# The human olfactory cleft mucus proteome and its age-related changes

**DOI:** 10.1038/s41598-018-35102-2

**Published:** 2018-11-21

**Authors:** Keiichi Yoshikawa, Hong Wang, Cristina Jaen, Mai Haneoka, Naoko Saito, Junji Nakamura, Nithin D. Adappa, Noam A. Cohen, Pamela Dalton

**Affiliations:** 1 Kao Corporation, Haga-Gun, Tochigi, 321-3497 Japan; 2 Monell Chemical Senses Center, Philadelphia, Pennsylvania USA; 3 Department of Otorhinolaryngology, Head and Neck Surgery, University of Pennsylvania, Philadelphia, Pennsylvania USA; 4 Philadelphia Veterans Affairs Medical Center Surgical Services, Philadelphia, Pennsylvania USA

## Abstract

Age-related decreases in olfactory sensitivity are often accompanied by a decrease in the quality of life. However, the molecular mechanisms underlying these changes are not well described. Inhaled substances including odorants are detected by sensory neurons in the olfactory cleft covered with a layer of mucus. This olfactory mucus is the first molecular machinery responsible for tissue protection and for detection of environmental odorants. Yet, little is known about the molecular identities of the actors because of the lack of information on the mucus proteome and its age-related changes. Here, we sampled human mucus from different nasal locations and from young and elderly subjects. The composition of the mucus was extensively analyzed by shotgun proteomic analysis for a vast array of proteins. We also explored correlations between the levels of each mucus proteins with the olfactory sensitivity of subjects. This analysis revealed previously unrecognized proteins with potentially important functions in olfaction. Taken together, this report describes the most comprehensive catalogue of the nasal mucus proteins to date, their positional and age-related differences, and candidate proteins associated with olfaction. This catalogue will provide fundamental information useful for future studies, such as identification of olfactory auxiliary proteins, causes of age-related declines in olfaction, and biomarkers for neurodegenerative disorders.

## Introduction

Olfaction, the sense of smell, is important for daily life. Odorants, toxic substances, and microorganisms are inhaled into the nasal cavity and make direct contact with the nasal mucosal layer. During this process, however, information is obtained from the external environment at the risk of tissue damage and infection. The information includes early warning for fire fume and polluted environments, flavor and palatability of food, and identities of other individuals. The decline in the ability to detect and discriminate odorants in older individuals renders one less aware of potential danger, diminished appetite supporting nutritional status, and decreases in the quality of life. The age-related decrease in olfactory function also tends to parallel the development of neurodegenerative disorders such as Alzheimer’s disease and sporadic Parkinson’s disease^1–3^. Clarifying the molecular nature of the olfactory system and its age-dependent changes could pave ways for not only understanding how the sense of smell is constructed and maintained but also developing tools for diagnosing neurodegenerative disorders.

The discovery of putative odorant receptors in olfactory sensory neurons (OSNs) initiated the molecular era of olfactory research^4,5^. OSNs scattered in the olfactory epithelium (OE) function to detect environmental odorants and convey odor information to higher brain areas^6^. In humans, the OE is located at the level of two narrow passages, the olfactory clefts (OCs), at the upper part of each nasal cavity. The OC is covered by a layer of mucus, which is secreted from Bowman’s glands and supporting cells^7^. Olfactory mucus is assumed to play multiple roles: maintenance of tissue integrity of the OE, protecting olfactory neurons, which are connected to the brain, from harmful volatiles and pathogenic microorganisms, and contributing to the detection of odorants. Recent studies using animal models have revealed that the olfactory mucus contributes more directly to odorant detection. Proteins in the olfactory mucus contribute to several pre-receptor events such as enzymatic metabolism of odorants and recruiting odorants to receptor sites^8–13^. Despite the importance of the presumed functions of olfactory mucus, the molecular identities of the actors responsible for these functions remain largely unknown.

There are only a few studies of human olfactory mucus, providing us with a small window into the range of olfactory mucus proteins. Mucus from the OC was compared to mucus from more anterior parts in the nasal cavity using a liquid chromatography-UV (LC-UV) spectrum^14^. This analysis indicated distinct molecular compositions, likely because the mucus in the OC and that in other parts of the nose are produced by different glands. Recently, a proteome analysis was conducted, and 83 proteins in human OC mucus were identified^15^. Despite these advances, considerable gaps have still remained. In the LC-UV spectrum study, odorant-binding proteins OBP2A and OBP2B, two members of the lipocalin family, were proposed as specific to OC mucus^14^. In contrast, a RNA-seq analysis indicated that the OBP genes did not show any OE-specific expression and the expression levels of the OBPs in the OE were quite low^16^. Alternatively, the OE was highly enriched with the transcripts of another lipocalin protein, which was not in the list of the 83 proteins determined by the above mentioned proteome analysis study^15^. Taken together, the following points remain to be determined: (1) the identities of additional OC mucus proteins beyond the ones identified so far, (2) the differences in the mucus protein composition across different nasal locations, and (3) the age-related changes of OC mucus proteins.

A previous proteomic analysis of human olfactory mucus was conducted using two-dimensional gel electrophoresis followed by MALDI-TOF-MS analysis^15^. This methodology has, in most cases, been replaced by a direct analysis using shotgun proteomics, which allows identification of a much larger number of proteins with higher reproducibility^17^. However, this methodology has not been applied to the analysis of olfactory mucus. Here, we employed a shotgun proteomics approach to provide the most comprehensive analysis of the protein composition of human olfactory mucus. Olfactory mucus from healthy, young (21–40 years old) and elderly (65–80 years old) individuals was collected using a technique that has previously been used to collect nasal secretions^18^. In order to determine the degree to which mucus collected from the OC truly differs from mucus collected from other locations in the nasal cavity, we also analyzed mucus from the anterior nasal cavity (hereafter referred to as ANC and see *Method*). Finally, we conducted a correlation analysis to investigate a relationship between olfactory sensitivity of participants and the levels of each mucus protein. This report describes the best understanding of the olfactory mucus protein composition to date, its tissue specificity, its age-related changes and candidate proteins which are involved in olfaction.

## Result and Discussion

### Data overview of olfactory cleft mucus

Olfactory mucus samples were obtained from the OC of 12 young and 12 elderly subjects (Table S1). Protein concentrations of OC mucus were 17 ± 4.8 mg/ml in young and 15 ± 5.6 mg/ml in elderly (Table 1 and Table S1). These concentrations were much higher than in saliva (1–3 mg/ml)^19^. 2.5 μg protein equivalent of each mucus sample was digested by trypsin and applied for shotgun proteome analysis. As a result, the average number of identified proteins with high confidence was 1236 ± 230 in young and 1227 ± 274 in elderly subjects (Mean ± S.D., FDR = 1.0%, Table 1). The total number of identified OC protein species is 2987 from 24 subjects (young: 2502, elderly: 2586, respectively, Table 1, Tables S2 and S3). This number is much larger than 83 reported in a previous proteomic analysis and covers 75 of the 83 protein species (90%) identified in the report^15^.

**Table 1:** Summary of olfactory mucus proteome analysis.

	Young (21–40, n = 12)		Elderly (65–80, n = 12)	
	ANC	OC	ANC	OC
Protein conc. (mg/ml)	15.2 ± 5.4	17.3 ± 4.8	12.9 ± 5.6	15.3 ± 5.6
No. of identified proteins	959 ± 234	1236 ± 230	995 ± 196	1227 ± 274
Total Identified proteins	2053	2502	1928	2586
ANC-OC-common proteins	1861		1866	
Young-Elderly-common proteins	1577			

The identified proteins include those with known intracellular functions. The presence of these proteins could potentially represent tissue damage from our sampling methodology. Alternatively, the fact that olfactory cells undergo continuous turnover during which they release a variety of intracellular proteins suggests that these proteins are natural components of olfactory mucus.

It is worthwhile to note that the shotgun proteomics approach is insensitive for growth factors and cytokines because these proteins are usually small in size and produced in small quantities. Thus we conducted antibody-based Luminex multiplex assays to analyze the profile of 30 cytokines and growth factors in the mucus, its age-related change and association with olfactory sensitivity of subjects as reported recently^20^.

We compared our OC mucus proteome with the previously reported transcriptome of the elderly OE^16^. The transcripts of the 2209 of the 2586 identified OC mucus proteins (85%) were detected from the OE in the RNA-seq analysis. The levels of mRNA in the OE biopsies and the levels of corresponding proteins in the mucus show a slight but significant correlation (*r* = 0.51, *P* < 0.0001; Supplementary Fig. S1). In contrast, mRNA from the respiratory epithelium showed less correlation (*r* = 0.21). These observations suggest that the OC proteome reflects gene expression in the OE, ensuring validity of our analysis.

Thus, we describe below the most comprehensive view of the olfactory mucus proteins identified in humans using shotgun proteomics analyses.

### Comparison between the OC and the ANC proteomes

There is broad consensus that tissue-specific expression has functional implications^16,21^ Because OSNs are located in the OC, proteins relevant for odorant recognition should be more abundant in the OC mucus than in the mucus covering any other nasal regions. A previous study reported a comparison of HPLC profiles of mucus sampled at different nasal sub-locations: the OC and more anterior regions in the nasal cavity, implying differences in the protein composition despite their anatomical proximity^14^. However, the observed HPLC profiles were based on UV absorption at 215 nm and therefore were not necessarily derived from proteins. Here we directly examined proteomic differences between OC and ANC mucus in order to identify candidate proteins which play important roles in olfaction.

**Figure 1:**
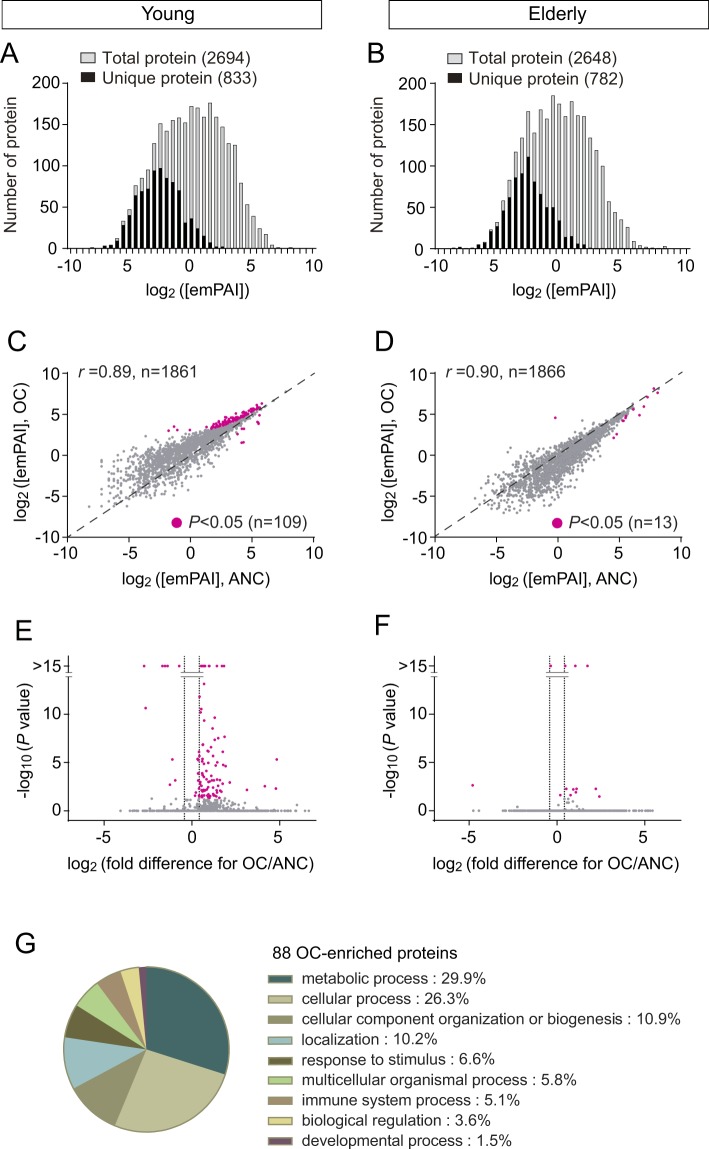
Comparison of proteome between the OC and the ANC. (**A,B**) Histogram shows the numbers of proteins in each bin corresponding to estimated proteins concentration ([emPAI]) range. Grey bars show distribution of the maximum [emPAI] value of each protein from two different positions of mucus (entire proteome). Black bars indicate the numbers of unique proteins which were specifically detected either in the OC or the ANC. (**C,D**) Scatter plots of average protein concentrations from two nasal regions. Common protein constituents of the OC and the ANC were used for this analysis (Young: 1861, elderly: 1866). Average [emPAI] value of each protein was plotted. Magenta dots represent statistically enriched proteins with *P* < 0.05, after adjusting for multiple comparisons. Spearman rank correlation coefficient (r) was used as a measure of the divergence of protein. (**E,F**) Volcano plot showing enrichment of proteins either in the OC or the ANC. Magenta dots correspond to the dots shown in Fig. 2C,D. Horizontal line shows log_2_ of OC/ANC value calculated using [emPAI] of each protein. Dashed lines represent ± 1.3-fold change which is second criteria for selecting OC enriched proteins. (**G**) Functional distribution of 88 OC enriched proteins according to biological process. Classification was conducted by PANTHER.

We sampled ANC mucus from the same subjects and analyzed protein composition by shotgun proteomics. Protein concentration of ANC mucus was lower than that of OC mucus (*P* = 0.025, Wilcoxon matched-pairs signed rank test, n = 24; Table 1). Shotgun proteomics identified 959 ± 234 proteins in young and 995 ± 196 proteins in elderly on average (Table 1). In the 12 young subjects, the number of protein species identified was 2053 from the ANC mucus versus 2502 from the OC mucus (Table 1). They comprised in total 2694 protein species, including 1861 that were detected in both ANC and OC mucus and 833 that were detected only in the OC or the ANC mucus. On the other hand, we detected 2648 proteins from the elderly mucus samples, of which 1866 were detected in both the ANC and OC mucus and 782 were detected either in the ANC or the OC mucus. The abundance of each protein was estimated using emPAI, a reliable quantification method, especially when the difference is above 1.3-fold^22–24^. We calculated emPAI concentration (emPAI per 1 μl mucus) for each protein and made comparisons between the OC and the ANC. The distribution of each protein concentration indicated that the 833 and 782 unique proteins identified from the young or the elderly, respectively, were quantitatively minor components within the entire proteome of the OC and ANC mucus (Fig. 1A,B). Then we compared the average concentrations of the proteins that were detected from both the OC and the ANC mucus (Young: 1861, Elderly: 1866). Scatter plots of the concentration distribution in the OC mucus relative to the ANC mucus revealed a high correlation coefficient (Young: *r* = 0.89, *P* < 0.0001, Elderly: *r* = 0.90, *P* < 0.0001, Fig. 1C,D). Wide non-uniform spread at the low abundance area was also observed, probably due to reduced accuracy of measurements of the peptides that are present at close to the background level as previously described^25^. These results indicate that the overall protein contents of the OC and ANC mucus are similar in young and elderly. Mucus in the OC and the ANC has been suggested to be produced by different glands and therefore differ in composition^14^. However, our result suggests that a continuous layer of mucus bathes the nasal epithelium and some mixing occurs.

Although the vast majority of proteins in the OC and ANC mucus were detected at comparable abundances, a subset of proteins was enriched in the mucus from either of the two positions. When the *P* value (concentration in the OC vs. ANC mucus) is below 0.05 (FDR-adjusted) and the OC/ANC ratio of the average concentration (emPAI/μl) is above 1.3, the protein is considered OC-enriched^24^. 109 out of 2694 proteins identified from the young subjects met the statistical criteria of adjusted *P* values below 0.05 (Magenta in Fig. 1C). The same analysis on the elderly proteome resulted in 13 proteins with significant differences (Magenta in Fig. 1D). Thus, we selected 109 and 13 proteins as candidates for either ANC- or OC-enriched proteins. Using the second criteria of OC/ANC ratios above 1.3, we identified 87 out of the 109 proteins from the young subjects as OC-enriched (Fig. 1C,F and Table S4). Similarly, 10 out of the 13 elderly proteins were identified as OC-enriched (Table S5). Collectively, we concluded that these 87 and 10 proteins were OC-enriched proteins in young and elderly, respectively. Young OC-enriched proteins were not necessarily abundant in the elderly, potentially due to age-dependent alterations. Therefore, we describe below an overview of the OC mucus-enriched proteins by focusing on young subjects (Table 2).

**Table 2:** OC-enriched proteins.

No.	Accession	Mass	Description	emPAI/μl					Significance	No. in other tables
				OC (n = 12)		ANC (n = 12)				
				Average	SEM	Average	SEM	OC/ANC	−log10 (*P*)	
1	O60739	12930	Eukaryotic translation initiation factor 1b	11.4	1.1	0.4	0.1	28.4	5.3	
2	P54652	70263	Heat shock-related 70 kDa protein 2	8.0	0.6	0.3	0.1	27.4	2.3	
3	P62314	13273	Small nuclear ribonucleoprotein Sm D1	8.5	0.6	0.5	0.1	17.8	2.6	
4	Q8WYR4	35159	Radial spoke head 1 homolog	8.5	0.7	1.0	0.2	8.8	2.2	
5	P08758	35971	Annexin A5	11.0	1.1	2.5	0.5	4.4	2.9	
6	Q99417	11959	C-Myc-binding protein	14.0	1.0	3.7	0.6	3.8	4.6	
7	Q9Y3Z3	72896	SAM domain and HD domain-containing protein 1	17.9	0.9	4.9	0.4	3.6	7.7	
8	P63208	18817	S-phase kinase-associated protein 1	25.2	1.6	7.1	0.6	3.5	>15	
9	Q9NQR4	30988	Omega-amidase NIT2	14.7	0.9	4.3	0.3	3.4	4.7	
10	P00325	40684	Alcohol dehydrogenase 1B	16.6	0.8	4.9	0.6	3.4	6.1	
11	P02144	17230	Myoglobin	11.9	0.7	3.5	0.4	3.4	2.8	
12	P80723	22680	Brain acid soluble protein 1	24.7	1.5	7.5	0.4	3.3	>15	
13	P49458	10219	Signal recognition particle 9 kDa protein	10.6	0.7	3.3	0.4	3.2	2.0	
14	Q96NY7	73196	Chloride intracellular channel protein 6	11.1	0.6	3.6	0.2	3.0	2.1	
15	P62888	12947	60S ribosomal protein L30	13.2	0.9	4.4	0.4	3.0	3.2	
16	Q02878	32765	60S ribosomal protein L6	9.6	0.8	3.3	0.4	2.9	1.3	
17	Q13885	50274	Tubulin beta-2A chain	19.9	1.0	7.1	0.7	2.8	7.5	
18	Q9NQ48	34628	Leucine zipper transcription factor-like protein 1	12.6	0.7	4.5	0.5	2.8	2.6	
19	P07355	38808	Annexin A2	16.9	0.8	6.2	0.5	2.7	5.0	
20	Q9Y265	50538	RuvB-like 1	14.1	0.7	5.3	0.5	2.7	3.2	
21	P0CW22	15597	40S ribosomal protein S17-like	11.4	0.7	4.2	0.3	2.7	1.9	
22	Q71U36	50788	Tubulin alpha-1A chain	34.5	1.4	12.9	1.0	2.7	>15	
23	P60900	27838	Proteasome subunit alpha type-6	12.0	0.5	4.5	0.2	2.6	2.1	
24	P18085	20612	ADP-ribosylation factor 4	10.7	0.7	4.1	0.4	2.6	1.5	
25	P31150	51177	Rab GDP dissociation inhibitor alpha	17.1	0.7	6.7	0.5	2.5	4.7	
26	P09104	47581	Gamma-enolase	11.3	0.8	4.6	0.4	2.5	1.6	
27	P41567	12839	Eukaryotic translation initiation factor 1	21.4	0.8	8.7	0.6	2.5	7.4	
28	P51858	26886	Hepatoma-derived growth factor	24.3	1.1	9.9	0.7	2.4	9.6	
29	P36405	20614	ADP-ribosylation factor-like protein 3	15.2	0.7	6.4	0.5	2.4	3.1	
30	P05386	11621	60S acidic ribosomal protein P1	11.8	0.6	5.0	0.4	2.3	1.6	
31	Q9H4A4	73234	Aminopeptidase B	12.3	0.6	5.3	0.3	2.3	1.7	
32	P49773	13907	Histidine triad nucleotide-binding protein 1	20.0	1.1	8.7	0.3	2.3	5.7	
33	P61204	20645	ADP-ribosylation factor 3	20.0	0.8	8.7	0.5	2.3	5.7	
34	P10768	31956	S-formylglutathione hydrolase	13.9	0.9	6.1	0.6	2.3	2.4	
35	P30050	17979	60S ribosomal protein L12	24.5	1.2	10.9	0.9	2.2	8.5	
36	P62820	22891	Ras-related protein Rab-1A	16.1	0.8	7.4	0.9	2.2	3.1	
37	P30043	22219	Flavin reductase (NADPH)	17.4	0.7	8.1	0.5	2.1	3.6	
38	P68371	50255	Tubulin beta-4B chain	22.9	1.1	10.8	0.8	2.1	6.6	
39	Q99832	59842	T-complex protein 1 subunit eta	13.0	0.7	6.3	0.4	2.1	1.5	
40	P17987	60819	T-complex protein 1 subunit alpha	17.3	0.8	8.6	0.5	2.0	3.0	
41	O00764	35308	Pyridoxal kinase	12.7	0.7	6.4	0.4	2.0	1.3	
42	P00441	16154	Superoxide dismutase [Cu-Zn]	38.2	1.3	19.2	0.8	2.0	>15	
43	Q9BW30	19145	Tubulin polymerization-promoting protein family member 3	39.6	1.6	20.1	1.1	2.0	>15	
44	Q99497	20050	Protein DJ-1	37.2	1.4	19.0	0.7	2.0	>15	
45	P23396	26842	40S ribosomal protein S3	17.9	0.6	9.4	0.5	1.9	3.0	
46	P16050	75498	Arachidonate 15-lipoxygenase	24.6	0.9	12.8	0.6	1.9	6.1	
47	P06454	12196	Prothymosin alpha	14.5	0.5	7.6	0.3	1.9	1.7	
48	P07437	50095	Tubulin beta chain	22.4	1.1	11.7	0.6	1.9	5.0	
49	O60701	55674	UDP-glucose 6-dehydrogenase	16.8	0.5	8.8	0.4	1.9	2.5	
50	P25786	29822	Proteasome subunit alpha type-1	13.7	0.6	7.2	0.3	1.9	1.4	
51	P37802	22548	Transgelin-2	25.6	1.0	13.8	0.3	1.9	6.2	
52	P05388	34423	60S acidic ribosomal protein P0	14.8	0.8	8.1	0.6	1.8	1.6	
53	P22626	37464	Heterogeneous nuclear ribonucleoproteins A2/B1	17.1	0.8	9.5	0.4	1.8	2.2	
54	Q96KP4	53187	Cytosolic non-specific dipeptidase	20.4	0.7	11.4	0.5	1.8	3.3	
55	Q13228	52928	Selenium-binding protein 1	22.7	0.9	12.9	0.6	1.8	4.1	
56	P28838	56530	Cytosol aminopeptidase	18.2	0.6	10.7	0.5	1.7	2.1	
57	P55072	89950	Transitional endoplasmic reticulum ATPase	18.3	0.7	10.8	0.4	1.7	2.1	
58	P09211	23569	Glutathione S-transferase P	70.3	2.8	42.1	2.0	1.7	>15	#20 in Table 3
59	P00326	40697	Alcohol dehydrogenase 1C	49.7	2.1	30.3	1.2	1.6	>15	
60	O00151	36505	PDZ and LIM domain protein 1	17.7	0.7	10.9	0.4	1.6	1.6	
61	P50395	51087	Rab GDP dissociation inhibitor beta	37.0	1.6	22.8	0.9	1.6	9.4	#14 in Table 3
62	P04083	38918	Annexin A1	43.4	2.5	26.9	1.7	1.6	13.1	#27 in Table 4
63	P68366	50634	Tubulin alpha-4A chain	19.6	1.3	12.2	0.8	1.6	2.1	
64	P07900	85006	Heat shock protein HSP 90-alpha	24.3	0.7	15.1	0.6	1.6	3.5	
65	P10599	12015	Thioredoxin	27.9	1.2	17.4	0.9	1.6	4.9	
66	P05387	11658	60S acidic ribosomal protein P2	51.0	2.0	32.0	2.6	1.6	>15	
67	P29401	68519	Transketolase	20.5	0.8	13.0	0.4	1.6	2.2	
68	P68036	18021	Ubiquitin-conjugating enzyme E2 L3	20.6	0.8	13.1	0.5	1.6	2.1	
69	P14550	36892	Alcohol dehydrogenase [NADP(+)]	35.1	1.4	22.8	0.8	1.5	6.9	#15 in Table 3
70	P62258	29326	14-3-3 protein epsilon	35.4	1.0	23.1	0.9	1.5	6.8	
71	P06703	10230	Protein S100-A6	27.2	1.2	17.8	1.1	1.5	3.7	
72	P02652	11282	Apolipoprotein A-II	32.3	1.0	21.3	1.0	1.5	5.4	
73	P13489	51766	Ribonuclease inhibitor	21.5	0.9	14.2	0.5	1.5	2.0	
74	P40394	42253	Alcohol dehydrogenase class 4 mu/sigma chain	28.5	1.2	18.9	0.9	1.5	3.9	
75	P30044	22301	Peroxiredoxin-5, mitochondrial	52.0	2.1	34.5	1.4	1.5	>15	
76	P08263	25672	Glutathione S-transferase A1	32.3	1.3	21.5	1.4	1.5	5.1	
77	P30085	22436	UMP-CMP kinase	20.3	0.6	13.6	0.8	1.5	1.6	
78	P60981	18950	Destrin	19.6	0.6	13.1	0.7	1.5	1.4	
79	P32119	22049	Peroxiredoxin-2	23.5	1.0	16.0	0.5	1.5	2.1	
80	P08238	83554	Heat shock protein HSP 90-beta	25.9	0.7	17.8	0.6	1.5	2.6	
81	P04075	39851	Fructose-bisphosphate aldolase A	48.5	1.6	33.5	1.4	1.4	10.5	
82	P00352	55454	Retinal dehydrogenase 1	80.3	2.7	55.9	2.7	1.4	>15	
83	Q13938	21068	Calcyphosin	50.8	1.8	36.0	1.9	1.4	10.2	
84	P30041	25133	Peroxiredoxin-6	30.2	1.2	21.7	1.6	1.4	3.0	
85	P27348	28032	14-3-3 protein theta	30.3	1.0	21.9	0.7	1.4	2.8	
86	P60174	31057	Triosephosphate isomerase	59.9	2.5	44.2	2.0	1.4	11.8	
87	P31949	11847	Protein S100-A11	43.7	2.5	32.4	1.6	1.3	5.7	

### OC mucus-enriched proteins

Because not much information on positional differences of mucus proteins is available, to evaluate the identified OC-enriched proteins we compared our data with the data from a transcriptome analysis of human OE-expressed genes^16^. This transcriptome analysis did not clearly identify OBP2A and OBP2B, two human lipocalins whose orthologs are highly expressed in the olfactory tissue in other mammalian species^16,26^. Instead, proteins with the highest enrichment in the OE compared with other tissues were another lipocalin member and the bactericidal/permeability-increasing (BPI)-fold containing proteins^27^. Consistently, our shotgun proteome analysis was unable to identify OBP2A and OBP2B in the OC mucus, although we cannot exclude the possibility that there exists degradation products of OBPs or OBPs with post-translational modifications^15,28^. Alternatively, a lipocalin, LCN15, and a BPI-fold containing protein, BPIFB4, were detected with a clear trend of OC-enrichment (Supplementary Fig. S2). Although these two proteins did not meet our criteria of OC-enriched described above and applied in Fig. 1C, statistically significant differences between their levels in the OC and the ANC mucus were observed when the levels were individually compared using student’s *t*-test (*p* < 0.003 for LCN15, *p* < 0.043 for BPIFB4). Lipocalins and BPI-fold containing proteins were suggested to function as odorant-binding proteins to carry or remove odorants^27,29^. Thus, an evolutionary process seems to have selected LCN15 and BPIFB4 instead of OBP2A and OBP2B for human olfaction. The consistency of the enrichments supports the reliability of our identification of OC-enriched proteins.

One of the proteins showing the largest enrichment in the OC mucus is heat shock-related 70 kDa protein 2 (HSPA2). HSP70 family of heat shock proteins are expressed in human OSNs, and one such protein, HSC70t, acts as a molecular chaperone for odorant receptors^30,31^. Interestingly, HSP70 appears to be secreted into olfactory mucus and play additional roles, in a secreted form, in immune and inflammatory responses^32,33^.

Then we classified OC-enriched proteins based on PANTHER (protein analysis through evolutionary relationships) classification system (Fig. 1G). The analysis identified the most dominant category of the OC-enriched proteins is proteins involved in metabolic processes. The large number of proteins in this category may reflect the requirement of protection mechanisms that shield OSNs from inhaled harmful compounds including some odorants. This category comprises three dehydrogenases (ADH1B, ADH1C and ADH7) which are known to metabolize toxic compounds with alcohol moiety^34^. Xenobiotic metabolizing enzymes, glutathione *S*-transfrases of GSTA1 and GSTP1, which were shown to convert odorant structures in model animals, were also enriched in the OC mucus, in agreement with a previous observation^35^. A member of chloride intracellular ion channels (CLIC6) was also identified. CLICs have been known to function not only as membrane channels but also GST-like enzymes in soluble forms^36^. These metabolizing enzymes are likely involved not only in detoxification but also for efficient odorant detection by enzymatic conversion before binding of chemosensory receptors^10^. Taken together, we provide a list of OC-enriched proteins which could be used to search for candidate proteins involved in olfactory-specific processes.

### Age-related changes in nasal mucus protein compositions

**Figure 2:**
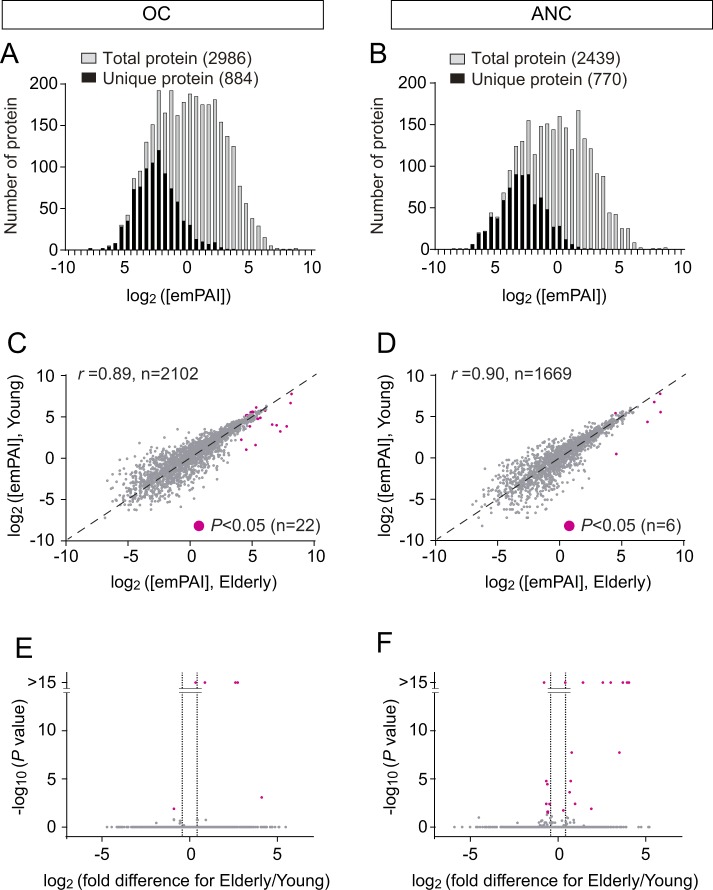
Comparison of proteome between young and elderly. (**A,B**) Histogram shows the numbers of proteins in each bin corresponding to estimated proteins concentration ([emPAI]) range. Grey bars represent entire proteome. Black bars indicate the numbers of unique proteins which were specifically detected either in young mucus or elderly mucus. (**C,D**) Scatter plots of average protein concentrations from two age groups. Common protein constituents of young and elderly were used for this analysis (OC: 2102, elderly: 1669). Average [emPAI] value of each protein was plotted. Magenta dots represent statistically enriched proteins with *P* < 0.05, after adjusting for multiple comparisons. Spearman rank correlation coefficient (r) was used as a measure of the divergence of protein. (**E,F**) Volcano plot shows enrichment of proteins either in young or elderly. Magenta dots correspond to the dots shown in Fig. 2C,D.

To investigate age-related difference in the mucus proteome, olfactory mucus from young and elderly individuals was similarly analyzed. The protein concentrations of the OC and ANC mucus were not significantly different between young and elderly (*P* = 0.41 for OC, *P* = 0.39 for ANC, Mann-Whitney test). The number of the identified proteins did not differ significantly between the two age groups (Table 1). The overall protein compositions are also similar: the unique proteins in either of young or elderly were found to be minor components and concentrations of the common protein constituents are comparable (Fig. 2A–D). Nonetheless, we successfully discovered 22 proteins showing age-related differences using similar criteria applied for classifying OC-enriched proteins. In our shotgun proteome analysis, 22 out of 2986 proteins showed adjusted *P* values below 0.05 (protein levels in young vs. elderly, Magenta, Fig. 2C–F). Our second criteria based on elderly/young or young/elderly ratios of average concentration (emPAI/μl) above 1.3 excluded two proteins, thus, 20 proteins from our shotgun proteome analysis show age-related changes (Table 3 and Table S6).

**Table 3:** Age-related OC Proteins.

No.	Accession	Mass	Description	emPAI/μl					Significance	No. in other tables
				EmPAI/μl from elderly (n = 12)		EmPAI/μl from young (n = 12)				
				Average	SEM	Average	SEM	Elderly/Young	−log10 (*P*)	
1	P06702	13291	Protein S100-A9	155.2	19.2	9.5	1.0	16.4	>15	#31 in Table 4
2	P05109	10885	Protein S100-A8	221.8	24.6	14.5	1.9	15.3	>15	#15 in Table 4
3	P02042	16159	Hemoglobin subunit delta	38.9	3.7	3.0	0.8	12.9	>15	
4	P62805	11360	Histone H4	23.3	3.5	2.1	0.3	11.3	7.7	
5	P68871	16102	Hemoglobin subunit beta	126.5	11.8	15.9	1.4	8.0	>15	
6	P69905	15305	Hemoglobin subunit alpha	98.7	13.3	16.9	2.0	5.8	>15	
7	P02766	15991	Transthyretin	17.6	1.8	4.7	0.4	3.7	1.9	
8	P01834	11773	Ig kappa chain C region	274.5	13.2	102.6	4.0	2.7	>15	
9	P80188	22745	Neutrophil gelatinase-associated lipocalin	28.4	1.2	14.5	0.5	2.0	2.4	
10	P02788	80014	Lactotransferrin	51.0	2.2	29.8	1.4	1.7	7.7	
11	P07737	15216	Profilin-1	44.8	1.3	27.3	0.7	1.6	4.8	
12	P60709	42052	Actin, cytoplasmic 1	43.2	1.1	27.4	0.6	1.6	3.6	
13	O00299	27248	Chloride intracellular channel protein 1	34.6	13.4	48.3	7.0	0.7	2.4	
14	P50395	51087	Rab GDP dissociation inhibitor beta	24.8	3.0	37.0	1.8	0.7	1.6	#61 in Table 2
15	P14550	36892	Alcohol dehydrogenase [NADP(+)]	23.2	1.1	35.1	1.6	0.7	1.5	#69 in Table 2
16	P04080	11190	Cystatin-B	23.5	0.9	35.7	1.6	0.7	1.6	
17	P01040	11000	Cystatin-A	32.3	1.0	49.4	1.4	0.7	4.4	#19 in Table 5
18	P30086	21158	Phosphatidylethanolamine-binding protein 1	29.7	1.3	47.2	1.7	0.6	4.8	
19	Q99497	20050	Protein DJ-1	23.3	1.0	37.2	2.0	0.6	2.4	#44 in Table 2
20	P09211	23569	Glutathione S-transferase P	40.5	1.3	70.3	1.8	0.6	>15	#58 in Table 2

These 20 identified age-related proteins include some previously known age-related proteins. It has been suggested that alpha and pi members of glutathione S-transferases (GSTA and GSTP) in olfactory mucus were decreased in older subjects^35^. Consistently, GSTP showed the largest age-dependent difference among the 20 proteins from our shotgun proteome analysis (Table 3). Similar declining trends were also observed in the major GSTAs (A1 and A3) from the elderly mucus (Supplementary Fig. S3). We also detected elderly mucus-specific enrichments of two pro-inflammatory proteins, S100a8 and S100a9 (Supplementary Fig. S5), which are known to robustly increase during normal aging in the central nervous system^37^.

Aging is commonly associated with a state of chronic inflammation which contributes to DNA damage and cognitive decline^38–40^. Correspondingly, we found that mucus from the elderly was enriched with inflammation-related proteins (Table 3). OC mucus proteins with the largest differences between the two age groups were S100-A9 and S100-A8, which were central inflammatory regulators capable of driving and responding to inflammation signals^41–43^. It is worth noting that the heterocomplex of S100A8-S100A9 contributes to amyloid plaque accumulation and a decline of cognitive performance in an Alzheimer’s disease mouse model^44^. Hemoglobin subunits which are elevated in elderly individuals may imply inflammation-related bleeding as is the case reported in the saliva of subjects with early stages of periodontal disease^45^. Transthyretin, which is a tetrameric protein, plays a role in transporting vitamin A and thyroxine and has been suggested to be related to inflammation and neurodegenerative deseases^46,47^. A light chain, Ig kappa chain C region, was also increased in the elderly mucus. Increased free light chain concentration has been described in a variety of inflammatory and autoimmune diseases including rhinitis^48,49^. Neutrophil gelatinase-associated lipocalin (also called lipocalin 2) was enriched in the elderly OC mucus. This protein was derived from macrophages and was shown to increase in patients with inflammation^50^.

Several proteins that showed reduced levels in the mucus from elderly subjects are also associated with inflammation and neural dysfunction. Dj-1 has a neuronal protective role against oxidative stress: loss of function of Dj-1 variant has been linked to early onset forms of parkinsonism^51,52^. Phosphatidylethanolamine binding protein 1 (PEBP-1), which is also known as Raf kinase inhibitory protein, is involved in several cellular processes^53^. In *Drosophila*, RKIP orthologs are expressed in an olfactory organ and function in carrying odorants into chemoreceptors^54^. The disruption of PEBP1 was not only associated with a wide range of diseases, including Alzheimer’s disease, but also resulted in low olfactory performance in older mice^53,55^. Cystatins A and B (also known as stefin A and B, respectively) were also lower in the mucus of the elderly than of the young. Cystatin A functions in tissues which participate in the first line of defense against pathogens. Polymorphysm in Cystatin A has been linked with atopic dermatitis, a chronic inflammatory skin disease^56^. Cystatin B is linked to inflammation and neurodegenerative disorders^56–58^. Interestingly, deficiency in cystatin B gene results in decrease of the anti-inflammatory cytokine, IL-10, which play a role in preventing inflammation^59^.

Some proteins that decreased with age are likely involved in metabolism and detection of odorants. Metabolic enzymes, including a glutathione S-transferase (GSTP), chloride intracellular channel protein 1 and alcohol dehydrogenase [NADP(+)], were decreased in elderly subjects. They may contribute to the clearance of recognized odorants from mucus in order to protect the OE from harmful compounds as well as to prevent prolonged adaptation which is often reported among the elderly^60^. Interestingly, glutathione S-transferase(s) was also suggested to affect odor perception by rapidly metabolizing inhaled odorants before receptor binding^11^. Chloride intracellular channel proteins may have a similar metabolic function as glutathione S-transferase^35^. PEBP-1 may also function as odorant-binding proteins, which recruit hydrophobic odorants into receptor sites^54^. These proteins probably metabolize and carry only a small fraction of odorants based on their substrate selectivity. Therefore the age-related decrease seems to explain odorant-selective decline of olfaction among the elderly as reported previously^61^. But decrease of these mucus proteins may also explain a general decline of olfaction. Odors in our daily life are composed of complex mixture of odorant components. Declines in the function of mucus for detecting a small fraction of odorant components may cause different patterns of activation of the receptors and consequently different odor quality perception, resulting in errors in identification. Thus these age-related proteins associated directly with odorants likely contribute to the peripheral causes of age-related decline of olfaction.

### Mucus proteins associated with olfactory sensitivity

**Figure 3:**
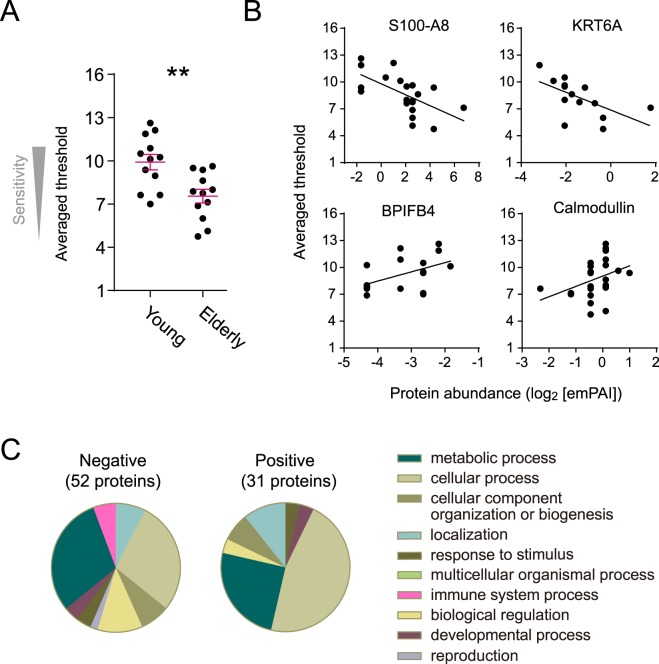
OC proteins associated with olfactory sensitivity. (**A**) Olfactory sensitivity of subjects. An averaged value of thresholds for phenylethylalcohol and n-butanol of each subject is plotted. Mean ± SE are shown in magenta. Asterisks (**) denote significant *p*-values (Student’s *t*-test *p*-value < 0.01). **(B**) Examples of OC proteins which show significantly negative or positive correlation with olfactory sensitivity of participants. Data are from 24 participants including young and elderly. Data from participants with protein level under detection limit (zero) are not displayed on a log axis. (**C**) Functional distribution of 52 and 31 proteins which are negatively and positively correlated with olfactory sensitivity. Classification was conducted by PANTHER according to biological process.

In order to associate individual differences in the composition of olfactory mucus with a functional outcome, we conducted sensory tests to characterize one of the types of olfactory function in the participants. Olfactory thresholds for two odorants, phenylethyl alcohol and n-butanol were measured for each participant. The average value of thresholds for the two odorants was utilized as an index of olfactory sensitivity. The olfactory sensitivity values showed a statistically significant difference between the two age groups (Fig. 3A and Table S1, P = 0.003, Student’s *t*-test), consistent with general knowledge on age-related decline in olfaction^62,63^. This analysis also highlighted substantial individual variability. The origins for the observed variability are both of individual and age-related differences. In addition, the variability could be caused by differences in not only nasal mucus but also molecular and cellular compositions in the olfactory epithelium and the brain. But in the present study, we searched for mucus proteins that may account for the variability. A Spearman correlation was used to test 2989 OC-mucus proteins for their correlation with the averaged olfactory sensitivity. As a result, a total of 83 proteins were significantly correlated at *P* values < 0.05 across 24 subjects. 52 and 31 proteins were negatively and positively correlated, respectively (Tables 4 and 5). The full set of proteins with significant correlations was listed in Table S7.

**Table 4:** Negatively-correlated OC protein.

No.	Accession	Mass	Description	Spearman’s rho	P value	EmPAI/μl from young (n = 12)		EmPAI/μl from elderly (n = 12)		EmPAI/μl from total subjects (n = 24)	
						Average	SE	Average	SE	Average	SE
1	P53611	37585	Geranylgeranyl transferase type-2 subunit beta	−0.58	0.0029	0.7	0.1	1.3	0.1	1.0	0.0
2	P02533	51872	Keratin, type I cytoskeletal 14	−0.57	0.0033	0.7	0.2	8.2	0.8	4.4	0.3
3	O43237	54351	Cytoplasmic dynein 1 light intermediate chain 2	−0.52	0.0085	2.4	0.2	2.3	0.1	2.3	0.1
4	Q6UX06	57529	Olfactomedin-4	−0.52	0.0086	0.1	0.0	0.9	0.2	0.5	0.1
5	P19012	49409	Keratin, type I cytoskeletal 15	−0.52	0.0091	0.6	0.2	1.9	0.3	1.2	0.1
6	P18065	35875	Insulin-like growth factor-binding protein 2	−0.52	0.0097	0.0	0.0	0.5	0.1	0.2	0.0
7	P21333	283301	Filamin-A	−0.52	0.0099	0.0	0.0	0.6	0.1	0.3	0.0
8	P19013	57649	Keratin, type II cytoskeletal 4	−0.51	0.0116	0.3	0.1	1.2	0.2	0.7	0.1
9	O76071	38842	Probable cytosolic iron-sulfur protein assembly protein CIAO1	−0.50	0.012	0.5	0.1	0.3	0.1	0.4	0.0
10	Q96B54	20867	Zinc finger protein 428	−0.50	0.012	1.2	0.2	0.8	0.1	1.0	0.1
11	Q14D04	95484	Ventricular zone-expressed PH domain-containing protein homolog 1	−0.50	0.0127	0.1	0.0	0.1	0.0	0.1	0.0
12	Q53FA7	35685	Quinone oxidoreductase PIG3	−0.50	0.0128	0.5	0.1	1.3	0.1	0.9	0.1
13	Q9UHD9	65655	Ubiquilin-2	−0.50	0.0128	0.2	0.0	0.4	0.0	0.3	0.0
14	P17213	54093	Bactericidal permeability-increasing protein	−0.49	0.015	0.1	0.0	0.5	0.1	0.3	0.0
15	P05109	10885	Protein S100-A8	−0.49	0.0158	14.5	1.9	221.8	24.6	118.2	9.7
16	P07738	30158	Bisphosphoglycerate mutase	−0.48	0.0169	0.2	0.1	0.2	0.0	0.2	0.0
17	Q9Y6G5	23009	COMM domain-containing protein 10	−0.48	0.0169	0.3	0.1	0.3	0.1	0.3	0.0
18	Q6IA69	80545	Glutamine-dependent NAD(+) synthetase	−0.48	0.0178	0.0	0.0	0.1	0.0	0.1	0.0
19	Q9HA65	73367	TBC1 domain family member 17	−0.48	0.0178	0.0	0.0	0.1	0.0	0.1	0.0
20	Q8TC07	80352	TBC1 domain family member 15	−0.48	0.0179	0.4	0.0	0.2	0.0	0.3	0.0
21	P13646	49900	Keratin, type I cytoskeletal 13	−0.48	0.0189	0.4	0.1	4.4	0.6	2.4	0.2
22	A6NHG4	14414	D-dopachrome decarboxylase	−0.47	0.0197	0.9	0.2	0.2	0.1	0.5	0.1
23	Q8IZP0	55161	Abl interactor 1	−0.47	0.0204	0.2	0.0	0.3	0.0	0.3	0.0
24	Q06033	100072	Inter-alpha-trypsin inhibitor heavy chain H3	−0.46	0.0239	0.0	0.0	0.4	0.0	0.2	0.0
25	P04434	12863	Ig kappa chain V-III region VH (Fragment)	−0.46	0.0252	0.0	0.0	6.7	0.5	3.3	0.2
26	Q8N9W5	59773	UPF0470 protein C19orf51	−0.45	0.026	0.1	0.0	0.5	0.1	0.3	0.0
27	P04083	38918	Annexin A1	−0.45	0.0287	43.4	2.5	40.2	1.4	41.8	1.0
28	P04745	58415	Alpha-amylase 1	−0.44	0.0296	0.0	0.0	0.5	0.0	0.3	0.0
29	P52566	23031	Rho GDP-dissociation inhibitor 2	−0.44	0.032	11.9	0.4	22.1	0.9	17.0	0.4
30	Q9Y6I9	34452	Testis-expressed sequence 264 protein	−0.44	0.0333	0.0	0.0	0.3	0.1	0.1	0.0
31	P06702	13291	Protein S100-A9	−0.43	0.0348	9.5	1.0	155.2	19.2	82.4	7.5
32	Q99615	57203	DnaJ homolog subfamily C member 7	−0.43	0.0362	0.0	0.0	0.4	0.1	0.2	0.0
33	Q5JSH3	101874	WD repeat-containing protein 44	−0.43	0.037	0.4	0.0	0.4	0.1	0.4	0.0
34	P15924	334021	Desmoplakin	−0.43	0.0375	0.0	0.0	0.3	0.0	0.2	0.0
35	Q13546	76453	Receptor-interacting serine/threonine-protein kinase 1	−0.42	0.0387	0.1	0.0	0.1	0.0	0.1	0.0
36	Q86XW9	37232	Thioredoxin domain-containing protein 6	−0.42	0.0402	0.4	0.1	0.6	0.1	0.5	0.0
37	Q96JH7	135604	Deubiquitinating protein VCIP135	−0.42	0.0403	0.2	0.0	0.1	0.0	0.1	0.0
38	P07996	133291	Thrombospondin-1	−0.42	0.0417	0.2	0.1	0.5	0.0	0.4	0.0
39	P39880	164544	Homeobox protein cut-like 1	−0.42	0.0417	0.0	0.0	0.1	0.0	0.0	0.0
40	P02538	60293	Keratin, type II cytoskeletal 6A	−0.42	0.0434	1.5	0.3	8.5	0.8	5.0	0.3
41	P52943	23276	Cysteine-rich protein 2	−0.42	0.0435	0.0	0.0	0.5	0.1	0.2	0.0
42	P38919	47126	Eukaryotic initiation factor 4A-III	−0.41	0.0438	0.0	0.0	1.4	0.1	0.7	0.1
43	Q9NXR7	43980	BRCA1-A complex subunit BRE	−0.41	0.0443	0.0	0.0	0.2	0.0	0.1	0.0
44	O15198	53429	Mothers against decapentaplegic homolog 9	−0.41	0.0443	0.0	0.0	0.1	0.0	0.1	0.0
45	Q9NR19	79613	Acetyl-coenzyme A synthetase, cytoplasmic	−0.41	0.0443	0.0	0.0	0.1	0.0	0.0	0.0
46	Q9H7C9	13438	UPF0366 protein C11orf67	−0.41	0.0443	0.0	0.0	0.5	0.1	0.3	0.0
47	P41218	46092	Myeloid cell nuclear differentiation antigen	−0.41	0.0453	0.9	0.1	1.7	0.1	1.3	0.1
48	P53609	43196	Geranylgeranyl transferase type-1 subunit beta	−0.41	0.0455	0.1	0.0	0.7	0.1	0.4	0.0
49	P35908	65678	Keratin, type II cytoskeletal 2 epidermal	−0.41	0.0455	2.0	0.3	4.9	0.3	3.5	0.2
50	P63104	27899	14-3-3 protein zeta/delta	−0.41	0.0457	55.4	1.8	52.5	1.7	54.0	0.9
51	O60256	41299	Phosphoribosyl pyrophosphate synthase-associated protein 2	−0.41	0.0462	1.4	0.2	0.9	0.1	1.2	0.1
52	P35527	62255	Keratin, type I cytoskeletal 9	−0.40	0.0499	1.8	0.2	5.1	0.2	3.5	0.1

**Table 5:** Positively-correlated OC protein.

No.	Accession	Mass	Description	Spearman’s rho	P value	EmPAI/μl from young (n = 12)		EmPAI/μl from elderly (n = 12)		EmPAI/μl from total subjects (n = 24)	
						Average	SE	Average	SE	Average	SE
1	P13693	19697	Translationally-controlled tumor protein	0.54	0.0061	4.9	0.3	2.8	0.1	3.8	0.1
2	Q14157	114579	Ubiquitin-associated protein 2-like	0.53	0.0082	0.2	0.0	0.0	0.0	0.1	0.0
3	Q8TD33	10578	Secretoglobin family 1 C member 1	0.52	0.0085	5.0	0.2	0.0	0.0	2.5	0.1
4	P08185	45283	Corticosteroid-binding globulin	0.50	0.0126	2.4	0.2	1.4	0.1	1.9	0.1
5	Q92597	43264	Protein NDRG1	0.49	0.0152	0.6	0.1	0.0	0.0	0.3	0.0
6	P59827	65356	BPI fold-containing family B member 4	0.49	0.0163	2.2	0.1	0.5	0.1	1.3	0.1
7	Q9UJU6	48463	Drebrin-like protein	0.48	0.0187	3.7	0.2	1.8	0.1	2.7	0.1
8	Q99436	30288	Proteasome subunit beta type-7	0.47	0.0196	0.6	0.1	0.4	0.1	0.5	0.0
9	P27216	35621	Annexin A13	0.46	0.0223	1.2	0.1	0.2	0.1	0.7	0.0
10	Q9NQ39	20279	Putative 40 S ribosomal protein S10-like	0.46	0.0227	0.8	0.2	0.5	0.1	0.7	0.1
11	O95236	44650	Apolipoprotein L3	0.46	0.0238	0.5	0.1	0.0	0.0	0.3	0.0
12	P35268	14835	60 S ribosomal protein L22	0.45	0.0282	8.4	0.5	5.2	0.4	6.8	0.2
13	P49458	10219	Signal recognition particle 9 kDa protein	0.44	0.0326	10.6	0.7	3.3	0.3	6.9	0.3
14	P62158	16827	Calmodulin	0.44	0.0327	12.1	0.5	16.1	0.8	14.1	0.4
15	P41567	12839	Eukaryotic translation initiation factor 1	0.44	0.0332	21.4	0.8	11.4	0.8	16.4	0.4
16	Q99426	27594	Tubulin-folding cofactor B	0.43	0.0339	1.1	0.1	0.4	0.1	0.7	0.0
17	Q6UWW0	20612	Lipocalin-15	0.43	0.0349	7.6	0.3	2.0	0.2	4.8	0.2
18	Q9HB40	51083	Retinoid-inducible serine carboxypeptidase	0.43	0.0364	0.8	0.0	0.1	0.0	0.5	0.0
19	P01040	11000	Cystatin-A	0.42	0.0417	49.4	2.0	32.3	1.0	40.8	0.9
20	Q13838	49416	Spliceosome RNA helicase DDX39B	0.42	0.0423	8.0	0.3	3.0	0.2	5.5	0.2
21	Q9UBI6	8115	Guanine nucleotide-binding protein G(I)/G(S)/G(O) subunit gamma-12	0.42	0.0425	3.7	0.3	0.0	0.0	1.8	0.1
22	Q8NBS9	48283	Thioredoxin domain-containing protein 5	0.42	0.0435	0.3	0.1	0.0	0.0	0.1	0.0
23	Q9Y5K3	42199	Choline-phosphate cytidylyltransferase B	0.42	0.0436	0.5	0.1	0.1	0.0	0.3	0.0
24	O14791	44004	Apolipoprotein L1	0.42	0.0437	0.6	0.1	0.2	0.0	0.4	0.0
25	P01019	53406	Angiotensinogen	0.42	0.0437	6.5	0.2	5.5	0.2	6.0	0.1
26	P52597	45985	Heterogeneous nuclear ribonucleoprotein F	0.41	0.0438	7.3	0.3	3.6	0.2	5.5	0.2
27	Q9H4A6	34075	Golgi phosphoprotein 3	0.41	0.0447	1.2	0.2	0.1	0.0	0.7	0.1
28	P14174	12639	Macrophage migration inhibitory factor	0.41	0.0463	6.0	0.3	4.1	0.1	5.1	0.1
29	Q9NWV4	18379	UPF0587 protein C1orf123	0.41	0.0462	3.6	0.2	0.1	0.0	1.8	0.1
30	Q9NRV9	21198	Heme-binding protein 1	0.41	0.0468	0.3	0.1	0.3	0.1	0.3	0.0
31	O00625	32207	Pirin	0.40	0.0498	4.6	0.2	2.9	0.2	3.8	0.1

Negatively-correlated proteins include molecular markers for normal aging, S100a8 and S100a9^37^, verifying the fact that olfactory decline is in parallel with aging (Fig. 3B and Table 4, No. **15** and **31**). The proteins which account for the largest percentage of negatively-correlated proteins are keratins (KRTs 2, 4, 6A, 9, 13, 14, 15), which protect epithelial cells (No. **49**, **8**, **40**, **52**, **21**, **2 and 4**). Although keratins are often regarded as major contaminants in proteomic experiments, the observed significant correlations with olfactory sensitivity suggest that they were derived from an olfaction-related origin, such as the olfactory mucus. Secretion of keratins into bodily fluid results from tissue injury^64^. Therefore, damage in the OC may cause increased amount of mucus keratins and a corresponding decline in olfactory performance. PANTHER classification identified a unique category of proteins correlated negatively with olfactory sensitivity (Fig. 3C). This category is immune system process and includes three proteins (BPI (No. **14**), Acetyl-coenzyme A synthetase (ACSS2, No. **45**) and Cysteine-rich protein 2 (CRIP2, No. **41**)). All of these proteins are related to inflammation. Murine BPI-fold containing protein has been known to be expressed peripherally only with inflammatory stimuli and to exhibit antimicrobial activity^65^. ACSS2 is involved in macrophage inflammatory cytokine responses by synthesizing metabolically available acetyl-coA^66^. Secretion of CRIP2, which regulates cytokine expression, is stimulated by lipopolysaccharide which induces inflammatory response^67^. Thus inflammation is associated tightly with olfactory decline, consistent with the results in Wang *et al*.^20^.

Positively-associated proteins include BPIFB4 and LCN15 (Fig. 3B and Table 5, No. **6 and 17**). These proteins are putative odorant binding proteins and enriched in olfactory tissue in RNA and protein levels, implying functional involvement with olfaction^16^. The observed positive correlations with olfactory sensitivity are consistent with this implication and suggest that BPIFB4 and LCN15 function in binding and recruiting PEA and n-butanol. Identification of calmodulin ((Fig. 3B and Table 5, No. **14**) seems to be also reasonable because a similar case was reported in saliva. Salivary calmodulin concentration of patients with taste and smell dysfunction is significantly lower than in normal subjects^68^. This observation indicates association between amount of secreted calmodulin and olfactory performance although no knowledge on origin of calmodulin in olfactory mucus impedes further speculation. Other identified proteins are involved in a variety of biological processes as shown in PANTHER classification but have currently little or no previous association with olfactory function. Taken together, these protein lists can be the basis for future studies to reveal molecular mechanisms of olfaction from the point of view of olfactory mucus function.

## Conclusion

This report, to our knowledge, is the first to apply the shotgun proteomic approach to analyze human olfactory mucus and its positional and age-related differences. This study revealed many more proteins than had been previously documented. Protein compositions from different nasal areas were more similar than expected, but regional differences in protein composition were identified. Age-related differences in mucus composition were also delineated, implicating peripheral causes of age-related olfactory decline. Thus, we believe that the present dataset provides the most comprehensive database of the olfactory mucus proteome and its age-related changes. We identified a list of mucus proteins which accounted for the observe variability in olfactory sensitivity of our participants. We acknowledge that the number of subjects for our analysis is relatively small given the fact that the sense of smell shows large individual variability^69–71^. Therefore, we cannot exclude the possibility that some mucus proteins which are critically involved in olfaction were overlooked. However, the present lists are reliable and informative because our analysis consistently identified proteins previously associated with olfactory function and inflammatory processes. This proteome dataset will support future research on the mechanism of human olfaction, causes of age-dependent decline and biomarkers for neurodegenerative disorders in our aging society.

## Methods

### Human subjects

Subjects were recruited by advertisement and compensated for their time and effort. All subjects provided informed consent prior to participation. The study protocol was approved by the University of Pennsylvania Institutional Review Board and the research was performed in accordance with all U.S. Department of Health and Human Services regulations for the protection of human subjects (45 CFR 46). Twelve young subjects between the ages of 21–40 and twelve older subjects between the ages of 65–80 were tested. The sexes were roughly the same in each group. Subjects did not have any subjective or objective evidence of sinonasal inflammation based on subject history and nasal endoscopy. Pregnant women and smokers were excluded from the study.

### Sensory analyses

In order to associate changes in the composition of olfactory mucus with functional outcomes, we conducted sensory tests to characterize olfactory function in our participants. We measured olfactory thresholds for two compounds (PEA and n-butanol) and odor identification for 16 odorants. Sensory thresholds were collected using Sniffin’ Sticks in a 3-alternative forced-choice procedure, as previously described^72,73^.

### Collection of mucus

The mucus collections were conducted at the Department of Otorhinolaryngology, Head and Neck Surgery of the Hospital of the University of Pennsylvania. Mucus samples were collected using the sponge region of an eye spear (bvi ultraClean^TM^, Beaver-Visitec International, USA) that is cut from the handle, soaked in double-distilled water and dried. Following instillation of topical lidocaine, the sponges were placed bilaterally under direct visualization using a 4 mm 30° endoscope (Karl Storz, El Segundo, CA) in the olfactory groove between the middle turbinate and superior nasal septum (the olfactory cleft: OC) and just deep to the nasal vestibule, between the inferior turbinate and inferior nasal septum (Anterior part: ANC), for 10 minutes. Then the sponge was removed from the nose and placed in a tube (1.5 ml) with a hole in the bottom poked by a 20 gauge needle. Another tube was placed below the punctured one and centrifuged at 10000 rpm for 2 min. The collected nasal secretions were frozen until use.

### Reagents

Procedures were performed with distilled water (Cat No. W6-500, Thermo, USA). Acetonitrile (ACN; Cat No. A955-500) and formic acid (FA; LS120-500), Pierce BCA Protein Assay kit were also obtained from Thermo. 1M Tris-HCl (Cat No. 312-90061), iodoacetamide (IAA; Cat No. 099-05591), trifluoroacetic acid (TFA; Cat No. 204-10771), lysyl endopeptidas (Lys-C; Cat No. 125-05061), trypsin from Porcine Pancreas (Cat No. 202-15951) were obtained from Wako (Japan). Dithiothreitol (DTT; Cat No. 43817) was obtained by Sigma-Aldrich (USA). 2.0 ml of Protein LoBind Tube was obtained from Eppendorf (German). GL-Tip SDB (Cat No. 7820-11200) and GL-TIP (Cat No. 7820-11201) were obtained by JL Science (Japan).

### Tryptic digestion

Protein concentrations were determined using the Pierce BCA Protein Assay by using bovine serum albumin (BSA) as standard. 50 μg protein equivalent of mucus samples in 90 μl of 100 mM Tris-HCl (pH.8.0) were transferred to 2.0 ml Protein LoBind tubes. The cysteines of mucus proteins were reduced by incubating with 5 μl of 15 mg/ml DTT in 100 mM Tris-HCl for 30 min at 37 °C. Alkylation was performed by adding 5 μl of IAA solution (100 mM Tris-HCl) to a final concentration of 50 mM and samples were incubated at 37 °C for 30 min. The samples were then mixed with 20 μl of 0.1 mg/ml Lys-C in 100 mM Tris-HCl for 3 h at 37 °C. Tryptic digestion was performed by adding 20 μl of 0.1 mg/ml trypsin from Porcine Pancreas in 100 mM Tris-HCl. The samples were cleaved overnight at 37 °C. On the following day, TFA was added to a final concentration of 0.5% and sample solutions were evaporated in a vacuum concentrator which comprised a centrifugal concentrator (VC-15SP; TAITEC, Japan) and a vacuum controller (FTP-10; Iwaki, Japan). The samples were reconstituted in solution A (5% ACN with 0.1% TFA in distilled water). 40 μg protein equivalent of each sample was desalted on GL-Tip SDB combined with GL-Tip GC (Top). The tips loaded with samples were washed with 20 μl of solution A and eluted with solution B (80% ACN with 0.1% TFA). After desalting, the samples were evaporated in the vacuum concentrator and dissolved in 50 μl of 2% ACN with 0.1% FA.

### LC-MS/MS

In order to determine the relative peptide concentrations of samples after tryptic digestion, 3 μl out of 50 μl sample solutions was analyzed using nanoAcquity UPLC (Waters). Estimation of relative peptides quantity was based on UV peak area at 214 nm in comparison with that from trypsine-digested BSA. Peptides were firstly loaded onto a trapping column (SymmetryC18TrapColumn 180 µm I.D., 20 mm, 5 µm, Waters, USA) using a buffer of 2% ACN with 0.1% FA for 3 min at a flow rate of 5 µl/min. The trapping column was set in-line with an analytical column: nanoAcquity UPLC BEH130 C18 (Waters, USA). The column temperature was maintained at 35 °C. The peptides were eluted with a linear gradient of 95% of phase A (0.1% FA in distilled water): 5% of phase B (0.1% FA with 80% ACN) to 50% of phase A: 50% of phase B over 30 min at a flow rate 0.4 µl/min. Then the percentage of phase B was held at 95% for 15 min, followed by decrease to 5% which was maintained for 15 min.

2.5 μg equivalent of peptide samples were analyzed using Ultimate 3000 RSLCnano System (Dionex) coupled to a TripleTOF 5600^+^ (AB SCIEX, Japan). After injection, the peptides were trapped on Acclaim PepMap 100 Nano Trap C18, nanoViper (100 µm I.D., 20 mm, 3 µm, Thermo, USA) under a buffer of 0.1% TFA in distilled water for 5 min at a flow rate of 5 µl/min. The eluted peptides were then applied onto an analytical column, MonoCap C18 High Resolution 2000 (JL Science, Japan) maintained at 40 °C. The peptides were eluted with a linear gradient of 95% of phase A (0.1% FA in distilled water): 5% of phase B (0.1% FA with 80% ACN) to 50% of phase A: 50% of phase B over 365 min at a flow rate 0.5 µl/min. Then the percentage of phase B was held at 95% for 55 min. The effluent introduced directly into the integrated nano-electrospray ionization source operating in positive ion mode. The TOF-MS mass range was set to m/z 350–1250. MS/MS scan was operated in data dependent scan mode performed to acquire fragmentation spectra within the range of m/z 100–2000 at mass tolerance of 50 mDa and at accumulation time of 100 msec. Fragmentation of the peptides was by Collision Energy of 37 V with collision energy spread of 15 V. Dynamic exclusion of m/z values to prevent repeated fragmentation of the same peptide was applied with an exclusion time of 15 s.

### Database search

MS/MS data acquired with LC-MS/MS was performed using the Mascot Daemon software (version 2.4, Matrix Science, UK). Data were searched against human entries in the SwissProt (2012). Carbamidomethylation of cysteine was set as a static modification and oxidation of methionine was set as a variable modification. The mass tolerance was set to 20 ppm and 0.1 Da for precursor and fragment, respectively. The false discovery rate (FDR) by searching decoy databases was estimated to be 1.0%. The abundance of identified proteins was estimated by calculating emPAI, which is one of semi-quantitative approaches widely used in comparative proteomic studies^21-23^. The emPAI is an exponential form of PAI (the number of detected peptides divided by the number of observable peptides per protein).

### Statistical analyses

Results are expressed as mean ± standard deviation. Statistical analysis was conducted using Graphpad prism7. For the results of the shotgun proteome, multiple *t*-tests were conducted in a following condition. Discovery determined using the two-stage linear step-up procedure of Benjamini, Krieger and Yekutieli, with desired FDR (Q) = 5%. Computations assume that all rows are sample from populations with the same scatter. Quantitative differences of each protein between two nasal regions or two age groups were considered to be statistically significant for FDR adjusted *P* < 0.05, and for concentration ratio of a protein from two groups >1.3. Correlations were evaluated using the Spearman rank correlation coefficient method for the data presented in Tables 4 and 5. The statistical significance of the difference in averaged threshold among two aged groups was analyzed by student’s t-test. Normal distributions were verified by Shapiro-Wilk test with a significance level of 5%.

## Electronic supplementary material

Supplementary Information

## Data Availability

Data generated or analyzed during this study are included in this published article (and its Supplementary information files) or are available from the corresponding authors on reasonable request.
