# Supplementary material for: The human olfactory cleft mucus proteome and its age-related changes
Source: Sci Rep. 2018 Nov 21;8:17170. doi: 10.1038/s41598-018-35102-2 (PMC6249231; doi:10.1038/s41598-018-35102-2)
Supplement: Supplementary file 1 — Supplementary Information [file 41598_2018_35102_MOESM1_ESM.pdf]

Supplementary Materials

The human olfactory cleft mucus proteome and its age-related changes

Keiichi Yoshikawa, Hong Wang, Cristina Jaen, Mai Haneoka, Naoko Saito,

Junji Nakamura, Nithin D. Adappa, Noam A. Cohen, Pamela Dalton

Supplementary Table S1. Subject information
Supplementary Table S2. OC and ANC proteome from young subjects.
Supplementary Table S3. OC and ANC proteome from elderly subjects.
Supplementary Table S4. Young OC-enriched proteins.
Supplementary Table S5. Elderly OC-enriched proteins.
Supplementary Table S6. Age-associated OC proteins.
Supplementary Table S7. Proteins associated with olfaction.
Supplementary Figure S1. Comparison between shotgun proteomics and anti-body based multiplex assay.
Supplementary Figure S2. Regional difference in levels of lipocalins and BPI-fold containing proteins.
Supplementary Figure S3. Age-related change in GST gene family in proteome analysis.
Supplementary Figure S4. Age-related change in S100a8 and S100a9 gene family in proteome analysis.

Table S1. Subject information

ID	Age	Gender	OC protein (mg/ml)	AP protein (mg/ml)	Combined PEA and Butanol threshold
Y1	21	F	20.4	23.5	7.625
Y2	30	F	17.2	16.1	10.5
Y3	31	M	23.9	23.8	7
Y4	27	M	21.4	16.8	8.958
Y5	29	F	18.9	12.7	9.375
Y6	25	F	23.6	15.3	10.875
Y7	26	F	17.1	18.1	12.625
Y8	26	M	9.0	13.0	10.125
Y9	19	F	13.7	16.9	11.875
Y10	38	M	18.5	8.0	7.625
Y11	39	M	15.7	14.2	10.25
Y12	28	M	8.2	4.0	12.125
E1	74	M	24.4	8.8	9.625
E2	72	M	23.0	20.1	4.75
E3	69	F	11.2	11.1	9.5
E4	72	F	15.2	6.9	9.375
E5	67	F	19.2	18.9	8
E6	85	F	14.6	15.3	5.125
E7	79	F	16.9	15.9	6
E8	84	F	15.9	13.0	7.75
E9	67	F	4.0	3.1	7.875
E10	76	F	10.4	20.8	7.125
E11	74	F	18.9	15.3	8.625
E12	69	F	10.0	5.7	6.875

Table S2 OC and ANC proteome from young subjects

emPAI/1 uL mucus		OC												ANC												Average		Ajusted *P* value	Fold difference
Accession	Description	Y1	Y2	Y3	Y4	Y5	Y6	Y7	Y8	Y9	Y10	Y11	Y12	Y1	Y2	Y3	Y4	Y5	Y6	Y7	Y8	Y9	Y10	Y11	Y12	OC	ANC	Multiple *t*-test	OC/ANC
P63208	S-phase kinase-associated protein 1 OS=Homo sapiens GN=SKP1 PE=1 SV=2	7.95	21.79	40.16	35.92	17.54	64.27	15.90	1.63	2.47	50.39	33.97	10.36	9.17	10.28	30.29	3.03	2.28	5.98	7.05	0.00	0.00	3.12	9.08	5.08	25.19	7.11	<0.000000000000001	3.5
P80723	Brain acid soluble protein 1 OS=Homo sapiens GN=BASP1 PE=1 SV=2	15.09	16.98	38.96	21.17	24.32	47.73	27.87	2.89	6.98	65.95	25.63	2.61	7.53	15.90	12.16	5.38	6.45	11.35	0.00	4.15	12.49	7.91	7.24	0.00	24.68	7.55	<0.000000000000001	3.3
Q71U36	Tubulin alpha-1A chain OS=Homo sapiens GN=TUBA1A PE=1 SV=1	20.39	26.93	65.50	44.90	39.60	54.35	32.66	12.73	13.70	46.50	43.09	14.11	0.00	22.64	33.63	21.20	12.65	24.09	0.00	0.00	0.00	13.82	27.10	0.00	34.54	12.93	<0.000000000000001	2.7
P00441	Superoxide dismutase [Cu-Zn] OS=Homo sapiens GN=SOD1 PE=1 SV=2	32.22	36.37	66.45	59.44	39.97	50.10	47.53	25.11	15.61	29.27	43.72	12.89	10.82	25.37	37.68	19.18	26.82	24.24	13.93	5.97	13.00	9.11	30.08	14.29	38.22	19.21	<0.000000000000001	2.0
Q9BW30	Tubulin polymerization-promoting protein family member 3 OS=Homo sapiens GN=TPPP3 PE=1 SV=1	25.49	36.37	63.83	35.28	50.34	78.45	36.25	24.11	17.12	61.51	33.34	13.46	14.82	42.87	39.35	27.76	26.82	32.53	6.87	0.00	6.41	13.18	23.41	6.60	39.63	20.05	<0.000000000000001	2.0
Q99497	Protein DJ-1 OS=Homo sapiens GN=PARK7 PE=1 SV=2	31.20	33.45	58.57	52.38	28.85	71.60	26.16	17.61	26.71	56.13	30.67	12.48	27.52	9.47	20.51	25.74	14.80	13.19	27.67	4.67	25.83	24.20	27.67	6.12	37.15	18.95	<0.000000000000001	2.0
P09211	Glutathione S-transferase P OS=Homo sapiens GN=GSTP1 PE=1 SV=2	38.74	56.95	110.68	116.09	102.38	128.31	39.50	35.49	38.07	72.81	72.82	32.06	36.22	44.64	110.42	55.86	35.18	35.44	41.78	19.99	38.99	26.52	47.11	13.29	70.33	42.12	<0.000000000000001	1.7
P00326	Alcohol dehydrogenase 1C OS=Homo sapiens GN=ADH1C PE=1 SV=2	27.73	23.33	99.20	53.24	70.90	80.58	64.29	17.88	27.12	46.13	65.27	20.31	46.57	24.89	66.06	33.31	25.05	27.00	35.81	13.24	22.96	17.73	39.31	11.09	49.66	30.25	<0.000000000000001	1.6
P05387	60S acidic ribosomal protein P2 OS=Homo sapiens GN=RPLP2 PE=1 SV=1	104.20	45.29	63.11	38.70	70.14	62.38	63.61	10.57	24.79	48.91	58.50	21.53	120.19	42.39	62.96	19.68	14.80	4.60	21.16	3.89	19.75	9.35	37.46	27.70	50.98	32.00	<0.000000000000001	1.6
P30044	Peroxiredoxin-5, mitochondrial OS=Homo sapiens GN=PRDX5 PE=1 SV=4	33.85	35.34	87.49	65.21	47.52	103.03	35.22	27.54	22.74	67.80	68.57	29.85	39.04	40.46	72.74	42.40	31.89	38.66	45.58	9.74	28.02	20.13	35.76	10.09	52.01	34.54	<0.000000000000001	1.5
P00352	Retinal dehydrogenase 1 OS=Homo sapiens GN=ALDH1A1 PE=1 SV=2	70.76	79.42	136.26	130.20	100.31	102.08	73.87	27.00	44.10	85.77	78.16	35.24	70.33	51.70	135.94	58.38	37.83	45.87	58.24	25.84	33.59	36.98	98.76	17.29	80.26	55.90	<0.000000000000001	1.4
P02788	Lactotransferrin OS=Homo sapiens GN=LTF PE=1 SV=6	77.29	31.56	7.65	16.03	32.62	26.94	37.62	35.94	28.49	8.15	20.76	34.18	166.29	27.78	21.46	17.83	50.36	61.06	61.86	32.07	60.77	19.73	41.43	27.02	29.77	48.97	<0.000000000000001	0.6
P69905	Hemoglobin subunit alpha OS=Homo sapiens GN=HBA1 PE=1 SV=2	4.49	8.41	11.71	10.48	32.43	40.64	86.52	4.43	0.00	0.00	3.46	0.00	11.52	7.87	0.00	3.70	81.11	191.94	115.94	83.22	29.03	1.76	0.00	0.00	16.88	43.84	<0.000000000000001	0.4
P68871	Hemoglobin subunit beta OS=Homo sapiens GN=HBB PE=1 SV=2	15.70	13.21	11.24	16.46	14.52	37.81	61.73	4.24	6.44	3.89	3.30	1.71	18.11	3.37	0.00	0.00	72.88	88.37	130.04	175.52	47.43	9.19	2.98	0.00	15.85	45.66	<0.000000000000001	0.3
P05109	Protein S100-A8 OS=Homo sapiens GN=S100A8 PE=1 SV=1	86.26	22.13	0.00	6.84	6.03	0.00	5.47	26.82	4.38	0.00	0.00	16.40	138.54	67.92	17.65	33.82	37.58	45.57	36.35	76.47	50.13	10.30	10.50	32.30	14.53	46.43	<0.000000000000001	0.3
P02042	Hemoglobin subunit delta OS=Homo sapiens GN=HBD PE=1 SV=2	0.00	0.00	0.00	0.00	0.00	0.00	36.25	0.00	0.00	0.00	0.00	0.00	0.00	0.00	0.00	0.00	57.19	42.65	64.57	73.74	0.00	0.00	0.00	0.84	3.02	19.92	<0.000000000000001	0.2
P04083	Annexin A1 OS=Homo sapiens GN=ANXA1 PE=1 SV=2	54.85	32.42	108.53	31.00	50.72	97.12	32.32	14.99	10.55	39.46	33.50	15.42	34.10	26.65	79.66	27.93	18.35	51.24	19.53	8.18	6.58	11.58	26.82	12.01	43.41	26.89	7.1E-14	1.6
P60174	Triosephosphate isomerase OS=Homo sapiens GN=TPI1 PE=1 SV=3	56.08	47.17	140.80	58.79	76.93	64.98	53.86	13.55	28.21	66.51	64.17	48.04	55.98	44.15	110.42	53.00	45.42	27.00	49.74	16.23	25.32	36.98	44.70	20.93	59.92	44.16	1.507E-12	1.4
A0M8Q6	Ig lambda-7 chain C region OS=Homo sapiens GN=IGLC7 PE=1 SV=2	0.00	0.00	0.00	0.00	0.00	0.00	0.00	34.77	0.00	0.00	0.00	0.00	64.21	0.00	0.00	0.00	34.54	0.00	49.38	35.44	31.56	0.00	0.00	0.00	2.90	17.93	2.3006E-11	0.2
P04075	Fructose-bisphosphate aldolase A OS=Homo sapiens GN=ALDOA PE=1 SV=2	41.80	49.40	61.67	68.42	90.13	43.01	44.12	20.86	28.08	42.79	68.25	23.49	54.33	25.69	76.32	34.49	25.94	35.44	37.08	13.63	38.99	16.38	32.78	11.53	48.50	33.55	2.9121E-11	1.4
Q13938	Calcyphosin OS=Homo sapiens GN=CAPS PE=1 SV=1	46.09	47.69	66.45	59.44	76.93	96.41	24.45	30.52	38.07	51.50	53.16	18.43	53.16	65.51	80.61	46.77	35.18	42.65	19.71	7.27	18.40	18.05	39.45	5.72	50.76	36.04	6.4082E-11	1.4
P51858	Hepatoma-derived growth factor OS=Homo sapiens GN=HDGF PE=1 SV=1	12.24	13.72	37.05	27.15	29.23	36.63	21.72	11.47	8.22	48.54	34.91	10.36	6.12	9.63	36.97	7.07	7.59	12.27	10.85	1.56	4.39	6.39	14.47	1.68	24.27	9.92	2.26971E-10	2.4
P50395	Rab GDP dissociation inhibitor beta OS=Homo sapiens GN=GDI2 PE=1 SV=2	35.07	21.44	59.52	63.28	46.95	69.95	26.67	7.86	21.37	46.13	27.05	18.60	26.34	27.62	45.31	26.25	14.17	23.93	37.62	8.44	18.90	16.62	24.41	4.48	36.99	22.84	4.43643E-10	1.6
P30050	60S ribosomal protein L12 OS=Homo sapiens GN=RPL12 PE=1 SV=1	13.87	16.98	55.70	29.08	34.13	42.77	30.95	1.72	13.56	25.20	21.39	8.08	23.29	21.84	32.44	11.44	5.19	0.00	17.91	0.00	0.00	3.28	9.65	5.44	24.45	10.87	2.97615E-09	2.2
Q9Y3Z3	SAM domain and HD domain-containing protein 1 OS=Homo sapiens GN=SAMHD1 PE=1 SV=2	6.12	24.19	38.96	21.81	19.23	35.92	18.98	3.25	4.93	15.75	20.60	5.06	2.12	11.24	14.79	4.21	3.80	5.52	4.52	0.00	0.00	6.79	5.11	1.20	17.90	4.94	2.17366E-08	3.6
Q13885	Tubulin beta-2A chain OS=Homo sapiens GN=TUBB2A PE=1 SV=1	18.15	11.32	42.07	33.99	24.13	37.57	13.17	0.00	10.55	23.71	17.93	6.28	10.82	0.00	27.19	11.10	0.00	0.00	10.13	0.00	0.00	10.22	12.63	2.64	19.91	7.06	2.99764E-08	2.8
P41567	Eukaryotic translation initiation factor 1 OS=Homo sapiens GN=EIF1 PE=1 SV=1	21.00	38.60	24.62	33.57	11.31	24.34	17.61	5.42	14.11	29.09	24.69	12.81	0.00	9.63	24.57	10.09	3.42	9.21	10.85	3.51	0.00	4.79	22.28	6.28	21.43	8.72	4.3951E-08	2.5
P14550	Alcohol dehydrogenase [NADP(+)] OS=Homo sapiens GN=AKR1A1 PE=1 SV=3	32.22	23.33	70.76	44.04	44.12	48.68	23.25	12.28	16.02	43.35	46.55	16.80	19.52	25.37	43.17	30.45	17.21	20.87	32.74	10.78	14.01	23.64	29.23	6.32	35.12	22.78	1.33484E-07	1.5
P62258	14-3-3 protein epsilon OS=Homo sapiens GN=YWHAE PE=1 SV=1	27.73	45.29	54.26	48.53	36.39	53.64	38.81	20.50	22.32	35.75	25.63	15.74	45.39	17.98	32.44	27.42	24.42	20.87	24.60	9.22	22.96	13.02	32.21	6.52	35.38	23.09	1.48182E-07	1.5
P68371	Tubulin beta-4B chain OS=Homo sapiens GN=TUBB4B PE=1 SV=1	20.60	17.33	51.16	37.63	24.13	37.57	13.17	5.96	12.19	26.49	20.13	8.24	0.00	16.22	34.11	14.97	8.35	15.50	10.13	0.00	0.00	12.70	14.33	3.08	22.88	10.78	2.57307E-07	2.1
P37802	Transgelin-2 OS=Homo sapiens GN=TAGLN2 PE=1 SV=3	26.51	22.30	39.20	27.79	38.28	30.72	51.30	6.68	13.70	18.53	11.64	20.23	7.53	16.06	17.65	16.82	16.45	15.34	18.09	9.61	12.49	13.10	14.19	8.13	25.57	13.79	6.33544E-07	1.9
P16050	Arachidonate 15-lipoxygenase OS=Homo sapiens GN=ALOX15 PE=1 SV=3	21.62	15.27	42.55	35.49	38.09	31.66	21.20	6.68	19.72	28.72	21.07	12.64	24.93	11.88	23.37	13.63	19.61	11.35	5.25	3.12	15.02	6.47	13.91	5.36	24.56	12.83	7.11729E-07	1.9
P00325	Alcohol dehydrogenase 1B OS=Homo sapiens GN=ADH1B PE=1 SV=2	0.00	14.92	28.21	18.60	25.64	27.88	23.25	0.00	10.00	25.20	18.56	7.10	0.00	0.00	24.33	0.00	0.00	13.35	0.00	0.00	0.00	6.95	10.36	4.08	16.61	4.92	7.8267E-07	3.4
P30086	Phosphatidylethanolamine-binding protein 1 OS=Homo sapiens GN=PEBP1 PE=1 SV=3	36.71	38.42	65.74	58.79	91.26	79.16	30.78	24.83	24.65	50.95	43.25	22.43	33.40	53.79	115.43	37.69	28.34	34.37	32.56	10.39	18.40	17.89	39.02	5.68	47.25	35.58	8.12736E-07	1.3
P49773	Histidine triad nucleotide-binding protein 1 OS=Homo sapiens GN=HINT1 PE=1 SV=2	18.96	15.95	47.81	19.88	37.71	13.00	9.40	4.97	7.53	26.12	31.45	7.59	5.88	8.83	13.12	4.21	6.96	14.27	9.95	3.25	9.28	7.43	13.20	8.01	20.03	8.70	2.05801E-06	2.3
P31949	Protein S100-A11 OS=Homo sapiens GN=S100A11 PE=1 SV=2	35.89	11.32	61.20	37.63	92.77	116.26	30.09	23.12	35.06	32.61	27.68	20.88	60.21	57.64	61.06	19.18	22.27	27.00	46.30	22.85	11.14	20.45	36.33	4.56	43.71	32.42	2.1681E-06	1.3
P61204	ADP-ribosylation factor 3 OS=Homo sapiens GN=ARF3 PE=1 SV=2	23.04	14.24	35.14	39.98	21.31	26.70	19.32	5.15	11.37	15.38	8.96	19.09	19.52	2.57	19.80	9.59	7.21	2.45	10.31	4.54	5.91	6.63	8.09	7.48	19.97	8.68	2.1681E-06	2.3
P29508	Serpin B3 OS=Homo sapiens GN=SERPINB3 PE=1 SV=2	70.96	54.38	75.78	89.15	71.84	56.00	49.24	13.82	35.75	22.05	54.73	56.53	55.74	46.24	68.69	58.55	36.44	44.19	52.09	19.86	40.00	13.74	40.87	41.35	54.19	43.15	4.27355E-06	1.3
P02652	Apolipoprotein A-II OS=Homo sapiens GN=APOA2 PE=1 SV=1	38.54	5.15	45.18	40.41	35.64	44.66	32.32	11.02	37.94	35.01	29.72	31.98	44.45	4.82	29.10	20.53	23.91	18.72	34.18	24.54	31.90	5.59	9.93	7.56	32.30	21.27	4.28961E-06	1.5
P63104	14-3-3 protein zeta/delta OS=Homo sapiens GN=YWHAZ PE=1 SV=1	50.16	35.85	92.27	94.93	62.98	68.05	57.11	26.01	39.44	61.88	52.53	23.49	67.74	33.56	68.69	56.19	36.44	51.24	60.41	31.94	41.52	19.65	47.39	17.77	55.39	44.38	4.3579E-06	1.2
P06702	Protein S100-A9 OS=Homo sapiens GN=S100A9 PE=1 SV=1	43.43	16.81	0.00	12.40	0.00	0.00	9.92	5.24	0.00	4.82	9.12	12.15	69.15	34.20	13.83	9.76	12.40	8.90	10.49	51.28	16.54	4.63	8.23	5.96	9.49	20.45	4.84543E-06	0.5
O60739	Eukaryotic translation initiation factor 1b OS=Homo sapiens GN=EIF1B PE=1 SV=2	0.00	0.00	24.62	33.57	0.00	24.34	0.00	0.00	0.00	29.09	24.69	0.00	0.00	0.00	0.00	0.00	0.00	0.00	0.00	0.00	0.00	4.79	0.00	0.00	11.36	0.40	4.84543E-06	28.4
O00299	Chloride intracellular channel protein 1 OS=Homo sapiens GN=CLIC1 PE=1 SV=4	52.41	26.07	83.67	86.59	48.46	51.51	43.94	16.53	41.22	55.76	40.42	33.04	29.40	35.00	96.59	50.64	19.23	46.18	54.44	16.23	30.89	17.41	42.71	10.29	48.30	37.42	5.76331E-06	1.3
P08263	Glutathione S-transferase A1 OS=Homo sapiens GN=GSTA1 PE=1 SV=3	8.97	41.17	39.68	42.97	31.30	67.11	28.38	18.15	18.63	37.24	44.66	8.81	14.82	17.34	67.73	22.88	21.00	36.82	24.60	17.66	4.73	6.71	19.30	4.32	32.26	21.49	7.80009E-06	1.5
P07355	Annexin A2 OS=Homo sapiens GN=ANXA2 PE=1 SV=2	12.85	10.81	30.12	16.46	31.49	34.50	13.17	5.69	0.00	20.19	17.14	10.28	0.00	12.36	15.03	10.60	9.74	7.67	0.00	0.00	0.00	0.00	15.47	3.68	16.89	6.21	9.54917E-06	2.7
P07437	Tubulin beta chain OS=Homo sapiens GN=TUBB PE=1 SV=2	26.31	13.38	46.85	37.84	24.32	41.83	15.22	4.24	12.19	26.68	14.00	5.47	18.35	16.38	27.43	13.12	5.95	11.97	12.12	3.76	3.54	10.30	14.47	3.56	22.36	11.75	1.10756E-05	1.9
P10599	Thioredoxin OS=Homo sapiens GN=TXN PE=1 SV=3	5.91	29.85	41.59	37.20	32.81	41.12	19.32	10.20	9.04	32.24	55.52	20.56	15.52	18.14	41.50	4.88	14.30	17.34	31.47	8.57	19.07	9.03	24.69	4.52	27.95	17.42	1.36517E-05	1.6
Q9NQR4	Omega-amidase NIT2 OS=Homo sapiens GN=NIT2 PE=1 SV=1	7.34	6.18	30.12	26.94	19.61	35.45	8.55	3.25	1.51	15.56	19.82	1.88	0.00	5.78	5.49	8.41	0.00	5.52	4.16	0.00	1.86	6.71	11.92	1.44	14.68	4.27	1.81688E-05	3.4
P31150	Rab GDP dissociation inhibitor alpha OS=Homo sapiens GN=GDI1 PE=1 SV=2	17.74	14.92	29.88	18.60	23.57	33.08	11.11	3.34	7.53	18.34	19.66	7.10	6.59	10.44	23.61	0.00	4.68	8.44	9.95	0.00	0.00	4.39	10.64	1.80	17.07	6.71	2.02175E-05	2.5
Q99417	C-Myc-binding protein OS=Homo sapiens GN=MYCBP PE=1 SV=3	0.00	11.32	27.01	24.16	5.47	26.70	11.29	0.00	0.00	32.24	27.36	2.37	0.00	0.00	26.95	0.00	0.00	4.45	0.00	0.00	0.00	9.03	4.12	0.00	13.99	3.71	2.43502E-05	3.8
Q13228	Selenium-binding protein 1 OS=Homo sapiens GN=SELENBP1 PE=1 SV=2	21.62	22.81	38.96	28.44	19.99	46.31	14.19	6.50	14.52	22.05	28.15	8.65	14.58	15.09	28.38	13.96	9.11	14.42	17.00	1.69	4.56	10.62	23.13	1.76	22.68	12.86	7.52799E-05	1.8
P40394	Alcohol dehydrogenase class 4 mu/sigma chain OS=Homo sapiens GN=ADH7 PE=1 SV=2	14.07	16.64	30.84	55.37	35.26	44.19	22.06	16.89	17.67	20.75	55.04	13.54	16.23	13.33	35.06	24.73	14.17	25.47	26.59	4.54	9.62	14.94	36.75	5.88	28.53	18.94	0.000132414	1.5
P14618	Pyruvate kinase isozymes M1/M2 OS=Homo sapiens GN=PKM2 PE=1 SV=4	47.51	43.23	65.02	72.26	55.25	55.06	34.03	19.51	23.01	40.02	42.78	27.57	43.04	24.73	51.52	42.40	29.48	41.73	45.58	16.49	36.46	20.13	47.96	10.89	43.77	34.20	0.000134418	1.3
P31946	14-3-3 protein beta/alpha OS=Homo sapiens GN=YWHAB PE=1 SV=3	24.27	41.51	49.24	51.74	71.27	57.18	48.22	18.60	23.69	44.83	44.35	26.67	33.87	33.08	57.72	47.45	35.68	37.13	37.26	18.69	24.31	16.46	34.34	11.29	41.80	32.27	0.000147252	1.3
P06703	Protein S100-A6 OS=Homo sapiens GN=S100A6 PE=1 SV=1	28.55	24.02	53.07	47.46	26.40	52.46	13.51	7.13	19.17	14.64	22.02	18.11	32.93	12.68	52.95	13.29	28.09	12.12	14.29	10.26	5.74	6.31	19.87	5.60	27.21	17.84	0.000209708	1.5
P30043	Flavin reductase (NADPH) OS=Homo sapiens GN=BLVRB PE=1 SV=3	27.12	8.92	24.38	21.81	25.08	31.43	13.00	4.70	4.38	18.90	20.92	8.32	3.53	8.35	24.33	8.75	4.05	7.98	9.41	6.75	5.40	6.07	10.78	2.08	17.41	8.12	0.000247046	2.1
P07900	Heat shock protein HSP 90-alpha OS=Homo sapiens GN=HSP90AA1 PE=1 SV=5	19.98	20.76	39.92	29.72	27.91	34.97	32.15	11.74	14.38	25.75	23.28	11.34	23.05	14.45	31.00	19.01	14.30	17.34	13.75	4.54	15.19	8.39	17.17	3.60	24.32	15.15	0.000316484	1.6
Q96KP4	Cytosolic non-specific dipeptidase OS=Homo sapiens GN=CNDP2 PE=1 SV=2	21.62	16.12	34.90	28.22	19.99	34.50	20.18	7.41	9.86	21.86	22.96	6.69	14.58	18.95	22.42	13.80	7.84	11.05	17.00	1.69	3.38	6.55	16.74	3.28	20.36	11.44	0.000558649	1.8
Q9Y265	RuvB-like 1 OS=Homo sapiens GN=RUVBL1 PE=1 SV=1	9.38	17.15	23.90	16.46	18.86	30.01	11.29	2.62	2.88	16.30	17.93	3.02	1.65	10.60	20.99	2.19	2.66	5.68	0.00	0.00	0.00	7.03	10.93	1.48	14.15	5.27	0.000596362	2.7
P62888	60S ribosomal protein L30 OS=Homo sapiens GN=RPL30 PE=1 SV=2	12.24	4.46	14.34	5.56	29.23	36.63	10.26	2.35	3.56	18.90	16.04	4.89	14.11	4.17	14.31	4.37	0.00	0.00	4.70	0.00	0.00	4.79	3.69	2.40	13.20	4.38	0.000664442	3.0
P36405	ADP-ribosylation factor-like protein 3 OS=Homo sapiens GN=ARL3 PE=1 SV=2	23.04	9.78	8.37	24.16	21.31	19.61	14.19	7.50	4.79	15.38	29.41	4.65	3.76	9.15	13.59	5.89	7.21	5.37	2.89	0.00	0.00	6.63	20.86	1.40	15.18	6.40	0.000708113	2.4
P62820	Ras-related protein Rab-1A OS=Homo sapiens GN=RAB1A PE=1 SV=3	19.98	8.75	7.41	27.15	23.95	23.16	21.72	0.00	6.98	23.53	25.16	5.95	0.00	0.00	17.41	0.00	12.40	0.00	35.99	0.00	0.00	10.14	10.36	2.04	16.15	7.36	0.000708113	2.2
P31025	Lipocalin-1 OS=Homo sapiens GN=LCN1 PE=1 SV=1	7.75	20.93	21.51	0.00	11.69	8.98	15.39	0.00	0.00	0.00	5.98	21.13	112.90	6.10	9.06	6.39	15.44	9.51	29.12	4.93	6.41	3.04	5.39	10.37	9.45	18.22	0.000709884	0.5
P06733	Alpha-enolase OS=Homo sapiens GN=ENO1 PE=1 SV=2	39.36	33.11	41.59	66.28	58.45	50.33	29.75	19.24	35.34	35.75	44.51	19.17	36.69	45.44	50.80	32.47	24.42	29.61	34.91	30.51	26.33	17.01	30.22	9.41	39.41	30.65	0.000730465	1.3
P17987	T-complex protein 1 subunit alpha OS=Homo sapiens GN=TCP1 PE=1 SV=1	22.23	18.70	34.66	23.30	20.55	28.36	11.80	2.71	1.51	22.23	15.41	5.63	8.70	15.73	20.99	13.12	1.39	9.36	11.03	0.00	0.00	7.03	13.91	2.44	17.26	8.64	0.000976602	2.0
P23396	40S ribosomal protein S3 OS=Homo sapiens GN=RPS3 PE=1 SV=2	16.31	7.20	14.34	17.10	29.23	30.01	21.72	9.21	13.97	23.53	24.38	8.32	18.82	12.84	19.08	7.07	7.59	3.99	14.47	1.56	4.39	6.39	14.47	1.68	17.94	9.36	0.00103799	1.9
P30041	Peroxiredoxin-6 OS=Homo sapiens GN=PRDX6 PE=1 SV=3	17.74	35.68	41.12	53.24	26.40	58.84	23.94	18.78	19.17	25.94	32.71	9.14	15.29	33.40	70.60	23.55	11.01	17.18	11.76	3.64	7.76	13.74	49.38	2.60	30.22	21.66	0.001056364	1.4
P08758	Annexin A5 OS=Homo sapiens GN=ANXA5 PE=1 SV=2	6.12	0.00	28.92	4.06	26.59	44.42	1.54	0.81	0.00	10.19	6.61	2.45	0.00	1.45	20.27	1.51	0.00	1.38	0.00	0.00	0.00	0.00	4.26	0.76	10.98	2.47	0.00118298	4.4
P27348	14-3-3 protein theta OS=Homo sapiens GN=YWHAQ PE=1 SV=1	19.58	29.85	28.45	37.20	32.81	57.42	20.35	13.09	23.83	45.02	32.55	23.17	40.93	23.28	34.58	20.02	22.02	22.25	21.52	12.46	16.20	19.41	20.58	9.73	30.28	21.91	0.001567337	1.4
P02144	Myoglobin OS=Homo sapiens GN=MB PE=1 SV=2	4.08	18.01	16.97	15.18	8.11	24.81	3.42	3.88	2.74	19.45	22.80	3.51	0.00	3.21	16.93	3.36	2.53	0.00	0.00	0.00	0.00	5.67	10.07	0.80	11.91	3.55	0.001567337	3.4
O75556	Mammaglobin-B OS=Homo sapiens GN=SCGB2A1 PE=1 SV=1	6.32	5.32	7.41	0.00	0.00	0.00	21.37	6.50	4.25	0.00	4.88	15.91	95.73	4.98	29.81	5.22	3.92	4.76	13.02	4.02	0.00	0.00	4.40	5.00	6.00	14.24	0.001990447	0.4
P08238	Heat shock protein HSP 90-beta OS=Homo sapiens GN=HSP90AB1 PE=1 SV=4	23.65	22.81	31.79	28.44	36.58	38.28	33.17	13.73	16.98	28.16	23.90	13.21	25.40	18.63	31.72	16.82	16.83	19.02	22.43	7.66	15.53	12.14	21.57	5.68	25.89	17.79	0.002590258	1.5
Q9NQ48	Leucine zipper transcription factor-like protein 1 OS=Homo sapiens GN=LZTFL1 PE=1 SV=1	0.00	12.52	25.82	19.24	13.76	25.52	9.92	2.89	2.74	16.67	16.98	4.73	0.00	11.72	13.83	0.00	2.53	1.53	0.00	0.00	0.00	8.63	15.33	0.00	12.57	4.46	0.002590258	2.8
P62314	Small nuclear ribonucleoprotein Sm D1 OS=Homo sapiens GN=SNRPD1 PE=1 SV=1	0.00	4.46	13.86	20.95	10.94	23.16	4.45	0.00	0.00	10.75	9.12	4.73	0.00	0.00	0.00	0.00	0.00	0.00	0.00	0.00	0.00	2.08	3.69	0.00	8.53	0.48	0.002817703	17.8
O60701	UDP-glucose 6-dehydrogenase OS=Homo sapiens GN=UGDH PE=1 SV=1	11.83	13.38	26.53	21.17	23.19	23.39	16.93	10.02	6.71	16.30	23.75	8.08	4.47	10.92	20.99	9.76	8.60	8.90	10.49	0.78	5.57	7.03	15.75	2.72	16.77	8.83	0.003497315	1.9
P10768	S-formylglutathione hydrolase OS=Homo sapiens GN=ESD PE=1 SV=2	7.14	8.41	34.18	36.13	9.24	19.14	17.10	4.43	1.37	9.08	15.73	5.22	0.00	7.87	23.85	3.70	2.78	3.38	3.98	0.00	0.00	7.99	17.17	1.96	13.93	6.06	0.003955297	2.3
Q9NP55	BPI fold-containing family A member 1 OS=Homo sapiens GN=BPIFA1 PE=1 SV=1	45.27	38.08	44.70	33.14	29.23	44.19	26.50	16.89	25.61	23.53	24.38	25.04	36.46	30.02	36.97	31.46	16.07	19.49	22.97	28.82	31.56	10.14	14.47	5.08	31.38	23.63	0.004897989	1.3
P62937	Peptidyl-prolyl cis-trans isomerase A OS=Homo sapiens GN=PPIA PE=1 SV=2	36.09	22.99	69.32	48.96	43.18	41.83	30.27	12.10	13.29	24.82	45.61	14.44	31.52	21.52	54.62	29.78	16.96	20.56	41.42	12.59	16.37	14.14	41.15	9.17	33.57	25.82	0.004897989	1.3
P54652	Heat shock-related 70 kDa protein 2 OS=Homo sapiens GN=HSPA2 PE=1 SV=1	10.40	0.00	10.52	13.90	15.27	23.16	0.00	4.61	0.00	9.45	9.12	0.00	0.00	0.00	0.00	0.00	0.00	0.00	0.00	0.00	0.00	3.51	0.00	0.00	8.04	0.29	0.004940997	27.4
P22626	Heterogeneous nuclear ribonucleoproteins A2/B1 OS=Homo sapiens GN=HNRNPA2B1 PE=1 SV=2	10.81	13.89	19.36	17.32	15.27	41.59	19.49	4.79	7.26	24.64	24.22	6.61	6.82	13.01	19.32	13.63	5.06	10.13	14.65	5.19	4.89	7.75	11.49	1.60	17.10	9.46	0.005951501	1.8
P29401	Transketolase OS=Homo sapiens GN=TKT PE=1 SV=3	13.66	11.49	39.92	26.08	23.00	36.63	19.15	8.40	10.27	20.75	26.26	10.77	15.76	13.49	24.33	12.62	11.77	15.65	13.57	9.74	11.31	6.71	15.89	4.88	20.53	12.98	0.006885755	1.6
Q8WYR4	Radial spoke head 1 homolog OS=Homo sapiens GN=RSPH1 PE=2 SV=1	0.00	15.10	21.04	15.39	0.00	20.79	5.30	0.81	0.00	13.34	8.96	1.63	0.00	1.45	7.39	0.00	0.00	0.00	0.00	0.00	0.00	0.00	2.84	0.00	8.53	0.97	0.006885755	8.8
P32119	Peroxiredoxin-2 OS=Homo sapiens GN=PRDX2 PE=1 SV=5	15.50	17.67	24.62	22.02	39.60	49.62	28.90	9.30	14.11	14.08	33.03	13.79	24.23	16.54	31.72	17.33	16.83	15.80	13.75	13.37	12.83	8.23	14.62	6.76	23.52	16.00	0.007291099	1.5
P28838	Cytosol aminopeptidase OS=Homo sapiens GN=LAP3 PE=1 SV=3	27.33	11.32	23.19	23.30	30.55	20.32	20.69	5.15	9.04	17.97	17.14	11.99	20.23	13.81	15.74	9.59	7.21	11.66	24.24	1.56	3.21	6.87	9.37	4.36	18.17	10.65	0.007309319	1.7
P55072	Transitional endoplasmic reticulum ATPase OS=Homo sapiens GN=VCP PE=1 SV=4	11.01	14.41	26.77	22.45	24.13	36.39	17.95	8.13	8.90	19.45	18.87	11.09	11.29	12.36	21.46	12.95	7.47	11.81	16.28	4.28	4.73	8.39	13.76	4.80	18.30	10.80	0.007423246	1.7
P68036	Ubiquitin-conjugating enzyme E2 L3 OS=Homo sapiens GN=UBE2L3 PE=1 SV=1	20.19	16.98	23.67	21.17	25.64	42.77	16.93	8.94	9.31	25.20	28.47	8.08	23.29	10.92	23.61	16.66	8.60	15.19	17.91	5.32	11.48	7.91	14.05	2.72	20.61	13.14	0.007585404	1.6
P60900	Proteasome subunit alpha type-6 OS=Homo sapiens GN=PSMA6 PE=1 SV=1	15.50	9.78	18.17	16.25	22.81	13.47	13.00	3.61	5.48	4.63	15.25	6.20	5.88	6.42	5.96	6.73	1.52	1.84	7.23	0.00	0.00	7.75	8.09	3.04	12.01	4.54	0.007585404	2.6
Q96NY7	Chloride intracellular channel protein 6 OS=Homo sapiens GN=CLIC6 PE=2 SV=3	9.79	11.84	28.69	11.76	10.37	16.30	5.13	1.72	4.11	17.23	14.63	1.14	3.29	7.71	10.02	3.20	3.16	2.92	4.52	0.00	0.00	2.40	5.96	0.36	11.06	3.63	0.00814815	3.0
P68366	Tubulin alpha-4A chain OS=Homo sapiens GN=TUBA4A PE=1 SV=1	17.94	15.10	41.59	0.00	32.81	49.86	21.72	0.00	10.41	0.00	30.20	15.66	26.58	0.00	23.85	12.79	8.35	19.49	15.92	0.00	0.00	12.62	22.42	4.52	19.61	12.21	0.008612478	1.6
P13489	Ribonuclease inhibitor OS=Homo sapiens GN=RNH1 PE=1 SV=2	15.09	10.98	32.75	47.68	25.83	32.37	16.59	8.76	13.29	17.97	21.55	15.17	17.41	10.28	23.13	23.05	9.36	13.04	19.90	8.31	9.11	9.83	21.71	4.92	21.50	14.17	0.009617991	1.5
P49458	Signal recognition particle 9 kDa protein OS=Homo sapiens GN=SRP9 PE=1 SV=2	6.93	5.83	8.13	7.27	14.90	33.08	13.51	3.07	4.66	14.64	12.42	2.77	0.00	5.46	8.11	0.00	0.00	0.00	14.29	4.41	0.00	2.72	4.82	0.00	10.60	3.32	0.010418244	3.2
P23528	Cofilin-1 OS=Homo sapiens GN=CFL1 PE=1 SV=3	69.74	37.40	30.60	73.12	64.48	64.98	47.02	11.56	37.66	50.95	81.15	22.43	39.98	35.00	100.65	57.54	34.80	64.75	49.74	22.07	28.69	27.32	30.93	13.69	49.26	42.10	0.012807196	1.2
P0CW22	40S ribosomal protein S17-like OS=Homo sapiens GN=RPS17L PE=3 SV=1	9.79	3.77	19.12	17.10	22.44	28.12	8.21	0.00	3.01	8.89	12.58	3.92	11.29	3.53	5.25	8.08	6.07	0.00	8.68	0.00	0.00	1.76	3.12	3.20	11.41	4.25	0.012807196	2.7
Q9H4A4	Aminopeptidase B OS=Homo sapiens GN=RNPEP PE=1 SV=2	9.79	7.20	16.49	21.81	17.54	26.23	10.60	3.25	2.60	17.23	10.85	3.43	4.47	6.74	14.79	8.08	1.77	5.52	3.44	0.00	3.21	4.95	9.79	0.76	12.25	5.29	0.018339284	2.3
P06454	Prothymosin alpha OS=Homo sapiens GN=PTMA PE=1 SV=2	22.43	4.80	15.30	13.68	20.74	25.99	10.94	5.78	15.07	20.38	10.07	8.97	15.05	4.50	6.68	4.71	8.10	9.82	11.58	8.31	10.80	5.11	3.97	2.56	14.51	7.60	0.019675279	1.9
O00151	PDZ and LIM domain protein 1 OS=Homo sapiens GN=PDLIM1 PE=1 SV=4	20.39	14.41	23.90	29.50	22.25	32.61	11.63	6.14	7.40	18.53	15.73	9.63	9.88	8.67	16.22	19.85	10.63	10.43	12.30	2.47	7.09	11.02	19.58	2.72	17.68	10.91	0.02504267	1.6
P05386	60S acidic ribosomal protein P1 OS=Homo sapiens GN=RPLP1 PE=1 SV=1	6.12	5.15	27.97	14.54	22.06	16.07	5.13	6.14	9.31	12.60	10.69	5.55	0.00	4.82	16.22	5.05	0.00	10.43	5.43	0.00	5.06	2.40	9.65	1.20	11.78	5.02	0.025484393	2.3
P30085	UMP-CMP kinase OS=Homo sapiens GN=CMPK1 PE=1 SV=3	15.29	17.33	31.32	28.01	24.70	23.87	28.21	9.12	10.27	18.71	25.95	10.69	7.53	16.22	39.35	22.04	12.78	11.51	18.27	1.95	5.40	5.99	18.59	3.00	20.29	13.55	0.026087192	1.5
P05388	60S acidic ribosomal protein P0 OS=Homo sapiens GN=RPLP0 PE=1 SV=1	18.35	5.49	26.06	19.24	20.55	30.48	15.39	0.00	0.00	20.19	11.64	10.52	17.41	17.50	21.46	7.57	9.36	0.00	10.49	0.00	0.00	1.60	8.23	3.60	14.83	8.10	0.02613127	1.8
P09104	Gamma-enolase OS=Homo sapiens GN=ENO2 PE=1 SV=3	0.00	12.18	22.71	23.30	11.31	25.76	0.00	2.80	0.00	22.79	11.17	4.00	0.00	9.63	14.31	10.09	0.00	6.14	0.00	0.00	0.00	6.63	8.51	0.00	11.33	4.61	0.02613127	2.5
Q06830	Peroxiredoxin-1 OS=Homo sapiens GN=PRDX1 PE=1 SV=1	74.63	74.79	72.91	78.25	47.52	59.55	52.15	33.05	34.51	56.50	68.57	24.88	71.74	40.46	123.30	51.31	38.59	38.66	45.58	26.74	34.77	34.83	73.36	17.45	56.44	49.73	0.026521332	1.1
P08107	Heat shock 70 kDa protein 1A/1B OS=Homo sapiens GN=HSPA1A PE=1 SV=5	35.28	18.53	23.43	27.37	37.52	38.04	29.58	13.46	22.05	25.57	35.86	17.37	32.46	20.55	35.54	19.68	16.20	19.64	33.64	11.68	15.19	10.22	22.85	5.96	27.00	20.30	0.026530158	1.3
Q99832	T-complex protein 1 subunit eta OS=Homo sapiens GN=CCT7 PE=1 SV=2	9.18	6.52	21.51	21.59	25.45	26.47	6.50	2.80	1.51	16.67	14.15	3.10	8.94	6.10	16.93	7.57	3.92	8.13	4.34	0.00	0.00	6.39	11.35	2.12	12.95	6.32	0.029457196	2.1
P18085	ADP-ribosylation factor 4 OS=Homo sapiens GN=ARF4 PE=1 SV=3	16.93	9.78	19.84	24.16	0.00	13.47	19.32	0.00	0.00	0.00	5.50	19.09	0.00	2.57	13.59	9.59	4.43	0.00	6.33	0.00	0.00	6.63	0.00	5.88	10.67	4.09	0.031756112	2.6
P30838	Aldehyde dehydrogenase, dimeric NADP-preferring OS=Homo sapiens GN=ALDH3A1 PE=1 SV=3	15.50	17.15	37.53	30.15	29.60	37.10	21.72	12.73	15.48	32.24	33.18	26.51	29.87	14.13	33.63	21.37	19.87	17.34	20.44	12.98	14.85	10.14	24.69	11.01	25.74	19.19	0.033783104	1.3
P25786	Proteasome subunit alpha type-1 OS=Homo sapiens GN=PSMA1 PE=1 SV=1	4.89	12.01	12.67	28.44	20.55	20.79	15.05	6.32	7.26	6.85	20.92	8.89	12.47	5.94	12.64	4.04	4.68	8.13	12.66	1.43	4.05	5.59	12.49	2.80	13.72	7.24	0.03775074	1.9
P60981	Destrin OS=Homo sapiens GN=DSTN PE=1 SV=3	12.85	10.81	30.12	19.67	23.76	21.74	10.77	11.38	22.87	30.94	26.26	13.62	4.23	10.12	21.94	15.48	7.97	14.12	30.20	5.06	10.63	10.06	23.70	3.68	19.57	13.10	0.03803777	1.5
Q02878	60S ribosomal protein L6 OS=Homo sapiens GN=RPL6 PE=1 SV=3	2.04	3.60	23.19	13.26	3.96	27.65	5.81	0.00	0.00	17.97	15.25	2.77	8.00	3.37	14.79	0.00	1.27	0.00	6.15	0.00	0.00	0.00	4.82	0.84	9.63	3.27	0.045457972	2.9
O00764	Pyridoxal kinase OS=Homo sapiens GN=PDXK PE=1 SV=1	6.32	9.78	20.80	22.45	8.11	34.26	12.14	5.15	4.25	7.97	16.51	4.65	4.70	13.97	13.59	7.23	2.53	6.60	10.31	1.17	1.52	4.55	8.09	2.28	12.70	6.38	0.047673897	2.0
O95777	N-alpha-acetyltransferase 38, NatC auxiliary subunit OS=Homo sapiens GN=NAA38 PE=1 SV=3	0.00	5.66	7.89	29.08	14.52	18.20	5.64	2.98	0.00	14.26	5.19	0.00	0.00	0.00	7.87	12.95	0.00	0.00	0.00	0.00	0.00	2.64	4.68	0.00	8.62	2.35	0.051100737	3.7
P01621	Ig kappa chain V-III region NG9 (Fragment) OS=Homo sapiens PE=1 SV=1	0.00	0.00	0.00	0.00	0.00	0.00	12.65	6.68	0.00	0.00	0.00	0.00	30.34	0.00	7.63	0.00	0.00	11.35	13.38	9.61	21.77	0.00	0.00	0.00	1.61	7.84	0.05445256	0.2
P07195	L-lactate dehydrogenase B chain OS=Homo sapiens GN=LDHB PE=1 SV=2	23.86	23.33	43.27	38.70	34.13	37.34	16.93	8.94	16.02	25.20	21.39	12.89	19.52	18.79	27.90	22.88	17.21	17.95	28.58	15.19	19.75	9.35	25.68	4.68	25.17	18.96	0.05568564	1.3
P01036	Cystatin-S OS=Homo sapiens GN=CST4 PE=1 SV=3	9.18	12.87	10.76	0.00	3.77	0.00	18.98	4.06	21.09	0.00	0.00	3.67	80.91	7.23	10.73	3.36	2.53	6.90	20.08	0.00	18.74	3.59	0.00	4.40	7.03	13.21	0.058196347	0.5
P05787	Keratin, type II cytoskeletal 8 OS=Homo sapiens GN=KRT8 PE=1 SV=7	16.52	8.92	21.99	15.18	19.61	27.65	10.43	7.32	9.72	21.68	20.44	10.60	14.35	5.62	14.55	13.63	10.25	12.43	12.84	5.58	11.98	5.67	7.38	2.84	15.84	9.76	0.067710005	1.6
P62424	60S ribosomal protein L7a OS=Homo sapiens GN=RPL7A PE=1 SV=2	14.07	8.92	20.80	28.01	16.40	25.52	8.89	0.00	1.51	16.12	13.68	4.24	8.70	13.97	25.76	6.23	4.68	1.69	9.41	0.00	0.00	1.84	9.79	3.48	13.18	7.13	0.070143392	1.8
P01834	Ig kappa chain C region OS=Homo sapiens GN=IGKC PE=1 SV=1	244.29	86.11	62.15	78.25	127.84	61.44	115.93	61.23	92.86	93.00	78.95	128.72	159.47	108.86	215.84	201.56	85.79	77.02	122.63	47.52	84.73	40.10	96.21	63.16	102.56	108.57	0.073580672	0.9
Q16851	UTP--glucose-1-phosphate uridylyltransferase OS=Homo sapiens GN=UGP2 PE=1 SV=5	19.58	12.87	20.32	25.44	20.18	28.12	12.82	3.61	6.57	12.23	16.83	4.65	15.52	6.42	22.90	6.73	8.35	8.75	13.57	1.56	5.40	5.99	13.62	2.28	15.27	9.26	0.073580672	1.6
P01857	Ig gamma-1 chain C region OS=Homo sapiens GN=IGHG1 PE=1 SV=1	54.24	23.67	43.75	44.47	39.22	27.88	23.60	16.53	41.09	33.90	21.70	16.97	48.92	48.17	89.44	56.53	47.45	24.39	33.10	30.64	26.84	16.62	29.52	7.32	32.25	38.24	0.074913108	0.8
P61626	Lysozyme C OS=Homo sapiens GN=LYZ PE=1 SV=1	66.89	25.39	10.52	15.39	37.14	17.01	18.30	9.66	14.65	27.42	11.32	20.96	77.15	41.26	25.52	18.00	18.73	22.71	19.35	25.58	33.25	20.53	36.47	7.89	22.89	28.87	0.074939839	0.8
Q9BUT1	3-hydroxybutyrate dehydrogenase type 2 OS=Homo sapiens GN=BDH2 PE=1 SV=2	8.56	2.06	24.14	16.89	14.90	13.94	7.18	1.08	3.56	7.78	9.28	4.81	0.00	4.17	6.20	7.07	0.00	3.99	7.60	0.00	0.00	6.31	5.96	1.04	9.52	3.53	0.074939839	2.7
P12429	Annexin A3 OS=Homo sapiens GN=ANXA3 PE=1 SV=3	13.87	11.66	12.91	11.55	26.02	19.85	5.13	2.71	0.00	10.00	1.42	4.40	0.00	3.05	23.85	3.20	1.14	8.29	0.00	0.00	0.00	0.00	7.66	0.76	9.96	4.00	0.076506343	2.5
P48594	Serpin B4 OS=Homo sapiens GN=SERPINB4 PE=1 SV=2	30.79	25.90	32.03	50.03	35.82	17.96	32.49	8.04	20.68	19.08	16.20	27.98	39.98	12.20	21.23	31.97	21.51	15.80	24.24	9.87	19.92	8.23	21.43	19.53	26.42	20.49	0.080812085	1.3
P15311	Ezrin OS=Homo sapiens GN=EZR PE=1 SV=4	12.03	10.12	17.69	19.46	22.44	33.08	15.56	7.41	9.04	13.71	12.90	6.04	10.58	7.23	12.40	13.80	9.36	11.35	10.67	4.93	8.78	7.27	10.50	1.80	14.96	9.06	0.082470557	1.7
Q01469	Fatty acid-binding protein, epidermal OS=Homo sapiens GN=FABP5 PE=1 SV=3	16.52	51.29	11.47	48.53	31.87	39.93	28.90	15.26	16.44	22.23	26.58	24.39	39.75	36.45	54.14	38.19	28.72	45.87	54.08	29.47	28.53	9.59	23.98	15.45	27.78	33.68	0.082470557	0.8
P62269	40S ribosomal protein S18 OS=Homo sapiens GN=RPS18 PE=1 SV=3	14.07	7.20	10.04	14.75	19.04	23.87	11.80	6.23	5.75	12.78	37.43	1.55	23.76	6.74	10.02	11.61	5.31	6.44	7.60	0.00	3.21	8.07	9.79	1.68	13.71	7.85	0.087646219	1.7
Q9BY32	Inosine triphosphate pyrophosphatase OS=Homo sapiens GN=ITPA PE=1 SV=2	0.00	2.57	18.41	22.24	6.22	18.20	13.17	0.00	0.00	9.82	12.11	0.00	0.00	5.30	7.87	2.52	0.00	0.00	2.71	0.00	0.00	2.64	10.93	0.60	8.56	2.71	0.088198119	3.2
Q8N392	Rho GTPase-activating protein 18 OS=Homo sapiens GN=ARHGAP18 PE=1 SV=3	0.82	10.29	19.60	14.32	5.47	15.83	4.10	1.72	0.00	13.71	10.54	0.73	0.00	7.55	9.78	0.00	0.00	1.38	0.00	0.00	0.00	3.28	4.97	0.16	8.09	2.26	0.089332692	3.6
P62241	40S ribosomal protein S8 OS=Homo sapiens GN=RPS8 PE=1 SV=2	23.65	11.49	21.51	19.24	16.97	27.41	15.39	0.00	3.97	12.41	14.15	2.37	21.17	7.55	21.46	7.91	5.95	2.15	16.28	0.00	0.00	2.32	12.77	1.16	14.05	8.23	0.090600607	1.7
P51991	Heterogeneous nuclear ribonucleoprotein A3 OS=Homo sapiens GN=HNRNPA3 PE=1 SV=2	1.63	4.63	6.45	16.03	7.16	17.72	2.91	2.44	1.10	13.89	14.00	2.20	1.88	1.28	4.05	1.35	1.01	1.23	4.88	0.00	0.00	1.36	3.83	0.00	7.51	1.74	0.096480772	4.3
P67936	Tropomyosin alpha-4 chain OS=Homo sapiens GN=TPM4 PE=1 SV=3	0.00	9.43	13.15	11.76	17.54	21.98	9.40	6.59	5.34	13.52	22.17	9.46	2.82	6.26	17.41	9.25	4.93	5.98	13.20	0.00	0.00	3.12	5.53	2.92	11.70	5.95	0.100373353	2.0
P60953	Cell division control protein 42 homolog OS=Homo sapiens GN=CDC42 PE=1 SV=2	11.01	9.26	32.99	16.68	19.99	25.05	18.12	4.88	10.68	14.45	12.27	8.65	8.00	5.46	18.60	17.83	6.83	16.26	6.15	4.41	13.17	6.23	11.07	2.16	15.34	9.68	0.113920392	1.6
O95336	6-phosphogluconolactonase OS=Homo sapiens GN=PGLS PE=1 SV=2	8.16	6.86	18.17	20.74	18.29	22.92	13.00	3.61	5.48	10.56	11.95	3.26	5.88	6.42	13.59	9.59	1.52	1.84	7.23	0.00	4.22	9.67	13.76	1.60	11.92	6.28	0.115644744	1.9
P61978	Heterogeneous nuclear ribonucleoprotein K OS=Homo sapiens GN=HNRNPK PE=1 SV=1	7.55	11.15	23.43	23.73	16.22	26.23	11.11	8.85	6.16	18.16	21.86	7.02	6.59	15.73	20.51	10.94	6.96	13.19	11.76	1.69	3.54	7.83	12.20	3.00	15.12	9.50	0.116955548	1.6
Q9BW83	Intraflagellar transport protein 27 homolog OS=Homo sapiens GN=IFT27 PE=1 SV=1	0.00	9.78	19.60	17.53	15.46	19.38	2.74	1.44	2.19	10.56	8.96	0.00	0.00	2.57	26.71	0.00	0.00	0.00	0.00	0.00	0.00	2.80	8.09	0.00	8.97	3.35	0.116958693	2.7
O75083	WD repeat-containing protein 1 OS=Homo sapiens GN=WDR1 PE=1 SV=4	8.16	14.92	16.73	20.52	21.87	29.77	14.88	4.88	6.44	16.12	21.55	5.06	7.76	11.24	14.79	10.43	7.84	11.97	15.74	5.19	6.75	6.95	12.35	2.48	15.07	9.46	0.116967704	1.6
P09651	Heterogeneous nuclear ribonucleoprotein A1 OS=Homo sapiens GN=HNRNPA1 PE=1 SV=5	0.00	0.00	0.00	16.46	11.88	21.74	15.73	4.52	3.83	11.67	17.14	3.18	6.59	8.03	0.00	0.00	2.28	0.00	7.05	0.00	4.73	0.00	8.94	1.56	8.85	3.26	0.122662458	2.7
P07384	Calpain-1 catalytic subunit OS=Homo sapiens GN=CAPN1 PE=1 SV=1	5.30	9.26	15.78	21.81	10.18	22.21	13.51	4.33	5.07	11.12	12.42	4.89	7.29	4.98	14.31	7.07	4.68	7.36	5.61	2.21	3.71	4.79	5.96	1.68	11.32	5.80	0.133459881	2.0
P25787	Proteasome subunit alpha type-2 OS=Homo sapiens GN=PSMA2 PE=1 SV=2	16.93	7.55	14.82	28.44	15.65	19.61	7.52	5.60	8.49	15.38	25.63	5.06	6.35	7.06	25.52	10.43	5.57	6.75	15.01	3.51	4.56	4.95	11.78	3.32	14.22	8.73	0.138494373	1.6
Q9H098	Protein FAM107B OS=Homo sapiens GN=FAM107B PE=1 SV=1	9.79	13.89	19.36	17.32	4.15	11.34	0.00	1.99	0.00	15.01	7.55	6.61	5.17	3.53	11.45	3.70	0.00	12.43	0.00	0.00	0.00	0.00	3.12	1.92	8.92	3.44	0.140567174	2.6
Q96FJ2	Dynein light chain 2, cytoplasmic OS=Homo sapiens GN=DYNLL2 PE=1 SV=1	0.00	5.66	7.89	7.06	6.22	18.20	13.17	2.98	0.00	6.11	12.11	2.69	0.00	0.00	0.00	0.00	4.18	5.06	0.00	0.00	0.00	2.64	4.68	0.00	6.84	1.38	0.142322886	5.0
Q9Y230	RuvB-like 2 OS=Homo sapiens GN=RUVBL2 PE=1 SV=3	4.28	11.15	20.56	13.90	14.14	13.00	12.82	4.97	2.88	12.04	11.80	2.28	1.41	12.04	15.50	3.53	3.54	5.68	1.09	0.00	0.00	4.39	10.64	0.84	10.32	4.89	0.146796144	2.1
O43598	Deoxyribonucleoside 5'-monophosphate N-glycosidase OS=Homo sapiens GN=RCL PE=1 SV=1	0.00	15.61	14.82	8.12	7.16	21.50	6.50	3.43	2.47	22.97	9.75	0.00	0.00	9.95	14.79	6.39	2.28	2.76	0.00	0.00	0.00	1.44	8.80	0.72	9.36	3.93	0.146796144	2.4
Q13185	Chromobox protein homolog 3 OS=Homo sapiens GN=CBX3 PE=1 SV=4	0.00	6.00	19.36	11.97	0.00	19.14	13.85	0.00	0.00	0.00	2.52	1.31	0.00	0.00	3.82	0.00	0.00	2.45	0.00	0.00	0.00	0.00	2.27	0.64	6.18	0.77	0.149175136	8.1
P17655	Calpain-2 catalytic subunit OS=Homo sapiens GN=CAPN2 PE=1 SV=6	8.77	11.66	35.86	19.03	14.14	21.03	15.22	1.99	5.07	7.97	14.00	5.55	4.00	7.87	31.24	11.44	4.05	5.68	7.78	2.21	0.68	3.43	13.76	3.56	13.36	7.97	0.155116057	1.7
Q9Y547	Heat shock protein beta-11 OS=Homo sapiens GN=HSPB11 PE=1 SV=1	0.00	3.60	10.76	9.62	21.12	10.63	3.59	1.90	0.00	13.89	17.61	1.71	0.00	3.37	10.73	0.00	2.66	3.22	0.00	0.00	0.00	3.59	6.39	0.00	7.87	2.50	0.156294201	3.2
Q9Y333	U6 snRNA-associated Sm-like protein LSm2 OS=Homo sapiens GN=LSM2 PE=1 SV=1	6.32	12.52	30.36	27.15	0.00	17.25	5.30	2.80	0.00	13.52	11.48	5.95	7.29	0.00	17.41	12.28	0.00	4.76	5.61	0.00	0.00	5.83	10.36	5.08	11.06	5.72	0.163661007	1.9
Q15185	Prostaglandin E synthase 3 OS=Homo sapiens GN=PTGES3 PE=1 SV=1	3.67	3.09	15.06	13.47	17.35	14.89	15.73	5.69	2.47	11.67	9.91	3.18	9.17	10.12	15.03	0.00	2.28	5.98	3.26	0.00	0.00	0.00	5.53	1.56	9.68	4.41	0.178407915	2.2
P12956	X-ray repair cross-complementing protein 6 OS=Homo sapiens GN=XRCC6 PE=1 SV=2	7.75	7.55	9.08	15.61	9.62	19.14	6.50	2.89	2.05	10.75	9.12	5.38	3.53	4.17	7.63	7.40	1.90	3.07	3.62	0.65	0.84	3.04	4.54	2.04	8.79	3.54	0.182317931	2.5
P48643	T-complex protein 1 subunit epsilon OS=Homo sapiens GN=CCT5 PE=1 SV=1	4.89	6.52	16.73	14.97	13.20	16.54	7.69	0.99	1.51	18.53	14.15	2.53	2.59	4.98	14.79	10.43	2.15	3.68	1.99	0.00	0.84	3.59	8.80	1.80	9.85	4.64	0.189853758	2.1
Q9UL46	Proteasome activator complex subunit 2 OS=Homo sapiens GN=PSME2 PE=1 SV=4	59.95	21.10	29.40	38.48	40.35	23.39	30.78	11.11	13.56	14.26	19.34	23.98	42.34	19.75	42.93	25.24	18.98	23.01	32.56	7.53	13.00	14.38	17.45	6.00	27.14	21.93	0.189959793	1.2
Q96C86	Scavenger mRNA-decapping enzyme DcpS OS=Homo sapiens GN=DCPS PE=1 SV=2	5.71	10.98	22.23	13.68	5.28	9.22	3.08	0.00	3.83	17.23	8.02	0.73	2.12	8.19	9.30	4.71	0.00	4.30	1.63	0.00	0.00	2.24	3.97	1.12	8.33	3.13	0.189959793	2.7
Q9Y224	UPF0568 protein C14orf166 OS=Homo sapiens GN=C14orf166 PE=1 SV=1	2.45	4.29	9.56	16.03	4.71	13.23	12.82	0.00	1.64	4.63	11.80	2.04	0.00	0.00	9.54	2.02	1.52	0.00	2.17	0.00	0.00	0.96	3.55	1.00	6.93	1.73	0.189959793	4.0
Q7RTV2	Glutathione S-transferase A5 OS=Homo sapiens GN=GSTA5 PE=2 SV=1	0.00	10.81	15.06	13.47	0.00	14.89	0.00	0.00	0.00	8.15	0.00	0.00	0.00	0.00	0.00	0.00	0.00	0.00	0.00	0.00	0.00	0.00	0.00	0.00	5.20	0.00	0.189959793	#DIV/0!
P50990	T-complex protein 1 subunit theta OS=Homo sapiens GN=CCT8 PE=1 SV=4	10.81	17.15	19.12	19.03	15.08	23.63	10.43	4.06	2.33	16.49	17.46	4.98	5.64	9.79	19.08	13.46	6.71	8.13	12.66	1.43	0.84	4.87	14.19	1.52	13.38	8.19	0.191627062	1.6
P50225	Sulfotransferase 1A1 OS=Homo sapiens GN=SULT1A1 PE=1 SV=3	0.00	0.00	17.69	9.62	3.77	7.56	5.47	0.00	0.00	10.93	17.14	3.67	0.00	0.00	0.00	0.00	0.00	0.00	0.00	0.00	0.00	1.60	10.50	1.80	6.32	1.16	0.197192435	5.5
P01623	Ig kappa chain V-III region WOL OS=Homo sapiens PE=1 SV=1	0.00	0.00	0.00	0.00	48.27	0.00	0.00	0.00	0.00	0.00	0.00	0.00	0.00	0.00	0.00	0.00	0.00	27.00	0.00	0.00	83.05	0.00	0.00	0.00	4.02	9.17	0.199852387	0.4
P02647	Apolipoprotein A-I OS=Homo sapiens GN=APOA1 PE=1 SV=1	65.46	21.79	49.96	68.63	89.19	42.30	54.89	21.95	57.11	51.87	28.15	29.94	111.25	44.96	76.56	61.75	46.44	32.07	58.06	61.41	79.84	22.37	39.73	8.37	48.44	53.57	0.203491453	0.9
P00568	Adenylate kinase isoenzyme 1 OS=Homo sapiens GN=AK1 PE=1 SV=3	6.73	9.26	18.41	11.55	19.80	12.76	13.17	9.48	2.05	19.45	21.39	4.40	3.53	12.36	12.88	9.09	6.83	8.29	13.93	0.00	0.00	6.15	10.93	3.08	12.37	7.25	0.206711571	1.7
P30520	Adenylosuccinate synthetase isozyme 2 OS=Homo sapiens GN=ADSS PE=1 SV=3	7.55	7.89	18.41	11.97	5.47	13.23	6.33	5.96	2.88	8.52	13.84	3.75	1.65	2.09	8.82	3.53	0.00	3.22	3.80	0.00	9.45	3.67	6.53	1.84	8.82	3.72	0.209736848	2.4
O43175	D-3-phosphoglycerate dehydrogenase OS=Homo sapiens GN=PHGDH PE=1 SV=4	1.22	6.86	15.54	11.97	3.39	15.36	4.27	0.54	0.82	8.89	6.29	0.49	0.00	1.93	4.29	1.01	0.00	0.92	0.00	0.00	0.00	3.83	2.55	0.48	6.31	1.25	0.222571038	5.0
Q13765	Nascent polypeptide-associated complex subunit alpha OS=Homo sapiens GN=NACA PE=1 SV=1	9.99	5.32	11.71	10.48	17.91	16.78	8.38	2.80	0.00	9.08	14.94	4.00	3.29	4.98	11.69	5.22	8.98	7.52	5.61	0.00	2.36	0.00	0.00	1.24	9.28	4.24	0.224510406	2.2
Q96C23	Aldose 1-epimerase OS=Homo sapiens GN=GALM PE=1 SV=1	1.84	6.86	22.71	11.12	7.54	22.45	4.96	1.63	1.23	9.63	10.22	1.47	0.00	4.66	12.40	3.03	5.06	4.45	3.26	0.00	0.00	4.15	4.12	0.36	8.47	3.46	0.231702347	2.5
Q8TDL5	BPI fold-containing family B member 1 OS=Homo sapiens GN=BPIFB1 PE=2 SV=1	48.33	18.35	12.67	20.31	48.65	8.27	56.43	16.35	38.35	15.56	14.94	29.04	42.57	21.52	22.66	10.60	25.05	14.58	32.74	23.50	36.63	11.90	17.03	8.69	27.27	22.29	0.240728159	1.2
Q9H1X1	Radial spoke head protein 9 homolog OS=Homo sapiens GN=RSPH9 PE=1 SV=1	0.00	8.58	11.95	10.69	4.15	8.27	3.76	0.99	0.00	12.23	10.38	0.00	0.00	1.77	5.25	0.00	0.00	3.38	0.00	0.00	0.00	0.88	0.00	0.00	5.92	0.94	0.240894122	6.3
P0C0S8	Histone H2A type 1 OS=Homo sapiens GN=HIST1H2AG PE=1 SV=2	0.00	4.12	0.00	5.13	17.16	21.50	0.00	0.00	0.00	4.45	3.77	4.40	0.00	0.00	0.00	0.00	0.00	0.00	0.00	0.00	0.00	0.00	0.00	0.96	5.04	0.08	0.243536795	63.0
P18124	60S ribosomal protein L7 OS=Homo sapiens GN=RPL7 PE=1 SV=1	2.24	1.89	5.74	8.12	21.12	16.78	9.23	0.99	1.51	13.15	8.49	1.96	5.64	1.77	12.88	0.00	0.00	4.34	0.00	0.00	1.86	0.88	3.41	0.96	7.60	2.64	0.245545909	2.9
P19338	Nucleolin OS=Homo sapiens GN=NCL PE=1 SV=3	9.38	5.83	11.00	8.55	15.08	18.90	12.48	4.70	2.47	10.93	13.84	3.26	10.82	4.66	9.54	6.73	6.58	1.38	5.25	1.17	3.04	1.04	5.68	1.16	9.70	4.75	0.245484498	2.0
P40925	Malate dehydrogenase, cytoplasmic OS=Homo sapiens GN=MDH1 PE=1 SV=4	16.93	11.66	23.90	21.38	22.25	16.07	17.10	10.66	18.90	29.46	21.70	6.77	23.52	13.33	23.85	11.44	12.65	8.29	18.09	12.98	16.88	5.43	7.66	3.32	18.06	13.12	0.245484498	1.4
P09429	High mobility group protein B1 OS=Homo sapiens GN=HMGB1 PE=1 SV=3	5.71	11.15	6.69	18.60	12.26	15.36	11.11	1.17	3.83	12.04	4.40	7.10	6.59	4.50	15.50	10.94	5.82	1.99	0.00	0.00	0.00	0.00	3.97	1.84	9.12	4.26	0.275097704	2.1
Q92598	Heat shock protein 105 kDa OS=Homo sapiens GN=HSPH1 PE=1 SV=1	3.67	6.69	10.52	10.48	12.07	13.94	6.67	1.26	1.37	10.00	6.92	3.18	0.71	3.53	7.15	2.36	1.77	2.15	1.81	0.00	0.51	2.08	5.53	1.04	7.23	2.39	0.277765281	3.0
P28074	Proteasome subunit beta type-5 OS=Homo sapiens GN=PSMB5 PE=1 SV=3	0.00	6.69	17.45	5.34	2.26	21.98	0.00	1.08	0.00	7.23	11.48	0.00	0.00	6.26	2.86	4.21	0.00	0.00	0.00	0.00	0.00	0.00	1.70	0.48	6.13	1.29	0.280149635	4.7
Q9P2T1	GMP reductase 2 OS=Homo sapiens GN=GMPR2 PE=1 SV=1	1.84	6.69	9.32	8.34	12.07	12.05	3.08	0.81	1.23	14.45	10.07	1.47	0.00	1.45	2.15	3.03	1.14	1.38	5.06	0.00	0.00	3.12	5.53	0.72	6.78	1.96	0.283495519	3.5
Q15181	Inorganic pyrophosphatase OS=Homo sapiens GN=PPA1 PE=1 SV=2	12.44	13.38	32.51	16.68	17.91	22.45	10.43	1.90	6.44	11.30	9.59	4.98	7.76	7.55	22.66	7.91	4.18	5.06	20.80	2.73	1.69	7.59	11.07	3.80	13.33	8.57	0.301674118	1.6
P37108	Signal recognition particle 14 kDa protein OS=Homo sapiens GN=SRP14 PE=1 SV=2	17.74	8.92	12.43	11.12	9.80	20.56	8.89	4.70	7.12	16.12	20.60	7.10	12.23	8.35	12.40	8.75	2.91	3.53	9.41	2.99	8.78	4.15	12.35	2.08	12.09	7.33	0.301674118	1.7
P0CG05	Ig lambda-2 chain C regions OS=Homo sapiens GN=IGLC2 PE=1 SV=1	147.23	0.00	0.00	58.37	100.31	90.98	65.83	34.77	72.86	98.56	83.67	43.40	125.13	85.42	126.88	64.77	67.32	81.62	96.22	69.07	64.99	42.50	0.00	28.90	66.33	71.07	0.310962679	0.9
P01871	Ig mu chain C region OS=Homo sapiens GN=IGHM PE=1 SV=3	36.30	13.38	3.35	14.32	21.68	8.98	15.22	7.04	12.19	5.37	5.98	2.37	37.87	25.85	6.92	19.35	20.37	15.65	23.51	18.82	21.94	3.04	7.95	1.52	12.18	16.90	0.31627154	0.7
Q12792	Twinfilin-1 OS=Homo sapiens GN=TWF1 PE=1 SV=3	7.55	12.52	11.47	12.83	11.31	14.18	8.21	2.44	1.10	11.12	5.82	4.89	1.88	1.28	11.45	10.09	3.42	0.00	3.07	0.00	0.00	3.83	10.36	1.48	8.62	3.91	0.31627154	2.2
P39687	Acidic leucine-rich nuclear phosphoprotein 32 family member A OS=Homo sapiens GN=ANP32A PE=1 SV=1	7.95	12.52	17.45	15.61	10.37	17.25	9.40	3.52	1.64	13.52	11.48	2.04	9.17	8.83	13.12	4.21	1.52	3.84	4.52	1.56	9.28	3.12	5.53	1.56	10.23	5.52	0.317582276	1.9
Q00610	Clathrin heavy chain 1 OS=Homo sapiens GN=CLTC PE=1 SV=5	11.22	6.35	16.97	21.17	12.82	20.32	13.17	1.44	3.56	12.60	12.11	6.28	5.17	9.15	21.23	10.60	4.43	2.76	10.85	0.39	0.34	3.75	10.50	2.52	11.50	6.81	0.319892154	1.7
Q9BRA2	Thioredoxin domain-containing protein 17 OS=Homo sapiens GN=TXNDC17 PE=1 SV=1	18.56	9.26	21.75	11.55	17.16	21.50	33.00	2.17	12.46	4.45	14.31	4.40	5.64	3.85	21.70	15.31	11.51	8.29	16.46	0.00	4.05	10.86	12.91	3.64	14.21	9.52	0.319892154	1.5
Q9Y266	Nuclear migration protein nudC OS=Homo sapiens GN=NUDC PE=1 SV=1	5.71	6.69	9.32	10.90	14.90	18.67	10.94	4.61	6.98	14.64	14.78	2.28	9.17	6.26	12.16	4.71	4.93	5.98	3.26	0.00	3.04	4.07	9.08	1.56	10.04	5.35	0.322279774	1.9
Q7Z406	Myosin-14 OS=Homo sapiens GN=MYH14 PE=1 SV=2	7.55	4.29	19.36	13.26	12.07	19.85	10.94	4.06	2.74	12.78	12.42	3.83	5.88	4.66	18.13	8.24	3.42	2.92	6.69	0.00	1.69	5.91	8.09	1.52	10.26	5.59	0.327500388	1.8
Q6UWW0	Lipocalin-15 OS=Homo sapiens GN=LCN15 PE=1 SV=1	7.14	6.00	8.37	7.48	10.75	8.27	9.75	3.16	15.48	10.56	2.52	1.31	0.00	0.00	3.82	0.00	4.43	5.37	0.00	2.08	5.91	4.55	8.09	0.64	7.56	2.91	0.329236578	2.6
O43707	Alpha-actinin-4 OS=Homo sapiens GN=ACTN4 PE=1 SV=2	24.88	20.93	27.49	37.41	25.64	25.76	24.45	10.39	16.71	22.60	23.75	16.40	30.34	15.41	30.77	24.06	10.76	15.65	31.65	9.61	21.77	9.75	13.62	7.32	23.03	18.39	0.334559695	1.3
P61981	14-3-3 protein gamma OS=Homo sapiens GN=YWHAG PE=1 SV=2	15.09	9.61	17.69	25.01	38.28	40.41	29.24	15.44	12.87	26.49	37.43	19.41	33.63	11.88	34.11	28.77	21.64	26.24	25.86	12.20	12.49	7.51	10.50	6.84	23.91	19.31	0.344319301	1.2
P21980	Protein-glutamine gamma-glutamyltransferase 2 OS=Homo sapiens GN=TGM2 PE=1 SV=2	13.05	2.23	15.30	12.19	19.04	7.80	21.72	0.36	3.15	4.26	6.13	5.22	13.41	3.69	7.87	3.03	4.93	0.00	15.37	0.00	0.00	2.64	3.26	0.92	9.20	4.59	0.344319301	2.0
Q14240	Eukaryotic initiation factor 4A-II OS=Homo sapiens GN=EIF4A2 PE=1 SV=2	6.32	5.32	12.19	13.04	0.00	12.05	10.43	0.00	0.00	7.60	11.48	3.34	0.00	0.00	12.16	0.00	2.91	0.00	0.00	4.02	2.53	3.28	0.00	1.64	6.81	2.21	0.344319301	3.1
P25713	Metallothionein-3 OS=Homo sapiens GN=MT3 PE=1 SV=1	0.00	0.00	0.00	0.00	8.11	25.05	7.35	0.00	0.00	7.97	6.76	0.00	0.00	0.00	0.00	0.00	0.00	0.00	0.00	0.00	0.00	0.00	0.00	0.00	4.60	0.00	0.344319301	#DIV/0!
P21291	Cysteine and glycine-rich protein 1 OS=Homo sapiens GN=CSRP1 PE=1 SV=3	0.00	2.74	13.15	7.27	10.37	25.28	9.40	1.44	4.66	2.96	12.42	1.31	0.00	2.57	8.11	2.69	2.02	2.45	6.15	0.00	0.00	2.72	7.80	1.36	7.58	2.99	0.346164857	2.5
Q6GMV3	Putative peptidyl-tRNA hydrolase PTRHD1 OS=Homo sapiens GN=PTRHD1 PE=1 SV=1	4.28	3.60	18.88	0.00	8.86	18.67	0.00	1.90	6.44	8.71	12.42	0.00	0.00	0.00	5.01	3.53	2.66	7.21	0.00	0.00	0.00	3.75	6.67	0.00	6.98	2.40	0.35174075	2.9
P12273	Prolactin-inducible protein OS=Homo sapiens GN=PIP PE=1 SV=1	85.24	71.70	79.36	70.98	48.83	78.45	44.29	37.75	45.47	77.44	65.74	27.08	98.32	103.88	79.18	55.86	42.01	64.13	94.41	54.27	70.56	33.39	59.31	31.86	61.03	65.60	0.35288094	0.9
P50991	T-complex protein 1 subunit delta OS=Homo sapiens GN=CCT4 PE=1 SV=4	13.05	14.24	17.45	17.75	15.65	27.17	15.90	4.97	3.42	17.23	16.36	3.18	11.05	11.72	22.18	12.28	4.93	8.92	15.01	0.00	1.01	8.31	13.20	2.20	13.86	9.31	0.358175705	1.5
Q9UNM6	26S proteasome non-ATPase regulatory subunit 13 OS=Homo sapiens GN=PSMD13 PE=1 SV=2	5.10	2.74	10.76	14.54	10.56	13.23	7.69	1.44	2.19	2.96	12.58	4.57	0.00	1.28	10.73	4.21	4.30	2.45	4.52	0.00	0.00	2.72	2.27	1.36	7.36	2.82	0.361627832	2.6
P35321	Cornifin-A OS=Homo sapiens GN=SPRR1A PE=1 SV=2	0.00	13.38	0.00	7.27	0.00	0.00	0.00	7.04	0.00	0.00	0.00	0.00	0.00	5.46	18.60	5.72	17.46	0.00	14.11	10.13	0.00	2.72	4.82	3.12	2.31	6.85	0.36242949	0.3
P46779	60S ribosomal protein L28 OS=Homo sapiens GN=RPL28 PE=1 SV=3	16.11	8.23	18.88	10.26	9.05	18.67	8.21	0.00	6.57	4.08	7.55	3.92	11.29	3.53	11.45	0.00	6.07	0.00	8.68	0.00	3.71	3.83	6.81	1.92	9.29	4.78	0.368629223	1.9
P62829	60S ribosomal protein L23 OS=Homo sapiens GN=RPL23 PE=1 SV=1	4.69	8.75	12.19	10.90	9.62	20.09	8.72	0.00	6.98	15.75	8.02	1.88	19.99	3.69	12.16	0.00	0.00	3.53	4.16	0.00	0.00	1.84	7.24	0.92	8.97	4.46	0.373743119	2.0
P61019	Ras-related protein Rab-2A OS=Homo sapiens GN=RAB2A PE=1 SV=1	2.85	5.15	11.47	14.75	5.66	11.34	11.80	2.71	1.92	8.89	10.85	3.92	0.00	0.00	11.45	5.05	0.00	0.00	0.00	0.00	2.36	5.51	13.20	0.00	7.61	3.13	0.38450091	2.4
Q16772	Glutathione S-transferase A3 OS=Homo sapiens GN=GSTA3 PE=1 SV=3	0.00	0.00	20.56	0.00	16.22	20.32	0.00	0.00	0.00	15.93	13.53	0.00	0.00	0.00	26.23	0.00	0.00	0.00	0.00	0.00	0.00	6.87	0.00	0.00	7.21	2.76	0.393877512	2.6
P62913	60S ribosomal protein L11 OS=Homo sapiens GN=RPL11 PE=1 SV=2	7.34	9.95	8.61	7.70	15.84	13.71	9.92	0.00	2.19	10.75	9.12	2.94	3.76	5.78	8.59	2.69	2.02	5.52	6.51	0.00	0.00	2.88	5.11	2.32	8.17	3.77	0.415712745	2.2
P63244	Guanine nucleotide-binding protein subunit beta-2-like 1 OS=Homo sapiens GN=GNB2L1 PE=1 SV=3	4.08	14.92	13.39	18.60	16.40	24.58	17.78	2.80	0.00	10.37	16.36	7.10	16.70	11.40	7.39	5.22	7.09	6.60	12.84	0.00	1.52	3.43	14.76	6.68	12.20	7.80	0.417552088	1.6
P0CF74	Ig lambda-6 chain C region OS=Homo sapiens GN=IGLC6 PE=4 SV=1	0.00	46.83	44.70	0.00	0.00	0.00	0.00	0.00	0.00	0.00	0.00	0.00	0.00	0.00	0.00	0.00	0.00	0.00	0.00	0.00	0.00	38.74	0.00	0.00	7.63	3.23	0.417552088	2.4
P52597	Heterogeneous nuclear ribonucleoprotein F OS=Homo sapiens GN=HNRNPF PE=1 SV=3	4.69	5.49	9.80	11.12	7.73	14.89	7.01	4.70	3.15	2.78	13.68	2.61	0.00	5.14	9.78	2.52	0.89	1.07	4.16	0.00	1.18	5.03	4.54	0.92	7.30	2.94	0.429794993	2.5
Q9BTT0	Acidic leucine-rich nuclear phosphoprotein 32 family member E OS=Homo sapiens GN=ANP32E PE=1 SV=1	0.00	1.89	16.02	10.90	9.62	12.05	1.88	0.00	0.00	6.67	5.66	4.16	0.00	0.00	8.59	0.00	1.39	0.00	0.00	0.00	1.86	0.00	3.26	1.44	5.74	1.38	0.432516778	4.2
P35268	60S ribosomal protein L22 OS=Homo sapiens GN=RPL22 PE=1 SV=2	4.69	3.95	0.00	10.90	16.03	20.09	14.53	4.61	6.98	4.26	8.02	6.93	5.41	13.65	5.49	0.00	2.91	0.00	9.22	0.00	3.88	4.07	3.26	0.92	8.42	4.07	0.436147498	2.1
P05783	Keratin, type I cytoskeletal 18 OS=Homo sapiens GN=KRT18 PE=1 SV=2	2.85	0.00	11.71	2.99	13.20	22.21	6.67	2.71	4.11	5.56	14.78	4.81	3.29	0.00	7.15	0.00	1.77	3.38	8.86	0.00	3.71	1.76	8.37	1.20	7.63	3.29	0.437128515	2.3
P04406	Glyceraldehyde-3-phosphate dehydrogenase OS=Homo sapiens GN=GAPDH PE=1 SV=3	37.93	31.91	44.46	39.77	64.86	56.95	52.32	16.80	22.19	39.27	48.12	19.66	56.68	29.86	50.56	45.76	34.42	41.73	43.59	24.15	31.40	21.73	30.08	12.25	39.52	35.18	0.438565139	1.1
Q7L266	L-asparaginase OS=Homo sapiens GN=ASRGL1 PE=1 SV=2	16.31	8.23	8.13	13.47	11.88	11.34	10.77	4.33	6.57	14.82	7.55	3.92	5.17	5.46	15.03	5.72	2.78	5.22	8.68	2.86	0.00	3.83	8.94	1.92	9.78	5.47	0.450861096	1.8
P22314	Ubiquitin-like modifier-activating enzyme 1 OS=Homo sapiens GN=UBA1 PE=1 SV=3	12.85	10.81	17.21	16.46	12.63	21.74	14.88	5.33	6.85	10.00	14.47	6.28	9.88	10.12	14.07	9.09	6.83	10.28	12.12	2.34	6.41	5.03	9.51	2.52	12.46	8.18	0.459489944	1.5
P15121	Aldose reductase OS=Homo sapiens GN=AKR1B1 PE=1 SV=3	3.87	9.43	16.49	8.98	7.92	9.92	3.25	0.00	2.60	12.78	4.72	1.55	0.00	4.82	7.15	3.20	1.14	2.92	1.63	0.00	0.00	5.51	2.70	1.20	6.79	2.52	0.459489944	2.7
P78371	T-complex protein 1 subunit beta OS=Homo sapiens GN=CCT2 PE=1 SV=4	6.53	6.69	22.47	15.82	10.56	13.23	9.58	2.26	3.42	8.71	8.81	2.61	4.23	6.26	11.21	9.42	3.16	4.91	7.05	0.00	1.01	3.75	6.67	1.88	9.22	4.96	0.459489944	1.9
P04792	Heat shock protein beta-1 OS=Homo sapiens GN=HSPB1 PE=1 SV=2	32.63	16.81	23.43	20.95	37.52	57.18	27.36	17.97	21.91	23.53	31.30	13.05	23.05	25.69	30.29	26.92	20.25	19.49	28.94	20.77	33.59	12.78	22.70	7.97	26.97	22.70	0.459489944	1.2
Q3LXA3	Bifunctional ATP-dependent dihydroxyacetone kinase/FAD-AMP lyase (cyclizing) OS=Homo sapiens GN=DAK PE=	2.24	5.32	9.08	11.55	4.53	17.01	1.88	0.99	1.51	8.52	8.49	1.47	0.00	0.96	7.39	3.03	0.00	2.76	0.00	0.00	0.00	1.44	5.39	0.24	6.05	1.77	0.459489944	3.4
O75223	Gamma-glutamylcyclotransferase OS=Homo sapiens GN=GGCT PE=1 SV=1	6.93	5.83	19.12	17.10	10.37	25.76	13.68	4.97	7.53	20.19	17.14	2.77	8.00	8.83	13.12	9.25	6.96	8.44	9.95	2.08	9.28	8.71	15.47	0.00	12.62	8.34	0.459489944	1.5
Q14011	Cold-inducible RNA-binding protein OS=Homo sapiens GN=CIRBP PE=1 SV=1	3.67	6.69	9.32	3.85	12.26	15.36	11.11	3.52	5.34	7.23	10.22	3.18	0.00	2.89	4.29	3.03	2.28	2.76	7.05	2.34	0.00	5.19	9.22	1.56	7.65	3.38	0.459489944	2.3
Q99873	Protein arginine N-methyltransferase 1 OS=Homo sapiens GN=PRMT1 PE=1 SV=2	1.63	2.74	8.37	14.97	6.60	13.47	5.98	1.44	2.19	6.48	11.01	2.04	0.00	1.28	8.35	4.21	1.01	1.23	6.33	0.00	0.00	2.00	1.14	0.00	6.41	2.13	0.459489944	3.0
O14737	Programmed cell death protein 5 OS=Homo sapiens GN=PDCD5 PE=1 SV=3	0.00	4.12	12.67	11.33	4.53	12.52	0.00	0.00	0.00	0.00	8.34	1.96	0.00	0.00	0.00	0.00	0.00	0.00	0.00	0.00	0.00	0.00	3.41	0.96	4.62	0.36	0.459489944	12.7
P01620	Ig kappa chain V-III region SIE OS=Homo sapiens PE=1 SV=1	23.25	19.56	15.78	14.11	0.00	26.94	30.09	10.30	24.10	0.00	0.00	5.38	84.44	18.30	27.19	19.18	14.42	0.00	20.62	14.80	0.00	9.11	9.37	2.64	14.13	18.34	0.482747187	0.8
P05455	Lupus La protein OS=Homo sapiens GN=SSB PE=1 SV=2	10.20	3.95	11.95	6.63	7.54	14.41	8.55	2.80	5.48	13.34	6.29	4.98	5.41	6.42	7.39	2.52	2.91	2.30	5.61	1.95	1.18	3.20	5.68	1.24	8.01	3.82	0.492623889	2.1
Q8WUM4	Programmed cell death 6-interacting protein OS=Homo sapiens GN=PDCD6IP PE=1 SV=1	8.16	9.26	15.54	11.55	11.12	15.36	9.23	1.99	2.47	9.08	10.22	3.26	5.17	4.98	10.49	6.73	0.38	2.76	7.23	0.39	3.04	5.59	9.22	1.04	8.94	4.75	0.494476214	1.9
P25815	Protein S100-P OS=Homo sapiens GN=S100P PE=1 SV=2	27.73	23.33	18.41	29.08	25.64	18.20	23.25	6.95	10.55	25.20	21.39	6.28	18.11	12.36	32.44	22.88	9.74	11.81	24.60	10.00	13.00	6.15	19.30	5.44	19.67	15.49	0.494476214	1.3
Q15631	Translin OS=Homo sapiens GN=TSN PE=1 SV=1	5.51	14.07	10.28	13.04	11.50	14.41	7.35	2.44	1.78	11.30	6.76	1.06	3.06	4.34	10.26	2.19	1.64	4.14	11.03	0.00	4.56	3.43	3.83	1.08	8.29	4.13	0.502776081	2.0
Q14558	Phosphoribosyl pyrophosphate synthase-associated protein 1 OS=Homo sapiens GN=PRPSAP1 PE=1 SV=2	1.63	1.37	14.82	8.12	3.21	11.58	1.37	0.00	0.00	7.04	7.71	0.65	0.00	1.28	1.91	1.35	0.00	0.00	0.00	0.00	0.00	3.04	0.00	0.00	4.79	0.63	0.502776081	7.6
P20962	Parathymosin OS=Homo sapiens GN=PTMS PE=1 SV=2	6.12	5.15	7.17	6.41	13.01	28.12	11.80	2.71	4.11	12.78	4.72	2.45	7.06	4.82	7.15	5.05	3.80	4.60	5.43	3.89	5.06	2.40	4.26	1.20	8.71	4.56	0.504981483	1.9
P07203	Glutathione peroxidase 1 OS=Homo sapiens GN=GPX1 PE=1 SV=4	10.60	2.57	17.93	21.59	9.80	12.29	12.82	1.35	2.05	5.93	8.18	4.24	3.53	8.35	17.89	5.38	1.90	2.30	5.79	0.00	0.00	2.56	10.64	1.28	9.11	4.97	0.507699786	1.8
Q15257	Serine/threonine-protein phosphatase 2A activator OS=Homo sapiens GN=PPP2R4 PE=1 SV=3	3.47	6.18	14.10	12.61	8.86	17.01	8.04	0.72	3.56	6.67	7.39	2.94	0.00	2.73	8.59	6.06	3.29	3.99	6.51	1.04	1.35	3.75	3.69	1.04	7.63	3.50	0.51648067	2.2
P36871	Phosphoglucomutase-1 OS=Homo sapiens GN=PGM1 PE=1 SV=3	8.97	10.29	16.26	14.54	8.30	18.20	5.13	5.42	1.51	5.56	9.44	4.16	5.41	4.82	10.49	1.85	3.80	5.68	1.99	0.65	5.06	7.67	8.51	2.40	8.98	4.86	0.517227192	1.8
O60664	Perilipin-3 OS=Homo sapiens GN=PLIN3 PE=1 SV=3	4.49	3.77	11.95	13.04	11.50	14.41	6.84	2.80	3.01	5.74	9.59	1.79	3.29	2.25	9.54	3.70	0.89	1.07	1.27	0.91	5.23	3.20	7.10	1.24	7.41	3.31	0.52563206	2.2
P47755	F-actin-capping protein subunit alpha-2 OS=Homo sapiens GN=CAPZA2 PE=1 SV=3	9.38	16.30	14.58	24.37	8.67	14.41	7.87	2.98	2.88	6.11	7.23	6.28	4.94	5.30	18.36	5.55	0.00	5.06	13.93	0.00	5.57	4.87	6.53	1.84	10.09	6.00	0.530004099	1.7
P28066	Proteasome subunit alpha type-5 OS=Homo sapiens GN=PSMA5 PE=1 SV=3	8.77	13.89	19.36	17.32	8.11	10.16	7.35	5.51	1.78	11.30	6.76	6.61	14.35	6.90	10.26	7.23	5.44	4.14	7.78	0.00	0.00	4.87	6.10	1.08	9.74	5.68	0.542268654	1.7
P61106	Ras-related protein Rab-14 OS=Homo sapiens GN=RAB14 PE=1 SV=4	9.79	11.66	3.35	10.26	12.82	21.74	11.63	2.71	4.11	8.89	14.47	2.45	7.06	7.71	11.45	8.08	1.77	4.60	8.68	1.82	0.00	2.40	9.65	1.92	9.49	5.43	0.542268654	1.7
P60866	40S ribosomal protein S20 OS=Homo sapiens GN=RPS20 PE=1 SV=1	5.10	9.78	13.63	12.19	10.75	5.91	0.00	0.00	0.00	4.63	3.93	0.00	0.00	9.15	5.96	0.00	0.00	0.00	0.00	0.00	0.00	2.00	0.00	0.00	5.49	1.43	0.542268654	3.9
P31939	Bifunctional purine biosynthesis protein PURH OS=Homo sapiens GN=ATIC PE=1 SV=3	4.49	5.83	17.21	13.68	9.05	11.34	12.31	3.70	2.19	11.86	15.57	3.34	3.76	6.58	13.36	4.71	2.02	4.30	7.42	0.00	0.00	5.11	12.77	1.92	9.22	5.16	0.546239439	1.8
P78417	Glutathione S-transferase omega-1 OS=Homo sapiens GN=GSTO1 PE=1 SV=2	11.62	13.04	13.63	31.43	14.33	22.92	13.00	5.15	5.48	14.08	19.03	6.20	9.41	6.42	18.13	12.79	7.21	11.66	10.31	9.87	9.62	6.07	17.17	3.04	14.16	10.14	0.56582328	1.4
Q15370	Transcription elongation factor B polypeptide 2 OS=Homo sapiens GN=TCEB2 PE=1 SV=1	0.00	16.98	13.86	12.40	10.94	6.14	4.45	0.00	0.00	4.82	9.12	0.00	0.00	4.17	13.83	0.00	3.29	0.00	0.00	0.00	0.00	0.00	8.23	1.04	6.56	2.55	0.566661705	2.6
P21964	Catechol O-methyltransferase OS=Homo sapiens GN=COMT PE=1 SV=2	13.87	11.66	20.56	14.54	12.82	16.07	8.72	4.61	4.93	6.67	13.53	5.55	12.00	1.77	16.22	14.47	4.56	5.52	12.30	1.43	1.86	5.43	7.24	2.72	11.13	7.13	0.570000285	1.6
P01781	Ig heavy chain V-III region GAL OS=Homo sapiens PE=1 SV=1	12.24	4.63	0.00	0.00	5.09	0.00	0.00	0.00	0.00	0.00	0.00	2.20	14.11	4.34	0.00	10.09	7.59	9.21	4.88	7.79	4.56	0.00	8.51	1.08	2.01	6.01	0.570000285	0.3
Q99471	Prefoldin subunit 5 OS=Homo sapiens GN=PFDN5 PE=1 SV=2	14.27	7.38	10.28	14.97	8.11	16.54	7.35	0.00	5.89	7.97	2.99	1.55	0.00	6.90	10.26	7.23	2.40	6.60	7.78	0.00	0.00	1.52	6.10	0.76	8.11	4.13	0.577010473	2.0
Q9H910	Hematological and neurological expressed 1-like protein OS=Homo sapiens GN=HN1L PE=1 SV=1	3.47	6.18	8.61	7.70	6.79	8.51	6.16	1.54	2.33	6.67	5.66	2.94	0.00	2.73	4.05	0.00	0.00	2.61	0.00	0.00	0.00	2.88	5.11	1.44	5.54	1.57	0.577010473	3.5
O94760	N(G),N(G)-dimethylarginine dimethylaminohydrolase 1 OS=Homo sapiens GN=DDAH1 PE=1 SV=3	0.00	6.00	15.54	17.75	2.07	11.82	1.88	4.52	3.01	9.26	16.04	1.79	0.00	5.62	11.92	3.70	1.39	3.38	0.00	0.00	0.00	6.63	9.22	0.00	7.47	3.49	0.577010473	2.1
Q13838	Spliceosome RNA helicase DDX39B OS=Homo sapiens GN=DDX39B PE=1 SV=1	7.75	6.52	13.63	8.12	8.86	18.67	6.50	4.24	6.44	5.37	7.39	3.10	8.94	3.37	5.01	3.53	0.00	4.45	6.87	2.73	3.54	3.75	5.39	1.52	8.05	4.09	0.588238462	2.0

O95968	Secretoglobin family 1D member 1 OS=Homo sapiens GN=SCGB1D1 PE=1 SV=1	0.00	0.00	8.13	0.00	0.00	0.00	0.00	3.07	0.00	0.00	0.00	0.00	32.93	0.00	8.11	0.00	0.00	5.22	0.00	4.41	0.00	0.00	4.82	3.16	0.93	4.89	0.588238462	0.2
Q12905	Interleukin enhancer-binding factor 2 OS=Homo sapiens GN=ILF2 PE=1 SV=2	5.10	2.74	13.15	7.27	6.41	13.00	7.52	2.26	1.10	8.15	8.65	3.59	1.88	2.57	3.82	2.69	2.02	2.45	6.15	1.04	1.35	2.72	3.55	1.36	6.58	2.63	0.591954716	2.5
P17174	Aspartate aminotransferase, cytoplasmic OS=Homo sapiens GN=GOT1 PE=1 SV=3	8.36	12.52	14.82	15.61	7.73	9.69	7.01	2.89	2.05	7.60	8.02	5.06	1.65	6.58	12.16	3.87	0.89	3.53	4.16	0.91	3.88	6.87	7.24	2.48	8.45	4.52	0.598890112	1.9
O75832	26S proteasome non-ATPase regulatory subunit 10 OS=Homo sapiens GN=PSMD10 PE=1 SV=1	2.85	7.89	15.78	9.83	5.47	10.87	4.96	0.00	0.00	8.52	10.38	1.14	0.00	2.25	3.34	0.00	1.77	2.15	5.25	0.00	0.00	5.27	9.37	1.16	6.47	2.55	0.598890112	2.5
P48506	Glutamate--cysteine ligase catalytic subunit OS=Homo sapiens GN=GCLC PE=1 SV=2	3.87	9.43	16.49	14.75	13.01	21.98	7.18	4.97	1.23	15.56	17.30	6.20	3.29	8.83	20.03	7.07	6.96	7.36	8.68	0.52	0.68	7.43	11.92	2.20	11.00	7.08	0.603811022	1.6
P52895	Aldo-keto reductase family 1 member C2 OS=Homo sapiens GN=AKR1C2 PE=1 SV=3	10.81	0.00	23.43	20.95	21.87	0.00	0.00	0.00	0.00	0.00	28.15	9.46	23.05	10.76	37.21	0.00	14.68	0.00	24.42	0.00	0.00	14.30	28.95	8.17	9.56	13.46	0.607444907	0.7
Q96K17	Transcription factor BTF3 homolog 4 OS=Homo sapiens GN=BTF3L4 PE=1 SV=1	4.08	3.43	10.28	0.00	8.11	10.16	7.35	1.81	0.00	7.97	6.76	0.00	0.00	0.00	10.26	0.00	0.00	0.00	0.00	0.00	0.00	0.00	2.84	0.00	5.00	1.09	0.607444907	4.6
P25789	Proteasome subunit alpha type-4 OS=Homo sapiens GN=PSMA4 PE=1 SV=1	7.55	6.35	16.73	19.03	13.20	16.54	11.97	4.79	5.07	12.97	14.00	7.26	5.64	11.24	21.23	18.51	0.00	5.68	9.59	3.12	1.86	2.96	7.52	1.48	11.29	7.40	0.617627536	1.5
Q03013	Glutathione S-transferase Mu 4 OS=Homo sapiens GN=GSTM4 PE=1 SV=3	2.65	0.00	0.00	5.99	11.88	14.89	4.79	0.00	1.78	11.67	9.91	1.06	3.06	0.00	3.10	0.00	0.00	4.30	0.00	0.00	2.19	1.04	3.97	0.52	5.38	1.52	0.626188022	3.6
Q7Z5L0	Vitelline membrane outer layer protein 1 homolog OS=Homo sapiens GN=VMO1 PE=1 SV=1	6.73	2.57	7.89	3.21	2.83	7.80	5.64	1.35	4.52	6.11	5.19	1.22	0.00	0.00	0.00	0.00	0.00	2.30	0.00	0.00	2.53	1.20	2.13	0.60	4.59	0.73	0.631890751	6.3
P51149	Ras-related protein Rab-7a OS=Homo sapiens GN=RAB7A PE=1 SV=1	6.12	5.15	28.69	19.88	5.66	11.34	5.13	6.23	4.11	5.56	7.55	5.63	3.29	2.25	7.15	15.65	1.77	2.15	12.48	0.00	2.36	7.43	9.79	0.56	9.25	5.41	0.634516018	1.7
P62277	40S ribosomal protein S13 OS=Homo sapiens GN=RPS13 PE=1 SV=2	4.08	3.43	10.28	9.19	8.11	10.16	3.42	0.00	5.89	7.97	6.76	5.79	0.00	0.00	10.26	3.36	2.53	6.60	0.00	0.00	0.00	1.60	2.84	1.72	6.26	2.41	0.634516018	2.6
Q16881	Thioredoxin reductase 1, cytoplasmic OS=Homo sapiens GN=TXNRD1 PE=1 SV=3	6.32	10.98	22.95	10.69	16.40	16.78	9.58	5.06	3.42	13.15	12.42	5.22	4.70	8.03	18.84	8.41	8.10	7.67	9.04	1.82	0.84	6.31	11.21	2.00	11.08	7.25	0.642075724	1.5
P35579	Myosin-9 OS=Homo sapiens GN=MYH9 PE=1 SV=4	16.72	6.35	13.15	17.96	17.35	17.01	12.65	2.80	3.97	8.71	10.85	5.47	13.64	5.94	14.79	9.76	5.69	6.60	8.86	3.38	4.89	2.96	9.22	1.80	11.08	7.29	0.669647706	1.5
P36952	Serpin B5 OS=Homo sapiens GN=SERPINB5 PE=1 SV=2	9.18	6.00	1.91	5.34	4.71	5.91	4.27	3.16	6.16	0.00	5.50	6.69	3.76	4.01	10.73	13.80	7.21	10.59	17.36	5.84	11.65	10.22	6.39	2.76	4.90	8.69	0.669647706	0.6
P22532	Small proline-rich protein 2D OS=Homo sapiens GN=SPRR2D PE=2 SV=2	0.00	0.00	0.00	0.00	0.00	0.00	0.00	0.00	0.00	0.00	0.00	0.00	0.00	0.00	0.00	0.00	0.00	45.26	0.00	0.00	0.00	0.00	0.00	0.00	0.00	3.77	0.679405353	0.0
P30711	Glutathione S-transferase theta-1 OS=Homo sapiens GN=GSTT1 PE=1 SV=4	0.00	13.21	0.00	8.77	0.00	9.69	4.45	0.00	0.00	10.75	1.89	0.00	0.00	0.00	0.00	0.00	0.00	0.00	0.00	0.00	0.00	0.00	3.69	0.00	4.06	0.31	0.688645076	13.2
P23381	Tryptophan--tRNA ligase, cytoplasmic OS=Homo sapiens GN=WARS PE=1 SV=2	16.72	2.23	6.45	9.19	24.70	3.07	14.02	0.54	4.79	3.71	3.15	8.56	19.29	0.96	3.10	3.36	6.58	1.99	7.78	0.78	3.38	1.60	1.84	1.72	8.10	4.37	0.700866494	1.9
P35998	26S protease regulatory subunit 7 OS=Homo sapiens GN=PSMC2 PE=1 SV=3	4.49	6.69	9.32	10.26	10.94	16.07	5.13	0.63	1.92	5.56	9.12	4.73	3.29	2.25	9.30	3.70	0.00	0.00	8.68	0.00	1.18	3.83	5.53	2.32	7.07	3.34	0.700866494	2.1
P32969	60S ribosomal protein L9 OS=Homo sapiens GN=RPL9 PE=1 SV=1	3.06	0.00	7.89	3.21	9.99	17.96	0.00	0.00	0.00	9.82	2.36	1.22	3.53	0.00	0.00	2.52	1.90	0.00	0.00	0.00	0.00	0.00	2.13	0.60	4.63	0.89	0.700866494	5.2
Q9Y2Z0	Suppressor of G2 allele of SKP1 homolog OS=Homo sapiens GN=SUGT1 PE=1 SV=3	1.63	8.06	11.24	7.70	6.79	6.14	4.45	0.00	0.00	8.71	4.09	0.65	1.88	2.73	4.05	0.00	0.00	0.00	0.00	0.00	0.00	0.64	5.11	0.68	4.95	1.26	0.722671844	3.9
Q14764	Major vault protein OS=Homo sapiens GN=MVP PE=1 SV=4	8.77	3.60	11.24	19.46	13.76	13.47	7.35	0.90	0.41	10.56	8.96	7.42	5.88	6.10	14.79	9.59	7.21	3.22	7.78	0.91	0.00	1.68	2.55	2.08	8.83	5.15	0.737007935	1.7
Q01105	Protein SET OS=Homo sapiens GN=SET PE=1 SV=3	12.24	10.29	7.89	12.83	17.72	10.87	10.26	2.98	6.30	8.52	7.23	6.20	10.82	7.39	14.31	7.74	4.18	3.22	10.85	0.00	5.57	0.00	2.98	2.40	9.44	5.79	0.750217938	1.6
P62195	26S protease regulatory subunit 8 OS=Homo sapiens GN=PSMC5 PE=1 SV=1	6.53	3.95	10.04	6.84	9.80	14.89	7.18	0.63	0.96	5.93	8.18	2.61	3.53	0.00	5.49	7.07	2.91	3.53	4.16	0.00	0.00	1.84	4.54	0.92	6.46	2.83	0.766184691	2.3
Q8TD33	Secretoglobin family 1C member 1 OS=Homo sapiens GN=SCGB1C1 PE=2 SV=2	0.00	5.66	7.89	7.06	6.22	7.80	5.64	6.86	4.52	6.11	0.00	2.69	0.00	5.30	0.00	0.00	0.00	0.00	0.00	4.28	0.00	2.64	4.68	0.00	5.04	1.41	0.766184691	3.6
Q9UJ70	N-acetyl-D-glucosamine kinase OS=Homo sapiens GN=NAGK PE=1 SV=4	10.60	8.92	15.78	17.10	12.44	15.60	4.96	1.63	0.00	9.63	12.58	1.47	6.82	8.35	19.08	6.73	1.14	2.76	7.23	1.17	1.52	4.15	7.38	1.16	9.23	5.62	0.78605788	1.6
O00232	26S proteasome non-ATPase regulatory subunit 12 OS=Homo sapiens GN=PSMD12 PE=1 SV=3	4.08	6.00	8.37	11.12	5.09	12.29	5.98	1.81	2.74	3.71	5.50	1.63	0.00	0.96	6.44	3.36	0.76	0.92	2.35	0.78	1.01	2.80	4.97	1.08	5.69	2.12	0.806329935	2.7
O14818	Proteasome subunit alpha type-7 OS=Homo sapiens GN=PSMA7 PE=1 SV=1	15.29	12.87	17.93	20.52	14.14	22.68	16.41	3.61	10.27	17.78	11.80	4.65	13.41	19.11	17.89	12.62	5.06	11.51	10.31	5.19	4.22	5.99	16.89	3.00	14.00	10.43	0.813300765	1.3
P06727	Apolipoprotein A-IV OS=Homo sapiens GN=APOA4 PE=1 SV=3	34.05	10.81	24.38	35.70	28.09	20.79	22.57	13.46	22.87	21.49	18.24	13.62	39.28	26.81	35.54	61.91	18.85	15.65	30.20	29.86	19.58	9.27	18.73	3.00	22.17	25.72	0.817850221	0.9
P49189	4-trimethylaminobutyraldehyde dehydrogenase OS=Homo sapiens GN=ALDH9A1 PE=1 SV=3	3.87	4.46	8.13	5.56	6.41	14.18	7.18	2.35	1.64	9.45	8.02	2.12	0.00	4.17	4.53	3.20	2.40	1.84	4.70	0.00	0.00	4.07	4.82	1.04	6.11	2.57	0.817850221	2.4
Q02543	60S ribosomal protein L18a OS=Homo sapiens GN=RPL18A PE=1 SV=2	3.26	0.00	8.37	0.00	15.27	8.27	5.98	0.00	0.00	6.48	5.50	1.31	3.76	2.57	0.00	0.00	2.02	0.00	2.89	0.00	0.00	0.00	0.00	0.64	4.54	0.99	0.817850221	4.6
Q9NR45	Sialic acid synthase OS=Homo sapiens GN=NANS PE=1 SV=2	9.79	10.29	20.80	21.81	13.76	17.25	12.48	3.34	6.57	11.12	9.44	2.12	14.11	7.71	17.41	8.08	6.07	5.68	6.69	4.80	2.87	6.95	14.47	1.48	11.56	8.03	0.819731209	1.4
P11216	Glycogen phosphorylase, brain form OS=Homo sapiens GN=PYGB PE=1 SV=5	7.95	6.00	11.71	14.97	10.18	15.12	7.52	1.26	4.11	5.56	10.07	4.40	6.12	5.62	11.69	7.40	2.78	3.99	7.96	0.00	0.51	4.31	4.26	1.76	8.24	4.70	0.819731209	1.8
P15374	Ubiquitin carboxyl-terminal hydrolase isozyme L3 OS=Homo sapiens GN=UCHL3 PE=1 SV=1	5.51	4.63	14.58	9.19	11.50	10.16	10.43	1.17	1.78	5.00	4.25	4.98	0.00	4.34	10.26	7.23	1.64	1.99	2.35	0.00	0.00	3.43	8.66	1.08	6.93	3.42	0.833940534	2.0
Q9UBI6	Guanine nucleotide-binding protein G(I)/G(S)/G(O) subunit gamma-12 OS=Homo sapiens GN=GNG12 PE=1 SV=3	0.00	7.38	0.00	0.00	8.11	10.16	0.00	0.00	0.00	7.97	6.76	3.51	0.00	0.00	0.00	0.00	0.00	0.00	0.00	0.00	0.00	0.00	0.00	1.72	3.66	0.14	0.833940534	25.5
P18669	Phosphoglycerate mutase 1 OS=Homo sapiens GN=PGAM1 PE=1 SV=2	34.05	9.43	17.45	24.59	31.49	17.25	15.90	8.40	10.00	13.52	14.63	11.42	21.87	22.48	22.18	15.65	9.24	11.20	20.80	7.14	9.28	5.83	16.32	4.60	17.34	13.88	0.874445988	1.2
P40227	T-complex protein 1 subunit zeta OS=Homo sapiens GN=CCT6A PE=1 SV=3	11.22	10.98	19.84	11.76	17.54	17.25	10.94	2.17	1.64	15.38	14.63	7.59	7.29	14.93	19.80	7.91	8.10	4.76	13.20	0.78	1.01	8.31	10.36	2.92	11.74	8.28	0.874445988	1.4
P12081	Histidine--tRNA ligase, cytoplasmic OS=Homo sapiens GN=HARS PE=1 SV=2	5.10	3.09	11.24	13.68	6.03	4.25	6.67	2.89	2.47	7.23	11.64	3.18	1.41	1.93	9.30	4.21	0.76	3.84	2.17	0.00	1.01	3.75	6.67	1.00	6.45	3.00	0.880364401	2.1
P00390	Glutathione reductase, mitochondrial OS=Homo sapiens GN=GSR PE=1 SV=2	8.16	8.23	15.78	16.25	7.54	15.60	6.84	2.26	1.64	12.23	11.95	5.38	5.88	7.71	13.59	8.08	6.07	6.14	8.68	0.78	0.00	3.83	8.09	1.92	9.32	5.90	0.897549063	1.6
P61077	Ubiquitin-conjugating enzyme E2 D3 OS=Homo sapiens GN=UBE2D3 PE=1 SV=1	8.97	3.43	10.52	4.28	8.30	17.25	3.42	0.00	2.74	8.15	6.92	3.59	0.00	3.21	10.49	7.40	2.53	0.00	0.00	0.00	0.00	5.83	6.24	0.80	6.46	3.04	0.897549063	2.1
P62942	Peptidyl-prolyl cis-trans isomerase FKBP1A OS=Homo sapiens GN=FKBP1A PE=1 SV=2	13.46	4.97	15.78	0.00	12.44	6.85	11.29	2.62	9.04	5.37	4.56	5.38	6.82	4.66	6.92	4.88	3.67	4.45	5.25	0.00	4.89	2.32	4.12	2.64	7.65	4.22	0.897549063	1.8
Q5TD94	Radial spoke head protein 4 homolog A OS=Homo sapiens GN=RSPH4A PE=1 SV=1	0.00	4.63	14.58	5.77	1.51	10.16	0.68	1.17	0.00	9.08	5.03	0.00	0.00	2.73	5.25	0.00	0.00	1.23	0.00	0.00	0.00	0.64	1.84	0.00	4.39	0.97	0.903486076	4.5
P62316	Small nuclear ribonucleoprotein Sm D2 OS=Homo sapiens GN=SNRPD2 PE=1 SV=1	0.00	4.29	13.39	20.31	10.56	13.23	0.00	2.26	0.00	10.37	8.81	2.04	0.00	4.01	22.66	4.21	0.00	0.00	4.52	0.00	0.00	0.00	7.95	1.00	7.10	3.70	0.903486076	1.9
Q15365	Poly(rC)-binding protein 1 OS=Homo sapiens GN=PCBP1 PE=1 SV=2	10.60	8.92	12.43	17.10	9.80	22.45	11.11	4.70	7.12	7.41	12.58	6.53	15.29	12.84	12.40	6.73	5.06	7.98	9.41	1.17	4.89	4.15	7.38	2.60	10.90	7.49	0.904928323	1.5
Q9H0W9	Ester hydrolase C11orf54 OS=Homo sapiens GN=C11orf54 PE=1 SV=1	1.84	1.54	7.41	6.63	3.58	10.16	3.25	0.81	1.23	5.74	6.76	0.00	0.00	0.00	2.15	0.00	0.00	1.38	1.63	0.00	0.00	0.72	2.70	0.00	4.08	0.71	0.939297769	5.7
Q9H3K6	BolA-like protein 2 OS=Homo sapiens GN=BOLA2 PE=1 SV=1	6.93	0.00	0.00	29.50	6.41	8.03	5.81	0.00	0.00	6.30	0.00	2.77	0.00	0.00	0.00	5.72	0.00	0.00	6.15	0.00	0.00	2.72	11.07	0.00	5.48	2.14	0.955066587	2.6
Q9UDX4	SEC14-like protein 3 OS=Homo sapiens GN=SEC14L3 PE=2 SV=1	0.00	2.57	23.43	0.00	0.00	5.43	0.00	0.00	0.00	7.60	1.10	0.00	0.00	0.00	0.00	0.00	0.00	0.00	0.00	0.00	0.00	0.00	0.00	0.00	3.34	0.00	0.955066587	#DIV/0!
O43776	Asparagine--tRNA ligase, cytoplasmic OS=Homo sapiens GN=NARS PE=1 SV=1	1.02	4.97	15.54	7.48	7.92	11.82	5.98	0.99	2.19	9.26	10.22	1.79	1.18	5.62	6.92	2.69	2.02	2.45	6.33	0.65	0.00	3.35	7.10	0.88	6.60	3.27	0.959794123	2.0
P00492	Hypoxanthine-guanine phosphoribosyltransferase OS=Homo sapiens GN=HPRT1 PE=1 SV=2	5.91	4.97	11.00	9.83	8.67	6.85	7.87	1.26	3.97	8.52	4.56	2.37	6.82	4.66	0.00	0.00	0.00	2.15	2.53	0.00	2.36	3.67	12.49	1.16	6.32	2.99	0.960501165	2.1
P01877	Ig alpha-2 chain C region OS=Homo sapiens GN=IGHA2 PE=1 SV=3	41.19	19.73	12.67	0.00	33.56	19.14	16.76	12.10	18.35	24.82	10.54	10.93	31.52	32.43	27.43	22.54	14.55	30.99	28.03	14.93	22.62	12.38	13.91	8.09	18.32	21.62	0.964075135	0.8
P13639	Elongation factor 2 OS=Homo sapiens GN=EEF2 PE=1 SV=4	15.70	11.15	18.41	12.83	13.39	19.61	17.44	5.87	9.72	10.00	14.00	9.46	12.70	7.06	15.50	12.95	8.98	9.97	16.10	4.54	8.27	5.19	12.63	4.08	13.13	9.83	0.964075135	1.3
P00739	Haptoglobin-related protein OS=Homo sapiens GN=HPR PE=1 SV=2	12.64	0.00	0.00	0.00	16.97	0.00	0.00	8.13	0.00	0.00	0.00	0.00	0.00	0.00	0.00	12.79	13.41	0.00	11.21	15.97	15.19	0.00	8.80	0.00	3.15	6.45	0.964075135	0.5
P62263	40S ribosomal protein S14 OS=Homo sapiens GN=RPS14 PE=1 SV=3	9.18	12.87	10.76	9.62	8.48	10.63	7.69	0.00	0.00	13.89	11.80	3.67	10.58	7.23	10.73	7.57	2.66	3.22	0.00	2.73	0.00	5.99	6.39	1.80	8.22	4.91	0.964075135	1.7
P02654	Apolipoprotein C-I OS=Homo sapiens GN=APOC1 PE=1 SV=1	7.55	0.00	0.00	0.00	0.00	0.00	0.00	3.34	5.07	0.00	0.00	3.02	8.70	5.94	0.00	6.23	4.68	5.68	6.69	11.55	6.25	2.96	0.00	0.00	1.58	4.89	0.964075135	0.3
P20618	Proteasome subunit beta type-1 OS=Homo sapiens GN=PSMB1 PE=1 SV=2	0.00	2.06	6.22	17.10	7.92	9.92	4.45	0.00	1.64	4.82	9.44	3.43	2.82	0.00	6.20	4.37	1.52	0.00	2.17	0.00	2.03	3.35	3.69	1.04	5.58	2.27	0.964075135	2.5
P01619	Ig kappa chain V-III region B6 OS=Homo sapiens PE=1 SV=1	0.00	0.00	0.00	0.00	0.00	0.00	0.00	0.00	0.00	0.00	4.56	0.00	0.00	0.00	27.67	11.27	0.00	0.00	0.00	0.00	0.00	5.35	0.00	0.00	0.38	3.69	0.964075135	0.1
O94811	Tubulin polymerization-promoting protein OS=Homo sapiens GN=TPPP PE=1 SV=1	0.00	8.23	16.49	2.99	5.66	16.30	2.39	1.26	0.00	8.89	2.20	1.14	0.00	4.82	11.45	0.00	0.00	0.00	0.00	0.00	0.00	2.40	6.81	0.56	5.46	2.17	0.96888256	2.5
Q8NCW5	NAD(P)H-hydrate epimerase OS=Homo sapiens GN=APOA1BP PE=1 SV=2	0.00	1.72	15.30	4.70	6.60	5.20	5.98	0.00	0.00	4.08	10.07	0.82	0.00	0.00	8.35	1.68	0.00	0.00	1.81	0.00	0.00	1.76	1.42	0.00	4.54	1.25	0.970472695	3.6
Q9UBQ7	Glyoxylate reductase/hydroxypyruvate reductase OS=Homo sapiens GN=GRHPR PE=1 SV=1	8.56	5.15	10.04	8.98	5.66	9.92	3.25	0.81	0.00	10.19	4.72	3.43	0.00	4.82	7.15	3.20	1.14	4.60	3.44	0.00	1.52	0.72	2.70	2.20	5.89	2.62	0.979350711	2.2
P49368	T-complex protein 1 subunit gamma OS=Homo sapiens GN=CCT3 PE=1 SV=4	3.47	3.95	10.52	6.41	9.80	12.29	7.52	1.54	0.00	12.78	10.85	4.89	5.41	3.69	10.49	2.86	3.80	4.60	4.16	0.65	0.00	2.40	5.25	1.48	7.00	3.73	0.979350711	1.9
P01037	Cystatin-SN OS=Homo sapiens GN=CST1 PE=1 SV=3	0.00	0.00	0.00	0.00	0.00	0.00	0.00	0.00	20.82	0.00	0.00	0.00	35.75	0.00	0.00	3.36	0.00	0.00	13.38	0.00	7.60	0.00	0.00	0.00	1.73	5.01	0.979350711	0.3
P25311	Zinc-alpha-2-glycoprotein OS=Homo sapiens GN=AZGP1 PE=1 SV=2	9.18	12.69	10.76	9.62	8.48	10.63	9.92	8.13	7.94	10.75	7.08	14.27	25.64	14.45	13.83	12.45	9.36	16.72	16.28	9.61	12.49	10.30	10.50	7.00	9.95	13.22	0.979351963	0.8
O75874	Isocitrate dehydrogenase [NADP] cytoplasmic OS=Homo sapiens GN=IDH1 PE=1 SV=2	10.20	6.86	23.19	15.39	13.58	22.92	14.36	4.52	11.50	17.97	15.25	6.85	14.35	13.49	20.03	8.41	9.11	7.67	13.02	7.92	8.44	5.75	11.92	3.36	13.55	10.29	0.980191115	1.3
Q9Y5Z4	Heme-binding protein 2 OS=Homo sapiens GN=HEBP2 PE=1 SV=1	10.40	12.52	17.45	6.63	13.76	17.25	8.72	2.80	4.25	9.45	11.48	4.16	7.29	8.19	12.16	8.58	3.92	7.82	9.22	4.02	5.23	4.07	7.24	2.04	9.91	6.65	0.980191115	1.5
P20810	Calpastatin OS=Homo sapiens GN=CAST PE=1 SV=4	4.69	6.86	9.56	8.55	4.34	10.87	3.93	1.17	1.78	10.75	7.23	3.26	3.06	2.89	8.11	3.03	2.28	3.53	5.06	0.52	0.68	2.24	1.84	0.72	6.08	2.83	0.981840104	2.2
Q06323	Proteasome activator complex subunit 1 OS=Homo sapiens GN=PSME1 PE=1 SV=1	28.55	24.02	33.47	29.93	26.40	33.08	23.94	12.64	19.17	21.30	22.02	13.62	27.05	22.48	33.39	33.31	11.77	17.64	35.81	5.06	19.41	15.82	23.70	3.72	24.01	20.76	0.982685049	1.2
P61604	10 kDa heat shock protein, mitochondrial OS=Homo sapiens GN=HSPE1 PE=1 SV=2	0.00	12.69	0.00	15.82	6.03	7.56	5.47	0.00	10.14	5.93	20.29	2.61	0.00	11.88	0.00	12.45	0.00	0.00	13.38	0.00	0.00	2.56	4.54	2.96	7.21	3.98	0.996598163	1.8
O95861	3'(2'),5'-bisphosphate nucleotidase 1 OS=Homo sapiens GN=BPNT1 PE=1 SV=1	0.00	3.60	5.02	9.83	6.03	10.87	5.47	1.90	2.88	3.89	3.30	0.82	0.00	1.61	2.38	0.00	0.00	4.91	1.81	0.00	0.00	0.80	2.98	0.40	4.47	1.24	0.997458428	3.6
Q9H0U4	Ras-related protein Rab-1B OS=Homo sapiens GN=RAB1B PE=1 SV=1	0.00	0.00	0.00	28.22	0.00	17.72	0.00	6.77	0.00	0.00	15.88	0.00	0.00	8.35	0.00	12.62	0.00	2.30	0.00	4.15	2.53	0.00	0.00	0.00	5.72	2.50	>0.999999999999999	2.3
P61313	60S ribosomal protein L15 OS=Homo sapiens GN=RPL15 PE=1 SV=2	6.12	0.00	3.35	6.41	8.86	11.11	5.13	1.26	0.00	5.56	2.20	1.14	0.00	0.00	3.34	2.36	3.80	0.00	0.00	0.00	0.00	1.12	1.99	0.00	4.26	1.05	>0.999999999999999	4.1
P02768	Serum albumin OS=Homo sapiens GN=ALB PE=1 SV=2	270.40	113.04	287.57	297.61	262.46	379.74	226.73	98.53	220.09	222.87	127.23	119.09	311.88	150.93	486.30	359.54	152.22	224.00	197.33	252.38	184.15	100.89	120.76	58.44	218.78	216.57	>0.999999999999999	1.0
P01024	Complement C3 OS=Homo sapiens GN=C3 PE=1 SV=2	30.18	13.04	19.60	14.32	29.60	24.10	31.63	16.26	26.02	22.97	17.77	23.98	42.34	24.57	29.57	26.41	24.04	24.70	32.56	28.04	36.46	11.50	18.73	6.44	22.46	25.45	>0.999999999999999	0.9
P01023	Alpha-2-macroglobulin OS=Homo sapiens GN=A2M PE=1 SV=3	19.37	7.72	9.56	16.46	22.44	8.74	18.30	7.86	17.53	10.56	5.35	8.08	27.05	13.49	20.03	23.89	17.97	13.35	17.18	14.93	16.71	5.83	9.51	1.92	12.66	15.15	>0.999999999999999	0.8
P01876	Ig alpha-1 chain C region OS=Homo sapiens GN=IGHA1 PE=1 SV=2	62.40	28.99	22.23	46.39	57.70	25.99	28.90	19.60	26.30	31.31	23.28	17.70	64.45	43.99	35.30	32.30	34.67	42.04	44.13	24.93	46.25	15.34	23.98	10.97	32.57	34.86	>0.999999999999999	0.9
P01833	Polymeric immunoglobulin receptor OS=Homo sapiens GN=PIGR PE=1 SV=4	32.42	30.88	18.41	22.66	24.70	16.54	25.48	13.46	24.65	25.94	20.60	14.68	37.40	23.92	25.28	22.04	20.12	22.86	28.76	21.94	25.15	11.90	19.87	7.60	22.53	22.24	>0.999999999999999	1.0
P02787	Serotransferrin OS=Homo sapiens GN=TF PE=1 SV=3	60.56	30.19	29.88	30.79	31.11	38.99	38.30	24.02	38.49	30.57	25.95	18.27	62.56	26.49	39.35	25.91	31.89	36.52	38.16	36.48	40.17	17.89	20.43	6.16	33.09	31.84	>0.999999999999999	1.0
P12814	Alpha-actinin-1 OS=Homo sapiens GN=ACTN1 PE=1 SV=2	12.03	6.18	13.15	18.39	8.48	13.00	13.85	3.70	4.38	8.34	5.66	4.81	16.46	5.78	11.92	8.41	5.19	6.90	11.76	6.49	6.92	5.19	5.82	1.80	9.33	7.72	>0.999999999999999	1.2
P11142	Heat shock cognate 71 kDa protein OS=Homo sapiens GN=HSPA8 PE=1 SV=1	25.69	13.72	21.04	20.74	36.96	29.77	23.25	9.57	17.26	21.49	16.67	9.46	27.28	10.28	25.28	21.20	18.60	26.08	19.17	13.76	16.37	10.86	13.76	4.64	20.47	17.27	>0.999999999999999	1.2
P34931	Heat shock 70 kDa protein 1-like OS=Homo sapiens GN=HSPA1L PE=1 SV=2	8.97	5.32	7.41	5.34	10.75	0.00	0.00	0.00	0.00	0.00	10.22	5.30	0.00	5.94	10.49	0.00	0.00	0.00	0.00	0.00	0.00	2.96	7.10	2.88	4.44	2.45	>0.999999999999999	1.8
P11021	78 kDa glucose-regulated protein OS=Homo sapiens GN=HSPA5 PE=1 SV=2	9.99	8.41	10.28	11.97	4.71	8.51	7.35	3.88	5.89	4.63	6.76	5.71	7.06	8.99	5.96	10.60	6.20	7.52	8.86	5.58	7.26	3.43	6.10	3.80	7.34	6.78	>0.999999999999999	1.1
Q58FF6	Putative heat shock protein HSP 90-beta 4 OS=Homo sapiens GN=HSP90AB4P PE=5 SV=1	4.89	4.12	5.74	6.63	5.85	5.67	5.30	2.17	3.29	4.45	4.88	2.53	5.64	3.85	7.39	5.22	3.92	2.76	5.61	2.34	3.04	1.92	4.40	1.20	4.62	3.94	>0.999999999999999	1.2
P14625	Endoplasmin OS=Homo sapiens GN=HSP90B1 PE=1 SV=1	2.24	1.20	0.96	1.50	1.32	3.54	2.56	0.99	0.55	0.74	2.36	4.65	2.59	1.77	1.67	1.85	0.89	2.30	2.71	0.91	2.53	0.88	3.26	1.48	1.88	1.90	>0.999999999999999	1.0
P01860	Ig gamma-3 chain C region OS=Homo sapiens GN=IGHG3 PE=1 SV=2	38.13	14.24	23.19	27.58	24.32	26.47	22.06	11.65	22.74	27.23	13.05	11.99	43.98	23.60	39.59	39.20	26.45	14.88	30.02	21.55	24.81	8.95	18.31	4.48	21.89	24.65	>0.999999999999999	0.9
P01859	Ig gamma-2 chain C region OS=Homo sapiens GN=IGHG2 PE=1 SV=2	48.33	23.67	16.26	21.38	18.86	23.63	14.36	7.59	21.91	15.56	13.21	6.85	37.63	29.54	32.91	30.96	20.25	18.10	24.96	17.92	19.92	9.43	16.74	4.72	19.30	21.92	>0.999999999999999	0.9
P01861	Ig gamma-4 chain C region OS=Homo sapiens GN=IGHG4 PE=1 SV=1	20.60	9.43	16.49	14.75	13.01	16.30	9.40	4.97	25.20	15.56	8.65	9.71	19.76	19.11	20.03	16.99	15.06	8.44	7.60	13.11	11.65	4.39	7.80	4.04	13.67	12.33	>0.999999999999999	1.1
P06396	Gelsolin OS=Homo sapiens GN=GSN PE=1 SV=1	21.00	11.84	22.95	13.47	22.44	24.34	21.89	12.28	16.30	16.49	17.46	9.71	20.93	13.17	21.23	14.97	13.03	17.03	17.36	17.66	20.09	8.87	15.75	3.28	17.51	15.28	>0.999999999999999	1.1
P60709	Actin, cytoplasmic 1 OS=Homo sapiens GN=ACTB PE=1 SV=1	43.03	22.30	31.08	31.64	24.51	26.70	36.08	13.37	25.75	27.42	23.28	23.66	55.27	23.76	26.95	21.87	21.13	28.84	30.20	24.41	48.95	16.85	21.00	5.92	27.40	27.10	>0.999999999999999	1.0
P09972	Fructose-bisphosphate aldolase C OS=Homo sapiens GN=ALDOC PE=1 SV=2	5.51	4.63	9.08	16.03	9.24	6.38	6.50	4.43	0.00	3.15	19.19	2.20	6.35	4.34	9.06	6.39	6.20	7.52	6.87	3.51	0.00	3.04	8.66	2.44	7.19	5.36	>0.999999999999999	1.3
P0C0L4	Complement C4-A OS=Homo sapiens GN=C4A PE=1 SV=1	7.14	0.00	5.74	4.28	7.92	0.00	0.00	5.15	0.00	9.63	4.09	0.00	8.23	0.00	12.40	0.00	0.00	0.00	0.00	0.00	7.43	0.00	0.00	0.00	3.66	2.34	>0.999999999999999	1.6
P02675	Fibrinogen beta chain OS=Homo sapiens GN=FGB PE=1 SV=2	22.02	5.66	5.98	10.48	25.08	11.58	9.75	5.96	11.78	7.41	6.29	5.38	22.81	12.20	11.69	16.32	10.88	13.19	15.55	15.71	24.81	3.20	13.76	1.60	10.61	13.48	>0.999999999999999	0.8
Q9Y490	Talin-1 OS=Homo sapiens GN=TLN1 PE=1 SV=3	5.10	2.23	4.06	5.77	3.77	5.67	3.59	0.45	1.51	1.67	2.83	2.69	4.00	1.61	4.05	3.03	0.63	2.15	3.26	0.78	1.52	1.36	2.84	0.84	3.28	2.17	>0.999999999999999	1.5
P02790	Hemopexin OS=Homo sapiens GN=HPX PE=1 SV=2	17.13	7.55	20.08	20.52	18.10	17.25	16.41	8.67	18.49	15.56	11.48	12.24	25.40	17.34	25.76	16.15	15.31	18.56	15.19	15.71	18.23	6.71	17.17	2.52	15.29	16.17	>0.999999999999999	0.9
Q04917	14-3-3 protein eta OS=Homo sapiens GN=YWHAH PE=1 SV=4	23.86	12.69	17.69	25.01	26.96	27.65	24.45	15.44	12.87	21.68	22.49	13.95	33.63	15.09	27.90	15.82	14.80	21.94	25.86	7.27	24.14	16.22	13.34	4.68	20.40	18.39	>0.999999999999999	1.1
P31947	14-3-3 protein sigma OS=Homo sapiens GN=SFN PE=1 SV=1	19.78	9.78	18.17	20.74	33.19	34.74	20.69	8.76	20.13	22.42	19.03	27.24	34.57	15.57	28.86	24.73	22.27	37.74	44.49	15.71	24.81	11.74	8.09	5.88	21.22	22.87	>0.999999999999999	0.9
P00450	Ceruloplasmin OS=Homo sapiens GN=CP PE=1 SV=1	9.79	4.63	7.17	7.06	13.01	11.34	11.80	4.33	12.74	8.15	8.18	6.36	16.23	5.30	8.82	8.08	13.67	10.59	15.01	7.79	14.01	3.83	8.51	2.60	8.71	9.54	>0.999999999999999	0.9
Q01518	Adenylyl cyclase-associated protein 1 OS=Homo sapiens GN=CAP1 PE=1 SV=5	22.02	18.53	25.82	25.87	22.81	31.90	14.36	7.59	14.79	20.01	13.21	7.83	28.46	13.49	32.20	14.13	12.15	18.56	15.19	14.02	14.18	7.67	11.92	4.32	18.73	15.52	>0.999999999999999	1.2
Q14204	Cytoplasmic dynein 1 heavy chain 1 OS=Homo sapiens GN=DYNC1H1 PE=1 SV=5	2.45	3.26	6.69	5.77	4.53	8.27	3.42	0.27	0.68	3.52	4.72	1.22	0.94	2.25	5.01	1.85	0.63	0.92	2.53	0.00	0.00	0.88	2.41	0.56	3.73	1.50	>0.999999999999999	2.5
P13796	Plastin-2 OS=Homo sapiens GN=LCP1 PE=1 SV=6	18.15	7.55	5.98	31.64	18.29	8.74	16.59	13.37	6.03	4.63	4.88	9.46	24.93	9.15	5.96	10.94	7.21	13.65	13.02	10.39	26.84	7.75	6.24	2.00	12.11	11.51	>0.999999999999999	1.1
P13797	Plastin-3 OS=Homo sapiens GN=PLS3 PE=1 SV=4	10.20	4.29	5.98	12.19	12.07	10.16	6.33	0.00	4.25	2.59	6.76	5.22	11.76	4.01	20.99	12.11	3.16	7.67	13.02	0.00	8.44	6.39	7.10	2.56	6.67	8.10	>0.999999999999999	0.8
Q14651	Plastin-1 OS=Homo sapiens GN=PLS1 PE=1 SV=2	4.08	3.43	6.22	6.63	10.94	8.74	9.92	1.35	1.37	2.78	6.92	4.73	0.00	3.21	8.82	7.40	2.53	3.07	6.69	0.00	0.00	2.48	6.24	1.48	5.59	3.49	>0.999999999999999	1.6
P46940	Ras GTPase-activating-like protein IQGAP1 OS=Homo sapiens GN=IQGAP1 PE=1 SV=1	6.93	7.38	13.86	14.32	8.48	12.52	5.81	2.26	3.15	8.34	8.02	4.32	5.41	3.69	15.26	7.57	2.66	3.22	6.87	1.69	4.22	4.23	7.95	2.12	7.95	5.41	>0.999999999999999	1.5
B9A064	Immunoglobulin lambda-like polypeptide 5 OS=Homo sapiens GN=IGLL5 PE=2 SV=2	31.61	21.10	22.71	20.31	23.19	29.07	16.24	11.11	16.85	17.60	19.34	10.03	36.46	19.75	22.66	20.69	19.61	18.87	28.03	20.12	20.76	9.83	13.48	6.20	19.93	19.71	>0.999999999999999	1.0
P52209	6-phosphogluconate dehydrogenase, decarboxylating OS=Homo sapiens GN=PGD PE=1 SV=3	14.48	12.18	16.97	25.01	17.54	16.78	20.01	6.41	9.72	13.15	14.63	9.54	14.35	9.79	31.00	11.95	11.77	14.27	16.82	5.58	10.30	4.87	10.07	5.20	14.70	12.16	>0.999999999999999	1.2
P00558	Phosphoglycerate kinase 1 OS=Homo sapiens GN=PGK1 PE=1 SV=3	13.05	13.04	24.62	19.03	32.05	21.03	20.18	8.04	18.35	21.86	18.56	13.87	27.75	12.20	24.57	19.85	19.11	15.80	18.63	13.37	15.02	9.43	21.43	5.36	18.64	16.88	>0.999999999999999	1.1
Q96DA0	Zymogen granule protein 16 homolog B OS=Homo sapiens GN=ZG16B PE=1 SV=3	26.10	16.98	23.67	34.64	24.13	30.25	16.93	18.06	22.19	23.71	20.13	19.90	23.29	15.90	30.53	27.26	30.87	30.69	23.15	21.03	27.34	15.98	28.38	8.01	23.06	23.53	>0.999999999999999	1.0
P02679	Fibrinogen gamma chain OS=Homo sapiens GN=FGG PE=1 SV=3	24.88	9.26	10.76	20.52	20.55	12.76	12.65	6.68	14.93	8.34	7.08	6.93	28.69	15.41	22.90	20.53	9.36	13.04	17.36	15.84	28.19	4.31	13.62	2.16	12.95	15.95	>0.999999999999999	0.8
Q09666	Neuroblast differentiation-associated protein AHNAK OS=Homo sapiens GN=AHNAK PE=1 SV=2	1.63	2.23	3.82	4.70	2.45	7.80	2.39	0.99	1.37	3.15	4.09	1.79	1.18	1.28	3.34	2.86	1.27	2.15	2.35	0.26	0.84	0.80	2.55	0.88	3.04	1.65	>0.999999999999999	1.8
P26038	Moesin OS=Homo sapiens GN=MSN PE=1 SV=3	4.28	0.86	3.59	7.06	3.96	3.54	2.56	3.52	0.00	0.93	0.79	2.20	7.76	0.80	1.19	2.52	2.66	3.22	5.97	5.06	7.76	2.16	2.13	1.32	2.77	3.55	>0.999999999999999	0.8
P02671	Fibrinogen alpha chain OS=Homo sapiens GN=FGA PE=1 SV=2	10.20	3.09	8.37	9.62	10.37	11.82	5.98	3.61	8.22	7.41	4.25	3.26	9.41	4.34	6.44	6.73	13.92	6.14	6.33	10.00	11.14	1.76	3.12	0.44	7.18	6.65	>0.999999999999999	1.1
P01591	Immunoglobulin J chain OS=Homo sapiens GN=IGJ PE=1 SV=4	71.37	38.08	31.32	20.31	41.86	40.88	37.96	31.61	38.49	24.27	34.91	18.11	66.09	45.12	41.26	37.35	44.29	34.06	40.15	28.82	59.08	13.82	31.50	8.89	35.76	37.54	>0.999999999999999	1.0
P08603	Complement factor H OS=Homo sapiens GN=CFH PE=1 SV=4	7.55	1.54	2.15	1.92	5.85	2.84	4.27	2.80	5.89	1.30	0.79	4.08	5.17	4.50	4.77	7.91	6.33	4.76	7.78	7.40	5.74	1.12	3.12	1.24	3.41	4.99	>0.999999999999999	0.7
P04114	Apolipoprotein B-100 OS=Homo sapiens GN=APOB PE=1 SV=2	1.63	0.00	0.00	0.64	2.26	0.00	0.68	0.00	0.96	0.19	0.00	2.04	2.59	0.16	0.72	2.19	3.42	0.31	0.72	1.69	0.51	0.00	0.28	0.40	0.70	1.08	>0.999999999999999	0.6
P19971	Thymidine phosphorylase OS=Homo sapiens GN=TYMP PE=1 SV=2	18.15	4.97	1.67	19.03	29.98	4.96	27.19	1.26	6.30	6.85	5.82	7.26	23.76	8.99	5.01	11.10	11.26	7.06	42.32	0.91	6.25	10.22	10.93	2.24	11.12	11.67	>0.999999999999999	1.0
Q06210	Glucosamine--fructose-6-phosphate aminotransferase [isomerizing] 1 OS=Homo sapiens GN=GFPT1 PE=1 SV=3	10.20	6.52	13.39	10.69	10.56	7.80	11.80	2.98	6.85	6.11	10.85	4.08	6.35	8.03	13.36	6.39	4.81	1.99	7.96	0.52	0.68	4.95	11.78	1.32	8.48	5.68	>0.999999999999999	1.5
P06744	Glucose-6-phosphate isomerase OS=Homo sapiens GN=GPI PE=1 SV=4	13.46	12.69	22.23	22.02	12.44	17.49	17.61	5.24	7.94	13.71	16.20	7.59	19.52	10.60	19.80	12.45	9.36	14.27	10.49	7.53	12.49	5.27	13.20	4.12	14.05	11.59	>0.999999999999999	1.2
P02774	Vitamin D-binding protein OS=Homo sapiens GN=GC PE=1 SV=1	14.27	8.75	19.12	21.81	19.23	21.50	15.56	6.32	17.39	14.82	5.35	9.30	14.11	5.46	21.70	13.46	11.51	13.96	16.46	11.81	13.50	7.27	11.35	2.04	14.45	11.89	>0.999999999999999	1.2
Q5VTE0	Putative elongation factor 1-alpha-like 3 OS=Homo sapiens GN=EEF1A1P5 PE=5 SV=1	23.25	11.32	27.25	14.11	18.86	26.94	17.10	6.95	12.05	21.12	19.97	9.30	15.52	16.06	30.29	23.89	9.74	15.34	22.97	7.27	11.14	6.15	14.19	2.64	17.35	14.60	>0.999999999999999	1.2
P00747	Plasminogen OS=Homo sapiens GN=PLG PE=1 SV=2	11.62	3.26	6.45	7.70	17.54	6.38	7.87	6.68	15.61	7.60	3.62	4.65	15.99	9.95	17.65	13.46	11.77	7.82	19.17	8.05	9.62	3.67	7.24	0.92	8.25	10.44	>0.999999999999999	0.8
P08727	Keratin, type I cytoskeletal 19 OS=Homo sapiens GN=KRT19 PE=1 SV=4	18.76	15.78	28.92	25.87	33.00	46.31	20.69	12.46	14.52	19.64	41.99	15.99	24.93	8.67	32.91	36.68	19.74	10.13	39.43	17.92	26.33	8.47	24.83	7.00	24.49	21.42	>0.999999999999999	1.1
P02763	Alpha-1-acid glycoprotein 1 OS=Homo sapiens GN=ORM1 PE=1 SV=1	55.87	15.95	28.69	32.50	22.63	35.92	25.99	13.73	20.82	50.76	18.87	15.25	43.98	19.27	54.14	38.19	19.23	34.83	27.49	19.73	25.66	14.94	17.03	7.48	28.08	26.83	>0.999999999999999	1.0
P19652	Alpha-1-acid glycoprotein 2 OS=Homo sapiens GN=ORM2 PE=1 SV=2	24.47	5.15	11.47	10.26	9.05	11.34	15.90	8.40	16.44	8.89	10.85	7.59	21.87	11.08	16.46	20.19	8.73	14.27	12.48	15.58	20.26	5.51	6.81	3.72	11.65	13.08	>0.999999999999999	0.9
P01009	Alpha-1-antitrypsin OS=Homo sapiens GN=SERPINA1 PE=1 SV=3	14.68	12.35	20.32	26.94	23.76	26.23	18.98	10.02	29.86	23.34	11.32	17.78	19.99	11.56	33.87	21.20	20.12	17.03	25.68	20.64	26.84	8.87	10.22	4.44	19.63	18.37	>0.999999999999999	1.1
P09960	Leukotriene A-4 hydrolase OS=Homo sapiens GN=LTA4H PE=1 SV=2	10.40	6.52	17.69	21.17	7.16	12.05	8.72	5.24	5.20	12.23	10.38	4.16	10.35	9.31	17.65	8.58	5.57	8.90	10.49	4.93	9.79	5.91	11.64	2.32	10.08	8.79	>0.999999999999999	1.1
P07737	Profilin-1 OS=Homo sapiens GN=PFN1 PE=1 SV=2	35.48	40.31	19.84	37.20	32.81	29.30	29.75	11.20	23.83	22.97	19.50	25.29	40.93	19.91	29.57	20.86	29.74	26.70	31.47	22.59	29.37	18.77	33.35	9.41	27.29	26.05	>0.999999999999999	1.0
P18206	Vinculin OS=Homo sapiens GN=VCL PE=1 SV=4	9.79	3.95	8.84	8.55	6.22	13.00	6.33	0.99	2.74	4.26	4.09	3.02	7.06	3.69	14.31	5.05	2.91	3.99	7.23	2.21	5.06	4.07	4.26	1.92	5.98	5.15	>0.999999999999999	1.2
P00738	Haptoglobin OS=Homo sapiens GN=HP PE=1 SV=1	26.51	17.15	17.69	13.47	31.11	30.72	31.46	18.42	27.94	21.30	8.18	20.39	34.57	18.46	31.00	24.73	25.81	31.30	23.51	35.70	31.06	13.18	14.19	8.17	22.03	24.31	>0.999999999999999	0.9
Q14624	Inter-alpha-trypsin inhibitor heavy chain H4 OS=Homo sapiens GN=ITIH4 PE=1 SV=4	7.34	2.23	7.65	9.62	6.79	7.56	7.01	4.06	7.53	3.89	3.30	4.49	8.47	3.85	10.73	8.41	6.33	5.52	7.42	5.32	6.92	2.88	3.97	0.40	5.96	5.85	>0.999999999999999	1.0
P43490	Nicotinamide phosphoribosyltransferase OS=Homo sapiens GN=NAMPT PE=1 SV=1	22.64	5.66	7.89	10.48	16.59	11.58	13.34	1.72	2.60	4.82	12.27	9.05	11.52	9.31	16.22	11.44	7.34	10.43	29.84	1.56	2.03	5.43	9.65	3.52	9.89	9.86	>0.999999999999999	1.0
P00751	Complement factor B OS=Homo sapiens GN=CFB PE=1 SV=2	15.09	2.74	5.98	5.34	12.82	5.91	6.84	3.61	13.97	8.34	4.72	6.04	15.99	6.42	8.35	8.41	9.36	8.59	15.92	8.83	12.49	4.95	5.68	1.40	7.62	8.87	>0.999999999999999	0.9
P00338	L-lactate dehydrogenase A chain OS=Homo sapiens GN=LDHA PE=1 SV=2	16.72	7.03	16.02	29.08	22.06	15.83	16.93	7.41	9.18	12.41	10.54	11.09	27.52	13.17	27.90	19.68	12.53	15.19	24.60	17.66	13.84	12.54	16.60	5.44	14.52	17.22	>0.999999999999999	0.8
Q14508	WAP four-disulfide core domain protein 2 OS=Homo sapiens GN=WFDC2 PE=1 SV=2	18.76	23.84	13.15	29.72	17.35	13.00	9.40	8.31	12.60	10.19	8.65	16.15	12.94	14.77	21.94	15.48	6.96	14.12	9.95	7.14	33.42	15.82	19.72	5.56	15.09	14.82	>0.999999999999999	1.0
P80188	Neutrophil gelatinase-associated lipocalin OS=Homo sapiens GN=LCN2 PE=1 SV=2	26.10	12.52	12.19	15.61	9.62	7.56	21.89	14.63	22.19	13.52	8.02	10.44	38.10	15.90	12.16	12.28	12.53	19.64	23.15	16.62	27.34	7.91	22.99	3.96	14.52	17.72	>0.999999999999999	0.8
Q8N4F0	BPI fold-containing family B member 2 OS=Homo sapiens GN=BPIFB2 PE=1 SV=2	6.12	8.06	7.17	10.05	7.16	3.31	9.75	7.13	10.82	3.89	12.42	8.48	13.41	7.55	13.59	7.91	7.21	8.75	10.31	6.10	13.33	9.43	8.09	4.16	7.86	9.15	>0.999999999999999	0.9
P49327	Fatty acid synthase OS=Homo sapiens GN=FASN PE=1 SV=3	2.04	3.26	6.22	5.13	4.71	2.36	2.39	0.36	0.55	0.93	2.99	1.14	0.00	0.64	3.58	1.85	0.63	1.07	1.81	0.00	0.17	0.88	2.27	0.48	2.67	1.12	>0.999999999999999	2.4
P30740	Leukocyte elastase inhibitor OS=Homo sapiens GN=SERPINB1 PE=1 SV=1	19.37	16.30	26.30	34.85	27.15	22.45	18.81	9.93	15.07	20.38	25.63	6.61	33.87	15.25	30.05	15.98	10.25	14.58	26.04	10.52	24.31	11.50	20.43	2.24	20.24	17.92	>0.999999999999999	1.1
P02751	Fibronectin OS=Homo sapiens GN=FN1 PE=1 SV=4	3.26	0.00	0.24	0.43	2.45	0.00	1.54	0.54	1.10	0.00	0.16	0.98	2.82	2.09	2.86	4.88	4.05	0.77	0.72	2.21	1.35	0.00	1.14	0.08	0.89	1.91	>0.999999999999999	0.5
P42330	Aldo-keto reductase family 1 member C3 OS=Homo sapiens GN=AKR1C3 PE=1 SV=4	10.81	7.03	12.67	17.53	12.63	12.52	9.06	8.85	7.26	12.41	18.24	12.72	9.64	8.51	15.98	11.27	5.19	10.28	14.83	6.88	0.00	9.27	13.91	4.64	11.81	9.20	>0.999999999999999	1.3
Q9NY33	Dipeptidyl peptidase 3 OS=Homo sapiens GN=DPP3 PE=1 SV=2	6.32	4.46	6.22	8.98	8.86	6.14	6.16	2.80	3.56	8.71	6.61	4.32	4.00	4.98	7.39	7.07	4.56	6.44	7.60	0.00	2.03	4.23	5.96	1.24	6.09	4.62	>0.999999999999999	1.3
Q14894	Thiomorpholine-carboxylate dehydrogenase OS=Homo sapiens GN=CRYM PE=1 SV=1	12.03	3.43	21.99	6.84	6.03	10.63	10.09	5.33	6.16	8.34	14.47	7.50	13.88	7.23	10.73	7.57	7.47	3.07	13.57	2.60	1.69	4.71	10.64	0.80	9.40	7.00	>0.999999999999999	1.3
P06737	Glycogen phosphorylase, liver form OS=Homo sapiens GN=PYGL PE=1 SV=4	5.30	1.72	8.37	7.48	7.35	8.27	7.52	0.90	1.37	4.08	7.71	3.18	6.12	2.25	3.34	3.03	1.77	2.76	2.53	3.89	2.36	3.51	6.24	0.72	5.27	3.21	>0.999999999999999	1.6
P01019	Angiotensinogen OS=Homo sapiens GN=AGT PE=1 SV=1	7.14	3.43	8.37	5.77	11.50	8.27	7.35	3.16	7.12	5.00	6.76	4.24	6.35	3.21	8.35	7.23	5.44	6.60	11.03	5.58	7.26	3.43	3.83	1.40	6.51	5.81	>0.999999999999999	1.1
P20061	Transcobalamin-1 OS=Homo sapiens GN=TCN1 PE=1 SV=2	11.83	11.84	7.17	17.10	15.08	13.71	8.21	6.23	7.94	10.75	7.55	6.53	18.82	14.77	7.15	9.76	8.73	14.12	8.68	7.53	9.79	6.39	6.81	4.24	10.33	9.73	>0.999999999999999	1.1
P08729	Keratin, type II cytoskeletal 7 OS=Homo sapiens GN=KRT7 PE=1 SV=5	13.05	0.00	8.61	7.70	20.93	20.32	9.40	5.78	10.27	10.19	15.41	9.05	8.47	0.00	15.26	16.49	6.96	6.90	27.85	3.64	7.60	2.88	13.91	2.20	10.89	9.35	>0.999999999999999	1.2
P08670	Vimentin OS=Homo sapiens GN=VIM PE=1 SV=4	10.60	2.23	0.00	5.77	19.61	6.38	15.73	7.32	8.35	2.41	5.50	9.54	16.70	5.62	6.44	3.36	4.43	7.98	14.65	15.19	15.53	4.15	6.10	1.08	7.79	8.44	>0.999999999999999	0.9
Q5XKE5	Keratin, type II cytoskeletal 79 OS=Homo sapiens GN=KRT79 PE=1 SV=2	2.45	2.06	2.87	3.85	2.26	2.84	2.05	0.00	0.00	4.63	2.83	1.47	0.00	0.00	0.00	2.02	0.00	1.84	3.26	0.00	0.00	0.96	0.00	0.00	2.28	0.67	>0.999999999999999	3.4
O95678	Keratin, type II cytoskeletal 75 OS=Homo sapiens GN=KRT75 PE=1 SV=2	2.24	0.00	0.00	0.00	0.00	0.00	0.00	0.00	0.00	0.00	0.00	0.00	0.00	0.00	0.00	0.00	0.00	0.00	0.00	0.00	0.00	0.00	0.00	0.00	0.19	0.00	>0.999999999999999	#DIV/0!
P11766	Alcohol dehydrogenase class-3 OS=Homo sapiens GN=ADH5 PE=1 SV=4	12.24	12.52	11.47	12.83	6.98	20.56	8.21	4.33	3.70	11.12	11.48	3.02	11.29	11.72	11.45	8.08	6.07	5.68	6.69	3.51	1.35	5.83	10.36	1.92	9.87	7.00	>0.999999999999999	1.4
Q86VP6	Cullin-associated NEDD8-dissociated protein 1 OS=Homo sapiens GN=CAND1 PE=1 SV=2	5.30	4.97	9.32	9.62	6.79	7.56	7.69	1.63	2.05	4.26	7.71	2.94	3.53	3.37	5.49	3.87	1.52	2.76	5.79	0.00	0.34	3.59	6.39	1.68	5.82	3.19	>0.999999999999999	1.8
P20591	Interferon-induced GTP-binding protein Mx1 OS=Homo sapiens GN=MX1 PE=1 SV=4	10.81	1.54	5.74	8.55	1.70	0.95	17.95	0.00	0.00	6.30	7.23	4.81	15.52	2.89	8.11	3.03	0.00	2.15	16.10	0.00	0.00	0.00	4.12	1.36	5.47	4.44	>0.999999999999999	1.2
P02765	Alpha-2-HS-glycoprotein OS=Homo sapiens GN=AHSG PE=1 SV=1	9.99	2.92	17.69	7.91	13.95	17.49	8.38	6.68	8.35	16.49	2.67	8.48	11.52	7.87	14.55	12.45	6.20	7.52	11.03	6.36	15.02	3.91	3.83	1.96	10.08	8.52	>0.999999999999999	1.2
P02812	Basic salivary proline-rich protein 2 OS=Homo sapiens GN=PRB2 PE=1 SV=3	5.30	0.00	0.00	0.00	1.51	0.00	1.37	0.00	0.00	0.00	0.00	1.39	6.12	0.00	0.00	0.00	2.15	1.23	1.45	0.00	0.00	0.00	0.00	0.32	0.80	0.94	>0.999999999999999	0.8
P31146	Coronin-1A OS=Homo sapiens GN=CORO1A PE=1 SV=4	9.18	2.23	0.00	9.62	3.77	1.42	4.79	4.06	7.40	1.11	0.00	3.67	8.47	2.09	1.43	2.19	4.56	4.30	6.51	5.84	14.35	1.60	0.85	0.00	3.94	4.35	>0.999999999999999	0.9
P01040	Cystatin-A OS=Homo sapiens GN=CSTA PE=1 SV=1	59.54	71.19	17.45	27.15	78.25	30.01	70.96	37.48	56.84	36.68	90.59	16.15	46.57	46.88	47.22	49.13	72.88	63.67	104.18	37.91	49.29	23.32	28.10	11.69	49.36	48.40	>0.999999999999999	1.0
P15104	Glutamine synthetase OS=Homo sapiens GN=GLUL PE=1 SV=4	7.14	7.72	5.98	3.42	10.75	8.27	4.27	1.44	1.10	1.48	8.96	2.85	5.88	5.62	5.96	11.61	3.16	8.75	10.31	3.25	1.35	2.00	9.79	1.00	5.28	5.72	>0.999999999999999	0.9
P23142	Fibulin-1 OS=Homo sapiens GN=FBLN1 PE=1 SV=4	5.51	0.69	0.96	0.00	1.51	0.95	0.00	0.00	0.00	0.74	0.63	1.06	3.06	0.00	1.91	4.54	1.01	0.00	0.72	0.00	0.00	0.00	1.14	0.32	1.00	1.06	>0.999999999999999	0.9
P01042	Kininogen-1 OS=Homo sapiens GN=KNG1 PE=1 SV=2	3.87	1.54	4.54	4.06	9.24	5.91	5.13	4.97	8.49	4.63	3.93	4.49	12.94	4.82	5.96	6.06	6.96	5.52	6.51	7.14	9.28	1.52	2.70	0.76	5.07	5.85	>0.999999999999999	0.9
P09467	Fructose-1,6-bisphosphatase 1 OS=Homo sapiens GN=FBP1 PE=1 SV=5	16.72	9.09	12.67	17.53	9.99	12.52	11.46	3.70	9.18	5.37	10.54	5.47	6.82	10.76	15.98	11.27	10.38	10.28	17.73	2.47	11.31	6.55	9.51	3.28	10.35	9.69	>0.999999999999999	1.1
Q9Y6R7	IgGFc-binding protein OS=Homo sapiens GN=FCGBP PE=1 SV=3	1.02	0.17	2.39	2.35	3.02	1.89	1.71	2.08	5.48	1.30	3.15	0.08	2.12	1.61	5.72	2.86	2.78	5.06	3.26	1.69	4.56	2.88	3.41	0.04	2.05	3.00	>0.999999999999999	0.7
P37837	Transaldolase OS=Homo sapiens GN=TALDO1 PE=1 SV=2	16.31	8.92	12.43	11.12	21.31	22.68	13.68	13.73	15.48	9.63	23.90	12.40	22.58	4.66	15.74	8.75	12.15	14.73	23.87	12.46	13.50	6.39	7.38	3.20	15.13	12.12	>0.999999999999999	1.2
Q16378	Proline-rich protein 4 OS=Homo sapiens GN=PRR4 PE=1 SV=3	17.13	30.19	20.08	0.00	33.19	0.00	0.00	0.00	0.00	15.56	19.66	0.00	41.40	28.26	0.00	0.00	15.82	0.00	0.00	0.00	0.00	9.99	24.97	0.00	11.32	10.04	>0.999999999999999	1.1
P04196	Histidine-rich glycoprotein OS=Homo sapiens GN=HRG PE=1 SV=1	9.18	0.86	8.84	7.91	4.53	5.67	4.10	5.51	6.16	6.85	2.67	4.32	4.00	0.80	7.15	8.92	3.80	10.74	5.43	7.92	1.86	2.96	4.26	0.20	5.55	4.84	>0.999999999999999	1.1
Q16719	Kynureninase OS=Homo sapiens GN=KYNU PE=1 SV=1	7.14	2.23	0.00	4.28	5.09	0.00	4.62	1.17	0.00	0.00	5.50	3.59	6.35	3.21	6.44	8.92	1.64	3.07	9.59	1.69	0.00	2.80	6.24	1.40	2.80	4.28	>0.999999999999999	0.7
P10909	Clusterin OS=Homo sapiens GN=CLU PE=1 SV=1	7.14	8.92	8.37	9.41	11.69	12.29	10.60	6.50	8.49	8.15	6.92	5.87	19.52	7.06	10.49	10.43	6.58	12.73	9.41	9.35	8.78	4.95	7.38	2.48	8.70	9.10	>0.999999999999999	1.0
Q12906	Interleukin enhancer-binding factor 3 OS=Homo sapiens GN=ILF3 PE=1 SV=3	3.67	2.40	5.26	7.48	5.09	7.33	3.76	0.99	1.51	4.08	5.50	1.47	1.65	1.12	2.62	3.70	0.89	2.76	3.26	0.00	0.00	2.16	2.55	0.28	4.05	1.75	>0.999999999999999	2.3
Q14974	Importin subunit beta-1 OS=Homo sapiens GN=KPNB1 PE=1 SV=2	4.49	0.51	4.30	6.41	5.66	4.25	4.45	0.90	1.92	3.33	4.09	2.45	1.65	1.61	5.25	3.03	0.38	1.53	3.98	0.39	1.69	1.76	3.12	0.56	3.56	2.08	>0.999999999999999	1.7
P01008	Antithrombin-III OS=Homo sapiens GN=SERPINC1 PE=1 SV=1	10.60	7.55	10.52	11.12	13.58	14.65	5.98	4.70	3.70	11.49	8.18	3.59	10.35	4.34	12.40	8.75	6.58	5.37	6.33	8.05	4.56	2.80	6.24	1.08	8.80	6.40	>0.999999999999999	1.4
O75368	SH3 domain-binding glutamic acid-rich-like protein OS=Homo sapiens GN=SH3BGRL PE=1 SV=1	21.21	17.84	24.86	22.24	11.50	24.58	27.19	20.59	14.24	11.30	25.01	8.48	14.35	16.70	37.92	10.26	7.72	24.39	28.76	13.50	26.84	12.70	22.56	4.16	19.09	18.32	>0.999999999999999	1.0
Q9Y678	Coatomer subunit gamma OS=Homo sapiens GN=COPG PE=1 SV=1	3.67	1.72	11.47	5.56	4.90	8.03	4.45	0.63	0.00	2.59	5.98	3.92	0.00	1.12	7.15	5.72	0.89	0.46	4.70	0.00	0.00	3.04	6.10	1.36	4.41	2.55	>0.999999999999999	1.7
P30153	Serine/threonine-protein phosphatase 2A 65 kDa regulatory subunit A alpha isoform OS=Homo sapiens GN=PPP2R	5.51	3.60	6.45	5.77	7.54	4.96	6.84	1.90	6.44	3.89	7.39	3.83	6.35	6.42	8.11	4.54	2.02	4.14	7.23	0.00	0.84	3.75	5.68	1.88	5.34	4.25	>0.999999999999999	1.3
O15143	Actin-related protein 2/3 complex subunit 1B OS=Homo sapiens GN=ARPC1B PE=1 SV=3	9.38	4.46	6.22	3.42	4.90	6.14	4.45	4.15	2.19	6.67	4.09	2.12	10.82	5.78	6.20	2.69	1.01	5.52	8.32	2.08	6.08	1.28	3.69	1.44	4.85	4.58	>0.999999999999999	1.1
P05156	Complement factor I OS=Homo sapiens GN=CFI PE=1 SV=2	5.51	0.86	1.20	2.14	5.09	2.36	4.62	2.44	4.52	3.89	0.00	1.22	4.94	3.37	2.38	2.52	4.18	4.14	3.80	4.28	3.54	1.68	3.83	0.40	2.82	3.26	>0.999999999999999	0.9
Q9UQ80	Proliferation-associated protein 2G4 OS=Homo sapiens GN=PA2G4 PE=1 SV=3	6.93	7.55	12.91	14.11	8.30	12.76	11.29	2.17	3.29	10.00	8.49	5.38	1.65	5.46	12.88	9.09	4.30	5.22	9.77	0.00	4.05	5.27	9.37	1.76	8.60	5.73	>0.999999999999999	1.5
P46459	Vesicle-fusing ATPase OS=Homo sapiens GN=NSF PE=1 SV=3	7.34	2.92	12.67	7.70	7.92	6.14	5.30	1.54	1.10	2.22	4.88	3.83	0.94	2.73	10.02	4.37	3.29	3.99	5.61	0.00	0.00	3.35	2.98	1.04	5.30	3.19	>0.999999999999999	1.7
P52907	F-actin-capping protein subunit alpha-1 OS=Homo sapiens GN=CAPZA1 PE=1 SV=3	12.44	13.38	14.58	16.68	11.50	14.41	13.34	4.24	8.35	11.30	12.27	2.69	11.05	7.55	14.55	13.12	12.02	1.53	17.18	4.28	16.04	7.59	11.07	2.44	11.27	9.87	>0.999999999999999	1.1
P34932	Heat shock 70 kDa protein 4 OS=Homo sapiens GN=HSPA4 PE=1 SV=4	3.67	4.63	10.76	7.70	6.79	8.51	5.30	1.26	1.92	7.41	7.08	2.94	0.71	3.69	8.59	4.54	0.89	2.76	4.16	0.39	1.18	2.48	3.83	1.24	5.66	2.87	>0.999999999999999	2.0
P61158	Actin-related protein 3 OS=Homo sapiens GN=ACTR3 PE=1 SV=3	9.99	2.40	5.26	10.48	9.24	11.58	10.09	1.99	5.48	5.74	9.28	4.81	7.29	2.25	7.39	3.70	2.78	3.38	8.86	5.19	5.23	5.59	5.68	2.36	7.19	4.98	>0.999999999999999	1.4
P56537	Eukaryotic translation initiation factor 6 OS=Homo sapiens GN=EIF6 PE=1 SV=1	20.60	7.20	6.22	16.89	11.12	6.14	7.18	3.79	3.56	2.22	4.09	2.12	6.12	4.17	14.07	9.93	7.47	6.44	7.60	0.00	2.03	8.07	8.37	2.36	7.60	6.39	>0.999999999999999	1.2
Q00796	Sorbitol dehydrogenase OS=Homo sapiens GN=SORD PE=1 SV=4	12.85	6.69	21.99	16.46	5.28	9.22	13.17	5.69	5.34	3.33	1.26	7.50	6.59	8.03	30.05	10.60	0.00	0.00	13.93	2.34	1.35	0.64	2.55	3.08	9.07	6.60	>0.999999999999999	1.4
Q96QK1	Vacuolar protein sorting-associated protein 35 OS=Homo sapiens GN=VPS35 PE=1 SV=2	3.87	3.95	3.59	4.92	2.07	2.60	2.56	0.36	0.00	4.26	2.99	2.28	1.65	1.12	7.63	4.71	0.51	1.07	2.71	0.52	0.00	0.88	5.25	1.48	2.79	2.29	>0.999999999999999	1.2
O14745	Na(+)/H(+) exchange regulatory cofactor NHE-RF1 OS=Homo sapiens GN=SLC9A3R1 PE=1 SV=4	7.75	6.52	6.69	8.12	9.43	8.98	6.50	3.43	2.47	5.19	5.98	2.28	8.94	6.10	6.68	6.39	1.01	2.76	5.06	0.00	4.73	2.24	3.97	1.52	6.11	4.12	>0.999999999999999	1.5
Q9UGM3	Deleted in malignant brain tumors 1 protein OS=Homo sapiens GN=DMBT1 PE=1 SV=2	1.84	1.72	0.96	1.28	3.21	2.36	1.54	0.90	1.51	2.04	1.42	1.63	1.18	1.45	1.43	1.01	1.64	1.53	1.63	1.04	1.69	1.12	1.84	0.52	1.70	1.34	>0.999999999999999	1.3
P36955	Pigment epithelium-derived factor OS=Homo sapiens GN=SERPINF1 PE=1 SV=4	10.40	3.95	12.19	4.92	11.69	9.69	10.60	6.59	17.53	5.93	3.62	6.93	12.00	5.14	7.63	12.28	10.76	6.29	7.42	14.67	12.32	4.95	5.82	2.04	8.67	8.44	>0.999999999999999	1.0
P01031	Complement C5 OS=Homo sapiens GN=C5 PE=1 SV=4	3.47	0.34	0.48	0.00	3.21	0.47	1.20	1.54	3.15	0.00	0.00	3.10	1.65	1.12	1.67	4.21	4.30	1.38	3.07	3.76	1.52	0.72	2.13	0.28	1.41	2.15	>0.999999999999999	0.7
P19827	Inter-alpha-trypsin inhibitor heavy chain H1 OS=Homo sapiens GN=ITIH1 PE=1 SV=3	2.65	0.51	1.67	2.14	5.47	3.07	6.33	2.26	6.98	1.85	1.10	3.02	2.35	1.61	4.05	5.55	4.68	3.22	6.69	4.28	7.09	2.00	1.84	0.68	3.09	3.67	>0.999999999999999	0.8
P19823	Inter-alpha-trypsin inhibitor heavy chain H2 OS=Homo sapiens GN=ITIH2 PE=1 SV=2	4.89	1.03	4.78	4.28	4.53	4.73	5.98	3.16	5.34	4.45	3.15	3.18	5.64	3.21	4.77	5.22	3.42	4.14	6.33	8.70	7.43	1.60	3.41	1.24	4.12	4.59	>0.999999999999999	0.9
P24158	Myeloblastin OS=Homo sapiens GN=PRTN3 PE=1 SV=3	5.10	2.06	0.00	5.34	0.00	0.00	6.84	2.26	1.64	0.00	0.00	0.00	5.88	1.93	0.00	2.02	3.16	3.84	2.17	3.25	6.75	0.00	0.00	0.00	1.94	2.42	>0.999999999999999	0.8
P11413	Glucose-6-phosphate 1-dehydrogenase OS=Homo sapiens GN=G6PD PE=1 SV=4	7.75	2.92	2.63	9.83	23.19	8.98	5.30	2.17	1.51	7.04	12.58	3.75	4.00	2.73	9.06	7.74	7.84	3.68	8.32	0.00	5.23	3.04	14.33	1.84	7.30	5.65	>0.999999999999999	1.3
P29692	Elongation factor 1-delta OS=Homo sapiens GN=EEF1D PE=1 SV=5	7.14	14.41	20.08	17.96	12.44	19.85	5.98	1.99	3.01	12.23	13.21	6.85	15.52	8.03	15.74	17.33	2.78	5.37	15.19	1.43	3.71	3.99	9.37	4.12	11.26	8.55	>0.999999999999999	1.3
P03973	Antileukoproteinase OS=Homo sapiens GN=SLPI PE=1 SV=2	16.93	21.27	19.84	17.75	75.80	55.53	40.18	73.96	70.40	22.97	48.75	14.19	94.55	19.91	11.92	13.96	39.22	78.86	31.47	66.73	109.88	24.76	11.78	4.96	39.80	42.34	>0.999999999999999	0.9
P54108	Cysteine-rich secretory protein 3 OS=Homo sapiens GN=CRISP3 PE=1 SV=1	5.10	4.29	2.87	5.34	2.26	2.84	4.27	5.06	3.42	4.63	3.93	2.04	9.17	4.01	5.96	4.21	3.16	3.84	4.52	3.25	4.22	2.00	5.53	2.24	3.84	4.34	>0.999999999999999	0.9
Q9ULZ3	Apoptosis-associated speck-like protein containing a CARD OS=Homo sapiens GN=PYCARD PE=1 SV=2	11.01	2.74	12.91	22.66	10.18	18.43	9.23	2.98	7.40	14.45	8.49	4.40	7.76	12.52	18.60	13.12	4.18	8.29	9.77	7.01	2.70	8.47	7.66	3.12	10.41	8.60	>0.999999999999999	1.2
Q16531	DNA damage-binding protein 1 OS=Homo sapiens GN=DDB1 PE=1 SV=1	2.24	2.74	3.82	1.07	2.07	1.89	1.88	0.00	0.00	2.41	2.52	0.24	0.71	0.80	1.91	0.50	0.00	0.00	0.54	0.00	0.00	0.40	2.70	0.12	1.74	0.64	>0.999999999999999	2.7
P01614	Ig kappa chain V-II region Cum OS=Homo sapiens PE=1 SV=1	12.44	4.63	6.45	5.77	5.09	6.38	4.62	0.00	0.00	5.00	4.25	2.20	14.35	4.34	6.44	4.54	3.42	9.36	11.03	3.51	10.30	2.16	3.83	1.08	4.74	6.20	>0.999999999999999	0.8
P02649	Apolipoprotein E OS=Homo sapiens GN=APOE PE=1 SV=1	8.56	0.00	0.00	4.06	5.66	0.00	9.40	0.81	13.83	0.00	1.42	3.43	9.88	4.82	2.15	7.07	5.31	1.38	5.43	7.14	5.06	0.00	5.96	0.00	3.93	4.52	>0.999999999999999	0.9
O60763	General vesicular transport factor p115 OS=Homo sapiens GN=USO1 PE=1 SV=2	1.84	3.26	7.41	3.42	1.70	3.78	3.25	0.54	0.82	2.41	3.62	1.06	2.12	1.45	3.10	1.01	0.38	0.46	1.63	0.00	0.00	0.72	2.27	0.24	2.76	1.11	>0.999999999999999	2.5
P07237	Protein disulfide-isomerase OS=Homo sapiens GN=P4HB PE=1 SV=3	8.16	3.09	2.87	11.97	4.71	11.34	6.84	5.87	6.57	3.33	8.81	8.65	7.53	7.71	4.29	10.94	4.05	7.36	11.76	5.19	10.97	3.20	6.81	4.72	6.85	7.04	>0.999999999999999	1.0
P00966	Argininosuccinate synthase OS=Homo sapiens GN=ASS1 PE=1 SV=2	10.40	3.95	9.80	15.61	30.17	9.69	12.48	4.61	6.98	5.74	9.59	4.98	12.00	2.41	17.41	10.26	9.24	7.82	11.03	2.99	8.61	6.79	8.66	1.64	10.33	8.24	>0.999999999999999	1.3
Q14103	Heterogeneous nuclear ribonucleoprotein D0 OS=Homo sapiens GN=HNRNPD PE=1 SV=1	3.67	4.80	6.69	5.99	5.28	15.12	4.79	2.53	5.34	3.33	8.02	1.47	4.23	2.89	9.30	4.71	3.54	2.76	5.06	2.34	3.04	3.12	7.24	0.72	5.59	4.08	>0.999999999999999	1.4
O14979	Heterogeneous nuclear ribonucleoprotein D-like OS=Homo sapiens GN=HNRNPDL PE=1 SV=3	3.06	3.95	5.50	4.92	5.85	5.43	3.93	2.08	3.15	4.26	6.45	1.22	3.53	3.69	5.49	2.52	2.91	3.53	2.71	0.00	0.00	1.84	4.40	0.60	4.15	2.60	>0.999999999999999	1.6
P00734	Prothrombin OS=Homo sapiens GN=F2 PE=1 SV=2	6.32	0.00	3.35	4.28	4.71	3.31	1.54	5.15	9.72	3.71	2.20	3.51	1.18	4.01	5.96	8.41	8.10	5.68	9.04	6.49	6.25	2.00	2.84	0.36	3.98	5.03	>0.999999999999999	0.8
P02735	Serum amyloid A protein OS=Homo sapiens GN=SAA1 PE=1 SV=2	11.62	0.00	0.00	12.19	0.00	0.00	4.27	0.00	3.42	0.00	0.00	0.00	5.88	0.00	0.00	0.00	3.16	0.00	0.00	0.00	0.00	0.00	0.00	0.00	2.63	0.75	>0.999999999999999	3.5
P04080	Cystatin-B OS=Homo sapiens GN=CSTB PE=1 SV=2	57.51	48.37	67.41	26.51	75.42	29.30	21.20	11.20	26.30	35.57	19.50	10.11	29.17	45.28	131.89	67.30	15.69	10.89	22.43	9.22	20.93	22.53	27.24	4.96	35.70	33.96	>0.999999999999999	1.1
P02748	Complement component C9 OS=Homo sapiens GN=C9 PE=1 SV=2	8.56	1.72	3.82	1.07	5.28	3.78	2.74	3.16	7.67	4.08	3.46	4.00	13.17	4.50	8.35	2.69	5.31	8.59	7.60	6.36	7.09	1.28	4.97	0.88	4.11	5.90	>0.999999999999999	0.7
P63241	Eukaryotic translation initiation factor 5A-1 OS=Homo sapiens GN=EIF5A PE=1 SV=2	21.62	18.18	17.21	15.39	19.99	17.01	18.12	6.50	9.86	7.97	6.76	5.87	10.11	3.21	35.06	17.83	9.11	11.05	13.02	0.00	7.26	5.75	27.67	5.88	13.71	12.16	>0.999999999999999	1.1
P01717	Ig lambda chain V-IV region Hil OS=Homo sapiens PE=1 SV=1	13.87	5.15	7.17	14.54	12.82	16.07	11.63	6.14	9.31	0.00	4.72	5.55	15.99	4.82	7.15	11.44	8.60	10.43	12.30	8.83	11.48	2.40	4.26	1.20	8.91	8.24	>0.999999999999999	1.1
P60842	Eukaryotic initiation factor 4A-I OS=Homo sapiens GN=EIF4A1 PE=1 SV=1	10.40	0.00	0.00	15.61	9.62	14.65	12.48	3.70	2.05	0.00	13.53	3.34	5.41	3.69	12.16	5.38	0.00	6.29	7.42	0.00	0.00	3.28	5.82	1.64	7.12	4.26	>0.999999999999999	1.7
P52566	Rho GDP-dissociation inhibitor 2 OS=Homo sapiens GN=ARHGDIB PE=1 SV=3	19.78	8.58	11.95	15.39	18.29	11.82	16.59	4.52	13.29	9.26	7.86	5.87	16.93	11.56	17.17	8.41	9.11	11.05	9.04	16.36	33.25	5.75	7.10	2.88	11.93	12.38	>0.999999999999999	1.0
Q96G03	Phosphoglucomutase-2 OS=Homo sapiens GN=PGM2 PE=1 SV=4	3.06	7.72	6.22	12.83	4.90	6.14	2.56	1.35	2.88	3.89	5.03	3.18	6.12	4.17	7.63	4.37	1.90	5.98	4.70	1.30	1.69	3.12	4.54	1.80	4.98	3.94	>0.999999999999999	1.3
Q16401	26S proteasome non-ATPase regulatory subunit 5 OS=Homo sapiens GN=PSMD5 PE=1 SV=3	5.10	1.03	4.30	7.06	2.26	5.91	4.27	0.00	0.00	1.11	2.83	0.98	1.41	0.00	5.96	1.01	0.00	1.84	3.26	0.00	1.01	0.96	3.55	1.00	2.90	1.67	>0.999999999999999	1.7
P00491	Purine nucleoside phosphorylase OS=Homo sapiens GN=PNP PE=1 SV=2	9.79	1.72	5.26	4.70	4.15	8.03	10.77	3.07	4.66	6.30	3.46	3.92	8.00	3.53	5.25	1.68	4.30	9.67	8.68	4.41	16.54	3.83	1.42	1.36	5.49	5.72	>0.999999999999999	1.0
P31943	Heterogeneous nuclear ribonucleoprotein H OS=Homo sapiens GN=HNRNPH1 PE=1 SV=4	5.91	2.40	6.93	6.20	7.16	8.98	4.96	2.62	3.97	5.37	7.39	1.71	6.82	2.25	5.01	0.00	0.00	1.07	6.87	0.00	1.18	3.04	0.00	0.28	5.30	2.21	>0.999999999999999	2.4
P33241	Lymphocyte-specific protein 1 OS=Homo sapiens GN=LSP1 PE=1 SV=1	8.16	1.54	2.15	8.55	0.00	2.13	3.08	3.61	1.23	0.00	2.83	1.47	2.12	2.89	2.15	1.51	1.14	2.76	1.63	2.34	4.89	0.00	1.28	0.72	2.90	1.95	>0.999999999999999	1.5
P01766	Ig heavy chain V-III region BRO OS=Homo sapiens PE=1 SV=1	11.83	9.95	6.22	5.56	18.48	6.14	16.76	8.85	7.94	10.75	4.09	4.73	13.64	15.73	13.83	16.49	3.29	15.04	10.49	12.72	16.54	4.63	3.69	2.32	9.27	10.70	>0.999999999999999	0.9
P62979	Ubiquitin-40S ribosomal protein S27a OS=Homo sapiens GN=RPS27A PE=1 SV=2	13.46	11.32	23.19	3.85	18.29	31.43	16.59	8.76	9.04	17.97	20.92	5.38	15.52	6.42	31.72	11.10	12.27	14.88	17.54	8.57	22.45	5.27	5.68	2.64	15.02	12.84	>0.999999999999999	1.2
P08865	40S ribosomal protein SA OS=Homo sapiens GN=RPSA PE=1 SV=4	9.58	8.06	18.65	16.68	8.86	14.65	13.34	1.90	4.52	14.45	12.27	6.36	18.35	7.55	14.79	10.43	4.18	7.21	17.36	0.00	1.69	4.95	8.80	1.88	10.78	8.10	>0.999999999999999	1.3
P12277	Creatine kinase B-type OS=Homo sapiens GN=CKB PE=1 SV=1	5.10	7.72	19.36	7.48	8.48	10.63	9.58	0.72	6.16	12.60	10.69	1.31	5.88	4.01	19.32	2.69	2.02	3.84	2.89	0.00	7.60	5.43	9.65	0.64	8.32	5.33	>0.999999999999999	1.6
P53634	Dipeptidyl peptidase 1 OS=Homo sapiens GN=CTSC PE=1 SV=2	4.08	4.63	1.43	0.00	1.13	3.07	4.62	0.00	3.70	0.00	0.94	1.06	1.41	3.21	1.43	1.01	3.42	3.07	3.62	0.78	2.19	0.48	1.84	0.24	2.06	1.89	>0.999999999999999	1.1
Q13813	Spectrin alpha chain, brain OS=Homo sapiens GN=SPTAN1 PE=1 SV=3	1.63	3.26	3.82	4.28	2.83	8.51	1.71	0.63	0.68	4.26	6.61	1.55	0.71	1.12	3.58	1.85	0.63	0.92	1.81	0.00	0.00	0.48	2.70	0.60	3.31	1.20	>0.999999999999999	2.8
Q6XQN6	Nicotinate phosphoribosyltransferase OS=Homo sapiens GN=NAPRT1 PE=1 SV=2	5.10	3.09	7.65	3.85	2.26	2.84	5.47	1.08	2.47	2.22	2.83	2.04	2.82	0.96	5.96	3.03	1.52	0.92	8.50	0.00	0.00	3.12	2.55	1.00	3.41	2.53	>0.999999999999999	1.3
P01714	Ig lambda chain V-III region SH OS=Homo sapiens PE=1 SV=1	14.07	0.00	0.00	6.41	13.01	16.30	11.80	6.23	0.00	5.56	0.00	5.63	16.23	4.82	7.15	5.05	15.06	10.59	0.00	3.89	5.06	2.40	4.26	2.76	6.58	6.44	>0.999999999999999	1.0
O43396	Thioredoxin-like protein 1 OS=Homo sapiens GN=TXNL1 PE=1 SV=3	6.93	5.83	11.24	13.26	11.69	14.65	5.81	1.90	4.66	11.49	12.42	0.82	4.94	5.46	14.79	10.43	2.66	5.22	8.50	1.30	3.54	3.75	6.67	1.36	8.39	5.72	>0.999999999999999	1.5
Q07020	60S ribosomal protein L18 OS=Homo sapiens GN=RPL18 PE=1 SV=2	11.01	5.66	12.91	11.55	14.52	12.76	5.64	1.35	7.40	2.78	12.11	2.69	12.70	8.67	12.88	9.09	9.74	2.30	5.97	0.00	5.57	2.64	14.90	1.32	8.36	7.15	>0.999999999999999	1.2
P61006	Ras-related protein Rab-8A OS=Homo sapiens GN=RAB8A PE=1 SV=1	6.12	0.00	3.35	0.00	13.01	11.34	0.00	0.00	0.00	0.00	10.85	5.63	7.06	7.71	0.00	8.08	0.00	0.00	0.00	0.00	0.00	0.00	0.00	0.00	4.19	1.90	>0.999999999999999	2.2
P21281	V-type proton ATPase subunit B, brain isoform OS=Homo sapiens GN=ATP6V1B2 PE=1 SV=3	3.67	0.00	2.87	0.00	3.39	0.00	4.27	1.08	0.82	1.11	0.94	0.49	1.41	0.00	4.29	3.03	0.76	1.84	4.52	0.00	0.00	2.00	2.55	0.48	1.55	1.74	>0.999999999999999	0.9
O95994	Anterior gradient protein 2 homolog OS=Homo sapiens GN=AGR2 PE=1 SV=1	17.54	2.92	14.10	12.61	3.21	13.94	14.71	3.34	5.07	6.85	18.56	9.63	8.70	2.73	20.51	9.93	7.47	5.68	15.55	4.80	2.87	6.87	12.20	0.68	10.21	8.17	>0.999999999999999	1.2
Q8NC51	Plasminogen activator inhibitor 1 RNA-binding protein OS=Homo sapiens GN=SERBP1 PE=1 SV=2	4.89	4.12	7.89	9.19	4.53	10.16	5.64	2.98	3.29	6.11	3.77	3.51	1.65	2.41	3.58	4.04	4.18	5.06	2.71	0.91	1.18	1.92	3.41	0.96	5.51	2.67	>0.999999999999999	2.1
P31948	Stress-induced-phosphoprotein 1 OS=Homo sapiens GN=STIP1 PE=1 SV=1	3.26	3.77	10.28	7.70	5.47	8.51	4.96	1.99	2.19	6.67	9.12	0.90	2.59	5.78	6.92	2.69	1.39	2.45	2.89	1.43	0.84	3.43	6.10	0.64	5.40	3.10	>0.999999999999999	1.7
P16152	Carbonyl reductase [NADPH] 1 OS=Homo sapiens GN=CBR1 PE=1 SV=3	7.34	8.75	30.60	10.90	24.13	30.25	18.12	6.05	11.78	12.41	13.53	12.48	8.47	5.78	36.49	11.27	13.41	7.82	23.15	2.99	3.88	8.47	29.94	5.12	15.53	13.07	>0.999999999999999	1.2
O75828	Carbonyl reductase [NADPH] 3 OS=Homo sapiens GN=CBR3 PE=1 SV=3	7.14	0.00	20.08	10.69	9.43	15.60	8.55	3.16	0.00	6.48	19.66	4.08	5.17	5.62	29.81	0.00	0.00	3.38	15.19	0.00	0.00	6.71	21.14	0.00	8.74	7.25	>0.999999999999999	1.2
P54577	Tyrosine--tRNA ligase, cytoplasmic OS=Homo sapiens GN=YARS PE=1 SV=4	4.89	5.32	5.74	8.12	3.39	8.98	6.50	0.54	0.82	1.11	4.88	0.90	1.41	1.77	4.29	1.85	1.39	1.69	5.61	0.00	0.00	1.92	3.41	0.72	4.27	2.00	>0.999999999999999	2.1
P58546	Myotrophin OS=Homo sapiens GN=MTPN PE=1 SV=2	12.03	17.33	24.14	21.59	11.12	23.87	17.27	9.12	13.83	18.71	34.44	8.24	35.99	9.47	72.03	9.93	12.78	9.05	10.67	0.00	9.96	4.71	8.37	4.04	17.64	15.58	>0.999999999999999	1.1
P21283	V-type proton ATPase subunit C 1 OS=Homo sapiens GN=ATP6V1C1 PE=1 SV=4	1.43	1.20	5.74	5.13	3.02	5.67	1.20	0.00	0.00	2.96	1.10	0.00	1.65	2.57	3.82	2.69	0.00	0.00	6.15	0.00	0.00	0.56	0.00	0.64	2.29	1.51	>0.999999999999999	1.5
P42574	Caspase-3 OS=Homo sapiens GN=CASP3 PE=1 SV=2	4.49	3.77	2.39	4.70	4.15	2.36	1.71	0.90	1.37	1.85	1.57	1.79	2.35	0.00	2.38	1.68	1.27	1.53	1.81	0.00	1.69	1.76	3.12	0.40	2.59	1.50	>0.999999999999999	1.7
P24534	Elongation factor 1-beta OS=Homo sapiens GN=EEF1B2 PE=1 SV=3	5.91	7.89	11.00	9.83	12.44	15.60	4.96	4.15	1.78	5.37	10.38	3.75	10.82	7.39	10.97	4.88	5.82	7.06	8.32	0.00	7.76	2.32	4.12	2.64	7.76	6.01	>0.999999999999999	1.3
P80748	Ig lambda chain V-III region LOI OS=Homo sapiens PE=1 SV=1	13.46	11.32	15.78	14.11	12.44	15.60	11.29	5.96	9.04	12.23	10.38	5.38	15.52	10.60	26.95	19.01	8.35	10.13	5.25	8.57	4.89	5.27	16.03	2.64	11.42	11.10	>0.999999999999999	1.0
Q9NQW7	Xaa-Pro aminopeptidase 1 OS=Homo sapiens GN=XPNPEP1 PE=1 SV=3	4.08	2.57	4.78	5.56	4.90	7.33	4.45	0.90	1.37	3.71	4.09	2.12	0.00	1.61	4.77	2.52	1.27	0.77	1.81	0.00	0.00	2.96	5.25	0.80	3.82	1.81	>0.999999999999999	2.1
P55060	Exportin-2 OS=Homo sapiens GN=CSE1L PE=1 SV=3	2.45	3.26	3.82	4.06	3.02	3.78	1.54	0.00	0.00	1.67	1.42	1.31	0.00	0.00	0.72	0.50	0.76	0.00	0.54	0.00	0.00	0.00	1.70	0.48	2.19	0.39	>0.999999999999999	5.6
P07357	Complement component C8 alpha chain OS=Homo sapiens GN=C8A PE=1 SV=2	3.06	0.00	1.20	0.00	0.00	1.18	0.85	0.45	2.05	2.78	0.00	2.69	4.94	1.61	0.00	5.55	1.90	0.77	2.71	1.95	0.84	0.40	0.00	0.60	1.19	1.77	>0.999999999999999	0.7
P35754	Glutaredoxin-1 OS=Homo sapiens GN=GLRX PE=1 SV=2	13.25	4.80	6.69	13.90	21.12	6.62	19.15	5.87	3.83	5.19	4.40	20.31	6.59	10.44	6.68	10.94	8.22	9.97	5.06	3.64	10.97	5.19	9.22	4.48	10.43	7.62	>0.999999999999999	1.4
P67809	Nuclease-sensitive element-binding protein 1 OS=Homo sapiens GN=YBX1 PE=1 SV=3	3.87	3.26	4.54	4.06	3.58	7.09	5.13	0.81	0.00	5.56	6.61	2.45	0.00	0.00	4.53	0.00	0.00	2.92	3.44	0.00	0.00	1.52	0.00	1.20	3.91	1.13	>0.999999999999999	3.5
P16989	DNA-binding protein A OS=Homo sapiens GN=CSDA PE=1 SV=4	3.47	2.92	4.06	0.00	0.00	4.02	4.62	0.00	0.00	0.00	0.00	2.20	0.00	0.00	6.44	1.35	0.00	0.00	0.00	0.00	0.00	0.00	0.00	0.00	1.77	0.65	>0.999999999999999	2.7
Q9Y3F4	Serine-threonine kinase receptor-associated protein OS=Homo sapiens GN=STRAP PE=1 SV=1	5.71	3.09	6.69	5.99	9.62	9.22	3.08	0.81	1.23	5.19	4.40	1.47	2.12	0.00	4.29	1.51	1.14	1.38	3.26	0.00	1.52	4.07	3.97	1.12	4.71	2.03	>0.999999999999999	2.3
P09914	Interferon-induced protein with tetratricopeptide repeats 1 OS=Homo sapiens GN=IFIT1 PE=1 SV=2	3.87	0.00	0.00	1.28	3.58	0.00	8.38	0.00	0.00	0.00	0.00	1.55	7.76	0.00	0.00	0.00	0.76	0.00	5.97	0.00	0.00	0.00	0.00	0.00	1.56	1.21	>0.999999999999999	1.3
O43866	CD5 antigen-like OS=Homo sapiens GN=CD5L PE=1 SV=1	5.51	0.00	0.00	0.00	0.00	0.00	1.37	0.72	2.33	0.00	0.00	0.00	6.35	4.34	0.00	4.54	4.81	0.00	4.88	1.04	0.00	0.64	1.14	0.00	0.83	2.31	>0.999999999999999	0.4

P42224	Signal transducer and activator of transcription 1-alpha/beta OS=Homo sapiens GN=STAT1 PE=1 SV=2	4.08	1.37	0.00	4.28	8.30	1.89	5.81	0.00	0.00	1.48	0.63	3.59	4.70	0.64	0.95	0.67	1.01	0.61	4.52	0.00	0.00	1.28	1.70	0.32	2.62	1.37	>0.999999999999999	1.9
P06318	Ig lambda chain V-VI region WLT OS=Homo sapiens PE=1 SV=1	5.71	4.80	6.69	5.99	5.28	0.00	4.79	2.53	3.83	5.19	4.40	0.00	0.00	4.50	6.68	4.71	3.54	4.30	0.00	3.64	0.00	0.00	9.22	1.12	4.10	3.14	>0.999999999999999	1.3
P32455	Interferon-induced guanylate-binding protein 1 OS=Homo sapiens GN=GBP1 PE=1 SV=2	4.28	0.00	0.00	7.06	8.67	0.00	9.06	0.90	0.88	0.00	0.00	3.75	6.12	0.80	0.00	0.00	1.27	0.77	5.97	0.00	0.00	1.20	2.13	0.60	2.87	1.57	>0.999999999999999	1.8
P32456	Interferon-induced guanylate-binding protein 2 OS=Homo sapiens GN=GBP2 PE=2 SV=3	2.04	0.00	0.00	0.00	0.00	0.00	0.00	0.00	0.00	0.00	0.00	1.71	0.00	0.00	0.00	0.00	0.00	0.00	0.00	0.00	0.00	0.00	0.00	0.00	0.31	0.00	>0.999999999999999	#DIV/0!
P05155	Plasma protease C1 inhibitor OS=Homo sapiens GN=SERPING1 PE=1 SV=2	3.87	0.00	4.54	4.06	4.90	2.84	7.01	2.98	4.52	3.52	1.89	3.34	7.76	1.93	6.20	4.37	3.29	5.06	4.70	6.49	4.39	2.08	2.70	1.04	3.62	4.17	>0.999999999999999	0.9
P07858	Cathepsin B OS=Homo sapiens GN=CTSB PE=1 SV=3	5.71	4.80	0.00	0.00	0.00	2.13	6.67	3.52	2.47	0.00	2.83	3.18	12.00	4.50	6.68	8.58	3.54	5.98	7.05	2.34	3.04	3.12	3.97	3.12	2.61	5.33	>0.999999999999999	0.5
P11586	C-1-tetrahydrofolate synthase, cytoplasmic OS=Homo sapiens GN=MTHFD1 PE=1 SV=3	4.28	3.60	5.02	8.77	3.21	4.96	1.71	0.63	0.96	3.15	2.04	0.82	1.65	1.61	3.10	1.68	0.89	0.00	3.07	0.00	0.00	2.32	3.55	0.68	3.26	1.54	>0.999999999999999	2.1
Q96TA1	Niban-like protein 1 OS=Homo sapiens GN=FAM129B PE=1 SV=3	5.30	3.60	5.02	4.49	3.96	6.14	1.37	1.08	2.88	2.22	2.52	2.45	2.82	1.28	3.82	3.53	2.02	1.84	3.80	0.00	0.68	0.96	1.70	0.84	3.42	1.94	>0.999999999999999	1.8
Q9P2E9	Ribosome-binding protein 1 OS=Homo sapiens GN=RRBP1 PE=1 SV=4	2.24	0.00	1.67	2.35	1.32	1.65	0.68	0.36	1.92	0.00	1.42	0.73	0.47	0.00	0.95	0.67	1.14	0.31	2.89	0.00	1.18	0.16	1.99	0.28	1.20	0.84	>0.999999999999999	1.4
O14950	Myosin regulatory light chain 12B OS=Homo sapiens GN=MYL12B PE=1 SV=2	7.55	2.92	8.84	7.91	11.31	8.74	6.33	3.34	0.00	11.12	9.44	4.89	4.00	2.73	8.82	10.09	2.15	2.61	3.07	2.21	2.87	2.96	5.25	0.68	6.87	3.95	>0.999999999999999	1.7
Q16181	Septin-7 OS=Homo sapiens GN=SEPT7 PE=1 SV=2	4.28	3.60	5.02	4.49	2.45	6.62	4.79	1.17	0.00	3.89	5.82	0.00	0.00	3.37	6.68	0.00	1.64	4.30	5.06	0.00	0.00	1.68	3.97	0.52	3.51	2.27	>0.999999999999999	1.5
P15880	40S ribosomal protein S2 OS=Homo sapiens GN=RPS2 PE=1 SV=2	4.49	3.77	11.71	13.90	12.26	8.27	5.98	3.16	3.01	4.08	10.22	2.85	2.59	5.62	11.69	3.70	4.43	1.69	6.33	0.00	0.00	5.19	6.95	0.88	6.98	4.09	>0.999999999999999	1.7
P47756	F-actin-capping protein subunit beta OS=Homo sapiens GN=CAPZB PE=1 SV=4	4.49	1.89	8.37	7.48	9.24	8.27	3.76	1.99	4.79	4.08	5.50	4.00	8.23	3.53	5.25	5.89	2.78	3.38	8.86	1.43	3.71	3.91	6.95	2.60	5.32	4.71	>0.999999999999999	1.1
P50135	Histamine N-methyltransferase OS=Homo sapiens GN=HNMT PE=1 SV=1	4.28	1.72	7.89	7.06	3.96	4.96	5.64	1.90	0.00	3.89	1.57	0.82	2.35	0.00	2.38	1.68	1.27	0.00	1.81	0.00	0.00	1.68	1.42	0.40	3.64	1.08	>0.999999999999999	3.4
P02747	Complement C1q subcomponent subunit C OS=Homo sapiens GN=C1QC PE=1 SV=3	8.97	0.00	0.00	2.78	5.09	0.00	4.62	1.17	3.70	8.15	2.04	2.20	14.58	7.06	6.44	10.43	7.84	1.99	4.88	3.51	2.19	2.16	8.80	1.08	3.23	5.91	>0.999999999999999	0.5
P01880	Ig delta chain C region OS=Homo sapiens GN=IGHD PE=1 SV=2	7.14	1.37	8.37	11.97	8.48	1.89	1.37	0.72	1.10	2.96	5.50	0.65	5.88	1.28	10.73	13.63	2.02	1.23	4.52	0.00	0.00	1.28	6.39	0.32	4.29	3.94	>0.999999999999999	1.1
Q9BRF8	Calcineurin-like phosphoesterase domain-containing protein 1 OS=Homo sapiens GN=CPPED1 PE=1 SV=3	3.87	5.15	10.04	8.98	7.92	9.92	7.18	1.72	1.23	5.56	6.61	0.00	2.12	1.45	7.15	0.00	2.40	6.44	3.44	0.00	1.52	2.40	4.26	0.36	5.68	2.63	>0.999999999999999	2.2
Q7KZF4	Staphylococcal nuclease domain-containing protein 1 OS=Homo sapiens GN=SND1 PE=1 SV=1	3.47	2.92	1.43	2.78	4.71	3.07	3.59	0.54	0.41	1.11	5.19	2.04	0.00	0.48	1.43	1.68	1.27	1.53	3.07	0.00	0.51	0.48	1.42	1.00	2.61	1.07	>0.999999999999999	2.4
P15531	Nucleoside diphosphate kinase A OS=Homo sapiens GN=NME1 PE=1 SV=1	8.77	7.38	24.86	15.18	3.77	24.58	17.78	0.00	5.89	7.97	11.17	11.75	10.11	16.70	10.26	11.95	8.98	10.89	12.84	5.58	7.26	5.67	6.10	2.84	11.59	9.10	>0.999999999999999	1.3
P22392	Nucleoside diphosphate kinase B OS=Homo sapiens GN=NME2 PE=1 SV=1	8.77	0.00	0.00	0.00	0.00	0.00	17.61	3.88	0.00	0.00	11.01	11.66	0.00	16.54	0.00	11.78	8.86	0.00	0.00	9.09	7.26	0.00	9.93	2.80	4.41	5.52	>0.999999999999999	0.8
P55058	Phospholipid transfer protein OS=Homo sapiens GN=PLTP PE=1 SV=1	3.87	3.26	0.00	0.00	3.58	1.42	2.05	1.72	1.64	0.00	0.94	2.12	2.82	5.46	0.00	2.02	3.29	3.99	2.17	3.38	3.21	1.52	1.70	1.68	1.72	2.60	>0.999999999999999	0.7
Q32MZ4	Leucine-rich repeat flightless-interacting protein 1 OS=Homo sapiens GN=LRRFIP1 PE=1 SV=2	3.06	5.66	9.08	7.06	0.75	6.85	3.42	1.35	1.51	4.45	5.98	1.22	1.65	1.77	6.92	3.36	0.89	2.30	1.99	0.00	1.18	2.32	2.84	0.80	4.20	2.17	>0.999999999999999	1.9
Q8IZP2	Putative protein FAM10A4 OS=Homo sapiens GN=ST13P4 PE=5 SV=1	5.30	9.95	13.86	16.46	7.73	13.71	0.00	3.70	5.62	14.26	0.00	3.34	2.82	6.58	18.36	9.76	5.19	3.99	7.42	0.00	2.03	3.28	5.82	2.32	7.83	5.63	>0.999999999999999	1.4
Q8N335	Glycerol-3-phosphate dehydrogenase 1-like protein OS=Homo sapiens GN=GPD1L PE=1 SV=1	3.67	4.80	11.95	10.69	17.35	6.62	4.79	3.43	2.47	11.67	9.91	0.65	1.88	6.10	15.03	3.03	4.81	2.76	3.26	0.00	4.73	8.63	15.33	0.72	7.33	5.52	>0.999999999999999	1.3
P17931	Galectin-3 OS=Homo sapiens GN=LGALS3 PE=1 SV=5	5.51	4.63	3.11	5.77	8.11	14.65	10.60	2.44	1.78	5.00	12.90	10.69	6.35	6.90	3.10	7.23	13.29	9.51	14.83	3.51	2.19	2.16	8.80	5.24	7.10	6.93	>0.999999999999999	1.0
P01033	Metalloproteinase inhibitor 1 OS=Homo sapiens GN=TIMP1 PE=1 SV=1	14.07	8.23	11.47	6.41	17.54	3.31	11.80	8.40	6.57	8.89	2.20	9.79	3.29	11.08	7.15	8.08	6.07	14.27	8.68	6.23	8.10	9.59	6.81	3.72	9.06	7.76	>0.999999999999999	1.2
Q15847	Adipose most abundant gene transcript 2 protein OS=Homo sapiens GN=APM2 PE=1 SV=1	22.23	7.72	48.53	23.30	38.28	25.76	18.64	9.84	14.93	20.19	31.93	8.89	10.58	7.23	48.41	34.15	13.79	31.15	19.71	14.15	7.60	16.22	28.81	4.36	22.52	19.68	>0.999999999999999	1.1
P57721	Poly(rC)-binding protein 3 OS=Homo sapiens GN=PCBP3 PE=1 SV=2	5.51	0.00	0.00	0.00	0.00	0.00	0.00	0.00	0.00	0.00	0.00	2.20	0.00	2.73	0.00	0.00	0.00	0.00	0.00	0.00	0.00	0.00	0.00	0.00	0.64	0.23	>0.999999999999999	2.8
P61247	40S ribosomal protein S3a OS=Homo sapiens GN=RPS3A PE=1 SV=2	4.69	6.35	8.84	4.92	16.40	16.30	8.89	0.99	1.51	6.85	16.98	4.24	8.70	3.69	12.40	3.87	2.91	5.68	9.41	0.00	0.00	5.51	9.79	1.48	8.08	5.29	>0.999999999999999	1.5
P04632	Calpain small subunit 1 OS=Homo sapiens GN=CAPNS1 PE=1 SV=1	7.95	4.29	17.69	8.34	13.95	13.23	6.67	3.52	5.34	10.37	8.81	6.04	0.00	6.26	13.36	9.42	3.16	8.59	10.13	3.25	4.22	3.12	10.50	1.56	8.85	6.13	>0.999999999999999	1.4
O95433	Activator of 90 kDa heat shock protein ATPase homolog 1 OS=Homo sapiens GN=AHSA1 PE=1 SV=1	5.71	4.80	9.32	8.34	5.28	12.05	6.67	2.53	3.83	9.45	6.13	1.47	2.12	2.89	9.30	3.03	1.14	5.98	3.26	0.00	1.52	4.07	5.53	0.72	6.30	3.30	>0.999999999999999	1.9
P61960	Ubiquitin-fold modifier 1 OS=Homo sapiens GN=UFM1 PE=1 SV=1	18.35	15.44	9.08	8.12	7.16	21.27	15.39	3.43	5.20	7.04	25.48	3.10	8.94	14.45	9.06	43.91	0.00	5.83	6.87	0.00	6.41	12.94	12.77	3.60	11.59	10.40	>0.999999999999999	1.1
Q9P1F3	Costars family protein C6orf115 OS=Homo sapiens GN=C6orf115 PE=1 SV=1	7.75	6.52	9.08	8.12	7.16	21.50	6.50	3.43	5.20	7.04	5.98	3.10	8.94	6.10	9.06	6.39	4.81	5.83	6.87	4.93	0.00	3.04	23.41	1.52	7.62	6.74	>0.999999999999999	1.1
A6NL28	Putative tropomyosin alpha-3 chain-like protein OS=Homo sapiens PE=5 SV=2	2.65	2.23	6.45	2.78	2.45	3.07	4.62	1.17	1.78	2.41	4.25	0.00	3.06	2.09	6.44	4.54	1.64	1.99	2.35	1.69	2.19	1.04	1.84	0.52	2.82	2.45	>0.999999999999999	1.2
Q9NZT1	Calmodulin-like protein 5 OS=Homo sapiens GN=CALML5 PE=1 SV=2	9.58	27.96	5.02	16.89	3.96	0.00	8.04	1.90	2.88	14.64	18.40	3.83	4.94	35.32	11.21	19.68	14.80	3.22	14.29	6.10	7.93	6.31	2.98	4.68	9.42	10.96	>0.999999999999999	0.9
P15153	Ras-related C3 botulinum toxin substrate 2 OS=Homo sapiens GN=RAC2 PE=1 SV=1	10.81	0.00	0.00	11.33	0.00	0.00	5.64	2.98	0.00	6.11	0.00	4.32	3.53	5.30	0.00	5.55	4.18	0.00	0.00	4.28	8.95	0.00	0.00	1.32	3.43	2.76	>0.999999999999999	1.2
P63000	Ras-related C3 botulinum toxin substrate 1 OS=Homo sapiens GN=RAC1 PE=1 SV=1	10.81	9.09	12.67	11.33	9.99	7.80	0.00	0.00	7.26	0.00	0.00	0.00	0.00	0.00	12.64	0.00	0.00	8.13	9.59	0.00	0.00	4.23	7.52	0.00	5.75	3.51	>0.999999999999999	1.6
P04004	Vitronectin OS=Homo sapiens GN=VTN PE=1 SV=1	5.30	1.03	1.43	4.06	9.43	1.42	3.25	1.72	3.56	2.22	1.89	2.77	8.00	3.05	2.86	2.02	7.47	3.99	3.44	4.41	5.74	0.00	0.00	0.48	3.17	3.45	>0.999999999999999	0.9
P40121	Macrophage-capping protein OS=Homo sapiens GN=CAPG PE=1 SV=2	5.71	3.09	2.15	5.99	3.39	9.22	6.67	3.52	1.23	5.19	2.83	2.28	12.00	4.50	6.68	6.56	2.28	5.98	5.06	1.17	4.73	3.12	2.55	1.12	4.27	4.65	>0.999999999999999	0.9
Q16666	Gamma-interferon-inducible protein 16 OS=Homo sapiens GN=IFI16 PE=1 SV=3	2.24	1.89	3.82	6.20	3.02	5.67	3.42	0.36	0.00	2.04	3.15	0.90	2.59	0.00	0.95	1.85	0.51	1.69	1.99	0.00	0.00	0.64	1.56	0.32	2.73	1.01	>0.999999999999999	2.7
P48637	Glutathione synthetase OS=Homo sapiens GN=GSS PE=1 SV=1	5.71	7.55	6.69	17.96	8.30	17.25	7.52	3.25	3.83	6.67	8.34	4.32	8.47	8.51	10.49	6.06	5.57	6.75	9.59	0.00	1.01	5.03	5.11	0.80	8.12	5.62	>0.999999999999999	1.4
P55036	26S proteasome non-ATPase regulatory subunit 4 OS=Homo sapiens GN=PSMD4 PE=1 SV=1	1.63	4.46	11.24	10.05	1.51	11.11	4.45	0.00	0.00	6.67	7.39	2.94	1.88	2.73	11.21	1.35	0.00	0.00	3.07	0.00	0.00	1.36	2.41	0.68	5.12	2.06	>0.999999999999999	2.5
Q9BS40	Latexin OS=Homo sapiens GN=LXN PE=1 SV=2	5.51	10.81	6.45	9.41	8.30	10.40	7.52	2.44	3.70	5.00	6.92	2.20	10.35	10.12	10.49	7.40	3.42	4.14	2.35	0.00	0.00	3.51	6.24	1.08	6.55	4.93	>0.999999999999999	1.3
P15170	Eukaryotic peptide chain release factor GTP-binding subunit ERF3A OS=Homo sapiens GN=GSPT1 PE=1 SV=1	3.87	7.03	2.87	2.57	4.71	4.49	4.27	0.54	0.00	4.63	3.93	0.98	0.00	3.05	4.53	2.02	0.00	2.92	7.42	0.00	1.01	0.96	3.55	0.48	3.33	2.16	>0.999999999999999	1.5
P06748	Nucleophosmin OS=Homo sapiens GN=NPM1 PE=1 SV=2	2.04	3.60	8.13	4.49	8.86	4.96	1.71	1.90	1.37	8.71	7.39	2.77	4.94	1.61	2.38	1.68	1.27	1.53	3.80	0.00	1.69	0.00	6.67	0.84	4.66	2.20	>0.999999999999999	2.1
P10643	Complement component C7 OS=Homo sapiens GN=C7 PE=1 SV=2	2.24	0.00	0.72	0.64	0.00	0.71	0.51	0.63	0.96	0.56	0.00	0.90	0.71	0.48	1.67	3.70	0.89	0.00	3.98	0.39	0.51	0.56	2.55	0.00	0.66	1.29	>0.999999999999999	0.5
Q9GZZ8	Extracellular glycoprotein lacritin OS=Homo sapiens GN=LACRT PE=1 SV=1	18.56	15.61	21.75	11.55	17.16	21.50	15.56	8.22	3.29	4.45	14.31	7.42	31.99	14.61	21.70	9.09	11.51	13.96	16.46	11.81	9.11	7.27	12.91	3.64	13.28	13.67	>0.999999999999999	1.0
Q13630	GDP-L-fucose synthase OS=Homo sapiens GN=TSTA3 PE=1 SV=1	3.87	3.26	2.15	4.06	1.70	4.49	3.25	0.81	1.23	0.00	1.42	0.73	4.47	1.45	4.53	1.51	1.14	1.38	5.43	2.47	3.21	0.00	1.28	1.68	2.25	2.38	>0.999999999999999	0.9
P14780	Matrix metalloproteinase-9 OS=Homo sapiens GN=MMP9 PE=1 SV=3	1.63	0.69	0.00	0.86	0.00	0.00	0.68	2.98	0.00	0.00	0.00	0.00	8.94	0.64	0.00	0.00	0.51	3.38	0.72	4.28	14.01	0.00	0.00	0.00	0.57	2.71	>0.999999999999999	0.2
Q96C19	EF-hand domain-containing protein D2 OS=Homo sapiens GN=EFHD2 PE=1 SV=1	5.30	7.20	10.04	33.35	11.31	9.92	7.18	3.79	8.22	7.78	12.58	6.53	2.82	9.63	14.31	7.07	10.12	6.44	14.47	1.56	4.39	10.22	18.16	1.04	10.27	8.35	>0.999999999999999	1.2
P48444	Coatomer subunit delta OS=Homo sapiens GN=ARCN1 PE=1 SV=1	3.67	1.03	5.98	11.97	3.39	5.91	5.47	2.89	2.47	3.33	6.29	3.26	4.23	0.96	9.54	4.21	1.52	0.92	4.52	0.78	3.04	3.20	4.54	0.72	4.64	3.18	>0.999999999999999	1.5
Q15008	26S proteasome non-ATPase regulatory subunit 6 OS=Homo sapiens GN=PSMD6 PE=1 SV=1	4.69	3.95	10.04	4.92	14.14	7.56	3.93	1.35	0.00	7.78	3.62	2.61	1.65	2.41	7.63	3.87	1.90	3.53	4.16	0.91	1.18	1.20	3.26	0.60	5.38	2.69	>0.999999999999999	2.0
Q6PCE3	Glucose 1,6-bisphosphate synthase OS=Homo sapiens GN=PGM2L1 PE=1 SV=3	2.85	1.54	10.28	2.99	1.70	4.73	2.39	1.26	0.68	2.59	4.88	1.63	0.00	1.45	7.39	0.84	0.63	0.77	2.53	0.00	0.00	1.60	6.10	1.00	3.13	1.86	>0.999999999999999	1.7
P63151	Serine/threonine-protein phosphatase 2A 55 kDa regulatory subunit B alpha isoform OS=Homo sapiens GN=PPP2R	2.65	2.23	12.91	5.99	6.79	10.40	3.42	1.17	1.78	3.71	6.92	1.06	3.06	0.00	8.59	2.19	0.00	0.00	3.62	0.00	0.00	1.60	2.84	0.52	4.92	1.87	>0.999999999999999	2.6
P05452	Tetranectin OS=Homo sapiens GN=CLEC3B PE=1 SV=3	6.32	2.57	7.41	6.63	5.85	3.54	0.00	2.80	2.05	9.45	0.00	1.22	12.00	0.00	7.39	16.49	3.92	2.30	5.61	1.95	2.53	5.83	4.40	0.60	3.99	5.25	>0.999999999999999	0.8
P13010	X-ray repair cross-complementing protein 5 OS=Homo sapiens GN=XRCC5 PE=1 SV=3	2.45	2.92	10.04	6.63	3.21	11.11	2.05	0.72	1.10	4.82	4.88	2.53	1.88	3.37	2.86	2.02	2.15	1.23	3.07	0.00	0.00	1.36	2.41	0.84	4.37	1.77	>0.999999999999999	2.5
Q99613	Eukaryotic translation initiation factor 3 subunit C OS=Homo sapiens GN=EIF3C PE=1 SV=1	2.65	0.51	1.43	1.28	1.13	3.78	0.51	0.00	0.00	2.41	2.04	0.49	0.00	0.48	1.43	0.50	0.00	0.00	0.00	0.00	0.00	0.00	1.42	0.00	1.35	0.32	>0.999999999999999	4.2
P14923	Junction plakoglobin OS=Homo sapiens GN=JUP PE=1 SV=3	1.63	2.06	6.22	5.56	2.26	5.20	0.00	0.00	0.00	1.48	2.67	2.12	0.94	0.64	4.05	2.86	0.00	0.61	1.45	0.00	0.00	1.36	3.12	0.68	2.43	1.31	>0.999999999999999	1.9
P04207	Ig kappa chain V-III region CLL OS=Homo sapiens PE=1 SV=2	4.89	0.00	0.00	0.00	0.00	0.00	4.10	4.79	7.26	0.00	0.00	0.00	0.00	3.85	0.00	0.00	3.04	0.00	4.34	0.00	8.95	0.00	7.52	0.00	1.75	2.31	>0.999999999999999	0.8
P01011	Alpha-1-antichymotrypsin OS=Homo sapiens GN=SERPINA3 PE=1 SV=2	4.49	1.20	0.00	0.00	0.00	0.00	1.20	1.99	3.01	0.00	0.00	0.00	1.65	1.12	0.00	2.36	0.00	0.00	0.00	1.82	0.00	2.48	1.99	0.00	0.99	0.95	>0.999999999999999	1.0
O14980	Exportin-1 OS=Homo sapiens GN=XPO1 PE=1 SV=1	1.02	1.89	1.20	1.71	0.94	1.89	1.37	0.27	0.00	1.48	0.79	0.24	0.71	0.48	1.91	1.85	0.38	0.00	0.54	0.39	0.51	0.40	1.14	0.44	1.07	0.73	>0.999999999999999	1.5
P22059	Oxysterol-binding protein 1 OS=Homo sapiens GN=OSBP PE=1 SV=1	2.24	1.89	6.69	2.35	3.77	3.54	1.88	0.00	0.55	2.04	1.10	0.57	0.00	0.64	2.62	0.00	0.89	0.00	0.00	0.00	0.00	0.32	1.56	0.44	2.22	0.54	>0.999999999999999	4.1
P35080	Profilin-2 OS=Homo sapiens GN=PFN2 PE=1 SV=3	4.49	8.41	11.71	10.48	9.24	11.58	3.76	4.43	3.01	9.08	7.71	4.00	5.17	7.87	5.25	8.24	6.20	7.52	3.98	0.00	0.00	1.76	6.95	0.88	7.32	4.49	>0.999999999999999	1.6
O15371	Eukaryotic translation initiation factor 3 subunit D OS=Homo sapiens GN=EIF3D PE=1 SV=1	3.26	2.74	6.69	1.07	3.02	5.20	2.74	0.45	0.68	2.96	2.52	0.00	2.35	0.80	5.25	0.00	1.27	0.00	1.81	0.00	0.00	0.00	2.27	0.20	2.61	1.16	>0.999999999999999	2.2
P35237	Serpin B6 OS=Homo sapiens GN=SERPINB6 PE=1 SV=3	5.10	6.00	5.98	5.34	6.60	8.27	5.98	2.26	3.42	4.63	5.50	2.04	0.00	4.01	5.96	4.21	3.16	3.84	2.89	0.00	2.70	3.59	4.97	0.64	5.09	3.00	>0.999999999999999	1.7
P50452	Serpin B8 OS=Homo sapiens GN=SERPINB8 PE=1 SV=2	3.26	2.74	0.00	1.71	0.00	1.89	2.74	0.00	2.19	0.00	0.00	2.04	3.76	2.57	5.96	2.69	0.00	3.84	4.52	0.00	0.00	2.00	0.00	1.00	1.38	2.20	>0.999999999999999	0.6
P23246	Splicing factor, proline- and glutamine-rich OS=Homo sapiens GN=SFPQ PE=1 SV=2	2.65	3.09	4.30	4.92	3.39	5.43	1.54	0.81	0.00	3.33	4.56	1.06	0.00	2.09	4.29	3.03	1.14	0.61	1.63	0.00	0.00	1.04	2.55	0.52	2.92	1.41	>0.999999999999999	2.1
P55210	Caspase-7 OS=Homo sapiens GN=CASP7 PE=1 SV=1	4.08	3.43	0.00	2.14	3.77	0.00	1.71	0.00	1.37	0.00	0.00	2.53	2.35	0.00	0.00	1.68	1.27	0.00	3.62	0.00	0.00	1.60	2.84	0.40	1.59	1.15	>0.999999999999999	1.4
Q99536	Synaptic vesicle membrane protein VAT-1 homolog OS=Homo sapiens GN=VAT1 PE=1 SV=2	3.26	1.37	1.91	1.71	0.00	1.89	2.74	0.72	2.19	0.00	0.00	0.00	0.00	0.00	1.91	1.35	0.00	0.00	0.00	1.04	4.22	0.64	1.14	0.32	1.32	0.88	>0.999999999999999	1.5
P55209	Nucleosome assembly protein 1-like 1 OS=Homo sapiens GN=NAP1L1 PE=1 SV=1	3.06	2.57	10.04	6.84	9.80	7.56	2.56	1.35	2.05	5.93	3.62	1.88	3.53	1.12	7.63	3.87	2.91	2.30	1.27	0.00	1.18	1.20	3.26	0.60	4.77	2.41	>0.999999999999999	2.0
Q99733	Nucleosome assembly protein 1-like 4 OS=Homo sapiens GN=NAP1L4 PE=1 SV=1	3.26	0.00	10.76	0.00	4.71	5.91	2.74	0.00	0.00	4.63	3.93	0.00	0.00	0.00	3.82	0.00	2.02	2.45	0.00	0.00	0.00	0.00	2.27	0.32	3.00	0.91	>0.999999999999999	3.3
P22102	Trifunctional purine biosynthetic protein adenosine-3 OS=Homo sapiens GN=GART PE=1 SV=1	2.65	2.23	8.13	4.06	4.34	3.78	3.25	0.00	0.00	3.52	4.25	1.55	0.00	0.96	6.44	1.51	0.00	1.38	0.54	0.00	0.00	1.28	2.70	0.36	3.15	1.26	>0.999999999999999	2.5
Q07960	Rho GTPase-activating protein 1 OS=Homo sapiens GN=ARHGAP1 PE=1 SV=1	4.28	4.97	5.02	9.83	3.96	6.85	3.59	1.17	2.88	2.41	4.56	1.71	1.65	2.09	6.92	3.53	2.66	1.99	3.80	0.00	3.54	1.04	1.84	0.52	4.27	2.47	>0.999999999999999	1.7
P12830	Cadherin-1 OS=Homo sapiens GN=CDH1 PE=1 SV=3	0.61	0.51	0.72	0.64	1.89	0.71	0.00	0.63	1.37	1.30	0.00	1.14	0.71	0.00	0.72	0.00	0.89	0.00	1.27	0.91	0.51	0.00	0.00	0.40	0.79	0.45	>0.999999999999999	1.8
P60660	Myosin light polypeptide 6 OS=Homo sapiens GN=MYL6 PE=1 SV=2	8.77	0.00	10.28	9.19	8.11	10.16	7.35	6.50	0.00	7.97	6.76	5.87	4.70	0.00	10.26	12.11	5.44	3.07	13.02	5.58	12.15	5.75	10.22	2.88	6.75	7.10	>0.999999999999999	1.0
P04217	Alpha-1B-glycoprotein OS=Homo sapiens GN=A1BG PE=1 SV=4	6.93	2.06	8.13	5.56	6.41	8.03	5.81	1.08	6.85	7.78	1.89	5.63	6.12	9.47	8.11	9.93	4.30	6.44	9.04	4.41	5.74	2.72	1.70	1.04	5.51	5.75	>0.999999999999999	1.0
P61086	Ubiquitin-conjugating enzyme E2 K OS=Homo sapiens GN=UBE2K PE=1 SV=3	6.53	5.49	7.65	6.84	6.03	3.54	8.89	1.35	2.05	9.63	2.36	2.61	3.53	5.14	7.63	5.38	1.90	2.30	0.00	1.95	2.53	1.20	2.13	4.00	5.25	3.14	>0.999999999999999	1.7
P09871	Complement C1s subcomponent OS=Homo sapiens GN=C1S PE=1 SV=1	1.84	0.69	0.00	2.78	4.34	0.00	3.08	1.17	2.47	1.67	0.00	2.69	3.06	1.45	2.15	5.55	4.93	0.61	4.16	2.34	0.00	0.32	1.84	0.36	1.73	2.23	>0.999999999999999	0.8
P31151	Protein S100-A7 OS=Homo sapiens GN=S100A7 PE=1 SV=4	24.27	65.01	16.49	81.03	13.01	0.00	11.80	16.62	16.30	34.09	0.00	2.45	16.23	60.85	43.88	45.26	23.28	10.59	12.48	34.92	11.65	14.70	9.79	4.76	23.42	24.03	>0.999999999999999	1.0
O75363	Breast carcinoma-amplified sequence 1 OS=Homo sapiens GN=BCAS1 PE=1 SV=2	2.24	3.95	5.50	3.63	4.34	7.09	3.93	0.99	4.11	2.04	5.66	1.88	2.59	1.77	8.59	2.86	1.39	1.69	6.51	0.00	0.84	3.51	4.26	0.68	3.78	2.89	>0.999999999999999	1.3
P26599	Polypyrimidine tract-binding protein 1 OS=Homo sapiens GN=PTBP1 PE=1 SV=1	2.45	2.06	4.30	10.26	6.03	9.45	4.27	1.63	0.82	3.33	6.29	3.26	4.23	1.93	4.29	2.02	0.76	3.84	3.26	0.78	2.03	1.44	3.55	1.28	4.51	2.45	>0.999999999999999	1.8
Q16222	UDP-N-acetylhexosamine pyrophosphorylase OS=Homo sapiens GN=UAP1 PE=1 SV=3	4.89	1.89	4.30	3.85	1.13	2.60	6.50	0.00	1.51	2.04	2.83	0.90	2.59	0.00	5.72	1.01	0.00	0.00	3.26	0.00	0.00	1.44	1.56	0.44	2.70	1.33	>0.999999999999999	2.0
P36873	Serine/threonine-protein phosphatase PP1-gamma catalytic subunit OS=Homo sapiens GN=PPP1CC PE=1 SV=1	8.16	3.09	0.00	6.20	7.54	15.60	4.96	0.00	0.00	5.37	0.00	0.00	0.00	0.00	6.92	0.00	0.00	2.76	0.00	0.00	1.52	0.00	0.00	1.60	4.24	1.07	>0.999999999999999	4.0
Q9H299	SH3 domain-binding glutamic acid-rich-like protein 3 OS=Homo sapiens GN=SH3BGRL3 PE=1 SV=1	6.73	5.66	7.89	16.46	25.64	18.20	13.17	6.95	10.55	6.11	12.11	6.28	7.76	0.00	0.00	12.95	17.21	11.81	13.93	17.66	22.96	2.64	4.68	1.32	11.31	9.41	>0.999999999999999	1.2
P62495	Eukaryotic peptide chain release factor subunit 1 OS=Homo sapiens GN=ETF1 PE=1 SV=3	2.85	2.40	9.08	6.41	3.96	7.09	3.59	0.63	0.96	2.59	7.55	1.71	0.00	2.25	7.15	2.36	0.89	3.22	2.53	0.00	0.00	2.40	4.26	0.28	4.07	2.11	>0.999999999999999	1.9
Q13421	Mesothelin OS=Homo sapiens GN=MSLN PE=1 SV=2	2.04	3.43	4.78	3.21	11.12	4.73	5.47	4.61	9.04	0.93	2.36	2.12	3.53	6.10	2.38	3.36	4.81	5.83	1.81	3.38	6.41	3.59	2.84	0.80	4.49	3.74	>0.999999999999999	1.2
Q7Z4S6	Kinesin-like protein KIF21A OS=Homo sapiens GN=KIF21A PE=1 SV=2	1.02	3.60	6.93	4.92	1.32	7.56	0.85	0.18	0.27	3.89	3.30	0.24	0.00	0.80	2.62	0.00	0.25	0.00	0.00	0.00	0.00	0.00	0.99	0.08	2.84	0.40	>0.999999999999999	7.2
P62826	GTP-binding nuclear protein Ran OS=Homo sapiens GN=RAN PE=1 SV=3	5.91	15.27	11.24	14.32	12.63	15.83	11.46	6.05	12.19	12.41	18.09	2.37	15.76	7.55	11.21	14.97	5.95	10.28	12.12	8.70	11.31	5.35	6.67	1.88	11.48	9.31	>0.999999999999999	1.2
P26640	Valine--tRNA ligase OS=Homo sapiens GN=VARS PE=1 SV=4	1.02	1.72	2.39	2.14	1.32	0.47	1.20	0.00	0.00	0.93	1.10	0.16	0.00	0.80	1.67	1.18	0.25	0.31	1.81	0.00	0.00	0.96	0.99	0.20	1.04	0.68	>0.999999999999999	1.5
Q14258	E3 ubiquitin/ISG15 ligase TRIM25 OS=Homo sapiens GN=TRIM25 PE=1 SV=2	1.02	0.86	5.98	1.92	1.70	3.31	2.39	0.00	0.00	0.93	2.20	1.55	0.00	0.80	2.15	1.51	0.00	0.00	0.90	0.00	0.00	0.72	1.28	0.20	1.82	0.63	>0.999999999999999	2.9
P29966	Myristoylated alanine-rich C-kinase substrate OS=Homo sapiens GN=MARCKS PE=1 SV=4	4.49	10.98	8.37	4.70	1.89	2.36	5.98	0.00	1.37	1.85	3.46	1.79	2.35	0.00	2.38	1.68	1.27	3.38	1.81	0.00	3.71	3.91	0.00	0.40	3.94	1.74	>0.999999999999999	2.3
Q10567	AP-1 complex subunit beta-1 OS=Homo sapiens GN=AP1B1 PE=1 SV=2	2.65	3.43	3.11	7.70	2.45	4.73	3.42	0.90	0.41	2.41	5.03	2.28	1.41	0.00	5.72	3.36	0.76	0.92	4.34	0.00	0.00	0.80	2.84	0.96	3.21	1.76	>0.999999999999999	1.8
P48147	Prolyl endopeptidase OS=Homo sapiens GN=PREP PE=1 SV=2	1.63	1.37	6.45	6.84	2.45	3.07	3.76	0.36	0.00	3.15	1.26	1.06	0.00	1.28	4.05	2.19	1.64	0.61	4.88	0.00	0.00	2.16	3.83	0.68	2.62	1.78	>0.999999999999999	1.5
P11940	Polyadenylate-binding protein 1 OS=Homo sapiens GN=PABPC1 PE=1 SV=2	4.08	3.43	4.78	1.92	4.71	4.73	2.56	0.00	0.00	4.63	4.88	0.00	2.12	0.00	2.15	2.52	0.00	0.00	0.90	0.00	0.00	0.00	1.28	1.00	2.98	0.83	>0.999999999999999	3.6
Q13310	Polyadenylate-binding protein 4 OS=Homo sapiens GN=PABPC4 PE=1 SV=1	1.84	0.00	3.59	0.00	3.77	4.73	2.56	0.00	0.00	0.00	3.15	0.00	0.00	0.00	2.15	0.00	0.00	0.00	0.00	0.00	0.00	0.00	0.00	0.00	1.64	0.18	>0.999999999999999	9.1
Q15019	Septin-2 OS=Homo sapiens GN=SEPT2 PE=1 SV=1	3.26	1.37	11.00	7.70	4.90	13.71	2.74	0.72	0.00	4.82	5.66	3.75	1.88	1.28	8.59	1.35	2.02	0.00	4.70	0.00	1.35	2.08	6.53	1.04	4.97	2.57	>0.999999999999999	1.9
Q32P51	Heterogeneous nuclear ribonucleoprotein A1-like 2 OS=Homo sapiens GN=HNRNPA1L2 PE=2 SV=2	6.53	5.49	7.65	0.00	0.00	0.00	0.00	0.00	0.00	0.00	0.00	0.00	0.00	0.00	7.63	5.38	0.00	3.07	0.00	1.30	0.00	3.59	0.00	0.00	1.64	1.75	>0.999999999999999	0.9
Q00341	Vigilin OS=Homo sapiens GN=HDLBP PE=1 SV=2	0.41	0.34	1.67	1.07	0.94	0.47	0.34	0.00	0.00	0.93	0.31	0.41	0.00	0.00	0.00	0.34	0.00	0.00	0.36	0.00	0.00	0.16	0.71	0.08	0.57	0.14	>0.999999999999999	4.2
P13671	Complement component C6 OS=Homo sapiens GN=C6 PE=1 SV=3	1.22	1.03	2.15	1.28	1.70	0.71	0.51	0.81	2.74	1.11	0.00	3.18	1.41	1.45	2.15	3.87	2.02	1.99	2.89	1.69	0.51	0.72	2.27	0.64	1.37	1.80	>0.999999999999999	0.8
Q96FW1	Ubiquitin thioesterase OTUB1 OS=Homo sapiens GN=OTUB1 PE=1 SV=2	4.49	6.00	11.71	10.48	6.60	8.27	5.98	0.99	3.01	6.48	5.50	2.85	0.00	1.77	8.35	3.70	2.78	0.00	3.98	0.00	1.86	3.91	11.64	0.88	6.03	3.24	>0.999999999999999	1.9
Q15435	Protein phosphatase 1 regulatory subunit 7 OS=Homo sapiens GN=PPP1R7 PE=1 SV=1	7.34	4.46	6.22	7.70	6.79	6.14	8.04	0.00	1.10	1.48	5.66	2.12	0.00	2.73	4.05	2.86	1.01	1.23	4.70	0.00	0.00	1.36	2.41	1.04	4.75	1.78	>0.999999999999999	2.7
P05164	Myeloperoxidase OS=Homo sapiens GN=MPO PE=1 SV=1	2.45	0.69	0.00	0.86	0.00	0.00	2.05	2.35	0.00	0.00	0.00	0.65	0.94	1.93	0.00	0.00	2.66	3.22	3.80	3.38	6.92	0.00	0.00	0.00	0.75	1.90	>0.999999999999999	0.4
P26641	Elongation factor 1-gamma OS=Homo sapiens GN=EEF1G PE=1 SV=3	4.28	2.40	8.84	14.11	6.98	6.85	6.33	1.90	1.92	2.59	5.82	3.75	4.94	3.37	6.92	6.23	2.66	2.15	3.80	1.82	2.36	2.96	2.98	1.48	5.48	3.47	>0.999999999999999	1.6
P61769	Beta-2-microglobulin OS=Homo sapiens GN=B2M PE=1 SV=1	18.96	4.29	22.23	19.88	17.54	21.98	24.11	18.06	7.53	17.23	14.63	7.59	21.87	22.64	22.18	9.25	11.77	21.63	25.50	25.97	23.80	7.43	13.20	3.72	16.17	17.41	>0.999999999999999	0.9
Q04637	Eukaryotic translation initiation factor 4 gamma 1 OS=Homo sapiens GN=EIF4G1 PE=1 SV=4	0.82	0.34	1.91	1.28	0.75	0.47	1.03	0.00	0.00	0.00	1.26	0.16	0.00	0.32	0.95	0.34	0.25	0.31	0.36	0.00	0.00	0.00	0.85	0.32	0.67	0.31	>0.999999999999999	2.2
P07741	Adenine phosphoribosyltransferase OS=Homo sapiens GN=APRT PE=1 SV=2	3.47	2.92	4.06	12.83	16.59	20.79	10.26	1.54	0.00	0.00	2.67	9.79	0.00	5.94	14.31	6.23	0.00	2.61	6.69	2.21	0.00	7.03	5.25	2.40	7.08	4.39	>0.999999999999999	1.6
Q9BZQ8	Protein Niban OS=Homo sapiens GN=FAM129A PE=1 SV=1	0.61	2.23	6.69	5.99	3.21	8.51	5.47	1.17	0.82	3.15	1.57	0.82	0.71	2.09	4.77	1.68	0.00	0.92	2.35	0.39	1.01	2.24	1.84	0.24	3.35	1.52	>0.999999999999999	2.2
P28062	Proteasome subunit beta type-8 OS=Homo sapiens GN=PSMB8 PE=1 SV=3	4.69	3.95	5.50	7.70	6.79	8.51	3.93	3.25	3.15	4.26	3.62	1.88	5.41	3.69	2.62	3.87	2.91	3.53	9.22	0.00	1.86	4.07	3.26	0.44	4.77	3.41	>0.999999999999999	1.4
Q13561	Dynactin subunit 2 OS=Homo sapiens GN=DCTN2 PE=1 SV=4	3.06	4.12	10.28	7.06	8.11	7.80	4.10	0.63	0.00	7.97	5.19	2.69	0.00	3.85	10.26	2.52	1.90	2.30	2.71	0.00	0.00	1.92	3.41	0.96	5.08	2.49	>0.999999999999999	2.0
P62191	26S protease regulatory subunit 4 OS=Homo sapiens GN=PSMC1 PE=1 SV=1	4.28	2.40	7.17	8.12	2.64	7.09	2.39	0.63	0.00	3.89	7.39	2.45	0.00	0.00	7.15	6.39	1.77	2.15	6.87	0.00	0.00	2.40	5.39	1.20	4.04	2.78	>0.999999999999999	1.5
Q99584	Protein S100-A13 OS=Homo sapiens GN=S100A13 PE=1 SV=1	6.12	5.15	7.17	6.41	13.01	7.09	5.13	2.71	4.11	5.56	4.72	2.45	7.06	4.82	7.15	5.05	3.80	4.60	5.43	0.00	5.06	2.40	4.26	1.20	5.80	4.23	>0.999999999999999	1.4
P05198	Eukaryotic translation initiation factor 2 subunit 1 OS=Homo sapiens GN=EIF2S1 PE=1 SV=3	6.12	5.15	7.17	6.41	13.01	2.13	7.18	1.72	1.23	7.78	4.72	2.45	2.12	3.05	2.15	7.07	1.14	0.00	5.43	0.00	0.00	2.40	14.33	0.76	5.42	3.20	>0.999999999999999	1.7
P49913	Cathelicidin antimicrobial peptide OS=Homo sapiens GN=CAMP PE=1 SV=1	7.55	2.92	0.00	0.00	0.00	0.00	0.00	5.51	0.00	0.00	0.00	0.00	4.00	2.73	0.00	0.00	2.15	5.68	0.00	7.92	6.25	0.00	0.00	0.00	1.33	2.39	>0.999999999999999	0.6
O00391	Sulfhydryl oxidase 1 OS=Homo sapiens GN=QSOX1 PE=1 SV=3	3.47	3.60	0.96	4.49	3.96	2.84	6.16	3.79	4.93	0.74	4.88	4.32	2.82	4.17	4.05	2.86	2.66	4.76	4.70	2.21	5.23	2.88	5.11	1.44	3.68	3.57	>0.999999999999999	1.0
P52565	Rho GDP-dissociation inhibitor 1 OS=Homo sapiens GN=ARHGDIA PE=1 SV=3	6.32	12.18	16.97	10.69	9.43	16.78	5.30	2.80	4.25	5.74	11.17	5.79	16.70	8.03	16.93	8.41	1.77	7.67	12.84	4.02	8.44	3.99	7.10	1.24	8.95	8.10	>0.999999999999999	1.1
Q9UDY2	Tight junction protein ZO-2 OS=Homo sapiens GN=TJP2 PE=1 SV=2	0.41	0.34	0.48	1.07	0.38	0.47	0.34	0.18	0.27	0.37	1.10	0.16	0.00	0.32	0.48	0.84	0.25	0.00	0.00	0.00	0.00	0.00	0.28	0.08	0.46	0.19	>0.999999999999999	2.5
P39019	40S ribosomal protein S19 OS=Homo sapiens GN=RPS19 PE=1 SV=2	9.58	3.60	5.02	10.05	14.52	18.20	13.17	4.24	2.88	8.71	12.11	3.83	18.11	3.37	11.21	3.53	2.66	7.21	3.80	2.73	13.00	1.68	2.98	0.84	8.83	5.93	>0.999999999999999	1.5
P13798	Acylamino-acid-releasing enzyme OS=Homo sapiens GN=APEH PE=1 SV=4	2.45	2.06	1.91	4.70	4.90	6.14	3.76	0.72	1.64	0.74	2.67	0.98	0.00	1.28	0.95	2.02	2.15	1.84	2.17	0.00	1.35	0.64	1.70	0.16	2.72	1.19	>0.999999999999999	2.3
P45974	Ubiquitin carboxyl-terminal hydrolase 5 OS=Homo sapiens GN=USP5 PE=1 SV=2	2.24	3.09	5.26	5.56	3.39	5.20	2.39	0.99	0.96	4.08	3.46	1.79	0.00	2.25	7.39	4.37	0.38	2.15	1.99	0.00	0.00	1.76	3.12	0.44	3.20	1.99	>0.999999999999999	1.6
P40306	Proteasome subunit beta type-10 OS=Homo sapiens GN=PSMB10 PE=1 SV=1	2.24	1.89	5.74	5.13	7.16	8.98	6.50	0.99	1.51	4.45	5.98	3.10	5.64	0.00	5.72	6.39	3.04	1.69	4.34	1.43	0.00	0.88	1.56	1.52	4.47	2.68	>0.999999999999999	1.7
P46926	Glucosamine-6-phosphate isomerase 1 OS=Homo sapiens GN=GNPDA1 PE=1 SV=1	2.04	3.60	2.39	2.14	8.86	7.80	1.71	0.00	0.00	3.89	5.19	1.71	4.94	1.61	5.01	7.91	0.00	0.00	1.81	0.00	0.00	1.68	4.68	0.40	3.28	2.34	>0.999999999999999	1.4
P01780	Ig heavy chain V-III region JON OS=Homo sapiens PE=1 SV=1	5.51	0.00	0.00	0.00	0.00	0.00	0.00	0.00	0.00	0.00	0.00	0.00	6.35	4.34	0.00	0.00	3.42	0.00	0.00	3.51	4.56	0.00	3.83	0.00	0.46	2.17	>0.999999999999999	0.2
P55884	Eukaryotic translation initiation factor 3 subunit B OS=Homo sapiens GN=EIF3B PE=1 SV=3	1.43	1.20	4.54	3.21	3.58	2.60	1.88	0.36	0.55	2.04	2.99	1.22	1.65	1.12	3.58	0.67	0.89	0.61	2.71	0.00	0.00	0.56	2.70	0.60	2.13	1.26	>0.999999999999999	1.7
O95786	Probable ATP-dependent RNA helicase DDX58 OS=Homo sapiens GN=DDX58 PE=1 SV=2	1.84	0.00	0.72	0.64	1.70	1.42	2.74	0.00	0.00	0.56	0.94	0.49	0.71	0.00	0.72	0.00	0.00	0.00	1.63	0.00	0.00	0.00	0.00	0.00	0.92	0.25	>0.999999999999999	3.6
Q7L5N1	COP9 signalosome complex subunit 6 OS=Homo sapiens GN=COPS6 PE=1 SV=1	1.84	0.00	4.54	4.06	0.00	4.49	1.54	0.00	0.00	1.67	2.99	0.73	0.00	1.45	0.00	0.00	0.00	0.00	0.00	0.00	0.00	0.72	0.00	0.00	1.82	0.18	>0.999999999999999	10.1
P13693	Translationally-controlled tumor protein OS=Homo sapiens GN=TPT1 PE=1 SV=1	3.47	2.92	0.00	7.91	11.50	8.74	10.43	1.54	5.07	0.00	2.67	4.98	8.70	2.73	8.82	6.23	4.68	5.68	15.92	2.21	2.87	2.96	2.41	1.48	4.94	5.39	>0.999999999999999	0.9
P17858	6-phosphofructokinase, liver type OS=Homo sapiens GN=PFKL PE=1 SV=6	1.63	2.06	3.82	7.48	3.02	7.09	1.37	0.36	1.10	5.56	3.30	1.71	1.88	0.64	3.82	2.69	0.51	0.61	2.89	0.00	0.00	1.28	1.70	0.32	3.21	1.36	>0.999999999999999	2.4
P29144	Tripeptidyl-peptidase 2 OS=Homo sapiens GN=TPP2 PE=1 SV=4	1.02	1.20	1.67	0.43	1.32	0.47	1.20	0.18	0.00	0.93	0.79	0.16	0.00	0.32	1.19	0.00	0.00	0.00	0.00	0.00	0.00	0.16	0.28	0.08	0.78	0.17	>0.999999999999999	4.6
P14174	Macrophage migration inhibitory factor OS=Homo sapiens GN=MIF PE=1 SV=4	5.51	4.63	0.00	5.77	11.50	14.41	4.62	5.51	3.70	5.00	9.59	2.20	6.35	4.34	6.44	4.54	7.72	0.00	11.03	7.92	4.56	2.16	3.83	1.08	6.04	5.00	>0.999999999999999	1.2
P59827	BPI fold-containing family B member 4 OS=Homo sapiens GN=BPIFB4 PE=2 SV=2	2.04	2.74	3.82	0.00	3.02	2.36	3.76	2.53	3.01	0.93	0.79	0.82	0.00	3.53	0.00	0.00	1.27	0.77	0.90	0.65	0.84	1.76	3.12	0.20	2.15	1.09	>0.999999999999999	2.0
O60218	Aldo-keto reductase family 1 member B10 OS=Homo sapiens GN=AKR1B10 PE=1 SV=2	3.87	5.15	4.54	8.98	5.66	16.30	5.13	6.23	1.23	3.52	13.37	8.32	4.47	4.82	16.46	9.25	3.80	6.44	7.60	2.47	1.52	3.35	9.79	2.20	6.86	6.01	>0.999999999999999	1.1
Q14914	Prostaglandin reductase 1 OS=Homo sapiens GN=PTGR1 PE=1 SV=2	1.84	5.15	13.15	6.41	10.37	13.00	3.25	1.72	4.11	5.56	10.85	1.55	2.12	3.05	13.12	3.20	3.80	2.92	3.44	1.17	1.52	2.40	5.96	1.20	6.41	3.66	>0.999999999999999	1.8
P26583	High mobility group protein B2 OS=Homo sapiens GN=HMGB2 PE=1 SV=2	6.12	2.40	3.35	6.41	0.00	7.09	2.39	2.71	1.92	2.59	2.20	1.14	3.29	2.25	3.34	2.36	1.77	2.15	2.53	6.10	5.06	1.12	1.99	0.56	3.19	2.71	>0.999999999999999	1.2
O15144	Actin-related protein 2/3 complex subunit 2 OS=Homo sapiens GN=ARPC2 PE=1 SV=1	6.53	3.43	10.76	19.24	13.95	13.71	9.92	5.24	4.38	10.75	5.03	6.04	10.58	3.21	17.65	9.76	2.53	6.90	8.14	2.60	3.38	4.63	8.23	1.80	9.08	6.62	>0.999999999999999	1.4
P01700	Ig lambda chain V-I region HA OS=Homo sapiens PE=1 SV=1	5.91	0.00	6.93	6.20	12.44	6.85	0.00	2.62	3.97	0.00	4.56	0.00	6.82	4.66	6.92	11.10	0.00	0.00	11.94	0.00	4.89	2.32	4.12	2.64	4.12	4.62	>0.999999999999999	0.9
Q8WZA9	Immunity-related GTPase family Q protein OS=Homo sapiens GN=IRGQ PE=1 SV=1	2.24	0.86	1.20	2.35	2.07	0.00	1.88	0.00	0.68	0.00	0.00	1.31	1.18	0.80	0.00	1.85	0.00	0.77	0.90	0.00	0.00	0.88	1.56	0.44	1.05	0.70	>0.999999999999999	1.5
Q13155	Aminoacyl tRNA synthase complex-interacting multifunctional protein 2 OS=Homo sapiens GN=AIMP2 PE=1 SV=2	1.84	1.54	2.15	1.92	1.70	2.13	1.54	0.00	0.00	1.67	1.42	0.73	0.00	0.00	4.53	1.51	0.00	0.00	1.63	0.00	0.00	0.00	0.00	0.36	1.39	0.67	>0.999999999999999	2.1
P14866	Heterogeneous nuclear ribonucleoprotein L OS=Homo sapiens GN=HNRNPL PE=1 SV=2	3.26	1.72	3.82	5.99	6.60	6.62	3.76	0.45	0.68	5.19	6.45	0.41	0.00	0.80	2.38	1.68	0.00	0.77	1.81	0.00	0.00	1.28	1.42	0.20	3.75	0.86	>0.999999999999999	4.3
P21333	Filamin-A OS=Homo sapiens GN=FLNA PE=1 SV=4	0.20	0.00	0.00	0.00	0.00	0.00	0.00	0.00	0.00	0.00	0.00	0.00	0.00	0.00	0.00	0.00	0.00	0.00	0.00	0.00	0.17	0.00	0.00	0.00	0.02	0.01	>0.999999999999999	1.2
P48507	Glutamate--cysteine ligase regulatory subunit OS=Homo sapiens GN=GCLM PE=1 SV=1	2.24	3.95	8.61	7.70	4.34	5.43	6.16	4.52	1.51	2.04	7.86	2.94	2.59	5.78	8.59	1.85	2.91	1.69	4.16	0.00	0.00	2.88	11.92	0.44	4.77	3.57	>0.999999999999999	1.3
P02766	Transthyretin OS=Homo sapiens GN=TTR PE=1 SV=1	4.28	0.00	5.02	10.05	14.71	0.00	3.59	1.90	0.00	3.89	0.00	13.21	4.94	3.37	11.21	3.53	2.66	3.22	3.80	10.13	0.00	0.00	2.98	1.88	4.72	3.98	>0.999999999999999	1.2
P14868	Aspartate--tRNA ligase, cytoplasmic OS=Homo sapiens GN=DARS PE=1 SV=2	5.10	2.06	7.65	10.26	2.26	7.56	2.05	0.00	0.00	1.11	3.93	1.47	0.00	4.01	7.63	3.03	1.52	0.00	1.09	0.00	0.00	2.00	2.55	0.48	3.62	1.86	>0.999999999999999	1.9
O14791	Apolipoprotein L1 OS=Homo sapiens GN=APOL1 PE=1 SV=5	1.63	0.00	0.00	0.00	1.51	1.89	0.00	0.72	1.10	0.00	0.00	0.65	1.88	1.28	0.00	1.35	1.01	1.23	1.45	0.00	1.35	0.64	0.00	0.32	0.63	0.88	>0.999999999999999	0.7
P07951	Tropomyosin beta chain OS=Homo sapiens GN=TPM2 PE=1 SV=1	4.28	0.00	11.24	0.00	0.00	0.00	0.00	0.00	0.00	0.00	9.75	0.00	0.00	0.00	11.21	0.00	0.00	0.00	0.00	1.30	1.69	0.00	0.00	0.00	2.11	1.18	>0.999999999999999	1.8
Q92973	Transportin-1 OS=Homo sapiens GN=TNPO1 PE=1 SV=2	1.22	0.51	0.00	1.28	1.89	1.42	0.51	0.00	0.41	0.56	0.94	0.00	0.00	0.00	0.72	0.00	0.38	0.46	0.54	0.00	0.00	0.24	0.43	0.00	0.73	0.23	>0.999999999999999	3.2
P15559	NAD(P)H dehydrogenase [quinone] 1 OS=Homo sapiens GN=NQO1 PE=1 SV=1	2.24	0.00	16.02	14.32	16.03	12.05	8.72	7.68	1.51	0.00	13.37	2.94	2.59	0.00	20.27	14.30	8.48	7.82	9.22	1.43	0.00	0.00	12.06	0.92	7.91	6.42	>0.999999999999999	1.2
Q9BWD1	Acetyl-CoA acetyltransferase, cytosolic OS=Homo sapiens GN=ACAT2 PE=1 SV=2	3.26	4.46	8.61	5.56	6.79	10.87	2.74	1.44	3.56	8.52	9.12	0.00	0.00	1.28	6.20	1.35	3.29	2.45	1.45	0.00	0.00	3.67	6.53	1.04	5.41	2.27	>0.999999999999999	2.4
P04280	Basic salivary proline-rich protein 1 OS=Homo sapiens GN=PRB1 PE=1 SV=2	7.95	0.00	0.00	0.00	0.00	0.00	0.00	0.00	0.00	0.00	0.00	1.47	2.12	0.00	0.00	0.00	0.00	1.38	0.00	0.00	0.00	0.00	0.00	3.72	0.79	0.60	>0.999999999999999	1.3
Q6P5S2	UPF0762 protein C6orf58 OS=Homo sapiens GN=C6orf58 PE=1 SV=2	1.84	1.54	2.15	1.92	1.70	2.13	3.08	1.63	2.47	1.67	4.40	3.18	4.23	2.89	2.15	4.71	3.54	4.30	1.63	3.64	4.73	2.24	2.55	2.04	2.31	3.22	>0.999999999999999	0.7
Q16890	Tumor protein D53 OS=Homo sapiens GN=TPD52L1 PE=1 SV=1	6.53	2.57	3.59	6.84	6.03	7.56	2.56	1.35	0.00	0.00	2.36	2.61	3.53	0.00	3.58	0.00	0.00	2.30	2.71	0.00	0.00	2.56	2.13	0.60	3.50	1.45	>0.999999999999999	2.4
P08118	Beta-microseminoprotein OS=Homo sapiens GN=MSMB PE=1 SV=1	11.62	0.00	5.98	12.19	0.00	0.00	4.27	2.26	3.42	0.00	3.93	0.00	5.88	0.00	5.96	9.59	3.16	0.00	4.52	0.00	0.00	0.00	3.55	0.00	3.64	2.72	>0.999999999999999	1.3
P46782	40S ribosomal protein S5 OS=Homo sapiens GN=RPS5 PE=1 SV=4	6.32	2.57	7.41	10.69	13.58	11.82	12.31	2.80	4.25	9.26	7.86	1.22	11.76	4.98	11.92	5.22	6.33	2.30	5.61	0.00	2.53	2.48	7.10	0.60	7.51	5.07	>0.999999999999999	1.5
Q14247	Src substrate cortactin OS=Homo sapiens GN=CTTN PE=1 SV=2	2.24	1.89	8.84	6.41	3.21	10.40	2.91	2.71	0.68	4.26	4.72	1.88	0.00	1.77	5.49	3.87	2.15	2.61	1.99	0.00	0.00	2.40	4.26	1.48	4.18	2.17	>0.999999999999999	1.9
Q15691	Microtubule-associated protein RP/EB family member 1 OS=Homo sapiens GN=MAPRE1 PE=1 SV=3	2.24	3.95	5.50	4.92	2.07	5.43	1.88	0.00	1.51	4.26	3.62	0.90	0.00	1.77	5.49	3.87	0.00	1.69	1.99	1.43	0.00	2.96	3.26	0.92	3.02	1.95	>0.999999999999999	1.6
P49720	Proteasome subunit beta type-3 OS=Homo sapiens GN=PSMB3 PE=1 SV=2	6.32	2.40	7.41	6.63	9.43	7.33	8.55	2.80	4.25	2.59	15.10	5.79	7.29	8.03	7.39	8.41	1.77	2.15	9.04	1.82	0.00	3.99	7.10	2.00	6.55	4.92	>0.999999999999999	1.3
P84098	60S ribosomal protein L19 OS=Homo sapiens GN=RPL19 PE=1 SV=1	2.85	0.00	3.35	2.99	5.66	3.31	5.13	1.26	1.92	2.59	4.72	0.00	0.00	2.25	3.34	2.36	0.00	2.15	5.43	0.00	0.00	1.12	4.26	0.56	2.82	1.79	>0.999999999999999	1.6
Q13200	26S proteasome non-ATPase regulatory subunit 2 OS=Homo sapiens GN=PSMD2 PE=1 SV=3	1.43	1.72	2.39	6.20	3.21	6.85	3.59	0.63	0.41	5.37	3.30	2.04	1.65	1.12	5.01	2.36	1.27	1.07	2.53	0.00	0.51	1.12	0.99	0.56	3.09	1.52	>0.999999999999999	2.0
Q9H008	Phospholysine phosphohistidine inorganic pyrophosphate phosphatase OS=Homo sapiens GN=LHPP PE=1 SV=2	2.24	1.89	2.63	5.13	4.53	2.60	1.88	0.00	0.00	2.04	1.73	0.90	0.00	1.77	2.62	1.85	1.39	0.00	1.99	0.00	0.00	0.88	3.41	0.44	2.13	1.20	>0.999999999999999	1.8
P01610	Ig kappa chain V-I region WEA OS=Homo sapiens PE=1 SV=1	5.91	0.00	0.00	0.00	5.47	0.00	0.00	2.62	0.00	0.00	0.00	2.37	6.82	4.66	0.00	4.88	0.00	0.00	0.00	0.00	4.89	0.00	4.12	0.00	1.36	2.11	>0.999999999999999	0.6
Q15437	Protein transport protein Sec23B OS=Homo sapiens GN=SEC23B PE=1 SV=2	0.82	0.00	2.87	1.71	0.75	0.95	1.37	0.00	0.00	0.00	1.26	0.98	0.00	0.64	0.95	0.67	0.51	0.61	0.72	0.00	0.00	0.32	0.57	0.32	0.89	0.44	>0.999999999999999	2.0
P55327	Tumor protein D52 OS=Homo sapiens GN=TPD52 PE=1 SV=2	5.91	4.97	6.93	10.05	5.47	11.11	8.04	4.24	1.92	0.00	10.54	3.83	3.29	4.66	6.92	4.88	1.77	4.45	0.00	0.00	4.89	3.75	6.67	2.68	6.08	3.66	>0.999999999999999	1.7
P25788	Proteasome subunit alpha type-3 OS=Homo sapiens GN=PSMA3 PE=1 SV=2	5.10	6.69	2.87	2.57	4.71	13.00	6.67	4.97	3.42	2.22	6.13	2.04	2.82	1.93	5.96	2.02	3.16	1.84	7.05	3.25	2.03	3.12	5.53	1.00	5.03	3.31	>0.999999999999999	1.5
O60234	Glia maturation factor gamma OS=Homo sapiens GN=GMFG PE=1 SV=1	8.97	0.00	0.00	9.41	0.00	0.00	1.81	0.00	0.00	0.00	0.00	0.00	0.00	0.00	0.00	0.00	0.00	3.07	0.00	0.00	3.38	0.00	0.00	0.00	1.68	0.54	>0.999999999999999	3.1
P60983	Glia maturation factor beta OS=Homo sapiens GN=GMFB PE=1 SV=2	8.97	0.00	4.78	0.00	3.77	0.00	7.52	0.00	0.00	3.71	6.92	1.63	0.00	0.00	0.00	7.40	0.00	0.00	3.62	0.00	0.00	0.00	0.00	0.80	3.11	0.99	>0.999999999999999	3.2
Q9UHY7	Enolase-phosphatase E1 OS=Homo sapiens GN=ENOPH1 PE=1 SV=1	2.24	1.89	9.08	11.55	7.16	8.98	4.10	0.99	1.51	2.04	11.32	0.90	0.00	3.85	5.72	4.04	0.00	3.68	1.99	0.00	0.00	1.92	3.41	0.44	5.15	2.09	>0.999999999999999	2.5
O43242	26S proteasome non-ATPase regulatory subunit 3 OS=Homo sapiens GN=PSMD3 PE=1 SV=2	3.47	5.15	5.50	6.41	4.34	7.09	2.91	0.99	0.68	0.93	4.72	1.39	1.18	3.69	4.05	5.05	0.00	0.77	4.16	0.00	0.84	2.96	5.25	0.68	3.63	2.39	>0.999999999999999	1.5
P43652	Afamin OS=Homo sapiens GN=AFM PE=1 SV=1	1.02	0.86	4.78	4.28	6.98	4.73	5.30	1.81	2.74	2.78	1.42	2.53	1.18	1.45	3.58	2.52	3.16	2.30	2.71	3.25	5.23	1.60	0.71	1.00	3.27	2.39	>0.999999999999999	1.4
O75347	Tubulin-specific chaperone A OS=Homo sapiens GN=TBCA PE=1 SV=3	12.24	10.29	6.45	5.77	11.31	14.18	10.26	9.30	8.22	5.00	4.25	8.40	14.11	9.63	6.44	0.00	7.59	9.21	10.85	3.51	4.56	2.16	14.62	1.08	8.81	6.98	>0.999999999999999	1.3
Q96EK6	Glucosamine 6-phosphate N-acetyltransferase OS=Homo sapiens GN=GNPNAT1 PE=1 SV=1	3.26	2.74	3.82	3.42	6.41	3.78	9.58	1.44	4.66	2.96	5.35	2.77	3.76	5.46	8.11	2.69	2.02	5.22	10.13	0.00	0.00	1.28	4.82	1.36	4.18	3.74	>0.999999999999999	1.1
A0AVT1	Ubiquitin-like modifier-activating enzyme 6 OS=Homo sapiens GN=UBA6 PE=1 SV=1	0.61	1.03	3.35	1.71	2.07	1.42	1.37	0.00	0.41	1.48	1.73	0.90	0.71	0.48	1.91	0.50	1.01	0.92	1.99	0.00	0.00	0.64	1.14	0.24	1.34	0.79	>0.999999999999999	1.7
P25398	40S ribosomal protein S12 OS=Homo sapiens GN=RPS12 PE=1 SV=3	10.40	8.75	5.50	18.17	9.62	20.09	14.53	0.00	3.15	9.45	20.13	4.16	12.00	8.19	30.53	14.30	6.45	3.53	4.16	0.00	3.88	6.79	18.16	2.04	10.33	9.17	>0.999999999999999	1.1
O14602	Eukaryotic translation initiation factor 1A, Y-chromosomal OS=Homo sapiens GN=EIF1AY PE=1 SV=4	4.08	3.43	4.78	4.28	3.77	4.73	0.00	1.81	2.74	3.71	3.15	1.63	4.70	3.21	4.77	3.36	2.53	3.07	3.62	0.00	0.00	0.00	0.00	0.00	3.17	2.11	>0.999999999999999	1.5
Q9UNH7	Sorting nexin-6 OS=Homo sapiens GN=SNX6 PE=1 SV=1	3.06	2.57	5.50	3.21	2.83	11.82	3.93	1.35	0.00	2.78	3.62	1.22	3.53	1.12	1.67	2.52	0.00	2.30	0.00	0.00	0.00	0.56	5.68	0.60	3.49	1.50	>0.999999999999999	2.3
P01593	Ig kappa chain V-I region AG OS=Homo sapiens PE=1 SV=1	5.71	0.00	6.69	5.99	12.26	6.62	0.00	2.53	3.83	0.00	0.00	2.28	6.59	0.00	6.68	4.71	8.22	9.97	0.00	3.64	0.00	2.24	3.97	1.12	3.83	3.93	>0.999999999999999	1.0
O60749	Sorting nexin-2 OS=Homo sapiens GN=SNX2 PE=1 SV=2	3.67	2.06	2.87	3.85	1.13	7.33	2.05	0.00	0.00	2.22	4.88	0.00	0.00	0.00	1.43	0.00	1.52	0.00	3.26	0.00	0.00	1.44	5.53	0.48	2.50	1.14	>0.999999999999999	2.2
P43034	Platelet-activating factor acetylhydrolase IB subunit alpha OS=Homo sapiens GN=PAFAH1B1 PE=1 SV=2	2.85	3.77	9.56	6.63	4.15	7.33	3.76	0.00	1.92	4.08	6.29	1.14	1.65	1.12	5.25	2.36	1.77	2.15	2.53	0.91	1.18	2.48	3.12	0.88	4.29	2.12	>0.999999999999999	2.0
P35606	Coatomer subunit beta' OS=Homo sapiens GN=COPB2 PE=1 SV=2	0.61	1.03	5.02	5.99	2.45	9.69	3.59	1.17	1.37	3.15	4.40	2.28	2.35	0.48	4.05	2.86	1.27	0.00	2.35	0.00	0.00	1.04	2.98	0.52	3.40	1.49	>0.999999999999999	2.3
P20042	Eukaryotic translation initiation factor 2 subunit 2 OS=Homo sapiens GN=EIF2S2 PE=1 SV=2	3.67	4.80	4.30	3.85	7.35	4.25	3.08	0.81	2.47	1.67	4.40	1.47	2.12	1.45	0.00	3.03	3.54	1.38	1.63	0.00	3.04	1.44	3.97	0.36	3.51	1.83	>0.999999999999999	1.9
P29350	Tyrosine-protein phosphatase non-receptor type 6 OS=Homo sapiens GN=PTPN6 PE=1 SV=1	1.02	1.72	3.59	2.14	0.94	2.36	2.56	0.45	0.00	0.93	0.79	1.22	1.18	1.61	1.19	0.84	0.63	1.53	0.90	0.65	1.69	1.20	1.42	0.00	1.48	1.07	>0.999999999999999	1.4
P27695	DNA-(apurinic or apyrimidinic site) lyase OS=Homo sapiens GN=APEX1 PE=1 SV=2	1.84	3.26	7.17	8.98	1.70	4.49	3.25	2.71	2.60	3.52	8.65	0.73	4.47	1.45	2.15	0.00	1.14	2.92	5.43	2.47	5.06	1.52	1.28	1.68	4.07	2.46	>0.999999999999999	1.7
P09913	Interferon-induced protein with tetratricopeptide repeats 2 OS=Homo sapiens GN=IFIT2 PE=1 SV=1	2.45	0.00	0.00	0.00	2.26	0.00	4.45	0.00	0.00	0.00	0.00	1.55	4.47	0.00	0.00	0.00	0.00	0.00	2.17	0.00	0.00	0.00	0.00	0.00	0.89	0.55	>0.999999999999999	1.6
P41218	Myeloid cell nuclear differentiation antigen OS=Homo sapiens GN=MNDA PE=1 SV=1	3.06	1.20	0.00	3.21	0.00	0.00	0.00	2.89	0.00	0.00	0.00	0.00	1.65	1.12	0.00	0.00	0.89	2.30	2.71	1.95	5.40	0.00	0.00	0.00	0.86	1.33	>0.999999999999999	0.6
Q6EEV6	Small ubiquitin-related modifier 4 OS=Homo sapiens GN=SUMO4 PE=1 SV=2	6.53	5.49	7.65	6.84	6.03	7.56	0.00	2.89	4.38	0.00	0.00	0.00	7.53	0.00	7.63	5.38	4.05	4.91	5.79	0.00	5.40	2.56	4.54	0.00	3.95	3.98	>0.999999999999999	1.0
Q9Y6U3	Adseverin OS=Homo sapiens GN=SCIN PE=1 SV=4	3.47	3.77	6.45	6.84	6.98	8.74	2.91	0.72	3.01	0.00	4.25	1.39	0.00	2.73	4.05	0.00	0.00	2.61	3.98	1.04	1.35	2.16	3.83	0.88	4.04	1.89	>0.999999999999999	2.1
Q9UNZ2	NSFL1 cofactor p47 OS=Homo sapiens GN=NSFL1C PE=1 SV=2	1.63	4.63	4.06	3.63	3.21	1.89	1.37	1.54	0.00	6.85	2.67	2.20	0.00	1.28	6.44	2.86	1.01	1.23	1.45	1.04	0.00	1.36	2.41	0.68	2.81	1.65	>0.999999999999999	1.7
P36578	60S ribosomal protein L4 OS=Homo sapiens GN=RPL4 PE=1 SV=5	4.49	3.77	9.32	1.50	4.15	11.58	3.76	0.00	0.96	7.23	6.13	0.00	5.17	4.82	7.15	5.05	1.77	0.00	2.53	0.00	0.00	0.56	3.12	0.56	4.41	2.56	>0.999999999999999	1.7
P47929	Galectin-7 OS=Homo sapiens GN=LGALS7 PE=1 SV=2	4.69	3.95	0.00	4.92	0.00	11.82	0.00	2.08	0.00	0.00	0.00	6.85	5.41	3.69	0.00	8.41	15.82	7.67	0.00	2.99	3.88	1.84	0.00	5.00	2.86	4.56	>0.999999999999999	0.6
Q9Y2V2	Calcium-regulated heat stable protein 1 OS=Homo sapiens GN=CARHSP1 PE=1 SV=2	4.28	3.60	5.02	4.49	3.96	4.96	8.04	0.00	2.88	3.89	7.39	0.00	11.05	3.37	5.01	3.53	0.00	3.22	3.80	0.00	0.00	6.15	6.67	0.00	4.04	3.57	>0.999999999999999	1.1
O95969	Secretoglobin family 1D member 2 OS=Homo sapiens GN=SCGB1D2 PE=2 SV=1	6.93	13.55	18.88	16.89	14.90	8.03	13.51	12.64	10.82	14.64	12.42	6.44	8.00	22.48	8.11	13.29	10.00	5.22	14.29	18.18	23.63	6.31	11.21	3.16	12.47	11.99	>0.999999999999999	1.0
P07910	Heterogeneous nuclear ribonucleoproteins C1/C2 OS=Homo sapiens GN=HNRNPC PE=1 SV=4	4.28	3.60	7.65	6.84	8.67	17.96	5.47	2.89	2.88	5.93	9.44	4.89	4.94	1.61	5.01	5.38	1.27	7.06	8.32	1.30	1.69	2.56	6.53	0.84	6.71	3.87	>0.999999999999999	1.7
Q6UXB2	VEGF co-regulated chemokine 1 OS=Homo sapiens GN=CXCL17 PE=1 SV=1	4.89	0.00	0.00	0.00	4.53	0.00	4.10	2.17	3.29	0.00	0.00	0.00	5.64	3.85	0.00	0.00	3.04	3.68	0.00	3.12	9.11	1.92	3.41	0.96	1.58	2.89	>0.999999999999999	0.5
P49721	Proteasome subunit beta type-2 OS=Homo sapiens GN=PSMB2 PE=1 SV=1	6.32	12.35	7.41	20.74	13.58	17.01	5.30	4.52	0.00	9.26	7.86	1.22	7.29	4.98	17.17	5.22	6.33	4.76	5.61	1.95	2.53	7.75	7.10	1.24	8.80	5.99	>0.999999999999999	1.5
Q9NTK5	Obg-like ATPase 1 OS=Homo sapiens GN=OLA1 PE=1 SV=2	1.43	4.12	10.28	3.21	1.32	10.16	2.56	0.63	0.96	4.45	3.77	0.57	5.64	2.41	7.87	4.04	0.00	3.68	2.71	0.00	0.00	1.92	2.13	0.00	3.62	2.53	>0.999999999999999	1.4
P11908	Ribose-phosphate pyrophosphokinase 2 OS=Homo sapiens GN=PRPS2 PE=1 SV=2	1.84	1.54	7.41	15.39	8.11	10.16	7.35	0.00	0.00	5.74	4.88	3.51	0.00	4.98	7.39	5.22	1.14	0.00	5.61	0.00	0.00	5.75	6.10	0.80	5.49	3.08	>0.999999999999999	1.8
P38606	V-type proton ATPase catalytic subunit A OS=Homo sapiens GN=ATP6V1A PE=1 SV=2	2.04	3.60	5.02	2.14	2.83	3.54	1.71	0.45	0.68	2.78	2.36	2.12	1.18	0.80	5.01	3.53	0.63	0.77	0.90	0.65	0.00	1.68	2.98	0.60	2.44	1.56	>0.999999999999999	1.6
P51665	26S proteasome non-ATPase regulatory subunit 7 OS=Homo sapiens GN=PSMD7 PE=1 SV=2	3.87	4.97	4.54	6.20	5.47	4.49	4.96	0.81	0.00	5.37	2.99	3.34	2.12	3.05	6.92	4.88	2.40	0.00	5.25	0.00	0.00	1.52	5.82	0.76	3.92	2.73	>0.999999999999999	1.4
P46781	40S ribosomal protein S9 OS=Homo sapiens GN=RPS9 PE=1 SV=3	10.40	2.57	12.19	6.84	9.62	12.05	8.72	2.89	6.98	2.78	11.64	1.22	3.53	0.00	17.65	8.58	0.00	2.30	5.79	0.00	2.53	2.56	4.54	2.04	7.33	4.13	>0.999999999999999	1.8
P31153	S-adenosylmethionine synthase isoform type-2 OS=Homo sapiens GN=MAT2A PE=1 SV=1	4.89	5.83	1.91	3.42	1.51	5.67	4.10	1.44	0.00	2.96	3.77	1.96	1.88	2.57	3.82	0.00	1.01	1.23	2.89	0.00	1.35	1.28	2.27	1.36	3.12	1.64	>0.999999999999999	1.9
Q9NUU7	ATP-dependent RNA helicase DDX19A OS=Homo sapiens GN=DDX19A PE=1 SV=1	1.22	2.06	8.13	1.28	3.58	4.49	1.03	0.00	0.82	1.11	4.25	1.55	0.00	0.96	1.43	0.00	0.76	0.92	1.09	0.00	0.00	0.96	1.70	0.48	2.46	0.69	>0.999999999999999	3.6
Q5JXB2	Putative ubiquitin-conjugating enzyme E2 N-like OS=Homo sapiens GN=UBE2NL PE=5 SV=1	8.77	0.00	0.00	0.00	8.11	0.00	0.00	3.88	5.89	0.00	0.00	0.00	10.11	0.00	0.00	0.00	0.00	0.00	7.78	2.60	3.38	0.00	0.00	0.00	2.22	1.99	>0.999999999999999	1.1
Q9Y6D6	Brefeldin A-inhibited guanine nucleotide-exchange protein 1 OS=Homo sapiens GN=ARFGEF1 PE=1 SV=2	0.41	0.00	1.91	0.64	1.13	1.18	0.51	0.00	0.00	0.00	1.57	0.49	0.00	0.32	0.72	0.00	0.00	0.00	0.00	0.00	0.00	0.16	1.14	0.12	0.65	0.20	>0.999999999999999	3.2
O15305	Phosphomannomutase 2 OS=Homo sapiens GN=PMM2 PE=1 SV=1	5.10	4.29	9.32	11.97	4.71	0.00	4.27	2.26	1.64	4.63	6.13	2.04	0.00	1.93	13.36	0.00	0.00	0.00	0.00	0.00	2.03	4.47	5.53	0.00	4.70	2.28	>0.999999999999999	2.1
P62750	60S ribosomal protein L23a OS=Homo sapiens GN=RPL23A PE=1 SV=1	3.87	3.26	4.54	4.06	7.92	9.92	3.25	0.00	0.00	0.00	10.85	1.55	9.88	0.00	4.53	3.20	0.00	0.00	3.44	0.00	0.00	3.35	2.70	0.00	4.10	2.26	>0.999999999999999	1.8
Q15714	TSC22 domain family protein 1 OS=Homo sapiens GN=TSC22D1 PE=1 SV=3	0.61	0.51	0.72	0.64	0.00	0.71	0.51	0.00	0.00	0.56	0.47	0.00	0.00	0.48	0.72	0.50	0.00	0.00	0.00	0.00	0.00	0.00	0.43	0.00	0.39	0.18	>0.999999999999999	2.2
P01719	Ig lambda chain V-V region DEL OS=Homo sapiens PE=1 SV=1	14.07	0.00	0.00	0.00	5.66	0.00	0.00	0.00	0.00	0.00	0.00	0.00	0.00	4.82	0.00	5.05	0.00	0.00	0.00	0.00	0.00	2.40	0.00	0.00	1.64	1.02	>0.999999999999999	1.6
P22061	Protein-L-isoaspartate(D-aspartate) O-methyltransferase OS=Homo sapiens GN=PCMT1 PE=1 SV=4	5.91	2.40	6.93	18.81	2.64	3.31	2.39	1.26	1.92	2.59	7.23	1.14	3.29	2.25	10.97	4.88	0.00	0.00	2.53	0.00	0.00	1.12	9.37	1.84	4.71	3.02	>0.999999999999999	1.6
Q14677	Clathrin interactor 1 OS=Homo sapiens GN=CLINT1 PE=1 SV=1	3.06	0.86	2.39	5.56	0.94	3.54	0.00	0.45	0.68	0.93	3.30	0.82	0.00	0.80	3.58	1.68	0.63	0.77	0.90	0.00	0.84	0.40	1.42	0.40	1.88	0.95	>0.999999999999999	2.0
Q99460	26S proteasome non-ATPase regulatory subunit 1 OS=Homo sapiens GN=PSMD1 PE=1 SV=2	1.22	1.54	4.78	4.28	5.09	7.33	2.74	0.54	0.82	2.96	6.13	2.20	0.00	1.45	4.77	2.19	0.38	1.38	3.62	0.00	0.00	1.60	3.41	1.08	3.30	1.66	>0.999999999999999	2.0
P43686	26S protease regulatory subunit 6B OS=Homo sapiens GN=PSMC4 PE=1 SV=2	1.43	1.20	5.26	6.63	1.32	5.20	1.20	0.63	0.00	1.30	1.10	2.53	0.00	0.00	3.34	2.36	0.89	2.15	0.00	0.00	0.00	2.48	3.12	1.24	2.32	1.30	>0.999999999999999	1.8
Q9HC35	Echinoderm microtubule-associated protein-like 4 OS=Homo sapiens GN=EML4 PE=1 SV=3	1.22	1.03	2.87	3.42	1.70	3.78	1.03	0.54	0.00	1.11	2.52	0.73	1.41	0.96	0.72	0.50	0.38	0.92	2.17	0.00	0.00	0.24	0.43	0.36	1.66	0.67	>0.999999999999999	2.5
O95379	Tumor necrosis factor alpha-induced protein 8 OS=Homo sapiens GN=TNFAIP8 PE=2 SV=1	2.85	2.40	3.35	2.99	2.64	3.31	2.39	0.00	0.00	0.00	0.00	1.14	0.00	0.00	0.00	0.00	0.00	0.00	0.00	0.00	0.00	0.00	0.00	0.00	1.76	0.00	>0.999999999999999	#DIV/0!
P26639	Threonine--tRNA ligase, cytoplasmic OS=Homo sapiens GN=TARS PE=1 SV=3	2.45	2.74	6.22	4.49	3.96	4.96	2.05	0.36	0.00	2.22	1.89	2.12	1.88	1.28	3.82	1.35	0.51	0.61	0.00	0.00	0.00	2.08	2.98	0.16	2.79	1.22	>0.999999999999999	2.3
Q9Y5P6	Mannose-1-phosphate guanyltransferase beta OS=Homo sapiens GN=GMPPB PE=1 SV=2	3.47	2.92	8.84	5.77	5.09	6.38	8.21	0.00	0.00	5.00	5.82	3.02	0.00	4.34	14.31	4.54	1.01	1.23	1.45	0.00	0.00	2.96	3.83	1.08	4.54	2.90	>0.999999999999999	1.6
Q96A49	Synapse-associated protein 1 OS=Homo sapiens GN=SYAP1 PE=1 SV=1	1.63	6.35	4.06	0.00	0.00	4.02	4.62	0.00	0.00	3.15	2.67	1.39	0.00	1.28	1.91	1.35	0.00	1.23	0.00	0.00	0.00	0.00	1.14	0.32	2.32	0.60	>0.999999999999999	3.9
O60506	Heterogeneous nuclear ribonucleoprotein Q OS=Homo sapiens GN=SYNCRIP PE=1 SV=2	2.04	2.57	2.39	1.07	3.77	4.73	1.71	0.00	0.00	3.71	2.36	1.63	1.18	0.00	1.19	0.84	0.00	0.00	2.71	0.00	0.84	0.40	2.13	0.60	2.16	0.82	>0.999999999999999	2.6
P62857	40S ribosomal protein S28 OS=Homo sapiens GN=RPS28 PE=1 SV=1	9.18	0.00	0.00	0.00	8.48	10.63	0.00	9.84	6.16	0.00	7.08	3.67	10.58	0.00	0.00	0.00	13.79	6.90	8.14	0.00	7.60	3.59	0.00	1.80	4.59	4.37	>0.999999999999999	1.1
Q9BR76	Coronin-1B OS=Homo sapiens GN=CORO1B PE=1 SV=1	2.45	1.03	4.54	2.57	2.26	6.14	3.25	0.54	0.82	3.52	0.94	2.12	2.82	0.96	2.86	2.02	0.76	0.92	3.44	0.78	1.01	2.08	1.70	0.48	2.52	1.65	>0.999999999999999	1.5
P61201	COP9 signalosome complex subunit 2 OS=Homo sapiens GN=COPS2 PE=1 SV=1	2.65	2.23	6.69	4.28	2.45	4.73	3.42	0.54	0.00	3.71	4.40	0.49	1.41	3.21	3.10	2.19	0.76	0.92	0.00	0.00	0.00	0.48	1.84	0.24	2.97	1.18	>0.999999999999999	2.5
P23526	Adenosylhomocysteinase OS=Homo sapiens GN=AHCY PE=1 SV=4	4.49	5.15	11.71	12.61	11.12	11.58	8.38	2.71	3.01	2.59	9.28	3.18	3.29	2.25	14.07	8.24	2.78	4.60	7.05	0.91	5.06	5.59	8.37	0.88	7.15	5.26	>0.999999999999999	1.4
Q13325	Interferon-induced protein with tetratricopeptide repeats 5 OS=Homo sapiens GN=IFIT5 PE=1 SV=1	1.22	0.00	0.00	1.28	2.26	0.00	1.03	0.00	0.00	0.00	0.00	0.49	0.00	0.00	0.00	0.00	0.00	0.00	1.09	0.00	0.00	0.00	0.00	0.00	0.52	0.09	>0.999999999999999	5.8
P35221	Catenin alpha-1 OS=Homo sapiens GN=CTNNA1 PE=1 SV=1	0.61	2.92	4.06	8.12	4.71	8.98	2.91	0.00	0.41	3.15	3.30	1.71	0.00	1.12	2.38	4.21	0.38	0.00	2.53	0.00	0.51	0.00	2.98	1.16	3.41	1.27	>0.999999999999999	2.7
Q92597	Protein NDRG1 OS=Homo sapiens GN=NDRG1 PE=1 SV=1	1.63	1.37	0.00	0.00	0.00	1.89	1.37	0.00	0.00	0.00	0.00	0.65	0.00	0.00	0.00	0.00	0.00	0.00	0.00	0.00	0.00	0.64	0.00	0.32	0.58	0.08	>0.999999999999999	7.2
P01034	Cystatin-C OS=Homo sapiens GN=CST3 PE=1 SV=1	9.58	8.06	5.02	4.49	8.86	4.96	3.59	7.04	6.44	8.71	7.39	13.21	11.05	7.55	5.01	3.53	5.95	17.80	3.80	2.73	19.58	6.23	11.07	1.88	7.28	8.01	>0.999999999999999	0.9
P01598	Ig kappa chain V-I region EU OS=Homo sapiens PE=1 SV=1	5.91	0.00	0.00	6.20	5.47	0.00	0.00	0.00	0.00	0.00	4.56	2.37	6.82	0.00	6.92	4.88	3.67	0.00	5.25	0.00	4.89	2.32	4.12	1.16	2.04	3.33	>0.999999999999999	0.6
P49589	Cysteine--tRNA ligase, cytoplasmic OS=Homo sapiens GN=CARS PE=1 SV=3	0.82	1.37	0.00	0.00	1.51	1.89	0.68	0.36	0.00	0.00	1.26	0.33	0.94	0.00	0.95	0.00	0.00	0.00	0.00	0.00	0.00	0.00	0.00	0.00	0.68	0.16	>0.999999999999999	4.3
P40763	Signal transducer and activator of transcription 3 OS=Homo sapiens GN=STAT3 PE=1 SV=2	1.63	0.69	5.74	2.35	3.02	2.60	4.10	0.00	0.00	0.74	2.52	0.90	0.00	0.64	0.95	0.67	0.00	0.00	0.72	0.00	0.00	0.88	2.84	0.64	2.02	0.61	>0.999999999999999	3.3
Q9UHV9	Prefoldin subunit 2 OS=Homo sapiens GN=PFDN2 PE=1 SV=1	4.08	3.43	10.76	9.62	8.48	10.63	7.69	1.81	2.74	8.34	0.00	1.63	4.70	3.21	4.77	7.57	2.53	3.07	3.62	0.00	3.38	3.59	6.39	1.80	5.77	3.72	>0.999999999999999	1.6
Q13126	S-methyl-5'-thioadenosine phosphorylase OS=Homo sapiens GN=MTAP PE=1 SV=2	4.49	1.72	5.26	4.70	1.89	8.27	0.00	0.00	1.37	4.08	1.57	1.79	2.35	0.00	5.25	1.68	0.00	0.00	0.00	0.00	0.00	1.76	0.00	0.88	2.93	0.99	>0.999999999999999	2.9
Q8WVM8	Sec1 family domain-containing protein 1 OS=Homo sapiens GN=SCFD1 PE=1 SV=4	1.02	0.86	2.15	1.92	0.00	2.13	0.85	0.00	0.00	1.67	0.79	0.41	0.00	0.00	1.19	0.84	0.00	0.00	0.90	0.00	0.00	0.00	0.00	0.20	0.98	0.26	>0.999999999999999	3.8
P02545	Prelamin-A/C OS=Homo sapiens GN=LMNA PE=1 SV=1	0.82	1.54	2.15	2.99	1.70	5.67	0.68	0.81	0.00	2.59	0.63	1.96	2.12	1.45	4.53	7.91	1.14	4.60	3.44	0.52	0.00	0.72	2.70	0.96	1.80	2.51	>0.999999999999999	0.7
P54578	Ubiquitin carboxyl-terminal hydrolase 14 OS=Homo sapiens GN=USP14 PE=1 SV=3	2.45	4.29	2.87	4.06	1.13	7.80	3.25	0.54	0.82	2.22	3.93	0.98	0.00	0.96	2.86	2.02	0.76	1.84	3.44	0.00	1.01	0.96	2.70	0.24	2.86	1.40	>0.999999999999999	2.0
Q01813	6-phosphofructokinase type C OS=Homo sapiens GN=PFKP PE=1 SV=2	2.45	2.06	1.91	3.42	1.51	2.84	2.05	0.00	1.10	3.71	3.15	2.04	0.00	0.00	1.91	1.35	0.00	0.61	2.89	0.00	0.00	1.28	2.84	0.32	2.19	0.93	>0.999999999999999	2.3
P05546	Heparin cofactor 2 OS=Homo sapiens GN=SERPIND1 PE=1 SV=3	3.67	1.03	1.43	3.85	6.03	1.42	4.27	1.63	5.48	4.63	2.83	2.04	4.23	4.01	4.29	5.38	5.06	4.91	4.52	4.15	6.75	3.20	0.85	0.48	3.19	3.99	>0.999999999999999	0.8
P53396	ATP-citrate synthase OS=Homo sapiens GN=ACLY PE=1 SV=3	0.61	0.51	1.91	1.71	2.64	1.89	1.37	0.00	0.00	1.48	0.79	0.24	0.00	0.48	1.19	0.84	0.38	0.46	0.90	0.00	0.00	0.88	0.71	0.00	1.10	0.49	>0.999999999999999	2.3
P43487	Ran-specific GTPase-activating protein OS=Homo sapiens GN=RANBP1 PE=1 SV=1	2.85	2.40	7.41	15.18	5.85	3.31	5.30	2.80	0.00	5.74	7.71	2.53	3.29	4.98	3.34	5.22	1.77	7.52	2.53	1.82	2.36	3.91	6.95	1.24	5.09	3.74	>0.999999999999999	1.4
P51884	Lumican OS=Homo sapiens GN=LUM PE=1 SV=2	3.67	0.00	9.32	5.99	5.28	6.62	3.08	1.63	3.83	3.33	4.40	5.22	2.12	2.89	12.16	6.56	3.54	2.76	1.63	1.17	0.00	0.00	2.55	1.56	4.36	3.08	>0.999999999999999	1.4
P22234	Multifunctional protein ADE2 OS=Homo sapiens GN=PAICS PE=1 SV=3	1.43	3.77	5.26	6.63	1.32	1.65	2.39	0.63	1.92	1.30	4.88	1.14	0.00	2.25	5.25	1.18	0.00	0.00	0.00	0.00	0.00	1.76	1.99	0.28	2.69	1.06	>0.999999999999999	2.5
Q16186	Proteasomal ubiquitin receptor ADRM1 OS=Homo sapiens GN=ADRM1 PE=1 SV=2	1.63	1.37	1.91	1.71	1.51	3.78	0.00	0.00	0.00	1.48	1.26	0.65	0.00	1.28	3.82	1.35	0.00	0.00	0.00	1.04	0.00	0.64	1.14	0.64	1.28	0.82	>0.999999999999999	1.5
P63172	Dynein light chain Tctex-type 1 OS=Homo sapiens GN=DYNLT1 PE=1 SV=1	5.51	4.63	6.45	5.77	11.50	6.38	4.62	2.44	0.00	5.00	9.59	2.20	0.00	4.34	6.44	4.54	3.42	0.00	4.88	0.00	0.00	2.16	3.83	2.44	5.34	2.67	>0.999999999999999	2.0
O94903	Proline synthase co-transcribed bacterial homolog protein OS=Homo sapiens GN=PROSC PE=1 SV=1	2.24	0.00	2.63	7.70	0.00	2.60	1.88	0.00	0.00	2.04	3.62	0.90	2.59	1.77	0.00	0.00	0.00	1.69	1.99	0.00	0.00	0.88	1.56	0.44	1.97	0.91	>0.999999999999999	2.2
Q8TF05	Serine/threonine-protein phosphatase 4 regulatory subunit 1 OS=Homo sapiens GN=PPP4R1 PE=1 SV=1	0.61	0.51	0.72	0.64	0.57	0.71	0.00	0.00	0.00	0.56	0.47	0.00	0.00	0.00	0.72	0.00	0.00	0.00	0.00	0.00	0.00	0.48	0.00	0.12	0.40	0.11	>0.999999999999999	3.6
O00231	26S proteasome non-ATPase regulatory subunit 11 OS=Homo sapiens GN=PSMD11 PE=1 SV=3	2.85	1.20	3.35	8.55	5.85	7.33	5.30	1.99	0.00	4.08	6.29	3.26	1.65	0.00	9.54	3.70	0.89	1.07	8.86	0.00	0.00	5.67	4.40	0.56	4.17	3.03	>0.999999999999999	1.4
P08185	Corticosteroid-binding globulin OS=Homo sapiens GN=SERPINA6 PE=1 SV=1	3.06	0.00	0.00	3.21	4.53	1.65	7.18	1.35	5.75	1.30	0.00	1.22	5.64	1.12	7.87	5.55	4.18	5.06	7.60	4.28	4.05	1.20	0.99	0.60	2.44	4.01	>0.999999999999999	0.6
Q92499	ATP-dependent RNA helicase DDX1 OS=Homo sapiens GN=DDX1 PE=1 SV=2	2.45	1.37	6.22	6.63	4.90	6.14	2.05	0.00	0.55	3.15	3.30	1.39	1.88	0.64	5.01	2.02	0.51	0.00	0.00	0.00	0.00	0.00	1.14	0.16	3.18	0.95	>0.999999999999999	3.4

P04003	C4b-binding protein alpha chain OS=Homo sapiens GN=C4BPA PE=1 SV=2	2.04	0.00	0.00	0.00	0.94	0.00	0.85	0.00	0.00	0.00	0.00	0.82	2.35	0.00	1.19	2.52	1.90	0.00	0.90	0.65	0.00	0.00	0.00	0.20	0.39	0.81	>0.999999999999999	0.5
P53618	Coatomer subunit beta OS=Homo sapiens GN=COPB1 PE=1 SV=3	1.84	1.54	4.78	5.77	3.02	3.07	3.93	0.54	1.23	3.71	2.52	1.31	0.00	0.48	3.82	1.51	1.14	0.46	1.63	0.00	0.00	1.60	1.28	0.52	2.77	1.04	>0.999999999999999	2.7
Q14376	UDP-glucose 4-epimerase OS=Homo sapiens GN=GALE PE=1 SV=2	3.67	1.54	0.00	5.99	1.70	0.00	3.08	0.81	1.23	0.00	1.42	1.47	0.00	1.45	2.15	1.51	3.54	2.76	3.26	0.00	3.04	1.44	5.53	1.12	1.74	2.15	>0.999999999999999	0.8
Q9Y285	Phenylalanine--tRNA ligase alpha subunit OS=Homo sapiens GN=FARSA PE=1 SV=3	1.22	0.00	2.87	2.57	1.13	1.42	1.03	0.54	0.00	2.22	0.94	0.49	0.00	0.96	1.43	1.01	0.00	0.00	1.09	0.00	0.00	0.48	0.85	0.24	1.20	0.50	>0.999999999999999	2.4
Q15056	Eukaryotic translation initiation factor 4H OS=Homo sapiens GN=EIF4H PE=1 SV=5	2.45	2.06	6.22	5.56	4.90	6.14	4.45	1.08	1.64	4.82	1.89	2.12	2.82	1.93	6.20	2.02	0.00	0.00	4.70	0.00	4.39	2.08	1.70	1.04	3.61	2.24	>0.999999999999999	1.6
P02760	Protein AMBP OS=Homo sapiens GN=AMBP PE=1 SV=1	3.47	0.00	4.06	5.77	1.51	0.00	1.37	1.54	2.33	3.15	1.26	3.10	4.00	1.28	4.05	6.39	2.15	2.61	1.45	6.36	1.35	1.36	1.14	1.52	2.30	2.81	>0.999999999999999	0.8
P20339	Ras-related protein Rab-5A OS=Homo sapiens GN=RAB5A PE=1 SV=2	6.12	0.00	0.00	0.00	0.00	7.09	0.00	0.00	1.92	0.00	0.00	0.00	3.29	0.00	7.15	0.00	0.00	0.00	0.00	0.00	0.00	0.00	1.99	0.00	1.26	1.04	>0.999999999999999	1.2
P61160	Actin-related protein 2 OS=Homo sapiens GN=ACTR2 PE=1 SV=1	3.06	2.57	1.67	5.13	2.83	5.67	4.10	1.35	0.96	2.78	5.19	1.22	5.64	1.12	1.67	0.00	0.00	2.30	2.71	0.91	4.05	2.64	2.13	0.28	3.05	1.95	>0.999999999999999	1.6
Q9UM54	Unconventional myosin-VI OS=Homo sapiens GN=MYO6 PE=1 SV=4	0.82	0.69	2.63	0.86	1.70	2.13	0.68	0.36	0.00	2.04	3.30	0.73	0.00	0.32	3.82	1.18	0.89	0.61	0.36	0.00	0.00	0.32	2.98	0.16	1.33	0.89	>0.999999999999999	1.5
O00303	Eukaryotic translation initiation factor 3 subunit F OS=Homo sapiens GN=EIF3F PE=1 SV=1	1.84	0.00	0.00	3.85	3.39	4.25	1.54	0.00	0.00	0.00	2.83	0.00	0.00	0.00	0.00	0.00	0.00	0.00	0.00	0.00	0.00	0.00	1.28	0.00	1.48	0.11	>0.999999999999999	13.9
O75594	Peptidoglycan recognition protein 1 OS=Homo sapiens GN=PGLYRP1 PE=1 SV=1	3.06	0.00	0.00	0.00	0.00	0.00	0.00	2.98	0.00	0.00	0.00	0.00	0.00	2.41	0.00	0.00	0.00	0.00	0.00	6.88	8.95	0.00	0.00	0.00	0.50	1.52	>0.999999999999999	0.3
O43237	Cytoplasmic dynein 1 light intermediate chain 2 OS=Homo sapiens GN=DYNC1LI2 PE=1 SV=1	2.45	2.06	8.13	4.06	3.58	2.84	0.00	0.00	0.00	2.22	1.89	0.98	1.41	1.93	4.53	1.01	0.00	0.00	0.00	0.00	0.00	0.96	2.70	0.24	2.35	1.06	>0.999999999999999	2.2
Q00688	Peptidyl-prolyl cis-trans isomerase FKBP3 OS=Homo sapiens GN=FKBP3 PE=1 SV=1	2.65	2.23	6.69	5.99	2.45	3.07	2.22	0.00	1.78	2.41	2.04	0.00	0.00	2.09	3.10	4.71	1.64	0.00	2.35	0.00	0.00	1.04	1.84	0.00	2.63	1.40	>0.999999999999999	1.9
P01824	Ig heavy chain V-II region WAH OS=Homo sapiens PE=1 SV=1	4.89	0.00	0.00	5.13	4.53	0.00	0.00	2.17	0.00	0.00	3.77	0.00	5.64	0.00	0.00	4.04	3.04	3.68	9.77	3.12	9.11	1.92	3.41	0.00	1.71	3.64	>0.999999999999999	0.5
Q96IJ6	Mannose-1-phosphate guanyltransferase alpha OS=Homo sapiens GN=GMPPA PE=1 SV=1	1.43	1.20	5.50	4.92	2.83	1.65	1.20	0.00	0.00	0.00	3.62	0.57	0.00	0.00	3.58	0.00	0.00	0.00	0.00	0.00	0.00	1.84	0.99	0.00	1.91	0.53	>0.999999999999999	3.6
Q00169	Phosphatidylinositol transfer protein alpha isoform OS=Homo sapiens GN=PITPNA PE=1 SV=2	2.04	0.00	8.37	4.92	1.89	0.00	0.00	0.00	0.00	0.00	0.00	0.00	0.00	0.00	0.00	0.00	0.00	1.53	0.00	0.00	0.00	0.00	3.12	0.00	1.43	0.39	>0.999999999999999	3.7
P55957	BH3-interacting domain death agonist OS=Homo sapiens GN=BID PE=1 SV=1	3.06	2.57	3.59	3.21	0.00	3.54	2.56	1.35	2.05	2.78	2.36	1.22	0.00	2.41	3.58	2.52	0.00	2.30	2.71	0.00	0.00	0.00	2.13	0.00	2.36	1.30	>0.999999999999999	1.8
P49591	Serine--tRNA ligase, cytoplasmic OS=Homo sapiens GN=SARS PE=1 SV=3	2.24	5.32	7.41	5.13	3.39	4.25	3.08	0.00	1.51	3.33	4.88	0.90	0.00	4.98	1.43	3.03	1.39	0.00	1.99	0.00	1.01	3.04	4.40	0.44	3.45	1.81	>0.999999999999999	1.9
O76003	Glutaredoxin-3 OS=Homo sapiens GN=GLRX3 PE=1 SV=2	1.84	1.54	0.00	1.92	7.54	2.13	3.08	0.81	2.47	1.67	4.56	1.47	0.00	2.89	0.00	0.00	2.28	2.76	1.63	0.00	0.00	2.32	4.12	0.36	2.42	1.36	>0.999999999999999	1.8
O00625	Pirin OS=Homo sapiens GN=PIR PE=1 SV=1	4.49	5.83	5.26	7.27	6.41	8.03	5.81	0.90	1.37	4.08	5.35	0.82	0.00	1.61	8.11	3.70	2.78	3.38	0.00	0.00	0.00	1.76	3.12	0.40	4.63	2.07	>0.999999999999999	2.2
Q9UN36	Protein NDRG2 OS=Homo sapiens GN=NDRG2 PE=1 SV=2	1.63	2.92	4.06	5.56	3.21	4.02	2.91	0.00	1.10	1.48	4.09	0.65	0.00	0.00	1.91	0.00	2.15	1.23	0.00	0.00	0.00	0.64	2.41	0.68	2.63	0.75	>0.999999999999999	3.5
Q8WVY7	Ubiquitin-like domain-containing CTD phosphatase 1 OS=Homo sapiens GN=UBLCP1 PE=1 SV=2	3.87	3.26	2.15	1.92	0.00	4.49	1.54	0.00	0.00	0.00	1.42	0.73	2.12	0.00	2.15	1.51	1.14	0.00	0.00	0.00	0.00	0.72	1.28	0.36	1.62	0.77	>0.999999999999999	2.1
Q92743	Serine protease HTRA1 OS=Homo sapiens GN=HTRA1 PE=1 SV=1	2.65	0.00	0.00	0.00	0.00	0.00	0.00	0.00	0.82	0.00	0.00	1.63	0.00	0.00	0.00	0.00	0.76	0.00	1.09	0.00	1.01	0.48	0.00	0.24	0.43	0.30	>0.999999999999999	1.4
P08134	Rho-related GTP-binding protein RhoC OS=Homo sapiens GN=RHOC PE=1 SV=1	10.60	2.57	12.43	28.22	14.14	12.29	5.47	0.00	4.38	0.00	8.18	6.12	7.53	5.14	12.40	8.75	1.90	15.50	13.57	1.95	2.53	4.15	10.64	0.00	8.70	7.00	>0.999999999999999	1.2
P80511	Protein S100-A12 OS=Homo sapiens GN=S100A12 PE=1 SV=2	6.73	5.66	0.00	16.25	6.22	7.80	5.64	12.10	4.52	6.11	5.19	2.69	7.76	5.30	18.13	5.55	16.96	5.06	5.97	17.40	5.57	2.64	4.68	0.00	6.58	7.92	>0.999999999999999	0.8
Q9UHD8	Septin-9 OS=Homo sapiens GN=SEPT9 PE=1 SV=2	2.04	0.86	3.82	2.14	3.02	6.62	2.74	0.45	0.00	5.19	4.40	0.00	1.18	1.61	3.82	0.84	0.63	0.77	2.89	0.00	0.84	0.80	2.27	0.00	2.61	1.30	>0.999999999999999	2.0
P62280	40S ribosomal protein S11 OS=Homo sapiens GN=RPS11 PE=1 SV=3	8.16	0.00	9.56	8.55	12.26	15.36	6.84	0.00	2.47	12.04	10.22	3.26	15.29	6.42	4.29	3.03	5.06	0.00	11.76	0.00	3.04	0.00	2.55	0.72	7.39	4.35	>0.999999999999999	1.7
O75821	Eukaryotic translation initiation factor 3 subunit G OS=Homo sapiens GN=EIF3G PE=1 SV=2	3.87	1.54	2.15	1.92	5.66	2.13	1.54	0.00	0.00	1.67	1.42	0.00	0.00	0.00	2.15	0.00	0.00	1.38	0.00	1.17	1.52	0.72	0.00	0.00	1.82	0.58	>0.999999999999999	3.2
Q5TDH0	Protein DDI1 homolog 2 OS=Homo sapiens GN=DDI2 PE=1 SV=1	1.43	1.20	3.59	1.50	1.32	0.00	0.00	0.63	0.00	2.78	3.77	0.00	0.00	1.12	1.67	0.00	0.00	0.00	0.00	0.00	0.00	1.20	0.00	0.00	1.35	0.33	>0.999999999999999	4.1
P28065	Proteasome subunit beta type-9 OS=Homo sapiens GN=PSMB9 PE=1 SV=2	2.85	0.00	7.41	6.63	5.85	3.31	2.39	1.26	1.92	2.59	2.20	2.53	0.00	2.25	3.34	2.36	0.00	2.15	2.53	0.00	0.00	1.12	1.99	0.00	3.25	1.31	>0.999999999999999	2.5
O43399	Tumor protein D54 OS=Homo sapiens GN=TPD52L2 PE=1 SV=2	6.53	2.57	7.65	6.84	14.14	17.72	2.56	2.89	0.00	5.93	8.18	6.12	7.53	0.00	12.40	5.38	1.90	7.98	13.57	1.95	5.40	2.56	4.54	2.08	6.76	5.44	>0.999999999999999	1.2
Q03154	Aminoacylase-1 OS=Homo sapiens GN=ACY1 PE=1 SV=1	1.43	1.20	5.50	8.77	1.32	1.65	0.00	0.00	0.00	1.30	1.10	0.00	1.65	1.12	5.49	1.18	0.89	1.07	0.00	0.00	0.00	1.20	2.13	0.00	1.86	1.23	>0.999999999999999	1.5
Q9BZZ5	Apoptosis inhibitor 5 OS=Homo sapiens GN=API5 PE=1 SV=3	1.22	1.03	1.43	2.35	1.13	2.60	1.03	0.00	0.00	3.33	0.94	0.90	1.41	0.00	1.43	0.00	0.00	0.00	0.00	0.00	0.00	0.00	0.00	0.24	1.33	0.26	>0.999999999999999	5.2
Q14166	Tubulin--tyrosine ligase-like protein 12 OS=Homo sapiens GN=TTLL12 PE=1 SV=2	2.85	0.00	0.96	1.92	0.75	0.95	0.00	0.00	0.00	0.00	0.00	0.33	0.00	0.00	0.95	0.67	0.00	0.61	0.72	0.00	0.00	0.00	1.99	0.36	0.65	0.44	>0.999999999999999	1.5
P49788	Retinoic acid receptor responder protein 1 OS=Homo sapiens GN=RARRES1 PE=2 SV=2	2.04	0.00	0.00	2.14	1.89	0.00	3.59	0.90	0.00	0.00	1.57	1.71	2.35	3.37	0.00	1.68	1.27	1.53	3.80	0.00	1.69	1.68	2.98	0.00	1.15	1.70	>0.999999999999999	0.7
Q9Y365	PCTP-like protein OS=Homo sapiens GN=STARD10 PE=2 SV=2	2.04	5.66	18.41	13.04	6.22	4.96	13.17	0.90	1.37	1.85	5.19	4.98	2.35	0.00	10.97	10.26	1.27	0.00	5.97	1.30	0.00	3.67	8.66	0.84	6.48	3.77	>0.999999999999999	1.7
P02749	Beta-2-glycoprotein 1 OS=Homo sapiens GN=APOH PE=1 SV=3	1.63	0.00	1.91	1.71	3.21	0.00	2.91	2.44	2.33	5.00	0.00	2.20	4.00	0.00	1.91	2.86	3.42	1.23	3.07	6.36	4.56	0.64	0.00	1.08	1.94	2.43	>0.999999999999999	0.8
P01742	Ig heavy chain V-I region EU OS=Homo sapiens PE=1 SV=1	5.51	0.00	0.00	0.00	5.09	0.00	4.62	2.44	0.00	0.00	0.00	2.20	0.00	0.00	0.00	4.54	3.42	4.14	4.88	3.51	4.56	0.00	3.83	1.08	1.65	2.50	>0.999999999999999	0.7
P61081	NEDD8-conjugating enzyme Ubc12 OS=Homo sapiens GN=UBE2M PE=1 SV=1	3.26	0.00	3.82	3.42	0.00	3.78	2.74	0.00	0.00	2.96	2.52	1.31	0.00	2.57	3.82	2.69	0.00	2.45	0.00	0.00	0.00	1.28	4.82	0.00	1.98	1.47	>0.999999999999999	1.4
P61163	Alpha-centractin OS=Homo sapiens GN=ACTR1A PE=1 SV=1	1.63	1.37	3.82	9.62	4.71	3.78	0.00	0.00	0.00	2.96	5.50	2.04	0.00	2.57	5.96	4.21	0.00	1.23	0.00	0.00	0.00	2.00	3.55	0.64	2.95	1.68	>0.999999999999999	1.8
Q04446	1,4-alpha-glucan-branching enzyme OS=Homo sapiens GN=GBE1 PE=1 SV=3	1.63	1.37	3.11	1.71	0.00	1.89	0.68	0.36	0.00	0.00	1.26	0.00	0.00	0.00	0.00	0.00	0.51	0.00	0.00	0.00	0.00	0.32	0.00	0.00	1.00	0.07	>0.999999999999999	14.6
O14879	Interferon-induced protein with tetratricopeptide repeats 3 OS=Homo sapiens GN=IFIT3 PE=1 SV=1	1.22	0.00	0.00	0.00	0.00	0.00	3.08	0.00	0.00	0.00	0.00	6.20	2.82	0.00	0.00	0.00	0.00	0.00	4.52	0.00	0.00	0.00	0.00	0.24	0.88	0.63	>0.999999999999999	1.4
P27797	Calreticulin OS=Homo sapiens GN=CALR PE=1 SV=1	4.49	2.40	3.35	6.41	1.32	1.65	1.20	1.26	0.96	5.56	1.10	4.81	3.29	1.12	1.67	2.36	0.89	1.07	2.53	0.91	5.06	1.12	3.12	2.36	2.88	2.13	>0.999999999999999	1.4
Q2VIR3	Putative eukaryotic translation initiation factor 2 subunit 3-like protein OS=Homo sapiens GN=EIF2S3L PE=5 SV=2	2.65	0.00	0.00	0.00	0.00	0.00	0.00	1.17	0.82	0.00	3.15	0.00	1.41	0.00	0.00	0.00	0.00	0.76	0.92	0.00	0.00	0.00	0.00	0.00	0.65	0.26	>0.999999999999999	2.5
P40429	60S ribosomal protein L13a OS=Homo sapiens GN=RPL13A PE=1 SV=2	2.85	5.15	7.17	6.41	5.66	7.09	5.13	0.00	0.00	2.59	4.72	0.00	0.00	0.00	3.34	0.00	0.00	0.00	2.53	0.00	0.00	0.00	4.26	0.00	3.90	0.84	>0.999999999999999	4.6
Q96HC4	PDZ and LIM domain protein 5 OS=Homo sapiens GN=PDLIM5 PE=1 SV=5	1.02	0.86	1.20	1.07	0.00	1.18	0.00	0.00	0.68	0.00	0.79	0.00	0.00	0.80	0.00	0.84	0.00	0.00	0.00	0.00	0.00	0.80	0.71	0.00	0.57	0.26	>0.999999999999999	2.2
Q00839	Heterogeneous nuclear ribonucleoprotein U OS=Homo sapiens GN=HNRNPU PE=1 SV=6	1.43	1.20	1.67	0.86	2.07	1.65	1.20	0.63	0.96	2.78	2.36	1.55	0.00	0.00	0.95	0.00	0.00	0.61	1.27	0.00	0.00	0.88	0.99	0.44	1.53	0.43	>0.999999999999999	3.6
P28070	Proteasome subunit beta type-4 OS=Homo sapiens GN=PSMB4 PE=1 SV=4	4.89	1.89	5.74	5.13	4.53	2.60	1.88	2.17	0.00	0.00	3.77	0.90	5.64	1.77	5.72	1.85	1.39	1.69	1.99	0.00	0.00	0.88	3.41	0.00	2.79	2.03	>0.999999999999999	1.4
Q13296	Mammaglobin-A OS=Homo sapiens GN=SCGB2A2 PE=1 SV=1	6.53	0.00	0.00	0.00	0.00	0.00	5.47	2.89	0.00	0.00	0.00	0.00	0.00	0.00	0.00	0.00	0.00	4.91	5.79	9.61	0.00	2.56	0.00	2.96	1.24	2.15	>0.999999999999999	0.6
Q9H4M9	EH domain-containing protein 1 OS=Homo sapiens GN=EHD1 PE=1 SV=2	1.02	0.00	0.00	0.00	0.00	0.00	0.00	0.00	0.68	0.00	0.00	0.00	1.18	0.00	0.00	0.00	0.63	0.00	0.00	0.65	0.84	0.00	0.00	0.20	0.14	0.29	>0.999999999999999	0.5
P59998	Actin-related protein 2/3 complex subunit 4 OS=Homo sapiens GN=ARPC4 PE=1 SV=3	3.47	10.29	4.06	7.91	22.63	4.02	15.05	10.84	8.22	0.00	13.84	7.18	8.70	5.94	4.05	10.09	4.68	2.61	21.70	11.42	10.13	4.79	8.51	1.48	8.96	7.84	>0.999999999999999	1.1
O15511	Actin-related protein 2/3 complex subunit 5 OS=Homo sapiens GN=ARPC5 PE=1 SV=3	4.28	3.60	11.00	9.83	3.96	4.96	3.59	1.90	0.00	3.89	3.30	3.75	10.82	3.37	10.97	3.53	2.66	3.22	8.32	5.97	3.54	1.68	2.98	3.04	4.51	5.01	>0.999999999999999	0.9
Q16629	Serine/arginine-rich splicing factor 7 OS=Homo sapiens GN=SRSF7 PE=1 SV=1	2.45	0.00	0.00	0.00	0.00	0.00	0.00	0.00	0.00	2.22	0.00	0.00	0.00	0.00	0.00	0.00	0.00	0.00	0.00	0.00	0.00	0.00	0.00	0.00	0.39	0.00	>0.999999999999999	#DIV/0!
P62081	40S ribosomal protein S7 OS=Homo sapiens GN=RPS7 PE=1 SV=1	3.06	0.00	7.89	7.06	6.22	12.52	13.00	2.98	4.52	6.11	5.19	4.32	7.76	8.51	12.64	0.00	4.18	5.06	2.71	0.00	0.00	1.20	4.68	2.12	6.07	4.07	>0.999999999999999	1.5
Q14847	LIM and SH3 domain protein 1 OS=Homo sapiens GN=LASP1 PE=1 SV=2	4.69	0.00	8.84	2.35	13.01	5.43	8.89	0.99	7.12	2.04	5.82	5.63	8.70	3.69	5.49	0.00	1.39	1.69	1.99	0.00	3.88	1.84	3.26	1.48	5.40	2.78	>0.999999999999999	1.9
Q9UKK9	ADP-sugar pyrophosphatase OS=Homo sapiens GN=NUDT5 PE=1 SV=1	2.85	4.97	6.93	2.99	8.67	10.87	4.96	1.26	1.92	12.23	7.23	3.75	6.82	4.66	0.00	4.88	3.67	0.00	5.25	0.00	0.00	5.27	4.12	0.00	5.72	2.89	>0.999999999999999	2.0
P55786	Puromycin-sensitive aminopeptidase OS=Homo sapiens GN=NPEPPS PE=1 SV=2	1.22	2.23	6.69	9.62	6.03	6.62	4.79	1.81	1.37	5.93	7.86	2.94	2.35	3.21	8.59	2.86	0.76	2.61	5.06	0.39	1.69	1.60	5.82	0.96	4.76	2.99	>0.999999999999999	1.6
P54920	Alpha-soluble NSF attachment protein OS=Homo sapiens GN=NAPA PE=1 SV=3	4.28	0.00	11.00	4.49	11.31	2.36	5.64	1.90	0.00	3.89	1.57	3.75	2.35	0.00	0.00	0.00	1.27	1.53	1.81	0.00	0.00	1.68	2.98	0.40	4.18	1.00	>0.999999999999999	4.2
Q7Z589	Protein EMSY OS=Homo sapiens GN=EMSY PE=1 SV=2	0.41	0.00	0.00	0.00	0.38	0.00	0.34	0.00	0.00	0.00	0.00	0.16	0.00	0.00	0.00	0.00	0.25	0.31	0.00	0.26	0.00	0.00	0.00	0.08	0.11	0.07	>0.999999999999999	1.4
Q12840	Kinesin heavy chain isoform 5A OS=Homo sapiens GN=KIF5A PE=1 SV=2	0.61	0.00	0.00	0.00	0.00	0.00	0.00	0.00	0.00	0.00	0.00	0.00	0.71	0.00	0.00	0.00	0.00	0.46	0.54	0.00	0.00	0.00	0.00	0.00	0.05	0.14	>0.999999999999999	0.4
Q9UEW8	STE20/SPS1-related proline-alanine-rich protein kinase OS=Homo sapiens GN=STK39 PE=1 SV=3	1.02	0.00	2.63	2.35	2.07	2.60	1.88	0.00	0.68	2.04	1.73	0.41	0.00	0.00	2.62	0.84	0.63	0.77	0.90	0.00	0.00	0.88	1.56	0.20	1.45	0.70	>0.999999999999999	2.1
Q7L1Q6	Basic leucine zipper and W2 domain-containing protein 1 OS=Homo sapiens GN=BZW1 PE=1 SV=1	1.43	2.40	1.67	2.99	4.15	3.31	3.76	1.26	0.96	0.00	2.20	1.14	0.00	0.00	1.67	3.70	1.77	1.07	0.00	0.00	0.00	0.00	0.99	0.88	2.11	0.84	>0.999999999999999	2.5
P18621	60S ribosomal protein L17 OS=Homo sapiens GN=RPL17 PE=1 SV=3	3.26	2.74	7.89	3.42	6.22	12.76	2.74	0.00	0.00	2.96	2.52	0.00	3.76	2.57	0.00	2.69	0.00	0.00	2.89	0.00	0.00	0.00	0.00	0.00	3.71	0.99	>0.999999999999999	3.7
P42166	Lamina-associated polypeptide 2, isoform alpha OS=Homo sapiens GN=TMPO PE=1 SV=2	0.82	0.69	0.00	0.86	0.75	0.95	2.39	0.36	0.55	0.00	0.00	0.00	0.00	0.64	0.00	0.00	0.00	0.61	0.72	0.00	0.68	0.32	0.57	0.00	0.61	0.30	>0.999999999999999	2.1
P50224	Sulfotransferase 1A3/1A4 OS=Homo sapiens GN=SULT1A3 PE=1 SV=1	4.08	5.49	10.76	6.84	3.77	7.56	5.47	2.89	2.74	5.93	9.28	2.61	4.70	7.23	7.63	1.68	1.27	3.07	1.81	0.00	0.00	0.00	0.00	1.80	5.62	2.43	>0.999999999999999	2.3
P09496	Clathrin light chain A OS=Homo sapiens GN=CLTA PE=1 SV=1	2.45	0.00	6.22	5.56	2.26	2.84	2.05	0.00	1.64	2.22	1.89	0.98	0.00	0.00	6.20	0.00	1.52	0.00	0.00	0.00	0.00	0.96	5.82	0.00	2.34	1.21	>0.999999999999999	1.9
O43390	Heterogeneous nuclear ribonucleoprotein R OS=Homo sapiens GN=HNRNPR PE=1 SV=1	1.02	1.54	2.15	1.07	2.64	5.91	1.54	0.81	0.68	3.71	1.42	0.73	0.00	1.45	1.19	0.00	0.00	0.77	2.53	0.00	0.00	0.72	1.28	0.56	1.94	0.71	>0.999999999999999	2.7
O14618	Copper chaperone for superoxide dismutase OS=Homo sapiens GN=CCS PE=1 SV=1	2.24	1.89	2.63	0.00	2.07	2.60	1.88	0.99	0.00	2.04	0.00	0.00	2.59	1.77	0.00	0.00	0.00	1.69	1.99	0.00	0.00	0.00	1.56	0.44	1.36	0.84	>0.999999999999999	1.6
P14735	Insulin-degrading enzyme OS=Homo sapiens GN=IDE PE=1 SV=4	1.22	1.03	0.72	0.64	1.13	1.42	0.51	0.27	0.00	0.56	1.26	0.49	0.00	0.48	0.72	1.85	0.76	0.92	1.09	0.00	0.00	0.64	1.14	0.24	0.77	0.65	>0.999999999999999	1.2
Q9GZT8	NIF3-like protein 1 OS=Homo sapiens GN=NIF3L1 PE=1 SV=2	1.63	1.37	1.91	1.71	0.00	3.78	2.74	0.72	0.00	1.48	1.26	0.00	0.00	1.28	1.91	1.35	0.00	0.00	2.89	0.00	0.00	1.28	1.14	0.00	1.38	0.82	>0.999999999999999	1.7
P14317	Hematopoietic lineage cell-specific protein OS=Homo sapiens GN=HCLS1 PE=1 SV=3	1.22	2.23	1.43	1.28	2.45	4.49	2.22	0.54	0.00	2.41	2.04	0.49	1.41	2.09	1.43	0.00	0.00	0.92	1.09	0.00	0.00	1.04	3.83	0.24	1.73	1.00	>0.999999999999999	1.7
P19878	Neutrophil cytosol factor 2 OS=Homo sapiens GN=NCF2 PE=1 SV=2	2.24	0.00	0.00	0.00	0.00	0.00	0.00	0.00	0.00	0.00	0.00	0.00	0.00	0.00	0.00	0.00	0.00	0.00	0.00	0.00	0.84	0.00	0.00	0.00	0.19	0.07	>0.999999999999999	2.7
O75937	DnaJ homolog subfamily C member 8 OS=Homo sapiens GN=DNAJC8 PE=1 SV=2	2.24	1.89	0.00	0.00	2.07	0.00	0.00	0.00	0.00	0.00	1.73	0.90	0.00	0.00	2.62	0.00	0.00	0.00	0.00	0.00	0.00	0.88	0.00	0.00	0.74	0.29	>0.999999999999999	2.5
Q1KMD3	Heterogeneous nuclear ribonucleoprotein U-like protein 2 OS=Homo sapiens GN=HNRNPUL2 PE=1 SV=1	1.63	0.69	0.00	1.71	0.75	1.89	0.00	0.00	0.00	0.74	0.00	0.00	0.00	0.00	0.00	0.00	0.00	0.00	0.00	0.00	0.00	0.00	0.00	0.00	0.62	0.00	>0.999999999999999	#DIV/0!
Q13404	Ubiquitin-conjugating enzyme E2 variant 1 OS=Homo sapiens GN=UBE2V1 PE=1 SV=2	4.08	3.43	4.78	4.28	8.48	4.73	0.00	0.00	2.74	3.71	3.15	1.63	4.70	0.00	4.77	0.00	2.53	3.07	0.00	2.60	0.00	1.60	2.84	0.80	3.42	1.91	>0.999999999999999	1.8
Q9UBQ0	Vacuolar protein sorting-associated protein 29 OS=Homo sapiens GN=VPS29 PE=1 SV=1	3.26	2.74	3.82	12.19	10.75	8.27	9.75	0.00	0.00	6.48	8.96	2.85	0.00	2.57	13.59	9.59	0.00	2.45	0.00	0.00	0.00	2.80	2.27	0.00	5.76	2.77	>0.999999999999999	2.1
P13861	cAMP-dependent protein kinase type II-alpha regulatory subunit OS=Homo sapiens GN=PRKAR2A PE=1 SV=2	1.43	5.49	12.43	13.47	4.34	7.56	2.56	0.63	2.05	5.93	3.62	2.61	1.65	3.69	12.40	3.87	0.89	1.07	2.71	0.00	1.18	2.56	4.54	0.60	5.18	2.93	>0.999999999999999	1.8
Q9H444	Charged multivesicular body protein 4b OS=Homo sapiens GN=CHMP4B PE=1 SV=1	2.65	4.97	3.11	6.20	0.00	0.00	2.22	0.00	0.00	0.00	0.00	2.37	0.00	2.09	3.10	2.19	0.00	2.35	0.00	0.00	0.00	1.04	1.84	0.00	1.79	1.05	>0.999999999999999	1.7
P56192	Methionine--tRNA ligase, cytoplasmic OS=Homo sapiens GN=MARS PE=1 SV=2	0.61	0.00	5.02	2.78	1.32	3.07	0.51	0.00	0.00	0.56	1.57	0.57	0.71	0.48	3.10	0.50	0.00	0.00	0.54	0.00	0.00	0.24	0.43	0.40	1.33	0.53	>0.999999999999999	2.5
P04264	Keratin, type II cytoskeletal 1 OS=Homo sapiens GN=KRT1 PE=1 SV=6	2.04	12.01	0.00	11.76	7.54	13.00	2.74	1.44	3.70	21.68	8.65	7.18	4.94	9.95	5.01	4.54	8.86	0.00	12.66	2.73	23.46	5.59	3.83	3.16	7.64	7.06	>0.999999999999999	1.1
O75369	Filamin-B OS=Homo sapiens GN=FLNB PE=1 SV=2	0.20	0.00	1.43	1.28	0.94	0.47	1.03	0.00	0.27	0.00	0.63	0.33	0.24	0.00	1.43	0.84	0.63	0.15	1.27	0.00	0.00	0.00	0.14	0.28	0.55	0.42	>0.999999999999999	1.3
Q9BV40	Vesicle-associated membrane protein 8 OS=Homo sapiens GN=VAMP8 PE=1 SV=1	6.12	5.15	0.00	6.41	0.00	0.00	5.13	2.71	4.11	0.00	4.72	0.00	0.00	0.00	7.15	11.61	3.80	4.60	5.43	0.00	5.06	2.40	4.26	0.00	2.86	3.69	>0.999999999999999	0.8
Q5QNW6	Histone H2B type 2-F OS=Homo sapiens GN=HIST2H2BF PE=1 SV=3	11.22	0.00	0.00	0.00	17.54	0.00	4.27	2.26	7.53	4.63	3.93	4.49	0.00	4.01	0.00	0.00	11.77	8.44	4.52	12.07	9.28	0.00	0.00	0.00	4.66	4.17	>0.999999999999999	1.1
P52272	Heterogeneous nuclear ribonucleoprotein M OS=Homo sapiens GN=HNRNPM PE=1 SV=3	2.65	2.23	4.30	7.27	3.39	8.03	1.54	1.63	0.00	3.33	5.35	1.88	3.06	1.45	5.49	2.19	1.14	1.99	2.35	0.00	0.00	1.04	3.26	0.72	3.47	1.89	>0.999999999999999	1.8
Q8TC07	TBC1 domain family member 15 OS=Homo sapiens GN=TBC1D15 PE=1 SV=2	0.82	0.00	1.91	0.00	0.75	0.95	0.00	0.00	0.00	0.00	0.00	0.00	0.00	0.00	0.95	0.00	0.00	0.00	0.00	0.00	0.00	0.64	0.57	0.00	0.37	0.18	>0.999999999999999	2.0
Q9NZT2	Opioid growth factor receptor OS=Homo sapiens GN=OGFR PE=1 SV=3	0.82	1.54	2.15	1.92	0.00	0.00	0.68	0.00	0.00	1.67	1.42	0.00	0.00	0.00	0.95	0.67	0.00	0.61	0.72	0.00	0.00	0.72	0.00	0.00	0.85	0.31	>0.999999999999999	2.8
A6NKL6	Transmembrane protein 200C OS=Homo sapiens GN=TMEM200C PE=2 SV=2	1.02	0.00	0.00	0.00	0.94	1.18	0.85	0.45	0.68	0.93	0.79	0.41	1.18	0.80	0.00	0.00	0.63	0.77	0.90	0.65	0.84	0.00	0.00	0.20	0.60	0.50	>0.999999999999999	1.2
Q6UX06	Olfactomedin-4 OS=Homo sapiens GN=OLFM4 PE=1 SV=1	1.22	0.00	0.00	0.00	0.00	0.00	0.00	0.00	0.00	0.00	0.00	0.00	2.82	0.00	0.00	0.00	0.00	0.00	0.00	0.00	1.56	0.00	0.00	0.00	0.10	0.37	>0.999999999999999	0.3
Q9Y5X3	Sorting nexin-5 OS=Homo sapiens GN=SNX5 PE=1 SV=1	1.43	1.20	1.67	1.50	1.32	9.45	0.00	0.00	0.00	1.30	3.62	0.00	0.00	0.00	0.00	0.00	0.00	0.00	0.00	0.00	0.00	0.56	2.13	0.00	1.79	0.22	>0.999999999999999	8.0
O75436	Vacuolar protein sorting-associated protein 26A OS=Homo sapiens GN=VPS26A PE=1 SV=2	1.84	0.00	4.30	0.00	1.70	2.13	1.54	0.00	0.00	0.00	0.00	0.00	0.00	0.00	2.15	0.00	0.00	0.00	0.00	0.00	0.00	0.72	1.28	0.00	0.96	0.35	>0.999999999999999	2.8
P62701	40S ribosomal protein S4, X isoform OS=Homo sapiens GN=RPS4X PE=1 SV=2	2.24	1.89	5.74	5.13	4.53	8.74	1.88	0.99	3.29	2.04	11.01	0.00	8.70	1.77	5.72	1.85	1.39	1.69	4.34	1.43	0.00	1.92	9.93	0.00	3.96	3.23	>0.999999999999999	1.2
Q5VWZ2	Lysophospholipase-like protein 1 OS=Homo sapiens GN=LYPLAL1 PE=1 SV=3	2.65	0.00	0.00	0.00	0.00	0.00	0.00	0.00	0.00	0.00	0.00	0.00	0.00	0.00	0.00	0.00	0.00	0.00	0.00	0.00	0.00	0.00	1.84	0.00	0.22	0.15	>0.999999999999999	1.4
Q9Y3C8	Ubiquitin-fold modifier-conjugating enzyme 1 OS=Homo sapiens GN=UFC1 PE=1 SV=3	7.55	2.92	4.06	3.63	3.21	4.02	2.91	3.34	5.07	6.85	2.67	1.39	4.00	2.73	4.05	2.86	2.15	2.61	3.07	0.00	0.00	2.96	5.25	0.00	3.97	2.47	>0.999999999999999	1.6
Q12955	Ankyrin-3 OS=Homo sapiens GN=ANK3 PE=1 SV=3	0.20	0.00	0.24	0.21	0.19	0.24	0.00	0.00	0.00	0.19	0.16	0.08	0.00	0.00	0.00	0.00	0.00	0.00	0.00	0.00	0.00	0.00	0.00	0.00	0.13	0.00	>0.999999999999999	#DIV/0!
Q15907	Ras-related protein Rab-11B OS=Homo sapiens GN=RAB11B PE=1 SV=4	5.91	4.97	6.93	19.03	8.67	21.03	7.87	1.26	6.30	2.59	4.56	5.38	15.52	2.25	21.23	2.36	8.35	7.06	11.94	0.00	7.76	5.27	12.63	1.84	7.88	8.02	>0.999999999999999	1.0
P34896	Serine hydroxymethyltransferase, cytosolic OS=Homo sapiens GN=SHMT1 PE=1 SV=1	2.65	0.00	8.37	4.28	0.00	3.07	1.03	0.00	0.00	0.00	0.00	0.49	0.00	0.00	1.43	0.00	0.00	0.00	0.00	0.00	0.00	1.04	0.85	0.00	1.66	0.28	>0.999999999999999	6.0
P55263	Adenosine kinase OS=Homo sapiens GN=ADK PE=1 SV=2	1.63	4.46	1.91	1.71	3.21	1.89	1.37	0.00	0.00	1.48	0.00	0.00	1.88	1.28	8.59	2.86	2.15	1.23	1.45	0.00	0.00	0.64	2.41	0.32	1.47	1.90	>0.999999999999999	0.8
O00571	ATP-dependent RNA helicase DDX3X OS=Homo sapiens GN=DDX3X PE=1 SV=3	1.84	0.00	0.00	4.06	6.79	8.51	1.54	0.00	0.00	3.52	3.77	1.14	0.94	0.00	4.53	1.51	0.51	0.61	2.53	0.00	0.00	0.32	1.99	0.96	2.60	1.16	>0.999999999999999	2.2
P49321	Nuclear autoantigenic sperm protein OS=Homo sapiens GN=NASP PE=1 SV=2	2.45	2.06	3.82	3.42	2.26	2.84	2.05	0.36	1.10	1.48	2.52	0.33	0.00	1.28	1.91	2.02	0.00	0.61	0.72	0.00	0.00	0.96	0.57	0.16	2.06	0.69	>0.999999999999999	3.0
O95817	BAG family molecular chaperone regulator 3 OS=Homo sapiens GN=BAG3 PE=1 SV=3	1.02	0.00	0.00	0.00	0.00	0.00	0.00	0.00	0.00	0.00	0.00	0.41	0.00	0.00	0.00	0.00	0.00	0.00	0.00	0.00	0.00	0.00	0.00	0.20	0.12	0.02	>0.999999999999999	7.1
P46777	60S ribosomal protein L5 OS=Homo sapiens GN=RPL5 PE=1 SV=3	2.04	0.00	4.78	4.28	3.77	4.73	3.42	0.90	0.00	3.71	3.15	1.63	0.00	1.61	2.38	1.68	1.27	0.00	0.00	0.00	0.00	1.60	1.42	0.40	2.70	0.86	>0.999999999999999	3.1
Q9UGI8	Testin OS=Homo sapiens GN=TES PE=1 SV=1	1.43	1.20	0.00	1.50	2.64	1.65	1.20	0.63	0.00	1.30	1.10	0.57	0.00	0.00	0.00	0.00	0.89	0.00	0.00	0.00	0.00	1.12	1.99	0.28	1.10	0.36	>0.999999999999999	3.1
Q9UBT2	SUMO-activating enzyme subunit 2 OS=Homo sapiens GN=UBA2 PE=1 SV=2	1.02	0.86	2.15	1.92	1.70	1.18	0.00	0.00	0.00	1.67	1.42	0.73	0.00	0.80	2.15	0.84	0.00	0.00	0.00	0.00	0.00	0.40	0.71	0.36	1.05	0.44	>0.999999999999999	2.4
P20290	Transcription factor BTF3 OS=Homo sapiens GN=BTF3 PE=1 SV=1	3.06	2.57	3.59	3.21	2.83	3.54	2.56	1.35	0.00	0.00	5.03	0.00	0.00	2.41	3.58	2.52	0.00	0.00	0.00	0.00	0.00	1.20	2.13	0.60	2.31	1.04	>0.999999999999999	2.2
P46776	60S ribosomal protein L27a OS=Homo sapiens GN=RPL27A PE=1 SV=2	4.08	3.43	10.76	9.62	3.77	10.63	3.42	1.81	0.00	3.71	11.64	3.67	0.00	0.00	10.73	7.57	2.53	0.00	3.62	0.00	0.00	3.59	6.39	0.00	5.54	2.87	>0.999999999999999	1.9
P42766	60S ribosomal protein L35 OS=Homo sapiens GN=RPL35 PE=1 SV=2	4.69	3.95	5.50	11.12	4.34	5.43	3.93	0.00	3.15	4.26	3.62	0.00	5.41	3.69	0.00	3.87	2.91	3.53	4.16	0.00	3.88	1.84	0.00	0.00	4.17	2.44	>0.999999999999999	1.7
P19525	Interferon-induced, double-stranded RNA-activated protein kinase OS=Homo sapiens GN=EIF2AK2 PE=1 SV=2	2.24	0.86	1.20	2.35	2.07	2.60	0.85	0.00	0.00	0.93	1.73	0.90	2.59	0.00	1.19	0.00	0.00	0.00	0.90	0.00	0.00	0.40	0.71	0.00	1.31	0.48	>0.999999999999999	2.7
Q9HD89	Resistin OS=Homo sapiens GN=RETN PE=2 SV=1	5.71	0.00	0.00	0.00	0.00	0.00	0.00	2.53	0.00	0.00	0.00	0.00	6.59	0.00	0.00	0.00	0.00	4.30	0.00	0.00	4.73	0.00	0.00	0.00	0.69	1.30	>0.999999999999999	0.5
P80303	Nucleobindin-2 OS=Homo sapiens GN=NUCB2 PE=1 SV=2	2.85	2.40	3.35	4.49	10.56	3.31	2.39	10.30	7.67	2.59	3.30	10.44	8.70	7.39	3.34	6.23	8.35	17.49	3.80	4.80	13.00	6.15	5.25	3.56	5.30	7.34	>0.999999999999999	0.7
P62861	40S ribosomal protein S30 OS=Homo sapiens GN=FAU PE=1 SV=1	11.01	0.00	12.91	11.55	10.18	12.76	9.23	4.88	0.00	10.00	8.49	0.00	12.70	8.67	12.88	9.09	6.83	0.00	0.00	0.00	0.00	0.00	7.66	2.16	7.58	5.00	>0.999999999999999	1.5
Q6P996	Pyridoxal-dependent decarboxylase domain-containing protein 1 OS=Homo sapiens GN=PDXDC1 PE=1 SV=2	1.63	1.37	6.93	7.27	2.26	3.78	4.27	0.36	0.00	0.74	3.15	1.63	0.00	0.64	2.86	0.67	0.00	1.23	2.17	0.00	0.00	0.96	3.55	1.00	2.78	1.09	>0.999999999999999	2.6
Q14152	Eukaryotic translation initiation factor 3 subunit A OS=Homo sapiens GN=EIF3A PE=1 SV=1	0.82	0.34	2.39	2.14	2.83	3.54	1.37	0.00	0.27	3.52	3.30	0.65	0.00	0.00	2.38	0.00	0.00	0.00	0.36	0.00	0.00	0.00	1.14	0.24	1.76	0.34	>0.999999999999999	5.1
P62910	60S ribosomal protein L32 OS=Homo sapiens GN=RPL32 PE=1 SV=2	4.28	3.60	5.02	4.49	3.96	0.00	0.00	0.00	0.00	3.89	3.30	1.71	4.94	3.37	5.01	0.00	0.00	0.00	0.00	0.00	0.00	0.00	2.98	0.00	2.52	1.36	>0.999999999999999	1.9
P07339	Cathepsin D OS=Homo sapiens GN=CTSD PE=1 SV=1	1.43	0.00	0.00	1.50	2.83	5.67	4.10	5.78	3.29	1.30	5.19	6.20	5.64	2.41	3.58	8.92	3.04	3.68	18.63	3.12	5.57	3.35	5.96	2.12	3.11	5.50	>0.999999999999999	0.6
P35637	RNA-binding protein FUS OS=Homo sapiens GN=FUS PE=1 SV=1	1.22	1.03	1.43	1.28	1.13	1.42	1.03	0.00	0.82	1.11	0.94	0.00	0.00	0.00	1.43	0.00	0.76	0.92	1.09	0.78	0.00	0.00	0.00	0.24	0.95	0.43	>0.999999999999999	2.2
Q13045	Protein flightless-1 homolog OS=Homo sapiens GN=FLII PE=1 SV=2	0.41	2.06	3.35	3.63	1.32	4.02	2.05	0.18	0.27	3.15	3.46	1.55	1.65	0.32	4.05	1.18	0.00	0.77	0.00	0.00	0.00	0.72	1.99	0.36	2.12	0.92	>0.999999999999999	2.3
P30101	Protein disulfide-isomerase A3 OS=Homo sapiens GN=PDIA3 PE=1 SV=4	2.45	3.09	9.56	13.90	1.13	5.91	4.27	4.33	3.42	2.22	8.81	5.30	2.82	7.71	4.29	8.08	3.16	3.84	5.79	0.78	5.40	2.00	6.81	2.24	5.37	4.41	>0.999999999999999	1.2
O95352	Ubiquitin-like modifier-activating enzyme ATG7 OS=Homo sapiens GN=ATG7 PE=1 SV=1	0.82	0.69	0.96	0.86	0.00	0.00	0.68	0.00	0.00	0.00	0.63	0.65	0.00	0.00	0.00	0.00	0.00	0.00	0.72	0.00	0.00	0.64	0.00	0.00	0.44	0.11	>0.999999999999999	3.9
P00748	Coagulation factor XII OS=Homo sapiens GN=F12 PE=1 SV=3	2.04	0.00	0.00	1.07	0.94	0.00	1.71	0.90	2.05	0.00	0.00	0.00	0.00	0.00	1.19	0.00	1.90	0.00	1.81	0.65	3.38	0.00	1.42	0.00	0.73	0.86	>0.999999999999999	0.8
Q9Y6W5	Wiskott-Aldrich syndrome protein family member 2 OS=Homo sapiens GN=WASF2 PE=1 SV=3	1.22	2.06	1.43	4.06	1.13	0.00	1.03	0.00	0.00	1.11	0.94	0.49	1.41	0.00	1.43	0.00	0.00	0.00	1.09	0.00	0.00	0.00	0.00	0.24	1.12	0.35	>0.999999999999999	3.2
Q12805	EGF-containing fibulin-like extracellular matrix protein 1 OS=Homo sapiens GN=EFEMP1 PE=1 SV=2	1.22	0.00	1.43	2.57	1.13	1.42	1.03	0.54	0.00	0.00	0.00	0.49	0.00	0.00	0.00	2.02	0.00	0.00	1.09	0.00	0.00	0.00	0.85	0.00	0.82	0.33	>0.999999999999999	2.5
P04211	Ig lambda chain V region 4A OS=Homo sapiens PE=4 SV=1	5.51	0.00	0.00	5.77	5.09	0.00	4.62	5.51	0.00	0.00	0.00	2.20	14.35	4.34	6.44	4.54	7.72	4.14	4.88	3.51	10.30	2.16	3.83	0.00	2.39	5.52	>0.999999999999999	0.4
O00757	Fructose-1,6-bisphosphatase isozyme 2 OS=Homo sapiens GN=FBP2 PE=1 SV=2	1.84	1.54	0.00	1.92	1.70	2.13	1.54	0.81	0.00	0.00	1.42	0.73	0.00	1.45	2.15	1.51	1.14	1.38	1.63	0.00	1.52	0.72	1.28	0.00	1.14	1.06	>0.999999999999999	1.1
Q02790	Peptidyl-prolyl cis-trans isomerase FKBP4 OS=Homo sapiens GN=FKBP4 PE=1 SV=3	1.22	2.23	4.78	1.28	5.28	8.51	0.00	0.00	0.00	3.71	5.66	1.06	0.00	0.96	3.10	0.00	0.00	1.99	2.35	0.00	0.00	0.48	1.84	0.24	2.81	0.91	>0.999999999999999	3.1
Q96AT9	Ribulose-phosphate 3-epimerase OS=Homo sapiens GN=RPE PE=1 SV=1	2.65	0.00	6.69	0.00	0.00	0.00	0.00	0.00	0.00	2.41	0.00	0.00	0.00	0.00	0.00	0.00	0.00	0.00	0.00	0.00	0.00	2.24	0.00	0.00	0.98	0.19	>0.999999999999999	5.3
P01769	Ig heavy chain V-III region GA OS=Homo sapiens PE=1 SV=1	11.83	0.00	0.00	0.00	0.00	0.00	0.00	2.35	0.00	0.00	0.00	0.00	6.12	0.00	0.00	4.37	0.00	3.99	0.00	3.38	0.00	0.00	3.69	0.00	1.18	1.80	>0.999999999999999	0.7
Q6IBS0	Twinfilin-2 OS=Homo sapiens GN=TWF2 PE=1 SV=2	1.63	1.37	1.91	3.63	5.09	8.98	2.91	0.72	1.10	3.15	1.26	3.10	1.88	2.73	0.00	1.35	1.01	2.61	3.07	0.00	2.87	2.16	2.41	1.52	2.90	1.80	>0.999999999999999	1.6
Q9UKK3	Poly [ADP-ribose] polymerase 4 OS=Homo sapiens GN=PARP4 PE=1 SV=3	0.61	0.00	0.48	0.43	0.38	0.71	0.34	0.00	0.00	0.56	0.79	0.00	0.00	0.00	0.72	0.34	0.00	0.00	0.00	0.00	0.00	0.00	0.28	0.00	0.36	0.11	>0.999999999999999	3.2
P08246	Neutrophil elastase OS=Homo sapiens GN=ELANE PE=1 SV=1	2.24	0.00	0.00	0.00	0.00	0.00	4.10	0.99	0.00	0.00	0.00	0.00	2.59	0.00	0.00	0.00	0.00	1.69	1.99	3.12	4.05	0.00	0.00	0.00	0.61	1.12	>0.999999999999999	0.5
Q99439	Calponin-2 OS=Homo sapiens GN=CNN2 PE=1 SV=4	2.04	1.72	0.00	2.14	1.89	2.36	0.00	0.00	0.00	0.00	0.00	0.82	0.00	0.00	0.00	1.68	0.00	0.00	1.81	0.00	0.00	0.00	0.00	0.00	0.91	0.29	>0.999999999999999	3.1
Q93052	Lipoma-preferred partner OS=Homo sapiens GN=LPP PE=1 SV=1	1.02	0.00	3.59	0.00	0.94	2.36	0.85	0.00	0.00	0.93	1.57	0.41	0.00	0.00	1.19	0.00	0.00	0.77	0.00	0.00	0.00	0.40	0.71	0.00	0.97	0.26	>0.999999999999999	3.8
P23284	Peptidyl-prolyl cis-trans isomerase B OS=Homo sapiens GN=PPIB PE=1 SV=2	2.85	0.00	0.00	6.41	2.64	0.00	2.39	2.71	6.57	0.00	2.20	2.45	11.29	2.25	0.00	5.05	6.07	10.59	2.53	6.23	8.10	1.12	1.99	2.76	2.35	4.83	>0.999999999999999	0.5
Q5VZK9	Leucine-rich repeat-containing protein 16A OS=Homo sapiens GN=LRRC16A PE=1 SV=1	0.41	0.00	0.48	0.86	0.38	0.95	0.34	0.00	0.00	0.37	0.00	0.00	0.00	0.00	0.95	0.00	0.00	0.00	0.36	0.00	0.00	0.16	0.00	0.00	0.31	0.12	>0.999999999999999	2.6
Q04760	Lactoylglutathione lyase OS=Homo sapiens GN=GLO1 PE=1 SV=4	3.26	6.00	3.82	7.48	0.00	8.27	5.98	1.44	0.00	2.96	5.50	1.31	0.00	5.62	3.82	2.69	2.02	2.45	2.89	0.00	0.00	4.47	7.95	0.64	3.84	2.71	>0.999999999999999	1.4
P13647	Keratin, type II cytoskeletal 5 OS=Homo sapiens GN=KRT5 PE=1 SV=3	2.24	2.92	0.00	0.00	2.07	2.60	0.00	0.99	2.33	0.00	0.00	2.37	2.59	1.77	0.00	4.88	1.39	0.00	4.16	2.21	6.08	2.32	0.00	1.16	1.29	2.21	>0.999999999999999	0.6
O60841	Eukaryotic translation initiation factor 5B OS=Homo sapiens GN=EIF5B PE=1 SV=4	0.41	0.86	1.20	0.00	1.32	1.65	0.85	0.00	0.00	0.93	0.79	0.00	0.00	0.00	0.00	0.00	0.00	0.00	0.00	0.00	0.00	0.00	0.00	0.08	0.67	0.01	>0.999999999999999	100.0
O95747	Serine/threonine-protein kinase OSR1 OS=Homo sapiens GN=OXSR1 PE=1 SV=1	1.22	1.03	1.43	0.00	2.26	4.25	3.08	0.00	0.82	3.33	3.93	2.04	0.00	0.96	1.43	0.00	0.00	0.00	2.17	0.00	0.00	0.96	1.70	0.24	1.95	0.62	>0.999999999999999	3.1
Q8IVT2	Uncharacterized protein C19orf21 OS=Homo sapiens GN=C19orf21 PE=1 SV=1	0.82	0.00	0.96	0.86	0.00	0.00	0.00	0.00	0.00	0.00	1.42	0.00	0.00	0.00	0.95	0.67	0.00	0.00	0.00	0.00	0.00	0.32	0.57	0.16	0.34	0.22	>0.999999999999999	1.5
P22079	Lactoperoxidase OS=Homo sapiens GN=LPO PE=1 SV=2	0.82	2.23	0.00	4.70	0.00	0.00	0.68	6.05	5.89	0.00	0.63	3.02	1.88	3.53	0.00	2.86	1.01	5.68	0.72	4.80	3.71	1.76	0.57	1.28	2.00	2.32	>0.999999999999999	0.9
Q92841	Probable ATP-dependent RNA helicase DDX17 OS=Homo sapiens GN=DDX17 PE=1 SV=2	1.63	0.00	1.91	2.78	1.51	5.20	0.00	0.36	0.55	2.41	4.25	0.00	0.00	0.00	1.91	1.35	0.00	0.00	0.00	0.00	0.00	0.64	1.84	0.16	1.72	0.49	>0.999999999999999	3.5
Q9NZL9	Methionine adenosyltransferase 2 subunit beta OS=Homo sapiens GN=MAT2B PE=1 SV=1	1.84	0.00	0.00	1.92	3.39	2.13	1.54	0.81	1.23	0.00	2.83	0.73	0.00	0.00	2.15	0.00	1.14	0.00	1.63	0.00	0.00	0.72	4.12	0.00	1.37	0.81	>0.999999999999999	1.7
Q9P287	BRCA2 and CDKN1A-interacting protein OS=Homo sapiens GN=BCCIP PE=1 SV=1	1.84	0.00	4.54	0.00	1.70	2.13	1.54	0.00	0.00	1.67	1.42	0.00	0.00	0.00	0.00	0.00	0.00	0.00	0.00	0.00	0.00	0.00	0.00	0.00	1.24	0.00	>0.999999999999999	#DIV/0!
P41252	Isoleucine--tRNA ligase, cytoplasmic OS=Homo sapiens GN=IARS PE=1 SV=2	1.02	0.34	3.35	2.57	1.32	2.13	0.34	0.00	0.00	0.00	1.10	0.00	0.00	1.12	1.19	0.00	0.00	0.00	0.00	0.00	0.00	0.00	0.28	0.08	1.01	0.22	>0.999999999999999	4.5
O75131	Copine-3 OS=Homo sapiens GN=CPNE3 PE=1 SV=1	1.02	0.00	0.00	0.00	0.94	2.60	0.00	0.00	0.00	0.93	0.00	0.00	0.00	0.00	1.19	0.00	0.00	0.00	0.00	0.00	0.00	0.00	0.00	0.00	0.46	0.10	>0.999999999999999	4.6
Q01082	Spectrin beta chain, brain 1 OS=Homo sapiens GN=SPTBN1 PE=1 SV=2	0.20	1.72	5.02	3.63	2.26	4.02	0.34	0.18	0.00	3.89	2.83	0.82	0.00	0.96	2.62	0.67	0.13	0.15	0.18	0.13	0.00	0.72	2.41	0.16	2.08	0.68	>0.999999999999999	3.1
P12004	Proliferating cell nuclear antigen OS=Homo sapiens GN=PCNA PE=1 SV=1	2.24	1.89	5.74	5.13	2.07	5.67	0.00	0.00	0.00	0.00	3.77	0.00	0.00	1.77	2.62	0.00	1.39	0.00	0.00	0.00	0.00	0.00	1.56	0.44	2.21	0.65	>0.999999999999999	3.4
P62633	Cellular nucleic acid-binding protein OS=Homo sapiens GN=CNBP PE=1 SV=1	3.26	0.00	8.37	0.00	0.00	8.27	5.98	1.44	2.19	2.96	8.96	1.31	3.76	0.00	0.00	0.00	4.43	2.45	2.89	0.00	5.91	0.00	0.00	0.64	3.56	1.67	>0.999999999999999	2.1
Q9BYE4	Small proline-rich protein 2G OS=Homo sapiens GN=SPRR2G PE=2 SV=1	8.16	0.00	0.00	0.00	0.00	0.00	0.00	3.61	0.00	7.41	0.00	3.26	9.41	0.00	0.00	0.00	5.06	0.00	0.00	5.19	6.75	0.00	0.00	3.84	1.87	2.52	>0.999999999999999	0.7
P02745	Complement C1q subcomponent subunit A OS=Homo sapiens GN=C1QA PE=1 SV=2	2.65	0.00	0.00	0.00	0.00	0.00	2.22	0.00	0.00	0.00	0.00	0.00	0.00	2.09	3.10	2.19	1.64	0.00	0.00	0.00	0.00	0.00	1.84	0.52	0.41	0.95	>0.999999999999999	0.4
P53621	Coatomer subunit alpha OS=Homo sapiens GN=COPA PE=1 SV=2	0.41	2.57	6.93	6.20	4.90	7.56	4.96	0.45	2.47	3.33	7.08	2.37	1.65	1.12	7.63	3.87	1.52	1.07	2.17	0.00	0.00	3.04	4.97	0.40	4.10	2.29	>0.999999999999999	1.8
Q9NTX5	Ethylmalonyl-CoA decarboxylase OS=Homo sapiens GN=ECHDC1 PE=1 SV=2	2.04	1.72	7.65	2.14	1.89	2.36	0.00	0.00	0.00	0.00	1.57	1.71	0.00	0.00	0.00	0.00	0.00	0.00	0.00	0.00	0.00	0.00	1.42	0.00	1.76	0.12	>0.999999999999999	14.9
P17213	Bactericidal permeability-increasing protein OS=Homo sapiens GN=BPI PE=1 SV=4	1.22	0.00	0.00	0.00	0.00	0.00	0.00	0.00	0.00	0.00	0.00	0.00	0.00	0.00	0.00	0.00	0.00	0.00	0.00	0.00	1.01	0.00	0.00	0.00	0.10	0.08	>0.999999999999999	1.2
P61011	Signal recognition particle 54 kDa protein OS=Homo sapiens GN=SRP54 PE=1 SV=1	1.22	1.03	0.00	0.00	0.00	2.84	0.00	0.00	0.00	1.11	0.94	0.49	0.00	0.00	1.43	0.00	0.00	0.00	0.00	0.00	0.00	0.00	0.00	0.00	0.64	0.12	>0.999999999999999	5.3
O95361	Tripartite motif-containing protein 16 OS=Homo sapiens GN=TRIM16 PE=1 SV=3	1.02	0.86	0.00	1.07	0.94	0.00	0.85	0.00	0.00	0.00	0.00	0.82	0.00	0.80	1.19	0.00	0.00	0.77	1.81	0.00	0.00	0.40	1.42	0.00	0.46	0.53	>0.999999999999999	0.9
Q16204	Coiled-coil domain-containing protein 6 OS=Homo sapiens GN=CCDC6 PE=1 SV=2	1.22	1.03	1.43	1.28	0.00	1.42	1.03	0.00	0.82	2.41	2.04	0.00	0.00	0.96	1.43	0.00	0.00	0.00	1.09	0.00	0.00	0.00	1.84	0.24	1.06	0.46	>0.999999999999999	2.3
Q15208	Serine/threonine-protein kinase 38 OS=Homo sapiens GN=STK38 PE=1 SV=1	1.22	1.03	0.00	0.00	0.00	0.00	0.00	0.00	0.00	0.00	0.94	0.00	0.00	0.00	0.00	0.00	0.00	0.00	0.00	0.00	0.00	0.00	0.00	0.24	0.27	0.02	>0.999999999999999	13.3
O75534	Cold shock domain-containing protein E1 OS=Homo sapiens GN=CSDE1 PE=1 SV=2	0.82	0.00	0.96	0.86	1.32	1.65	0.00	0.00	0.00	1.30	1.10	0.33	0.00	0.00	0.00	0.00	0.00	0.00	0.00	0.00	0.00	0.56	0.57	0.28	0.69	0.12	>0.999999999999999	5.9
Q8NBJ4	Golgi membrane protein 1 OS=Homo sapiens GN=GOLM1 PE=1 SV=1	1.43	2.57	1.67	0.00	1.32	0.00	3.93	1.35	3.15	0.00	2.36	2.61	1.65	3.69	1.67	3.87	0.89	1.07	0.00	0.00	0.00	5.99	4.54	0.28	1.70	1.97	>0.999999999999999	0.9
Q92905	COP9 signalosome complex subunit 5 OS=Homo sapiens GN=COPS5 PE=1 SV=4	1.84	0.00	4.30	1.92	1.70	4.25	0.00	0.00	0.00	1.67	1.42	0.73	0.00	1.45	2.15	0.00	0.00	0.00	0.00	0.00	0.00	0.00	1.28	0.00	1.49	0.41	>0.999999999999999	3.7
O60547	GDP-mannose 4,6 dehydratase OS=Homo sapiens GN=GMDS PE=1 SV=1	1.63	1.37	1.91	1.71	1.51	0.00	2.74	0.00	0.00	0.00	1.26	0.65	1.88	0.00	1.91	0.00	1.01	0.00	0.00	0.00	0.00	1.28	0.00	0.64	1.07	0.56	>0.999999999999999	1.9
Q07866	Kinesin light chain 1 OS=Homo sapiens GN=KLC1 PE=1 SV=2	2.04	1.72	0.00	1.07	0.94	0.00	0.00	0.00	0.00	0.00	0.00	0.00	0.00	0.80	0.00	0.00	0.00	0.00	0.00	0.00	0.00	0.00	0.00	0.40	0.48	0.10	>0.999999999999999	4.8
P07814	Bifunctional glutamate/proline--tRNA ligase OS=Homo sapiens GN=EPRS PE=1 SV=5	0.41	0.69	3.82	3.42	1.13	2.84	1.71	0.00	0.00	2.59	1.89	0.49	0.00	0.64	1.91	0.67	0.00	0.00	0.36	0.00	0.00	0.16	1.14	0.24	1.58	0.43	>0.999999999999999	3.7
Q15628	Tumor necrosis factor receptor type 1-associated DEATH domain protein OS=Homo sapiens GN=TRADD PE=1 SV	2.04	0.00	0.00	0.00	0.00	0.00	0.00	0.00	0.00	0.00	0.00	0.00	0.00	0.00	0.00	0.00	0.00	0.00	0.00	0.00	0.00	0.00	0.00	0.00	0.17	0.00	>0.999999999999999	#DIV/0!
Q14444	Caprin-1 OS=Homo sapiens GN=CAPRIN1 PE=1 SV=2	0.82	0.69	0.96	2.78	2.45	3.07	1.54	0.36	0.55	2.41	3.62	0.00	0.94	0.64	2.15	0.67	0.51	0.61	1.63	0.52	0.00	0.32	1.28	0.00	1.60	0.77	>0.999999999999999	2.1
O14579	Coatomer subunit epsilon OS=Homo sapiens GN=COPE PE=1 SV=3	4.08	1.72	4.78	4.28	3.77	4.73	3.42	0.90	1.37	1.85	4.88	1.63	0.00	0.00	7.39	3.36	1.27	1.53	1.81	0.00	1.69	1.60	2.84	0.00	3.12	1.79	>0.999999999999999	1.7
P31689	DnaJ homolog subfamily A member 1 OS=Homo sapiens GN=DNAJA1 PE=1 SV=2	1.43	3.95	5.50	4.92	2.83	7.56	1.20	0.00	0.00	4.26	2.36	1.22	3.53	1.12	3.58	0.00	0.00	0.00	0.00	0.00	0.00	0.00	0.00	0.28	2.93	0.71	>0.999999999999999	4.1
O94979	Protein transport protein Sec31A OS=Homo sapiens GN=SEC31A PE=1 SV=3	0.41	0.34	2.39	1.71	1.51	3.78	1.37	0.45	0.68	0.37	1.57	0.65	1.18	0.00	1.91	0.84	1.01	0.00	0.36	0.00	0.00	0.40	0.71	0.20	1.27	0.55	>0.999999999999999	2.3
P60228	Eukaryotic translation initiation factor 3 subunit E OS=Homo sapiens GN=EIF3E PE=1 SV=1	1.22	1.03	4.78	4.28	3.77	1.42	1.03	0.00	0.00	1.11	4.25	0.49	0.00	0.96	3.10	0.00	0.00	0.00	0.00	0.00	0.00	0.00	3.83	0.00	1.95	0.66	>0.999999999999999	3.0
P51580	Thiopurine S-methyltransferase OS=Homo sapiens GN=TPMT PE=1 SV=1	2.45	2.06	2.87	2.57	2.26	0.00	0.00	0.00	0.00	0.00	1.89	0.00	0.00	0.00	0.00	0.00	0.00	0.00	0.00	0.00	0.00	0.96	0.00	0.00	1.17	0.08	>0.999999999999999	14.7
P19957	Elafin OS=Homo sapiens GN=PI3 PE=1 SV=3	5.51	0.00	0.00	0.00	0.00	0.00	0.00	2.44	3.70	0.00	4.25	0.00	0.00	0.00	0.00	4.54	0.00	0.00	4.88	7.92	4.56	4.87	3.83	0.00	1.32	2.55	>0.999999999999999	0.5
Q02818	Nucleobindin-1 OS=Homo sapiens GN=NUCB1 PE=1 SV=4	2.65	1.03	0.00	2.78	3.58	0.00	5.98	3.88	7.12	0.00	0.94	4.24	6.35	6.90	0.00	4.54	2.40	4.14	2.35	6.75	10.30	7.35	3.83	1.40	2.68	4.69	>0.999999999999999	0.6
O00534	von Willebrand factor A domain-containing protein 5A OS=Homo sapiens GN=VWA5A PE=2 SV=2	0.82	1.37	0.96	2.57	2.26	2.84	1.37	0.00	0.00	0.00	2.52	0.33	0.00	0.00	1.91	0.67	0.00	0.00	0.00	0.00	0.00	0.00	1.70	0.00	1.25	0.36	>0.999999999999999	3.5
O00203	AP-3 complex subunit beta-1 OS=Homo sapiens GN=AP3B1 PE=1 SV=3	0.61	0.00	0.72	1.07	0.94	0.71	0.85	0.00	0.00	0.00	0.47	0.00	0.00	0.00	0.00	0.00	0.00	0.00	0.54	0.00	0.51	0.00	0.43	0.20	0.45	0.14	>0.999999999999999	3.2
Q9ULV4	Coronin-1C OS=Homo sapiens GN=CORO1C PE=1 SV=1	2.65	0.00	0.00	1.28	0.00	1.42	1.03	0.00	0.82	1.11	2.99	1.06	0.00	0.00	1.43	1.01	0.00	0.00	0.00	0.00	0.00	1.04	0.00	0.24	1.03	0.31	>0.999999999999999	3.3
O95394	Phosphoacetylglucosamine mutase OS=Homo sapiens GN=PGM3 PE=1 SV=1	1.02	4.12	4.06	5.13	0.94	1.18	5.13	1.54	0.00	0.93	1.73	2.45	1.18	0.00	2.62	0.00	0.63	0.77	1.99	0.00	0.00	2.40	3.41	1.20	2.35	1.18	>0.999999999999999	2.0
Q9P2X3	Protein IMPACT OS=Homo sapiens GN=IMPACT PE=1 SV=3	1.84	0.00	2.15	0.00	0.00	0.00	0.00	0.00	0.00	0.00	0.00	0.00	0.00	0.00	0.00	0.00	0.00	0.00	0.00	0.00	0.00	0.00	0.00	0.00	0.33	0.00	>0.999999999999999	#DIV/0!
Q92616	Translational activator GCN1 OS=Homo sapiens GN=GCN1L1 PE=1 SV=6	0.20	0.00	1.67	1.92	0.19	0.71	0.17	0.00	0.00	0.19	0.16	0.49	0.00	0.00	0.72	0.00	0.00	0.00	0.00	0.00	0.00	0.00	0.43	0.04	0.48	0.10	>0.999999999999999	4.8
Q07812	Apoptosis regulator BAX OS=Homo sapiens GN=BAX PE=1 SV=1	3.26	0.00	3.82	3.42	3.02	3.78	0.00	1.44	0.00	2.96	2.52	1.31	0.00	0.00	3.82	0.00	0.00	0.00	0.00	0.00	0.00	1.28	2.27	0.64	2.13	0.67	>0.999999999999999	3.2
P68400	Casein kinase II subunit alpha OS=Homo sapiens GN=CSNK2A1 PE=1 SV=1	1.43	1.20	5.74	3.21	2.83	5.67	1.20	0.00	0.00	0.00	5.19	0.57	0.00	0.00	3.58	1.18	0.89	0.00	0.00	0.00	0.00	0.00	0.00	0.28	2.25	0.49	>0.999999999999999	4.6
Q9Y262	Eukaryotic translation initiation factor 3 subunit L OS=Homo sapiens GN=EIF3L PE=1 SV=1	1.02	0.86	5.02	5.77	6.22	4.96	2.56	0.00	0.68	1.85	0.00	0.41	0.00	1.61	6.44	0.84	0.63	0.00	0.00	0.00	0.00	0.00	1.42	0.40	3.15	0.94	>0.999999999999999	3.3
P59665	Neutrophil defensin 1 OS=Homo sapiens GN=DEFA1 PE=1 SV=1	6.73	0.00	0.00	0.00	0.00	0.00	0.00	6.86	0.00	0.00	0.00	0.00	0.00	0.00	0.00	0.00	0.00	0.00	0.00	4.28	12.83	0.00	0.00	0.00	1.13	1.43	>0.999999999999999	0.8
Q9Y5P4	Collagen type IV alpha-3-binding protein OS=Homo sapiens GN=COL4A3BP PE=1 SV=1	1.02	0.86	4.78	2.99	0.00	2.13	0.85	0.00	0.00	0.93	1.42	0.00	0.00	0.00	2.15	0.00	0.00	0.00	0.00	0.00	0.00	0.00	0.71	0.00	1.25	0.24	>0.999999999999999	5.2
P53041	Serine/threonine-protein phosphatase 5 OS=Homo sapiens GN=PPP5C PE=1 SV=1	1.22	0.00	2.87	2.57	2.26	4.25	2.05	0.54	0.00	3.33	3.93	0.49	0.00	0.96	2.86	1.01	0.00	0.00	0.00	0.00	0.00	2.00	2.55	0.00	1.96	0.78	>0.999999999999999	2.5
Q02539	Histone H1.1 OS=Homo sapiens GN=HIST1H1A PE=1 SV=3	3.06	0.00	0.00	0.00	0.00	3.54	0.00	0.00	0.00	0.00	0.00	0.00	3.53	2.41	0.00	0.00	0.00	0.00	0.00	0.00	0.00	1.20	0.00	0.00	0.55	0.59	>0.999999999999999	0.9
P62158	Calmodulin OS=Homo sapiens GN=CALM1 PE=1 SV=2	4.08	12.52	10.52	0.00	13.76	25.52	18.47	6.59	14.79	13.52	16.98	8.81	25.40	0.00	10.49	7.40	18.85	16.57	13.20	9.48	25.15	1.60	10.36	0.80	12.13	11.61	>0.999999999999999	1.0
P17980	26S protease regulatory subunit 6A OS=Homo sapiens GN=PSMC3 PE=1 SV=3	1.43	0.00	5.02	2.99	3.96	6.85	2.39	1.26	1.92	3.89	3.30	2.37	1.65	1.12	5.01	1.18	1.77	2.15	2.53	0.00	1.18	1.68	2.98	0.56	2.95	1.82	>0.999999999999999	1.6
Q9Y4E8	Ubiquitin carboxyl-terminal hydrolase 15 OS=Homo sapiens GN=USP15 PE=1 SV=3	0.61	0.51	1.43	0.00	0.00	0.71	0.51	0.00	0.00	0.00	0.00	0.00	0.00	0.00	0.00	0.00	0.00	0.00	0.00	0.00	0.00	0.24	0.43	0.12	0.32	0.07	>0.999999999999999	4.8
Q9Y3I0	tRNA-splicing ligase RtcB homolog OS=Homo sapiens GN=C22orf28 PE=1 SV=1	1.22	5.66	6.22	5.56	3.58	9.69	3.25	0.54	0.82	3.52	5.19	0.98	0.00	1.93	4.53	2.02	0.00	0.92	1.09	0.00	0.00	0.96	1.70	0.48	3.85	1.14	>0.999999999999999	3.4
P49902	Cytosolic purine 5'-nucleotidase OS=Homo sapiens GN=NT5C2 PE=1 SV=1	1.02	0.86	3.82	3.42	0.94	2.36	2.74	0.00	0.00	0.93	0.79	0.00	0.00	0.00	2.38	1.68	0.00	0.77	0.90	0.00	0.00	0.80	2.27	0.40	1.41	0.77	>0.999999999999999	1.8
P51812	Ribosomal protein S6 kinase alpha-3 OS=Homo sapiens GN=RPS6KA3 PE=1 SV=1	0.82	1.37	2.87	0.86	1.51	4.02	2.05	0.00	0.00	0.74	2.67	0.00	0.00	1.28	2.86	0.00	0.00	0.00	0.00	0.00	0.00	0.96	0.00	0.00	1.41	0.43	>0.999999999999999	3.3
Q9NWS1	PCNA-interacting partner OS=Homo sapiens GN=PARPBP PE=1 SV=3	1.02	0.86	1.20	1.07	0.94	1.18	0.85	0.45	0.68	0.93	0.79	0.41	1.18	0.80	1.19	0.84	0.63	0.77	0.90	0.65	0.84	0.00	0.71	0.20	0.86	0.73	>0.999999999999999	1.2
Q92900	Regulator of nonsense transcripts 1 OS=Homo sapiens GN=UPF1 PE=1 SV=2	0.61	0.00	1.91	0.64	0.94	1.18	0.51	0.00	0.00	0.93	1.26	0.65	0.00	0.00	0.00	0.00	0.00	0.00	0.00	0.00	0.00	0.24	0.00	0.12	0.74	0.03	>0.999999999999999	24.8
P22894	Neutrophil collagenase OS=Homo sapiens GN=MMP8 PE=1 SV=1	1.22	0.00	0.00	0.00	0.00	0.00	0.00	0.54	0.00	0.00	0.00	0.00	0.00	0.00	0.00	0.00	0.00	0.00	0.00	1.69	4.56	0.00	0.00	0.00	0.15	0.52	>0.999999999999999	0.3
Q96EP5	DAZ-associated protein 1 OS=Homo sapiens GN=DAZAP1 PE=1 SV=1	1.63	1.37	1.91	1.71	3.02	1.89	1.37	0.72	0.00	0.00	2.52	0.00	0.00	1.28	1.91	1.35	0.00	0.00	0.00	0.00	0.00	1.28	1.14	0.00	1.35	0.58	>0.999999999999999	2.3
P30622	CAP-Gly domain-containing linker protein 1 OS=Homo sapiens GN=CLIP1 PE=1 SV=2	0.41	0.69	0.96	0.86	0.00	0.00	0.34	0.00	0.00	0.00	0.31	0.00	0.00	0.00	0.48	0.00	0.00	0.00	0.00	0.00	0.00	0.00	0.00	0.08	0.30	0.05	>0.999999999999999	6.4
Q9NZD2	Glycolipid transfer protein OS=Homo sapiens GN=GLTP PE=1 SV=3	2.85	2.40	3.35	2.99	5.66	7.09	2.39	0.00	1.92	2.59	4.72	3.92	0.00	0.00	3.34	0.00	1.77	0.00	8.68	1.82	2.36	1.12	1.99	1.92	3.32	1.92	>0.999999999999999	1.7
P25774	Cathepsin S OS=Homo sapiens GN=CTSS PE=1 SV=3	1.84	0.00	0.00	1.92	0.00	0.00	1.54	1.63	0.00	0.00	0.00	1.47	2.12	0.00	4.29	1.51	0.00	0.00	1.63	1.17	0.00	2.24	2.55	0.36	0.70	1.32	>0.999999999999999	0.5
Q6NXT2	Histone H3.3C OS=Homo sapiens GN=H3F3C PE=1 SV=3	4.69	3.95	0.00	4.92	4.34	0.00	3.76	0.00	0.00	0.00	0.00	1.88	5.41	3.69	5.49	0.00	2.91	0.00	4.16	2.99	0.00	0.00	3.26	0.00	1.96	2.33	>0.999999999999999	0.8
O00584	Ribonuclease T2 OS=Homo sapiens GN=RNASET2 PE=1 SV=2	2.24	0.00	2.63	2.35	0.00	0.00	1.88	0.99	1.51	2.04	1.73	1.88	2.59	3.69	2.62	1.85	1.39	1.69	0.00	1.43	1.86	2.96	1.56	0.92	1.44	1.88	>0.999999999999999	0.8
P48163	NADP-dependent malic enzyme OS=Homo sapiens GN=ME1 PE=1 SV=1	1.02	6.00	6.69	2.14	7.73	3.78	2.74	1.99	0.68	4.08	3.46	1.31	0.00	3.53	5.25	3.70	2.02	0.00	2.89	0.00	0.84	3.28	5.82	0.88	3.47	2.35	>0.999999999999999	1.5
P07358	Complement component C8 beta chain OS=Homo sapiens GN=C8B PE=1 SV=3	1.02	1.72	2.39	2.14	1.89	1.18	1.71	0.90	2.05	1.85	0.00	1.71	0.00	2.41	3.58	5.38	2.66	2.30	4.70	2.73	3.54	0.80	0.71	0.40	1.55	2.43	>0.999999999999999	0.6
P24666	Low molecular weight phosphotyrosine protein phosphatase OS=Homo sapiens GN=ACP1 PE=1 SV=3	3.67	3.09	4.30	8.55	3.39	4.25	3.08	0.00	2.47	3.33	6.29	1.47	0.00	2.89	4.29	0.00	0.00	0.00	3.26	0.00	0.00	5.19	5.68	0.00	3.66	1.78	>0.999999999999999	2.1
Q9BRP8	Partner of Y14 and mago OS=Homo sapiens GN=WIBG PE=1 SV=1	3.06	2.57	3.59	0.00	0.00	3.54	0.00	0.00	2.05	0.00	0.00	2.61	0.00	0.00	0.00	0.00	1.90	0.00	0.00	0.00	0.00	2.56	4.54	1.28	1.45	0.86	>0.999999999999999	1.7
P14902	Indoleamine 2,3-dioxygenase 1 OS=Homo sapiens GN=IDO1 PE=1 SV=1	1.43	0.00	0.00	0.00	0.00	0.00	1.20	0.00	0.00	0.00	0.00	1.22	3.53	0.00	0.00	0.00	0.00	0.00	0.00	0.00	0.00	0.00	0.00	0.00	0.32	0.29	>0.999999999999999	1.1
P12955	Xaa-Pro dipeptidase OS=Homo sapiens GN=PEPD PE=1 SV=3	1.22	0.00	0.00	1.28	0.00	0.00	2.05	1.08	0.00	0.00	0.00	0.49	1.41	0.96	1.43	0.00	0.00	0.92	2.17	0.00	0.00	1.52	0.85	0.00	0.51	0.77	>0.999999999999999	0.7
Q9UBE0	SUMO-activating enzyme subunit 1 OS=Homo sapiens GN=SAE1 PE=1 SV=1	1.84	3.09	2.15	1.92	1.70	4.25	0.00	0.00	0.00	5.19	4.40	1.47	2.12	0.00	0.00	0.00	0.00	1.38	0.00	0.00	0.00	0.00	0.00	0.36	2.17	0.32	>0.999999999999999	6.7
Q01844	RNA-binding protein EWS OS=Homo sapiens GN=EWSR1 PE=1 SV=1	1.02	0.86	1.20	1.07	0.00	1.18	0.85	0.00	0.00	0.93	0.79	0.00	0.00	0.00	1.19	0.00	0.00	0.77	0.00	0.00	0.00	0.00	0.00	0.00	0.66	0.16	>0.999999999999999	4.0
Q15063	Periostin OS=Homo sapiens GN=POSTN PE=1 SV=2	0.61	0.00	0.72	0.00	5.09	0.00	0.00	0.27	5.62	0.00	0.00	0.24	0.00	0.00	0.72	3.20	4.56	0.00	0.54	0.00	1.18	0.00	2.13	0.00	1.05	1.03	>0.999999999999999	1.0
O43681	ATPase ASNA1 OS=Homo sapiens GN=ASNA1 PE=1 SV=2	1.63	3.09	9.08	3.85	3.39	4.25	0.00	0.72	0.00	3.33	5.98	1.47	0.00	0.00	9.06	1.35	0.00	1.23	0.00	0.00	0.00	3.04	3.97	0.72	3.07	1.61	>0.999999999999999	1.9
P52790	Hexokinase-3 OS=Homo sapiens GN=HK3 PE=1 SV=2	0.61	0.00	0.00	0.00	0.00	0.00	1.20	0.00	0.00	0.00	0.00	0.00	1.65	0.48	0.00	0.00	0.00	1.07	0.00	0.00	2.87	0.00	0.00	0.00	0.15	0.51	>0.999999999999999	0.3
Q9UMF0	Intercellular adhesion molecule 5 OS=Homo sapiens GN=ICAM5 PE=1 SV=3	0.61	0.00	0.72	0.64	0.57	0.00	0.51	0.00	0.41	0.56	0.00	0.24	0.71	0.48	0.00	0.00	0.38	0.00	0.00	0.00	0.00	0.00	0.00	0.12	0.36	0.14	>0.999999999999999	2.5
P28482	Mitogen-activated protein kinase 1 OS=Homo sapiens GN=MAPK1 PE=1 SV=3	1.63	2.74	13.86	1.71	3.02	10.87	4.45	0.00	0.00	2.96	4.09	0.65	0.00	0.00	8.59	0.00	0.00	1.23	0.00	0.00	0.00	2.08	2.27	0.32	3.83	1.21	>0.999999999999999	3.2
Q03591	Complement factor H-related protein 1 OS=Homo sapiens GN=CFHR1 PE=1 SV=2	1.84	0.00	0.00	0.00	0.00	0.00	0.00	1.63	0.00	0.00	0.00	0.00	0.00	0.00	2.15	1.51	0.00	0.00	7.05	0.00	0.00	0.00	0.00	0.00	0.29	0.89	>0.999999999999999	0.3
P00488	Coagulation factor XIII A chain OS=Homo sapiens GN=F13A1 PE=1 SV=4	0.82	0.00	0.00	0.00	1.51	0.00	0.00	0.00	0.00	0.00	0.00	0.00	0.00	0.00	0.00	0.67	0.00	0.00	0.00	0.00	0.00	0.00	0.00	0.00	0.19	0.06	>0.999999999999999	3.5
P61221	ATP-binding cassette sub-family E member 1 OS=Homo sapiens GN=ABCE1 PE=1 SV=1	1.02	0.86	2.39	1.07	2.83	2.36	2.56	0.00	0.68	1.85	0.79	0.41	1.18	0.00	1.19	0.00	0.00	1.53	0.00	0.00	0.00	0.00	1.42	0.00	1.40	0.44	>0.999999999999999	3.2
Q8WXF1	Paraspeckle component 1 OS=Homo sapiens GN=PSPC1 PE=1 SV=1	1.22	1.03	1.43	1.28	1.13	1.42	0.00	0.00	0.00	2.04	1.73	0.49	0.00	0.00	2.62	0.00	0.00	0.00	0.00	0.00	0.00	0.00	0.00	0.00	0.98	0.22	>0.999999999999999	4.5
Q9HA64	Ketosamine-3-kinase OS=Homo sapiens GN=FN3KRP PE=1 SV=2	2.04	0.00	2.39	0.00	0.00	0.00	0.00	0.00	0.00	0.00	0.00	0.00	0.00	0.00	0.00	0.00	0.00	0.00	0.00	0.00	0.00	0.00	0.00	0.00	0.37	0.00	>0.999999999999999	#DIV/0!
Q13263	Transcription intermediary factor 1-beta OS=Homo sapiens GN=TRIM28 PE=1 SV=5	0.82	1.89	3.59	1.50	2.07	3.54	1.20	0.36	0.00	2.78	2.36	0.57	0.00	1.12	0.00	0.00	0.00	0.61	0.00	0.00	0.00	0.56	0.57	0.16	1.72	0.25	>0.999999999999999	6.8
P04433	Ig kappa chain V-III region VG (Fragment) OS=Homo sapiens PE=1 SV=1	5.51	4.63	0.00	5.77	5.09	0.00	4.62	2.44	0.00	0.00	0.00	2.20	6.35	4.34	6.44	4.54	3.42	4.14	4.88	3.51	4.56	2.16	3.83	0.00	2.52	4.01	>0.999999999999999	0.6
P40926	Malate dehydrogenase, mitochondrial OS=Homo sapiens GN=MDH2 PE=1 SV=3	1.84	5.15	0.00	6.41	0.00	4.49	0.00	4.97	0.00	1.67	4.72	6.93	2.12	8.83	2.15	7.07	1.14	2.92	12.66	1.17	3.21	1.52	4.26	3.40	3.01	4.20	>0.999999999999999	0.7
P53999	Activated RNA polymerase II transcriptional coactivator p15 OS=Homo sapiens GN=SUB1 PE=1 SV=3	4.89	0.00	0.00	0.00	4.53	5.67	0.00	2.17	0.00	4.45	3.77	0.00	0.00	0.00	5.72	0.00	3.04	0.00	0.00	0.00	0.00	1.92	0.00	0.00	2.12	0.89	>0.999999999999999	2.4
P62244	40S ribosomal protein S15a OS=Homo sapiens GN=RPS15A PE=1 SV=2	4.69	0.00	0.00	0.00	0.00	5.67	0.00	2.08	3.15	0.00	0.00	0.00	0.00	0.00	0.00	0.00	0.00	0.00	0.00	0.00	0.00	0.00	3.26	0.00	1.30	0.27	>0.999999999999999	4.8
O15027	Protein transport protein Sec16A OS=Homo sapiens GN=SEC16A PE=1 SV=3	0.20	0.17	0.00	0.00	0.00	0.00	0.00	0.00	0.00	0.00	0.47	0.00	0.00	0.00	0.00	0.00	0.00	0.00	0.00	0.00	0.00	0.00	0.00	0.00	0.07	0.00	>0.999999999999999	#DIV/0!
Q7Z4H3	HD domain-containing protein 2 OS=Homo sapiens GN=HDDC2 PE=1 SV=1	2.85	2.40	7.17	2.99	0.00	7.09	0.00	0.00	1.92	2.59	2.20	1.14	0.00	0.00	0.00	0.00	0.00	0.00	2.53	0.00	0.00	1.12	1.99	0.00	2.53	0.47	>0.999999999999999	5.4
Q93009	Ubiquitin carboxyl-terminal hydrolase 7 OS=Homo sapiens GN=USP7 PE=1 SV=2	0.61	0.51	1.91	0.64	0.00	1.89	0.00	0.00	0.00	0.93	0.79	0.00	0.00	0.00	0.72	0.00	0.00	0.00	0.00	0.00	0.00	0.24	0.00	0.12	0.61	0.09	>0.999999999999999	6.8
Q8TBC4	NEDD8-activating enzyme E1 catalytic subunit OS=Homo sapiens GN=UBA3 PE=1 SV=2	1.22	1.03	8.61	5.99	1.13	4.73	2.22	0.00	0.00	0.00	0.94	1.63	0.00	0.00	1.43	3.36	0.00	0.00	1.09	0.00	0.00	1.04	3.97	0.80	2.29	0.97	>0.999999999999999	2.4
Q9HC84	Mucin-5B OS=Homo sapiens GN=MUC5B PE=1 SV=3	0.20	2.06	0.00	0.00	0.75	0.00	0.00	0.09	0.00	0.74	0.00	0.08	1.18	1.45	0.00	0.00	0.00	0.15	0.00	3.12	0.00	0.00	0.00	0.44	0.33	0.53	>0.999999999999999	0.6
Q9NR31	GTP-binding protein SAR1a OS=Homo sapiens GN=SAR1A PE=1 SV=1	3.06	0.00	0.00	0.00	2.83	0.00	0.00	2.89	2.05	0.00	2.36	0.00	3.53	0.00	0.00	0.00	0.00	0.00	2.71	0.00	0.00	0.00	2.13	0.00	1.10	0.70	>0.999999999999999	1.6
Q9H993	UPF0364 protein C6orf211 OS=Homo sapiens GN=C6orf211 PE=1 SV=1	1.22	1.03	1.43	1.28	1.13	3.07	1.03	0.54	0.00	0.00	0.94	0.49	0.00	0.00	0.00	0.00	0.00	0.00	1.09	0.00	0.00	0.48	0.00	0.00	1.01	0.13	>0.999999999999999	7.8
P61923	Coatomer subunit zeta-1 OS=Homo sapiens GN=COPZ1 PE=1 SV=1	3.47	0.00	8.61	3.63	11.12	13.94	0.00	0.00	0.00	0.00	2.67	1.39	0.00	0.00	8.59	0.00	0.00	0.00	0.00	0.00	0.00	0.00	2.41	0.68	3.74	0.97	>0.999999999999999	3.8
Q53EL6	Programmed cell death protein 4 OS=Homo sapiens GN=PDCD4 PE=1 SV=2	1.22	4.80	8.61	4.28	2.45	4.73	0.00	0.00	0.00	2.41	3.15	1.06	0.00	0.96	1.43	3.36	0.00	0.92	0.00	0.00	0.00	3.59	2.84	0.52	2.72	1.14	>0.999999999999999	2.4
Q9UNX3	60S ribosomal protein L26-like 1 OS=Homo sapiens GN=RPL26L1 PE=1 SV=1	4.08	0.00	4.78	4.28	3.77	4.73	3.42	0.00	2.74	3.71	3.15	0.00	0.00	3.21	4.77	3.36	2.53	0.00	0.00	0.00	0.00	1.60	2.84	0.80	2.89	1.59	>0.999999999999999	1.8
P02814	Submaxillary gland androgen-regulated protein 3B OS=Homo sapiens GN=SMR3B PE=1 SV=2	8.77	0.00	0.00	0.00	0.00	0.00	0.00	0.00	0.00	0.00	0.00	3.51	44.92	0.00	0.00	0.00	0.00	0.00	0.00	0.00	0.00	0.00	0.00	4.16	1.02	4.09	>0.999999999999999	0.3
Q86V81	THO complex subunit 4 OS=Homo sapiens GN=ALYREF PE=1 SV=3	2.45	0.00	0.00	0.00	0.00	2.84	0.00	0.00	0.00	0.00	1.89	0.00	0.00	0.00	0.00	0.00	0.00	0.00	0.00	0.00	0.00	0.00	0.00	0.00	0.60	0.00	>0.999999999999999	#DIV/0!
Q96HN2	Putative adenosylhomocysteinase 3 OS=Homo sapiens GN=AHCYL2 PE=1 SV=1	1.02	0.00	0.00	4.49	0.00	7.80	1.71	0.45	0.00	3.89	3.30	0.00	0.00	1.61	0.00	0.00	0.63	0.00	1.81	0.00	0.00	0.40	2.13	0.00	1.89	0.55	>0.999999999999999	3.4
A6NJ16	Putative V-set and immunoglobulin domain-containing protein 6 OS=Homo sapiens GN=VSIG6 PE=5 SV=2	5.10	0.00	0.00	0.00	4.71	0.00	0.00	2.26	0.00	0.00	0.00	0.00	5.88	4.01	0.00	0.00	0.00	3.84	0.00	0.00	0.00	0.00	3.55	0.00	1.01	1.44	>0.999999999999999	0.7
Q99622	Protein C10 OS=Homo sapiens GN=C12orf57 PE=1 SV=1	5.30	0.00	0.00	0.00	0.00	0.00	0.00	0.00	0.00	0.00	0.00	2.12	0.00	0.00	0.00	0.00	0.00	0.00	0.00	0.00	0.00	0.00	0.00	0.00	0.62	0.00	>0.999999999999999	#DIV/0!
P04899	Guanine nucleotide-binding protein G(i) subunit alpha-2 OS=Homo sapiens GN=GNAI2 PE=1 SV=3	1.63	0.00	0.00	0.00	0.00	0.00	0.00	0.00	0.00	0.00	0.00	0.00	0.00	0.00	0.00	1.35	0.00	0.00	0.00	0.00	0.00	0.00	0.00	0.00	0.14	0.11	>0.999999999999999	1.2
Q9Y295	Developmentally-regulated GTP-binding protein 1 OS=Homo sapiens GN=DRG1 PE=1 SV=1	1.63	0.00	0.00	0.00	1.51	1.89	0.00	0.00	0.00	0.00	2.67	0.65	0.00	0.00	0.00	0.00	0.00	0.00	0.00	0.00	0.00	0.00	1.14	0.00	0.70	0.09	>0.999999999999999	7.4
O75608	Acyl-protein thioesterase 1 OS=Homo sapiens GN=LYPLA1 PE=1 SV=1	2.65	2.23	6.93	6.20	5.47	6.85	0.00	0.00	0.00	5.37	4.56	0.00	0.00	0.00	3.10	2.19	1.64	4.45	0.00	0.00	0.00	1.04	6.53	0.52	3.36	1.62	>0.999999999999999	2.1
P06753	Tropomyosin alpha-3 chain OS=Homo sapiens GN=TPM3 PE=1 SV=1	2.04	3.60	2.39	2.14	3.96	7.80	1.71	0.90	1.37	0.00	7.39	3.83	2.35	1.61	5.01	1.68	1.27	1.53	3.80	2.73	1.69	0.80	1.42	0.84	3.09	2.06	>0.999999999999999	1.5
Q8NFU3	Thiosulfate sulfurtransferase/rhodanese-like domain-containing protein 1 OS=Homo sapiens GN=TSTD1 PE=1 SV=(	5.51	0.00	0.00	0.00	0.00	6.38	4.62	0.00	3.70	5.00	4.25	0.00	0.00	0.00	0.00	0.00	0.00	0.00	0.00	0.00	0.00	0.00	0.00	0.00	2.45	0.00	>0.999999999999999	#DIV/0!
O95372	Acyl-protein thioesterase 2 OS=Homo sapiens GN=LYPLA2 PE=1 SV=1	2.65	0.00	0.00	0.00	0.00	0.00	0.00	0.00	1.78	0.00	0.00	1.06	0.00	0.00	0.00	2.19	1.64	1.99	0.00	0.00	0.00	0.00	0.00	0.52	0.46	0.53	>0.999999999999999	0.9
P19838	Nuclear factor NF-kappa-B p105 subunit OS=Homo sapiens GN=NFKB1 PE=1 SV=2	0.61	0.00	0.72	0.00	0.00	0.71	0.00	0.00	0.00	0.00	0.47	0.00	0.00	0.00	0.00	0.00	0.00	0.00	0.00	0.00	0.00	0.00	0.00	0.00	0.21	0.00	>0.999999999999999	#DIV/0!

P27216	Annexin A13 OS=Homo sapiens GN=ANXA13 PE=1 SV=3	1.84	1.54	0.00	0.00	1.70	4.49	0.00	0.00	1.23	1.67	1.42	0.73	0.00	0.00	2.15	1.51	0.00	0.00	0.00	0.00	0.00	0.72	0.00	0.00	1.22	0.36	>0.999999999999999	3.3
Q13492	Phosphatidylinositol-binding clathrin assembly protein OS=Homo sapiens GN=PICALM PE=1 SV=2	1.84	1.54	3.59	3.21	1.70	3.54	1.54	0.81	0.00	0.00	1.42	0.73	2.12	0.00	2.15	0.84	0.00	0.00	0.90	0.00	1.52	1.20	2.13	0.60	1.66	0.95	>0.999999999999999	1.7
P06311	Ig kappa chain V-III region IARC/BL41 OS=Homo sapiens PE=1 SV=1	4.89	0.00	0.00	0.00	0.00	0.00	0.00	0.00	0.00	0.00	0.00	1.96	0.00	0.00	0.00	0.00	0.00	0.00	0.00	0.00	0.00	1.92	0.00	0.00	0.57	0.16	>0.999999999999999	3.6
Q9UK76	Hematological and neurological expressed 1 protein OS=Homo sapiens GN=HN1 PE=1 SV=3	4.28	0.00	0.00	0.00	14.71	0.00	0.00	0.00	2.88	0.00	3.30	3.83	0.00	0.00	5.01	0.00	0.00	0.00	0.00	0.00	0.00	3.75	0.00	0.84	2.42	0.80	>0.999999999999999	3.0
O60493	Sorting nexin-3 OS=Homo sapiens GN=SNX3 PE=1 SV=3	3.67	3.09	4.30	8.34	7.35	4.25	3.08	0.00	0.00	3.33	6.13	1.47	0.00	2.89	0.00	0.00	2.28	0.00	3.26	0.00	0.00	1.44	2.55	0.72	3.75	1.09	>0.999999999999999	3.4
Q9BXX3	Ankyrin repeat domain-containing protein 30A OS=Homo sapiens GN=ANKRD30A PE=1 SV=3	0.41	0.00	0.48	0.43	0.38	0.47	0.34	0.18	0.27	0.37	0.31	0.00	0.47	0.32	0.48	0.34	0.25	0.00	0.36	0.00	0.00	0.16	0.28	0.00	0.30	0.22	>0.999999999999999	1.4
Q9Y2A7	Nck-associated protein 1 OS=Homo sapiens GN=NCKAP1 PE=1 SV=1	0.61	0.86	1.20	1.07	0.00	3.07	0.85	0.00	0.00	0.56	0.47	0.41	0.00	0.00	0.72	0.00	0.00	0.00	0.00	0.00	0.00	0.00	0.43	0.20	0.76	0.11	>0.999999999999999	6.8
P61576	HERV-K_5q13.3 provirus Rec protein OS=Homo sapiens PE=1 SV=1	5.91	4.97	6.93	6.20	5.47	6.85	4.96	2.62	3.97	5.37	4.56	2.37	6.82	4.66	6.92	4.88	3.67	4.45	5.25	3.76	4.89	2.32	4.12	1.16	5.02	4.41	>0.999999999999999	1.1
Q7Z4V5	Hepatoma-derived growth factor-related protein 2 OS=Homo sapiens GN=HDGFRP2 PE=1 SV=1	0.82	0.00	0.96	0.00	0.75	3.31	0.00	0.00	0.00	0.74	0.63	0.00	0.00	0.00	0.00	0.00	0.00	0.00	0.00	0.00	0.00	0.00	0.00	0.00	0.60	0.00	>0.999999999999999	#DIV/0!
P82979	SAP domain-containing ribonucleoprotein OS=Homo sapiens GN=SARNP PE=1 SV=3	2.85	2.40	0.00	6.41	2.64	7.09	2.39	1.26	0.00	2.59	7.55	1.14	0.00	0.00	3.34	2.36	0.00	4.60	2.53	0.00	0.00	1.12	6.81	0.56	3.03	1.78	>0.999999999999999	1.7
O00507	Probable ubiquitin carboxyl-terminal hydrolase FAF-Y OS=Homo sapiens GN=USP9Y PE=1 SV=2	0.20	0.00	0.00	0.00	0.00	0.00	0.00	0.00	0.00	0.19	0.00	0.00	0.00	0.00	0.00	0.00	0.00	0.00	0.00	0.00	0.00	0.00	0.00	0.00	0.03	0.00	>0.999999999999999	#DIV/0!
P62906	60S ribosomal protein L10a OS=Homo sapiens GN=RPL10A PE=1 SV=2	2.65	2.23	3.11	2.78	8.67	10.87	4.96	0.00	1.78	2.41	2.04	1.06	0.00	2.09	0.00	2.19	0.00	0.00	2.35	0.00	0.00	2.32	1.84	0.52	3.55	0.94	>0.999999999999999	3.8
Q9GZZ9	Ubiquitin-like modifier-activating enzyme 5 OS=Homo sapiens GN=UBA5 PE=1 SV=1	1.43	0.00	3.59	0.00	0.00	0.00	0.00	0.00	0.00	0.00	0.00	1.96	0.00	0.00	1.67	0.00	0.00	0.00	0.00	0.00	0.00	0.00	0.00	0.96	0.58	0.22	>0.999999999999999	2.7
O43278	Kunitz-type protease inhibitor 1 OS=Homo sapiens GN=SPINT1 PE=1 SV=2	1.02	0.00	0.00	0.00	0.00	0.00	0.00	0.00	0.00	0.00	0.00	0.00	0.00	0.00	0.00	0.00	0.00	0.00	0.00	0.00	0.00	0.00	0.00	0.00	0.08	0.00	>0.999999999999999	#DIV/0!
Q9BU76	Multiple myeloma tumor-associated protein 2 OS=Homo sapiens GN=MMTAG2 PE=1 SV=1	2.24	0.00	0.00	0.00	0.00	0.00	0.00	0.00	0.00	0.00	0.00	0.00	0.00	0.00	0.00	0.00	0.00	0.00	0.00	0.00	0.00	0.00	0.00	0.00	0.19	0.00	>0.999999999999999	#DIV/0!
P30566	Adenylosuccinate lyase OS=Homo sapiens GN=ADSL PE=1 SV=2	1.22	1.03	4.54	5.56	2.26	6.14	1.03	0.00	0.00	1.11	2.99	0.49	0.00	0.00	4.53	1.01	0.00	1.84	1.09	0.00	0.00	0.48	0.85	0.00	2.20	0.82	>0.999999999999999	2.7
P18510	Interleukin-1 receptor antagonist protein OS=Homo sapiens GN=IL1RN PE=1 SV=1	3.26	6.18	8.61	7.70	0.00	8.51	9.92	1.44	0.00	6.67	0.00	1.31	0.00	2.57	0.00	6.06	2.02	5.52	2.89	4.67	2.70	2.88	2.27	1.44	4.47	2.75	>0.999999999999999	1.6
P09238	Stromelysin-2 OS=Homo sapiens GN=MMP10 PE=1 SV=1	1.22	0.00	2.87	5.77	0.00	1.42	2.05	0.54	0.00	0.00	0.94	0.00	0.00	0.00	0.00	0.00	0.00	0.00	0.00	0.00	0.00	2.16	0.00	0.00	1.23	0.18	>0.999999999999999	6.9
Q13546	Receptor-interacting serine/threonine-protein kinase 1 OS=Homo sapiens GN=RIPK1 PE=1 SV=3	0.82	0.00	0.96	0.00	0.00	0.00	0.00	0.00	0.00	0.00	0.00	0.00	0.00	0.00	0.00	0.00	0.00	0.00	0.00	0.00	0.00	0.00	0.00	0.00	0.15	0.00	>0.999999999999999	#DIV/0!
P01604	Ig kappa chain V-I region Kue OS=Homo sapiens PE=1 SV=1	5.71	0.00	0.00	0.00	0.00	0.00	0.00	0.00	0.00	0.00	0.00	0.00	6.59	4.50	0.00	4.71	3.54	0.00	0.00	0.00	0.00	0.00	0.00	0.00	0.48	1.61	>0.999999999999999	0.3
P51570	Galactokinase OS=Homo sapiens GN=GALK1 PE=1 SV=1	1.63	0.00	1.91	0.00	0.00	1.89	0.00	0.00	0.00	0.00	1.26	0.00	0.00	0.00	0.00	0.00	0.00	0.00	0.00	0.00	0.00	0.00	0.00	0.00	0.56	0.00	>0.999999999999999	#DIV/0!
P50570	Dynamin-2 OS=Homo sapiens GN=DNM2 PE=1 SV=2	0.61	0.51	0.72	2.14	1.32	0.71	0.51	0.00	0.00	1.30	2.20	0.24	0.00	0.00	0.00	0.00	0.00	0.46	0.54	0.00	0.00	0.00	0.43	0.12	0.86	0.13	>0.999999999999999	6.6
P01625	Ig kappa chain V-IV region Len OS=Homo sapiens PE=1 SV=2	5.51	0.00	0.00	0.00	0.00	0.00	0.00	0.00	0.00	0.00	0.00	0.00	0.00	0.00	0.00	0.00	0.00	0.00	0.00	3.51	4.56	0.00	3.83	0.00	0.46	0.99	>0.999999999999999	0.5
P24821	Tenascin OS=Homo sapiens GN=TNC PE=1 SV=3	0.20	0.00	0.00	0.64	0.19	0.24	0.17	0.00	0.00	0.19	0.00	0.00	0.24	0.16	0.24	0.17	0.13	0.00	0.00	0.00	0.00	0.08	0.00	0.00	0.14	0.08	>0.999999999999999	1.6
Q9C0F3	Zinc finger protein 436 OS=Homo sapiens GN=ZNF436 PE=2 SV=2	1.22	1.03	0.00	1.28	0.00	0.00	0.00	0.00	0.00	0.00	0.94	0.00	0.00	0.00	0.00	0.00	0.00	0.00	1.09	0.00	0.00	0.00	0.00	0.24	0.37	0.11	>0.999999999999999	3.4
O75592	Probable E3 ubiquitin-protein ligase MYCBP2 OS=Homo sapiens GN=MYCBP2 PE=1 SV=3	0.20	0.17	0.00	0.00	0.00	0.00	0.00	0.09	0.14	0.00	0.00	0.00	0.24	0.16	0.00	0.00	0.00	0.15	0.18	0.00	0.00	0.08	0.14	0.00	0.05	0.08	>0.999999999999999	0.6
Q9HC38	Glyoxalase domain-containing protein 4 OS=Homo sapiens GN=GLOD4 PE=1 SV=1	1.84	7.38	10.28	15.39	5.85	7.33	3.42	1.81	2.74	5.74	8.96	2.53	7.29	4.98	10.26	3.36	2.53	3.07	7.78	0.00	1.52	7.03	8.09	0.80	6.10	4.73	>0.999999999999999	1.3
Q6ZTZ1	Uncharacterized protein C4orf44 OS=Homo sapiens GN=C4orf44 PE=2 SV=2	2.24	0.00	0.00	0.00	0.00	0.00	0.00	0.00	0.00	0.00	0.00	0.00	0.00	0.00	2.62	0.00	0.00	0.00	0.00	0.00	0.00	0.00	0.00	0.00	0.19	0.22	>0.999999999999999	0.9
P08697	Alpha-2-antiplasmin OS=Homo sapiens GN=SERPINF2 PE=1 SV=3	1.22	1.03	2.87	5.56	4.90	4.49	1.03	3.07	2.60	3.52	4.09	1.55	1.41	1.93	8.11	5.72	3.29	5.22	3.44	3.38	2.03	2.08	1.70	0.00	2.99	3.19	>0.999999999999999	0.9
Q9Y376	Calcium-binding protein 39 OS=Homo sapiens GN=CAB39 PE=1 SV=1	1.63	0.00	0.00	0.00	0.00	4.02	0.00	0.00	0.00	0.00	0.00	0.65	0.00	0.00	4.05	0.00	0.00	0.00	0.00	0.00	0.00	0.00	2.41	0.00	0.53	0.54	>0.999999999999999	1.0
Q9H446	RWD domain-containing protein 1 OS=Homo sapiens GN=RWDD1 PE=1 SV=1	2.45	0.00	5.98	2.57	2.26	2.84	0.00	0.00	0.00	2.22	0.00	0.98	0.00	1.93	2.86	2.02	0.00	0.00	0.00	0.00	0.00	0.00	0.00	0.48	1.61	0.61	>0.999999999999999	2.6
Q9BY44	Eukaryotic translation initiation factor 2A OS=Homo sapiens GN=EIF2A PE=1 SV=3	1.02	0.00	1.20	2.14	1.89	3.78	0.00	0.00	0.00	0.00	2.52	1.31	0.00	0.00	1.19	0.00	0.00	0.00	0.00	0.00	0.00	0.00	0.71	0.20	1.15	0.18	>0.999999999999999	6.6
P19021	Peptidyl-glycine alpha-amidating monooxygenase OS=Homo sapiens GN=PAM PE=1 SV=2	0.61	0.51	1.43	0.00	0.00	0.00	0.00	0.00	0.41	0.56	0.00	0.24	0.00	0.00	0.72	0.00	0.00	0.00	0.00	0.00	0.00	0.48	0.43	0.24	0.31	0.16	>0.999999999999999	2.0
P60510	Serine/threonine-protein phosphatase 4 catalytic subunit OS=Homo sapiens GN=PPP4C PE=1 SV=1	1.84	0.00	0.00	1.92	1.70	7.33	0.00	0.81	0.00	0.00	1.42	0.00	0.00	1.45	4.53	0.00	1.14	1.38	1.63	0.00	1.52	0.00	0.00	0.00	1.25	0.97	>0.999999999999999	1.3
O15372	Eukaryotic translation initiation factor 3 subunit H OS=Homo sapiens GN=EIF3H PE=1 SV=1	1.63	0.00	1.91	1.71	1.51	4.02	0.00	0.00	0.00	1.48	4.25	0.65	0.00	0.00	0.00	0.00	2.15	0.00	0.00	0.00	0.00	0.00	0.00	0.32	1.43	0.21	>0.999999999999999	6.9
P55160	Nck-associated protein 1-like OS=Homo sapiens GN=NCKAP1L PE=1 SV=3	0.61	0.00	0.00	0.00	0.00	0.00	0.00	0.00	0.00	0.00	0.00	0.00	0.00	0.00	0.00	0.00	0.00	0.00	0.00	0.00	0.00	0.00	0.00	0.00	0.05	0.00	>0.999999999999999	#DIV/0!
Q14232	Translation initiation factor eIF-2B subunit alpha OS=Homo sapiens GN=EIF2B1 PE=1 SV=1	2.04	1.72	2.39	2.14	1.89	2.36	0.00	0.00	0.00	0.00	0.00	0.00	0.00	0.00	0.00	0.00	0.00	0.00	0.00	0.00	0.00	0.00	0.00	0.00	1.04	0.00	>0.999999999999999	#DIV/0!
Q9BQA1	Methylosome protein 50 OS=Homo sapiens GN=WDR77 PE=1 SV=1	1.84	1.54	2.15	1.92	3.39	4.25	1.54	0.00	0.00	1.67	2.83	0.73	0.00	0.00	2.15	0.00	0.00	0.00	0.00	0.00	0.00	0.72	1.28	0.00	1.82	0.35	>0.999999999999999	5.3
Q9UQ88	Cyclin-dependent kinase 11A OS=Homo sapiens GN=CDK11A PE=1 SV=4	0.82	0.00	0.00	0.00	0.00	0.00	0.00	0.00	0.00	0.00	0.00	0.00	0.00	0.00	0.00	0.67	0.00	0.00	0.00	0.00	0.00	0.00	0.00	0.00	0.07	0.06	>0.999999999999999	1.2
O76071	Probable cytosolic iron-sulfur protein assembly protein CIAO1 OS=Homo sapiens GN=CIAO1 PE=1 SV=1	1.84	0.00	2.15	1.92	0.00	0.00	0.00	0.00	0.00	0.00	0.00	0.00	0.00	0.00	0.00	0.00	0.00	0.00	0.00	0.00	0.00	0.00	0.00	0.00	0.49	0.00	>0.999999999999999	#DIV/0!
P31942	Heterogeneous nuclear ribonucleoprotein H3 OS=Homo sapiens GN=HNRNPH3 PE=1 SV=2	1.84	0.00	4.54	0.00	0.00	4.49	3.25	0.81	0.00	3.52	6.45	0.00	0.00	0.00	0.00	0.00	0.00	0.00	3.44	0.00	0.00	0.00	0.00	0.00	2.07	0.29	>0.999999999999999	7.2
P29218	Inositol monophosphatase 1 OS=Homo sapiens GN=IMPA1 PE=1 SV=1	2.24	11.66	16.26	10.90	9.62	12.05	6.16	3.25	0.00	12.60	10.69	2.94	0.00	10.92	16.22	8.58	0.00	3.53	4.16	0.00	1.86	4.07	12.20	0.92	8.20	5.20	>0.999999999999999	1.6
O14744	Protein arginine N-methyltransferase 5 OS=Homo sapiens GN=PRMT5 PE=1 SV=4	0.82	2.40	7.17	4.06	2.64	3.31	0.68	0.00	0.00	2.59	1.42	0.33	0.00	0.00	2.15	0.67	0.00	0.00	0.72	0.00	0.00	1.12	1.28	0.00	2.12	0.49	>0.999999999999999	4.3
P01702	Ig lambda chain V-I region NIG-64 OS=Homo sapiens PE=1 SV=1	6.12	0.00	0.00	0.00	13.01	0.00	0.00	0.00	4.11	0.00	0.00	0.00	0.00	4.82	0.00	5.05	8.73	0.00	5.43	0.00	0.00	2.40	4.26	0.00	1.94	2.56	>0.999999999999999	0.8
P25685	DnaJ homolog subfamily B member 1 OS=Homo sapiens GN=DNAJB1 PE=1 SV=4	1.84	1.54	2.15	1.92	1.70	2.13	0.00	0.00	0.00	1.67	1.42	0.73	0.00	0.00	0.00	1.51	0.00	0.00	0.00	0.00	0.00	0.72	1.28	0.00	1.26	0.29	>0.999999999999999	4.3
Q9UBC2	Epidermal growth factor receptor substrate 15-like 1 OS=Homo sapiens GN=EPS15L1 PE=1 SV=1	0.61	0.51	1.67	1.50	0.57	3.54	0.00	0.00	0.00	1.30	2.36	0.57	0.00	0.00	1.67	0.00	0.00	0.00	0.00	0.00	0.00	0.00	0.43	0.00	1.05	0.17	>0.999999999999999	6.0
Q9HAB8	Phosphopantothenate--cysteine ligase OS=Homo sapiens GN=PPCS PE=1 SV=2	2.04	1.72	2.39	4.28	1.89	2.36	5.47	0.90	0.00	3.71	5.03	0.82	0.00	1.61	7.63	1.68	1.27	1.53	5.79	0.00	1.69	0.00	2.84	0.40	2.55	2.04	>0.999999999999999	1.3
Q9Y6K5	2'-5'-oligoadenylate synthase 3 OS=Homo sapiens GN=OAS3 PE=1 SV=3	0.61	0.00	0.00	0.64	0.00	0.00	0.00	0.00	0.00	0.00	0.00	0.24	0.00	0.00	0.72	0.00	0.00	0.00	0.00	0.00	0.00	0.24	0.00	0.12	0.12	0.09	>0.999999999999999	1.4
P07996	Thrombospondin-1 OS=Homo sapiens GN=THBS1 PE=1 SV=2	0.41	2.23	0.00	0.00	0.00	0.00	0.00	0.00	0.00	0.00	0.00	0.16	0.00	3.37	0.00	0.00	1.01	0.31	0.36	0.26	0.84	0.00	0.71	0.20	0.23	0.59	>0.999999999999999	0.4
Q92831	Histone acetyltransferase KAT2B OS=Homo sapiens GN=KAT2B PE=1 SV=3	0.61	0.00	0.72	0.00	0.00	0.71	0.00	0.00	0.00	0.00	0.00	0.00	0.00	0.48	0.00	0.00	0.00	0.00	0.00	0.00	0.00	0.00	0.00	0.00	0.17	0.04	>0.999999999999999	4.2
P01765	Ig heavy chain V-III region TIL OS=Homo sapiens PE=1 SV=1	5.71	10.81	6.69	0.00	5.28	6.62	4.79	2.53	8.63	5.19	0.00	2.28	0.00	4.50	6.68	10.60	7.97	4.30	11.39	8.18	18.23	5.03	8.94	1.12	4.88	7.24	>0.999999999999999	0.7
Q96GA7	Serine dehydratase-like OS=Homo sapiens GN=SDSL PE=1 SV=1	1.84	0.00	2.15	1.92	0.00	2.13	1.54	0.00	0.00	0.00	1.42	0.00	0.00	0.00	2.15	0.00	0.00	0.00	0.00	0.00	0.00	0.00	0.00	0.36	0.92	0.21	>0.999999999999999	4.4
P36507	Dual specificity mitogen-activated protein kinase kinase 2 OS=Homo sapiens GN=MAP2K2 PE=1 SV=1	1.43	2.57	3.59	0.00	0.00	1.65	1.20	0.00	0.00	2.78	1.10	0.00	0.00	1.12	1.67	1.18	0.00	0.00	0.00	0.00	0.00	0.56	0.99	0.28	1.19	0.48	>0.999999999999999	2.5
Q13740	CD166 antigen OS=Homo sapiens GN=ALCAM PE=1 SV=2	1.02	0.86	5.26	2.14	1.89	5.20	0.85	0.00	1.37	1.85	4.40	1.31	0.00	0.00	3.82	0.00	0.00	0.00	0.00	0.00	0.00	1.28	2.27	0.00	2.18	0.61	>0.999999999999999	3.6
Q02750	Dual specificity mitogen-activated protein kinase kinase 1 OS=Homo sapiens GN=MAP2K1 PE=1 SV=2	1.63	0.00	0.00	0.00	0.00	1.89	0.00	0.00	0.00	0.00	0.00	0.00	0.00	0.00	0.00	0.00	0.00	0.00	0.00	0.00	0.00	0.00	0.00	0.00	0.29	0.00	>0.999999999999999	#DIV/0!
Q5HYA8	Meckelin OS=Homo sapiens GN=TMEM67 PE=1 SV=2	0.61	0.00	0.72	0.64	0.00	0.00	0.51	0.00	0.00	0.56	0.47	0.00	0.00	0.00	0.72	0.00	0.38	0.46	0.00	0.00	0.00	0.00	0.00	0.12	0.29	0.14	>0.999999999999999	2.1
Q9HB71	Calcyclin-binding protein OS=Homo sapiens GN=CACYBP PE=1 SV=2	2.65	7.38	10.28	5.77	0.00	3.07	4.62	0.00	1.78	5.00	4.25	0.00	0.00	2.09	0.00	0.00	0.00	1.99	0.00	0.00	0.00	2.16	1.84	0.00	3.73	0.67	>0.999999999999999	5.5
Q9UBB4	Ataxin-10 OS=Homo sapiens GN=ATXN10 PE=1 SV=1	1.22	1.03	3.11	0.00	1.13	1.42	0.00	0.00	0.00	0.00	0.94	0.00	0.00	0.00	0.00	0.00	0.00	0.00	0.00	0.00	0.00	0.00	0.00	0.00	0.74	0.00	>0.999999999999999	#DIV/0!
Q96B54	Zinc finger protein 428 OS=Homo sapiens GN=ZNF428 PE=1 SV=2	3.26	0.00	8.37	0.00	3.02	0.00	0.00	0.00	0.00	0.00	0.00	0.00	0.00	0.00	0.00	0.00	0.00	0.00	0.00	0.00	0.00	0.00	0.00	0.00	1.22	0.00	>0.999999999999999	#DIV/0!
O75884	Putative hydrolase RBBP9 OS=Homo sapiens GN=RBBP9 PE=1 SV=2	3.26	0.00	3.82	7.27	0.00	0.00	0.00	0.00	0.00	0.00	0.00	0.00	0.00	0.00	0.00	0.00	0.00	0.00	0.00	0.00	0.00	0.00	0.00	0.00	1.20	0.00	>0.999999999999999	#DIV/0!
P63279	SUMO-conjugating enzyme UBC9 OS=Homo sapiens GN=UBE2I PE=1 SV=1	3.87	0.00	4.54	8.77	3.58	4.49	3.25	0.00	2.60	0.00	2.99	0.00	0.00	0.00	4.53	6.90	0.00	6.29	0.00	0.00	0.00	1.52	2.70	0.76	2.84	1.89	>0.999999999999999	1.5
P02746	Complement C1q subcomponent subunit B OS=Homo sapiens GN=C1QB PE=1 SV=3	2.45	0.00	0.00	2.57	2.26	0.00	2.05	1.08	1.64	0.00	0.00	0.98	2.82	4.17	2.86	7.07	3.29	1.84	2.17	3.38	0.00	0.96	3.69	0.48	1.09	2.73	>0.999999999999999	0.4
Q04756	Hepatocyte growth factor activator OS=Homo sapiens GN=HGFAC PE=1 SV=1	1.02	0.00	0.00	0.00	0.00	0.00	0.00	0.00	0.00	0.00	0.00	0.00	0.00	0.00	0.00	0.00	0.00	0.00	0.00	0.00	0.00	0.00	0.00	0.00	0.08	0.00	>0.999999999999999	#DIV/0!
P53004	Biliverdin reductase A OS=Homo sapiens GN=BLVRA PE=1 SV=2	2.04	5.49	11.00	4.49	3.96	4.96	3.59	1.90	2.88	3.89	5.03	2.61	2.35	1.61	2.38	5.38	1.27	3.22	1.81	0.00	0.00	2.56	2.98	1.28	4.32	2.07	>0.999999999999999	2.1
P04438	Ig heavy chain V-II region SESS OS=Homo sapiens PE=2 SV=1	4.28	0.00	0.00	0.00	14.14	0.00	12.82	4.06	2.88	0.00	0.00	1.71	10.58	0.00	0.00	7.74	9.49	11.51	13.57	5.84	3.54	0.00	6.53	0.84	3.33	5.80	>0.999999999999999	0.6
A6NLC5	UPF0524 protein C3orf70 OS=Homo sapiens GN=C3orf70 PE=2 SV=1	2.45	0.00	0.00	0.00	0.00	0.00	0.00	0.00	0.00	0.00	0.00	0.00	0.00	0.00	0.00	0.00	0.00	0.00	0.00	0.00	0.00	0.00	0.00	0.00	0.20	0.00	>0.999999999999999	#DIV/0!
Q8IUG5	Unconventional myosin-XVIIIb OS=Homo sapiens GN=MYO18B PE=1 SV=1	0.20	0.00	0.00	0.00	0.00	0.00	0.00	0.00	0.00	0.00	0.00	0.00	0.00	0.00	0.00	0.00	0.00	0.00	0.18	0.00	0.00	0.00	0.00	0.00	0.02	0.02	>0.999999999999999	1.1
P18077	60S ribosomal protein L35a OS=Homo sapiens GN=RPL35A PE=1 SV=2	5.51	0.00	0.00	0.00	5.09	14.65	0.00	0.00	0.00	5.19	9.75	0.00	6.59	4.34	0.00	0.00	0.00	0.00	0.00	0.00	4.56	0.00	0.00	0.00	3.35	1.29	>0.999999999999999	2.6
P30419	Glycylpeptide N-tetradecanoyltransferase 1 OS=Homo sapiens GN=NMT1 PE=1 SV=2	1.22	0.00	2.87	1.28	3.39	4.25	2.05	0.00	0.00	0.00	1.89	0.98	0.00	0.00	2.86	0.00	0.00	0.00	0.00	0.00	0.00	0.48	0.85	0.24	1.50	0.37	>0.999999999999999	4.0
P29034	Protein S100-A2 OS=Homo sapiens GN=S100A2 PE=1 SV=3	6.12	0.00	0.00	0.00	0.00	0.00	0.00	0.00	0.00	0.00	0.00	0.00	7.06	4.82	0.00	5.05	3.80	4.60	5.43	4.15	0.00	0.00	0.00	1.20	0.51	3.01	>0.999999999999999	0.2
O14802	DNA-directed RNA polymerase III subunit RPC1 OS=Homo sapiens GN=POLR3A PE=1 SV=2	0.41	0.00	0.00	0.00	0.00	0.00	0.00	0.00	0.00	0.00	0.00	0.00	0.00	0.00	0.00	0.00	0.25	0.31	0.00	0.00	0.00	0.00	0.00	0.00	0.03	0.05	>0.999999999999999	0.7
Q13177	Serine/threonine-protein kinase PAK 2 OS=Homo sapiens GN=PAK2 PE=1 SV=3	1.22	2.06	4.30	3.85	0.00	2.84	2.05	0.00	0.00	2.22	0.00	0.00	0.00	0.00	0.00	1.01	0.76	0.00	0.00	0.00	0.00	1.44	1.70	0.00	1.55	0.41	>0.999999999999999	3.8
P10619	Lysosomal protective protein OS=Homo sapiens GN=CTSA PE=1 SV=2	1.22	0.00	0.00	0.00	0.00	0.00	0.00	1.08	0.00	0.00	0.00	0.49	0.00	0.96	0.00	1.01	0.00	0.92	1.09	0.00	1.01	0.48	0.00	0.24	0.23	0.48	>0.999999999999999	0.5
P10644	cAMP-dependent protein kinase type I-alpha regulatory subunit OS=Homo sapiens GN=PRKAR1A PE=1 SV=1	1.63	0.00	1.91	0.00	0.00	0.00	0.00	0.00	0.00	1.48	0.00	0.00	0.00	0.00	0.00	0.00	0.00	0.00	0.00	0.00	0.00	0.00	1.14	0.00	0.42	0.09	>0.999999999999999	4.4
P62333	26S protease regulatory subunit 10B OS=Homo sapiens GN=PSMC6 PE=1 SV=1	1.43	0.00	3.59	3.21	6.22	1.65	7.35	4.88	3.29	4.45	5.19	1.22	0.00	1.12	5.72	1.18	0.00	0.00	4.34	0.00	0.00	1.92	4.68	0.60	3.54	1.63	>0.999999999999999	2.2
Q15149	Plectin OS=Homo sapiens GN=PLEC PE=1 SV=3	0.20	0.17	0.24	0.21	0.19	0.95	0.00	0.00	0.14	0.00	1.57	0.33	0.00	0.00	0.72	0.34	0.13	0.15	0.72	0.00	0.17	0.08	1.14	0.12	0.33	0.30	>0.999999999999999	1.1
P17096	High mobility group protein HMG-I/HMG-Y OS=Homo sapiens GN=HMGA1 PE=1 SV=3	6.12	0.00	0.00	0.00	5.66	0.00	0.00	0.00	0.00	0.00	0.00	0.00	0.00	0.00	0.00	0.00	0.00	0.00	0.00	0.00	0.00	0.00	0.00	0.00	0.98	0.00	>0.999999999999999	#DIV/0!
Q96A72	Protein mago nashi homolog 2 OS=Homo sapiens GN=MAGOHB PE=1 SV=1	4.08	0.00	0.00	4.28	0.00	4.73	0.00	0.00	0.00	0.00	3.15	0.00	0.00	0.00	0.00	0.00	0.00	0.00	3.62	0.00	0.00	1.60	0.00	0.00	1.35	0.43	>0.999999999999999	3.1
O60437	Periplakin OS=Homo sapiens GN=PPL PE=1 SV=4	0.41	0.34	0.72	0.43	0.57	1.65	0.34	0.63	0.27	0.56	1.26	0.98	0.71	0.00	0.72	1.68	0.63	1.07	2.17	0.00	0.34	0.16	2.13	0.28	0.68	0.82	>0.999999999999999	0.8
Q9NYL9	Tropomodulin-3 OS=Homo sapiens GN=TMOD3 PE=1 SV=1	1.63	2.92	9.08	1.71	0.00	1.89	0.00	0.72	0.00	0.00	2.67	0.65	0.00	1.28	1.91	0.00	0.00	2.61	0.00	1.04	0.00	0.00	0.00	0.00	1.77	0.57	>0.999999999999999	3.1
P12532	Creatine kinase U-type, mitochondrial OS=Homo sapiens GN=CKMT1A PE=1 SV=1	1.43	0.00	0.00	0.00	1.32	0.00	1.20	0.00	0.96	0.00	1.10	1.79	0.00	0.00	0.00	1.18	0.00	0.00	1.27	0.00	0.00	0.56	0.99	1.24	0.65	0.44	>0.999999999999999	1.5
Q8NHQ9	ATP-dependent RNA helicase DDX55 OS=Homo sapiens GN=DDX55 PE=1 SV=3	1.02	0.86	1.20	1.07	0.94	1.18	0.00	0.00	0.00	0.93	0.79	0.41	1.18	0.80	1.19	0.00	0.00	0.00	0.90	0.00	0.00	0.00	0.71	0.20	0.70	0.42	>0.999999999999999	1.7
O00410	Importin-5 OS=Homo sapiens GN=IPO5 PE=1 SV=4	0.61	0.00	3.35	1.71	3.21	4.73	1.37	0.00	0.41	3.15	2.67	1.14	0.71	0.00	4.05	0.50	0.00	0.77	0.00	0.00	0.00	0.64	1.99	0.00	1.86	0.72	>0.999999999999999	2.6
Q96AE4	Far upstream element-binding protein 1 OS=Homo sapiens GN=FUBP1 PE=1 SV=3	1.02	0.00	3.59	4.49	2.83	4.96	0.85	0.90	0.00	3.89	5.19	0.82	0.00	0.00	1.19	1.68	0.00	0.00	0.90	0.00	0.00	1.20	3.83	0.00	2.38	0.73	>0.999999999999999	3.2
P53990	IST1 homolog OS=Homo sapiens GN=IST1 PE=1 SV=1	1.63	1.37	0.00	0.00	1.51	4.02	2.91	0.00	0.00	0.00	2.67	0.65	0.00	0.00	0.00	0.00	0.00	0.00	1.45	0.00	0.00	0.00	0.00	0.32	1.23	0.15	>0.999999999999999	8.4
P30613	Pyruvate kinase isozymes R/L OS=Homo sapiens GN=PKLR PE=1 SV=2	0.00	1.89	0.00	0.00	0.00	0.00	0.00	0.00	0.00	0.00	0.00	0.00	0.00	0.00	0.00	0.00	0.00	0.00	0.00	0.00	0.00	0.00	0.00	0.00	0.16	0.00	>0.999999999999999	#DIV/0!
Q9BQE3	Tubulin alpha-1C chain OS=Homo sapiens GN=TUBA1C PE=1 SV=1	0.00	27.10	55.46	0.00	0.00	0.00	0.00	0.00	0.00	0.00	39.95	0.00	29.87	0.00	0.00	0.00	0.00	0.00	18.09	0.00	16.88	0.00	27.39	7.73	10.21	8.33	>0.999999999999999	1.2
P98088	Mucin-5AC (Fragments) OS=Homo sapiens GN=MUC5AC PE=1 SV=3	0.00	0.51	0.24	0.00	0.75	0.24	0.00	0.09	0.14	0.00	0.31	0.65	0.24	0.00	0.95	0.50	0.25	0.00	0.00	3.89	0.17	0.08	1.99	0.60	0.24	0.72	>0.999999999999999	0.3
P0C0L5	Complement C4-B OS=Homo sapiens GN=C4B PE=1 SV=1	0.00	3.09	0.00	0.00	0.00	11.11	6.84	0.00	7.40	0.00	0.00	10.28	0.00	7.55	0.00	6.23	8.22	13.04	11.76	12.72	0.00	4.95	9.22	1.60	3.23	6.27	>0.999999999999999	0.5
P35527	Keratin, type I cytoskeletal 9 OS=Homo sapiens GN=KRT9 PE=1 SV=3	0.00	8.75	0.00	0.00	3.21	1.18	1.88	0.00	0.68	4.26	0.79	1.39	5.41	3.69	2.62	1.85	0.63	0.00	5.25	2.21	25.66	2.32	1.56	1.72	1.84	4.41	>0.999999999999999	0.4
P02538	Keratin, type II cytoskeletal 6A OS=Homo sapiens GN=KRT6A PE=1 SV=3	0.00	4.12	0.00	0.00	0.00	0.00	0.00	1.54	1.51	11.30	0.00	0.00	2.59	3.85	0.00	5.05	3.04	2.61	8.14	2.21	6.41	3.04	0.00	2.12	1.54	3.25	>0.999999999999999	0.5
P13645	Keratin, type I cytoskeletal 10 OS=Homo sapiens GN=KRT10 PE=1 SV=6	0.00	17.50	4.30	8.12	8.67	10.87	3.08	1.63	4.25	33.35	9.91	13.46	7.29	13.17	5.72	9.09	12.91	0.92	9.77	3.12	12.15	9.11	6.53	4.56	9.59	7.86	>0.999999999999999	1.2
Q04828	Aldo-keto reductase family 1 member C1 OS=Homo sapiens GN=AKR1C1 PE=1 SV=1	0.00	11.49	0.00	33.35	0.00	19.38	14.02	14.09	9.18	18.16	36.17	0.00	0.00	0.00	42.45	34.15	0.00	20.71	32.19	10.65	3.21	0.00	0.00	10.41	12.99	12.81	>0.999999999999999	1.0
P48735	Isocitrate dehydrogenase [NADP], mitochondrial OS=Homo sapiens GN=IDH2 PE=1 SV=2	0.00	2.23	3.11	4.49	0.00	0.00	0.00	0.00	0.00	0.00	3.30	2.28	0.00	2.09	3.10	2.19	0.00	0.00	0.00	0.00	0.00	1.04	0.00	0.00	1.28	0.70	>0.999999999999999	1.8
Q92526	T-complex protein 1 subunit zeta-2 OS=Homo sapiens GN=CCT6B PE=1 SV=5	0.00	5.49	4.30	2.57	0.00	4.25	0.00	0.00	0.00	2.22	0.00	0.00	0.00	0.00	4.29	0.00	0.00	0.00	0.00	0.00	0.00	0.00	0.00	0.00	1.57	0.36	>0.999999999999999	4.4
P63010	AP-2 complex subunit beta OS=Homo sapiens GN=AP2B1 PE=1 SV=1	0.00	2.23	4.06	5.99	1.13	4.02	0.00	0.00	0.00	3.71	8.34	0.00	0.00	0.00	4.77	0.00	0.00	0.00	0.00	0.00	0.00	0.00	2.84	0.52	2.46	0.68	>0.999999999999999	3.6
P27816	Microtubule-associated protein 4 OS=Homo sapiens GN=MAP4 PE=1 SV=3	0.00	1.37	0.72	1.71	0.00	1.18	0.51	0.00	0.00	1.48	1.73	0.24	0.00	0.48	0.00	0.00	0.00	0.00	0.00	0.00	0.00	0.24	0.00	0.12	0.75	0.07	>0.999999999999999	10.6
Q8N142	Adenylosuccinate synthetase isozyme 1 OS=Homo sapiens GN=ADSSL1 PE=1 SV=1	0.00	2.23	3.11	2.78	0.00	3.07	0.00	0.00	0.00	2.41	5.82	0.00	0.00	0.00	3.10	0.00	0.00	0.00	0.00	0.00	0.00	0.00	1.84	0.00	1.62	0.41	>0.999999999999999	3.9
P33176	Kinesin-1 heavy chain OS=Homo sapiens GN=KIF5B PE=1 SV=1	0.00	3.26	5.50	4.06	4.90	7.09	2.05	0.27	0.00	6.30	6.61	1.31	0.00	2.57	8.11	2.02	0.76	0.00	0.00	0.00	0.00	0.96	3.69	0.48	3.45	1.55	>0.999999999999999	2.2
A0MZ66	Shootin-1 OS=Homo sapiens GN=KIAA1598 PE=1 SV=4	0.00	2.40	2.15	2.99	1.70	3.31	0.85	0.00	0.00	0.93	3.93	0.73	0.00	0.00	3.34	0.84	0.00	0.77	0.90	0.00	0.00	1.52	3.55	0.56	1.58	0.96	>0.999999999999999	1.7
P41091	Eukaryotic translation initiation factor 2 subunit 3 OS=Homo sapiens GN=EIF2S3 PE=1 SV=3	0.00	4.80	8.61	11.55	8.48	6.62	3.42	0.00	0.00	5.19	0.00	1.63	0.00	0.96	10.73	3.36	0.00	0.00	5.06	0.00	0.00	2.24	3.97	1.12	4.19	2.29	>0.999999999999999	1.8
P23141	Liver carboxylesterase 1 OS=Homo sapiens GN=CES1 PE=1 SV=2	0.00	4.97	1.20	3.63	0.00	6.85	0.00	0.00	0.00	4.26	9.12	3.51	0.00	1.77	0.00	0.00	0.00	0.00	0.00	0.00	0.00	1.36	6.10	0.92	2.80	0.85	>0.999999999999999	3.3
P61088	Ubiquitin-conjugating enzyme E2 N OS=Homo sapiens GN=UBE2N PE=1 SV=1	0.00	18.01	16.97	15.18	0.00	24.81	12.14	0.00	0.00	7.97	11.17	3.51	0.00	11.40	16.93	11.95	5.44	10.89	0.00	0.00	0.00	5.67	20.58	1.72	9.15	7.05	>0.999999999999999	1.3
Q9H4G0	Band 4.1-like protein 1 OS=Homo sapiens GN=EPB41L1 PE=1 SV=2	0.00	4.46	2.39	2.14	2.64	3.31	0.00	0.00	0.00	1.85	2.20	0.57	0.71	0.00	0.72	1.18	0.38	0.00	0.54	0.00	0.00	0.24	1.42	0.56	1.63	0.48	>0.999999999999999	3.4
Q96M27	Protein PRRC1 OS=Homo sapiens GN=PRRC1 PE=1 SV=1	0.00	1.20	5.50	4.92	2.83	5.43	2.56	0.00	0.00	0.00	3.62	1.88	0.00	0.00	0.00	2.52	0.00	0.00	0.00	0.00	0.00	0.56	2.13	0.28	2.33	0.46	>0.999999999999999	5.1
Q13409	Cytoplasmic dynein 1 intermediate chain 2 OS=Homo sapiens GN=DYNC1I2 PE=1 SV=3	0.00	3.43	4.78	1.92	3.77	4.73	2.39	0.00	0.00	2.59	3.93	0.00	0.00	1.45	3.34	0.84	0.63	2.15	0.90	0.00	0.84	0.40	1.28	0.36	2.30	1.02	>0.999999999999999	2.3
O43583	Density-regulated protein OS=Homo sapiens GN=DENR PE=1 SV=2	0.00	5.49	3.59	3.21	2.83	3.54	2.56	0.00	2.05	2.78	5.03	1.22	0.00	0.00	7.63	2.52	0.00	0.00	2.71	0.00	0.00	1.20	2.13	0.00	2.69	1.35	>0.999999999999999	2.0
Q8IZ83	Aldehyde dehydrogenase family 16 member A1 OS=Homo sapiens GN=ALDH16A1 PE=1 SV=2	0.00	1.37	0.96	2.57	2.26	0.95	0.00	0.00	0.00	1.48	1.89	0.65	0.94	0.00	0.00	0.00	0.00	0.00	1.45	0.00	0.00	0.64	0.00	0.00	1.01	0.25	>0.999999999999999	4.0
Q96FV2	Secernin-2 OS=Homo sapiens GN=SCRN2 PE=2 SV=3	0.00	2.57	0.00	1.50	0.00	3.54	0.00	0.00	0.00	1.30	0.00	0.57	0.00	1.12	0.00	0.00	0.00	0.00	0.00	0.00	0.00	1.20	0.99	0.00	0.79	0.28	>0.999999999999999	2.9
P49354	Protein farnesyltransferase/geranylgeranyltransferase type-1 subunit alpha OS=Homo sapiens GN=FNTA PE=1 SV=	0.00	1.20	3.59	1.50	2.83	7.80	2.56	1.35	0.00	4.45	5.19	1.96	0.00	2.41	1.67	2.52	0.00	1.07	0.00	0.00	0.00	1.20	3.41	0.60	2.70	1.07	>0.999999999999999	2.5
P49588	Alanine--tRNA ligase, cytoplasmic OS=Homo sapiens GN=AARS PE=1 SV=2	0.00	2.23	5.50	2.78	1.70	7.33	0.51	0.00	0.00	2.96	0.94	0.24	0.00	1.45	1.43	0.00	0.00	0.00	0.00	0.00	0.00	0.72	1.84	0.00	2.02	0.45	>0.999999999999999	4.4
Q9NT62	Ubiquitin-like-conjugating enzyme ATG3 OS=Homo sapiens GN=ATG3 PE=1 SV=1	0.00	3.26	4.54	1.92	1.70	7.09	3.25	0.00	0.00	1.67	1.42	1.55	0.00	0.00	4.53	5.05	1.14	2.92	1.63	0.00	0.00	2.40	4.26	0.36	2.20	1.86	>0.999999999999999	1.2
Q9UNF1	Melanoma-associated antigen D2 OS=Homo sapiens GN=MAGED2 PE=1 SV=2	0.00	1.72	2.39	2.14	0.94	3.78	0.85	0.00	0.00	0.93	2.52	0.41	0.00	0.00	5.25	0.00	0.63	0.00	0.00	0.00	0.00	0.40	1.42	0.00	1.31	0.64	>0.999999999999999	2.0
Q13564	NEDD8-activating enzyme E1 regulatory subunit OS=Homo sapiens GN=NAE1 PE=1 SV=1	0.00	2.92	4.06	3.63	2.07	1.18	1.88	0.00	0.00	3.15	3.62	1.39	0.00	0.80	1.19	0.84	1.39	0.00	0.00	0.00	0.00	0.88	1.56	0.68	1.99	0.61	>0.999999999999999	3.3
Q9BT78	COP9 signalosome complex subunit 4 OS=Homo sapiens GN=COPS4 PE=1 SV=1	0.00	3.95	7.65	6.84	4.34	5.43	7.01	0.00	0.96	7.60	5.03	2.61	0.00	2.41	9.78	2.52	0.00	1.07	1.27	0.00	0.00	1.84	7.24	0.60	4.28	2.23	>0.999999999999999	1.9
P36959	GMP reductase 1 OS=Homo sapiens GN=GMPR PE=1 SV=1	0.00	4.97	9.56	3.85	0.00	6.85	0.00	0.00	0.00	3.33	2.83	0.00	0.00	0.00	2.15	0.00	0.00	0.00	0.00	0.00	0.00	0.00	1.28	0.00	2.62	0.29	>0.999999999999999	9.2
Q9UBC9	Small proline-rich protein 3 OS=Homo sapiens GN=SPRR3 PE=1 SV=2	0.00	16.12	0.00	8.34	3.39	4.25	3.08	15.44	0.00	3.33	14.78	3.18	0.00	0.00	4.29	15.82	2.28	19.95	17.00	5.06	15.87	7.51	9.22	0.72	5.99	8.14	>0.999999999999999	0.7
O95757	Heat shock 70 kDa protein 4L OS=Homo sapiens GN=HSPA4L PE=1 SV=3	0.00	1.89	3.35	4.70	0.00	3.31	1.20	0.00	0.00	5.00	1.73	0.00	0.00	0.00	4.29	0.00	0.00	0.00	0.00	0.00	0.00	0.88	1.56	0.00	1.76	0.56	>0.999999999999999	3.1
Q8NDH3	Probable aminopeptidase NPEPL1 OS=Homo sapiens GN=NPEPL1 PE=1 SV=3	0.00	2.06	4.30	2.57	0.00	4.25	1.03	0.54	0.00	2.22	2.83	0.00	0.00	1.93	2.86	0.00	0.00	1.84	0.00	0.00	0.00	0.48	0.00	0.00	1.65	0.59	>0.999999999999999	2.8
Q9UJU6	Drebrin-like protein OS=Homo sapiens GN=DBNL PE=1 SV=1	0.00	3.77	3.35	1.50	5.66	9.22	5.13	2.71	1.92	4.08	4.72	2.45	1.65	1.12	5.25	1.18	0.89	2.15	3.98	0.00	2.36	1.12	1.99	1.56	3.71	1.94	>0.999999999999999	1.9
Q9UH65	Switch-associated protein 70 OS=Homo sapiens GN=SWAP70 PE=1 SV=1	0.00	3.43	2.39	3.21	2.83	3.54	0.85	0.00	0.00	0.93	2.36	0.82	0.00	1.61	4.77	2.52	0.00	0.77	0.90	0.00	0.00	1.20	1.42	0.40	1.70	1.13	>0.999999999999999	1.5
P23588	Eukaryotic translation initiation factor 4B OS=Homo sapiens GN=EIF4B PE=1 SV=2	0.00	2.57	2.39	3.21	0.94	3.54	2.56	0.90	0.68	2.78	5.03	1.22	1.18	0.80	3.58	0.84	0.63	1.53	0.90	0.00	0.84	1.20	2.13	0.40	2.15	1.17	>0.999999999999999	1.8
Q13153	Serine/threonine-protein kinase PAK 1 OS=Homo sapiens GN=PAK1 PE=1 SV=2	0.00	1.89	4.06	4.92	0.94	2.60	0.00	0.00	0.00	0.00	0.79	0.90	0.00	0.00	1.19	0.00	0.00	0.00	0.00	0.00	0.00	0.88	0.00	0.44	1.34	0.21	>0.999999999999999	6.4
Q5TFE4	5'-nucleotidase domain-containing protein 1 OS=Homo sapiens GN=NT5DC1 PE=1 SV=1	0.00	3.43	6.69	0.00	1.13	3.07	1.03	0.54	0.00	2.41	3.15	0.49	1.41	2.09	3.10	1.01	0.00	0.00	1.09	0.00	0.00	0.00	2.84	0.00	1.83	0.96	>0.999999999999999	1.9
O75351	Vacuolar protein sorting-associated protein 4B OS=Homo sapiens GN=VPS4B PE=1 SV=2	0.00	3.60	1.67	1.50	0.00	3.31	2.39	0.00	0.00	0.00	3.30	0.57	0.00	0.00	1.67	0.00	0.00	0.00	0.00	0.00	0.00	0.00	1.99	0.00	1.36	0.30	>0.999999999999999	4.5
Q8N6N7	Acyl-CoA-binding domain-containing protein 7 OS=Homo sapiens GN=ACBD7 PE=1 SV=1	0.00	6.00	8.37	7.48	6.60	8.27	0.00	0.00	0.00	0.00	0.00	0.00	0.00	0.00	8.35	0.00	0.00	0.00	0.00	0.00	0.00	2.80	0.00	0.00	3.06	0.93	>0.999999999999999	3.3
P16083	Ribosyldihydronicotinamide dehydrogenase [quinone] OS=Homo sapiens GN=NQO2 PE=1 SV=5	0.00	7.55	6.45	2.78	5.09	6.38	0.00	1.17	0.00	5.00	4.25	0.00	0.00	2.09	3.10	0.00	1.64	1.99	0.00	1.69	2.19	2.16	8.80	0.52	3.22	2.02	>0.999999999999999	1.6
P31944	Caspase-14 OS=Homo sapiens GN=CASP14 PE=1 SV=2	0.00	9.78	0.00	2.57	0.00	0.00	2.05	5.15	0.00	7.41	0.00	0.00	2.82	9.15	2.86	4.21	1.52	1.84	7.23	1.56	6.75	2.00	0.00	0.48	2.25	3.37	>0.999999999999999	0.7
Q96BW5	Phosphotriesterase-related protein OS=Homo sapiens GN=PTER PE=1 SV=1	0.00	6.52	0.00	0.00	1.51	0.00	0.00	0.00	0.00	0.00	4.25	0.65	0.00	0.00	0.00	6.39	1.01	2.61	0.00	0.00	0.00	0.64	5.39	0.00	1.08	1.34	>0.999999999999999	0.8
P39023	60S ribosomal protein L3 OS=Homo sapiens GN=RPL3 PE=1 SV=2	0.00	2.57	5.50	3.21	4.34	7.56	3.93	0.00	0.96	2.78	5.03	0.57	0.00	3.69	3.58	2.52	0.89	1.07	2.71	0.00	0.00	0.00	2.13	0.28	3.04	1.41	>0.999999999999999	2.2
P09936	Ubiquitin carboxyl-terminal hydrolase isozyme L1 OS=Homo sapiens GN=UCHL1 PE=1 SV=2	0.00	2.23	0.00	0.00	2.45	3.07	0.00	0.00	0.00	2.41	0.00	0.00	0.00	2.09	0.00	0.00	0.00	0.00	0.00	0.00	0.00	1.04	0.00	0.00	0.85	0.26	>0.999999999999999	3.3
Q15366	Poly(rC)-binding protein 2 OS=Homo sapiens GN=PCBP2 PE=1 SV=1	0.00	3.09	15.06	13.47	9.43	11.82	8.55	1.63	0.00	3.33	9.91	0.00	4.23	0.00	11.92	6.56	3.54	4.30	0.00	0.00	0.00	2.24	5.53	1.56	6.36	3.32	>0.999999999999999	1.9
Q9NP79	Vacuolar protein sorting-associated protein VTA1 homolog OS=Homo sapiens GN=VTA1 PE=1 SV=1	0.00	3.43	2.39	2.14	0.00	2.36	0.00	0.00	0.00	1.85	1.57	0.00	0.00	0.00	0.00	0.00	0.00	1.53	0.00	0.00	0.00	0.80	1.42	0.00	1.15	0.31	>0.999999999999999	3.7
P27694	Replication protein A 70 kDa DNA-binding subunit OS=Homo sapiens GN=RPA1 PE=1 SV=2	0.00	0.86	2.39	3.21	2.83	4.96	1.71	0.00	0.00	0.93	4.09	1.71	0.00	0.00	2.38	0.84	0.00	0.77	0.00	0.00	0.00	0.80	1.42	0.00	1.89	0.52	>0.999999999999999	3.7
Q7Z6Z7	E3 ubiquitin-protein ligase HUWE1 OS=Homo sapiens GN=HUWE1 PE=1 SV=3	0.00	0.51	1.20	0.21	0.19	0.47	0.17	0.00	0.00	0.74	0.16	0.00	0.00	0.00	0.24	0.17	0.00	0.00	0.00	0.00	0.00	0.00	0.28	0.04	0.30	0.06	>0.999999999999999	5.0
Q9H977	WD repeat-containing protein 54 OS=Homo sapiens GN=WDR54 PE=1 SV=1	0.00	3.26	7.17	4.06	3.58	4.49	1.54	0.81	0.00	3.52	2.99	0.00	0.00	1.45	0.00	0.00	0.00	0.00	0.00	0.00	0.00	0.00	2.70	0.00	2.62	0.35	>0.999999999999999	7.6
Q9UHX1	Poly(U)-binding-splicing factor PUF60 OS=Homo sapiens GN=PUF60 PE=1 SV=1	0.00	1.89	0.00	1.07	0.00	2.60	0.00	0.00	0.00	0.93	0.79	0.00	0.00	0.00	1.19	0.00	0.00	0.00	0.00	0.00	0.00	0.88	0.71	0.00	0.61	0.23	>0.999999999999999	2.6
Q8N573	Oxidation resistance protein 1 OS=Homo sapiens GN=OXR1 PE=1 SV=2	0.00	1.72	7.17	0.00	0.00	0.71	0.00	0.00	0.00	1.85	0.00	0.24	0.00	0.48	0.72	0.00	0.00	0.00	0.00	0.00	0.00	0.00	0.43	0.00	0.97	0.14	>0.999999999999999	7.2
P43243	Matrin-3 OS=Homo sapiens GN=MATR3 PE=1 SV=2	0.00	1.20	1.67	0.64	0.57	4.25	0.00	0.00	0.00	2.04	1.10	0.00	0.00	0.00	0.00	0.50	0.00	0.00	0.00	0.00	0.00	0.00	0.00	0.00	0.96	0.04	>0.999999999999999	22.7
O43865	Putative adenosylhomocysteinase 2 OS=Homo sapiens GN=AHCYL1 PE=1 SV=2	0.00	2.92	10.76	0.00	3.21	0.00	0.00	0.00	0.00	4.45	0.00	0.00	0.00	0.00	5.72	0.00	0.00	0.00	0.00	0.00	0.00	0.00	2.41	0.68	1.78	0.73	>0.999999999999999	2.4
Q15121	Astrocytic phosphoprotein PEA-15 OS=Homo sapiens GN=PEA15 PE=1 SV=2	0.00	8.58	11.95	4.92	9.43	11.82	3.93	2.08	3.15	9.26	7.86	4.08	0.00	3.69	11.92	8.41	2.91	7.67	4.16	2.99	0.00	1.84	7.10	0.92	6.42	4.30	>0.999999999999999	1.5
P62714	Serine/threonine-protein phosphatase 2A catalytic subunit beta isoform OS=Homo sapiens GN=PPP2CB PE=1 SV=	0.00	3.26	2.15	4.06	0.00	4.49	1.54	0.00	1.23	1.67	0.00	0.00	0.00	0.00	2.15	1.51	0.00	1.38	0.00	0.00	0.00	3.35	2.70	1.20	1.53	1.02	>0.999999999999999	1.5
Q14697	Neutral alpha-glucosidase AB OS=Homo sapiens GN=GANAB PE=1 SV=3	0.00	0.51	0.72	1.92	0.00	0.71	0.51	0.00	0.00	0.56	0.47	1.06	0.00	0.00	0.00	1.01	0.00	0.00	0.54	0.00	0.00	0.72	0.43	0.24	0.54	0.24	>0.999999999999999	2.2
P50851	Lipopolysaccharide-responsive and beige-like anchor protein OS=Homo sapiens GN=LRBA PE=1 SV=4	0.00	0.34	1.91	1.28	0.75	2.84	0.68	0.18	0.00	1.11	1.42	0.33	0.00	0.16	0.24	0.34	0.00	0.00	0.18	0.00	0.00	0.16	0.71	0.00	0.90	0.15	>0.999999999999999	6.1
O95865	N(G),N(G)-dimethylarginine dimethylaminohydrolase 2 OS=Homo sapiens GN=DDAH2 PE=1 SV=1	0.00	4.12	12.67	2.35	2.07	8.74	1.88	0.00	0.00	12.97	1.73	0.90	0.00	0.00	5.72	0.00	1.39	0.00	4.34	0.00	0.00	1.92	1.56	0.44	3.95	1.28	>0.999999999999999	3.1
P18440	Arylamine N-acetyltransferase 1 OS=Homo sapiens GN=NAT1 PE=1 SV=2	0.00	5.49	7.65	0.00	3.77	2.36	3.42	1.81	1.37	3.71	3.15	0.82	2.35	1.61	2.38	1.68	1.27	1.53	1.81	0.00	0.00	0.80	2.84	0.00	2.79	1.36	>0.999999999999999	2.1
Q08257	Quinone oxidoreductase OS=Homo sapiens GN=CRYZ PE=1 SV=1	0.00	7.38	13.39	6.63	1.70	16.78	1.54	0.81	0.00	3.71	0.00	2.53	0.00	3.21	4.77	1.51	3.92	0.00	1.63	0.00	0.00	2.48	1.28	0.00	4.54	1.57	>0.999999999999999	2.9
Q13526	Peptidyl-prolyl cis-trans isomerase NIMA-interacting 1 OS=Homo sapiens GN=PIN1 PE=1 SV=1	0.00	3.09	4.30	3.85	0.00	4.25	3.08	0.00	0.00	3.33	2.83	0.00	0.00	0.00	4.29	0.00	0.00	2.76	0.00	0.00	0.00	1.44	0.00	0.00	2.06	0.71	>0.999999999999999	2.9
Q07617	Sperm-associated antigen 1 OS=Homo sapiens GN=SPAG1 PE=1 SV=3	0.00	0.51	1.43	0.64	0.00	0.71	0.00	0.00	0.00	1.11	0.47	0.00	0.00	0.00	0.00	0.00	0.00	0.00	0.00	0.00	0.00	0.00	0.00	0.00	0.41	0.00	>0.999999999999999	#DIV/0!
Q9NUQ9	Protein FAM49B OS=Homo sapiens GN=FAM49B PE=1 SV=1	0.00	1.54	0.00	1.92	3.58	2.13	3.25	0.81	0.00	0.00	0.00	1.55	2.12	0.00	0.00	1.51	0.00	2.92	3.44	0.00	6.92	0.72	4.12	0.76	1.23	1.87	>0.999999999999999	0.7
Q9UBG3	Cornulin OS=Homo sapiens GN=CRNN PE=1 SV=1	0.00	1.03	0.00	0.00	0.00	0.00	0.00	1.17	0.00	1.11	0.00	0.00	0.00	0.96	0.00	0.00	0.00	0.00	0.00	0.78	0.00	0.48	0.85	0.52	0.28	0.30	>0.999999999999999	0.9
Q8NBF2	NHL repeat-containing protein 2 OS=Homo sapiens GN=NHLRC2 PE=1 SV=1	0.00	1.37	3.11	1.71	1.51	0.00	2.22	0.00	0.00	0.74	2.04	0.65	0.00	0.64	1.91	0.67	0.00	0.00	0.00	0.00	0.00	1.36	0.57	0.16	1.11	0.44	>0.999999999999999	2.5
O15067	Phosphoribosylformylglycinamidine synthase OS=Homo sapiens GN=PFAS PE=1 SV=4	0.00	0.86	2.15	1.50	0.38	1.18	0.85	0.00	0.00	0.00	0.00	0.00	0.00	0.00	0.00	0.00	0.00	0.00	0.00	0.00	0.00	0.16	0.28	0.00	0.58	0.04	>0.999999999999999	15.6
Q96C74	Ropporin-1-like protein OS=Homo sapiens GN=ROPN1L PE=1 SV=2	0.00	2.23	6.45	2.78	8.11	3.07	0.00	0.00	0.00	11.30	4.25	0.00	0.00	0.00	3.10	0.00	0.00	0.00	0.00	0.00	0.00	0.00	1.84	0.00	3.18	0.41	>0.999999999999999	7.7
Q9Y3C4	TP53RK-binding protein OS=Homo sapiens GN=TPRKB PE=1 SV=1	0.00	6.35	4.06	3.63	3.21	4.02	0.00	0.00	0.00	3.15	0.00	1.39	0.00	2.73	4.05	0.00	0.00	0.00	0.00	0.00	0.00	1.36	2.41	0.68	2.15	0.94	>0.999999999999999	2.3
Q86X76	Nitrilase homolog 1 OS=Homo sapiens GN=NIT1 PE=1 SV=2	0.00	3.26	2.15	4.06	1.70	2.13	3.25	0.00	1.23	1.67	2.99	1.55	2.12	1.45	7.15	0.00	0.00	0.00	0.00	0.00	0.00	2.40	2.70	0.36	2.00	1.35	>0.999999999999999	1.5
P07954	Fumarate hydratase, mitochondrial OS=Homo sapiens GN=FH PE=1 SV=3	0.00	1.03	0.00	1.28	0.00	0.00	1.03	0.00	0.00	0.00	0.00	0.49	0.00	0.00	0.00	3.20	0.00	0.00	0.00	0.00	0.00	0.48	0.85	0.00	0.32	0.38	>0.999999999999999	0.8
Q969E4	Transcription elongation factor A protein-like 3 OS=Homo sapiens GN=TCEAL3 PE=1 SV=1	0.00	2.57	3.59	3.21	2.83	3.54	0.00	0.00	0.00	2.78	2.36	0.00	0.00	2.41	3.58	0.00	0.00	0.00	0.00	0.00	0.00	1.20	2.13	0.00	1.74	0.78	>0.999999999999999	2.2
P22528	Cornifin-B OS=Homo sapiens GN=SPRR1B PE=1 SV=2	0.00	13.38	0.00	7.27	6.41	0.00	0.00	7.04	4.66	6.30	5.35	2.77	0.00	5.46	0.00	5.72	9.87	21.17	14.11	0.00	13.17	0.00	4.82	1.36	4.43	6.31	>0.999999999999999	0.7
P04040	Catalase OS=Homo sapiens GN=CAT PE=1 SV=3	0.00	1.89	4.06	1.07	0.00	2.60	0.85	1.54	0.00	0.93	0.79	0.41	0.00	2.73	2.62	0.00	0.00	0.00	0.00	3.12	2.87	0.00	0.71	0.00	1.18	1.00	>0.999999999999999	1.2
P55735	Protein SEC13 homolog OS=Homo sapiens GN=SEC13 PE=1 SV=3	0.00	1.54	2.15	1.92	1.70	4.49	3.25	0.81	0.00	3.52	4.72	0.73	2.12	1.45	7.15	5.05	1.14	1.38	1.63	0.00	1.52	1.52	1.28	0.36	2.07	2.05	>0.999999999999999	1.0
Q00577	Transcriptional activator protein Pur-alpha OS=Homo sapiens GN=PURA PE=1 SV=2	0.00	3.43	4.78	4.28	5.85	7.33	1.54	0.81	0.00	8.15	8.96	0.00	2.12	1.45	7.39	1.51	0.00	3.07	0.00	0.00	0.00	1.60	2.84	0.36	3.76	1.69	>0.999999999999999	2.2
P07602	Proactivator polypeptide OS=Homo sapiens GN=PSAP PE=1 SV=2	0.00	1.89	0.00	2.35	3.21	2.60	1.88	2.80	2.33	0.93	2.67	3.10	4.00	1.77	4.05	5.22	3.04	4.76	3.07	2.21	5.23	4.23	2.41	1.52	1.98	3.46	>0.999999999999999	0.6
P27482	Calmodulin-like protein 3 OS=Homo sapiens GN=CALML3 PE=1 SV=2	0.00	3.43	0.00	4.28	0.00	0.00	0.00	0.00	0.00	3.71	3.15	1.63	0.00	7.06	0.00	0.00	0.00	3.07	0.00	0.00	0.00	3.51	10.22	1.76	1.35	2.14	>0.999999999999999	0.6
P52434	DNA-directed RNA polymerases I, II, and III subunit RPABC3 OS=Homo sapiens GN=POLR2H PE=1 SV=4	0.00	3.43	4.78	4.28	0.00	4.73	3.42	0.00	0.00	3.71	3.15	0.00	0.00	3.21	4.77	3.36	0.00	0.00	0.00	0.00	0.00	1.60	2.84	0.00	2.29	1.32	>0.999999999999999	1.7
P26373	60S ribosomal protein L13 OS=Homo sapiens GN=RPL13 PE=1 SV=4	0.00	4.97	6.93	2.99	2.64	11.11	0.00	0.00	0.00	5.37	4.56	0.00	0.00	4.66	3.34	0.00	0.00	0.00	0.00	0.00	0.00	0.00	4.12	0.00	3.21	1.01	>0.999999999999999	3.2
Q8TEA8	D-tyrosyl-tRNA(Tyr) deacylase 1 OS=Homo sapiens GN=DTD1 PE=1 SV=2	0.00	2.40	3.35	2.99	0.00	3.31	0.00	0.00	0.00	0.00	2.20	0.00	0.00	0.00	3.34	0.00	0.00	0.00	0.00	0.00	0.00	0.00	0.00	0.00	1.19	0.28	>0.999999999999999	4.3
Q5T1M5	FK506-binding protein 15 OS=Homo sapiens GN=FKBP15 PE=1 SV=2	0.00	0.86	1.20	0.00	0.38	1.18	0.00	0.00	0.00	0.37	0.31	0.16	0.00	0.00	0.48	0.00	0.00	0.00	0.00	0.00	0.00	0.00	0.00	0.08	0.37	0.05	>0.999999999999999	8.0
Q13557	Calcium/calmodulin-dependent protein kinase type II subunit delta OS=Homo sapiens GN=CAMK2D PE=1 SV=3	0.00	2.06	1.43	2.57	0.00	0.00	0.00	0.00	0.00	0.00	0.94	0.00	0.00	0.00	1.43	0.00	0.00	0.00	1.09	0.00	0.00	0.00	0.00	0.00	0.58	0.21	>0.999999999999999	2.8
Q96FV0	Leucine-rich repeat-containing protein 46 OS=Homo sapiens GN=LRRC46 PE=2 SV=1	0.00	3.26	7.41	4.06	0.00	4.49	1.54	0.00	0.00	1.67	0.00	0.73	0.00	0.00	2.15	0.00	0.00	0.00	0.00	0.00	0.00	0.00	0.00	0.00	1.93	0.18	>0.999999999999999	10.8
Q9UN86	Ras GTPase-activating protein-binding protein 2 OS=Homo sapiens GN=G3BP2 PE=1 SV=2	0.00	1.03	1.43	1.28	0.00	0.00	1.03	0.00	0.00	1.11	0.00	0.00	0.00	0.96	1.43	0.00	0.00	0.00	0.00	0.00	0.00	0.00	0.00	0.00	0.49	0.20	>0.999999999999999	2.5
P10809	60 kDa heat shock protein, mitochondrial OS=Homo sapiens GN=HSPD1 PE=1 SV=2	0.00	2.92	0.00	1.07	0.94	0.00	0.00	0.00	0.00	0.00	0.79	1.88	0.00	0.80	0.00	1.85	0.00	0.00	0.00	0.00	0.00	0.88	0.71	0.44	0.63	0.39	>0.999999999999999	1.6
Q96A65	Exocyst complex component 4 OS=Homo sapiens GN=EXOC4 PE=1 SV=1	0.00	0.51	1.43	0.00	0.00	0.00	0.00	0.00	0.00	0.00	0.47	0.00	0.00	0.00	0.72	0.00	0.00	0.00	0.00	0.00	0.00	0.00	0.00	0.00	0.20	0.06	>0.999999999999999	3.4
Q9NSK0	Kinesin light chain 4 OS=Homo sapiens GN=KLC4 PE=1 SV=3	0.00	2.57	3.59	0.00	0.00	0.00	0.00	0.00	0.00	0.00	1.57	0.00	0.00	0.00	1.19	0.00	0.00	0.00	0.00	0.00	0.00	0.00	0.71	0.00	0.64	0.16	>0.999999999999999	4.1
P54727	UV excision repair protein RAD23 homolog B OS=Homo sapiens GN=RAD23B PE=1 SV=1	0.00	4.29	8.13	5.34	6.41	8.03	4.27	1.44	1.10	1.48	3.93	2.77	8.00	4.01	1.91	1.35	0.00	0.00	0.00	2.08	4.22	1.28	3.55	1.00	3.93	2.28	>0.999999999999999	1.7
O43795	Unconventional myosin-Ib OS=Homo sapiens GN=MYO1B PE=2 SV=3	0.00	0.86	0.48	0.43	0.38	0.47	0.00	0.00	0.00	0.00	0.79	0.65	0.00	0.00	0.00	0.00	0.00	0.00	0.00	0.00	0.00	0.00	0.28	0.08	0.34	0.03	>0.999999999999999	11.1
Q9H2G2	STE20-like serine/threonine-protein kinase OS=Homo sapiens GN=SLK PE=1 SV=1	0.00	1.20	0.48	1.92	1.32	0.00	0.85	0.00	0.27	0.00	1.42	1.14	0.00	0.00	0.48	2.86	0.00	0.00	0.90	0.00	0.84	0.56	0.00	0.48	0.72	0.51	>0.999999999999999	1.4
O60936	Nucleolar protein 3 OS=Homo sapiens GN=NOL3 PE=1 SV=1	0.00	2.40	6.93	2.99	0.00	3.31	0.00	0.00	0.00	2.59	2.20	1.14	0.00	0.00	0.00	0.00	0.00	0.00	0.00	0.00	0.00	0.00	0.00	0.56	1.80	0.05	>0.999999999999999	38.5
P36543	V-type proton ATPase subunit E 1 OS=Homo sapiens GN=ATP6V1E1 PE=1 SV=1	0.00	4.63	10.28	2.78	2.45	6.38	0.00	0.00	0.00	2.41	0.00	2.20	0.00	4.34	3.10	4.54	0.00	0.00	2.35	0.00	0.00	2.16	3.83	1.72	2.59	1.84	>0.999999999999999	1.4
Q8TCD5	5'(3')-deoxyribonucleotidase, cytosolic type OS=Homo sapiens GN=NT5C PE=1 SV=2	0.00	8.41	11.71	6.41	2.64	7.09	0.00	0.00	0.00	2.59	7.71	1.14	0.00	4.82	11.69	5.05	1.77	0.00	0.00	0.00	0.00	2.40	4.26	0.56	3.98	2.54	>0.999999999999999	1.6
Q6ZN66	Guanylate-binding protein 6 OS=Homo sapiens GN=GBP6 PE=2 SV=1	0.00	2.40	2.15	1.07	0.94	1.18	2.39	0.00	0.00	0.93	1.42	0.73	0.00	1.45	1.19	0.00	0.00	0.00	0.00	0.00	0.00	0.40	2.70	0.20	1.10	0.49	>0.999999999999999	2.2
Q16555	Dihydropyrimidinase-related protein 2 OS=Homo sapiens GN=DPYSL2 PE=1 SV=1	0.00	1.89	2.63	2.35	0.94	5.43	0.00	0.00	0.00	0.93	0.00	0.00	0.00	0.00	0.00	0.84	0.00	0.00	0.00	0.00	0.00	0.40	0.00	0.00	1.18	0.10	>0.999999999999999	11.4
Q9UNF0	Protein kinase C and casein kinase substrate in neurons protein 2 OS=Homo sapiens GN=PACSIN2 PE=1 SV=2	0.00	2.06	0.00	1.28	0.00	0.00	0.00	0.00	0.00	0.00	0.94	0.00	0.00	1.93	1.43	0.00	0.00	0.00	0.00	0.00	0.00	0.00	0.00	0.00	0.36	0.28	>0.999999999999999	1.3
Q9NQP4	Prefoldin subunit 4 OS=Homo sapiens GN=PFDN4 PE=1 SV=1	0.00	3.77	5.26	10.48	0.00	5.20	3.76	0.00	0.00	0.00	0.00	1.79	0.00	3.53	0.00	0.00	0.00	0.00	0.00	0.00	0.00	0.00	3.12	0.88	2.52	0.63	>0.999999999999999	4.0
Q8WWI1	LIM domain only protein 7 OS=Homo sapiens GN=LMO7 PE=1 SV=3	0.00	0.51	0.72	0.43	0.00	0.00	0.00	0.27	0.00	0.56	2.20	0.98	0.71	0.48	0.72	1.85	0.25	0.31	0.90	0.00	0.34	0.16	1.56	0.36	0.47	0.64	>0.999999999999999	0.7
Q8NEU8	DCC-interacting protein 13-beta OS=Homo sapiens GN=APPL2 PE=1 SV=3	0.00	1.54	5.74	0.00	0.00	4.49	0.00	0.00	0.00	3.52	3.77	0.00	0.00	0.00	0.00	0.00	0.00	0.00	0.00	0.00	0.00	0.72	0.57	0.00	1.59	0.11	>0.999999999999999	14.8
Q05655	Protein kinase C delta type OS=Homo sapiens GN=PRKCD PE=1 SV=2	0.00	1.54	2.15	1.28	0.00	0.95	0.68	0.00	0.00	0.00	0.00	0.00	0.00	0.00	3.10	0.00	0.00	0.00	0.00	0.00	0.00	0.32	1.28	0.16	0.60	0.40	>0.999999999999999	1.5
Q01581	Hydroxymethylglutaryl-CoA synthase, cytoplasmic OS=Homo sapiens GN=HMGCS1 PE=1 SV=2	0.00	2.06	2.87	3.85	1.13	1.42	0.00	0.00	0.00	1.11	0.94	0.49	0.00	0.96	2.86	0.00	0.00	0.92	0.00	0.00	0.00	0.96	0.85	0.00	1.16	0.55	>0.999999999999999	2.1
Q9H6S3	Epidermal growth factor receptor kinase substrate 8-like protein 2 OS=Homo sapiens GN=EPS8L2 PE=1 SV=2	0.00	0.69	0.00	0.00	0.00	1.89	0.00	0.36	0.00	0.00	0.63	1.39	0.00	1.28	1.91	0.00	0.00	0.00	0.72	0.00	0.00	0.00	0.57	0.52	0.41	0.42	>0.999999999999999	1.0
Q9UHD9	Ubiquilin-2 OS=Homo sapiens GN=UBQLN2 PE=1 SV=2	0.00	0.86	1.20	0.00	0.00	0.00	0.00	0.00	0.00	0.00	0.00	0.00	0.00	0.00	0.00	0.00	0.00	0.00	0.00	0.00	0.00	0.40	1.42	0.00	0.17	0.15	>0.999999999999999	1.1
Q8IWE2	Protein NOXP20 OS=Homo sapiens GN=FAM114A1 PE=1 SV=2	0.00	1.89	5.50	2.35	4.34	2.60	3.93	0.45	1.51	2.04	3.62	1.39	1.18	0.80	2.62	0.84	1.39	0.77	3.07	0.00	0.00	1.36	3.26	0.68	2.47	1.33	>0.999999999999999	1.9
P00367	Glutamate dehydrogenase 1, mitochondrial OS=Homo sapiens GN=GLUD1 PE=1 SV=2	0.00	0.86	0.00	0.00	0.00	1.18	0.00	0.00	0.00	0.00	1.73	1.39	0.00	0.80	0.00	0.84	0.00	0.00	0.00	0.00	0.00	0.00	0.00	0.20	0.43	0.15	>0.999999999999999	2.8
Q9UPY8	Microtubule-associated protein RP/EB family member 3 OS=Homo sapiens GN=MAPRE3 PE=1 SV=1	0.00	1.72	2.39	2.14	1.89	0.00	0.00	0.00	0.00	1.85	3.46	0.00	0.00	0.00	0.00	0.00	0.00	0.00	0.00	0.00	1.69	1.76	3.12	0.00	1.12	0.55	>0.999999999999999	2.0
O60888	Protein CutA OS=Homo sapiens GN=CUTA PE=1 SV=2	0.00	3.09	9.08	3.85	3.39	4.25	3.08	1.63	2.47	3.33	5.98	1.47	0.00	0.00	4.29	3.03	0.00	0.00	0.00	0.00	0.00	1.44	2.55	0.72	3.47	1.00	>0.999999999999999	3.5
P15313	V-type proton ATPase subunit B, kidney isoform OS=Homo sapiens GN=ATP6V1B1 PE=1 SV=3	0.00	1.03	0.00	2.57	0.00	0.00	0.00	0.00	0.00	0.00	0.00	0.00	0.00	0.00	0.00	0.00	0.00	0.00	0.00	0.00	0.00	1.44	0.00	0.00	0.30	0.12	>0.999999999999999	2.5
P35270	Sepiapterin reductase OS=Homo sapiens GN=SPR PE=1 SV=1	0.00	2.06	5.98	11.97	2.26	5.91	0.00	0.00	0.00	7.41	1.89	3.26	0.00	1.93	5.96	0.00	0.00	0.00	0.00	0.00	0.00	2.00	5.68	0.00	3.39	1.30	>0.999999999999999	2.6
P07686	Beta-hexosaminidase subunit beta OS=Homo sapiens GN=HEXB PE=1 SV=3	0.00	1.89	0.00	2.35	0.00	0.00	0.00	0.99	0.68	0.00	0.79	0.00	0.00	0.80	1.19	3.70	0.00	0.00	1.99	0.00	0.00	0.40	1.56	0.44	0.56	0.84	>0.999999999999999	0.7
O14929	Histone acetyltransferase type B catalytic subunit OS=Homo sapiens GN=HAT1 PE=1 SV=1	0.00	1.20	0.00	0.00	0.00	0.00	0.00	0.00	0.00	0.00	1.10	0.00	0.00	1.12	0.00	0.00	0.00	1.07	0.00	0.00	0.00	0.00	0.00	0.00	0.19	0.18	>0.999999999999999	1.0
P49419	Alpha-aminoadipic semialdehyde dehydrogenase OS=Homo sapiens GN=ALDH7A1 PE=1 SV=5	0.00	1.89	1.43	3.85	0.00	7.33	1.03	0.00	0.00	4.45	3.77	1.47	1.41	0.96	0.00	1.01	0.00	0.00	1.09	0.00	0.00	0.88	1.56	0.44	2.10	0.61	>0.999999999999999	3.4
Q9Y6G9	Cytoplasmic dynein 1 light intermediate chain 1 OS=Homo sapiens GN=DYNC1LI1 PE=1 SV=3	0.00	2.06	2.87	2.57	0.00	2.84	0.00	0.00	0.00	2.22	1.89	1.47	0.00	0.00	0.00	0.00	0.00	0.00	0.00	0.00	0.00	0.00	0.00	1.00	1.33	0.08	>0.999999999999999	15.9
Q9NP97	Dynein light chain roadblock-type 1 OS=Homo sapiens GN=DYNLRB1 PE=1 SV=3	0.00	5.49	7.65	6.84	6.03	7.56	5.47	0.00	0.00	5.93	0.00	2.61	0.00	5.14	7.63	5.38	0.00	0.00	5.79	0.00	0.00	2.56	0.00	1.28	3.97	2.31	>0.999999999999999	1.7
Q93008	Probable ubiquitin carboxyl-terminal hydrolase FAF-X OS=Homo sapiens GN=USP9X PE=1 SV=3	0.00	0.34	0.96	0.64	1.13	0.71	0.00	0.00	0.00	0.00	0.31	0.00	0.00	0.00	0.24	0.00	0.00	0.00	0.00	0.00	0.00	0.00	0.14	0.00	0.34	0.03	>0.999999999999999	10.8
P62136	Serine/threonine-protein phosphatase PP1-alpha catalytic subunit OS=Homo sapiens GN=PPP1CA PE=1 SV=1	0.00	3.09	18.88	8.34	7.35	0.00	0.00	1.63	0.00	0.00	0.00	0.00	0.00	4.50	0.00	10.94	0.00	0.00	5.06	0.00	0.00	3.12	5.53	0.00	3.27	2.43	>0.999999999999999	1.3
Q9H1E3	Nuclear ubiquitous casein and cyclin-dependent kinases substrate OS=Homo sapiens GN=NUCKS1 PE=1 SV=1	0.00	2.06	6.22	0.00	0.00	0.00	0.00	1.08	0.00	0.00	0.00	0.00	0.00	0.00	0.00	0.00	0.00	0.00	2.17	0.00	0.00	0.00	0.00	0.00	0.78	0.18	>0.999999999999999	4.3
P25325	3-mercaptopyruvate sulfurtransferase OS=Homo sapiens GN=MPST PE=1 SV=3	0.00	1.72	5.02	2.14	3.96	4.96	3.59	0.90	1.37	1.85	3.30	0.00	0.00	1.61	2.38	0.00	1.27	0.00	3.80	0.00	0.00	1.68	0.00	0.40	2.40	0.93	>0.999999999999999	2.6
P04424	Argininosuccinate lyase OS=Homo sapiens GN=ASL PE=1 SV=4	0.00	1.03	3.11	5.99	1.13	3.07	4.79	0.00	0.00	5.19	3.15	0.49	0.00	0.96	3.10	0.00	0.00	1.99	0.00	0.00	0.00	2.24	3.97	0.24	2.33	1.04	>0.999999999999999	2.2
Q96DH6	RNA-binding protein Musashi homolog 2 OS=Homo sapiens GN=MSI2 PE=1 SV=1	0.00	1.54	2.15	0.00	3.77	2.13	1.54	0.00	0.00	1.67	4.88	0.00	0.00	0.00	0.00	0.00	0.00	0.00	0.00	0.00	0.00	0.00	0.00	0.00	1.47	0.00	>0.999999999999999	#DIV/0!
Q9BPX5	Actin-related protein 2/3 complex subunit 5-like protein OS=Homo sapiens GN=ARPC5L PE=1 SV=1	0.00	3.43	10.52	9.41	8.30	10.40	7.52	3.97	0.00	8.15	3.15	3.59	4.70	7.06	10.49	3.36	2.53	0.00	3.62	0.00	0.00	5.75	10.22	0.80	5.70	4.05	>0.999999999999999	1.4
P50479	PDZ and LIM domain protein 4 OS=Homo sapiens GN=PDLIM4 PE=1 SV=2	0.00	3.26	2.15	0.00	0.00	0.00	0.00	0.00	0.00	0.00	1.42	0.00	0.00	0.00	0.00	0.00	0.00	0.00	0.00	0.00	0.00	0.72	1.28	0.00	0.57	0.17	>0.999999999999999	3.4
P78527	DNA-dependent protein kinase catalytic subunit OS=Homo sapiens GN=PRKDC PE=1 SV=3	0.00	0.17	1.91	1.92	0.75	3.07	0.34	0.18	0.00	1.11	1.10	0.16	0.00	0.16	0.72	0.50	0.13	0.15	0.18	0.00	0.00	0.24	0.28	0.00	0.89	0.20	>0.999999999999999	4.5
P54136	Arginine--tRNA ligase, cytoplasmic OS=Homo sapiens GN=RARS PE=1 SV=2	0.00	3.09	9.56	3.85	2.45	6.85	3.93	0.00	0.00	2.41	4.56	1.06	2.12	2.09	9.54	2.19	0.00	0.00	0.72	0.00	0.00	1.04	1.28	0.16	3.15	1.59	>0.999999999999999	2.0
P40855	Peroxisomal biogenesis factor 19 OS=Homo sapiens GN=PEX19 PE=1 SV=1	0.00	1.72	0.00	0.00	0.00	0.00	0.00	0.00	0.00	0.00	0.00	0.00	0.00	0.00	0.00	0.00	0.00	0.00	0.00	0.00	0.00	0.00	0.00	0.40	0.14	0.03	>0.999999999999999	4.3
P49441	Inositol polyphosphate 1-phosphatase OS=Homo sapiens GN=INPP1 PE=1 SV=1	0.00	1.20	1.67	3.21	1.32	1.65	0.00	0.00	0.00	1.30	1.10	1.22	0.00	0.00	1.67	0.00	0.89	0.00	0.00	0.00	0.00	0.00	0.99	0.28	1.06	0.32	>0.999999999999999	3.3
P54105	Methylosome subunit pICln OS=Homo sapiens GN=CLNS1A PE=1 SV=1	0.00	2.23	0.00	2.78	2.45	3.07	0.00	0.00	0.00	0.00	0.00	1.06	0.00	2.09	0.00	0.00	0.00	0.00	0.00	0.00	0.00	0.00	0.00	0.52	0.97	0.22	>0.999999999999999	4.4
O00204	Sulfotransferase family cytosolic 2B member 1 OS=Homo sapiens GN=SULT2B1 PE=1 SV=2	0.00	1.37	4.06	1.71	0.00	4.02	0.00	0.00	0.00	0.00	0.00	0.00	0.00	0.00	0.00	0.00	0.00	0.00	0.00	0.00	0.00	0.00	0.00	0.00	0.93	0.00	>0.999999999999999	#DIV/0!
P57737	Coronin-7 OS=Homo sapiens GN=CORO7 PE=1 SV=2	0.00	0.51	4.06	4.49	0.57	3.31	1.20	0.63	0.00	2.59	3.93	0.00	0.00	0.00	1.67	0.50	0.00	1.07	0.00	0.00	0.00	0.56	2.41	0.00	1.77	0.52	>0.999999999999999	3.4
P82970	High mobility group nucleosome-binding domain-containing protein 5 OS=Homo sapiens GN=HMGN5 PE=1 SV=1	0.00	1.89	2.63	0.00	0.00	5.20	0.00	0.99	0.00	6.48	1.73	0.00	0.00	0.00	0.00	0.00	0.00	0.00	0.00	0.00	0.00	0.00	0.00	0.44	1.58	0.04	>0.999999999999999	43.0
O95989	Diphosphoinositol polyphosphate phosphohydrolase 1 OS=Homo sapiens GN=NUDT3 PE=1 SV=1	0.00	2.92	4.06	3.63	3.21	4.02	0.00	0.00	0.00	3.15	2.67	0.00	0.00	0.00	0.00	0.00	0.00	0.00	0.00	0.00	0.00	1.36	0.00	0.00	1.97	0.11	>0.999999999999999	17.4
Q8WWF8	Calcyphosin-like protein OS=Homo sapiens GN=CAPSL PE=2 SV=4	0.00	4.97	6.93	10.05	8.86	0.00	2.39	0.00	0.00	8.71	7.39	2.37	0.00	2.25	6.92	0.00	0.00	0.00	0.00	0.00	0.00	3.75	4.12	0.56	4.31	1.47	>0.999999999999999	2.9
P40123	Adenylyl cyclase-associated protein 2 OS=Homo sapiens GN=CAP2 PE=1 SV=1	0.00	2.23	4.78	0.00	2.45	6.38	0.00	0.00	0.00	3.71	2.04	0.00	0.00	0.00	3.10	0.00	0.00	0.00	0.00	0.00	0.00	0.00	0.85	0.00	1.80	0.33	>0.999999999999999	5.5
Q9NVA2	Septin-11 OS=Homo sapiens GN=SEPT11 PE=1 SV=3	0.00	2.40	0.00	1.50	1.32	1.65	0.00	0.00	0.00	0.00	3.30	0.00	0.00	1.12	0.00	0.00	0.00	0.00	0.00	0.00	0.00	0.00	1.99	0.00	0.85	0.26	>0.999999999999999	3.3
P10155	60 kDa SS-A/Ro ribonucleoprotein OS=Homo sapiens GN=TROVE2 PE=1 SV=2	0.00	0.86	1.20	2.35	2.07	4.02	0.00	0.00	0.00	0.93	0.79	0.90	0.00	0.00	1.19	0.00	0.00	0.00	0.00	0.00	0.00	0.88	2.41	0.00	1.09	0.37	>0.999999999999999	2.9
Q13576	Ras GTPase-activating-like protein IQGAP2 OS=Homo sapiens GN=IQGAP2 PE=1 SV=4	0.00	0.69	0.96	1.92	0.75	1.42	0.00	0.00	0.00	0.37	1.73	0.00	0.00	0.00	0.00	0.34	0.00	0.00	0.00	0.00	0.00	0.00	0.57	0.08	0.65	0.08	>0.999999999999999	8.0
P06730	Eukaryotic translation initiation factor 4E OS=Homo sapiens GN=EIF4E PE=1 SV=2	0.00	2.23	3.11	0.00	0.00	0.00	2.22	0.00	0.00	0.00	0.00	0.00	0.00	2.09	3.10	0.00	0.00	0.00	0.00	0.00	2.19	2.24	3.97	0.00	0.63	1.13	>0.999999999999999	0.6
Q6UWP8	Suprabasin OS=Homo sapiens GN=SBSN PE=2 SV=1	0.00	2.23	0.00	0.00	0.00	0.00	0.00	0.00	0.00	0.00	0.00	0.00	0.00	2.09	0.00	2.19	1.64	0.00	0.00	0.00	0.00	0.00	0.00	0.52	0.19	0.54	>0.999999999999999	0.3
P15927	Replication protein A 32 kDa subunit OS=Homo sapiens GN=RPA2 PE=1 SV=1	0.00	1.89	2.63	2.35	0.00	2.60	0.00	0.00	0.00	0.00	0.00	0.00	0.00	0.00	2.62	0.00	0.00	0.00	0.00	0.00	0.00	0.00	0.00	0.00	0.79	0.22	>0.999999999999999	3.6
O60925	Prefoldin subunit 1 OS=Homo sapiens GN=PFDN1 PE=1 SV=2	0.00	4.12	0.00	5.13	4.53	0.00	0.00	0.00	0.00	0.00	3.77	0.00	0.00	3.85	0.00	0.00	0.00	0.00	0.00	0.00	0.00	0.00	0.00	0.00	1.46	0.32	>0.999999999999999	4.6
Q69YN2	CWF19-like protein 1 OS=Homo sapiens GN=CWF19L1 PE=1 SV=2	0.00	0.86	0.00	0.00	0.00	0.00	0.00	0.00	0.00	0.00	0.00	0.00	0.00	0.00	0.00	0.00	0.00	0.00	0.00	0.00	0.00	0.00	0.00	0.00	0.07	0.00	>0.999999999999999	#DIV/0!
Q5T6V5	UPF0553 protein C9orf64 OS=Homo sapiens GN=C9orf64 PE=1 SV=1	0.00	2.92	1.91	0.00	0.00	4.02	0.00	0.00	0.00	0.00	0.00	0.00	0.00	1.28	1.91	0.00	0.00	0.00	0.00	0.00	0.00	0.64	1.14	0.32	0.74	0.44	>0.999999999999999	1.7
P07311	Acylphosphatase-1 OS=Homo sapiens GN=ACYP1 PE=1 SV=2	0.00	5.32	7.41	6.63	0.00	16.78	0.00	0.00	0.00	5.74	11.17	0.00	0.00	4.98	7.39	0.00	0.00	0.00	0.00	0.00	0.00	2.48	4.40	0.00	4.42	1.60	>0.999999999999999	2.8
O94874	E3 UFM1-protein ligase 1 OS=Homo sapiens GN=UFL1 PE=1 SV=2	0.00	1.20	0.96	2.35	0.75	1.65	1.20	0.00	0.55	0.00	0.63	0.00	0.00	0.00	1.67	0.00	0.00	0.00	0.00	0.00	0.00	0.00	0.00	0.16	0.77	0.15	>0.999999999999999	5.1
Q8TDQ7	Glucosamine-6-phosphate isomerase 2 OS=Homo sapiens GN=GNPDA2 PE=1 SV=1	0.00	1.89	5.26	4.70	6.60	8.27	1.88	0.99	0.00	2.04	5.50	0.90	0.00	3.53	0.00	1.85	0.00	3.38	1.99	0.00	0.00	0.88	3.12	0.44	3.17	1.27	>0.999999999999999	2.5
Q9UKY7	Protein CDV3 homolog OS=Homo sapiens GN=CDV3 PE=1 SV=1	0.00	2.06	2.87	5.56	2.26	0.00	2.05	0.00	0.00	2.22	1.89	0.00	0.00	0.00	2.86	2.02	0.00	0.00	0.00	0.00	0.00	0.96	0.00	0.00	1.58	0.49	>0.999999999999999	3.2
P06681	Complement C2 OS=Homo sapiens GN=C2 PE=1 SV=2	0.00	0.69	0.00	0.86	1.51	1.89	0.68	0.36	1.10	1.48	0.63	2.12	1.88	1.28	0.95	1.35	1.01	1.23	1.45	0.52	0.68	0.64	0.00	0.32	0.94	0.94	>0.999999999999999	1.0
P06331	Ig heavy chain V-II region ARH-77 OS=Homo sapiens PE=4 SV=1	0.00	3.60	0.00	4.49	0.00	0.00	0.00	1.90	2.88	3.89	3.30	0.00	4.94	3.37	5.01	3.53	0.00	0.00	3.80	2.73	3.54	1.68	2.98	0.00	1.67	2.63	>0.999999999999999	0.6
Q9UBW8	COP9 signalosome complex subunit 7a OS=Homo sapiens GN=COPS7A PE=1 SV=1	0.00	3.95	2.63	2.35	0.00	5.43	1.88	0.00	0.00	2.04	0.00	0.90	0.00	0.00	0.00	0.00	0.00	0.00	0.00	0.00	0.00	0.00	1.56	0.00	1.60	0.13	>0.999999999999999	12.3
O76094	Signal recognition particle 72 kDa protein OS=Homo sapiens GN=SRP72 PE=1 SV=3	0.00	0.69	2.15	0.86	0.75	0.95	1.54	0.00	0.00	0.00	1.42	0.73	0.00	0.64	0.95	0.00	0.00	0.00	0.00	0.00	0.00	0.00	0.00	0.16	0.76	0.15	>0.999999999999999	5.2
Q5SXH7	PH domain-containing protein C10orf81 OS=Homo sapiens GN=C10orf81 PE=1 SV=2	0.00	1.03	4.78	1.28	0.00	6.62	1.03	0.00	0.00	2.41	2.04	0.49	0.00	0.00	0.00	0.00	0.00	0.00	0.00	0.00	0.00	0.48	0.85	0.00	1.64	0.11	>0.999999999999999	14.8
P42025	Beta-centractin OS=Homo sapiens GN=ACTR1B PE=1 SV=1	0.00	1.37	3.82	7.48	3.02	5.91	0.00	0.72	0.00	1.48	5.50	0.00	0.00	0.00	5.96	0.00	0.00	0.00	0.00	0.00	0.00	2.00	3.55	0.00	2.44	0.96	>0.999999999999999	2.5
Q9Y570	Protein phosphatase methylesterase 1 OS=Homo sapiens GN=PPME1 PE=1 SV=3	0.00	2.74	1.91	0.00	1.51	0.00	0.00	0.00	0.00	0.00	1.26	0.00	0.00	0.00	0.00	0.00	0.00	0.00	0.00	0.00	0.00	1.28	0.00	0.64	0.62	0.16	>0.999999999999999	3.9
P09455	Retinol-binding protein 1 OS=Homo sapiens GN=RBP1 PE=1 SV=2	0.00	8.06	0.00	4.49	3.96	0.00	0.00	1.90	0.00	0.00	3.30	0.00	0.00	7.55	5.01	3.53	5.95	0.00	3.80	0.00	0.00	3.75	6.67	0.00	1.81	3.02	>0.999999999999999	0.6
O95834	Echinoderm microtubule-associated protein-like 2 OS=Homo sapiens GN=EML2 PE=2 SV=1	0.00	1.54	4.78	6.63	2.64	5.91	3.42	0.81	1.23	2.59	11.17	0.41	2.12	3.21	8.82	2.36	0.63	2.15	0.90	0.00	0.00	3.43	3.55	0.00	3.43	2.26	>0.999999999999999	1.5
Q9Y5S9	RNA-binding protein 8A OS=Homo sapiens GN=RBM8A PE=1 SV=1	0.00	2.92	0.00	3.63	0.00	8.74	0.00	0.00	0.00	0.00	2.67	1.39	0.00	0.00	4.05	0.00	2.15	0.00	0.00	0.00	0.00	0.00	2.41	0.00	1.61	0.72	>0.999999999999999	2.2
P61513	60S ribosomal protein L37a OS=Homo sapiens GN=RPL37A PE=1 SV=2	0.00	5.66	7.89	7.06	0.00	0.00	0.00	0.00	0.00	0.00	5.19	0.00	0.00	5.30	0.00	0.00	0.00	0.00	0.00	0.00	0.00	0.00	0.00	0.00	2.15	0.44	>0.999999999999999	4.9

Q96LJ8	UBX domain-containing protein 10 OS=Homo sapiens GN=UBXN10 PE=2 SV=1	0.00	3.95	11.95	0.00	0.00	2.60	0.00	0.00	0.00	0.00	0.00	0.00	0.00	0.00	0.00	0.00	0.00	0.00	0.00	0.00	0.00	0.00	0.00	0.00	1.54	0.00	>0.999999999999999	#DIV/0!
Q4LDG9	Dynein light chain 1, axonemal OS=Homo sapiens GN=DNAL1 PE=1 SV=1	0.00	2.74	3.82	3.42	3.02	3.78	0.00	0.00	0.00	2.96	2.52	1.31	0.00	0.00	0.00	0.00	0.00	0.00	0.00	0.00	0.00	0.00	0.00	0.00	1.96	0.00	>0.999999999999999	#DIV/0!
Q9UI42	Carboxypeptidase A4 OS=Homo sapiens GN=CPA4 PE=1 SV=2	0.00	1.20	0.00	0.00	0.00	0.00	0.00	0.63	0.00	0.00	0.00	0.00	0.00	2.25	0.00	1.18	0.00	0.00	0.00	0.00	0.00	0.56	0.00	0.28	0.15	0.36	>0.999999999999999	0.4
Q9H4A6	Golgi phosphoprotein 3 OS=Homo sapiens GN=GOLPH3 PE=1 SV=1	0.00	1.72	0.00	6.84	0.00	0.00	1.71	0.90	0.00	0.00	3.15	0.00	0.00	0.00	0.00	0.00	0.00	0.00	0.00	0.00	0.00	0.00	0.00	0.00	1.19	0.00	>0.999999999999999	#DIV/0!
Q12765	Secernin-1 OS=Homo sapiens GN=SCRN1 PE=1 SV=2	0.00	1.20	3.59	3.21	2.83	1.65	1.20	0.00	0.00	1.30	3.62	0.57	0.00	2.41	1.67	0.00	0.00	0.00	0.00	0.00	0.00	0.00	4.40	0.28	1.60	0.73	>0.999999999999999	2.2
O75822	Eukaryotic translation initiation factor 3 subunit J OS=Homo sapiens GN=EIF3J PE=1 SV=2	0.00	1.89	0.00	0.00	0.00	0.00	1.88	0.00	0.00	0.00	0.00	0.90	0.00	0.00	0.00	1.85	0.00	0.00	0.00	0.00	0.00	0.88	0.00	0.44	0.39	0.26	>0.999999999999999	1.5
Q53FT3	Uncharacterized protein C11orf73 OS=Homo sapiens GN=C11orf73 PE=1 SV=2	0.00	2.74	0.00	0.00	0.00	3.78	0.00	0.00	0.00	0.00	0.00	0.00	0.00	0.00	0.00	0.00	0.00	0.00	0.00	0.00	0.00	0.00	0.00	0.00	0.54	0.00	>0.999999999999999	#DIV/0!
Q8WZA0	Protein LZIC OS=Homo sapiens GN=LZIC PE=1 SV=1	0.00	2.74	3.82	3.42	0.00	3.78	5.81	0.00	0.00	2.96	0.00	1.31	0.00	0.00	0.00	0.00	0.00	0.00	0.00	0.00	0.00	4.31	2.27	0.00	1.99	0.55	>0.999999999999999	3.6
Q8WX92	Negative elongation factor B OS=Homo sapiens GN=COBRA1 PE=1 SV=1	0.00	0.86	1.20	1.07	0.00	1.18	0.00	0.00	0.00	0.00	0.79	0.00	0.00	0.80	1.19	0.00	0.00	0.00	0.00	0.00	0.00	0.00	0.00	0.00	0.42	0.17	>0.999999999999999	2.6
Q9BW04	Specifically androgen-regulated gene protein OS=Homo sapiens GN=SARG PE=1 SV=2	0.00	0.86	0.00	0.00	0.00	0.00	0.00	0.00	0.00	0.00	0.00	0.00	0.00	0.00	1.19	0.00	0.00	0.00	0.00	0.00	0.00	0.00	0.00	0.00	0.07	0.10	>0.999999999999999	0.7
O43633	Charged multivesicular body protein 2a OS=Homo sapiens GN=CHMP2A PE=1 SV=1	0.00	2.23	3.11	2.78	2.45	0.00	2.22	1.26	0.00	5.19	7.23	0.00	0.00	0.00	3.34	2.19	0.00	0.00	2.35	0.00	0.00	0.00	1.99	0.52	2.21	0.87	>0.999999999999999	2.5
Q8N4P3	Guanosine-3',5'-bis(diphosphate) 3'-pyrophosphohydrolase MESH1 OS=Homo sapiens GN=HDDC3 PE=1 SV=3	0.00	2.74	0.00	3.42	3.02	3.78	0.00	1.44	0.00	0.00	0.00	0.00	0.00	0.00	3.82	2.69	0.00	0.00	0.00	0.00	0.00	0.00	0.00	0.00	1.20	0.54	>0.999999999999999	2.2
Q13685	Angio-associated migratory cell protein OS=Homo sapiens GN=AAMP PE=1 SV=2	0.00	1.20	0.00	0.00	0.00	0.00	0.00	0.00	0.00	0.00	0.00	0.00	0.00	0.00	0.00	0.00	0.00	0.00	0.00	0.00	0.00	0.00	0.00	0.00	0.10	0.00	>0.999999999999999	#DIV/0!
Q9H069	Leucine-rich repeat-containing protein 48 OS=Homo sapiens GN=LRRC48 PE=2 SV=2	0.00	0.86	0.00	1.07	0.00	0.00	0.00	0.00	0.00	2.04	0.00	0.00	0.00	0.80	0.00	0.00	0.00	0.00	0.00	0.00	0.00	0.00	0.71	0.00	0.33	0.13	>0.999999999999999	2.6
P13667	Protein disulfide-isomerase A4 OS=Homo sapiens GN=PDIA4 PE=1 SV=2	0.00	1.54	0.96	2.99	0.75	3.31	0.00	0.81	0.00	0.74	1.42	2.45	0.94	0.64	0.95	0.67	1.14	0.00	1.63	0.00	0.68	0.72	0.57	1.20	1.25	0.76	>0.999999999999999	1.6
Q9H2H8	Peptidyl-prolyl cis-trans isomerase-like 3 OS=Homo sapiens GN=PPIL3 PE=1 SV=1	0.00	3.09	0.00	3.85	0.00	0.00	0.00	0.00	0.00	0.00	2.83	0.00	0.00	2.89	4.29	3.03	0.00	0.00	3.26	0.00	0.00	0.00	2.55	0.72	0.81	1.40	>0.999999999999999	0.6
P21399	Cytoplasmic aconitate hydratase OS=Homo sapiens GN=ACO1 PE=1 SV=3	0.00	1.20	2.39	2.99	0.00	2.36	1.71	0.00	0.41	1.30	0.47	0.57	0.00	0.48	1.67	1.18	0.00	0.00	0.54	0.00	0.00	1.44	0.99	0.12	1.12	0.54	>0.999999999999999	2.1
P63098	Calcineurin subunit B type 1 OS=Homo sapiens GN=PPP3R1 PE=1 SV=2	0.00	2.92	9.08	0.00	0.00	4.02	0.00	0.00	0.00	0.00	2.67	3.10	0.00	2.73	0.00	0.00	0.00	0.00	3.07	0.00	0.00	1.36	2.41	0.68	1.82	0.85	>0.999999999999999	2.1
Q9C0C2	182 kDa tankyrase-1-binding protein OS=Homo sapiens GN=TNKS1BP1 PE=1 SV=4	0.00	0.34	0.00	0.43	0.00	0.47	0.00	0.00	0.00	0.37	0.00	0.00	0.00	0.00	0.00	0.00	0.00	0.00	0.00	0.00	0.00	0.00	0.00	0.16	0.13	0.01	>0.999999999999999	10.1
Q9NR46	Endophilin-B2 OS=Homo sapiens GN=SH3GLB2 PE=1 SV=1	0.00	2.74	1.67	1.50	1.32	0.00	2.74	0.00	0.00	1.30	1.10	0.00	0.00	0.00	1.67	0.00	0.00	0.00	0.00	0.00	0.00	0.56	0.99	0.00	1.03	0.27	>0.999999999999999	3.8
Q92945	Far upstream element-binding protein 2 OS=Homo sapiens GN=KHSRP PE=1 SV=4	0.00	1.54	2.15	2.99	3.58	4.49	1.54	1.26	0.55	1.67	3.77	0.73	0.00	0.00	2.15	0.67	0.00	0.61	0.00	0.00	0.00	0.32	2.70	0.16	2.02	0.55	>0.999999999999999	3.7
Q96S44	TP53-regulating kinase OS=Homo sapiens GN=TP53RK PE=1 SV=2	0.00	2.06	2.87	2.57	0.00	2.84	0.00	0.00	0.00	2.22	1.89	0.00	0.00	0.00	0.00	0.00	0.00	0.00	0.00	0.00	0.00	0.00	0.00	0.00	1.20	0.00	>0.999999999999999	#DIV/0!
P78344	Eukaryotic translation initiation factor 4 gamma 2 OS=Homo sapiens GN=EIF4G2 PE=1 SV=1	0.00	1.03	0.00	0.00	1.13	0.00	0.00	0.00	0.00	0.00	0.47	0.00	0.00	0.00	1.43	0.00	0.00	0.00	0.54	0.00	0.00	0.24	0.85	0.00	0.22	0.26	>0.999999999999999	0.9
Q5RHP9	Uncharacterized protein C1orf173 OS=Homo sapiens GN=C1orf173 PE=2 SV=1	0.00	0.34	2.39	0.86	0.00	1.42	0.00	0.00	0.00	1.11	1.57	0.00	0.00	0.00	0.48	0.00	0.00	0.00	0.00	0.00	0.00	0.16	1.14	0.00	0.64	0.15	>0.999999999999999	4.3
O15212	Prefoldin subunit 6 OS=Homo sapiens GN=PFDN6 PE=1 SV=1	0.00	3.95	5.50	4.92	4.34	5.43	0.00	0.00	0.00	4.26	3.62	4.24	0.00	3.69	5.49	3.87	2.91	0.00	4.16	0.00	0.00	1.84	3.26	0.92	3.02	2.18	>0.999999999999999	1.4
P14649	Myosin light chain 6B OS=Homo sapiens GN=MYL6B PE=1 SV=1	0.00	2.57	0.00	0.00	0.00	0.00	0.00	0.00	2.05	0.00	0.00	0.00	0.00	2.41	0.00	0.00	0.00	0.00	0.00	0.00	0.00	0.00	0.00	0.00	0.39	0.20	>0.999999999999999	1.9
O43684	Mitotic checkpoint protein BUB3 OS=Homo sapiens GN=BUB3 PE=1 SV=1	0.00	1.54	0.00	1.92	1.70	2.13	0.00	0.00	0.00	3.33	1.42	0.00	0.00	0.00	0.00	0.00	0.00	0.00	0.00	0.00	0.00	0.72	0.00	0.36	1.00	0.09	>0.999999999999999	11.2
P53602	Diphosphomevalonate decarboxylase OS=Homo sapiens GN=MVD PE=1 SV=1	0.00	1.37	1.91	1.71	0.00	1.89	0.00	0.00	0.00	1.48	1.26	0.00	0.00	1.28	0.00	0.00	0.00	0.00	0.00	0.00	0.00	0.64	1.14	0.00	0.80	0.25	>0.999999999999999	3.1
Q9BTW9	Tubulin-specific chaperone D OS=Homo sapiens GN=TBCD PE=1 SV=2	0.00	0.34	0.48	0.43	0.00	0.47	0.00	0.00	0.00	0.00	0.31	0.00	0.00	0.00	0.00	0.00	0.00	0.00	0.00	0.00	0.00	0.00	0.00	0.00	0.17	0.00	>0.999999999999999	#DIV/0!
P47897	Glutamine--tRNA ligase OS=Homo sapiens GN=QARS PE=1 SV=1	0.00	0.69	3.82	2.35	2.07	3.78	1.37	0.00	0.00	2.04	1.73	1.63	2.59	0.00	2.62	1.35	0.51	0.00	0.00	0.00	0.00	0.00	1.14	0.64	1.62	0.74	>0.999999999999999	2.2
P53611	Geranylgeranyl transferase type-2 subunit beta OS=Homo sapiens GN=RABGGTB PE=1 SV=2	0.00	1.54	2.15	1.92	1.70	0.00	0.00	0.00	0.00	1.67	0.00	0.00	0.00	0.00	0.00	1.51	1.14	1.38	1.63	0.00	0.00	0.72	0.00	0.00	0.75	0.53	>0.999999999999999	1.4
Q9HB40	Retinoid-inducible serine carboxypeptidase OS=Homo sapiens GN=SCPEP1 PE=1 SV=1	0.00	1.03	0.00	1.28	1.13	1.42	1.03	0.54	0.82	1.11	0.94	0.49	0.00	0.96	0.00	2.19	0.00	0.00	1.09	0.00	0.00	0.48	0.85	0.00	0.82	0.46	>0.999999999999999	1.8
Q9NX46	Poly(ADP-ribose) glycohydrolase ARH3 OS=Homo sapiens GN=ADPRHL2 PE=1 SV=1	0.00	1.37	4.30	1.71	3.39	1.89	3.08	0.00	0.00	1.48	0.00	0.65	0.00	0.00	1.91	0.00	0.00	0.00	0.00	0.00	0.00	0.64	1.14	0.00	1.49	0.31	>0.999999999999999	4.9
P46379	Large proline-rich protein BAG6 OS=Homo sapiens GN=BAG6 PE=1 SV=2	0.00	0.51	1.43	1.28	1.13	0.71	0.00	0.00	0.00	0.00	0.00	0.00	0.00	0.00	0.72	0.00	0.00	0.00	0.00	0.00	0.00	0.00	0.43	0.00	0.42	0.10	>0.999999999999999	4.4
Q8WW22	DnaJ homolog subfamily A member 4 OS=Homo sapiens GN=DNAJA4 PE=1 SV=1	0.00	1.20	10.04	4.92	0.00	9.92	0.00	0.00	0.00	1.30	1.10	0.00	0.00	1.12	1.67	0.00	0.00	0.00	0.00	0.00	0.00	0.56	0.99	0.00	2.37	0.36	>0.999999999999999	6.6
Q9P2J5	Leucine--tRNA ligase, cytoplasmic OS=Homo sapiens GN=LARS PE=1 SV=2	0.00	0.86	2.39	1.50	0.38	1.65	0.34	0.00	0.00	1.30	1.57	0.41	0.00	0.32	1.19	0.84	0.00	0.00	0.36	0.00	0.00	0.00	0.28	0.00	0.87	0.25	>0.999999999999999	3.5
Q92696	Geranylgeranyl transferase type-2 subunit alpha OS=Homo sapiens GN=RABGGTA PE=1 SV=2	0.00	0.86	5.02	2.14	1.89	1.18	0.85	0.45	0.00	0.93	2.52	0.41	0.00	1.61	1.19	0.00	0.00	0.00	0.00	0.00	0.00	1.28	2.27	0.00	1.35	0.53	>0.999999999999999	2.6
Q6V1X1	Dipeptidyl peptidase 8 OS=Homo sapiens GN=DPP8 PE=1 SV=1	0.00	0.51	0.72	0.64	0.57	0.71	0.00	0.00	0.00	0.00	0.47	0.00	0.00	0.00	0.72	0.00	0.00	0.00	0.00	0.00	0.00	0.00	0.00	0.00	0.30	0.06	>0.999999999999999	5.1
P35813	Protein phosphatase 1A OS=Homo sapiens GN=PPM1A PE=1 SV=1	0.00	1.37	3.82	3.42	1.51	0.00	2.74	0.00	0.00	0.00	1.26	0.00	0.00	0.00	3.82	0.00	0.00	0.00	0.00	0.00	0.00	0.00	1.14	0.32	1.18	0.44	>0.999999999999999	2.7
O15231	Zinc finger protein 185 OS=Homo sapiens GN=ZNF185 PE=1 SV=3	0.00	0.69	0.00	0.86	0.00	0.00	0.00	0.00	0.00	0.00	0.00	0.00	0.00	0.00	0.00	2.36	0.51	0.00	0.72	0.00	0.00	0.00	1.99	0.56	0.13	0.51	>0.999999999999999	0.3
P08571	Monocyte differentiation antigen CD14 OS=Homo sapiens GN=CD14 PE=1 SV=2	0.00	1.37	0.00	0.00	0.00	0.00	0.00	0.72	0.00	0.00	0.00	1.39	0.00	0.00	0.00	2.86	0.00	1.23	0.00	0.00	0.00	0.64	1.14	1.04	0.29	0.58	>0.999999999999999	0.5
P52788	Spermine synthase OS=Homo sapiens GN=SMS PE=1 SV=2	0.00	2.74	6.22	9.83	3.02	6.14	0.00	0.72	1.10	8.52	9.12	4.73	0.00	2.57	3.82	0.00	0.00	1.23	1.45	0.00	1.35	2.08	3.69	0.32	4.35	1.37	>0.999999999999999	3.2
Q9BVG4	UPF0368 protein Cxorf26 OS=Homo sapiens GN=CXorf26 PE=1 SV=1	0.00	4.63	0.00	0.00	0.00	2.45	0.00	0.00	1.17	0.00	2.41	0.00	0.00	0.00	0.00	0.00	0.00	0.00	0.00	0.00	0.00	0.00	0.00	1.08	0.89	0.09	>0.999999999999999	9.9
Q96IZ0	PRKC apoptosis WT1 regulator protein OS=Homo sapiens GN=PAWR PE=1 SV=1	0.00	1.54	0.00	0.00	0.00	0.00	0.00	0.00	0.00	0.00	0.00	0.00	0.00	0.00	0.00	0.00	0.00	0.00	0.00	0.00	0.00	0.00	0.00	0.00	0.13	0.00	>0.999999999999999	#DIV/0!
P50583	Bis(5'-nucleosyl)-tetraphosphatase [asymmetrical] OS=Homo sapiens GN=NUDT2 PE=1 SV=3	0.00	3.43	4.78	4.28	3.77	4.73	3.42	0.00	2.74	3.71	0.00	1.63	0.00	3.21	4.77	0.00	0.00	0.00	0.00	0.00	0.00	0.00	0.00	0.00	2.71	0.67	>0.999999999999999	4.1
Q9H4A3	Serine/threonine-protein kinase WNK1 OS=Homo sapiens GN=WNK1 PE=1 SV=2	0.00	0.17	0.24	0.21	0.19	0.00	0.00	0.00	0.00	0.19	0.00	0.00	0.00	0.00	0.00	0.00	0.00	0.00	0.00	0.00	0.00	0.08	0.43	0.00	0.08	0.04	>0.999999999999999	2.0
P30990	Neurotensin/neuromedin N OS=Homo sapiens GN=NTS PE=1 SV=2	0.00	2.92	4.06	3.63	0.00	4.02	0.00	0.00	0.00	0.00	0.00	0.00	0.00	0.00	0.00	0.00	0.00	0.00	0.00	0.00	0.00	0.00	0.00	0.00	1.22	0.00	>0.999999999999999	#DIV/0!
Q08J23	tRNA (cytosine(34)-C(5))-methyltransferase OS=Homo sapiens GN=NSUN2 PE=1 SV=2	0.00	0.69	1.91	0.86	0.00	0.95	0.00	0.00	0.00	0.00	0.00	0.00	0.00	0.00	0.95	0.00	0.00	0.00	0.00	0.00	0.00	0.32	0.57	0.00	0.37	0.15	>0.999999999999999	2.4
Q9H9Q2	COP9 signalosome complex subunit 7b OS=Homo sapiens GN=COPS7B PE=1 SV=1	0.00	1.89	2.63	2.35	0.00	0.00	0.00	0.00	0.00	0.00	0.00	0.00	0.00	0.00	0.00	0.00	0.00	0.00	0.00	0.00	0.00	0.00	0.00	0.00	0.57	0.00	>0.999999999999999	#DIV/0!
Q14157	Ubiquitin-associated protein 2-like OS=Homo sapiens GN=UBAP2L PE=1 SV=2	0.00	0.51	0.00	0.64	0.00	0.00	0.51	0.00	0.41	0.00	0.47	0.00	0.00	0.00	0.72	0.00	0.00	0.00	0.00	0.00	0.00	0.00	0.43	0.00	0.21	0.10	>0.999999999999999	2.2
Q9UI12	V-type proton ATPase subunit H OS=Homo sapiens GN=ATP6V1H PE=1 SV=1	0.00	2.06	1.43	0.00	1.13	0.00	0.00	0.00	0.00	1.11	0.00	0.00	0.00	0.00	0.00	1.01	0.00	0.00	2.17	0.00	0.00	0.00	0.00	0.24	0.48	0.29	>0.999999999999999	1.7
P28161	Glutathione S-transferase Mu 2 OS=Homo sapiens GN=GSTM2 PE=1 SV=2	0.00	2.23	10.52	0.00	0.00	0.00	0.00	0.00	0.00	0.00	4.25	0.00	0.00	2.09	0.00	0.00	0.00	0.00	0.00	0.00	0.00	0.00	0.00	0.00	1.42	0.17	>0.999999999999999	8.1
Q13442	28 kDa heat- and acid-stable phosphoprotein OS=Homo sapiens GN=PDAP1 PE=1 SV=1	0.00	2.74	13.63	3.42	10.75	13.47	5.98	3.16	4.79	2.96	2.52	2.85	3.76	2.57	8.35	2.69	4.43	2.45	2.89	0.00	2.70	2.80	4.97	0.64	5.52	3.19	>0.999999999999999	1.7
Q8WWY7	WAP four-disulfide core domain protein 12 OS=Homo sapiens GN=WFDC12 PE=2 SV=1	0.00	4.63	0.00	0.00	0.00	0.00	0.00	0.00	0.00	0.00	0.00	0.00	0.00	0.00	0.00	0.00	0.00	0.00	0.00	0.00	0.00	0.00	0.00	0.00	0.39	0.00	>0.999999999999999	#DIV/0!
Q8NFW8	N-acylneuraminate cytidylyltransferase OS=Homo sapiens GN=CMAS PE=1 SV=2	0.00	1.20	0.00	1.50	1.32	1.65	0.00	0.00	0.00	0.00	2.20	0.00	0.00	0.00	1.67	0.00	0.00	0.00	2.53	0.00	0.00	0.00	0.99	0.00	0.66	0.43	>0.999999999999999	1.5
Q9P258	Protein RCC2 OS=Homo sapiens GN=RCC2 PE=1 SV=2	0.00	1.03	1.43	0.00	2.26	1.42	2.05	0.00	0.00	0.00	0.00	0.00	0.00	0.00	0.00	0.00	0.00	0.00	0.00	0.00	0.00	0.00	0.00	0.24	0.68	0.02	>0.999999999999999	34.1
Q96NG3	Tetratricopeptide repeat protein 25 OS=Homo sapiens GN=TTC25 PE=1 SV=2	0.00	0.69	2.15	0.00	0.00	0.95	0.00	0.00	0.00	0.00	0.63	0.00	0.00	0.00	0.00	0.00	0.00	0.00	0.00	0.00	0.00	0.00	0.00	0.00	0.37	0.00	>0.999999999999999	#DIV/0!
P55042	GTP-binding protein RAD OS=Homo sapiens GN=RRAD PE=1 SV=2	0.00	1.72	2.39	0.00	3.96	0.00	3.59	0.00	0.00	1.85	3.30	0.00	0.00	0.00	2.38	0.00	0.00	0.00	0.00	0.00	0.00	0.00	1.42	0.00	1.40	0.32	>0.999999999999999	4.4
P50552	Vasodilator-stimulated phosphoprotein OS=Homo sapiens GN=VASP PE=1 SV=3	0.00	1.37	1.91	0.00	1.51	0.00	1.37	2.44	2.33	0.00	1.26	0.65	1.88	0.00	0.00	1.35	0.00	2.61	0.00	2.21	6.25	0.64	2.41	1.08	1.07	1.54	>0.999999999999999	0.7
O15347	High mobility group protein B3 OS=Homo sapiens GN=HMGB3 PE=1 SV=4	0.00	2.57	7.41	6.63	2.83	7.33	0.00	0.00	2.05	2.78	0.00	1.22	3.53	0.00	3.58	0.00	0.00	0.00	2.71	0.00	0.00	0.00	2.13	0.60	2.74	1.05	>0.999999999999999	2.6
P48739	Phosphatidylinositol transfer protein beta isoform OS=Homo sapiens GN=PITPNB PE=1 SV=2	0.00	1.72	5.26	4.70	0.00	5.20	1.71	0.00	0.00	1.85	0.00	0.82	0.00	1.61	5.25	1.68	0.00	0.00	1.81	0.00	0.00	0.80	3.12	0.00	1.77	1.19	>0.999999999999999	1.5
O60825	6-phosphofructo-2-kinase/fructose-2,6-bisphosphatase 2 OS=Homo sapiens GN=PFKFB2 PE=1 SV=2	0.00	1.03	1.43	0.00	1.13	1.42	0.00	0.00	0.00	0.00	0.94	0.49	0.00	0.00	1.43	0.00	0.00	0.00	0.00	0.00	0.00	0.00	0.85	0.24	0.54	0.21	>0.999999999999999	2.6
Q53GD3	Choline transporter-like protein 4 OS=Homo sapiens GN=SLC44A4 PE=2 SV=2	0.00	1.37	0.96	0.00	0.00	0.00	0.00	0.00	0.00	0.00	0.63	0.00	0.00	0.00	0.00	0.00	0.00	0.00	0.00	0.00	0.00	0.32	0.00	0.00	0.25	0.03	>0.999999999999999	9.3
Q00535	Cyclin-dependent kinase 5 OS=Homo sapiens GN=CDK5 PE=1 SV=3	0.00	1.72	0.00	0.00	0.00	0.00	0.00	0.00	0.00	0.00	0.00	0.00	0.00	0.00	0.00	0.00	0.00	0.00	0.00	0.00	0.00	0.00	0.00	0.00	0.14	0.00	>0.999999999999999	#DIV/0!
A6NDG6	Phosphoglycolate phosphatase OS=Homo sapiens GN=PGP PE=1 SV=1	0.00	1.72	2.39	2.14	1.89	0.00	0.00	0.00	0.00	1.85	1.57	0.00	0.00	0.00	4.77	0.00	0.00	0.00	0.00	0.00	0.00	1.60	2.84	0.00	0.96	0.77	>0.999999999999999	1.3
P62917	60S ribosomal protein L8 OS=Homo sapiens GN=RPL8 PE=1 SV=2	0.00	4.29	2.87	2.57	4.71	5.91	2.05	0.00	0.00	0.00	6.29	0.00	0.00	1.93	5.96	0.00	0.00	0.00	0.00	0.00	0.00	0.00	1.70	0.00	2.39	0.80	>0.999999999999999	3.0
Q9UL15	BAG family molecular chaperone regulator 5 OS=Homo sapiens GN=BAG5 PE=1 SV=1	0.00	1.03	3.11	1.28	0.00	3.07	1.03	0.00	0.00	0.00	0.94	0.00	0.00	0.00	0.00	0.00	0.00	0.00	0.00	0.00	0.00	0.00	0.00	0.00	0.87	0.00	>0.999999999999999	#DIV/0!
Q15785	Mitochondrial import receptor subunit TOM34 OS=Homo sapiens GN=TOMM34 PE=1 SV=2	0.00	1.54	4.78	6.63	3.77	10.40	0.00	0.00	0.00	1.67	16.67	0.73	0.00	0.00	2.15	0.00	1.14	0.00	0.00	0.00	0.00	0.00	4.40	0.36	3.85	0.67	>0.999999999999999	5.7
P49862	Kallikrein-7 OS=Homo sapiens GN=KLK7 PE=1 SV=1	0.00	2.06	0.00	0.00	0.00	0.00	0.00	0.00	0.00	0.00	0.00	0.00	0.00	1.93	0.00	0.00	0.00	0.00	0.00	0.00	0.00	0.96	0.00	0.00	0.17	0.24	>0.999999999999999	0.7
Q9NPF4	Probable tRNA threonylcarbamoyladenosine biosynthesis protein OSGEP OS=Homo sapiens GN=OSGEP PE=1 S\	0.00	1.54	2.15	1.92	0.00	2.13	0.00	0.00	0.00	1.67	1.42	0.00	0.00	0.00	0.00	0.00	0.00	0.00	0.00	0.00	0.00	0.72	1.28	0.00	0.90	0.17	>0.999999999999999	5.4
Q15717	ELAV-like protein 1 OS=Homo sapiens GN=ELAVL1 PE=1 SV=2	0.00	1.54	0.00	4.06	1.70	4.49	1.54	0.81	0.00	1.67	2.99	0.73	0.00	0.00	2.15	0.00	0.00	1.38	1.63	0.00	0.00	0.00	2.70	0.00	1.63	0.65	>0.999999999999999	2.5
Q9H2U2	Inorganic pyrophosphatase 2, mitochondrial OS=Homo sapiens GN=PPA2 PE=1 SV=2	0.00	1.54	4.30	1.92	0.00	0.00	0.00	0.00	0.00	0.00	0.00	0.73	0.00	0.00	0.00	0.00	0.00	0.00	3.26	0.00	0.00	0.00	0.00	0.72	0.71	0.33	>0.999999999999999	2.1
Q9NS86	LanC-like protein 2 OS=Homo sapiens GN=LANCL2 PE=1 SV=1	0.00	1.03	4.78	0.00	0.00	1.42	0.00	0.00	0.00	1.11	0.00	0.00	0.00	0.00	1.43	0.00	0.00	0.00	0.00	0.00	0.00	0.00	0.00	0.00	0.69	0.12	>0.999999999999999	5.8
Q13098	COP9 signalosome complex subunit 1 OS=Homo sapiens GN=GPS1 PE=1 SV=4	0.00	2.06	6.22	4.06	1.13	0.00	2.05	0.00	0.00	2.22	0.94	0.49	0.00	0.96	2.86	0.00	0.00	0.00	0.00	0.00	0.00	0.96	2.70	0.24	1.60	0.64	>0.999999999999999	2.5
Q9GZS3	WD repeat-containing protein 61 OS=Homo sapiens GN=WDR61 PE=1 SV=1	0.00	1.72	2.39	2.14	1.89	4.96	0.00	0.00	0.00	3.89	1.57	0.82	0.00	0.00	5.01	0.00	0.00	0.00	0.00	0.00	0.00	0.80	1.42	0.00	1.61	0.60	>0.999999999999999	2.7
P31323	cAMP-dependent protein kinase type II-beta regulatory subunit OS=Homo sapiens GN=PRKAR2B PE=1 SV=3	0.00	1.20	1.67	1.50	1.32	0.00	1.20	0.00	0.00	1.30	0.00	0.57	0.00	1.12	0.00	1.18	0.00	0.00	0.00	0.00	0.00	0.56	0.99	0.28	0.73	0.34	>0.999999999999999	2.1
Q13907	Isopentenyl-diphosphate Delta-isomerase 1 OS=Homo sapiens GN=IDI1 PE=1 SV=2	0.00	2.23	3.11	5.77	5.09	3.07	0.00	0.00	0.00	2.41	4.25	0.00	0.00	2.09	0.00	2.19	0.00	0.00	0.00	0.00	0.00	1.04	1.84	0.52	2.16	0.64	>0.999999999999999	3.4
Q9H3S7	Tyrosine-protein phosphatase non-receptor type 23 OS=Homo sapiens GN=PTPN23 PE=1 SV=1	0.00	0.34	0.48	0.00	0.00	0.95	0.00	0.00	0.00	0.00	0.31	0.00	0.00	0.00	0.00	0.00	0.00	0.00	0.00	0.00	0.00	0.00	0.00	0.00	0.17	0.00	>0.999999999999999	#DIV/0!
Q9H0N0	Ras-related protein Rab-6C OS=Homo sapiens GN=RAB6C PE=2 SV=2	0.00	2.06	2.87	0.00	2.26	2.84	2.05	0.00	0.00	2.22	1.89	0.00	0.00	1.93	2.86	2.02	0.00	1.84	2.17	0.00	0.00	0.96	0.00	0.48	1.35	1.02	>0.999999999999999	1.3
P18283	Glutathione peroxidase 2 OS=Homo sapiens GN=GPX2 PE=1 SV=3	0.00	2.57	0.00	0.00	6.03	0.00	0.00	1.35	0.00	2.78	5.03	1.22	0.00	0.00	3.58	2.52	0.00	0.00	2.71	0.00	0.00	2.56	4.54	0.60	1.58	1.38	>0.999999999999999	1.2
P14314	Glucosidase 2 subunit beta OS=Homo sapiens GN=PRKCSH PE=1 SV=2	0.00	0.86	0.00	2.35	0.00	1.18	0.00	0.00	0.68	0.93	0.79	1.39	0.00	0.80	1.19	0.00	0.00	0.00	1.99	0.65	0.84	0.88	2.41	0.68	0.68	0.79	>0.999999999999999	0.9
P50579	Methionine aminopeptidase 2 OS=Homo sapiens GN=METAP2 PE=1 SV=1	0.00	1.03	0.00	0.00	1.13	1.42	1.03	0.00	0.00	0.00	0.94	0.00	0.00	0.96	0.00	0.00	0.00	0.00	0.00	0.00	0.00	0.00	0.00	0.00	0.46	0.08	>0.999999999999999	5.8
Q13347	Eukaryotic translation initiation factor 3 subunit I OS=Homo sapiens GN=EIF3I PE=1 SV=1	0.00	1.54	6.93	0.00	3.58	12.76	1.54	0.00	2.60	1.67	8.49	0.73	2.12	0.00	2.15	0.00	0.00	0.00	3.44	0.00	0.00	0.00	4.12	0.36	3.32	1.01	>0.999999999999999	3.3
Q15418	Ribosomal protein S6 kinase alpha-1 OS=Homo sapiens GN=RPS6KA1 PE=1 SV=2	0.00	0.69	1.91	0.00	0.00	0.00	0.00	0.00	0.00	0.00	1.89	0.33	0.00	0.00	0.00	0.00	0.00	0.00	0.00	0.00	0.00	0.64	0.57	0.00	0.40	0.10	>0.999999999999999	4.0
Q9NVE7	Pantothenate kinase 4 OS=Homo sapiens GN=PANK4 PE=1 SV=1	0.00	0.69	0.00	0.00	0.00	0.95	0.00	0.00	0.00	0.00	0.00	0.00	0.00	0.00	0.00	0.00	0.00	0.00	0.00	0.00	0.00	0.00	0.57	0.00	0.14	0.05	>0.999999999999999	2.9
Q969Q0	60S ribosomal protein L36a-like OS=Homo sapiens GN=RPL36AL PE=1 SV=3	0.00	4.63	6.45	5.77	11.50	0.00	4.62	0.00	3.70	5.00	4.25	0.00	0.00	0.00	6.44	0.00	3.42	0.00	0.00	0.00	0.00	0.00	0.00	0.00	3.83	0.82	>0.999999999999999	4.7
Q86VN1	Vacuolar protein-sorting-associated protein 36 OS=Homo sapiens GN=VPS36 PE=1 SV=1	0.00	1.20	0.00	0.00	0.00	0.00	0.00	0.00	0.00	1.30	1.10	0.00	0.00	0.00	0.00	0.00	0.00	0.00	0.00	0.00	0.00	0.00	0.00	0.00	0.30	0.00	>0.999999999999999	#DIV/0!
P61966	AP-1 complex subunit sigma-1A OS=Homo sapiens GN=AP1S1 PE=1 SV=1	0.00	3.09	4.30	3.85	0.00	0.00	0.00	0.00	0.00	3.33	2.83	0.00	0.00	0.00	4.29	3.03	0.00	0.00	0.00	0.00	0.00	0.00	8.94	0.00	1.45	1.36	>0.999999999999999	1.1
P34949	Mannose-6-phosphate isomerase OS=Homo sapiens GN=MPI PE=1 SV=2	0.00	1.20	5.26	1.50	0.00	1.65	0.00	0.00	0.00	0.00	0.00	0.00	0.00	0.00	0.00	0.00	0.00	0.00	0.00	0.00	0.00	0.00	0.99	0.28	0.80	0.11	>0.999999999999999	7.5
P09417	Dihydropteridine reductase OS=Homo sapiens GN=QDPR PE=1 SV=2	0.00	2.23	6.45	2.78	2.45	6.38	0.00	0.00	0.00	0.00	2.04	0.00	0.00	2.09	3.10	2.19	0.00	0.00	0.00	0.00	0.00	0.00	0.00	0.00	1.86	0.61	>0.999999999999999	3.0
Q9NWV4	UPF0587 protein C1orf123 OS=Homo sapiens GN=C1orf123 PE=1 SV=1	0.00	6.86	4.30	8.55	0.00	4.25	6.84	0.00	2.47	3.33	6.29	0.00	0.00	0.00	4.29	3.03	0.00	0.00	3.26	0.00	0.00	3.20	0.00	0.00	3.57	1.15	>0.999999999999999	3.1
Q96FT9	Intraflagellar transport protein 43 homolog OS=Homo sapiens GN=IFT43 PE=1 SV=3	0.00	2.40	3.35	2.99	0.00	3.31	0.00	0.00	0.00	2.59	2.20	0.00	0.00	0.00	0.00	0.00	0.00	0.00	0.00	0.00	0.00	0.00	0.00	0.00	1.40	0.00	>0.999999999999999	#DIV/0!
P28799	Granulins OS=Homo sapiens GN=GRN PE=1 SV=2	0.00	0.86	0.00	0.00	0.00	0.00	0.00	0.45	0.00	0.00	0.00	0.41	1.18	0.00	1.19	0.00	0.00	0.00	0.00	0.65	0.00	0.00	0.71	0.00	0.14	0.31	>0.999999999999999	0.5
Q9UBR2	Cathepsin Z OS=Homo sapiens GN=CTSZ PE=1 SV=1	0.00	1.72	2.39	0.00	0.00	2.36	1.71	0.90	0.00	0.00	1.57	0.00	2.35	1.61	2.38	0.00	0.00	0.00	1.81	1.30	0.00	1.60	2.84	0.00	0.89	1.16	>0.999999999999999	0.8
Q08554	Desmocollin-1 OS=Homo sapiens GN=DSC1 PE=1 SV=2	0.00	0.51	0.00	0.00	0.00	0.00	0.00	0.00	0.00	0.00	0.00	0.00	0.00	0.00	0.00	0.00	0.00	0.00	0.00	0.00	0.00	0.00	0.00	0.00	0.04	0.00	>0.999999999999999	#DIV/0!
Q15046	Lysine--tRNA ligase OS=Homo sapiens GN=KARS PE=1 SV=3	0.00	0.86	1.20	1.07	0.00	1.18	0.00	0.00	0.00	0.00	0.00	0.82	0.00	0.00	1.19	0.00	0.00	0.00	0.00	0.00	0.00	0.00	0.71	0.20	0.43	0.18	>0.999999999999999	2.4
Q13148	TAR DNA-binding protein 43 OS=Homo sapiens GN=TARDBP PE=1 SV=1	0.00	1.20	3.59	1.50	0.00	3.54	0.00	0.00	0.00	1.30	0.00	0.57	0.00	0.00	0.00	1.18	0.00	0.00	0.00	0.00	0.00	0.00	0.00	0.00	0.97	0.10	>0.999999999999999	9.9
P60891	Ribose-phosphate pyrophosphokinase 1 OS=Homo sapiens GN=PRPS1 PE=1 SV=2	0.00	1.54	10.28	15.18	5.85	13.47	5.30	0.81	0.00	7.97	8.96	0.00	0.00	3.21	13.59	3.36	0.00	3.07	0.00	0.00	0.00	4.55	6.10	0.00	5.78	2.82	>0.999999999999999	2.0
P62312	U6 snRNA-associated Sm-like protein LSm6 OS=Homo sapiens GN=LSM6 PE=1 SV=1	0.00	6.52	0.00	8.12	7.16	8.98	6.50	0.00	0.00	0.00	5.98	0.00	0.00	0.00	0.00	0.00	4.81	0.00	7.05	0.00	0.00	0.00	5.39	0.00	3.61	1.44	>0.999999999999999	2.5
P61353	60S ribosomal protein L27 OS=Homo sapiens GN=RPL27 PE=1 SV=2	0.00	3.77	5.26	10.26	0.00	11.34	0.00	0.00	0.00	0.00	3.46	0.00	5.17	3.53	5.25	3.70	0.00	0.00	0.00	0.00	0.00	1.76	6.81	0.00	2.84	2.19	>0.999999999999999	1.3
P09622	Dihydrolipoyl dehydrogenase, mitochondrial OS=Homo sapiens GN=DLD PE=1 SV=2	0.00	1.03	0.00	0.00	0.00	1.42	0.00	0.00	0.00	0.00	0.94	0.98	0.00	0.96	0.00	0.00	0.00	0.00	0.00	0.00	0.00	0.00	0.00	0.48	0.36	0.12	>0.999999999999999	3.0
O76054	SEC14-like protein 2 OS=Homo sapiens GN=SEC14L2 PE=1 SV=1	0.00	1.20	9.80	0.00	0.00	9.69	0.00	0.00	0.00	2.78	0.00	0.00	0.00	0.00	0.00	0.00	0.00	0.00	0.00	0.00	0.00	0.00	0.00	0.00	1.96	0.00	>0.999999999999999	#DIV/0!
O00154	Cytosolic acyl coenzyme A thioester hydrolase OS=Homo sapiens GN=ACOT7 PE=1 SV=3	0.00	1.37	0.00	0.00	0.00	0.00	0.00	0.00	0.00	0.00	0.00	0.65	0.00	0.00	0.00	1.35	0.00	0.00	0.00	0.00	0.00	0.00	1.14	0.32	0.17	0.23	>0.999999999999999	0.7
O43488	Aflatoxin B1 aldehyde reductase member 2 OS=Homo sapiens GN=AKR7A2 PE=1 SV=3	0.00	1.37	8.84	7.91	0.00	4.02	0.00	0.00	0.00	0.00	2.67	1.39	0.00	0.00	1.91	1.35	0.00	0.00	0.00	0.00	0.00	0.00	3.83	0.32	2.18	0.62	>0.999999999999999	3.5
Q16543	Hsp90 co-chaperone Cdc37 OS=Homo sapiens GN=CDC37 PE=1 SV=1	0.00	1.20	3.59	3.21	4.53	5.67	2.56	0.00	0.96	1.30	5.19	1.22	0.00	0.00	5.72	2.52	3.04	1.07	2.71	0.00	2.53	1.20	2.13	0.96	2.45	1.82	>0.999999999999999	1.3
P05089	Arginase-1 OS=Homo sapiens GN=ARG1 PE=1 SV=2	0.00	3.43	0.00	2.14	0.00	0.00	0.00	0.00	0.00	0.00	0.00	0.00	4.70	0.00	0.00	0.00	0.00	0.00	0.00	0.00	1.69	0.00	0.00	0.00	0.46	0.53	>0.999999999999999	0.9
P17612	cAMP-dependent protein kinase catalytic subunit alpha OS=Homo sapiens GN=PRKACA PE=1 SV=2	0.00	2.92	8.84	3.63	3.21	4.02	2.91	0.00	0.00	3.15	4.09	1.39	1.88	1.28	1.91	4.37	0.00	0.00	3.07	0.00	0.00	0.64	3.69	1.04	2.85	1.49	>0.999999999999999	1.9
Q9UIC8	Leucine carboxyl methyltransferase 1 OS=Homo sapiens GN=LCMT1 PE=1 SV=2	0.00	1.37	0.00	0.00	0.00	0.00	0.00	0.00	0.00	0.00	0.00	0.00	0.00	0.00	0.00	0.00	0.00	0.00	0.00	0.00	0.00	0.00	0.00	0.00	0.11	0.00	>0.999999999999999	#DIV/0!
Q8NE71	ATP-binding cassette sub-family F member 1 OS=Homo sapiens GN=ABCF1 PE=1 SV=2	0.00	0.51	0.00	0.64	0.57	0.00	0.00	0.00	0.00	0.56	0.00	0.24	0.00	0.00	0.00	0.00	0.00	0.00	0.00	0.00	0.00	0.24	0.43	0.12	0.21	0.07	>0.999999999999999	3.2
Q14C86	GTPase-activating protein and VPS9 domain-containing protein 1 OS=Homo sapiens GN=GAPVD1 PE=1 SV=2	0.00	0.69	1.43	1.28	0.38	2.84	0.68	0.18	0.00	0.74	1.57	0.33	0.00	0.96	2.38	0.67	0.00	0.31	0.36	0.00	0.00	0.32	1.42	0.08	0.84	0.54	>0.999999999999999	1.6
P50895	Basal cell adhesion molecule OS=Homo sapiens GN=BCAM PE=1 SV=2	0.00	0.86	0.00	1.07	0.00	1.18	0.00	0.00	0.00	0.00	0.00	0.00	0.00	0.00	0.00	0.00	0.00	0.00	0.00	0.00	0.00	0.00	0.00	0.00	0.26	0.00	>0.999999999999999	#DIV/0!
P35573	Glycogen debranching enzyme OS=Homo sapiens GN=AGL PE=1 SV=3	0.00	0.34	0.48	1.71	0.00	2.36	0.00	0.00	0.00	2.59	1.57	0.65	0.00	0.00	0.00	0.34	0.00	0.00	0.00	0.00	0.00	0.16	0.28	0.32	0.81	0.09	>0.999999999999999	8.8
O43653	Prostate stem cell antigen OS=Homo sapiens GN=PSCA PE=1 SV=1	0.00	4.29	5.98	5.34	0.00	0.00	0.00	0.00	0.00	4.63	8.96	2.04	0.00	4.01	5.96	4.21	0.00	0.00	0.00	0.00	0.00	2.00	8.09	1.00	2.60	2.11	>0.999999999999999	1.2
O43747	AP-1 complex subunit gamma-1 OS=Homo sapiens GN=AP1G1 PE=1 SV=5	0.00	0.69	0.00	0.86	0.00	0.00	0.00	0.00	0.00	0.00	0.63	0.33	0.00	0.00	0.00	0.00	0.00	0.00	0.72	0.00	0.00	0.00	0.57	0.16	0.21	0.12	>0.999999999999999	1.7
O43149	Zinc finger ZZ-type and EF-hand domain-containing protein 1 OS=Homo sapiens GN=ZZEF1 PE=1 SV=6	0.00	0.17	0.72	0.00	0.00	0.00	0.00	0.00	0.00	0.00	0.00	0.00	0.00	0.00	0.00	0.00	0.00	0.00	0.00	0.00	0.00	0.00	0.00	0.00	0.07	0.00	>0.999999999999999	#DIV/0!
P49247	Ribose-5-phosphate isomerase OS=Homo sapiens GN=RPIA PE=1 SV=3	0.00	1.72	5.02	4.49	3.96	10.87	5.64	0.00	0.00	0.00	0.00	0.82	0.00	0.00	2.38	0.00	0.00	0.00	0.00	0.00	0.00	1.68	2.98	0.84	2.71	0.66	>0.999999999999999	4.1
Q9Y5K6	CD2-associated protein OS=Homo sapiens GN=CD2AP PE=1 SV=1	0.00	0.86	1.20	1.92	1.70	1.18	0.85	0.00	0.00	0.00	1.42	0.41	0.00	0.00	1.19	0.00	0.00	0.00	0.00	0.00	0.00	0.00	0.00	0.00	0.79	0.10	>0.999999999999999	8.0
Q6MZZ7	Calpain-13 OS=Homo sapiens GN=CAPN13 PE=1 SV=2	0.00	0.69	2.15	0.86	1.70	2.13	0.68	0.00	0.00	0.74	0.63	0.33	0.00	0.00	0.95	0.00	0.51	0.00	1.63	0.00	0.00	0.00	0.57	0.00	0.82	0.30	>0.999999999999999	2.7
Q9Y5K3	Choline-phosphate cytidylyltransferase B OS=Homo sapiens GN=PCYT1B PE=1 SV=1	0.00	1.37	0.00	0.00	1.51	1.89	0.00	0.00	0.00	0.00	1.26	0.00	0.00	0.00	0.00	0.00	0.00	0.00	0.00	0.00	0.00	0.00	0.00	0.00	0.50	0.00	>0.999999999999999	#DIV/0!
O60271	C-Jun-amino-terminal kinase-interacting protein 4 OS=Homo sapiens GN=SPAG9 PE=1 SV=4	0.00	0.34	0.00	0.00	0.00	0.00	0.00	0.00	0.00	0.00	0.00	0.00	0.00	0.00	0.00	0.00	0.00	0.00	0.00	0.00	0.00	0.00	0.00	0.00	0.03	0.00	>0.999999999999999	#DIV/0!
P46778	60S ribosomal protein L21 OS=Homo sapiens GN=RPL21 PE=1 SV=2	0.00	3.09	4.30	4.06	0.00	0.00	0.00	0.00	0.00	3.33	0.00	1.47	0.00	0.00	4.53	0.00	0.00	0.00	0.00	0.00	0.00	0.00	0.00	0.00	1.35	0.38	>0.999999999999999	3.6
P13987	CD59 glycoprotein OS=Homo sapiens GN=CD59 PE=1 SV=1	0.00	3.95	5.50	4.92	0.00	5.43	3.93	0.00	3.15	4.26	0.00	1.88	0.00	0.00	5.49	0.00	0.00	0.00	0.00	2.99	3.88	1.84	0.00	2.04	2.75	1.35	>0.999999999999999	2.0
Q9NZL4	Hsp70-binding protein 1 OS=Homo sapiens GN=HSPBP1 PE=1 SV=1	0.00	1.37	1.91	0.00	0.00	0.00	0.00	0.00	0.00	0.00	0.00	0.00	0.00	0.00	0.00	0.00	0.00	0.00	0.00	0.00	0.00	0.00	1.14	0.00	0.27	0.09	>0.999999999999999	2.9
Q3B8N2	Galectin-9B OS=Homo sapiens GN=LGALS9B PE=2 SV=3	0.00	1.37	0.00	0.00	0.00	0.00	0.00	0.00	0.00	0.00	0.00	0.00	0.00	0.00	0.00	0.00	0.00	0.00	0.00	0.00	0.00	0.00	0.00	0.00	0.11	0.00	>0.999999999999999	#DIV/0!
Q92625	Ankyrin repeat and SAM domain-containing protein 1A OS=Homo sapiens GN=ANKS1A PE=1 SV=4	0.00	0.51	0.00	0.00	0.00	0.71	0.00	0.00	0.00	0.56	0.47	0.00	0.00	0.00	0.00	0.00	0.00	0.00	0.00	0.00	0.00	0.00	0.43	0.00	0.19	0.04	>0.999999999999999	5.3
P55196	Afadin OS=Homo sapiens GN=MLLT4 PE=1 SV=3	0.00	0.34	0.72	0.00	0.00	0.00	0.00	0.00	0.00	0.00	0.31	0.00	0.00	0.32	0.00	0.00	0.00	0.00	0.00	0.00	0.00	0.00	0.00	0.00	0.11	0.03	>0.999999999999999	4.3
Q96GX9	Probable methylthioribulose-1-phosphate dehydratase OS=Homo sapiens GN=APIP PE=1 SV=1	0.00	2.06	0.00	0.00	0.00	0.00	0.00	0.00	0.00	2.22	0.00	0.00	0.00	0.00	0.00	0.00	0.00	0.00	0.00	0.00	0.00	0.00	0.00	0.00	0.36	0.00	>0.999999999999999	#DIV/0!
O14908	PDZ domain-containing protein GIPC1 OS=Homo sapiens GN=GIPC1 PE=1 SV=2	0.00	1.54	2.15	1.92	0.00	0.00	0.00	0.00	0.00	0.00	0.00	0.00	0.00	0.00	0.00	0.00	0.00	0.00	0.00	0.00	0.00	1.52	4.26	0.00	0.47	0.48	>0.999999999999999	1.0
Q14203	Dynactin subunit 1 OS=Homo sapiens GN=DCTN1 PE=1 SV=3	0.00	0.34	4.78	1.50	0.94	2.36	0.34	0.00	0.00	1.30	0.79	0.41	0.47	0.00	1.67	0.34	0.00	0.00	0.00	0.00	0.00	0.40	0.00	0.20	1.06	0.26	>0.999999999999999	4.1
Q96CP2	FLYWCH family member 2 OS=Homo sapiens GN=FLYWCH2 PE=1 SV=1	0.00	3.95	0.00	0.00	0.00	0.00	0.00	0.00	0.00	0.00	0.00	0.00	0.00	0.00	0.00	0.00	0.00	0.00	0.00	0.00	0.00	0.00	0.00	0.00	0.33	0.00	>0.999999999999999	#DIV/0!
P30039	Phenazine biosynthesis-like domain-containing protein OS=Homo sapiens GN=PBLD PE=1 SV=2	0.00	1.72	11.47	0.00	0.00	0.00	0.00	0.00	0.00	0.00	3.46	0.00	0.00	5.46	0.00	0.00	0.00	0.00	0.00	0.00	0.00	0.00	0.00	0.00	1.39	0.45	>0.999999999999999	3.0
Q9Y3E1	Hepatoma-derived growth factor-related protein 3 OS=Homo sapiens GN=HDGFRP3 PE=1 SV=1	0.00	2.57	0.00	0.00	0.00	0.00	0.00	0.00	0.00	2.78	0.00	0.00	0.00	0.00	0.00	0.00	0.00	0.00	0.00	0.00	0.00	0.00	0.00	0.00	0.45	0.00	>0.999999999999999	#DIV/0!
P78318	Immunoglobulin-binding protein 1 OS=Homo sapiens GN=IGBP1 PE=1 SV=1	0.00	1.37	1.91	0.00	0.00	1.89	1.37	0.00	0.00	0.00	2.83	0.00	0.00	0.00	0.00	0.00	0.00	0.00	0.00	0.00	0.00	0.00	1.14	0.00	0.78	0.09	>0.999999999999999	8.3
O43847	Nardilysin OS=Homo sapiens GN=NRD1 PE=1 SV=2	0.00	0.86	0.48	2.14	0.38	1.18	0.34	0.00	0.00	0.37	0.79	0.00	0.00	0.00	0.00	0.00	0.00	0.00	0.00	0.00	0.00	0.00	0.28	0.20	0.54	0.04	>0.999999999999999	13.5
Q03426	Mevalonate kinase OS=Homo sapiens GN=MVK PE=1 SV=1	0.00	1.37	0.00	0.00	0.00	0.00	0.00	0.00	0.00	0.00	1.26	0.00	0.00	0.00	0.00	0.00	0.00	0.00	0.00	0.00	0.00	0.64	1.14	0.32	0.22	0.17	>0.999999999999999	1.3
A0AVF1	Tetratricopeptide repeat protein 26 OS=Homo sapiens GN=TTC26 PE=2 SV=1	0.00	0.86	2.39	1.07	0.00	0.00	0.00	0.00	0.00	0.00	0.00	0.00	0.00	0.00	0.00	0.00	0.00	0.00	0.00	0.00	0.00	0.00	0.00	0.00	0.36	0.00	>0.999999999999999	#DIV/0!
O43708	Maleylacetoacetate isomerase OS=Homo sapiens GN=GSTZ1 PE=1 SV=3	0.00	2.40	3.35	6.20	0.00	0.00	0.00	0.00	0.00	2.59	2.20	0.00	0.00	2.25	0.00	2.36	1.77	2.15	2.53	0.00	0.00	2.32	6.67	0.56	1.40	1.72	>0.999999999999999	0.8
P17844	Probable ATP-dependent RNA helicase DDX5 OS=Homo sapiens GN=DDX5 PE=1 SV=1	0.00	0.86	0.00	0.00	0.94	3.54	1.71	0.00	0.00	0.00	0.00	1.22	0.00	0.00	0.00	1.68	0.00	0.77	0.90	0.00	0.00	0.00	0.00	0.00	0.69	0.28	>0.999999999999999	2.5
Q9UNP9	Peptidyl-prolyl cis-trans isomerase E OS=Homo sapiens GN=PPIE PE=1 SV=1	0.00	1.72	0.00	0.00	0.00	0.00	0.00	0.00	0.00	0.00	0.00	0.00	0.00	0.00	0.00	0.00	0.00	0.00	0.00	0.00	0.00	0.00	0.00	0.00	0.14	0.00	>0.999999999999999	#DIV/0!
Q9NVZ3	Adaptin ear-binding coat-associated protein 2 OS=Homo sapiens GN=NECAP2 PE=1 SV=1	0.00	2.06	0.00	0.00	0.00	2.84	0.00	0.00	0.00	0.00	0.00	0.00	0.00	0.00	0.00	0.00	0.00	0.00	0.00	0.00	0.00	0.00	0.00	0.00	0.41	0.00	>0.999999999999999	#DIV/0!
Q8IVF2	Protein AHNAK2 OS=Homo sapiens GN=AHNAK2 PE=1 SV=2	0.00	0.17	0.00	0.21	0.00	0.24	0.00	0.00	0.00	0.00	0.16	0.00	0.00	0.00	0.24	0.17	0.00	0.15	0.00	0.00	0.00	0.08	0.14	0.00	0.06	0.07	>0.999999999999999	1.0
Q14116	Interleukin-18 OS=Homo sapiens GN=IL18 PE=1 SV=1	0.00	2.57	0.00	3.21	0.00	0.00	2.56	1.35	0.00	0.00	2.36	1.22	0.00	2.41	7.63	0.00	0.00	0.00	5.79	0.00	0.00	0.00	2.13	0.00	1.11	1.50	>0.999999999999999	0.7
Q8N0U7	Uncharacterized protein C1orf87 OS=Homo sapiens GN=C1orf87 PE=2 SV=1	0.00	0.86	2.63	0.00	0.00	2.60	0.00	0.00	0.00	2.04	2.67	0.00	0.00	0.00	0.00	0.00	0.00	0.00	0.00	0.00	0.00	0.40	0.00	0.00	0.90	0.03	>0.999999999999999	27.0
O43765	Small glutamine-rich tetratricopeptide repeat-containing protein alpha OS=Homo sapiens GN=SGTA PE=1 SV=1	0.00	1.72	2.39	2.14	1.89	2.36	0.00	0.00	0.00	0.00	0.00	0.00	0.00	0.00	7.63	0.00	0.00	0.00	0.00	0.00	1.69	0.80	2.84	0.40	0.87	1.11	>0.999999999999999	0.8
Q86V21	Acetoacetyl-CoA synthetase OS=Homo sapiens GN=AACS PE=1 SV=1	0.00	0.69	0.00	0.00	0.00	0.00	0.00	0.00	0.00	0.00	0.63	0.00	0.00	0.00	0.00	0.00	0.00	0.00	0.00	0.00	0.00	0.00	0.00	0.00	0.11	0.00	>0.999999999999999	#DIV/0!
O75569	Interferon-inducible double stranded RNA-dependent protein kinase activator A OS=Homo sapiens GN=PRKRA PE=	0.00	1.72	4.78	2.14	1.89	4.73	0.00	0.00	0.00	0.00	0.00	1.63	0.00	0.00	2.38	0.00	0.00	0.00	0.00	0.00	0.00	0.80	0.00	0.00	1.41	0.27	>0.999999999999999	5.3
Q9UHD1	Cysteine and histidine-rich domain-containing protein 1 OS=Homo sapiens GN=CHORDC1 PE=1 SV=2	0.00	1.54	6.69	0.00	0.00	0.00	0.00	0.00	0.00	1.67	2.83	0.00	0.00	0.00	0.00	0.00	0.00	0.00	0.00	0.00	0.00	0.00	0.00	0.00	1.06	0.00	>0.999999999999999	#DIV/0!
Q9UIV8	Serpin B13 OS=Homo sapiens GN=SERPINB13 PE=2 SV=2	0.00	1.20	0.00	0.00	4.53	0.00	0.00	1.35	0.96	0.00	1.10	0.57	1.65	0.00	0.00	1.18	3.04	0.00	2.71	0.00	1.18	1.92	0.00	0.60	0.81	1.02	>0.999999999999999	0.8
Q96E29	mTERF domain-containing protein 1, mitochondrial OS=Homo sapiens GN=MTERFD1 PE=1 SV=2	0.00	1.20	0.00	0.00	0.00	0.00	0.00	0.00	0.00	0.00	0.00	0.00	0.00	0.00	0.00	0.00	0.00	0.00	0.00	0.00	0.00	0.00	0.00	0.00	0.10	0.00	>0.999999999999999	#DIV/0!
Q8NEZ5	F-box only protein 22 OS=Homo sapiens GN=FBXO22 PE=1 SV=1	0.00	1.20	0.00	0.00	0.00	1.65	1.20	0.00	0.00	1.30	0.00	0.00	0.00	0.00	1.67	0.00	0.00	0.00	0.00	0.00	0.00	0.56	0.00	0.00	0.45	0.19	>0.999999999999999	2.4
P61289	Proteasome activator complex subunit 3 OS=Homo sapiens GN=PSME3 PE=1 SV=1	0.00	1.89	0.00	2.35	0.00	2.60	0.00	0.00	0.00	4.45	3.77	0.90	0.00	0.00	2.62	0.00	0.00	0.00	0.00	0.00	0.00	1.92	1.56	0.00	1.33	0.51	>0.999999999999999	2.6
P99999	Cytochrome c OS=Homo sapiens GN=CYCS PE=1 SV=2	0.00	4.97	0.00	0.00	0.00	0.00	4.96	0.00	0.00	0.00	10.38	5.38	0.00	0.00	0.00	11.10	0.00	0.00	11.94	0.00	0.00	0.00	9.37	1.16	2.14	2.80	>0.999999999999999	0.8
Q07666	KH domain-containing, RNA-binding, signal transduction-associated protein 1 OS=Homo sapiens GN=KHDRBS1 PE	0.00	2.40	0.00	2.99	1.32	1.65	0.00	0.00	0.00	2.59	0.00	0.00	0.00	0.00	1.67	0.00	0.00	0.00	0.00	0.00	0.00	0.00	0.00	0.00	0.91	0.14	>0.999999999999999	6.6
P61916	Epididymal secretory protein E1 OS=Homo sapiens GN=NPC2 PE=1 SV=1	0.00	3.43	0.00	0.00	3.77	0.00	3.42	3.97	2.74	0.00	0.00	3.59	4.70	3.21	0.00	0.00	2.53	3.07	3.62	2.60	3.38	3.51	2.84	1.76	1.74	2.60	>0.999999999999999	0.7
Q5T4S7	E3 ubiquitin-protein ligase UBR4 OS=Homo sapiens GN=UBR4 PE=1 SV=1	0.00	0.17	1.20	0.21	0.19	0.24	0.17	0.00	0.00	0.00	0.16	0.08	0.00	0.00	0.48	0.00	0.00	0.00	0.00	0.00	0.00	0.00	0.14	0.04	0.20	0.05	>0.999999999999999	3.7
Q9NZZ3	Charged multivesicular body protein 5 OS=Homo sapiens GN=CHMP5 PE=1 SV=1	0.00	2.40	3.35	0.00	0.00	0.00	0.00	0.00	0.00	0.00	2.20	0.00	0.00	0.00	0.00	0.00	0.00	0.00	0.00	0.00	0.00	0.00	1.99	0.00	0.66	0.17	>0.999999999999999	4.0
Q05086	Ubiquitin-protein ligase E3A OS=Homo sapiens GN=UBE3A PE=1 SV=4	0.00	0.51	0.72	0.64	0.00	0.00	0.00	0.00	0.00	0.56	0.47	0.00	0.00	0.00	0.00	0.00	0.00	0.00	0.00	0.00	0.00	0.00	0.00	0.12	0.24	0.01	>0.999999999999999	24.2
O75663	TIP41-like protein OS=Homo sapiens GN=TIPRL PE=1 SV=2	0.00	1.89	5.26	2.35	0.00	0.00	0.00	0.00	0.00	0.00	3.46	0.00	0.00	1.77	0.00	0.00	0.00	0.00	0.00	0.00	0.00	0.88	1.56	0.00	1.08	0.35	>0.999999999999999	3.1
O75582	Ribosomal protein S6 kinase alpha-5 OS=Homo sapiens GN=RPS6KA5 PE=1 SV=1	0.00	0.69	0.96	0.00	0.00	0.00	0.00	0.00	0.00	0.00	0.00	0.00	0.00	0.00	0.00	0.00	0.00	0.00	0.00	0.00	0.00	0.00	0.00	0.00	0.14	0.00	>0.999999999999999	#DIV/0!
Q99598	Translin-associated protein X OS=Homo sapiens GN=TSNAX PE=1 SV=1	0.00	1.72	5.02	2.14	3.96	2.36	1.71	0.00	0.00	3.89	1.57	0.00	0.00	1.61	5.01	0.00	0.00	0.00	0.00	0.00	0.00	1.68	1.42	0.00	1.86	0.81	>0.999999999999999	2.3
Q06124	Tyrosine-protein phosphatase non-receptor type 11 OS=Homo sapiens GN=PTPN11 PE=1 SV=2	0.00	0.86	1.20	2.14	0.00	2.36	0.85	0.00	0.00	1.85	3.15	0.00	0.00	0.00	1.19	0.84	0.00	0.00	0.00	0.00	0.00	0.40	1.42	0.20	1.03	0.34	>0.999999999999999	3.1
O00487	26S proteasome non-ATPase regulatory subunit 14 OS=Homo sapiens GN=PSMD14 PE=1 SV=1	0.00	1.72	0.00	2.14	0.00	2.36	1.71	0.90	0.00	1.85	3.15	0.00	0.00	0.00	2.38	0.00	0.00	0.00	1.81	0.00	0.00	0.80	1.42	0.00	1.15	0.53	>0.999999999999999	2.2
Q96GE6	Calmodulin-like protein 4 OS=Homo sapiens GN=CALML4 PE=2 SV=3	0.00	2.57	0.00	0.00	0.00	0.00	0.00	0.00	0.00	0.00	0.00	0.00	0.00	0.00	0.00	0.00	0.00	0.00	0.00	0.00	0.00	0.00	0.00	0.00	0.21	0.00	>0.999999999999999	#DIV/0!
Q9Y3B8	Oligoribonuclease, mitochondrial OS=Homo sapiens GN=REXO2 PE=1 SV=3	0.00	2.06	0.00	0.00	2.26	0.00	2.05	1.08	0.00	0.00	1.89	0.98	0.00	1.93	0.00	0.00	0.00	0.00	2.17	0.00	0.00	0.96	0.00	0.48	0.86	0.46	>0.999999999999999	1.9
Q9HB07	UPF0160 protein MYG1, mitochondrial OS=Homo sapiens GN=C12orf10 PE=1 SV=2	0.00	1.37	5.98	3.42	0.00	5.91	0.00	0.00	0.00	2.96	3.93	0.00	0.00	2.57	1.91	0.00	0.00	1.23	0.00	0.00	0.00	0.00	0.00	0.00	1.96	0.48	>0.999999999999999	4.1
Q92623	Tetratricopeptide repeat protein 9A OS=Homo sapiens GN=TTC9 PE=2 SV=3	0.00	2.40	0.00	0.00	0.00	0.00	0.00	0.00	0.00	0.00	2.20	0.00	0.00	0.00	0.00	0.00	0.00	0.00	0.00	0.00	0.00	0.00	0.00	0.00	0.38	0.00	>0.999999999999999	#DIV/0!
Q08209	Serine/threonine-protein phosphatase 2B catalytic subunit alpha isoform OS=Homo sapiens GN=PPP3CA PE=1 SV=	0.00	1.03	1.43	1.28	0.00	0.00	1.03	0.00	0.00	0.00	0.94	0.00	0.00	0.00	0.00	0.00	0.00	0.00	0.00	0.00	0.00	0.48	0.00	0.00	0.48	0.04	>0.999999999999999	11.9
Q9UL25	Ras-related protein Rab-21 OS=Homo sapiens GN=RAB21 PE=1 SV=3	0.00	2.40	3.35	0.00	0.00	3.31	0.00	0.00	0.00	2.59	0.00	1.14	0.00	0.00	3.34	0.00	0.00	0.00	2.53	0.00	0.00	0.00	0.00	0.00	1.07	0.49	>0.999999999999999	2.2
P38646	Stress-70 protein, mitochondrial OS=Homo sapiens GN=HSPA9 PE=1 SV=2	0.00	0.69	0.00	0.86	0.00	0.00	0.00	0.00	0.00	0.00	2.20	3.43	0.00	0.00	0.00	1.51	0.00	0.00	0.00	0.00	0.00	0.00	0.57	0.76	0.60	0.24	>0.999999999999999	2.5
Q9UJT2	Testis-specific serine kinase substrate OS=Homo sapiens GN=TSKS PE=1 SV=3	0.00	0.86	0.00	1.07	0.94	0.00	0.85	0.00	0.00	0.00	0.00	0.00	0.00	0.00	0.00	0.84	0.00	0.00	0.00	0.00	0.00	0.00	0.00	0.00	0.31	0.07	>0.999999999999999	4.4
P09382	Galectin-1 OS=Homo sapiens GN=LGALS1 PE=1 SV=2	0.00	3.95	5.50	0.00	4.34	5.43	0.00	0.00	0.00	4.26	0.00	6.85	5.41	0.00	0.00	0.00	0.00	0.00	9.04	0.00	0.00	0.00	0.00	0.00	2.53	1.20	>0.999999999999999	2.1
Q13136	Liprin-alpha-1 OS=Homo sapiens GN=PPFIA1 PE=1 SV=1	0.00	0.34	0.00	0.00	0.00	0.00	0.00	0.00	0.00	0.00	0.00	0.00	0.00	0.00	0.00	0.00	0.00	0.00	0.00	0.00	0.00	0.00	0.00	0.00	0.03	0.00	>0.999999999999999	#DIV/0!
Q7L576	Cytoplasmic FMR1-interacting protein 1 OS=Homo sapiens GN=CYFIP1 PE=1 SV=1	0.00	0.34	1.67	1.50	0.00	1.18	1.20	0.00	0.00	0.37	2.20	0.57	0.00	0.00	0.48	0.34	0.25	0.00	0.36	0.00	0.00	0.00	0.28	0.08	0.75	0.15	>0.999999999999999	5.0
Q8IWB7	WD repeat and FYVE domain-containing protein 1 OS=Homo sapiens GN=WDFY1 PE=1 SV=1	0.00	1.20	0.00	0.00	0.00	0.00	0.00	0.00	0.00	0.00	0.00	0.00	0.00	0.00	0.00	0.00	0.00	0.00	0.00	0.00	0.00	0.00	0.00	0.00	0.10	0.00	>0.999999999999999	#DIV/0!
O00244	Copper transport protein ATOX1 OS=Homo sapiens GN=ATOX1 PE=1 SV=1	0.00	8.06	11.24	10.05	0.00	0.00	0.00	4.24	0.00	21.49	0.00	3.83	0.00	7.55	11.21	0.00	0.00	0.00	0.00	0.00	0.00	3.75	6.67	0.00	4.91	2.43	>0.999999999999999	2.0
O00429	Dynamin-1-like protein OS=Homo sapiens GN=DNM1L PE=1 SV=2	0.00	0.69	0.00	0.86	0.00	1.89	0.00	0.00	0.00	0.74	2.67	0.33	0.00	0.00	0.00	0.00	0.00	0.00	0.00	0.00	0.00	0.00	0.00	0.16	0.60	0.01	>0.999999999999999	44.8
Q5JNZ5	Putative 40S ribosomal protein S26-like 1 OS=Homo sapiens GN=RPS26P11 PE=5 SV=1	0.00	4.46	6.22	0.00	0.00	6.14	4.45	0.00	0.00	4.82	4.09	0.00	0.00	0.00	6.20	0.00	0.00	0.00	0.00	0.00	0.00	0.00	0.00	0.00	2.51	0.52	>0.999999999999999	4.9
Q4G0F5	Vacuolar protein sorting-associated protein 26B OS=Homo sapiens GN=VPS26B PE=1 SV=2	0.00	1.54	0.00	0.00	0.00	0.00	0.00	0.00	0.00	0.00	0.00	0.00	0.00	0.00	0.00	0.00	0.00	0.00	0.00	0.00	0.00	0.00	0.00	0.00	0.13	0.00	>0.999999999999999	#DIV/0!
Q96E40	Uncharacterized protein C9orf9 OS=Homo sapiens GN=C9orf9 PE=2 SV=1	0.00	2.23	6.69	0.00	0.00	0.00	0.00	0.00	0.00	0.00	0.00	0.00	0.00	0.00	0.00	0.00	0.00	0.00	0.00	0.00	0.00	0.00	0.00	0.00	0.74	0.00	>0.999999999999999	#DIV/0!
Q5XUX1	F-box/WD repeat-containing protein 9 OS=Homo sapiens GN=FBXW9 PE=1 SV=2	0.00	1.03	4.54	1.28	0.00	1.42	1.03	0.54	0.00	1.11	2.99	0.00	0.00	0.00	1.43	0.00	0.00	0.00	0.00	0.00	0.00	0.48	0.00	0.00	1.16	0.16	>0.999999999999999	7.3
Q13363	C-terminal-binding protein 1 OS=Homo sapiens GN=CTBP1 PE=1 SV=2	0.00	1.20	1.67	0.00	0.00	0.00	0.00	0.00	0.00	0.00	2.20	0.00	0.00	0.00	0.00	0.00	0.00	0.00	0.00	0.00	0.00	0.00	0.99	0.00	0.42	0.08	>0.999999999999999	5.1
P11233	Ras-related protein Ral-A OS=Homo sapiens GN=RALA PE=1 SV=1	0.00	2.40	0.00	0.00	0.00	0.00	0.00	0.00	0.00	0.00	0.00	1.14	0.00	0.00	0.00	0.00	0.00	0.00	0.00	0.00	0.00	0.00	0.00	0.00	0.30	0.00	>0.999999999999999	#DIV/0!
P26196	Probable ATP-dependent RNA helicase DDX6 OS=Homo sapiens GN=DDX6 PE=1 SV=2	0.00	1.03	0.00	0.00	0.00	0.00	0.00	0.00	0.00	2.22	0.00	0.00	0.00	0.00	0.00	0.00	0.00	0.00	0.00	0.00	0.00	0.00	0.00	0.00	0.27	0.00	>0.999999999999999	#DIV/0!
Q8N7X1	RNA-binding motif protein, X-linked-like-3 OS=Homo sapiens GN=RBMXL3 PE=2 SV=2	0.00	0.51	0.00	0.64	0.00	0.71	0.00	0.27	0.00	0.56	0.00	0.00	0.00	0.00	0.00	0.50	0.00	0.00	0.54	0.00	0.00	0.00	0.43	0.00	0.22	0.12	>0.999999999999999	1.8
Q86VM9	Zinc finger CCCH domain-containing protein 18 OS=Homo sapiens GN=ZC3H18 PE=1 SV=2	0.00	0.51	0.00	0.00	0.00	0.00	0.00	0.00	0.00	0.00	0.00	0.00	0.00	0.00	0.00	0.00	0.00	0.00	0.00	0.00	0.00	0.00	0.00	0.00	0.04	0.00	>0.999999999999999	#DIV/0!
Q96LB3	Intraflagellar transport protein 74 homolog OS=Homo sapiens GN=IFT74 PE=2 SV=1	0.00	0.86	3.59	1.07	0.00	2.36	0.00	0.00	0.00	1.85	2.36	0.00	0.00	0.00	1.19	0.00	0.00	0.00	0.00	0.00	0.00	0.00	0.00	0.00	1.01	0.10	>0.999999999999999	10.1
Q13435	Splicing factor 3B subunit 2 OS=Homo sapiens GN=SF3B2 PE=1 SV=2	0.00	0.51	0.00	0.64	0.00	0.71	0.51	0.27	0.00	0.56	0.47	0.00	0.00	0.00	0.72	0.00	0.00	0.00	0.00	0.00	0.00	0.00	0.43	0.00	0.31	0.10	>0.999999999999999	3.2
P36639	7,8-dihydro-8-oxoguanine triphosphatase OS=Homo sapiens GN=NUDT1 PE=1 SV=3	0.00	2.57	0.00	0.00	0.00	0.00	0.00	0.00	0.00	0.00	0.00	0.00	0.00	0.00	0.00	0.00	0.00	0.00	0.00	0.00	0.00	0.00	2.13	0.00	0.21	0.18	>0.999999999999999	1.2
Q9NVR5	Protein kintoun OS=Homo sapiens GN=DNAAF2 PE=1 SV=2	0.00	0.69	1.67	0.00	0.00	0.00	0.00	0.00	0.00	0.00	0.00	0.00	0.00	0.00	0.00	0.00	0.00	0.00	0.00	0.00	0.00	0.00	0.00	0.00	0.20	0.00	>0.999999999999999	#DIV/0!
Q9UGP4	LIM domain-containing protein 1 OS=Homo sapiens GN=LIMD1 PE=1 SV=1	0.00	0.69	0.00	0.00	0.00	0.00	0.00	0.00	0.00	0.00	0.00	0.00	0.00	0.00	0.00	0.00	0.00	0.00	0.00	0.00	0.00	0.00	0.00	0.00	0.06	0.00	>0.999999999999999	#DIV/0!
P51808	Dynein light chain Tctex-type 3 OS=Homo sapiens GN=DYNLT3 PE=1 SV=1	0.00	4.29	0.00	0.00	0.00	0.00	0.00	0.00	0.00	0.00	3.93	2.04	0.00	0.00	0.00	0.00	0.00	0.00	0.00	0.00	0.00	0.00	0.00	0.00	0.85	0.00	>0.999999999999999	#DIV/0!
P18827	Syndecan-1 OS=Homo sapiens GN=SDC1 PE=1 SV=3	0.00	1.72	0.00	0.00	0.00	0.00	0.00	0.00	0.00	0.00	1.57	0.82	0.00	0.00	0.00	0.00	0.00	0.00	0.00	0.00	0.00	0.80	1.42	0.40	0.34	0.22	>0.999999999999999	1.6
Q8WX93	Palladin OS=Homo sapiens GN=PALLD PE=1 SV=3	0.00	0.34	0.00	0.00	0.00	0.00	0.00	0.00	0.00	0.00	0.31	0.00	0.00	0.00	0.00	0.00	0.00	0.00	0.00	0.00	0.00	0.00	0.00	0.00	0.05	0.00	>0.999999999999999	#DIV/0!
Q9UP83	Conserved oligomeric Golgi complex subunit 5 OS=Homo sapiens GN=COG5 PE=1 SV=3	0.00	0.69	0.00	0.00	0.00	0.00	0.00	0.00	0.00	0.74	0.00	0.33	0.00	0.00	0.00	0.00	0.00	0.00	0.00	0.00	0.00	0.00	0.57	0.16	0.15	0.06	>0.999999999999999	2.4
Q9BYT3	Serine/threonine-protein kinase 33 OS=Homo sapiens GN=STK33 PE=1 SV=1	0.00	1.03	2.87	2.57	0.00	5.91	0.00	0.00	0.00	5.93	2.83	0.00	0.00	0.00	1.43	0.00	0.00	0.00	0.00	0.00	0.00	0.48	1.70	0.00	1.76	0.30	>0.999999999999999	5.8
Q99426	Tubulin-folding cofactor B OS=Homo sapiens GN=TBCB PE=1 SV=2	0.00	2.06	0.00	2.57	0.00	2.84	0.00	0.00	1.64	2.22	1.89	0.00	0.00	0.00	2.86	0.00	0.00	0.00	0.00	0.00	0.00	0.96	1.70	0.00	1.10	0.46	>0.999999999999999	2.4
A8K2U0	Alpha-2-macroglobulin-like protein 1 OS=Homo sapiens GN=A2ML1 PE=1 SV=3	0.00	0.34	0.00	0.00	0.00	0.00	0.00	0.00	0.00	0.00	0.00	0.00	0.00	0.32	0.00	0.00	0.00	0.00	0.00	0.00	0.00	0.00	0.00	0.00	0.03	0.03	>0.999999999999999	1.1
Q6BCY4	NADH-cytochrome b5 reductase 2 OS=Homo sapiens GN=CYB5R2 PE=1 SV=1	0.00	1.89	11.71	7.48	2.07	8.27	0.00	0.00	1.51	2.04	5.50	0.90	0.00	1.77	5.25	1.85	0.00	0.00	0.00	0.00	0.00	1.76	0.00	0.00	3.45	0.89	>0.999999999999999	3.9
P09668	Pro-cathepsin H OS=Homo sapiens GN=CTSH PE=1 SV=4	0.00	1.54	0.00	0.00	0.00	2.13	0.00	0.00	0.00	0.00	0.00	0.00	0.00	1.45	0.00	0.00	0.00	0.00	0.00	0.00	0.00	0.00	0.00	0.00	0.31	0.12	>0.999999999999999	2.5
O00443	Phosphatidylinositol-4-phosphate 3-kinase C2 domain-containing subunit alpha OS=Homo sapiens GN=PIK3C2A PE	0.00	0.34	0.00	0.00	0.00	0.00	0.00	0.00	0.00	0.00	0.00	0.00	0.00	0.00	0.00	0.00	0.00	0.00	0.00	0.00	0.00	0.00	0.00	0.00	0.03	0.00	>0.999999999999999	#DIV/0!
P49006	MARCKS-related protein OS=Homo sapiens GN=MARCKSL1 PE=1 SV=2	0.00	2.92	0.00	0.00	0.00	0.00	6.33	0.00	0.00	0.00	0.00	1.39	0.00	0.00	0.00	0.00	0.00	0.00	0.00	0.00	0.00	0.00	0.00	0.00	0.89	0.00	>0.999999999999999	#DIV/0!
Q9NRV9	Heme-binding protein 1 OS=Homo sapiens GN=HEBP1 PE=1 SV=1	0.00	2.74	0.00	0.00	0.00	0.00	0.00	0.00	0.00	0.00	0.00	1.31	0.00	0.00	0.00	0.00	0.00	0.00	0.00	0.00	0.00	0.00	4.82	0.00	0.34	0.40	>0.999999999999999	0.8
Q86XW9	Thioredoxin domain-containing protein 6 OS=Homo sapiens GN=NME9 PE=1 SV=1	0.00	1.54	2.15	0.00	0.00	0.00	0.00	0.00	0.00	0.00	0.00	0.73	0.00	0.00	0.00	0.00	0.00	0.00	0.00	0.00	0.00	0.00	0.00	0.00	0.37	0.00	>0.999999999999999	#DIV/0!
P10253	Lysosomal alpha-glucosidase OS=Homo sapiens GN=GAA PE=1 SV=4	0.00	0.51	0.00	1.28	0.00	0.00	0.00	0.00	0.00	0.00	0.00	0.49	0.00	0.00	0.00	0.50	0.00	0.00	0.00	0.00	0.00	0.48	0.00	0.00	0.19	0.08	>0.999999999999999	2.3
O95782	AP-2 complex subunit alpha-1 OS=Homo sapiens GN=AP2A1 PE=1 SV=3	0.00	0.51	1.43	1.28	0.00	2.13	0.00	0.00	0.00	0.00	3.15	0.49	0.00	0.00	1.43	1.01	0.00	0.00	0.00	0.00	0.00	0.00	1.84	0.12	0.75	0.37	>0.999999999999999	2.0
Q8WVJ2	NudC domain-containing protein 2 OS=Homo sapiens GN=NUDCD2 PE=1 SV=1	0.00	3.26	9.80	8.77	3.58	9.69	0.00	0.00	0.00	3.52	2.99	0.00	0.00	0.00	9.78	3.20	0.00	0.00	0.00	0.00	0.00	3.28	0.00	0.00	3.47	1.35	>0.999999999999999	2.6
P16471	Prolactin receptor OS=Homo sapiens GN=PRLR PE=1 SV=1	0.00	0.86	0.00	0.00	0.00	0.00	0.00	0.00	0.00	0.00	0.00	0.00	0.00	0.00	0.00	0.00	0.00	0.00	0.00	0.00	0.00	0.00	0.00	0.00	0.07	0.00	>0.999999999999999	#DIV/0!
P47914	60S ribosomal protein L29 OS=Homo sapiens GN=RPL29 PE=1 SV=2	0.00	3.26	4.54	4.06	3.58	4.49	3.25	1.72	2.60	3.52	2.99	1.55	4.47	3.05	4.53	3.20	0.00	0.00	0.00	0.00	0.00	0.00	0.00	0.00	2.96	1.27	>0.999999999999999	2.3
Q9UBS8	E3 ubiquitin-protein ligase RNF14 OS=Homo sapiens GN=RNF14 PE=1 SV=1	0.00	1.03	0.00	0.00	0.00	1.42	0.00	0.00	0.00	0.00	0.00	0.00	0.00	0.00	0.00	0.00	0.00	0.00	0.00	0.00	0.00	0.00	0.00	0.00	0.20	0.00	>0.999999999999999	#DIV/0!
Q09028	Histone-binding protein RBBP4 OS=Homo sapiens GN=RBBP4 PE=1 SV=3	0.00	1.20	3.35	2.99	0.00	3.31	0.00	0.63	0.00	1.30	0.00	0.00	0.00	0.00	0.00	0.00	0.00	0.00	0.00	0.00	0.00	0.00	0.99	0.00	1.06	0.08	>0.999999999999999	12.9
Q9P0R6	GSK3-beta interaction protein OS=Homo sapiens GN=GSKIP PE=1 SV=2	0.00	3.77	0.00	0.00	4.15	0.00	0.00	0.00	0.00	0.00	0.00	0.00	0.00	0.00	0.00	0.00	0.00	0.00	0.00	0.00	0.00	0.00	0.00	0.00	0.66	0.00	>0.999999999999999	#DIV/0!
O15498	Synaptobrevin homolog YKT6 OS=Homo sapiens GN=YKT6 PE=1 SV=1	0.00	2.57	0.00	0.00	0.00	0.00	2.56	0.00	0.00	0.00	0.00	1.22	0.00	0.00	3.58	0.00	0.00	0.00	0.00	0.00	0.00	0.00	0.00	0.00	0.53	0.30	>0.999999999999999	1.8
P62249	40S ribosomal protein S16 OS=Homo sapiens GN=RPS16 PE=1 SV=2	0.00	3.43	26.53	4.28	8.48	10.63	7.69	0.00	0.00	0.00	3.15	1.63	4.70	7.23	10.73	7.57	2.53	0.00	3.62	0.00	0.00	3.59	15.75	0.80	5.49	4.71	>0.999999999999999	1.2
P14324	Farnesyl pyrophosphate synthase OS=Homo sapiens GN=FDPS PE=1 SV=4	0.00	1.20	1.67	0.00	1.32	1.65	1.20	0.63	0.96	0.00	3.46	1.14	1.65	0.00	0.00	0.00	0.00	1.07	1.27	0.00	1.18	1.12	1.99	0.28	1.10	0.71	>0.999999999999999	1.5
Q15102	Platelet-activating factor acetylhydrolase IB subunit gamma OS=Homo sapiens GN=PAFAH1B3 PE=1 SV=1	0.00	2.23	3.11	2.78	2.45	3.07	2.22	0.00	0.00	2.41	2.04	0.00	0.00	0.00	0.00	0.00	0.00	0.00	0.00	0.00	0.00	1.04	1.84	0.00	1.69	0.24	>0.999999999999999	7.0
Q8NEP3	Dynein assembly factor 1, axonemal OS=Homo sapiens GN=DNAAF1 PE=1 SV=5	0.00	0.69	3.11	0.00	0.75	1.89	0.68	0.00	0.00	2.41	0.63	0.00	0.00	0.00	0.00	0.00	0.00	0.00	0.00	0.00	0.00	0.00	0.00	0.00	0.85	0.00	>0.999999999999999	#DIV/0!
Q8WWA0	Intelectin-1 OS=Homo sapiens GN=ITLN1 PE=1 SV=1	0.00	1.54	2.15	6.63	1.70	0.00	1.54	0.81	7.67	1.67	6.76	0.00	0.00	3.21	2.15	7.23	1.14	1.38	0.00	0.00	5.23	1.60	6.10	0.00	2.54	2.34	>0.999999999999999	1.1
Q969H8	UPF0556 protein C19orf10 OS=Homo sapiens GN=C19orf10 PE=1 SV=1	0.00	3.09	0.00	0.00	0.00	4.25	0.00	0.00	0.00	0.00	2.83	1.47	4.23	2.89	0.00	3.03	0.00	0.00	0.00	0.00	0.00	0.00	2.55	0.72	0.97	1.12	>0.999999999999999	0.9
Q9UHR4	Brain-specific angiogenesis inhibitor 1-associated protein 2-like protein 1 OS=Homo sapiens GN=BAIAP2L1 PE=1 S	0.00	1.03	1.43	1.28	0.00	0.00	0.00	0.00	0.00	1.11	0.00	0.00	0.00	0.00	0.00	0.00	0.00	0.00	0.00	0.00	0.00	0.00	0.00	0.00	0.40	0.00	>0.999999999999999	#DIV/0!
O15516	Circadian locomoter output cycles protein kaput OS=Homo sapiens GN=CLOCK PE=1 SV=1	0.00	0.51	0.00	0.00	0.00	0.00	0.00	0.00	0.00	0.00	0.00	0.00	0.00	0.00	0.00	0.00	0.00	0.00	0.00	0.00	0.00	0.00	0.00	0.00	0.04	0.00	>0.999999999999999	#DIV/0!
O15078	Centrosomal protein of 290 kDa OS=Homo sapiens GN=CEP290 PE=1 SV=2	0.00	0.17	0.24	0.00	0.00	0.00	0.00	0.00	0.00	0.00	0.00	0.00	0.00	0.00	0.00	0.00	0.00	0.00	0.00	0.00	0.00	0.00	0.00	0.00	0.03	0.00	>0.999999999999999	#DIV/0!
P11279	Lysosome-associated membrane glycoprotein 1 OS=Homo sapiens GN=LAMP1 PE=1 SV=3	0.00	1.20	0.00	0.00	0.00	0.00	0.00	0.00	0.00	0.00	0.00	1.88	0.00	0.00	0.00	1.18	0.00	0.00	0.00	0.00	0.00	0.00	0.00	0.28	0.26	0.12	>0.999999999999999	2.1
Q7Z3T8	Zinc finger FYVE domain-containing protein 16 OS=Homo sapiens GN=ZFYVE16 PE=1 SV=3	0.00	0.34	0.00	0.00	0.00	0.00	0.00	0.00	0.00	0.00	0.00	0.00	0.00	0.00	0.00	0.00	0.00	0.00	0.00	0.00	0.00	0.00	0.00	0.00	0.03	0.00	>0.999999999999999	#DIV/0!
Q8IWI9	MAX gene-associated protein OS=Homo sapiens GN=MGA PE=1 SV=2	0.00	0.17	0.24	0.00	0.19	0.00	0.00	0.00	0.00	0.19	0.16	0.00	0.00	0.00	0.24	0.00	0.00	0.00	0.00	0.00	0.00	0.00	0.00	0.00	0.08	0.02	>0.999999999999999	3.9
Q16513	Serine/threonine-protein kinase N2 OS=Homo sapiens GN=PKN2 PE=1 SV=1	0.00	0.51	0.00	0.00	0.00	0.00	0.00	0.00	0.00	0.00	0.00	0.00	0.00	0.00	0.00	0.00	0.00	0.00	0.00	0.00	0.00	0.00	0.00	0.00	0.04	0.00	>0.999999999999999	#DIV/0!
Q9HA90	Coiled-coil domain-containing protein 48 OS=Homo sapiens GN=CCDC48 PE=2 SV=2	0.00	0.86	0.00	0.00	0.00	0.00	0.00	0.00	0.00	0.00	0.00	0.00	0.00	0.00	0.00	0.00	0.00	0.00	0.00	0.00	0.00	0.00	0.00	0.00	0.07	0.00	>0.999999999999999	#DIV/0!
P10412	Histone H1.4 OS=Homo sapiens GN=HIST1H1E PE=1 SV=2	0.00	2.57	0.00	0.00	0.00	0.00	0.00	6.95	4.52	0.00	0.00	1.22	0.00	0.00	0.00	0.00	0.00	0.00	5.97	10.00	5.57	0.00	0.00	0.60	1.27	1.84	>0.999999999999999	0.7
P46109	Crk-like protein OS=Homo sapiens GN=CRKL PE=1 SV=1	0.00	1.72	2.39	2.14	0.00	2.36	1.71	0.00	0.00	0.00	1.57	0.00	0.00	0.00	0.00	0.00	0.00	0.00	0.00	0.00	0.00	0.00	0.00	0.00	0.99	0.00	>0.999999999999999	#DIV/0!
Q96L46	Calpain small subunit 2 OS=Homo sapiens GN=CAPNS2 PE=2 SV=2	0.00	2.06	0.00	0.00	0.00	0.00	0.00	0.00	0.00	0.00	0.00	0.00	0.00	0.00	0.00	0.00	0.00	0.00	0.00	0.00	0.00	0.00	0.00	0.00	0.17	0.00	>0.999999999999999	#DIV/0!
O00170	AH receptor-interacting protein OS=Homo sapiens GN=AIP PE=1 SV=2	0.00	1.54	2.15	1.92	0.00	2.13	0.00	0.00	0.00	0.00	1.42	0.73	0.00	0.00	2.15	0.00	0.00	0.00	0.00	0.00	0.00	0.00	0.00	0.00	0.82	0.18	>0.999999999999999	4.6
P09758	Tumor-associated calcium signal transducer 2 OS=Homo sapiens GN=TACSTD2 PE=1 SV=3	0.00	1.54	0.00	1.92	0.00	0.00	0.00	0.00	0.00	0.00	0.00	0.73	0.00	0.00	0.00	3.20	0.00	0.00	0.00	0.00	0.00	0.00	0.00	0.76	0.35	0.33	>0.999999999999999	1.1
Q02413	Desmoglein-1 OS=Homo sapiens GN=DSG1 PE=1 SV=2	0.00	0.51	0.00	0.00	0.00	0.00	0.00	0.00	0.00	0.00	0.00	0.00	0.00	0.48	0.00	0.00	0.00	0.00	0.00	0.00	0.00	0.00	0.00	0.00	0.04	0.04	>0.999999999999999	1.1
O15228	Dihydroxyacetone phosphate acyltransferase OS=Homo sapiens GN=GNPAT PE=1 SV=1	0.00	0.69	0.00	0.00	0.00	0.00	0.00	0.00	0.00	0.00	0.00	0.33	0.00	0.00	0.00	0.00	0.00	0.00	0.00	0.00	0.00	0.00	0.00	0.00	0.08	0.00	>0.999999999999999	#DIV/0!
Q01432	AMP deaminase 3 OS=Homo sapiens GN=AMPD3 PE=1 SV=1	0.00	0.69	0.96	0.86	0.00	0.00	0.00	0.00	0.00	0.00	1.10	0.00	0.00	0.00	0.00	0.00	0.00	0.00	0.00	0.00	0.00	0.00	0.00	0.00	0.30	0.00	>0.999999999999999	#DIV/0!
Q16774	Guanylate kinase OS=Homo sapiens GN=GUK1 PE=1 SV=2	0.00	2.57	0.00	7.06	0.00	7.80	2.56	0.00	0.00	2.78	2.36	1.22	0.00	0.00	0.00	2.52	1.90	0.00	2.71	0.00	0.00	0.00	0.00	0.00	2.20	0.59	>0.999999999999999	3.7
P61457	Pterin-4-alpha-carbinolamine dehydratase OS=Homo sapiens GN=PCBD1 PE=1 SV=2	0.00	4.80	0.00	23.95	0.00	0.00	0.00	0.00	0.00	0.00	0.00	0.00	0.00	0.00	0.00	0.00	0.00	0.00	0.00	0.00	0.00	2.24	0.00	0.00	2.40	0.19	>0.999999999999999	12.9
P16885	1-phosphatidylinositol-4,5-bisphosphate phosphodiesterase gamma-2 OS=Homo sapiens GN=PLCG2 PE=1 SV=4	0.00	0.34	0.00	0.00	0.00	0.00	0.00	0.00	0.00	0.00	0.00	0.00	0.00	0.00	0.00	0.00	0.00	0.00	0.00	0.00	0.00	0.00	0.00	0.00	0.03	0.00	>0.999999999999999	#DIV/0!
Q12904	Aminoacyl tRNA synthase complex-interacting multifunctional protein 1 OS=Homo sapiens GN=AIMP1 PE=1 SV=2	0.00	1.72	4.78	9.41	1.89	4.73	1.71	0.00	0.00	1.85	3.15	0.82	0.00	1.61	4.77	1.68	1.27	0.00	1.81	0.00	0.00	0.00	4.54	0.00	2.50	1.31	>0.999999999999999	1.9
O75116	Rho-associated protein kinase 2 OS=Homo sapiens GN=ROCK2 PE=1 SV=4	0.00	0.34	0.96	0.00	0.00	0.00	0.00	0.00	0.00	0.00	0.00	0.00	0.00	0.00	0.00	0.00	0.00	0.00	0.00	0.00	0.00	0.00	0.00	0.00	0.11	0.00	>0.999999999999999	#DIV/0!
P08621	U1 small nuclear ribonucleoprotein 70 kDa OS=Homo sapiens GN=SNRNP70 PE=1 SV=2	0.00	1.03	0.00	2.78	1.13	1.42	0.00	0.00	0.00	2.41	2.04	0.00	0.00	0.00	0.00	0.00	0.00	0.00	0.00	0.00	0.00	0.00	0.00	0.00	0.90	0.00	>0.999999999999999	#DIV/0!
Q9H201	Epsin-3 OS=Homo sapiens GN=EPN3 PE=1 SV=1	0.00	0.86	0.00	0.00	0.00	0.00	0.00	0.00	0.00	0.00	0.00	0.00	0.00	0.00	0.00	0.00	0.00	0.00	0.00	0.00	0.00	0.00	0.00	0.00	0.07	0.00	>0.999999999999999	#DIV/0!
Q9P2D3	HEAT repeat-containing protein 5B OS=Homo sapiens GN=HEATR5B PE=1 SV=2	0.00	0.17	0.00	0.00	0.00	0.00	0.00	0.00	0.00	0.00	0.16	0.00	0.00	0.00	0.00	0.00	0.00	0.00	0.00	0.00	0.00	0.08	0.14	0.00	0.03	0.02	>0.999999999999999	1.5
O15119	T-box transcription factor TBX3 OS=Homo sapiens GN=TBX3 PE=1 SV=4	0.00	0.69	0.00	0.00	0.00	0.00	0.00	0.00	0.00	0.00	0.00	0.00	0.00	0.00	0.00	0.00	0.00	0.00	0.00	0.00	0.00	0.00	0.00	0.00	0.06	0.00	>0.999999999999999	#DIV/0!
Q3ZCW2	Galectin-related protein OS=Homo sapiens GN=LGALSL PE=1 SV=2	0.00	3.09	0.00	0.00	0.00	0.00	0.00	0.00	0.00	0.00	0.00	0.00	0.00	0.00	4.29	0.00	0.00	0.00	0.00	0.00	0.00	0.00	0.00	0.00	0.26	0.36	>0.999999999999999	0.7

P57772	Selenocysteine-specific elongation factor OS=Homo sapiens GN=EEFSEC PE=1 SV=4	0.00	0.86	0.00	1.07	0.00	1.18	0.00	0.00	0.00	0.00	0.00	0.00	0.00	0.00	0.00	0.00	0.00	0.00	0.00	0.00	0.00	0.00	0.71	0.20	0.26	0.08	>0.999999999999999	3.4
P35580	Myosin-10 OS=Homo sapiens GN=MYH10 PE=1 SV=3	0.00	0.00	2.87	0.00	0.00	2.84	2.56	0.00	0.00	0.00	1.57	0.00	0.00	0.00	2.38	0.00	0.00	0.00	0.00	0.00	0.00	0.00	0.00	0.00	0.82	0.20	>0.999999999999999	4.1
P43353	Aldehyde dehydrogenase family 3 member B1 OS=Homo sapiens GN=ALDH3B1 PE=1 SV=1	0.00	0.00	3.11	0.00	0.00	4.73	0.00	0.00	0.00	0.00	3.15	0.00	0.00	0.00	0.00	0.00	0.00	0.00	0.00	0.00	0.00	0.00	0.00	0.00	0.91	0.00	>0.999999999999999	#DIV/0!
Q6S8J3	POTE ankyrin domain family member E OS=Homo sapiens GN=POTEE PE=1 SV=3	0.00	0.00	5.50	0.00	0.00	0.00	4.62	0.00	0.00	5.00	0.00	0.00	0.00	0.00	0.00	0.00	0.00	0.00	0.00	0.00	0.00	0.00	0.00	0.00	1.26	0.00	>0.999999999999999	#DIV/0!
P68032	Actin, alpha cardiac muscle 1 OS=Homo sapiens GN=ACTC1 PE=1 SV=1	0.00	0.00	13.63	0.00	0.00	0.00	0.00	0.00	0.00	0.00	0.00	9.14	0.00	0.00	0.00	0.00	0.00	0.00	0.00	0.00	0.00	0.00	0.00	0.00	1.90	0.00	>0.999999999999999	#DIV/0!
Q15029	116 kDa U5 small nuclear ribonucleoprotein component OS=Homo sapiens GN=EFTUD2 PE=1 SV=1	0.00	0.00	1.43	1.28	0.00	2.13	0.00	0.00	0.00	0.00	0.00	0.00	0.00	0.00	1.43	0.00	0.00	0.00	0.00	0.00	0.00	0.00	0.00	0.00	0.40	0.12	>0.999999999999999	3.4
Q9UBF2	Coatomer subunit gamma-2 OS=Homo sapiens GN=COPG2 PE=1 SV=1	0.00	0.00	1.67	1.50	0.00	0.00	0.00	0.00	0.00	0.00	0.00	1.14	0.71	0.48	0.00	1.68	0.00	0.00	0.00	0.00	0.00	0.00	0.00	0.40	0.36	0.27	>0.999999999999999	1.3
O75602	Sperm-associated antigen 6 OS=Homo sapiens GN=SPAG6 PE=1 SV=1	0.00	0.00	4.54	1.28	0.00	0.00	0.00	0.00	0.00	2.22	0.00	0.00	0.00	0.00	1.43	0.00	0.00	0.00	0.00	0.00	0.00	0.00	0.00	0.00	0.67	0.12	>0.999999999999999	5.6
Q14117	Dihydropyrimidinase OS=Homo sapiens GN=DPYS PE=1 SV=1	0.00	0.00	11.47	1.28	0.00	13.47	1.03	0.00	0.00	2.22	0.94	0.00	0.00	0.00	0.00	0.00	0.00	0.00	0.00	0.00	0.00	0.00	0.00	0.00	2.53	0.00	>0.999999999999999	#DIV/0!
O60256	Phosphoribosyl pyrophosphate synthase-associated protein 2 OS=Homo sapiens GN=PRPSAP2 PE=1 SV=1	0.00	0.00	6.22	0.00	0.00	6.14	0.00	0.00	0.00	4.82	0.00	0.00	0.00	0.00	1.91	0.00	0.00	0.00	0.00	0.00	0.00	0.00	0.00	0.00	1.43	0.16	>0.999999999999999	9.0
Q8TEX9	Importin-4 OS=Homo sapiens GN=IPO4 PE=1 SV=2	0.00	0.00	3.35	1.71	2.07	1.89	1.37	0.00	0.00	1.11	0.47	0.00	0.00	0.00	0.72	0.00	0.00	0.00	0.00	0.00	0.00	0.00	0.43	0.00	1.00	0.10	>0.999999999999999	10.5
Q9Y6D5	Brefeldin A-inhibited guanine nucleotide-exchange protein 2 OS=Homo sapiens GN=ARFGEF2 PE=1 SV=3	0.00	0.00	1.20	0.00	0.00	0.00	0.00	0.00	0.00	0.37	0.00	0.00	0.00	0.00	0.00	0.34	0.00	0.00	0.00	0.00	0.00	0.00	0.43	0.12	0.13	0.07	>0.999999999999999	1.8
Q9Y2T3	Guanine deaminase OS=Homo sapiens GN=GDA PE=1 SV=1	0.00	0.00	6.69	0.00	0.00	3.07	0.00	0.00	0.00	0.00	0.94	0.00	0.00	0.00	0.00	0.00	0.00	0.00	0.00	0.00	0.00	0.48	0.00	0.00	0.89	0.04	>0.999999999999999	22.3
Q5VW32	BRO1 domain-containing protein BROX OS=Homo sapiens GN=BROX PE=1 SV=1	0.00	0.00	5.50	4.92	1.32	5.43	2.56	0.00	0.00	2.78	2.36	0.57	0.00	0.00	5.49	2.52	1.90	0.00	0.00	0.00	0.00	1.20	2.13	0.00	2.12	1.10	>0.999999999999999	1.9
P35222	Catenin beta-1 OS=Homo sapiens GN=CTNNB1 PE=1 SV=1	0.00	0.00	2.87	0.00	1.51	1.89	1.37	0.00	0.00	1.48	2.52	0.65	0.00	0.00	1.91	0.00	0.00	0.00	0.00	0.00	0.00	0.00	0.00	0.00	1.02	0.16	>0.999999999999999	6.4
P12268	Inosine-5'-monophosphate dehydrogenase 2 OS=Homo sapiens GN=IMPDH2 PE=1 SV=2	0.00	0.00	6.22	5.56	2.26	6.14	0.00	0.00	0.00	0.00	0.94	0.00	0.00	0.96	2.86	1.01	0.00	0.00	0.00	0.00	0.00	0.00	0.00	0.00	1.76	0.40	>0.999999999999999	4.4
P41250	Glycine--tRNA ligase OS=Homo sapiens GN=GARS PE=1 SV=3	0.00	0.00	4.06	3.63	3.96	2.84	2.91	0.36	0.00	1.48	1.89	1.39	0.00	0.00	2.86	2.86	1.01	1.84	1.45	0.00	0.68	0.64	2.98	0.84	1.88	1.26	>0.999999999999999	1.5
P15428	15-hydroxyprostaglandin dehydrogenase [NAD(+)] OS=Homo sapiens GN=HPGD PE=1 SV=1	0.00	0.00	9.08	0.00	0.00	5.67	0.00	0.00	0.00	0.00	0.00	0.90	0.00	1.77	0.00	0.00	0.00	0.00	1.99	0.00	0.00	0.00	0.00	0.44	1.30	0.35	>0.999999999999999	3.7
O94973	AP-2 complex subunit alpha-2 OS=Homo sapiens GN=AP2A2 PE=1 SV=2	0.00	0.00	4.06	2.14	1.13	2.36	0.51	0.00	0.41	1.11	2.04	0.00	0.00	0.00	0.00	0.00	0.00	0.00	0.54	0.00	0.00	0.00	0.00	0.00	1.15	0.05	>0.999999999999999	25.4
O95373	Importin-7 OS=Homo sapiens GN=IPO7 PE=1 SV=1	0.00	0.00	2.63	1.07	1.51	0.71	0.00	0.00	0.00	0.93	0.47	0.65	0.00	0.00	1.19	0.50	0.00	0.46	0.54	0.00	0.00	0.00	0.71	0.20	0.66	0.30	>0.999999999999999	2.2
Q08211	ATP-dependent RNA helicase A OS=Homo sapiens GN=DHX9 PE=1 SV=4	0.00	0.00	1.20	1.07	0.38	2.84	1.20	0.00	0.00	0.37	1.57	0.00	0.00	0.00	0.48	0.00	0.00	0.00	0.00	0.00	0.00	0.16	0.00	0.00	0.72	0.05	>0.999999999999999	13.5
Q86Y56	HEAT repeat-containing protein 2 OS=Homo sapiens GN=HEATR2 PE=1 SV=4	0.00	0.00	2.63	1.50	0.57	3.54	0.00	0.00	0.00	2.04	1.73	0.00	0.00	0.00	0.72	0.00	0.00	0.00	0.00	0.00	0.00	0.00	0.43	0.00	1.00	0.10	>0.999999999999999	10.5
O75891	Cytosolic 10-formyltetrahydrofolate dehydrogenase OS=Homo sapiens GN=ALDH1L1 PE=1 SV=2	0.00	0.00	4.30	2.14	1.32	1.65	0.00	0.00	0.00	0.00	1.57	0.00	0.00	0.00	5.01	0.00	0.00	0.00	0.00	0.00	0.00	0.00	1.42	0.00	0.92	0.54	>0.999999999999999	1.7
Q92747	Actin-related protein 2/3 complex subunit 1A OS=Homo sapiens GN=ARPC1A PE=1 SV=2	0.00	0.00	3.82	3.42	4.71	8.27	5.98	0.00	2.19	2.96	3.93	0.00	0.00	0.00	0.00	0.00	0.00	2.45	2.89	0.00	0.00	2.80	2.27	0.00	2.94	0.87	>0.999999999999999	3.4
P54725	UV excision repair protein RAD23 homolog A OS=Homo sapiens GN=RAD23A PE=1 SV=1	0.00	0.00	4.06	3.63	7.16	6.38	0.00	0.00	0.00	0.00	4.25	1.39	0.00	2.73	0.00	0.00	1.01	1.23	0.00	0.00	0.00	0.00	0.00	1.08	2.24	0.50	>0.999999999999999	4.4
Q04206	Transcription factor p65 OS=Homo sapiens GN=RELA PE=1 SV=2	0.00	0.00	2.63	1.07	0.00	0.00	0.85	0.00	0.00	2.04	0.79	0.00	0.00	0.80	1.19	0.00	0.00	0.00	0.00	0.00	0.00	0.00	0.71	0.00	0.61	0.23	>0.999999999999999	2.7
P09488	Glutathione S-transferase Mu 1 OS=Homo sapiens GN=GSTM1 PE=1 SV=3	0.00	0.00	6.45	0.00	0.00	14.65	0.00	0.00	0.00	0.00	0.00	0.00	0.00	0.00	0.00	0.00	0.00	0.00	0.00	0.00	0.00	0.00	0.00	0.00	1.76	0.00	>0.999999999999999	#DIV/0!
Q99685	Monoglyceride lipase OS=Homo sapiens GN=MGLL PE=1 SV=2	0.00	0.00	5.02	0.00	0.00	4.96	0.00	0.00	0.00	0.00	0.00	0.00	0.00	0.00	0.00	0.00	0.00	0.00	0.00	0.00	0.00	0.00	0.00	0.00	0.83	0.00	>0.999999999999999	#DIV/0!
Q5SRE7	Phytanoyl-CoA dioxygenase domain-containing protein 1 OS=Homo sapiens GN=PHYHD1 PE=1 SV=2	0.00	0.00	8.13	4.49	3.96	8.03	1.71	0.00	1.37	3.89	1.57	1.71	0.00	0.00	2.38	0.00	0.00	0.00	0.00	0.00	0.00	0.80	0.00	0.00	2.91	0.27	>0.999999999999999	11.0
Q15393	Splicing factor 3B subunit 3 OS=Homo sapiens GN=SF3B3 PE=1 SV=4	0.00	0.00	1.20	0.43	1.32	0.00	0.34	0.00	0.00	1.30	0.00	0.16	0.00	0.00	0.00	0.00	0.00	0.00	0.00	0.00	0.00	0.00	0.00	0.08	0.40	0.01	>0.999999999999999	59.3
Q8NFP9	Neurobeachin OS=Homo sapiens GN=NBEA PE=1 SV=3	0.00	0.00	0.72	0.00	0.00	0.00	0.00	0.00	0.00	0.00	0.16	0.00	0.00	0.00	0.00	0.00	0.00	0.00	0.00	0.00	0.00	0.00	0.00	0.00	0.07	0.00	>0.999999999999999	#DIV/0!
Q9BV20	Methylthioribose-1-phosphate isomerase OS=Homo sapiens GN=MRI1 PE=1 SV=1	0.00	0.00	9.08	3.63	0.00	1.89	1.37	0.72	0.00	1.48	5.98	2.20	0.00	0.00	4.05	1.35	1.01	1.23	0.00	0.00	0.00	0.64	3.83	0.32	2.20	1.04	>0.999999999999999	2.1
Q8TC71	Mitochondria-eating protein OS=Homo sapiens GN=SPATA18 PE=1 SV=1	0.00	0.00	5.50	0.00	3.21	2.60	0.85	0.00	0.00	2.04	3.62	0.00	0.00	0.80	1.19	0.00	0.00	0.00	0.00	0.00	0.00	0.00	0.71	0.00	1.48	0.23	>0.999999999999999	6.6
Q8N1G4	Leucine-rich repeat-containing protein 47 OS=Homo sapiens GN=LRRC47 PE=1 SV=1	0.00	0.00	2.63	2.35	0.94	3.78	0.85	0.00	0.00	0.00	0.79	1.31	0.00	0.80	0.00	0.84	2.02	0.00	0.00	0.00	0.00	0.88	0.71	0.20	1.05	0.45	>0.999999999999999	2.3
Q8NCM8	Cytoplasmic dynein 2 heavy chain 1 OS=Homo sapiens GN=DYNC2H1 PE=1 SV=4	0.00	0.00	0.72	0.64	0.00	0.71	0.17	0.00	0.00	0.19	0.16	0.00	0.00	0.00	0.00	0.00	0.00	0.00	0.00	0.00	0.00	0.00	0.14	0.00	0.22	0.01	>0.999999999999999	18.2
Q16576	Histone-binding protein RBBP7 OS=Homo sapiens GN=RBBP7 PE=1 SV=1	0.00	0.00	3.35	4.70	1.32	3.31	0.00	0.00	0.00	1.30	0.00	0.00	0.00	0.00	0.00	0.00	0.00	0.00	0.00	0.00	0.00	1.12	0.00	0.28	1.16	0.12	>0.999999999999999	10.0
Q13618	Cullin-3 OS=Homo sapiens GN=CUL3 PE=1 SV=2	0.00	0.00	4.78	2.35	2.07	2.60	0.68	0.00	0.00	2.78	1.73	0.90	0.94	0.64	1.67	0.00	0.00	0.61	0.00	0.00	0.00	0.56	0.99	0.00	1.49	0.45	>0.999999999999999	3.3
Q9UI46	Dynein intermediate chain 1, axonemal OS=Homo sapiens GN=DNAI1 PE=1 SV=1	0.00	0.00	1.91	0.00	0.00	0.00	0.00	0.00	0.00	0.74	2.04	0.00	0.00	0.00	0.00	0.00	0.00	0.00	0.00	0.00	0.00	0.00	0.00	0.00	0.39	0.00	>0.999999999999999	#DIV/0!
O43813	LanC-like protein 1 OS=Homo sapiens GN=LANCL1 PE=1 SV=1	0.00	0.00	5.50	4.92	4.34	9.69	0.00	0.00	0.00	4.26	1.10	0.00	0.00	0.00	1.67	0.00	0.00	0.00	1.27	0.00	0.00	0.56	0.99	0.28	2.48	0.40	>0.999999999999999	6.3
P57076	Uncharacterized protein C21orf59 OS=Homo sapiens GN=C21orf59 PE=1 SV=1	0.00	0.00	5.02	4.49	1.89	7.80	0.00	0.00	0.00	3.89	3.30	0.00	0.00	0.00	0.00	0.00	0.00	0.00	0.00	0.00	0.00	0.00	0.00	0.00	2.20	0.00	>0.999999999999999	#DIV/0!
Q709C8	Vacuolar protein sorting-associated protein 13C OS=Homo sapiens GN=VPS13C PE=1 SV=1	0.00	0.00	0.24	0.00	0.00	0.47	0.00	0.00	0.00	0.00	0.00	0.08	0.00	0.00	0.00	0.00	0.00	0.00	0.00	0.00	0.00	0.00	0.14	0.00	0.07	0.01	>0.999999999999999	5.6
P21266	Glutathione S-transferase Mu 3 OS=Homo sapiens GN=GSTM3 PE=1 SV=3	0.00	0.00	6.22	0.00	2.26	9.92	0.00	0.00	0.00	2.22	1.89	0.98	0.00	0.00	0.00	0.00	0.00	0.00	0.00	0.00	0.00	0.00	0.00	0.48	1.96	0.04	>0.999999999999999	48.9
O95671	N-acetylserotonin O-methyltransferase-like protein OS=Homo sapiens GN=ASMTL PE=1 SV=3	0.00	0.00	2.39	0.00	0.00	0.00	0.00	0.00	0.00	1.85	0.00	0.00	0.00	0.00	1.19	0.84	0.00	1.53	0.00	0.00	0.00	1.20	0.71	0.00	0.35	0.46	>0.999999999999999	0.8
O75382	Tripartite motif-containing protein 3 OS=Homo sapiens GN=TRIM3 PE=1 SV=2	0.00	0.00	4.06	0.00	0.00	0.95	0.00	0.00	0.00	0.00	0.00	0.00	0.00	0.00	0.00	0.00	0.00	0.00	0.00	0.00	0.00	0.00	0.00	0.00	0.42	0.00	>0.999999999999999	#DIV/0!
P53365	Arfaptin-2 OS=Homo sapiens GN=ARFIP2 PE=1 SV=1	0.00	0.00	4.30	1.92	0.00	2.13	0.00	0.00	0.00	0.00	1.42	0.00	0.00	0.00	2.15	0.00	0.00	0.00	0.00	0.00	0.00	0.00	2.55	0.00	0.81	0.39	>0.999999999999999	2.1
O60610	Protein diaphanous homolog 1 OS=Homo sapiens GN=DIAPH1 PE=1 SV=2	0.00	0.00	1.67	1.50	1.32	0.47	0.34	0.00	0.27	0.00	0.00	0.57	0.00	0.32	1.19	0.34	0.00	0.00	0.00	0.00	0.00	0.56	1.42	0.20	0.51	0.34	>0.999999999999999	1.5
Q9NQG5	Regulation of nuclear pre-mRNA domain-containing protein 1B OS=Homo sapiens GN=RPRD1B PE=1 SV=1	0.00	0.00	4.54	0.00	0.00	0.00	0.00	0.00	0.00	1.67	0.00	0.00	0.00	0.00	0.00	0.00	0.00	0.00	0.00	0.00	0.00	0.72	0.00	0.00	0.52	0.06	>0.999999999999999	8.6
P09874	Poly [ADP-ribose] polymerase 1 OS=Homo sapiens GN=PARP1 PE=1 SV=4	0.00	0.00	1.43	0.64	0.00	1.42	0.51	0.00	0.00	1.11	0.00	0.00	0.00	0.00	0.00	0.00	0.00	0.00	0.00	0.00	0.00	0.00	0.00	0.00	0.43	0.00	>0.999999999999999	#DIV/0!
Q9NZB2	Constitutive coactivator of PPAR-gamma-like protein 1 OS=Homo sapiens GN=FAM120A PE=1 SV=2	0.00	0.00	1.20	1.07	0.00	1.18	0.00	0.00	0.00	0.56	0.00	0.00	0.00	0.00	0.00	0.00	0.00	0.00	0.00	0.00	0.00	0.00	0.00	0.00	0.33	0.00	>0.999999999999999	#DIV/0!
Q8WYA0	Intraflagellar transport protein 81 homolog OS=Homo sapiens GN=IFT81 PE=1 SV=1	0.00	0.00	1.91	0.86	0.00	1.89	0.00	0.00	0.00	0.74	2.04	0.00	0.00	0.00	0.00	0.00	0.00	0.00	0.00	0.00	0.00	0.00	0.00	0.00	0.62	0.00	>0.999999999999999	#DIV/0!
P13716	Delta-aminolevulinic acid dehydratase OS=Homo sapiens GN=ALAD PE=1 SV=1	0.00	0.00	4.54	4.06	1.70	4.49	0.00	0.00	0.00	1.67	2.99	0.73	0.00	0.00	7.15	1.51	0.00	1.38	0.00	0.00	0.00	0.72	1.28	0.00	1.68	1.00	>0.999999999999999	1.7
O14964	Hepatocyte growth factor-regulated tyrosine kinase substrate OS=Homo sapiens GN=HGS PE=1 SV=1	0.00	0.00	0.96	0.86	0.00	1.89	0.00	0.00	0.00	0.00	0.63	0.33	0.00	0.64	0.95	0.00	0.00	0.61	0.00	0.00	0.00	0.00	0.57	0.00	0.39	0.23	>0.999999999999999	1.7
Q14155	Rho guanine nucleotide exchange factor 7 OS=Homo sapiens GN=ARHGEF7 PE=1 SV=2	0.00	0.00	2.63	0.86	0.75	0.95	0.00	0.00	0.00	0.00	0.63	0.33	0.00	0.00	0.00	0.67	0.00	0.00	0.00	0.00	0.00	0.00	0.00	0.00	0.51	0.06	>0.999999999999999	9.1
Q99447	Ethanolamine-phosphate cytidylyltransferase OS=Homo sapiens GN=PCYT2 PE=1 SV=1	0.00	0.00	3.59	1.50	0.00	3.54	0.00	0.00	0.00	4.45	3.77	0.00	0.00	0.00	0.00	0.00	0.00	0.00	0.00	0.00	0.00	0.00	0.00	0.28	1.40	0.02	>0.999999999999999	60.1
O60884	DnaJ homolog subfamily A member 2 OS=Homo sapiens GN=DNAJA2 PE=1 SV=1	0.00	0.00	3.59	1.50	2.83	5.43	1.20	0.00	0.00	0.00	2.36	0.57	0.00	1.12	1.67	0.00	0.00	0.00	0.00	0.00	0.00	1.20	3.26	0.28	1.46	0.63	>0.999999999999999	2.3
P78559	Microtubule-associated protein 1A OS=Homo sapiens GN=MAP1A PE=1 SV=6	0.00	0.00	0.72	0.21	0.00	0.00	0.00	0.00	0.00	1.67	0.63	0.00	0.00	0.00	0.00	0.00	0.00	0.00	0.00	0.00	0.00	0.00	0.00	0.00	0.27	0.00	>0.999999999999999	#DIV/0!
Q9Y2S2	Lambda-crystallin homolog OS=Homo sapiens GN=CRYL1 PE=1 SV=3	0.00	0.00	7.17	6.41	3.58	7.09	0.00	0.00	0.00	3.52	1.42	0.00	0.00	0.00	2.15	1.51	0.00	0.00	3.44	0.00	0.00	1.52	1.28	0.00	2.43	0.82	>0.999999999999999	3.0
P06132	Uroporphyrinogen decarboxylase OS=Homo sapiens GN=UROD PE=1 SV=2	0.00	0.00	4.06	1.71	0.00	1.89	4.45	0.00	0.00	1.48	4.09	1.39	0.00	0.00	0.00	0.00	0.00	0.00	0.00	0.00	0.00	0.00	0.00	0.00	1.59	0.00	>0.999999999999999	#DIV/0!
P34913	Epoxide hydrolase 2 OS=Homo sapiens GN=EPHX2 PE=1 SV=2	0.00	0.00	3.82	1.07	0.00	3.78	0.00	0.00	0.00	0.93	0.00	0.41	0.00	0.00	1.19	0.00	0.00	0.00	0.00	0.00	0.00	0.00	0.71	0.00	0.83	0.16	>0.999999999999999	5.3
O95049	Tight junction protein ZO-3 OS=Homo sapiens GN=TJP3 PE=1 SV=2	0.00	0.00	1.43	0.64	0.00	0.71	0.00	0.00	0.00	0.00	0.47	0.00	0.00	0.00	0.72	0.00	0.00	0.00	0.00	0.00	0.00	0.00	0.00	0.00	0.27	0.06	>0.999999999999999	4.6
O14976	Cyclin-G-associated kinase OS=Homo sapiens GN=GAK PE=1 SV=2	0.00	0.00	0.48	0.43	0.38	0.00	0.00	0.00	0.00	0.00	0.00	0.16	0.00	0.00	0.00	0.00	0.00	0.00	0.00	0.00	0.00	0.00	0.28	0.00	0.12	0.02	>0.999999999999999	5.1
Q9UG01	Intraflagellar transport protein 172 homolog OS=Homo sapiens GN=IFT172 PE=1 SV=2	0.00	0.00	1.67	0.00	0.00	0.47	0.00	0.00	0.00	0.37	0.47	0.00	0.00	0.00	0.48	0.00	0.00	0.00	0.00	0.00	0.00	0.00	0.00	0.00	0.25	0.04	>0.999999999999999	6.3
Q13619	Cullin-4A OS=Homo sapiens GN=CUL4A PE=1 SV=3	0.00	0.00	2.87	0.00	0.75	1.89	0.00	0.00	0.00	0.00	0.00	0.00	0.00	0.00	0.00	0.00	0.00	0.00	0.00	0.00	0.00	0.00	0.00	0.00	0.46	0.00	>0.999999999999999	#DIV/0!
Q8TAT6	Nuclear protein localization protein 4 homolog OS=Homo sapiens GN=NPLOC4 PE=1 SV=3	0.00	0.00	2.39	1.07	1.89	0.00	1.71	0.00	0.00	0.93	4.09	0.41	0.00	0.00	1.19	0.84	0.63	0.00	0.00	0.00	0.00	0.40	0.71	0.20	1.04	0.33	>0.999999999999999	3.1
O60784	Target of Myb protein 1 OS=Homo sapiens GN=TOM1 PE=1 SV=2	0.00	0.00	3.11	0.00	0.00	3.07	0.00	0.00	0.00	1.11	0.00	0.49	0.00	0.96	3.10	0.00	0.00	0.00	0.00	0.00	0.00	0.48	0.85	0.00	0.65	0.45	>0.999999999999999	1.4
O75533	Splicing factor 3B subunit 1 OS=Homo sapiens GN=SF3B1 PE=1 SV=3	0.00	0.00	1.67	0.00	0.00	1.65	0.00	0.00	0.00	1.30	1.89	0.41	0.00	0.00	0.00	0.00	0.00	0.00	0.00	0.00	0.00	0.00	0.00	0.08	0.58	0.01	>0.999999999999999	86.4
Q9Y383	Putative RNA-binding protein Luc7-like 2 OS=Homo sapiens GN=LUC7L2 PE=1 SV=2	0.00	0.00	1.67	0.00	0.00	1.65	0.00	0.00	0.00	1.30	0.00	0.00	0.00	0.00	0.00	0.00	0.00	0.00	0.00	0.00	0.00	0.00	0.00	0.00	0.39	0.00	>0.999999999999999	#DIV/0!
Q8TE77	Protein phosphatase Slingshot homolog 3 OS=Homo sapiens GN=SSH3 PE=1 SV=2	0.00	0.00	0.96	0.86	0.00	0.95	0.00	0.00	0.00	0.74	0.63	0.33	0.00	0.00	0.95	0.67	0.00	0.00	0.00	0.00	0.00	0.32	0.00	0.16	0.37	0.18	>0.999999999999999	2.1
Q8N1V2	WD repeat-containing protein 16 OS=Homo sapiens GN=WDR16 PE=1 SV=3	0.00	0.00	1.20	0.00	0.00	0.00	0.00	0.00	0.00	0.00	0.00	0.00	0.00	0.00	0.00	0.00	0.00	0.00	0.00	0.00	0.00	0.00	0.71	0.00	0.10	0.06	>0.999999999999999	1.7
Q96CV9	Optineurin OS=Homo sapiens GN=OPTN PE=1 SV=2	0.00	0.00	1.20	2.14	0.00	3.78	0.00	0.00	0.00	0.93	0.00	0.82	0.00	0.00	2.38	0.00	0.00	0.00	0.00	0.00	0.00	0.40	0.00	0.20	0.74	0.25	>0.999999999999999	3.0
Q96T76	MMS19 nucleotide excision repair protein homolog OS=Homo sapiens GN=MMS19 PE=1 SV=2	0.00	0.00	1.43	0.64	0.00	1.42	0.00	0.00	0.00	0.00	0.47	0.00	0.00	0.00	0.72	0.00	0.00	0.00	0.00	0.00	0.00	0.00	0.00	0.00	0.33	0.06	>0.999999999999999	5.5
Q86UP2	Kinectin OS=Homo sapiens GN=KTN1 PE=1 SV=1	0.00	0.00	1.43	0.86	0.75	0.47	0.00	0.00	0.00	1.11	0.31	0.00	0.00	0.00	0.00	0.00	0.00	0.00	0.00	0.00	0.00	0.00	0.00	0.00	0.41	0.00	>0.999999999999999	#DIV/0!
Q5VYK3	Proteasome-associated protein ECM29 homolog OS=Homo sapiens GN=ECM29 PE=1 SV=2	0.00	0.00	0.72	0.64	0.38	0.47	0.34	0.00	0.00	0.56	0.31	0.16	0.00	0.00	0.72	0.34	0.00	0.00	0.00	0.00	0.00	0.16	0.28	0.12	0.30	0.13	>0.999999999999999	2.2
P20073	Annexin A7 OS=Homo sapiens GN=ANXA7 PE=1 SV=3	0.00	0.00	1.43	0.00	0.00	1.42	0.00	0.00	0.00	1.11	0.00	0.00	0.00	0.00	1.43	0.00	0.00	0.00	0.00	0.00	0.00	0.00	0.00	0.00	0.33	0.12	>0.999999999999999	2.8
Q15404	Ras suppressor protein 1 OS=Homo sapiens GN=RSU1 PE=1 SV=3	0.00	0.00	2.63	2.35	0.00	0.00	1.88	0.00	0.00	0.00	0.00	0.90	0.00	0.00	0.00	0.00	0.00	0.00	0.00	0.00	0.00	0.00	0.00	0.00	0.65	0.00	>0.999999999999999	#DIV/0!
Q53H96	Pyrroline-5-carboxylate reductase 3 OS=Homo sapiens GN=PYCRL PE=1 SV=2	0.00	0.00	2.63	0.00	0.00	5.67	0.00	0.00	0.00	2.04	1.73	0.00	0.00	0.00	2.62	0.00	0.00	0.00	0.00	0.00	0.00	0.00	0.00	0.00	1.01	0.22	>0.999999999999999	4.6
Q9H4A5	Golgi phosphoprotein 3-like OS=Homo sapiens GN=GOLPH3L PE=1 SV=1	0.00	0.00	2.39	4.49	0.00	0.00	0.00	0.00	0.00	0.00	0.00	0.82	0.00	0.00	2.38	0.00	0.00	0.00	0.00	0.00	0.00	0.00	1.42	0.00	0.64	0.32	>0.999999999999999	2.0
Q14008	Cytoskeleton-associated protein 5 OS=Homo sapiens GN=CKAP5 PE=1 SV=3	0.00	0.00	0.72	0.00	0.00	0.71	0.00	0.00	0.00	0.19	0.16	0.00	0.00	0.00	0.24	0.00	0.00	0.00	0.00	0.00	0.00	0.00	0.00	0.00	0.15	0.02	>0.999999999999999	7.4
Q9Y3D0	Mitotic spindle-associated MMXD complex subunit MIP18 OS=Homo sapiens GN=FAM96B PE=1 SV=1	0.00	0.00	10.04	0.00	0.00	4.49	3.25	0.00	0.00	0.00	2.99	0.00	0.00	0.00	0.00	3.20	0.00	0.00	0.00	0.00	0.00	0.00	2.70	0.00	1.73	0.49	>0.999999999999999	3.5
Q96AJ1	Clusterin-associated protein 1 OS=Homo sapiens GN=CLUAP1 PE=1 SV=4	0.00	0.00	3.35	0.00	0.00	1.65	0.00	0.00	0.00	0.00	0.00	0.00	0.00	0.00	0.00	0.00	0.00	0.00	0.00	0.00	0.00	0.00	0.00	0.00	0.42	0.00	>0.999999999999999	#DIV/0!
Q7Z5L9	Interferon regulatory factor 2-binding protein 2 OS=Homo sapiens GN=IRF2BP2 PE=1 SV=2	0.00	0.00	1.20	0.00	0.00	0.00	0.00	0.00	0.00	0.00	0.00	0.00	0.00	0.00	0.00	0.00	0.00	0.00	0.00	0.00	0.00	0.00	0.00	0.00	0.10	0.00	>0.999999999999999	#DIV/0!
Q9C0B1	Alpha-ketoglutarate-dependent dioxygenase FTO OS=Homo sapiens GN=FTO PE=1 SV=3	0.00	0.00	1.43	1.28	0.00	0.00	0.00	0.00	0.00	0.00	0.00	0.00	0.00	0.00	0.00	0.00	0.00	0.00	0.00	0.00	0.00	0.00	0.00	0.00	0.23	0.00	>0.999999999999999	#DIV/0!
Q6P6B1	Uncharacterized protein C8orf47 OS=Homo sapiens GN=C8orf47 PE=2 SV=1	0.00	0.00	4.06	1.71	0.00	1.89	0.00	0.00	0.00	1.48	1.26	0.00	0.00	0.00	1.91	0.00	0.00	0.00	0.00	0.00	0.00	0.00	0.00	0.00	0.87	0.16	>0.999999999999999	5.5
P52888	Thimet oligopeptidase OS=Homo sapiens GN=THOP1 PE=1 SV=2	0.00	0.00	1.91	0.86	1.51	0.95	0.00	0.00	0.00	1.48	0.63	0.00	0.00	0.00	0.95	0.00	0.00	0.00	0.00	0.00	0.00	0.00	0.00	0.00	0.61	0.08	>0.999999999999999	7.7
P26368	Splicing factor U2AF 65 kDa subunit OS=Homo sapiens GN=U2AF2 PE=1 SV=4	0.00	0.00	3.11	2.78	2.45	0.00	1.03	0.00	0.00	1.11	0.00	0.49	0.00	0.00	0.00	0.00	0.00	1.09	0.00	0.00	0.00	0.00	0.85	0.24	0.91	0.18	>0.999999999999999	5.0
P61758	Prefoldin subunit 3 OS=Homo sapiens GN=VBP1 PE=1 SV=3	0.00	0.00	7.41	3.21	0.00	0.00	0.00	0.00	0.00	0.00	0.00	1.22	0.00	0.00	0.00	0.00	0.00	0.00	0.00	0.00	0.00	0.00	0.00	0.60	0.99	0.05	>0.999999999999999	19.7
P11172	Uridine 5'-monophosphate synthase OS=Homo sapiens GN=UMPS PE=1 SV=1	0.00	0.00	3.11	1.28	1.13	1.42	0.00	0.54	0.00	1.11	3.15	0.00	0.00	0.96	0.00	1.01	0.00	0.00	0.00	0.00	0.00	0.48	0.85	0.00	0.98	0.28	>0.999999999999999	3.6
Q9P2L0	WD repeat-containing protein 35 OS=Homo sapiens GN=WDR35 PE=1 SV=3	0.00	0.00	0.48	0.43	0.00	0.47	0.00	0.00	0.00	0.00	0.31	0.00	0.00	0.00	0.48	0.00	0.00	0.00	0.00	0.00	0.00	0.00	0.00	0.00	0.14	0.04	>0.999999999999999	3.5
O15523	ATP-dependent RNA helicase DDX3Y OS=Homo sapiens GN=DDX3Y PE=1 SV=2	0.00	0.00	2.15	0.00	0.00	0.00	0.00	0.00	0.00	0.00	0.00	0.00	0.00	0.00	0.00	0.00	0.00	0.00	0.00	0.00	0.00	0.00	0.00	0.00	0.18	0.00	>0.999999999999999	#DIV/0!
Q96CN7	Isochorismatase domain-containing protein 1 OS=Homo sapiens GN=ISOC1 PE=1 SV=3	0.00	0.00	2.39	2.14	0.00	0.00	0.00	0.00	0.00	0.00	1.57	0.00	0.00	0.00	2.38	0.00	0.00	0.00	0.00	0.00	0.00	1.68	2.98	0.40	0.51	0.62	>0.999999999999999	0.8
P67870	Casein kinase II subunit beta OS=Homo sapiens GN=CSNK2B PE=1 SV=1	0.00	0.00	3.11	0.00	0.00	0.00	0.00	0.00	0.00	0.00	7.08	1.06	0.00	0.00	0.00	0.00	0.00	0.00	0.00	0.00	0.00	0.00	0.00	0.00	0.94	0.00	>0.999999999999999	#DIV/0!
Q9NQA3	WAS protein family homolog 6 OS=Homo sapiens GN=WASH6P PE=1 SV=3	0.00	0.00	1.67	0.00	0.00	0.00	0.00	0.00	0.00	0.00	0.00	0.00	0.00	0.00	0.00	0.00	0.00	0.00	0.00	0.00	0.00	0.00	0.00	0.00	0.14	0.00	>0.999999999999999	#DIV/0!
P00167	Cytochrome b5 OS=Homo sapiens GN=CYB5A PE=1 SV=2	0.00	0.00	11.71	0.00	4.15	0.00	0.00	0.00	0.00	0.00	0.00	0.00	0.00	0.00	0.00	0.00	0.00	0.00	0.00	0.00	0.00	0.00	0.00	0.00	1.32	0.00	>0.999999999999999	#DIV/0!
Q2M389	WASH complex subunit 7 OS=Homo sapiens GN=KIAA1033 PE=1 SV=2	0.00	0.00	1.67	0.00	0.00	1.18	0.00	0.00	0.00	0.00	0.00	0.00	0.00	0.00	0.00	0.00	0.00	0.00	0.00	0.00	0.00	0.00	0.00	0.00	0.24	0.00	>0.999999999999999	#DIV/0!
Q9BXC9	Bardet-Biedl syndrome 2 protein OS=Homo sapiens GN=BBS2 PE=1 SV=1	0.00	0.00	0.96	0.00	0.00	0.00	0.00	0.00	0.00	0.00	0.00	0.00	0.00	0.00	0.00	0.00	0.00	0.00	0.00	0.00	0.00	0.00	0.00	0.00	0.08	0.00	>0.999999999999999	#DIV/0!
Q13099	Intraflagellar transport protein 88 homolog OS=Homo sapiens GN=IFT88 PE=2 SV=2	0.00	0.00	2.63	0.00	0.00	2.60	0.00	0.00	0.00	0.56	1.10	0.00	0.00	0.00	0.00	0.00	0.00	0.00	0.00	0.00	0.00	0.00	0.00	0.00	0.57	0.00	>0.999999999999999	#DIV/0!
Q969H4	Connector enhancer of kinase suppressor of ras 1 OS=Homo sapiens GN=CNKSR1 PE=1 SV=1	0.00	0.00	0.96	0.00	0.00	0.95	0.00	0.00	0.00	0.00	0.00	0.33	0.00	0.00	0.00	0.00	0.00	0.00	0.00	0.00	0.00	0.00	0.00	0.00	0.19	0.00	>0.999999999999999	#DIV/0!
Q93034	Cullin-5 OS=Homo sapiens GN=CUL5 PE=1 SV=4	0.00	0.00	1.67	0.00	0.75	0.95	0.68	0.00	0.00	0.74	0.00	0.00	0.00	0.00	0.95	0.00	0.00	0.00	0.00	0.00	0.00	0.32	0.57	0.00	0.40	0.15	>0.999999999999999	2.6
P56597	Nucleoside diphosphate kinase homolog 5 OS=Homo sapiens GN=NME5 PE=1 SV=1	0.00	0.00	3.35	0.00	2.64	3.31	0.00	0.00	0.00	8.71	2.20	0.00	0.00	0.00	0.00	0.00	0.00	0.00	0.00	0.00	0.00	1.12	0.00	0.00	1.68	0.09	>0.999999999999999	18.1
Q9UKG1	DCC-interacting protein 13-alpha OS=Homo sapiens GN=APPL1 PE=1 SV=1	0.00	0.00	0.96	0.00	0.00	0.00	0.00	0.00	0.00	0.00	0.00	0.00	0.00	0.00	0.00	0.00	0.00	0.00	0.00	0.00	0.00	0.00	0.00	0.00	0.08	0.00	>0.999999999999999	#DIV/0!
P53582	Methionine aminopeptidase 1 OS=Homo sapiens GN=METAP1 PE=1 SV=2	0.00	0.00	1.67	1.50	0.00	1.65	0.00	0.00	0.00	0.00	2.52	0.57	0.00	0.00	0.00	0.00	0.00	0.00	0.00	0.00	0.00	0.56	0.99	0.28	0.66	0.15	>0.999999999999999	4.3
P08397	Porphobilinogen deaminase OS=Homo sapiens GN=HMBS PE=1 SV=2	0.00	0.00	1.91	1.71	0.00	0.00	0.00	0.00	0.00	0.00	0.00	0.65	0.00	0.00	0.00	0.00	0.00	0.00	0.00	0.00	0.00	0.64	1.14	0.00	0.36	0.15	>0.999999999999999	2.4
Q96IU4	Abhydrolase domain-containing protein 14B OS=Homo sapiens GN=ABHD14B PE=1 SV=1	0.00	0.00	7.65	6.84	2.83	12.29	2.56	0.00	2.05	2.78	8.18	1.22	0.00	2.41	3.58	5.38	0.00	4.91	5.79	0.00	2.53	2.56	14.33	0.00	3.87	3.46	>0.999999999999999	1.1
Q13951	Core-binding factor subunit beta OS=Homo sapiens GN=CBFB PE=1 SV=2	0.00	0.00	3.59	7.06	0.00	0.00	0.00	0.00	0.00	0.00	0.00	0.00	0.00	0.00	0.00	0.00	2.52	0.00	0.00	0.00	0.00	0.00	0.00	0.00	0.89	0.21	>0.999999999999999	4.2
P09132	Signal recognition particle 19 kDa protein OS=Homo sapiens GN=SRP19 PE=1 SV=3	0.00	0.00	5.02	0.00	0.00	10.63	0.00	0.00	0.00	3.89	0.00	1.71	0.00	0.00	0.00	0.00	0.00	0.00	0.00	0.00	0.00	0.00	0.00	0.00	1.77	0.00	>0.999999999999999	#DIV/0!
Q9BVJ7	Dual specificity protein phosphatase 23 OS=Homo sapiens GN=DUSP23 PE=1 SV=1	0.00	0.00	4.78	0.00	0.00	0.00	0.00	0.00	0.00	0.00	3.15	0.00	0.00	0.00	10.49	0.00	0.00	0.00	0.00	0.00	0.00	0.00	0.00	0.00	0.66	0.87	>0.999999999999999	0.8
Q53EV4	Leucine-rich repeat-containing protein 23 OS=Homo sapiens GN=LRRC23 PE=2 SV=2	0.00	0.00	1.91	1.71	0.00	0.00	0.00	0.00	0.00	1.48	0.00	0.00	0.00	0.00	0.00	0.00	0.00	0.00	0.00	0.00	0.00	0.00	0.00	0.00	0.43	0.00	>0.999999999999999	#DIV/0!
Q8IYI6	Exocyst complex component 8 OS=Homo sapiens GN=EXOC8 PE=1 SV=2	0.00	0.00	0.96	0.00	0.00	0.00	0.00	0.00	0.00	0.00	0.63	0.00	0.00	0.00	0.00	0.00	0.00	0.00	0.00	0.00	0.00	0.00	0.00	0.00	0.13	0.00	>0.999999999999999	#DIV/0!
Q92882	Osteoclast-stimulating factor 1 OS=Homo sapiens GN=OSTF1 PE=1 SV=2	0.00	0.00	3.35	2.99	0.00	3.31	2.39	0.00	0.00	2.59	2.20	2.45	3.29	0.00	3.34	2.36	0.00	0.00	0.00	0.00	0.00	1.12	1.99	0.56	1.61	1.05	>0.999999999999999	1.5
Q9NSD9	Phenylalanine--tRNA ligase beta subunit OS=Homo sapiens GN=FARSB PE=1 SV=3	0.00	0.00	3.82	5.77	0.94	4.96	1.71	0.00	0.00	0.93	1.57	0.41	0.00	0.00	1.19	1.68	0.00	0.00	0.00	0.00	0.00	0.80	2.27	0.00	1.68	0.50	>0.999999999999999	3.4
Q8NFF5	FAD synthase OS=Homo sapiens GN=FLAD1 PE=1 SV=1	0.00	0.00	2.39	1.07	0.00	1.18	0.00	0.00	0.00	0.93	0.00	0.00	0.00	0.00	1.19	0.00	0.00	0.00	0.00	0.00	0.00	0.40	0.00	0.00	0.46	0.13	>0.999999999999999	3.5
P53367	Arfaptin-1 OS=Homo sapiens GN=ARFIP1 PE=1 SV=2	0.00	0.00	3.82	5.56	0.00	1.89	0.00	0.00	0.00	0.00	5.66	0.00	0.00	0.00	0.00	0.00	0.00	0.00	0.00	0.00	0.00	0.00	3.69	1.04	1.41	0.39	>0.999999999999999	3.6
O15355	Protein phosphatase 1G OS=Homo sapiens GN=PPM1G PE=1 SV=1	0.00	0.00	2.63	1.07	3.21	1.18	0.85	0.00	0.00	2.04	0.79	0.41	0.00	0.00	0.00	0.00	0.00	0.00	0.00	0.00	0.00	0.00	0.00	0.20	1.01	0.02	>0.999999999999999	60.8
P18615	Negative elongation factor E OS=Homo sapiens GN=RDBP PE=1 SV=3	0.00	0.00	1.91	0.00	0.00	0.00	0.00	0.00	0.00	0.00	0.00	0.00	0.00	0.00	0.00	0.00	0.00	0.00	0.00	0.00	0.00	0.00	0.00	0.00	0.16	0.00	>0.999999999999999	#DIV/0!
Q92845	Kinesin-associated protein 3 OS=Homo sapiens GN=KIFAP3 PE=1 SV=2	0.00	0.00	1.67	0.00	0.00	0.95	0.00	0.00	0.00	0.00	0.63	0.00	0.00	0.00	0.00	0.00	0.00	0.00	0.00	0.00	0.00	0.00	0.00	0.00	0.27	0.00	>0.999999999999999	#DIV/0!
Q7L5Y1	Mitochondrial enolase superfamily member 1 OS=Homo sapiens GN=ENOSF1 PE=1 SV=1	0.00	0.00	1.67	1.50	3.96	1.65	0.00	0.00	0.96	1.30	0.00	0.00	0.00	0.00	0.00	0.00	0.00	0.00	0.00	0.00	0.00	0.00	0.00	0.00	0.92	0.00	>0.999999999999999	#DIV/0!
Q96JH7	Deubiquitinating protein VCIP135 OS=Homo sapiens GN=VCPIP1 PE=1 SV=2	0.00	0.00	1.20	0.43	0.38	0.00	0.00	0.00	0.00	0.37	0.00	0.00	0.00	0.00	0.00	0.00	0.00	0.00	0.00	0.00	0.00	0.00	0.00	0.00	0.20	0.00	>0.999999999999999	#DIV/0!
Q13057	Bifunctional coenzyme A synthase OS=Homo sapiens GN=COASY PE=1 SV=4	0.00	0.00	1.20	1.07	0.94	2.60	0.85	0.00	0.00	0.00	0.79	0.41	0.00	0.00	0.00	0.00	0.00	0.00	0.00	0.00	0.00	0.00	0.00	0.20	0.65	0.02	>0.999999999999999	39.3
Q9Y5K5	Ubiquitin carboxyl-terminal hydrolase isozyme L5 OS=Homo sapiens GN=UCHL5 PE=1 SV=3	0.00	0.00	2.15	0.00	0.00	0.00	0.00	0.00	0.00	1.67	1.42	1.47	0.00	0.00	0.00	0.00	0.00	1.63	0.00	0.00	0.00	0.00	1.28	0.00	0.56	0.24	>0.999999999999999	2.3
Q96KP1	Exocyst complex component 2 OS=Homo sapiens GN=EXOC2 PE=1 SV=1	0.00	0.00	0.72	0.00	0.00	0.00	0.00	0.00	0.00	0.00	0.00	0.00	0.00	0.00	0.72	0.00	0.00	0.00	0.00	0.00	0.00	0.00	0.00	0.00	0.06	0.06	>0.999999999999999	1.0
A1Z1Q3	MACRO domain-containing protein 2 OS=Homo sapiens GN=MACROD2 PE=1 SV=1	0.00	0.00	1.67	0.00	0.00	0.00	0.00	0.00	0.00	0.00	0.00	0.00	0.00	0.00	0.00	0.00	0.00	0.00	0.00	0.00	0.00	0.00	0.00	0.00	0.14	0.00	>0.999999999999999	#DIV/0!
Q99627	COP9 signalosome complex subunit 8 OS=Homo sapiens GN=COPS8 PE=1 SV=1	0.00	0.00	7.41	2.99	0.00	7.33	0.00	0.00	0.00	0.00	2.20	0.00	0.00	0.00	0.00	0.00	0.00	0.00	2.53	0.00	0.00	0.00	0.00	0.00	1.66	0.21	>0.999999999999999	7.9
Q8TE73	Dynein heavy chain 5, axonemal OS=Homo sapiens GN=DNAH5 PE=1 SV=3	0.00	0.00	0.48	0.00	0.00	0.00	0.00	0.00	0.00	0.00	0.16	0.00	0.00	0.00	0.00	0.00	0.00	0.00	0.00	0.00	0.00	0.00	0.00	0.00	0.05	0.00	>0.999999999999999	#DIV/0!
P02750	Leucine-rich alpha-2-glycoprotein OS=Homo sapiens GN=LRG1 PE=1 SV=2	0.00	0.00	2.15	0.00	1.70	2.13	0.00	0.81	3.83	0.00	0.00	1.47	0.00	0.00	0.00	0.00	2.28	0.00	1.63	1.17	1.52	0.00	0.00	1.12	1.01	0.64	>0.999999999999999	1.6
Q5JR98	Tctex1 domain-containing protein 4 OS=Homo sapiens GN=TCTEX1D4 PE=1 SV=1	0.00	0.00	3.35	0.00	0.00	3.31	0.00	0.00	0.00	0.00	0.00	0.00	0.00	0.00	0.00	0.00	0.00	0.00	0.00	0.00	0.00	0.00	0.00	0.00	0.55	0.00	>0.999999999999999	#DIV/0!
P14621	Acylphosphatase-2 OS=Homo sapiens GN=ACYP2 PE=1 SV=2	0.00	0.00	7.41	6.63	5.85	7.33	0.00	0.00	0.00	0.00	4.88	2.53	0.00	0.00	7.39	5.22	0.00	0.00	0.00	0.00	0.00	2.48	0.00	1.24	2.88	1.36	>0.999999999999999	2.1
Q8N668	COMM domain-containing protein 1 OS=Homo sapiens GN=COMMD1 PE=1 SV=1	0.00	0.00	3.82	0.00	0.00	0.00	0.00	0.00	0.00	0.00	2.52	0.00	0.00	0.00	0.00	0.00	0.00	0.00	0.00	0.00	0.00	0.00	0.00	0.00	0.53	0.00	>0.999999999999999	#DIV/0!
Q9C040	Tripartite motif-containing protein 2 OS=Homo sapiens GN=TRIM2 PE=1 SV=1	0.00	0.00	1.91	0.00	0.00	0.95	0.00	0.00	0.00	0.00	0.00	0.00	0.00	0.00	0.00	0.00	0.00	0.00	0.00	0.00	0.00	0.00	0.00	0.00	0.24	0.00	>0.999999999999999	#DIV/0!
Q13616	Cullin-1 OS=Homo sapiens GN=CUL1 PE=1 SV=2	0.00	0.00	0.96	0.86	0.75	0.95	0.00	0.00	0.00	0.00	0.00	0.00	0.00	0.00	0.00	0.00	0.00	0.00	0.00	0.00	0.00	0.00	0.57	0.00	0.29	0.05	>0.999999999999999	6.2
Q9GZS0	Dynein intermediate chain 2, axonemal OS=Homo sapiens GN=DNAI2 PE=1 SV=2	0.00	0.00	1.20	0.00	0.00	0.00	0.00	0.00	0.00	0.93	0.00	0.00	0.00	0.00	0.00	0.00	0.00	0.00	0.00	0.00	0.00	0.00	0.00	0.00	0.18	0.00	>0.999999999999999	#DIV/0!
Q9NUQ8	ATP-binding cassette sub-family F member 3 OS=Homo sapiens GN=ABCF3 PE=1 SV=2	0.00	0.00	0.96	0.00	0.00	0.00	0.00	0.00	0.00	0.74	0.63	0.33	0.00	0.00	0.95	0.67	0.00	0.00	0.00	0.00	0.00	0.00	0.57	0.16	0.22	0.20	>0.999999999999999	1.1
P09525	Annexin A4 OS=Homo sapiens GN=ANXA4 PE=1 SV=4	0.00	0.00	2.15	0.00	0.00	7.09	0.00	0.00	0.00	1.67	0.00	0.00	0.00	0.00	0.00	0.00	0.00	0.00	0.00	0.00	0.00	0.00	0.00	0.00	0.91	0.00	>0.999999999999999	#DIV/0!
Q96P70	Importin-9 OS=Homo sapiens GN=IPO9 PE=1 SV=3	0.00	0.00	0.72	0.64	0.00	0.00	0.51	0.00	0.00	0.00	0.00	0.00	0.00	0.00	0.00	0.00	0.00	0.00	0.00	0.00	0.00	0.00	0.00	0.00	0.16	0.00	>0.999999999999999	#DIV/0!
Q9BUH6	Uncharacterized protein C9orf142 OS=Homo sapiens GN=C9orf142 PE=1 SV=2	0.00	0.00	3.59	3.21	2.83	0.00	0.00	0.00	0.00	0.00	0.00	0.00	0.00	2.41	0.00	0.00	0.00	0.00	0.00	0.00	0.00	0.00	2.13	0.00	0.80	0.38	>0.999999999999999	2.1
P56945	Breast cancer anti-estrogen resistance protein 1 OS=Homo sapiens GN=BCAR1 PE=1 SV=2	0.00	0.00	0.96	0.00	0.00	0.00	0.00	0.00	0.00	0.00	0.63	0.00	0.00	0.00	0.00	0.00	0.00	0.00	0.00	0.00	0.00	0.32	0.00	0.16	0.13	0.04	>0.999999999999999	3.3
O95396	Adenylyltransferase and sulfurtransferase MOCS3 OS=Homo sapiens GN=MOCS3 PE=1 SV=1	0.00	0.00	1.67	0.00	0.00	0.00	0.00	0.00	0.00	0.00	2.04	0.00	0.00	0.00	0.00	0.00	0.00	0.00	0.00	0.00	0.00	0.56	0.00	0.00	0.31	0.05	>0.999999999999999	6.6
Q96CX2	BTB/POZ domain-containing protein KCTD12 OS=Homo sapiens GN=KCTD12 PE=1 SV=1	0.00	0.00	2.15	1.92	0.00	0.00	0.00	0.00	0.00	0.00	0.00	0.73	0.00	0.00	0.00	0.00	0.00	0.00	0.00	0.00	0.00	0.72	0.00	0.00	0.40	0.06	>0.999999999999999	6.7
Q15560	Transcription elongation factor A protein 2 OS=Homo sapiens GN=TCEA2 PE=1 SV=1	0.00	0.00	2.39	2.14	1.89	2.36	1.71	0.00	0.00	1.85	0.00	0.00	0.00	0.00	0.00	0.00	0.00	0.00	0.00	0.00	0.00	0.80	1.42	0.00	1.03	0.18	>0.999999999999999	5.6
Q9UHB9	Signal recognition particle 68 kDa protein OS=Homo sapiens GN=SRP68 PE=1 SV=2	0.00	0.00	2.15	4.28	1.70	2.13	1.54	0.00	0.00	0.00	2.20	1.14	0.00	0.00	0.00	0.84	0.00	0.00	0.00	0.00	0.00	0.00	0.00	0.20	1.26	0.09	>0.999999999999999	14.5
Q7L2H7	Eukaryotic translation initiation factor 3 subunit M OS=Homo sapiens GN=EIF3M PE=1 SV=1	0.00	0.00	1.91	1.71	0.00	1.89	0.00	0.00	0.00	0.00	0.00	0.65	0.00	0.00	1.91	0.00	0.00	0.00	0.00	0.00	0.00	0.00	0.00	0.00	0.51	0.16	>0.999999999999999	3.2
Q7Z2T5	TRMT1-like protein OS=Homo sapiens GN=TRMT1L PE=1 SV=2	0.00	0.00	0.96	0.86	0.00	0.00	0.00	0.00	0.00	0.74	0.00	0.00	0.00	0.00	0.00	0.00	0.00	0.00	0.00	0.00	0.00	0.00	0.00	0.00	0.21	0.00	>0.999999999999999	#DIV/0!
O43639	Cytoplasmic protein NCK2 OS=Homo sapiens GN=NCK2 PE=1 SV=2	0.00	0.00	1.91	0.00	0.00	0.00	0.00	0.00	0.00	0.00	0.00	0.00	0.00	0.00	0.00	0.00	0.00	0.00	0.00	0.00	0.00	0.00	0.00	0.00	0.16	0.00	>0.999999999999999	#DIV/0!
Q9NWB7	Intraflagellar transport protein 57 homolog OS=Homo sapiens GN=IFT57 PE=1 SV=1	0.00	0.00	1.67	0.00	1.32	3.31	0.00	0.00	0.00	0.00	1.10	0.00	0.00	0.00	0.00	0.00	0.00	0.00	0.00	0.00	0.00	0.00	0.00	0.00	0.62	0.00	>0.999999999999999	#DIV/0!
P35228	Nitric oxide synthase, inducible OS=Homo sapiens GN=NOS2 PE=1 SV=2	0.00	0.00	0.48	0.00	0.00	0.00	0.00	0.00	0.00	0.00	0.00	0.16	0.00	0.00	0.00	0.00	0.00	0.00	0.00	0.00	0.00	0.00	0.00	0.00	0.05	0.00	>0.999999999999999	#DIV/0!
Q9BRT3	Migration and invasion enhancer 1 OS=Homo sapiens GN=MIEN1 PE=1 SV=1	0.00	0.00	6.45	5.77	0.00	0.00	0.00	0.00	0.00	0.00	0.00	0.00	0.00	0.00	0.00	0.00	0.00	0.00	0.00	0.00	0.00	0.00	0.00	0.00	1.02	0.00	>0.999999999999999	#DIV/0!
P28072	Proteasome subunit beta type-6 OS=Homo sapiens GN=PSMB6 PE=1 SV=4	0.00	0.00	3.11	2.78	2.45	3.07	0.00	2.53	0.00	2.41	2.04	2.28	3.06	0.00	6.68	2.19	0.00	4.30	2.35	1.69	4.73	2.24	3.97	1.12	1.72	2.69	>0.999999999999999	0.6
Q9H0P0	Cytosolic 5'-nucleotidase 3 OS=Homo sapiens GN=NT5C3 PE=1 SV=3	0.00	0.00	2.15	3.85	0.00	0.00	1.54	0.00	0.00	0.00	2.83	0.73	0.00	0.00	2.15	0.00	0.00	0.00	0.00	0.00	0.00	0.00	0.00	0.00	0.93	0.18	>0.999999999999999	5.2
Q8N1Q1	Carbonic anhydrase 13 OS=Homo sapiens GN=CA13 PE=1 SV=1	0.00	0.00	2.63	0.00	2.07	2.60	0.00	0.00	0.00	2.04	0.00	0.90	0.00	0.00	9.06	1.85	0.00	0.00	1.99	0.00	0.00	3.04	0.00	0.00	0.85	1.33	>0.999999999999999	0.6
Q99933	BAG family molecular chaperone regulator 1 OS=Homo sapiens GN=BAG1 PE=1 SV=4	0.00	0.00	1.91	1.71	0.00	0.00	1.37	0.00	0.00	1.48	1.26	0.65	0.00	0.00	1.91	1.35	0.00	0.00	1.45	0.00	0.00	0.64	2.55	2.00	0.70	0.82	>0.999999999999999	0.8
Q9Y6E0	Serine/threonine-protein kinase 24 OS=Homo sapiens GN=STK24 PE=1 SV=1	0.00	0.00	3.35	0.00	0.00	4.96	0.00	0.00	0.00	0.00	3.30	1.14	0.00	0.00	0.00	0.00	0.00	0.00	0.00	0.00	0.00	0.00	0.00	0.84	1.06	0.07	>0.999999999999999	15.2
Q8TCX1	Cytoplasmic dynein 2 light intermediate chain 1 OS=Homo sapiens GN=DYNC2LI1 PE=2 SV=1	0.00	0.00	6.45	0.00	0.00	0.00	0.00	0.00	0.00	0.00	0.00	0.00	0.00	0.00	0.00	0.00	0.00	0.00	0.00	0.00	0.00	0.00	0.00	0.00	0.54	0.00	>0.999999999999999	#DIV/0!
O14841	5-oxoprolinase OS=Homo sapiens GN=OPLAH PE=1 SV=3	0.00	0.00	0.48	1.07	0.38	0.00	0.34	0.00	0.00	0.37	0.00	0.00	0.00	0.00	0.48	0.00	0.00	0.00	0.00	0.00	0.00	0.16	0.00	0.08	0.22	0.06	>0.999999999999999	3.7
P35611	Alpha-adducin OS=Homo sapiens GN=ADD1 PE=1 SV=2	0.00	0.00	1.91	0.86	0.00	0.00	0.00	0.00	0.00	0.00	0.63	0.00	0.00	0.00	0.00	0.00	0.00	0.00	0.00	0.00	0.00	0.00	0.57	0.16	0.28	0.06	>0.999999999999999	4.7
Q9UID3	Protein fat-free homolog OS=Homo sapiens GN=FFR PE=1 SV=2	0.00	0.00	0.96	0.00	0.00	0.00	0.00	0.00	0.00	0.00	0.00	0.00	0.00	0.00	0.00	0.00	0.00	0.00	0.00	0.00	0.00	0.00	0.00	0.00	0.08	0.00	>0.999999999999999	#DIV/0!
Q8IW45	ATP-dependent (S)-NAD(P)H-hydrate dehydratase OS=Homo sapiens GN=CARKD PE=1 SV=1	0.00	0.00	2.15	0.00	0.00	2.13	0.00	0.00	0.00	1.67	1.42	0.00	0.00	0.00	0.00	0.00	0.00	0.00	0.00	0.00	0.00	0.72	1.28	0.36	0.61	0.20	>0.999999999999999	3.1
P41214	Eukaryotic translation initiation factor 2D OS=Homo sapiens GN=EIF2D PE=1 SV=3	0.00	0.00	1.20	0.00	0.00	0.00	0.00	0.00	0.00	0.00	0.00	0.00	0.00	0.00	0.00	0.00	0.00	0.00	0.00	0.00	0.00	0.00	0.00	0.00	0.10	0.00	>0.999999999999999	#DIV/0!
O95232	Luc7-like protein 3 OS=Homo sapiens GN=LUC7L3 PE=1 SV=2	0.00	0.00	1.43	0.00	0.00	0.00	0.00	0.00	0.00	1.11	0.00	0.00	0.00	0.00	0.00	0.00	0.00	0.00	0.00	0.00	0.00	0.00	0.00	0.00	0.21	0.00	>0.999999999999999	#DIV/0!
Q8N339	Metallothionein-1M OS=Homo sapiens GN=MT1M PE=2 SV=2	0.00	0.00	11.71	10.48	9.24	0.00	8.38	4.43	6.71	9.08	7.71	4.00	11.52	0.00	0.00	0.00	6.20	7.52	0.00	0.00	8.27	0.00	0.00	1.96	5.98	2.96	>0.999999999999999	2.0
P36969	Phospholipid hydroperoxide glutathione peroxidase, mitochondrial OS=Homo sapiens GN=GPX4 PE=1 SV=3	0.00	0.00	3.59	3.21	0.00	0.00	2.56	0.00	0.00	2.78	2.36	0.00	0.00	2.41	0.00	0.00	0.00	2.30	2.71	0.00	0.00	1.20	2.13	0.00	1.21	0.90	>0.999999999999999	1.3
Q8N4P6	Leucine-rich repeat-containing protein 71 OS=Homo sapiens GN=LRRC71 PE=2 SV=1	0.00	0.00	1.20	0.00	0.00	0.00	0.00	0.00	0.00	0.93	0.00	0.00	0.00	0.00	0.00	0.00	0.00	0.00	0.00	0.00	0.00	0.00	0.00	0.00	0.18	0.00	>0.999999999999999	#DIV/0!
O95163	Elongator complex protein 1 OS=Homo sapiens GN=IKBKAP PE=1 SV=3	0.00	0.00	0.48	0.86	0.00	0.00	0.00	0.00	0.00	0.00	0.31	0.00	0.00	0.00	0.00	0.00	0.00	0.00	0.00	0.00	0.00	0.00	0.00	0.00	0.14	0.00	>0.999999999999999	#DIV/0!
A6NHG4	D-dopachrome decarboxylase-like protein OS=Homo sapiens GN=DDTL PE=2 SV=1	0.00	0.00	5.74	0.00	4.53	0.00	0.00	0.00	0.00	0.00	0.00	0.00	0.00	3.85	5.72	0.00	3.04	3.68	4.34	0.00	4.05	0.00	3.41	0.00	0.86	2.34	>0.999999999999999	0.4
Q14019	Coactosin-like protein OS=Homo sapiens GN=COTL1 PE=1 SV=3	0.00	0.00	5.02	0.00	0.00	0.00	0.00	0.00	2.88	3.89	0.00	1.71	4.94	3.37	0.00	0.00	0.00	0.00	0.00	0.00	13.00	3.75	0.00	0.00	1.12	2.09	>0.999999999999999	0.5
Q9UMS4	Pre-mRNA-processing factor 19 OS=Homo sapiens GN=PRPF19 PE=1 SV=1	0.00	0.00	2.87	0.00	1.13	2.84	0.00	0.00	0.00	1.11	0.94	0.00	0.00	0.96	0.00	1.01	0.00	0.00	0.00	0.00	0.00	0.00	0.00	0.00	0.74	0.16	>0.999999999999999	4.5
Q9P000	COMM domain-containing protein 9 OS=Homo sapiens GN=COMMD9 PE=1 SV=2	0.00	0.00	3.59	0.00	0.00	0.00	0.00	0.00	0.00	0.00	0.00	1.22	0.00	0.00	3.58	0.00	0.00	0.00	0.00	0.00	0.00	0.00	0.00	0.00	0.40	0.30	>0.999999999999999	1.3
Q9H223	EH domain-containing protein 4 OS=Homo sapiens GN=EHD4 PE=1 SV=1	0.00	0.00	1.20	0.00	0.00	0.00	0.00	0.00	0.00	0.00	0.00	0.41	0.00	0.00	0.00	0.00	0.00	0.77	0.00	0.00	0.00	0.00	0.71	0.00	0.13	0.12	>0.999999999999999	1.1
P61764	Syntaxin-binding protein 1 OS=Homo sapiens GN=STXBP1 PE=1 SV=1	0.00	0.00	2.39	0.00	0.00	2.36	0.00	0.00	0.00	0.00	0.00	0.00	0.00	0.00	0.00	0.00	0.00	0.00	0.00	0.00	0.00	0.00	0.00	0.00	0.40	0.00	>0.999999999999999	#DIV/0!
Q15506	Sperm surface protein Sp17 OS=Homo sapiens GN=SPA17 PE=1 SV=1	0.00	0.00	4.54	4.06	3.58	10.16	3.25	0.00	0.00	12.97	2.99	0.00	0.00	0.00	4.53	0.00	0.00	0.00	0.00	0.00	0.00	0.00	2.70	0.00	3.46	0.60	>0.999999999999999	5.7
Q15147	1-phosphatidylinositol-4,5-bisphosphate phosphodiesterase beta-4 OS=Homo sapiens GN=PLCB4 PE=1 SV=3	0.00	0.00	0.48	0.00	0.00	0.00	0.00	0.00	0.00	0.00	0.31	0.00	0.00	0.00	0.00	0.00	0.00	0.00	0.00	0.00	0.00	0.00	0.00	0.00	0.07	0.00	>0.999999999999999	#DIV/0!
Q5JVL4	EF-hand domain-containing protein 1 OS=Homo sapiens GN=EFHC1 PE=1 SV=1	0.00	0.00	0.96	0.86	0.00	0.00	0.00	0.00	0.00	0.74	0.00	0.00	0.00	0.00	0.00	0.00	0.00	0.00	0.00	0.00	0.00	0.00	0.00	0.00	0.21	0.00	>0.999999999999999	#DIV/0!
Q13107	Ubiquitin carboxyl-terminal hydrolase 4 OS=Homo sapiens GN=USP4 PE=1 SV=3	0.00	0.00	0.72	0.00	0.00	0.71	0.00	0.00	0.00	0.00	0.47	0.00	0.00	0.00	0.00	0.00	0.00	0.00	0.00	0.00	0.00	0.00	0.00	0.00	0.16	0.00	>0.999999999999999	#DIV/0!
Q5SNT6	WASH complex subunit FAM21B OS=Homo sapiens GN=FAM21B PE=1 SV=2	0.00	0.00	0.48	1.07	0.00	0.47	0.00	0.00	0.00	0.00	0.00	0.16	0.00	0.00	0.00	0.00	0.00	0.00	0.00	0.00	0.00	0.00	0.00	0.00	0.18	0.00	>0.999999999999999	#DIV/0!
P53384	Cytosolic Fe-S cluster assembly factor NUBP1 OS=Homo sapiens GN=NUBP1 PE=1 SV=2	0.00	0.00	2.15	1.92	1.70	0.00	0.00	0.00	0.00	0.00	0.00	0.73	0.00	0.00	0.00	0.00	0.00	0.00	0.00	0.00	0.00	0.72	0.00	0.00	0.54	0.06	>0.999999999999999	9.1
P55011	Solute carrier family 12 member 2 OS=Homo sapiens GN=SLC12A2 PE=1 SV=1	0.00	0.00	1.20	0.00	0.00	0.00	0.00	0.00	0.00	0.37	0.31	0.00	0.00	0.00	0.48	0.00	0.00	0.00	0.00	0.00	0.00	0.00	0.00	0.00	0.16	0.04	>0.999999999999999	3.9
Q12798	Centrin-1 OS=Homo sapiens GN=CETN1 PE=1 SV=1	0.00	0.00	4.06	0.00	0.00	4.02	2.91	0.00	0.00	0.00	0.00	0.00	0.00	0.00	0.00	0.00	0.00	0.00	0.00	0.00	0.00	1.36	0.00	0.00	0.92	0.11	>0.999999999999999	8.1
P50914	60S ribosomal protein L14 OS=Homo sapiens GN=RPL14 PE=1 SV=4	0.00	0.00	3.35	2.99	9.24	7.09	11.97	1.26	0.00	0.00	11.01	1.14	3.29	2.25	7.15	0.00	1.77	0.00	5.43	0.00	2.36	0.00	4.26	0.00	4.00	2.21	>0.999999999999999	1.8
Q6Y7W6	PERQ amino acid-rich with GYF domain-containing protein 2 OS=Homo sapiens GN=GIGYF2 PE=1 SV=1	0.00	0.00	0.48	0.00	0.00	0.00	0.00	0.00	0.00	0.00	0.00	0.00	0.00	0.00	0.00	0.00	0.00	0.00	0.00	0.00	0.00	0.00	0.00	0.00	0.04	0.00	>0.999999999999999	#DIV/0!
P09661	U2 small nuclear ribonucleoprotein A' OS=Homo sapiens GN=SNRPA1 PE=1 SV=2	0.00	0.00	5.98	2.57	2.26	5.91	0.00	0.00	0.00	4.63	1.89	0.00	0.00	0.00	0.00	0.00	0.00	0.00	0.00	0.00	0.00	0.00	0.00	0.00	1.94	0.00	>0.999999999999999	#DIV/0!
Q96GY0	Protein FAM164A OS=Homo sapiens GN=FAM164A PE=1 SV=2	0.00	0.00	4.54	0.00	0.00	4.49	0.00	0.00	0.00	1.67	2.99	0.00	0.00	0.00	0.00	0.00	0.00	0.00	0.00	0.00	0.00	0.00	0.00	0.00	1.14	0.00	>0.999999999999999	#DIV/0!
P49915	GMP synthase [glutamine-hydrolyzing] OS=Homo sapiens GN=GMPS PE=1 SV=1	0.00	0.00	2.15	0.00	0.00	0.00	0.00	0.00	0.00	0.00	0.63	0.00	0.00	0.64	0.00	0.00	0.00	0.00	0.00	0.00	0.00	0.32	0.00	0.00	0.23	0.08	>0.999999999999999	2.9
P61224	Ras-related protein Rap-1b OS=Homo sapiens GN=RAP1B PE=1 SV=1	0.00	0.00	8.13	0.00	0.00	0.00	0.00	1.44	0.00	0.00	2.52	4.57	0.00	0.00	0.00	0.00	0.00	0.00	6.15	0.00	0.00	2.72	2.27	0.00	1.39	0.93	>0.999999999999999	1.5
Q8IY31	Intraflagellar transport protein 20 homolog OS=Homo sapiens GN=IFT20 PE=1 SV=1	0.00	0.00	5.26	0.00	0.00	0.00	0.00	0.00	0.00	4.08	3.46	0.00	0.00	0.00	0.00	0.00	0.00	0.00	0.00	0.00	0.00	0.00	0.00	0.00	1.07	0.00	>0.999999999999999	#DIV/0!
Q8IUC4	Rhophilin-2 OS=Homo sapiens GN=RHPN2 PE=1 SV=1	0.00	0.00	2.15	0.00	0.00	0.00	0.00	0.00	0.00	0.00	0.00	0.00	0.00	0.00	0.00	0.00	0.00	0.00	0.00	0.00	0.00	0.00	0.00	0.00	0.18	0.00	>0.999999999999999	#DIV/0!
P11441	Ubiquitin-like protein 4A OS=Homo sapiens GN=UBL4A PE=1 SV=1	0.00	0.00	9.80	0.00	0.00	0.00	0.00	0.00	0.00	0.00	0.00	0.00	0.00	0.00	0.00	0.00	2.40	0.00	0.00	0.00	0.00	0.00	0.00	0.00	0.82	0.20	>0.999999999999999	4.1
Q9P289	Serine/threonine-protein kinase MST4 OS=Homo sapiens GN=MST4 PE=1 SV=2	0.00	0.00	1.67	1.50	1.32	5.43	3.93	0.00	0.00	0.00	2.36	0.00	0.00	1.12	1.67	0.00	0.00	1.07	2.71	0.00	0.00	0.00	0.00	0.00	1.35	0.55	>0.999999999999999	2.5
Q5JSH3	WD repeat-containing protein 44 OS=Homo sapiens GN=WDR44 PE=1 SV=1	0.00	0.00	1.67	0.00	0.57	0.71	0.00	0.00	0.00	1.30	1.10	0.00	0.00	0.00	0.00	0.00	0.00	0.00	0.00	0.00	0.00	0.00	0.00	0.00	0.45	0.00	>0.999999999999999	#DIV/0!
Q7L5D6	Golgi to ER traffic protein 4 homolog OS=Homo sapiens GN=GET4 PE=1 SV=1	0.00	0.00	2.15	0.00	0.00	0.00	0.00	0.00	0.00	0.00	0.00	0.00	0.00	0.00	2.15	0.00	0.00	0.00	0.00	0.00	0.00	0.00	0.00	0.00	0.18	0.18	>0.999999999999999	1.0
P55010	Eukaryotic translation initiation factor 5 OS=Homo sapiens GN=EIF5 PE=1 SV=2	0.00	0.00	3.35	1.50	2.64	1.65	1.20	0.00	0.00	0.00	1.10	0.00	0.00	1.12	1.67	0.00	0.89	1.07	2.53	0.00	0.00	0.56	0.99	0.00	0.95	0.74	>0.999999999999999	1.3
Q9UI10	Translation initiation factor eIF-2B subunit delta OS=Homo sapiens GN=EIF2B4 PE=1 SV=2	0.00	0.00	1.43	0.00	0.00	0.00	0.00	0.00	0.00	0.00	0.00	0.00	0.00	0.00	0.00	0.00	0.00	0.00	0.00	0.00	0.00	0.00	0.00	0.00	0.12	0.00	>0.999999999999999	#DIV/0!
Q9Y263	Phospholipase A-2-activating protein OS=Homo sapiens GN=PLAA PE=1 SV=2	0.00	0.00	2.63	0.86	0.00	0.95	0.00	0.00	0.00	0.00	0.00	0.33	0.00	0.00	0.00	0.00	0.00	0.00	0.00	0.00	0.00	0.00	0.00	0.16	0.40	0.01	>0.999999999999999	29.7
O43290	U4/U6.U5 tri-snRNP-associated protein 1 OS=Homo sapiens GN=SART1 PE=1 SV=1	0.00	0.00	0.96	0.00	0.00	0.00	0.00	0.00	0.00	0.00	0.00	0.00	0.00	0.00	0.00	0.00	0.00	0.00	0.00	0.00	0.00	0.00	0.00	0.00	0.08	0.00	>0.999999999999999	#DIV/0!
O43447	Peptidyl-prolyl cis-trans isomerase H OS=Homo sapiens GN=PPIH PE=1 SV=1	0.00	0.00	4.06	0.00	0.00	0.00	0.00	0.00	0.00	0.00	0.00	0.00	0.00	0.00	0.00	0.00	0.00	0.00	0.00	0.00	0.00	0.00	2.41	0.00	0.34	0.20	>0.999999999999999	1.7
Q9Y5S2	Serine/threonine-protein kinase MRCK beta OS=Homo sapiens GN=CDC42BPB PE=1 SV=2	0.00	0.00	0.48	0.00	0.00	0.00	0.00	0.00	0.00	0.00	0.00	0.00	0.00	0.00	0.00	0.00	0.00	0.00	0.00	0.00	0.00	0.00	0.00	0.00	0.04	0.00	>0.999999999999999	#DIV/0!
Q96K76	Ubiquitin carboxyl-terminal hydrolase 47 OS=Homo sapiens GN=USP47 PE=1 SV=3	0.00	0.00	0.48	0.00	0.38	0.00	0.00	0.00	0.00	0.00	0.63	0.00	0.00	0.00	0.00	0.00	0.00	0.00	0.00	0.00	0.00	0.00	0.28	0.00	0.12	0.02	>0.999999999999999	5.2
Q9HBG6	Intraflagellar transport protein 122 homolog OS=Homo sapiens GN=IFT122 PE=1 SV=2	0.00	0.00	1.20	0.00	0.00	0.47	0.00	0.00	0.00	0.00	0.00	0.00	0.00	0.00	0.00	0.00	0.00	0.00	0.00	0.00	0.00	0.00	0.00	0.00	0.14	0.00	>0.999999999999999	#DIV/0!
Q05397	Focal adhesion kinase 1 OS=Homo sapiens GN=PTK2 PE=1 SV=2	0.00	0.00	1.43	0.64	0.00	0.00	0.00	0.00	0.00	0.56	0.47	0.00	0.00	0.00	0.00	0.00	0.00	0.00	0.00	0.00	0.00	0.24	0.00	0.00	0.26	0.02	>0.999999999999999	12.9
Q9P2H3	Intraflagellar transport protein 80 homolog OS=Homo sapiens GN=IFT80 PE=1 SV=3	0.00	0.00	0.96	0.00	0.00	3.78	0.00	0.00	0.00	1.48	0.63	0.00	0.00	0.00	0.95	0.00	0.00	0.00	0.00	0.00	0.00	0.00	0.00	0.00	0.57	0.08	>0.999999999999999	7.2
O15116	U6 snRNA-associated Sm-like protein LSm1 OS=Homo sapiens GN=LSM1 PE=1 SV=1	0.00	0.00	5.26	0.00	0.00	5.20	0.00	0.00	0.00	4.08	3.46	0.00	0.00	0.00	5.25	0.00	0.00	0.00	0.00	0.00	0.00	0.00	0.00	0.00	1.50	0.44	>0.999999999999999	3.4
Q15075	Early endosome antigen 1 OS=Homo sapiens GN=EEA1 PE=1 SV=2	0.00	0.00	0.48	0.43	0.00	0.47	0.00	0.00	0.00	0.37	0.31	0.16	0.00	0.00	0.00	0.00	0.00	0.00	0.00	0.00	0.00	0.00	0.00	0.00	0.19	0.00	>0.999999999999999	#DIV/0!
P31513	Dimethylaniline monooxygenase [N-oxide-forming] 3 OS=Homo sapiens GN=FMO3 PE=1 SV=5	0.00	0.00	1.20	0.00	0.00	0.00	0.00	0.00	0.00	0.00	0.00	0.00	0.00	0.00	0.00	0.00	0.00	0.00	0.00	0.00	0.00	0.00	0.00	0.00	0.10	0.00	>0.999999999999999	#DIV/0!
P30876	DNA-directed RNA polymerase II subunit RPB2 OS=Homo sapiens GN=POLR2B PE=1 SV=1	0.00	0.00	0.48	0.00	0.00	0.00	0.00	0.00	0.00	0.00	0.00	0.00	0.00	0.00	0.00	0.00	0.00	0.00	0.00	0.00	0.00	0.00	0.00	0.00	0.04	0.00	>0.999999999999999	#DIV/0!
O75688	Protein phosphatase 1B OS=Homo sapiens GN=PPM1B PE=1 SV=1	0.00	0.00	1.43	1.28	0.00	0.00	0.00	0.00	0.00	1.11	2.04	0.00	0.00	0.00	1.43	0.00	0.00	1.09	0.00	0.00	0.00	0.00	0.85	0.24	0.49	0.30	>0.999999999999999	1.6
O43143	Putative pre-mRNA-splicing factor ATP-dependent RNA helicase DHX15 OS=Homo sapiens GN=DHX15 PE=1 SV=	0.00	0.00	0.96	0.00	0.00	0.00	0.00	0.00	0.00	0.00	1.10	0.00	0.94	0.00	0.00	0.00	0.00	0.00	0.00	0.00	0.00	0.32	0.57	0.00	0.17	0.15	>0.999999999999999	1.1
Q8N3F0	UPF0452 protein C7orf41 OS=Homo sapiens GN=C7orf41 PE=2 SV=2	0.00	0.00	5.26	0.00	0.00	0.00	0.00	0.00	0.00	0.00	0.00	0.00	0.00	0.00	0.00	0.00	0.00	0.00	0.00	0.00	0.00	0.00	0.00	0.00	0.44	0.00	>0.999999999999999	#DIV/0!
Q5TH69	Brefeldin A-inhibited guanine nucleotide-exchange protein 3 OS=Homo sapiens GN=ARFGEF3 PE=1 SV=3	0.00	0.00	0.24	0.00	0.00	0.00	0.00	0.00	0.00	0.00	0.00	0.00	0.00	0.00	0.00	0.00	0.00	0.00	0.00	0.00	0.00	0.00	0.00	0.00	0.02	0.00	>0.999999999999999	#DIV/0!
Q96PD5	N-acetylmuramoyl-L-alanine amidase OS=Homo sapiens GN=PGLYRP2 PE=1 SV=1	0.00	0.00	1.20	2.35	2.07	1.18	0.00	0.45	0.00	0.00	0.00	1.39	0.00	0.80	1.19	2.86	0.63	0.00	0.00	0.65	0.00	0.40	0.00	0.44	0.72	0.58	>0.999999999999999	1.2
Q9H0Q0	Protein FAM49A OS=Homo sapiens GN=FAM49A PE=1 SV=1	0.00	0.00	2.15	0.00	0.00	0.00	0.00	0.00	0.00	0.00	0.00	0.00	0.00	0.00	0.00	0.00	0.00	0.00	0.00	0.00	0.00	0.00	0.00	0.00	0.18	0.00	>0.999999999999999	#DIV/0!
Q13188	Serine/threonine-protein kinase 3 OS=Homo sapiens GN=STK3 PE=1 SV=2	0.00	0.00	1.43	0.00	0.00	0.00	0.00	0.00	0.00	0.00	0.00	0.00	0.00	0.00	0.00	0.00	0.00	0.00	0.00	0.00	0.00	0.00	0.85	0.00	0.12	0.07	>0.999999999999999	1.7
Q6PGP7	Tetratricopeptide repeat protein 37 OS=Homo sapiens GN=TTC37 PE=1 SV=1	0.00	0.00	0.48	0.86	0.38	0.47	0.00	0.00	0.00	0.00	0.31	0.00	0.00	0.00	0.00	0.00	0.00	0.00	0.00	0.00	0.00	0.00	0.00	0.00	0.21	0.00	>0.999999999999999	#DIV/0!
Q9Y5B9	FACT complex subunit SPT16 OS=Homo sapiens GN=SUPT16H PE=1 SV=1	0.00	0.00	0.72	0.64	0.00	0.71	0.00	0.00	0.00	0.00	0.00	0.00	0.00	0.00	0.00	0.00	0.00	0.00	0.00	0.00	0.00	0.00	0.00	0.00	0.17	0.00	>0.999999999999999	#DIV/0!
Q9UHB6	LIM domain and actin-binding protein 1 OS=Homo sapiens GN=LIMA1 PE=1 SV=1	0.00	0.00	1.91	0.00	0.75	0.95	0.00	0.00	0.00	0.00	1.89	0.00	0.00	0.00	1.91	0.67	1.01	0.00	0.00	0.00	0.00	0.00	1.14	0.00	0.46	0.39	>0.999999999999999	1.2
Q96M32	Adenylate kinase 7 OS=Homo sapiens GN=AK7 PE=1 SV=3	0.00	0.00	0.96	0.00	0.00	0.00	0.00	0.00	0.00	1.48	0.00	0.00	0.00	0.00	0.95	0.00	0.00	0.00	0.00	0.00	0.00	0.00	0.00	0.00	0.20	0.08	>0.999999999999999	2.6
Q07157	Tight junction protein ZO-1 OS=Homo sapiens GN=TJP1 PE=1 SV=3	0.00	0.00	0.48	0.00	0.00	0.00	0.00	0.00	0.00	0.00	0.00	0.00	0.00	0.00	0.00	0.00	0.00	0.00	0.00	0.00	0.00	0.00	0.00	0.00	0.04	0.00	>0.999999999999999	#DIV/0!
Q7L014	Probable ATP-dependent RNA helicase DDX46 OS=Homo sapiens GN=DDX46 PE=1 SV=2	0.00	0.00	0.72	0.00	0.00	0.00	0.00	0.00	0.00	0.00	0.47	0.00	0.00	0.00	0.72	0.00	0.00	0.00	0.00	0.00	0.00	0.00	0.00	0.00	0.10	0.06	>0.999999999999999	1.7
Q5EBL8	PDZ domain-containing protein 11 OS=Homo sapiens GN=PDZD11 PE=1 SV=2	0.00	0.00	5.02	0.00	0.00	4.96	0.00	0.00	0.00	3.89	0.00	0.00	0.00	0.00	0.00	0.00	0.00	0.00	0.00	0.00	0.00	0.00	0.00	0.00	1.16	0.00	>0.999999999999999	#DIV/0!
Q70E73	Ras-associated and pleckstrin homology domains-containing protein 1 OS=Homo sapiens GN=RAPH1 PE=1 SV=3	0.00	0.00	0.48	0.00	0.00	0.00	0.00	0.00	0.00	0.00	0.00	0.16	0.00	0.00	0.00	0.00	0.00	0.00	0.00	0.00	0.00	0.00	0.00	0.00	0.05	0.00	>0.999999999999999	#DIV/0!
Q8N3I7	Bardet-Biedl syndrome 5 protein OS=Homo sapiens GN=BBS5 PE=1 SV=1	0.00	0.00	2.15	0.00	0.00	0.00	0.00	0.00	0.00	0.00	0.00	0.00	0.00	0.00	0.00	0.00	0.00	0.00	0.00	0.00	0.00	0.00	0.00	0.00	0.18	0.00	>0.999999999999999	#DIV/0!
Q13247	Serine/arginine-rich splicing factor 6 OS=Homo sapiens GN=SRSF6 PE=1 SV=2	0.00	0.00	4.06	0.00	1.51	4.02	0.00	0.00	0.00	0.00	0.00	0.00	0.00	0.00	0.00	0.00	0.00	0.00	0.00	0.00	0.00	0.00	0.00	0.00	0.80	0.00	>0.999999999999999	#DIV/0!
Q53H82	Beta-lactamase-like protein 2 OS=Homo sapiens GN=LACTB2 PE=1 SV=2	0.00	0.00	5.02	2.14	1.89	0.00	0.00	0.00	0.00	1.85	0.00	0.00	0.00	0.00	2.38	0.00	0.00	0.00	0.00	0.00	0.00	0.00	1.42	0.00	0.91	0.32	>0.999999999999999	2.9
Q9BSL1	Ubiquitin-associated domain-containing protein 1 OS=Homo sapiens GN=UBAC1 PE=1 SV=1	0.00	0.00	1.67	1.50	0.00	0.00	0.00	0.00	0.00	0.00	0.00	0.00	0.00	0.00	0.00	0.00	0.00	0.00	0.00	0.00	0.00	0.00	0.00	0.00	0.26	0.00	>0.999999999999999	#DIV/0!
Q9UBK7	Rab-like protein 2A OS=Homo sapiens GN=RABL2A PE=2 SV=1	0.00	0.00	6.45	0.00	0.00	0.00	0.00	0.00	0.00	0.00	0.00	0.00	0.00	2.09	0.00	0.00	0.00	0.00	0.00	0.00	0.00	0.00	0.00	0.00	0.54	0.17	>0.999999999999999	3.1
O43324	Eukaryotic translation elongation factor 1 epsilon-1 OS=Homo sapiens GN=EEF1E1 PE=1 SV=1	0.00	0.00	8.84	0.00	0.00	0.00	0.00	0.00	0.00	0.00	0.00	1.39	0.00	0.00	0.00	0.00	0.00	0.00	0.00	0.00	0.00	0.00	0.00	0.00	0.85	0.00	>0.999999999999999	#DIV/0!
Q6FI81	Anamorsin OS=Homo sapiens GN=CIAPIN1 PE=1 SV=2	0.00	0.00	2.39	2.14	0.00	2.36	0.00	0.00	0.00	0.00	0.00	0.82	0.00	1.61	0.00	0.00	1.27	0.00	1.81	0.00	0.00	2.56	1.42	0.00	0.64	0.72	>0.999999999999999	0.9
Q8WVF1	Protein OSCP1 OS=Homo sapiens GN=OSCP1 PE=1 SV=4	0.00	0.00	1.67	0.00	1.32	1.65	1.20	0.00	0.00	1.30	1.10	0.00	0.00	0.00	0.00	0.00	0.00	0.00	0.00	0.00	0.00	0.00	0.00	0.00	0.69	0.00	>0.999999999999999	#DIV/0!
Q9Y3D6	Mitochondrial fission 1 protein OS=Homo sapiens GN=FIS1 PE=1 SV=2	0.00	0.00	4.78	4.28	0.00	0.00	3.42	0.00	0.00	3.71	0.00	0.00	0.00	0.00	0.00	0.00	0.00	0.00	0.00	0.00	0.00	0.00	2.84	0.00	1.35	0.24	>0.999999999999999	5.7
P25686	DnaJ homolog subfamily B member 2 OS=Homo sapiens GN=DNAJB2 PE=1 SV=3	0.00	0.00	2.15	1.92	0.00	0.00	0.00	0.00	0.00	0.00	0.00	0.00	0.00	0.00	0.00	0.00	0.00	0.00	0.00	0.00	0.00	0.00	0.00	0.00	0.34	0.00	>0.999999999999999	#DIV/0!
P62753	40S ribosomal protein S6 OS=Homo sapiens GN=RPS6 PE=1 SV=1	0.00	0.00	2.87	0.00	0.00	2.84	2.05	0.00	0.00	0.00	3.77	0.00	5.64	1.93	2.86	2.02	0.00	0.00	0.00	0.00	0.00	0.00	0.00	0.00	0.96	1.04	>0.999999999999999	0.9
P05090	Apolipoprotein D OS=Homo sapiens GN=APOD PE=1 SV=1	0.00	0.00	8.13	7.27	3.02	0.00	0.00	3.07	0.00	2.96	0.00	2.77	0.00	0.00	3.82	5.72	0.00	2.45	2.89	4.41	2.70	2.72	0.00	1.36	2.27	2.17	>0.999999999999999	1.0
O75643	U5 small nuclear ribonucleoprotein 200 kDa helicase OS=Homo sapiens GN=SNRNP200 PE=1 SV=2	0.00	0.00	0.72	0.00	0.19	0.71	0.00	0.00	0.00	0.00	0.00	0.00	0.00	0.00	0.00	0.00	0.00	0.00	0.00	0.00	0.00	0.00	0.00	0.00	0.13	0.00	>0.999999999999999	#DIV/0!
Q8N584	Tetratricopeptide repeat protein 39C OS=Homo sapiens GN=TTC39C PE=1 SV=2	0.00	0.00	1.20	0.00	0.00	0.00	0.00	0.00	0.00	0.00	0.00	0.00	0.00	0.00	0.00	0.00	0.00	0.00	0.00	0.00	0.00	0.00	0.00	0.00	0.10	0.00	>0.999999999999999	#DIV/0!
O00505	Importin subunit alpha-3 OS=Homo sapiens GN=KPNA3 PE=1 SV=2	0.00	0.00	1.43	1.28	0.00	1.42	2.05	0.00	0.00	0.00	0.00	0.00	0.00	0.96	0.00	0.00	0.00	0.00	0.00	0.00	0.00	0.00	1.70	0.00	0.52	0.22	>0.999999999999999	2.3
Q9H3U1	Protein unc-45 homolog A OS=Homo sapiens GN=UNC45A PE=1 SV=1	0.00	0.00	0.72	0.64	0.00	0.00	0.00	0.00	0.00	0.00	0.00	0.24	0.00	0.00	0.00	0.00	0.00	0.00	0.00	0.00	0.00	0.00	0.00	0.12	0.13	0.01	>0.999999999999999	13.4
Q5H9R7	Serine/threonine-protein phosphatase 6 regulatory subunit 3 OS=Homo sapiens GN=PPP6R3 PE=1 SV=2	0.00	0.00	0.72	0.00	0.00	0.71	0.51	0.00	0.00	0.00	0.47	0.00	0.00	0.00	0.00	0.00	0.00	0.00	0.00	0.00	0.00	0.00	0.00	0.00	0.20	0.00	>0.999999999999999	#DIV/0!
Q0VDF9	Heat shock 70 kDa protein 14 OS=Homo sapiens GN=HSPA14 PE=1 SV=1	0.00	0.00	1.43	0.00	0.00	0.00	1.03	0.00	0.00	0.00	0.94	0.49	0.00	0.00	0.00	0.00	0.00	0.00	0.00	0.00	0.00	0.00	0.85	0.00	0.32	0.07	>0.999999999999999	4.6
Q15477	Helicase SKI2W OS=Homo sapiens GN=SKIV2L PE=1 SV=3	0.00	0.00	0.48	0.00	0.00	0.00	0.00	0.00	0.00	0.00	0.00	0.16	0.00	0.00	0.00	0.00	0.00	0.00	0.00	0.00	0.00	0.00	0.00	0.00	0.05	0.00	>0.999999999999999	#DIV/0!
Q9UJC3	Protein Hook homolog 1 OS=Homo sapiens GN=HOOK1 PE=1 SV=2	0.00	0.00	1.91	0.00	0.00	0.00	0.00	0.00	0.00	0.00	0.63	0.00	0.00	0.00	0.00	0.00	0.00	0.00	0.00	0.00	0.00	0.00	0.00	0.16	0.21	0.01	>0.999999999999999	15.9
Q9UNN5	FAS-associated factor 1 OS=Homo sapiens GN=FAF1 PE=1 SV=2	0.00	0.00	0.96	0.86	0.00	0.95	0.00	0.00	0.00	0.74	0.00	0.00	0.00	0.00	0.00	0.00	0.00	0.00	0.00	0.00	0.00	0.00	0.00	0.00	0.29	0.00	>0.999999999999999	#DIV/0!
Q9NR50	Translation initiation factor eIF-2B subunit gamma OS=Homo sapiens GN=EIF2B3 PE=1 SV=1	0.00	0.00	1.43	0.00	0.00	0.00	0.00	0.00	0.00	0.00	0.94	0.49	0.00	0.00	0.00	0.00	0.00	0.00	0.00	0.00	0.00	0.00	0.00	0.00	0.24	0.00	>0.999999999999999	#DIV/0!
Q15599	Na(+)/H(+) exchange regulatory cofactor NHE-RF2 OS=Homo sapiens GN=SLC9A3R2 PE=1 SV=2	0.00	0.00	2.15	0.00	0.00	0.00	0.00	0.00	0.00	0.00	0.00	0.00	0.00	0.00	0.00	0.00	0.00	0.00	0.00	0.00	0.00	0.00	0.00	0.00	0.18	0.00	>0.999999999999999	#DIV/0!
Q86XP3	ATP-dependent RNA helicase DDX42 OS=Homo sapiens GN=DDX42 PE=1 SV=1	0.00	0.00	0.72	0.00	0.00	2.36	0.00	0.00	0.00	0.56	0.00	0.00	0.00	0.00	0.00	0.00	0.00	0.00	0.00	0.00	0.00	0.00	0.00	0.00	0.30	0.00	>0.999999999999999	#DIV/0!
Q9UIA9	Exportin-7 OS=Homo sapiens GN=XPO7 PE=1 SV=3	0.00	0.00	1.20	1.71	0.57	1.89	0.00	0.00	0.00	0.00	0.79	0.24	0.00	0.00	1.19	0.00	0.00	0.00	0.00	0.00	0.00	0.24	0.00	0.00	0.53	0.12	>0.999999999999999	4.5
Q9Y6G5	COMM domain-containing protein 10 OS=Homo sapiens GN=COMMD10 PE=1 SV=1	0.00	0.00	3.59	0.00	0.00	0.00	0.00	0.00	0.00	0.00	0.00	0.00	0.00	2.41	0.00	0.00	0.00	0.00	0.00	0.00	0.00	0.00	2.13	0.00	0.30	0.38	>0.999999999999999	0.8
Q5GLZ8	Probable E3 ubiquitin-protein ligase HERC4 OS=Homo sapiens GN=HERC4 PE=1 SV=1	0.00	0.00	1.43	0.64	0.00	0.00	0.00	0.00	0.00	0.00	0.47	0.00	0.00	0.00	0.00	0.00	0.00	0.00	0.00	0.00	0.00	0.48	0.43	0.00	0.21	0.08	>0.999999999999999	2.8
P35244	Replication protein A 14 kDa subunit OS=Homo sapiens GN=RPA3 PE=1 SV=1	0.00	0.00	5.98	0.00	4.71	5.91	4.27	2.26	0.00	4.63	0.00	0.00	0.00	0.00	0.00	0.00	0.00	0.00	4.52	0.00	0.00	2.00	0.00	1.00	2.31	0.63	>0.999999999999999	3.7

Q14790	Caspase-8 OS=Homo sapiens GN=CASP8 PE=1 SV=1	0.00	0.00	1.43	0.00	0.00	0.00	0.00	0.00	0.00	0.00	0.00	0.00	0.00	0.00	0.00	0.00	0.00	0.00	0.00	0.00	0.00	0.00	0.00	0.00	0.12	0.00	>0.999999999999999	#DIV/0!
P63173	60S ribosomal protein L38 OS=Homo sapiens GN=RPL38 PE=1 SV=2	0.00	0.00	10.04	8.98	7.92	9.92	0.00	0.00	0.00	0.00	0.00	3.43	9.88	0.00	0.00	0.00	5.31	0.00	0.00	0.00	0.00	0.00	5.96	1.68	3.36	1.90	>0.999999999999999	1.8
O15131	Importin subunit alpha-6 OS=Homo sapiens GN=KPNA5 PE=1 SV=2	0.00	0.00	1.20	1.07	0.00	1.18	0.00	0.00	0.00	0.00	0.00	0.41	0.00	0.00	0.00	0.00	0.00	0.00	0.00	0.00	0.00	0.00	0.00	0.20	0.32	0.02	>0.999999999999999	19.3
O60716	Catenin delta-1 OS=Homo sapiens GN=CTNND1 PE=1 SV=1	0.00	0.00	0.72	0.64	0.00	0.71	0.00	0.00	0.00	0.00	0.00	0.00	0.00	0.00	0.00	0.50	0.00	0.00	0.00	0.00	0.00	0.00	0.00	0.00	0.17	0.04	>0.999999999999999	4.1
Q7Z2Z2	Elongation factor Tu GTP-binding domain-containing protein 1 OS=Homo sapiens GN=EFTUD1 PE=1 SV=2	0.00	0.00	0.72	1.07	0.00	0.71	0.00	0.00	0.00	0.00	0.47	0.00	0.00	0.00	0.72	0.00	0.00	0.00	0.00	0.00	0.00	0.00	0.00	0.00	0.25	0.06	>0.999999999999999	4.1
Q9UEY8	Gamma-adducin OS=Homo sapiens GN=ADD3 PE=1 SV=1	0.00	0.00	0.96	0.86	0.00	0.95	0.00	0.00	0.00	0.00	0.00	0.00	0.00	0.00	0.00	0.00	0.00	0.00	0.00	0.00	0.00	0.00	0.00	0.00	0.23	0.00	>0.999999999999999	#DIV/0!
P07738	Bisphosphoglycerate mutase OS=Homo sapiens GN=BPGM PE=1 SV=2	0.00	0.00	2.63	0.00	0.00	0.00	0.00	0.00	0.00	0.00	0.00	0.00	0.00	0.00	0.00	0.00	0.00	0.00	0.00	0.00	0.00	0.00	0.00	0.00	0.22	0.00	>0.999999999999999	#DIV/0!
P31749	RAC-alpha serine/threonine-protein kinase OS=Homo sapiens GN=AKT1 PE=1 SV=2	0.00	0.00	1.43	1.28	0.00	0.00	0.00	0.00	0.00	0.00	0.00	0.00	0.00	0.00	1.43	0.00	0.00	0.00	0.00	0.00	0.00	0.00	0.00	0.24	0.23	0.14	>0.999999999999999	1.6
Q9Y315	Putative deoxyribose-phosphate aldolase OS=Homo sapiens GN=DERA PE=1 SV=2	0.00	0.00	2.15	4.28	1.70	4.73	1.54	0.00	1.23	1.67	0.00	1.63	0.00	1.45	2.15	0.00	1.14	0.00	1.63	0.00	0.00	1.60	2.84	0.00	1.58	0.90	>0.999999999999999	1.8
Q9H477	Ribokinase OS=Homo sapiens GN=RBKS PE=1 SV=1	0.00	0.00	2.39	0.00	0.00	4.73	0.00	0.00	0.00	0.00	3.15	0.00	0.00	1.61	0.00	0.00	0.00	0.00	0.00	0.00	0.00	0.00	0.00	0.00	0.86	0.13	>0.999999999999999	6.4
Q9BT73	Proteasome assembly chaperone 3 OS=Homo sapiens GN=PSMG3 PE=1 SV=1	0.00	0.00	6.22	5.56	0.00	0.00	4.45	0.00	0.00	0.00	4.09	0.00	0.00	0.00	0.00	0.00	0.00	0.00	0.00	0.00	0.00	0.00	0.00	0.00	1.69	0.00	>0.999999999999999	#DIV/0!
O15066	Kinesin-like protein KIF3B OS=Homo sapiens GN=KIF3B PE=1 SV=1	0.00	0.00	0.96	0.00	0.00	0.00	0.00	0.00	0.00	0.00	0.00	0.00	0.00	0.00	0.95	0.00	0.00	0.00	0.00	0.00	0.00	0.00	0.00	0.00	0.08	0.08	>0.999999999999999	1.0
Q96MX6	WD repeat-containing protein 92 OS=Homo sapiens GN=WDR92 PE=1 SV=1	0.00	0.00	1.91	1.71	0.00	0.00	0.00	0.00	0.00	0.00	0.00	0.00	0.00	0.00	0.00	0.00	0.00	0.00	0.00	0.00	0.00	0.00	0.00	0.00	0.30	0.00	>0.999999999999999	#DIV/0!
Q96DG6	Carboxymethylenebutenolidase homolog OS=Homo sapiens GN=CMBL PE=1 SV=1	0.00	0.00	5.98	5.34	0.00	5.91	0.00	0.00	0.00	2.22	8.81	0.00	0.00	0.00	5.96	0.00	0.00	0.00	0.00	2.17	0.00	0.00	1.70	0.00	2.35	0.82	>0.999999999999999	2.9
Q9P0V9	Septin-10 OS=Homo sapiens GN=SEPT10 PE=1 SV=2	0.00	0.00	1.43	1.28	0.00	3.07	0.00	0.00	0.00	0.00	0.94	0.00	0.00	0.00	0.00	0.00	0.00	0.00	0.00	0.00	0.00	0.00	0.00	0.00	0.56	0.00	>0.999999999999999	#DIV/0!
Q03393	6-pyruvoyl tetrahydrobiopterin synthase OS=Homo sapiens GN=PTS PE=1 SV=1	0.00	0.00	4.78	4.28	0.00	4.73	0.00	0.00	0.00	0.00	3.15	0.00	0.00	0.00	4.77	0.00	0.00	0.00	0.00	0.00	0.00	0.00	2.84	0.00	1.41	0.63	>0.999999999999999	2.2
Q8NEZ3	WD repeat-containing protein 19 OS=Homo sapiens GN=WDR19 PE=2 SV=2	0.00	0.00	0.48	0.00	0.00	0.00	0.00	0.00	0.00	0.00	0.00	0.00	0.00	0.00	0.00	0.00	0.00	0.00	0.00	0.00	0.00	0.00	0.00	0.00	0.04	0.00	>0.999999999999999	#DIV/0!
O15160	DNA-directed RNA polymerases I and III subunit RPAC1 OS=Homo sapiens GN=POLR1C PE=1 SV=1	0.00	0.00	1.91	0.00	0.00	0.00	0.00	0.00	0.00	0.00	1.26	0.00	0.00	0.00	4.05	0.00	0.00	1.23	0.00	0.00	0.00	0.00	1.14	0.00	0.26	0.53	>0.999999999999999	0.5
O14645	Axonemal dynein light intermediate polypeptide 1 OS=Homo sapiens GN=DNALI1 PE=2 SV=2	0.00	0.00	2.63	2.35	0.00	2.60	0.00	0.00	0.00	2.04	1.73	0.00	0.00	0.00	2.62	0.00	0.00	0.00	0.00	0.00	0.00	0.88	0.00	0.00	0.95	0.29	>0.999999999999999	3.2
Q96QR8	Transcriptional activator protein Pur-beta OS=Homo sapiens GN=PURB PE=1 SV=3	0.00	0.00	2.39	4.49	0.00	2.36	0.00	0.00	0.00	1.85	1.57	0.82	0.00	0.00	2.38	0.00	0.00	0.00	0.00	0.00	0.00	0.00	0.00	0.40	1.12	0.23	>0.999999999999999	4.8
Q13131	5'-AMP-activated protein kinase catalytic subunit alpha-1 OS=Homo sapiens GN=PRKAA1 PE=1 SV=4	0.00	0.00	1.20	0.00	0.00	0.00	0.85	0.00	0.00	0.00	0.79	0.00	0.00	0.00	0.00	0.00	0.00	0.00	0.00	0.00	0.00	0.80	0.71	0.20	0.24	0.14	>0.999999999999999	1.7
O00533	Neural cell adhesion molecule L1-like protein OS=Homo sapiens GN=CHL1 PE=1 SV=4	0.00	0.00	1.20	0.43	0.94	0.47	0.00	0.00	0.00	0.00	0.79	0.16	0.00	0.32	0.00	0.00	0.00	0.00	0.00	0.00	0.00	0.00	0.00	0.00	0.33	0.03	>0.999999999999999	12.4
Q9P2M7	Cingulin OS=Homo sapiens GN=CGN PE=1 SV=2	0.00	0.00	0.48	0.00	0.38	0.00	0.34	0.00	0.00	0.00	0.31	0.00	0.00	0.00	0.00	0.00	0.00	0.00	0.00	0.00	0.00	0.00	0.28	0.00	0.13	0.02	>0.999999999999999	5.3
O14787	Transportin-2 OS=Homo sapiens GN=TNPO2 PE=1 SV=3	0.00	0.00	0.72	0.64	0.00	0.00	0.00	0.00	0.00	0.00	0.47	0.00	0.00	0.00	0.00	0.00	0.00	0.00	0.00	0.00	0.00	0.24	0.00	0.00	0.15	0.02	>0.999999999999999	7.6
P49407	Beta-arrestin-1 OS=Homo sapiens GN=ARRB1 PE=1 SV=2	0.00	0.00	1.67	0.00	0.00	1.65	0.00	0.00	0.00	0.00	0.00	0.00	0.00	0.00	1.67	0.00	0.00	0.00	1.27	0.00	0.00	0.56	0.00	0.00	0.28	0.29	>0.999999999999999	1.0
O15084	Serine/threonine-protein phosphatase 6 regulatory ankyrin repeat subunit A OS=Homo sapiens GN=ANKRD28 PE=1	0.00	0.00	0.72	0.00	0.00	0.00	0.00	0.00	0.00	0.56	0.94	0.00	0.00	0.00	0.00	0.00	0.00	0.00	0.00	0.00	0.00	0.00	0.00	0.00	0.18	0.00	>0.999999999999999	#DIV/0!
P08237	6-phosphofructokinase, muscle type OS=Homo sapiens GN=PFKM PE=1 SV=2	0.00	0.00	0.96	0.86	0.00	1.89	0.00	0.00	0.00	0.00	0.00	0.00	0.00	0.00	0.00	0.00	0.00	0.00	0.00	0.00	0.00	0.00	0.00	0.00	0.31	0.00	>0.999999999999999	#DIV/0!
Q7RTR2	Protein NLRC3 OS=Homo sapiens GN=NLRC3 PE=2 SV=2	0.00	0.00	0.72	0.00	0.00	0.00	0.00	0.00	0.00	0.00	0.00	0.00	0.00	0.00	0.00	0.00	0.00	0.00	0.00	0.00	0.00	0.00	0.00	0.00	0.06	0.00	>0.999999999999999	#DIV/0!
Q96IM9	DPY30 domain-containing protein 2 OS=Homo sapiens GN=DYDC2 PE=2 SV=1	0.00	0.00	3.82	0.00	0.00	0.00	0.00	0.00	0.00	0.00	0.00	0.00	0.00	0.00	0.00	0.00	0.00	0.00	0.00	0.00	0.00	0.00	0.00	0.00	0.32	0.00	>0.999999999999999	#DIV/0!
Q15276	Rab GTPase-binding effector protein 1 OS=Homo sapiens GN=RABEP1 PE=1 SV=2	0.00	0.00	0.72	0.00	0.00	0.00	0.00	0.00	0.00	0.00	0.00	0.00	0.00	0.00	0.00	0.00	0.00	0.00	0.00	0.00	0.00	0.00	0.00	0.00	0.06	0.00	>0.999999999999999	#DIV/0!
Q8N4P2	Tetratricopeptide repeat protein 30B OS=Homo sapiens GN=TTC30B PE=2 SV=2	0.00	0.00	0.96	0.00	0.00	0.00	0.00	0.00	0.00	0.74	0.00	0.00	0.00	0.00	0.00	0.00	0.00	0.00	0.00	0.00	0.00	0.00	0.00	0.00	0.14	0.00	>0.999999999999999	#DIV/0!
P07099	Epoxide hydrolase 1 OS=Homo sapiens GN=EPHX1 PE=1 SV=1	0.00	0.00	1.43	1.28	0.00	4.73	0.00	0.00	0.00	1.11	2.04	0.00	0.00	0.00	0.00	0.00	0.00	0.00	0.00	0.00	0.00	0.00	0.00	0.00	0.88	0.00	>0.999999999999999	#DIV/0!
P33240	Cleavage stimulation factor subunit 2 OS=Homo sapiens GN=CSTF2 PE=1 SV=1	0.00	0.00	1.20	0.00	0.00	0.00	0.00	0.00	0.00	0.00	0.00	0.00	0.00	0.00	0.00	0.00	0.00	0.00	0.00	0.00	0.00	0.00	0.00	0.00	0.10	0.00	>0.999999999999999	#DIV/0!
Q9ULH7	MKL/myocardin-like protein 2 OS=Homo sapiens GN=MKL2 PE=1 SV=3	0.00	0.00	1.43	0.64	0.00	0.00	0.00	0.00	0.00	0.00	0.00	0.00	0.00	0.00	0.00	0.00	0.00	0.00	0.00	0.00	0.00	0.00	0.00	0.00	0.17	0.00	>0.999999999999999	#DIV/0!
P02753	Retinol-binding protein 4 OS=Homo sapiens GN=RBP4 PE=1 SV=3	0.00	0.00	3.35	0.00	0.00	0.00	0.00	1.26	1.92	0.00	0.00	1.14	0.00	0.00	3.34	0.00	1.77	2.15	2.53	1.82	2.36	0.00	0.00	0.00	0.64	1.16	>0.999999999999999	0.5
Q13144	Translation initiation factor eIF-2B subunit epsilon OS=Homo sapiens GN=EIF2B5 PE=1 SV=3	0.00	0.00	0.96	0.86	0.00	0.95	0.00	0.00	0.00	0.00	0.00	0.00	0.00	0.00	0.00	0.00	0.00	0.00	0.00	0.00	0.00	0.00	0.00	0.00	0.23	0.00	>0.999999999999999	#DIV/0!
Q8TDR0	TRAF3-interacting protein 1 OS=Homo sapiens GN=TRAF3IP1 PE=1 SV=1	0.00	0.00	0.96	0.00	0.00	0.95	0.00	0.00	0.00	1.48	0.63	0.00	0.00	0.00	0.00	0.00	0.00	0.00	0.00	0.00	0.00	0.00	0.00	0.00	0.33	0.00	>0.999999999999999	#DIV/0!
O14933	Ubiquitin/ISG15-conjugating enzyme E2 L6 OS=Homo sapiens GN=UBE2L6 PE=1 SV=4	0.00	0.00	4.54	4.06	3.58	0.00	7.01	0.00	0.00	3.52	2.99	1.55	4.47	3.05	4.53	3.20	2.40	0.00	3.44	0.00	0.00	1.52	0.00	0.76	2.27	1.95	>0.999999999999999	1.2
Q16537	Serine/threonine-protein phosphatase 2A 56 kDa regulatory subunit epsilon isoform OS=Homo sapiens GN=PPP2R5	0.00	0.00	1.43	0.00	0.00	0.00	0.00	0.00	0.00	0.00	0.94	0.49	0.00	0.00	0.00	0.00	0.00	0.00	0.00	0.00	0.00	0.00	0.00	0.00	0.24	0.00	>0.999999999999999	#DIV/0!
Q9GZQ3	COMM domain-containing protein 5 OS=Homo sapiens GN=COMMD5 PE=1 SV=1	0.00	0.00	3.11	2.78	0.00	0.00	0.00	0.00	0.00	0.00	0.00	0.00	0.00	0.00	0.00	0.00	0.00	0.00	0.00	0.00	0.00	0.00	0.00	0.00	0.49	0.00	>0.999999999999999	#DIV/0!
Q6NXG1	Epithelial splicing regulatory protein 1 OS=Homo sapiens GN=ESRP1 PE=1 SV=2	0.00	0.00	0.96	0.00	0.00	0.00	0.00	0.00	0.00	0.74	0.63	0.00	0.00	0.00	0.00	0.00	0.00	0.00	0.00	0.00	0.00	0.32	0.00	0.00	0.19	0.03	>0.999999999999999	7.3
Q9Y250	Leucine zipper putative tumor suppressor 1 OS=Homo sapiens GN=LZTS1 PE=1 SV=3	0.00	0.00	1.20	0.00	0.00	0.00	0.00	0.00	0.00	0.00	0.00	0.00	0.00	0.00	0.00	0.00	0.00	0.00	0.00	0.00	0.00	0.00	0.00	0.00	0.10	0.00	>0.999999999999999	#DIV/0!
Q13451	Peptidyl-prolyl cis-trans isomerase FKBP5 OS=Homo sapiens GN=FKBP5 PE=1 SV=2	0.00	0.00	1.43	0.00	0.00	0.00	0.00	0.00	0.00	0.00	0.00	0.00	0.00	0.00	0.00	0.00	0.00	0.00	0.00	0.00	0.00	0.00	0.00	0.00	0.12	0.00	>0.999999999999999	#DIV/0!
Q9UJC5	SH3 domain-binding glutamic acid-rich-like protein 2 OS=Homo sapiens GN=SH3BGRL2 PE=1 SV=2	0.00	0.00	6.69	0.00	0.00	0.00	0.00	0.00	0.00	0.00	0.00	0.00	0.00	0.00	6.68	0.00	0.00	0.00	0.00	0.00	0.00	0.00	0.00	0.00	0.56	0.56	>0.999999999999999	1.0
P62304	Small nuclear ribonucleoprotein E OS=Homo sapiens GN=SNRPE PE=1 SV=1	0.00	0.00	7.65	0.00	0.00	7.56	0.00	0.00	0.00	5.93	0.00	0.00	0.00	0.00	0.00	0.00	0.00	0.00	0.00	0.00	0.00	0.00	0.00	0.00	1.76	0.00	>0.999999999999999	#DIV/0!
Q9UJW0	Dynactin subunit 4 OS=Homo sapiens GN=DCTN4 PE=1 SV=1	0.00	0.00	1.43	0.00	0.00	0.00	0.00	0.00	0.00	0.00	0.00	0.00	0.00	0.00	0.00	0.00	0.00	0.00	0.00	0.00	0.00	0.00	0.00	0.00	0.12	0.00	>0.999999999999999	#DIV/0!
Q96FN9	Probable D-tyrosyl-tRNA(Tyr) deacylase 2 OS=Homo sapiens GN=C14orf126 PE=2 SV=1	0.00	0.00	4.30	0.00	0.00	0.00	0.00	0.00	0.00	0.00	0.00	0.00	0.00	0.00	0.00	0.00	0.00	0.00	0.00	0.00	0.00	0.00	0.00	0.00	0.36	0.00	>0.999999999999999	#DIV/0!
Q14409	Putative glycerol kinase 3 OS=Homo sapiens GN=GK3P PE=5 SV=2	0.00	0.00	1.20	2.35	0.94	1.18	0.85	0.00	0.00	2.04	2.67	0.00	0.00	0.00	0.00	0.00	0.00	0.77	0.00	0.00	0.00	0.00	0.00	0.00	0.94	0.06	>0.999999999999999	14.6
Q9BZ23	Pantothenate kinase 2, mitochondrial OS=Homo sapiens GN=PANK2 PE=1 SV=3	0.00	0.00	2.63	0.00	0.00	0.00	0.00	0.00	0.00	0.00	0.00	0.00	0.00	0.00	0.00	0.00	0.00	0.00	0.00	0.00	0.00	0.00	0.00	0.00	0.22	0.00	>0.999999999999999	#DIV/0!
Q6DT37	Serine/threonine-protein kinase MRCK gamma OS=Homo sapiens GN=CDC42BPG PE=1 SV=2	0.00	0.00	0.48	0.00	0.00	0.00	0.00	0.00	0.00	0.00	0.00	0.00	0.00	0.00	0.00	0.00	0.00	0.00	0.00	0.00	0.00	0.00	0.00	0.00	0.04	0.00	>0.999999999999999	#DIV/0!
Q9Y6M1	Insulin-like growth factor 2 mRNA-binding protein 2 OS=Homo sapiens GN=IGF2BP2 PE=1 SV=2	0.00	0.00	1.20	0.00	0.00	0.00	0.00	0.00	0.00	0.00	0.00	0.00	0.00	0.00	0.00	0.00	0.00	0.00	0.00	0.00	0.00	0.00	0.00	0.00	0.10	0.00	>0.999999999999999	#DIV/0!
Q9H0R8	Gamma-aminobutyric acid receptor-associated protein-like 1 OS=Homo sapiens GN=GABARAPL1 PE=1 SV=1	0.00	0.00	6.22	0.00	0.00	6.14	0.00	0.00	0.00	4.82	4.09	0.00	0.00	0.00	6.20	4.37	0.00	0.00	0.00	0.00	0.00	0.00	0.00	0.00	1.77	0.88	>0.999999999999999	2.0
P36954	DNA-directed RNA polymerase II subunit RPB9 OS=Homo sapiens GN=POLR2I PE=1 SV=1	0.00	0.00	5.50	0.00	0.00	0.00	0.00	0.00	0.00	4.26	0.00	0.00	0.00	0.00	0.00	0.00	0.00	0.00	0.00	0.00	0.00	0.00	0.00	0.00	0.81	0.00	>0.999999999999999	#DIV/0!
Q53FA7	Quinone oxidoreductase PIG3 OS=Homo sapiens GN=TP53I3 PE=1 SV=2	0.00	0.00	2.15	1.92	0.00	0.00	0.00	0.81	0.00	1.67	0.00	0.00	0.00	0.00	0.00	0.00	0.00	0.00	0.00	0.00	0.00	1.52	0.00	0.00	0.55	0.13	>0.999999999999999	4.3
Q08378	Golgin subfamily A member 3 OS=Homo sapiens GN=GOLGA3 PE=1 SV=2	0.00	0.00	0.48	0.00	0.00	0.00	0.00	0.00	0.00	0.00	0.00	0.00	0.00	0.00	0.48	0.00	0.00	0.00	0.00	0.00	0.00	0.00	0.00	0.08	0.04	0.05	>0.999999999999999	0.9
P49888	Estrogen sulfotransferase OS=Homo sapiens GN=SULT1E1 PE=1 SV=1	0.00	0.00	2.15	0.00	0.00	2.13	0.00	0.00	0.00	0.00	0.00	0.00	0.00	0.00	0.00	0.00	0.00	0.00	0.00	0.00	0.00	0.00	0.00	0.00	0.36	0.00	>0.999999999999999	#DIV/0!
Q6P1N9	Putative deoxyribonuclease TATDN1 OS=Homo sapiens GN=TATDN1 PE=1 SV=2	0.00	0.00	2.39	0.00	0.00	0.00	0.00	0.00	0.00	0.00	0.00	0.00	0.00	0.00	0.00	0.00	0.00	0.00	0.00	0.00	0.00	0.00	0.00	0.00	0.20	0.00	>0.999999999999999	#DIV/0!
Q9UK45	U6 snRNA-associated Sm-like protein LSm7 OS=Homo sapiens GN=LSM7 PE=1 SV=1	0.00	0.00	7.17	6.41	0.00	0.00	0.00	0.00	0.00	0.00	0.00	2.45	0.00	0.00	0.00	0.00	3.80	0.00	0.00	0.00	0.00	0.00	0.00	0.00	1.34	0.32	>0.999999999999999	4.2
Q9UJX2	Cell division cycle protein 23 homolog OS=Homo sapiens GN=CDC23 PE=1 SV=3	0.00	0.00	1.20	0.00	0.00	0.00	0.00	0.00	0.00	0.00	0.00	0.00	0.00	0.00	0.00	0.00	0.00	0.00	0.00	0.00	0.00	0.00	0.00	0.00	0.10	0.00	>0.999999999999999	#DIV/0!
Q9Y3L3	SH3 domain-binding protein 1 OS=Homo sapiens GN=SH3BP1 PE=1 SV=3	0.00	0.00	0.96	0.00	0.75	0.00	0.00	0.00	0.55	0.00	0.00	0.33	0.00	0.00	0.00	0.00	0.00	0.00	0.00	0.00	0.00	0.00	0.00	0.00	0.22	0.00	>0.999999999999999	#DIV/0!
Q9UJY5	ADP-ribosylation factor-binding protein GGA1 OS=Homo sapiens GN=GGA1 PE=1 SV=1	0.00	0.00	1.20	0.00	0.00	0.00	0.00	0.00	0.00	0.00	0.00	0.00	0.00	0.00	0.00	0.00	0.00	0.00	0.00	0.00	0.00	0.00	0.00	0.00	0.10	0.00	>0.999999999999999	#DIV/0!
Q7LBR1	Charged multivesicular body protein 1b OS=Homo sapiens GN=CHMP1B PE=1 SV=1	0.00	0.00	3.59	3.21	2.83	0.00	0.00	0.00	0.00	0.00	2.36	0.00	0.00	0.00	3.58	0.00	1.90	2.30	0.00	0.00	0.00	0.00	0.00	0.00	1.00	0.65	>0.999999999999999	1.5
Q15637	Splicing factor 1 OS=Homo sapiens GN=SF1 PE=1 SV=4	0.00	0.00	1.20	0.00	0.00	1.18	0.00	0.00	0.00	0.00	0.00	0.00	0.00	0.00	0.00	0.00	0.00	0.00	0.00	0.00	0.00	0.00	0.00	0.00	0.20	0.00	>0.999999999999999	#DIV/0!
Q8IYB7	DIS3-like exonuclease 2 OS=Homo sapiens GN=DIS3L2 PE=1 SV=4	0.00	0.00	0.72	0.00	0.00	0.00	0.00	0.00	0.00	0.00	0.00	0.00	0.00	0.00	0.00	0.00	0.00	0.00	0.00	0.00	0.00	0.00	0.00	0.00	0.06	0.00	>0.999999999999999	#DIV/0!
Q9BUL8	Programmed cell death protein 10 OS=Homo sapiens GN=PDCD10 PE=1 SV=1	0.00	0.00	3.35	2.99	2.64	6.85	2.39	0.00	0.00	0.00	2.20	0.00	0.00	0.00	3.34	0.00	0.00	0.00	0.00	0.00	0.00	0.00	1.99	0.00	1.70	0.44	>0.999999999999999	3.8
Q9UQN3	Charged multivesicular body protein 2b OS=Homo sapiens GN=CHMP2B PE=1 SV=1	0.00	0.00	3.35	0.00	0.00	0.00	0.00	0.00	0.00	0.00	0.00	0.00	0.00	0.00	0.00	0.00	0.00	0.00	0.00	0.00	0.00	0.00	0.00	0.56	0.28	0.05	>0.999999999999999	6.0
Q16527	Cysteine and glycine-rich protein 2 OS=Homo sapiens GN=CSRP2 PE=1 SV=3	0.00	0.00	3.59	0.00	0.00	0.00	0.00	0.00	0.00	0.00	0.00	0.00	0.00	0.00	0.00	0.00	0.00	0.00	0.00	0.00	0.00	0.00	0.00	0.00	0.30	0.00	>0.999999999999999	#DIV/0!
O75886	Signal transducing adapter molecule 2 OS=Homo sapiens GN=STAM2 PE=1 SV=1	0.00	0.00	1.43	0.00	0.00	0.00	0.00	0.54	0.00	0.00	0.00	0.00	0.00	0.00	0.00	0.00	0.00	0.00	0.00	0.00	0.00	0.00	0.00	0.00	0.16	0.00	>0.999999999999999	#DIV/0!
Q7Z4W1	L-xylulose reductase OS=Homo sapiens GN=DCXR PE=1 SV=2	0.00	0.00	3.11	5.77	2.45	6.38	2.22	0.00	0.00	0.00	2.04	2.20	0.00	0.00	0.00	0.00	0.00	0.00	0.00	0.00	0.00	2.16	3.83	0.52	2.02	0.54	>0.999999999999999	3.7
Q9C091	GREB1-like protein OS=Homo sapiens GN=GREB1L PE=2 SV=2	0.00	0.00	0.48	0.00	0.00	0.00	0.00	0.00	0.00	0.00	0.00	0.00	0.00	0.00	0.00	0.00	0.00	0.00	0.00	0.00	0.00	0.00	0.00	0.00	0.04	0.00	>0.999999999999999	#DIV/0!
Q9ULR3	Protein phosphatase 1H OS=Homo sapiens GN=PPM1H PE=1 SV=2	0.00	0.00	1.43	0.00	0.00	1.42	0.00	0.00	0.00	0.00	0.00	0.00	0.00	0.00	0.00	0.00	0.00	0.00	0.00	0.00	0.00	0.00	0.00	0.00	0.24	0.00	>0.999999999999999	#DIV/0!
Q5JS54	Proteasome assembly chaperone 4 OS=Homo sapiens GN=PSMG4 PE=2 SV=2	0.00	0.00	5.98	5.34	0.00	0.00	0.00	0.00	0.00	0.00	0.00	0.00	0.00	0.00	0.00	0.00	0.00	0.00	0.00	0.00	0.00	2.00	0.00	0.00	0.94	0.17	>0.999999999999999	5.7
Q08AM6	Protein VAC14 homolog OS=Homo sapiens GN=VAC14 PE=1 SV=1	0.00	0.00	0.96	0.00	0.00	0.00	0.00	0.00	0.00	0.00	0.00	0.00	0.00	0.64	0.95	0.00	0.00	0.00	0.00	0.00	0.00	0.00	0.00	0.00	0.08	0.13	>0.999999999999999	0.6
Q9BXS5	AP-1 complex subunit mu-1 OS=Homo sapiens GN=AP1M1 PE=1 SV=3	0.00	0.00	1.67	0.00	0.00	0.00	0.00	0.00	0.00	1.30	0.00	0.00	0.00	0.00	0.00	0.00	0.00	0.00	0.00	0.00	0.00	0.00	0.00	0.00	0.25	0.00	>0.999999999999999	#DIV/0!
P83436	Conserved oligomeric Golgi complex subunit 7 OS=Homo sapiens GN=COG7 PE=1 SV=1	0.00	0.00	0.96	0.00	0.00	0.95	0.00	0.00	0.00	0.00	0.00	0.00	0.00	0.00	0.95	0.00	0.00	0.00	0.00	0.00	0.00	0.00	0.00	0.00	0.16	0.08	>0.999999999999999	2.0
P42858	Huntingtin OS=Homo sapiens GN=HTT PE=1 SV=2	0.00	0.00	0.24	0.00	0.00	0.00	0.00	0.00	0.00	0.00	0.00	0.00	0.00	0.00	0.00	0.00	0.00	0.00	0.00	0.00	0.00	0.00	0.00	0.00	0.02	0.00	>0.999999999999999	#DIV/0!
Q13283	Ras GTPase-activating protein-binding protein 1 OS=Homo sapiens GN=G3BP1 PE=1 SV=1	0.00	0.00	1.43	0.00	0.00	1.42	0.00	0.00	0.00	0.00	0.94	0.49	0.00	0.00	0.00	0.00	0.00	0.00	0.00	0.00	0.00	0.00	0.00	0.00	0.36	0.00	>0.999999999999999	#DIV/0!
Q8TAC1	Rieske domain-containing protein OS=Homo sapiens GN=RFESD PE=1 SV=1	0.00	0.00	4.54	0.00	0.00	0.00	0.00	0.00	0.00	0.00	0.00	0.00	0.00	0.00	0.00	0.00	0.00	0.00	0.00	0.00	0.00	0.00	0.00	0.00	0.38	0.00	>0.999999999999999	#DIV/0!
Q92878	DNA repair protein RAD50 OS=Homo sapiens GN=RAD50 PE=1 SV=1	0.00	0.00	0.48	0.00	0.00	0.00	0.00	0.00	0.00	0.00	0.00	0.00	0.00	0.00	0.00	0.00	0.00	0.00	0.00	0.00	0.00	0.00	0.00	0.00	0.04	0.00	>0.999999999999999	#DIV/0!
P57764	Gasdermin-D OS=Homo sapiens GN=GSDMD PE=1 SV=1	0.00	0.00	1.43	0.00	1.13	1.42	0.00	0.00	0.00	0.00	0.00	0.49	0.00	0.00	0.00	0.00	0.00	0.00	0.00	0.00	0.00	0.00	0.00	0.00	0.37	0.00	>0.999999999999999	#DIV/0!
P62310	U6 snRNA-associated Sm-like protein LSm3 OS=Homo sapiens GN=LSM3 PE=1 SV=2	0.00	0.00	6.93	6.20	5.47	6.85	0.00	0.00	0.00	0.00	0.00	2.37	6.82	0.00	0.00	0.00	4.45	0.00	0.00	0.00	0.00	0.00	0.00	1.16	2.32	1.04	>0.999999999999999	2.2
P63302	Selenoprotein W OS=Homo sapiens GN=SEPW1 PE=1 SV=3	0.00	0.00	8.61	0.00	0.00	0.00	0.00	0.00	0.00	0.00	0.00	0.00	0.00	0.00	0.00	0.00	0.00	0.00	0.00	0.00	0.00	0.00	0.00	0.00	0.72	0.00	>0.999999999999999	#DIV/0!
P11047	Laminin subunit gamma-1 OS=Homo sapiens GN=LAMC1 PE=1 SV=3	0.00	0.00	0.48	0.00	0.00	0.00	0.00	0.00	0.00	0.00	0.00	0.00	0.00	0.00	0.00	0.00	0.00	0.00	0.00	0.00	0.00	0.00	0.00	0.00	0.04	0.00	>0.999999999999999	#DIV/0!
Q5T447	E3 ubiquitin-protein ligase HECTD3 OS=Homo sapiens GN=HECTD3 PE=1 SV=1	0.00	0.00	0.72	0.00	0.00	0.71	0.00	0.00	0.00	0.00	0.00	0.00	0.00	0.00	0.00	0.00	0.00	0.00	0.00	0.00	0.00	0.00	0.00	0.00	0.12	0.00	>0.999999999999999	#DIV/0!
A5YKK6	CCR4-NOT transcription complex subunit 1 OS=Homo sapiens GN=CNOT1 PE=1 SV=2	0.00	0.00	0.24	0.00	0.00	0.00	0.00	0.00	0.00	0.00	0.00	0.00	0.00	0.00	0.24	0.00	0.00	0.00	0.00	0.00	0.00	0.00	0.00	0.00	0.02	0.02	>0.999999999999999	1.0
P49207	60S ribosomal protein L34 OS=Homo sapiens GN=RPL34 PE=1 SV=3	0.00	0.00	5.98	5.34	4.71	5.91	0.00	0.00	0.00	0.00	8.96	0.00	0.00	0.00	0.00	0.00	0.00	0.00	0.00	0.00	0.00	0.00	0.00	0.00	2.58	0.00	>0.999999999999999	#DIV/0!
Q8N0X7	Spartin OS=Homo sapiens GN=SPG20 PE=1 SV=1	0.00	0.00	0.96	0.00	0.00	0.00	0.00	0.00	0.00	0.00	0.00	0.00	0.00	0.00	0.00	0.00	0.00	0.00	0.00	0.00	0.00	0.00	0.00	0.00	0.08	0.00	>0.999999999999999	#DIV/0!
Q9Y5X1	Sorting nexin-9 OS=Homo sapiens GN=SNX9 PE=1 SV=1	0.00	0.00	1.20	0.00	0.00	0.00	0.00	0.00	0.00	0.00	0.00	0.00	0.00	0.00	0.00	0.00	0.00	0.00	0.00	0.00	0.00	0.00	0.00	0.00	0.10	0.00	>0.999999999999999	#DIV/0!
P30414	NK-tumor recognition protein OS=Homo sapiens GN=NKTR PE=1 SV=2	0.00	0.00	0.48	0.00	0.00	0.00	0.00	0.00	0.00	0.00	0.00	0.00	0.00	0.00	0.00	0.00	0.00	0.00	0.00	0.00	0.00	0.00	0.00	0.00	0.04	0.00	>0.999999999999999	#DIV/0!
O95487	Protein transport protein Sec24B OS=Homo sapiens GN=SEC24B PE=1 SV=2	0.00	0.00	0.48	0.00	0.00	0.00	0.00	0.00	0.00	0.00	0.31	0.00	0.00	0.00	0.00	0.00	0.00	0.00	0.00	0.00	0.00	0.00	0.00	0.00	0.07	0.00	>0.999999999999999	#DIV/0!
Q9BVM2	Protein DPCD OS=Homo sapiens GN=DPCD PE=1 SV=2	0.00	0.00	3.59	2.99	2.64	0.00	0.00	0.00	0.00	0.00	4.88	0.00	0.00	2.25	0.00	0.00	0.00	0.00	0.00	0.00	0.00	0.00	1.99	0.00	1.17	0.35	>0.999999999999999	3.3
O00629	Importin subunit alpha-4 OS=Homo sapiens GN=KPNA4 PE=1 SV=1	0.00	0.00	1.43	0.00	0.00	1.42	0.00	0.00	0.00	0.00	0.00	0.98	0.00	0.00	0.00	0.00	0.00	0.00	0.00	0.00	0.00	0.00	0.00	0.00	0.32	0.00	>0.999999999999999	#DIV/0!
P15924	Desmoplakin OS=Homo sapiens GN=DSP PE=1 SV=3	0.00	0.00	0.24	0.00	0.00	0.00	0.00	0.00	0.00	0.00	0.31	0.00	0.00	0.16	0.00	0.00	0.00	0.15	0.18	0.00	0.00	0.08	0.00	0.08	0.05	0.05	>0.999999999999999	0.8
O15061	Synemin OS=Homo sapiens GN=SYNM PE=1 SV=2	0.00	0.00	0.48	0.00	0.00	0.00	0.00	0.00	0.00	0.00	0.00	0.00	0.00	0.00	0.00	0.00	0.00	0.00	0.00	0.00	0.00	0.00	0.00	0.00	0.04	0.00	>0.999999999999999	#DIV/0!
Q9UBB5	Methyl-CpG-binding domain protein 2 OS=Homo sapiens GN=MBD2 PE=1 SV=1	0.00	0.00	1.91	1.71	0.00	1.89	0.00	0.00	0.00	0.00	0.00	0.65	0.00	1.28	0.00	0.00	1.01	0.00	0.00	0.00	0.00	0.00	0.00	0.00	0.51	0.19	>0.999999999999999	2.7
O60826	Coiled-coil domain-containing protein 22 OS=Homo sapiens GN=CCDC22 PE=1 SV=1	0.00	0.00	1.20	0.00	0.00	0.00	0.00	0.00	0.00	0.00	0.00	0.00	0.00	0.00	0.00	0.00	0.00	0.00	0.00	0.00	0.00	0.00	0.00	0.00	0.10	0.00	>0.999999999999999	#DIV/0!
Q92734	Protein TFG OS=Homo sapiens GN=TFG PE=1 SV=2	0.00	0.00	1.91	1.71	0.00	0.00	1.37	0.00	0.00	0.00	1.26	0.00	0.00	1.28	1.91	1.35	0.00	0.00	0.00	0.00	0.00	0.64	1.14	0.00	0.52	0.53	>0.999999999999999	1.0
A4UGR9	Xin actin-binding repeat-containing protein 2 OS=Homo sapiens GN=XIRP2 PE=1 SV=2	0.00	0.00	0.24	0.00	0.00	0.00	0.00	0.00	0.00	0.00	0.00	0.00	0.00	0.00	0.00	0.00	0.00	0.00	0.00	0.00	0.00	0.00	0.00	0.00	0.02	0.00	>0.999999999999999	#DIV/0!
Q8IYB5	Stromal membrane-associated protein 1 OS=Homo sapiens GN=SMAP1 PE=1 SV=2	0.00	0.00	1.43	0.00	0.00	0.00	0.00	0.00	0.00	0.00	0.94	0.00	0.00	0.00	0.00	0.00	0.00	0.00	0.00	0.00	0.00	0.00	0.00	0.00	0.20	0.00	>0.999999999999999	#DIV/0!
Q9Y2R9	28S ribosomal protein S7, mitochondrial OS=Homo sapiens GN=MRPS7 PE=1 SV=2	0.00	0.00	2.87	0.00	0.00	0.00	0.00	0.00	0.00	0.00	0.00	0.00	0.00	0.00	0.00	0.00	0.00	0.00	0.00	0.00	0.00	0.00	0.00	0.00	0.24	0.00	>0.999999999999999	#DIV/0!
Q7L190	Developmental pluripotency-associated protein 4 OS=Homo sapiens GN=DPPA4 PE=1 SV=2	0.00	0.00	2.39	0.00	0.00	0.00	0.00	0.00	0.00	1.85	0.00	0.00	0.00	0.00	2.38	0.00	0.00	0.00	0.00	0.00	0.00	0.00	0.00	0.00	0.35	0.20	>0.999999999999999	1.8
Q01415	N-acetylgalactosamine kinase OS=Homo sapiens GN=GALK2 PE=1 SV=1	0.00	0.00	1.43	0.00	0.00	0.00	0.00	0.00	0.00	0.00	0.00	0.00	0.00	0.00	0.00	0.00	0.00	0.00	0.00	0.00	0.00	0.00	0.00	0.00	0.12	0.00	>0.999999999999999	#DIV/0!
Q15642	Cdc42-interacting protein 4 OS=Homo sapiens GN=TRIP10 PE=1 SV=3	0.00	0.00	1.20	0.00	0.00	0.00	0.00	0.00	0.00	0.00	0.00	0.00	0.00	0.00	0.00	0.00	0.00	0.00	0.90	0.00	0.00	0.00	0.00	0.00	0.10	0.08	>0.999999999999999	1.3
Q8N1B4	Vacuolar protein sorting-associated protein 52 homolog OS=Homo sapiens GN=VPS52 PE=1 SV=1	0.00	0.00	0.96	0.00	0.00	0.00	0.00	0.00	0.00	0.00	0.00	0.00	0.00	0.00	0.00	0.00	0.00	0.00	0.00	0.00	0.00	0.00	0.00	0.00	0.08	0.00	>0.999999999999999	#DIV/0!
Q9Y3C6	Peptidyl-prolyl cis-trans isomerase-like 1 OS=Homo sapiens GN=PPIL1 PE=1 SV=1	0.00	0.00	4.30	0.00	0.00	0.00	0.00	0.00	0.00	0.00	0.00	0.00	0.00	0.00	0.00	0.00	0.00	0.00	0.00	0.00	0.00	1.44	0.00	0.00	0.36	0.12	>0.999999999999999	3.0
Q9UPT5	Exocyst complex component 7 OS=Homo sapiens GN=EXOC7 PE=1 SV=3	0.00	0.00	0.96	0.00	0.00	0.00	0.00	0.00	0.00	0.00	0.00	0.00	0.00	0.00	0.00	0.00	0.00	0.00	0.00	0.00	0.00	0.00	0.00	0.00	0.08	0.00	>0.999999999999999	#DIV/0!
Q8N960	Centrosomal protein of 120 kDa OS=Homo sapiens GN=CEP120 PE=2 SV=2	0.00	0.00	0.72	0.00	0.00	0.00	0.00	0.00	0.00	0.00	0.00	0.24	0.00	0.00	0.00	0.00	0.00	0.00	0.00	0.00	0.00	0.00	0.00	0.00	0.08	0.00	>0.999999999999999	#DIV/0!
Q8WVV9	Heterogeneous nuclear ribonucleoprotein L-like OS=Homo sapiens GN=HNRPLL PE=1 SV=1	0.00	0.00	1.20	1.07	0.00	1.18	0.00	0.00	0.00	0.00	0.00	0.00	0.00	0.00	0.00	0.00	0.00	0.00	0.00	0.00	0.00	0.00	0.00	0.00	0.29	0.00	>0.999999999999999	#DIV/0!
P51452	Dual specificity protein phosphatase 3 OS=Homo sapiens GN=DUSP3 PE=1 SV=1	0.00	0.00	3.82	0.00	0.00	0.00	2.74	0.00	0.00	0.00	2.52	0.00	0.00	0.00	0.00	0.00	0.00	0.00	2.89	0.00	0.00	2.80	2.27	0.00	0.76	0.66	>0.999999999999999	1.1
P19784	Casein kinase II subunit alpha' OS=Homo sapiens GN=CSNK2A2 PE=1 SV=1	0.00	0.00	1.91	0.00	0.00	4.02	0.00	0.00	0.00	0.00	0.00	0.00	0.00	0.00	0.00	0.00	0.00	0.00	0.00	0.00	0.00	0.00	0.00	0.00	0.49	0.00	>0.999999999999999	#DIV/0!
Q9BZV1	UBX domain-containing protein 6 OS=Homo sapiens GN=UBXN6 PE=1 SV=1	0.00	0.00	1.67	0.00	0.00	1.65	0.00	0.00	0.00	0.00	0.00	0.00	0.00	0.00	0.00	0.00	0.00	0.00	0.00	0.00	0.00	0.00	0.00	0.00	0.28	0.00	>0.999999999999999	#DIV/0!
Q8N5R6	Coiled-coil domain-containing protein 33 OS=Homo sapiens GN=CCDC33 PE=1 SV=3	0.00	0.00	0.72	0.00	0.00	0.00	0.00	0.00	0.00	0.00	0.00	0.00	0.00	0.00	0.00	0.00	0.00	0.00	0.00	0.00	0.00	0.00	0.00	0.00	0.06	0.00	>0.999999999999999	#DIV/0!
O00178	GTP-binding protein 1 OS=Homo sapiens GN=GTPBP1 PE=1 SV=3	0.00	0.00	0.96	0.86	0.75	0.00	0.00	0.00	0.00	0.00	0.00	0.33	0.00	0.00	0.00	0.00	0.00	0.00	0.00	0.00	0.00	0.00	0.00	0.00	0.24	0.00	>0.999999999999999	#DIV/0!
Q86TP1	Protein prune homolog OS=Homo sapiens GN=PRUNE PE=1 SV=2	0.00	0.00	1.43	1.28	0.00	0.00	0.00	0.00	0.00	1.11	0.00	0.49	0.00	0.00	0.00	0.00	0.00	0.00	0.00	0.00	0.00	0.00	0.00	0.00	0.36	0.00	>0.999999999999999	#DIV/0!
O15195	Villin-like protein OS=Homo sapiens GN=VILL PE=2 SV=3	0.00	0.00	0.72	0.64	0.00	0.71	0.00	0.00	0.00	0.00	0.47	0.00	0.00	0.00	0.00	0.00	0.00	0.00	0.00	0.00	0.00	0.56	1.56	0.00	0.21	0.18	>0.999999999999999	1.2
Q9Y2M0	Fanconi-associated nuclease 1 OS=Homo sapiens GN=FAN1 PE=1 SV=4	0.00	0.00	0.72	0.00	0.00	0.00	0.00	0.00	0.00	1.11	0.00	0.00	0.00	0.00	0.00	0.00	0.00	0.00	0.00	0.00	0.00	0.00	0.00	0.00	0.15	0.00	>0.999999999999999	#DIV/0!
Q9P0W8	Spermatogenesis-associated protein 7 OS=Homo sapiens GN=SPATA7 PE=2 SV=3	0.00	0.00	1.20	1.07	0.94	0.00	0.00	0.00	0.00	0.00	0.79	0.41	1.18	0.80	1.19	0.84	0.63	0.77	0.00	0.65	0.84	0.40	0.71	0.00	0.37	0.67	>0.999999999999999	0.5
Q6IC98	GRAM domain-containing protein 4 OS=Homo sapiens GN=GRAMD4 PE=1 SV=1	0.00	0.00	1.20	0.00	0.00	0.00	0.00	0.00	0.00	0.00	0.00	0.00	0.00	0.00	0.00	0.00	0.00	0.00	0.00	0.00	0.00	0.00	0.00	0.00	0.10	0.00	>0.999999999999999	#DIV/0!
Q2TAA2	Isoamyl acetate-hydrolyzing esterase 1 homolog OS=Homo sapiens GN=IAH1 PE=1 SV=1	0.00	0.00	2.87	0.00	0.00	0.00	0.00	0.00	0.00	0.00	0.00	0.00	0.00	0.00	0.00	0.00	0.00	0.00	0.00	0.00	0.00	0.00	0.00	0.00	0.24	0.00	>0.999999999999999	#DIV/0!
Q13670	Putative postmeiotic segregation increased 2-like protein 11 OS=Homo sapiens GN=PMS2P11 PE=5 SV=1	0.00	0.00	2.63	0.00	0.00	0.00	0.00	0.00	0.00	0.00	0.00	0.00	0.00	0.00	0.00	0.00	0.00	0.00	0.00	0.00	0.00	0.00	0.00	0.00	0.22	0.00	>0.999999999999999	#DIV/0!
Q99611	Selenide, water dikinase 2 OS=Homo sapiens GN=SEPHS2 PE=1 SV=3	0.00	0.00	1.67	0.00	0.00	0.00	0.00	0.00	0.00	0.00	0.00	0.00	0.00	0.00	0.00	0.00	0.00	0.00	0.00	0.00	0.00	0.00	0.00	0.00	0.14	0.00	>0.999999999999999	#DIV/0!
Q86XH1	IQ and AAA domain-containing protein 1 OS=Homo sapiens GN=IQCA1 PE=2 SV=1	0.00	0.00	0.72	0.00	0.00	0.00	0.00	0.00	0.00	0.00	0.00	0.00	0.00	0.00	0.00	0.00	0.00	0.00	0.00	0.00	0.00	0.00	0.00	0.00	0.06	0.00	>0.999999999999999	#DIV/0!
P49789	Bis(5'-adenosyl)-triphosphatase OS=Homo sapiens GN=FHIT PE=1 SV=3	0.00	0.00	4.78	0.00	0.00	4.73	0.00	0.00	0.00	0.00	0.00	0.00	0.00	0.00	0.00	0.00	0.00	0.00	0.00	0.00	0.00	0.00	0.00	0.00	0.79	0.00	>0.999999999999999	#DIV/0!
Q9HD15	Steroid receptor RNA activator 1 OS=Homo sapiens GN=SRA1 PE=1 SV=1	0.00	0.00	3.11	0.00	0.00	0.00	0.00	0.00	0.00	0.00	0.00	1.06	0.00	0.00	0.00	0.00	0.00	0.00	0.00	0.00	0.00	0.00	0.00	0.00	0.35	0.00	>0.999999999999999	#DIV/0!
Q9Y4P1	Cysteine protease ATG4B OS=Homo sapiens GN=ATG4B PE=1 SV=2	0.00	0.00	1.67	1.50	0.00	0.00	0.00	0.00	0.00	0.00	0.00	0.00	0.00	0.00	0.00	0.00	0.00	0.00	0.00	0.00	0.00	0.00	0.99	0.00	0.26	0.08	>0.999999999999999	3.2
Q8TES7	Fas-binding factor 1 OS=Homo sapiens GN=FBF1 PE=1 SV=2	0.00	0.00	0.72	0.00	0.00	0.00	0.00	0.00	0.00	0.00	0.00	0.00	0.00	0.00	0.00	0.00	0.00	0.00	0.00	0.00	0.00	0.00	0.00	0.00	0.06	0.00	>0.999999999999999	#DIV/0!
P46939	Utrophin OS=Homo sapiens GN=UTRN PE=1 SV=2	0.00	0.00	0.24	0.21	0.00	0.24	0.00	0.00	0.00	0.00	0.00	0.00	0.00	0.00	0.00	0.00	0.00	0.00	0.00	0.00	0.00	0.00	0.00	0.00	0.06	0.00	>0.999999999999999	#DIV/0!
Q6GMV2	SET and MYND domain-containing protein 5 OS=Homo sapiens GN=SMYD5 PE=1 SV=2	0.00	0.00	1.67	0.00	0.00	0.00	0.00	0.00	0.00	0.00	0.00	0.00	0.00	0.00	0.00	0.00	0.00	0.00	0.00	0.00	0.00	0.00	0.00	0.00	0.14	0.00	>0.999999999999999	#DIV/0!
Q08752	Peptidyl-prolyl cis-trans isomerase D OS=Homo sapiens GN=PPID PE=1 SV=3	0.00	0.00	1.91	0.00	0.00	0.00	0.00	0.00	0.00	0.00	2.67	0.00	0.00	0.00	4.05	0.00	0.00	0.00	0.00	0.00	0.00	0.64	0.00	0.00	0.38	0.39	>0.999999999999999	1.0
Q9UKZ1	UPF0760 protein C2orf29 OS=Homo sapiens GN=C2orf29 PE=1 SV=1	0.00	0.00	1.43	0.00	0.00	0.00	0.00	0.00	0.00	0.00	0.00	0.00	0.00	0.00	0.00	0.00	0.00	0.00	0.00	0.00	0.00	0.00	0.00	0.00	0.12	0.00	>0.999999999999999	#DIV/0!
Q9UL36	Zinc finger protein 236 OS=Homo sapiens GN=ZNF236 PE=2 SV=2	0.00	0.00	0.48	0.00	0.00	0.00	0.00	0.00	0.00	0.00	0.00	0.00	0.00	0.00	0.00	0.00	0.00	0.00	0.00	0.00	0.00	0.00	0.00	0.00	0.04	0.00	>0.999999999999999	#DIV/0!
Q96T17	MAP7 domain-containing protein 2 OS=Homo sapiens GN=MAP7D2 PE=1 SV=2	0.00	0.00	0.96	0.00	0.00	0.00	0.00	0.00	0.00	0.00	0.00	0.00	0.00	0.00	0.00	0.00	0.00	0.00	0.00	0.00	0.00	0.00	0.00	0.00	0.08	0.00	>0.999999999999999	#DIV/0!
Q14126	Desmoglein-2 OS=Homo sapiens GN=DSG2 PE=1 SV=2	0.00	0.00	0.72	0.00	0.00	1.18	0.00	0.00	0.00	0.00	0.47	0.00	0.00	0.00	0.00	0.50	0.38	0.00	0.00	0.00	0.00	0.24	0.71	0.12	0.20	0.16	>0.999999999999999	1.2
Q9P260	LisH domain and HEAT repeat-containing protein KIAA1468 OS=Homo sapiens GN=KIAA1468 PE=1 SV=2	0.00	0.00	0.48	0.00	0.00	0.00	0.00	0.00	0.00	0.00	0.00	0.00	0.00	0.00	0.00	0.00	0.00	0.00	0.00	0.00	0.00	0.00	0.00	0.00	0.04	0.00	>0.999999999999999	#DIV/0!
Q8NFQ5	BPI fold-containing family B member 6 OS=Homo sapiens GN=BPIFB6 PE=2 SV=1	0.00	0.00	1.67	0.00	0.00	0.00	0.00	0.00	0.00	0.00	0.00	0.00	0.00	0.00	0.00	0.00	0.00	0.00	0.00	0.00	0.00	0.00	0.00	0.00	0.14	0.00	>0.999999999999999	#DIV/0!
Q8N136	Outer row dynein assembly protein 16 homolog OS=Homo sapiens GN=WDR69 PE=1 SV=1	0.00	0.00	1.67	0.00	0.00	1.65	0.00	0.00	0.00	0.00	0.00	0.00	0.00	0.00	0.00	0.00	0.00	0.00	0.00	0.00	0.00	0.00	0.00	0.00	0.28	0.00	>0.999999999999999	#DIV/0!
P46108	Adapter molecule crk OS=Homo sapiens GN=CRK PE=1 SV=2	0.00	0.00	2.39	0.00	1.89	0.00	1.71	0.00	0.00	0.00	1.57	0.00	0.00	0.00	0.00	0.00	0.00	1.53	0.00	0.00	0.00	0.80	1.42	0.84	0.63	0.38	>0.999999999999999	1.6
Q14D04	Ventricular zone-expressed PH domain-containing protein homolog 1 OS=Homo sapiens GN=VEPH1 PE=1 SV=1	0.00	0.00	0.72	0.00	0.00	0.00	0.00	0.00	0.00	0.00	0.00	0.00	0.00	0.00	0.00	0.00	0.00	0.00	0.00	0.00	0.00	0.00	0.00	0.00	0.06	0.00	>0.999999999999999	#DIV/0!
Q9UKX3	Myosin-13 OS=Homo sapiens GN=MYH13 PE=1 SV=2	0.00	0.00	0.24	0.00	0.00	0.00	0.00	0.00	0.00	0.00	0.00	0.00	0.00	0.00	0.00	0.00	0.00	0.00	0.00	0.00	0.00	0.00	0.00	0.00	0.02	0.00	>0.999999999999999	#DIV/0!
O14975	Very long-chain acyl-CoA synthetase OS=Homo sapiens GN=SLC27A2 PE=1 SV=2	0.00	0.00	1.20	0.00	0.00	0.00	0.00	0.00	0.00	0.00	0.00	0.00	0.00	0.00	0.00	0.00	0.00	0.00	0.00	0.00	0.00	0.00	0.00	0.00	0.10	0.00	>0.999999999999999	#DIV/0!
Q8NFW1	Collagen alpha-1(XXII) chain OS=Homo sapiens GN=COL22A1 PE=1 SV=2	0.00	0.00	0.48	0.00	0.00	0.00	0.00	0.00	0.00	0.00	0.00	0.00	0.00	0.00	0.00	0.00	0.00	0.00	0.00	0.00	0.00	0.00	0.00	0.00	0.04	0.00	>0.999999999999999	#DIV/0!
Q8N8S7	Protein enabled homolog OS=Homo sapiens GN=ENAH PE=1 SV=2	0.00	0.00	1.20	0.00	0.00	1.18	0.00	0.00	0.00	0.00	1.57	0.41	0.00	0.00	0.00	0.00	0.00	0.00	0.00	0.00	0.00	0.00	0.00	0.00	0.36	0.00	>0.999999999999999	#DIV/0!
P03950	Angiogenin OS=Homo sapiens GN=ANG PE=1 SV=1	0.00	0.00	4.78	0.00	0.00	0.00	0.00	0.00	2.74	0.00	0.00	1.63	0.00	3.21	0.00	0.00	2.53	3.07	0.00	0.00	0.00	1.60	0.00	0.80	0.76	0.93	>0.999999999999999	0.8
P43246	DNA mismatch repair protein Msh2 OS=Homo sapiens GN=MSH2 PE=1 SV=1	0.00	0.00	0.72	0.00	0.00	0.00	0.00	0.00	0.00	0.00	0.00	0.00	0.00	0.00	0.00	0.00	0.00	0.00	0.00	0.00	0.00	0.00	0.00	0.00	0.06	0.00	>0.999999999999999	#DIV/0!
P68363	Tubulin alpha-1B chain OS=Homo sapiens GN=TUBA1B PE=1 SV=1	0.00	0.00	0.00	44.90	0.00	0.00	0.00	14.18	0.00	0.00	0.00	0.00	0.00	22.64	0.00	0.00	0.00	0.00	0.00	8.44	0.00	0.00	0.00	0.00	4.92	2.59	>0.999999999999999	1.9
Q9BVA1	Tubulin beta-2B chain OS=Homo sapiens GN=TUBB2B PE=1 SV=1	0.00	0.00	0.00	33.99	0.00	0.00	0.00	0.00	0.00	0.00	0.00	0.00	0.00	0.00	0.00	0.00	0.00	0.00	0.00	0.00	0.00	10.22	0.00	0.00	2.83	0.85	>0.999999999999999	3.3
P04259	Keratin, type II cytoskeletal 6B OS=Homo sapiens GN=KRT6B PE=1 SV=5	0.00	0.00	0.00	3.63	0.00	0.00	0.00	0.00	0.00	0.00	0.00	0.00	0.00	0.00	0.00	0.00	0.00	0.00	0.00	0.00	0.00	0.00	2.41	0.00	0.30	0.20	>0.999999999999999	1.5
P62140	Serine/threonine-protein phosphatase PP1-beta catalytic subunit OS=Homo sapiens GN=PPP1CB PE=1 SV=3	0.00	0.00	0.00	6.20	7.54	0.00	0.00	1.63	2.47	0.00	8.18	5.30	0.00	0.00	0.00	0.00	0.00	0.00	5.25	0.00	0.00	3.20	5.68	0.00	2.61	1.18	>0.999999999999999	2.2
Q8TAX9	Gasdermin-B OS=Homo sapiens GN=GSDMB PE=1 SV=2	0.00	0.00	0.00	3.21	0.00	3.54	3.93	0.00	0.00	0.00	0.00	0.57	0.00	0.00	0.00	0.00	0.00	0.00	1.27	0.00	0.00	0.56	0.00	0.00	0.94	0.15	>0.999999999999999	6.2
Q96HY6	DDRGK domain-containing protein 1 OS=Homo sapiens GN=DDRGK1 PE=1 SV=2	0.00	0.00	0.00	4.06	0.00	0.00	0.00	0.00	0.00	1.67	0.00	1.55	0.00	0.00	0.00	0.00	0.00	0.00	0.00	0.00	0.00	0.00	0.00	0.36	0.61	0.03	>0.999999999999999	20.2
Q13596	Sorting nexin-1 OS=Homo sapiens GN=SNX1 PE=1 SV=3	0.00	0.00	0.00	2.35	1.13	0.00	0.00	0.54	0.82	0.00	1.73	0.49	1.41	0.96	1.43	0.00	0.00	0.92	0.00	0.00	1.01	0.88	0.00	0.44	0.59	0.59	>0.999999999999999	1.0
Q9UNS2	COP9 signalosome complex subunit 3 OS=Homo sapiens GN=COPS3 PE=1 SV=3	0.00	0.00	0.00	2.99	0.00	1.65	0.00	0.00	0.00	0.00	2.20	0.00	0.00	1.12	1.67	1.18	0.00	0.00	0.00	0.00	0.00	0.00	1.99	0.00	0.57	0.50	>0.999999999999999	1.1
Q52LJ0	Protein FAM98B OS=Homo sapiens GN=FAM98B PE=1 SV=1	0.00	0.00	0.00	3.85	1.70	4.25	0.00	0.00	0.00	1.67	1.42	0.00	0.00	0.00	2.15	0.00	1.14	0.00	0.00	0.00	0.00	0.00	2.55	0.00	1.07	0.49	>0.999999999999999	2.2
Q8TBX8	Phosphatidylinositol-5-phosphate 4-kinase type-2 gamma OS=Homo sapiens GN=PIP4K2C PE=1 SV=3	0.00	0.00	0.00	1.50	1.32	0.00	1.20	0.00	0.00	1.30	2.20	0.57	0.00	0.00	0.00	0.00	0.00	0.00	0.00	0.00	0.00	0.00	0.99	0.00	0.67	0.08	>0.999999999999999	8.1
Q96PP9	Guanylate-binding protein 4 OS=Homo sapiens GN=GBP4 PE=1 SV=2	0.00	0.00	0.00	0.86	0.00	0.00	1.54	0.00	0.00	0.00	0.00	0.73	0.00	0.00	0.00	0.00	0.00	0.00	0.00	0.00	0.00	0.00	0.00	0.00	0.26	0.00	>0.999999999999999	#DIV/0!
P46783	40S ribosomal protein S10 OS=Homo sapiens GN=RPS10 PE=1 SV=1	0.00	0.00	0.00	8.34	7.35	0.00	0.00	0.00	0.00	0.00	10.07	0.00	0.00	0.00	0.00	0.00	0.00	0.00	0.00	0.00	0.00	0.00	0.00	0.00	2.15	0.00	>0.999999999999999	#DIV/0!
P54619	5'-AMP-activated protein kinase subunit gamma-1 OS=Homo sapiens GN=PRKAG1 PE=1 SV=1	0.00	0.00	0.00	3.85	0.00	2.13	1.54	0.00	0.00	0.00	0.00	0.73	0.00	0.00	0.00	0.00	0.00	0.00	0.00	0.00	0.00	0.00	0.00	0.00	0.69	0.00	>0.999999999999999	#DIV/0!
O43252	Bifunctional 3'-phosphoadenosine 5'-phosphosulfate synthase 1 OS=Homo sapiens GN=PAPSS1 PE=1 SV=2	0.00	0.00	0.00	2.99	0.00	0.00	0.00	0.00	0.00	0.93	0.79	0.00	0.00	0.00	0.00	0.00	0.00	0.00	0.00	0.00	0.00	0.00	0.40	0.00	0.39	0.03	>0.999999999999999	11.8
Q16658	Fascin OS=Homo sapiens GN=FSCN1 PE=1 SV=3	0.00	0.00	0.00	2.57	0.00	0.00	0.00	0.00	0.00	0.00	0.00	1.55	0.00	0.00	1.43	2.02	0.00	0.92	3.44	0.78	0.00	0.48	0.00	0.76	0.34	0.82	>0.999999999999999	0.4
P04430	Ig kappa chain V-I region BAN OS=Homo sapiens PE=1 SV=1	0.00	0.00	0.00	6.20	0.00	0.00	0.00	0.00	0.00	0.00	0.00	0.00	0.00	0.00	6.92	11.10	0.00	4.45	5.25	0.00	0.00	5.27	4.12	0.00	0.52	3.09	>0.999999999999999	0.2
Q8TAX7	Mucin-7 OS=Homo sapiens GN=MUC7 PE=1 SV=2	0.00	0.00	0.00	1.71	0.00	0.00	0.00	0.00	1.10	0.00	0.00	1.39	0.00	0.00	0.00	1.35	1.01	0.00	0.00	0.00	1.35	0.00	1.14	0.68	0.35	0.46	>0.999999999999999	0.8
Q8IZP0	Abl interactor 1 OS=Homo sapiens GN=ABI1 PE=1 SV=4	0.00	0.00	0.00	1.28	0.00	0.00	0.00	0.00	0.00	0.00	0.94	0.00	0.00	0.00	0.00	0.00	0.00	0.00	0.00	0.00	0.00	0.00	0.00	0.00	0.19	0.00	>0.999999999999999	#DIV/0!
Q9Y2L1	Exosome complex exonuclease RRP44 OS=Homo sapiens GN=DIS3 PE=1 SV=2	0.00	0.00	0.00	0.64	0.00	1.42	0.00	0.00	0.00	1.11	0.47	0.24	0.00	0.00	0.00	0.00	0.00	0.00	0.00	0.00	0.00	0.24	0.43	0.00	0.32	0.06	>0.999999999999999	5.8
Q9NP72	Ras-related protein Rab-18 OS=Homo sapiens GN=RAB18 PE=1 SV=1	0.00	0.00	0.00	10.69	0.00	3.31	0.00	0.00	0.00	0.00	2.20	0.00	0.00	0.00	0.00	0.00	0.00	0.00	0.00	0.00	0.00	1.12	0.00	0.00	1.35	0.09	>0.999999999999999	14.5
Q96AG4	Leucine-rich repeat-containing protein 59 OS=Homo sapiens GN=LRRC59 PE=1 SV=1	0.00	0.00	0.00	1.92	0.00	2.13	1.54	0.00	0.00	0.00	0.00	1.63	2.12	0.00	2.15	1.51	0.00	0.00	3.62	0.00	0.00	0.00	0.00	0.00	0.60	0.78	>0.999999999999999	0.8
Q9H0D6	5'-3' exoribonuclease 2 OS=Homo sapiens GN=XRN2 PE=1 SV=1	0.00	0.00	0.00	0.64	0.57	1.42	0.51	0.00	0.00	1.11	0.47	0.49	0.00	0.00	0.72	0.00	0.00	0.00	0.00	0.00	0.00	0.00	0.00	0.12	0.43	0.07	>0.999999999999999	6.2
P16070	CD44 antigen OS=Homo sapiens GN=CD44 PE=1 SV=3	0.00	0.00	0.00	0.86	0.75	1.89	0.68	0.00	0.55	0.00	0.63	0.33	0.00	0.64	0.95	0.67	1.01	1.23	2.17	0.00	0.68	0.32	1.14	0.00	0.47	0.73	>0.999999999999999	0.6
Q9BQS8	FYVE and coiled-coil domain-containing protein 1 OS=Homo sapiens GN=FYCO1 PE=1 SV=3	0.00	0.00	0.00	0.43	0.00	0.47	0.00	0.00	0.00	0.00	0.00	0.00	0.00	0.00	0.00	0.00	0.00	0.00	0.00	0.00	0.00	0.00	0.00	0.00	0.08	0.00	>0.999999999999999	#DIV/0!
P03952	Plasma kallikrein OS=Homo sapiens GN=KLKB1 PE=1 SV=1	0.00	0.00	0.00	0.86	1.70	0.00	1.54	0.36	1.92	0.74	0.00	1.14	2.12	0.00	0.95	0.67	5.31	1.38	1.63	3.12	2.36	0.00	0.00	0.36	0.69	1.49	>0.999999999999999	0.5
P27169	Serum paraoxonase/arylesterase 1 OS=Homo sapiens GN=PON1 PE=1 SV=3	0.00	0.00	0.00	5.77	5.09	0.00	2.91	0.00	5.20	3.15	0.00	0.65	1.88	0.00	4.05	1.35	4.81	2.61	1.45	6.36	1.35	0.64	0.00	0.32	1.90	2.07	>0.999999999999999	0.9
P83731	60S ribosomal protein L24 OS=Homo sapiens GN=RPL24 PE=1 SV=1	0.00	0.00	0.00	8.77	0.00	0.00	0.00	0.00	0.00	0.00	0.00	0.00	0.00	0.00	4.53	0.00	0.00	0.00	0.00	0.00	0.00	1.52	2.70	0.00	0.73	0.73	>0.999999999999999	1.0
P29466	Caspase-1 OS=Homo sapiens GN=CASP1 PE=1 SV=1	0.00	0.00	0.00	1.50	1.32	0.00	1.20	0.00	0.00	0.00	0.00	0.00	0.00	0.00	0.00	0.00	0.00	0.00	0.00	0.00	0.00	0.56	0.00	0.00	0.33	0.05	>0.999999999999999	7.2
Q96HR9	Receptor expression-enhancing protein 6 OS=Homo sapiens GN=REEP6 PE=1 SV=1	0.00	0.00	0.00	3.42	0.00	0.00	0.00	0.00	0.00	0.00	0.00	0.00	0.00	0.00	0.00	0.00	0.00	0.00	0.00	0.00	0.00	0.00	0.00	0.00	0.29	0.00	>0.999999999999999	#DIV/0!
Q9H3P7	Golgi resident protein GCP60 OS=Homo sapiens GN=ACBD3 PE=1 SV=4	0.00	0.00	0.00	1.07	0.00	0.00	0.00	0.00	0.00	0.00	0.00	1.39	0.00	0.00	1.19	0.84	0.00	0.00	0.90	0.00	0.00	0.00	0.00	0.20	0.20	0.26	>0.999999999999999	0.8
Q9H5N1	Rab GTPase-binding effector protein 2 OS=Homo sapiens GN=RABEP2 PE=1 SV=2	0.00	0.00	0.00	1.07	0.00	0.00	0.00	0.00	0.00	0.00	1.73	0.00	0.00	0.00	0.00	0.00	0.00	0.00	0.00	0.00	0.00	0.00	0.71	0.44	0.23	0.10	>0.999999999999999	2.4
Q9NVD7	Alpha-parvin OS=Homo sapiens GN=PARVA PE=1 SV=1	0.00	0.00	0.00	1.71	0.00	1.89	0.00	0.00	0.00	0.00	0.00	0.00	0.00	0.00	0.00	0.00	0.00	0.00	0.00	0.00	0.00	0.00	0.00	0.00	0.30	0.00	>0.999999999999999	#DIV/0!
P41240	Tyrosine-protein kinase CSK OS=Homo sapiens GN=CSK PE=1 SV=1	0.00	0.00	0.00	1.28	2.45	0.00	2.22	0.00	0.00	0.00	0.00	0.00	0.00	0.00	0.00	0.00	0.00	0.00	0.00	0.00	0.00	1.04	0.00	0.52	0.50	0.13	>0.999999999999999	3.8
P62899	60S ribosomal protein L31 OS=Homo sapiens GN=RPL31 PE=1 SV=1	0.00	0.00	0.00	11.33	4.53	5.67	4.10	0.00	0.00	4.45	3.77	1.96	0.00	0.00	5.72	0.00	0.00	0.00	0.00	0.00	0.00	1.92	7.52	0.00	2.98	1.26	>0.999999999999999	2.4
P49411	Elongation factor Tu, mitochondrial OS=Homo sapiens GN=TUFM PE=1 SV=2	0.00	0.00	0.00	1.50	0.00	0.00	0.00	0.00	0.00	0.00	2.20	1.14	0.00	0.00	0.00	0.00	0.00	0.00	1.27	0.00	0.00	0.00	0.99	0.28	0.40	0.21	>0.999999999999999	1.9
P62851	40S ribosomal protein S25 OS=Homo sapiens GN=RPS25 PE=1 SV=1	0.00	0.00	0.00	5.34	4.71	13.23	0.00	0.00	0.00	0.00	8.81	2.04	5.88	4.01	5.96	4.21	3.16	3.84	4.52	0.00	0.00	0.00	7.95	1.00	2.84	3.38	>0.999999999999999	0.8
Q8N8N7	Prostaglandin reductase 2 OS=Homo sapiens GN=PTGR2 PE=1 SV=1	0.00	0.00	0.00	3.85	0.00	0.00	1.37	0.72	0.00	0.00	2.83	0.65	0.00	1.28	1.91	0.00	0.00	1.23	0.00	0.00	0.00	0.64	2.55	0.32	0.79	0.66	>0.999999999999999	1.2
O60232	Sjogren syndrome/scleroderma autoantigen 1 OS=Homo sapiens GN=SSSCA1 PE=1 SV=1	0.00	0.00	0.00	3.21	0.00	0.00	0.00	0.00	0.00	0.00	0.00	1.22	0.00	0.00	0.00	0.00	0.00	0.00	0.00	0.00	0.00	0.00	2.13	0.60	0.37	0.23	>0.999999999999999	1.6
O00233	26S proteasome non-ATPase regulatory subunit 9 OS=Homo sapiens GN=PSMD9 PE=1 SV=3	0.00	0.00	0.00	6.20	2.45	3.07	2.22	1.17	0.00	2.41	4.56	0.00	0.00	0.00	6.92	2.19	0.00	0.00	0.00	0.00	0.00	1.04	4.12	0.52	1.84	1.23	>0.999999999999999	1.5
Q9NQ88	Probable fructose-2,6-bisphosphatase TIGAR OS=Homo sapiens GN=TIGAR PE=1 SV=1	0.00	0.00	0.00	2.35	0.00	0.00	0.00	0.00	0.00	0.00	1.73	0.00	0.00	0.00	0.00	0.00	0.00	0.00	0.00	0.00	0.00	0.88	0.00	0.00	0.34	0.07	>0.999999999999999	4.6
P55008	Allograft inflammatory factor 1 OS=Homo sapiens GN=AIF1 PE=1 SV=1	0.00	0.00	0.00	4.28	0.00	0.00	0.00	0.00	0.00	0.00	0.00	1.63	0.00	0.00	0.00	0.00	0.00	0.00	0.00	0.00	3.38	0.00	0.00	0.00	0.49	0.28	>0.999999999999999	1.7
Q15459	Splicing factor 3A subunit 1 OS=Homo sapiens GN=SF3A1 PE=1 SV=1	0.00	0.00	0.00	1.71	0.00	0.00	0.00	0.00	0.00	0.00	0.00	0.00	0.00	0.00	0.00	0.00	0.00	0.00	0.00	0.00	0.00	0.00	0.00	0.00	0.14	0.00	>0.999999999999999	#DIV/0!
Q9UMX0	Ubiquilin-1 OS=Homo sapiens GN=UBQLN1 PE=1 SV=2	0.00	0.00	0.00	1.07	0.00	0.00	0.85	0.00	0.00	0.00	0.79	0.00	0.00	0.00	0.00	0.00	0.00	0.00	0.00	0.00	0.00	0.00	0.00	0.00	0.23	0.00	>0.999999999999999	#DIV/0!
Q8WXX5	DnaJ homolog subfamily C member 9 OS=Homo sapiens GN=DNAJC9 PE=1 SV=1	0.00	0.00	0.00	4.92	2.07	0.00	0.00	0.00	0.00	0.00	0.00	0.00	0.00	0.00	0.00	0.00	0.00	0.00	0.00	0.00	0.00	0.00	0.00	0.00	0.58	0.00	>0.999999999999999	#DIV/0!
P62266	40S ribosomal protein S23 OS=Homo sapiens GN=RPS23 PE=1 SV=3	0.00	0.00	0.00	4.49	0.00	4.96	0.00	0.00	0.00	0.00	0.00	0.00	0.00	3.37	0.00	3.53	0.00	0.00	3.80	0.00	0.00	1.68	2.98	0.00	0.79	1.28	>0.999999999999999	0.6
Q9Y4X5	E3 ubiquitin-protein ligase ARIH1 OS=Homo sapiens GN=ARIH1 PE=1 SV=2	0.00	0.00	0.00	1.07	0.00	0.00	0.00	0.00	0.00	0.00	0.00	0.00	0.00	0.00	0.00	0.00	0.00	0.00	0.00	0.00	0.00	0.00	0.00	0.00	0.09	0.00	>0.999999999999999	#DIV/0!
P17900	Ganglioside GM2 activator OS=Homo sapiens GN=GM2A PE=1 SV=4	0.00	0.00	0.00	3.42	0.00	0.00	0.00	1.44	2.19	2.96	0.00	1.31	3.76	5.46	3.82	2.69	2.02	2.45	2.89	2.08	2.70	1.28	0.00	0.64	0.94	2.48	>0.999999999999999	0.4
P30046	D-dopachrome decarboxylase OS=Homo sapiens GN=DDT PE=1 SV=3	0.00	0.00	0.00	13.04	0.00	0.00	0.00	0.00	0.00	0.00	0.00	0.00	0.00	0.00	0.00	0.00	0.00	0.00	0.00	0.00	0.00	0.00	0.00	0.00	1.09	0.00	>0.999999999999999	#DIV/0!
P51148	Ras-related protein Rab-5C OS=Homo sapiens GN=RAB5C PE=1 SV=2	0.00	0.00	0.00	2.99	5.66	16.54	8.38	0.00	0.00	5.56	0.00	2.45	0.00	0.00	0.00	0.00	0.00	0.00	5.43	0.00	0.00	0.00	0.00	1.96	3.46	0.62	>0.999999999999999	5.6
Q9Y496	Kinesin-like protein KIF3A OS=Homo sapiens GN=KIF3A PE=1 SV=4	0.00	0.00	0.00	0.86	0.00	0.75	0.00	0.00	0.00	0.00	0.00	0.00	0.00	0.00	0.95	0.00	0.00	0.00	0.00	0.00	0.00	0.00	0.00	0.00	0.13	0.08	>0.999999999999999	1.7
Q96FQ6	Protein S100-A16 OS=Homo sapiens GN=S100A16 PE=1 SV=1	0.00	0.00	0.00	6.20	12.44	6.85	4.96	2.62	0.00	5.37	10.38	2.37	6.82	0.00	0.00	0.00	3.67	4.45	11.94	0.00	4.89	0.00	0.00	1.16	4.27	2.74	>0.999999999999999	1.6
P36980	Complement factor H-related protein 2 OS=Homo sapiens GN=CFHR2 PE=1 SV=1	0.00	0.00	0.00	4.70	0.00	0.00	5.98	0.00	0.00	0.00	0.00	1.79	0.00	3.53	0.00	3.70	2.78	0.00	0.00	0.00	0.00	1.56	0.00	0.00	1.04	0.96	>0.999999999999999	1.1
Q16864	V-type proton ATPase subunit F OS=Homo sapiens GN=ATP6V1F PE=1 SV=2	0.00	0.00	0.00	5.56	0.00	0.00	0.00	0.00	0.00	0.00	0.00	0.00	0.00	0.00	0.00	0.00	0.00	0.00	0.00	0.00	0.00	0.00	0.00	0.00	0.46	0.00	>0.999999999999999	#DIV/0!
P40616	ADP-ribosylation factor-like protein 1 OS=Homo sapiens GN=ARL1 PE=1 SV=1	0.00	0.00	0.00	3.42	0.00	0.00	0.00	0.00	0.00	0.00	0.00	0.00	0.00	0.00	0.00	0.00	0.00	0.00	0.00	0.00	0.00	0.00	0.00	0.00	0.29	0.00	>0.999999999999999	#DIV/0!
Q08AF3	Schlafen family member 5 OS=Homo sapiens GN=SLFN5 PE=1 SV=1	0.00	0.00	0.00	0.64	0.57	0.00	0.00	0.00	0.00	0.00	0.00	0.49	0.00	0.00	0.00	0.00	0.00	0.00	0.00	0.00	0.00	0.00	0.00	0.00	0.14	0.00	>0.999999999999999	#DIV/0!
Q9H9S4	Calcium-binding protein 39-like OS=Homo sapiens GN=CAB39L PE=1 SV=3	0.00	0.00	0.00	1.71	0.00	0.00	0.00	0.00	0.00	0.00	0.00	0.00	0.00	0.00	0.00	0.00	0.00	0.00	0.00	0.00	0.00	0.64	0.00	0.00	0.14	0.05	>0.999999999999999	2.7
P00918	Carbonic anhydrase 2 OS=Homo sapiens GN=CA2 PE=1 SV=2	0.00	0.00	0.00	2.35	4.53	0.00	1.88	0.00	3.29	0.00	0.00	0.90	0.00	0.00	9.06	1.85	3.04	3.68	1.99	1.43	1.86	1.92	1.56	0.44	1.08	2.24	>0.999999999999999	0.5
P15498	Proto-oncogene vav OS=Homo sapiens GN=VAV1 PE=1 SV=4	0.00	0.00	0.00	1.50	0.00	0.00	0.00	0.00	0.00	0.00	0.00	0.00	0.00	0.00	0.00	0.00	0.00	0.00	0.00	0.00	0.00	0.00	0.00	0.00	0.12	0.00	>0.999999999999999	#DIV/0!
Q9BQE5	Apolipoprotein L2 OS=Homo sapiens GN=APOL2 PE=1 SV=1	0.00	0.00	0.00	1.92	3.58	0.00	1.54	0.00	0.00	0.00	0.00	1.55	4.47	0.00	0.00	0.00	0.00	0.00	1.63	0.00	0.00	0.00	0.00	0.36	0.72	0.54	>0.999999999999999	1.3
Q8WYA6	Beta-catenin-like protein 1 OS=Homo sapiens GN=CTNNBL1 PE=1 SV=1	0.00	0.00	0.00	1.07	0.00	0.00	0.00	0.00	0.00	0.00	0.00	0.00	0.00	0.00	0.00	0.00	0.00	0.00	0.00	0.00	0.00	0.00	0.00	0.00	0.09	0.00	>0.999999999999999	#DIV/0!
Q8NB37	Parkinson disease 7 domain-containing protein 1 OS=Homo sapiens GN=PDDC1 PE=1 SV=1	0.00	0.00	0.00	2.99	0.00	0.00	0.00	0.00	0.00	0.00	0.00	0.00	0.00	0.00	0.00	0.00	0.00	0.00	0.00	0.00	0.00	0.00	0.00	0.00	0.25	0.00	>0.999999999999999	#DIV/0!
P30084	Enoyl-CoA hydratase, mitochondrial OS=Homo sapiens GN=ECHS1 PE=1 SV=4	0.00	0.00	0.00	2.14	0.00	0.00	0.00	0.00	0.00	0.00	1.57	6.61	0.00	0.00	0.00	0.00	0.00	0.00	0.00	0.00	0.00	0.00	0.00	0.88	0.86	0.07	>0.999999999999999	11.7
Q9Y450	HBS1-like protein OS=Homo sapiens GN=HBS1L PE=1 SV=1	0.00	0.00	0.00	0.86	0.00	0.00	0.00	0.00	0.00	0.00	0.63	0.00	0.00	0.00	0.00	0.00	0.00	0.00	0.00	0.00	0.68	0.00	0.00	0.00	0.12	0.06	>0.999999999999999	2.2
Q9BRG1	Vacuolar protein-sorting-associated protein 25 OS=Homo sapiens GN=VPS25 PE=1 SV=1	0.00	0.00	0.00	3.42	0.00	0.00	0.00	0.00	0.00	0.00	0.00	0.00	0.00	0.00	0.00	0.00	0.00	0.00	0.00	0.00	0.00	0.00	0.00	0.00	0.29	0.00	>0.999999999999999	#DIV/0!
Q00765	Receptor expression-enhancing protein 5 OS=Homo sapiens GN=REEP5 PE=1 SV=3	0.00	0.00	0.00	3.21	0.00	0.00	0.00	0.00	0.00	0.00	2.36	1.22	0.00	0.00	0.00	0.00	0.00	0.00	0.00	0.00	0.00	0.00	2.13	0.00	0.57	0.18	>0.999999999999999	3.2
Q9H7X7	Rab-like protein 5 OS=Homo sapiens GN=RABL5 PE=2 SV=1	0.00	0.00	0.00	3.42	0.00	8.03	0.00	0.00	0.00	2.96	2.52	0.00	0.00	0.00	0.00	0.00	0.00	0.00	0.00	0.00	0.00	0.00	0.00	0.00	1.41	0.00	>0.999999999999999	#DIV/0!
O00743	Serine/threonine-protein phosphatase 6 catalytic subunit OS=Homo sapiens GN=PPP6C PE=1 SV=1	0.00	0.00	0.00	1.92	0.00	0.00	0.00	0.00	0.00	0.00	0.00	0.00	0.00	0.00	2.15	1.51	0.00	0.00	0.00	0.00	0.00	0.00	1.28	0.00	0.16	0.41	>0.999999999999999	0.4
Q9H1Z4	WD repeat-containing protein 13 OS=Homo sapiens GN=WDR13 PE=1 SV=2	0.00	0.00	0.00	1.28	0.00	0.00	0.00	0.00	0.00	0.00	0.00	0.00	0.00	0.00	0.00	0.00	0.00	0.00	0.00	0.00	0.00	0.00	0.00	0.00	0.11	0.00	>0.999999999999999	#DIV/0!
A6NIH7	Protein unc-119 homolog B OS=Homo sapiens GN=UNC119B PE=1 SV=1	0.00	0.00	0.00	2.57	0.00	0.00	0.00	0.00	0.00	0.00	0.00	0.00	0.00	0.00	0.00	0.00	0.00	0.00	0.00	0.00	0.00	0.00	0.00	0.00	0.21	0.00	>0.999999999999999	#DIV/0!
Q5VSP4	Putative lipocalin 1-like protein 1 OS=Homo sapiens GN=LCN1P1 PE=5 SV=1	0.00	0.00	0.00	4.06	0.00	0.00	0.00	1.72	2.60	0.00	0.00	0.00	0.00	0.00	0.00	0.00	0.00	0.00	0.00	0.00	0.00	0.00	0.00	0.00	0.70	0.00	>0.999999999999999	#DIV/0!
Q86UE4	Protein LYRIC OS=Homo sapiens GN=MTDH PE=1 SV=2	0.00	0.00	0.00	1.07	0.00	0.00	0.00	0.00	0.00	0.00	0.00	0.00	0.00	0.00	0.00	0.84	0.00	0.00	0.00	0.00	0.00	0.00	0.00	0.00	0.09	0.07	>0.999999999999999	1.3
O60502	Bifunctional protein NCOAT OS=Homo sapiens GN=MGEA5 PE=1 SV=2	0.00	0.00	0.00	0.64	0.00	0.71	0.00	0.00	0.00	0.00	0.47	0.24	0.00	0.00	0.72	0.00	0.00	0.46	0.00	0.00	0.00	0.00	0.00	0.00	0.17	0.10	>0.999999999999999	1.8
Q96T51	RUN and FYVE domain-containing protein 1 OS=Homo sapiens GN=RUFY1 PE=1 SV=2	0.00	0.00	0.00	0.86	0.00	0.00	0.00	0.00	0.00	0.00	0.00	0.00	0.00	0.00	0.00	0.00	0.00	0.00	0.00	0.00	0.00	0.00	0.00	0.00	0.07	0.00	>0.999999999999999	#DIV/0!
P09497	Clathrin light chain B OS=Homo sapiens GN=CLTB PE=1 SV=1	0.00	0.00	0.00	2.78	0.00	0.00	0.00	0.00	0.00	0.00	0.00	0.00	0.00	0.00	3.10	0.00	0.00	0.00	0.00	0.00	0.00	1.04	0.00	0.00	0.23	0.34	>0.999999999999999	0.7
P62993	Growth factor receptor-bound protein 2 OS=Homo sapiens GN=GRB2 PE=1 SV=1	0.00	0.00	0.00	5.99	2.45	0.00	0.00	0.00	0.00	0.00	0.00	0.00	0.00	0.00	0.00	0.00	0.00	0.00	0.00	0.00	2.19	0.00	0.00	0.00	0.70	0.18	>0.999999999999999	3.8
Q6TDU7	Cancer susceptibility candidate protein 1 OS=Homo sapiens GN=CASC1 PE=2 SV=2	0.00	0.00	0.00	0.86	0.00	0.00	0.00	0.00	0.00	0.00	0.00	0.00	0.00	0.00	0.00	0.00	0.00	0.00	0.00	0.00	0.00	0.00	0.00	0.00	0.07	0.00	>0.999999999999999	#DIV/0!
Q6IN85	Serine/threonine-protein phosphatase 4 regulatory subunit 3A OS=Homo sapiens GN=SMEK1 PE=1 SV=1	0.00	0.00	0.00	0.64	0.00	0.00	0.00	0.00	0.00	0.00	0.00	0.24	0.00	0.00	0.00	0.00	0.00	0.00	0.00	0.00	0.00	0.00	0.00	0.00	0.07	0.00	>0.999999999999999	#DIV/0!
Q9Y608	Leucine-rich repeat flightless-interacting protein 2 OS=Homo sapiens GN=LRRFIP2 PE=1 SV=1	0.00	0.00	0.00	0.86	0.00	0.95	0.00	0.00	0.00	0.00	0.00	0.00	0.00	0.00	0.95	0.00	0.00	0.00	0.00	0.00	0.00	0.00	0.57	0.00	0.15	0.13	>0.999999999999999	1.2
Q9NRF8	CTP synthase 2 OS=Homo sapiens GN=CTPS2 PE=1 SV=1	0.00	0.00	0.00	1.07	0.00	0.00	0.00	0.00	0.00	0.00	0.00	0.00	0.00	0.00	1.19	0.00	0.00	0.00	0.00	0.00	0.00	0.00	0.00	0.00	0.09	0.10	>0.999999999999999	0.9
Q15084	Protein disulfide-isomerase A6 OS=Homo sapiens GN=PDIA6 PE=1 SV=1	0.00	0.00	0.00	1.50	0.00	3.31	0.00	0.63	1.92	0.00	3.46	3.18	0.00	0.00	0.00	0.00	0.00	1.07	1.27	0.91	1.18	0.56	0.99	0.56	1.17	0.55	>0.999999999999999	2.1
P49903	Selenide, water dikinase 1 OS=Homo sapiens GN=SEPHS1 PE=1 SV=2	0.00	0.00	0.00	1.71	0.00	0.00	0.00	0.00	0.00	0.00	0.00	0.00	0.00	0.00	0.00	0.00	0.00	0.00	0.00	0.00	0.00	0.00	0.00	0.00	0.14	0.00	>0.999999999999999	#DIV/0!
Q9Y6Q5	AP-1 complex subunit mu-2 OS=Homo sapiens GN=AP1M2 PE=1 SV=4	0.00	0.00	0.00	2.99	0.00	5.20	2.39	0.00	0.00	0.00	6.13	0.57	0.00	0.00	3.34	0.00	0.00	0.00	0.00	0.00	0.00	0.00	3.12	0.00	1.44	0.54	>0.999999999999999	2.7
O95218	Zinc finger Ran-binding domain-containing protein 2 OS=Homo sapiens GN=ZRANB2 PE=1 SV=2	0.00	0.00	0.00	1.92	0.00	2.13	0.00	0.00	0.00	0.00	0.00	0.00	0.00	0.00	0.00	0.00	0.00	0.00	0.00	0.00	0.00	0.00	0.00	0.00	0.34	0.00	>0.999999999999999	#DIV/0!
Q9P2R3	Ankyrin repeat and FYVE domain-containing protein 1 OS=Homo sapiens GN=ANKFY1 PE=1 SV=2	0.00	0.00	0.00	0.64	0.57	0.00	0.00	0.00	0.00	0.00	0.47	0.00	0.00	0.00	0.00	0.00	0.00	0.00	0.00	0.00	0.00	0.00	0.00	0.12	0.14	0.01	>0.999999999999999	14.0
Q9BRQ4	Uncharacterized protein C11orf70 OS=Homo sapiens GN=C11orf70 PE=2 SV=3	0.00	0.00	0.00	2.35	0.00	0.00	0.00	0.00	0.00	0.00	0.00	0.00	0.00	0.00	0.00	0.00	0.00	0.00	0.00	0.00	0.00	0.88	0.00	0.00	0.20	0.07	>0.999999999999999	2.7
Q9NV70	Exocyst complex component 1 OS=Homo sapiens GN=EXOC1 PE=1 SV=4	0.00	0.00	0.00	0.64	0.00	0.00	0.00	0.00	0.00	0.00	0.00	0.00	0.00	0.00	0.00	0.00	0.00	0.00	0.00	0.00	0.00	0.00	0.00	0.00	0.05	0.00	>0.999999999999999	#DIV/0!
Q96H20	Vacuolar-sorting protein SNF8 OS=Homo sapiens GN=SNF8 PE=1 SV=1	0.00	0.00	0.00	2.35	0.00	0.00	0.00	0.00	0.00	0.00	0.00	0.00	0.00	0.00	0.00	0.00	0.00	0.00	0.00	0.00	0.00	0.88	0.00	0.00	0.20	0.07	>0.999999999999999	2.7
P01815	Ig heavy chain V-II region COR OS=Homo sapiens PE=1 SV=1	0.00	0.00	0.00	5.56	0.00	0.00	0.00	0.00	0.00	0.00	0.00	0.00	0.00	0.00	6.20	0.00	0.00	0.00	0.00	0.00	0.00	0.00	0.00	0.00	0.46	0.52	>0.999999999999999	0.9
Q96MW5	Conserved oligomeric Golgi complex subunit 8 OS=Homo sapiens GN=COG8 PE=1 SV=2	0.00	0.00	0.00	1.07	0.00	0.00	0.00	0.00	0.00	0.00	0.00	0.00	0.00	0.00	0.00	0.00	0.00	0.00	0.00	0.00	0.00	0.00	0.00	0.00	0.09	0.00	>0.999999999999999	#DIV/0!
O94855	Protein transport protein Sec24D OS=Homo sapiens GN=SEC24D PE=1 SV=2	0.00	0.00	0.00	1.28	0.00	0.71	0.00	0.27	0.00	0.00	0.94	0.24	1.41	0.48	0.00	0.00	0.00	0.00	0.00	0.00	0.00	0.00	1.28	0.24	0.29	0.28	>0.999999999999999	1.0
Q9P253	Vacuolar protein sorting-associated protein 18 homolog OS=Homo sapiens GN=VPS18 PE=1 SV=2	0.00	0.00	0.00	0.64	0.00	0.00	0.00	0.00	0.00	0.00	0.00	0.00	0.00	0.00	0.00	0.00	0.00	0.00	0.00	0.00	0.00	0.00	0.00	0.00	0.05	0.00	>0.999999999999999	#DIV/0!
P20700	Lamin-B1 OS=Homo sapiens GN=LMNB1 PE=1 SV=2	0.00	0.00	0.00	1.07	0.00	0.00	0.00	0.00	0.00	0.00	0.00	0.00	0.00	0.00	0.00	0.00	0.00	0.00	0.00	2.73	2.70	0.00	0.00	0.00	0.09	0.45	>0.999999999999999	0.2
Q9P016	Thymocyte nuclear protein 1 OS=Homo sapiens GN=THYN1 PE=1 SV=1	0.00	0.00	0.00	2.78	0.00	6.38	0.00	0.00	0.00	0.00	2.04	0.00	0.00	0.00	0.00	0.00	0.00	0.00	0.00	0.00	0.00	0.00	0.00	0.00	0.93	0.00	>0.999999999999999	#DIV/0!
O75935	Dynactin subunit 3 OS=Homo sapiens GN=DCTN3 PE=1 SV=1	0.00	0.00	0.00	3.42	0.00	0.00	0.00	0.00	0.00	0.00	2.52	0.00	0.00	0.00	0.00	0.00	0.00	0.00	0.00	0.00	0.00	0.00	0.00	0.00	0.49	0.00	>0.999999999999999	#DIV/0!
Q9Y5L0	Transportin-3 OS=Homo sapiens GN=TNPO3 PE=1 SV=3	0.00	0.00	0.00	0.64	0.00	0.71	0.00	0.00	0.00	0.00	0.00	0.00	0.00	0.00	0.00	0.00	0.00	0.00	0.00	0.00	0.00	0.00	0.00	0.00	0.11	0.00	>0.999999999999999	#DIV/0!
Q9H2X0	Chordin OS=Homo sapiens GN=CHRD PE=1 SV=2	0.00	0.00	0.00	0.64	0.00	0.00	0.00	0.00	0.00	0.00	0.00	0.24	0.00	0.00	0.00	0.00	0.00	0.00	0.00	0.00	0.00	0.00	0.00	0.12	0.07	0.01	>0.999999999999999	7.4
Q9UPE1	SRSF protein kinase 3 OS=Homo sapiens GN=SRPK3 PE=2 SV=2	0.00	0.00	0.00	1.07	0.00	0.00	0.00	0.00	0.00	0.00	0.00	0.00	0.00	0.00	0.00	0.00	0.00	0.00	0.00	0.00	0.00	0.00	0.71	0.20	0.09	0.08	>0.999999999999999	1.2
Q9NV35	Probable 8-oxo-dGTP diphosphatase NUDT15 OS=Homo sapiens GN=NUDT15 PE=1 SV=1	0.00	0.00	0.00	3.85	0.00	0.00	0.00	0.00	0.00	0.00	0.00	0.00	0.00	0.00	0.00	0.00	0.00	0.00	0.00	0.00	0.00	0.00	0.00	0.00	0.32	0.00	>0.999999999999999	#DIV/0!
P19367	Hexokinase-1 OS=Homo sapiens GN=HK1 PE=1 SV=3	0.00	0.00	0.00	1.28	0.00	0.00	0.00	0.00	0.00	0.00	0.00	1.06	0.00	0.00	0.00	0.00	0.00	0.00	0.00	0.00	0.00	0.24	0.00	0.12	0.20	0.03	>0.999999999999999	6.5
O94832	Unconventional myosin-Id OS=Homo sapiens GN=MYO1D PE=1 SV=2	0.00	0.00	0.00	0.64	0.57	0.71	0.00	0.00	0.00	0.00	0.00	0.24	0.00	0.00	0.00	0.00	0.00	0.00	0.00	0.00	0.00	0.24	0.00	0.12	0.18	0.03	>0.999999999999999	6.0

Q8NGK0	Olfactory receptor 51G2 OS=Homo sapiens GN=OR51G2 PE=2 SV=1	0.00	0.00	0.00	1.92	0.00	0.00	0.00	0.00	0.00	0.00	0.00	0.00	0.00	0.00	0.00	0.00	0.00	0.00	0.00	0.00	0.00	0.00	0.00	0.00	0.16	0.00	>0.999999999999999	#DIV/0!
Q96I15	Selenocysteine lyase OS=Homo sapiens GN=SCLY PE=1 SV=2	0.00	0.00	0.00	1.50	0.00	0.00	0.00	0.00	0.00	0.00	1.10	0.00	0.00	0.00	0.00	0.00	0.00	0.00	0.00	0.00	0.00	0.00	0.00	0.00	0.22	0.00	>0.999999999999999	#DIV/0!
Q96CW1	AP-2 complex subunit mu OS=Homo sapiens GN=AP2M1 PE=1 SV=2	0.00	0.00	0.00	1.50	0.00	0.00	0.00	0.00	0.00	0.00	2.20	0.00	0.00	0.00	0.00	0.00	0.00	0.00	0.00	0.00	0.00	0.00	0.00	0.00	0.31	0.00	>0.999999999999999	#DIV/0!
Q7RTV0	PHD finger-like domain-containing protein 5A OS=Homo sapiens GN=PHF5A PE=1 SV=1	0.00	0.00	0.00	5.56	0.00	0.00	0.00	0.00	0.00	0.00	4.09	0.00	0.00	0.00	0.00	0.00	0.00	0.00	0.00	0.00	0.00	0.00	0.00	0.00	0.80	0.00	>0.999999999999999	#DIV/0!
Q96RP9	Elongation factor G, mitochondrial OS=Homo sapiens GN=GFM1 PE=1 SV=2	0.00	0.00	0.00	0.86	0.00	0.00	0.00	0.00	0.00	0.00	0.00	0.00	0.00	0.00	0.00	0.00	0.00	0.00	0.00	0.00	0.00	0.00	0.00	0.00	0.07	0.00	>0.999999999999999	#DIV/0!
P27361	Mitogen-activated protein kinase 3 OS=Homo sapiens GN=MAPK3 PE=1 SV=4	0.00	0.00	0.00	1.71	0.00	0.00	0.00	0.00	0.00	0.00	1.26	0.00	0.00	0.00	0.00	0.00	0.00	0.00	0.00	0.00	0.00	0.00	0.00	0.00	0.25	0.00	>0.999999999999999	#DIV/0!
Q53T59	HCLS1-binding protein 3 OS=Homo sapiens GN=HS1BP3 PE=1 SV=1	0.00	0.00	0.00	1.71	0.00	0.00	0.00	0.00	0.00	0.00	0.00	0.00	0.00	0.00	0.00	0.00	0.00	0.00	0.00	0.00	0.00	0.00	0.00	0.00	0.14	0.00	>0.999999999999999	#DIV/0!
O94817	Ubiquitin-like protein ATG12 OS=Homo sapiens GN=ATG12 PE=1 SV=1	0.00	0.00	0.00	4.70	4.15	0.00	0.00	1.99	3.01	0.00	0.00	0.00	0.00	0.00	0.00	0.00	0.00	0.00	0.00	0.00	0.00	0.00	3.12	0.00	1.15	0.26	>0.999999999999999	4.4
Q9UNE7	E3 ubiquitin-protein ligase CHIP OS=Homo sapiens GN=STUB1 PE=1 SV=2	0.00	0.00	0.00	1.92	0.00	0.00	0.00	0.00	0.00	0.00	0.00	0.00	0.00	0.00	0.00	0.00	0.00	0.00	0.00	0.00	0.00	0.00	0.00	0.00	0.16	0.00	>0.999999999999999	#DIV/0!
P16455	Methylated-DNA--protein-cysteine methyltransferase OS=Homo sapiens GN=MGMT PE=1 SV=1	0.00	0.00	0.00	3.21	0.00	0.00	0.00	0.00	0.00	0.00	2.36	0.00	0.00	0.00	0.00	0.00	0.00	0.00	0.00	0.00	0.00	0.00	0.00	0.00	0.46	0.00	>0.999999999999999	#DIV/0!
O43414	ERI1 exoribonuclease 3 OS=Homo sapiens GN=ERI3 PE=1 SV=2	0.00	0.00	0.00	1.92	0.00	2.13	0.00	0.00	0.00	0.00	0.00	0.00	0.00	0.00	0.00	0.00	0.00	0.00	0.00	0.00	0.00	0.00	0.00	0.00	0.34	0.00	>0.999999999999999	#DIV/0!
Q7Z3J2	UPF0505 protein C16orf62 OS=Homo sapiens GN=C16orf62 PE=1 SV=2	0.00	0.00	0.00	0.64	0.00	0.00	0.00	0.00	0.00	0.00	0.00	0.24	0.00	0.00	0.00	0.00	0.00	0.00	0.00	0.00	0.00	0.00	0.00	0.00	0.07	0.00	>0.999999999999999	#DIV/0!
Q9H6T3	RNA polymerase II-associated protein 3 OS=Homo sapiens GN=RPAP3 PE=1 SV=2	0.00	0.00	0.00	0.86	0.00	0.95	0.00	0.00	0.00	0.00	0.00	0.00	0.00	0.00	0.00	0.00	0.00	0.00	0.00	0.00	0.00	0.00	0.00	0.00	0.15	0.00	>0.999999999999999	#DIV/0!
P09234	U1 small nuclear ribonucleoprotein C OS=Homo sapiens GN=SNRPC PE=1 SV=1	0.00	0.00	0.00	4.06	0.00	4.49	0.00	0.00	0.00	0.00	2.99	0.00	0.00	0.00	0.00	0.00	0.00	0.00	0.00	0.00	0.00	0.00	0.00	0.00	0.96	0.00	>0.999999999999999	#DIV/0!
P04626	Receptor tyrosine-protein kinase erbB-2 OS=Homo sapiens GN=ERBB2 PE=1 SV=1	0.00	0.00	0.00	0.43	0.00	0.47	0.34	0.00	0.00	0.00	0.00	0.00	0.00	0.00	0.48	0.00	0.00	0.00	0.00	0.00	0.00	0.16	0.00	0.00	0.10	0.05	>0.999999999999999	2.0
O00418	Eukaryotic elongation factor 2 kinase OS=Homo sapiens GN=EEF2K PE=1 SV=2	0.00	0.00	0.00	0.86	0.00	0.00	0.00	0.00	0.00	0.00	0.00	0.00	0.00	0.00	0.00	0.00	0.00	0.00	0.00	0.00	0.00	0.00	0.00	0.00	0.07	0.00	>0.999999999999999	#DIV/0!
Q8N9Z9	Intermediate filament tail domain-containing protein 1 OS=Homo sapiens GN=IFLTD1 PE=2 SV=2	0.00	0.00	0.00	1.71	0.00	0.00	0.00	0.00	0.00	0.00	0.00	0.00	0.00	0.00	0.00	0.00	0.00	0.00	0.00	0.00	0.00	0.00	0.00	0.00	0.14	0.00	>0.999999999999999	#DIV/0!
Q14244	Ensconsin OS=Homo sapiens GN=MAP7 PE=1 SV=1	0.00	0.00	0.00	0.86	0.75	0.00	0.00	0.00	0.55	0.74	0.63	0.65	0.00	0.00	0.00	0.00	0.00	0.00	0.00	0.00	0.00	0.00	0.00	0.00	0.35	0.00	>0.999999999999999	#DIV/0!
P02656	Apolipoprotein C-III OS=Homo sapiens GN=APOC3 PE=1 SV=1	0.00	0.00	0.00	6.84	13.95	0.00	12.65	6.68	4.38	0.00	0.00	2.61	7.53	0.00	0.00	12.45	9.36	0.00	5.79	16.75	12.49	5.91	0.00	0.00	3.93	5.86	>0.999999999999999	0.7
P23743	Diacylglycerol kinase alpha OS=Homo sapiens GN=DGKA PE=1 SV=3	0.00	0.00	0.00	0.86	0.00	0.00	0.00	0.00	0.00	0.00	0.00	0.33	0.00	0.00	0.00	0.00	0.00	0.00	0.00	0.00	0.00	0.00	0.00	0.00	0.10	0.00	>0.999999999999999	#DIV/0!
O14896	Interferon regulatory factor 6 OS=Homo sapiens GN=IRF6 PE=1 SV=1	0.00	0.00	0.00	1.28	1.13	3.07	1.03	0.00	0.00	0.00	0.94	0.00	0.00	0.00	1.43	0.00	0.00	0.00	0.00	0.00	0.00	0.00	0.85	0.00	0.62	0.19	>0.999999999999999	3.3
Q96CD0	F-box/LRR-repeat protein 8 OS=Homo sapiens GN=FBXL8 PE=1 SV=1	0.00	0.00	0.00	1.71	3.21	1.89	0.00	0.00	0.00	0.00	1.26	0.00	0.00	1.28	0.00	0.00	0.00	0.00	0.00	0.00	0.00	0.00	1.14	0.00	0.67	0.20	>0.999999999999999	3.3
Q9H2F3	3 beta-hydroxysteroid dehydrogenase type 7 OS=Homo sapiens GN=HSD3B7 PE=1 SV=2	0.00	0.00	0.00	1.71	1.51	1.89	0.00	0.00	0.00	0.00	1.26	0.00	0.00	0.00	0.00	0.00	0.00	0.00	0.00	0.00	0.00	0.00	1.14	0.00	0.53	0.09	>0.999999999999999	5.6
P53609	Geranylgeranyl transferase type-1 subunit beta OS=Homo sapiens GN=PGGT1B PE=1 SV=2	0.00	0.00	0.00	1.71	0.00	0.00	0.00	0.00	0.00	0.00	0.00	0.00	0.00	0.00	0.00	0.00	0.00	0.00	0.00	0.00	0.00	0.00	0.00	0.00	0.14	0.00	>0.999999999999999	#DIV/0!
Q6NUN9	Zinc finger protein 746 OS=Homo sapiens GN=ZNF746 PE=1 SV=1	0.00	0.00	0.00	1.07	0.00	0.00	0.00	0.00	0.00	0.00	0.00	0.00	0.00	0.00	0.00	0.00	0.00	0.00	0.00	0.00	0.00	0.00	0.00	0.00	0.09	0.00	>0.999999999999999	#DIV/0!
O15514	DNA-directed RNA polymerase II subunit RPB4 OS=Homo sapiens GN=POLR2D PE=1 SV=1	0.00	0.00	0.00	4.49	0.00	0.00	0.00	0.00	0.00	0.00	0.00	0.00	0.00	0.00	0.00	0.00	0.00	0.00	0.00	0.00	0.00	0.00	0.00	0.00	0.37	0.00	>0.999999999999999	#DIV/0!
Q8TD06	Anterior gradient protein 3 homolog OS=Homo sapiens GN=AGR3 PE=1 SV=1	0.00	0.00	0.00	3.63	0.00	0.00	0.00	0.00	0.00	0.00	0.00	0.00	0.00	0.00	0.00	0.00	0.00	0.00	0.00	0.00	0.00	0.00	0.00	0.00	0.30	0.00	>0.999999999999999	#DIV/0!
P61970	Nuclear transport factor 2 OS=Homo sapiens GN=NUTF2 PE=1 SV=1	0.00	0.00	0.00	4.92	0.00	0.00	0.00	0.00	0.00	0.00	0.00	0.00	0.00	0.00	0.00	3.87	0.00	3.53	0.00	0.00	0.00	1.84	0.00	0.92	0.41	0.85	>0.999999999999999	0.5
Q9UK22	F-box only protein 2 OS=Homo sapiens GN=FBXO2 PE=1 SV=2	0.00	0.00	0.00	2.14	0.00	2.36	0.00	0.00	0.00	0.00	0.00	0.00	0.00	0.00	0.00	0.00	0.00	0.00	0.00	0.00	0.00	0.00	0.00	0.00	0.38	0.00	>0.999999999999999	#DIV/0!
Q15020	Squamous cell carcinoma antigen recognized by T-cells 3 OS=Homo sapiens GN=SART3 PE=1 SV=1	0.00	0.00	0.00	0.64	0.00	0.00	0.00	0.00	0.00	0.00	0.00	0.00	0.00	0.00	0.00	0.00	0.00	0.00	0.00	0.00	0.00	0.00	0.00	0.00	0.05	0.00	>0.999999999999999	#DIV/0!
Q96S53	Dual specificity testis-specific protein kinase 2 OS=Homo sapiens GN=TESK2 PE=2 SV=1	0.00	0.00	0.00	1.07	0.00	0.00	0.00	0.00	0.00	0.00	0.00	0.00	0.00	0.00	0.00	0.00	0.00	0.00	0.00	0.00	0.00	0.00	0.00	0.00	0.09	0.00	>0.999999999999999	#DIV/0!
O75340	Programmed cell death protein 6 OS=Homo sapiens GN=PDCD6 PE=1 SV=1	0.00	0.00	0.00	3.21	0.00	0.00	0.00	0.00	0.00	0.00	0.00	0.00	0.00	0.00	0.00	0.00	0.00	0.00	0.00	0.00	0.00	0.00	0.00	0.00	0.27	0.00	>0.999999999999999	#DIV/0!
Q9H1R3	Myosin light chain kinase 2, skeletal/cardiac muscle OS=Homo sapiens GN=MYLK2 PE=1 SV=3	0.00	0.00	0.00	1.07	0.00	0.00	0.85	0.00	0.00	0.00	0.00	0.00	0.00	0.00	0.00	0.00	0.00	0.00	0.00	0.00	0.00	0.00	0.00	0.00	0.16	0.00	>0.999999999999999	#DIV/0!
P49756	RNA-binding protein 25 OS=Homo sapiens GN=RBM25 PE=1 SV=3	0.00	0.00	0.00	0.64	0.00	0.00	0.00	0.00	0.00	0.00	0.00	0.00	0.00	0.00	0.00	0.00	0.00	0.00	0.00	0.00	0.00	0.00	0.00	0.00	0.05	0.00	>0.999999999999999	#DIV/0!
P62805	Histone H4 OS=Homo sapiens GN=HIST1H4A PE=1 SV=2	0.00	0.00	0.00	6.41	13.20	0.00	5.13	0.00	0.00	0.00	0.00	0.00	0.00	4.82	0.00	5.05	8.86	4.60	0.00	15.84	0.00	0.00	4.26	0.00	2.06	3.62	>0.999999999999999	0.6
Q71UM5	40S ribosomal protein S27-like OS=Homo sapiens GN=RPS27L PE=1 SV=3	0.00	0.00	0.00	7.70	6.79	0.00	0.00	0.00	0.00	0.00	5.66	0.00	8.47	0.00	0.00	0.00	0.00	0.00	0.00	0.00	0.00	0.00	5.11	1.44	1.68	1.25	>0.999999999999999	1.3
Q9NZC9	SWI/SNF-related matrix-associated actin-dependent regulator of chromatin subfamily A-like protein 1 OS=Homo sap	0.00	0.00	0.00	0.64	0.00	0.00	0.00	0.00	0.00	0.00	0.00	0.00	0.00	0.00	0.00	0.00	0.00	0.00	0.00	0.00	0.00	0.00	0.00	0.00	0.05	0.00	>0.999999999999999	#DIV/0!
P14678	Small nuclear ribonucleoprotein-associated proteins B and B' OS=Homo sapiens GN=SNRPB PE=1 SV=2	0.00	0.00	0.00	2.99	0.00	3.31	0.00	0.00	0.00	0.00	2.20	0.00	0.00	0.00	0.00	0.00	0.00	0.00	0.00	0.00	0.00	0.00	0.00	0.00	0.71	0.00	>0.999999999999999	#DIV/0!
Q9HC24	Protein lifeguard 4 OS=Homo sapiens GN=TMBIM4 PE=1 SV=3	0.00	0.00	0.00	2.78	2.45	3.07	0.00	0.00	0.00	0.00	2.04	0.00	3.06	0.00	3.10	2.19	0.00	0.00	0.00	0.00	0.00	1.84	0.00	0.00	0.86	0.85	>0.999999999999999	1.0
Q9NXV6	CDKN2A-interacting protein OS=Homo sapiens GN=CDKN2AIP PE=1 SV=3	0.00	0.00	0.00	1.07	0.00	0.00	0.00	0.00	0.00	0.00	0.00	0.00	0.00	0.00	0.00	0.00	0.00	0.00	0.00	0.00	0.00	0.00	0.00	0.00	0.09	0.00	>0.999999999999999	#DIV/0!
P30048	Thioredoxin-dependent peroxide reductase, mitochondrial OS=Homo sapiens GN=PRDX3 PE=1 SV=3	0.00	0.00	0.00	2.57	0.00	0.00	0.00	0.00	0.00	0.00	0.00	3.26	0.00	0.00	0.00	4.21	0.00	0.00	0.00	0.00	0.00	0.00	1.70	0.00	0.49	0.49	>0.999999999999999	1.0
Q14192	Four and a half LIM domains protein 2 OS=Homo sapiens GN=FHL2 PE=1 SV=3	0.00	0.00	0.00	2.14	0.00	2.36	0.00	0.00	0.00	0.00	0.00	0.00	0.00	0.00	0.00	0.00	0.00	0.00	0.00	0.00	0.00	0.00	0.00	0.00	0.38	0.00	>0.999999999999999	#DIV/0!
Q8IX04	Ubiquitin-conjugating enzyme E2 variant 3 OS=Homo sapiens GN=UEVLD PE=1 SV=2	0.00	0.00	0.00	1.28	0.00	0.00	0.00	0.00	0.00	1.11	2.04	0.00	0.00	0.00	0.00	0.00	0.00	0.00	0.00	0.00	0.00	0.00	0.00	0.00	0.37	0.00	>0.999999999999999	#DIV/0!
Q99747	Gamma-soluble NSF attachment protein OS=Homo sapiens GN=NAPG PE=1 SV=1	0.00	0.00	0.00	1.92	0.00	0.00	0.00	0.00	0.00	0.00	1.42	0.00	0.00	0.00	2.15	0.00	0.00	0.00	1.63	0.00	0.00	0.00	1.28	0.00	0.28	0.42	>0.999999999999999	0.7
Q8N163	Protein KIAA1967 OS=Homo sapiens GN=KIAA1967 PE=1 SV=2	0.00	0.00	0.00	0.64	0.00	2.36	0.00	0.27	0.00	1.11	1.57	0.00	0.00	0.00	0.00	0.00	0.00	0.00	0.00	0.00	0.00	0.00	0.00	0.00	0.50	0.00	>0.999999999999999	#DIV/0!
Q9Y5A7	NEDD8 ultimate buster 1 OS=Homo sapiens GN=NUB1 PE=1 SV=2	0.00	0.00	0.00	1.07	0.00	0.00	0.00	0.00	0.00	0.00	0.00	0.00	0.00	0.00	0.00	0.00	0.00	0.00	0.00	0.00	0.00	0.00	0.00	0.00	0.09	0.00	>0.999999999999999	#DIV/0!
P47895	Aldehyde dehydrogenase family 1 member A3 OS=Homo sapiens GN=ALDH1A3 PE=1 SV=2	0.00	0.00	0.00	0.00	3.39	0.00	0.00	0.00	0.00	0.00	2.83	2.04	0.00	0.00	5.96	0.00	0.00	0.00	4.52	0.00	0.00	0.00	6.81	0.72	0.69	1.50	>0.999999999999999	0.5
P20742	Pregnancy zone protein OS=Homo sapiens GN=PZP PE=1 SV=4	0.00	0.00	0.00	0.00	2.83	0.00	5.47	0.00	2.33	0.00	0.00	0.00	0.00	0.00	0.00	0.00	0.00	3.04	0.00	5.25	0.00	3.71	0.00	0.00	0.89	1.00	>0.999999999999999	0.9
P07205	Phosphoglycerate kinase 2 OS=Homo sapiens GN=PGK2 PE=1 SV=3	0.00	0.00	0.00	0.00	6.22	7.80	0.00	0.00	0.00	0.00	5.19	0.00	0.00	0.00	0.00	0.00	0.00	0.00	0.00	0.00	0.00	0.00	0.00	0.00	1.60	0.00	>0.999999999999999	#DIV/0!
P07864	L-lactate dehydrogenase C chain OS=Homo sapiens GN=LDHC PE=2 SV=4	0.00	0.00	0.00	0.00	3.58	0.00	0.00	0.00	0.00	0.00	0.00	0.00	0.00	0.00	0.00	0.00	0.00	0.00	0.00	0.00	0.00	0.00	0.00	0.00	0.30	0.00	>0.999999999999999	#DIV/0!
P19013	Keratin, type II cytoskeletal 4 OS=Homo sapiens GN=KRT4 PE=1 SV=4	0.00	0.00	0.00	0.00	3.39	0.00	0.00	0.00	0.00	0.00	0.00	0.00	0.00	0.00	0.00	1.43	3.03	1.52	0.00	3.26	0.00	0.00	0.00	0.00	0.28	0.77	>0.999999999999999	0.4
Q13151	Heterogeneous nuclear ribonucleoprotein A0 OS=Homo sapiens GN=HNRNPA0 PE=1 SV=1	0.00	0.00	0.00	0.00	4.34	0.00	3.93	0.00	0.00	0.00	0.00	0.00	0.00	1.77	0.00	0.00	0.00	0.00	0.00	0.00	0.00	0.00	0.00	0.00	0.69	0.15	>0.999999999999999	4.7
P84085	ADP-ribosylation factor 5 OS=Homo sapiens GN=ARF5 PE=1 SV=2	0.00	0.00	0.00	0.00	15.65	0.00	0.00	0.00	0.00	0.00	0.00	0.00	0.00	0.00	19.80	0.00	0.00	0.00	0.00	0.00	0.00	0.00	0.00	5.88	1.30	2.14	>0.999999999999999	0.6
P04208	Ig lambda chain V-I region WAH OS=Homo sapiens PE=1 SV=1	0.00	0.00	0.00	0.00	12.44	0.00	11.29	0.00	0.00	12.23	0.00	5.38	0.00	0.00	0.00	0.00	0.00	8.35	10.13	11.94	8.57	0.00	0.00	0.00	3.45	3.25	>0.999999999999999	1.1
Q08380	Galectin-3-binding protein OS=Homo sapiens GN=LGALS3BP PE=1 SV=1	0.00	0.00	0.00	0.00	1.89	3.78	0.85	1.44	1.37	0.00	0.79	2.77	2.35	1.61	0.00	0.00	2.02	0.77	0.90	0.65	0.84	1.28	0.71	1.36	1.07	1.04	>0.999999999999999	1.0
P18428	Lipopolysaccharide-binding protein OS=Homo sapiens GN=LBP PE=1 SV=3	0.00	0.00	0.00	0.00	2.45	0.00	0.00	0.00	0.00	0.00	0.00	0.49	0.00	0.00	1.43	1.01	0.76	0.00	0.00	0.78	0.00	0.00	0.00	0.00	0.25	0.33	>0.999999999999999	0.7
A6NKN8	Purkinje cell protein 4-like protein 1 OS=Homo sapiens GN=PCP4L1 PE=2 SV=3	0.00	0.00	0.00	0.00	9.05	11.34	0.00	0.00	0.00	8.89	7.55	0.00	0.00	0.00	0.00	0.00	0.00	0.00	0.00	0.00	0.00	0.00	0.00	1.92	3.07	0.16	>0.999999999999999	19.2
P07360	Complement component C8 gamma chain OS=Homo sapiens GN=C8G PE=1 SV=3	0.00	0.00	0.00	0.00	2.83	0.00	0.00	4.70	0.00	5.93	0.00	8.24	3.53	2.41	0.00	5.38	4.05	2.30	5.79	1.95	0.00	2.56	4.54	1.28	1.81	2.82	>0.999999999999999	0.6
P05114	Non-histone chromosomal protein HMG-14 OS=Homo sapiens GN=HMGN1 PE=1 SV=3	0.00	0.00	0.00	0.00	14.14	7.56	0.00	6.77	0.00	5.93	11.80	2.61	0.00	0.00	0.00	0.00	0.00	4.05	4.91	0.00	0.00	12.66	0.00	1.28	4.07	1.91	>0.999999999999999	2.1
P08579	U2 small nuclear ribonucleoprotein B'' OS=Homo sapiens GN=SNRPB2 PE=1 SV=1	0.00	0.00	0.00	0.00	2.45	6.62	0.00	0.00	0.00	2.41	2.04	0.00	0.00	0.00	0.00	0.00	0.00	0.00	0.00	0.00	0.00	0.00	0.00	0.00	1.13	0.00	>0.999999999999999	#DIV/0!
Q14997	Proteasome activator complex subunit 4 OS=Homo sapiens GN=PSME4 PE=1 SV=2	0.00	0.00	0.00	0.00	0.38	0.00	0.00	0.00	0.00	0.00	0.00	0.16	0.00	0.00	0.00	0.00	0.00	0.00	0.00	0.00	0.00	0.00	0.00	0.00	0.05	0.00	>0.999999999999999	#DIV/0!
O75800	Zinc finger MYND domain-containing protein 10 OS=Homo sapiens GN=ZMYND10 PE=1 SV=2	0.00	0.00	0.00	0.00	1.13	3.07	0.00	0.00	0.00	1.11	0.94	0.00	0.00	0.00	0.00	0.00	0.00	0.00	0.00	0.00	0.00	0.00	0.00	0.00	0.52	0.00	>0.999999999999999	#DIV/0!
P02743	Serum amyloid P-component OS=Homo sapiens GN=APCS PE=1 SV=2	0.00	0.00	0.00	0.00	2.45	0.00	0.00	0.00	1.78	0.00	0.00	3.67	3.06	0.00	0.00	2.19	1.64	0.00	2.35	1.69	2.19	0.00	1.84	0.52	0.66	1.29	>0.999999999999999	0.5
Q9GZP8	Immortalization up-regulated protein OS=Homo sapiens GN=IMUP PE=1 SV=1	0.00	0.00	0.00	0.00	6.03	7.56	0.00	0.00	0.00	0.00	5.03	0.00	0.00	0.00	0.00	0.00	4.05	0.00	0.00	0.00	0.00	0.00	0.00	0.00	1.55	0.34	>0.999999999999999	4.6
P57735	Ras-related protein Rab-25 OS=Homo sapiens GN=RAB25 PE=1 SV=2	0.00	0.00	0.00	0.00	2.64	3.31	0.00	0.00	0.00	0.00	0.00	1.14	0.00	0.00	0.00	0.00	0.00	0.00	0.00	0.00	0.00	1.12	0.00	0.00	0.59	0.09	>0.999999999999999	6.3
O00442	RNA 3'-terminal phosphate cyclase OS=Homo sapiens GN=RTCD1 PE=1 SV=1	0.00	0.00	0.00	0.00	1.51	0.00	0.00	0.00	0.00	1.48	0.00	0.65	0.00	0.00	0.00	0.00	0.00	0.00	0.00	0.00	0.00	0.00	0.00	0.00	0.30	0.00	>0.999999999999999	#DIV/0!
Q96E39	RNA binding motif protein, X-linked-like-1 OS=Homo sapiens GN=RBMXL1 PE=1 SV=1	0.00	0.00	0.00	0.00	3.02	0.00	1.37	0.00	0.00	0.00	2.52	0.65	1.88	0.00	0.00	0.00	0.00	0.00	0.00	0.00	0.00	0.64	0.00	0.00	0.63	0.21	>0.999999999999999	3.0
Q13243	Serine/arginine-rich splicing factor 5 OS=Homo sapiens GN=SRSF5 PE=1 SV=1	0.00	0.00	0.00	0.00	2.07	0.00	0.00	0.00	0.00	0.00	0.00	0.00	0.00	0.00	0.00	0.00	0.00	0.00	0.00	0.00	0.00	0.00	0.00	0.00	0.17	0.00	>0.999999999999999	#DIV/0!
Q63HN8	E3 ubiquitin-protein ligase RNF213 OS=Homo sapiens GN=RNF213 PE=1 SV=3	0.00	0.00	0.00	0.00	0.19	0.00	0.17	0.00	0.00	0.00	0.00	0.08	0.00	0.00	0.00	0.00	0.00	0.00	0.00	0.00	0.00	0.08	0.00	0.00	0.04	0.01	>0.999999999999999	5.5
P84103	Serine/arginine-rich splicing factor 3 OS=Homo sapiens GN=SRSF3 PE=1 SV=1	0.00	0.00	0.00	0.00	6.98	14.41	0.00	0.00	0.00	0.00	5.82	0.00	0.00	0.00	0.00	0.00	0.00	0.00	0.00	0.00	0.00	2.96	0.00	0.00	2.27	0.25	>0.999999999999999	9.2
Q9Y6Y8	SEC23-interacting protein OS=Homo sapiens GN=SEC23IP PE=1 SV=1	0.00	0.00	0.00	0.00	0.57	0.71	0.51	0.00	0.00	0.00	0.00	0.24	0.00	0.00	0.72	0.00	0.00	0.00	0.00	0.00	0.00	0.00	0.43	0.00	0.17	0.10	>0.999999999999999	1.8
Q8TF65	PDZ domain-containing protein GIPC2 OS=Homo sapiens GN=GIPC2 PE=1 SV=1	0.00	0.00	0.00	0.00	1.89	0.00	1.71	0.00	0.00	0.00	0.00	0.00	0.00	0.00	0.00	0.00	0.00	0.00	0.00	0.00	0.00	0.00	0.00	0.00	0.30	0.00	>0.999999999999999	#DIV/0!
P26447	Protein S100-A4 OS=Homo sapiens GN=S100A4 PE=1 SV=1	0.00	0.00	0.00	0.00	12.44	6.85	4.96	2.62	3.97	5.37	4.56	14.19	6.82	0.00	6.92	4.88	8.35	17.34	5.25	3.76	4.89	5.27	0.00	4.52	4.58	5.67	>0.999999999999999	0.8
Q99614	Tetratricopeptide repeat protein 1 OS=Homo sapiens GN=TTC1 PE=1 SV=1	0.00	0.00	0.00	0.00	1.89	0.00	0.00	0.00	0.00	0.00	1.57	0.00	0.00	0.00	0.00	0.00	0.00	0.00	0.00	0.00	0.00	0.00	0.00	0.00	0.29	0.00	>0.999999999999999	#DIV/0!
A6NIZ1	Ras-related protein Rap-1b-like protein OS=Homo sapiens PE=2 SV=1	0.00	0.00	0.00	0.00	3.02	8.03	5.81	0.00	0.00	0.00	0.00	0.00	3.76	0.00	3.82	2.69	0.00	0.00	0.00	0.00	0.00	0.00	0.00	0.00	1.41	0.86	>0.999999999999999	1.6
Q5T2P8	Annexin A8-like protein 1 OS=Homo sapiens GN=ANXA8L1 PE=2 SV=1	0.00	0.00	0.00	0.00	1.70	0.00	0.00	0.00	0.00	0.00	0.00	0.00	0.00	0.00	0.00	0.00	0.00	0.00	0.00	0.00	0.00	0.00	0.00	0.00	0.14	0.00	>0.999999999999999	#DIV/0!
O96007	Molybdopterin synthase catalytic subunit OS=Homo sapiens GN=MOCS2 PE=1 SV=1	0.00	0.00	0.00	0.00	3.02	0.00	2.74	0.00	0.00	0.00	2.52	0.00	0.00	0.00	3.82	0.00	0.00	0.00	0.00	0.00	0.00	0.00	2.27	0.00	0.69	0.51	>0.999999999999999	1.4
O43809	Cleavage and polyadenylation specificity factor subunit 5 OS=Homo sapiens GN=NUDT21 PE=1 SV=1	0.00	0.00	0.00	0.00	2.45	3.07	0.00	0.00	0.00	2.41	2.04	0.00	0.00	0.00	0.00	0.00	0.00	0.00	0.00	0.00	0.00	0.00	0.00	0.00	0.83	0.00	>0.999999999999999	#DIV/0!
Q15427	Splicing factor 3B subunit 4 OS=Homo sapiens GN=SF3B4 PE=1 SV=1	0.00	0.00	0.00	0.00	1.32	1.65	0.00	0.00	0.00	0.00	0.00	0.00	0.00	0.00	0.00	0.00	0.00	0.00	0.00	0.00	0.00	0.00	0.00	0.00	0.25	0.00	>0.999999999999999	#DIV/0!
Q9H0A8	COMM domain-containing protein 4 OS=Homo sapiens GN=COMMD4 PE=1 SV=1	0.00	0.00	0.00	0.00	2.83	0.00	2.56	1.35	0.00	0.00	0.00	0.00	0.00	2.41	0.00	0.00	0.00	0.00	0.00	0.00	0.00	0.00	0.00	0.00	0.56	0.20	>0.999999999999999	2.8
Q6ZV29	Patatin-like phospholipase domain-containing protein 7 OS=Homo sapiens GN=PNPLA7 PE=2 SV=3	0.00	0.00	0.00	0.00	0.38	0.00	0.00	0.00	0.00	0.00	0.00	0.00	0.00	0.00	0.00	0.00	0.00	0.00	0.00	0.00	0.00	0.00	0.00	0.00	0.03	0.00	>0.999999999999999	#DIV/0!
Q99623	Prohibitin-2 OS=Homo sapiens GN=PHB2 PE=1 SV=2	0.00	0.00	0.00	0.00	1.89	0.00	0.00	0.00	0.00	0.00	0.00	0.00	0.00	0.00	0.00	0.00	0.00	0.00	0.00	0.00	0.00	0.00	0.00	0.00	0.16	0.00	>0.999999999999999	#DIV/0!
Q9H0B6	Kinesin light chain 2 OS=Homo sapiens GN=KLC2 PE=1 SV=1	0.00	0.00	0.00	0.00	0.94	1.18	0.00	0.00	0.00	0.00	0.00	0.00	0.00	0.00	0.00	0.00	0.00	0.00	0.00	0.00	0.00	0.40	0.00	0.00	0.18	0.03	>0.999999999999999	5.3
P05161	Ubiquitin-like protein ISG15 OS=Homo sapiens GN=ISG15 PE=1 SV=5	0.00	0.00	0.00	0.00	3.58	0.00	23.25	0.00	0.00	0.00	0.00	1.55	0.00	0.00	0.00	0.00	0.00	0.00	3.44	0.00	0.00	0.00	0.00	0.00	2.37	0.29	>0.999999999999999	8.3
P41236	Protein phosphatase inhibitor 2 OS=Homo sapiens GN=PPP1R2 PE=1 SV=2	0.00	0.00	0.00	0.00	2.83	0.00	0.00	0.00	0.00	0.00	2.36	0.00	0.00	0.00	0.00	0.00	0.00	0.00	0.00	0.00	0.00	0.00	0.00	0.00	0.43	0.00	>0.999999999999999	#DIV/0!
Q16610	Extracellular matrix protein 1 OS=Homo sapiens GN=ECM1 PE=1 SV=2	0.00	0.00	0.00	0.00	0.94	0.00	0.00	0.00	0.00	0.00	0.00	0.00	0.00	0.00	0.00	0.00	0.63	0.00	0.00	0.00	0.00	0.00	0.00	0.00	0.08	0.05	>0.999999999999999	1.5
Q5R3I4	Tetratricopeptide repeat protein 38 OS=Homo sapiens GN=TTC38 PE=1 SV=1	0.00	0.00	0.00	0.00	1.13	0.00	0.00	0.00	0.00	0.00	0.94	0.00	0.00	0.00	0.00	0.00	0.00	0.00	0.00	0.00	1.01	0.00	0.85	0.00	0.17	0.16	>0.999999999999999	1.1
Q8WU90	Zinc finger CCCH domain-containing protein 15 OS=Homo sapiens GN=ZC3H15 PE=1 SV=1	0.00	0.00	0.00	0.00	1.32	0.00	0.00	0.00	0.00	0.00	0.00	0.00	0.00	0.00	0.00	0.00	0.89	0.00	0.00	0.00	0.00	0.56	0.00	0.00	0.11	0.12	>0.999999999999999	0.9
P06312	Ig kappa chain V-IV region (Fragment) OS=Homo sapiens GN=IGKV4-1 PE=4 SV=1	0.00	0.00	0.00	0.00	10.75	0.00	4.27	8.76	3.42	0.00	0.00	4.65	22.81	0.00	0.00	0.00	12.27	0.00	17.54	0.00	0.00	0.00	0.00	1.00	2.65	4.47	>0.999999999999999	0.6
P48556	26S proteasome non-ATPase regulatory subunit 8 OS=Homo sapiens GN=PSMD8 PE=1 SV=2	0.00	0.00	0.00	0.00	1.51	0.00	1.37	0.00	0.00	0.00	1.26	0.00	0.00	0.00	1.91	1.35	0.00	0.00	0.00	0.00	0.00	0.64	0.00	0.32	0.34	0.35	>0.999999999999999	1.0
Q9Y223	Bifunctional UDP-N-acetylglucosamine 2-epimerase/N-acetylmannosamine kinase OS=Homo sapiens GN=GNE PE	0.00	0.00	0.00	0.00	0.75	0.95	0.68	0.00	0.00	0.00	0.00	0.65	0.00	0.00	0.00	0.67	0.51	0.00	0.72	0.00	0.00	0.64	0.00	0.16	0.25	0.23	>0.999999999999999	1.1
Q9UHD2	Serine/threonine-protein kinase TBK1 OS=Homo sapiens GN=TBK1 PE=1 SV=1	0.00	0.00	0.00	0.00	0.75	0.00	0.00	0.00	0.00	0.00	0.00	0.00	0.00	0.00	0.00	0.00	0.00	0.00	0.00	0.00	0.00	0.00	0.00	0.00	0.06	0.00	>0.999999999999999	#DIV/0!
P13807	Glycogen [starch] synthase, muscle OS=Homo sapiens GN=GYS1 PE=1 SV=2	0.00	0.00	0.00	0.00	0.75	0.95	0.00	0.00	0.00	0.74	0.00	0.00	0.00	0.00	0.00	0.00	0.00	0.00	0.00	0.00	0.00	0.00	0.00	0.00	0.20	0.00	>0.999999999999999	#DIV/0!
Q8NBL1	Protein O-glucosyltransferase 1 OS=Homo sapiens GN=POGLUT1 PE=1 SV=1	0.00	0.00	0.00	0.00	1.32	0.00	0.00	0.00	0.00	0.00	0.00	0.00	0.00	0.00	0.00	0.00	0.00	0.00	0.00	0.00	0.00	0.00	0.00	0.00	0.11	0.00	>0.999999999999999	#DIV/0!
P01889	HLA class I histocompatibility antigen, B-7 alpha chain OS=Homo sapiens GN=HLA-B PE=1 SV=3	0.00	0.00	0.00	0.00	1.51	0.00	0.00	0.72	1.10	0.00	1.26	0.00	0.00	0.00	0.00	1.01	0.00	0.00	0.00	0.00	0.00	0.00	0.00	0.00	0.38	0.08	>0.999999999999999	4.5
P32929	Cystathionine gamma-lyase OS=Homo sapiens GN=CTH PE=1 SV=3	0.00	0.00	0.00	0.00	1.32	0.00	0.00	0.00	0.00	0.00	0.00	0.57	0.00	0.00	0.00	0.00	0.00	0.00	0.00	0.00	0.00	0.00	0.00	0.00	0.16	0.00	>0.999999999999999	#DIV/0!
Q9BQ61	Uncharacterized protein C19orf43 OS=Homo sapiens GN=C19orf43 PE=1 SV=1	0.00	0.00	0.00	0.00	3.39	4.25	0.00	0.00	0.00	0.00	2.83	0.00	0.00	0.00	0.00	0.00	0.00	0.00	0.00	0.00	0.00	0.00	0.00	0.72	0.87	0.06	>0.999999999999999	14.5
Q9UKM9	RNA-binding protein Raly OS=Homo sapiens GN=RALY PE=1 SV=1	0.00	0.00	0.00	0.00	1.89	2.36	1.71	0.90	0.00	0.00	0.00	0.82	0.00	0.00	0.00	0.00	0.00	0.00	3.80	0.00	1.69	0.00	0.00	0.00	0.64	0.46	>0.999999999999999	1.4
Q92530	Proteasome inhibitor PI31 subunit OS=Homo sapiens GN=PSMF1 PE=1 SV=2	0.00	0.00	0.00	0.00	2.07	0.00	0.00	0.00	0.00	0.00	1.73	0.00	0.00	0.00	2.62	0.00	0.00	0.00	0.00	0.00	0.00	0.00	1.56	0.00	0.32	0.35	>0.999999999999999	0.9
P09543	2',3'-cyclic-nucleotide 3'-phosphodiesterase OS=Homo sapiens GN=CNP PE=1 SV=2	0.00	0.00	0.00	0.00	2.64	0.00	0.00	0.00	0.00	0.00	0.00	0.57	0.00	0.00	0.00	0.00	0.00	0.00	0.00	0.00	0.00	0.00	0.00	0.00	0.27	0.00	>0.999999999999999	#DIV/0!
Q8IWZ3	Ankyrin repeat and KH domain-containing protein 1 OS=Homo sapiens GN=ANKHD1 PE=1 SV=1	0.00	0.00	0.00	0.00	0.19	0.00	0.00	0.00	0.00	0.00	0.00	0.00	0.00	0.00	0.00	0.00	0.00	0.00	0.00	0.00	0.00	0.00	0.00	0.00	0.02	0.00	>0.999999999999999	#DIV/0!
Q99436	Proteasome subunit beta type-7 OS=Homo sapiens GN=PSMB7 PE=1 SV=1	0.00	0.00	0.00	0.00	2.07	2.60	0.00	0.00	0.00	0.00	1.73	0.90	0.00	0.00	0.00	0.00	1.69	0.00	0.00	0.00	0.00	0.00	0.00	0.00	0.61	0.14	>0.999999999999999	4.3
Q9UDY4	DnaJ homolog subfamily B member 4 OS=Homo sapiens GN=DNAJB4 PE=1 SV=1	0.00	0.00	0.00	0.00	1.70	0.00	0.00	0.00	0.00	1.67	1.42	0.00	0.00	0.00	0.00	0.00	0.00	0.00	0.00	0.00	0.00	0.00	0.00	0.00	0.40	0.00	>0.999999999999999	#DIV/0!
Q15154	Pericentriolar material 1 protein OS=Homo sapiens GN=PCM1 PE=1 SV=4	0.00	0.00	0.00	0.00	0.19	0.00	0.17	0.00	0.00	0.00	0.16	0.00	0.00	0.00	0.00	0.00	0.00	0.00	0.00	0.00	0.00	0.00	0.00	0.00	0.04	0.00	>0.999999999999999	#DIV/0!
P62306	Small nuclear ribonucleoprotein F OS=Homo sapiens GN=SNRPF PE=1 SV=1	0.00	0.00	0.00	0.00	6.79	8.51	0.00	0.00	0.00	0.00	0.00	0.00	0.00	0.00	0.00	0.00	0.00	0.00	0.00	0.00	0.00	0.00	5.11	1.44	1.27	0.55	>0.999999999999999	2.3
P62318	Small nuclear ribonucleoprotein Sm D3 OS=Homo sapiens GN=SNRPD3 PE=1 SV=1	0.00	0.00	0.00	0.00	4.53	5.67	0.00	0.00	0.00	4.45	14.47	0.00	0.00	0.00	0.00	0.00	0.00	0.00	4.34	3.12	0.00	0.00	0.00	0.00	2.43	0.62	>0.999999999999999	3.9
Q9BV57	1,2-dihydroxy-3-keto-5-methylthiopentene dioxygenase OS=Homo sapiens GN=ADI1 PE=1 SV=1	0.00	0.00	0.00	0.00	3.02	0.00	0.00	0.00	0.00	0.00	0.00	0.00	0.00	0.00	0.00	0.00	0.00	0.00	0.00	0.00	0.00	0.00	2.27	0.00	0.25	0.19	>0.999999999999999	1.3
Q8IXM2	Chromatin complexes subunit BAP18 OS=Homo sapiens GN=BAP18 PE=1 SV=1	0.00	0.00	0.00	0.00	3.58	0.00	3.25	0.00	0.00	0.00	0.00	0.00	4.47	0.00	0.00	0.00	0.00	0.00	0.00	0.00	0.00	0.00	0.00	0.00	0.57	0.37	>0.999999999999999	1.5
Q9NU02	Ankyrin repeat domain-containing protein 5 OS=Homo sapiens GN=ANKRD5 PE=2 SV=2	0.00	0.00	0.00	0.00	0.75	0.00	0.00	0.00	0.00	0.00	0.00	0.00	0.00	0.00	0.00	0.00	0.00	0.00	0.00	0.00	0.00	0.00	0.00	0.00	0.06	0.00	>0.999999999999999	#DIV/0!
Q04118	Basic salivary proline-rich protein 3 OS=Homo sapiens GN=PRB3 PE=1 SV=2	0.00	0.00	0.00	0.00	2.07	2.60	0.00	0.99	0.00	0.00	1.73	0.00	0.00	0.00	0.00	0.00	2.91	0.00	0.00	0.00	1.86	0.00	0.00	0.00	0.62	0.40	>0.999999999999999	1.6
P12035	Keratin, type II cytoskeletal 3 OS=Homo sapiens GN=KRT3 PE=1 SV=3	0.00	0.00	0.00	0.00	1.89	0.00	0.00	0.00	0.00	0.00	0.00	0.00	0.00	0.00	0.00	1.68	0.00	0.00	0.00	0.00	0.00	0.00	0.00	0.00	0.16	0.14	>0.999999999999999	1.1
P50995	Annexin A11 OS=Homo sapiens GN=ANXA11 PE=1 SV=1	0.00	0.00	0.00	0.00	1.13	1.42	0.00	0.00	0.00	0.00	0.00	0.00	0.00	0.00	1.43	0.00	0.00	0.00	0.00	0.00	0.00	0.00	0.00	0.00	0.21	0.12	>0.999999999999999	1.8
P62330	ADP-ribosylation factor 6 OS=Homo sapiens GN=ARF6 PE=1 SV=2	0.00	0.00	0.00	0.00	6.79	4.02	0.00	0.00	0.00	0.00	0.00	0.00	0.00	0.00	0.00	0.00	0.00	0.00	0.00	0.00	0.00	0.00	0.00	0.00	0.90	0.00	>0.999999999999999	#DIV/0!
Q15843	NEDD8 OS=Homo sapiens GN=NEDD8 PE=1 SV=1	0.00	0.00	0.00	0.00	7.16	0.00	6.50	3.43	0.00	0.00	0.00	3.10	0.00	0.00	0.00	0.00	4.81	0.00	6.87	0.00	0.00	0.00	0.00	0.00	1.68	0.97	>0.999999999999999	1.7
Q8NHP1	Aflatoxin B1 aldehyde reductase member 4 OS=Homo sapiens GN=AKR7L PE=2 SV=6	0.00	0.00	0.00	0.00	1.70	0.00	1.54	0.00	0.00	0.00	0.00	0.00	0.00	0.00	0.00	0.00	1.38	0.00	0.00	0.00	0.00	0.00	0.00	0.00	0.27	0.12	>0.999999999999999	2.3
O43670	Zinc finger protein 207 OS=Homo sapiens GN=ZNF207 PE=1 SV=1	0.00	0.00	0.00	0.00	1.13	0.00	0.00	0.00	0.00	0.00	0.00	0.00	0.00	0.00	0.00	0.00	0.00	0.00	0.00	0.00	0.00	0.00	0.00	0.00	0.09	0.00	>0.999999999999999	#DIV/0!
Q9ULV0	Unconventional myosin-Vb OS=Homo sapiens GN=MYO5B PE=1 SV=3	0.00	0.00	0.00	0.00	0.38	0.47	0.00	0.00	0.00	0.00	0.31	0.16	0.00	0.00	1.19	0.00	0.00	0.00	0.00	0.00	0.00	0.00	0.00	0.00	0.11	0.10	>0.999999999999999	1.1
Q9NRY5	Protein FAM114A2 OS=Homo sapiens GN=FAM114A2 PE=1 SV=4	0.00	0.00	0.00	0.00	1.13	0.00	0.00	0.00	0.00	0.00	0.00	0.00	0.00	0.00	0.00	0.00	0.00	0.00	0.00	0.00	0.00	0.00	0.00	0.00	0.09	0.00	>0.999999999999999	#DIV/0!
Q6P1N0	Coiled-coil and C2 domain-containing protein 1A OS=Homo sapiens GN=CC2D1A PE=1 SV=1	0.00	0.00	0.00	0.00	0.57	0.00	0.51	0.00	0.00	0.00	0.00	0.00	0.00	0.00	0.00	0.00	0.00	0.00	0.00	0.00	0.00	0.00	0.00	0.00	0.09	0.00	>0.999999999999999	#DIV/0!
P31431	Syndecan-4 OS=Homo sapiens GN=SDC4 PE=1 SV=2	0.00	0.00	0.00	0.00	3.02	7.80	0.00	0.00	2.19	6.11	0.00	2.69	0.00	0.00	0.00	0.00	0.00	2.45	2.89	0.00	0.00	0.00	0.00	0.00	1.82	0.45	>0.999999999999999	4.1
P16949	Stathmin OS=Homo sapiens GN=STMN1 PE=1 SV=3	0.00	0.00	0.00	0.00	3.77	0.00	3.42	1.81	0.00	0.00	0.00	0.00	4.70	0.00	0.00	0.00	0.00	0.00	0.00	0.00	3.38	0.00	0.00	0.00	0.75	0.67	>0.999999999999999	1.1
Q8TF72	Protein Shroom3 OS=Homo sapiens GN=SHROOM3 PE=1 SV=2	0.00	0.00	0.00	0.00	0.19	0.00	0.00	0.00	0.00	0.19	0.00	0.00	0.00	0.00	0.00	0.00	0.00	0.00	0.18	0.00	0.00	0.00	0.00	0.00	0.03	0.02	>0.999999999999999	2.1
Q8IYT8	Serine/threonine-protein kinase ULK2 OS=Homo sapiens GN=ULK2 PE=1 SV=3	0.00	0.00	0.00	0.00	0.57	0.00	0.00	0.00	0.00	0.00	0.00	0.00	0.00	0.00	0.00	0.00	0.00	0.00	0.00	0.00	0.00	0.24	0.00	0.00	0.05	0.02	>0.999999999999999	2.4
Q9NWV8	BRISC and BRCA1-A complex member 1 OS=Homo sapiens GN=BABAM1 PE=1 SV=1	0.00	0.00	0.00	0.00	1.70	0.00	1.54	0.00	0.00	0.00	0.00	0.00	0.00	0.00	0.00	0.00	0.00	0.00	0.00	0.00	0.00	0.00	0.00	0.00	0.27	0.00	>0.999999999999999	#DIV/0!
Q9C005	Protein dpy-30 homolog OS=Homo sapiens GN=DPY30 PE=1 SV=1	0.00	0.00	0.00	0.00	5.85	0.00	0.00	0.00	0.00	0.00	4.88	0.00	0.00	4.98	0.00	0.00	0.00	0.00	5.61	0.00	0.00	0.00	4.40	0.00	0.89	1.25	>0.999999999999999	0.7
Q9NZ32	Actin-related protein 10 OS=Homo sapiens GN=ACTR10 PE=1 SV=1	0.00	0.00	0.00	0.00	1.32	0.00	0.00	0.00	0.00	0.00	0.00	0.00	0.00	0.00	0.00	0.00	0.00	0.00	0.00	0.00	0.00	0.00	0.00	0.00	0.11	0.00	>0.999999999999999	#DIV/0!
P60673	Profilin-3 OS=Homo sapiens GN=PFN3 PE=2 SV=1	0.00	0.00	0.00	0.00	4.34	0.00	0.00	0.00	0.00	0.00	0.00	0.00	0.00	0.00	0.00	0.00	0.00	0.00	0.00	0.00	0.00	0.00	0.00	0.00	0.36	0.00	>0.999999999999999	#DIV/0!
Q15057	Arf-GAP with coiled-coil, ANK repeat and PH domain-containing protein 2 OS=Homo sapiens GN=ACAP2 PE=1 SV=	0.00	0.00	0.00	0.00	0.75	2.60	0.00	0.00	0.00	0.00	0.00	0.33	0.00	0.00	0.00	0.00	0.00	0.00	0.00	0.00	0.00	0.00	0.00	0.00	0.31	0.00	>0.999999999999999	#DIV/0!
P27635	60S ribosomal protein L10 OS=Homo sapiens GN=RPL10 PE=1 SV=4	0.00	0.00	0.00	0.00	2.45	0.00	0.00	0.00	0.00	0.00	0.00	0.00	0.00	0.00	0.00	0.00	0.00	0.00	0.00	0.00	0.00	0.00	0.00	0.00	0.20	0.00	>0.999999999999999	#DIV/0!
O14815	Calpain-9 OS=Homo sapiens GN=CAPN9 PE=1 SV=1	0.00	0.00	0.00	0.00	0.75	0.00	0.00	0.00	0.00	0.00	0.00	0.00	0.00	0.00	0.95	0.00	0.00	0.00	0.00	0.00	0.00	0.00	0.00	0.00	0.06	0.08	>0.999999999999999	0.8
Q66K14	TBC1 domain family member 9B OS=Homo sapiens GN=TBC1D9B PE=1 SV=3	0.00	0.00	0.00	0.00	0.38	0.00	0.00	0.00	0.00	0.00	0.00	0.00	0.00	0.00	0.00	0.00	0.00	0.00	0.00	0.00	0.00	0.00	0.00	0.00	0.03	0.00	>0.999999999999999	#DIV/0!
Q9Y3A5	Ribosome maturation protein SBDS OS=Homo sapiens GN=SBDS PE=1 SV=4	0.00	0.00	0.00	0.00	2.07	0.00	0.00	0.00	0.00	0.00	1.73	0.00	0.00	1.77	0.00	0.00	0.00	0.00	1.99	0.00	0.00	0.88	1.56	0.44	0.32	0.55	>0.999999999999999	0.6
Q969Z0	Protein TBRG4 OS=Homo sapiens GN=TBRG4 PE=1 SV=1	0.00	0.00	0.00	0.00	0.94	0.00	0.00	0.00	0.00	0.00	0.00	0.00	0.00	0.00	0.00	0.00	0.00	0.00	0.00	0.00	0.00	0.00	0.00	0.00	0.08	0.00	>0.999999999999999	#DIV/0!
Q12874	Splicing factor 3A subunit 3 OS=Homo sapiens GN=SF3A3 PE=1 SV=1	0.00	0.00	0.00	0.00	1.13	1.42	0.00	0.00	0.00	1.11	0.94	0.00	0.00	0.00	0.00	0.00	0.00	0.00	0.00	0.00	0.00	0.00	0.00	0.00	0.38	0.00	>0.999999999999999	#DIV/0!
P17066	Heat shock 70 kDa protein 6 OS=Homo sapiens GN=HSPA6 PE=1 SV=2	0.00	0.00	0.00	0.00	0.00	11.82	0.00	0.00	0.00	0.00	0.00	0.00	0.00	0.00	0.00	0.00	0.00	0.00	0.00	0.00	0.00	0.00	0.00	0.00	0.98	0.00	>0.999999999999999	#DIV/0!
Q04695	Keratin, type I cytoskeletal 17 OS=Homo sapiens GN=KRT17 PE=1 SV=2	0.00	0.00	0.00	0.00	0.00	7.09	0.00	0.00	0.00	9.08	0.00	3.18	0.00	0.00	0.00	0.00	0.00	0.00	0.00	5.06	0.00	0.00	0.00	1.96	1.61	0.59	>0.999999999999999	2.8
P55795	Heterogeneous nuclear ribonucleoprotein H2 OS=Homo sapiens GN=HNRNPH2 PE=1 SV=1	0.00	0.00	0.00	0.00	0.00	6.85	0.00	0.00	0.00	0.00	5.98	0.00	0.00	0.00	0.00	2.36	0.00	0.00	0.00	5.25	0.00	0.00	1.99	0.00	1.07	0.80	>0.999999999999999	1.3
P20337	Ras-related protein Rab-3B OS=Homo sapiens GN=RAB3B PE=1 SV=2	0.00	0.00	0.00	0.00	0.00	6.85	0.00	0.00	0.00	0.00	0.00	0.00	0.00	0.00	0.00	0.00	0.00	0.00	0.00	0.00	0.00	0.00	0.00	0.00	0.57	0.00	>0.999999999999999	#DIV/0!
P09493	Tropomyosin alpha-1 chain OS=Homo sapiens GN=TPM1 PE=1 SV=2	0.00	0.00	0.00	0.00	0.00	8.03	0.00	0.00	0.00	0.00	0.00	0.00	0.00	0.00	0.00	0.00	0.00	0.00	0.00	0.00	0.00	0.00	1.42	0.00	0.67	0.12	>0.999999999999999	5.7
P61020	Ras-related protein Rab-5B OS=Homo sapiens GN=RAB5B PE=1 SV=1	0.00	0.00	0.00	0.00	0.00	7.09	0.00	0.00	0.00	0.00	4.72	0.00	0.00	0.00	0.00	0.00	0.00	0.00	0.00	0.00	0.00	0.00	0.00	0.00	0.98	0.00	>0.999999999999999	#DIV/0!
Q86TU7	Histone-lysine N-methyltransferase setd3 OS=Homo sapiens GN=SETD3 PE=1 SV=1	0.00	0.00	0.00	0.00	0.00	2.36	0.00	0.00	0.00	0.00	0.79	0.00	0.00	0.00	0.00	0.00	0.00	0.00	0.00	0.00	0.00	0.00	0.00	0.20	0.26	0.02	>0.999999999999999	15.7
Q8TD19	Serine/threonine-protein kinase Nek9 OS=Homo sapiens GN=NEK9 PE=1 SV=2	0.00	0.00	0.00	0.00	0.00	1.42	0.00	0.00	0.00	0.00	0.00	0.00	0.00	0.00	0.00	0.00	0.00	0.00	0.00	0.00	0.00	0.00	0.00	0.00	0.12	0.00	>0.999999999999999	#DIV/0!
P02795	Metallothionein-2 OS=Homo sapiens GN=MT2A PE=1 SV=1	0.00	0.00	0.00	0.00	0.00	29.07	0.00	0.00	0.00	0.00	0.00	0.00	0.00	0.00	0.00	0.00	0.00	0.00	0.00	0.00	0.00	0.00	0.00	0.00	2.42	0.00	>0.999999999999999	#DIV/0!
Q9NRX4	14 kDa phosphohistidine phosphatase OS=Homo sapiens GN=PHPT1 PE=1 SV=1	0.00	0.00	0.00	0.00	0.00	5.67	0.00	0.00	0.00	0.00	3.77	1.96	0.00	0.00	0.00	0.00	3.04	0.00	0.00	0.00	0.00	0.00	3.41	0.96	0.95	0.62	>0.999999999999999	1.5
Q9BUJ2	Heterogeneous nuclear ribonucleoprotein U-like protein 1 OS=Homo sapiens GN=HNRNPUL1 PE=1 SV=2	0.00	0.00	0.00	0.00	0.00	0.71	0.00	0.00	0.00	0.00	0.47	0.24	0.00	0.00	0.00	0.00	0.00	0.00	0.00	0.00	0.00	0.00	0.00	0.00	0.12	0.00	>0.999999999999999	#DIV/0!
O14974	Protein phosphatase 1 regulatory subunit 12A OS=Homo sapiens GN=PPP1R12A PE=1 SV=1	0.00	0.00	0.00	0.00	0.00	0.71	0.00	0.00	0.00	0.00	0.00	0.00	0.00	0.00	0.00	0.00	0.00	0.00	0.00	0.00	0.00	0.00	0.00	0.00	0.06	0.00	>0.999999999999999	#DIV/0!
Q9H773	dCTP pyrophosphatase 1 OS=Homo sapiens GN=DCTPP1 PE=1 SV=1	0.00	0.00	0.00	0.00	0.00	4.25	0.00	0.00	0.00	0.00	2.83	0.00	0.00	0.00	0.00	0.00	0.00	0.00	0.00	0.00	0.00	0.00	0.00	0.72	0.59	0.06	>0.999999999999999	9.8
Q6UWP2	Dehydrogenase/reductase SDR family member 11 OS=Homo sapiens GN=DHRS11 PE=1 SV=1	0.00	0.00	0.00	0.00	0.00	2.84	0.00	0.00	0.00	0.00	0.00	0.00	0.00	0.00	0.00	0.00	0.00	0.00	0.00	0.00	0.00	0.00	0.00	0.00	0.24	0.00	>0.999999999999999	#DIV/0!
Q9H6Z4	Ran-binding protein 3 OS=Homo sapiens GN=RANBP3 PE=1 SV=1	0.00	0.00	0.00	0.00	0.00	1.18	0.00	0.00	0.00	0.93	0.79	0.41	0.00	0.00	0.00	0.63	0.00	0.00	0.00	0.00	0.00	0.00	0.00	0.44	0.28	0.09	>0.999999999999999	3.1
Q7Z739	YTH domain family protein 3 OS=Homo sapiens GN=YTHDF3 PE=1 SV=1	0.00	0.00	0.00	0.00	0.00	1.18	0.00	0.00	0.00	0.00	0.00	0.00	0.00	0.00	0.00	0.00	0.00	0.00	0.00	0.00	0.00	0.00	0.00	0.00	0.10	0.00	>0.999999999999999	#DIV/0!
Q9NXG2	THUMP domain-containing protein 1 OS=Homo sapiens GN=THUMPD1 PE=1 SV=2	0.00	0.00	0.00	0.00	0.00	4.02	0.00	0.00	0.00	0.00	1.26	0.00	0.00	0.00	0.00	0.00	0.00	0.00	0.00	0.00	1.36	0.00	0.00	0.00	0.44	0.11	>0.999999999999999	3.9
P46060	Ran GTPase-activating protein 1 OS=Homo sapiens GN=RANGAP1 PE=1 SV=1	0.00	0.00	0.00	0.00	0.00	1.18	0.00	0.00	0.00	0.00	0.00	0.41	0.00	0.00	0.00	0.84	0.63	0.00	0.90	0.00	0.00	0.00	0.00	0.64	0.13	0.25	>0.999999999999999	0.5
Q9NRG4	N-lysine methyltransferase SMYD2 OS=Homo sapiens GN=SMYD2 PE=1 SV=2	0.00	0.00	0.00	0.00	0.00	1.65	0.00	0.00	0.00	0.00	0.00	0.00	0.00	0.00	0.00	0.00	0.00	0.00	0.00	0.00	0.00	0.00	0.00	0.00	0.14	0.00	>0.999999999999999	#DIV/0!
Q08170	Serine/arginine-rich splicing factor 4 OS=Homo sapiens GN=SRSF4 PE=1 SV=2	0.00	0.00	0.00	0.00	0.00	2.84	0.00	0.00	0.00	0.00	0.94	0.00	0.00	0.00	0.00	0.00	0.00	0.00	0.00	0.00	0.00	0.00	0.00	0.00	0.31	0.00	>0.999999999999999	#DIV/0!
Q7LG56	Ribonucleoside-diphosphate reductase subunit M2 B OS=Homo sapiens GN=RRM2B PE=1 SV=1	0.00	0.00	0.00	0.00	0.00	1.89	0.00	0.00	0.00	0.00	0.00	0.00	0.00	0.00	0.00	0.00	0.00	0.00	0.00	0.00	0.00	0.00	1.14	0.00	0.16	0.09	>0.999999999999999	1.7
Q9Y2S0	DNA-directed RNA polymerases I and III subunit RPAC2 OS=Homo sapiens GN=POLR1D PE=1 SV=1	0.00	0.00	0.00	0.00	0.00	5.20	0.00	0.00	0.00	0.00	0.00	0.00	0.00	0.00	0.00	0.00	0.00	0.00	0.00	0.00	0.00	1.76	0.00	0.00	0.43	0.15	>0.999999999999999	3.0
P37840	Alpha-synuclein OS=Homo sapiens GN=SNCA PE=1 SV=1	0.00	0.00	0.00	0.00	0.00	5.67	0.00	0.00	0.00	0.00	0.00	0.00	0.00	0.00	0.00	0.00	0.00	0.00	0.00	0.00	0.00	0.00	0.00	0.00	0.47	0.00	>0.999999999999999	#DIV/0!
Q14498	RNA-binding protein 39 OS=Homo sapiens GN=RBM39 PE=1 SV=2	0.00	0.00	0.00	0.00	0.00	1.42	0.00	0.00	0.00	1.11	1.73	0.00	0.00	0.00	0.00	0.00	0.00	0.00	0.00	0.00	0.00	0.00	0.00	0.00	0.35	0.00	>0.999999999999999	#DIV/0!
Q9H0E2	Toll-interacting protein OS=Homo sapiens GN=TOLLIP PE=1 SV=1	0.00	0.00	0.00	0.00	0.00	2.60	0.00	0.00	0.00	0.00	0.00	0.00	0.00	0.00	0.00	0.00	0.00	0.00	0.00	0.00	0.00	0.00	0.00	0.00	0.22	0.00	>0.999999999999999	#DIV/0!
Q9Y5K8	V-type proton ATPase subunit D OS=Homo sapiens GN=ATP6V1D PE=1 SV=1	0.00	0.00	0.00	0.00	0.00	2.84	0.00	0.00	0.00	0.00	0.00	0.98	2.82	0.00	0.00	0.00	0.00	0.00	0.00	0.00	0.00	0.00	0.00	0.48	0.32	0.28	>0.999999999999999	1.2
O43768	Alpha-endosulfine OS=Homo sapiens GN=ENSA PE=1 SV=1	0.00	0.00	0.00	0.00	0.00	5.91	0.00	0.00	0.00	4.63	0.00	2.04	0.00	0.00	0.00	0.00	0.00	0.00	0.00	0.00	0.00	2.00	0.00	1.00	1.05	0.25	>0.999999999999999	4.2
P29373	Cellular retinoic acid-binding protein 2 OS=Homo sapiens GN=CRABP2 PE=1 SV=2	0.00	0.00	0.00	0.00	0.00	4.96	0.00	0.00	0.00	0.00	0.00	0.00	0.00	0.00	0.00	0.00	0.00	0.00	0.00	0.00	0.00	0.00	0.00	0.00	0.41	0.00	>0.999999999999999	#DIV/0!
Q9NZU5	LIM and cysteine-rich domains protein 1 OS=Homo sapiens GN=LMCD1 PE=1 SV=1	0.00	0.00	0.00	0.00	0.00	1.89	0.00	0.00	0.00	0.00	0.00	0.00	0.00	0.00	0.00	0.00	0.00	0.00	0.00	0.00	0.00	0.00	0.00	0.00	0.16	0.00	>0.999999999999999	#DIV/0!
P0CG29	Glutathione S-transferase theta-2 OS=Homo sapiens GN=GSTT2 PE=1 SV=1	0.00	0.00	0.00	0.00	0.00	2.84	0.00	0.00	0.00	2.22	1.89	0.00	0.00	0.00	0.00	0.00	0.00	0.00	0.00	0.00	0.96	0.00	0.00	0.00	0.58	0.08	>0.999999999999999	7.2
B5MCN3	Putative SEC14-like protein 6 OS=Homo sapiens GN=SEC14L6 PE=5 SV=1	0.00	0.00	0.00	0.00	0.00	1.65	0.00	0.00	0.00	0.00	0.00	0.00	0.00	0.00	0.00	0.00	0.00	0.00	0.00	0.00	0.00	0.00	0.00	0.00	0.14	0.00	>0.999999999999999	#DIV/0!
Q8NBS9	Thioredoxin domain-containing protein 5 OS=Homo sapiens GN=TXNDC5 PE=1 SV=2	0.00	0.00	0.00	0.00	0.00	1.65	0.00	0.00	0.00	0.00	0.00	1.79	0.00	0.00	0.00	0.00	0.00	0.00	0.00	0.00	0.00	0.00	0.00	0.88	0.29	0.07	>0.999999999999999	3.9
P84090	Enhancer of rudimentary homolog OS=Homo sapiens GN=ERH PE=1 SV=1	0.00	0.00	0.00	0.00	0.00	6.62	0.00	0.00	0.00	5.19	0.00	0.00	0.00	0.00	0.00	0.00	0.00	0.00	0.00	0.00	0.00	0.00	0.00	0.00	0.98	0.00	>0.999999999999999	#DIV/0!
P19388	DNA-directed RNA polymerases I, II, and III subunit RPABC1 OS=Homo sapiens GN=POLR2E PE=1 SV=4	0.00	0.00	0.00	0.00	0.00	3.31	0.00	0.00	0.00	0.00	0.00	0.00	0.00	0.00	0.00	0.00	0.00	0.00	0.00	0.00	0.00	0.00	0.00	0.00	0.28	0.00	>0.999999999999999	#DIV/0!
P41227	N-alpha-acetyltransferase 10 OS=Homo sapiens GN=NAA10 PE=1 SV=1	0.00	0.00	0.00	0.00	0.00	3.07	0.00	0.00	0.00	0.00	0.00	0.00	0.00	0.00	0.00	0.00	0.00	0.00	0.00	0.00	1.04	0.00	0.00	0.00	0.26	0.09	>0.999999999999999	3.0
Q5W041	Armadillo repeat-containing protein 3 OS=Homo sapiens GN=ARMC3 PE=2 SV=2	0.00	0.00	0.00	0.00	0.00	0.71	0.00	0.00	0.00	0.00	0.00	0.00	0.00	0.00	0.00	0.00	0.00	0.00	0.00	0.00	0.00	0.00	0.00	0.00	0.06	0.00	>0.999999999999999	#DIV/0!
O43852	Calumenin OS=Homo sapiens GN=CALU PE=1 SV=2	0.00	0.00	0.00	0.00	0.00	2.13	0.00	0.00	0.00	0.00	0.00	3.34	2.12	0.00	0.00	0.00	0.00	0.00	0.00	1.17	1.52	0.00	0.00	0.36	0.46	0.43	>0.999999999999999	1.1
P58107	Epiplakin OS=Homo sapiens GN=EPPK1 PE=1 SV=2	0.00	0.00	0.00	0.00	0.00	0.24	0.00	0.09	0.00	0.00	0.00	0.16	0.00	0.00	0.00	0.00	0.00	0.00	0.00	0.13	0.00	0.00	0.00	0.00	0.04	0.01	>0.999999999999999	3.8
Q9GZU8	Protein FAM192A OS=Homo sapiens GN=FAM192A PE=1 SV=1	0.00	0.00	0.00	0.00	0.00	2.60	0.00	0.00	0.00	0.00	0.00	0.00	0.00	0.00	0.00	0.00	0.00	0.00	0.00	0.00	0.00	0.00	0.00	0.00	0.22	0.00	>0.999999999999999	#DIV/0!
P07305	Histone H1.0 OS=Homo sapiens GN=H1F0 PE=1 SV=3	0.00	0.00	0.00	0.00	0.00	3.78	0.00	0.00	0.00	0.00	0.00	0.00	0.00	0.00	0.00	0.00	0.00	0.00	0.00	0.00	0.00	0.00	0.00	0.00	0.32	0.00	>0.999999999999999	#DIV/0!
O00160	Unconventional myosin-If OS=Homo sapiens GN=MYO1F PE=1 SV=3	0.00	0.00	0.00	0.00	0.00	0.71	0.00	0.00	0.00	0.47	0.00	0.00	0.00	0.00	0.00	0.00	0.00	0.00	0.00	0.00	0.00	0.00	0.00	0.00	0.10	0.00	>0.999999999999999	#DIV/0!
O95236	Apolipoprotein L3 OS=Homo sapiens GN=APOL3 PE=2 SV=3	0.00	0.00	0.00	0.00	0.00	1.65	1.20	0.00	0.96	1.30	1.10	0.00	1.65	0.00	0.00	0.00	0.89	1.07	0.00	0.00	0.00	0.00	0.00	0.00	0.52	0.30	>0.999999999999999	1.7
Q9Y6A4	UPF0468 protein C16orf80 OS=Homo sapiens GN=C16orf80 PE=1 SV=1	0.00	0.00	0.00	0.00	0.00	3.54	0.00	0.00	0.00	0.00	0.00	0.00	0.00	0.00	0.00	0.00	0.00	0.00	0.00	0.00	0.00	0.00	0.00	0.00	0.30	0.00	>0.999999999999999	#DIV/0!
O60645	Exocyst complex component 3 OS=Homo sapiens GN=EXOC3 PE=1 SV=2	0.00	0.00	0.00	0.00	0.00	0.95	0.00	0.00	0.00	0.00	0.00	0.00	0.00	0.00	0.00	0.00	0.00	0.00	0.00	0.00	0.00	0.00	0.00	0.00	0.08	0.00	>0.999999999999999	#DIV/0!
Q96G28	Coiled-coil domain-containing protein 104 OS=Homo sapiens GN=CCDC104 PE=1 SV=2	0.00	0.00	0.00	0.00	0.00	1.89	0.00	0.00	0.00	0.00	0.00	0.00	0.00	0.00	0.00	0.00	0.00	0.00	0.00	0.00	0.00	0.00	0.00	0.00	0.16	0.00	>0.999999999999999	#DIV/0!
Q96MW1	Coiled-coil domain-containing protein 43 OS=Homo sapiens GN=CCDC43 PE=1 SV=2	0.00	0.00	0.00	0.00	0.00	3.07	0.00	0.00	0.00	2.41	2.04	0.00	0.00	2.09	0.00	0.00	0.00	0.00	0.00	0.00	0.00	2.24	0.00	0.52	0.63	0.40	>0.999999999999999	1.6
P62995	Transformer-2 protein homolog beta OS=Homo sapiens GN=TRA2B PE=1 SV=1	0.00	0.00	0.00	0.00	0.00	2.36	0.00	0.00	0.00	0.00	0.00	0.00	0.00	0.00	0.00	0.00	0.00	0.00	0.00	0.00	0.00	0.00	0.00	0.00	0.20	0.00	>0.999999999999999	#DIV/0!
P09012	U1 small nuclear ribonucleoprotein A OS=Homo sapiens GN=SNRPA PE=1 SV=3	0.00	0.00	0.00	0.00	0.00	2.60	1.88	0.00	0.00	2.04	1.73	0.00	0.00	0.00	0.00	0.00	0.00	0.00	0.00	0.00	0.00	0.00	0.00	0.00	0.69	0.00	>0.999999999999999	#DIV/0!
O00423	Echinoderm microtubule-associated protein-like 1 OS=Homo sapiens GN=EML1 PE=1 SV=3	0.00	0.00	0.00	0.00	0.00	0.95	0.00	0.00	0.00	0.00	0.00	0.00	0.00	0.00	0.00	0.00	0.00	0.00	0.00	0.00	0.00	0.00	0.00	0.00	0.08	0.00	>0.999999999999999	#DIV/0!
Q9UI30	tRNA methyltransferase 112 homolog OS=Homo sapiens GN=TRMT112 PE=1 SV=1	0.00	0.00	0.00	0.00	0.00	5.67	0.00	0.00	0.00	0.00	0.00	0.00	0.00	0.00	0.00	0.00	0.00	0.00	0.00	0.00	0.00	0.00	0.00	0.00	0.47	0.00	>0.999999999999999	#DIV/0!
O76041	Nebulette OS=Homo sapiens GN=NEBL PE=2 SV=1	0.00	0.00	0.00	0.00	0.00	0.71	0.00	0.00	0.00	0.00	0.00	0.00	0.00	0.00	0.00	0.00	0.00	0.00	0.00	0.00	0.00	0.00	0.00	0.00	0.06	0.00	>0.999999999999999	#DIV/0!
Q9UII2	ATPase inhibitor, mitochondrial OS=Homo sapiens GN=ATPIF1 PE=1 SV=1	0.00	0.00	0.00	0.00	0.00	6.62	0.00	0.00	0.00	5.19	4.40	2.28	0.00	0.00	0.00	0.00	0.00	0.00	0.00	0.00	0.00	0.00	0.00	1.12	1.54	0.09	>0.999999999999999	16.5
Q99816	Tumor susceptibility gene 101 protein OS=Homo sapiens GN=TSG101 PE=1 SV=2	0.00	0.00	0.00	0.00	0.00	1.65	0.00	0.00	0.00	0.00	0.00	0.00	0.00	0.00	3.82	0.00	0.00	0.00	0.00	0.00	0.00	0.00	2.27	0.00	0.14	0.51	>0.999999999999999	0.3
Q9C0E2	Exportin-4 OS=Homo sapiens GN=XPO4 PE=1 SV=2	0.00	0.00	0.00	0.00	0.00	0.47	0.00	0.00	0.00	0.00	0.00	0.00	0.00	0.00	0.00	0.00	0.00	0.00	0.00	0.00	0.00	0.00	0.00	0.00	0.04	0.00	>0.999999999999999	#DIV/0!
Q9BQG0	Myb-binding protein 1A OS=Homo sapiens GN=MYBBP1A PE=1 SV=2	0.00	0.00	0.00	0.00	0.00	0.47	0.00	0.00	0.00	0.00	0.00	0.00	0.00	0.00	0.00	0.00	0.00	0.00	0.00	0.00	0.00	0.00	0.00	0.00	0.04	0.00	>0.999999999999999	#DIV/0!
Q9H0N5	Pterin-4-alpha-carbinolamine dehydratase 2 OS=Homo sapiens GN=PCBD2 PE=1 SV=4	0.00	0.00	0.00	0.00	0.00	5.67	0.00	2.17	0.00	0.00	0.00	0.00	0.00	0.00	0.00	0.00	0.00	0.00	0.00	0.00	0.00	0.00	0.00	0.00	0.65	0.00	>0.999999999999999	#DIV/0!
O95816	BAG family molecular chaperone regulator 2 OS=Homo sapiens GN=BAG2 PE=1 SV=1	0.00	0.00	0.00	0.00	0.00	3.31	0.00	0.00	0.00	0.00	0.00	0.00	0.00	0.00	0.00	0.00	0.00	0.00	0.00	0.00	0.00	0.00	0.00	0.00	0.28	0.00	>0.999999999999999	#DIV/0!
P18754	Regulator of chromosome condensation OS=Homo sapiens GN=RCC1 PE=1 SV=1	0.00	0.00	0.00	0.00	0.00	1.65	0.00	0.00	0.00	0.00	0.00	0.00	0.00	0.00	0.00	0.00	0.00	0.00	0.00	0.00	0.00	0.00	0.00	0.00	0.14	0.00	>0.999999999999999	#DIV/0!
Q00978	Interferon regulatory factor 9 OS=Homo sapiens GN=IRF9 PE=1 SV=1	0.00	0.00	0.00	0.00	0.00	1.65	0.00	0.00	0.00	0.00	0.00	0.00	0.00	0.00	0.00	0.00	0.00	0.00	0.00	0.00	0.00	0.00	0.00	0.00	0.14	0.00	>0.999999999999999	#DIV/0!
Q92572	AP-3 complex subunit sigma-1 OS=Homo sapiens GN=AP3S1 PE=1 SV=1	0.00	0.00	0.00	0.00	0.00	3.54	0.00	0.00	0.00	0.00	0.00	0.00	0.00	0.00	0.00	0.00	0.00	0.00	0.00	0.00	0.00	0.00	0.00	0.00	0.30	0.00	>0.999999999999999	#DIV/0!
Q9BRK5	45 kDa calcium-binding protein OS=Homo sapiens GN=SDF4 PE=1 SV=1	0.00	0.00	0.00	0.00	0.00	1.89	0.00	0.72	2.19	0.00	0.00	0.65	0.00	0.00	0.00	0.00	1.01	1.23	1.45	1.04	1.35	1.28	1.14	0.32	0.45	0.73	>0.999999999999999	0.6
Q6P2E9	Enhancer of mRNA-decapping protein 4 OS=Homo sapiens GN=EDC4 PE=1 SV=1	0.00	0.00	0.00	0.00	0.00	0.47	0.00	0.00	0.00	0.00	0.00	0.00	0.00	0.00	0.00	0.00	0.00	0.00	0.00	0.00	0.00	0.00	0.00	0.00	0.04	0.00	>0.999999999999999	#DIV/0!
O75113	NEDD4-binding protein 1 OS=Homo sapiens GN=N4BP1 PE=1 SV=4	0.00	0.00	0.00	0.00	0.00	0.71	0.00	0.00	0.00	0.00	0.00	0.00	0.00	0.00	0.00	0.00	0.00	0.00	0.00	0.00	0.00	0.00	0.00	0.00	0.06	0.00	>0.999999999999999	#DIV/0!
Q9Y2X3	Nucleolar protein 58 OS=Homo sapiens GN=NOP58 PE=1 SV=1	0.00	0.00	0.00	0.00	0.00	1.18	0.85	0.00	0.00	0.00	0.00	0.00	0.00	0.00	0.00	0.00	0.00	0.00	0.00	0.00	0.00	0.00	0.00	0.00	0.17	0.00	>0.999999999999999	#DIV/0!
P01814	Ig heavy chain V-II region OU OS=Homo sapiens PE=1 SV=1	0.00	0.00	0.00	0.00	0.00	5.67	0.00	0.00	0.00	0.00	3.77	0.00	0.00	3.85	0.00	0.00	0.00	0.00	0.00	0.00	0.00	1.92	0.00	0.00	0.79	0.48	>0.999999999999999	1.6
Q7Z5P4	17-beta-hydroxysteroid dehydrogenase 13 OS=Homo sapiens GN=HSD17B13 PE=2 SV=1	0.00	0.00	0.00	0.00	0.00	2.36	0.00	0.00	0.00	0.00	1.57	0.00	0.00	0.00	0.00	0.00	0.00	0.00	0.00	0.00	0.00	0.00	0.00	0.00	0.33	0.00	>0.999999999999999	#DIV/0!
Q8NDA8	HEAT repeat-containing protein 7A OS=Homo sapiens GN=HEATR7A PE=1 SV=3	0.00	0.00	0.00	0.00	0.00	0.47	0.00	0.00	0.00	0.00	0.00	0.00	0.00	0.00	0.00	0.00	0.00	0.00	0.00	0.00	0.00	0.00	0.00	0.00	0.04	0.00	>0.999999999999999	#DIV/0!
Q9BTE6	Alanyl-tRNA editing protein Aarsd1 OS=Homo sapiens GN=AARSD1 PE=1 SV=2	0.00	0.00	0.00	0.00	0.00	1.65	0.00	0.00	0.00	0.00	0.00	0.00	0.00	0.00	0.00	0.00	0.00	0.00	0.00	0.00	0.00	0.00	0.00	0.00	0.14	0.00	>0.999999999999999	#DIV/0!
Q9Y237	Peptidyl-prolyl cis-trans isomerase NIMA-interacting 4 OS=Homo sapiens GN=PIN4 PE=1 SV=1	0.00	0.00	0.00	0.00	0.00	5.91	0.00	0.00	0.00	0.00	0.00	0.00	0.00	0.00	0.00	0.00	0.00	0.00	0.00	0.00	0.00	0.00	0.00	0.00	0.49	0.00	>0.999999999999999	#DIV/0!
P05023	Sodium/potassium-transporting ATPase subunit alpha-1 OS=Homo sapiens GN=ATP1A1 PE=1 SV=1	0.00	0.00	0.00	0.00	0.00	0.71	0.00	0.00	0.00	0.00	0.00	0.73	0.00	0.00	0.00	0.00	0.00	0.00	0.00	0.00	0.00	0.00	0.00	0.12	0.12	0.01	>0.999999999999999	12.0
Q9H169	Stathmin-4 OS=Homo sapiens GN=STMN4 PE=2 SV=1	0.00	0.00	0.00	0.00	0.00	3.54	0.00	0.00	0.00	0.00	0.00	0.00	0.00	0.00	0.00	0.00	0.00	0.00	0.00	0.00	0.00	0.00	0.00	0.00	0.30	0.00	>0.999999999999999	#DIV/0!
Q9GZZ1	N-alpha-acetyltransferase 50 OS=Homo sapiens GN=NAA50 PE=1 SV=1	0.00	0.00	0.00	0.00	0.00	4.02	2.91	0.00	0.00	3.15	0.00	0.00	0.00	0.00	0.00	0.00	0.00	0.00	0.00	0.00	0.00	2.41	0.00	0.00	0.84	0.20	>0.999999999999999	4.2
Q9BXJ9	N-alpha-acetyltransferase 15, NatA auxiliary subunit OS=Homo sapiens GN=NAA15 PE=1 SV=1	0.00	0.00	0.00	0.00	0.00	0.71	1.03	0.00	0.00	0.00	0.47	0.24	0.00	0.00	0.72	0.00	0.00	0.00	0.00	0.00	0.00	0.00	0.00	0.00	0.20	0.06	>0.999999999999999	3.4
Q01081	Splicing factor U2AF 35 kDa subunit OS=Homo sapiens GN=U2AF1 PE=1 SV=3	0.00	0.00	0.00	0.00	0.00	2.84	0.00	0.00	0.00	0.00	1.89	0.00	0.00	0.00	0.00	0.00	0.00	0.00	0.00	0.00	0.00	0.00	0.00	0.00	0.39	0.00	>0.999999999999999	#DIV/0!
Q14134	Tripartite motif-containing protein 29 OS=Homo sapiens GN=TRIM29 PE=1 SV=2	0.00	0.00	0.00	0.00	0.00	1.18	0.00	0.45	0.00	0.93	0.00	0.82	0.00	0.00	1.19	0.00	0.00	0.00	0.00	0.00	0.00	0.40	0.71	0.64	0.28	0.25	>0.999999999999999	1.1
P35269	General transcription factor IIF subunit 1 OS=Homo sapiens GN=GTF2F1 PE=1 SV=2	0.00	0.00	0.00	0.00	0.00	1.42	0.00	0.00	0.00	0.00	0.00	0.00	0.00	0.00	0.00	0.00	0.00	0.00	0.00	0.00	0.00	0.00	0.00	0.00	0.12	0.00	>0.999999999999999	#DIV/0!
Q9NQ39	Putative 40S ribosomal protein S10-like OS=Homo sapiens GN=RPS10P5 PE=5 SV=1	0.00	0.00	0.00	0.00	0.00	4.02	6.16	0.00	0.00	0.00	0.00	0.00	0.00	0.00	4.05	0.00	0.00	0.00	0.00	0.00	0.00	2.41	0.00	0.00	0.85	0.54	>0.999999999999999	1.6
O43504	Hepatitis B virus X-interacting protein OS=Homo sapiens GN=HBXIP PE=1 SV=1	0.00	0.00	0.00	0.00	0.00	8.51	0.00	3.25	0.00	0.00	0.00	0.00	0.00	0.00	0.00	0.00	0.00	0.00	0.00	0.00	0.00	0.00	0.00	0.00	0.98	0.00	>0.999999999999999	#DIV/0!
Q8WWY3	U4/U6 small nuclear ribonucleoprotein Prp31 OS=Homo sapiens GN=PRPF31 PE=1 SV=2	0.00	0.00	0.00	0.00	0.00	1.42	0.00	0.00	0.00	0.00	0.00	0.00	0.00	0.00	0.00	0.00	0.00	0.00	0.00	0.00	0.00	0.00	0.00	0.00	0.12	0.00	>0.999999999999999	#DIV/0!
P11684	Uteroglobin OS=Homo sapiens GN=SCGB1A1 PE=1 SV=1	0.00	0.00	0.00	0.00	0.00	8.03	0.00	3.07	0.00	0.00	0.00	0.00	0.00	0.00	0.00	0.00	0.00	5.22	0.00	4.41	0.00	0.00	0.00	0.00	0.93	0.80	>0.999999999999999	1.2
Q99798	Aconitate hydratase, mitochondrial OS=Homo sapiens GN=ACO2 PE=1 SV=2	0.00	0.00	0.00	0.00	0.00	0.95	0.00	0.00	0.00	0.00	0.00	1.31	0.00	0.00	0.00	0.00	0.00	0.00	0.00	0.00	0.00	0.00	0.57	0.16	0.19	0.06	>0.999999999999999	3.1
Q8NA03	Fibrous sheath-interacting protein 1 OS=Homo sapiens GN=FSIP1 PE=2 SV=1	0.00	0.00	0.00	0.00	0.00	1.18	0.00	0.00	0.00	0.00	0.79	0.00	0.00	0.00	0.00	0.00	0.00	0.00	0.00	0.00	0.00	0.00	0.00	0.00	0.16	0.00	>0.999999999999999	#DIV/0!
Q6P9B6	TLD domain-containing protein KIAA1609 OS=Homo sapiens GN=KIAA1609 PE=1 SV=2	0.00	0.00	0.00	0.00	0.00	1.42	0.00	0.00	0.00	0.00	0.00	0.00	0.00	0.00	0.00	0.00	0.00	0.00	0.00	0.00	0.00	0.00	0.00	0.00	0.12	0.00	>0.999999999999999	#DIV/0!
Q15018	BRISC complex subunit Abro1 OS=Homo sapiens GN=FAM175B PE=1 SV=2	0.00	0.00	0.00	0.00	0.00	1.65	1.20	0.00	0.00	1.30	1.10	0.57	1.65	0.00	0.00	0.00	0.00	0.00	0.00	0.00	0.00	0.00	0.00	0.00	0.48	0.14	>0.999999999999999	3.5
Q676U5	Autophagy-related protein 16-1 OS=Homo sapiens GN=ATG16L1 PE=1 SV=2	0.00	0.00	0.00	0.00	0.00	1.18	0.85	0.00	0.00	0.93	0.00	0.00	0.00	0.00	0.00	0.00	0.00	0.00	0.00	0.00	0.00	0.00	0.00	0.00	0.25	0.00	>0.999999999999999	#DIV/0!
Q9UNT1	Rab-like protein 2B OS=Homo sapiens GN=RABL2B PE=2 SV=1	0.00	0.00	0.00	0.00	0.00	3.07	0.00	0.00	0.00	0.00	0.00	0.00	0.00	0.00	0.00	0.00	0.00	0.00	0.00	0.00	0.00	0.00	0.00	0.00	0.26	0.00	>0.999999999999999	#DIV/0!
O14948	Transcription factor EC OS=Homo sapiens GN=TFEC PE=1 SV=1	0.00	0.00	0.00	0.00	0.00	1.89	0.00	0.00	0.00	0.00	1.26	0.00	0.00	0.00	0.00	0.00	0.00	0.00	0.00	0.00	0.00	0.64	0.00	0.00	0.26	0.05	>0.999999999999999	4.9
Q13510	Acid ceramidase OS=Homo sapiens GN=ASAH1 PE=1 SV=5	0.00	0.00	0.00	0.00	0.00	1.65	0.00	0.63	0.00	0.00	1.10	1.22	0.00	0.00	1.91	2.52	0.00	0.00	4.34	0.00	0.00	1.92	0.99	0.28	0.38	1.00	>0.999999999999999	0.4
Q92817	Envoplakin OS=Homo sapiens GN=EVPL PE=1 SV=3	0.00	0.00	0.00	0.00	0.00	0.24	0.00	0.00	0.00	0.00	0.00	0.00	0.00	0.00	0.00	0.00	0.00	0.00	0.00	0.00	0.00	0.00	0.00	0.00	0.02	0.00	>0.999999999999999	#DIV/0!
P55769	NHP2-like protein 1 OS=Homo sapiens GN=NHP2L1 PE=1 SV=3	0.00	0.00	0.00	0.00	0.00	5.67	0.00	0.00	0.00	0.00	3.77	1.96	0.00	0.00	0.00	0.00	0.00	0.00	0.00	0.00	0.00	1.92	0.00	0.00	0.95	0.16	>0.999999999999999	5.9
Q9NQC8	Intraflagellar transport protein 46 homolog OS=Homo sapiens GN=IFT46 PE=2 SV=1	0.00	0.00	0.00	0.00	0.00	2.36	0.00	0.00	0.00	0.00	3.15	0.00	0.00	0.00	0.00	0.00	0.00	0.00	0.00	0.00	0.00	0.00	0.00	0.00	0.46	0.00	>0.999999999999999	#DIV/0!
P30626	Sorcin OS=Homo sapiens GN=SRI PE=1 SV=1	0.00	0.00	0.00	0.00	0.00	3.54	0.00	0.00	0.00	0.00	0.00	0.00	0.00	0.00	0.00	0.00	0.00	0.00	0.00	0.00	0.00	0.00	0.00	0.00	0.30	0.00	>0.999999999999999	#DIV/0!
Q9UK99	F-box only protein 3 OS=Homo sapiens GN=FBXO3 PE=1 SV=3	0.00	0.00	0.00	0.00	0.00	1.42	0.00	0.00	0.00	0.00	0.00	0.00	0.00	0.00	0.00	0.00	0.00	0.00	0.00	0.00	0.00	0.00	0.85	0.00	0.12	0.07	>0.999999999999999	1.7
Q96RS6	NudC domain-containing protein 1 OS=Homo sapiens GN=NUDCD1 PE=1 SV=2	0.00	0.00	0.00	0.00	0.00	1.18	0.00	0.00	0.00	0.00	0.00	0.00	0.00	0.00	0.00	0.00	0.00	0.00	0.00	0.00	0.00	0.00	0.00	0.00	0.10	0.00	>0.999999999999999	#DIV/0!
Q6PII5	Hydroxyacylglutathione hydrolase-like protein OS=Homo sapiens GN=HAGHL PE=2 SV=1	0.00	0.00	0.00	0.00	0.00	2.36	0.00	0.00	0.00	0.00	0.00	0.00	0.00	0.00	0.00	0.00	0.00	0.00	0.00	0.00	0.00	0.00	0.00	0.00	0.20	0.00	>0.999999999999999	#DIV/0!
Q96JQ2	Calmin OS=Homo sapiens GN=CLMN PE=1 SV=1	0.00	0.00	0.00	0.00	0.00	0.71	0.00	0.00	0.00	0.00	0.00	0.00	0.00	0.00	0.00	0.00	0.00	0.00	0.00	0.00	0.00	0.00	0.00	0.00	0.06	0.00	>0.999999999999999	#DIV/0!
Q6PD74	Alpha- and gamma-adaptin-binding protein p34 OS=Homo sapiens GN=AAGAB PE=1 SV=1	0.00	0.00	0.00	0.00	0.00	2.13	0.00	0.00	0.00	0.00	0.00	0.73	0.00	0.00	0.00	0.00	0.00	0.00	0.00	0.00	0.00	0.00	0.00	0.00	0.24	0.00	>0.999999999999999	#DIV/0!
P36915	Guanine nucleotide-binding protein-like 1 OS=Homo sapiens GN=GNL1 PE=1 SV=2	0.00	0.00	0.00	0.00	0.00	1.18	0.00	0.00	0.00	0.00	0.00	0.00	0.00	0.00	0.00	0.00	0.00	0.00	0.00	0.00	0.00	0.00	0.00	0.20	0.10	0.02	>0.999999999999999	5.9
Q6P1M3	Lethal(2) giant larvae protein homolog 2 OS=Homo sapiens GN=LLGL2 PE=1 SV=2	0.00	0.00	0.00	0.00	0.00	0.71	0.00	0.00	0.00	0.00	0.00	0.00	0.00	0.00	0.00	0.00	0.00	0.00	0.00	0.00	0.00	0.00	0.00	0.00	0.06	0.00	>0.999999999999999	#DIV/0!
Q8IX12	Cell division cycle and apoptosis regulator protein 1 OS=Homo sapiens GN=CCAR1 PE=1 SV=2	0.00	0.00	0.00	0.00	0.00	0.47	0.00	0.00	0.00	0.00	0.00	0.00	0.00	0.00	0.00	0.00	0.00	0.00	0.00	0.00	0.00	0.00	0.00	0.00	0.04	0.00	>0.999999999999999	#DIV/0!
O96033	Molybdopterin synthase sulfur carrier subunit OS=Homo sapiens GN=MOCS2 PE=1 SV=1	0.00	0.00	0.00	0.00	0.00	8.51	0.00	0.00	0.00	0.00	0.00	0.00	0.00	5.78	0.00	0.00	0.00	0.00	0.00	0.00	0.00	0.00	0.00	0.00	0.71	0.48	>0.999999999999999	1.5
Q14980	Nuclear mitotic apparatus protein 1 OS=Homo sapiens GN=NUMA1 PE=1 SV=2	0.00	0.00	0.00	0.00	0.00	0.24	0.00	0.00	0.00	0.00	0.00	0.00	0.00	0.00	0.00	0.00	0.00	0.00	0.00	0.00	0.00	0.00	0.00	0.00	0.02	0.00	>0.999999999999999	#DIV/0!
Q9H4G4	Golgi-associated plant pathogenesis-related protein 1 OS=Homo sapiens GN=GLIPR2 PE=1 SV=3	0.00	0.00	0.00	0.00	0.00	4.73	0.00	0.00	0.00	0.00	0.00	0.00	0.00	0.00	0.00	0.00	0.00	0.00	0.00	0.00	0.00	0.00	0.00	0.00	0.39	0.00	>0.999999999999999	#DIV/0!
Q86X83	COMM domain-containing protein 2 OS=Homo sapiens GN=COMMD2 PE=1 SV=2	0.00	0.00	0.00	0.00	0.00	3.54	0.00	0.00	0.00	0.00	0.00	0.00	0.00	0.00	0.00	0.00	0.00	0.00	0.00	0.00	0.00	0.00	0.00	0.00	0.30	0.00	>0.999999999999999	#DIV/0!
Q8N752	Casein kinase I isoform alpha-like OS=Homo sapiens GN=CSNK1A1L PE=2 SV=2	0.00	0.00	0.00	0.00	0.00	1.89	0.00	0.00	0.00	0.00	0.00	0.00	0.00	0.00	0.00	0.00	0.00	0.00	0.00	0.00	0.00	0.00	0.00	0.00	0.16	0.00	>0.999999999999999	#DIV/0!
Q96HJ3	Coiled-coil domain-containing protein 34 OS=Homo sapiens GN=CCDC34 PE=2 SV=2	0.00	0.00	0.00	0.00	0.00	1.89	0.00	0.00	0.00	0.00	0.00	0.00	0.00	0.00	0.00	1.35	0.00	0.00	0.00	0.00	0.00	0.00	0.00	0.00	0.16	0.11	>0.999999999999999	1.4
Q712K3	Ubiquitin-conjugating enzyme E2 R2 OS=Homo sapiens GN=UBE2R2 PE=1 SV=1	0.00	0.00	0.00	0.00	0.00	2.84	0.00	0.00	0.00	0.00	0.00	0.00	0.00	0.00	0.00	0.00	0.00	0.00	0.00	0.00	0.00	0.00	0.00	0.00	0.24	0.00	>0.999999999999999	#DIV/0!
P55265	Double-stranded RNA-specific adenosine deaminase OS=Homo sapiens GN=ADAR PE=1 SV=4	0.00	0.00	0.00	0.00	0.00	0.47	0.00	0.00	0.00	0.00	0.00	0.00	0.00	0.00	0.00	0.00	0.00	0.00	0.00	0.00	0.00	0.00	0.00	0.00	0.04	0.00	>0.999999999999999	#DIV/0!
Q15286	Ras-related protein Rab-35 OS=Homo sapiens GN=RAB35 PE=1 SV=1	0.00	0.00	0.00	0.00	0.00	0.00	8.55	0.00	0.00	0.00	0.00	0.00	0.00	0.00	0.00	0.00	0.00	0.00	0.00	0.00	0.00	0.00	0.00	0.00	0.71	0.00	>0.999999999999999	#DIV/0!
P04278	Sex hormone-binding globulin OS=Homo sapiens GN=SHBG PE=1 SV=2	0.00	0.00	0.00	0.00	0.00	0.00	1.37	0.00	1.10	0.00	0.00	0.00	0.00	0.00	0.00	1.35	0.00	0.00	1.45	0.00	0.00	0.00	0.00	0.00	0.21	0.23	>0.999999999999999	0.9

P50502	Hsc70-interacting protein OS=Homo sapiens GN=ST13 PE=1 SV=2	0.00	0.00	0.00	0.00	0.00	0.00	8.04	0.00	0.00	0.00	5.66	0.00	0.00	0.00	0.00	0.00	0.00	0.00	0.00	0.00	0.00	0.00	0.00	0.00	1.14	0.00	>0.999999999999999	#DIV/0!
Q9Y6E2	Basic leucine zipper and W2 domain-containing protein 2 OS=Homo sapiens GN=BZW2 PE=1 SV=1	0.00	0.00	0.00	0.00	0.00	0.00	2.39	0.00	0.00	0.00	1.10	0.00	0.00	0.00	0.00	2.36	0.00	0.00	0.00	0.00	0.00	0.00	0.00	0.00	0.29	0.20	>0.999999999999999	1.5
P61956	Small ubiquitin-related modifier 2 OS=Homo sapiens GN=SUMO2 PE=1 SV=3	0.00	0.00	0.00	0.00	0.00	0.00	12.65	0.00	0.00	13.71	11.64	2.61	0.00	0.00	0.00	0.00	0.00	0.00	0.00	9.61	0.00	0.00	0.00	0.00	3.38	0.80	>0.999999999999999	4.2
P04222	HLA class I histocompatibility antigen, Cw-3 alpha chain OS=Homo sapiens GN=HLA-C PE=1 SV=2	0.00	0.00	0.00	0.00	0.00	0.00	2.91	0.00	0.00	0.00	0.00	0.00	0.00	0.00	0.00	0.00	0.00	0.00	0.00	0.00	0.00	0.00	0.00	0.00	0.24	0.00	>0.999999999999999	#DIV/0!
Q8TBF2	Prostamide/prostaglandin F synthase OS=Homo sapiens GN=FAM213B PE=2 SV=1	0.00	0.00	0.00	0.00	0.00	0.00	2.74	0.00	0.00	0.00	0.00	0.00	0.00	0.00	0.00	0.00	0.00	0.00	0.00	0.00	0.00	0.00	0.00	0.00	0.23	0.00	>0.999999999999999	#DIV/0!
Q969R2	Oxysterol-binding protein 2 OS=Homo sapiens GN=OSBP2 PE=1 SV=2	0.00	0.00	0.00	0.00	0.00	0.00	0.51	0.00	0.00	0.00	0.00	0.00	0.00	0.00	0.00	0.00	0.00	0.46	0.00	0.00	0.00	0.00	0.00	0.00	0.04	0.04	>0.999999999999999	1.1
Q9NSC7	Alpha-N-acetylgalactosaminide alpha-2,6-sialyltransferase 1 OS=Homo sapiens GN=ST6GALNAC1 PE=1 SV=1	0.00	0.00	0.00	0.00	0.00	0.00	0.85	0.00	0.68	0.00	0.00	0.00	0.00	0.00	0.00	0.00	0.00	0.00	0.00	0.00	0.00	0.00	0.00	0.00	0.13	0.00	>0.999999999999999	#DIV/0!
Q96PP8	Guanylate-binding protein 5 OS=Homo sapiens GN=GBP5 PE=2 SV=1	0.00	0.00	0.00	0.00	0.00	0.00	0.85	0.00	0.00	0.00	0.00	0.82	0.00	0.00	0.00	0.00	0.00	0.00	0.00	0.00	0.00	0.00	0.00	0.00	0.14	0.00	>0.999999999999999	#DIV/0!
P29728	2'-5'-oligoadenylate synthase 2 OS=Homo sapiens GN=OAS2 PE=1 SV=3	0.00	0.00	0.00	0.00	0.00	0.00	0.68	0.00	0.00	0.00	0.00	0.33	0.00	0.00	0.00	0.00	0.00	0.00	0.00	0.00	0.00	0.00	0.00	0.00	0.08	0.00	>0.999999999999999	#DIV/0!
Q9H269	Vacuolar protein sorting-associated protein 16 homolog OS=Homo sapiens GN=VPS16 PE=1 SV=2	0.00	0.00	0.00	0.00	0.00	0.00	0.51	0.00	0.00	0.00	0.00	0.00	0.00	0.00	0.00	0.00	0.00	0.00	0.00	0.00	0.00	0.00	0.00	0.00	0.04	0.00	>0.999999999999999	#DIV/0!
P00736	Complement C1r subcomponent OS=Homo sapiens GN=C1R PE=1 SV=2	0.00	0.00	0.00	0.00	0.00	0.00	0.68	0.00	0.00	0.00	0.00	0.65	0.00	0.00	0.00	0.67	1.64	0.00	2.35	0.00	0.00	0.32	0.00	0.16	0.11	0.43	>0.999999999999999	0.3
P07711	Cathepsin L1 OS=Homo sapiens GN=CTSL1 PE=1 SV=2	0.00	0.00	0.00	0.00	0.00	0.00	1.54	0.00	0.00	0.00	0.00	0.73	0.00	0.00	0.00	0.00	0.00	0.00	1.63	0.00	0.00	0.00	0.00	0.36	0.19	0.17	>0.999999999999999	1.1
Q5EBM0	UMP-CMP kinase 2, mitochondrial OS=Homo sapiens GN=CMPK2 PE=1 SV=3	0.00	0.00	0.00	0.00	0.00	0.00	1.20	0.00	0.00	0.00	0.00	0.00	0.00	0.00	0.00	0.00	0.00	0.00	0.00	0.00	0.00	0.00	0.00	0.00	0.10	0.00	>0.999999999999999	#DIV/0!
Q6ZN28	Metastasis-associated in colon cancer protein 1 OS=Homo sapiens GN=MACC1 PE=1 SV=2	0.00	0.00	0.00	0.00	0.00	0.00	0.51	0.00	0.00	0.00	0.00	0.00	0.00	0.00	0.00	0.00	0.00	0.00	0.00	0.00	0.00	0.00	0.00	0.00	0.04	0.00	>0.999999999999999	#DIV/0!
P00915	Carbonic anhydrase 1 OS=Homo sapiens GN=CA1 PE=1 SV=2	0.00	0.00	0.00	0.00	0.00	0.00	2.05	0.00	0.00	0.00	0.00	0.00	0.00	0.00	0.00	0.00	3.04	3.68	2.17	9.48	0.00	0.00	0.00	0.00	0.17	1.53	>0.999999999999999	0.1
Q92954	Proteoglycan 4 OS=Homo sapiens GN=PRG4 PE=1 SV=2	0.00	0.00	0.00	0.00	0.00	0.00	0.34	0.00	0.00	0.00	0.00	0.00	0.00	0.00	0.00	0.00	0.00	0.00	0.00	0.00	0.00	0.00	0.00	0.00	0.03	0.00	>0.999999999999999	#DIV/0!
Q9NP61	ADP-ribosylation factor GTPase-activating protein 3 OS=Homo sapiens GN=ARFGAP3 PE=1 SV=1	0.00	0.00	0.00	0.00	0.00	0.00	1.03	0.00	0.00	0.00	0.94	0.00	0.00	0.00	0.00	0.00	0.00	0.00	0.00	0.00	0.00	0.00	0.00	0.00	0.16	0.00	>0.999999999999999	#DIV/0!
Q9H0R4	Haloacid dehalogenase-like hydrolase domain-containing protein 2 OS=Homo sapiens GN=HDHD2 PE=1 SV=1	0.00	0.00	0.00	0.00	0.00	0.00	4.27	0.00	0.00	0.00	0.00	0.00	0.00	1.93	0.00	0.00	0.00	0.00	0.00	0.00	0.00	0.00	0.00	0.00	0.36	0.16	>0.999999999999999	2.2
Q13790	Apolipoprotein F OS=Homo sapiens GN=APOF PE=1 SV=2	0.00	0.00	0.00	0.00	0.00	0.00	1.54	0.00	1.23	1.67	0.00	0.00	2.12	0.00	0.00	0.00	1.14	1.38	0.00	2.47	0.00	0.72	0.00	0.00	0.37	0.65	>0.999999999999999	0.6
O43592	Exportin-T OS=Homo sapiens GN=XPOT PE=1 SV=2	0.00	0.00	0.00	0.00	0.00	0.00	0.51	0.00	0.00	0.00	0.00	0.00	0.00	0.00	0.00	0.00	0.00	0.00	0.00	0.00	0.00	0.24	0.00	0.00	0.04	0.02	>0.999999999999999	2.1
P42229	Signal transducer and activator of transcription 5A OS=Homo sapiens GN=STAT5A PE=1 SV=1	0.00	0.00	0.00	0.00	0.00	0.00	0.68	0.00	0.00	0.74	0.63	0.00	0.00	0.00	0.95	0.00	0.00	0.00	0.00	0.00	0.00	0.00	0.00	0.00	0.17	0.08	>0.999999999999999	2.2
Q96HW7	Integrator complex subunit 4 OS=Homo sapiens GN=INTS4 PE=1 SV=2	0.00	0.00	0.00	0.00	0.00	0.00	0.51	0.00	0.00	0.00	0.00	0.00	0.00	0.00	0.00	0.00	0.00	0.00	0.00	0.00	0.00	0.00	0.00	0.00	0.04	0.00	>0.999999999999999	#DIV/0!
Q9Y4B5	Coiled-coil domain-containing protein 165 OS=Homo sapiens GN=CCDC165 PE=1 SV=4	0.00	0.00	0.00	0.00	0.00	0.00	0.34	0.00	0.00	0.00	0.00	0.00	0.00	0.00	0.00	0.00	0.00	0.00	0.00	0.00	0.00	0.00	0.00	0.00	0.03	0.00	>0.999999999999999	#DIV/0!
P48059	LIM and senescent cell antigen-like-containing domain protein 1 OS=Homo sapiens GN=LIMS1 PE=1 SV=4	0.00	0.00	0.00	0.00	0.00	0.00	1.37	0.00	0.00	0.00	0.00	0.00	0.00	0.00	0.00	0.00	0.00	0.00	0.00	0.00	0.00	0.00	0.00	0.00	0.11	0.00	>0.999999999999999	#DIV/0!
Q9HBL8	NmrA-like family domain-containing protein 1 OS=Homo sapiens GN=NMRAL1 PE=1 SV=1	0.00	0.00	0.00	0.00	0.00	0.00	1.71	0.00	0.00	0.00	1.57	0.00	0.00	0.00	0.00	0.00	0.00	0.00	0.00	0.00	0.00	0.00	0.00	0.00	0.27	0.00	>0.999999999999999	#DIV/0!
Q13287	N-myc-interactor OS=Homo sapiens GN=NMI PE=1 SV=2	0.00	0.00	0.00	0.00	0.00	0.00	1.54	0.00	0.00	0.00	0.00	0.00	0.00	0.00	0.00	0.00	0.00	0.00	0.00	0.00	0.00	0.00	0.00	0.00	0.13	0.00	>0.999999999999999	#DIV/0!
P0C0S5	Histone H2A.Z OS=Homo sapiens GN=H2AFZ PE=1 SV=2	0.00	0.00	0.00	0.00	0.00	0.00	4.27	2.26	0.00	0.00	0.00	0.00	0.00	0.00	0.00	4.21	3.16	0.00	4.52	3.25	0.00	0.00	0.00	0.00	0.54	1.26	>0.999999999999999	0.4
P12259	Coagulation factor V OS=Homo sapiens GN=F5 PE=1 SV=4	0.00	0.00	0.00	0.00	0.00	0.00	0.00	0.81	0.55	0.00	0.00	0.90	0.00	0.48	0.00	0.50	0.13	0.15	0.00	0.00	0.00	0.40	0.00	0.20	0.19	0.16	>0.999999999999999	1.2
P16401	Histone H1.5 OS=Homo sapiens GN=HIST1H1B PE=1 SV=3	0.00	0.00	0.00	0.00	0.00	0.00	0.00	4.70	2.05	0.00	0.00	0.00	0.00	0.00	0.00	0.00	0.00	4.91	0.00	4.15	8.78	0.00	0.00	0.00	0.56	1.49	>0.999999999999999	0.4
P05120	Plasminogen activator inhibitor 2 OS=Homo sapiens GN=SERPINB2 PE=1 SV=2	0.00	0.00	0.00	0.00	0.00	0.00	0.00	0.63	3.15	0.00	0.00	0.00	0.00	0.00	0.00	0.00	0.00	2.30	4.16	0.00	8.44	0.56	2.13	0.60	0.32	1.52	>0.999999999999999	0.2
Q14118	Dystroglycan OS=Homo sapiens GN=DAG1 PE=1 SV=2	0.00	0.00	0.00	0.00	0.00	0.00	0.00	0.27	0.00	0.00	0.47	0.24	0.71	0.48	0.00	0.00	0.00	0.00	0.00	0.00	0.00	0.56	0.99	0.12	0.08	0.24	>0.999999999999999	0.3
O15145	Actin-related protein 2/3 complex subunit 3 OS=Homo sapiens GN=ARPC3 PE=1 SV=3	0.00	0.00	0.00	0.00	0.00	0.00	0.00	1.44	0.00	0.00	0.00	1.31	0.00	0.00	0.00	0.00	2.02	0.00	0.00	0.00	0.00	0.00	0.00	0.00	0.23	0.17	>0.999999999999999	1.4
Q92520	Protein FAM3C OS=Homo sapiens GN=FAM3C PE=1 SV=1	0.00	0.00	0.00	0.00	0.00	0.00	0.00	1.17	0.00	0.00	0.00	0.00	0.00	0.00	0.00	0.00	0.00	0.00	0.00	0.00	0.00	1.04	0.00	0.00	0.10	0.09	>0.999999999999999	1.1
Q9H173	Nucleotide exchange factor SIL1 OS=Homo sapiens GN=SIL1 PE=1 SV=1	0.00	0.00	0.00	0.00	0.00	0.00	0.00	0.54	0.00	0.00	0.00	0.00	0.00	0.00	0.00	0.00	0.00	0.00	0.00	0.00	0.00	0.00	0.00	0.00	0.05	0.00	>0.999999999999999	#DIV/0!
P16278	Beta-galactosidase OS=Homo sapiens GN=GLB1 PE=1 SV=2	0.00	0.00	0.00	0.00	0.00	0.00	0.00	0.36	0.00	0.00	0.00	0.00	0.00	0.00	0.00	0.67	0.00	0.00	0.00	0.00	0.00	0.32	0.00	0.00	0.03	0.08	>0.999999999999999	0.4
P68402	Platelet-activating factor acetylhydrolase IB subunit beta OS=Homo sapiens GN=PAFAH1B2 PE=1 SV=1	0.00	0.00	0.00	0.00	0.00	0.00	0.00	1.17	0.00	0.00	0.00	1.06	0.00	0.00	0.00	0.00	0.00	0.00	2.35	0.00	0.00	1.04	1.84	0.52	0.19	0.48	>0.999999999999999	0.4
O60613	15 kDa selenoprotein OS=Homo sapiens GN=SEP15 PE=1 SV=3	0.00	0.00	0.00	0.00	0.00	0.00	0.00	1.72	0.00	0.00	2.99	3.34	0.00	0.00	0.00	0.00	0.00	0.00	0.00	0.00	0.00	0.00	0.00	1.64	0.67	0.14	>0.999999999999999	4.9
O00592	Podocalyxin OS=Homo sapiens GN=PODXL PE=1 SV=2	0.00	0.00	0.00	0.00	0.00	0.00	0.00	0.54	0.82	0.00	0.00	0.49	0.00	0.00	0.00	0.00	0.00	0.92	0.00	0.00	1.86	0.00	0.85	0.24	0.15	0.32	>0.999999999999999	0.5
Q15782	Chitinase-3-like protein 2 OS=Homo sapiens GN=CHI3L2 PE=1 SV=1	0.00	0.00	0.00	0.00	0.00	0.00	0.00	0.72	0.00	0.00	0.00	0.00	0.00	0.00	0.00	0.00	0.00	0.00	0.00	0.00	0.00	0.64	0.00	0.00	0.06	0.05	>0.999999999999999	1.1
Q8NHW5	60S acidic ribosomal protein P0-like OS=Homo sapiens GN=RPLP0P6 PE=5 SV=1	0.00	0.00	0.00	0.00	0.00	0.00	0.00	0.90	2.74	0.00	0.00	0.00	0.00	0.00	0.00	0.00	0.00	3.07	0.00	0.00	1.52	0.00	0.00	0.00	0.30	0.38	>0.999999999999999	0.8
Q15233	Non-POU domain-containing octamer-binding protein OS=Homo sapiens GN=NONO PE=1 SV=4	0.00	0.00	0.00	0.00	0.00	0.00	0.00	0.54	0.00	0.00	0.00	0.00	0.00	0.00	0.00	0.00	0.00	0.00	0.00	0.00	0.00	0.00	0.00	0.00	0.05	0.00	>0.999999999999999	#DIV/0!
O75531	Barrier-to-autointegration factor OS=Homo sapiens GN=BANF1 PE=1 SV=1	0.00	0.00	0.00	0.00	0.00	0.00	0.00	3.07	0.00	0.00	0.00	0.00	0.00	0.00	0.00	0.00	0.00	0.00	0.00	0.00	0.00	0.00	0.00	0.00	0.26	0.00	>0.999999999999999	#DIV/0!
Q8NCL4	Polypeptide N-acetylgalactosaminyltransferase 6 OS=Homo sapiens GN=GALNT6 PE=2 SV=2	0.00	0.00	0.00	0.00	0.00	0.00	0.00	0.45	0.68	0.00	0.00	0.00	0.00	0.00	0.00	0.00	0.00	0.00	0.00	0.65	0.00	0.40	0.00	0.00	0.09	0.09	>0.999999999999999	1.1
P58499	Protein FAM3B OS=Homo sapiens GN=FAM3B PE=1 SV=2	0.00	0.00	0.00	0.00	0.00	0.00	0.00	1.17	0.00	0.00	0.00	0.00	0.00	0.00	0.00	0.00	0.00	0.00	0.00	0.00	0.00	2.16	0.00	0.52	0.10	0.22	>0.999999999999999	0.4
Q96BQ1	Protein FAM3D OS=Homo sapiens GN=FAM3D PE=1 SV=1	0.00	0.00	0.00	0.00	0.00	0.00	0.00	1.17	0.00	0.00	0.00	0.00	0.00	0.00	0.00	0.00	0.00	0.00	0.00	0.00	1.69	4.73	1.04	0.00	0.10	0.62	>0.999999999999999	0.2
P30040	Endoplasmic reticulum resident protein 29 OS=Homo sapiens GN=ERP29 PE=1 SV=4	0.00	0.00	0.00	0.00	0.00	0.00	0.00	0.99	0.00	0.00	0.00	1.96	0.00	0.00	0.00	1.85	0.00	0.00	0.00	0.00	1.43	0.00	0.00	0.44	0.25	0.31	>0.999999999999999	0.8
Q3KQV9	UDP-N-acetylhexosamine pyrophosphorylase-like protein 1 OS=Homo sapiens GN=UAP1L1 PE=2 SV=2	0.00	0.00	0.00	0.00	0.00	0.00	0.00	0.54	0.00	0.00	0.00	0.00	0.00	0.00	0.00	0.00	0.00	0.00	0.00	0.00	0.00	0.00	0.00	0.00	0.05	0.00	>0.999999999999999	#DIV/0!
Q8N4H5	Mitochondrial import receptor subunit TOM5 homolog OS=Homo sapiens GN=TOMM5 PE=1 SV=1	0.00	0.00	0.00	0.00	0.00	0.00	0.00	5.78	0.00	0.00	0.00	0.00	0.00	0.00	0.00	0.00	0.00	0.00	0.00	0.00	0.00	0.00	0.00	0.00	0.48	0.00	>0.999999999999999	#DIV/0!
Q15166	Serum paraoxonase/lactonase 3 OS=Homo sapiens GN=PON3 PE=1 SV=3	0.00	0.00	0.00	0.00	0.00	0.00	0.00	0.72	0.00	0.00	0.00	0.00	0.00	0.00	0.00	0.00	0.00	0.00	0.00	0.00	0.00	0.00	0.00	0.00	0.06	0.00	>0.999999999999999	#DIV/0!
Q96IY4	Carboxypeptidase B2 OS=Homo sapiens GN=CPB2 PE=1 SV=2	0.00	0.00	0.00	0.00	0.00	0.00	0.00	0.63	0.00	0.00	0.00	0.00	0.00	2.25	0.00	0.00	1.77	0.00	2.53	2.86	1.18	0.00	0.00	0.00	0.05	0.88	>0.999999999999999	0.1
Q96S96	Phosphatidylethanolamine-binding protein 4 OS=Homo sapiens GN=PEBP4 PE=1 SV=3	0.00	0.00	0.00	0.00	0.00	0.00	0.00	1.17	0.00	0.00	0.00	0.00	0.00	0.00	0.00	0.00	0.00	0.00	0.00	0.00	0.00	0.00	3.83	0.00	0.10	0.32	>0.999999999999999	0.3
Q6P4A8	Phospholipase B-like 1 OS=Homo sapiens GN=PLBD1 PE=1 SV=2	0.00	0.00	0.00	0.00	0.00	0.00	0.00	0.45	0.00	0.00	0.00	0.00	0.00	0.00	0.00	0.00	0.00	0.00	0.90	0.00	1.86	0.00	0.00	0.00	0.04	0.23	>0.999999999999999	0.2
Q9BZR9	Probable E3 ubiquitin-protein ligase TRIM8 OS=Homo sapiens GN=TRIM8 PE=2 SV=2	0.00	0.00	0.00	0.00	0.00	0.00	0.00	0.45	0.00	0.00	0.00	0.00	0.00	0.00	0.00	0.00	0.00	0.00	0.00	0.00	0.00	0.00	0.00	0.00	0.04	0.00	>0.999999999999999	#DIV/0!
O76031	ATP-dependent Clp protease ATP-binding subunit clpX-like, mitochondrial OS=Homo sapiens GN=CLPX PE=1 SV=	0.00	0.00	0.00	0.00	0.00	0.00	0.00	0.45	0.00	0.00	0.00	0.00	0.00	0.00	0.00	0.00	0.00	0.00	0.00	0.00	0.00	0.00	0.00	0.00	0.04	0.00	>0.999999999999999	#DIV/0!
Q6ZVX7	Non-specific cytotoxic cell receptor protein 1 homolog OS=Homo sapiens GN=NCCRP1 PE=1 SV=1	0.00	0.00	0.00	0.00	0.00	0.00	0.00	0.99	0.00	0.00	0.00	1.88	0.00	0.00	0.00	0.00	1.39	1.69	1.99	0.00	0.00	0.00	0.00	0.92	0.24	0.50	>0.999999999999999	0.5
P04066	Tissue alpha-L-fucosidase OS=Homo sapiens GN=FUCA1 PE=1 SV=4	0.00	0.00	0.00	0.00	0.00	0.00	0.00	0.54	0.00	0.00	0.00	0.49	0.00	0.00	0.00	1.01	0.00	0.00	0.00	0.00	1.01	0.00	0.00	0.00	0.09	0.17	>0.999999999999999	0.5
Q9BTY2	Plasma alpha-L-fucosidase OS=Homo sapiens GN=FUCA2 PE=1 SV=2	0.00	0.00	0.00	0.00	0.00	0.00	0.00	0.54	0.00	0.00	0.00	0.00	0.00	0.00	0.00	0.00	0.00	0.00	0.00	0.00	0.00	0.48	0.00	0.00	0.05	0.04	>0.999999999999999	1.1
P16870	Carboxypeptidase E OS=Homo sapiens GN=CPE PE=1 SV=1	0.00	0.00	0.00	0.00	0.00	0.00	0.00	0.54	0.00	0.00	0.00	0.00	0.00	0.00	0.00	0.00	0.00	0.00	0.00	0.00	0.00	1.04	0.00	0.00	0.05	0.09	>0.999999999999999	0.5
Q02383	Semenogelin-2 OS=Homo sapiens GN=SEMG2 PE=1 SV=1	0.00	0.00	0.00	0.00	0.00	0.00	0.00	0.45	0.00	0.00	0.00	0.00	0.00	0.00	0.00	0.00	0.00	0.00	0.00	0.00	1.69	0.00	0.71	0.00	0.04	0.20	>0.999999999999999	0.2
P35908	Keratin, type II cytoskeletal 2 epidermal OS=Homo sapiens GN=KRT2 PE=1 SV=2	0.00	0.00	0.00	0.00	0.00	0.00	0.00	0.00	3.01	13.15	5.35	2.77	0.00	4.50	0.00	0.00	0.00	0.00	0.00	0.00	5.74	2.24	0.00	1.92	2.02	1.20	>0.999999999999999	1.7
A6NMY6	Putative annexin A2-like protein OS=Homo sapiens GN=ANXA2P2 PE=5 SV=2	0.00	0.00	0.00	0.00	0.00	0.00	0.00	0.00	2.47	0.00	0.00	0.00	2.12	0.00	0.00	0.00	0.00	0.00	0.00	0.00	0.00	1.44	0.00	0.00	0.21	0.30	>0.999999999999999	0.7
Q9Y2T7	Y-box-binding protein 2 OS=Homo sapiens GN=YBX2 PE=1 SV=2	0.00	0.00	0.00	0.00	0.00	0.00	0.00	0.00	1.23	0.00	0.00	0.00	0.00	1.45	0.00	0.00	1.14	0.00	0.00	0.00	0.00	0.00	0.00	0.00	0.10	0.22	>0.999999999999999	0.5
P35858	Insulin-like growth factor-binding protein complex acid labile subunit OS=Homo sapiens GN=IGFALS PE=1 SV=1	0.00	0.00	0.00	0.00	0.00	0.00	0.00	0.00	2.19	0.00	0.00	0.41	1.18	0.00	1.19	0.84	0.00	0.00	0.00	0.65	0.00	0.00	0.00	0.20	0.22	0.34	>0.999999999999999	0.6
P59826	BPI fold-containing family B member 3 OS=Homo sapiens GN=BPIFB3 PE=2 SV=2	0.00	0.00	0.00	0.00	0.00	0.00	0.00	0.00	0.96	0.00	0.00	0.00	0.00	0.00	0.00	0.00	0.00	0.00	0.00	0.00	0.00	0.00	0.00	0.00	0.08	0.00	>0.999999999999999	#DIV/0!
P07998	Ribonuclease pancreatic OS=Homo sapiens GN=RNASE1 PE=1 SV=4	0.00	0.00	0.00	0.00	0.00	0.00	0.00	0.00	2.60	3.52	0.00	1.55	0.00	0.00	0.00	0.00	0.00	0.00	0.00	0.00	0.00	1.52	0.00	0.00	0.64	0.13	>0.999999999999999	5.1
O60361	Putative nucleoside diphosphate kinase OS=Homo sapiens GN=NME2P1 PE=5 SV=1	0.00	0.00	0.00	0.00	0.00	0.00	0.00	0.00	6.57	8.89	0.00	0.00	11.29	0.00	0.00	0.00	0.00	0.00	0.00	0.00	0.00	0.00	0.00	0.00	1.29	0.94	>0.999999999999999	1.4
Q86SR0	Secreted Ly-6/uPAR-related protein 2 OS=Homo sapiens GN=SLURP2 PE=2 SV=1	0.00	0.00	0.00	0.00	0.00	0.00	0.00	0.00	4.38	0.00	0.00	0.00	0.00	0.00	0.00	0.00	0.00	0.00	0.00	0.00	0.00	0.00	4.54	1.28	0.37	0.49	>0.999999999999999	0.8
P01767	Ig heavy chain V-III region BUT OS=Homo sapiens PE=1 SV=1	0.00	0.00	0.00	0.00	0.00	0.00	0.00	0.00	3.83	0.00	0.00	2.28	25.40	0.00	0.00	10.60	0.00	0.00	0.00	0.00	0.00	0.00	3.97	0.00	0.51	3.33	>0.999999999999999	0.2
P12724	Eosinophil cationic protein OS=Homo sapiens GN=RNASE3 PE=1 SV=2	0.00	0.00	0.00	0.00	0.00	0.00	0.00	0.00	2.47	0.00	0.00	0.00	0.00	0.00	0.00	0.00	0.00	0.00	0.00	0.00	0.00	0.00	0.00	0.00	0.21	0.00	>0.999999999999999	#DIV/0!
P02655	Apolipoprotein C-II OS=Homo sapiens GN=APOC2 PE=1 SV=1	0.00	0.00	0.00	0.00	0.00	0.00	0.00	0.00	9.59	0.00	0.00	0.00	0.00	0.00	0.00	0.00	0.00	0.00	0.00	3.89	0.00	0.00	0.00	0.00	0.80	0.32	>0.999999999999999	2.5
P20930	Filaggrin OS=Homo sapiens GN=FLG PE=1 SV=3	0.00	0.00	0.00	0.00	0.00	0.00	0.00	0.00	0.14	0.00	0.00	0.16	0.00	0.00	0.00	0.00	0.00	0.00	0.00	0.13	0.17	0.00	0.00	0.08	0.03	0.03	>0.999999999999999	0.8
Q8NI51	Transcriptional repressor CTCFL OS=Homo sapiens GN=CTCFL PE=1 SV=2	0.00	0.00	0.00	0.00	0.00	0.00	0.00	0.00	0.55	0.00	0.00	0.00	0.00	0.00	0.00	0.00	0.00	0.00	0.00	0.00	0.00	0.00	0.00	0.00	0.05	0.00	>0.999999999999999	#DIV/0!
Q99538	Legumain OS=Homo sapiens GN=LGMN PE=1 SV=1	0.00	0.00	0.00	0.00	0.00	0.00	0.00	0.00	0.96	0.00	0.00	0.57	0.00	0.00	0.00	0.00	0.00	0.00	0.00	0.00	0.00	0.00	0.00	0.28	0.13	0.02	>0.999999999999999	5.5
P63220	40S ribosomal protein S21 OS=Homo sapiens GN=RPS21 PE=1 SV=1	0.00	0.00	0.00	0.00	0.00	0.00	0.00	0.00	5.20	0.00	0.00	3.10	0.00	0.00	0.00	6.39	0.00	0.00	0.00	0.00	0.00	3.04	5.39	0.00	0.69	1.24	>0.999999999999999	0.6
Q9H361	Polyadenylate-binding protein 3 OS=Homo sapiens GN=PABPC3 PE=1 SV=2	0.00	0.00	0.00	0.00	0.00	0.00	0.00	0.00	0.68	0.00	0.00	0.41	0.00	0.00	0.00	0.00	0.00	1.53	0.00	0.00	0.00	0.40	0.00	0.00	0.09	0.16	>0.999999999999999	0.6
Q13617	Cullin-2 OS=Homo sapiens GN=CUL2 PE=1 SV=2	0.00	0.00	0.00	0.00	0.00	0.00	0.00	0.00	0.55	0.00	0.00	0.00	0.00	0.00	0.00	0.00	0.00	0.00	0.00	0.00	0.00	0.00	0.57	0.00	0.05	0.05	>0.999999999999999	1.0
Q13217	DnaJ homolog subfamily C member 3 OS=Homo sapiens GN=DNAJC3 PE=1 SV=1	0.00	0.00	0.00	0.00	0.00	0.00	0.00	0.00	0.82	0.00	0.00	0.98	0.00	0.00	0.00	0.00	0.76	0.00	1.09	0.00	0.00	0.00	0.00	0.24	0.15	0.17	>0.999999999999999	0.9
P15907	Beta-galactoside alpha-2,6-sialyltransferase 1 OS=Homo sapiens GN=ST6GAL1 PE=1 SV=1	0.00	0.00	0.00	0.00	0.00	0.00	0.00	0.00	0.96	0.00	0.00	0.00	0.00	0.00	0.00	0.00	0.00	0.00	0.00	0.00	0.00	0.00	0.00	0.00	0.08	0.00	>0.999999999999999	#DIV/0!
Q9BZK3	Putative nascent polypeptide-associated complex subunit alpha-like protein OS=Homo sapiens GN=NACAP1 PE=5	0.00	0.00	0.00	0.00	0.00	0.00	0.00	0.00	1.92	0.00	0.00	0.00	0.00	0.00	0.00	0.00	0.00	0.00	0.00	0.00	0.00	1.12	0.00	0.00	0.16	0.09	>0.999999999999999	1.7
Q68DL7	Uncharacterized protein C18orf63 OS=Homo sapiens GN=C18orf63 PE=2 SV=2	0.00	0.00	0.00	0.00	0.00	0.00	0.00	0.00	0.55	0.00	0.00	0.00	0.00	0.00	0.00	0.00	0.00	0.00	0.00	0.00	0.00	0.00	0.00	0.00	0.05	0.00	>0.999999999999999	#DIV/0!
Q6NUJ1	Proactivator polypeptide-like 1 OS=Homo sapiens GN=PSAPL1 PE=2 SV=2	0.00	0.00	0.00	0.00	0.00	0.00	0.00	0.00	0.82	0.00	0.94	0.00	0.00	0.00	0.00	0.00	0.00	0.00	0.00	0.00	0.00	0.00	0.00	0.00	0.15	0.00	>0.999999999999999	#DIV/0!
P07225	Vitamin K-dependent protein S OS=Homo sapiens GN=PROS1 PE=1 SV=1	0.00	0.00	0.00	0.00	0.00	0.00	0.00	0.00	0.55	0.00	0.00	0.33	0.00	0.00	0.00	0.00	0.00	0.00	0.00	0.00	0.00	0.00	0.00	0.16	0.07	0.01	>0.999999999999999	5.5
P01708	Ig lambda chain V-II region BUR OS=Homo sapiens PE=1 SV=1	0.00	0.00	0.00	0.00	0.00	0.00	0.00	0.00	4.11	0.00	0.00	0.00	0.00	0.00	0.00	0.00	3.80	0.00	0.00	0.00	0.00	0.00	0.00	0.00	0.34	0.32	>0.999999999999999	1.1
O75976	Carboxypeptidase D OS=Homo sapiens GN=CPD PE=1 SV=2	0.00	0.00	0.00	0.00	0.00	0.00	0.00	0.00	0.27	0.00	0.00	0.00	0.00	0.00	0.00	0.00	0.00	0.00	0.00	0.00	0.00	0.00	0.00	0.00	0.02	0.00	>0.999999999999999	#DIV/0!
Q7L9L4	MOB kinase activator 1B OS=Homo sapiens GN=MOB1B PE=1 SV=3	0.00	0.00	0.00	0.00	0.00	0.00	0.00	0.00	1.78	0.00	0.00	0.00	0.00	0.00	0.00	0.00	0.00	0.00	0.00	0.00	2.19	0.00	0.00	0.00	0.15	0.18	>0.999999999999999	0.8
Q16270	Insulin-like growth factor-binding protein 7 OS=Homo sapiens GN=IGFBP7 PE=1 SV=1	0.00	0.00	0.00	0.00	0.00	0.00	0.00	0.00	1.51	0.00	0.00	0.00	0.00	0.00	0.00	0.00	0.00	0.00	0.00	0.00	0.00	0.88	0.00	0.00	0.13	0.07	>0.999999999999999	1.7
P0CB38	Polyadenylate-binding protein 4-like OS=Homo sapiens GN=PABPC4L PE=2 SV=1	0.00	0.00	0.00	0.00	0.00	0.00	0.00	0.00	1.10	0.00	0.00	0.00	0.00	0.00	0.00	0.00	0.00	0.00	0.00	0.00	0.00	0.00	0.00	0.00	0.09	0.00	>0.999999999999999	#DIV/0!
Q4VCS5	Angiomotin OS=Homo sapiens GN=AMOT PE=1 SV=1	0.00	0.00	0.00	0.00	0.00	0.00	0.00	0.00	0.41	0.00	0.00	0.00	0.00	0.00	0.00	0.00	0.00	0.00	0.00	0.00	0.00	0.00	0.00	0.00	0.03	0.00	>0.999999999999999	#DIV/0!
Q8WU03	Glycine N-acyltransferase-like protein 2 OS=Homo sapiens GN=GLYATL2 PE=2 SV=1	0.00	0.00	0.00	0.00	0.00	0.00	0.00	0.00	1.37	1.85	0.00	0.00	0.00	0.00	0.00	0.00	1.27	0.00	0.00	0.00	0.00	0.00	0.00	0.00	0.27	0.11	>0.999999999999999	2.5
Q9NY65	Tubulin alpha-8 chain OS=Homo sapiens GN=TUBA8 PE=1 SV=1	0.00	0.00	0.00	0.00	0.00	0.00	0.00	0.00	0.00	14.08	0.00	0.00	0.00	0.00	0.00	0.00	0.00	0.00	0.00	0.00	0.00	0.00	0.00	0.00	1.17	0.00	>0.999999999999999	#DIV/0!
P02533	Keratin, type I cytoskeletal 14 OS=Homo sapiens GN=KRT14 PE=1 SV=4	0.00	0.00	0.00	0.00	0.00	0.00	0.00	0.00	0.00	8.34	0.00	0.00	0.00	0.00	0.00	0.00	4.56	1.99	8.14	0.00	12.49	4.31	0.00	1.80	0.69	2.77	>0.999999999999999	0.3
P19012	Keratin, type I cytoskeletal 15 OS=Homo sapiens GN=KRT15 PE=1 SV=3	0.00	0.00	0.00	0.00	0.00	0.00	0.00	0.00	0.00	7.04	0.00	0.00	0.00	0.00	0.00	0.00	0.00	0.00	0.00	0.00	0.00	0.00	0.00	1.52	0.59	0.13	>0.999999999999999	4.6
P13646	Keratin, type I cytoskeletal 13 OS=Homo sapiens GN=KRT13 PE=1 SV=4	0.00	0.00	0.00	0.00	0.00	0.00	0.00	0.00	0.00	5.37	0.00	0.00	0.00	0.00	0.00	0.00	0.00	0.00	0.00	0.00	4.89	0.00	0.00	1.88	0.45	0.56	>0.999999999999999	0.8
Q7RTS7	Keratin, type II cytoskeletal 74 OS=Homo sapiens GN=KRT74 PE=1 SV=2	0.00	0.00	0.00	0.00	0.00	0.00	0.00	0.00	0.00	2.22	0.00	0.00	0.00	0.00	0.00	0.00	0.00	0.00	0.00	0.00	0.00	0.00	0.00	0.00	0.19	0.00	>0.999999999999999	#DIV/0!
P17661	Desmin OS=Homo sapiens GN=DES PE=1 SV=3	0.00	0.00	0.00	0.00	0.00	0.00	0.00	0.00	0.00	2.41	0.00	0.00	0.00	0.00	0.00	0.00	0.00	0.00	0.00	0.00	0.00	0.00	0.00	0.00	0.20	0.00	>0.999999999999999	#DIV/0!
P61586	Transforming protein RhoA OS=Homo sapiens GN=RHOA PE=1 SV=1	0.00	0.00	0.00	0.00	0.00	0.00	0.00	0.00	0.00	9.82	0.00	0.00	0.00	0.00	0.00	0.00	0.00	0.00	0.00	0.00	0.00	0.00	0.00	2.12	0.82	0.18	>0.999999999999999	4.6
Q04323	UBX domain-containing protein 1 OS=Homo sapiens GN=UBXN1 PE=1 SV=2	0.00	0.00	0.00	0.00	0.00	0.00	0.00	0.00	0.00	1.85	0.00	0.00	0.00	0.00	2.38	0.00	0.00	0.00	1.81	0.00	0.00	0.00	0.00	0.00	0.15	0.35	>0.999999999999999	0.4
Q9NXA8	NAD-dependent lysine demalonylase and desuccinylase sirtuin-5, mitochondrial OS=Homo sapiens GN=SIRT5 PE=	0.00	0.00	0.00	0.00	0.00	0.00	0.00	0.00	0.00	1.85	1.57	0.00	0.00	0.00	0.00	0.00	0.00	0.00	0.00	0.00	0.00	0.00	0.00	0.00	0.29	0.00	>0.999999999999999	#DIV/0!
Q6ZTR7	Protein FAM92B OS=Homo sapiens GN=FAM92B PE=2 SV=1	0.00	0.00	0.00	0.00	0.00	0.00	0.00	0.00	0.00	3.71	1.42	0.00	0.00	0.00	0.00	0.00	0.00	0.00	0.00	0.00	0.00	0.00	0.00	0.00	0.43	0.00	>0.999999999999999	#DIV/0!
Q3YEC7	Rab-like protein 1 OS=Homo sapiens GN=PARF PE=1 SV=2	0.00	0.00	0.00	0.00	0.00	0.00	0.00	0.00	0.00	0.74	0.00	0.00	0.00	0.00	0.00	0.00	0.00	0.00	0.00	0.00	0.00	0.00	0.57	0.16	0.06	0.06	>0.999999999999999	1.0
Q15113	Procollagen C-endopeptidase enhancer 1 OS=Homo sapiens GN=PCOLCE PE=1 SV=2	0.00	0.00	0.00	0.00	0.00	0.00	0.00	0.00	0.00	1.30	0.00	0.00	0.00	0.00	0.00	1.18	0.00	0.00	0.00	0.00	0.00	0.00	0.00	0.00	0.11	0.10	>0.999999999999999	1.1
Q5T764	Interferon-induced protein with tetratricopeptide repeats 1B OS=Homo sapiens GN=IFIT1B PE=2 SV=1	0.00	0.00	0.00	0.00	0.00	0.00	0.00	0.00	0.00	1.11	0.00	0.00	0.00	0.00	0.00	0.00	0.00	0.00	0.00	0.00	0.00	0.00	0.00	0.24	0.09	0.02	>0.999999999999999	4.6
Q9Y2Y0	ADP-ribosylation factor-like protein 2-binding protein OS=Homo sapiens GN=ARL2BP PE=1 SV=1	0.00	0.00	0.00	0.00	0.00	0.00	0.00	0.00	0.00	3.33	2.83	0.00	0.00	0.00	0.00	0.00	0.00	0.00	0.00	0.00	0.00	1.44	0.00	0.00	0.51	0.12	>0.999999999999999	4.3
Q8IVF4	Dynein heavy chain 10, axonemal OS=Homo sapiens GN=DNAH10 PE=1 SV=4	0.00	0.00	0.00	0.00	0.00	0.00	0.00	0.00	0.00	0.19	0.00	0.00	0.00	0.00	0.00	0.00	0.00	0.00	0.00	0.00	0.00	0.00	0.00	0.00	0.02	0.00	>0.999999999999999	#DIV/0!
Q8NHS0	DnaJ homolog subfamily B member 8 OS=Homo sapiens GN=DNAJB8 PE=1 SV=1	0.00	0.00	0.00	0.00	0.00	0.00	0.00	0.00	0.00	2.41	0.00	0.00	0.00	0.00	0.00	0.00	0.00	0.00	0.00	0.00	0.00	0.00	0.00	0.00	0.20	0.00	>0.999999999999999	#DIV/0!
Q5T124	UBX domain-containing protein 11 OS=Homo sapiens GN=UBXN11 PE=2 SV=2	0.00	0.00	0.00	0.00	0.00	0.00	0.00	0.00	0.00	1.11	0.00	0.00	0.00	0.00	0.00	0.00	0.00	0.00	0.00	0.00	0.00	0.00	0.00	0.00	0.09	0.00	>0.999999999999999	#DIV/0!
Q9H1H9	Kinesin-like protein KIF13A OS=Homo sapiens GN=KIF13A PE=1 SV=2	0.00	0.00	0.00	0.00	0.00	0.00	0.00	0.00	0.00	0.37	0.31	0.00	0.00	0.00	0.00	0.00	0.00	0.00	0.00	0.00	0.00	0.00	0.00	0.08	0.06	0.01	>0.999999999999999	8.6
P45877	Peptidyl-prolyl cis-trans isomerase C OS=Homo sapiens GN=PPIC PE=1 SV=1	0.00	0.00	0.00	0.00	0.00	0.00	0.00	0.00	0.00	2.78	2.36	0.00	0.00	0.00	0.00	0.00	0.00	0.00	0.00	0.00	0.00	1.20	0.00	0.00	0.43	0.10	>0.999999999999999	4.3
P22792	Carboxypeptidase N subunit 2 OS=Homo sapiens GN=CPN2 PE=1 SV=3	0.00	0.00	0.00	0.00	0.00	0.00	0.00	0.00	0.00	0.93	0.00	0.41	0.00	0.00	0.00	0.84	0.63	0.00	0.00	0.00	0.00	0.00	0.00	0.00	0.11	0.12	>0.999999999999999	0.9
Q96I24	Far upstream element-binding protein 3 OS=Homo sapiens GN=FUBP3 PE=1 SV=2	0.00	0.00	0.00	0.00	0.00	0.00	0.00	0.00	0.00	0.93	0.00	0.00	0.00	0.00	0.00	0.00	0.00	0.00	0.00	0.00	0.00	0.00	0.00	0.00	0.08	0.00	>0.999999999999999	#DIV/0!
P59910	DnaJ homolog subfamily B member 13 OS=Homo sapiens GN=DNAJB13 PE=2 SV=1	0.00	0.00	0.00	0.00	0.00	0.00	0.00	0.00	0.00	1.67	0.00	0.00	0.00	0.00	0.00	0.00	0.00	0.00	0.00	0.00	0.00	0.00	0.00	0.00	0.14	0.00	>0.999999999999999	#DIV/0!
P46821	Microtubule-associated protein 1B OS=Homo sapiens GN=MAP1B PE=1 SV=2	0.00	0.00	0.00	0.00	0.00	0.00	0.00	0.00	0.00	0.19	0.31	0.00	0.00	0.00	0.00	0.00	0.00	0.00	0.00	0.00	0.00	0.00	0.00	0.00	0.04	0.00	>0.999999999999999	#DIV/0!
P60903	Protein S100-A10 OS=Homo sapiens GN=S100A10 PE=1 SV=2	0.00	0.00	0.00	0.00	0.00	0.00	0.00	0.00	0.00	5.93	0.00	0.00	0.00	0.00	0.00	0.00	0.00	0.00	0.00	0.00	0.00	0.00	0.00	0.00	0.49	0.00	>0.999999999999999	#DIV/0!
Q8N474	Secreted frizzled-related protein 1 OS=Homo sapiens GN=SFRP1 PE=1 SV=1	0.00	0.00	0.00	0.00	0.00	0.00	0.00	0.00	0.00	1.67	0.00	0.73	0.00	1.45	0.00	0.00	0.00	0.00	0.00	0.00	0.00	0.00	0.00	0.00	0.20	0.12	>0.999999999999999	1.7
P39880	Homeobox protein cut-like 1 OS=Homo sapiens GN=CUX1 PE=1 SV=3	0.00	0.00	0.00	0.00	0.00	0.00	0.00	0.00	0.00	0.37	0.00	0.00	0.00	0.00	0.00	0.00	0.00	0.00	0.00	0.00	0.00	0.00	0.00	0.00	0.03	0.00	>0.999999999999999	#DIV/0!
P49959	Double-strand break repair protein MRE11A OS=Homo sapiens GN=MRE11A PE=1 SV=3	0.00	0.00	0.00	0.00	0.00	0.00	0.00	0.00	0.00	0.74	0.63	0.00	0.00	0.00	0.00	0.00	0.00	0.00	0.00	0.00	0.00	0.00	0.00	0.00	0.11	0.00	>0.999999999999999	#DIV/0!
Q9UJA5	tRNA (adenine(58)-N(1))-methyltransferase non-catalytic subunit TRM6 OS=Homo sapiens GN=TRMT6 PE=1 SV=1	0.00	0.00	0.00	0.00	0.00	0.00	0.00	0.00	0.00	1.11	0.00	0.00	0.00	0.00	0.00	0.00	0.00	0.00	0.00	0.00	0.00	0.00	0.00	0.00	0.09	0.00	>0.999999999999999	#DIV/0!
Q9BZH6	WD repeat-containing protein 11 OS=Homo sapiens GN=WDR11 PE=1 SV=1	0.00	0.00	0.00	0.00	0.00	0.00	0.00	0.00	0.00	0.37	0.00	0.00	0.00	0.00	0.00	0.00	0.00	0.00	0.00	0.00	0.00	0.00	0.00	0.00	0.03	0.00	>0.999999999999999	#DIV/0!
Q5T6J7	Probable gluconokinase OS=Homo sapiens GN=C9orf103 PE=2 SV=1	0.00	0.00	0.00	0.00	0.00	0.00	0.00	0.00	0.00	2.96	2.52	0.00	0.00	0.00	0.00	0.00	0.00	0.00	0.00	0.00	0.00	0.00	0.00	0.00	0.46	0.00	>0.999999999999999	#DIV/0!
Q8N9W5	UPF0470 protein C19orf51 OS=Homo sapiens GN=C19orf51 PE=2 SV=3	0.00	0.00	0.00	0.00	0.00	0.00	0.00	0.00	0.00	1.11	0.00	0.00	0.00	0.00	0.00	0.00	0.00	0.00	0.00	0.00	0.00	0.00	0.00	0.00	0.09	0.00	>0.999999999999999	#DIV/0!
Q12933	TNF receptor-associated factor 2 OS=Homo sapiens GN=TRAF2 PE=1 SV=2	0.00	0.00	0.00	0.00	0.00	0.00	0.00	0.00	0.00	1.11	0.00	0.00	0.00	0.00	0.00	0.00	0.00	0.00	0.00	0.00	0.00	0.00	0.00	0.00	0.09	0.00	>0.999999999999999	#DIV/0!
Q99719	Septin-5 OS=Homo sapiens GN=SEPT5 PE=1 SV=1	0.00	0.00	0.00	0.00	0.00	0.00	0.00	0.00	0.00	1.48	0.00	0.00	0.00	0.00	0.00	0.00	0.00	0.00	0.00	0.00	0.00	0.00	0.00	0.00	0.12	0.00	>0.999999999999999	#DIV/0!
P98171	Rho GTPase-activating protein 4 OS=Homo sapiens GN=ARHGAP4 PE=1 SV=2	0.00	0.00	0.00	0.00	0.00	0.00	0.00	0.00	0.00	0.56	0.00	0.00	0.00	0.00	0.00	0.00	0.00	0.00	0.00	0.00	0.00	0.00	0.00	0.00	0.05	0.00	>0.999999999999999	#DIV/0!
Q8WUW1	Protein BRICK1 OS=Homo sapiens GN=BRK1 PE=1 SV=1	0.00	0.00	0.00	0.00	0.00	0.00	0.00	0.00	0.00	7.78	0.00	0.00	0.00	0.00	0.00	0.00	0.00	0.00	0.00	0.00	0.00	0.00	0.00	0.00	0.65	0.00	>0.999999999999999	#DIV/0!
Q8N6T3	ADP-ribosylation factor GTPase-activating protein 1 OS=Homo sapiens GN=ARFGAP1 PE=1 SV=2	0.00	0.00	0.00	0.00	0.00	0.00	0.00	0.00	0.00	1.30	0.00	1.22	0.00	0.00	0.00	0.00	0.00	0.00	0.00	0.00	0.00	0.00	0.00	0.00	0.21	0.00	>0.999999999999999	#DIV/0!
Q15047	Histone-lysine N-methyltransferase SETDB1 OS=Homo sapiens GN=SETDB1 PE=1 SV=1	0.00	0.00	0.00	0.00	0.00	0.00	0.00	0.00	0.00	0.37	0.00	0.00	0.00	0.00	0.48	0.00	0.00	0.00	0.00	0.00	0.00	0.00	0.00	0.00	0.03	0.04	>0.999999999999999	0.8
P01701	Ig lambda chain V-I region NEW OS=Homo sapiens PE=1 SV=1	0.00	0.00	0.00	0.00	0.00	0.00	0.00	0.00	0.00	5.56	0.00	0.00	0.00	0.00	0.00	0.00	8.73	0.00	0.00	0.00	0.00	0.00	0.00	0.00	0.46	0.73	>0.999999999999999	0.6
Q05519	Serine/arginine-rich splicing factor 11 OS=Homo sapiens GN=SRSF11 PE=1 SV=1	0.00	0.00	0.00	0.00	0.00	0.00	0.00	0.00	0.00	1.11	2.04	0.00	0.00	0.00	0.00	0.00	0.00	0.00	0.00	0.00	0.00	0.00	0.00	0.00	0.26	0.00	>0.999999999999999	#DIV/0!
Q3ZCM7	Tubulin beta-8 chain OS=Homo sapiens GN=TUBB8 PE=1 SV=2	0.00	0.00	0.00	0.00	0.00	0.00	0.00	0.00	0.00	0.00	3.30	0.00	0.00	0.00	0.00	0.00	0.00	0.00	0.00	0.00	0.00	0.00	0.00	0.00	0.28	0.00	>0.999999999999999	#DIV/0!
Q17RN3	Protein FAM98C OS=Homo sapiens GN=FAM98C PE=2 SV=1	0.00	0.00	0.00	0.00	0.00	0.00	0.00	0.00	0.00	0.00	4.40	0.00	0.00	0.00	4.29	0.00	0.00	0.00	0.00	0.00	0.00	0.00	1.28	0.00	0.37	0.46	>0.999999999999999	0.8
Q96DM3	Uncharacterized protein C18orf8 OS=Homo sapiens GN=C18orf8 PE=1 SV=2	0.00	0.00	0.00	0.00	0.00	0.00	0.00	0.00	0.00	0.00	0.63	0.00	0.00	0.00	0.00	0.00	0.00	0.00	0.00	0.00	0.00	0.00	0.00	0.00	0.05	0.00	>0.999999999999999	#DIV/0!
Q92538	Golgi-specific brefeldin A-resistance guanine nucleotide exchange factor 1 OS=Homo sapiens GN=GBF1 PE=1 SV=	0.00	0.00	0.00	0.00	0.00	0.00	0.00	0.00	0.00	0.00	0.47	0.00	0.00	0.00	0.00	0.00	0.00	0.00	0.00	0.00	0.00	0.00	0.00	0.00	0.04	0.00	>0.999999999999999	#DIV/0!
P23193	Transcription elongation factor A protein 1 OS=Homo sapiens GN=TCEA1 PE=1 SV=2	0.00	0.00	0.00	0.00	0.00	0.00	0.00	0.00	0.00	0.00	3.15	0.00	0.00	0.00	0.00	0.00	0.00	0.00	0.00	0.00	0.00	0.00	0.00	0.00	0.26	0.00	>0.999999999999999	#DIV/0!
Q9NZJ9	Diphosphoinositol polyphosphate phosphohydrolase 2 OS=Homo sapiens GN=NUDT4 PE=1 SV=2	0.00	0.00	0.00	0.00	0.00	0.00	0.00	0.00	0.00	0.00	2.52	0.00	0.00	0.00	0.00	0.00	0.00	0.00	0.00	0.00	0.00	0.00	0.00	0.00	0.21	0.00	>0.999999999999999	#DIV/0!
Q9Y2Q3	Glutathione S-transferase kappa 1 OS=Homo sapiens GN=GSTK1 PE=1 SV=3	0.00	0.00	0.00	0.00	0.00	0.00	0.00	0.00	0.00	0.00	2.04	2.28	0.00	2.09	0.00	0.00	0.00	0.00	0.00	0.00	0.00	0.00	0.00	0.52	0.36	0.22	>0.999999999999999	1.7
Q4V328	GRIP1-associated protein 1 OS=Homo sapiens GN=GRIPAP1 PE=1 SV=1	0.00	0.00	0.00	0.00	0.00	0.00	0.00	0.00	0.00	0.00	1.10	0.00	0.00	0.00	0.00	0.00	0.00	0.00	0.00	0.00	0.00	0.00	0.00	0.00	0.09	0.00	>0.999999999999999	#DIV/0!
Q9BZL1	Ubiquitin-like protein 5 OS=Homo sapiens GN=UBL5 PE=1 SV=1	0.00	0.00	0.00	0.00	0.00	0.00	0.00	0.00	0.00	0.00	6.29	0.00	0.00	0.00	0.00	0.00	0.00	0.00	0.00	0.00	0.00	0.00	0.00	0.00	0.52	0.00	>0.999999999999999	#DIV/0!
P22307	Non-specific lipid-transfer protein OS=Homo sapiens GN=SCP2 PE=1 SV=2	0.00	0.00	0.00	0.00	0.00	0.00	0.00	0.00	0.00	0.00	1.73	0.49	0.00	0.00	0.00	0.00	0.00	0.00	0.00	0.00	0.00	0.00	0.85	0.24	0.18	0.09	>0.999999999999999	2.0
Q92934	Bcl2 antagonist of cell death OS=Homo sapiens GN=BAD PE=1 SV=3	0.00	0.00	0.00	0.00	0.00	0.00	0.00	0.00	0.00	0.00	2.83	0.00	0.00	0.00	0.00	0.00	0.00	0.00	0.00	0.00	0.00	0.00	0.00	0.00	0.24	0.00	>0.999999999999999	#DIV/0!
O75896	Tumor suppressor candidate 2 OS=Homo sapiens GN=TUSC2 PE=1 SV=3	0.00	0.00	0.00	0.00	0.00	0.00	0.00	0.00	0.00	0.00	4.40	0.00	0.00	0.00	0.00	0.00	0.00	0.00	0.00	0.00	0.00	0.00	0.00	0.00	0.37	0.00	>0.999999999999999	#DIV/0!
Q9NQT8	Kinesin-like protein KIF13B OS=Homo sapiens GN=KIF13B PE=1 SV=1	0.00	0.00	0.00	0.00	0.00	0.00	0.00	0.00	0.00	0.00	0.31	0.00	0.00	0.00	0.00	0.00	0.00	0.00	0.00	0.00	0.00	0.00	0.28	0.08	0.03	0.03	>0.999999999999999	0.9
Q9Y2X7	ARF GTPase-activating protein GIT1 OS=Homo sapiens GN=GIT1 PE=1 SV=2	0.00	0.00	0.00	0.00	0.00	0.00	0.00	0.00	0.00	0.00	0.63	0.00	0.00	0.00	0.95	0.00	0.00	0.00	0.00	0.00	0.00	0.00	0.00	0.00	0.05	0.08	>0.999999999999999	0.7
O14772	Fucose-1-phosphate guanylyltransferase OS=Homo sapiens GN=FPGT PE=1 SV=2	0.00	0.00	0.00	0.00	0.00	0.00	0.00	0.00	0.00	0.00	0.79	0.00	0.00	0.00	0.00	0.00	0.00	0.00	0.00	0.00	0.00	0.40	0.00	0.00	0.07	0.03	>0.999999999999999	2.0
P49770	Translation initiation factor eIF-2B subunit beta OS=Homo sapiens GN=EIF2B2 PE=1 SV=3	0.00	0.00	0.00	0.00	0.00	0.00	0.00	0.00	0.00	0.00	1.26	0.00	0.00	0.00	0.00	0.00	0.00	0.00	0.00	0.00	0.00	0.00	0.00	0.00	0.10	0.00	>0.999999999999999	#DIV/0!
O00139	Kinesin-like protein KIF2A OS=Homo sapiens GN=KIF2A PE=1 SV=3	0.00	0.00	0.00	0.00	0.00	0.00	0.00	0.00	0.00	0.00	0.63	0.00	0.00	0.00	0.00	0.00	0.00	0.00	0.00	0.00	0.00	0.00	0.00	0.00	0.05	0.00	>0.999999999999999	#DIV/0!
O00186	Syntaxin-binding protein 3 OS=Homo sapiens GN=STXBP3 PE=1 SV=2	0.00	0.00	0.00	0.00	0.00	0.00	0.00	0.00	0.00	0.00	1.57	0.00	0.00	0.00	0.00	0.00	0.00	0.00	0.00	0.00	0.00	0.00	0.00	0.00	0.13	0.00	>0.999999999999999	#DIV/0!
Q96L92	Sorting nexin-27 OS=Homo sapiens GN=SNX27 PE=1 SV=2	0.00	0.00	0.00	0.00	0.00	0.00	0.00	0.00	0.00	0.00	0.79	0.00	0.00	0.00	0.00	0.00	0.00	0.00	0.00	0.00	0.00	0.00	0.00	0.00	0.07	0.00	>0.999999999999999	#DIV/0!
Q9UPN7	Serine/threonine-protein phosphatase 6 regulatory subunit 1 OS=Homo sapiens GN=PPP6R1 PE=1 SV=5	0.00	0.00	0.00	0.00	0.00	0.00	0.00	0.00	0.00	0.00	0.47	0.00	0.00	0.00	0.00	0.00	0.00	0.00	0.00	0.00	0.00	0.00	0.00	0.00	0.04	0.00	>0.999999999999999	#DIV/0!
Q16775	Hydroxyacylglutathione hydrolase, mitochondrial OS=Homo sapiens GN=HAGH PE=1 SV=2	0.00	0.00	0.00	0.00	0.00	0.00	0.00	0.00	0.00	0.00	1.57	0.00	0.00	0.00	0.00	0.00	0.00	0.00	0.00	0.00	0.00	0.00	0.00	0.00	0.13	0.00	>0.999999999999999	#DIV/0!
Q4V339	COBW domain-containing protein 6 OS=Homo sapiens GN=CBWD6 PE=2 SV=1	0.00	0.00	0.00	0.00	0.00	0.00	0.00	0.00	0.00	0.00	1.10	0.00	0.00	0.00	0.00	0.00	0.00	0.00	0.00	0.00	0.00	0.00	0.00	0.00	0.09	0.00	>0.999999999999999	#DIV/0!
Q5W0U4	B box and SPRY domain-containing protein OS=Homo sapiens GN=BSPRY PE=2 SV=1	0.00	0.00	0.00	0.00	0.00	0.00	0.00	0.00	0.00	0.00	1.10	0.00	0.00	0.00	0.00	0.00	0.00	0.00	0.00	0.00	0.00	0.00	0.00	0.00	0.09	0.00	>0.999999999999999	#DIV/0!
Q07021	Complement component 1 Q subcomponent-binding protein, mitochondrial OS=Homo sapiens GN=C1QBP PE=1 S\	0.00	0.00	0.00	0.00	0.00	0.00	0.00	0.00	0.00	0.00	1.57	2.85	0.00	0.00	0.00	1.68	0.00	0.00	0.00	0.00	0.00	0.80	0.00	0.40	0.37	0.24	>0.999999999999999	1.5
O75190	DnaJ homolog subfamily B member 6 OS=Homo sapiens GN=DNAJB6 PE=1 SV=2	0.00	0.00	0.00	0.00	0.00	0.00	0.00	0.00	0.00	0.00	1.42	0.00	0.00	0.00	0.00	0.00	0.00	0.00	0.00	0.00	0.00	0.00	0.00	0.00	0.12	0.00	>0.999999999999999	#DIV/0!
A2RUB1	Uncharacterized protein C17orf104 OS=Homo sapiens GN=C17orf104 PE=2 SV=3	0.00	0.00	0.00	0.00	0.00	0.00	0.00	0.00	0.00	0.00	0.47	0.00	0.00	0.00	0.00	0.00	0.00	0.00	0.00	0.00	0.00	0.00	0.00	0.00	0.04	0.00	>0.999999999999999	#DIV/0!
O43490	Prominin-1 OS=Homo sapiens GN=PROM1 PE=1 SV=1	0.00	0.00	0.00	0.00	0.00	0.00	0.00	0.00	0.00	0.00	1.10	0.24	0.00	0.00	0.00	0.00	0.00	0.00	0.00	0.00	0.00	0.00	0.00	0.00	0.11	0.00	>0.999999999999999	#DIV/0!
Q9UQB8	Brain-specific angiogenesis inhibitor 1-associated protein 2 OS=Homo sapiens GN=BAIAP2 PE=1 SV=1	0.00	0.00	0.00	0.00	0.00	0.00	0.00	0.00	0.00	0.00	0.79	0.00	0.00	0.00	0.00	0.00	0.00	0.00	0.00	0.00	0.00	0.40	0.71	0.00	0.07	0.09	>0.999999999999999	0.7
Q9ULT8	E3 ubiquitin-protein ligase HECTD1 OS=Homo sapiens GN=HECTD1 PE=1 SV=3	0.00	0.00	0.00	0.00	0.00	0.00	0.00	0.00	0.00	0.00	0.16	0.00	0.00	0.00	0.00	0.00	0.00	0.00	0.00	0.00	0.00	0.00	0.00	0.00	0.01	0.00	>0.999999999999999	#DIV/0!
Q8IV38	Ankyrin repeat and MYND domain-containing protein 2 OS=Homo sapiens GN=ANKMY2 PE=1 SV=1	0.00	0.00	0.00	0.00	0.00	0.00	0.00	0.00	0.00	0.00	1.10	0.00	0.00	0.00	0.00	0.00	0.00	0.00	0.00	0.00	0.00	0.00	0.00	0.00	0.09	0.00	>0.999999999999999	#DIV/0!
P50897	Palmitoyl-protein thioesterase 1 OS=Homo sapiens GN=PPT1 PE=1 SV=1	0.00	0.00	0.00	0.00	0.00	0.00	0.00	0.00	0.00	0.00	1.57	0.00	0.00	0.00	0.00	0.00	0.00	0.00	0.00	0.00	0.00	0.00	0.00	0.40	0.13	0.03	>0.999999999999999	3.9
O00193	Small acidic protein OS=Homo sapiens GN=SMAP PE=1 SV=1	0.00	0.00	0.00	0.00	0.00	0.00	0.00	0.00	0.00	0.00	2.52	1.31	0.00	0.00	0.00	0.00	0.00	0.00	0.00	0.00	0.00	0.00	0.00	0.00	0.32	0.00	>0.999999999999999	#DIV/0!
Q9HD26	Golgi-associated PDZ and coiled-coil motif-containing protein OS=Homo sapiens GN=GOPC PE=1 SV=1	0.00	0.00	0.00	0.00	0.00	0.00	0.00	0.00	0.00	0.00	0.94	0.00	0.00	0.00	0.00	0.00	0.00	0.00	0.00	0.00	0.00	0.00	0.00	0.00	0.08	0.00	>0.999999999999999	#DIV/0!
Q5VTQ0	Tetratricopeptide repeat protein 39B OS=Homo sapiens GN=TTC39B PE=2 SV=3	0.00	0.00	0.00	0.00	0.00	0.00	0.00	0.00	0.00	0.00	0.79	0.00	0.00	0.00	0.00	0.00	0.00	0.00	0.00	0.00	0.00	0.00	0.00	0.00	0.07	0.00	>0.999999999999999	#DIV/0!
P35241	Radixin OS=Homo sapiens GN=RDX PE=1 SV=1	0.00	0.00	0.00	0.00	0.00	0.00	0.00	0.00	0.00	0.00	0.79	0.00	0.00	0.00	0.00	0.00	0.00	0.00	0.00	0.00	0.00	0.00	0.00	0.00	0.07	0.00	>0.999999999999999	#DIV/0!
Q9HBY8	Serine/threonine-protein kinase Sgk2 OS=Homo sapiens GN=SGK2 PE=1 SV=1	0.00	0.00	0.00	0.00	0.00	0.00	0.00	0.00	0.00	0.00	1.10	0.00	0.00	0.00	0.00	0.00	0.00	0.00	0.00	0.00	0.00	0.00	0.00	0.00	0.09	0.00	>0.999999999999999	#DIV/0!
P40818	Ubiquitin carboxyl-terminal hydrolase 8 OS=Homo sapiens GN=USP8 PE=1 SV=1	0.00	0.00	0.00	0.00	0.00	0.00	0.00	0.00	0.00	0.00	0.47	0.00	0.00	0.00	0.00	0.00	0.00	0.00	0.00	0.00	0.00	0.00	0.00	0.00	0.04	0.00	>0.999999999999999	#DIV/0!
P50402	Emerin OS=Homo sapiens GN=EMD PE=1 SV=1	0.00	0.00	0.00	0.00	0.00	0.00	0.00	0.00	0.00	0.00	1.73	0.00	0.00	0.00	0.00	0.00	0.00	0.00	0.00	0.00	0.00	0.00	0.00	0.00	0.14	0.00	>0.999999999999999	#DIV/0!
O95359	Transforming acidic coiled-coil-containing protein 2 OS=Homo sapiens GN=TACC2 PE=1 SV=3	0.00	0.00	0.00	0.00	0.00	0.00	0.00	0.00	0.00	0.00	0.16	0.00	0.00	0.00	0.00	0.00	0.00	0.00	0.00	0.00	0.00	0.00	0.00	0.04	0.01	0.00	>0.999999999999999	3.9
P50238	Cysteine-rich protein 1 OS=Homo sapiens GN=CRIP1 PE=1 SV=3	0.00	0.00	0.00	0.00	0.00	0.00	0.00	0.00	0.00	0.00	6.13	0.00	0.00	0.00	0.00	0.00	0.00	0.00	0.00	0.00	0.00	0.00	0.00	0.00	0.51	0.00	>0.999999999999999	#DIV/0!
Q96GG9	DCN1-like protein 1 OS=Homo sapiens GN=DCUN1D1 PE=1 SV=1	0.00	0.00	0.00	0.00	0.00	0.00	0.00	0.00	0.00	0.00	1.73	0.90	0.00	1.77	0.00	0.00	0.00	0.00	0.00	0.00	0.00	0.88	1.56	0.44	0.22	0.39	>0.999999999999999	0.6
Q9Y4F5	Protein KIAA0284 OS=Homo sapiens GN=KIAA0284 PE=1 SV=4	0.00	0.00	0.00	0.00	0.00	0.00	0.00	0.00	0.00	0.00	0.31	0.00	0.00	0.00	0.00	0.00	0.00	0.00	0.00	0.00	0.00	0.00	0.00	0.00	0.03	0.00	>0.999999999999999	#DIV/0!
A0AV96	RNA-binding protein 47 OS=Homo sapiens GN=RBM47 PE=1 SV=2	0.00	0.00	0.00	0.00	0.00	0.00	0.00	0.00	0.00	0.00	0.79	0.00	0.00	0.00	0.00	0.00	0.00	0.00	0.00	0.00	0.00	0.00	0.71	0.00	0.07	0.06	>0.999999999999999	1.1
Q6ZU80	Centrosomal protein of 128 kDa OS=Homo sapiens GN=CEP128 PE=1 SV=2	0.00	0.00	0.00	0.00	0.00	0.00	0.00	0.00	0.00	0.00	0.47	0.00	0.00	0.00	0.00	0.00	0.00	0.00	0.00	0.00	0.00	0.00	0.00	0.00	0.04	0.00	>0.999999999999999	#DIV/0!
Q8NF50	Dedicator of cytokinesis protein 8 OS=Homo sapiens GN=DOCK8 PE=1 SV=3	0.00	0.00	0.00	0.00	0.00	0.00	0.00	0.00	0.00	0.00	0.16	0.00	0.00	0.00	0.00	0.00	0.00	0.00	0.00	0.00	0.00	0.00	0.00	0.00	0.01	0.00	>0.999999999999999	#DIV/0!
P48378	DNA-binding protein RFX2 OS=Homo sapiens GN=RFX2 PE=2 SV=2	0.00	0.00	0.00	0.00	0.00	0.00	0.00	0.00	0.00	0.00	0.63	0.00	0.00	0.00	0.00	0.00	0.00	0.00	0.00	0.00	0.00	0.00	0.00	0.00	0.05	0.00	>0.999999999999999	#DIV/0!
Q9UBD9	Cardiotrophin-like cytokine factor 1 OS=Homo sapiens GN=CLCF1 PE=1 SV=1	0.00	0.00	0.00	0.00	0.00	0.00	0.00	0.00	0.00	0.00	2.04	0.00	0.00	0.00	0.00	0.00	0.00	0.00	0.00	0.00	0.00	0.00	0.00	0.00	0.17	0.00	>0.999999999999999	#DIV/0!
Q9Y2W2	WW domain-binding protein 11 OS=Homo sapiens GN=WBP11 PE=1 SV=1	0.00	0.00	0.00	0.00	0.00	0.00	0.00	0.00	0.00	0.00	0.79	0.00	0.00	0.00	0.00	0.00	0.00	0.00	0.00	0.00	0.00	0.00	0.00	0.00	0.07	0.00	>0.999999999999999	#DIV/0!
Q9Y5B8	Nucleoside diphosphate kinase 7 OS=Homo sapiens GN=NME7 PE=1 SV=1	0.00	0.00	0.00	0.00	0.00	0.00	0.00	0.00	0.00	0.00	1.26	0.00	0.00	0.00	0.00	0.00	0.00	0.00	0.00	0.00	0.00	0.00	0.00	0.00	0.10	0.00	>0.999999999999999	#DIV/0!
Q9HCD5	Nuclear receptor coactivator 5 OS=Homo sapiens GN=NCOA5 PE=1 SV=2	0.00	0.00	0.00	0.00	0.00	0.00	0.00	0.00	0.00	0.00	0.79	0.00	0.00	0.00	0.00	0.00	0.00	0.00	0.00	0.00	0.00	0.00	0.00	0.00	0.07	0.00	>0.999999999999999	#DIV/0!
O43924	Retinal rod rhodopsin-sensitive cGMP 3',5'-cyclic phosphodiesterase subunit delta OS=Homo sapiens GN=PDE6D F	0.00	0.00	0.00	0.00	0.00	0.00	0.00	0.00	0.00	0.00	2.99	0.00	0.00	0.00	4.53	0.00	0.00	0.00	0.00	0.00	0.00	0.00	0.00	0.00	0.25	0.38	>0.999999999999999	0.7
P05091	Aldehyde dehydrogenase, mitochondrial OS=Homo sapiens GN=ALDH2 PE=1 SV=2	0.00	0.00	0.00	0.00	0.00	0.00	0.00	0.00	0.00	0.00	0.00	2.04	0.00	0.00	0.00	0.00	0.00	0.00	0.00	0.00	0.00	0.00	0.00	0.72	0.17	0.06	>0.999999999999999	2.8
Q9Y4L1	Hypoxia up-regulated protein 1 OS=Homo sapiens GN=HYOU1 PE=1 SV=1	0.00	0.00	0.00	0.00	0.00	0.00	0.00	0.00	0.00	0.00	0.00	1.55	0.00	0.00	0.00	0.00	0.00	0.00	0.00	0.00	0.00	0.24	0.00	0.64	0.13	0.07	>0.999999999999999	1.8
P30479	HLA class I histocompatibility antigen, B-41 alpha chain OS=Homo sapiens GN=HLA-B PE=1 SV=1	0.00	0.00	0.00	0.00	0.00	0.00	0.00	0.00	0.00	0.00	0.00	3.02	0.00	0.00	0.00	0.00	0.00	0.00	0.00	0.00	0.00	0.00	0.00	0.00	0.25	0.00	>0.999999999999999	#DIV/0!
P10314	HLA class I histocompatibility antigen, A-32 alpha chain OS=Homo sapiens GN=HLA-A PE=2 SV=2	0.00	0.00	0.00	0.00	0.00	0.00	0.00	0.00	0.00	0.00	0.00	2.12	0.00	0.00	0.00	0.00	0.00	0.00	0.00	0.00	0.00	0.00	0.00	0.00	0.18	0.00	>0.999999999999999	#DIV/0!
Q99935	Proline-rich protein 1 OS=Homo sapiens GN=PROL1 PE=1 SV=2	0.00	0.00	0.00	0.00	0.00	0.00	0.00	0.00	0.00	0.00	0.00	2.12	9.64	0.00	6.20	0.00	1.52	1.84	0.00	0.00	0.00	0.00	1.70	1.04	0.18	1.83	>0.999999999999999	0.1
P67775	Serine/threonine-protein phosphatase 2A catalytic subunit alpha isoform OS=Homo sapiens GN=PPP2CA PE=1 SV:	0.00	0.00	0.00	0.00	0.00	0.00	0.00	0.00	0.00	0.00	0.00	3.43	0.00	0.00	0.00	0.00	0.00	0.00	0.00	0.00	0.00	0.00	0.00	0.00	0.29	0.00	>0.999999999999999	#DIV/0!
Q9UHJ6	Sedoheptulokinase OS=Homo sapiens GN=SHPK PE=1 SV=3	0.00	0.00	0.00	0.00	0.00	0.00	0.00	0.00	0.00	0.00	0.00	2.28	0.00	0.00	0.00	0.00	0.00	0.00	0.00	0.00	0.00	0.48	0.85	0.52	0.19	0.15	>0.999999999999999	1.2
P13804	Electron transfer flavoprotein subunit alpha, mitochondrial OS=Homo sapiens GN=ETFA PE=1 SV=1	0.00	0.00	0.00	0.00	0.00	0.00	0.00	0.00	0.00	0.00	0.00	2.53	0.00	0.00	0.00	0.00	0.00	0.00	0.00	0.00	0.00	0.00	0.00	0.00	0.21	0.00	>0.999999999999999	#DIV/0!
P06576	ATP synthase subunit beta, mitochondrial OS=Homo sapiens GN=ATP5B PE=1 SV=3	0.00	0.00	0.00	0.00	0.00	0.00	0.00	0.00	0.00	0.00	0.00	0.98	0.00	0.00	0.00	0.00	0.00	0.00	0.00	0.00	0.00	0.00	0.00	0.24	0.08	0.02	>0.999999999999999	4.1
P51659	Peroxisomal multifunctional enzyme type 2 OS=Homo sapiens GN=HSD17B4 PE=1 SV=3	0.00	0.00	0.00	0.00	0.00	0.00	0.00	0.00	0.00	0.00	0.00	1.39	0.00	0.00	0.00	0.00	0.00	0.00	0.00	0.00	0.00	0.00	0.00	0.16	0.12	0.01	>0.999999999999999	8.7
O75882	Attractin OS=Homo sapiens GN=ATRN PE=1 SV=2	0.00	0.00	0.00	0.00	0.00	0.00	0.00	0.00	0.00	0.00	0.00	0.49	0.00	0.00	0.00	0.00	0.25	0.31	0.00	0.00	0.34	0.00	0.00	0.00	0.04	0.07	>0.999999999999999	0.5
O95716	Ras-related protein Rab-3D OS=Homo sapiens GN=RAB3D PE=1 SV=1	0.00	0.00	0.00	0.00	0.00	0.00	0.00	0.00	0.00	0.00	0.00	3.83	0.00	0.00	0.00	0.00	0.00	0.00	0.00	0.00	0.00	0.00	0.00	1.16	0.32	0.10	>0.999999999999999	3.3
P61026	Ras-related protein Rab-10 OS=Homo sapiens GN=RAB10 PE=1 SV=1	0.00	0.00	0.00	0.00	0.00	0.00	0.00	0.00	0.00	0.00	0.00	4.16	7.53	0.00	0.00	0.00	4.05	0.00	0.00	0.00	0.00	0.00	7.24	1.28	0.35	1.67	>0.999999999999999	0.2
Q99714	3-hydroxyacyl-CoA dehydrogenase type-2 OS=Homo sapiens GN=HSD17B10 PE=1 SV=3	0.00	0.00	0.00	0.00	0.00	0.00	0.00	0.00	0.00	0.00	0.00	2.12	0.00	0.00	0.00	0.00	0.00	0.00	0.00	0.00	0.00	0.00	0.00	0.48	0.18	0.04	>0.999999999999999	4.4
O14907	Tax1-binding protein 3 OS=Homo sapiens GN=TAX1BP3 PE=1 SV=2	0.00	0.00	0.00	0.00	0.00	0.00	0.00	0.00	0.00	0.00	0.00	2.04	0.00	0.00	0.00	0.00	0.00	0.00	0.00	0.00	0.00	0.00	0.00	1.00	0.17	0.08	>0.999999999999999	2.0
Q9NUJ1	Abhydrolase domain-containing protein 10, mitochondrial OS=Homo sapiens GN=ABHD10 PE=1 SV=1	0.00	0.00	0.00	0.00	0.00	0.00	0.00	0.00	0.00	0.00	0.00	1.63	0.00	0.00	0.00	0.00	0.00	0.00	0.00	0.00	0.00	0.00	0.00	0.00	0.14	0.00	>0.999999999999999	#DIV/0!
Q9Y371	Endophilin-B1 OS=Homo sapiens GN=SH3GLB1 PE=1 SV=1	0.00	0.00	0.00	0.00	0.00	0.00	0.00	0.00	0.00	0.00	0.00	1.39	0.00	0.00	0.00	0.00	0.00	0.00	0.00	0.00	0.00	0.64	1.14	0.00	0.12	0.15	>0.999999999999999	0.8
Q9H2C0	Gigaxonin OS=Homo sapiens GN=GAN PE=1 SV=1	0.00	0.00	0.00	0.00	0.00	0.00	0.00	0.00	0.00	0.00	0.00	0.41	0.00	0.00	0.00	0.00	0.00	0.00	0.00	0.00	0.00	0.00	1.42	0.20	0.03	0.13	>0.999999999999999	0.3
P25705	ATP synthase subunit alpha, mitochondrial OS=Homo sapiens GN=ATP5A1 PE=1 SV=1	0.00	0.00	0.00	0.00	0.00	0.00	0.00	0.00	0.00	0.00	0.00	0.90	0.00	0.00	0.00	0.84	0.00	0.00	0.00	0.00	0.00	0.00	0.00	0.68	0.07	0.13	>0.999999999999999	0.6
Q9UBS4	DnaJ homolog subfamily B member 11 OS=Homo sapiens GN=DNAJB11 PE=1 SV=1	0.00	0.00	0.00	0.00	0.00	0.00	0.00	0.00	0.00	0.00	0.00	0.65	0.00	0.00	0.00	0.00	0.00	0.00	0.00	0.00	0.00	0.64	0.00	0.00	0.05	0.05	>0.999999999999999	1.0
Q9HCY8	Protein S100-A14 OS=Homo sapiens GN=S100A14 PE=1 SV=1	0.00	0.00	0.00	0.00	0.00	0.00	0.00	0.00	0.00	0.00	0.00	2.37	0.00	0.00	0.00	4.88	0.00	0.00	0.00	0.00	0.00	0.00	0.00	1.16	0.20	0.50	>0.999999999999999	0.4
Q16698	2,4-dienoyl-CoA reductase, mitochondrial OS=Homo sapiens GN=DECR1 PE=1 SV=1	0.00	0.00	0.00	0.00	0.00	0.00	0.00	0.00	0.00	0.00	0.00	1.55	0.00	0.00	0.00	0.00	0.00	0.00	0.00	0.00	0.00	0.00	0.00	0.36	0.13	0.03	>0.999999999999999	4.3
Q9BQG2	Peroxisomal NADH pyrophosphatase NUDT12 OS=Homo sapiens GN=NUDT12 PE=1 SV=1	0.00	0.00	0.00	0.00	0.00	0.00	0.00	0.00	0.00	0.00	0.00	0.49	0.00	0.00	0.00	0.00	0.00	0.00	0.00	0.00	0.00	0.00	0.00	0.00	0.04	0.00	>0.999999999999999	#DIV/0!
Q8IVU3	Probable E3 ubiquitin-protein ligase HERC6 OS=Homo sapiens GN=HERC6 PE=2 SV=2	0.00	0.00	0.00	0.00	0.00	0.00	0.00	0.00	0.00	0.00	0.00	0.24	0.00	0.00	0.00	0.00	0.00	0.00	0.00	0.00	0.00	0.00	0.00	0.00	0.02	0.00	>0.999999999999999	#DIV/0!
Q9P0J7	E3 ubiquitin-protein ligase KCMF1 OS=Homo sapiens GN=KCMF1 PE=1 SV=2	0.00	0.00	0.00	0.00	0.00	0.00	0.00	0.00	0.00	0.00	0.00	0.65	0.00	0.00	0.00	0.00	0.00	0.00	0.00	0.00	0.00	0.00	0.00	0.00	0.05	0.00	>0.999999999999999	#DIV/0!
Q96I99	Succinyl-CoA ligase [GDP-forming] subunit beta, mitochondrial OS=Homo sapiens GN=SUCLG2 PE=1 SV=2	0.00	0.00	0.00	0.00	0.00	0.00	0.00	0.00	0.00	0.00	0.00	0.57	0.00	0.00	0.00	0.00	0.00	0.00	0.00	0.00	0.00	0.00	0.00	0.00	0.05	0.00	>0.999999999999999	#DIV/0!
Q9Y6N5	Sulfide:quinone oxidoreductase, mitochondrial OS=Homo sapiens GN=SQRDL PE=1 SV=1	0.00	0.00	0.00	0.00	0.00	0.00	0.00	0.00	0.00	0.00	0.00	1.71	0.00	0.00	0.00	0.00	0.00	0.00	0.00	0.00	0.00	0.00	0.00	0.00	0.14	0.00	>0.999999999999999	#DIV/0!
P42785	Lysosomal Pro-X carboxypeptidase OS=Homo sapiens GN=PRCP PE=1 SV=1	0.00	0.00	0.00	0.00	0.00	0.00	0.00	0.00	0.00	0.00	0.00	0.49	0.00	0.00	0.00	0.00	0.00	0.00	0.00	0.00	0.00	0.00	0.00	0.24	0.04	0.02	>0.999999999999999	2.0
P35030	Trypsin-3 OS=Homo sapiens GN=PRSS3 PE=1 SV=2	0.00	0.00	0.00	0.00	0.00	0.00	0.00	0.00	0.00	0.00	0.00	0.82	0.00	0.00	0.00	0.00	0.00	0.00	0.00	0.00	1.69	0.00	0.00	0.00	0.07	0.14	>0.999999999999999	0.5
Q9BX68	Histidine triad nucleotide-binding protein 2, mitochondrial OS=Homo sapiens GN=HINT2 PE=1 SV=1	0.00	0.00	0.00	0.00	0.00	0.00	0.00	0.00	0.00	0.00	0.00	1.63	0.00	0.00	0.00	0.00	0.00	0.00	0.00	0.00	0.00	0.00	0.00	0.00	0.14	0.00	>0.999999999999999	#DIV/0!
Q5VV41	Rho guanine nucleotide exchange factor 16 OS=Homo sapiens GN=ARHGEF16 PE=1 SV=1	0.00	0.00	0.00	0.00	0.00	0.00	0.00	0.00	0.00	0.00	0.00	0.33	0.00	0.00	0.00	0.00	0.00	0.00	0.00	0.00	0.00	0.00	0.00	0.16	0.03	0.01	>0.999999999999999	2.0
Q8NFL0	UDP-GlcNAc:betaGal beta-1,3-N-acetylglucosaminyltransferase 7 OS=Homo sapiens GN=B3GNT7 PE=2 SV=1	0.00	0.00	0.00	0.00	0.00	0.00	0.00	0.00	0.00	0.00	0.00	0.57	0.00	0.00	0.00	0.00	0.00	0.00	0.00	0.00	0.00	0.00	0.00	0.00	0.05	0.00	>0.999999999999999	#DIV/0!
Q86X29	Lipolysis-stimulated lipoprotein receptor OS=Homo sapiens GN=LSR PE=1 SV=4	0.00	0.00	0.00	0.00	0.00	0.00	0.00	0.00	0.00	0.00	0.00	0.73	0.00	0.00	0.00	0.00	0.00	0.00	0.00	0.00	0.00	0.00	0.00	0.20	0.06	0.02	>0.999999999999999	3.7
P54819	Adenylate kinase 2, mitochondrial OS=Homo sapiens GN=AK2 PE=1 SV=2	0.00	0.00	0.00	0.00	0.00	0.00	0.00	0.00	0.00	0.00	0.00	2.20	0.00	0.00	0.00	0.00	0.00	0.00	0.00	0.00	0.00	0.00	0.00	0.00	0.18	0.00	>0.999999999999999	#DIV/0!
O15254	Peroxisomal acyl-coenzyme A oxidase 3 OS=Homo sapiens GN=ACOX3 PE=1 SV=2	0.00	0.00	0.00	0.00	0.00	0.00	0.00	0.00	0.00	0.00	0.00	0.33	0.00	0.00	0.00	0.00	0.00	0.00	0.00	0.00	0.00	0.00	0.00	0.00	0.03	0.00	>0.999999999999999	#DIV/0!
O95831	Apoptosis-inducing factor 1, mitochondrial OS=Homo sapiens GN=AIFM1 PE=1 SV=1	0.00	0.00	0.00	0.00	0.00	0.00	0.00	0.00	0.00	0.00	0.00	0.41	0.00	0.00	0.00	0.00	0.00	0.00	0.00	0.00	0.00	0.00	0.00	0.00	0.03	0.00	>0.999999999999999	#DIV/0!
P01764	Ig heavy chain V-III region VH26 OS=Homo sapiens PE=1 SV=1	0.00	0.00	0.00	0.00	0.00	0.00	0.00	0.00	0.00	0.00	0.00	2.20	0.00	0.00	0.00	0.00	3.42	0.00	0.00	0.00	0.00	0.00	0.00	0.00	0.18	0.28	>0.999999999999999	0.6
P01772	Ig heavy chain V-III region KOL OS=Homo sapiens PE=1 SV=1	0.00	0.00	0.00	0.00	0.00	0.00	0.00	0.00	0.00	0.00	0.00	1.96	0.00	0.00	0.00	0.00	0.00	3.68	4.34	0.00	0.00	0.00	3.41	0.96	0.16	1.03	>0.999999999999999	0.2
O75390	Citrate synthase, mitochondrial OS=Homo sapiens GN=CS PE=1 SV=2	0.00	0.00	0.00	0.00	0.00	0.00	0.00	0.00	0.00	0.00	0.00	1.06	0.00	0.00	0.00	0.00	0.00	0.00	0.00	0.00	0.00	0.00	0.00	0.52	0.09	0.04	>0.999999999999999	2.0
P54802	Alpha-N-acetylglucosaminidase OS=Homo sapiens GN=NAGLU PE=1 SV=2	0.00	0.00	0.00	0.00	0.00	0.00	0.00	0.00	0.00	0.00	0.00	0.65	0.00	0.00	0.00	0.00	0.00	0.00	0.00	0.00	0.00	0.32	0.00	0.16	0.05	0.04	>0.999999999999999	1.4
P31040	Succinate dehydrogenase [ubiquinone] flavoprotein subunit, mitochondrial OS=Homo sapiens GN=SDHA PE=1 SV=	0.00	0.00	0.00	0.00	0.00	0.00	0.00	0.00	0.00	0.00	0.00	0.33	0.00	0.00	0.00	0.00	0.00	0.00	0.00	0.00	0.00	0.00	0.00	0.00	0.03	0.00	>0.999999999999999	#DIV/0!
Q9H0C2	ADP/ATP translocase 4 OS=Homo sapiens GN=SLC25A31 PE=1 SV=1	0.00	0.00	0.00	0.00	0.00	0.00	0.00	0.00	0.00	0.00	0.00	1.63	0.00	0.00	0.00	0.00	0.00	0.00	0.00	0.00	0.00	0.00	0.00	0.00	0.14	0.00	>0.999999999999999	#DIV/0!
Q9UK41	Vacuolar protein sorting-associated protein 28 homolog OS=Homo sapiens GN=VPS28 PE=1 SV=1	0.00	0.00	0.00	0.00	0.00	0.00	0.00	0.00	0.00	0.00	0.00	1.06	0.00	0.00	0.00	0.00	0.00	0.00	0.00	0.00	0.00	0.00	0.00	0.00	0.09	0.00	>0.999999999999999	#DIV/0!
Q96HE7	ERO1-like protein alpha OS=Homo sapiens GN=ERO1L PE=1 SV=2	0.00	0.00	0.00	0.00	0.00	0.00	0.00	0.00	0.00	0.00	0.00	0.49	0.00	0.00	0.00	0.00	0.00	0.00	0.00	0.00	0.00	0.00	0.00	0.76	0.04	0.06	>0.999999999999999	0.6
P13726	Tissue factor OS=Homo sapiens GN=F3 PE=1 SV=1	0.00	0.00	0.00	0.00	0.00	0.00	0.00	0.00	0.00	0.00	0.00	0.82	0.00	0.00	0.00	0.00	0.00	0.00	0.00	0.00	0.00	0.00	0.00	0.84	0.07	0.07	>0.999999999999999	1.0
Q9H3G5	Probable serine carboxypeptidase CPVL OS=Homo sapiens GN=CPVL PE=1 SV=2	0.00	0.00	0.00	0.00	0.00	0.00	0.00	0.00	0.00	0.00	0.00	0.49	0.00	0.00	0.00	0.00	0.00	0.00	0.00	0.00	0.00	0.00	0.00	0.24	0.04	0.02	>0.999999999999999	2.0
Q86UX7	Fermitin family homolog 3 OS=Homo sapiens GN=FERMT3 PE=1 SV=1	0.00	0.00	0.00	0.00	0.00	0.00	0.00	0.00	0.00	0.00	0.00	0.33	0.00	0.00	0.00	0.00	0.00	0.61	0.00	0.00	0.68	0.00	0.00	0.00	0.03	0.11	>0.999999999999999	0.3
Q11128	Alpha-(1,3)-fucosyltransferase OS=Homo sapiens GN=FUT5 PE=2 SV=1	0.00	0.00	0.00	0.00	0.00	0.00	0.00	0.00	0.00	0.00	0.00	1.31	0.00	0.00	0.00	0.00	0.00	0.00	0.00	0.00	1.35	0.00	0.00	0.32	0.11	0.14	>0.999999999999999	0.8
P00387	NADH-cytochrome b5 reductase 3 OS=Homo sapiens GN=CYB5R3 PE=1 SV=3	0.00	0.00	0.00	0.00	0.00	0.00	0.00	0.00	0.00	0.00	0.00	0.82	0.00	0.00	0.00	0.00	0.00	0.00	0.00	0.00	0.00	0.00	0.00	0.00	0.07	0.00	>0.999999999999999	#DIV/0!
Q9NZ08	Endoplasmic reticulum aminopeptidase 1 OS=Homo sapiens GN=ERAP1 PE=1 SV=3	0.00	0.00	0.00	0.00	0.00	0.00	0.00	0.00	0.00	0.00	0.00	0.49	0.00	0.00	0.00	0.50	0.38	0.00	0.00	0.00	0.00	0.00	0.00	0.00	0.04	0.07	>0.999999999999999	0.6
O95395	Beta-1,3-galactosyl-O-glycosyl-glycoprotein beta-1,6-N-acetylglucosaminyltransferase 3 OS=Homo sapiens GN=G(	0.00	0.00	0.00	0.00	0.00	0.00	0.00	0.00	0.00	0.00	0.00	0.49	0.00	0.00	0.00	0.00	0.00	0.92	0.00	0.00	0.00	0.48	0.00	0.24	0.04	0.14	>0.999999999999999	0.3
Q8NFV4	Abhydrolase domain-containing protein 11 OS=Homo sapiens GN=ABHD11 PE=1 SV=1	0.00	0.00	0.00	0.00	0.00	0.00	0.00	0.00	0.00	0.00	0.00	0.82	0.00	0.00	0.00	0.00	0.00	0.00	0.00	0.00	0.00	0.00	0.00	0.00	0.07	0.00	>0.999999999999999	#DIV/0!
P52630	Signal transducer and activator of transcription 2 OS=Homo sapiens GN=STAT2 PE=1 SV=1	0.00	0.00	0.00	0.00	0.00	0.00	0.00	0.00	0.00	0.00	0.00	0.24	0.00	0.00	0.00	0.00	0.00	0.00	0.00	0.00	0.00	0.00	0.00	0.00	0.02	0.00	>0.999999999999999	#DIV/0!
O14773	Tripeptidyl-peptidase 1 OS=Homo sapiens GN=TPP1 PE=1 SV=2	0.00	0.00	0.00	0.00	0.00	0.00	0.00	0.00	0.00	0.00	0.00	0.41	0.00	0.00	0.00	0.00	0.00	0.00	0.00	0.00	0.00	0.00	0.00	0.20	0.03	0.02	>0.999999999999999	2.0
Q13501	Sequestosome-1 OS=Homo sapiens GN=SQSTM1 PE=1 SV=1	0.00	0.00	0.00	0.00	0.00	0.00	0.00	0.00	0.00	0.00	0.00	0.57	0.00	0.00	0.00	0.00	0.00	0.00	0.00	0.00	0.00	0.00	0.00	0.00	0.05	0.00	>0.999999999999999	#DIV/0!
Q5K651	Sterile alpha motif domain-containing protein 9 OS=Homo sapiens GN=SAMD9 PE=1 SV=1	0.00	0.00	0.00	0.00	0.00	0.00	0.00	0.00	0.00	0.00	0.00	0.16	0.00	0.00	0.00	0.34	0.00	0.00	0.00	0.00	0.00	0.00	0.00	0.00	0.01	0.03	>0.999999999999999	0.5
P00973	2'-5'-oligoadenylate synthase 1 OS=Homo sapiens GN=OAS1 PE=1 SV=4	0.00	0.00	0.00	0.00	0.00	0.00	0.00	0.00	0.00	0.00	0.00	0.57	1.65	0.00	0.00	0.00	0.00	0.00	1.27	0.00	0.00	0.00	0.00	0.00	0.05	0.24	>0.999999999999999	0.2
P07108	Acyl-CoA-binding protein OS=Homo sapiens GN=DBI PE=1 SV=2	0.00	0.00	0.00	0.00	0.00	0.00	0.00	0.00	0.00	0.00	0.00	11.83	0.00	0.00	0.00	0.00	0.00	0.00	0.00	0.00	0.00	2.80	4.97	1.40	0.99	0.76	>0.999999999999999	1.3
Q9Y6B6	GTP-binding protein SAR1b OS=Homo sapiens GN=SAR1B PE=1 SV=1	0.00	0.00	0.00	0.00	0.00	0.00	0.00	0.00	0.00	0.00	0.00	1.22	0.00	0.00	0.00	0.00	0.00	0.00	0.00	0.00	0.00	0.00	0.00	0.60	0.10	0.05	>0.999999999999999	2.0
Q9UMY4	Sorting nexin-12 OS=Homo sapiens GN=SNX12 PE=1 SV=3	0.00	0.00	0.00	0.00	0.00	0.00	0.00	0.00	0.00	0.00	0.00	1.39	0.00	0.00	0.00	0.00	0.00	0.00	0.00	0.00	0.00	0.00	0.00	0.00	0.12	0.00	>0.999999999999999	#DIV/0!
P62253	Ubiquitin-conjugating enzyme E2 G1 OS=Homo sapiens GN=UBE2G1 PE=1 SV=3	0.00	0.00	0.00	0.00	0.00	0.00	0.00	0.00	0.00	0.00	0.00	1.39	0.00	0.00	0.00	0.00	0.00	0.00	0.00	0.00	0.00	0.00	0.00	0.00	0.12	0.00	>0.999999999999999	#DIV/0!
P04844	Dolichyl-diphosphooligosaccharide--protein glycosyltransferase subunit 2 OS=Homo sapiens GN=RPN2 PE=1 SV=3	0.00	0.00	0.00	0.00	0.00	0.00	0.00	0.00	0.00	0.00	0.00	0.41	0.00	0.00	0.00	0.00	0.00	0.00	0.00	0.00	0.00	0.00	0.00	0.20	0.03	0.02	>0.999999999999999	2.0
Q8TDB6	E3 ubiquitin-protein ligase DTX3L OS=Homo sapiens GN=DTX3L PE=1 SV=1	0.00	0.00	0.00	0.00	0.00	0.00	0.00	0.00	0.00	0.00	0.00	0.33	0.00	0.00	0.00	0.00	0.00	0.00	0.00	0.00	0.00	0.00	0.00	0.00	0.03	0.00	>0.999999999999999	#DIV/0!
Q9NYU2	UDP-glucose:glycoprotein glucosyltransferase 1 OS=Homo sapiens GN=UGGT1 PE=1 SV=3	0.00	0.00	0.00	0.00	0.00	0.00	0.00	0.00	0.00	0.00	0.00	0.16	0.00	0.00	0.00	0.00	0.00	0.00	0.00	0.00	0.00	0.00	0.00	0.08	0.01	0.01	>0.999999999999999	2.0
P52789	Hexokinase-2 OS=Homo sapiens GN=HK2 PE=1 SV=2	0.00	0.00	0.00	0.00	0.00	0.00	0.00	0.00	0.00	0.00	0.00	0.24	0.00	0.00	0.00	0.00	0.00	0.00	0.00	0.00	0.00	0.00	0.00	0.12	0.02	0.01	>0.999999999999999	2.0

Q8TE68	Epidermal growth factor receptor kinase substrate 8-like protein 1 OS=Homo sapiens GN=EPS8L1 PE=1 SV=3	0.00	0.00	0.00	0.00	0.00	0.00	0.00	0.00	0.00	0.00	0.00	0.33	0.00	0.00	0.00	0.00	0.00	0.00	0.00	0.00	0.00	0.32	0.00	0.00	0.03	0.03	>0.999999999999999	1.0
Q9H788	SH2 domain-containing protein 4A OS=Homo sapiens GN=SH2D4A PE=1 SV=1	0.00	0.00	0.00	0.00	0.00	0.00	0.00	0.00	0.00	0.00	0.00	0.49	0.00	0.00	0.00	0.00	0.00	0.00	0.00	0.00	0.00	0.00	0.00	0.24	0.04	0.02	>0.999999999999999	2.0
P17050	Alpha-N-acetylgalactosaminidase OS=Homo sapiens GN=NAGA PE=1 SV=2	0.00	0.00	0.00	0.00	0.00	0.00	0.00	0.00	0.00	0.00	0.00	0.57	0.00	0.00	0.00	0.00	0.00	0.00	0.00	0.00	0.00	0.00	0.00	0.00	0.05	0.00	>0.999999999999999	#DIV/0!
P55809	Succinyl-CoA:3-ketoacid-coenzyme A transferase 1, mitochondrial OS=Homo sapiens GN=OXCT1 PE=1 SV=1	0.00	0.00	0.00	0.00	0.00	0.00	0.00	0.00	0.00	0.00	0.00	0.49	0.00	0.00	0.00	0.00	0.00	0.00	0.00	0.00	0.00	0.00	0.00	0.00	0.04	0.00	>0.999999999999999	#DIV/0!
Q9P2R7	Succinyl-CoA ligase [ADP-forming] subunit beta, mitochondrial OS=Homo sapiens GN=SUCLA2 PE=1 SV=3	0.00	0.00	0.00	0.00	0.00	0.00	0.00	0.00	0.00	0.00	0.00	0.57	0.00	0.00	0.00	0.00	0.00	0.00	0.00	0.00	0.00	0.00	0.00	0.00	0.05	0.00	>0.999999999999999	#DIV/0!
Q9BS26	Endoplasmic reticulum resident protein 44 OS=Homo sapiens GN=ERP44 PE=1 SV=1	0.00	0.00	0.00	0.00	0.00	0.00	0.00	0.00	0.00	0.00	0.00	0.57	0.00	0.00	0.00	0.00	0.00	0.00	0.00	0.00	0.00	0.00	0.00	0.00	0.05	0.00	>0.999999999999999	#DIV/0!
Q14194	Dihydropyrimidinase-related protein 1 OS=Homo sapiens GN=CRMP1 PE=1 SV=1	0.00	0.00	0.00	0.00	0.00	0.00	0.00	0.00	0.00	0.00	0.00	0.41	0.00	0.00	0.00	0.00	0.00	0.00	0.00	0.00	0.00	0.00	0.00	0.00	0.03	0.00	>0.999999999999999	#DIV/0!
Q7Z3D6	UPF0317 protein C14orf159, mitochondrial OS=Homo sapiens GN=C14orf159 PE=2 SV=2	0.00	0.00	0.00	0.00	0.00	0.00	0.00	0.00	0.00	0.00	0.00	0.41	0.00	0.00	0.00	0.00	0.00	0.00	0.00	0.00	0.00	0.00	0.00	0.00	0.03	0.00	>0.999999999999999	#DIV/0!
Q15582	Transforming growth factor-beta-induced protein ig-h3 OS=Homo sapiens GN=TGFBI PE=1 SV=1	0.00	0.00	0.00	0.00	0.00	0.00	0.00	0.00	0.00	0.00	0.00	0.33	0.00	0.00	0.00	0.00	0.00	0.00	0.00	0.00	0.00	0.00	0.00	0.00	0.03	0.00	>0.999999999999999	#DIV/0!
P01605	Ig kappa chain V-I region Lay OS=Homo sapiens PE=1 SV=1	0.00	0.00	0.00	0.00	0.00	0.00	0.00	0.00	0.00	0.00	0.00	2.37	0.00	0.00	0.00	4.88	0.00	0.00	0.00	0.00	0.00	2.32	0.00	0.00	0.20	0.60	>0.999999999999999	0.3
Q15126	Phosphomevalonate kinase OS=Homo sapiens GN=PMVK PE=1 SV=3	0.00	0.00	0.00	0.00	0.00	0.00	0.00	0.00	0.00	0.00	0.00	1.22	0.00	0.00	0.00	0.00	0.00	0.00	0.00	0.00	0.00	0.00	0.00	0.60	0.10	0.05	>0.999999999999999	2.0
P26885	Peptidyl-prolyl cis-trans isomerase FKBP2 OS=Homo sapiens GN=FKBP2 PE=1 SV=2	0.00	0.00	0.00	0.00	0.00	0.00	0.00	0.00	0.00	0.00	0.00	1.79	0.00	0.00	0.00	0.00	0.00	0.00	0.00	0.00	0.00	0.00	0.00	0.88	0.15	0.07	>0.999999999999999	2.0
Q04837	Single-stranded DNA-binding protein, mitochondrial OS=Homo sapiens GN=SSBP1 PE=1 SV=1	0.00	0.00	0.00	0.00	0.00	0.00	0.00	0.00	0.00	0.00	0.00	1.63	0.00	0.00	0.00	0.00	0.00	0.00	0.00	0.00	0.00	0.00	0.00	0.00	0.14	0.00	>0.999999999999999	#DIV/0!
P06865	Beta-hexosaminidase subunit alpha OS=Homo sapiens GN=HEXA PE=1 SV=2	0.00	0.00	0.00	0.00	0.00	0.00	0.00	0.00	0.00	0.00	0.00	0.41	0.00	0.00	0.00	1.01	0.00	0.00	0.00	0.00	0.00	0.00	0.00	0.20	0.03	0.10	>0.999999999999999	0.3
P38117	Electron transfer flavoprotein subunit beta OS=Homo sapiens GN=ETFB PE=1 SV=3	0.00	0.00	0.00	0.00	0.00	0.00	0.00	0.00	0.00	0.00	0.00	2.04	0.00	0.00	0.00	0.00	0.00	0.00	0.00	0.00	0.00	0.00	0.00	0.00	0.17	0.00	>0.999999999999999	#DIV/0!
P13284	Gamma-interferon-inducible lysosomal thiol reductase OS=Homo sapiens GN=IFI30 PE=1 SV=3	0.00	0.00	0.00	0.00	0.00	0.00	0.00	0.00	0.00	0.00	0.00	0.98	2.82	0.00	0.00	0.00	1.52	0.00	0.00	0.00	0.00	0.00	0.00	0.00	0.08	0.36	>0.999999999999999	0.2
Q9Y646	Plasma glutamate carboxypeptidase OS=Homo sapiens GN=PGCP PE=1 SV=1	0.00	0.00	0.00	0.00	0.00	0.00	0.00	0.00	0.00	0.00	0.00	0.49	0.00	0.00	0.00	0.00	0.00	0.00	0.00	0.00	0.00	1.60	0.00	0.00	0.04	0.13	>0.999999999999999	0.3
Q14210	Lymphocyte antigen 6D OS=Homo sapiens GN=LY6D PE=1 SV=1	0.00	0.00	0.00	0.00	0.00	0.00	0.00	0.00	0.00	0.00	0.00	2.04	0.00	4.01	0.00	0.00	0.00	0.00	0.00	0.00	0.00	2.00	3.55	0.00	0.17	0.80	>0.999999999999999	0.2
P30042	ES1 protein homolog, mitochondrial OS=Homo sapiens GN=C21orf33 PE=1 SV=3	0.00	0.00	0.00	0.00	0.00	0.00	0.00	0.00	0.00	0.00	0.00	0.98	0.00	0.00	0.00	0.00	0.00	0.00	0.00	0.00	0.00	0.00	0.00	0.00	0.08	0.00	>0.999999999999999	#DIV/0!
P02778	C-X-C motif chemokine 10 OS=Homo sapiens GN=CXCL10 PE=1 SV=2	0.00	0.00	0.00	0.00	0.00	0.00	0.00	0.00	0.00	0.00	0.00	2.53	0.00	0.00	0.00	0.00	0.00	0.00	0.00	0.00	0.00	0.00	0.00	0.00	0.21	0.00	>0.999999999999999	#DIV/0!
Q15293	Reticulocalbin-1 OS=Homo sapiens GN=RCN1 PE=1 SV=1	0.00	0.00	0.00	0.00	0.00	0.00	0.00	0.00	0.00	0.00	0.00	0.73	0.00	0.00	2.15	0.00	0.00	0.00	0.00	0.00	1.52	0.72	0.00	0.72	0.06	0.43	>0.999999999999999	0.1
Q6YP21	Kynurenine--oxoglutarate transaminase 3 OS=Homo sapiens GN=CCBL2 PE=1 SV=1	0.00	0.00	0.00	0.00	0.00	0.00	0.00	0.00	0.00	0.00	0.00	0.49	0.00	0.00	0.00	0.00	0.00	0.00	0.00	0.00	0.00	0.00	0.00	0.00	0.04	0.00	>0.999999999999999	#DIV/0!
P08254	Stromelysin-1 OS=Homo sapiens GN=MMP3 PE=1 SV=2	0.00	0.00	0.00	0.00	0.00	0.00	0.00	0.00	0.00	0.00	0.00	0.49	0.00	0.00	0.00	0.00	0.00	0.00	0.00	0.00	0.00	0.00	0.00	0.00	0.04	0.00	>0.999999999999999	#DIV/0!
P31937	3-hydroxyisobutyrate dehydrogenase, mitochondrial OS=Homo sapiens GN=HIBADH PE=1 SV=2	0.00	0.00	0.00	0.00	0.00	0.00	0.00	0.00	0.00	0.00	0.00	0.73	0.00	0.00	0.00	0.00	0.00	0.00	0.00	0.00	0.00	0.00	0.00	0.00	0.06	0.00	>0.999999999999999	#DIV/0!
Q9NPJ3	Acyl-coenzyme A thioesterase 13 OS=Homo sapiens GN=ACOT13 PE=1 SV=1	0.00	0.00	0.00	0.00	0.00	0.00	0.00	0.00	0.00	0.00	0.00	1.88	0.00	0.00	0.00	0.00	0.00	0.00	0.00	0.00	0.00	0.00	0.00	0.00	0.16	0.00	>0.999999999999999	#DIV/0!
P07476	Involucrin OS=Homo sapiens GN=IVL PE=1 SV=2	0.00	0.00	0.00	0.00	0.00	0.00	0.00	0.00	0.00	0.00	0.00	0.41	0.00	0.00	0.00	0.84	0.00	0.77	0.90	0.00	0.00	0.40	0.00	1.80	0.03	0.39	>0.999999999999999	0.1
O60313	Dynamin-like 120 kDa protein, mitochondrial OS=Homo sapiens GN=OPA1 PE=1 SV=3	0.00	0.00	0.00	0.00	0.00	0.00	0.00	0.00	0.00	0.00	0.00	0.24	0.00	0.00	0.00	0.00	0.00	0.00	0.00	0.00	0.00	0.00	0.00	0.00	0.02	0.00	>0.999999999999999	#DIV/0!
Q96KG9	N-terminal kinase-like protein OS=Homo sapiens GN=SCYL1 PE=1 SV=1	0.00	0.00	0.00	0.00	0.00	0.00	0.00	0.00	0.00	0.00	0.00	0.33	0.00	0.00	0.00	0.00	0.00	0.00	0.00	0.00	0.00	0.00	0.00	0.00	0.03	0.00	>0.999999999999999	#DIV/0!
P15941	Mucin-1 OS=Homo sapiens GN=MUC1 PE=1 SV=3	0.00	0.00	0.00	0.00	0.00	0.00	0.00	0.00	0.00	0.00	0.00	0.24	0.00	0.00	0.00	0.00	0.00	0.00	0.00	0.00	0.00	0.00	0.00	0.12	0.02	0.01	>0.999999999999999	2.0
P15328	Folate receptor alpha OS=Homo sapiens GN=FOLR1 PE=1 SV=3	0.00	0.00	0.00	0.00	0.00	0.00	0.00	0.00	0.00	0.00	0.00	0.90	0.00	0.00	0.00	0.00	1.39	0.00	0.00	0.00	0.00	0.00	0.00	0.44	0.07	0.15	>0.999999999999999	0.5
Q9Y287	Integral membrane protein 2B OS=Homo sapiens GN=ITM2B PE=1 SV=1	0.00	0.00	0.00	0.00	0.00	0.00	0.00	0.00	0.00	0.00	0.00	0.90	0.00	0.00	0.00	0.00	0.00	0.00	0.00	0.00	0.00	0.00	0.00	0.00	0.07	0.00	>0.999999999999999	#DIV/0!
Q92820	Gamma-glutamyl hydrolase OS=Homo sapiens GN=GGH PE=1 SV=2	0.00	0.00	0.00	0.00	0.00	0.00	0.00	0.00	0.00	0.00	0.00	0.73	0.00	0.00	0.00	0.00	0.00	0.00	0.00	0.00	0.00	0.72	0.00	0.00	0.06	0.06	>0.999999999999999	1.0
Q6ZSR9	Uncharacterized protein FLJ45252 OS=Homo sapiens PE=1 SV=2	0.00	0.00	0.00	0.00	0.00	0.00	0.00	0.00	0.00	0.00	0.00	0.73	0.00	0.00	0.00	0.00	0.00	0.00	0.00	0.00	0.00	0.00	0.00	0.00	0.06	0.00	>0.999999999999999	#DIV/0!
P60033	CD81 antigen OS=Homo sapiens GN=CD81 PE=1 SV=1	0.00	0.00	0.00	0.00	0.00	0.00	0.00	0.00	0.00	0.00	0.00	1.06	0.00	0.00	0.00	0.00	0.00	0.00	0.00	0.00	0.00	0.00	0.00	0.00	0.09	0.00	>0.999999999999999	#DIV/0!
Q5JWF2	Guanine nucleotide-binding protein G(s) subunit alpha isoforms XLas OS=Homo sapiens GN=GNAS PE=1 SV=2	0.00	0.00	0.00	0.00	0.00	0.00	0.00	0.00	0.00	0.00	0.00	0.24	0.00	0.00	0.00	0.00	0.00	0.00	0.00	0.00	0.00	0.00	0.00	0.00	0.02	0.00	>0.999999999999999	#DIV/0!
Q9Y2B0	Protein canopy homolog 2 OS=Homo sapiens GN=CNPY2 PE=1 SV=1	0.00	0.00	0.00	0.00	0.00	0.00	0.00	0.00	0.00	0.00	0.00	1.31	0.00	0.00	0.00	0.00	0.00	0.00	0.00	0.00	0.00	0.00	0.00	0.00	0.11	0.00	>0.999999999999999	#DIV/0!
P23786	Carnitine O-palmitoyltransferase 2, mitochondrial OS=Homo sapiens GN=CPT2 PE=1 SV=2	0.00	0.00	0.00	0.00	0.00	0.00	0.00	0.00	0.00	0.00	0.00	0.33	0.00	0.00	0.00	0.00	0.00	0.00	0.00	0.00	0.00	0.00	0.00	0.00	0.03	0.00	>0.999999999999999	#DIV/0!
Q9BWS9	Chitinase domain-containing protein 1 OS=Homo sapiens GN=CHID1 PE=1 SV=1	0.00	0.00	0.00	0.00	0.00	0.00	0.00	0.00	0.00	0.00	0.00	0.57	0.00	0.00	0.00	0.00	0.00	0.00	0.00	0.00	0.00	0.00	0.00	0.00	0.05	0.00	>0.999999999999999	#DIV/0!
Q04864	Proto-oncogene c-Rel OS=Homo sapiens GN=REL PE=1 SV=1	0.00	0.00	0.00	0.00	0.00	0.00	0.00	0.00	0.00	0.00	0.00	0.41	0.00	0.00	0.00	0.00	0.00	0.00	0.00	0.00	0.00	0.00	0.00	0.00	0.03	0.00	>0.999999999999999	#DIV/0!
Q96AZ6	Interferon-stimulated gene 20 kDa protein OS=Homo sapiens GN=ISG20 PE=1 SV=2	0.00	0.00	0.00	0.00	0.00	0.00	0.00	0.00	0.00	0.00	0.00	1.31	0.00	0.00	0.00	0.00	0.00	0.00	0.00	0.00	0.00	0.00	0.00	0.00	0.11	0.00	>0.999999999999999	#DIV/0!
Q6P2I3	Fumarylacetoacetate hydrolase domain-containing protein 2B OS=Homo sapiens GN=FAHD2B PE=1 SV=1	0.00	0.00	0.00	0.00	0.00	0.00	0.00	0.00	0.00	0.00	0.00	0.73	0.00	0.00	0.00	0.00	0.00	0.00	0.00	0.00	0.00	0.00	0.00	0.00	0.06	0.00	>0.999999999999999	#DIV/0!
Q9UHY1	Nuclear receptor-binding protein OS=Homo sapiens GN=NRBP1 PE=1 SV=1	0.00	0.00	0.00	0.00	0.00	0.00	0.00	0.00	0.00	0.00	0.00	0.41	0.00	0.00	0.00	0.00	0.00	0.00	0.00	0.00	0.00	0.00	0.71	0.00	0.03	0.06	>0.999999999999999	0.6
P49748	Very long-chain specific acyl-CoA dehydrogenase, mitochondrial OS=Homo sapiens GN=ACADVL PE=1 SV=1	0.00	0.00	0.00	0.00	0.00	0.00	0.00	0.00	0.00	0.00	0.00	0.41	0.00	0.00	0.00	0.00	0.00	0.00	0.00	0.00	0.00	0.00	0.00	0.00	0.03	0.00	>0.999999999999999	#DIV/0!
Q14257	Reticulocalbin-2 OS=Homo sapiens GN=RCN2 PE=1 SV=1	0.00	0.00	0.00	0.00	0.00	0.00	0.00	0.00	0.00	0.00	0.00	0.73	0.00	0.00	0.00	0.00	0.00	0.00	0.00	0.00	0.00	0.00	0.00	0.00	0.06	0.00	>0.999999999999999	#DIV/0!
P39748	Flap endonuclease 1 OS=Homo sapiens GN=FEN1 PE=1 SV=1	0.00	0.00	0.00	0.00	0.00	0.00	0.00	0.00	0.00	0.00	0.00	0.65	0.00	0.00	0.00	0.00	0.00	0.00	0.00	0.00	0.00	0.00	0.00	0.00	0.05	0.00	>0.999999999999999	#DIV/0!
P16435	NADPH--cytochrome P450 reductase OS=Homo sapiens GN=POR PE=1 SV=2	0.00	0.00	0.00	0.00	0.00	0.00	0.00	0.00	0.00	0.00	0.00	0.33	0.00	0.00	0.00	0.00	0.00	0.00	0.00	0.00	0.00	0.00	0.00	0.00	0.03	0.00	>0.999999999999999	#DIV/0!
A8MQ14	Zinc finger protein 850 OS=Homo sapiens GN=ZNF850 PE=3 SV=2	0.00	0.00	0.00	0.00	0.00	0.00	0.00	0.00	0.00	0.00	0.00	0.24	0.00	0.00	0.00	0.00	0.00	0.00	0.00	0.00	0.00	0.00	0.00	0.00	0.02	0.00	>0.999999999999999	#DIV/0!
P62877	E3 ubiquitin-protein ligase RBX1 OS=Homo sapiens GN=RBX1 PE=1 SV=1	0.00	0.00	0.00	0.00	0.00	0.00	0.00	0.00	0.00	0.00	0.00	2.20	0.00	0.00	0.00	0.00	0.00	0.00	0.00	0.00	0.00	0.00	0.00	0.00	0.18	0.00	>0.999999999999999	#DIV/0!
P49792	E3 SUMO-protein ligase RanBP2 OS=Homo sapiens GN=RANBP2 PE=1 SV=2	0.00	0.00	0.00	0.00	0.00	0.00	0.00	0.00	0.00	0.00	0.00	0.08	0.00	0.00	0.00	0.00	0.00	0.00	0.00	0.00	0.00	0.00	0.00	0.00	0.01	0.00	>0.999999999999999	#DIV/0!
P40939	Trifunctional enzyme subunit alpha, mitochondrial OS=Homo sapiens GN=HADHA PE=1 SV=2	0.00	0.00	0.00	0.00	0.00	0.00	0.00	0.00	0.00	0.00	0.00	0.33	0.00	0.00	0.00	0.00	0.00	0.00	0.00	0.00	0.00	0.00	0.00	0.00	0.03	0.00	>0.999999999999999	#DIV/0!
Q92896	Golgi apparatus protein 1 OS=Homo sapiens GN=GLG1 PE=1 SV=2	0.00	0.00	0.00	0.00	0.00	0.00	0.00	0.00	0.00	0.00	0.00	0.16	0.00	0.00	0.00	0.00	0.00	0.00	0.00	0.00	0.00	0.16	0.00	0.00	0.01	0.01	>0.999999999999999	1.0
P53779	Mitogen-activated protein kinase 10 OS=Homo sapiens GN=MAPK10 PE=1 SV=2	0.00	0.00	0.00	0.00	0.00	0.00	0.00	0.00	0.00	0.00	0.00	0.49	0.00	0.00	0.00	0.00	0.00	0.00	0.00	0.00	0.00	1.04	0.00	0.00	0.04	0.09	>0.999999999999999	0.5
O95571	Protein ETHE1, mitochondrial OS=Homo sapiens GN=ETHE1 PE=1 SV=2	0.00	0.00	0.00	0.00	0.00	0.00	0.00	0.00	0.00	0.00	0.00	0.98	0.00	0.00	0.00	0.00	0.00	0.00	0.00	0.00	0.00	0.00	0.00	0.00	0.08	0.00	>0.999999999999999	#DIV/0!
P09110	3-ketoacyl-CoA thiolase, peroxisomal OS=Homo sapiens GN=ACAA1 PE=1 SV=2	0.00	0.00	0.00	0.00	0.00	0.00	0.00	0.00	0.00	0.00	0.00	0.57	0.00	0.00	0.00	0.00	0.00	0.00	0.00	0.00	0.00	0.00	0.00	0.00	0.05	0.00	>0.999999999999999	#DIV/0!
Q8TD47	40S ribosomal protein S4, Y isoform 2 OS=Homo sapiens GN=RPS4Y2 PE=1 SV=3	0.00	0.00	0.00	0.00	0.00	0.00	0.00	0.00	0.00	0.00	0.00	0.90	0.00	0.00	0.00	0.00	0.00	0.00	0.00	0.00	0.00	0.00	7.52	0.00	0.07	0.63	>0.999999999999999	0.1
Q5VYY2	Lipase member M OS=Homo sapiens GN=LIPM PE=2 SV=2	0.00	0.00	0.00	0.00	0.00	0.00	0.00	0.00	0.00	0.00	0.00	0.57	0.00	0.00	0.00	0.00	0.00	0.00	0.00	0.00	0.00	0.00	0.00	0.00	0.05	0.00	>0.999999999999999	#DIV/0!
Q9Y5Y2	Cytosolic Fe-S cluster assembly factor NUBP2 OS=Homo sapiens GN=NUBP2 PE=1 SV=1	0.00	0.00	0.00	0.00	0.00	0.00	0.00	0.00	0.00	0.00	0.00	0.90	0.00	0.00	0.00	0.00	0.00	0.00	0.00	0.00	0.00	0.00	0.00	0.00	0.07	0.00	>0.999999999999999	#DIV/0!
Q9NRR5	Ubiquilin-4 OS=Homo sapiens GN=UBQLN4 PE=1 SV=2	0.00	0.00	0.00	0.00	0.00	0.00	0.00	0.00	0.00	0.00	0.00	0.41	0.00	0.00	0.00	0.00	0.00	0.00	0.00	0.00	0.00	0.00	0.00	0.00	0.03	0.00	>0.999999999999999	#DIV/0!
Q05823	2-5A-dependent ribonuclease OS=Homo sapiens GN=RNASEL PE=1 SV=2	0.00	0.00	0.00	0.00	0.00	0.00	0.00	0.00	0.00	0.00	0.00	0.33	0.00	0.00	0.00	0.00	0.00	0.00	0.00	0.00	0.00	0.00	0.00	0.00	0.03	0.00	>0.999999999999999	#DIV/0!
P98179	Putative RNA-binding protein 3 OS=Homo sapiens GN=RBM3 PE=1 SV=1	0.00	0.00	0.00	0.00	0.00	0.00	0.00	0.00	0.00	0.00	0.00	1.63	0.00	0.00	0.00	0.00	0.00	0.00	0.00	0.00	0.00	0.00	0.00	0.00	0.14	0.00	>0.999999999999999	#DIV/0!
Q8WWK9	Cytoskeleton-associated protein 2 OS=Homo sapiens GN=CKAP2 PE=1 SV=1	0.00	0.00	0.00	0.00	0.00	0.00	0.00	0.00	0.00	0.00	0.00	0.33	0.00	0.00	0.00	0.00	0.00	0.00	0.00	0.00	0.00	0.00	0.00	0.00	0.03	0.00	>0.999999999999999	#DIV/0!
P55084	Trifunctional enzyme subunit beta, mitochondrial OS=Homo sapiens GN=HADHB PE=1 SV=3	0.00	0.00	0.00	0.00	0.00	0.00	0.00	0.00	0.00	0.00	0.00	0.49	0.00	0.00	0.00	0.00	0.00	0.00	0.00	0.00	0.00	0.00	0.00	0.00	0.04	0.00	>0.999999999999999	#DIV/0!
P28325	Cystatin-D OS=Homo sapiens GN=CST5 PE=1 SV=1	0.00	0.00	0.00	0.00	0.00	0.00	0.00	0.00	0.00	0.00	0.00	1.71	0.00	0.00	0.00	0.00	2.66	0.00	0.00	0.00	0.00	0.00	6.53	0.00	0.14	0.77	>0.999999999999999	0.2
Q9NZM1	Myoferlin OS=Homo sapiens GN=MYOF PE=1 SV=1	0.00	0.00	0.00	0.00	0.00	0.00	0.00	0.00	0.00	0.00	0.00	0.08	0.00	0.00	0.00	0.00	0.00	0.00	0.00	0.00	0.00	0.00	0.00	0.00	0.01	0.00	>0.999999999999999	#DIV/0!
Q9UK10	Zinc finger protein 225 OS=Homo sapiens GN=ZNF225 PE=1 SV=2	0.00	0.00	0.00	0.00	0.00	0.00	0.00	0.00	0.00	0.00	0.00	0.33	0.00	0.00	0.00	0.00	0.00	0.00	0.00	0.00	0.00	0.00	0.00	0.00	0.03	0.00	>0.999999999999999	#DIV/0!
Q96LJ7	Dehydrogenase/reductase SDR family member 1 OS=Homo sapiens GN=DHRS1 PE=1 SV=1	0.00	0.00	0.00	0.00	0.00	0.00	0.00	0.00	0.00	0.00	0.00	0.82	0.00	0.00	0.00	0.00	0.00	0.00	0.00	0.00	0.00	0.00	0.00	0.00	0.07	0.00	>0.999999999999999	#DIV/0!
O00273	DNA fragmentation factor subunit alpha OS=Homo sapiens GN=DFFA PE=1 SV=1	0.00	0.00	0.00	0.00	0.00	0.00	0.00	0.00	0.00	0.00	0.00	0.73	0.00	0.00	0.00	0.00	0.00	0.00	0.00	0.00	0.00	0.72	0.00	0.00	0.06	0.06	>0.999999999999999	1.0
P09228	Cystatin-SA OS=Homo sapiens GN=CST2 PE=1 SV=1	0.00	0.00	0.00	0.00	0.00	0.00	0.00	0.00	0.00	0.00	0.00	0.00	25.64	0.00	0.00	0.00	0.00	0.00	0.00	0.00	0.00	0.00	0.00	0.00	0.00	2.14	>0.999999999999999	0.0
P62854	40S ribosomal protein S26 OS=Homo sapiens GN=RPS26 PE=1 SV=3	0.00	0.00	0.00	0.00	0.00	0.00	0.00	0.00	0.00	0.00	0.00	0.00	6.12	0.00	0.00	0.00	0.00	0.00	0.00	0.00	0.00	0.00	0.00	0.00	0.00	0.51	>0.999999999999999	0.0
Q6NVV1	Putative 60S ribosomal protein L13a-like MGC87657 OS=Homo sapiens PE=5 SV=1	0.00	0.00	0.00	0.00	0.00	0.00	0.00	0.00	0.00	0.00	0.00	0.00	6.59	4.50	0.00	4.71	0.00	0.00	0.00	0.00	0.00	0.00	0.00	0.00	0.00	1.32	>0.999999999999999	0.0
O95340	Bifunctional 3'-phosphoadenosine 5'-phosphosulfate synthase 2 OS=Homo sapiens GN=PAPSS2 PE=1 SV=2	0.00	0.00	0.00	0.00	0.00	0.00	0.00	0.00	0.00	0.00	0.00	0.00	2.35	0.00	0.00	0.00	0.00	0.00	0.00	0.00	0.00	0.00	0.71	0.00	0.00	0.26	>0.999999999999999	0.0
P81605	Dermcidin OS=Homo sapiens GN=DCD PE=1 SV=2	0.00	0.00	0.00	0.00	0.00	0.00	0.00	0.00	0.00	0.00	0.00	0.00	7.06	0.00	0.00	0.00	0.00	0.00	0.00	3.89	11.65	0.00	0.00	0.00	0.00	1.88	>0.999999999999999	0.0
O60762	Dolichol-phosphate mannosyltransferase OS=Homo sapiens GN=DPM1 PE=1 SV=1	0.00	0.00	0.00	0.00	0.00	0.00	0.00	0.00	0.00	0.00	0.00	0.00	2.82	0.00	0.00	0.00	0.00	0.00	0.00	1.43	0.00	0.00	0.00	0.00	0.00	0.35	>0.999999999999999	0.0
Q5FWF4	Zinc finger Ran-binding domain-containing protein 3 OS=Homo sapiens GN=ZRANB3 PE=2 SV=2	0.00	0.00	0.00	0.00	0.00	0.00	0.00	0.00	0.00	0.00	0.00	0.00	0.71	0.00	0.72	0.00	0.00	0.00	0.00	0.00	0.00	0.00	0.00	0.00	0.00	0.12	>0.999999999999999	0.0
O95445	Apolipoprotein M OS=Homo sapiens GN=APOM PE=1 SV=2	0.00	0.00	0.00	0.00	0.00	0.00	0.00	0.00	0.00	0.00	0.00	0.00	3.76	0.00	0.00	2.69	2.02	0.00	0.00	7.01	0.00	1.28	0.00	0.00	0.00	1.40	>0.999999999999999	0.0
Q9H2M9	Rab3 GTPase-activating protein non-catalytic subunit OS=Homo sapiens GN=RAB3GAP2 PE=1 SV=1	0.00	0.00	0.00	0.00	0.00	0.00	0.00	0.00	0.00	0.00	0.00	0.00	0.47	0.00	0.00	0.00	0.00	0.00	0.00	0.00	0.00	0.00	0.00	0.00	0.00	0.04	>0.999999999999999	0.0
Q8IYD1	Eukaryotic peptide chain release factor GTP-binding subunit ERF3B OS=Homo sapiens GN=GSPT2 PE=1 SV=2	0.00	0.00	0.00	0.00	0.00	0.00	0.00	0.00	0.00	0.00	0.00	0.00	1.18	0.00	0.00	0.00	0.63	0.00	0.00	0.00	0.00	0.00	0.00	0.00	0.00	0.15	>0.999999999999999	0.0
Q00005	Serine/threonine-protein phosphatase 2A 55 kDa regulatory subunit B beta isoform OS=Homo sapiens GN=PPP2R2	0.00	0.00	0.00	0.00	0.00	0.00	0.00	0.00	0.00	0.00	0.00	0.00	0.00	0.96	0.00	0.00	0.76	0.00	0.00	0.00	0.00	0.00	0.00	0.00	0.00	0.14	>0.999999999999999	0.0
Q5D862	Filaggrin-2 OS=Homo sapiens GN=FLG2 PE=1 SV=1	0.00	0.00	0.00	0.00	0.00	0.00	0.00	0.00	0.00	0.00	0.00	0.00	0.00	0.16	0.00	0.00	0.00	0.00	0.00	0.00	0.00	0.00	0.14	0.00	0.00	0.03	>0.999999999999999	0.0
Q4VXU2	Polyadenylate-binding protein 1-like OS=Homo sapiens GN=PABPC1L PE=2 SV=1	0.00	0.00	0.00	0.00	0.00	0.00	0.00	0.00	0.00	0.00	0.00	0.00	0.00	0.80	0.00	0.00	0.63	0.00	0.00	0.00	0.00	0.00	0.00	0.00	0.00	0.12	>0.999999999999999	0.0
P01703	Ig lambda chain V-I region NEWM OS=Homo sapiens PE=1 SV=1	0.00	0.00	0.00	0.00	0.00	0.00	0.00	0.00	0.00	0.00	0.00	0.00	0.00	4.98	0.00	5.22	0.00	0.00	0.00	0.00	5.23	2.48	4.40	0.00	0.00	1.86	>0.999999999999999	0.0
P10163	Basic salivary proline-rich protein 4 OS=Homo sapiens GN=PRB4 PE=1 SV=3	0.00	0.00	0.00	0.00	0.00	0.00	0.00	0.00	0.00	0.00	0.00	0.00	0.00	1.77	0.00	0.00	0.00	0.00	0.00	0.00	0.00	0.00	0.00	0.00	0.00	0.15	>0.999999999999999	0.0
Q9UKR3	Kallikrein-13 OS=Homo sapiens GN=KLK13 PE=1 SV=1	0.00	0.00	0.00	0.00	0.00	0.00	0.00	0.00	0.00	0.00	0.00	0.00	0.00	1.77	0.00	0.00	0.00	0.00	0.00	0.00	0.00	0.00	0.00	0.00	0.00	0.15	>0.999999999999999	0.0
P22570	NADPH:adrenodoxin oxidoreductase, mitochondrial OS=Homo sapiens GN=FDXR PE=1 SV=3	0.00	0.00	0.00	0.00	0.00	0.00	0.00	0.00	0.00	0.00	0.00	0.00	0.00	0.96	0.00	0.00	0.00	0.00	0.00	0.00	0.00	0.00	0.00	0.00	0.00	0.08	>0.999999999999999	0.0
P32926	Desmoglein-3 OS=Homo sapiens GN=DSG3 PE=1 SV=2	0.00	0.00	0.00	0.00	0.00	0.00	0.00	0.00	0.00	0.00	0.00	0.00	0.00	0.48	0.00	0.00	0.00	0.00	0.46	0.00	0.00	0.00	0.00	0.00	0.00	0.08	>0.999999999999999	0.0
P18065	Insulin-like growth factor-binding protein 2 OS=Homo sapiens GN=IGFBP2 PE=1 SV=2	0.00	0.00	0.00	0.00	0.00	0.00	0.00	0.00	0.00	0.00	0.00	0.00	0.00	1.45	0.00	0.00	0.00	0.00	0.00	0.00	0.00	0.00	0.00	0.00	0.00	0.12	>0.999999999999999	0.0
Q8TDX9	Polycystic kidney disease protein 1-like 1 OS=Homo sapiens GN=PKD1L1 PE=2 SV=1	0.00	0.00	0.00	0.00	0.00	0.00	0.00	0.00	0.00	0.00	0.00	0.00	0.00	0.16	0.00	0.00	0.00	0.00	0.00	0.00	0.00	0.00	0.00	0.00	0.00	0.01	>0.999999999999999	0.0
P04434	Ig kappa chain V-III region VH (Fragment) OS=Homo sapiens PE=4 SV=1	0.00	0.00	0.00	0.00	0.00	0.00	0.00	0.00	0.00	0.00	0.00	0.00	0.00	4.34	0.00	0.00	0.00	0.00	0.00	0.00	0.00	0.00	0.00	0.00	0.00	0.36	>0.999999999999999	0.0
Q92859	Neogenin OS=Homo sapiens GN=NEO1 PE=1 SV=2	0.00	0.00	0.00	0.00	0.00	0.00	0.00	0.00	0.00	0.00	0.00	0.00	0.00	0.32	0.00	0.34	0.00	0.00	0.00	0.26	0.00	0.00	0.00	0.00	0.00	0.08	>0.999999999999999	0.0
P01743	Ig heavy chain V-I region HG3 OS=Homo sapiens PE=4 SV=1	0.00	0.00	0.00	0.00	0.00	0.00	0.00	0.00	0.00	0.00	0.00	0.00	0.00	4.17	0.00	0.00	0.00	0.00	0.00	0.00	0.00	0.00	0.00	0.00	0.00	0.35	>0.999999999999999	0.0
O60235	Transmembrane protease serine 11D OS=Homo sapiens GN=TMPRSS11D PE=1 SV=1	0.00	0.00	0.00	0.00	0.00	0.00	0.00	0.00	0.00	0.00	0.00	0.00	0.00	1.12	0.00	0.00	0.00	0.00	0.00	0.00	0.00	0.56	0.00	0.00	0.00	0.14	>0.999999999999999	0.0
Q9H0R5	Guanylate-binding protein 3 OS=Homo sapiens GN=GBP3 PE=2 SV=3	0.00	0.00	0.00	0.00	0.00	0.00	0.00	0.00	0.00	0.00	0.00	0.00	0.00	0.00	2.38	0.00	0.00	0.00	0.00	0.00	0.00	0.00	0.00	0.00	0.00	0.20	>0.999999999999999	0.0
P32321	Deoxycytidylate deaminase OS=Homo sapiens GN=DCTD PE=1 SV=2	0.00	0.00	0.00	0.00	0.00	0.00	0.00	0.00	0.00	0.00	0.00	0.00	0.00	0.00	3.82	0.00	0.00	0.00	0.00	0.00	0.00	0.00	0.00	0.00	0.00	0.32	>0.999999999999999	0.0
Q86TX2	Acyl-coenzyme A thioesterase 1 OS=Homo sapiens GN=ACOT1 PE=1 SV=1	0.00	0.00	0.00	0.00	0.00	0.00	0.00	0.00	0.00	0.00	0.00	0.00	0.00	0.00	1.67	0.00	0.00	0.00	0.00	0.00	0.00	0.00	0.00	0.00	0.00	0.14	>0.999999999999999	0.0
P42773	Cyclin-dependent kinase 4 inhibitor C OS=Homo sapiens GN=CDKN2C PE=1 SV=1	0.00	0.00	0.00	0.00	0.00	0.00	0.00	0.00	0.00	0.00	0.00	0.00	0.00	0.00	4.53	0.00	0.00	0.00	0.00	0.00	0.00	0.00	0.00	0.00	0.00	0.38	>0.999999999999999	0.0
P55039	Developmentally-regulated GTP-binding protein 2 OS=Homo sapiens GN=DRG2 PE=1 SV=1	0.00	0.00	0.00	0.00	0.00	0.00	0.00	0.00	0.00	0.00	0.00	0.00	0.00	0.00	1.91	0.00	0.00	0.00	0.00	0.00	0.00	0.64	0.00	0.32	0.00	0.24	>0.999999999999999	0.0
P52943	Cysteine-rich protein 2 OS=Homo sapiens GN=CRIP2 PE=1 SV=1	0.00	0.00	0.00	0.00	0.00	0.00	0.00	0.00	0.00	0.00	0.00	0.00	0.00	0.00	3.34	0.00	0.00	0.00	0.00	0.00	0.00	1.12	0.00	0.00	0.00	0.37	>0.999999999999999	0.0
Q9NVS9	Pyridoxine-5'-phosphate oxidase OS=Homo sapiens GN=PNPO PE=1 SV=1	0.00	0.00	0.00	0.00	0.00	0.00	0.00	0.00	0.00	0.00	0.00	0.00	0.00	0.00	2.62	0.00	0.00	0.00	0.00	0.00	0.00	0.00	0.00	0.00	0.00	0.22	>0.999999999999999	0.0
Q9H9E3	Conserved oligomeric Golgi complex subunit 4 OS=Homo sapiens GN=COG4 PE=1 SV=3	0.00	0.00	0.00	0.00	0.00	0.00	0.00	0.00	0.00	0.00	0.00	0.00	0.00	0.00	0.95	0.00	0.00	0.00	0.00	0.00	0.00	0.00	0.00	0.00	0.00	0.08	>0.999999999999999	0.0
P15814	Immunoglobulin lambda-like polypeptide 1 OS=Homo sapiens GN=IGLL1 PE=1 SV=1	0.00	0.00	0.00	0.00	0.00	0.00	0.00	0.00	0.00	0.00	0.00	0.00	0.00	0.00	3.34	0.00	0.00	0.00	0.00	0.00	0.00	0.00	0.00	0.00	0.00	0.28	>0.999999999999999	0.0
Q9UBN7	Histone deacetylase 6 OS=Homo sapiens GN=HDAC6 PE=1 SV=2	0.00	0.00	0.00	0.00	0.00	0.00	0.00	0.00	0.00	0.00	0.00	0.00	0.00	0.00	0.48	0.00	0.00	0.00	0.00	0.00	0.00	0.00	0.00	0.00	0.00	0.04	>0.999999999999999	0.0
O14639	Actin-binding LIM protein 1 OS=Homo sapiens GN=ABLIM1 PE=1 SV=3	0.00	0.00	0.00	0.00	0.00	0.00	0.00	0.00	0.00	0.00	0.00	0.00	0.00	0.00	0.95	0.00	0.00	0.00	0.00	0.00	0.00	0.00	0.00	0.00	0.00	0.08	>0.999999999999999	0.0
P15586	N-acetylglucosamine-6-sulfatase OS=Homo sapiens GN=GNS PE=1 SV=3	0.00	0.00	0.00	0.00	0.00	0.00	0.00	0.00	0.00	0.00	0.00	0.00	0.00	0.00	1.19	0.00	0.00	0.00	0.00	0.00	0.00	0.00	0.00	0.00	0.00	0.10	>0.999999999999999	0.0
Q15654	Thyroid receptor-interacting protein 6 OS=Homo sapiens GN=TRIP6 PE=1 SV=3	0.00	0.00	0.00	0.00	0.00	0.00	0.00	0.00	0.00	0.00	0.00	0.00	0.00	0.00	1.43	0.00	0.00	0.00	0.00	0.00	0.00	0.00	0.00	0.00	0.00	0.12	>0.999999999999999	0.0
Q9BV36	Melanophilin OS=Homo sapiens GN=MLPH PE=1 SV=1	0.00	0.00	0.00	0.00	0.00	0.00	0.00	0.00	0.00	0.00	0.00	0.00	0.00	0.00	1.19	0.00	0.00	0.00	0.00	0.00	0.00	0.00	0.00	0.00	0.00	0.10	>0.999999999999999	0.0
Q8WXX0	Dynein heavy chain 7, axonemal OS=Homo sapiens GN=DNAH7 PE=1 SV=2	0.00	0.00	0.00	0.00	0.00	0.00	0.00	0.00	0.00	0.00	0.00	0.00	0.00	0.00	0.24	0.00	0.00	0.00	0.00	0.00	0.00	0.00	0.00	0.00	0.00	0.02	>0.999999999999999	0.0
Q8IVL5	Prolyl 3-hydroxylase 2 OS=Homo sapiens GN=LEPREL1 PE=1 SV=1	0.00	0.00	0.00	0.00	0.00	0.00	0.00	0.00	0.00	0.00	0.00	0.00	0.00	0.00	0.95	0.00	0.00	0.00	0.00	0.00	0.00	0.00	0.00	0.00	0.00	0.08	>0.999999999999999	0.0
A0AVI2	Fer-1-like protein 5 OS=Homo sapiens GN=FER1L5 PE=2 SV=2	0.00	0.00	0.00	0.00	0.00	0.00	0.00	0.00	0.00	0.00	0.00	0.00	0.00	0.00	0.24	0.00	0.00	0.00	0.00	0.00	0.00	0.00	0.00	0.00	0.00	0.02	>0.999999999999999	0.0
Q7Z7L9	Zinc finger and SCAN domain-containing protein 2 OS=Homo sapiens GN=ZSCAN2 PE=2 SV=2	0.00	0.00	0.00	0.00	0.00	0.00	0.00	0.00	0.00	0.00	0.00	0.00	0.00	0.00	1.19	0.00	0.00	0.00	0.00	0.00	0.00	0.00	0.00	0.00	0.00	0.10	>0.999999999999999	0.0
Q9UF83	Uncharacterized protein DKFZp434B061 OS=Homo sapiens PE=2 SV=2	0.00	0.00	0.00	0.00	0.00	0.00	0.00	0.00	0.00	0.00	0.00	0.00	0.00	0.00	1.43	0.00	0.00	0.00	0.00	0.00	0.00	0.00	0.00	0.00	0.00	0.12	>0.999999999999999	0.0
Q5SRH9	Tetratricopeptide repeat protein 39A OS=Homo sapiens GN=TTC39A PE=2 SV=1	0.00	0.00	0.00	0.00	0.00	0.00	0.00	0.00	0.00	0.00	0.00	0.00	0.00	0.00	1.19	0.00	0.00	0.00	0.00	0.00	0.00	0.00	0.00	0.00	0.00	0.10	>0.999999999999999	0.0
Q9UQ16	Dynamin-3 OS=Homo sapiens GN=DNM3 PE=1 SV=4	0.00	0.00	0.00	0.00	0.00	0.00	0.00	0.00	0.00	0.00	0.00	0.00	0.00	0.00	0.72	0.00	0.00	0.00	0.00	0.00	0.00	0.00	0.00	0.00	0.00	0.06	>0.999999999999999	0.0
P01768	Ig heavy chain V-III region CAM OS=Homo sapiens PE=1 SV=1	0.00	0.00	0.00	0.00	0.00	0.00	0.00	0.00	0.00	0.00	0.00	0.00	0.00	0.00	0.00	9.42	0.00	0.00	0.00	0.00	0.00	0.00	0.00	0.00	0.00	0.79	>0.999999999999999	0.0
P35326	Small proline-rich protein 2A OS=Homo sapiens GN=SPRR2A PE=2 SV=1	0.00	0.00	0.00	0.00	0.00	0.00	0.00	0.00	0.00	0.00	0.00	0.00	0.00	0.00	0.00	16.66	0.00	0.00	0.00	0.00	0.00	0.00	0.00	0.00	0.00	1.39	>0.999999999999999	0.0
P63165	Small ubiquitin-related modifier 1 OS=Homo sapiens GN=SUMO1 PE=1 SV=1	0.00	0.00	0.00	0.00	0.00	0.00	0.00	0.00	0.00	0.00	0.00	0.00	0.00	0.00	0.00	5.05	0.00	0.00	0.00	0.00	0.00	0.00	0.00	0.00	0.00	0.42	>0.999999999999999	0.0
O76070	Gamma-synuclein OS=Homo sapiens GN=SNCG PE=1 SV=2	0.00	0.00	0.00	0.00	0.00	0.00	0.00	0.00	0.00	0.00	0.00	0.00	0.00	0.00	0.00	4.37	0.00	0.00	0.00	0.00	0.00	0.00	0.00	0.00	0.00	0.36	>0.999999999999999	0.0
Q9Y4I1	Unconventional myosin-Va OS=Homo sapiens GN=MYO5A PE=1 SV=2	0.00	0.00	0.00	0.00	0.00	0.00	0.00	0.00	0.00	0.00	0.00	0.00	0.00	0.00	0.00	0.50	0.00	0.00	0.00	0.00	0.00	0.00	0.00	0.00	0.00	0.04	>0.999999999999999	0.0
P00746	Complement factor D OS=Homo sapiens GN=CFD PE=1 SV=5	0.00	0.00	0.00	0.00	0.00	0.00	0.00	0.00	0.00	0.00	0.00	0.00	0.00	0.00	0.00	2.02	0.00	0.00	0.00	0.00	0.00	0.00	1.70	0.00	0.00	0.31	>0.999999999999999	0.0
Q15750	TGF-beta-activated kinase 1 and MAP3K7-binding protein 1 OS=Homo sapiens GN=TAB1 PE=1 SV=1	0.00	0.00	0.00	0.00	0.00	0.00	0.00	0.00	0.00	0.00	0.00	0.00	0.00	0.00	0.00	1.01	0.00	0.00	0.00	0.00	0.00	0.00	0.00	0.00	0.00	0.08	>0.999999999999999	0.0
Q15349	Ribosomal protein S6 kinase alpha-2 OS=Homo sapiens GN=RPS6KA2 PE=1 SV=2	0.00	0.00	0.00	0.00	0.00	0.00	0.00	0.00	0.00	0.00	0.00	0.00	0.00	0.00	0.00	0.67	0.00	0.00	0.00	0.00	0.00	0.00	0.00	0.00	0.00	0.06	>0.999999999999999	0.0
Q15833	Syntaxin-binding protein 2 OS=Homo sapiens GN=STXBP2 PE=1 SV=2	0.00	0.00	0.00	0.00	0.00	0.00	0.00	0.00	0.00	0.00	0.00	0.00	0.00	0.00	0.00	0.84	0.00	0.00	0.00	0.00	0.00	0.40	0.00	0.00	0.00	0.10	>0.999999999999999	0.0
Q9Y2M2	Uncharacterized protein C3orf32 OS=Homo sapiens GN=C3orf32 PE=2 SV=1	0.00	0.00	0.00	0.00	0.00	0.00	0.00	0.00	0.00	0.00	0.00	0.00	0.00	0.00	0.00	1.35	0.00	0.00	0.00	0.00	0.00	0.00	0.00	0.00	0.00	0.11	>0.999999999999999	0.0
P01707	Ig lambda chain V-II region TRO OS=Homo sapiens PE=1 SV=1	0.00	0.00	0.00	0.00	0.00	0.00	0.00	0.00	0.00	0.00	0.00	0.00	0.00	0.00	0.00	4.88	0.00	0.00	0.00	0.00	0.00	0.00	0.00	0.00	0.00	0.41	>0.999999999999999	0.0
Q96DC9	Ubiquitin thioesterase OTUB2 OS=Homo sapiens GN=OTUB2 PE=1 SV=2	0.00	0.00	0.00	0.00	0.00	0.00	0.00	0.00	0.00	0.00	0.00	0.00	0.00	0.00	0.00	2.02	0.00	0.00	0.00	0.00	0.00	0.00	0.00	0.00	0.00	0.17	>0.999999999999999	0.0
P56211	cAMP-regulated phosphoprotein 19 OS=Homo sapiens GN=ARPP19 PE=1 SV=2	0.00	0.00	0.00	0.00	0.00	0.00	0.00	0.00	0.00	0.00	0.00	0.00	0.00	0.00	0.00	4.71	0.00	0.00	0.00	0.00	0.00	0.00	0.00	0.00	0.00	0.39	>0.999999999999999	0.0
P02100	Hemoglobin subunit epsilon OS=Homo sapiens GN=HBE1 PE=1 SV=2	0.00	0.00	0.00	0.00	0.00	0.00	0.00	0.00	0.00	0.00	0.00	0.00	0.00	0.00	0.00	0.00	5.82	0.00	0.00	5.97	0.00	0.00	0.00	0.00	0.00	0.98	>0.999999999999999	0.0
Q06033	Inter-alpha-trypsin inhibitor heavy chain H3 OS=Homo sapiens GN=ITIH3 PE=1 SV=2	0.00	0.00	0.00	0.00	0.00	0.00	0.00	0.00	0.00	0.00	0.00	0.00	0.00	0.00	0.00	0.00	0.38	0.00	0.00	0.39	0.00	0.00	0.00	0.00	0.00	0.06	>0.999999999999999	0.0
P06899	Histone H2B type 1-J OS=Homo sapiens GN=HIST1H2BJ PE=1 SV=3	0.00	0.00	0.00	0.00	0.00	0.00	0.00	0.00	0.00	0.00	0.00	0.00	0.00	0.00	0.00	0.00	11.77	0.00	0.00	0.00	0.00	0.00	0.00	0.00	0.00	0.98	>0.999999999999999	0.0
Q6P474	Pyridoxal-dependent decarboxylase domain-containing protein 2 OS=Homo sapiens GN=PDXDC2P PE=5 SV=3	0.00	0.00	0.00	0.00	0.00	0.00	0.00	0.00	0.00	0.00	0.00	0.00	0.00	0.00	0.00	0.00	0.76	0.00	0.00	0.00	0.00	0.00	0.00	0.00	0.00	0.06	>0.999999999999999	0.0
O00148	ATP-dependent RNA helicase DDX39A OS=Homo sapiens GN=DDX39A PE=1 SV=2	0.00	0.00	0.00	0.00	0.00	0.00	0.00	0.00	0.00	0.00	0.00	0.00	0.00	0.00	0.00	0.00	1.77	0.00	0.00	0.00	0.00	0.00	0.00	0.00	0.00	0.15	>0.999999999999999	0.0
P08519	Apolipoprotein(a) OS=Homo sapiens GN=LPA PE=1 SV=1	0.00	0.00	0.00	0.00	0.00	0.00	0.00	0.00	0.00	0.00	0.00	0.00	0.00	0.00	0.00	0.00	0.25	0.00	0.00	0.13	0.00	0.00	0.00	0.00	0.00	0.03	>0.999999999999999	0.0
Q8WUD1	Ras-related protein Rab-2B OS=Homo sapiens GN=RAB2B PE=1 SV=1	0.00	0.00	0.00	0.00	0.00	0.00	0.00	0.00	0.00	0.00	0.00	0.00	0.00	0.00	0.00	0.00	1.77	0.00	2.53	0.00	0.00	0.00	0.56	0.00	0.00	0.41	>0.999999999999999	0.0
P10153	Non-secretory ribonuclease OS=Homo sapiens GN=RNASE2 PE=1 SV=2	0.00	0.00	0.00	0.00	0.00	0.00	0.00	0.00	0.00	0.00	0.00	0.00	0.00	0.00	0.00	0.00	2.28	0.00	0.00	0.00	0.00	0.00	0.00	0.00	0.00	0.19	>0.999999999999999	0.0
Q8IWV2	Contactin-4 OS=Homo sapiens GN=CNTN4 PE=1 SV=1	0.00	0.00	0.00	0.00	0.00	0.00	0.00	0.00	0.00	0.00	0.00	0.00	0.00	0.00	0.00	0.00	0.38	0.46	0.54	0.00	0.00	0.00	0.00	0.00	0.00	0.12	>0.999999999999999	0.0
Q96GC5	39S ribosomal protein L48, mitochondrial OS=Homo sapiens GN=MRPL48 PE=1 SV=2	0.00	0.00	0.00	0.00	0.00	0.00	0.00	0.00	0.00	0.00	0.00	0.00	0.00	0.00	0.00	0.00	1.77	0.00	0.00	0.00	0.00	0.00	0.00	0.00	0.00	0.15	>0.999999999999999	0.0
Q6NUP7	Serine/threonine-protein phosphatase 4 regulatory subunit 4 OS=Homo sapiens GN=PPP4R4 PE=1 SV=1	0.00	0.00	0.00	0.00	0.00	0.00	0.00	0.00	0.00	0.00	0.00	0.00	0.00	0.00	0.00	0.00	0.38	0.46	0.00	0.00	0.00	0.00	0.00	0.00	0.00	0.07	>0.999999999999999	0.0
O15438	Canalicular multispecific organic anion transporter 2 OS=Homo sapiens GN=ABCC3 PE=1 SV=3	0.00	0.00	0.00	0.00	0.00	0.00	0.00	0.00	0.00	0.00	0.00	0.00	0.00	0.00	0.00	0.00	0.25	0.00	0.00	0.00	0.00	0.00	0.00	0.00	0.00	0.02	>0.999999999999999	0.0
Q9UGM5	Fetuin-B OS=Homo sapiens GN=FETUB PE=1 SV=2	0.00	0.00	0.00	0.00	0.00	0.00	0.00	0.00	0.00	0.00	0.00	0.00	0.00	0.00	0.00	0.00	1.01	0.00	1.45	0.00	0.00	0.00	0.00	0.00	0.00	0.20	>0.999999999999999	0.0
P05543	Thyroxine-binding globulin OS=Homo sapiens GN=SERPINA7 PE=1 SV=2	0.00	0.00	0.00	0.00	0.00	0.00	0.00	0.00	0.00	0.00	0.00	0.00	0.00	0.00	0.00	0.00	0.89	0.00	0.00	0.00	1.18	0.00	0.00	0.00	0.00	0.17	>0.999999999999999	0.0
Q96P31	Fc receptor-like protein 3 OS=Homo sapiens GN=FCRL3 PE=1 SV=1	0.00	0.00	0.00	0.00	0.00	0.00	0.00	0.00	0.00	0.00	0.00	0.00	0.00	0.00	0.00	0.00	0.51	0.00	0.00	0.00	0.00	0.00	0.00	0.00	0.00	0.04	>0.999999999999999	0.0
Q9Y2A9	UDP-GlcNAc:betaGal beta-1,3-N-acetylglucosaminyltransferase 3 OS=Homo sapiens GN=B3GNT3 PE=1 SV=2	0.00	0.00	0.00	0.00	0.00	0.00	0.00	0.00	0.00	0.00	0.00	0.00	0.00	0.00	0.00	0.00	1.01	0.00	0.00	0.00	0.00	0.64	0.00	0.00	0.00	0.14	>0.999999999999999	0.0
P48723	Heat shock 70 kDa protein 13 OS=Homo sapiens GN=HSPA13 PE=1 SV=1	0.00	0.00	0.00	0.00	0.00	0.00	0.00	0.00	0.00	0.00	0.00	0.00	0.00	0.00	0.00	0.00	0.00	0.92	0.00	0.00	0.00	0.00	0.00	0.00	0.00	0.08	>0.999999999999999	0.0
P32320	Cytidine deaminase OS=Homo sapiens GN=CDA PE=1 SV=2	0.00	0.00	0.00	0.00	0.00	0.00	0.00	0.00	0.00	0.00	0.00	0.00	0.00	0.00	0.00	0.00	0.00	3.07	0.00	0.00	0.00	0.00	0.00	0.00	0.00	0.26	>0.999999999999999	0.0
Q16651	Prostasin OS=Homo sapiens GN=PRSS8 PE=1 SV=1	0.00	0.00	0.00	0.00	0.00	0.00	0.00	0.00	0.00	0.00	0.00	0.00	0.00	0.00	0.00	0.00	0.00	1.38	0.00	0.00	0.00	0.00	0.00	0.00	0.00	0.12	>0.999999999999999	0.0
P01775	Ig heavy chain V-III region LAY OS=Homo sapiens PE=1 SV=1	0.00	0.00	0.00	0.00	0.00	0.00	0.00	0.00	0.00	0.00	0.00	0.00	0.00	0.00	0.00	0.00	0.00	3.99	0.00	0.00	0.00	0.00	0.00	0.00	0.00	0.33	>0.999999999999999	0.0
F5GYI3	Ubiquitin-associated protein 1-like OS=Homo sapiens GN=UBAP1L PE=2 SV=1	0.00	0.00	0.00	0.00	0.00	0.00	0.00	0.00	0.00	0.00	0.00	0.00	0.00	0.00	0.00	0.00	0.00	1.23	0.00	0.00	0.00	0.00	0.00	0.00	0.00	0.10	>0.999999999999999	0.0
Q15904	V-type proton ATPase subunit S1 OS=Homo sapiens GN=ATP6AP1 PE=1 SV=2	0.00	0.00	0.00	0.00	0.00	0.00	0.00	0.00	0.00	0.00	0.00	0.00	0.00	0.00	0.00	0.00	0.00	0.92	0.00	0.00	0.00	0.00	0.00	0.00	0.00	0.08	>0.999999999999999	0.0
P41226	Ubiquitin-like modifier-activating enzyme 7 OS=Homo sapiens GN=UBA7 PE=1 SV=2	0.00	0.00	0.00	0.00	0.00	0.00	0.00	0.00	0.00	0.00	0.00	0.00	0.00	0.00	0.00	0.00	0.00	0.54	0.00	0.00	0.00	0.00	0.12	0.00	0.00	0.06	>0.999999999999999	0.0
P01601	Ig kappa chain V-I region HK101 (Fragment) OS=Homo sapiens PE=4 SV=1	0.00	0.00	0.00	0.00	0.00	0.00	0.00	0.00	0.00	0.00	0.00	0.00	0.00	0.00	0.00	0.00	0.00	4.70	0.00	0.00	0.00	0.00	0.00	0.00	0.00	0.39	>0.999999999999999	0.0
O15400	Syntaxin-7 OS=Homo sapiens GN=STX7 PE=1 SV=4	0.00	0.00	0.00	0.00	0.00	0.00	0.00	0.00	0.00	0.00	0.00	0.00	0.00	0.00	0.00	0.00	0.00	1.99	0.00	0.00	0.00	0.00	0.00	0.00	0.00	0.17	>0.999999999999999	0.0
P54687	Branched-chain-amino-acid aminotransferase, cytosolic OS=Homo sapiens GN=BCAT1 PE=1 SV=3	0.00	0.00	0.00	0.00	0.00	0.00	0.00	0.00	0.00	0.00	0.00	0.00	0.00	0.00	0.00	0.00	0.00	1.45	0.00	0.00	0.00	0.00	0.00	0.00	0.00	0.12	>0.999999999999999	0.0
Q9NR28	Diablo homolog, mitochondrial OS=Homo sapiens GN=DIABLO PE=1 SV=1	0.00	0.00	0.00	0.00	0.00	0.00	0.00	0.00	0.00	0.00	0.00	0.00	0.00	0.00	0.00	0.00	0.00	2.17	0.00	0.00	0.00	0.00	0.00	0.00	0.00	0.18	>0.999999999999999	0.0
Q96SK2	Transmembrane protein 209 OS=Homo sapiens GN=TMEM209 PE=1 SV=2	0.00	0.00	0.00	0.00	0.00	0.00	0.00	0.00	0.00	0.00	0.00	0.00	0.00	0.00	0.00	0.00	0.00	0.90	0.00	0.00	0.00	0.00	0.00	0.00	0.00	0.08	>0.999999999999999	0.0
P01699	Ig lambda chain V-I region VOR OS=Homo sapiens PE=1 SV=1	0.00	0.00	0.00	0.00	0.00	0.00	0.00	0.00	0.00	0.00	0.00	0.00	0.00	0.00	0.00	0.00	0.00	5.43	0.00	0.00	0.00	0.00	0.00	0.00	0.00	0.45	>0.999999999999999	0.0
P55212	Caspase-6 OS=Homo sapiens GN=CASP6 PE=1 SV=2	0.00	0.00	0.00	0.00	0.00	0.00	0.00	0.00	0.00	0.00	0.00	0.00	0.00	0.00	0.00	0.00	0.00	1.81	0.00	0.00	0.00	0.00	0.00	0.00	0.00	0.15	>0.999999999999999	0.0
O95721	Synaptosomal-associated protein 29 OS=Homo sapiens GN=SNAP29 PE=1 SV=1	0.00	0.00	0.00	0.00	0.00	0.00	0.00	0.00	0.00	0.00	0.00	0.00	0.00	0.00	0.00	0.00	0.00	1.99	0.00	0.00	0.00	0.00	0.00	0.00	0.00	0.17	>0.999999999999999	0.0
Q9UBQ5	Eukaryotic translation initiation factor 3 subunit K OS=Homo sapiens GN=EIF3K PE=1 SV=1	0.00	0.00	0.00	0.00	0.00	0.00	0.00	0.00	0.00	0.00	0.00	0.00	0.00	0.00	0.00	0.00	0.00	2.35	0.00	0.00	0.00	0.00	0.00	0.00	0.00	0.20	>0.999999999999999	0.0
Q7Z6G8	Ankyrin repeat and sterile alpha motif domain-containing protein 1B OS=Homo sapiens GN=ANKS1B PE=1 SV=2	0.00	0.00	0.00	0.00	0.00	0.00	0.00	0.00	0.00	0.00	0.00	0.00	0.00	0.00	0.00	0.00	0.00	0.36	0.00	0.00	0.00	0.00	0.00	0.00	0.00	0.03	>0.999999999999999	0.0
P22531	Small proline-rich protein 2E OS=Homo sapiens GN=SPRR2E PE=2 SV=2	0.00	0.00	0.00	0.00	0.00	0.00	0.00	0.00	0.00	0.00	0.00	0.00	0.00	0.00	0.00	0.00	0.00	7.42	0.00	0.00	0.00	0.00	0.00	0.00	0.00	0.62	>0.999999999999999	0.0
Q02817	Mucin-2 OS=Homo sapiens GN=MUC2 PE=1 SV=2	0.00	0.00	0.00	0.00	0.00	0.00	0.00	0.00	0.00	0.00	0.00	0.00	0.00	0.00	0.00	0.00	0.00	0.26	0.00	0.00	0.00	0.00	0.00	0.00	0.00	0.02	>0.999999999999999	0.0
O75015	Low affinity immunoglobulin gamma Fc region receptor III-B OS=Homo sapiens GN=FCGR3B PE=1 SV=2	0.00	0.00	0.00	0.00	0.00	0.00	0.00	0.00	0.00	0.00	0.00	0.00	0.00	0.00	0.00	0.00	0.00	3.51	0.00	0.00	0.00	0.00	0.00	0.00	0.00	0.29	>0.999999999999999	0.0
P80108	Phosphatidylinositol-glycan-specific phospholipase D OS=Homo sapiens GN=GPLD1 PE=1 SV=3	0.00	0.00	0.00	0.00	0.00	0.00	0.00	0.00	0.00	0.00	0.00	0.00	0.00	0.00	0.00	0.00	0.00	0.91	0.00	0.00	0.00	0.00	0.00	0.00	0.00	0.08	>0.999999999999999	0.0
Q15485	Ficolin-2 OS=Homo sapiens GN=FCN2 PE=1 SV=2	0.00	0.00	0.00	0.00	0.00	0.00	0.00	0.00	0.00	0.00	0.00	0.00	0.00	0.00	0.00	0.00	0.00	1.30	0.00	0.00	0.00	0.00	0.00	0.00	0.00	0.11	>0.999999999999999	0.0
P06276	Cholinesterase OS=Homo sapiens GN=BCHE PE=1 SV=1	0.00	0.00	0.00	0.00	0.00	0.00	0.00	0.00	0.00	0.00	0.00	0.00	0.00	0.00	0.00	0.00	0.00	0.65	0.00	0.00	0.00	0.00	0.00	0.00	0.00	0.05	>0.999999999999999	0.0
Q9BRZ2	E3 ubiquitin-protein ligase TRIM56 OS=Homo sapiens GN=TRIM56 PE=1 SV=3	0.00	0.00	0.00	0.00	0.00	0.00	0.00	0.00	0.00	0.00	0.00	0.00	0.00	0.00	0.00	0.00	0.00	0.52	0.00	0.00	0.00	0.00	0.00	0.00	0.00	0.04	>0.999999999999999	0.0
P21741	Midkine OS=Homo sapiens GN=MDK PE=1 SV=1	0.00	0.00	0.00	0.00	0.00	0.00	0.00	0.00	0.00	0.00	0.00	0.00	0.00	0.00	0.00	0.00	0.00	2.73	0.00	0.00	0.00	0.00	0.00	0.00	0.00	0.23	>0.999999999999999	0.0
P60763	Ras-related C3 botulinum toxin substrate 3 OS=Homo sapiens GN=RAC3 PE=1 SV=1	0.00	0.00	0.00	0.00	0.00	0.00	0.00	0.00	0.00	0.00	0.00	0.00	0.00	0.00	0.00	0.00	0.00	0.00	0.00	0.00	9.11	0.00	0.00	0.00	0.00	0.76	>0.999999999999999	0.0
Q7Z5R6	Amyloid beta A4 precursor protein-binding family B member 1-interacting protein OS=Homo sapiens GN=APBB1IP F	0.00	0.00	0.00	0.00	0.00	0.00	0.00	0.00	0.00	0.00	0.00	0.00	0.00	0.00	0.00	0.00	0.00	0.00	0.00	0.00	0.68	0.00	0.00	0.00	0.00	0.06	>0.999999999999999	0.0
P16035	Metalloproteinase inhibitor 2 OS=Homo sapiens GN=TIMP2 PE=1 SV=2	0.00	0.00	0.00	0.00	0.00	0.00	0.00	0.00	0.00	0.00	0.00	0.00	0.00	0.00	0.00	0.00	0.00	0.00	0.00	0.00	2.19	0.00	0.00	0.00	0.00	0.18	>0.999999999999999	0.0
Q86U42	Polyadenylate-binding protein 2 OS=Homo sapiens GN=PABPN1 PE=1 SV=3	0.00	0.00	0.00	0.00	0.00	0.00	0.00	0.00	0.00	0.00	0.00	0.00	0.00	0.00	0.00	0.00	0.00	0.00	0.00	0.00	1.69	0.00	0.00	0.00	0.00	0.14	>0.999999999999999	0.0
Q15080	Neutrophil cytosol factor 4 OS=Homo sapiens GN=NCF4 PE=1 SV=2	0.00	0.00	0.00	0.00	0.00	0.00	0.00	0.00	0.00	0.00	0.00	0.00	0.00	0.00	0.00	0.00	0.00	0.00	0.00	0.00	3.04	0.00	0.00	0.00	0.00	0.25	>0.999999999999999	0.0
C9JRZ8	Aldo-keto reductase family 1 member B15 OS=Homo sapiens GN=AKR1B15 PE=2 SV=1	0.00	0.00	0.00	0.00	0.00	0.00	0.00	0.00	0.00	0.00	0.00	0.00	0.00	0.00	0.00	0.00	0.00	0.00	0.00	0.00	1.35	0.00	0.00	0.00	0.00	0.11	>0.999999999999999	0.0
Q05315	Eosinophil lysophospholipase OS=Homo sapiens GN=CLC PE=1 SV=3	0.00	0.00	0.00	0.00	0.00	0.00	0.00	0.00	0.00	0.00	0.00	0.00	0.00	0.00	0.00	0.00	0.00	0.00	0.00	0.00	3.71	0.00	0.00	0.00	0.00	0.31	>0.999999999999999	0.0
B2RPK0	Putative high mobility group protein B1-like 1 OS=Homo sapiens GN=HMGB1P1 PE=5 SV=1	0.00	0.00	0.00	0.00	0.00	0.00	0.00	0.00	0.00	0.00	0.00	0.00	0.00	0.00	0.00	0.00	0.00	0.00	0.00	0.00	2.36	1.12	0.00	0.00	0.00	0.29	>0.999999999999999	0.0
A8MW06	Thymosin beta-4-like protein 3 OS=Homo sapiens GN=TMSL3 PE=2 SV=1	0.00	0.00	0.00	0.00	0.00	0.00	0.00	0.00	0.00	0.00	0.00	0.00	0.00	0.00	0.00	0.00	0.00	0.00	0.00	0.00	12.66	0.00	0.00	0.00	0.00	1.05	>0.999999999999999	0.0
P51512	Matrix metalloproteinase-16 OS=Homo sapiens GN=MMP16 PE=1 SV=2	0.00	0.00	0.00	0.00	0.00	0.00	0.00	0.00	0.00	0.00	0.00	0.00	0.00	0.00	0.00	0.00	0.00	0.00	0.00	0.00	0.84	0.00	0.00	0.00	0.00	0.07	>0.999999999999999	0.0
P27918	Properdin OS=Homo sapiens GN=CFP PE=1 SV=2	0.00	0.00	0.00	0.00	0.00	0.00	0.00	0.00	0.00	0.00	0.00	0.00	0.00	0.00	0.00	0.00	0.00	0.00	0.00	0.00	1.01	0.00	0.00	0.00	0.00	0.08	>0.999999999999999	0.0
Q99729	Heterogeneous nuclear ribonucleoprotein A/B OS=Homo sapiens GN=HNRNPAB PE=1 SV=2	0.00	0.00	0.00	0.00	0.00	0.00	0.00	0.00	0.00	0.00	0.00	0.00	0.00	0.00	0.00	0.00	0.00	0.00	0.00	0.00	0.00	2.40	2.70	0.00	0.00	0.42	>0.999999999999999	0.0
P32189	Glycerol kinase OS=Homo sapiens GN=GK PE=1 SV=3	0.00	0.00	0.00	0.00	0.00	0.00	0.00	0.00	0.00	0.00	0.00	0.00	0.00	0.00	0.00	0.00	0.00	0.00	0.00	0.00	0.00	1.84	0.71	0.00	0.00	0.21	>0.999999999999999	0.0
P34096	Ribonuclease 4 OS=Homo sapiens GN=RNASE4 PE=1 SV=3	0.00	0.00	0.00	0.00	0.00	0.00	0.00	0.00	0.00	0.00	0.00	0.00	0.00	0.00	0.00	0.00	0.00	0.00	0.00	0.00	0.00	3.43	2.84	0.00	0.00	0.52	>0.999999999999999	0.0
O15327	Type II inositol-3,4-bisphosphate 4-phosphatase OS=Homo sapiens GN=INPP4B PE=2 SV=4	0.00	0.00	0.00	0.00	0.00	0.00	0.00	0.00	0.00	0.00	0.00	0.00	0.00	0.00	0.00	0.00	0.00	0.00	0.00	0.00	0.00	0.24	0.00	0.00	0.00	0.02	>0.999999999999999	0.0
Q86SF2	N-acetylgalactosaminyltransferase 7 OS=Homo sapiens GN=GALNT7 PE=1 SV=1	0.00	0.00	0.00	0.00	0.00	0.00	0.00	0.00	0.00	0.00	0.00	0.00	0.00	0.00	0.00	0.00	0.00	0.00	0.00	0.00	0.00	0.32	0.00	0.00	0.00	0.03	>0.999999999999999	0.0
P15291	Beta-1,4-galactosyltransferase 1 OS=Homo sapiens GN=B4GALT1 PE=1 SV=5	0.00	0.00	0.00	0.00	0.00	0.00	0.00	0.00	0.00	0.00	0.00	0.00	0.00	0.00	0.00	0.00	0.00	0.00	0.00	0.00	0.00	0.56	0.00	0.00	0.00	0.05	>0.999999999999999	0.0
P43251	Biotinidase OS=Homo sapiens GN=BTD PE=1 SV=2	0.00	0.00	0.00	0.00	0.00	0.00	0.00	0.00	0.00	0.00	0.00	0.00	0.00	0.00	0.00	0.00	0.00	0.00	0.00	0.00	0.00	0.40	0.00	0.00	0.00	0.03	>0.999999999999999	0.0
O95848	Uridine diphosphate glucose pyrophosphatase OS=Homo sapiens GN=NUDT14 PE=1 SV=2	0.00	0.00	0.00	0.00	0.00	0.00	0.00	0.00	0.00	0.00	0.00	0.00	0.00	0.00	0.00	0.00	0.00	0.00	0.00	0.00	0.00	1.12	0.00	0.00	0.00	0.09	>0.999999999999999	0.0
Q9UHG2	ProSAAS OS=Homo sapiens GN=PCSK1N PE=1 SV=1	0.00	0.00	0.00	0.00	0.00	0.00	0.00	0.00	0.00	0.00	0.00	0.00	0.00	0.00	0.00	0.00	0.00	0.00	0.00	0.00	0.00	2.08	0.00	0.00	0.00	0.17	>0.999999999999999	0.0
Q9Y2E5	Epididymis-specific alpha-mannosidase OS=Homo sapiens GN=MAN2B2 PE=1 SV=4	0.00	0.00	0.00	0.00	0.00	0.00	0.00	0.00	0.00	0.00	0.00	0.00	0.00	0.00	0.00	0.00	0.00	0.00	0.00	0.00	0.00	0.24	0.00	0.00	0.00	0.02	>0.999999999999999	0.0
P26232	Catenin alpha-2 OS=Homo sapiens GN=CTNNA2 PE=1 SV=5	0.00	0.00	0.00	0.00	0.00	0.00	0.00	0.00	0.00	0.00	0.00	0.00	0.00	0.00	0.00	0.00	0.00	0.00	0.00	0.00	0.00	0.24	0.00	0.00	0.00	0.02	>0.999999999999999	0.0
Q66K66	Transmembrane protein 198 OS=Homo sapiens GN=TMEM198 PE=2 SV=1	0.00	0.00	0.00	0.00	0.00	0.00	0.00	0.00	0.00	0.00	0.00	0.00	0.00	0.00	0.00	0.00	0.00	0.00	0.00	0.00	0.00	0.64	0.00	0.00	0.00	0.05	>0.999999999999999	0.0
Q9Y2G9	Protein strawberry notch homolog 2 OS=Homo sapiens GN=SBNO2 PE=2 SV=3	0.00	0.00	0.00	0.00	0.00	0.00	0.00	0.00	0.00	0.00	0.00	0.00	0.00	0.00	0.00	0.00	0.00	0.00	0.00	0.00	0.00	0.16	0.57	0.00	0.00	0.06	>0.999999999999999	0.0
P12931	Proto-oncogene tyrosine-protein kinase Src OS=Homo sapiens GN=SRC PE=1 SV=3	0.00	0.00	0.00	0.00	0.00	0.00	0.00	0.00	0.00	0.00	0.00	0.00	0.00	0.00	0.00	0.00	0.00	0.00	0.00	0.00	0.00	0.40	0.71	0.00	0.00	0.09	>0.999999999999999	0.0
O00115	Deoxyribonuclease-2-alpha OS=Homo sapiens GN=DNASE2 PE=1 SV=2	0.00	0.00	0.00	0.00	0.00	0.00	0.00	0.00	0.00	0.00	0.00	0.00	0.00	0.00	0.00	0.00	0.00	0.00	0.00	0.00	0.00	0.64	0.00	0.00	0.00	0.05	>0.999999999999999	0.0
Q9BUK6	Protein misato homolog 1 OS=Homo sapiens GN=MSTO1 PE=1 SV=1	0.00	0.00	0.00	0.00	0.00	0.00	0.00	0.00	0.00	0.00	0.00	0.00	0.00	0.00	0.00	0.00	0.00	0.00	0.00	0.00	0.00	0.40	0.00	0.00	0.00	0.03	>0.999999999999999	0.0
Q9UJ68	Mitochondrial peptide methionine sulfoxide reductase OS=Homo sapiens GN=MSRA PE=1 SV=1	0.00	0.00	0.00	0.00	0.00	0.00	0.00	0.00	0.00	0.00	0.00	0.00	0.00	0.00	0.00	0.00	0.00	0.00	0.00	0.00	0.00	1.04	1.84	0.00	0.00	0.24	>0.999999999999999	0.0
Q9BXT2	Voltage-dependent calcium channel gamma-6 subunit OS=Homo sapiens GN=CACNG6 PE=2 SV=1	0.00	0.00	0.00	0.00	0.00	0.00	0.00	0.00	0.00	0.00	0.00	0.00	0.00	0.00	0.00	0.00	0.00	0.00	0.00	0.00	0.00	0.96	0.00	0.00	0.00	0.08	>0.999999999999999	0.0
Q99961	Endophilin-A2 OS=Homo sapiens GN=SH3GL1 PE=1 SV=1	0.00	0.00	0.00	0.00	0.00	0.00	0.00	0.00	0.00	0.00	0.00	0.00	0.00	0.00	0.00	0.00	0.00	0.00	0.00	0.00	0.00	0.64	1.14	0.00	0.00	0.15	>0.999999999999999	0.0
Q8N3K9	Cardiomyopathy-associated protein 5 OS=Homo sapiens GN=CMYA5 PE=1 SV=3	0.00	0.00	0.00	0.00	0.00	0.00	0.00	0.00	0.00	0.00	0.00	0.00	0.00	0.00	0.00	0.00	0.00	0.00	0.00	0.00	0.00	0.08	0.00	0.00	0.00	0.01	>0.999999999999999	0.0
Q6A1A2	Putative 3-phosphoinositide-dependent protein kinase 2 OS=Homo sapiens GN=PDPK2 PE=5 SV=1	0.00	0.00	0.00	0.00	0.00	0.00	0.00	0.00	0.00	0.00	0.00	0.00	0.00	0.00	0.00	0.00	0.00	0.00	0.00	0.00	0.00	0.56	0.00	0.00	0.00	0.05	>0.999999999999999	0.0
Q6ZTC4	Putative uncharacterized protein FLJ44790 OS=Homo sapiens PE=2 SV=1	0.00	0.00	0.00	0.00	0.00	0.00	0.00	0.00	0.00	0.00	0.00	0.00	0.00	0.00	0.00	0.00	0.00	0.00	0.00	0.00	0.00	1.12	1.99	0.00	0.00	0.26	>0.999999999999999	0.0
Q8NI22	Multiple coagulation factor deficiency protein 2 OS=Homo sapiens GN=MCFD2 PE=1 SV=1	0.00	0.00	0.00	0.00	0.00	0.00	0.00	0.00	0.00	0.00	0.00	0.00	0.00	0.00	0.00	0.00	0.00	0.00	0.00	0.00	0.00	1.60	0.00	0.80	0.00	0.20	>0.999999999999999	0.0
O00462	Beta-mannosidase OS=Homo sapiens GN=MANBA PE=1 SV=3	0.00	0.00	0.00	0.00	0.00	0.00	0.00	0.00	0.00	0.00	0.00	0.00	0.00	0.00	0.00	0.00	0.00	0.00	0.00	0.00	0.00	0.24	0.00	0.00	0.00	0.02	>0.999999999999999	0.0
Q16674	Melanoma-derived growth regulatory protein OS=Homo sapiens GN=MIA PE=1 SV=1	0.00	0.00	0.00	0.00	0.00	0.00	0.00	0.00	0.00	0.00	0.00	0.00	0.00	0.00	0.00	0.00	0.00	0.00	0.00	0.00	0.00	1.84	0.00	0.00	0.00	0.15	>0.999999999999999	0.0
Q9H7C9	UPF0366 protein C11orf67 OS=Homo sapiens GN=C11orf67 PE=1 SV=1	0.00	0.00	0.00	0.00	0.00	0.00	0.00	0.00	0.00	0.00	0.00	0.00	0.00	0.00	0.00	0.00	0.00	0.00	0.00	0.00	0.00	2.00	0.00	0.00	0.00	0.17	>0.999999999999999	0.0
Q66K74	Microtubule-associated protein 1S OS=Homo sapiens GN=MAP1S PE=1 SV=2	0.00	0.00	0.00	0.00	0.00	0.00	0.00	0.00	0.00	0.00	0.00	0.00	0.00	0.00	0.00	0.00	0.00	0.00	0.00	0.00	0.00	0.00	0.43	0.00	0.00	0.04	>0.999999999999999	0.0
Q9NXJ5	Pyroglutamyl-peptidase 1 OS=Homo sapiens GN=PGPEP1 PE=1 SV=1	0.00	0.00	0.00	0.00	0.00	0.00	0.00	0.00	0.00	0.00	0.00	0.00	0.00	0.00	0.00	0.00	0.00	0.00	0.00	0.00	0.00	0.00	1.99	0.00	0.00	0.17	>0.999999999999999	0.0
Q92575	UBX domain-containing protein 4 OS=Homo sapiens GN=UBXN4 PE=1 SV=2	0.00	0.00	0.00	0.00	0.00	0.00	0.00	0.00	0.00	0.00	0.00	0.00	0.00	0.00	0.00	0.00	0.00	0.00	0.00	0.00	0.00	0.00	0.85	0.00	0.00	0.07	>0.999999999999999	0.0
P01608	Ig kappa chain V-I region Roy OS=Homo sapiens PE=1 SV=1	0.00	0.00	0.00	0.00	0.00	0.00	0.00	0.00	0.00	0.00	0.00	0.00	0.00	0.00	0.00	0.00	0.00	0.00	0.00	0.00	0.00	0.00	4.12	0.00	0.00	0.34	>0.999999999999999	0.0
P42345	Serine/threonine-protein kinase mTOR OS=Homo sapiens GN=MTOR PE=1 SV=1	0.00	0.00	0.00	0.00	0.00	0.00	0.00	0.00	0.00	0.00	0.00	0.00	0.00	0.00	0.00	0.00	0.00	0.00	0.00	0.00	0.00	0.00	0.28	0.00	0.00	0.02	>0.999999999999999	0.0
Q9UIJ7	GTP:AMP phosphotransferase, mitochondrial OS=Homo sapiens GN=AK3 PE=1 SV=4	0.00	0.00	0.00	0.00	0.00	0.00	0.00	0.00	0.00	0.00	0.00	0.00	0.00	0.00	0.00	0.00	0.00	0.00	0.00	0.00	0.00	0.00	1.84	0.00	0.00	0.15	>0.999999999999999	0.0
Q9NRA1	Platelet-derived growth factor C OS=Homo sapiens GN=PDGFC PE=1 SV=2	0.00	0.00	0.00	0.00	0.00	0.00	0.00	0.00	0.00	0.00	0.00	0.00	0.00	0.00	0.00	0.00	0.00	0.00	0.00	0.00	0.00	0.00	1.14	0.00	0.00	0.09	>0.999999999999999	0.0
Q6SZW1	Sterile alpha and TIR motif-containing protein 1 OS=Homo sapiens GN=SARM1 PE=1 SV=1	0.00	0.00	0.00	0.00	0.00	0.00	0.00	0.00	0.00	0.00	0.00	0.00	0.00	0.00	0.00	0.00	0.00	0.00	0.00	0.00	0.00	0.00	0.57	0.00	0.00	0.05	>0.999999999999999	0.0
Q96JB5	CDK5 regulatory subunit-associated protein 3 OS=Homo sapiens GN=CDK5RAP3 PE=1 SV=2	0.00	0.00	0.00	0.00	0.00	0.00	0.00	0.00	0.00	0.00	0.00	0.00	0.00	0.00	0.00	0.00	0.00	0.00	0.00	0.00	0.00	0.00	0.85	0.00	0.00	0.07	>0.999999999999999	0.0
P01597	Ig kappa chain V-I region DEE OS=Homo sapiens PE=1 SV=1	0.00	0.00	0.00	0.00	0.00	0.00	0.00	0.00	0.00	0.00	0.00	0.00	0.00	0.00	0.00	0.00	0.00	0.00	0.00	0.00	0.00	0.00	4.12	0.00	0.00	0.34	>0.999999999999999	0.0
Q99543	DnaJ homolog subfamily C member 2 OS=Homo sapiens GN=DNAJC2 PE=1 SV=4	0.00	0.00	0.00	0.00	0.00	0.00	0.00	0.00	0.00	0.00	0.00	0.00	0.00	0.00	0.00	0.00	0.00	0.00	0.00	0.00	0.00	0.00	0.71	0.00	0.00	0.06	>0.999999999999999	0.0
P58340	Myeloid leukemia factor 1 OS=Homo sapiens GN=MLF1 PE=1 SV=1	0.00	0.00	0.00	0.00	0.00	0.00	0.00	0.00	0.00	0.00	0.00	0.00	0.00	0.00	0.00	0.00	0.00	0.00	0.00	0.00	0.00	0.00	1.56	0.00	0.00	0.13	>0.999999999999999	0.0
P47989	Xanthine dehydrogenase/oxidase OS=Homo sapiens GN=XDH PE=1 SV=4	0.00	0.00	0.00	0.00	0.00	0.00	0.00	0.00	0.00	0.00	0.00	0.00	0.00	0.00	0.00	0.00	0.00	0.00	0.00	0.00	0.00	0.00	0.28	0.00	0.00	0.02	>0.999999999999999	0.0
Q9BYN0	Sulfiredoxin-1 OS=Homo sapiens GN=SRXN1 PE=1 SV=2	0.00	0.00	0.00	0.00	0.00	0.00	0.00	0.00	0.00	0.00	0.00	0.00	0.00	0.00	0.00	0.00	0.00	0.00	0.00	0.00	0.00	0.00	3.41	0.00	0.00	0.28	>0.999999999999999	0.0
P62736	Actin, aortic smooth muscle OS=Homo sapiens GN=ACTA2 PE=1 SV=1	0.00	0.00	0.00	0.00	0.00	0.00	0.00	0.00	0.00	0.00	0.00	0.00	0.00	0.00	0.00	0.00	0.00	0.00	0.00	0.00	0.00	0.00	0.00	1.40	0.00	0.12	>0.999999999999999	0.0
P30508	HLA class I histocompatibility antigen, Cw-12 alpha chain OS=Homo sapiens GN=HLA-C PE=1 SV=2	0.00	0.00	0.00	0.00	0.00	0.00	0.00	0.00	0.00	0.00	0.00	0.00	0.00	0.00	0.00	0.00	0.00	0.00	0.00	0.00	0.00	0.00	0.00	1.88	0.00	0.16	>0.999999999999999	0.0
O75396	Vesicle-trafficking protein SEC22b OS=Homo sapiens GN=SEC22B PE=1 SV=4	0.00	0.00	0.00	0.00	0.00	0.00	0.00	0.00	0.00	0.00	0.00	0.00	0.00	0.00	0.00	0.00	0.00	0.00	0.00	0.00	0.00	0.00	0.00	0.56	0.00	0.05	>0.999999999999999	0.0
Q9NQC3	Reticulon-4 OS=Homo sapiens GN=RTN4 PE=1 SV=2	0.00	0.00	0.00	0.00	0.00	0.00	0.00	0.00	0.00	0.00	0.00	0.00	0.00	0.00	0.00	0.00	0.00	0.00	0.00	0.00	0.00	0.00	0.00	0.12	0.00	0.01	>0.999999999999999	0.0
Q08174	Protocadherin-1 OS=Homo sapiens GN=PCDH1 PE=1 SV=2	0.00	0.00	0.00	0.00	0.00	0.00	0.00	0.00	0.00	0.00	0.00	0.00	0.00	0.00	0.00	0.00	0.00	0.00	0.00	0.00	0.00	0.00	0.00	0.24	0.00	0.02	>0.999999999999999	0.0
Q9H9H4	Vacuolar protein sorting-associated protein 37B OS=Homo sapiens GN=VPS37B PE=1 SV=1	0.00	0.00	0.00	0.00	0.00	0.00	0.00	0.00	0.00	0.00	0.00	0.00	0.00	0.00	0.00	0.00	0.00	0.00	0.00	0.00	0.00	0.00	0.00	0.44	0.00	0.04	>0.999999999999999	0.0
P52594	Arf-GAP domain and FG repeat-containing protein 1 OS=Homo sapiens GN=AGFG1 PE=1 SV=2	0.00	0.00	0.00	0.00	0.00	0.00	0.00	0.00	0.00	0.00	0.00	0.00	0.00	0.00	0.00	0.00	0.00	0.00	0.00	0.00	0.00	0.00	0.00	0.24	0.00	0.02	>0.999999999999999	0.0
P02810	Salivary acidic proline-rich phosphoprotein 1/2 OS=Homo sapiens GN=PRH1 PE=1 SV=2	0.00	0.00	0.00	0.00	0.00	0.00	0.00	0.00	0.00	0.00	0.00	0.00	0.00	0.00	0.00	0.00	0.00	0.00	0.00	0.00	0.00	0.00	0.00	1.76	0.00	0.15	>0.999999999999999	0.0
P16615	Sarcoplasmic/endoplasmic reticulum calcium ATPase 2 OS=Homo sapiens GN=ATP2A2 PE=1 SV=1	0.00	0.00	0.00	0.00	0.00	0.00	0.00	0.00	0.00	0.00	0.00	0.00	0.00	0.00	0.00	0.00	0.00	0.00	0.00	0.00	0.00	0.00	0.00	0.12	0.00	0.01	>0.999999999999999	0.0
Q99519	Sialidase-1 OS=Homo sapiens GN=NEU1 PE=1 SV=1	0.00	0.00	0.00	0.00	0.00	0.00	0.00	0.00	0.00	0.00	0.00	0.00	0.00	0.00	0.00	0.00	0.00	0.00	0.00	0.00	0.00	0.00	0.00	0.28	0.00	0.02	>0.999999999999999	0.0
P45985	Dual specificity mitogen-activated protein kinase kinase 4 OS=Homo sapiens GN=MAP2K4 PE=1 SV=1	0.00	0.00	0.00	0.00	0.00	0.00	0.00	0.00	0.00	0.00	0.00	0.00	0.00	0.00	0.00	0.00	0.00	0.00	0.00	0.00	0.00	0.00	0.00	0.28	0.00	0.02	>0.999999999999999	0.0
Q99959	Plakophilin-2 OS=Homo sapiens GN=PKP2 PE=1 SV=2	0.00	0.00	0.00	0.00	0.00	0.00	0.00	0.00	0.00	0.00	0.00	0.00	0.00	0.00	0.00	0.00	0.00	0.00	0.00	0.00	0.00	0.00	0.00	0.12	0.00	0.01	>0.999999999999999	0.0
O14545	TRAF-type zinc finger domain-containing protein 1 OS=Homo sapiens GN=TRAFD1 PE=1 SV=1	0.00	0.00	0.00	0.00	0.00	0.00	0.00	0.00	0.00	0.00	0.00	0.00	0.00	0.00	0.00	0.00	0.00	0.00	0.00	0.00	0.00	0.00	0.00	0.20	0.00	0.02	>0.999999999999999	0.0
Q9NUQ6	SPATS2-like protein OS=Homo sapiens GN=SPATS2L PE=1 SV=2	0.00	0.00	0.00	0.00	0.00	0.00	0.00	0.00	0.00	0.00	0.00	0.00	0.00	0.00	0.00	0.00	0.00	0.00	0.00	0.00	0.00	0.00	0.00	0.20	0.00	0.02	>0.999999999999999	0.0
Q9BPW9	Dehydrogenase/reductase SDR family member 9 OS=Homo sapiens GN=DHRS9 PE=1 SV=1	0.00	0.00	0.00	0.00	0.00	0.00	0.00	0.00	0.00	0.00	0.00	0.00	0.00	0.00	0.00	0.00	0.00	0.00	0.00	0.00	0.00	0.00	0.00	0.36	0.00	0.03	>0.999999999999999	0.0
Q00534	Cyclin-dependent kinase 6 OS=Homo sapiens GN=CDK6 PE=1 SV=1	0.00	0.00	0.00	0.00	0.00	0.00	0.00	0.00	0.00	0.00	0.00	0.00	0.00	0.00	0.00	0.00	0.00	0.00	0.00	0.00	0.00	0.00	0.00	0.36	0.00	0.03	>0.999999999999999	0.0
P21926	CD9 antigen OS=Homo sapiens GN=CD9 PE=1 SV=4	0.00	0.00	0.00	0.00	0.00	0.00	0.00	0.00	0.00	0.00	0.00	0.00	0.00	0.00	0.00	0.00	0.00	0.00	0.00	0.00	0.00	0.00	0.00	0.52	0.00	0.04	>0.999999999999999	0.0
Q309B1	Tripartite motif-containing protein 16-like protein OS=Homo sapiens GN=TRIM16L PE=2 SV=3	0.00	0.00	0.00	0.00	0.00	0.00	0.00	0.00	0.00	0.00	0.00	0.00	0.00	0.00	0.00	0.00	0.00	0.00	0.00	0.00	0.00	0.00	0.00	0.32	0.00	0.03	>0.999999999999999	0.0
Q9Y3Q8	TSC22 domain family protein 4 OS=Homo sapiens GN=TSC22D4 PE=1 SV=2	0.00	0.00	0.00	0.00	0.00	0.00	0.00	0.00	0.00	0.00	0.00	0.00	0.00	0.00	0.00	0.00	0.00	0.00	0.00	0.00	0.00	0.00	0.00	0.32	0.00	0.03	>0.999999999999999	0.0
Q6UX73	UPF0764 protein C16orf89 OS=Homo sapiens GN=C16orf89 PE=2 SV=2	0.00	0.00	0.00	0.00	0.00	0.00	0.00	0.00	0.00	0.00	0.00	0.00	0.00	0.00	0.00	0.00	0.00	0.00	0.00	0.00	0.00	0.00	0.00	0.28	0.00	0.02	>0.999999999999999	0.0
Q5SGD2	Protein phosphatase 1L OS=Homo sapiens GN=PPM1L PE=1 SV=1	0.00	0.00	0.00	0.00	0.00	0.00	0.00	0.00	0.00	0.00	0.00	0.00	0.00	0.00	0.00	0.00	0.00	0.00	0.00	0.00	0.00	0.00	0.00	0.32	0.00	0.03	>0.999999999999999	0.0
P55145	Mesencephalic astrocyte-derived neurotrophic factor OS=Homo sapiens GN=MANF PE=1 SV=3	0.00	0.00	0.00	0.00	0.00	0.00	0.00	0.00	0.00	0.00	0.00	0.00	0.00	0.00	0.00	0.00	0.00	0.00	0.00	0.00	0.00	0.00	0.00	0.64	0.00	0.05	>0.999999999999999	0.0
Q9BQ04	RNA-binding protein 4B OS=Homo sapiens GN=RBM4B PE=1 SV=1	0.00	0.00	0.00	0.00	0.00	0.00	0.00	0.00	0.00	0.00	0.00	0.00	0.00	0.00	0.00	0.00	0.00	0.00	0.00	0.00	0.00	0.00	0.00	0.32	0.00	0.03	>0.999999999999999	0.0
O95477	ATP-binding cassette sub-family A member 1 OS=Homo sapiens GN=ABCA1 PE=1 SV=3	0.00	0.00	0.00	0.00	0.00	0.00	0.00	0.00	0.00	0.00	0.00	0.00	0.00	0.00	0.00	0.00	0.00	0.00	0.00	0.00	0.00	0.00	0.00	0.04	0.00	0.00	>0.999999999999999	0.0
P62487	DNA-directed RNA polymerase II subunit RPB7 OS=Homo sapiens GN=POLR2G PE=1 SV=1	0.00	0.00	0.00	0.00	0.00	0.00	0.00	0.00	0.00	0.00	0.00	0.00	0.00	0.00	0.00	0.00	0.00	0.00	0.00	0.00	0.00	0.00	0.00	0.68	0.00	0.06	>0.999999999999999	0.0
O43493	Trans-Golgi network integral membrane protein 2 OS=Homo sapiens GN=TGOLN2 PE=1 SV=2	0.00	0.00	0.00	0.00	0.00	0.00	0.00	0.00	0.00	0.00	0.00	0.00	0.00	0.00	0.00	0.00	0.00	0.00	0.00	0.00	0.00	0.00	0.00	0.24	0.00	0.02	>0.999999999999999	0.0
A2IDD5	Coiled-coil domain-containing protein 78 OS=Homo sapiens GN=CCDC78 PE=2 SV=1	0.00	0.00	0.00	0.00	0.00	0.00	0.00	0.00	0.00	0.00	0.00	0.00	0.00	0.00	0.00	0.00	0.00	0.00	0.00	0.00	0.00	0.00	0.00	0.28	0.00	0.02	>0.999999999999999	0.0

Q12884	Seprase OS=Homo sapiens GN=FAP PE=1 SV=5	0.00	0.00	0.00	0.00	0.00	0.00	0.00	0.00	0.00	0.00	0.00	0.00	0.00	0.00	0.00	0.00	0.00	0.00	0.00	0.00	0.00	0.00	0.00	0.16	0.00	0.01	>0.999999999999999	0.0
O14672	Disintegrin and metalloproteinase domain-containing protein 10 OS=Homo sapiens GN=ADAM10 PE=1 SV=1	0.00	0.00	0.00	0.00	0.00	0.00	0.00	0.00	0.00	0.00	0.00	0.00	0.00	0.00	0.00	0.00	0.00	0.00	0.00	0.00	0.00	0.00	0.00	0.16	0.00	0.01	>0.999999999999999	0.0
Q9NR19	Acetyl-coenzyme A synthetase, cytoplasmic OS=Homo sapiens GN=ACSS2 PE=1 SV=1	0.00	0.00	0.00	0.00	0.00	0.00	0.00	0.00	0.00	0.00	0.00	0.00	0.00	0.00	0.00	0.00	0.00	0.00	0.00	0.00	0.00	0.00	0.00	0.16	0.00	0.01	>0.999999999999999	0.0
O60513	Beta-1,4-galactosyltransferase 4 OS=Homo sapiens GN=B4GALT4 PE=1 SV=1	0.00	0.00	0.00	0.00	0.00	0.00	0.00	0.00	0.00	0.00	0.00	0.00	0.00	0.00	0.00	0.00	0.00	0.00	0.00	0.00	0.00	0.00	0.00	0.32	0.00	0.03	>0.999999999999999	0.0
Q49MG5	Microtubule-associated protein 9 OS=Homo sapiens GN=MAP9 PE=1 SV=3	0.00	0.00	0.00	0.00	0.00	0.00	0.00	0.00	0.00	0.00	0.00	0.00	0.00	0.00	0.00	0.00	0.00	0.00	0.00	0.00	0.00	0.00	0.00	0.16	0.00	0.01	>0.999999999999999	0.0
P19438	Tumor necrosis factor receptor superfamily member 1A OS=Homo sapiens GN=TNFRSF1A PE=1 SV=1	0.00	0.00	0.00	0.00	0.00	0.00	0.00	0.00	0.00	0.00	0.00	0.00	0.00	0.00	0.00	0.00	0.00	0.00	0.00	0.00	0.00	0.00	0.00	0.24	0.00	0.02	>0.999999999999999	0.0
P15882	N-chimaerin OS=Homo sapiens GN=CHN1 PE=1 SV=3	0.00	0.00	0.00	0.00	0.00	0.00	0.00	0.00	0.00	0.00	0.00	0.00	0.00	0.00	0.00	0.00	0.00	0.00	0.00	0.00	0.00	0.00	0.00	0.24	0.00	0.02	>0.999999999999999	0.0
Q96PC3	AP-1 complex subunit sigma-3 OS=Homo sapiens GN=AP1S3 PE=2 SV=1	0.00	0.00	0.00	0.00	0.00	0.00	0.00	0.00	0.00	0.00	0.00	0.00	0.00	0.00	0.00	0.00	0.00	0.00	0.00	0.00	0.00	0.00	0.00	0.80	0.00	0.07	>0.999999999999999	0.0
P10915	Hyaluronan and proteoglycan link protein 1 OS=Homo sapiens GN=HAPLN1 PE=2 SV=2	0.00	0.00	0.00	0.00	0.00	0.00	0.00	0.00	0.00	0.00	0.00	0.00	0.00	0.00	0.00	0.00	0.00	0.00	0.00	0.00	0.00	0.00	0.00	0.32	0.00	0.03	>0.999999999999999	0.0
O95171	Sciellin OS=Homo sapiens GN=SCEL PE=1 SV=2	0.00	0.00	0.00	0.00	0.00	0.00	0.00	0.00	0.00	0.00	0.00	0.00	0.00	0.00	0.00	0.00	0.00	0.00	0.00	0.00	0.00	0.00	0.00	0.16	0.00	0.01	>0.999999999999999	0.0
Q9UKF5	Disintegrin and metalloproteinase domain-containing protein 29 OS=Homo sapiens GN=ADAM29 PE=1 SV=3	0.00	0.00	0.00	0.00	0.00	0.00	0.00	0.00	0.00	0.00	0.00	0.00	0.00	0.00	0.00	0.00	0.00	0.00	0.00	0.00	0.00	0.00	0.00	0.12	0.00	0.01	>0.999999999999999	0.0
Q6P499	NIPA-like protein 3 OS=Homo sapiens GN=NIPAL3 PE=1 SV=1	0.00	0.00	0.00	0.00	0.00	0.00	0.00	0.00	0.00	0.00	0.00	0.00	0.00	0.00	0.00	0.00	0.00	0.00	0.00	0.00	0.00	0.00	0.00	0.28	0.00	0.02	>0.999999999999999	0.0
O00560	Syntenin-1 OS=Homo sapiens GN=SDCBP PE=1 SV=1	0.00	0.00	0.00	0.00	0.00	0.00	0.00	0.00	0.00	0.00	0.00	0.00	0.00	0.00	0.00	0.00	0.00	0.00	0.00	0.00	0.00	0.00	0.00	0.40	0.00	0.03	>0.999999999999999	0.0
Q9NWK9	Box C/D snoRNA protein 1 OS=Homo sapiens GN=ZNHIT6 PE=1 SV=1	0.00	0.00	0.00	0.00	0.00	0.00	0.00	0.00	0.00	0.00	0.00	0.00	0.00	0.00	0.00	0.00	0.00	0.00	0.00	0.00	0.00	0.00	0.00	0.24	0.00	0.02	>0.999999999999999	0.0

Table S3 OC and ANC proteome from elderly subjects

emPAI/1 uL mucus		OC												ANC												Average		Ajusted *P* value	Fold difference
Accession	Description	E1	E2	E3	E4	E5	E6	E7	E8	E9	E10	E11	E12	E1	E2	E3	E4	E5	E6	E7	E8	E9	E10	E11	E12	OC	ANC	Multiple *t*-test	OC/ANC
P68871	Hemoglobin subunit beta OS=Homo sapiens GN=HBB PE=1 SV=2	0.00	253.54	0.00	109.03	388.94	16.74	12.99	3.33	11.14	173.03	383.99	165.15	18.86	92.46	0.00	0.00	40.53	0.00	12.21	0.00	299.32	33.25	207.36	32.70	126.49	61.39	<0.000000000000001	2.1
P69905	Hemoglobin subunit alpha OS=Homo sapiens GN=HBA1 PE=1 SV=2	5.37	70.53	0.00	35.33	239.92	7.13	8.27	3.49	3.25	24.32	550.99	235.48	10.79	101.93	0.00	0.00	15.46	0.00	3.49	0.00	72.80	10.18	123.47	17.37	98.67	29.62	<0.000000000000001	3.3
P01834	Ig kappa chain C region OS=Homo sapiens GN=IGKC PE=1 SV=1	505.08	622.79	176.81	181.67	396.61	174.37	266.19	189.91	107.14	125.03	391.56	157.18	237.04	136.58	75.32	186.36	389.87	183.74	250.15	267.93	36.89	248.94	317.18	51.38	274.53	198.45	<0.000000000000001	1.4
P05109	Protein S100-A8 OS=Homo sapiens GN=S100A8 PE=1 SV=1	143.86	455.22	47.40	299.50	81.12	85.73	99.36	93.37	23.36	1121.69	152.80	58.67	51.67	297.13	22.33	20.48	79.75	123.77	31.86	76.31	9.15	2233.38	403.83	45.82	221.84	282.96	<0.000000000000001	0.8
P35326	Small proline-rich protein 2A OS=Homo sapiens GN=SPRR2A PE=2 SV=1	0.00	0.00	0.00	0.00	0.00	0.00	0.00	0.00	0.00	10.33	0.00	0.00	25.88	59.43	0.00	0.00	0.00	27.61	28.53	38.22	0.00	61.30	45.25	0.00	0.86	23.85	0.002353285	0.0
P60174	Triosephosphate isomerase OS=Homo sapiens GN=TPI1 PE=1 SV=3	113.08	171.72	66.00	61.87	33.75	85.73	68.83	64.68	18.36	10.85	77.25	66.04	40.62	72.32	22.89	28.14	38.84	48.31	56.91	52.86	11.06	96.21	80.21	29.69	69.85	48.17	0.005500171	1.4
P04083	Annexin A1 OS=Homo sapiens GN=ANXA1 PE=1 SV=2	73.27	69.15	33.61	21.99	31.84	39.15	45.38	33.77	14.71	38.72	50.93	29.88	18.69	29.21	12.00	8.69	23.75	22.24	47.56	16.32	9.24	16.00	11.81	7.15	40.20	18.56	0.005500171	2.2
Q71U36	Tubulin alpha-1A chain OS=Homo sapiens GN=TUBA1A PE=1 SV=1	56.17	39.87	28.12	0.00	33.18	55.46	32.22	0.00	8.33	0.00	51.88	25.00	16.76	0.00	15.66	0.00	0.00	0.00	0.00	29.80	0.00	0.00	0.00	8.91	27.52	5.93	0.005500171	4.6
Q13938	Calcyphosin OS=Homo sapiens GN=CAPS PE=1 SV=1	82.55	64.08	31.15	42.16	64.82	40.46	38.12	44.07	13.40	14.92	64.00	40.64	24.39	28.81	25.11	12.48	42.61	21.93	2.54	43.79	6.96	37.61	27.76	10.28	45.03	23.69	0.006139041	1.9
P02042	Hemoglobin subunit delta OS=Homo sapiens GN=HBD PE=1 SV=2	5.13	104.18	0.00	42.16	108.93	0.00	0.00	0.00	0.00	29.01	107.55	70.42	0.00	56.00	0.00	1.45	0.00	3.22	0.00	0.00	40.87	0.00	108.43	12.04	38.95	18.50	0.012229126	2.1
P06702	Protein S100-A9 OS=Homo sapiens GN=S100A9 PE=1 SV=1	36.39	685.71	10.98	103.27	56.38	21.69	66.63	15.54	15.66	626.18	214.53	9.76	45.79	42.91	10.89	4.00	98.41	22.85	15.54	27.60	6.56	988.07	359.97	5.56	155.23	135.68	0.02397551	1.1
P30044	Peroxiredoxin-5, mitochondrial OS=Homo sapiens GN=PRDX5 PE=1 SV=4	74.49	58.08	48.85	31.24	70.19	63.46	51.45	39.95	12.09	10.54	69.30	30.38	14.56	50.76	28.00	9.10	31.30	38.65	5.07	32.65	5.11	52.36	38.65	20.78	46.67	27.25	0.02446064	1.7
P62805	Histone H4 OS=Homo sapiens GN=HIST1H4A PE=1 SV=2	0.00	124.93	0.00	4.55	0.00	10.19	11.81	0.00	1.19	103.63	23.10	0.00	0.00	14.10	7.78	2.07	5.66	0.00	11.10	0.00	0.92	6.23	4.60	0.00	23.28	4.37	0.034265153	5.3
P00352	Retinal dehydrogenase 1 OS=Homo sapiens GN=ALDH1A1 PE=1 SV=2	159.00	79.98	59.61	75.37	77.10	77.43	54.32	63.73	31.44	12.94	94.10	69.33	43.60	122.68	41.55	27.73	44.30	61.66	23.94	84.34	14.26	77.72	61.66	34.57	71.20	53.17	0.059946814	1.3
P05387	60S acidic ribosomal protein P2 OS=Homo sapiens GN=RPLP2 PE=1 SV=1	124.81	60.85	41.68	27.45	50.63	74.38	62.75	28.69	20.26	27.55	34.27	50.90	23.16	36.46	29.33	12.48	49.77	40.49	41.85	48.20	11.46	37.61	27.76	29.01	50.35	32.30	0.059946814	1.6
P06703	Protein S100-A6 OS=Homo sapiens GN=S100A6 PE=1 SV=1	80.84	32.27	37.09	21.23	42.58	48.18	37.45	35.19	8.80	23.17	42.03	32.97	19.48	44.72	15.55	9.66	26.39	12.12	12.52	28.76	6.84	29.09	12.12	18.79	36.82	19.67	0.109976959	1.9
P02788	Lactotransferrin OS=Homo sapiens GN=LTF PE=1 SV=6	47.87	91.74	30.92	41.85	56.00	32.02	90.08	93.05	15.03	24.42	55.29	34.07	17.19	28.61	18.00	15.17	71.45	47.24	69.75	42.11	8.99	43.22	33.74	16.58	51.03	34.34	0.144767414	1.5
P31949	Protein S100-A11 OS=Homo sapiens GN=S100A11 PE=1 SV=2	87.68	82.75	40.22	54.44	33.75	25.62	29.69	56.91	14.24	18.37	67.97	25.50	10.00	51.57	28.44	12.14	33.18	39.26	18.07	33.17	5.42	53.20	39.26	14.53	44.76	28.19	0.148704637	1.6
P02768	Serum albumin OS=Homo sapiens GN=ALB PE=1 SV=2	392.49	251.47	250.99	255.68	624.25	158.80	549.09	164.55	84.74	159.88	404.63	184.68	155.10	391.61	248.85	75.25	509.40	203.38	515.99	148.48	75.79	535.90	327.76	105.26	290.10	274.40	0.26016987	1.1
P40394	Alcohol dehydrogenase class 4 mu/sigma chain OS=Homo sapiens GN=ADH7 PE=1 SV=2	45.67	38.26	32.16	22.29	24.74	37.70	28.00	23.30	10.27	1.67	35.41	34.86	14.56	25.99	12.44	10.14	10.75	8.74	5.55	19.05	3.45	20.16	14.88	6.36	27.86	12.67	0.351650504	2.2
P35321	Cornifin-A OS=Homo sapiens GN=SPRR1A PE=1 SV=2	0.00	0.00	0.00	11.83	0.00	0.00	23.28	12.36	0.00	0.00	0.00	0.00	0.00	94.08	15.33	45.38	0.00	21.17	0.00	17.88	6.71	16.21	5.21	4.43	3.96	18.87	0.402807042	0.2
P80723	Brain acid soluble protein 1 OS=Homo sapiens GN=BASP1 PE=1 SV=2	49.34	22.82	8.29	15.01	24.74	18.78	27.50	39.00	0.59	13.46	30.86	9.86	11.32	14.91	1.67	1.03	6.03	7.82	2.38	1.94	0.46	20.57	25.00	0.00	21.69	7.76	0.722973693	2.8
P68371	Tubulin beta-4B chain OS=Homo sapiens GN=TUBB4B PE=1 SV=1	52.27	36.65	16.02	26.69	30.49	34.06	12.99	16.01	6.30	0.00	36.73	15.84	7.81	22.96	6.22	5.31	10.56	11.81	7.29	11.53	2.74	16.00	10.12	5.73	23.67	9.84	0.733972734	2.4
P31946	14-3-3 protein beta/alpha OS=Homo sapiens GN=YWHAB PE=1 SV=3	68.87	65.00	36.64	36.70	54.08	35.22	63.76	51.84	14.99	18.05	82.18	43.23	24.74	56.81	36.33	14.21	32.61	26.53	38.36	48.97	10.07	58.60	43.25	16.01	47.55	33.87	0.775532913	1.4
Q9BW30	Tubulin polymerization-promoting protein family member 3 OS=Homo sapiens GN=TPPP3 P	40.30	48.86	29.92	32.15	40.66	24.02	35.76	19.82	8.41	9.60	62.86	16.44	18.60	33.24	13.89	6.35	39.97	19.17	0.00	16.20	2.83	25.97	25.31	5.22	30.73	17.23	0.825103921	1.8
P0CG05	Ig lambda-2 chain C regions OS=Homo sapiens GN=IGLC2 PE=1 SV=1	94.03	88.74	59.61	58.38	102.03	77.43	89.74	43.28	15.27	40.18	100.73	38.35	63.34	107.17	59.10	26.55	182.68	59.05	114.45	68.93	5.76	150.03	81.59	21.86	67.31	78.38	>0.999999999999999	0.9
P29508	Serpin B3 OS=Homo sapiens GN=SERPINB3 PE=1 SV=2	84.99	104.87	38.99	48.07	32.99	66.23	58.70	33.77	70.07	10.85	120.80	129.79	16.84	91.66	26.33	28.76	44.68	69.79	37.57	82.66	34.40	132.57	63.96	79.88	66.68	59.09	>0.999999999999999	1.1
P12273	Prolactin-inducible protein OS=Homo sapiens GN=PIP PE=1 SV=1	102.09	76.52	58.49	63.39	100.11	60.84	88.06	66.26	10.27	20.77	79.15	33.07	36.67	84.20	46.44	28.83	78.80	80.06	31.55	43.01	10.22	86.86	80.06	18.85	63.25	52.13	>0.999999999999999	1.2
Q06830	Peroxiredoxin-1 OS=Homo sapiens GN=PRDX1 PE=1 SV=1	74.49	84.36	94.01	66.12	83.62	75.25	51.45	39.95	20.50	21.50	82.55	36.46	45.35	104.15	79.54	25.24	57.50	38.65	20.93	66.98	22.05	90.60	66.87	29.35	60.85	53.94	>0.999999999999999	1.1
P02647	Apolipoprotein A-I OS=Homo sapiens GN=APOA1 PE=1 SV=1	51.05	35.03	35.97	91.44	79.97	53.42	79.79	28.38	18.76	29.22	89.56	47.12	28.16	48.95	66.99	28.76	78.62	27.45	95.59	27.08	14.57	111.17	63.96	10.16	53.31	50.12	>0.999999999999999	1.1
P63104	14-3-3 protein zeta/delta OS=Homo sapiens GN=YWHAZ PE=1 SV=1	60.08	88.97	57.14	43.67	40.08	56.18	74.90	52.95	13.24	25.67	73.09	44.23	29.30	67.28	42.88	19.86	62.97	37.73	45.65	43.27	10.29	69.40	51.23	18.96	52.52	41.57	>0.999999999999999	1.3
P23528	Cofilin-1 OS=Homo sapiens GN=CFL1 PE=1 SV=3	53.24	97.27	30.81	63.99	65.59	61.42	46.39	20.29	10.90	28.70	79.90	42.04	24.12	43.92	37.99	18.97	97.28	52.45	34.56	35.63	10.53	71.07	52.45	19.42	50.05	41.53	>0.999999999999999	1.2
P07737	Profilin-1 OS=Homo sapiens GN=PFN1 PE=1 SV=2	57.40	71.45	34.73	47.01	59.45	45.12	39.64	49.14	12.29	53.64	44.50	23.41	27.20	62.45	34.44	21.38	58.44	26.69	49.14	22.54	7.24	64.42	47.55	13.34	44.82	36.24	>0.999999999999999	1.2
P60709	Actin, cytoplasmic 1 OS=Homo sapiens GN=ACTB PE=1 SV=1	57.40	38.49	29.36	59.14	50.25	46.72	48.92	50.89	12.73	48.95	49.61	26.10	22.98	47.34	18.55	24.42	49.39	32.36	33.45	21.64	8.07	81.04	40.18	14.87	43.21	32.86	>0.999999999999999	1.3
P14618	Pyruvate kinase isozymes M1/M2 OS=Homo sapiens GN=PKM2 PE=1 SV=4	82.55	49.79	43.59	35.33	38.16	60.55	39.30	34.24	14.39	20.77	47.71	41.44	20.44	63.46	32.55	30.69	37.52	35.74	29.01	53.90	8.38	70.23	51.84	13.23	42.32	37.25	>0.999999999999999	1.1
P01591	Immunoglobulin J chain OS=Homo sapiens GN=IGJ PE=1 SV=4	54.22	51.17	39.22	42.61	42.58	40.90	47.40	44.54	11.14	29.33	81.80	17.23	24.65	70.51	47.99	15.31	81.44	53.68	35.19	36.41	8.65	46.13	43.10	15.95	41.84	39.92	>0.999999999999999	1.0
P01876	Ig alpha-1 chain C region OS=Homo sapiens GN=IGHA1 PE=1 SV=2	41.28	63.15	34.29	41.55	65.40	24.60	57.52	48.51	9.68	43.83	41.09	19.13	19.04	76.35	30.44	21.11	87.66	37.42	60.08	31.61	6.68	50.70	42.02	10.90	40.83	39.50	>0.999999999999999	1.0
P09211	Glutathione S-transferase P OS=Homo sapiens GN=GSTP1 PE=1 SV=2	37.61	53.24	37.20	35.03	36.44	57.20	46.90	30.12	18.36	19.83	74.41	39.15	13.51	46.53	36.88	15.93	52.41	35.43	14.90	29.93	8.56	68.99	42.64	18.85	40.46	32.05	>0.999999999999999	1.3
P04075	Fructose-bisphosphate aldolase A OS=Homo sapiens GN=ALDOA PE=1 SV=2	70.34	47.25	32.27	43.67	44.30	51.67	38.97	40.90	17.21	14.61	38.82	31.88	14.04	64.46	32.00	19.86	38.65	35.43	32.50	41.46	10.93	73.77	35.43	16.35	39.32	34.57	>0.999999999999999	1.1
P00326	Alcohol dehydrogenase 1C OS=Homo sapiens GN=ADH1C PE=1 SV=2	67.65	70.99	27.90	23.51	29.73	54.73	22.94	27.90	13.52	3.86	78.58	41.34	15.44	68.69	19.55	15.31	33.18	30.37	0.00	35.89	6.10	70.86	30.37	14.14	38.55	28.32	>0.999999999999999	1.4
P01857	Ig gamma-1 chain C region OS=Homo sapiens GN=IGHG1 PE=1 SV=1	44.70	42.18	33.61	45.49	89.18	30.28	35.09	25.21	7.26	24.63	50.37	13.75	26.32	41.90	41.66	20.69	70.70	36.20	53.26	20.60	7.27	69.82	36.20	13.40	36.81	36.50	>0.999999999999999	1.0
P37802	Transgelin-2 OS=Homo sapiens GN=TAGLN2 PE=1 SV=3	87.93	37.80	33.61	37.61	47.56	43.67	27.67	32.18	16.97	10.44	31.05	29.88	26.32	33.04	18.22	14.00	24.51	19.94	20.61	55.45	6.25	51.53	25.15	9.31	36.36	25.36	>0.999999999999999	1.4
P02652	Apolipoprotein A-II OS=Homo sapiens GN=APOA2 PE=1 SV=1	46.16	43.56	43.92	42.01	75.18	27.51	31.88	19.34	15.54	7.31	52.45	18.83	24.30	55.80	30.77	19.10	52.22	28.99	43.91	35.89	8.53	57.56	28.99	15.73	35.31	33.48	>0.999999999999999	1.1
P62258	14-3-3 protein epsilon OS=Homo sapiens GN=YWHAE PE=1 SV=1	33.22	60.85	25.43	53.23	31.26	38.43	32.56	48.35	10.47	7.41	57.75	22.61	19.91	38.88	21.44	15.66	25.64	20.86	21.56	39.52	5.02	47.17	25.00	7.72	35.13	24.03	>0.999999999999999	1.5
O00299	Chloride intracellular channel protein 1 OS=Homo sapiens GN=CLIC1 PE=1 SV=4	44.70	50.25	39.22	38.97	29.15	50.94	25.64	29.01	10.19	13.05	48.66	34.86	19.12	60.64	24.22	20.76	28.66	23.31	12.36	52.47	9.27	84.16	39.42	14.59	34.55	32.41	>0.999999999999999	1.1
P02787	Serotransferrin OS=Homo sapiens GN=TF PE=1 SV=3	35.17	26.74	35.07	50.19	45.64	20.96	58.70	31.55	9.99	24.84	37.68	26.50	24.65	33.24	36.77	12.14	62.40	25.31	52.47	22.80	5.08	58.39	28.68	8.18	33.59	30.84	>0.999999999999999	1.1
P61626	Lysozyme C OS=Homo sapiens GN=LYZ PE=1 SV=1	36.15	34.11	46.28	38.97	62.90	21.54	55.33	52.00	13.01	7.51	20.26	14.74	22.55	39.69	21.89	22.62	48.45	22.70	31.23	25.52	7.91	53.40	30.21	14.59	33.57	28.40	>0.999999999999999	1.2
P00441	Superoxide dismutase [Cu-Zn] OS=Homo sapiens GN=SOD1 PE=1 SV=2	87.19	26.28	17.70	23.96	40.66	65.79	12.99	33.61	8.41	8.04	29.92	45.02	31.32	56.00	17.55	14.62	14.52	32.52	3.33	27.47	6.53	44.05	24.23	4.37	33.30	23.04	>0.999999999999999	1.4
P06733	Alpha-enolase OS=Homo sapiens GN=ENO1 PE=1 SV=2	52.02	49.09	28.91	35.64	37.01	41.19	29.35	24.73	12.29	18.16	36.54	28.19	22.63	62.45	28.66	17.79	44.30	36.04	24.73	36.67	7.24	58.81	36.04	16.07	32.76	32.62	>0.999999999999999	1.0
Q01469	Fatty acid-binding protein, epidermal OS=Homo sapiens GN=FABP5 PE=1 SV=3	55.44	38.95	33.50	34.42	23.01	33.04	38.29	47.40	9.00	17.64	32.00	29.78	10.53	45.73	25.22	15.66	42.80	45.86	12.84	29.41	6.99	35.12	34.82	21.91	32.71	27.24	>0.999999999999999	1.2
P01040	Cystatin-A OS=Homo sapiens GN=CSTA PE=1 SV=1	48.36	45.64	22.19	44.28	24.36	28.82	21.42	31.39	16.46	20.66	55.29	29.09	17.37	39.89	46.10	20.14	55.05	30.37	31.39	37.83	3.91	60.68	30.37	23.56	32.33	33.05	>0.999999999999999	1.0
P48594	Serpin B4 OS=Homo sapiens GN=SERPINB4 PE=1 SV=2	51.53	72.14	19.05	32.00	14.58	34.06	35.59	16.33	27.00	5.53	39.95	37.35	9.04	42.51	11.44	16.14	28.47	26.07	26.95	36.80	9.64	48.62	23.16	25.43	32.09	25.36	>0.999999999999999	1.3
P30838	Aldehyde dehydrogenase, dimeric NADP-preferring OS=Homo sapiens GN=ALDH3A1 PE=1	79.38	63.39	15.80	32.00	36.82	36.68	19.06	27.58	11.86	5.74	24.05	19.13	20.26	42.51	17.44	20.62	26.58	35.43	15.85	38.74	5.36	32.62	19.48	10.90	30.96	23.82	>0.999999999999999	1.3
P01024	Complement C3 OS=Homo sapiens GN=C3 PE=1 SV=2	34.19	38.26	24.76	53.38	64.63	22.85	37.28	13.47	6.38	21.39	37.87	16.93	21.41	50.36	34.99	18.07	58.07	26.07	37.73	17.10	5.85	64.00	26.84	10.22	30.95	30.89	>0.999999999999999	1.0
P27348	14-3-3 protein theta OS=Homo sapiens GN=YWHAQ PE=1 SV=1	35.41	56.01	19.50	26.39	39.70	30.13	29.35	27.58	9.64	15.13	53.77	24.21	12.72	41.70	19.33	12.00	14.14	14.72	22.99	31.48	7.48	59.01	22.24	8.23	30.57	22.17	>0.999999999999999	1.4
P08727	Keratin, type I cytoskeletal 19 OS=Homo sapiens GN=KRT19 PE=1 SV=4	33.70	45.18	33.05	18.35	23.21	31.73	17.88	24.73	12.85	57.92	50.55	17.43	13.69	31.43	32.77	12.07	14.71	33.44	34.56	20.21	4.80	16.21	16.26	11.13	30.55	20.11	>0.999999999999999	1.5
P03973	Antileukoproteinase OS=Homo sapiens GN=SLPI PE=1 SV=2	20.27	54.17	9.30	26.39	23.78	18.05	86.71	27.58	6.90	8.66	58.70	23.41	27.20	35.05	13.78	12.00	58.44	19.02	19.66	10.75	7.24	36.16	26.69	7.04	30.33	22.75	>0.999999999999999	1.3
Q9NP55	BPI fold-containing family A member 1 OS=Homo sapiens GN=BPIFA1 PE=1 SV=1	37.86	70.76	29.36	28.36	24.36	32.31	26.15	29.64	8.80	23.17	24.05	26.10	8.95	20.55	17.22	7.04	29.22	19.48	29.64	20.08	6.84	21.20	15.64	10.62	30.08	17.21	>0.999999999999999	1.7
P31947	14-3-3 protein sigma OS=Homo sapiens GN=SFN PE=1 SV=1	35.90	56.70	32.27	37.30	28.19	25.62	41.50	23.30	8.29	10.12	39.57	20.82	10.61	29.61	27.33	19.86	27.71	22.55	39.00	15.68	6.44	59.84	26.99	6.87	29.97	24.37	>0.999999999999999	1.2
P30086	Phosphatidylethanolamine-binding protein 1 OS=Homo sapiens GN=PEBP1 PE=1 SV=3	67.17	41.49	25.10	33.97	34.52	32.60	18.39	22.51	7.14	5.74	34.08	33.37	19.65	45.12	12.11	23.11	10.37	27.61	8.72	23.32	5.54	22.65	12.27	19.02	29.67	19.12	>0.999999999999999	1.6
Q8TDL5	BPI fold-containing family B member 1 OS=Homo sapiens GN=BPIFB1 PE=2 SV=1	44.21	37.80	36.98	35.94	25.70	15.57	27.67	34.40	13.09	31.73	22.72	27.89	28.95	10.68	26.33	8.28	37.33	22.85	34.40	15.55	11.86	41.14	12.88	14.65	29.47	22.07	>0.999999999999999	1.3
P01833	Polymeric immunoglobulin receptor OS=Homo sapiens GN=PIGR PE=1 SV=4	38.83	49.09	20.17	32.30	36.44	31.00	32.05	37.73	9.44	17.64	32.00	15.84	17.72	40.69	20.00	10.97	42.61	30.98	20.77	26.17	6.22	55.07	30.98	9.59	29.38	25.98	>0.999999999999999	1.1
P04406	Glyceraldehyde-3-phosphate dehydrogenase OS=Homo sapiens GN=GAPDH PE=1 SV=3	51.78	55.55	20.84	28.21	23.01	35.08	17.21	19.02	8.41	22.12	35.22	30.48	10.53	61.64	13.33	8.28	30.54	24.85	22.19	44.57	5.73	50.08	32.52	12.04	28.91	26.36	>0.999999999999999	1.1
P80188	Neutrophil gelatinase-associated lipocalin OS=Homo sapiens GN=LCN2 PE=1 SV=2	17.83	56.24	18.15	44.74	24.55	35.51	49.76	20.29	9.68	13.36	30.67	19.92	17.55	99.92	22.22	16.83	30.54	37.42	20.29	25.91	6.16	87.27	24.85	13.85	28.39	33.57	>0.999999999999999	0.8
P30041	Peroxiredoxin-6 OS=Homo sapiens GN=PRDX6 PE=1 SV=3	27.35	57.39	23.31	26.08	26.85	59.39	18.89	13.79	6.82	2.92	56.05	20.72	25.97	28.20	23.11	11.86	8.67	26.38	4.44	26.95	3.45	18.08	21.47	11.81	28.30	17.53	>0.999999999999999	1.6
P00738	Haptoglobin OS=Homo sapiens GN=HP PE=1 SV=1	31.75	29.96	18.49	41.70	39.12	18.92	55.84	18.23	6.54	13.57	31.24	24.90	17.90	37.07	14.44	8.97	47.13	19.94	52.47	16.84	6.28	42.39	31.29	10.45	27.52	25.43	>0.999999999999999	1.1
P04792	Heat shock protein beta-1 OS=Homo sapiens GN=HSPB1 PE=1 SV=2	48.60	36.88	27.12	36.70	38.16	23.29	26.99	20.13	7.89	16.70	30.30	15.94	11.14	32.23	38.88	16.69	30.16	24.54	15.54	25.78	6.13	41.35	15.03	13.74	27.39	22.60	>0.999999999999999	1.2
P02763	Alpha-1-acid glycoprotein 1 OS=Homo sapiens GN=ORM1 PE=1 SV=1	29.31	27.66	20.95	34.42	35.86	22.12	31.55	14.74	6.03	28.60	51.88	22.61	13.33	55.20	25.22	12.90	42.80	28.68	51.68	15.55	6.99	38.86	34.82	12.89	27.14	28.24	>0.999999999999999	1.0
P10599	Thioredoxin OS=Homo sapiens GN=TXN PE=1 SV=3	42.50	26.05	28.24	17.14	33.37	51.38	42.51	17.91	9.99	11.79	21.40	11.26	15.26	35.05	12.55	7.79	12.44	26.69	10.46	32.65	3.48	36.16	26.69	14.31	26.13	19.46	>0.999999999999999	1.3
P60981	Destrin OS=Homo sapiens GN=DSTN PE=1 SV=3	30.77	21.21	30.25	32.45	24.16	48.76	10.63	19.97	8.49	0.00	51.12	21.32	14.65	25.38	14.00	18.62	17.34	19.33	2.85	34.98	3.88	44.47	9.66	15.33	24.93	18.37	>0.999999999999999	1.4
P01009	Alpha-1-antitrypsin OS=Homo sapiens GN=SERPINA1 PE=1 SV=3	43.47	16.60	27.00	29.87	30.49	18.34	40.65	25.21	9.56	4.28	33.70	19.62	19.12	32.03	19.77	8.69	18.48	17.02	28.22	20.60	3.88	29.51	21.78	7.15	24.90	18.85	>0.999999999999999	1.3
P50395	Rab GDP dissociation inhibitor beta OS=Homo sapiens GN=GDI2 PE=1 SV=2	42.01	32.27	25.55	16.98	26.85	43.08	21.09	12.05	8.25	10.33	35.98	22.71	12.28	34.65	17.33	7.72	16.40	23.93	13.79	29.54	3.85	47.38	23.93	8.86	24.76	19.97	>0.999999999999999	1.2
P62937	Peptidyl-prolyl cis-trans isomerase A OS=Homo sapiens GN=PPIA PE=1 SV=2	32.73	40.80	19.83	20.32	18.60	25.76	38.63	15.38	7.02	18.47	33.51	22.81	20.09	35.66	25.44	4.62	33.37	14.88	10.62	17.36	8.93	36.78	20.55	13.00	24.49	20.11	>0.999999999999999	1.2
Q14508	WAP four-disulfide core domain protein 2 OS=Homo sapiens GN=WFDC2 PE=1 SV=2	22.47	45.64	22.19	30.03	26.66	39.30	23.45	31.39	7.85	20.66	17.42	5.48	12.19	28.00	15.44	13.66	37.33	30.37	14.58	25.65	4.28	19.12	30.37	11.24	24.38	20.19	>0.999999999999999	1.2
Q96DA0	Zymogen granule protein 16 homolog B OS=Homo sapiens GN=ZG16B PE=1 SV=3	31.26	29.50	27.34	30.33	46.80	18.63	27.33	20.29	7.93	16.91	13.82	19.92	14.21	32.63	18.00	8.83	46.00	19.63	25.68	16.58	6.16	33.66	19.63	13.85	24.17	21.24	>0.999999999999999	1.1
P61981	14-3-3 protein gamma OS=Homo sapiens GN=YWHAG PE=1 SV=2	49.58	46.79	22.75	21.69	22.44	24.89	24.12	14.90	6.78	9.81	27.08	17.03	8.25	18.94	10.44	6.48	17.72	17.94	11.73	15.16	4.40	49.46	21.93	6.64	23.99	15.76	>0.999999999999999	1.5
P08107	Heat shock 70 kDa protein 1A/1B OS=Homo sapiens GN=HSPA1A PE=1 SV=5	23.94	39.87	22.30	28.21	30.88	28.97	27.16	21.88	6.86	5.32	30.48	21.22	10.26	30.02	19.22	11.93	22.06	21.17	14.27	19.30	3.33	28.68	19.63	9.14	23.92	17.42	>0.999999999999999	1.4
P01877	Ig alpha-2 chain C region OS=Homo sapiens GN=IGHA2 PE=1 SV=3	0.00	35.73	19.94	20.32	38.74	14.26	38.63	45.97	7.06	23.90	25.37	13.35	10.09	46.13	19.77	13.93	48.64	17.64	36.30	23.06	4.77	32.21	20.55	6.53	23.61	23.30	>0.999999999999999	1.0
P04080	Cystatin-B OS=Homo sapiens GN=CSTB PE=1 SV=2	46.89	44.25	13.89	18.80	54.08	27.95	20.92	11.26	11.18	7.41	13.44	12.35	10.88	38.68	21.33	19.45	13.39	19.02	11.26	16.07	5.91	25.77	29.45	7.04	23.54	18.19	>0.999999999999999	1.3
Q99497	Protein DJ-1 OS=Homo sapiens GN=PARK7 PE=1 SV=2	37.37	35.27	27.45	17.74	11.32	22.27	41.33	30.91	6.07	1.77	28.97	19.42	17.11	17.32	13.00	8.07	6.79	17.94	5.71	15.16	1.82	40.52	17.94	6.64	23.32	14.00	>0.999999999999999	1.7
P14550	Alcohol dehydrogenase [NADP(+)] OS=Homo sapiens GN=AKR1A1 PE=1 SV=3	33.22	41.72	20.28	20.62	22.44	48.32	22.94	18.55	8.17	0.00	22.15	20.52	10.26	31.83	20.11	6.83	12.82	27.76	6.50	17.62	2.09	32.83	15.18	11.70	23.24	16.29	>0.999999999999999	1.4
P08238	Heat shock protein HSP 90-beta OS=Homo sapiens GN=HSP90AB1 PE=1 SV=4	42.01	35.03	20.50	23.05	19.18	31.59	19.57	21.08	9.64	6.16	30.67	17.13	9.47	24.98	12.89	7.45	11.12	19.02	8.40	28.11	4.10	25.77	13.04	6.59	22.97	14.24	>0.999999999999999	1.6
Q04917	14-3-3 protein eta OS=Homo sapiens GN=YWHAH PE=1 SV=4	34.93	26.97	16.02	21.69	22.44	34.64	28.85	18.55	5.67	12.21	32.38	17.03	10.26	28.81	8.22	8.07	13.95	8.59	14.90	7.26	3.60	49.46	21.93	4.20	22.61	14.94	>0.999999999999999	1.5
P01859	Ig gamma-2 chain C region OS=Homo sapiens GN=IGHG2 PE=1 SV=2	28.82	23.05	13.22	31.69	40.08	14.56	23.28	18.71	3.33	19.20	44.87	9.96	16.14	27.80	17.78	16.35	39.40	28.22	15.85	17.88	2.09	49.25	32.05	4.77	22.56	22.30	>0.999999999999999	1.0
O75368	SH3 domain-binding glutamic acid-rich-like protein OS=Homo sapiens GN=SH3BGRL PE=1	38.83	23.97	11.65	24.11	43.73	23.14	17.54	25.21	6.30	6.37	30.11	15.84	9.12	32.03	11.55	7.17	29.98	15.95	16.49	20.60	3.20	47.38	34.97	9.03	22.23	19.79	>0.999999999999999	1.1
P52566	Rho GDP-dissociation inhibitor 2 OS=Homo sapiens GN=ARHGDIB PE=1 SV=3	17.59	45.41	10.87	36.40	24.16	14.12	26.82	25.21	6.30	25.05	23.86	9.66	11.05	19.54	8.00	4.97	13.57	19.33	0.00	16.32	1.54	49.87	19.32	7.15	22.12	14.22	>0.999999999999999	1.6
Q9UL46	Proteasome activator complex subunit 2 OS=Homo sapiens GN=PSME2 PE=1 SV=4	36.64	34.57	28.24	8.80	23.59	36.68	12.99	9.19	11.66	2.71	34.08	21.32	8.68	24.78	16.66	10.35	18.66	8.90	9.19	23.32	3.79	44.47	18.86	14.31	21.71	16.83	>0.999999999999999	1.3
P07900	Heat shock protein HSP 90-alpha OS=Homo sapiens GN=HSP90AA1 PE=1 SV=5	33.95	36.19	18.71	18.35	23.21	29.11	14.00	20.61	8.84	4.80	33.70	13.85	9.21	22.76	12.55	6.76	13.20	16.10	8.24	23.06	4.00	32.62	10.74	7.38	21.28	13.89	>0.999999999999999	1.5
P01860	Ig gamma-3 chain C region OS=Homo sapiens GN=IGHG3 PE=1 SV=2	23.69	25.81	16.47	28.36	40.08	18.78	21.76	15.38	4.44	10.12	35.41	11.16	11.32	25.99	20.77	12.90	43.93	19.79	26.31	14.51	2.56	34.49	32.05	5.51	20.95	20.84	>0.999999999999999	1.0
P16050	Arachidonate 15-lipoxygenase OS=Homo sapiens GN=ALOX15 PE=1 SV=3	52.51	30.89	16.13	12.28	22.06	25.91	15.01	15.54	6.15	1.46	35.98	16.54	8.60	12.09	6.67	4.62	7.73	9.20	26.31	9.59	5.11	8.52	10.28	5.05	20.87	9.48	>0.999999999999999	2.2
P08263	Glutathione S-transferase A1 OS=Homo sapiens GN=GSTA1 PE=1 SV=3	40.54	31.35	22.52	30.48	46.03	19.80	14.17	13.32	6.58	4.59	0.00	20.02	14.56	21.76	15.11	9.38	15.84	16.56	2.06	13.99	3.33	22.44	16.56	9.42	20.78	13.42	>0.999999999999999	1.5
P00558	Phosphoglycerate kinase 1 OS=Homo sapiens GN=PGK1 PE=1 SV=3	32.73	27.20	15.01	20.32	22.63	37.55	15.01	10.15	7.53	10.75	32.19	16.93	11.76	30.42	11.44	9.24	19.42	18.10	12.05	24.62	4.65	27.84	23.16	7.61	20.67	16.69	>0.999999999999999	1.2
P06727	Apolipoprotein A-IV OS=Homo sapiens GN=APOA4 PE=1 SV=3	15.39	14.52	25.77	31.39	28.58	7.57	31.55	16.17	11.06	7.83	39.19	14.84	11.58	23.37	28.22	11.52	35.25	13.50	36.46	9.72	4.59	38.86	11.50	10.62	20.32	19.60	>0.999999999999999	1.0
P11142	Heat shock cognate 71 kDa protein OS=Homo sapiens GN=HSPA8 PE=1 SV=1	25.89	33.88	14.12	25.78	15.34	28.53	19.57	16.80	4.60	7.51	37.11	14.64	13.86	25.38	11.78	10.14	16.59	13.50	12.68	22.03	3.57	28.26	17.79	6.02	20.31	15.13	>0.999999999999999	1.3
P01620	Ig kappa chain V-III region SIE OS=Homo sapiens PE=1 SV=1	27.84	40.57	19.72	26.69	33.75	9.61	19.23	10.46	10.15	11.90	21.59	11.36	10.00	35.45	28.44	12.14	33.18	10.12	18.07	8.55	5.42	36.57	17.48	3.75	20.24	18.27	>0.999999999999999	1.1
P31151	Protein S100-A7 OS=Homo sapiens GN=S100A7 PE=1 SV=4	44.94	62.00	30.14	4.55	0.00	0.00	45.38	10.94	1.19	12.42	22.53	6.87	10.44	54.19	7.67	8.21	22.43	10.58	18.86	23.84	8.28	38.23	18.25	10.45	20.08	19.29	>0.999999999999999	1.0
P02679	Fibrinogen gamma chain OS=Homo sapiens GN=FGG PE=1 SV=3	23.45	28.12	10.76	36.55	42.38	12.37	28.17	9.99	2.93	12.73	25.75	7.37	16.14	33.64	34.44	5.86	34.69	13.04	9.99	11.01	3.76	41.97	30.98	4.83	20.05	20.03	>0.999999999999999	1.0
P02675	Fibrinogen beta chain OS=Homo sapiens GN=FGB PE=1 SV=2	18.56	37.11	6.39	47.16	33.95	7.13	20.41	10.46	2.62	20.14	30.48	4.88	16.93	24.38	17.89	7.45	27.71	11.66	10.46	6.35	2.34	33.45	29.60	6.13	19.94	16.20	>0.999999999999999	1.2
P13489	Ribonuclease inhibitor OS=Homo sapiens GN=RNH1 PE=1 SV=2	26.87	22.36	27.34	11.22	18.60	29.69	28.51	13.47	7.38	6.68	28.97	16.83	10.79	27.60	20.66	7.59	12.07	14.88	17.44	24.10	4.22	35.12	14.88	6.25	19.83	16.30	>0.999999999999999	1.2
O43707	Alpha-actinin-4 OS=Homo sapiens GN=ACTN4 PE=1 SV=2	28.09	38.26	17.70	20.62	11.12	25.47	18.39	13.47	7.61	13.46	28.59	15.04	7.90	30.42	9.44	9.38	13.95	17.64	9.99	23.71	2.96	36.36	17.64	7.32	19.82	15.56	>0.999999999999999	1.3
Q06323	Proteasome activator complex subunit 1 OS=Homo sapiens GN=PSME1 PE=1 SV=1	28.09	26.51	18.71	21.23	26.85	28.82	15.69	14.74	6.62	2.50	31.62	13.95	8.16	28.20	15.55	11.52	13.76	11.20	3.80	25.65	3.54	34.70	25.61	9.48	19.61	15.93	>0.999999999999999	1.2
P00338	L-lactate dehydrogenase A chain OS=Homo sapiens GN=LDHA PE=1 SV=2	33.22	31.35	13.11	20.62	26.08	26.20	16.70	10.62	8.13	12.21	22.15	11.65	10.26	36.26	17.44	9.38	22.06	24.08	13.00	30.19	4.84	42.60	27.61	8.91	19.34	20.55	>0.999999999999999	0.9
P63208	S-phase kinase-associated protein 1 OS=Homo sapiens GN=SKP1 PE=1 SV=2	52.76	38.72	10.42	9.71	24.36	31.44	10.80	6.18	5.04	0.00	24.05	12.65	14.74	25.58	4.33	4.41	7.35	5.98	2.85	8.29	2.86	13.30	14.26	5.28	18.84	9.10	>0.999999999999999	2.1
P68366	Tubulin alpha-4A chain OS=Homo sapiens GN=TUBA4A PE=1 SV=1	38.59	26.05	17.70	19.26	0.00	33.77	0.00	13.95	8.37	5.84	32.95	23.11	9.91	20.14	9.78	8.76	0.00	11.66	13.95	0.00	2.71	18.29	8.59	6.42	18.30	9.18	>0.999999999999999	2.0
P07195	L-lactate dehydrogenase B chain OS=Homo sapiens GN=LDHB PE=1 SV=2	28.58	31.35	15.24	20.62	22.44	17.03	19.74	13.16	3.93	5.64	29.92	11.65	11.93	27.40	11.00	9.38	22.06	20.86	13.16	12.83	3.60	28.26	24.23	6.64	18.27	15.95	>0.999999999999999	1.1
P61204	ADP-ribosylation factor 3 OS=Homo sapiens GN=ARF3 PE=1 SV=2	27.60	13.14	20.95	28.36	6.71	16.45	14.00	5.55	9.28	5.95	44.31	23.31	3.07	0.00	3.89	2.41	6.60	8.74	9.04	10.75	2.56	11.84	5.37	8.35	17.97	6.05	>0.999999999999999	3.0
P55072	Transitional endoplasmic reticulum ATPase OS=Homo sapiens GN=VCP PE=1 SV=4	33.22	31.35	11.76	18.20	18.60	18.63	16.36	14.27	6.11	5.01	24.24	16.24	8.51	21.15	10.00	6.21	5.28	13.80	9.35	14.51	3.23	21.82	13.80	7.72	17.83	11.28	>0.999999999999999	1.6
P02766	Transthyretin OS=Homo sapiens GN=TTR PE=1 SV=1	19.05	4.84	2.35	11.83	22.25	6.84	13.16	12.36	1.86	12.11	88.04	16.14	0.00	4.23	0.00	1.45	14.71	3.22	34.40	2.72	4.99	4.36	7.21	2.67	17.57	6.66	>0.999999999999999	2.6
P32119	Peroxiredoxin-2 OS=Homo sapiens GN=PRDX2 PE=1 SV=5	32.48	23.74	11.54	15.62	19.75	24.60	17.38	12.05	5.27	5.53	25.18	16.83	4.65	34.04	8.44	9.17	14.33	8.13	5.23	9.85	4.10	15.79	8.13	5.85	17.50	10.64	>0.999999999999999	1.6
Q01518	Adenylyl cyclase-associated protein 1 OS=Homo sapiens GN=CAP1 PE=1 SV=5	32.97	34.57	13.56	22.75	16.11	15.72	12.31	13.32	4.28	10.02	20.45	10.76	8.42	33.44	9.33	10.35	18.10	11.20	9.99	17.49	2.25	34.49	20.71	8.52	17.23	15.36	>0.999999999999999	1.1
B9A064	Immunoglobulin lambda-like polypeptide 5 OS=Homo sapiens GN=IGLL5 PE=2 SV=2	30.04	21.90	13.78	14.41	18.22	17.90	20.75	15.06	3.77	12.84	23.29	12.25	13.60	24.78	17.22	8.48	29.22	18.87	24.57	15.94	2.19	32.21	18.86	6.98	17.02	17.74	>0.999999999999999	1.0
P18669	Phosphoglycerate mutase 1 OS=Homo sapiens GN=PGAM1 PE=1 SV=2	34.19	21.44	10.42	17.44	7.48	24.31	19.40	8.72	7.85	9.71	26.51	13.95	6.40	23.17	12.78	11.52	10.37	11.20	8.72	21.64	3.54	29.09	25.61	7.95	16.78	14.33	>0.999999999999999	1.2
P13796	Plastin-2 OS=Homo sapiens GN=LCP1 PE=1 SV=6	10.75	29.04	6.39	27.90	22.25	11.64	24.97	4.91	2.26	25.05	30.11	4.38	11.05	17.93	10.78	4.97	23.75	9.97	9.04	4.79	2.22	71.90	41.72	2.50	16.64	17.55	>0.999999999999999	0.9
P07355	Annexin A2 OS=Homo sapiens GN=ANXA2 PE=1 SV=2	30.77	21.21	10.31	11.68	9.59	24.31	15.52	23.14	5.00	11.38	17.42	18.83	9.56	12.69	0.00	8.69	3.39	11.81	17.28	11.92	3.88	8.10	5.98	2.21	16.60	7.96	>0.999999999999999	2.1
P02790	Hemopexin OS=Homo sapiens GN=HPX PE=1 SV=2	20.52	24.89	10.76	22.75	25.89	12.23	27.83	11.57	5.35	8.77	15.90	10.76	9.47	27.20	16.66	8.35	37.71	11.20	28.85	13.99	3.73	34.29	14.72	6.13	16.43	17.69	>0.999999999999999	0.9
P00568	Adenylate kinase isoenzyme 1 OS=Homo sapiens GN=AK1 PE=1 SV=3	18.81	39.87	11.76	20.62	10.36	25.18	5.57	8.56	2.14	0.00	40.71	13.55	11.93	15.51	15.11	7.24	6.22	5.06	2.38	13.60	1.66	3.12	5.06	3.07	16.43	7.50	>0.999999999999999	2.2
P52209	6-phosphogluconate dehydrogenase, decarboxylating OS=Homo sapiens GN=PGD PE=1 S	31.75	21.44	11.76	12.43	17.84	18.92	8.77	16.64	5.15	10.96	24.61	14.44	9.21	32.23	13.00	7.24	22.06	9.36	9.67	10.62	2.86	33.25	12.58	9.08	16.23	14.26	>0.999999999999999	1.1
P29401	Transketolase OS=Homo sapiens GN=TKT PE=1 SV=3	18.32	25.81	11.43	20.02	16.11	20.96	11.30	17.75	4.84	9.71	24.99	13.15	6.58	20.55	7.44	5.79	11.31	10.28	10.62	14.51	2.31	34.70	17.18	2.95	16.20	12.02	>0.999999999999999	1.3
P30085	UMP-CMP kinase OS=Homo sapiens GN=CMPK1 PE=1 SV=3	24.67	23.28	14.68	19.87	9.97	29.84	12.65	11.89	6.54	1.57	24.80	13.05	6.58	20.35	8.33	6.97	9.80	7.98	11.89	26.56	4.03	20.99	11.50	7.44	16.07	11.87	>0.999999999999999	1.4
P62158	Calmodulin OS=Homo sapiens GN=CALM1 PE=1 SV=2	36.39	16.83	8.18	30.18	20.71	15.72	12.31	17.12	2.89	4.59	20.45	7.27	13.07	14.71	16.55	7.45	13.76	6.75	11.57	19.30	2.25	22.44	16.56	6.13	16.05	12.55	>0.999999999999999	1.3
P35754	Glutaredoxin-1 OS=Homo sapiens GN=GLRX PE=1 SV=2	27.35	14.98	12.55	9.86	12.47	16.30	18.89	17.75	6.82	11.69	32.57	11.16	15.09	22.56	12.44	11.86	12.25	9.97	4.44	22.28	3.45	23.27	17.18	6.36	16.03	13.43	>0.999999999999999	1.2
P42330	Aldo-keto reductase family 1 member C3 OS=Homo sapiens GN=AKR1C3 PE=1 SV=4	32.97	26.74	19.94	10.16	15.73	22.71	13.83	15.54	4.60	0.00	18.56	11.55	8.60	16.52	12.89	8.00	12.63	12.58	0.00	17.49	3.02	20.36	12.58	6.59	16.03	10.94	>0.999999999999999	1.5
P62979	Ubiquitin-40S ribosomal protein S27a OS=Homo sapiens GN=RPS27A PE=1 SV=2	32.48	15.21	7.40	10.01	25.51	25.62	11.13	27.90	3.85	13.88	12.50	6.57	11.67	13.30	7.33	6.69	18.29	14.88	15.38	17.23	2.99	20.16	14.88	5.51	16.00	12.36	>0.999999999999999	1.3
P51858	Hepatoma-derived growth factor OS=Homo sapiens GN=HDGF PE=1 SV=1	45.67	43.10	14.23	6.37	8.05	18.49	13.50	6.66	1.67	1.25	15.15	15.44	2.28	12.09	2.89	4.14	2.26	12.27	4.12	10.36	1.29	5.40	1.84	7.21	15.80	5.51	>0.999999999999999	2.9
P16152	Carbonyl reductase [NADPH] 1 OS=Homo sapiens GN=CBR1 PE=1 SV=3	21.00	35.27	14.34	7.73	12.85	26.20	11.30	20.29	8.37	1.15	20.07	10.56	5.88	42.51	11.78	3.52	9.61	7.82	5.71	23.32	5.54	17.87	19.63	7.27	15.76	13.37	>0.999999999999999	1.2
P28838	Cytosol aminopeptidase OS=Homo sapiens GN=LAP3 PE=1 SV=3	21.00	25.12	12.21	13.04	18.60	23.58	14.51	9.04	7.02	1.25	22.91	16.14	8.51	21.96	8.44	5.93	4.71	13.19	0.00	11.14	2.34	33.66	16.72	4.88	15.37	10.96	>0.999999999999999	1.4
Q13228	Selenium-binding protein 1 OS=Homo sapiens GN=SELENBP1 PE=1 SV=2	25.89	24.43	9.30	10.92	13.81	21.40	10.46	14.90	4.20	1.36	22.53	23.31	4.65	21.35	6.89	6.48	9.99	12.73	3.17	8.03	1.63	17.25	11.04	5.34	15.21	9.05	>0.999999999999999	1.7
P30050	60S ribosomal protein L12 OS=Homo sapiens GN=RPL12 PE=1 SV=1	10.01	22.82	20.28	15.01	18.99	26.35	11.47	10.78	5.39	1.98	25.75	13.55	5.97	27.40	0.00	4.69	7.73	15.18	6.50	17.62	1.26	3.95	0.00	3.86	15.20	7.85	>0.999999999999999	1.9
P25815	Protein S100-P OS=Homo sapiens GN=S100P PE=1 SV=2	33.22	17.75	15.24	11.68	26.08	11.21	12.99	12.21	5.39	8.04	14.58	13.55	6.75	43.31	8.55	9.38	14.52	11.81	12.21	9.98	2.37	16.00	11.81	7.72	15.16	12.87	>0.999999999999999	1.2
P40925	Malate dehydrogenase, cytoplasmic OS=Homo sapiens GN=MDH1 PE=1 SV=4	20.27	19.13	9.30	17.89	26.47	23.14	14.00	18.71	4.68	4.28	15.72	8.27	12.11	20.14	13.11	8.14	10.18	15.34	8.56	12.96	3.08	38.03	8.28	5.68	15.15	12.97	>0.999999999999999	1.2
P01023	Alpha-2-macroglobulin OS=Homo sapiens GN=A2M PE=1 SV=3	14.65	16.83	8.63	26.99	24.55	4.22	34.75	5.07	3.93	13.36	20.26	7.67	16.05	26.79	16.33	7.38	23.38	15.80	43.28	7.00	4.99	41.56	19.02	5.17	15.08	18.89	>0.999999999999999	0.8
P78417	Glutathione S-transferase omega-1 OS=Homo sapiens GN=GSTO1 PE=1 SV=2	35.90	33.88	10.87	14.71	10.93	17.61	4.22	15.38	2.26	5.95	18.37	9.66	8.51	24.38	10.78	8.35	10.75	11.66	1.90	15.68	2.34	11.84	14.88	5.51	14.98	10.55	>0.999999999999999	1.4
P02774	Vitamin D-binding protein OS=Homo sapiens GN=GC PE=1 SV=1	19.54	13.83	14.23	19.26	27.04	14.85	21.42	9.51	4.52	4.38	19.31	10.16	15.00	16.12	15.66	6.28	29.41	9.20	18.07	7.77	3.91	35.53	15.64	5.79	14.84	14.87	>0.999999999999999	1.0
P01766	Ig heavy chain V-III region BRO OS=Homo sapiens PE=1 SV=1	23.94	13.37	10.98	14.86	28.58	8.44	16.53	15.54	5.91	10.23	18.56	9.76	5.09	19.74	10.89	6.76	18.48	15.03	9.19	7.51	1.79	20.36	3.99	3.29	14.72	10.18	>0.999999999999999	1.4
P13645	Keratin, type I cytoskeletal 10 OS=Homo sapiens GN=KRT10 PE=1 SV=6	5.86	31.81	2.69	15.47	7.29	20.09	25.47	19.97	2.85	30.06	5.87	9.16	8.95	7.65	7.00	6.35	11.88	19.33	33.61	4.02	0.95	2.29	15.64	6.47	14.72	10.34	>0.999999999999999	1.4
P30740	Leukocyte elastase inhibitor OS=Homo sapiens GN=SERPINB1 PE=1 SV=1	23.20	21.90	16.13	8.49	21.10	20.96	16.03	7.13	7.26	3.65	20.83	9.46	4.91	25.38	9.00	6.55	15.27	12.42	8.88	14.25	2.49	38.03	8.59	8.18	14.68	12.83	>0.999999999999999	1.1
P07437	Tubulin beta chain OS=Homo sapiens GN=TUBB PE=1 SV=2	43.23	0.00	11.43	24.26	0.00	31.29	0.00	10.62	7.02	3.97	27.27	15.94	6.84	17.93	5.22	4.62	0.00	11.96	7.45	10.11	2.74	13.92	0.00	4.43	14.59	7.10	>0.999999999999999	2.1
P06396	Gelsolin OS=Homo sapiens GN=GSN PE=1 SV=1	20.03	15.90	10.76	16.83	19.75	17.32	18.72	13.00	4.40	7.83	18.18	11.85	10.44	20.75	12.33	8.21	20.93	13.65	15.22	14.38	2.74	24.73	18.25	5.05	14.55	13.89	>0.999999999999999	1.0
P61769	Beta-2-microglobulin OS=Homo sapiens GN=B2M PE=1 SV=1	13.43	21.44	10.42	14.10	27.04	13.54	9.28	14.74	2.18	14.72	10.41	19.92	8.16	11.08	10.33	6.41	17.53	3.83	14.74	12.05	6.16	29.30	8.44	3.12	14.27	10.93	>0.999999999999999	1.3
Q9Y3Z3	SAM domain and HD domain-containing protein 1 OS=Homo sapiens GN=SAMHD1 PE=1 S	24.91	17.75	10.53	8.34	14.77	19.07	20.41	7.77	3.73	0.94	28.78	14.05	7.46	11.08	4.00	1.72	0.00	6.44	0.79	7.13	1.29	10.18	6.44	3.12	14.25	4.97	>0.999999999999999	2.9
P04264	Keratin, type II cytoskeletal 1 OS=Homo sapiens GN=KRT1 PE=1 SV=6	19.29	22.36	1.79	14.71	10.55	21.98	23.45	8.72	2.18	22.96	14.96	6.97	7.72	5.44	6.11	4.83	7.54	16.41	23.94	3.50	1.69	3.32	12.12	6.64	14.16	8.27	>0.999999999999999	1.7
P01861	Ig gamma-4 chain C region OS=Homo sapiens GN=IGHG4 PE=1 SV=1	20.52	15.90	6.16	12.74	22.82	14.70	17.04	13.32	2.74	14.51	19.12	10.06	12.19	20.35	13.22	8.21	26.21	12.88	10.94	10.88	2.59	24.73	18.25	5.73	14.14	13.85	>0.999999999999999	1.0
P15311	Ezrin OS=Homo sapiens GN=EZR PE=1 SV=4	26.62	17.06	12.21	11.22	8.63	18.92	15.35	15.85	3.97	3.97	22.53	11.85	8.77	10.48	7.33	4.55	7.16	7.98	5.07	11.79	2.03	20.78	12.58	3.75	14.02	8.52	>0.999999999999999	1.6
O95969	Secretoglobin family 1D member 2 OS=Homo sapiens GN=SCGB1D2 PE=2 SV=1	19.29	32.27	8.85	5.16	15.15	4.95	37.45	12.52	5.55	8.24	14.96	3.39	6.93	6.85	8.78	5.45	41.85	5.21	12.52	10.24	4.31	16.42	12.12	4.49	13.98	11.26	>0.999999999999999	1.2
P25311	Zinc-alpha-2-glycoprotein OS=Homo sapiens GN=AZGP1 PE=1 SV=2	18.07	20.74	6.50	8.80	17.26	10.77	15.18	32.02	4.32	7.72	14.01	10.86	2.81	9.07	6.44	8.90	8.48	13.80	11.73	7.51	3.97	15.38	8.90	2.55	13.86	8.30	>0.999999999999999	1.7
P05787	Keratin, type II cytoskeletal 8 OS=Homo sapiens GN=KRT8 PE=1 SV=7	19.78	26.97	10.31	12.28	13.62	18.92	11.98	11.26	2.82	13.57	17.42	7.07	5.35	12.29	9.00	4.21	8.11	9.36	11.26	9.20	1.88	8.94	9.36	4.60	13.83	7.79	>0.999999999999999	1.8
P80748	Ig lambda chain V-III region LOI OS=Homo sapiens PE=1 SV=1	16.12	15.21	12.66	17.14	33.37	9.61	11.13	10.46	2.62	18.16	12.50	6.57	5.79	13.30	12.55	4.55	12.44	10.12	17.91	8.55	2.03	23.48	17.33	6.42	13.80	11.21	>0.999999999999999	1.2
O60701	UDP-glucose 6-dehydrogenase OS=Homo sapiens GN=UGDH PE=1 SV=1	27.11	20.28	12.44	11.83	11.12	16.16	13.16	13.95	3.09	0.63	21.02	13.65	7.72	13.70	5.44	5.38	6.22	7.52	0.00	14.38	0.80	14.13	7.52	3.29	13.70	7.17	>0.999999999999999	1.9
P37837	Transaldolase OS=Homo sapiens GN=TALDO1 PE=1 SV=2	12.70	26.05	12.66	17.14	9.97	19.21	19.06	8.24	3.81	4.17	18.18	13.15	5.79	22.76	10.67	4.55	12.44	10.12	6.34	14.64	3.48	27.43	14.72	4.54	13.70	11.46	>0.999999999999999	1.2
P58546	Myotrophin OS=Homo sapiens GN=MTPN PE=1 SV=2	14.41	23.28	11.32	23.20	4.99	22.27	9.95	4.12	6.07	0.00	28.97	15.24	5.18	30.82	11.22	10.55	11.12	15.49	4.12	19.82	1.82	20.99	15.49	12.43	13.65	13.25	>0.999999999999999	1.0
P19652	Alpha-1-acid glycoprotein 2 OS=Homo sapiens GN=ORM2 PE=1 SV=2	16.85	21.44	13.45	14.10	17.84	10.04	15.69	10.94	2.74	15.76	17.61	6.87	4.21	13.90	16.78	4.76	28.47	14.26	19.02	3.89	2.12	31.38	18.40	5.28	13.61	13.54	>0.999999999999999	1.0
O00151	PDZ and LIM domain protein 1 OS=Homo sapiens GN=PDLIM1 PE=1 SV=4	24.42	15.67	13.22	12.74	10.36	14.56	11.47	13.32	3.97	0.00	22.34	20.82	5.97	8.46	9.33	6.90	5.66	10.43	4.76	8.81	2.59	6.23	10.43	3.86	13.57	6.95	>0.999999999999999	2.0
Q04828	Aldo-keto reductase family 1 member C1 OS=Homo sapiens GN=AKR1C1 PE=1 SV=1	0.00	41.03	0.00	0.00	15.73	33.48	13.83	21.40	0.00	3.03	18.56	15.54	11.84	0.00	0.00	0.00	15.46	23.93	3.01	0.00	0.00	32.42	15.03	11.52	13.55	9.43	>0.999999999999999	1.4
Q9NZT1	Calmodulin-like protein 5 OS=Homo sapiens GN=CALML5 PE=1 SV=2	5.13	26.97	18.26	7.13	4.03	3.06	19.74	25.84	3.13	17.01	22.15	7.87	6.93	23.57	8.78	8.07	30.73	12.12	7.45	15.16	0.65	24.31	7.21	4.49	13.36	12.45	>0.999999999999999	1.1
Q5VTE0	Putative elongation factor 1-alpha-like 3 OS=Homo sapiens GN=EEF1A1P5 PE=5 SV=1	21.49	23.05	11.20	10.01	12.66	14.56	11.13	18.07	3.97	4.80	18.93	9.96	4.91	15.51	8.55	6.07	10.56	10.12	8.88	12.96	2.71	13.71	7.06	5.00	13.32	8.84	>0.999999999999999	1.5
Q15847	Adipose most abundant gene transcript 2 protein OS=Homo sapiens GN=APM2 PE=1 SV=1	49.58	25.12	12.21	6.82	0.00	6.55	18.39	17.28	4.32	0.00	8.52	10.86	0.00	9.07	12.11	3.10	0.00	6.90	7.13	5.83	3.36	9.35	0.00	6.19	13.30	5.25	>0.999999999999999	2.5
P17655	Calpain-2 catalytic subunit OS=Homo sapiens GN=CAPN2 PE=1 SV=6	29.80	28.12	10.87	6.52	11.70	20.38	9.28	8.72	2.42	1.36	18.37	9.66	6.58	12.29	5.44	3.38	6.03	9.36	3.49	10.62	1.51	11.43	9.36	6.93	13.10	7.20	>0.999999999999999	1.8
Q96KP4	Cytosolic non-specific dipeptidase OS=Homo sapiens GN=CNDP2 PE=1 SV=2	20.03	24.43	11.88	7.89	13.81	19.21	8.77	9.83	4.20	0.63	24.99	9.36	4.56	18.94	8.00	4.28	8.11	9.51	3.17	12.18	2.89	14.96	9.51	5.34	12.92	8.45	>0.999999999999999	1.5
P15531	Nucleoside diphosphate kinase A OS=Homo sapiens GN=NME1 PE=1 SV=1	25.40	33.19	11.65	15.77	13.62	15.14	0.00	11.26	2.82	4.49	13.44	7.07	9.12	20.95	7.89	7.17	3.77	15.95	0.00	13.47	0.00	14.75	10.89	5.90	12.82	9.16	>0.999999999999999	1.4
P10768	S-formylglutathione hydrolase OS=Homo sapiens GN=ESD PE=1 SV=2	29.55	23.05	16.02	3.34	9.40	17.61	5.90	3.49	3.97	1.04	22.91	16.83	3.07	4.43	11.11	3.38	6.60	7.52	3.49	10.49	0.31	13.30	5.37	4.60	12.76	6.14	>0.999999999999999	2.1
P26447	Protein S100-A4 OS=Homo sapiens GN=S100A4 PE=1 SV=1	16.12	26.05	19.50	10.01	12.66	9.61	11.13	10.46	6.90	0.00	12.50	17.33	2.54	5.84	19.33	7.79	5.47	10.12	4.60	8.55	2.03	13.71	10.12	9.88	12.69	8.33	>0.999999999999999	1.5
P06744	Glucose-6-phosphate isomerase OS=Homo sapiens GN=GPI PE=1 SV=4	20.27	17.06	11.54	15.62	8.05	14.99	14.00	6.66	4.48	7.72	17.61	10.26	7.28	20.75	7.33	4.00	12.44	7.67	4.60	13.34	2.56	33.45	14.26	5.85	12.36	11.13	>0.999999999999999	1.1
O60218	Aldo-keto reductase family 1 member B10 OS=Homo sapiens GN=AKR1B10 PE=1 SV=2	34.19	12.68	13.45	12.89	16.30	20.38	5.06	8.72	4.04	0.94	10.41	8.47	7.46	17.12	6.11	5.86	13.01	6.44	1.43	7.13	1.29	8.73	4.60	2.38	12.29	6.80	>0.999999999999999	1.8
P02765	Alpha-2-HS-glycoprotein OS=Homo sapiens GN=AHSG PE=1 SV=1	21.74	6.22	13.56	15.77	26.66	5.39	15.01	5.87	3.53	5.11	19.69	8.87	10.61	20.95	13.44	8.35	22.81	7.52	25.21	7.90	2.74	21.61	18.56	4.20	12.28	13.66	>0.999999999999999	0.9
O14818	Proteasome subunit alpha type-7 OS=Homo sapiens GN=PSMA7 PE=1 SV=1	29.06	22.13	10.76	18.05	4.79	25.33	6.75	6.34	2.97	2.61	10.79	5.68	5.00	11.48	4.44	5.17	2.26	11.50	3.96	12.44	2.31	19.95	8.74	8.23	12.10	7.96	>0.999999999999999	1.5
P23396	40S ribosomal protein S3 OS=Homo sapiens GN=RPS3 PE=1 SV=2	10.26	18.44	11.43	9.10	0.00	32.31	7.08	16.17	4.04	4.38	19.31	12.65	7.02	16.12	8.89	7.04	0.00	19.48	4.12	16.45	1.85	5.40	6.44	5.79	12.10	8.22	>0.999999999999999	1.5
P01871	Ig mu chain C region OS=Homo sapiens GN=IGHM PE=1 SV=3	9.28	17.98	7.51	7.13	37.59	4.22	13.16	7.45	4.56	9.29	21.77	4.68	7.81	13.50	9.89	4.62	40.72	10.28	10.62	11.53	4.00	21.20	22.24	3.18	12.05	13.30	>0.999999999999999	0.9
P00325	Alcohol dehydrogenase 1B OS=Homo sapiens GN=ADH1B PE=1 SV=2	24.91	23.51	9.75	13.19	16.69	0.00	0.00	11.57	5.39	0.00	25.75	13.55	7.63	23.77	8.11	0.00	0.00	0.00	7.61	13.22	0.00	21.20	0.00	4.94	12.03	7.21	>0.999999999999999	1.7
P29692	Elongation factor 1-delta OS=Homo sapiens GN=EEF1D PE=1 SV=5	16.12	34.34	9.41	10.01	12.66	12.23	17.38	5.55	2.62	3.65	9.47	10.26	4.39	16.92	5.55	5.79	4.15	15.80	3.49	16.20	2.03	17.45	7.67	7.10	11.97	8.88	>0.999999999999999	1.3
P08670	Vimentin OS=Homo sapiens GN=VIM PE=1 SV=4	6.59	18.67	3.03	21.84	13.62	11.79	15.52	8.24	3.65	16.59	17.42	6.08	7.11	16.32	5.78	4.90	11.50	9.36	9.67	7.90	1.88	21.61	14.11	2.44	11.92	9.38	>0.999999999999999	1.3
O75223	Gamma-glutamylcyclotransferase OS=Homo sapiens GN=GGCT PE=1 SV=1	13.43	32.73	6.16	12.13	3.07	26.20	9.28	8.72	2.18	1.67	15.15	10.86	2.98	21.96	8.89	5.52	0.00	8.44	2.54	14.12	3.36	11.43	12.27	6.19	11.80	8.14	>0.999999999999999	1.4
P06454	Prothymosin alpha OS=Homo sapiens GN=PTMA PE=1 SV=2	26.87	14.75	7.17	9.71	12.27	9.32	10.80	17.44	2.54	2.92	20.83	6.38	5.61	12.89	12.22	4.41	12.07	9.82	10.15	8.29	1.97	13.30	9.82	3.63	11.75	8.68	>0.999999999999999	1.4
P48506	Glutamate--cysteine ligase catalytic subunit OS=Homo sapiens GN=GCLC PE=1 SV=2	20.52	25.35	9.41	8.34	16.11	20.38	5.06	7.61	2.74	0.00	14.39	10.96	4.82	7.25	4.00	3.31	4.52	6.44	0.00	12.05	1.91	4.99	3.68	3.92	11.74	4.74	>0.999999999999999	2.5
Q9Y333	U6 snRNA-associated Sm-like protein LSm2 OS=Homo sapiens GN=LSM2 PE=1 SV=1	17.83	16.83	14.23	4.70	24.36	18.49	12.31	11.57	1.23	3.24	5.87	7.27	2.72	6.24	3.44	5.03	0.00	4.75	4.91	9.46	0.00	26.39	4.75	7.21	11.49	6.24	>0.999999999999999	1.8
Q13885	Tubulin beta-2A chain OS=Homo sapiens GN=TUBB2A PE=1 SV=1	31.26	20.51	9.97	0.00	0.00	28.24	7.76	0.00	5.67	0.00	21.59	12.75	5.79	17.93	4.11	4.55	0.00	8.59	0.00	8.55	2.37	11.64	7.06	4.37	11.48	6.25	>0.999999999999999	1.8
P05388	60S acidic ribosomal protein P0 OS=Homo sapiens GN=RPLP0 PE=1 SV=1	21.98	13.37	19.61	8.80	0.00	18.78	15.18	0.00	4.32	0.00	24.43	10.86	5.09	14.91	10.00	6.21	8.48	19.79	11.73	16.71	4.65	0.00	0.00	6.19	11.44	8.65	>0.999999999999999	1.3
P41567	Eukaryotic translation initiation factor 1 OS=Homo sapiens GN=EIF1 PE=1 SV=1	38.35	6.22	11.54	4.09	19.75	14.99	10.12	4.28	4.08	6.26	11.36	5.98	5.26	12.09	6.67	7.10	5.09	9.20	0.00	7.77	1.85	5.61	4.14	3.41	11.42	5.68	>0.999999999999999	2.0
Q14894	Thiomorpholine-carboxylate dehydrogenase OS=Homo sapiens GN=CRYM PE=1 SV=1	18.32	21.21	8.40	13.95	8.63	16.16	9.95	9.35	3.65	0.00	14.20	11.06	6.58	15.11	6.55	4.07	6.03	9.05	0.00	9.72	1.39	15.58	14.11	6.30	11.24	7.87	>0.999999999999999	1.4
Q00796	Sorbitol dehydrogenase OS=Homo sapiens GN=SORD PE=1 SV=4	18.81	14.52	12.10	4.25	1.53	18.34	10.63	12.21	5.75	0.00	23.86	12.55	1.58	18.53	8.55	4.35	0.00	7.67	1.27	16.32	2.83	16.00	22.24	8.23	11.21	8.96	>0.999999999999999	1.3
P61960	Ubiquitin-fold modifier 1 OS=Homo sapiens GN=UFM1 PE=1 SV=1	21.98	20.74	4.26	5.76	17.26	13.10	6.41	14.27	3.57	0.00	17.04	8.96	7.90	32.63	4.22	6.21	0.00	13.80	0.00	11.66	2.77	7.90	5.83	5.11	11.11	8.17	>0.999999999999999	1.4
P20061	Transcobalamin-1 OS=Homo sapiens GN=TCN1 PE=1 SV=2	16.85	11.06	8.96	12.13	17.64	8.44	13.50	10.94	3.17	9.60	13.06	7.97	6.05	9.67	8.89	4.76	19.98	8.90	10.94	8.94	3.26	14.34	12.27	3.92	11.11	9.33	>0.999999999999999	1.2
P37108	Signal recognition particle 14 kDa protein OS=Homo sapiens GN=SRP14 PE=1 SV=2	32.00	30.19	5.83	7.89	4.41	19.07	3.88	0.00	2.06	2.40	16.47	8.67	4.56	17.53	0.00	6.00	0.00	7.98	0.00	11.27	0.71	4.78	3.53	4.94	11.07	5.11	>0.999999999999999	2.2
P63241	Eukaryotic translation initiation factor 5A-1 OS=Homo sapiens GN=EIF5A PE=1 SV=2	25.89	24.43	8.07	0.00	3.84	28.38	3.37	3.17	4.20	0.00	20.07	10.56	3.77	8.66	4.78	0.00	8.11	16.26	11.41	9.33	4.53	8.94	11.04	6.02	11.00	7.74	>0.999999999999999	1.4
P09467	Fructose-1,6-bisphosphatase 1 OS=Homo sapiens GN=FBP1 PE=1 SV=5	12.94	18.90	5.94	12.43	10.16	22.71	13.83	8.40	3.89	1.98	12.69	6.67	5.88	16.52	5.89	5.66	7.73	10.28	10.62	17.49	2.06	17.04	10.28	4.66	10.88	9.51	>0.999999999999999	1.1
P35579	Myosin-9 OS=Homo sapiens GN=MYH9 PE=1 SV=4	18.07	21.21	8.85	8.34	7.10	15.87	10.12	5.55	3.57	9.91	14.01	7.87	3.95	15.51	5.67	4.28	4.90	7.52	7.77	9.98	1.79	26.60	10.28	3.80	10.87	8.50	>0.999999999999999	1.3
P61978	Heterogeneous nuclear ribonucleoprotein K OS=Homo sapiens GN=HNRNPK PE=1 SV=1	18.32	14.98	12.44	9.86	8.63	14.26	7.59	5.87	3.41	4.70	16.28	13.85	3.95	15.11	7.22	5.17	8.48	8.44	8.72	9.72	1.39	7.69	8.44	3.12	10.85	7.29	>0.999999999999999	1.5
Q99417	C-Myc-binding protein OS=Homo sapiens GN=MYCBP PE=1 SV=3	42.50	15.21	3.25	4.40	0.00	16.45	4.89	4.60	1.15	0.00	12.50	25.10	5.79	0.00	0.00	0.00	0.00	0.00	0.00	3.76	0.00	0.00	0.00	1.65	10.84	0.93	>0.999999999999999	11.6
P17174	Aspartate aminotransferase, cytoplasmic OS=Homo sapiens GN=GOT1 PE=1 SV=3	24.18	26.05	2.58	2.27	9.78	26.20	2.53	8.08	2.89	0.73	16.28	7.27	0.61	6.45	2.56	3.52	2.83	4.91	0.00	9.46	1.26	12.88	2.30	2.90	10.74	4.14	>0.999999999999999	2.6
O95336	6-phosphogluconolactonase OS=Homo sapiens GN=PGLS PE=1 SV=2	23.69	17.52	10.87	6.07	2.30	25.62	2.02	1.90	5.83	0.00	18.37	14.64	3.51	11.48	2.78	6.69	2.26	6.14	0.00	5.18	0.37	15.79	6.13	4.31	10.74	5.39	>0.999999999999999	2.0
P40227	T-complex protein 1 subunit zeta OS=Homo sapiens GN=CCT6A PE=1 SV=3	25.40	21.44	10.42	7.13	7.48	18.49	7.93	4.91	2.89	0.63	13.82	8.27	2.72	11.08	6.11	2.69	2.26	8.44	2.85	6.09	0.95	13.30	7.21	3.12	10.73	5.57	>0.999999999999999	1.9
O00764	Pyridoxal kinase OS=Homo sapiens GN=PDXK PE=1 SV=1	21.25	24.20	9.75	4.70	8.25	21.11	9.62	4.91	2.82	0.94	10.79	10.46	3.77	21.15	4.78	6.00	5.84	3.07	1.43	13.60	2.19	18.08	6.60	4.94	10.73	7.62	>0.999999999999999	1.4
P59998	Actin-related protein 2/3 complex subunit 4 OS=Homo sapiens GN=ARPC4 PE=1 SV=3	9.04	20.28	13.45	13.34	11.51	12.81	14.84	5.87	2.38	6.26	7.01	11.95	5.26	31.63	9.78	8.28	11.31	13.50	5.87	11.40	1.85	32.62	13.50	3.41	10.73	12.37	>0.999999999999999	0.9
P68036	Ubiquitin-conjugating enzyme E2 L3 OS=Homo sapiens GN=UBE2L3 PE=1 SV=1	24.18	22.82	4.59	6.22	13.04	19.80	6.92	6.50	5.39	4.28	7.76	6.77	8.68	13.70	4.55	4.69	7.73	6.29	10.78	8.81	2.09	14.13	10.43	2.33	10.69	7.85	>0.999999999999999	1.4
P52907	F-actin-capping protein subunit alpha-1 OS=Homo sapiens GN=CAPZA1 PE=1 SV=3	19.05	17.98	6.83	7.13	9.01	23.29	5.57	7.45	3.09	3.44	14.77	9.46	5.35	15.71	5.22	6.55	11.50	9.36	3.33	14.90	3.54	12.68	14.57	2.67	10.59	8.78	>0.999999999999999	1.2
P12429	Annexin A3 OS=Homo sapiens GN=ANXA3 PE=1 SV=3	20.52	12.45	7.62	6.37	13.04	14.56	7.08	15.85	2.70	8.77	7.95	9.96	4.74	6.04	1.00	0.00	1.70	1.38	1.43	7.00	0.92	11.22	2.91	2.38	10.57	3.39	>0.999999999999999	3.1
P02144	Myoglobin OS=Homo sapiens GN=MB PE=1 SV=2	35.41	16.36	11.76	6.52	8.25	21.11	7.25	6.82	0.79	0.00	8.14	4.28	3.77	4.03	2.22	1.38	3.77	3.07	0.00	2.59	0.00	0.00	3.07	2.44	10.56	2.20	>0.999999999999999	4.8
O75874	Isocitrate dehydrogenase [NADP] cytoplasmic OS=Homo sapiens GN=IDH1 PE=1 SV=2	14.90	19.36	12.44	10.92	7.67	18.34	10.29	9.67	2.85	2.40	9.47	8.37	7.37	16.92	6.78	6.69	2.83	9.36	3.65	12.57	1.23	10.39	4.75	4.09	10.56	7.22	>0.999999999999999	1.5
P63244	Guanine nucleotide-binding protein subunit beta-2-like 1 OS=Homo sapiens GN=GNB2L1 PE	25.40	16.36	9.75	4.70	3.84	17.90	3.37	11.26	4.88	2.09	16.47	10.36	2.72	14.30	4.78	3.86	3.77	22.09	8.88	9.20	3.20	4.16	3.07	4.94	10.53	7.08	>0.999999999999999	1.5
P22314	Ubiquitin-like modifier-activating enzyme 1 OS=Homo sapiens GN=UBA1 PE=1 SV=3	16.36	14.52	8.63	12.43	11.32	14.85	5.90	10.62	3.25	2.19	15.53	10.76	3.33	14.50	6.00	4.62	6.60	7.67	4.44	9.98	2.06	11.22	6.44	4.09	10.53	6.75	>0.999999999999999	1.6
P13639	Elongation factor 2 OS=Homo sapiens GN=EEF2 PE=1 SV=4	15.88	13.83	10.64	9.86	11.51	12.95	10.96	7.77	4.04	3.24	15.72	9.46	2.72	17.93	7.89	5.31	5.84	10.89	9.51	15.03	3.79	13.51	7.52	8.35	10.49	9.02	>0.999999999999999	1.2
Q9BQE3	Tubulin alpha-1C chain OS=Homo sapiens GN=TUBA1C PE=1 SV=1	0.00	40.34	0.00	21.53	0.00	40.32	0.00	15.85	7.65	0.00	0.00	0.00	16.93	31.83	0.00	13.31	0.00	17.48	15.85	30.06	4.37	20.78	13.50	0.00	10.47	13.68	>0.999999999999999	0.8
P36405	ADP-ribosylation factor-like protein 3 OS=Homo sapiens GN=ARL3 PE=1 SV=2	20.27	33.88	3.92	5.31	6.71	16.45	2.70	5.55	2.26	1.67	15.72	11.26	1.40	11.48	1.78	1.10	6.60	5.37	0.00	4.53	2.56	3.32	5.37	0.91	10.47	3.70	>0.999999999999999	2.8
P31150	Rab GDP dissociation inhibitor alpha OS=Homo sapiens GN=GDI1 PE=1 SV=2	24.18	12.68	9.75	8.34	8.63	16.16	9.28	5.87	2.58	2.92	14.20	11.06	4.82	11.08	4.11	2.55	5.28	8.44	0.00	11.27	1.69	18.08	8.44	2.55	10.47	6.53	>0.999999999999999	1.6
P50991	T-complex protein 1 subunit delta OS=Homo sapiens GN=CCT4 PE=1 SV=4	22.71	14.75	12.89	8.34	9.01	20.38	5.40	8.72	2.18	0.63	10.41	9.26	5.61	12.89	3.56	3.79	3.39	5.98	2.85	7.13	1.97	11.43	4.91	4.14	10.39	5.64	>0.999999999999999	1.8
P62424	60S ribosomal protein L7a OS=Homo sapiens GN=RPL7A PE=1 SV=2	21.25	15.90	12.10	3.49	16.69	19.07	6.24	5.87	4.28	1.15	13.06	5.18	2.02	17.53	4.11	4.76	4.34	7.98	1.74	13.99	1.14	4.78	1.69	2.95	10.36	5.58	>0.999999999999999	1.9
P25787	Proteasome subunit alpha type-2 OS=Homo sapiens GN=PSMA2 PE=1 SV=2	15.14	37.57	6.95	4.09	8.44	15.57	7.42	6.98	2.46	1.36	11.74	6.18	3.86	12.49	4.89	4.28	5.09	6.75	2.06	10.75	1.36	22.23	6.75	7.55	10.32	7.34	>0.999999999999999	1.4
P49773	Histidine triad nucleotide-binding protein 1 OS=Homo sapiens GN=HINT1 PE=1 SV=2	48.85	12.68	10.42	8.34	4.79	8.01	4.22	8.72	3.69	0.00	4.73	9.26	2.19	28.40	2.78	1.72	0.00	14.26	0.00	18.27	0.00	19.32	3.83	1.42	10.31	7.68	>0.999999999999999	1.3
P60953	Cell division control protein 42 homolog OS=Homo sapiens GN=CDC42 PE=1 SV=2	19.05	0.00	8.74	11.83	14.96	7.86	17.88	5.39	5.47	8.14	14.77	7.77	6.84	21.35	8.67	7.31	6.41	8.28	5.39	7.00	1.66	28.68	11.96	6.02	10.15	9.96	>0.999999999999999	1.0
Q9NQR4	Omega-amidase NIT2 OS=Homo sapiens GN=NIT2 PE=1 SV=1	16.12	23.97	9.41	10.01	9.59	12.23	6.07	3.65	2.62	0.00	19.69	8.37	2.02	16.92	4.00	2.48	6.79	5.52	1.74	13.47	1.11	7.48	10.12	4.77	10.14	6.37	>0.999999999999999	1.6
Q01105	Protein SET OS=Homo sapiens GN=SET PE=1 SV=3	18.56	21.67	6.72	6.98	8.82	11.06	10.12	9.51	3.01	4.80	14.39	5.98	4.04	9.27	5.11	4.14	11.31	5.06	7.29	7.77	0.65	9.56	9.20	3.41	10.14	6.40	>0.999999999999999	1.6
Q9Y230	RuvB-like 2 OS=Homo sapiens GN=RUVBL2 PE=1 SV=3	18.32	12.68	8.40	9.86	12.47	14.26	9.28	7.13	0.52	0.00	18.56	9.76	3.95	11.08	0.00	0.90	0.00	0.92	0.00	4.79	0.18	1.25	1.99	1.59	10.10	2.22	>0.999999999999999	4.5
Q15181	Inorganic pyrophosphatase OS=Homo sapiens GN=PPA1 PE=1 SV=2	14.90	21.90	6.83	3.18	9.41	19.80	13.16	3.33	3.77	2.19	11.55	11.46	2.89	12.29	5.22	9.38	1.89	9.36	1.59	17.62	3.54	19.74	7.21	4.43	10.09	7.93	>0.999999999999999	1.3
P19971	Thymidine phosphorylase OS=Homo sapiens GN=TYMP PE=1 SV=2	7.08	20.51	9.97	10.01	7.10	25.62	9.45	0.00	8.49	0.73	19.12	2.89	1.84	22.96	6.22	5.31	5.47	15.49	4.60	7.26	3.11	29.71	8.59	4.37	10.08	9.58	>0.999999999999999	1.1

P43490	Nicotinamide phosphoribosyltransferase OS=Homo sapiens GN=NAMPT PE=1 SV=1	14.17	17.98	13.78	10.31	4.99	17.90	9.78	9.19	5.99	1.98	7.76	6.77	5.09	30.42	8.67	5.38	3.58	7.52	3.01	14.38	2.71	23.07	8.90	5.62	10.05	9.86	>0.999999999999999	1.0
Q00610	Clathrin heavy chain 1 OS=Homo sapiens GN=CLTC PE=1 SV=5	19.54	11.99	8.63	5.61	11.51	20.81	5.57	4.12	3.77	2.30	17.42	9.16	4.56	13.09	7.00	4.90	4.52	7.52	3.17	9.98	1.76	6.86	5.67	4.37	10.03	6.12	>0.999999999999999	1.6
Q14764	Major vault protein OS=Homo sapiens GN=MVP PE=1 SV=4	22.23	19.59	10.20	5.76	5.75	17.03	4.22	3.33	4.36	0.73	19.69	7.27	3.77	13.70	5.22	3.93	1.89	4.60	3.96	8.81	1.45	14.13	7.21	3.52	10.01	6.02	>0.999999999999999	1.7
O75347	Tubulin-specific chaperone A OS=Homo sapiens GN=TBCA PE=1 SV=3	14.65	6.22	6.72	4.09	11.51	22.85	10.12	16.33	4.08	2.82	5.11	15.64	9.04	12.09	6.67	1.86	0.00	9.20	0.00	13.34	1.85	21.40	9.20	1.53	10.01	7.18	>0.999999999999999	1.4
P22392	Nucleoside diphosphate kinase B OS=Homo sapiens GN=NME2 PE=1 SV=1	25.16	0.00	21.29	0.00	13.42	27.66	17.38	11.10	4.08	0.00	0.00	0.00	0.00	14.10	7.78	0.00	0.00	0.00	11.10	0.00	3.17	14.55	0.00	5.85	10.01	4.71	>0.999999999999999	2.1
P50990	T-complex protein 1 subunit theta OS=Homo sapiens GN=CCT8 PE=1 SV=4	10.99	18.44	8.96	8.04	10.16	17.76	8.94	7.13	2.10	2.50	16.85	7.97	4.65	12.29	5.89	2.62	3.20	8.13	0.00	10.36	1.63	11.01	5.83	4.54	9.99	5.85	>0.999999999999999	1.7
P62269	40S ribosomal protein S18 OS=Homo sapiens GN=RPS18 PE=1 SV=3	24.67	9.68	4.71	2.88	3.64	26.78	7.08	6.66	5.51	1.98	19.12	6.87	1.67	28.00	4.67	2.90	0.00	10.58	6.66	13.09	0.59	3.95	0.00	0.00	9.97	6.01	>0.999999999999999	1.7
Q9ULZ3	Apoptosis-associated speck-like protein containing a CARD OS=Homo sapiens GN=PYCAR	19.05	12.45	3.70	2.43	14.96	19.94	9.11	5.23	1.31	8.14	14.77	7.77	1.40	6.65	6.00	3.72	6.22	5.06	5.23	10.11	2.40	28.47	11.96	3.07	9.90	7.52	>0.999999999999999	1.3
O75083	WD repeat-containing protein 1 OS=Homo sapiens GN=WDR1 PE=1 SV=4	17.10	14.29	14.12	9.40	6.33	12.66	9.11	7.45	5.00	0.52	14.77	7.77	5.44	17.53	11.66	6.62	6.22	9.51	0.00	8.03	2.68	24.10	9.51	6.59	9.88	8.99	>0.999999999999999	1.1
P35080	Profilin-2 OS=Homo sapiens GN=PFN2 PE=1 SV=3	20.03	18.90	5.49	7.43	15.73	7.13	8.27	7.77	0.87	2.30	15.53	8.17	4.30	4.43	2.44	3.38	4.15	3.37	0.00	10.62	0.68	17.04	3.37	2.78	9.80	4.71	>0.999999999999999	2.1
Q99832	T-complex protein 1 subunit eta OS=Homo sapiens GN=CCT7 PE=1 SV=2	17.34	18.44	8.96	8.04	11.89	16.30	1.86	4.91	2.46	0.52	15.15	11.16	2.72	12.49	6.89	2.14	3.20	2.61	1.74	5.83	1.39	7.90	3.68	4.54	9.75	4.59	>0.999999999999999	2.1
P01768	Ig heavy chain V-III region CAM OS=Homo sapiens PE=1 SV=1	13.68	12.91	6.27	14.25	27.42	0.00	9.45	8.88	3.73	9.81	10.60	0.00	0.00	11.28	6.22	3.86	0.00	0.00	3.96	12.18	0.00	11.64	0.00	0.00	9.75	4.10	>0.999999999999999	2.4
P22626	Heterogeneous nuclear ribonucleoproteins A2/B1 OS=Homo sapiens GN=HNRNPA2B1 PE=	16.12	12.22	9.08	6.07	7.67	29.11	8.94	2.85	2.62	0.94	10.04	11.36	2.54	16.32	7.33	5.59	7.54	2.76	12.84	10.49	0.89	6.03	12.42	3.75	9.75	7.38	>0.999999999999999	1.3
Q02878	60S ribosomal protein L6 OS=Homo sapiens GN=RPL6 PE=1 SV=3	28.58	14.29	10.87	7.13	4.03	20.23	5.74	0.00	3.85	1.04	11.74	7.87	0.00	4.23	2.33	1.45	0.00	1.53	1.59	2.72	0.31	2.08	0.00	0.57	9.61	1.40	>0.999999999999999	6.9
Q9NQ48	Leucine zipper transcription factor-like protein 1 OS=Homo sapiens GN=LZTFL1 PE=1 SV=1	17.83	16.83	4.93	3.03	6.14	13.10	12.31	14.27	0.79	0.00	17.04	8.96	5.09	6.45	2.22	1.38	1.89	4.91	0.00	5.70	0.62	0.00	3.07	0.57	9.60	2.66	>0.999999999999999	3.6
Q9H299	SH3 domain-binding glutamic acid-rich-like protein 3 OS=Homo sapiens GN=SH3BGRL3 PE	18.81	17.75	8.63	5.00	0.00	11.21	22.94	5.23	3.05	0.00	14.58	7.67	6.75	6.65	3.67	5.31	14.52	11.81	5.23	9.98	1.02	16.00	20.86	4.37	9.57	8.85	>0.999999999999999	1.1
P60660	Myosin light polypeptide 6 OS=Homo sapiens GN=MYL6 PE=1 SV=2	10.50	9.91	4.82	16.07	8.25	10.48	7.25	11.41	4.20	15.34	8.14	7.17	6.32	8.66	4.78	4.97	13.57	6.60	11.41	5.57	1.32	22.03	16.26	2.44	9.46	8.66	>0.999999999999999	1.1
Q96C19	EF-hand domain-containing protein D2 OS=Homo sapiens GN=EFHD2 PE=1 SV=1	10.26	13.83	6.72	6.37	4.99	32.60	2.02	9.51	3.17	4.38	11.36	7.97	1.05	8.46	4.67	1.79	4.90	3.99	4.12	13.22	1.29	16.62	12.27	4.54	9.43	6.41	>0.999999999999999	1.5
P62826	GTP-binding nuclear protein Ran OS=Homo sapiens GN=RAN PE=1 SV=3	16.36	10.83	7.51	7.13	9.01	12.95	15.01	7.45	2.66	1.46	12.69	8.87	5.88	23.17	7.44	3.24	5.47	13.65	0.00	8.68	2.06	9.77	4.45	3.80	9.33	7.30	>0.999999999999999	1.3
Q9Y265	RuvB-like 1 OS=Homo sapiens GN=RUVBL1 PE=1 SV=1	18.81	10.60	8.63	5.61	10.74	16.59	9.45	5.87	2.22	0.00	14.58	8.77	2.54	5.84	4.11	2.00	5.47	1.99	0.00	0.91	0.22	2.70	8.59	0.74	9.32	2.93	>0.999999999999999	3.2
P36955	Pigment epithelium-derived factor OS=Homo sapiens GN=SERPINF1 PE=1 SV=4	5.62	9.45	10.98	14.86	18.79	10.63	10.46	5.07	3.37	4.28	11.74	6.18	7.46	14.71	12.55	5.03	16.02	11.20	13.47	4.15	1.91	17.66	7.82	4.14	9.29	9.68	>0.999999999999999	1.0
P00450	Ceruloplasmin OS=Homo sapiens GN=CP PE=1 SV=1	10.75	8.53	7.28	12.59	17.84	6.99	10.96	6.98	2.38	5.43	13.06	8.27	7.28	13.09	9.78	3.86	15.65	6.29	18.07	6.74	2.12	18.29	9.20	3.92	9.25	9.52	>0.999999999999999	1.0
P17987	T-complex protein 1 subunit alpha OS=Homo sapiens GN=TCP1 PE=1 SV=1	21.49	20.28	7.73	7.89	4.41	14.26	2.87	8.24	1.47	1.15	11.55	8.77	1.49	13.90	5.00	3.10	2.07	3.53	1.74	6.74	0.92	10.81	2.61	1.70	9.18	4.47	>0.999999999999999	2.1
P25786	Proteasome subunit alpha type-1 OS=Homo sapiens GN=PSMA1 PE=1 SV=1	12.94	12.22	7.84	5.61	7.10	23.14	6.24	5.87	4.32	2.50	13.25	8.77	4.65	14.10	9.78	4.83	6.98	5.67	0.00	6.87	2.16	22.65	8.13	3.97	9.15	7.48	>0.999999999999999	1.2
P52895	Aldo-keto reductase family 1 member C2 OS=Homo sapiens GN=AKR1C2 PE=1 SV=3	32.97	0.00	22.86	23.66	0.00	19.65	0.00	0.00	10.35	0.00	0.00	0.00	0.00	41.10	15.00	10.76	0.00	0.00	0.00	26.43	3.57	0.00	0.00	0.00	9.12	8.07	>0.999999999999999	1.1
P07741	Adenine phosphoribosyltransferase OS=Homo sapiens GN=APRT PE=1 SV=2	4.15	20.28	9.86	9.10	7.10	22.85	6.24	2.69	3.49	0.00	7.01	15.64	1.49	7.45	6.67	6.07	3.20	0.00	0.00	7.77	2.71	12.47	2.61	5.00	9.03	4.62	>0.999999999999999	2.0
P17931	Galectin-3 OS=Homo sapiens GN=LGALS3 PE=1 SV=5	6.59	9.91	11.76	6.52	5.18	15.28	2.19	13.00	5.19	1.36	24.80	6.18	3.77	16.52	11.66	9.04	0.00	9.51	2.06	16.97	1.91	17.04	9.51	4.66	9.00	8.55	>0.999999999999999	1.1
P12814	Alpha-actinin-1 OS=Homo sapiens GN=ACTN1 PE=1 SV=2	10.01	16.13	4.59	11.37	7.86	11.79	7.59	5.07	3.21	10.23	14.20	5.48	5.18	10.07	4.00	3.79	9.43	6.29	3.80	9.07	1.26	28.88	13.19	2.33	8.96	8.11	>0.999999999999999	1.1
P09960	Leukotriene A-4 hydrolase OS=Homo sapiens GN=LTA4H PE=1 SV=2	16.12	13.37	7.40	8.80	8.44	11.94	4.39	6.02	2.02	7.72	15.53	5.78	5.09	21.96	6.44	4.55	6.03	7.82	4.12	10.62	0.99	39.07	19.79	4.20	8.96	10.89	>0.999999999999999	0.8
P62241	40S ribosomal protein S8 OS=Homo sapiens GN=RPS8 PE=1 SV=2	21.98	10.83	3.25	4.40	5.56	13.10	2.36	7.45	1.86	3.03	21.96	11.55	4.12	9.47	5.22	4.62	5.47	13.80	10.62	15.03	1.45	2.91	4.45	3.80	8.95	6.75	>0.999999999999999	1.3
P15559	NAD(P)H dehydrogenase [quinone] 1 OS=Homo sapiens GN=NQO1 PE=1 SV=1	12.46	15.44	5.71	10.16	6.90	18.49	8.60	3.65	0.91	0.00	16.09	8.47	2.02	17.12	4.00	4.62	4.34	5.52	1.74	8.68	0.71	4.78	7.82	2.04	8.91	5.28	>0.999999999999999	1.7
Q15185	Prostaglandin E synthase 3 OS=Homo sapiens GN=PTGES3 PE=1 SV=1	15.39	14.52	10.31	5.91	7.48	18.34	10.63	2.85	2.50	0.00	11.93	6.28	3.42	3.63	0.00	1.24	0.00	5.98	2.85	8.16	1.94	8.10	2.76	3.58	8.84	3.47	>0.999999999999999	2.5
P84085	ADP-ribosylation factor 5 OS=Homo sapiens GN=ARF5 PE=1 SV=2	20.27	8.07	12.66	0.00	0.00	12.08	0.00	5.55	4.48	0.00	27.83	14.64	3.07	7.05	0.00	0.00	0.00	8.74	0.00	14.64	0.00	0.00	8.74	0.00	8.80	3.52	>0.999999999999999	2.5
O15144	Actin-related protein 2/3 complex subunit 2 OS=Homo sapiens GN=ARPC2 PE=1 SV=1	18.07	13.37	5.04	8.80	6.14	15.87	5.40	7.13	2.30	6.05	10.98	5.78	1.75	11.68	3.56	2.21	8.48	4.91	5.07	11.66	0.62	6.65	6.90	3.29	8.74	5.57	>0.999999999999999	1.6
Q9H098	Protein FAM107B OS=Homo sapiens GN=FAM107B PE=1 SV=1	29.31	5.07	9.08	3.34	4.22	11.79	3.71	12.84	1.90	0.00	15.34	8.07	10.53	24.17	5.33	3.31	0.00	0.00	3.49	10.49	1.48	4.57	3.37	1.25	8.72	5.67	>0.999999999999999	1.5
Q15365	Poly(rC)-binding protein 1 OS=Homo sapiens GN=PCBP1 PE=1 SV=2	12.70	11.99	5.83	6.07	12.47	16.30	8.77	6.34	3.77	0.94	12.31	6.47	2.54	10.48	5.78	2.76	3.39	4.45	6.34	6.74	1.60	8.31	9.97	3.69	8.66	5.50	>0.999999999999999	1.6
P09104	Gamma-enolase OS=Homo sapiens GN=ENO2 PE=1 SV=3	20.27	16.36	7.96	6.07	13.62	12.08	0.00	6.34	0.00	0.00	11.36	9.46	7.28	0.00	0.00	0.00	0.00	0.00	0.00	7.77	0.00	0.00	7.52	0.00	8.63	1.88	>0.999999999999999	4.6
P47755	F-actin-capping protein subunit alpha-2 OS=Homo sapiens GN=CAPZA2 PE=1 SV=3	14.90	14.06	6.83	6.98	6.33	16.59	5.57	7.29	1.31	2.19	11.55	9.46	2.89	9.27	0.00	5.31	3.96	7.06	0.00	12.31	2.37	16.00	11.81	3.46	8.59	6.20	>0.999999999999999	1.4
P10909	Clusterin OS=Homo sapiens GN=CLU PE=1 SV=1	10.75	6.22	6.95	10.92	13.81	9.02	8.77	8.24	2.46	8.66	11.74	5.18	6.32	12.49	6.89	4.28	11.69	12.73	11.41	9.33	1.91	17.25	11.04	2.95	8.56	9.02	>0.999999999999999	0.9
P31939	Bifunctional purine biosynthesis protein PURH OS=Homo sapiens GN=ATIC PE=1 SV=3	17.59	14.75	3.81	6.22	1.92	20.81	3.71	3.49	1.35	0.52	15.34	13.05	2.46	4.43	1.78	3.86	0.94	2.45	1.59	7.26	1.48	2.08	3.37	3.18	8.55	2.91	>0.999999999999999	2.9
P02538	Keratin, type II cytoskeletal 6A OS=Homo sapiens GN=KRT6A PE=1 SV=3	5.86	18.21	2.69	6.82	4.60	3.49	13.33	6.02	0.00	35.80	5.68	0.00	3.33	22.16	5.89	2.07	0.00	8.13	17.44	3.11	0.00	9.35	0.00	1.70	8.54	6.10	>0.999999999999999	1.4
P01717	Ig lambda chain V-IV region Hil OS=Homo sapiens PE=1 SV=1	7.33	15.67	7.62	10.31	13.04	4.37	11.47	4.76	1.19	7.10	12.88	6.77	2.63	13.70	3.33	4.69	12.82	4.60	10.78	8.81	2.09	14.13	10.43	3.86	8.54	7.66	>0.999999999999999	1.1
P11021	78 kDa glucose-regulated protein OS=Homo sapiens GN=HSPA5 PE=1 SV=2	8.79	14.52	7.06	4.55	4.79	16.45	8.27	12.36	2.50	5.84	11.93	4.88	4.30	15.71	9.55	3.38	10.56	9.66	8.88	11.14	1.32	16.21	10.74	2.78	8.50	8.69	>0.999999999999999	1.0
P01033	Metalloproteinase inhibitor 1 OS=Homo sapiens GN=TIMP1 PE=1 SV=1	7.33	6.91	10.42	10.46	13.23	2.04	11.64	14.74	1.90	0.00	13.06	9.26	2.63	2.82	13.33	6.41	22.62	4.60	7.61	8.94	4.65	6.23	10.58	6.81	8.42	8.10	>0.999999999999999	1.0
P00491	Purine nucleoside phosphorylase OS=Homo sapiens GN=PNP PE=1 SV=2	11.72	18.44	2.47	9.55	4.22	14.26	8.10	7.61	1.90	2.30	15.15	4.78	0.88	16.12	0.00	3.31	1.89	7.36	0.00	2.85	0.68	16.62	5.21	1.93	8.37	4.74	>0.999999999999999	1.8
P00751	Complement factor B OS=Homo sapiens GN=CFB PE=1 SV=2	10.99	6.91	11.43	10.31	18.22	8.15	8.43	3.96	2.70	4.17	9.47	5.58	7.72	12.49	10.55	4.28	17.91	6.14	16.17	6.48	2.09	11.64	13.50	3.52	8.36	9.37	>0.999999999999999	0.9
P02671	Fibrinogen alpha chain OS=Homo sapiens GN=FGA PE=1 SV=2	10.99	10.37	6.16	12.59	17.26	3.20	17.38	2.22	1.07	4.17	12.50	1.79	7.28	9.07	6.67	1.24	14.52	4.75	7.93	3.50	1.85	12.47	8.44	1.53	8.31	6.60	>0.999999999999999	1.3
Q9NR45	Sialic acid synthase OS=Homo sapiens GN=NANS PE=1 SV=2	17.83	16.83	5.38	5.61	9.21	8.73	6.24	7.61	2.38	0.00	13.82	5.98	4.21	12.09	5.33	4.14	3.20	9.20	4.12	9.46	1.14	5.40	3.99	2.73	8.30	5.42	>0.999999999999999	1.5
P08729	Keratin, type II cytoskeletal 7 OS=Homo sapiens GN=KRT7 PE=1 SV=5	6.84	8.30	6.16	11.37	3.84	22.42	3.37	10.15	3.41	6.68	14.20	2.79	2.46	15.11	9.55	5.93	3.77	11.50	5.71	7.13	0.62	5.82	4.29	3.12	8.29	6.25	>0.999999999999999	1.3
P61088	Ubiquitin-conjugating enzyme E2 N OS=Homo sapiens GN=UBE2N PE=1 SV=1	25.64	24.20	4.82	0.00	13.62	10.33	0.00	0.00	0.00	0.00	13.44	7.07	0.00	14.30	0.00	0.00	0.00	0.00	6.82	0.00	2.19	0.00	0.00	0.00	8.26	1.94	>0.999999999999999	4.3
P39687	Acidic leucine-rich nuclear phosphoprotein 32 family member A OS=Homo sapiens GN=ANF	13.43	8.99	8.18	5.91	7.48	20.52	6.58	0.00	3.69	1.25	13.82	9.26	3.42	11.08	8.11	3.79	10.37	3.83	6.18	9.46	0.77	24.10	8.44	3.12	8.26	7.72	>0.999999999999999	1.1
P05455	Lupus La protein OS=Homo sapiens GN=SSB PE=1 SV=2	17.59	16.60	4.48	7.58	7.67	12.23	6.75	7.93	1.98	0.73	11.55	3.98	3.51	4.63	2.56	3.45	2.83	3.53	1.11	5.18	0.95	6.44	6.13	1.31	8.26	3.47	>0.999999999999999	2.4
P28066	Proteasome subunit alpha type-5 OS=Homo sapiens GN=PSMA5 PE=1 SV=3	10.50	14.06	6.83	6.52	8.25	15.14	7.25	6.82	4.12	2.82	8.14	8.07	3.77	8.66	4.78	4.21	5.09	6.60	4.28	7.90	1.32	12.68	6.60	2.44	8.21	5.69	>0.999999999999999	1.4
P02533	Keratin, type I cytoskeletal 14 OS=Homo sapiens GN=KRT14 PE=1 SV=4	4.88	10.37	0.00	6.82	6.90	7.86	7.59	8.56	0.00	38.30	6.82	0.00	3.16	10.88	0.00	3.72	0.00	8.28	13.47	0.00	0.62	4.16	6.90	2.55	8.18	4.48	>0.999999999999999	1.8
P30043	Flavin reductase (NADPH) OS=Homo sapiens GN=BLVRB PE=1 SV=3	18.56	11.99	11.43	4.85	2.88	19.36	0.00	8.24	3.01	1.57	6.06	10.16	4.56	15.31	3.56	2.21	0.00	4.91	5.07	9.85	2.34	6.65	4.91	4.31	8.18	5.31	>0.999999999999999	1.5
P29218	Inositol monophosphatase 1 OS=Homo sapiens GN=IMPA1 PE=1 SV=1	16.61	11.76	4.03	3.49	9.78	9.90	8.60	5.71	2.02	1.15	16.28	8.57	4.47	10.27	4.00	1.59	2.07	3.53	0.00	8.81	0.71	10.60	7.82	2.90	8.16	4.73	>0.999999999999999	1.7
Q16881	Thioredoxin reductase 1, cytoplasmic OS=Homo sapiens GN=TXNRD1 PE=1 SV=3	17.34	12.91	7.96	4.70	9.59	11.50	7.25	6.82	2.54	0.94	10.60	4.98	4.91	8.66	5.55	2.97	5.84	3.83	1.43	4.79	0.77	7.69	5.67	2.44	8.09	4.55	>0.999999999999999	1.8
Q16851	UTP--glucose-1-phosphate uridylyltransferase OS=Homo sapiens GN=UGP2 PE=1 SV=5	16.12	11.06	7.40	6.07	4.79	17.32	3.04	5.07	3.81	3.34	12.50	6.57	2.81	19.34	6.33	3.93	4.71	10.12	1.90	11.01	2.03	11.84	7.36	3.24	8.09	7.05	>0.999999999999999	1.1
O95994	Anterior gradient protein 2 homolog OS=Homo sapiens GN=AGR2 PE=1 SV=1	14.41	13.60	4.15	2.58	11.32	12.52	6.24	5.87	3.41	0.00	16.28	5.88	1.49	17.32	9.55	4.07	0.00	13.19	5.87	11.14	1.82	3.53	2.61	2.10	8.02	6.06	>0.999999999999999	1.3
P01765	Ig heavy chain V-III region TIL OS=Homo sapiens PE=1 SV=1	6.84	14.52	7.06	4.25	12.08	9.17	4.72	9.99	2.50	6.57	11.93	6.28	2.46	5.64	7.00	4.35	20.36	16.56	9.99	8.16	1.94	13.09	16.56	1.59	7.99	8.98	>0.999999999999999	0.9
P00390	Glutathione reductase, mitochondrial OS=Homo sapiens GN=GSR PE=1 SV=2	18.56	15.21	7.40	5.00	7.67	9.61	5.57	7.61	2.62	1.88	9.09	5.68	4.21	15.31	6.33	3.31	3.39	7.36	3.96	5.18	1.48	9.97	8.74	3.24	7.99	6.04	>0.999999999999999	1.3
O75828	Carbonyl reductase [NADPH] 3 OS=Homo sapiens GN=CBR3 PE=1 SV=3	20.52	11.52	11.54	0.00	6.71	18.19	0.00	5.55	1.98	0.00	9.47	10.26	4.39	13.30	3.89	4.55	0.00	5.37	0.00	10.88	2.03	10.39	0.00	3.75	7.98	4.88	>0.999999999999999	1.6
P12277	Creatine kinase B-type OS=Homo sapiens GN=CKB PE=1 SV=1	13.68	21.90	9.08	8.49	4.79	9.90	9.45	3.96	0.63	0.83	8.52	4.48	4.91	11.28	6.22	2.41	4.71	0.00	0.00	2.07	0.25	5.19	3.83	0.91	7.98	3.48	>0.999999999999999	2.3
Q7Z406	Myosin-14 OS=Homo sapiens GN=MYH14 PE=1 SV=2	15.14	13.83	7.73	5.31	5.95	14.99	5.90	6.34	1.94	0.00	10.41	7.87	3.25	11.08	5.67	3.24	1.70	5.37	0.00	7.38	0.77	5.61	6.44	2.55	7.95	4.42	>0.999999999999999	1.8
P80511	Protein S100-A12 OS=Homo sapiens GN=S100A12 PE=1 SV=2	8.06	17.52	3.70	11.53	14.58	4.80	12.82	0.00	0.00	7.93	14.39	0.00	6.67	0.00	8.44	0.00	25.26	5.06	5.23	4.28	1.02	43.84	11.66	0.00	7.94	9.29	>0.999999999999999	0.9
Q5QNW6	Histone H2B type 2-F OS=Homo sapiens GN=HIST2H2BF PE=1 SV=3	6.11	12.68	6.16	8.34	17.84	8.01	4.22	3.96	0.00	14.72	10.41	2.49	0.00	11.08	6.11	9.72	10.37	0.00	22.35	7.13	0.00	0.00	14.26	0.00	7.91	6.75	>0.999999999999999	1.2
P01714	Ig lambda chain V-III region SH OS=Homo sapiens PE=1 SV=1	0.00	27.43	7.73	4.55	5.75	10.04	20.07	4.76	2.74	3.13	5.68	2.99	2.63	6.04	7.67	4.76	13.01	10.58	4.76	3.89	0.92	6.23	10.58	1.70	7.91	6.06	>0.999999999999999	1.3
P62820	Ras-related protein Rab-1A OS=Homo sapiens GN=RAB1A PE=1 SV=3	12.46	0.00	8.18	0.00	0.00	35.22	0.00	8.08	5.04	0.00	18.56	7.27	4.47	25.58	3.44	6.76	0.00	0.00	0.00	20.73	0.95	15.17	7.82	5.56	7.90	7.54	>0.999999999999999	1.0
P07237	Protein disulfide-isomerase OS=Homo sapiens GN=P4HB PE=1 SV=3	11.72	9.22	9.41	4.85	2.30	15.43	6.75	11.73	4.20	5.84	9.09	3.98	2.81	13.09	4.44	3.86	3.39	8.59	5.07	10.88	2.00	6.65	11.35	2.27	7.88	6.20	>0.999999999999999	1.3
P68363	Tubulin alpha-1B chain OS=Homo sapiens GN=TUBA1B PE=1 SV=1	0.00	0.00	0.00	0.00	36.63	0.00	32.22	15.85	0.00	9.08	0.00	0.00	0.00	31.63	0.00	0.00	21.30	19.33	0.00	32.52	0.00	0.00	0.00	0.00	7.82	8.73	>0.999999999999999	0.9
P0C0L5	Complement C4-B OS=Homo sapiens GN=C4B PE=1 SV=1	8.06	13.14	7.62	19.71	14.00	4.51	11.81	0.00	0.56	7.10	0.00	6.97	4.56	16.52	12.00	6.55	11.12	0.00	18.23	0.00	1.82	25.35	0.00	4.66	7.79	8.40	>0.999999999999999	0.9
P00747	Plasminogen OS=Homo sapiens GN=PLG PE=1 SV=2	10.01	1.61	4.59	19.56	26.27	2.18	8.60	1.11	0.59	4.28	7.76	6.77	6.49	12.49	16.11	7.31	7.73	6.29	20.45	1.94	0.59	9.56	2.30	1.82	7.78	7.76	>0.999999999999999	1.0
Q8N4F0	BPI fold-containing family B member 2 OS=Homo sapiens GN=BPIFB2 PE=1 SV=2	5.13	4.84	14.79	13.80	7.29	4.37	13.33	6.02	3.61	0.00	10.79	9.06	4.12	6.04	14.66	3.93	10.75	4.60	6.02	4.92	3.63	2.91	8.74	5.90	7.75	6.35	>0.999999999999999	1.2
O94760	N(G),N(G)-dimethylarginine dimethylaminohydrolase 1 OS=Homo sapiens GN=DDAH1 PE=1	8.55	14.98	9.30	9.86	6.71	9.46	8.43	5.55	1.39	0.00	12.31	6.47	5.70	13.09	1.22	3.45	0.00	1.69	0.00	8.42	0.68	4.57	3.37	2.84	7.75	3.75	>0.999999999999999	2.1
Q7L266	L-asparaginase OS=Homo sapiens GN=ASRGL1 PE=1 SV=2	19.54	11.06	5.38	3.34	6.52	17.18	3.71	5.39	2.50	0.00	11.93	6.28	2.98	16.12	3.78	9.72	4.15	5.21	1.59	6.22	1.48	13.09	5.21	2.73	7.73	6.02	>0.999999999999999	1.3
P09429	High mobility group protein B1 OS=Homo sapiens GN=HMGB1 PE=1 SV=3	15.88	6.45	9.75	6.98	5.37	4.08	4.72	7.29	4.44	0.00	21.21	6.47	1.14	9.27	0.00	1.93	0.00	13.34	2.06	5.96	0.40	2.70	0.00	0.00	7.72	3.07	>0.999999999999999	2.5
P00492	Hypoxanthine-guanine phosphoribosyltransferase OS=Homo sapiens GN=HPRT1 PE=1 SV	16.12	10.60	5.15	10.01	5.56	9.61	7.76	4.60	2.62	1.46	12.50	6.57	1.23	5.84	3.22	3.17	0.00	7.06	0.00	8.55	0.89	9.56	4.45	3.75	7.71	3.98	>0.999999999999999	1.9
Q13765	Nascent polypeptide-associated complex subunit alpha OS=Homo sapiens GN=NACA PE=1	23.20	16.36	5.49	4.70	5.95	7.13	8.27	7.77	1.23	0.00	9.28	3.09	2.72	9.87	3.44	4.90	0.00	4.75	4.91	9.20	1.51	0.00	7.52	4.03	7.71	4.40	>0.999999999999999	1.7
P01008	Antithrombin-III OS=Homo sapiens GN=SERPINC1 PE=1 SV=1	12.70	8.07	8.07	4.09	8.44	12.08	8.77	4.28	1.74	0.00	13.63	10.56	3.07	10.48	10.44	1.86	8.30	4.14	6.98	4.53	1.36	9.14	5.37	3.52	7.70	5.77	>0.999999999999999	1.3
P25789	Proteasome subunit alpha type-4 OS=Homo sapiens GN=PSMA4 PE=1 SV=1	12.94	8.53	7.84	5.61	7.10	19.50	6.24	1.74	2.78	2.50	10.04	6.97	4.65	7.45	2.67	0.76	2.07	1.69	0.00	3.11	1.14	11.01	5.67	3.97	7.65	3.68	>0.999999999999999	2.1
P08758	Annexin A5 OS=Homo sapiens GN=ANXA5 PE=1 SV=2	17.10	12.68	9.52	2.88	0.00	14.85	1.52	13.47	3.37	0.94	7.95	6.97	1.67	0.00	0.00	0.00	0.00	2.91	0.00	2.46	0.59	0.00	0.00	0.51	7.60	0.68	>0.999999999999999	11.2
P62316	Small nuclear ribonucleoprotein Sm D2 OS=Homo sapiens GN=SNRPD2 PE=1 SV=1	13.68	12.91	2.80	3.79	10.74	13.83	9.45	3.96	0.00	2.61	10.60	5.58	2.19	11.28	0.00	1.72	0.00	3.83	0.00	7.26	0.77	5.19	0.00	1.42	7.50	2.81	>0.999999999999999	2.7
P25398	40S ribosomal protein S12 OS=Homo sapiens GN=RPS12 PE=1 SV=3	12.46	11.76	9.52	7.73	4.41	12.37	0.00	13.47	3.37	0.00	9.66	5.08	2.02	25.79	5.67	3.52	0.00	13.04	3.65	11.01	0.71	4.78	0.00	7.27	7.49	6.45	>0.999999999999999	1.2
Q9UBC9	Small proline-rich protein 3 OS=Homo sapiens GN=SPRR3 PE=1 SV=2	4.40	21.67	7.28	14.25	0.00	9.46	15.86	6.18	1.55	1.88	3.41	3.88	1.58	44.32	19.00	23.86	3.39	19.94	6.18	36.02	2.89	8.10	14.42	7.38	7.48	15.59	>0.999999999999999	0.5
P07384	Calpain-1 catalytic subunit OS=Homo sapiens GN=CAPN1 PE=1 SV=1	16.12	12.45	8.18	6.37	3.26	11.50	4.39	5.87	2.14	1.25	9.09	8.67	1.93	10.88	4.67	3.31	1.51	4.75	0.00	6.22	0.68	7.69	3.99	2.38	7.44	4.00	>0.999999999999999	1.9
P21980	Protein-glutamine gamma-glutamyltransferase 2 OS=Homo sapiens GN=TGM2 PE=1 SV=2	9.53	16.13	2.58	1.97	1.73	21.25	5.57	2.85	4.68	0.42	13.25	8.47	1.58	20.35	0.44	0.90	0.00	7.82	0.63	6.61	1.97	10.60	5.06	4.43	7.37	5.03	>0.999999999999999	1.5
Q8IZP2	Putative protein FAM10A4 OS=Homo sapiens GN=ST13P4 PE=5 SV=1	14.17	13.37	6.50	8.80	7.86	14.41	6.92	9.19	1.63	1.25	0.00	4.08	2.28	8.26	6.44	4.00	4.90	6.29	1.90	7.51	1.26	8.52	1.84	2.33	7.35	4.63	>0.999999999999999	1.6
P01036	Cystatin-S OS=Homo sapiens GN=CST4 PE=1 SV=3	18.32	0.00	17.26	6.82	0.00	2.91	18.72	17.60	4.40	0.00	0.00	1.99	6.58	4.03	0.00	1.38	0.00	11.50	0.00	19.95	2.31	9.35	0.00	2.55	7.34	4.80	>0.999999999999999	1.5
Q9BRA2	Thioredoxin domain-containing protein 17 OS=Homo sapiens GN=TXNDC17 PE=1 SV=1	5.86	5.53	6.05	13.80	0.00	13.25	9.11	0.00	3.61	0.00	17.23	13.55	2.11	18.33	2.67	1.66	0.00	3.68	0.00	7.00	4.19	11.22	0.00	3.07	7.33	4.49	>0.999999999999999	1.6
P12956	X-ray repair cross-complementing protein 6 OS=Homo sapiens GN=XRCC6 PE=1 SV=2	12.46	13.37	6.50	6.67	6.14	13.10	5.40	5.07	1.51	0.00	9.66	8.07	3.86	8.86	4.89	3.03	3.77	5.83	3.17	8.55	0.80	5.40	3.99	3.29	7.33	4.62	>0.999999999999999	1.6
P01034	Cystatin-C OS=Homo sapiens GN=CST3 PE=1 SV=1	11.48	4.84	5.27	11.83	9.01	6.84	13.16	7.45	4.60	2.19	8.90	2.09	4.12	9.47	5.22	3.24	8.86	3.22	3.33	2.72	0.65	9.77	3.22	2.67	7.30	4.71	>0.999999999999999	1.6
P22528	Cornifin-B OS=Homo sapiens GN=SPRR1B PE=1 SV=2	0.00	17.98	8.74	11.83	0.00	4.95	23.28	12.36	1.35	3.55	0.00	3.39	2.98	65.47	0.00	22.42	0.00	21.17	5.39	17.88	4.25	16.21	5.21	4.43	7.29	13.78	>0.999999999999999	0.5
P61158	Actin-related protein 3 OS=Homo sapiens GN=ACTR3 PE=1 SV=3	9.77	13.60	6.61	4.70	7.67	8.59	8.27	4.91	2.78	4.17	11.17	4.88	5.18	8.06	4.44	2.14	5.84	6.14	4.91	7.64	0.95	19.74	10.74	2.78	7.26	6.55	>0.999999999999999	1.1
Q9H4A4	Aminopeptidase B OS=Homo sapiens GN=RNPEP PE=1 SV=2	13.43	12.68	6.95	6.37	3.64	11.21	7.08	3.01	0.56	0.42	13.06	8.47	2.11	8.46	3.33	2.48	0.75	7.36	0.63	6.22	1.11	6.23	3.68	3.12	7.24	3.79	>0.999999999999999	1.9
O43653	Prostate stem cell antigen OS=Homo sapiens GN=PSCA PE=1 SV=1	13.92	13.14	2.80	8.64	10.93	0.00	9.62	9.04	2.26	0.00	10.79	5.68	2.19	11.48	6.33	3.93	10.75	0.00	9.04	7.38	0.77	0.00	3.83	3.24	7.23	4.91	>0.999999999999999	1.5
P36952	Serpin B5 OS=Homo sapiens GN=SERPINB5 PE=1 SV=2	8.55	8.07	9.19	8.64	4.79	13.97	4.22	10.94	3.81	4.70	3.03	6.87	3.95	9.07	5.00	11.38	13.01	6.90	10.94	16.58	3.94	19.95	8.74	5.45	7.23	9.58	>0.999999999999999	0.8
P08865	40S ribosomal protein SA OS=Homo sapiens GN=RPSA PE=1 SV=4	15.14	10.83	8.74	1.52	4.03	11.35	7.93	5.23	3.09	2.19	8.90	7.77	1.84	6.65	8.67	3.24	3.96	7.21	5.23	8.03	1.91	4.36	1.53	4.43	7.23	4.76	>0.999999999999999	1.5
P05783	Keratin, type I cytoskeletal 18 OS=Homo sapiens GN=KRT18 PE=1 SV=2	11.97	11.29	6.61	4.55	5.75	13.68	1.18	3.49	1.19	4.07	17.80	4.88	1.93	2.82	2.44	1.52	2.64	3.37	2.22	0.00	0.68	0.00	2.15	0.79	7.21	1.71	>0.999999999999999	4.2
P13797	Plastin-3 OS=Homo sapiens GN=PLS3 PE=1 SV=4	10.50	11.52	8.96	8.64	0.00	9.32	9.62	3.96	1.98	3.86	10.79	7.17	3.25	12.89	8.00	6.69	9.43	5.67	3.17	10.36	1.76	18.29	9.82	3.24	7.19	7.71	>0.999999999999999	0.9
P48637	Glutathione synthetase OS=Homo sapiens GN=GSS PE=1 SV=1	12.94	14.52	3.14	6.67	2.49	13.83	3.37	6.98	1.74	0.00	13.82	6.28	2.46	8.86	2.22	2.48	0.00	3.07	0.00	4.66	0.62	7.48	4.29	2.50	7.15	3.22	>0.999999999999999	2.2
P62888	60S ribosomal protein L30 OS=Homo sapiens GN=RPL30 PE=1 SV=2	24.91	13.83	2.91	0.00	4.99	14.85	4.39	0.00	2.38	0.00	11.36	5.98	2.28	20.55	0.00	0.00	4.90	3.99	4.12	13.22	0.80	0.00	0.00	1.48	7.13	4.28	>0.999999999999999	1.7
P46779	60S ribosomal protein L28 OS=Homo sapiens GN=RPL28 PE=1 SV=3	11.72	18.21	5.38	7.28	4.22	11.50	3.71	7.61	1.90	2.30	9.09	2.19	1.93	9.67	5.33	1.52	4.15	3.37	3.49	6.22	0.68	4.57	3.37	2.73	7.09	3.92	>0.999999999999999	1.8
Q06210	Glucosamine--fructose-6-phosphate aminotransferase [isomerizing] 1 OS=Homo sapiens GI	12.21	12.91	6.27	6.67	7.29	12.08	5.57	6.02	3.29	0.42	7.20	4.98	1.58	11.28	3.67	3.03	2.45	11.66	0.00	8.94	1.36	5.61	5.06	2.84	7.08	4.79	>0.999999999999999	1.5
Q8N339	Metallothionein-1M OS=Homo sapiens GN=MT1M PE=2 SV=2	11.97	11.29	5.49	7.43	9.40	7.13	8.27	7.77	1.94	0.00	9.28	4.88	0.00	9.87	5.44	3.38	9.24	7.52	7.77	6.35	1.51	10.18	7.52	2.78	7.07	5.96	>0.999999999999999	1.2
P46940	Ras GTPase-activating-like protein IQGAP1 OS=Homo sapiens GN=IQGAP1 PE=1 SV=1	14.17	10.37	5.38	4.70	4.03	10.63	3.88	5.71	3.01	3.76	11.55	7.27	1.84	9.67	4.00	2.35	3.20	2.91	2.06	7.26	1.17	12.68	5.83	2.90	7.04	4.66	>0.999999999999999	1.5
P31025	Lipocalin-1 OS=Homo sapiens GN=LCN1 PE=1 SV=1	9.28	0.00	13.67	5.76	7.29	13.10	10.46	19.34	1.51	3.97	0.00	0.00	3.33	0.00	4.22	2.62	7.16	13.80	0.00	11.66	0.00	25.35	5.83	2.16	7.03	6.35	>0.999999999999999	1.1
P24534	Elongation factor 1-beta OS=Homo sapiens GN=EEF1B2 PE=1 SV=3	11.23	10.60	7.40	6.98	8.82	12.81	4.89	7.29	2.62	0.00	8.71	2.89	4.04	17.73	3.22	4.55	5.47	10.12	2.06	8.55	1.42	9.56	1.99	3.75	7.02	6.04	>0.999999999999999	1.2
P62314	Small nuclear ribonucleoprotein Sm D1 OS=Homo sapiens GN=SNRPD1 PE=1 SV=1	23.94	22.59	0.00	8.80	4.99	8.44	0.00	4.12	1.03	2.71	4.92	2.59	5.09	0.00	0.00	0.00	0.00	0.00	0.00	7.51	0.00	5.40	0.00	0.00	7.01	1.50	>0.999999999999999	4.7
P19338	Nucleolin OS=Homo sapiens GN=NCL PE=1 SV=3	12.70	11.99	5.83	6.98	3.45	10.63	6.75	6.34	1.82	2.40	8.71	5.18	2.54	10.48	3.78	4.07	5.47	5.21	3.65	7.64	0.71	6.03	3.53	1.93	6.90	4.59	>0.999999999999999	1.5
Q03013	Glutathione S-transferase Mu 4 OS=Homo sapiens GN=GSTM4 PE=1 SV=3	15.39	10.14	7.06	1.97	2.49	6.40	2.19	2.06	2.50	2.92	15.90	13.45	2.46	5.64	4.89	0.90	0.00	4.29	2.06	3.63	1.94	0.00	1.99	3.58	6.87	2.61	>0.999999999999999	2.6
P61106	Ras-related protein Rab-14 OS=Homo sapiens GN=RAB14 PE=1 SV=4	11.72	11.06	5.38	4.55	2.68	13.39	5.06	7.61	1.90	5.01	9.09	4.78	0.00	9.67	3.33	3.31	0.00	7.36	2.22	6.22	0.00	9.97	0.00	2.73	6.85	3.73	>0.999999999999999	1.8
P04217	Alpha-1B-glycoprotein OS=Homo sapiens GN=A1BG PE=1 SV=4	2.93	4.38	6.61	11.98	17.26	4.95	7.08	3.01	1.35	2.71	14.96	4.98	3.68	6.85	10.00	4.76	11.12	6.44	9.35	4.41	1.05	12.26	6.44	3.35	6.85	6.64	>0.999999999999999	1.0
Q9Y5Z4	Heme-binding protein 2 OS=Homo sapiens GN=HEBP2 PE=1 SV=1	7.57	11.76	3.47	4.70	5.95	18.49	5.23	4.91	1.23	0.00	13.82	5.08	1.32	6.24	5.67	2.14	2.83	7.82	0.00	1.94	0.95	10.60	7.82	4.14	6.85	4.29	>0.999999999999999	1.6
P61077	Ubiquitin-conjugating enzyme E2 D3 OS=Homo sapiens GN=UBE2D3 PE=1 SV=1	10.75	10.14	2.24	11.07	8.44	6.40	7.42	6.98	2.89	4.59	3.79	7.27	0.00	8.86	4.89	3.03	0.00	0.00	3.17	5.70	1.36	9.14	6.75	2.50	6.83	3.78	>0.999999999999999	1.8
P02749	Beta-2-glycoprotein 1 OS=Homo sapiens GN=APOH PE=1 SV=3	9.28	8.76	3.03	9.40	14.38	2.47	8.27	2.69	1.07	0.00	14.20	7.47	5.44	12.49	6.89	2.62	11.69	5.83	9.83	3.50	1.51	0.00	7.52	2.16	6.75	5.79	>0.999999999999999	1.2
P01621	Ig kappa chain V-III region NG9 (Fragment) OS=Homo sapiens PE=1 SV=1	0.00	17.06	0.00	18.96	0.00	10.77	5.40	11.73	2.85	0.00	14.01	0.00	2.81	0.00	8.22	8.90	24.32	4.91	0.00	4.15	2.28	0.00	0.00	2.90	6.73	4.63	>0.999999999999999	1.5
P36871	Phosphoglucomutase-1 OS=Homo sapiens GN=PGM1 PE=1 SV=3	12.46	13.83	4.15	4.55	4.41	18.78	2.87	3.65	1.74	1.77	5.68	6.77	2.63	10.27	3.33	4.69	2.07	9.20	1.74	7.77	1.14	10.60	2.61	1.70	6.72	4.91	>0.999999999999999	1.4
P49913	Cathelicidin antimicrobial peptide OS=Homo sapiens GN=CAMP PE=1 SV=1	4.15	27.89	0.00	5.61	3.26	2.47	6.24	9.67	0.00	9.29	11.55	0.00	14.04	17.93	0.00	0.00	6.98	0.00	0.00	0.00	0.52	33.25	24.54	0.00	6.68	8.10	>0.999999999999999	0.8
P04434	Ig kappa chain V-III region VH (Fragment) OS=Homo sapiens PE=4 SV=1	0.00	0.00	3.03	9.10	5.18	8.73	17.38	16.33	0.00	6.26	11.36	2.69	0.00	5.44	0.00	4.14	11.31	9.20	4.28	0.00	1.85	0.00	9.20	1.53	6.67	3.91	>0.999999999999999	1.7
P49720	Proteasome subunit beta type-3 OS=Homo sapiens GN=PSMB3 PE=1 SV=2	17.34	16.36	7.96	2.12	5.95	10.33	2.36	2.22	4.96	1.46	5.87	3.09	2.72	2.82	5.55	2.14	0.00	7.67	0.00	6.48	1.54	14.75	4.75	4.03	6.67	4.37	>0.999999999999999	1.5
P39019	40S ribosomal protein S19 OS=Homo sapiens GN=RPS19 PE=1 SV=2	0.00	10.83	2.35	11.68	4.03	16.74	3.54	7.45	0.83	0.00	14.58	7.67	1.84	15.51	5.22	3.24	3.96	7.21	3.33	6.09	0.00	4.36	7.21	1.19	6.64	4.93	>0.999999999999999	1.3
P18085	ADP-ribosylation factor 4 OS=Homo sapiens GN=ARF4 PE=1 SV=3	13.92	8.07	12.66	0.00	0.00	21.40	0.00	2.54	5.83	3.65	0.00	11.26	0.00	0.00	3.89	2.41	0.00	5.37	9.04	7.38	3.48	7.27	2.45	6.42	6.61	3.98	>0.999999999999999	1.7
O60664	Perilipin-3 OS=Homo sapiens GN=PLIN3 PE=1 SV=3	9.77	14.06	6.83	7.58	5.95	7.28	2.36	6.34	1.59	0.73	11.55	4.98	1.23	6.24	1.56	0.97	2.64	2.15	3.49	2.85	0.43	10.39	2.15	1.25	6.58	2.94	>0.999999999999999	2.2
Q9UKK9	ADP-sugar pyrophosphatase OS=Homo sapiens GN=NUDT5 PE=1 SV=1	11.23	10.60	5.15	6.98	8.82	6.70	0.00	10.46	1.82	0.00	12.50	4.58	4.04	5.84	5.11	2.00	2.64	7.06	0.00	5.96	0.43	18.49	4.45	1.65	6.57	4.81	>0.999999999999999	1.4
P05386	60S acidic ribosomal protein P1 OS=Homo sapiens GN=RPLP1 PE=1 SV=1	7.33	6.91	3.36	4.55	5.75	17.03	5.06	4.76	1.19	3.13	12.88	6.77	2.63	13.70	7.55	2.07	12.82	4.60	4.76	3.89	3.60	14.13	10.43	1.70	6.56	6.82	>0.999999999999999	1.0
P30520	Adenylosuccinate synthetase isozyme 2 OS=Homo sapiens GN=ADSS PE=1 SV=3	11.23	4.84	4.15	4.40	10.74	12.81	3.54	4.60	0.83	2.19	10.60	8.77	3.25	9.27	0.78	2.00	2.45	4.45	0.00	7.26	0.40	13.71	1.99	1.65	6.56	3.93	>0.999999999999999	1.7
P04632	Calpain small subunit 1 OS=Homo sapiens GN=CAPNS1 PE=1 SV=1	9.53	17.06	6.27	3.79	4.79	10.77	4.22	6.18	2.93	0.00	7.38	5.58	4.91	5.04	2.78	0.83	2.26	3.83	1.90	5.05	1.72	5.19	3.83	3.18	6.54	3.38	>0.999999999999999	1.9
P49368	T-complex protein 1 subunit gamma OS=Homo sapiens GN=CCT3 PE=1 SV=4	10.75	13.83	6.72	4.55	3.26	14.26	5.06	3.65	1.74	1.15	8.33	5.18	1.49	7.45	1.89	1.59	0.94	2.61	0.00	7.77	0.71	3.53	1.69	2.10	6.54	2.65	>0.999999999999999	2.5
P61586	Transforming protein RhoA OS=Homo sapiens GN=RHOA PE=1 SV=1	0.00	0.00	14.90	8.04	10.16	11.06	0.00	5.23	4.08	0.00	14.39	10.26	6.67	26.79	0.00	0.00	9.99	11.66	0.00	0.00	2.34	0.00	0.00	4.31	6.51	5.15	>0.999999999999999	1.3
Q8N392	Rho GTPase-activating protein 18 OS=Homo sapiens GN=ARHGAP18 PE=1 SV=3	12.94	9.45	5.27	6.22	10.16	8.73	3.21	6.50	0.75	0.00	10.04	4.68	1.67	1.81	0.44	0.97	0.00	0.61	0.00	2.46	0.00	0.83	2.15	0.23	6.50	0.93	>0.999999999999999	7.0
Q96C23	Aldose 1-epimerase OS=Homo sapiens GN=GALM PE=1 SV=1	19.54	9.22	1.01	2.73	9.97	7.57	3.04	2.85	0.36	0.00	9.85	11.16	2.54	1.81	2.00	0.00	0.00	1.38	0.00	3.76	0.28	1.87	6.13	1.65	6.44	1.79	>0.999999999999999	3.6
P60900	Proteasome subunit alpha type-6 OS=Homo sapiens GN=PSMA6 PE=1 SV=1	18.56	17.52	2.80	1.82	4.79	11.06	2.02	3.96	2.26	0.00	4.73	7.57	2.19	8.06	4.44	0.83	2.26	3.83	3.96	7.38	1.76	15.79	8.74	5.51	6.43	5.40	>0.999999999999999	1.2
P62942	Peptidyl-prolyl cis-trans isomerase FKBP1A OS=Homo sapiens GN=FKBP1A PE=1 SV=2	16.12	15.21	7.40	4.40	5.56	4.22	4.89	4.60	2.62	3.03	5.49	2.89	2.54	5.84	3.22	2.00	5.47	4.45	0.00	3.76	0.89	13.71	4.45	1.65	6.37	4.00	>0.999999999999999	1.6
Q9UQ80	Proliferation-associated protein 2G4 OS=Homo sapiens GN=PA2G4 PE=1 SV=3	10.75	7.84	4.93	5.16	4.60	15.43	5.74	5.39	2.14	0.73	8.33	5.38	2.98	10.88	3.78	3.72	1.32	10.12	5.39	10.11	1.05	7.07	1.07	3.07	6.37	5.05	>0.999999999999999	1.3
Q8WUM4	Programmed cell death 6-interacting protein OS=Homo sapiens GN=PDCD6IP PE=1 SV=1	13.19	9.22	4.93	4.70	4.99	8.59	3.04	6.34	1.94	0.73	12.31	5.88	4.30	11.89	3.44	4.48	2.07	4.75	1.74	7.00	1.51	6.44	2.76	1.99	6.32	4.36	>0.999999999999999	1.4
P0C0S8	Histone H2A type 1 OS=Homo sapiens GN=HIST1H2AG PE=1 SV=2	13.19	0.00	2.69	0.00	10.36	0.00	23.28	0.00	0.00	0.00	26.13	0.00	0.00	10.88	6.00	0.00	0.00	0.00	14.43	0.00	0.00	11.22	0.00	0.00	6.30	3.54	>0.999999999999999	1.8
P23381	Tryptophan--tRNA ligase, cytoplasmic OS=Homo sapiens GN=WARS PE=1 SV=2	6.59	9.91	2.24	4.09	3.84	11.94	10.29	3.17	6.34	4.49	9.85	2.69	1.14	8.66	2.22	2.97	2.45	9.36	2.06	3.50	2.86	14.75	10.89	1.14	6.29	5.17	>0.999999999999999	1.2
Q9BTT0	Acidic leucine-rich nuclear phosphoprotein 32 family member E OS=Homo sapiens GN=ANF	8.79	5.30	2.58	5.46	12.85	9.75	3.88	5.71	0.91	2.40	12.69	5.08	2.02	4.63	1.22	0.76	2.07	1.69	1.74	1.43	0.71	7.48	1.69	0.62	6.28	2.17	>0.999999999999999	2.9
P51149	Ras-related protein Rab-7a OS=Homo sapiens GN=RAB7A PE=1 SV=1	11.72	11.06	5.38	4.55	0.00	13.54	2.36	2.22	1.90	7.20	5.68	9.26	6.05	9.67	1.56	2.07	0.00	2.15	2.22	1.81	0.43	24.94	10.58	0.00	6.24	5.12	>0.999999999999999	1.2
Q14651	Plastin-1 OS=Homo sapiens GN=PLS1 PE=1 SV=2	10.75	11.76	6.50	5.61	3.84	10.63	4.39	2.38	2.89	0.00	9.66	6.47	3.86	13.09	3.44	4.00	5.84	6.75	2.38	11.53	0.62	10.60	3.99	2.10	6.24	5.68	>0.999999999999999	1.1
P11766	Alcohol dehydrogenase class-3 OS=Homo sapiens GN=ADH5 PE=1 SV=4	9.04	11.06	6.72	7.28	5.18	10.63	4.55	4.28	2.38	0.83	9.09	3.69	3.25	17.53	4.11	4.14	1.51	4.14	2.69	7.77	0.52	9.97	2.61	4.94	6.23	5.27	>0.999999999999999	1.2
P11216	Glycogen phosphorylase, brain form OS=Homo sapiens GN=PYGB PE=1 SV=5	11.97	8.99	6.61	7.43	4.22	9.32	5.90	2.22	3.21	2.30	6.63	5.88	2.28	12.89	3.89	3.72	4.15	5.98	6.18	12.18	1.82	11.22	3.99	3.07	6.22	5.95	>0.999999999999999	1.0
P78371	T-complex protein 1 subunit beta OS=Homo sapiens GN=CCT2 PE=1 SV=4	11.48	17.06	2.80	7.13	4.79	6.84	4.22	5.07	1.55	0.63	7.38	5.58	4.12	6.45	2.00	2.69	1.13	2.76	0.95	5.05	0.18	8.10	2.76	1.42	6.21	3.14	>0.999999999999999	2.0
P62913	60S ribosomal protein L11 OS=Homo sapiens GN=RPL11 PE=1 SV=2	14.17	13.37	4.03	2.43	6.90	8.44	2.70	5.71	1.43	1.67	10.98	1.59	1.40	7.25	0.00	2.48	0.00	5.52	0.00	7.51	0.00	0.00	0.00	3.29	6.12	2.29	>0.999999999999999	2.7
P13861	cAMP-dependent protein kinase type II-alpha regulatory subunit OS=Homo sapiens GN=PR	15.39	14.52	5.83	3.49	2.88	12.66	2.53	1.11	0.28	0.00	7.95	6.28	1.32	12.69	1.67	1.59	0.00	1.07	1.11	5.44	0.22	3.12	0.00	1.31	6.08	2.46	>0.999999999999999	2.5
P31146	Coronin-1A OS=Homo sapiens GN=CORO1A PE=1 SV=4	6.84	6.45	2.24	12.89	6.90	7.86	4.72	3.17	1.78	7.72	10.22	1.99	5.61	10.88	8.22	1.38	10.18	11.35	5.71	0.78	0.86	22.86	13.04	1.14	6.07	7.67	>0.999999999999999	0.8
P21964	Catechol O-methyltransferase OS=Homo sapiens GN=COMT PE=1 SV=2	8.79	8.30	5.71	5.46	2.11	9.90	6.07	1.74	4.20	0.00	9.66	10.56	3.16	13.70	4.00	2.48	6.79	7.82	5.71	8.81	2.09	14.13	1.69	3.86	6.04	6.19	>0.999999999999999	1.0
P00966	Argininosuccinate synthase OS=Homo sapiens GN=ASS1 PE=1 SV=2	7.57	11.76	5.71	3.49	2.88	14.26	5.23	3.65	3.89	0.00	9.66	4.08	3.60	14.71	5.67	3.52	5.84	6.29	3.65	11.01	1.26	15.17	7.82	4.83	6.01	6.95	>0.999999999999999	0.9
O43399	Tumor protein D54 OS=Homo sapiens GN=TPD52L2 PE=1 SV=2	7.82	11.99	3.59	7.89	2.88	14.70	5.40	5.07	2.97	1.57	2.84	5.18	4.56	20.35	3.56	0.00	6.03	2.30	0.00	9.72	0.99	10.81	4.91	2.95	5.99	5.51	>0.999999999999999	1.1
P34931	Heat shock 70 kDa protein 1-like OS=Homo sapiens GN=HSPA1L PE=1 SV=2	12.21	0.00	5.60	7.58	7.10	9.46	7.42	5.87	1.74	0.00	8.33	6.47	0.00	0.00	0.00	3.45	0.00	0.00	0.00	6.48	0.00	0.00	0.00	2.10	5.98	1.00	>0.999999999999999	6.0
Q15631	Translin OS=Homo sapiens GN=TSN PE=1 SV=1	14.90	14.06	4.82	6.52	0.00	6.26	2.19	0.00	1.71	1.36	15.53	4.28	2.37	8.66	1.44	4.21	0.00	9.36	0.00	3.50	0.00	5.61	4.14	2.44	5.97	3.48	>0.999999999999999	1.7
P08603	Complement factor H OS=Homo sapiens GN=CFH PE=1 SV=4	6.11	5.07	6.72	15.92	13.04	4.08	6.24	1.43	1.11	2.92	5.87	3.09	3.77	10.07	8.00	5.79	10.75	4.29	10.15	2.85	1.32	15.58	5.67	2.27	5.97	6.71	>0.999999999999999	0.9
P04196	Histidine-rich glycoprotein OS=Homo sapiens GN=HRG PE=1 SV=1	9.04	0.00	5.94	13.50	13.42	2.47	8.94	0.00	0.44	3.86	7.01	6.97	4.65	6.04	7.78	3.66	11.50	0.77	8.40	2.20	1.39	12.68	6.90	2.10	5.97	5.67	>0.999999999999999	1.1
P47756	F-actin-capping protein subunit beta OS=Homo sapiens GN=CAPZB PE=1 SV=4	8.55	11.29	3.92	5.31	9.40	9.46	3.71	5.55	1.94	2.30	6.63	3.49	3.07	9.87	3.89	3.38	4.15	1.69	3.49	4.53	0.68	7.27	5.37	2.78	5.96	4.18	>0.999999999999999	1.4
P35237	Serpin B6 OS=Homo sapiens GN=SERPINB6 PE=1 SV=3	10.99	12.91	3.92	3.79	6.71	8.15	4.22	2.54	1.78	0.00	10.60	5.58	3.95	5.04	2.78	2.41	3.02	3.83	2.54	5.83	0.77	3.32	6.90	0.91	5.93	3.44	>0.999999999999999	1.7
P40121	Macrophage-capping protein OS=Homo sapiens GN=CAPG PE=1 SV=2	9.53	6.45	3.14	9.71	3.45	7.42	6.58	4.44	1.55	5.32	9.66	3.88	1.58	3.63	3.11	3.52	5.28	2.76	1.43	6.61	1.20	19.32	11.96	2.21	5.93	5.22	>0.999999999999999	1.1
O15511	Actin-related protein 2/3 complex subunit 5 OS=Homo sapiens GN=ARPC5 PE=1 SV=3	11.23	4.84	5.15	6.98	4.03	6.70	7.76	3.33	3.01	4.80	8.71	4.58	4.04	9.27	2.33	3.17	0.00	11.66	0.00	5.96	1.42	9.56	11.66	4.31	5.93	5.28	>0.999999999999999	1.1
P21291	Cysteine and glycine-rich protein 1 OS=Homo sapiens GN=CSRP1 PE=1 SV=3	13.43	12.68	8.85	2.43	0.00	15.57	2.70	0.00	1.35	0.00	3.03	10.66	2.98	15.91	1.78	2.35	0.00	5.21	0.00	7.13	0.49	0.00	0.00	6.07	5.89	3.49	>0.999999999999999	1.7
Q12792	Twinfilin-1 OS=Homo sapiens GN=TWF1 PE=1 SV=3	11.72	11.06	6.72	5.61	3.26	12.81	2.87	2.69	1.47	1.77	7.01	3.69	2.37	7.45	5.33	2.55	1.51	5.67	2.69	6.22	0.52	1.66	1.23	2.73	5.89	3.33	>0.999999999999999	1.8
Q14914	Prostaglandin reductase 1 OS=Homo sapiens GN=PTGR1 PE=1 SV=2	7.33	6.91	9.52	2.88	8.05	14.85	3.21	3.01	1.19	0.00	7.95	5.48	1.67	6.04	2.11	2.90	1.70	1.38	1.43	5.44	0.92	1.87	1.38	3.12	5.87	2.50	>0.999999999999999	2.3
P07108	Acyl-CoA-binding protein OS=Homo sapiens GN=DBI PE=1 SV=2	8.55	18.90	9.19	5.31	0.00	5.09	0.00	5.55	3.25	0.00	0.00	14.44	0.00	16.52	3.89	10.00	0.00	5.37	0.00	0.00	1.08	0.00	0.00	1.99	5.86	3.24	>0.999999999999999	1.8
P40926	Malate dehydrogenase, mitochondrial OS=Homo sapiens GN=MDH2 PE=1 SV=3	7.33	9.68	4.71	2.88	0.00	17.61	3.21	19.18	1.67	1.98	0.00	1.89	2.63	6.04	3.33	0.62	1.70	4.60	3.01	2.46	0.00	1.87	2.91	1.08	5.84	2.52	>0.999999999999999	2.3
P12081	Histidine--tRNA ligase, cytoplasmic OS=Homo sapiens GN=HARS PE=1 SV=2	13.68	7.38	5.27	1.82	4.79	17.03	2.02	3.96	1.86	0.63	6.06	5.58	1.05	6.45	3.56	1.24	1.13	2.76	0.00	4.15	0.37	3.74	2.76	2.67	5.84	2.49	>0.999999999999999	2.3
P01042	Kininogen-1 OS=Homo sapiens GN=KNG1 PE=1 SV=2	4.64	4.38	4.71	9.40	10.55	5.24	8.27	6.66	1.19	4.38	6.82	3.59	4.82	11.08	4.00	2.90	10.37	4.60	9.83	4.66	0.77	11.43	6.44	2.04	5.82	6.08	>0.999999999999999	1.0
P61247	40S ribosomal protein S3a OS=Homo sapiens GN=RPS3A PE=1 SV=2	12.70	5.30	5.83	5.61	2.11	12.66	0.00	1.74	0.44	0.00	16.47	6.87	0.96	10.48	1.22	1.59	0.00	1.69	3.65	2.98	0.00	2.29	1.69	2.10	5.81	2.39	>0.999999999999999	2.4
P07360	Complement component C8 gamma chain OS=Homo sapiens GN=C8G PE=1 SV=3	0.00	0.00	8.40	25.02	14.38	0.00	8.77	0.00	0.00	5.43	6.06	1.49	1.32	20.35	8.33	5.17	14.14	2.30	8.24	4.15	0.46	15.58	0.00	2.95	5.80	6.92	>0.999999999999999	0.8
P54108	Cysteine-rich secretory protein 3 OS=Homo sapiens GN=CRISP3 PE=1 SV=1	9.53	8.99	4.37	3.79	7.48	3.64	9.45	6.18	1.55	5.84	7.38	1.20	2.19	7.86	2.78	2.69	10.56	5.98	6.18	5.05	1.20	8.10	5.98	1.42	5.78	5.00	>0.999999999999999	1.2
Q9GZZ8	Extracellular glycoprotein lacritin OS=Homo sapiens GN=LACRT PE=1 SV=1	13.19	0.00	6.05	0.00	0.00	13.25	15.35	14.43	2.14	2.50	0.00	2.39	4.74	4.83	10.11	1.66	0.00	13.96	0.00	11.79	0.68	18.91	0.00	3.07	5.77	5.81	>0.999999999999999	1.0
P48643	T-complex protein 1 subunit epsilon OS=Homo sapiens GN=CCT5 PE=1 SV=1	10.99	7.15	6.95	4.70	4.60	14.56	4.05	0.79	2.10	0.00	7.20	6.18	2.72	6.24	2.67	2.62	0.00	5.83	1.74	5.83	1.17	4.99	1.69	1.36	5.77	3.07	>0.999999999999999	1.9
P18206	Vinculin OS=Homo sapiens GN=VCL PE=1 SV=4	8.06	11.06	4.48	5.61	1.53	10.48	3.37	6.34	2.18	4.59	6.25	5.08	1.49	12.09	4.44	4.69	1.51	5.06	4.76	9.33	1.23	17.04	7.36	3.86	5.75	6.07	>0.999999999999999	0.9
P60842	Eukaryotic initiation factor 4A-I OS=Homo sapiens GN=EIF4A1 PE=1 SV=1	17.83	5.30	5.71	3.49	0.00	10.63	0.00	6.50	2.46	0.00	9.66	7.27	0.00	0.00	5.67	3.52	0.00	7.82	0.00	6.61	1.26	6.65	6.29	1.82	5.74	3.30	>0.999999999999999	1.7
P46781	40S ribosomal protein S9 OS=Homo sapiens GN=RPS9 PE=1 SV=3	12.46	7.38	8.29	2.27	2.88	10.77	5.40	2.38	2.02	1.57	6.06	7.37	2.81	10.27	3.56	2.21	2.83	11.35	0.00	9.59	0.99	3.32	0.00	2.90	5.74	4.15	>0.999999999999999	1.4
Q86VP6	Cullin-associated NEDD8-dissociated protein 1 OS=Homo sapiens GN=CAND1 PE=1 SV=2	10.99	9.68	4.71	4.85	4.03	10.33	3.04	1.90	2.06	0.21	9.85	7.07	1.05	9.87	2.89	2.48	2.26	4.91	0.79	6.74	0.99	6.65	1.53	2.38	5.73	3.55	>0.999999999999999	1.6
Q96C86	Scavenger mRNA-decapping enzyme DcpS OS=Homo sapiens GN=DCPS PE=1 SV=2	15.63	14.75	2.02	7.73	5.37	4.08	1.52	0.00	1.55	0.00	9.66	6.38	0.00	1.81	0.00	1.24	0.00	1.38	0.00	5.05	0.55	5.82	1.38	2.21	5.72	1.62	>0.999999999999999	3.5
Q14240	Eukaryotic initiation factor 4A-II OS=Homo sapiens GN=EIF4A2 PE=1 SV=2	20.76	0.00	5.71	3.49	0.00	7.42	6.92	4.91	0.00	1.57	11.55	6.08	3.60	6.24	0.00	0.00	1.32	0.00	2.38	5.31	0.00	0.00	0.00	1.76	5.70	1.72	>0.999999999999999	3.3
Q96NY7	Chloride intracellular channel protein 6 OS=Homo sapiens GN=CLIC6 PE=2 SV=3	22.71	12.68	2.13	2.12	2.68	6.99	5.06	2.22	0.99	0.00	9.09	1.39	1.67	3.83	0.44	0.28	0.75	0.00	0.00	1.17	0.12	0.00	0.61	0.00	5.67	0.74	>0.999999999999999	7.7
Q9Y266	Nuclear migration protein nudC OS=Homo sapiens GN=NUDC PE=1 SV=1	6.84	11.76	3.14	4.25	5.37	11.50	8.60	2.85	1.55	0.94	7.38	3.88	3.42	7.86	4.33	2.69	7.35	4.29	2.85	5.05	0.86	8.10	5.98	1.59	5.67	4.53	>0.999999999999999	1.3
P06748	Nucleophosmin OS=Homo sapiens GN=NPM1 PE=1 SV=2	15.14	10.83	3.81	7.13	1.92	6.84	3.54	1.59	0.83	1.04	11.74	3.39	1.84	6.85	1.11	2.35	1.89	5.21	1.59	2.72	0.31	2.08	5.21	2.67	5.65	2.82	>0.999999999999999	2.0
P23526	Adenosylhomocysteinase OS=Homo sapiens GN=AHCY PE=1 SV=4	9.53	11.29	6.61	4.55	4.22	11.79	3.71	2.22	2.34	0.00	7.38	3.88	0.61	9.87	3.33	2.69	0.00	4.60	0.00	5.05	1.82	8.10	4.60	2.78	5.63	3.62	>0.999999999999999	1.6
Q9Y547	Heat shock protein beta-11 OS=Homo sapiens GN=HSPB11 PE=1 SV=1	5.13	10.37	5.04	3.18	8.63	6.55	3.54	3.33	0.00	0.00	14.20	7.47	6.58	9.07	5.00	3.10	0.00	0.00	0.00	9.72	0.65	0.00	3.22	2.55	5.62	3.32	>0.999999999999999	1.7
P50224	Sulfotransferase 1A3/1A4 OS=Homo sapiens GN=SULT1A3 PE=1 SV=1	18.07	10.37	5.04	3.03	3.84	6.55	3.37	5.07	1.78	0.00	3.79	5.88	0.00	4.03	2.22	2.21	0.00	4.91	0.00	5.83	0.31	0.00	0.00	1.14	5.57	1.72	>0.999999999999999	3.2
P15121	Aldose reductase OS=Homo sapiens GN=AKR1B1 PE=1 SV=3	16.85	12.68	7.73	6.37	3.64	6.11	0.00	3.01	1.19	0.00	3.60	5.48	2.63	13.90	2.11	7.04	0.00	4.60	0.00	3.89	0.00	1.87	4.60	1.08	5.56	3.48	>0.999999999999999	1.6
P62195	26S protease regulatory subunit 8 OS=Homo sapiens GN=PSMC5 PE=1 SV=1	10.26	9.68	3.59	3.49	6.14	10.92	5.40	3.65	1.67	0.00	4.35	7.47	1.32	8.46	2.56	2.21	0.00	3.53	0.00	6.74	0.46	4.78	2.30	2.38	5.55	2.89	>0.999999999999999	1.9
P62280	40S ribosomal protein S11 OS=Homo sapiens GN=RPS11 PE=1 SV=3	9.77	14.98	7.28	6.07	7.67	5.82	0.00	2.85	2.58	0.00	7.57	1.79	1.58	3.63	0.00	1.24	0.00	2.76	2.85	5.18	0.55	0.00	0.00	2.27	5.53	1.67	>0.999999999999999	3.3
P52565	Rho GDP-dissociation inhibitor 1 OS=Homo sapiens GN=ARHGDIA PE=1 SV=3	12.21	11.52	3.47	4.70	5.95	4.51	2.36	7.93	1.23	3.24	5.87	3.09	2.72	14.30	3.44	3.45	5.84	7.67	4.91	4.02	0.95	14.75	4.75	2.84	5.51	5.80	>0.999999999999999	0.9
P01781	Ig heavy chain V-III region GAL OS=Homo sapiens PE=1 SV=1	6.59	0.00	3.03	9.10	19.75	3.93	4.55	4.28	4.08	2.82	5.11	2.69	2.37	5.44	3.00	1.86	11.31	4.14	4.28	7.77	0.83	5.61	4.14	1.53	5.50	4.36	>0.999999999999999	1.3
P01019	Angiotensinogen OS=Homo sapiens GN=AGT PE=1 SV=1	8.55	6.22	4.82	5.31	11.70	5.09	3.37	3.17	1.39	2.09	9.85	4.28	1.75	8.66	5.78	2.97	9.80	3.07	9.67	4.53	1.32	5.61	5.37	2.44	5.49	5.08	>0.999999999999999	1.1
P07910	Heterogeneous nuclear ribonucleoproteins C1/C2 OS=Homo sapiens GN=HNRNPC PE=1 S	5.13	7.38	5.15	4.85	6.14	6.70	5.40	5.07	1.82	2.19	11.36	4.58	2.81	4.23	3.56	2.21	1.89	4.91	0.00	4.15	0.99	4.36	4.91	1.82	5.48	2.98	>0.999999999999999	1.8
O14950	Myosin regulatory light chain 12B OS=Homo sapiens GN=MYL12B PE=1 SV=2	4.15	8.53	6.72	5.61	3.26	12.66	2.87	2.69	2.38	3.86	7.01	5.98	3.25	7.45	4.11	4.14	3.20	5.67	2.69	7.77	0.52	18.08	5.67	3.41	5.48	5.50	>0.999999999999999	1.0
Q14847	LIM and SH3 domain protein 1 OS=Homo sapiens GN=LASP1 PE=1 SV=2	12.70	11.99	4.15	3.49	2.11	7.57	3.88	8.24	2.06	0.00	4.35	5.18	2.02	10.48	5.78	2.55	2.07	7.98	1.74	2.98	0.71	4.78	3.53	2.95	5.48	3.96	>0.999999999999999	1.4
P27695	DNA-(apurinic or apyrimidinic site) lyase OS=Homo sapiens GN=APEX1 PE=1 SV=2	13.43	2.07	2.13	6.37	3.64	12.37	3.21	4.76	0.75	1.98	10.41	4.18	0.00	6.04	2.11	1.31	0.00	1.38	0.00	3.89	0.28	1.87	4.60	1.08	5.44	1.88	>0.999999999999999	2.9
Q14011	Cold-inducible RNA-binding protein OS=Homo sapiens GN=CIRBP PE=1 SV=1	9.53	8.99	4.37	5.91	7.48	9.46	0.00	0.00	0.71	0.00	12.31	6.47	3.42	3.63	2.00	1.24	3.39	0.00	2.85	5.05	0.55	0.00	0.00	2.21	5.44	2.03	>0.999999999999999	2.7
P62277	40S ribosomal protein S13 OS=Homo sapiens GN=RPS13 PE=1 SV=2	10.50	9.91	4.82	6.52	3.84	6.26	0.00	11.26	0.79	0.00	3.79	7.07	0.00	4.03	2.22	1.38	0.00	0.00	0.00	13.60	1.32	8.94	3.07	2.44	5.40	3.08	>0.999999999999999	1.8
P07203	Glutathione peroxidase 1 OS=Homo sapiens GN=GPX1 PE=1 SV=4	24.67	11.99	3.59	0.00	0.00	4.66	0.00	2.38	1.27	0.00	2.84	13.15	1.32	6.45	1.67	0.00	0.00	4.91	2.38	4.15	0.46	10.81	0.00	2.95	5.38	2.92	>0.999999999999999	1.8
Q9UBQ7	Glyoxylate reductase/hydroxypyruvate reductase OS=Homo sapiens GN=GRHPR PE=1 SV	10.26	9.68	4.71	2.88	1.73	12.37	3.21	6.66	1.67	0.00	5.68	5.48	0.79	11.08	1.00	2.07	0.00	1.38	1.43	5.44	0.92	1.87	2.91	1.70	5.36	2.55	>0.999999999999999	2.1
Q9UJ70	N-acetyl-D-glucosamine kinase OS=Homo sapiens GN=NAGK PE=1 SV=4	16.12	2.07	4.48	1.36	5.56	7.57	1.52	4.60	2.06	0.94	9.85	7.97	2.54	8.06	3.22	2.76	3.39	7.98	0.00	5.18	0.55	8.31	6.13	2.95	5.34	4.26	>0.999999999999999	1.3
P02748	Complement component C9 OS=Homo sapiens GN=C9 PE=1 SV=2	8.55	6.45	7.17	8.49	10.74	2.33	2.70	1.59	1.39	1.04	7.95	5.58	3.68	12.89	11.11	3.38	10.56	3.37	8.88	3.63	0.86	4.57	7.52	2.78	5.33	6.10	>0.999999999999999	0.9
P0CW22	40S ribosomal protein S17-like OS=Homo sapiens GN=RPS17L PE=3 SV=1	19.54	11.06	8.96	3.34	4.22	6.99	0.00	3.49	0.00	0.00	4.17	2.19	0.00	4.43	0.00	1.52	0.00	3.37	0.00	2.85	0.00	0.00	0.00	1.25	5.33	1.12	>0.999999999999999	4.8
Q14624	Inter-alpha-trypsin inhibitor heavy chain H4 OS=Homo sapiens GN=ITIH4 PE=1 SV=4	10.01	6.45	3.14	7.58	10.55	1.46	8.43	2.06	1.27	2.19	6.06	4.48	3.95	10.07	6.11	2.83	8.48	3.68	10.30	2.20	1.39	6.65	4.29	2.04	5.31	5.17	>0.999999999999999	1.0
P67936	Tropomyosin alpha-4 chain OS=Homo sapiens GN=TPM4 PE=1 SV=3	6.11	12.68	4.37	1.82	2.30	16.88	4.22	0.00	2.18	0.00	7.38	5.48	2.19	0.00	2.78	3.79	0.00	5.98	8.72	9.46	0.77	29.30	3.83	4.14	5.28	5.91	>0.999999999999999	0.9
O43776	Asparagine--tRNA ligase, cytoplasmic OS=Homo sapiens GN=NARS PE=1 SV=1	13.92	8.07	4.71	2.43	3.07	8.30	2.70	1.74	1.98	0.00	12.31	4.18	0.96	4.43	2.44	2.00	0.94	2.45	1.74	8.42	0.68	6.03	3.37	1.99	5.28	2.96	>0.999999999999999	1.8
P13647	Keratin, type II cytoskeletal 5 OS=Homo sapiens GN=KRT5 PE=1 SV=3	0.00	9.91	1.90	4.40	2.11	4.22	8.60	2.69	0.00	29.01	0.00	0.50	0.00	11.89	4.78	1.59	0.00	0.00	15.06	2.20	0.52	6.03	0.00	0.97	5.28	3.59	>0.999999999999999	1.5
Q9BW83	Intraflagellar transport protein 27 homolog OS=Homo sapiens GN=IFT27 PE=1 SV=1	13.92	3.69	3.92	5.31	3.07	11.94	5.90	2.54	0.63	0.00	6.63	5.68	1.40	7.05	1.78	1.10	0.00	2.45	0.00	4.53	0.00	0.00	0.00	1.99	5.27	1.69	>0.999999999999999	3.1
Q9H1X1	Radial spoke head protein 9 homolog OS=Homo sapiens GN=RSPH9 PE=1 SV=1	12.21	8.07	2.47	0.00	2.11	12.08	0.00	5.55	0.00	0.00	15.72	4.98	0.00	0.00	0.00	0.00	0.00	0.00	0.00	0.00	0.00	0.00	0.00	0.00	5.26	0.00	>0.999999999999999	#DIV/0!
P61006	Ras-related protein Rab-8A OS=Homo sapiens GN=RAB8A PE=1 SV=1	11.72	0.00	5.38	10.46	0.00	6.99	0.00	4.76	2.74	3.13	13.06	4.78	2.63	9.67	0.00	3.31	0.00	7.36	0.00	8.94	0.43	9.97	7.36	0.00	5.25	4.14	>0.999999999999999	1.3
Q9HC38	Glyoxalase domain-containing protein 4 OS=Homo sapiens GN=GLOD4 PE=1 SV=1	10.50	9.91	8.07	6.52	1.73	8.30	1.52	4.91	1.23	0.00	5.87	4.28	1.75	11.48	2.22	3.93	0.00	1.38	1.43	5.57	0.00	4.16	3.07	2.44	5.24	3.12	>0.999999999999999	1.7
Q15370	Transcription elongation factor B polypeptide 2 OS=Homo sapiens GN=TCEB2 PE=1 SV=1	14.17	13.37	6.50	0.00	0.00	8.44	4.39	4.12	1.03	0.00	4.92	5.78	5.09	5.24	2.89	4.00	0.00	0.00	0.00	7.51	0.00	0.00	0.00	1.48	5.23	2.18	>0.999999999999999	2.4
P62857	40S ribosomal protein S28 OS=Homo sapiens GN=RPS28 PE=1 SV=1	0.00	10.37	0.00	6.82	8.63	6.55	7.59	7.13	1.78	4.70	8.52	0.00	3.95	0.00	5.00	0.00	8.48	6.90	7.13	5.83	0.00	9.35	6.90	2.55	5.18	4.68	>0.999999999999999	1.1
P35268	60S ribosomal protein L22 OS=Homo sapiens GN=RPL22 PE=1 SV=2	12.46	11.76	2.58	3.49	0.00	12.37	0.00	8.08	2.02	2.40	4.35	2.29	0.00	10.27	2.56	3.52	0.00	13.04	0.00	6.61	0.71	0.00	13.04	2.90	5.15	4.39	>0.999999999999999	1.2
P30153	Serine/threonine-protein phosphatase 2A 65 kDa regulatory subunit A alpha isoform OS=Ho	6.59	9.22	4.48	4.09	6.52	6.84	5.74	1.59	1.86	1.04	8.90	4.68	1.40	6.85	5.22	2.35	3.96	5.21	2.54	3.50	0.83	7.07	4.14	1.93	5.13	3.75	>0.999999999999999	1.4
O75556	Mammaglobin-B OS=Homo sapiens GN=SCGB2A1 PE=1 SV=1	7.57	0.00	14.01	0.00	5.95	4.51	5.23	19.82	1.23	3.24	0.00	0.00	2.72	0.00	0.00	0.00	5.84	4.75	4.91	4.02	0.00	25.97	0.00	1.76	5.13	4.17	>0.999999999999999	1.2
Q04760	Lactoylglutathione lyase OS=Homo sapiens GN=GLO1 PE=1 SV=4	8.55	12.91	1.79	2.43	3.07	2.33	5.90	5.55	1.39	0.00	6.63	10.96	3.07	11.28	1.78	0.00	0.00	0.00	0.00	7.26	0.49	7.27	0.00	0.91	5.12	2.67	>0.999999999999999	1.9
Q9BS40	Latexin OS=Homo sapiens GN=LXN PE=1 SV=2	10.75	14.52	3.03	4.09	2.49	9.17	4.55	4.28	1.74	0.00	2.46	4.38	2.37	12.69	0.00	3.03	0.00	4.14	0.00	5.70	1.36	2.70	4.14	1.53	5.12	3.14	>0.999999999999999	1.6
P07858	Cathepsin B OS=Homo sapiens GN=CTSB PE=1 SV=3	6.84	14.75	3.14	4.25	1.73	9.32	1.52	8.08	2.54	1.88	3.41	3.88	5.61	10.27	3.11	6.41	7.35	7.82	4.44	8.29	0.55	5.82	4.29	4.43	5.11	5.70	>0.999999999999999	0.9
Q92598	Heat shock protein 105 kDa OS=Homo sapiens GN=HSPH1 PE=1 SV=1	9.53	8.99	4.37	3.94	1.92	12.66	6.58	2.22	1.19	0.31	5.68	3.88	1.23	2.82	1.11	0.97	1.89	1.53	0.48	2.85	0.00	2.91	2.15	0.79	5.11	1.56	>0.999999999999999	3.3
P47929	Galectin-7 OS=Homo sapiens GN=LGALS7 PE=1 SV=2	20.52	0.00	9.41	3.49	0.00	0.00	8.43	0.00	1.98	13.05	4.35	0.00	0.00	0.00	2.56	3.45	4.34	3.53	19.82	6.48	5.42	4.78	0.00	1.31	5.10	4.31	>0.999999999999999	1.2
Q99584	Protein S100-A13 OS=Homo sapiens GN=S100A13 PE=1 SV=1	7.33	6.91	3.36	4.55	5.75	10.04	11.64	4.76	1.19	0.00	5.68	0.00	2.63	6.04	3.33	2.07	5.66	4.60	4.76	3.89	0.92	14.34	4.60	1.70	5.10	4.55	>0.999999999999999	1.1
P23284	Peptidyl-prolyl cis-trans isomerase B OS=Homo sapiens GN=PPIB PE=1 SV=2	3.42	6.91	3.36	4.55	5.75	6.99	5.06	7.61	1.19	7.20	5.68	2.99	4.21	6.04	1.56	2.07	9.05	7.36	7.61	3.89	2.12	6.23	7.36	1.70	5.06	4.93	>0.999999999999999	1.0

P02656	Apolipoprotein C-III OS=Homo sapiens GN=APOC3 PE=1 SV=1	0.00	0.00	3.59	19.56	6.14	0.00	12.48	5.07	1.27	3.34	6.06	3.19	2.81	0.00	8.22	0.00	6.03	4.91	31.86	0.00	2.28	6.65	11.35	1.82	5.06	6.33	>0.999999999999999	0.8
P35527	Keratin, type I cytoskeletal 9 OS=Homo sapiens GN=KRT9 PE=1 SV=3	2.69	5.30	0.56	4.40	6.90	9.75	7.25	6.82	1.43	4.49	6.82	4.28	3.16	2.22	3.22	4.07	4.34	13.04	5.71	0.65	0.52	2.29	5.52	3.35	5.06	4.01	>0.999999999999999	1.3
P05452	Tetranectin OS=Homo sapiens GN=CLEC3B PE=1 SV=3	17.83	0.00	3.47	2.27	18.79	2.18	5.23	0.00	1.23	1.57	2.84	5.08	1.32	3.02	3.44	2.14	13.76	0.00	8.08	0.00	0.00	3.12	2.30	0.85	5.04	3.17	>0.999999999999999	1.6
P20810	Calpastatin OS=Homo sapiens GN=CAST PE=1 SV=4	12.70	11.99	5.15	1.97	4.41	4.08	3.88	5.39	0.91	0.00	5.30	4.58	1.58	3.63	2.00	1.24	1.70	3.53	0.63	4.41	1.05	2.70	1.38	1.31	5.03	2.10	>0.999999999999999	2.4
P62861	40S ribosomal protein S30 OS=Homo sapiens GN=FAU PE=1 SV=1	13.19	0.00	6.05	8.19	10.36	7.86	9.11	0.00	0.00	0.00	0.00	5.38	0.00	0.00	0.00	0.00	0.00	8.28	8.56	7.00	1.66	0.00	0.00	3.07	5.01	2.38	>0.999999999999999	2.1
P26641	Elongation factor 1-gamma OS=Homo sapiens GN=EEF1G PE=1 SV=3	13.68	6.68	6.27	2.12	2.68	8.15	3.54	1.11	1.82	1.46	8.71	3.69	2.54	11.28	3.22	4.55	2.64	5.67	1.11	9.98	1.42	9.56	3.22	2.61	4.99	4.82	>0.999999999999999	1.0
Q15121	Astrocytic phosphoprotein PEA-15 OS=Homo sapiens GN=PEA15 PE=1 SV=2	12.21	11.52	5.60	3.49	4.41	7.28	3.88	3.65	0.91	0.00	4.35	2.29	2.02	10.07	2.56	1.59	0.00	3.53	0.00	6.48	0.00	4.78	0.00	1.31	4.97	2.69	>0.999999999999999	1.8
P59665	Neutrophil defensin 1 OS=Homo sapiens GN=DEFA1 PE=1 SV=1	8.06	7.61	0.00	11.53	6.33	0.00	12.82	5.23	0.00	7.93	0.00	0.00	6.67	0.00	0.00	0.00	6.22	0.00	0.00	0.00	0.00	6.86	11.66	0.00	4.96	2.62	>0.999999999999999	1.9
Q13296	Mammaglobin-A OS=Homo sapiens GN=SCGB2A2 PE=1 SV=1	0.00	17.06	0.00	0.00	14.19	0.00	12.48	0.00	2.93	3.34	6.06	3.19	0.00	0.00	0.00	2.21	13.95	0.00	5.07	4.15	0.99	0.00	0.00	1.82	4.94	2.35	>0.999999999999999	2.1
P80303	Nucleobindin-2 OS=Homo sapiens GN=NUCB2 PE=1 SV=2	5.13	1.61	6.27	5.61	7.10	4.22	9.45	10.46	3.05	0.00	2.65	3.69	4.91	2.82	2.33	3.17	10.56	7.06	5.87	2.72	2.03	6.03	5.67	1.65	4.94	4.57	>0.999999999999999	1.1
Q07020	60S ribosomal protein L18 OS=Homo sapiens GN=RPL18 PE=1 SV=2	13.19	7.61	1.68	2.27	2.88	11.21	2.53	2.38	0.00	1.57	6.25	7.67	1.32	3.02	1.67	1.03	2.83	8.28	5.23	7.00	1.66	3.12	2.30	4.37	4.94	3.49	>0.999999999999999	1.4
P62263	40S ribosomal protein S14 OS=Homo sapiens GN=RPS14 PE=1 SV=3	10.99	4.84	5.04	6.82	4.03	6.55	3.54	3.33	0.83	0.00	8.52	4.48	1.84	4.23	2.33	3.10	3.96	3.22	0.00	5.83	0.65	4.36	3.22	1.19	4.92	2.83	>0.999999999999999	1.7
P35908	Keratin, type II cytoskeletal 2 epidermal OS=Homo sapiens GN=KRT2 PE=1 SV=2	5.37	9.45	0.00	6.22	1.92	10.33	6.92	3.49	0.87	8.35	4.17	1.59	2.46	0.00	3.11	2.35	3.02	5.21	8.72	0.65	0.00	0.00	4.29	2.33	4.89	2.68	>0.999999999999999	1.8
P63000	Ras-related C3 botulinum toxin substrate 1 OS=Homo sapiens GN=RAC1 PE=1 SV=1	12.94	12.22	0.00	0.00	0.00	11.21	0.00	0.00	0.00	0.00	14.58	7.67	0.00	10.68	5.89	3.66	0.00	8.13	0.00	6.87	0.00	0.00	0.00	0.00	4.88	2.93	>0.999999999999999	1.7
P15374	Ubiquitin carboxyl-terminal hydrolase isozyme L3 OS=Homo sapiens GN=UCHL3 PE=1 SV=	6.59	9.91	4.82	4.09	2.49	8.88	2.19	2.06	1.71	0.00	11.55	4.28	1.14	8.66	1.44	4.21	0.00	6.60	0.00	3.50	0.83	5.61	0.00	2.44	4.88	2.87	>0.999999999999999	1.7
P07339	Cathepsin D OS=Homo sapiens GN=CTSD PE=1 SV=1	3.66	14.75	7.17	5.00	0.00	9.32	2.53	5.23	3.01	0.73	2.84	4.18	1.32	8.46	7.11	9.24	1.32	5.06	1.11	9.85	1.63	8.73	1.07	4.31	4.87	4.93	>0.999999999999999	1.0
O94811	Tubulin polymerization-promoting protein OS=Homo sapiens GN=TPPP PE=1 SV=1	22.71	11.06	1.57	2.12	2.68	4.37	5.06	4.76	0.00	0.00	2.65	1.39	1.23	2.82	1.56	0.00	0.00	0.00	0.00	0.00	0.00	0.00	2.15	0.00	4.87	0.65	>0.999999999999999	7.5
O43175	D-3-phosphoglycerate dehydrogenase OS=Homo sapiens GN=PHGDH PE=1 SV=4	13.68	5.76	4.48	2.73	4.79	5.82	3.04	2.85	0.48	0.00	6.06	8.47	2.19	1.21	1.33	0.00	0.00	0.00	0.00	1.55	0.18	0.00	0.00	0.34	4.85	0.57	>0.999999999999999	8.5
Q12905	Interleukin enhancer-binding factor 2 OS=Homo sapiens GN=ILF2 PE=1 SV=2	8.30	5.76	4.93	5.16	4.79	6.40	2.70	2.54	0.63	0.83	12.69	3.39	0.70	6.85	1.78	1.72	1.51	2.45	0.00	4.41	0.25	3.32	3.83	0.91	4.84	2.31	>0.999999999999999	2.1
P06737	Glycogen phosphorylase, liver form OS=Homo sapiens GN=PYGL PE=1 SV=4	6.35	5.99	3.36	5.31	5.75	5.09	6.58	2.22	1.55	3.13	8.33	4.38	2.28	7.05	2.89	1.52	1.89	1.53	1.11	3.37	0.92	16.83	6.75	1.48	4.84	3.97	>0.999999999999999	1.2
P27482	Calmodulin-like protein 3 OS=Homo sapiens GN=CALML3 PE=1 SV=2	4.88	10.14	2.24	6.67	0.00	2.91	0.00	11.41	1.74	7.51	8.33	1.99	0.00	8.86	4.89	3.03	0.00	6.75	0.00	5.70	0.00	4.16	0.00	2.50	4.82	2.99	>0.999999999999999	1.6
O14745	Na(+)/H(+) exchange regulatory cofactor NHE-RF1 OS=Homo sapiens GN=SLC9A3R1 PE=	4.40	8.76	3.14	2.73	5.37	7.28	4.72	7.93	1.51	2.92	5.30	3.79	2.46	7.65	3.11	1.93	1.51	4.29	2.85	4.92	1.17	5.82	5.83	1.59	4.82	3.59	>0.999999999999999	1.3
P61916	Epididymal secretory protein E1 OS=Homo sapiens GN=NPC2 PE=1 SV=1	0.00	16.83	0.00	6.67	0.00	10.63	12.31	3.17	1.74	2.09	0.00	4.38	1.75	4.03	2.22	3.03	3.77	3.07	0.00	5.70	0.00	15.17	11.20	0.00	4.82	4.16	>0.999999999999999	1.2
P20962	Parathymosin OS=Homo sapiens GN=PTMS PE=1 SV=2	7.33	6.91	3.36	4.55	5.75	10.04	5.06	4.76	1.19	0.00	5.68	2.99	2.63	6.04	3.33	2.07	5.66	4.60	4.76	0.00	0.92	6.23	4.60	1.70	4.80	3.55	>0.999999999999999	1.4
P22061	Protein-L-isoaspartate(D-aspartate) O-methyltransferase OS=Homo sapiens GN=PCMT1 PE	11.23	3.23	3.25	4.40	2.68	12.81	4.89	7.29	0.56	0.00	2.65	4.58	2.54	9.27	1.56	4.55	2.64	4.45	2.22	5.96	0.89	6.03	2.15	3.75	4.80	3.83	>0.999999999999999	1.3
P05155	Plasma protease C1 inhibitor OS=Homo sapiens GN=SERPING1 PE=1 SV=2	6.35	5.99	3.70	5.00	6.33	3.78	6.92	0.95	1.31	3.44	9.47	4.08	2.28	8.26	5.55	1.79	9.43	3.99	9.35	2.46	1.26	8.52	3.99	2.84	4.78	4.98	>0.999999999999999	1.0
Q9UNM6	26S proteasome non-ATPase regulatory subunit 13 OS=Homo sapiens GN=PSMD13 PE=1	10.99	7.84	3.81	2.43	0.00	11.64	1.35	1.27	1.78	0.83	8.52	6.77	1.40	5.04	0.89	1.72	0.00	3.83	0.00	5.83	0.49	5.19	2.45	3.18	4.77	2.50	>0.999999999999999	1.9
P09455	Retinol-binding protein 1 OS=Homo sapiens GN=RBP1 PE=1 SV=2	19.05	4.84	5.27	3.18	0.00	16.88	0.00	3.33	0.00	0.00	0.00	4.68	10.18	4.23	0.00	1.45	0.00	0.00	0.00	20.99	0.65	4.36	0.00	0.00	4.77	3.49	>0.999999999999999	1.4
Q8NFU3	Thiosulfate sulfurtransferase/rhodanese-like domain-containing protein 1 OS=Homo sapiens	15.14	0.00	6.95	4.09	5.18	9.02	0.00	4.28	1.07	0.00	5.11	6.18	5.44	5.44	0.00	0.00	0.00	0.00	0.00	3.50	0.83	5.61	0.00	1.53	4.75	1.86	>0.999999999999999	2.6
O15143	Actin-related protein 2/3 complex subunit 1B OS=Homo sapiens GN=ARPC1B PE=1 SV=3	3.91	10.60	4.03	6.98	3.07	8.44	1.35	2.54	1.43	6.05	4.92	3.59	3.16	9.27	1.78	2.48	3.02	2.45	4.12	5.96	0.80	17.45	5.52	2.61	4.74	4.89	>0.999999999999999	1.0
P49721	Proteasome subunit beta type-2 OS=Homo sapiens GN=PSMB2 PE=1 SV=1	3.66	7.15	5.60	2.27	5.95	14.12	5.23	0.00	1.98	0.00	5.87	4.98	1.32	6.24	3.44	1.03	2.83	4.75	0.00	6.48	0.95	3.12	2.30	1.76	4.73	2.85	>0.999999999999999	1.7
P43487	Ran-specific GTPase-activating protein OS=Homo sapiens GN=RANBP1 PE=1 SV=1	11.97	3.23	3.47	4.70	2.68	7.13	5.23	2.22	0.56	0.00	5.87	9.46	2.72	6.24	3.44	0.97	0.00	2.15	2.22	6.35	0.43	10.18	2.15	2.78	4.71	3.30	>0.999999999999999	1.4
O00391	Sulfhydryl oxidase 1 OS=Homo sapiens GN=QSOX1 PE=1 SV=3	5.13	5.99	4.71	4.70	6.90	6.11	8.94	3.33	1.86	2.71	3.98	2.09	2.28	6.24	4.00	2.48	8.86	4.75	4.12	5.44	0.80	4.36	3.22	2.04	4.71	4.05	>0.999999999999999	1.2
P02760	Protein AMBP OS=Homo sapiens GN=AMBP PE=1 SV=1	4.15	3.92	3.03	9.25	7.29	1.16	8.27	1.27	1.07	2.82	9.28	4.88	3.33	7.65	3.00	1.86	9.24	2.61	9.67	1.04	1.51	5.61	7.52	1.53	4.70	4.55	>0.999999999999999	1.0
P51665	26S proteasome non-ATPase regulatory subunit 7 OS=Homo sapiens GN=PSMD7 PE=1 S\	10.01	15.44	5.94	2.88	1.73	7.71	0.00	3.01	1.15	0.00	5.49	2.89	0.79	8.26	2.11	2.83	0.00	1.38	1.43	3.76	0.59	1.87	1.38	0.51	4.69	2.07	>0.999999999999999	2.3
P01593	Ig kappa chain V-I region AG OS=Homo sapiens PE=1 SV=1	0.00	6.45	7.28	4.25	5.37	4.08	4.72	10.30	1.11	0.00	12.31	0.00	0.00	5.64	3.11	4.48	5.28	4.29	4.44	8.42	0.86	5.82	4.29	0.00	4.66	3.89	>0.999999999999999	1.2
Q13185	Chromobox protein homolog 3 OS=Homo sapiens GN=CBX3 PE=1 SV=4	13.68	3.69	3.92	2.43	0.00	8.15	2.70	2.54	0.63	3.65	10.60	3.49	1.40	3.22	1.78	1.10	0.00	2.45	0.00	7.26	0.49	3.32	5.37	0.91	4.62	2.28	>0.999999999999999	2.0
Q9Y224	UPF0568 protein C14orf166 OS=Homo sapiens GN=C14orf166 PE=1 SV=1	9.77	5.76	2.80	1.82	4.79	17.32	0.00	0.00	1.59	1.25	4.73	5.58	0.00	0.00	0.00	1.72	0.00	1.84	0.00	5.18	0.00	2.49	1.84	0.00	4.62	1.09	>0.999999999999999	4.2
P67809	Nuclease-sensitive element-binding protein 1 OS=Homo sapiens GN=YBX1 PE=1 SV=3	7.33	4.38	7.84	4.55	3.64	8.15	5.06	4.76	1.19	0.94	5.68	1.89	0.00	8.46	2.11	1.31	0.00	0.00	4.76	5.44	0.59	1.87	0.00	2.38	4.62	2.24	>0.999999999999999	2.1
P30101	Protein disulfide-isomerase A3 OS=Homo sapiens GN=PDIA3 PE=1 SV=4	6.11	7.38	5.38	0.91	2.30	15.57	3.04	5.07	1.27	2.61	2.27	3.19	2.19	8.06	4.44	1.72	3.39	8.59	6.34	7.26	0.77	5.19	2.76	2.27	4.59	4.42	>0.999999999999999	1.0
P01767	Ig heavy chain V-III region BUT OS=Homo sapiens PE=1 SV=1	0.00	0.00	7.06	16.38	20.71	0.00	0.00	0.00	4.28	6.57	0.00	0.00	0.00	0.00	0.00	4.35	11.88	0.00	0.00	0.00	0.00	13.09	0.00	0.00	4.58	2.44	>0.999999999999999	1.9
Q9Y365	PCTP-like protein OS=Homo sapiens GN=STARD10 PE=2 SV=2	11.23	7.61	5.15	1.52	4.03	8.88	0.00	3.33	2.42	0.00	6.25	4.58	0.88	6.65	1.11	1.45	3.96	5.06	0.00	7.90	0.31	4.36	1.53	2.61	4.58	2.99	>0.999999999999999	1.5
P04208	Ig lambda chain V-I region WAH OS=Homo sapiens PE=1 SV=1	0.00	0.00	12.77	10.01	0.00	0.00	0.00	10.46	1.15	11.90	5.49	2.89	5.79	22.96	12.66	4.55	12.44	17.48	10.46	8.55	2.03	13.71	0.00	3.75	4.56	9.53	>0.999999999999999	0.5
P00915	Carbonic anhydrase 1 OS=Homo sapiens GN=CA1 PE=1 SV=2	0.00	2.77	0.00	0.00	22.06	0.00	0.00	0.00	0.00	2.50	21.77	5.48	0.00	11.08	0.00	0.00	0.00	0.00	0.00	0.00	4.31	0.00	14.26	0.00	4.55	2.47	>0.999999999999999	1.8
P18124	60S ribosomal protein L7 OS=Homo sapiens GN=RPL7 PE=1 SV=1	9.28	2.54	6.05	0.00	2.11	10.33	1.86	6.02	2.14	1.15	7.20	5.38	0.00	0.00	0.00	0.76	0.00	3.68	1.74	7.00	0.74	0.00	0.00	0.62	4.50	1.21	>0.999999999999999	3.7
P01619	Ig kappa chain V-III region B6 OS=Homo sapiens PE=1 SV=1	0.00	0.00	0.00	17.59	0.00	0.00	0.00	10.62	7.10	18.68	0.00	0.00	0.00	0.00	19.89	0.00	0.00	0.00	0.00	0.00	0.00	0.00	0.00	0.00	4.50	1.66	>0.999999999999999	2.7
Q8N335	Glycerol-3-phosphate dehydrogenase 1-like protein OS=Homo sapiens GN=GPD1L PE=1 S	18.81	6.45	2.02	2.73	1.53	5.53	1.35	1.27	1.98	0.00	7.20	4.98	0.70	10.07	2.00	2.62	0.00	1.23	1.27	8.16	1.54	3.74	4.29	2.84	4.49	3.21	>0.999999999999999	1.4
Q9UHY7	Enolase-phosphatase E1 OS=Homo sapiens GN=ENOPH1 PE=1 SV=1	17.59	5.53	6.05	3.64	2.11	7.86	0.00	1.74	0.95	0.00	4.54	3.79	0.96	7.65	0.00	2.62	2.07	0.00	0.00	4.92	0.00	0.00	1.69	0.00	4.48	1.66	>0.999999999999999	2.7
Q9NY33	Dipeptidyl peptidase 3 OS=Homo sapiens GN=DPP3 PE=1 SV=2	8.79	8.30	5.94	3.18	3.26	6.11	2.02	1.90	0.83	0.00	7.95	5.28	1.49	8.46	2.33	2.14	1.51	1.84	0.63	8.55	0.95	5.40	1.23	2.04	4.46	3.05	>0.999999999999999	1.5
P15880	40S ribosomal protein S2 OS=Homo sapiens GN=RPS2 PE=1 SV=2	5.37	8.07	3.92	0.00	4.22	9.46	1.86	0.00	1.39	0.00	12.31	6.47	0.00	4.43	1.22	0.76	2.07	5.37	1.74	2.85	1.08	2.29	1.69	0.62	4.42	2.01	>0.999999999999999	2.2
Q7RTV2	Glutathione S-transferase A5 OS=Homo sapiens GN=GSTA5 PE=2 SV=1	10.75	10.14	0.00	0.00	12.08	0.00	4.72	6.98	0.00	0.00	8.33	0.00	3.86	0.00	0.00	3.03	0.00	0.00	0.00	5.70	0.00	0.00	0.00	0.00	4.42	1.05	>0.999999999999999	4.2
P49189	4-trimethylaminobutyraldehyde dehydrogenase OS=Homo sapiens GN=ALDH9A1 PE=1 SV	8.30	7.84	2.91	3.94	1.15	11.50	3.21	0.95	1.67	0.63	4.92	5.98	1.05	6.85	2.11	1.31	1.13	6.44	0.00	3.37	0.59	5.40	1.84	1.48	4.42	2.63	>0.999999999999999	1.7
P13646	Keratin, type I cytoskeletal 13 OS=Homo sapiens GN=KRT13 PE=1 SV=4	0.00	17.98	0.00	4.40	4.03	0.00	7.93	0.00	0.00	18.58	0.00	0.00	0.00	9.47	0.00	0.00	0.00	0.00	12.36	0.00	0.00	0.00	0.00	0.00	4.41	1.82	>0.999999999999999	2.4
P22532	Small proline-rich protein 2D OS=Homo sapiens GN=SPRR2D PE=2 SV=2	0.00	0.00	20.17	0.00	0.00	0.00	0.00	28.53	3.93	0.00	0.00	0.00	0.00	59.43	0.00	0.00	0.00	0.00	0.00	0.00	3.05	0.00	0.00	0.00	4.39	5.21	>0.999999999999999	0.8
P09488	Glutathione S-transferase Mu 1 OS=Homo sapiens GN=GSTM1 PE=1 SV=3	15.14	10.14	4.93	0.00	0.00	0.00	0.00	0.00	0.00	0.00	11.74	10.66	0.00	0.00	0.00	0.00	0.00	0.00	0.00	0.00	0.00	5.61	0.00	2.50	4.38	0.68	>0.999999999999999	6.5
P09651	Heterogeneous nuclear ribonucleoprotein A1 OS=Homo sapiens GN=HNRNPA1 PE=1 SV=5	9.53	0.00	0.00	0.00	3.45	9.17	6.58	4.44	1.98	0.00	9.47	7.67	1.58	10.07	3.11	2.69	0.00	0.00	0.00	6.48	0.55	0.00	4.29	2.21	4.36	2.58	>0.999999999999999	1.7
P06312	Ig kappa chain V-IV region (Fragment) OS=Homo sapiens GN=IGKV4-1 PE=4 SV=1	0.00	13.14	2.80	8.64	10.93	0.00	0.00	0.00	0.00	5.95	10.79	0.00	5.00	0.00	0.00	1.72	10.75	3.83	0.00	0.00	1.76	0.00	0.00	0.00	4.35	1.92	>0.999999999999999	2.3
Q9NZD2	Glycolipid transfer protein OS=Homo sapiens GN=GLTP PE=1 SV=3	11.72	6.91	5.38	7.28	2.68	2.04	2.36	2.22	1.19	0.00	5.68	4.78	1.23	6.04	3.33	2.07	2.64	4.60	2.22	3.89	1.48	2.91	2.15	2.73	4.35	2.94	>0.999999999999999	1.5
P26038	Moesin OS=Homo sapiens GN=MSN PE=1 SV=3	5.13	7.61	3.70	5.00	1.92	3.93	6.58	3.33	1.31	4.80	6.25	2.69	1.84	5.44	3.00	1.86	2.83	3.22	3.33	2.72	0.46	21.40	8.13	1.19	4.35	4.62	>0.999999999999999	0.9
P00734	Prothrombin OS=Homo sapiens GN=F2 PE=1 SV=2	6.11	0.00	3.47	10.77	10.93	0.00	4.22	1.43	0.79	2.09	8.14	4.28	7.72	5.04	7.89	2.55	13.39	2.15	9.04	0.65	0.95	4.16	3.07	1.42	4.35	4.83	>0.999999999999999	0.9
Q6PCE3	Glucose 1,6-bisphosphate synthase OS=Homo sapiens GN=PGM2L1 PE=1 SV=3	12.21	8.53	4.15	3.79	4.79	5.39	0.84	0.79	0.79	0.00	5.87	4.98	0.79	4.03	1.00	0.62	0.00	0.00	0.00	1.81	0.28	1.87	1.38	0.51	4.34	1.02	>0.999999999999999	4.2
P34932	Heat shock 70 kDa protein 4 OS=Homo sapiens GN=HSPA4 PE=1 SV=4	7.57	7.15	4.03	1.67	5.95	7.28	3.04	1.74	1.59	0.31	6.82	4.98	1.23	5.44	1.22	1.24	2.64	4.75	0.00	2.98	0.34	2.29	3.53	2.04	4.34	2.31	>0.999999999999999	1.9
Q96EK6	Glucosamine 6-phosphate N-acetyltransferase OS=Homo sapiens GN=GNPNAT1 PE=1 SV	3.91	7.84	6.27	2.43	3.07	4.95	2.70	2.54	2.22	0.00	10.60	5.58	0.00	6.85	1.78	2.35	0.00	5.21	0.00	4.41	1.05	7.07	5.21	3.18	4.34	3.09	>0.999999999999999	1.4
P35998	26S protease regulatory subunit 7 OS=Homo sapiens GN=PSMC2 PE=1 SV=3	11.72	13.37	2.47	2.12	1.34	8.44	1.18	1.11	2.30	0.73	4.17	2.99	0.61	6.04	1.56	1.52	1.32	3.37	1.11	7.51	0.43	2.91	2.15	2.21	4.33	2.56	>0.999999999999999	1.7
Q9BPX5	Actin-related protein 2/3 complex subunit 5-like protein OS=Homo sapiens GN=ARPC5L PE	4.88	10.14	4.93	3.03	3.84	15.57	3.37	0.00	1.74	0.00	0.00	4.38	0.00	0.00	2.22	1.38	0.00	3.07	0.00	2.59	0.00	0.00	0.00	4.09	4.33	1.11	>0.999999999999999	3.9
P15104	Glutamine synthetase OS=Homo sapiens GN=GLUL PE=1 SV=4	8.55	3.69	5.04	3.79	4.79	10.04	2.70	2.54	1.78	1.67	4.73	2.49	2.19	9.07	3.89	3.10	4.71	5.37	1.27	7.38	1.39	7.27	6.90	2.55	4.32	4.59	>0.999999999999999	0.9
P11908	Ribose-phosphate pyrophosphokinase 2 OS=Homo sapiens GN=PRPS2 PE=1 SV=2	2.20	9.91	3.47	1.36	1.73	15.43	1.52	0.00	2.26	0.00	8.14	5.68	0.00	4.03	2.22	0.62	0.00	3.07	0.00	0.00	0.95	1.87	1.38	1.14	4.31	1.27	>0.999999999999999	3.4
Q9Y678	Coatomer subunit gamma OS=Homo sapiens GN=COPG PE=1 SV=1	8.30	5.99	4.26	1.52	3.45	11.64	3.04	2.22	1.51	0.31	4.92	4.28	0.88	4.43	1.11	1.24	0.00	3.99	2.22	3.89	0.92	2.91	1.07	2.16	4.29	2.07	>0.999999999999999	2.1
Q96FW1	Ubiquitin thioesterase OTUB1 OS=Homo sapiens GN=OTUB1 PE=1 SV=2	11.97	5.07	1.23	3.34	4.22	9.46	1.86	0.00	1.39	0.00	9.28	3.49	3.07	4.43	3.89	2.41	0.00	1.69	0.00	1.43	1.08	4.57	0.00	1.99	4.27	2.05	>0.999999999999999	2.1
Q9BYE4	Small proline-rich protein 2G OS=Homo sapiens GN=SPRR2G PE=2 SV=1	9.77	0.00	0.00	6.07	7.67	0.00	16.19	0.00	0.00	0.00	7.57	3.98	0.00	0.00	10.67	2.76	18.10	0.00	0.00	0.00	0.00	0.00	0.00	5.45	4.27	3.08	>0.999999999999999	1.4
P46782	40S ribosomal protein S5 OS=Homo sapiens GN=RPS5 PE=1 SV=4	12.21	3.46	1.68	4.70	2.88	10.48	2.53	2.38	0.00	0.00	5.87	4.98	0.00	3.02	0.00	1.03	0.00	2.30	2.38	4.02	0.95	0.00	0.00	1.76	4.26	1.29	>0.999999999999999	3.3
Q9P2T1	GMP reductase 2 OS=Homo sapiens GN=GMPR2 PE=1 SV=1	15.63	6.45	2.02	0.00	3.45	9.32	3.04	2.85	0.71	0.00	7.38	0.00	1.58	5.64	1.00	0.62	0.00	1.38	0.00	3.63	0.28	0.00	4.29	1.02	4.24	1.62	>0.999999999999999	2.6
P20618	Proteasome subunit beta type-1 OS=Homo sapiens GN=PSMB1 PE=1 SV=2	2.93	13.83	0.00	1.82	4.99	11.64	0.00	0.00	3.17	0.00	7.95	4.18	1.05	5.24	4.67	1.79	0.00	1.84	0.00	3.37	1.29	5.40	0.00	3.41	4.21	2.34	>0.999999999999999	1.8
P50225	Sulfotransferase 1A1 OS=Homo sapiens GN=SULT1A1 PE=1 SV=3	22.23	0.00	5.04	0.00	0.00	10.77	0.00	5.07	0.00	0.00	0.00	7.37	5.18	0.00	0.00	0.00	0.00	0.00	0.00	0.00	0.00	4.16	1.53	0.00	4.21	0.91	>0.999999999999999	4.6
P11413	Glucose-6-phosphate 1-dehydrogenase OS=Homo sapiens GN=G6PD PE=1 SV=4	4.15	5.53	4.26	2.58	1.15	16.30	1.01	0.95	2.82	1.77	4.54	5.38	0.96	12.49	6.00	3.17	3.20	3.68	0.00	7.00	1.17	14.75	3.68	1.76	4.20	4.82	>0.999999999999999	0.9
P62899	60S ribosomal protein L31 OS=Homo sapiens GN=RPL31 PE=1 SV=1	12.94	0.00	5.94	0.00	0.00	12.95	4.05	0.00	2.10	0.00	10.04	2.39	0.00	10.68	0.00	3.66	0.00	8.13	0.00	6.87	0.00	0.00	0.00	5.05	4.20	2.87	>0.999999999999999	1.5
P40306	Proteasome subunit beta type-10 OS=Homo sapiens GN=PSMB10 PE=1 SV=1	5.86	12.45	1.23	1.67	4.60	5.53	4.05	1.74	2.14	1.15	7.20	2.39	3.33	2.22	0.00	0.00	0.00	1.69	0.00	1.43	0.00	2.29	1.69	1.36	4.17	1.17	>0.999999999999999	3.6
P28072	Proteasome subunit beta type-6 OS=Homo sapiens GN=PSMB6 PE=1 SV=4	10.99	3.00	5.04	6.82	8.63	1.89	2.19	2.06	1.11	0.00	5.30	2.79	3.95	9.07	5.00	1.93	2.45	6.90	0.00	8.29	0.86	5.82	1.99	2.55	4.15	4.07	>0.999999999999999	1.0
P14174	Macrophage migration inhibitory factor OS=Homo sapiens GN=MIF PE=1 SV=4	6.59	6.22	3.03	4.09	5.18	3.93	4.55	4.28	1.07	2.82	5.11	2.69	2.37	5.44	3.00	1.86	0.00	4.14	4.28	3.50	0.83	5.61	4.14	1.53	4.13	3.06	>0.999999999999999	1.4
Q15907	Ras-related protein Rab-11B OS=Homo sapiens GN=RAB11B PE=1 SV=4	7.08	10.60	1.57	2.12	0.00	9.61	2.36	2.22	1.82	4.80	2.65	4.58	4.04	9.27	3.22	2.00	0.00	2.15	0.00	1.81	0.00	13.71	0.00	0.00	4.12	3.02	>0.999999999999999	1.4
P61086	Ubiquitin-conjugating enzyme E2 K OS=Homo sapiens GN=UBE2K PE=1 SV=3	18.07	11.99	1.68	2.27	0.00	4.66	0.00	2.38	2.06	0.00	2.84	3.19	1.32	6.45	3.56	1.03	2.83	2.30	0.00	6.74	0.46	6.65	0.00	2.95	4.09	2.86	>0.999999999999999	1.4
Q15019	Septin-2 OS=Homo sapiens GN=SEPT2 PE=1 SV=1	8.79	8.30	2.91	1.21	3.07	8.44	1.35	1.27	1.03	0.00	6.82	5.78	1.40	5.24	1.78	1.79	0.00	3.99	1.27	5.96	0.25	1.66	1.23	1.48	4.08	2.17	>0.999999999999999	1.9
Q15257	Serine/threonine-protein phosphatase 2A activator OS=Homo sapiens GN=PPP2R4 PE=1 S	11.48	1.84	4.03	2.58	6.90	5.24	4.39	1.27	1.43	0.00	4.92	4.68	2.28	3.42	4.00	1.17	0.00	1.23	1.27	6.09	0.52	3.53	1.23	2.67	4.06	2.28	>0.999999999999999	1.8
O00244	Copper transport protein ATOX1 OS=Homo sapiens GN=ATOX1 PE=1 SV=1	0.00	10.83	5.27	7.13	0.00	6.84	7.93	0.00	1.86	0.00	8.90	0.00	4.12	9.47	0.00	3.24	0.00	0.00	7.45	6.09	1.45	9.77	7.21	0.00	4.06	4.07	>0.999999999999999	1.0
O43396	Thioredoxin-like protein 1 OS=Homo sapiens GN=TXNL1 PE=1 SV=3	5.13	10.83	3.81	3.18	4.03	4.95	1.69	3.33	1.86	0.00	6.44	3.39	2.98	6.85	5.22	4.28	1.89	1.53	1.59	8.03	0.31	7.07	3.22	1.93	4.05	3.74	>0.999999999999999	1.1
Q5TD94	Radial spoke head protein 4 homolog A OS=Homo sapiens GN=RSPH4A PE=1 SV=1	11.97	8.53	1.90	0.61	4.22	7.13	0.67	2.69	0.16	0.00	7.01	3.69	1.14	0.00	0.00	0.00	1.51	0.00	0.00	0.52	0.00	0.00	0.00	0.00	4.05	0.26	>0.999999999999999	15.3
P25788	Proteasome subunit alpha type-3 OS=Homo sapiens GN=PSMA3 PE=1 SV=2	2.93	8.99	1.34	3.79	4.79	5.68	2.02	6.18	1.55	0.00	7.38	3.88	0.00	5.04	4.33	2.69	2.26	1.84	0.00	0.00	0.77	11.43	1.84	2.21	4.05	2.70	>0.999999999999999	1.5
P55957	BH3-interacting domain death agonist OS=Homo sapiens GN=BID PE=1 SV=1	3.66	7.38	3.59	7.89	6.14	7.57	2.53	0.00	0.59	1.57	6.06	1.49	1.32	6.45	0.00	0.00	0.00	2.30	2.38	1.94	0.46	6.65	4.91	1.82	4.04	2.35	>0.999999999999999	1.7
Q3LXA3	Bifunctional ATP-dependent dihydroxyacetone kinase/FAD-AMP lyase (cyclizing) OS=Homo	13.19	8.76	2.02	3.64	2.11	6.70	1.01	0.00	0.71	0.00	7.20	3.09	0.96	1.21	1.22	0.76	1.13	0.92	0.00	1.43	0.18	2.29	1.69	0.34	4.03	1.01	>0.999999999999999	4.0
P63172	Dynein light chain Tctex-type 1 OS=Homo sapiens GN=DYNLT1 PE=1 SV=1	6.59	6.22	3.03	0.00	5.18	3.93	4.55	0.00	1.07	0.00	11.55	6.08	0.00	0.00	0.00	0.00	0.00	0.00	0.00	3.50	0.00	0.00	0.00	0.00	4.02	0.29	>0.999999999999999	13.8
Q14103	Heterogeneous nuclear ribonucleoprotein D0 OS=Homo sapiens GN=HNRNPD PE=1 SV=1	4.40	4.15	5.71	2.73	3.45	5.68	3.04	2.85	1.55	1.88	9.66	2.79	1.58	5.64	2.00	2.69	0.00	2.76	2.85	3.63	0.55	5.82	2.76	1.59	3.99	2.66	>0.999999999999999	1.5
Q13404	Ubiquitin-conjugating enzyme E2 variant 1 OS=Homo sapiens GN=UBE2V1 PE=1 SV=2	4.88	10.37	2.24	6.82	3.84	6.55	3.37	3.17	0.83	0.00	3.79	1.99	1.75	4.03	5.00	1.38	3.77	3.07	3.17	2.59	0.62	4.16	3.07	1.14	3.99	2.81	>0.999999999999999	1.4
P19823	Inter-alpha-trypsin inhibitor heavy chain H2 OS=Homo sapiens GN=ITIH2 PE=1 SV=2	4.88	4.61	3.03	8.64	4.60	1.89	5.90	2.06	1.23	2.50	4.54	3.88	3.42	9.67	5.89	3.31	7.35	4.75	8.40	3.50	1.36	9.97	5.37	1.53	3.98	5.38	>0.999999999999999	0.7
Q8NC51	Plasminogen activator inhibitor 1 RNA-binding protein OS=Homo sapiens GN=SERBP1 PE=	10.50	3.46	2.69	3.64	6.33	7.71	1.18	1.11	0.59	0.00	6.25	4.28	1.32	0.00	0.78	0.00	1.32	1.07	2.38	0.91	1.02	0.00	2.30	0.40	3.98	0.96	>0.999999999999999	4.2
P09972	Fructose-bisphosphate aldolase C OS=Homo sapiens GN=ALDOC PE=1 SV=2	9.28	6.22	4.26	2.58	5.18	5.53	0.00	0.00	1.94	1.77	9.28	1.69	2.37	7.65	0.00	3.38	0.00	4.14	2.69	0.00	0.00	0.00	4.14	2.78	3.98	2.26	>0.999999999999999	1.8
Q32MZ4	Leucine-rich repeat flightless-interacting protein 1 OS=Homo sapiens GN=LRRFIP1 PE=1 S\	11.72	7.61	1.23	3.03	3.84	5.53	4.05	3.17	0.59	0.42	2.84	3.29	2.11	4.03	1.22	0.76	0.00	0.61	0.63	3.11	0.46	4.16	1.69	0.62	3.94	1.62	>0.999999999999999	2.4
Q13421	Mesothelin OS=Homo sapiens GN=MSLN PE=1 SV=2	2.44	3.46	7.40	1.52	8.63	2.91	1.69	5.07	1.78	1.57	4.92	5.88	0.88	4.03	5.67	0.69	6.03	0.77	1.59	1.30	1.82	3.12	3.99	3.75	3.94	2.80	>0.999999999999999	1.4
P48444	Coatomer subunit delta OS=Homo sapiens GN=ARCN1 PE=1 SV=1	6.11	5.76	4.48	1.82	3.45	8.15	3.04	2.85	2.58	0.00	3.41	5.58	0.53	6.45	2.78	2.76	1.13	8.59	0.95	5.18	0.99	1.25	0.92	2.27	3.94	2.82	>0.999999999999999	1.4
Q9BUT1	3-hydroxybutyrate dehydrogenase type 2 OS=Homo sapiens GN=BDH2 PE=1 SV=2	6.35	13.60	2.91	3.94	0.00	3.78	2.02	0.00	0.48	0.00	11.17	2.59	0.00	5.24	1.33	1.79	0.00	0.00	0.00	1.55	0.80	5.40	3.99	1.48	3.90	1.80	>0.999999999999999	2.2
O43598	Deoxyribonucleoside 5'-monophosphate N-glycosidase OS=Homo sapiens GN=RCL PE=1 S	15.14	4.15	6.95	2.73	3.45	2.62	3.04	0.00	1.51	0.00	3.41	3.79	1.58	0.00	0.00	1.24	0.00	0.00	0.00	2.33	0.00	0.00	0.00	0.00	3.90	0.43	>0.999999999999999	9.1
Q6EEV6	Small ubiquitin-related modifier 4 OS=Homo sapiens GN=SUMO4 PE=1 SV=2	7.82	7.38	0.00	4.85	6.14	4.66	5.40	0.00	1.27	0.00	6.06	3.19	2.81	6.45	3.56	2.21	0.00	4.91	0.00	4.15	0.99	6.65	4.91	1.82	3.90	3.20	>0.999999999999999	1.2
Q9NQW7	Xaa-Pro aminopeptidase 1 OS=Homo sapiens GN=XPNPEP1 PE=1 SV=3	9.04	4.61	2.91	2.27	3.84	9.46	3.37	1.59	1.47	0.00	3.79	4.38	1.32	5.24	1.67	2.14	0.94	3.07	2.38	4.79	0.62	6.44	2.30	1.48	3.89	2.70	>0.999999999999999	1.4
P61019	Ras-related protein Rab-2A OS=Homo sapiens GN=RAB2A PE=1 SV=1	7.33	11.06	3.36	4.55	2.68	0.00	0.00	0.00	2.74	0.00	5.68	9.26	0.00	9.67	0.00	0.00	0.00	4.60	0.00	3.89	0.00	0.00	4.60	0.00	3.89	1.90	>0.999999999999999	2.1
P55786	Puromycin-sensitive aminopeptidase OS=Homo sapiens GN=NPEPPS PE=1 SV=2	4.88	7.38	3.59	2.58	2.49	9.32	1.01	2.06	1.63	0.31	6.82	4.48	1.14	4.83	2.67	1.93	0.00	1.53	0.00	5.31	0.62	7.48	3.07	0.97	3.88	2.46	>0.999999999999999	1.6
P23141	Liver carboxylesterase 1 OS=Homo sapiens GN=CES1 PE=1 SV=2	8.79	5.30	4.03	0.00	6.90	10.92	0.00	6.82	0.00	0.00	3.22	0.50	3.77	1.01	3.22	0.00	0.00	0.00	0.00	2.20	0.00	0.00	0.00	0.00	3.87	0.85	>0.999999999999999	4.6
P31944	Caspase-14 OS=Homo sapiens GN=CASP14 PE=1 SV=2	0.00	2.77	8.52	1.82	2.30	1.75	4.22	15.22	0.48	5.95	2.27	1.20	0.00	8.06	1.33	2.76	7.54	1.84	3.96	1.55	0.00	8.31	1.84	1.42	3.87	3.22	>0.999999999999999	1.2
P41091	Eukaryotic translation initiation factor 2 subunit 3 OS=Homo sapiens GN=EIF2S3 PE=1 SV=:	6.84	0.00	4.03	3.03	3.84	9.32	0.00	4.44	1.78	0.00	6.82	6.38	0.00	7.25	0.00	1.38	0.00	4.29	2.06	4.66	0.40	0.00	0.00	1.59	3.87	1.80	>0.999999999999999	2.1
P62851	40S ribosomal protein S25 OS=Homo sapiens GN=RPS25 PE=1 SV=1	6.11	5.76	6.27	0.00	0.00	13.68	4.22	0.00	0.00	0.00	4.73	5.58	0.00	11.28	0.00	1.72	0.00	3.83	0.00	3.24	0.77	0.00	0.00	0.00	3.86	1.74	>0.999999999999999	2.2
P02750	Leucine-rich alpha-2-glycoprotein OS=Homo sapiens GN=LRG1 PE=1 SV=2	2.20	6.45	3.14	2.73	3.45	1.31	8.60	0.00	1.55	2.92	7.38	6.38	0.00	0.00	3.11	1.24	9.61	1.38	12.36	0.00	0.55	1.87	5.98	0.00	3.84	3.01	>0.999999999999999	1.3
Q9P1F3	Costars family protein C6orf115 OS=Homo sapiens GN=C6orf115 PE=1 SV=1	9.28	8.76	4.26	5.76	0.00	5.53	0.00	0.00	1.51	0.00	7.20	3.79	0.00	7.65	4.22	2.62	7.16	5.83	6.02	4.92	1.17	18.91	5.83	5.17	3.84	5.79	>0.999999999999999	0.7
Q8WWF8	Calcyphosin-like protein OS=Homo sapiens GN=CAPSL PE=2 SV=4	7.08	3.23	1.57	4.40	0.00	6.84	2.36	4.60	0.00	0.00	8.90	6.67	2.54	0.00	0.00	0.00	0.00	0.00	0.00	1.81	0.00	0.00	2.15	0.00	3.80	0.54	>0.999999999999999	7.0
P52788	Spermine synthase OS=Homo sapiens GN=SMS PE=1 SV=2	8.79	5.99	4.03	1.21	3.07	6.70	1.35	2.54	0.63	0.00	8.71	2.59	0.70	3.22	0.00	1.79	0.00	0.00	1.27	1.04	0.00	3.32	0.00	0.91	3.80	1.02	>0.999999999999999	3.7
P24666	Low molecular weight phosphotyrosine protein phosphatase OS=Homo sapiens GN=ACP1 F	4.40	0.00	4.48	0.00	3.45	9.46	3.04	2.85	1.59	0.00	12.31	3.98	3.51	13.09	0.00	2.76	0.00	2.76	0.00	8.42	1.23	0.00	6.13	2.27	3.80	3.35	>0.999999999999999	1.1
P04040	Catalase OS=Homo sapiens GN=CAT PE=1 SV=3	5.86	7.15	0.56	3.64	3.26	2.47	2.87	4.91	0.00	8.35	5.87	0.50	0.44	10.68	0.56	0.00	0.00	0.77	1.74	0.00	0.15	18.70	6.90	0.00	3.79	3.33	>0.999999999999999	1.1
P61513	60S ribosomal protein L37a OS=Homo sapiens GN=RPL37A PE=1 SV=2	8.06	7.61	3.70	0.00	6.33	4.80	0.00	5.23	0.00	0.00	6.25	3.29	0.00	6.65	3.67	0.00	0.00	5.06	0.00	4.28	0.00	0.00	0.00	1.87	3.77	1.79	>0.999999999999999	2.1
Q15435	Protein phosphatase 1 regulatory subunit 7 OS=Homo sapiens GN=PPP1R7 PE=1 SV=1	8.79	3.92	1.90	1.21	1.53	8.44	2.87	2.69	1.43	0.83	6.82	4.68	1.49	3.42	2.89	1.79	1.51	3.99	0.00	6.09	0.52	5.40	2.61	2.04	3.76	2.65	>0.999999999999999	1.4
P0C0S5	Histone H2A.Z OS=Homo sapiens GN=H2AFZ PE=1 SV=2	0.00	13.14	0.00	3.79	0.00	0.00	0.00	0.00	0.00	10.02	18.18	0.00	0.00	11.48	0.00	1.72	0.00	0.00	0.00	0.00	0.00	0.00	3.83	0.00	3.76	1.42	>0.999999999999999	2.6
Q02543	60S ribosomal protein L18a OS=Homo sapiens GN=RPL18A PE=1 SV=2	8.55	0.00	3.92	2.43	3.07	5.09	2.70	2.54	0.63	0.00	10.60	5.58	1.40	3.22	3.89	0.00	3.02	5.37	0.00	7.26	0.00	0.00	2.45	0.91	3.76	2.29	>0.999999999999999	1.6
Q9UHV9	Prefoldin subunit 2 OS=Homo sapiens GN=PFDN2 PE=1 SV=1	10.99	4.61	2.24	3.03	3.84	6.55	0.00	0.00	0.79	0.00	8.52	4.48	1.75	9.07	2.22	3.10	0.00	6.90	0.00	5.83	0.00	4.16	3.07	2.55	3.75	3.22	>0.999999999999999	1.2
Q9NRX4	14 kDa phosphohistidine phosphatase OS=Homo sapiens GN=PHPT1 PE=1 SV=1	5.86	5.53	2.69	3.64	4.60	3.49	4.05	3.80	0.95	0.00	4.54	5.48	2.11	11.08	0.00	1.66	0.00	3.68	0.00	7.13	0.74	4.99	3.68	1.36	3.72	3.03	>0.999999999999999	1.2
P13987	CD59 glycoprotein OS=Homo sapiens GN=CD59 PE=1 SV=1	5.62	5.30	0.00	3.49	9.78	0.00	3.88	3.65	0.91	5.32	4.35	2.29	4.47	4.63	2.56	1.59	4.34	3.53	3.65	6.61	0.00	4.78	0.00	1.31	3.72	3.12	>0.999999999999999	1.2
Q15506	Sperm surface protein Sp17 OS=Homo sapiens GN=SPA17 PE=1 SV=1	17.10	4.38	0.00	0.00	8.25	6.26	0.00	3.01	0.00	0.00	3.60	1.89	0.00	0.00	0.00	0.00	0.00	0.00	0.00	0.00	0.00	0.00	0.00	0.00	3.71	0.00	>0.999999999999999	#DIV/0!
P00739	Haptoglobin-related protein OS=Homo sapiens GN=HPR PE=1 SV=2	0.00	0.00	13.78	13.65	0.00	0.00	0.00	0.00	0.00	0.00	17.04	0.00	0.00	18.13	13.66	0.00	0.00	0.00	16.80	0.00	0.00	15.79	16.26	0.00	3.71	6.72	>0.999999999999999	0.6
P01614	Ig kappa chain V-II region Cum OS=Homo sapiens PE=1 SV=1	6.59	6.22	3.03	4.09	0.00	3.93	4.55	4.28	1.07	2.82	5.11	2.69	2.37	12.29	6.78	1.86	5.09	4.14	9.67	7.90	0.83	5.61	4.14	1.53	3.70	5.18	>0.999999999999999	0.7
P29966	Myristoylated alanine-rich C-kinase substrate OS=Homo sapiens GN=MARCKS PE=1 SV=4	0.00	8.07	3.92	5.31	1.92	5.09	3.71	5.55	0.87	1.04	6.63	2.19	0.00	2.01	1.11	0.00	1.89	3.37	1.59	4.53	0.00	4.57	5.37	1.25	3.69	2.14	>0.999999999999999	1.7
Q96G03	Phosphoglucomutase-2 OS=Homo sapiens GN=PGM2 PE=1 SV=4	7.82	7.38	4.37	2.27	1.92	6.55	2.53	1.59	1.03	1.57	3.98	3.19	0.44	9.07	4.33	2.21	2.83	3.99	2.38	3.37	0.99	9.35	3.99	1.48	3.68	3.70	>0.999999999999999	1.0
P61604	10 kDa heat shock protein, mitochondrial OS=Homo sapiens GN=HSPE1 PE=1 SV=2	0.00	7.38	8.29	0.00	0.00	4.66	0.00	11.73	1.27	3.34	0.00	7.37	2.81	14.91	0.00	0.00	0.00	4.91	0.00	4.15	0.00	6.65	4.91	1.82	3.67	3.35	>0.999999999999999	1.1
P08779	Keratin, type I cytoskeletal 16 OS=Homo sapiens GN=KRT16 PE=1 SV=4	0.00	0.00	0.00	0.00	5.37	0.00	7.59	0.00	0.00	25.57	5.30	0.00	0.00	0.00	0.00	0.00	0.00	0.00	10.15	0.00	0.00	4.16	6.90	0.00	3.65	1.77	>0.999999999999999	2.1
P51884	Lumican OS=Homo sapiens GN=LUM PE=1 SV=2	9.53	0.00	4.37	4.25	7.48	1.31	4.72	1.43	1.55	0.94	5.30	2.79	3.42	1.81	8.67	3.52	5.28	1.38	8.08	1.17	0.28	3.74	5.98	1.59	3.64	3.74	>0.999999999999999	1.0
P62318	Small nuclear ribonucleoprotein Sm D3 OS=Homo sapiens GN=SNRPD3 PE=1 SV=1	5.86	5.53	2.69	3.64	0.00	3.49	0.00	0.00	0.00	2.50	17.42	2.39	0.00	4.83	0.00	1.66	0.00	0.00	0.00	3.11	0.00	0.00	0.00	0.00	3.63	0.80	>0.999999999999999	4.5
P52597	Heterogeneous nuclear ribonucleoprotein F OS=Homo sapiens GN=HNRNPF PE=1 SV=3	5.62	5.30	5.83	2.27	1.34	4.66	3.88	3.65	0.00	0.00	7.76	3.19	2.02	3.02	0.78	1.03	0.00	4.91	0.00	1.94	0.22	1.45	1.07	1.82	3.62	1.52	>0.999999999999999	2.4
Q96FJ2	Dynein light chain 2, cytoplasmic OS=Homo sapiens GN=DYNLL2 PE=1 SV=1	8.06	7.61	3.70	5.00	0.00	4.80	0.00	0.00	1.31	3.44	6.25	3.29	0.00	0.00	3.67	0.00	0.00	5.06	0.00	4.28	0.00	6.86	0.00	0.00	3.62	1.66	>0.999999999999999	2.2
Q9Y5P6	Mannose-1-phosphate guanyltransferase beta OS=Homo sapiens GN=GMPPB PE=1 SV=2	4.15	6.22	1.90	2.58	1.53	8.73	2.87	1.27	1.07	0.00	7.01	5.98	0.70	3.42	0.89	0.55	0.00	2.61	0.00	1.04	0.52	7.69	1.23	2.10	3.61	1.73	>0.999999999999999	2.1
P62136	Serine/threonine-protein phosphatase PP1-alpha catalytic subunit OS=Homo sapiens GN=P	15.88	0.00	5.83	0.00	0.00	13.68	4.72	0.00	3.13	0.00	0.00	0.00	0.00	0.00	2.00	0.00	0.00	4.29	0.00	0.00	0.55	0.00	0.00	1.59	3.60	0.70	>0.999999999999999	5.1
P36578	60S ribosomal protein L4 OS=Homo sapiens GN=RPL4 PE=1 SV=5	5.37	3.23	3.36	2.12	2.68	10.19	2.36	2.22	1.19	0.73	7.38	2.19	1.93	6.04	2.44	2.07	2.64	4.60	2.22	5.05	0.22	0.00	0.00	0.79	3.59	2.33	>0.999999999999999	1.5
Q6P5S2	UPF0762 protein C6orf58 OS=Homo sapiens GN=C6orf58 PE=1 SV=2	2.20	8.99	4.37	7.73	3.45	1.31	3.04	2.85	1.11	0.94	1.70	5.08	3.42	5.64	3.11	2.69	3.39	4.29	2.85	1.17	1.97	5.82	1.38	1.59	3.56	3.11	>0.999999999999999	1.1
Q9NQP4	Prefoldin subunit 4 OS=Homo sapiens GN=PFDN4 PE=1 SV=1	0.00	11.29	5.49	3.34	0.00	7.13	3.71	3.49	1.94	0.00	4.17	2.19	0.00	9.87	2.44	1.52	0.00	3.37	3.49	0.00	0.68	4.57	3.37	1.25	3.56	2.55	>0.999999999999999	1.4
P48507	Glutamate--cysteine ligase regulatory subunit OS=Homo sapiens GN=GCLM PE=1 SV=1	8.79	8.30	2.58	0.00	2.11	7.28	0.00	3.65	1.98	0.00	4.35	3.59	0.00	2.22	2.56	0.76	2.07	1.69	0.00	4.66	0.34	7.48	1.69	0.62	3.55	2.01	>0.999999999999999	1.8
Q13630	GDP-L-fucose synthase OS=Homo sapiens GN=TSTA3 PE=1 SV=1	4.64	6.91	2.13	1.36	1.73	10.04	5.06	1.43	0.75	0.94	5.68	1.89	1.67	6.04	2.11	1.31	1.70	6.44	1.43	5.44	0.59	6.23	2.91	1.70	3.55	3.13	>0.999999999999999	1.1
P02747	Complement C1q subcomponent subunit C OS=Homo sapiens GN=C1QC PE=1 SV=3	3.18	6.22	3.03	6.67	5.18	3.93	4.55	2.06	1.07	2.82	2.46	1.29	1.14	12.49	4.89	1.86	5.09	1.99	6.98	3.50	0.40	9.14	4.14	0.74	3.54	4.36	>0.999999999999999	0.8
Q13813	Spectrin alpha chain, brain OS=Homo sapiens GN=SPTAN1 PE=1 SV=3	5.86	5.30	2.24	1.67	3.07	8.44	0.51	1.74	0.32	1.77	7.95	3.59	0.88	3.22	0.56	0.55	0.19	1.69	0.32	2.20	0.09	3.53	1.07	0.68	3.54	1.25	>0.999999999999999	2.8
P28074	Proteasome subunit beta type-5 OS=Homo sapiens GN=PSMB5 PE=1 SV=3	17.83	5.76	2.80	3.79	2.30	1.75	0.00	1.90	0.00	0.00	2.27	3.88	1.05	7.86	1.33	0.00	2.26	0.00	0.00	0.00	0.00	0.00	0.00	1.42	3.52	1.16	>0.999999999999999	3.0
P24158	Myeloblastin OS=Homo sapiens GN=PRTN3 PE=1 SV=3	2.93	9.22	0.00	1.82	2.30	1.75	4.22	0.00	0.00	5.84	14.20	0.00	2.19	0.00	2.78	0.00	0.00	3.83	0.00	0.00	0.00	8.31	8.59	0.00	3.52	2.14	>0.999999999999999	1.6
P05114	Non-histone chromosomal protein HMG-14 OS=Homo sapiens GN=HMGN1 PE=1 SV=3	0.00	7.38	0.00	0.00	6.14	4.66	5.40	0.00	1.27	0.00	14.20	3.19	2.81	6.45	0.00	0.00	6.03	4.91	0.00	4.15	0.00	6.65	4.91	1.82	3.52	3.14	>0.999999999999999	1.1
P55036	26S proteasome non-ATPase regulatory subunit 4 OS=Homo sapiens GN=PSMD4 PE=1 S\	11.48	10.83	0.90	1.21	0.00	6.84	1.35	1.27	0.32	0.00	3.22	4.68	0.00	7.25	0.00	1.17	0.00	0.00	0.00	3.37	0.80	0.00	0.00	0.97	3.51	1.13	>0.999999999999999	3.1
P10412	Histone H1.4 OS=Homo sapiens GN=HIST1H1E PE=1 SV=2	0.00	0.00	0.00	8.04	0.00	2.18	8.94	8.40	0.00	8.04	6.25	0.00	2.89	0.00	0.00	0.00	6.22	5.06	0.00	0.00	0.00	6.86	5.06	0.85	3.49	2.25	>0.999999999999999	1.6
P15153	Ras-related C3 botulinum toxin substrate 2 OS=Homo sapiens GN=RAC2 PE=1 SV=1	0.00	0.00	0.00	8.04	0.00	0.00	5.57	5.23	2.10	10.85	10.04	0.00	2.89	10.68	0.00	0.00	9.99	0.00	5.23	0.00	1.02	21.61	8.13	1.87	3.49	5.12	>0.999999999999999	0.7
Q15056	Eukaryotic translation initiation factor 4H OS=Homo sapiens GN=EIF4H PE=1 SV=5	6.35	5.99	4.59	3.94	2.30	3.78	0.00	1.90	1.03	0.00	7.76	4.08	1.05	5.24	1.33	0.83	2.26	3.99	0.00	5.31	1.26	2.49	1.84	0.68	3.48	2.19	>0.999999999999999	1.6
P49354	Protein farnesyltransferase/geranylgeranyltransferase type-1 subunit alpha OS=Homo sapie	5.86	5.53	2.69	3.64	4.60	4.80	4.05	3.80	0.59	0.00	2.84	3.29	1.32	4.83	0.78	0.48	0.00	2.30	1.11	3.11	0.46	3.12	2.30	0.85	3.48	1.72	>0.999999999999999	2.0
P62854	40S ribosomal protein S26 OS=Homo sapiens GN=RPS26 PE=1 SV=3	14.17	0.00	2.91	0.00	4.99	3.78	4.39	0.00	1.03	2.71	4.92	2.59	0.00	0.00	2.89	0.00	4.90	0.00	4.12	0.00	0.80	5.40	0.00	1.48	3.46	1.63	>0.999999999999999	2.1
P62081	40S ribosomal protein S7 OS=Homo sapiens GN=RPS7 PE=1 SV=1	3.66	3.46	3.70	0.00	2.88	7.71	5.57	2.38	0.59	0.00	10.04	1.49	0.00	6.65	1.67	1.03	0.00	0.00	2.38	9.85	1.63	0.00	0.00	0.00	3.46	1.93	>0.999999999999999	1.8
Q04695	Keratin, type I cytoskeletal 17 OS=Homo sapiens GN=KRT17 PE=1 SV=2	0.00	11.29	0.00	5.91	0.00	0.00	6.58	0.00	0.00	17.64	0.00	0.00	3.42	11.89	0.00	0.00	5.66	0.00	9.35	0.00	0.92	6.23	5.98	0.00	3.45	3.62	>0.999999999999999	1.0
P0CF74	Ig lambda-6 chain C region OS=Homo sapiens GN=IGLC6 PE=4 SV=1	0.00	0.00	0.00	41.40	0.00	0.00	0.00	0.00	0.00	0.00	0.00	0.00	0.00	0.00	0.00	0.00	0.00	0.00	0.00	0.00	0.00	0.00	0.00	0.00	3.45	0.00	>0.999999999999999	#DIV/0!
P04004	Vitronectin OS=Homo sapiens GN=VTN PE=1 SV=1	4.64	5.99	2.91	5.16	6.52	1.75	2.02	1.90	0.75	2.71	3.60	3.39	3.68	3.83	2.89	1.31	4.90	0.92	6.66	1.55	0.80	5.40	2.91	1.48	3.45	3.03	>0.999999999999999	1.1
Q13561	Dynactin subunit 2 OS=Homo sapiens GN=DCTN2 PE=1 SV=4	5.86	5.53	3.70	3.64	2.88	6.26	4.05	1.11	0.59	0.73	4.54	2.39	2.11	6.65	1.67	1.03	1.32	3.68	0.00	3.11	0.46	3.12	3.68	1.36	3.44	2.35	>0.999999999999999	1.5
Q16181	Septin-7 OS=Homo sapiens GN=SEPT7 PE=1 SV=2	6.84	3.00	2.35	3.18	4.03	6.70	2.19	2.06	0.83	0.63	3.98	5.48	1.84	5.64	0.67	0.00	1.13	3.22	0.95	3.63	0.40	7.69	4.29	1.59	3.44	2.59	>0.999999999999999	1.3
Q9H910	Hematological and neurological expressed 1-like protein OS=Homo sapiens GN=HN1L PE='	8.79	3.92	4.03	2.58	3.26	2.47	2.87	5.71	0.67	0.00	3.22	3.59	1.49	3.42	0.00	1.17	0.00	0.00	0.00	2.20	1.11	0.00	2.61	0.97	3.43	1.08	>0.999999999999999	3.2
P17980	26S protease regulatory subunit 6A OS=Homo sapiens GN=PSMC3 PE=1 SV=3	7.08	3.23	2.35	1.06	5.56	5.53	1.18	3.33	0.83	0.73	5.49	4.68	1.23	4.23	2.33	1.45	0.00	2.15	0.00	2.72	0.22	4.36	0.00	1.19	3.42	1.66	>0.999999999999999	2.1
P62750	60S ribosomal protein L23a OS=Homo sapiens GN=RPL23A PE=1 SV=1	4.64	4.38	2.13	2.88	3.64	10.04	7.08	0.00	0.75	0.00	3.60	1.89	0.00	13.90	2.11	0.00	0.00	6.44	3.01	2.46	0.59	0.00	2.91	1.08	3.42	2.71	>0.999999999999999	1.3
Q9BY32	Inosine triphosphate pyrophosphatase OS=Homo sapiens GN=ITPA PE=1 SV=2	3.66	3.46	1.68	0.00	0.00	11.21	0.00	0.00	0.59	0.00	10.04	10.36	0.00	0.00	0.00	2.28	0.00	0.00	0.00	1.94	0.46	0.00	2.30	0.85	3.42	0.65	>0.999999999999999	5.2
Q9Y3I0	tRNA-splicing ligase RtcB homolog OS=Homo sapiens GN=C22orf28 PE=1 SV=1	6.35	4.38	2.91	1.82	4.99	3.78	2.02	3.01	0.75	0.63	6.25	4.08	1.05	2.42	1.33	0.83	0.00	1.84	1.90	2.46	0.37	3.95	1.84	1.08	3.42	1.59	>0.999999999999999	2.1
P13010	X-ray repair cross-complementing protein 5 OS=Homo sapiens GN=XRCC5 PE=1 SV=3	10.26	7.15	4.03	3.18	1.53	3.78	1.35	2.69	0.83	0.83	3.22	2.09	1.49	0.81	0.44	1.79	0.00	1.84	1.27	2.72	0.37	0.00	0.61	0.00	3.41	0.95	>0.999999999999999	3.6
P02649	Apolipoprotein E OS=Homo sapiens GN=APOE PE=1 SV=1	7.33	2.07	4.71	4.55	8.05	0.00	7.08	1.43	1.19	0.94	1.70	1.89	3.68	3.83	11.22	2.90	10.37	4.60	16.01	1.17	2.12	14.34	6.44	0.00	3.41	6.39	>0.999999999999999	0.5
P27797	Calreticulin OS=Homo sapiens GN=CALR PE=1 SV=1	1.71	5.07	2.47	1.06	1.34	8.59	5.06	11.10	0.87	2.30	1.33	0.00	1.93	4.43	2.44	0.48	2.64	5.98	6.18	2.85	0.43	10.18	2.15	0.40	3.41	3.34	>0.999999999999999	1.0
P62701	40S ribosomal protein S4, X isoform OS=Homo sapiens GN=RPS4X PE=1 SV=2	2.69	2.54	5.94	1.67	2.11	12.81	0.00	1.74	1.47	0.00	4.54	5.28	0.00	0.00	0.00	0.00	0.00	0.00	0.00	3.11	0.74	2.29	0.00	0.62	3.40	0.56	>0.999999999999999	6.0
P56537	Eukaryotic translation initiation factor 6 OS=Homo sapiens GN=EIF6 PE=1 SV=1	2.93	2.77	4.71	6.37	0.00	8.59	2.02	4.12	0.48	1.25	4.92	2.59	1.05	5.24	2.89	5.45	0.00	9.05	1.90	7.64	0.37	16.42	6.44	1.48	3.40	4.83	>0.999999999999999	0.7
P55327	Tumor protein D52 OS=Homo sapiens GN=TPD52 PE=1 SV=2	3.42	10.83	5.27	2.12	2.68	6.84	0.00	2.22	2.66	0.00	0.00	4.68	1.23	5.84	3.22	2.00	0.00	2.15	2.22	6.09	0.89	9.77	2.15	1.65	3.39	3.10	>0.999999999999999	1.1
Q07960	Rho GTPase-activating protein 1 OS=Homo sapiens GN=ARHGAP1 PE=1 SV=1	5.13	6.68	4.15	1.06	4.03	6.70	1.18	1.11	1.47	0.73	5.49	2.89	0.61	2.62	1.44	0.90	1.32	1.99	1.11	3.76	0.89	7.69	1.07	2.61	3.38	2.17	>0.999999999999999	1.6
Q99873	Protein arginine N-methyltransferase 1 OS=Homo sapiens GN=PRMT1 PE=1 SV=2	6.11	3.69	5.15	2.43	1.53	8.30	0.00	1.27	1.82	0.00	6.63	3.49	0.00	5.04	0.00	1.72	0.00	0.00	1.27	5.96	0.00	0.00	2.45	1.42	3.37	1.49	>0.999999999999999	2.3
Q96FQ6	Protein S100-A16 OS=Homo sapiens GN=S100A16 PE=1 SV=1	7.08	6.68	3.25	4.40	0.00	4.22	4.89	0.00	1.15	0.00	5.49	2.89	0.00	5.84	3.22	2.00	5.47	4.45	4.60	8.55	0.89	6.03	0.00	1.65	3.34	3.56	>0.999999999999999	0.9
P53621	Coatomer subunit alpha OS=Homo sapiens GN=COPA PE=1 SV=2	5.62	5.30	3.25	1.52	0.00	8.01	2.02	1.11	2.18	0.00	5.49	5.48	0.18	6.45	2.00	0.48	0.38	2.76	0.79	2.59	0.15	2.08	0.31	1.14	3.33	1.61	>0.999999999999999	2.1
P33176	Kinesin-1 heavy chain OS=Homo sapiens GN=KIF5B PE=1 SV=1	7.33	3.69	1.79	0.91	1.15	9.32	1.01	1.90	0.75	0.31	7.95	3.79	0.53	4.63	0.33	0.83	0.00	2.91	0.00	3.37	0.49	3.95	0.92	1.08	3.33	1.59	>0.999999999999999	2.1
O60493	Sorting nexin-3 OS=Homo sapiens GN=SNX3 PE=1 SV=3	4.40	4.15	4.37	0.00	3.45	2.62	3.04	2.85	3.73	0.00	7.38	3.88	1.58	0.00	4.33	1.24	0.00	2.76	0.00	5.05	0.55	0.00	0.00	1.02	3.32	1.38	>0.999999999999999	2.4
P62306	Small nuclear ribonucleoprotein F OS=Homo sapiens GN=SNRPF PE=1 SV=1	0.00	8.30	4.03	0.00	0.00	5.24	6.07	5.71	0.00	0.00	6.82	3.59	0.00	7.25	0.00	2.48	0.00	5.52	0.00	4.66	1.11	7.48	0.00	0.00	3.31	2.38	>0.999999999999999	1.4
Q15366	Poly(rC)-binding protein 2 OS=Homo sapiens GN=PCBP2 PE=1 SV=1	9.53	6.45	3.14	0.00	0.00	7.28	0.00	0.00	0.00	0.00	9.47	3.88	0.70	5.64	0.00	1.93	0.00	2.76	0.00	6.48	1.54	5.82	5.98	2.21	3.31	2.76	>0.999999999999999	1.2
P43034	Platelet-activating factor acetylhydrolase IB subunit alpha OS=Homo sapiens GN=PAFAH1B	5.37	3.23	2.47	3.34	2.68	4.51	2.36	3.49	0.87	0.73	7.57	3.09	1.93	4.43	1.56	2.14	2.64	3.37	0.00	7.90	0.43	8.31	6.13	2.84	3.31	3.47	>0.999999999999999	1.0
P49591	Serine--tRNA ligase, cytoplasmic OS=Homo sapiens GN=SARS PE=1 SV=3	7.57	5.53	2.69	1.67	3.45	9.17	1.01	0.00	1.23	0.63	1.14	5.38	0.53	4.83	1.22	1.24	0.00	2.76	0.00	5.96	0.74	2.29	3.68	1.02	3.29	2.02	>0.999999999999999	1.6
P49458	Signal recognition particle 9 kDa protein OS=Homo sapiens GN=SRP9 PE=1 SV=2	8.30	7.84	0.00	5.16	0.00	4.95	0.00	5.39	1.35	0.00	6.44	0.00	0.00	6.85	3.78	0.00	0.00	0.00	0.00	4.41	0.00	0.00	0.00	0.00	3.29	1.25	>0.999999999999999	2.6
P43652	Afamin OS=Homo sapiens GN=AFM PE=1 SV=1	6.11	2.07	2.24	3.79	8.44	2.91	2.53	0.00	0.79	0.94	7.01	2.49	1.75	3.02	4.11	1.38	8.30	1.38	4.91	1.17	1.14	5.19	1.38	1.14	3.28	2.91	>0.999999999999999	1.1
Q9Y2Z0	Suppressor of G2 allele of SKP1 homolog OS=Homo sapiens GN=SUGT1 PE=1 SV=3	8.79	8.30	0.90	1.21	1.53	8.59	0.00	0.00	0.32	0.00	6.82	2.59	0.00	1.61	0.00	0.55	0.00	1.23	0.00	1.04	0.25	1.66	2.61	0.97	3.25	0.83	>0.999999999999999	3.9
P01610	Ig kappa chain V-I region WEA OS=Homo sapiens PE=1 SV=1	0.00	0.00	7.40	4.40	0.00	4.22	0.00	10.46	0.00	0.00	12.50	0.00	0.00	5.84	3.22	4.55	5.47	4.45	0.00	8.55	0.00	0.00	0.00	1.65	3.25	2.81	>0.999999999999999	1.2
P05198	Eukaryotic translation initiation factor 2 subunit 1 OS=Homo sapiens GN=EIF2S1 PE=1 SV=:	4.64	2.07	3.36	1.36	3.64	6.11	3.21	3.01	1.67	0.00	5.68	4.18	0.79	3.83	2.11	0.62	1.70	4.60	1.43	3.89	0.28	3.95	2.91	1.70	3.25	2.32	>0.999999999999999	1.4
P61576	HERV-K_5q13.3 provirus Rec protein OS=Homo sapiens PE=1 SV=1	7.08	6.68	3.25	4.40	0.00	4.22	0.00	4.60	0.00	3.03	5.49	0.00	0.00	5.84	3.22	2.00	5.47	4.45	4.60	3.76	0.89	6.03	4.45	1.65	3.23	3.53	>0.999999999999999	0.9
P18283	Glutathione peroxidase 2 OS=Homo sapiens GN=GPX2 PE=1 SV=3	7.82	3.46	3.59	2.27	2.88	7.57	2.53	0.00	0.59	0.00	2.84	5.18	0.00	10.48	0.00	3.59	2.83	2.30	0.00	1.94	0.00	0.00	0.00	0.85	3.23	1.83	>0.999999999999999	1.8
P62495	Eukaryotic peptide chain release factor subunit 1 OS=Homo sapiens GN=ETF1 PE=1 SV=3	3.42	8.76	5.38	2.12	1.34	6.99	1.18	1.11	1.90	0.00	2.65	3.79	0.61	6.04	2.33	3.31	1.32	1.07	1.11	2.72	0.43	4.36	2.15	1.19	3.22	2.22	>0.999999999999999	1.4
P53004	Biliverdin reductase A OS=Homo sapiens GN=BLVRA PE=1 SV=2	7.82	4.84	1.12	3.18	4.03	6.70	1.69	0.00	0.83	0.00	6.06	2.09	0.88	4.23	2.33	1.45	0.00	3.22	0.00	4.15	0.65	4.36	1.53	1.19	3.20	2.00	>0.999999999999999	1.6
Q15008	26S proteasome non-ATPase regulatory subunit 6 OS=Homo sapiens GN=PSMD6 PE=1 S\	7.82	7.38	3.59	2.27	1.34	6.11	1.18	0.00	0.91	0.00	1.33	6.28	1.32	10.48	0.00	0.48	0.00	2.30	0.00	2.98	0.00	3.12	0.00	2.38	3.18	1.92	>0.999999999999999	1.7
P04433	Ig kappa chain V-III region VG (Fragment) OS=Homo sapiens PE=1 SV=1	6.59	0.00	3.03	4.09	0.00	3.93	4.55	4.28	1.07	2.82	5.11	2.69	2.37	5.44	3.00	1.86	5.09	4.14	4.28	3.50	0.83	5.61	4.14	1.53	3.18	3.48	>0.999999999999999	0.9
P06318	Ig lambda chain V-VI region WLT OS=Homo sapiens PE=1 SV=1	6.84	6.45	3.14	4.25	5.37	0.00	0.00	0.00	1.11	2.92	5.30	2.79	2.46	5.64	3.11	1.93	5.28	4.29	4.44	3.63	0.86	5.82	0.00	1.59	3.18	3.25	>0.999999999999999	1.0
P01037	Cystatin-SN OS=Homo sapiens GN=CST1 PE=1 SV=3	0.00	0.00	17.03	0.00	0.00	0.00	7.59	11.73	1.78	0.00	0.00	0.00	6.49	0.00	0.00	0.00	3.77	0.00	3.17	19.69	0.00	0.00	0.00	0.00	3.18	2.76	>0.999999999999999	1.2
P54725	UV excision repair protein RAD23 homolog A OS=Homo sapiens GN=RAD23A PE=1 SV=1	9.28	6.22	3.03	1.21	3.26	2.47	1.35	1.27	0.00	0.00	5.11	4.88	0.70	5.44	0.00	0.00	0.00	0.00	0.00	2.20	0.00	3.53	1.23	2.16	3.17	1.27	>0.999999999999999	2.5
P09210	Glutathione S-transferase A2 OS=Homo sapiens GN=GSTA2 PE=1 SV=4	0.00	0.00	0.00	0.00	0.00	0.00	0.00	0.00	0.00	0.00	38.06	0.00	0.00	0.00	0.00	0.00	0.00	0.00	0.00	0.00	0.00	0.00	0.00	0.00	3.17	0.00	>0.999999999999999	#DIV/0!
Q9BRF8	Calcineurin-like phosphoesterase domain-containing protein 1 OS=Homo sapiens GN=CPPE	10.26	4.38	2.13	2.88	5.75	2.77	1.52	1.43	0.36	0.00	3.60	2.99	0.79	1.81	0.00	2.90	1.70	1.38	0.00	5.44	0.59	6.23	4.60	1.08	3.17	2.21	>0.999999999999999	1.4
O14737	Programmed cell death protein 5 OS=Homo sapiens GN=PDCD5 PE=1 SV=3	5.86	0.00	2.69	0.00	0.00	13.10	0.00	0.00	0.95	0.00	10.04	5.28	2.11	0.00	0.00	0.00	0.00	3.68	0.00	3.11	0.74	0.00	0.00	3.01	3.16	1.05	>0.999999999999999	3.0
P54577	Tyrosine--tRNA ligase, cytoplasmic OS=Homo sapiens GN=YARS PE=1 SV=4	9.28	7.15	2.69	1.67	1.15	9.02	0.00	0.95	0.95	0.00	1.14	3.79	0.96	6.24	2.67	2.62	0.00	2.76	0.00	4.02	0.55	6.44	0.92	1.02	3.15	2.35	>0.999999999999999	1.3
P07357	Complement component C8 alpha chain OS=Homo sapiens GN=C8A PE=1 SV=2	3.66	1.15	4.48	8.19	10.36	0.73	4.55	0.79	0.20	2.19	0.95	0.50	0.88	4.23	4.44	1.45	7.54	0.77	3.33	1.30	0.15	5.61	3.22	0.28	3.15	2.77	>0.999999999999999	1.1
Q8WYR4	Radial spoke head 1 homolog OS=Homo sapiens GN=RSPH1 PE=2 SV=1	17.59	0.00	0.00	1.36	1.73	4.51	1.52	3.17	0.00	0.00	5.87	1.99	0.00	0.00	0.00	0.00	0.00	0.00	0.00	0.00	0.00	0.00	0.00	0.00	3.14	0.00	>0.999999999999999	#DIV/0!
O00231	26S proteasome non-ATPase regulatory subunit 11 OS=Homo sapiens GN=PSMD11 PE=1	7.57	5.07	2.47	1.06	1.34	8.73	1.18	3.49	1.23	0.73	2.65	2.19	1.23	8.06	1.56	0.97	1.32	3.37	0.00	5.18	0.43	6.44	2.15	1.25	3.14	2.66	>0.999999999999999	1.2
Q58FF6	Putative heat shock protein HSP 90-beta 4 OS=Homo sapiens GN=HSP90AB4P PE=5 SV=	5.86	5.53	3.47	2.73	0.00	4.51	1.86	3.80	1.23	0.00	7.38	1.10	1.58	3.63	2.00	1.24	2.07	3.68	1.74	4.02	0.74	3.74	1.69	0.62	3.12	2.23	>0.999999999999999	1.4
Q9H3K6	BolA-like protein 2 OS=Homo sapiens GN=BOLA2 PE=1 SV=1	19.05	0.00	0.00	0.00	0.00	4.95	0.00	0.00	0.00	3.55	6.44	3.39	0.00	0.00	3.78	0.00	0.00	0.00	0.00	4.66	1.05	0.00	5.21	1.93	3.11	1.39	>0.999999999999999	2.2
Q9H0W9	Ester hydrolase C11orf54 OS=Homo sapiens GN=C11orf54 PE=1 SV=1	13.68	4.38	4.82	1.36	3.64	4.51	0.00	0.00	0.36	0.00	3.60	0.90	0.00	1.81	1.00	0.62	0.00	0.00	0.00	1.17	0.28	0.00	0.00	0.00	3.10	0.41	>0.999999999999999	7.6
P07602	Proactivator polypeptide OS=Homo sapiens GN=PSAP PE=1 SV=2	4.15	5.53	3.47	2.58	3.26	5.53	0.84	4.91	0.67	2.50	2.08	1.69	2.11	6.24	1.89	2.14	2.07	5.83	0.79	4.92	0.95	9.35	3.68	3.01	3.10	3.58	>0.999999999999999	0.9
P43686	26S protease regulatory subunit 6B OS=Homo sapiens GN=PSMC4 PE=1 SV=2	5.37	7.15	3.47	2.12	4.22	7.28	1.18	0.00	0.56	0.00	2.65	3.09	1.23	4.43	1.56	1.52	0.00	3.37	0.00	1.81	0.00	0.00	0.00	1.76	3.09	1.31	>0.999999999999999	2.4
Q9Y490	Talin-1 OS=Homo sapiens GN=TLN1 PE=1 SV=3	6.11	4.61	2.24	3.18	2.68	5.24	1.86	1.74	1.07	1.77	4.17	2.39	0.88	5.64	1.11	0.90	1.70	0.77	0.79	4.15	0.52	10.39	5.52	1.02	3.09	2.78	>0.999999999999999	1.1
Q13442	28 kDa heat- and acid-stable phosphoprotein OS=Homo sapiens GN=PDAP1 PE=1 SV=1	8.55	3.69	3.92	2.43	0.00	5.09	2.70	2.54	1.39	0.00	3.03	3.49	0.00	3.22	1.78	1.10	0.00	0.00	0.00	2.07	0.00	3.32	5.37	0.00	3.07	1.41	>0.999999999999999	2.2
Q71DI3	Histone H3.2 OS=Homo sapiens GN=HIST2H3A PE=1 SV=3	0.00	28.12	0.00	0.00	0.00	0.00	0.00	0.00	0.00	8.56	0.00	0.00	0.00	0.00	0.00	0.00	0.00	0.00	3.65	0.00	0.00	0.00	0.00	0.00	3.06	0.30	>0.999999999999999	10.1
O00232	26S proteasome non-ATPase regulatory subunit 12 OS=Homo sapiens GN=PSMD12 PE=1	6.59	8.07	1.46	3.03	1.15	5.09	2.19	0.00	1.07	0.00	5.11	2.69	1.14	4.03	1.44	1.38	0.00	1.99	0.95	3.50	0.18	4.16	1.99	1.53	3.04	1.86	>0.999999999999999	1.6
O43583	Density-regulated protein OS=Homo sapiens GN=DENR PE=1 SV=2	7.82	7.38	1.68	2.27	2.88	7.57	2.53	0.00	0.00	0.00	2.84	1.49	1.32	6.45	3.56	2.21	2.83	2.30	0.00	1.94	0.46	3.12	4.91	0.00	3.04	2.42	>0.999999999999999	1.3
Q02818	Nucleobindin-1 OS=Homo sapiens GN=NUCB1 PE=1 SV=4	3.18	3.00	2.13	1.97	0.00	1.89	10.29	5.55	2.06	1.36	3.60	1.29	3.77	2.62	3.00	1.31	6.60	2.91	5.55	2.46	0.59	2.70	4.14	1.08	3.03	3.06	>0.999999999999999	1.0
O75363	Breast carcinoma-amplified sequence 1 OS=Homo sapiens GN=BCAS1 PE=1 SV=2	2.69	6.91	4.03	3.49	0.96	4.37	0.84	3.65	1.43	0.00	4.35	3.59	5.18	10.27	1.89	1.59	0.00	3.53	0.79	7.64	0.00	2.29	1.69	2.90	3.03	3.15	>0.999999999999999	1.0
P62736	Actin, aortic smooth muscle OS=Homo sapiens GN=ACTA2 PE=1 SV=1	16.85	0.00	0.00	19.41	0.00	0.00	0.00	0.00	0.00	0.00	0.00	0.00	0.00	0.00	0.00	0.00	0.00	0.00	0.00	0.00	0.00	0.00	0.00	0.00	3.02	0.00	>0.999999999999999	#DIV/0!
P46459	Vesicle-fusing ATPase OS=Homo sapiens GN=NSF PE=1 SV=3	1.95	7.15	4.71	1.21	3.26	6.11	1.35	0.63	2.34	0.42	3.98	3.09	0.35	5.24	0.89	1.79	1.51	1.84	0.00	5.44	0.80	1.66	1.84	2.04	3.02	1.95	>0.999999999999999	1.5
Q8NBJ4	Golgi membrane protein 1 OS=Homo sapiens GN=GOLM1 PE=1 SV=1	0.00	1.61	5.83	4.85	4.41	2.18	7.08	6.66	2.06	0.00	0.00	1.49	2.81	4.63	5.78	1.03	1.32	1.07	0.00	2.98	1.60	1.45	4.91	3.58	3.02	2.60	>0.999999999999999	1.2
P55209	Nucleosome assembly protein 1-like 1 OS=Homo sapiens GN=NAP1L1 PE=1 SV=1	3.66	7.38	2.58	2.27	4.41	3.35	1.18	3.65	1.67	0.00	2.84	3.19	2.02	4.63	2.56	1.59	1.32	2.30	0.00	1.94	0.71	6.65	1.07	1.82	3.01	2.22	>0.999999999999999	1.4
Q9Y262	Eukaryotic translation initiation factor 3 subunit L OS=Homo sapiens GN=EIF3L PE=1 SV=1	6.59	2.30	5.27	2.27	0.96	6.84	1.69	1.59	1.86	0.00	3.98	2.69	0.88	5.44	1.11	0.69	0.00	0.77	0.00	2.72	0.65	1.04	0.77	1.19	3.00	1.27	>0.999999999999999	2.4
O15212	Prefoldin subunit 6 OS=Homo sapiens GN=PFDN6 PE=1 SV=1	5.62	5.30	2.58	3.49	0.00	12.81	3.88	0.00	0.00	0.00	0.00	2.29	2.02	4.63	2.56	1.59	0.00	3.53	0.00	2.98	0.00	4.78	0.00	1.31	3.00	1.95	>0.999999999999999	1.5
Q13838	Spliceosome RNA helicase DDX39B OS=Homo sapiens GN=DDX39B PE=1 SV=1	7.08	6.68	4.26	3.18	0.00	5.53	0.00	0.00	2.26	0.00	3.98	2.89	0.00	5.84	0.00	1.45	0.00	3.22	2.22	3.76	0.65	0.00	0.00	2.16	2.99	1.61	>0.999999999999999	1.9
P26599	Polypyrimidine tract-binding protein 1 OS=Homo sapiens GN=PTBP1 PE=1 SV=1	2.93	4.15	2.80	1.82	3.45	3.64	2.02	0.95	0.99	0.00	9.09	3.98	0.53	3.63	1.33	1.24	1.13	0.92	0.95	0.78	0.18	2.49	0.00	1.82	2.99	1.25	>0.999999999999999	2.4

Q9UNZ2	NSFL1 cofactor p47 OS=Homo sapiens GN=NSFL1C PE=1 SV=2	4.15	6.22	1.90	2.58	3.26	3.93	2.87	4.28	0.67	0.00	3.22	2.69	0.70	5.44	4.11	1.86	1.51	2.61	1.27	3.50	0.25	5.61	2.61	0.97	2.98	2.54	>0.999999999999999	1.2
Q9HAB8	Phosphopantothenate--cysteine ligase OS=Homo sapiens GN=PPCS PE=1 SV=2	2.44	10.37	3.59	3.03	1.92	2.91	1.69	1.59	1.27	0.00	3.79	3.19	0.88	4.03	1.11	1.38	0.00	3.07	0.00	2.59	0.00	6.65	0.00	1.14	2.98	1.74	>0.999999999999999	1.7
P60983	Glia maturation factor beta OS=Homo sapiens GN=GMFB PE=1 SV=2	4.88	10.14	0.00	0.00	0.00	6.40	0.00	3.17	0.79	0.00	8.33	1.99	0.00	0.00	0.00	1.38	0.00	0.00	0.00	0.00	0.00	0.00	6.75	1.14	2.98	0.77	>0.999999999999999	3.9
Q969Q0	60S ribosomal protein L36a-like OS=Homo sapiens GN=RPL36AL PE=1 SV=3	14.90	0.00	3.03	0.00	0.00	8.88	0.00	0.00	1.07	0.00	5.11	2.69	0.00	5.44	0.00	0.00	0.00	4.14	0.00	7.90	0.00	0.00	0.00	0.00	2.97	1.46	>0.999999999999999	2.0
P51991	Heterogeneous nuclear ribonucleoprotein A3 OS=Homo sapiens GN=HNRNPA3 PE=1 SV=2	9.28	3.92	1.90	1.21	1.53	7.13	1.35	0.00	0.67	0.83	5.11	2.69	0.70	3.42	0.00	0.00	1.51	1.23	0.00	2.20	0.00	1.66	4.14	0.45	2.97	1.28	>0.999999999999999	2.3
P46776	60S ribosomal protein L27a OS=Homo sapiens GN=RPL27A PE=1 SV=2	4.88	4.61	5.04	3.03	3.84	6.55	0.00	0.00	1.78	0.00	3.79	1.99	0.00	0.00	0.00	0.00	0.00	6.90	0.00	5.83	0.62	0.00	0.00	1.14	2.96	1.21	>0.999999999999999	2.5
P31948	Stress-induced-phosphoprotein 1 OS=Homo sapiens GN=STIP1 PE=1 SV=1	5.37	2.54	3.25	2.43	0.96	8.44	1.86	1.74	1.71	0.52	3.03	3.59	3.16	4.43	1.78	1.52	0.94	4.45	0.00	2.85	0.49	6.03	3.37	1.25	2.95	2.52	>0.999999999999999	1.2
P01011	Alpha-1-antichymotrypsin OS=Homo sapiens GN=SERPINA3 PE=1 SV=2	3.42	1.61	4.48	0.00	1.34	2.04	5.23	11.10	0.56	1.46	4.17	0.00	3.51	0.00	4.44	0.48	2.64	2.15	0.00	0.91	0.22	0.00	9.05	0.00	2.95	1.95	>0.999999999999999	1.5
Q9BTM9	Ubiquitin-related modifier 1 homolog OS=Homo sapiens GN=URM1 PE=1 SV=1	7.33	6.91	3.36	0.00	0.00	4.37	0.00	4.76	0.00	0.00	5.68	2.99	2.63	6.04	3.33	2.07	0.00	4.60	0.00	0.00	0.00	6.23	0.00	1.70	2.95	2.22	>0.999999999999999	1.3
P31943	Heterogeneous nuclear ribonucleoprotein H OS=Homo sapiens GN=HNRNPH1 PE=1 SV=4	3.42	4.84	5.27	2.12	1.34	4.22	1.18	1.11	0.83	0.00	7.20	3.79	1.23	4.23	0.78	0.48	0.00	4.45	1.11	4.92	0.22	1.45	0.00	1.65	2.94	1.71	>0.999999999999999	1.7
O00625	Pirin OS=Homo sapiens GN=PIR PE=1 SV=1	8.30	2.30	3.81	3.34	4.22	3.20	1.69	1.59	0.40	0.00	4.17	2.19	1.93	0.00	2.44	0.69	1.89	1.53	1.59	1.30	0.31	2.08	1.53	0.57	2.93	1.32	>0.999999999999999	2.2
P68400	Casein kinase II subunit alpha OS=Homo sapiens GN=CSNK2A1 PE=1 SV=1	10.26	1.61	1.68	1.06	2.88	6.11	4.05	0.00	0.59	0.00	4.54	2.39	0.00	1.41	1.67	0.00	1.32	2.30	0.00	0.00	0.22	0.00	0.00	0.00	2.93	0.58	>0.999999999999999	5.1
Q16719	Kynureninase OS=Homo sapiens GN=KYNU PE=1 SV=1	3.18	10.14	3.03	1.97	1.15	5.09	2.19	0.00	2.46	0.63	2.46	2.69	1.14	4.03	3.00	4.97	0.00	6.75	0.00	8.03	0.62	12.88	1.99	3.52	2.92	3.91	>0.999999999999999	0.7
P06753	Tropomyosin alpha-3 chain OS=Homo sapiens GN=TPM3 PE=1 SV=1	2.44	4.84	1.12	3.18	1.92	9.02	1.69	1.59	0.83	2.19	3.98	2.09	0.88	2.01	1.11	0.69	3.96	3.22	0.00	2.72	0.65	12.88	3.22	1.19	2.91	2.71	>0.999999999999999	1.1
Q10567	AP-1 complex subunit beta-1 OS=Homo sapiens GN=AP1B1 PE=1 SV=2	7.82	4.61	3.59	3.64	1.92	4.66	0.00	0.95	1.11	0.00	3.79	2.79	0.88	4.03	1.11	0.69	0.00	2.61	0.95	3.63	0.00	2.08	0.00	1.36	2.91	1.44	>0.999999999999999	2.0
P28062	Proteasome subunit beta type-8 OS=Homo sapiens GN=PSMB8 PE=1 SV=3	5.62	2.54	1.23	1.67	4.41	5.24	1.86	1.74	1.43	0.00	6.82	2.29	0.96	2.22	1.22	0.00	2.07	1.69	1.74	1.43	0.00	4.78	5.52	2.04	2.90	1.97	>0.999999999999999	1.5
Q13347	Eukaryotic translation initiation factor 3 subunit I OS=Homo sapiens GN=EIF3I PE=1 SV=1	4.64	6.68	2.13	1.36	1.73	9.90	0.00	3.01	0.75	0.94	1.70	1.89	0.79	8.26	1.00	0.62	1.70	4.45	0.00	5.31	0.59	0.00	1.38	0.51	2.90	2.05	>0.999999999999999	1.4
P29034	Protein S100-A2 OS=Homo sapiens GN=S100A2 PE=1 SV=3	0.00	0.00	3.36	4.85	0.00	4.37	5.40	4.76	2.78	3.13	6.06	0.00	0.00	6.45	3.33	4.83	5.66	0.00	4.76	9.07	2.16	14.55	4.60	1.82	2.89	4.77	>0.999999999999999	0.6
O14979	Heterogeneous nuclear ribonucleoprotein D-like OS=Homo sapiens GN=HNRPDL PE=1 SV=	5.62	3.46	2.58	2.27	2.88	4.51	2.53	0.00	0.91	1.57	5.87	2.29	0.00	4.63	1.67	1.59	2.83	2.30	2.38	2.98	0.00	0.00	2.30	1.31	2.87	1.83	>0.999999999999999	1.6
P54136	Arginine--tRNA ligase, cytoplasmic OS=Homo sapiens GN=RARS PE=1 SV=2	3.18	10.60	2.02	0.61	0.77	7.57	1.52	2.06	1.35	0.00	2.46	2.29	0.00	4.63	1.00	0.90	0.00	4.45	0.00	5.96	0.12	2.70	0.00	0.74	2.87	1.71	>0.999999999999999	1.7
Q9BRP8	Partner of Y14 and mago OS=Homo sapiens GN=WIBG PE=1 SV=1	12.46	7.38	3.59	0.00	0.00	4.66	2.53	0.00	0.59	0.00	0.00	3.19	0.00	6.45	0.00	2.21	2.83	2.30	0.00	6.61	0.46	3.12	2.30	0.85	2.87	2.26	>0.999999999999999	1.3
P05546	Heparin cofactor 2 OS=Homo sapiens GN=SERPIND1 PE=1 SV=3	2.93	1.38	2.80	3.79	6.14	0.87	5.40	0.95	0.71	0.63	7.57	1.20	2.81	8.06	4.44	2.21	3.39	0.92	8.88	1.55	1.23	6.65	2.76	1.02	2.86	3.66	>0.999999999999999	0.8
P28161	Glutathione S-transferase Mu 2 OS=Homo sapiens GN=GSTM2 PE=1 SV=2	10.75	6.22	0.00	0.00	0.00	3.93	0.00	0.00	0.00	0.00	5.11	8.27	0.00	0.00	0.00	0.00	0.00	0.00	0.00	0.00	0.00	0.00	0.00	1.53	2.86	0.13	>0.999999999999999	22.4
P61163	Alpha-centractin OS=Homo sapiens GN=ACTR1A PE=1 SV=1	0.00	5.76	5.04	0.00	4.79	5.09	2.70	1.27	0.99	0.00	3.03	5.58	0.70	5.04	0.89	0.55	0.00	2.45	0.00	1.04	0.25	5.19	0.00	2.55	2.85	1.56	>0.999999999999999	1.8
P19827	Inter-alpha-trypsin inhibitor heavy chain H1 OS=Homo sapiens GN=ITIH1 PE=1 SV=3	0.73	3.00	1.90	6.98	5.56	1.89	4.89	0.48	0.52	1.77	3.98	2.49	1.14	4.23	1.44	1.17	4.71	1.53	5.87	1.30	0.89	5.19	2.61	0.97	2.85	2.59	>0.999999999999999	1.1
P18510	Interleukin-1 receptor antagonist protein OS=Homo sapiens GN=IL1RN PE=1 SV=1	3.91	8.30	1.79	2.43	0.00	2.33	0.00	5.71	1.43	1.67	3.03	3.59	1.40	16.92	0.00	1.10	3.02	5.52	2.54	0.00	1.11	12.05	2.45	3.29	2.85	4.12	>0.999999999999999	0.7
Q9Y6U3	Adseverin OS=Homo sapiens GN=SCIN PE=1 SV=4	7.82	3.00	4.15	2.58	0.77	7.13	2.19	1.27	1.07	0.00	2.46	1.69	0.35	2.62	0.44	1.17	0.00	2.61	0.00	2.20	0.40	0.00	0.61	0.74	2.84	0.93	>0.999999999999999	3.1
Q6XQN6	Nicotinate phosphoribosyltransferase OS=Homo sapiens GN=NAPRT1 PE=1 SV=2	4.40	4.15	4.37	3.79	0.00	4.66	3.04	1.90	0.99	0.63	2.27	3.88	1.05	6.45	2.00	3.24	0.00	1.84	0.00	1.55	0.18	6.65	1.84	2.67	2.84	2.29	>0.999999999999999	1.2
Q6IBS0	Twinfilin-2 OS=Homo sapiens GN=TWF2 PE=1 SV=2	4.15	8.76	3.03	1.21	5.18	3.93	1.35	0.00	1.51	0.83	3.22	0.80	0.70	5.44	0.89	0.00	0.00	2.61	0.00	1.04	0.25	7.90	4.14	0.45	2.83	1.95	>0.999999999999999	1.5
P68032	Actin, alpha cardiac muscle 1 OS=Homo sapiens GN=ACTC1 PE=1 SV=1	0.00	0.00	0.00	0.00	0.00	0.00	0.00	20.45	5.12	0.00	0.00	8.27	0.00	0.00	0.00	0.00	0.00	0.00	0.00	0.00	0.00	0.00	0.00	0.00	2.82	0.00	>0.999999999999999	#DIV/0!
P05156	Complement factor I OS=Homo sapiens GN=CFI PE=1 SV=2	1.22	3.46	3.03	5.91	4.03	3.06	4.55	0.79	0.59	2.19	2.84	2.09	0.88	5.44	5.11	1.45	5.09	1.53	5.23	0.65	0.46	4.36	4.14	0.85	2.81	2.93	>0.999999999999999	1.0
P26583	High mobility group protein B2 OS=Homo sapiens GN=HMGB2 PE=1 SV=2	3.42	6.91	0.00	2.12	2.68	2.04	2.36	2.22	0.00	3.13	5.68	2.99	2.63	6.04	0.00	0.97	5.66	7.21	2.22	0.00	0.00	9.77	7.21	0.00	2.80	3.47	>0.999999999999999	0.8
Q08380	Galectin-3-binding protein OS=Homo sapiens GN=LGALS3BP PE=1 SV=1	1.22	3.69	1.79	5.16	1.92	6.84	2.70	1.59	0.83	1.04	5.11	1.59	0.88	2.01	1.78	1.10	1.89	2.45	0.79	2.72	1.45	5.61	3.22	1.19	2.79	2.09	>0.999999999999999	1.3
Q9Y6R7	IgGFc-binding protein OS=Homo sapiens GN=FCGBP PE=1 SV=3	3.66	5.53	2.91	5.00	1.34	3.06	4.05	3.49	0.75	0.10	0.76	2.79	2.54	8.46	4.22	3.24	1.32	2.61	3.33	5.70	0.31	1.25	1.38	3.29	2.79	3.14	>0.999999999999999	0.9
Q99471	Prefoldin subunit 5 OS=Homo sapiens GN=PFDN5 PE=1 SV=2	4.64	4.38	2.13	2.88	3.64	6.26	3.21	0.00	0.75	0.00	3.60	1.89	0.00	3.83	2.11	1.31	3.58	2.91	0.00	2.46	0.59	3.95	0.00	1.08	2.78	1.82	>0.999999999999999	1.5
P01701	Ig lambda chain V-I region NEW OS=Homo sapiens PE=1 SV=1	7.33	0.00	3.36	0.00	0.00	4.37	5.06	0.00	0.00	0.00	13.06	0.00	0.00	6.04	0.00	0.00	0.00	4.60	4.76	0.00	0.92	6.23	0.00	0.00	2.77	1.88	>0.999999999999999	1.5
P13693	Translationally-controlled tumor protein OS=Homo sapiens GN=TPT1 PE=1 SV=1	0.00	3.92	4.15	5.61	3.26	2.47	2.87	2.69	1.47	1.77	3.22	1.69	1.49	0.00	1.89	1.17	3.20	5.67	2.69	2.20	1.14	3.53	5.67	0.97	2.76	2.47	>0.999999999999999	1.1
P01608	Ig kappa chain V-I region Roy OS=Homo sapiens PE=1 SV=1	0.00	6.68	7.40	4.40	0.00	0.00	0.00	10.46	1.15	3.03	0.00	0.00	2.54	5.84	3.22	4.55	5.47	0.00	0.00	0.00	0.89	6.03	4.45	1.65	2.76	2.89	>0.999999999999999	1.0
P04207	Ig kappa chain V-III region CLL OS=Homo sapiens PE=1 SV=2	0.00	0.00	0.00	8.04	0.00	7.71	8.94	8.40	0.00	0.00	0.00	0.00	2.11	0.00	0.00	3.66	9.99	0.00	3.80	0.00	0.00	0.00	0.00	0.00	2.76	1.63	>0.999999999999999	1.7
Q16890	Tumor protein D53 OS=Homo sapiens GN=TPD52L1 PE=1 SV=1	7.82	3.46	1.68	0.00	0.00	4.66	5.40	0.00	2.06	0.00	2.84	5.18	0.00	0.00	0.00	0.00	0.00	0.00	0.00	4.15	0.46	3.12	2.30	2.95	2.76	1.08	>0.999999999999999	2.5
P14625	Endoplasmin OS=Homo sapiens GN=HSP90B1 PE=1 SV=1	3.66	2.54	3.14	1.06	1.34	7.57	1.18	6.66	0.44	2.40	2.08	0.70	3.25	4.63	1.22	0.76	1.32	2.30	3.65	4.15	0.22	3.12	2.91	1.31	2.73	2.40	>0.999999999999999	1.1
P09228	Cystatin-SA OS=Homo sapiens GN=CST2 PE=1 SV=1	18.07	0.00	0.00	0.00	0.00	0.00	0.00	11.73	2.93	0.00	0.00	0.00	3.86	0.00	0.00	0.00	3.77	0.00	0.00	0.00	0.00	0.00	0.00	0.00	2.73	0.64	>0.999999999999999	4.3
O95861	3'(2'),5'-bisphosphate nucleotidase 1 OS=Homo sapiens GN=BPNT1 PE=1 SV=1	11.23	4.84	1.12	1.52	0.00	4.66	1.69	0.00	0.40	0.00	3.98	3.19	1.84	4.23	0.00	0.69	0.00	1.53	0.00	2.72	0.31	2.08	0.00	1.82	2.72	1.27	>0.999999999999999	2.1
P08246	Neutrophil elastase OS=Homo sapiens GN=ELANE PE=1 SV=1	0.00	12.45	0.00	3.64	2.11	0.00	4.05	1.74	0.00	3.97	4.54	0.00	0.96	2.22	1.22	0.00	4.52	0.00	0.00	0.00	0.34	0.00	3.68	0.00	2.71	1.08	>0.999999999999999	2.5
P14868	Aspartate--tRNA ligase, cytoplasmic OS=Homo sapiens GN=DARS PE=1 SV=2	7.82	4.15	1.34	0.91	1.15	9.46	1.01	0.00	1.27	0.63	3.41	1.20	0.00	2.42	0.00	0.41	0.00	0.92	0.00	6.22	0.00	1.25	0.00	1.02	2.70	1.02	>0.999999999999999	2.6
P62310	U6 snRNA-associated Sm-like protein LSm3 OS=Homo sapiens GN=LSM3 PE=1 SV=2	7.08	6.68	3.25	4.40	0.00	4.22	0.00	0.00	1.15	0.00	5.49	0.00	2.54	5.84	0.00	2.00	0.00	0.00	0.00	3.76	0.00	0.00	0.00	0.00	2.69	1.18	>0.999999999999999	2.3
Q14247	Src substrate cortactin OS=Homo sapiens GN=CTTN PE=1 SV=2	9.04	3.92	4.15	0.76	0.96	5.39	1.86	2.69	1.19	0.00	0.00	2.29	0.44	3.42	1.22	2.07	0.94	3.53	0.79	2.98	0.34	0.00	0.77	1.70	2.69	1.52	>0.999999999999999	1.8
P61224	Ras-related protein Rap-1b OS=Homo sapiens GN=RAP1B PE=1 SV=1	0.00	0.00	1.79	2.43	0.00	8.15	0.00	0.00	0.00	5.84	10.60	3.39	0.00	3.22	0.00	0.00	0.00	0.00	0.00	0.00	0.00	16.83	8.59	1.93	2.68	2.55	>0.999999999999999	1.1
P01880	Ig delta chain C region OS=Homo sapiens GN=IGHD PE=1 SV=2	6.11	0.00	6.27	3.79	6.71	1.16	0.00	0.00	0.63	0.00	6.63	0.80	1.40	1.61	7.55	3.86	3.02	0.00	0.00	0.00	0.00	0.00	2.45	0.45	2.68	1.70	>0.999999999999999	1.6
P02545	Prelamin-A/C OS=Homo sapiens GN=LMNA PE=1 SV=1	2.20	3.23	1.01	3.64	0.00	2.04	3.21	0.63	0.00	7.83	7.76	0.40	1.23	4.83	2.67	0.00	0.75	2.15	5.55	1.17	0.28	2.91	2.15	0.23	2.66	1.99	>0.999999999999999	1.3
P47914	60S ribosomal protein L29 OS=Homo sapiens GN=RPL29 PE=1 SV=2	4.64	4.38	2.13	2.88	3.64	2.77	3.21	0.00	0.75	1.98	3.60	1.89	1.67	3.83	2.11	1.31	0.00	0.00	3.01	2.46	0.59	0.00	2.91	1.08	2.66	1.58	>0.999999999999999	1.7
Q08257	Quinone oxidoreductase OS=Homo sapiens GN=CRYZ PE=1 SV=1	0.00	16.36	0.00	0.00	0.00	6.26	0.00	4.91	1.23	0.00	0.00	3.09	0.00	1.81	0.00	0.62	0.00	1.38	0.00	5.57	0.00	0.00	0.00	0.51	2.65	0.82	>0.999999999999999	3.2
P62191	26S protease regulatory subunit 4 OS=Homo sapiens GN=PSMC1 PE=1 SV=1	5.13	3.23	0.00	0.00	4.03	8.30	2.36	1.11	0.28	0.00	2.65	4.68	0.61	4.23	0.00	0.97	0.00	2.15	1.11	1.81	0.65	1.45	0.00	1.19	2.65	1.18	>0.999999999999999	2.2
P63173	60S ribosomal protein L38 OS=Homo sapiens GN=RPL38 PE=1 SV=2	0.00	0.00	4.71	0.00	0.00	6.11	7.08	0.00	1.67	0.00	7.95	4.18	0.00	8.46	0.00	0.00	0.00	0.00	0.00	5.44	1.29	0.00	0.00	2.38	2.64	1.47	>0.999999999999999	1.8
O95433	Activator of 90 kDa heat shock protein ATPase homolog 1 OS=Homo sapiens GN=AHSA1 F	6.84	6.45	2.02	2.73	1.73	2.62	3.04	1.43	0.36	0.00	1.70	2.79	1.58	5.64	0.00	0.62	0.00	1.38	0.00	3.63	0.00	1.87	0.00	0.51	2.64	1.27	>0.999999999999999	2.1
P05164	Myeloperoxidase OS=Homo sapiens GN=MPO PE=1 SV=1	2.93	8.07	0.45	2.43	1.53	1.75	3.54	3.33	0.16	6.68	0.76	0.00	1.40	6.04	0.89	0.55	3.96	1.23	0.00	0.52	0.12	10.81	2.45	0.00	2.64	2.33	>0.999999999999999	1.1
P55769	NHP2-like protein 1 OS=Homo sapiens GN=NHP2L1 PE=1 SV=3	5.86	0.00	2.69	3.64	0.00	0.00	4.05	0.00	0.00	0.00	10.04	5.28	0.00	0.00	2.67	1.66	0.00	0.00	0.00	3.11	0.00	0.00	0.00	1.36	2.63	0.73	>0.999999999999999	3.6
A0M8Q6	Ig lambda-7 chain C region OS=Homo sapiens GN=IGLC7 PE=1 SV=2	0.00	0.00	0.00	0.00	0.00	0.00	31.55	0.00	0.00	0.00	0.00	0.00	23.95	0.00	0.00	0.00	0.00	0.00	61.03	0.00	0.00	0.00	0.00	0.00	2.63	7.08	>0.999999999999999	0.4
P62906	60S ribosomal protein L10a OS=Homo sapiens GN=RPL10A PE=1 SV=2	0.00	3.00	1.46	1.97	2.49	9.46	2.19	2.06	0.52	0.00	5.49	2.89	0.00	2.62	1.44	0.90	2.45	1.99	2.06	5.96	0.00	2.70	1.99	0.74	2.63	1.90	>0.999999999999999	1.4
O43242	26S proteasome non-ATPase regulatory subunit 3 OS=Homo sapiens GN=PSMD3 PE=1 S\	2.69	5.30	4.15	0.76	0.00	8.73	0.84	3.65	1.47	0.00	2.08	1.69	0.44	3.42	3.33	1.17	0.94	3.53	0.79	4.79	0.71	4.78	1.69	0.62	2.61	2.19	>0.999999999999999	1.2
O60749	Sorting nexin-2 OS=Homo sapiens GN=SNX2 PE=1 SV=2	5.86	5.53	0.00	0.00	3.45	5.68	0.00	2.85	0.95	0.00	4.54	2.39	1.05	2.42	0.00	0.83	0.00	1.84	0.00	4.02	0.37	1.25	0.00	1.36	2.61	1.09	>0.999999999999999	2.4
Q00577	Transcriptional activator protein Pur-alpha OS=Homo sapiens GN=PURA PE=1 SV=2	10.75	0.00	2.24	1.36	1.73	4.51	0.00	0.00	0.36	0.00	5.87	4.38	1.75	0.00	1.00	0.62	0.00	0.00	0.00	2.59	0.00	0.00	0.00	0.51	2.60	0.54	>0.999999999999999	4.8
P36959	GMP reductase 1 OS=Homo sapiens GN=GMPR PE=1 SV=1	7.08	4.15	2.02	2.73	5.56	2.62	0.00	0.00	0.71	0.00	3.41	2.89	0.79	0.00	0.00	0.00	0.00	0.00	0.00	0.00	0.00	0.00	0.00	0.00	2.60	0.07	>0.999999999999999	39.5
Q9Y3F4	Serine-threonine kinase receptor-associated protein OS=Homo sapiens GN=STRAP PE=1 S	2.20	4.15	2.02	2.73	1.73	5.68	0.00	4.44	1.11	0.00	5.30	1.79	0.79	3.63	1.00	1.93	0.00	2.76	2.85	6.61	0.28	3.74	1.38	1.02	2.59	2.17	>0.999999999999999	1.2
Q9BT78	COP9 signalosome complex subunit 4 OS=Homo sapiens GN=COPS4 PE=1 SV=1	5.62	3.46	4.59	2.27	0.00	4.66	0.00	1.11	0.91	0.00	6.06	2.29	0.00	8.26	1.67	1.59	0.00	0.00	0.00	4.15	0.22	0.00	0.00	1.31	2.58	1.43	>0.999999999999999	1.8
P99999	Cytochrome c OS=Homo sapiens GN=CYCS PE=1 SV=2	7.08	0.00	0.00	0.00	0.00	16.59	0.00	4.60	2.62	0.00	0.00	0.00	0.00	5.84	0.00	0.00	0.00	0.00	0.00	8.55	0.00	0.00	0.00	1.65	2.57	1.34	>0.999999999999999	1.9
Q92747	Actin-related protein 2/3 complex subunit 1A OS=Homo sapiens GN=ARPC1A PE=1 SV=2	6.11	5.76	2.80	2.43	1.53	3.64	0.00	2.54	1.39	0.00	0.00	4.58	0.00	5.04	0.00	1.10	0.00	1.23	0.00	2.07	0.77	3.32	0.00	0.00	2.56	1.13	>0.999999999999999	2.3
Q9UII2	ATPase inhibitor, mitochondrial OS=Homo sapiens GN=ATPIF1 PE=1 SV=1	6.84	6.45	3.14	0.00	0.00	4.08	0.00	10.15	0.00	0.00	0.00	0.00	0.00	0.00	0.00	0.00	0.00	0.00	0.00	0.00	0.00	0.00	0.00	0.00	2.55	0.00	>0.999999999999999	#DIV/0!
P08134	Rho-related GTP-binding protein RhoC OS=Homo sapiens GN=RHOC PE=1 SV=1	7.82	11.99	0.00	0.00	0.00	0.00	5.40	0.00	0.00	5.43	0.00	0.00	0.00	0.00	8.33	3.59	0.00	0.00	5.07	6.74	0.00	10.81	11.50	0.00	2.55	3.84	>0.999999999999999	0.7
Q9Y3C4	TP53RK-binding protein OS=Homo sapiens GN=TPRKB PE=1 SV=1	14.65	3.92	0.00	0.00	0.00	2.47	2.87	0.00	0.67	0.00	0.00	5.98	0.00	3.42	0.00	0.00	0.00	0.00	0.00	2.20	0.00	0.00	2.61	0.97	2.55	0.77	>0.999999999999999	3.3
P55060	Exportin-2 OS=Homo sapiens GN=CSE1L PE=1 SV=3	6.35	2.07	1.34	1.82	1.73	5.53	0.51	0.95	0.36	0.00	5.68	4.18	0.26	2.42	0.67	0.41	0.00	0.46	0.00	2.07	0.18	1.87	0.00	0.68	2.54	0.75	>0.999999999999999	3.4
P20042	Eukaryotic translation initiation factor 2 subunit 2 OS=Homo sapiens GN=EIF2S2 PE=1 SV=	2.20	4.15	2.02	0.00	3.45	7.42	1.52	4.44	0.71	0.00	1.70	2.79	0.79	5.64	1.00	1.93	1.70	1.38	2.85	3.63	0.86	0.00	1.38	1.02	2.53	1.85	>0.999999999999999	1.4
P38606	V-type proton ATPase catalytic subunit A OS=Homo sapiens GN=ATP6V1A PE=1 SV=2	6.35	3.46	2.35	3.18	1.92	3.06	0.84	1.59	0.59	1.57	3.98	1.49	0.88	2.01	1.11	1.79	0.00	2.30	0.79	5.05	0.15	8.10	0.77	1.19	2.53	2.01	>0.999999999999999	1.3
Q13564	NEDD8-activating enzyme E1 regulatory subunit OS=Homo sapiens GN=NAE1 PE=1 SV=1	10.99	2.54	1.23	1.67	2.11	6.55	0.00	0.00	0.20	0.00	2.08	2.99	1.49	2.22	0.00	1.17	0.00	0.77	0.00	1.43	0.00	1.04	0.00	0.62	2.53	0.73	>0.999999999999999	3.5
P13671	Complement component C6 OS=Homo sapiens GN=C6 PE=1 SV=3	3.91	3.69	3.03	5.91	6.71	0.00	2.19	0.95	0.24	1.36	1.70	0.60	1.75	4.03	4.33	2.14	6.60	0.92	5.55	0.39	0.40	8.94	1.38	1.14	2.52	3.13	>0.999999999999999	0.8
P62312	U6 snRNA-associated Sm-like protein LSm6 OS=Homo sapiens GN=LSM6 PE=1 SV=1	9.28	0.00	4.26	0.00	0.00	5.53	0.00	0.00	0.00	0.00	7.20	3.79	0.00	7.65	0.00	0.00	0.00	5.83	0.00	4.92	1.17	7.90	0.00	2.16	2.50	2.47	>0.999999999999999	1.0
P35606	Coatomer subunit beta' OS=Homo sapiens GN=COPB2 PE=1 SV=2	4.15	4.84	3.14	2.58	0.58	8.01	0.51	0.95	1.27	0.00	1.89	2.09	1.14	4.23	1.11	0.90	0.00	1.99	0.48	3.63	0.31	0.62	1.53	0.74	2.50	1.39	>0.999999999999999	1.8
P20591	Interferon-induced GTP-binding protein Mx1 OS=Homo sapiens GN=MX1 PE=1 SV=4	4.40	5.53	2.69	0.00	1.73	8.59	0.67	0.63	2.62	0.00	1.70	1.39	1.23	1.81	1.00	0.97	0.75	0.00	0.00	1.17	0.89	0.83	0.00	0.23	2.50	0.74	>0.999999999999999	3.4
Q9UBQ0	Vacuolar protein sorting-associated protein 29 OS=Homo sapiens GN=VPS29 PE=1 SV=1	8.55	0.00	1.79	0.00	0.00	8.30	0.00	0.00	1.39	1.67	6.63	1.59	1.40	7.05	0.00	1.10	0.00	2.45	2.54	7.38	0.00	3.32	0.00	0.91	2.49	2.18	>0.999999999999999	1.1
Q01813	6-phosphofructokinase type C OS=Homo sapiens GN=PFKP PE=1 SV=2	3.91	2.77	2.24	1.82	1.53	7.42	2.02	3.17	0.99	0.00	1.51	2.49	1.75	4.03	1.33	1.38	0.75	1.84	0.00	4.53	0.00	1.66	0.61	0.68	2.49	1.55	>0.999999999999999	1.6
P02751	Fibronectin OS=Homo sapiens GN=FN1 PE=1 SV=4	1.47	1.38	1.90	6.22	8.05	0.15	3.37	0.16	0.16	2.92	3.60	0.50	2.46	3.83	6.78	2.21	8.30	2.45	4.91	0.26	0.89	3.32	3.37	0.51	2.49	3.27	>0.999999999999999	0.8
P07358	Complement component C8 beta chain OS=Homo sapiens GN=C8B PE=1 SV=3	3.66	1.15	2.91	5.91	11.51	0.00	1.69	0.79	0.20	0.52	0.00	1.49	0.44	5.24	4.33	1.45	2.83	0.00	5.07	1.94	0.31	2.08	0.77	0.57	2.49	2.09	>0.999999999999999	1.2
P61026	Ras-related protein Rab-10 OS=Homo sapiens GN=RAB10 PE=1 SV=1	0.00	11.76	0.00	0.00	0.00	0.00	0.00	0.00	0.00	3.34	9.66	5.08	0.00	10.27	0.00	0.00	0.00	0.00	0.00	6.61	0.00	0.00	0.00	0.00	2.49	1.41	>0.999999999999999	1.8
Q8TCD5	5'(3')-deoxyribonucleotidase, cytosolic type OS=Homo sapiens GN=NT5C PE=1 SV=2	11.97	3.23	1.57	0.00	0.00	4.37	0.00	0.00	0.00	0.00	5.68	2.99	1.23	2.82	0.00	0.97	0.00	2.15	0.00	3.89	0.92	2.91	2.15	1.70	2.48	1.56	>0.999999999999999	1.6
P35221	Catenin alpha-1 OS=Homo sapiens GN=CTNNA1 PE=1 SV=1	7.08	3.23	2.80	1.06	1.34	3.06	1.69	3.33	0.83	0.00	3.22	2.09	0.00	5.04	1.11	1.72	0.57	3.22	1.59	3.76	0.22	1.45	0.46	0.79	2.48	1.66	>0.999999999999999	1.5
P14866	Heterogeneous nuclear ribonucleoprotein L OS=Homo sapiens GN=HNRNPL PE=1 SV=2	3.91	5.07	3.92	3.34	0.96	3.20	0.00	0.79	1.11	0.52	5.30	1.59	0.00	2.01	0.56	0.69	0.00	1.53	0.00	2.85	0.31	1.04	0.00	0.00	2.48	0.75	>0.999999999999999	3.3
Q13126	S-methyl-5'-thioadenosine phosphorylase OS=Homo sapiens GN=MTAP PE=1 SV=2	2.44	5.07	2.47	3.34	0.00	9.32	0.00	1.59	1.39	0.00	1.89	2.19	0.88	0.00	0.00	0.00	0.00	0.00	0.00	1.30	0.31	4.57	1.53	1.25	2.47	0.82	>0.999999999999999	3.0
P49419	Alpha-aminoadipic semialdehyde dehydrogenase OS=Homo sapiens GN=ALDH7A1 PE=1 S	4.40	5.53	2.02	1.67	0.00	5.53	0.00	2.85	0.71	0.00	4.54	2.39	0.00	3.63	0.67	0.41	0.00	1.69	0.00	4.92	0.34	1.25	0.92	1.36	2.47	1.27	>0.999999999999999	2.0
P62140	Serine/threonine-protein phosphatase PP1-beta catalytic subunit OS=Homo sapiens GN=PF	0.00	0.00	0.00	0.00	0.00	9.46	4.89	0.00	0.00	0.00	9.85	5.18	0.00	10.48	0.00	0.00	0.00	0.00	0.00	0.00	0.00	0.00	0.00	1.65	2.45	1.01	>0.999999999999999	2.4
P32969	60S ribosomal protein L9 OS=Homo sapiens GN=RPL9 PE=1 SV=1	3.66	7.61	1.68	0.00	2.88	4.80	0.00	2.38	0.00	0.00	6.25	0.00	0.00	0.00	1.67	1.03	0.00	2.30	0.00	4.28	1.02	0.00	0.00	0.00	2.44	0.86	>0.999999999999999	2.8
Q9NUQ9	Protein FAM49B OS=Homo sapiens GN=FAM49B PE=1 SV=1	4.64	4.38	2.13	1.36	3.64	4.22	0.00	0.00	0.36	3.03	3.60	1.89	0.00	0.00	1.00	1.31	1.70	1.38	0.00	2.46	0.59	17.04	2.91	0.00	2.44	2.37	>0.999999999999999	1.0
Q99460	26S proteasome non-ATPase regulatory subunit 1 OS=Homo sapiens GN=PSMD1 PE=1 S\	6.59	7.15	2.24	0.45	1.73	4.51	0.51	0.95	0.52	0.00	2.46	1.99	0.00	4.03	0.67	1.66	0.57	1.99	0.48	4.02	0.18	4.16	0.46	1.14	2.43	1.61	>0.999999999999999	1.5
Q9NP97	Dynein light chain roadblock-type 1 OS=Homo sapiens GN=DYNLRB1 PE=1 SV=3	7.82	7.38	0.00	0.00	0.00	4.66	0.00	0.00	0.00	0.00	6.06	3.19	0.00	6.45	0.00	0.00	0.00	0.00	0.00	0.00	0.00	0.00	0.00	1.82	2.42	0.69	>0.999999999999999	3.5
Q12906	Interleukin enhancer-binding factor 3 OS=Homo sapiens GN=ILF3 PE=1 SV=3	4.40	4.15	1.57	2.12	1.34	4.51	1.18	1.74	0.44	0.31	5.11	2.19	0.26	3.63	0.33	0.97	0.00	1.69	0.00	1.81	0.09	1.45	1.07	0.62	2.42	0.99	>0.999999999999999	2.4
P61353	60S ribosomal protein L27 OS=Homo sapiens GN=RPL27 PE=1 SV=2	11.72	0.00	5.38	0.00	0.00	3.20	0.00	0.00	0.87	0.00	0.00	7.87	0.00	0.00	0.00	3.31	0.00	0.00	0.00	2.85	0.68	0.00	0.00	4.49	2.42	0.94	>0.999999999999999	2.6
Q96C74	Ropporin-1-like protein OS=Homo sapiens GN=ROPN1L PE=1 SV=2	6.59	3.00	0.00	0.00	2.49	6.26	2.19	2.06	0.00	0.00	5.11	1.29	0.00	2.62	0.00	0.00	0.00	0.00	0.00	1.68	0.00	0.00	0.00	0.00	2.42	0.36	>0.999999999999999	6.7
P14621	Acylphosphatase-2 OS=Homo sapiens GN=ACYP2 PE=1 SV=2	0.00	7.15	3.47	0.00	0.00	4.51	0.00	4.91	0.00	0.00	5.87	3.09	0.00	0.00	0.00	2.14	0.00	0.00	0.00	4.02	0.00	0.00	0.00	0.00	2.42	0.51	>0.999999999999999	4.7
P04259	Keratin, type II cytoskeletal 6B OS=Homo sapiens GN=KRT6B PE=1 SV=5	0.00	0.00	0.00	0.00	0.00	0.00	0.00	0.00	0.00	28.91	0.00	0.00	0.00	0.00	0.00	0.00	3.20	0.00	0.00	0.00	1.17	0.00	3.68	0.00	2.41	0.67	>0.999999999999999	3.6
Q9H444	Charged multivesicular body protein 4b OS=Homo sapiens GN=CHMP4B PE=1 SV=1	3.18	0.00	0.00	1.97	2.49	6.70	2.19	2.06	0.52	1.36	5.49	2.89	1.14	0.00	0.00	0.90	0.00	1.99	2.06	0.00	0.00	2.70	1.99	0.74	2.40	0.96	>0.999999999999999	2.5
P50552	Vasodilator-stimulated phosphoprotein OS=Homo sapiens GN=VASP PE=1 SV=3	0.00	1.84	0.00	2.58	3.26	2.47	1.35	2.69	0.67	3.86	9.28	0.80	0.70	1.61	0.89	0.55	6.98	1.23	1.27	2.20	0.00	10.18	5.67	0.45	2.40	2.64	>0.999999999999999	0.9
P02654	Apolipoprotein C-I OS=Homo sapiens GN=APOC1 PE=1 SV=1	0.00	0.00	0.00	5.61	7.10	0.00	6.24	5.87	0.00	3.86	0.00	0.00	3.25	0.00	4.11	0.00	6.98	5.67	14.11	4.79	0.00	7.69	5.67	0.00	2.39	4.36	>0.999999999999999	0.5
Q99536	Synaptic vesicle membrane protein VAT-1 homolog OS=Homo sapiens GN=VAT1 PE=1 SV	3.91	1.84	1.79	3.79	3.07	3.64	2.70	1.27	0.32	1.67	3.03	1.59	0.70	1.61	0.89	1.10	3.02	2.45	0.00	1.04	0.49	7.27	5.37	0.45	2.39	2.03	>0.999999999999999	1.2
Q15691	Microtubule-associated protein RP/EB family member 1 OS=Homo sapiens GN=MAPRE1 PI	2.69	2.54	4.15	1.67	2.11	3.35	3.88	0.00	0.44	1.15	4.35	2.29	0.00	4.63	1.22	2.55	2.07	5.67	0.00	2.98	0.34	2.29	0.00	1.31	2.38	1.92	>0.999999999999999	1.2
P45974	Ubiquitin carboxyl-terminal hydrolase 5 OS=Homo sapiens GN=USP5 PE=1 SV=2	5.37	5.07	2.02	1.67	1.34	5.09	1.86	1.74	0.87	0.00	1.33	2.19	1.58	2.82	0.33	1.24	0.00	3.37	0.00	3.37	0.34	1.45	0.46	1.02	2.38	1.33	>0.999999999999999	1.8
Q96TA1	Niban-like protein 1 OS=Homo sapiens GN=FAM129B PE=1 SV=3	7.33	2.77	2.35	1.82	1.53	2.33	1.35	2.54	0.83	0.42	2.27	2.99	0.70	6.04	1.33	1.79	1.51	3.22	1.90	5.31	0.65	2.49	1.23	0.91	2.38	2.26	>0.999999999999999	1.1
P16401	Histone H1.5 OS=Homo sapiens GN=HIST1H1B PE=1 SV=3	0.00	3.46	0.00	7.89	0.00	0.00	5.40	2.38	0.00	3.34	6.06	0.00	1.32	6.45	1.67	0.00	0.00	0.00	0.00	0.00	0.00	6.65	4.91	0.00	2.38	1.75	>0.999999999999999	1.4
P31689	DnaJ homolog subfamily A member 1 OS=Homo sapiens GN=DNAJA1 PE=1 SV=2	5.62	5.30	1.68	1.06	1.34	4.66	2.53	0.00	0.28	0.00	2.84	3.19	0.61	4.63	0.78	0.00	0.00	0.00	0.00	1.94	0.22	3.12	2.30	0.00	2.37	1.13	>0.999999999999999	2.1
Q9GZS3	WD repeat-containing protein 61 OS=Homo sapiens GN=WDR61 PE=1 SV=1	11.23	4.84	2.35	0.00	0.00	3.06	0.00	1.59	0.40	0.00	3.98	1.00	0.00	4.23	0.00	0.00	0.00	0.00	0.00	2.72	0.00	0.00	0.00	0.57	2.37	0.63	>0.999999999999999	3.8
Q14204	Cytoplasmic dynein 1 heavy chain 1 OS=Homo sapiens GN=DYNC1H1 PE=1 SV=5	3.91	4.61	2.13	1.21	1.92	5.09	1.52	0.95	0.75	0.21	3.98	2.09	0.61	3.22	0.44	0.55	0.19	1.07	0.32	2.07	0.18	1.25	0.46	0.68	2.36	0.92	>0.999999999999999	2.6
A6NL28	Putative tropomyosin alpha-3 chain-like protein OS=Homo sapiens PE=5 SV=2	3.18	3.00	1.46	4.09	2.49	1.89	4.55	2.06	0.52	1.36	2.46	1.29	1.14	2.62	1.44	0.90	0.00	1.99	0.00	1.68	0.40	2.70	1.99	0.74	2.36	1.30	>0.999999999999999	1.8
P53634	Dipeptidyl peptidase 1 OS=Homo sapiens GN=CTSC PE=1 SV=2	4.88	4.61	2.24	1.97	1.15	5.09	1.01	3.17	1.07	0.00	1.14	1.99	2.37	5.44	2.22	1.86	2.45	4.14	0.95	3.50	0.18	1.25	0.00	1.53	2.36	2.16	>0.999999999999999	1.1
O00571	ATP-dependent RNA helicase DDX3X OS=Homo sapiens GN=DDX3X PE=1 SV=3	5.86	0.92	1.57	1.36	1.73	6.11	1.52	2.22	0.95	0.42	2.65	2.99	0.35	2.82	1.00	1.31	0.00	1.38	2.22	4.66	0.59	0.83	0.61	1.08	2.36	1.40	>0.999999999999999	1.7
P48163	NADP-dependent malic enzyme OS=Homo sapiens GN=ME1 PE=1 SV=1	6.84	5.07	1.12	0.76	1.92	5.09	1.69	0.79	0.40	0.00	3.03	1.59	0.44	9.87	1.11	0.69	0.94	1.53	0.00	2.85	0.00	1.04	0.00	0.57	2.36	1.59	>0.999999999999999	1.5
Q96QK1	Vacuolar protein sorting-associated protein 35 OS=Homo sapiens GN=VPS35 PE=1 SV=2	4.64	4.38	1.23	0.00	0.77	7.57	1.86	0.63	0.75	0.00	4.35	1.89	0.61	4.63	0.44	0.76	0.00	0.61	0.63	2.46	0.22	1.45	0.00	1.31	2.34	1.09	>0.999999999999999	2.1
Q14558	Phosphoribosyl pyrophosphate synthase-associated protein 1 OS=Homo sapiens GN=PRPS	4.15	6.22	0.90	0.00	1.53	9.02	0.00	0.00	0.32	0.00	3.22	2.69	0.00	3.42	0.00	0.00	0.00	0.00	0.00	0.00	0.00	1.45	0.00	0.00	2.34	0.29	>0.999999999999999	8.2
Q14974	Importin subunit beta-1 OS=Homo sapiens GN=KPNB1 PE=1 SV=2	5.37	4.15	2.91	1.52	0.58	4.37	1.18	1.11	0.87	0.00	3.41	2.59	0.61	3.63	2.00	1.24	0.00	1.07	1.11	3.37	0.43	6.23	3.37	1.02	2.34	2.01	>0.999999999999999	1.2
P62829	60S ribosomal protein L23 OS=Homo sapiens GN=RPL23 PE=1 SV=1	5.62	11.76	0.00	0.00	0.00	7.42	0.00	0.00	0.91	0.00	0.00	2.29	0.00	4.63	0.00	1.59	0.00	7.82	0.00	6.61	0.00	0.00	0.00	1.31	2.33	1.83	>0.999999999999999	1.3
P62910	60S ribosomal protein L32 OS=Homo sapiens GN=RPL32 PE=1 SV=2	11.48	0.00	2.35	3.18	4.03	6.84	0.00	0.00	0.00	0.00	0.00	0.00	0.00	0.00	0.00	1.45	0.00	0.00	0.00	0.00	0.00	0.00	0.00	1.19	2.32	0.22	>0.999999999999999	10.6
P16989	DNA-binding protein A OS=Homo sapiens GN=CSDA PE=1 SV=4	4.15	1.84	3.03	2.58	3.26	5.39	0.00	2.69	0.00	0.00	3.22	1.69	1.49	3.42	0.00	0.00	0.00	2.61	2.69	2.20	0.00	0.00	0.00	0.00	2.32	1.04	>0.999999999999999	2.2
O43237	Cytoplasmic dynein 1 light intermediate chain 2 OS=Homo sapiens GN=DYNC1LI2 PE=1 SV	4.64	6.22	1.34	0.91	2.30	2.77	3.21	1.90	0.24	0.00	2.27	1.89	0.53	1.21	0.67	0.41	0.00	0.00	0.00	2.46	0.00	0.00	0.92	0.00	2.31	0.52	>0.999999999999999	4.5
P17612	cAMP-dependent protein kinase catalytic subunit alpha OS=Homo sapiens GN=PRKACA PE	6.35	1.84	1.90	1.21	1.53	5.39	1.35	1.27	1.03	0.00	3.22	2.59	0.00	1.61	0.89	0.55	1.51	1.23	0.00	0.00	0.00	1.66	0.00	0.00	2.31	0.62	>0.999999999999999	3.7
O95834	Echinoderm microtubule-associated protein-like 2 OS=Homo sapiens GN=EML2 PE=2 SV=1	6.11	3.23	2.24	0.76	2.68	2.91	0.00	0.79	0.36	0.52	3.79	4.28	0.44	1.81	1.00	0.62	0.94	0.00	0.79	1.17	0.28	2.91	1.38	0.79	2.31	1.01	>0.999999999999999	2.3
P54578	Ubiquitin carboxyl-terminal hydrolase 14 OS=Homo sapiens GN=USP14 PE=1 SV=3	2.93	4.38	2.80	1.82	1.15	3.64	1.01	1.90	0.75	0.63	4.73	1.89	1.05	5.04	0.67	1.72	1.13	2.91	0.00	3.24	0.37	2.49	0.00	1.08	2.30	1.64	>0.999999999999999	1.4
Q9Y3C8	Ubiquitin-fold modifier-conjugating enzyme 1 OS=Homo sapiens GN=UFC1 PE=1 SV=3	4.15	3.92	1.90	0.00	0.00	5.39	2.87	0.00	0.67	0.00	7.01	1.69	1.49	3.42	0.00	0.00	0.00	2.61	0.00	4.79	0.52	7.69	0.00	0.00	2.30	1.71	>0.999999999999999	1.3
O43488	Aflatoxin B1 aldehyde reductase member 2 OS=Homo sapiens GN=AKR7A2 PE=1 SV=3	6.59	6.22	0.00	1.21	0.00	5.39	0.00	1.27	0.00	0.00	3.22	3.69	0.00	0.00	1.89	1.17	0.00	0.00	0.00	4.79	0.00	0.00	2.61	0.97	2.30	0.95	>0.999999999999999	2.4
P33241	Lymphocyte-specific protein 1 OS=Homo sapiens GN=LSP1 PE=1 SV=1	2.20	4.15	0.00	2.73	3.45	4.22	1.52	2.85	0.00	3.03	3.41	0.00	1.58	3.63	1.00	0.62	3.39	1.38	0.00	0.00	0.00	13.71	6.13	0.00	2.30	2.62	>0.999999999999999	0.9
P17900	Ganglioside GM2 activator OS=Homo sapiens GN=GM2A PE=1 SV=4	3.91	3.69	1.79	2.43	0.00	0.00	2.70	5.39	1.35	1.67	3.03	1.59	1.40	3.22	1.78	1.10	3.02	2.45	2.54	2.07	0.49	7.07	0.00	0.91	2.30	2.17	>0.999999999999999	1.1
P01604	Ig kappa chain V-I region Kue OS=Homo sapiens PE=1 SV=1	0.00	0.00	0.00	4.25	5.37	0.00	4.72	10.15	0.00	2.92	0.00	0.00	0.00	0.00	0.00	0.00	0.00	0.00	0.00	0.00	0.00	5.82	0.00	0.00	2.28	0.48	>0.999999999999999	4.7
Q03591	Complement factor H-related protein 1 OS=Homo sapiens GN=CFHR1 PE=1 SV=2	0.00	6.45	4.37	7.73	3.45	0.00	0.00	0.00	0.00	1.88	1.70	1.79	0.00	5.64	5.67	1.93	0.00	0.00	0.00	3.63	0.00	5.82	0.00	1.02	2.28	1.98	>0.999999999999999	1.2
Q16378	Proline-rich protein 4 OS=Homo sapiens GN=PRR4 PE=1 SV=3	0.00	19.36	0.00	0.00	0.00	0.00	0.00	7.93	0.00	0.00	0.00	0.00	0.00	0.00	0.00	3.45	0.00	0.00	0.00	10.88	0.00	10.39	0.00	0.00	2.27	2.06	>0.999999999999999	1.1
P11586	C-1-tetrahydrofolate synthase, cytoplasmic OS=Homo sapiens GN=MTHFD1 PE=1 SV=3	5.13	3.92	2.80	0.45	0.58	3.64	1.69	1.59	1.31	0.00	4.73	1.29	1.14	2.62	1.11	1.17	0.00	2.61	0.00	4.28	0.52	2.70	0.46	1.19	2.26	1.48	>0.999999999999999	1.5
P50135	Histamine N-methyltransferase OS=Homo sapiens GN=HNMT PE=1 SV=1	8.06	4.84	3.70	1.52	0.00	1.46	1.69	0.00	0.83	0.00	3.98	1.00	1.84	4.23	2.33	2.28	0.00	1.53	0.00	4.28	0.31	4.36	1.53	1.19	2.26	1.99	>0.999999999999999	1.1
P26639	Threonine--tRNA ligase, cytoplasmic OS=Homo sapiens GN=TARS PE=1 SV=3	5.13	3.69	1.34	0.00	1.53	7.57	1.35	0.63	1.03	0.42	2.27	2.09	1.05	3.22	0.44	0.83	0.00	0.61	0.63	1.55	0.49	4.36	1.23	0.91	2.26	1.28	>0.999999999999999	1.8
O15305	Phosphomannomutase 2 OS=Homo sapiens GN=PMM2 PE=1 SV=1	6.11	2.77	1.34	0.00	0.00	5.68	2.02	0.00	0.48	0.00	4.73	3.88	0.00	7.86	0.00	0.83	0.00	1.84	1.90	7.26	0.00	8.10	1.84	2.21	2.25	2.65	>0.999999999999999	0.8
P54727	UV excision repair protein RAD23 homolog B OS=Homo sapiens GN=RAD23B PE=1 SV=1	6.11	3.69	1.79	0.00	3.07	3.64	0.00	2.54	0.63	0.00	3.03	2.49	0.00	0.00	2.78	1.10	1.51	2.45	1.27	2.07	0.49	3.32	0.00	1.42	2.25	1.37	>0.999999999999999	1.6
O75832	26S proteasome non-ATPase regulatory subunit 10 OS=Homo sapiens GN=PSMD10 PE=1	3.42	6.68	3.25	2.12	0.00	2.04	0.00	2.22	0.00	0.00	2.65	4.58	0.00	2.82	1.56	0.97	0.00	2.15	0.00	3.76	0.43	0.00	2.15	0.79	2.25	1.22	>0.999999999999999	1.8
Q9UHD8	Septin-9 OS=Homo sapiens GN=SEPT9 PE=1 SV=2	5.37	3.69	2.47	0.00	1.92	4.08	0.84	0.79	0.20	0.00	5.30	2.19	0.44	2.01	0.56	0.34	0.00	0.77	0.00	1.30	0.00	0.00	0.00	0.00	2.24	0.45	>0.999999999999999	5.0
P54652	Heat shock-related 70 kDa protein 2 OS=Homo sapiens GN=HSPA2 PE=1 SV=1	9.28	0.00	5.71	0.00	0.00	11.79	0.00	0.00	0.00	0.00	0.00	0.00	0.00	0.00	0.00	0.00	0.00	0.00	0.00	0.00	0.00	0.00	0.00	0.00	2.23	0.00	>0.999999999999999	#DIV/0!
Q9NTK5	Obg-like ATPase 1 OS=Homo sapiens GN=OLA1 PE=1 SV=2	5.86	3.46	2.69	1.06	1.34	6.26	1.18	0.00	0.59	0.00	2.84	1.49	0.00	1.41	0.78	1.03	0.00	1.07	0.00	0.91	0.22	1.45	0.00	0.00	2.23	0.57	>0.999999999999999	3.9
P02753	Retinol-binding protein 4 OS=Homo sapiens GN=RBP4 PE=1 SV=3	3.42	3.23	1.57	2.12	2.68	0.00	5.23	2.22	0.56	0.00	2.65	3.09	0.00	0.00	1.56	0.97	5.84	0.00	2.22	0.00	0.95	2.91	4.75	0.79	2.23	1.67	>0.999999999999999	1.3
P04438	Ig heavy chain V-II region SESS OS=Homo sapiens PE=2 SV=1	0.00	4.84	0.00	3.18	4.03	0.00	3.54	3.33	0.00	7.83	0.00	0.00	1.84	0.00	5.00	1.45	3.96	0.00	0.00	0.00	0.00	9.35	0.00	0.00	2.23	1.80	>0.999999999999999	1.2
P06331	Ig heavy chain V-II region ARH-77 OS=Homo sapiens PE=4 SV=1	5.13	4.84	2.35	3.18	0.00	3.06	0.00	3.33	0.83	0.00	3.98	0.00	0.00	9.07	0.00	0.00	3.96	0.00	0.00	0.00	0.00	0.00	0.00	0.00	2.23	1.09	>0.999999999999999	2.1
Q6NVV1	Putative 60S ribosomal protein L13a-like MGC87657 OS=Homo sapiens PE=5 SV=1	6.84	0.00	0.00	0.00	5.37	0.00	4.72	4.44	0.00	0.00	5.30	0.00	2.46	0.00	0.00	0.00	0.00	0.00	4.44	0.00	0.86	0.00	0.00	0.00	2.22	0.65	>0.999999999999999	3.4
O43768	Alpha-endosulfine OS=Homo sapiens GN=ENSA PE=1 SV=1	0.00	5.76	2.80	3.79	0.00	3.64	4.22	3.96	0.00	0.00	0.00	2.49	0.00	5.04	0.00	0.00	0.00	0.00	0.00	0.00	0.00	5.19	0.00	1.42	2.22	0.97	>0.999999999999999	2.3
Q8N6N7	Acyl-CoA-binding domain-containing protein 7 OS=Homo sapiens GN=ACBD7 PE=1 SV=1	8.55	0.00	3.92	0.00	0.00	5.09	0.00	5.55	0.00	0.00	0.00	3.49	3.07	0.00	0.00	0.00	0.00	0.00	0.00	0.00	0.00	0.00	0.00	0.00	2.22	0.26	>0.999999999999999	8.7
P36543	V-type proton ATPase subunit E 1 OS=Homo sapiens GN=ATP6V1E1 PE=1 SV=1	0.00	6.22	0.00	0.00	2.49	3.93	2.19	0.00	1.07	2.82	5.11	2.69	2.37	5.44	0.00	1.86	2.45	4.14	0.00	3.50	0.40	5.61	1.99	1.53	2.21	2.44	>0.999999999999999	0.9
P52272	Heterogeneous nuclear ribonucleoprotein M OS=Homo sapiens GN=HNRNPM PE=1 SV=3	4.40	4.15	3.81	0.00	0.00	4.95	0.67	0.00	0.71	0.00	4.35	3.39	0.35	0.81	1.00	0.62	0.00	0.00	0.00	2.98	0.00	1.87	1.38	0.74	2.20	0.81	>0.999999999999999	2.7
P31153	S-adenosylmethionine synthase isoform type-2 OS=Homo sapiens GN=MAT2A PE=1 SV=1	1.95	3.69	1.79	3.64	1.53	4.95	1.35	2.54	0.32	0.00	3.03	1.59	0.70	1.61	0.89	0.55	1.51	1.23	2.54	2.07	0.25	3.32	1.23	0.91	2.20	1.40	>0.999999999999999	1.6
Q00839	Heterogeneous nuclear ribonucleoprotein U OS=Homo sapiens GN=HNRNPU PE=1 SV=6	3.66	1.61	2.69	2.88	1.34	2.77	0.67	2.38	0.75	1.15	5.30	1.10	0.35	1.41	0.00	0.48	0.75	2.91	1.11	1.43	0.00	0.00	1.07	0.40	2.19	0.83	>0.999999999999999	2.7
P14780	Matrix metalloproteinase-9 OS=Homo sapiens GN=MMP9 PE=1 SV=3	0.00	8.76	0.00	3.34	1.53	0.00	4.55	1.27	0.00	3.44	3.41	0.00	2.89	4.43	0.89	0.00	5.09	0.00	0.00	0.00	0.00	12.88	13.96	0.00	2.19	3.35	>0.999999999999999	0.7
P16083	Ribosyldihydronicotinamide dehydrogenase [quinone] OS=Homo sapiens GN=NQO2 PE=1 S	6.59	3.00	4.93	4.09	0.00	3.93	0.00	0.00	0.00	0.00	2.46	1.29	1.14	0.00	1.44	3.03	0.00	0.00	0.00	0.00	0.00	0.00	4.14	0.00	2.19	0.81	>0.999999999999999	2.7
P07311	Acylphosphatase-1 OS=Homo sapiens GN=ACYP1 PE=1 SV=2	0.00	7.15	3.47	4.70	0.00	0.00	0.00	4.91	0.00	0.00	5.87	0.00	0.00	0.00	0.00	0.00	0.00	0.00	0.00	0.00	0.00	0.00	0.00	0.00	2.18	0.00	>0.999999999999999	#DIV/0!
P09871	Complement C1s subcomponent OS=Homo sapiens GN=C1S PE=1 SV=1	0.98	2.07	2.02	6.82	6.33	0.58	2.19	0.00	0.16	2.40	2.46	0.00	2.46	4.63	4.33	1.93	0.75	1.99	5.23	1.68	0.28	8.10	1.38	0.74	2.17	2.79	>0.999999999999999	0.8
Q6UXB2	VEGF co-regulated chemokine 1 OS=Homo sapiens GN=CXCL17 PE=1 SV=1	0.00	0.00	2.69	3.64	4.60	0.00	4.05	3.80	0.00	2.50	4.54	0.00	2.11	0.00	2.67	0.00	4.52	3.68	3.80	0.00	0.74	0.00	3.68	0.00	2.15	1.77	>0.999999999999999	1.2
P05090	Apolipoprotein D OS=Homo sapiens GN=APOD PE=1 SV=1	0.00	0.00	0.00	2.43	3.07	2.33	5.74	2.54	0.00	1.67	6.44	1.59	1.40	3.22	1.78	1.10	6.41	0.00	5.39	2.07	0.49	3.32	5.21	0.91	2.15	2.61	>0.999999999999999	0.8
Q9BR76	Coronin-1B OS=Homo sapiens GN=CORO1B PE=1 SV=1	2.93	4.38	2.13	1.82	1.15	6.11	0.00	0.95	0.75	0.00	3.60	1.89	1.05	2.42	1.33	0.83	1.13	1.84	0.95	1.55	0.59	2.49	0.92	0.68	2.14	1.32	>0.999999999999999	1.6
P20700	Lamin-B1 OS=Homo sapiens GN=LMNB1 PE=1 SV=2	0.00	6.22	0.00	3.18	1.92	0.00	2.70	0.00	0.00	6.47	5.11	0.00	0.88	0.00	0.00	0.00	0.94	0.00	1.59	0.00	0.00	6.86	6.13	0.00	2.13	1.37	>0.999999999999999	1.6
P61081	NEDD8-conjugating enzyme Ubc12 OS=Homo sapiens GN=UBE2M PE=1 SV=1	3.91	3.69	0.00	5.16	3.07	4.95	0.00	2.54	0.63	0.00	0.00	1.59	0.00	3.22	0.00	0.00	0.00	0.00	0.00	4.41	0.00	7.07	0.00	0.91	2.13	1.30	>0.999999999999999	1.6
Q99733	Nucleosome assembly protein 1-like 4 OS=Homo sapiens GN=NAP1L4 PE=1 SV=1	0.00	3.69	2.80	1.21	3.07	3.64	1.35	2.54	0.63	0.00	3.03	3.49	0.00	3.22	0.89	1.72	0.00	0.00	0.00	1.04	0.49	1.66	1.23	0.00	2.12	0.85	>0.999999999999999	2.5
P08697	Alpha-2-antiplasmin OS=Homo sapiens GN=SERPINF2 PE=1 SV=3	4.64	0.00	2.13	1.82	3.64	1.75	2.02	0.95	1.35	0.63	2.27	4.18	0.00	1.21	2.11	0.83	4.90	0.00	4.12	0.78	0.37	0.00	1.84	1.93	2.12	1.51	>0.999999999999999	1.4
P42574	Caspase-3 OS=Homo sapiens GN=CASP3 PE=1 SV=2	5.37	2.30	1.12	1.52	1.92	4.95	1.69	1.59	0.87	1.04	1.89	1.00	0.88	2.01	1.11	0.69	1.89	1.53	0.00	1.30	0.31	2.08	1.53	0.57	2.10	1.16	>0.999999999999999	1.8
P01625	Ig kappa chain V-IV region Len OS=Homo sapiens PE=1 SV=2	6.59	0.00	0.00	0.00	0.00	3.93	0.00	0.00	1.07	10.85	0.00	2.69	0.00	5.44	0.00	1.86	0.00	0.00	0.00	3.50	0.00	0.00	4.14	0.00	2.09	1.25	>0.999999999999999	1.7
O00584	Ribonuclease T2 OS=Homo sapiens GN=RNASET2 PE=1 SV=2	2.69	2.54	4.15	1.67	2.11	3.35	3.88	1.74	0.91	0.00	2.08	0.00	0.96	2.22	2.56	0.76	4.34	0.00	0.00	1.43	0.00	4.78	1.69	0.62	2.09	1.61	>0.999999999999999	1.3
P50583	Bis(5'-nucleosyl)-tetraphosphatase [asymmetrical] OS=Homo sapiens GN=NUDT2 PE=1 SV	0.00	9.91	0.00	0.00	0.00	6.26	0.00	0.00	0.79	0.00	3.79	4.28	0.00	8.66	2.22	0.00	0.00	3.07	0.00	2.59	0.00	0.00	0.00	0.00	2.09	1.38	>0.999999999999999	1.5
O76003	Glutaredoxin-3 OS=Homo sapiens GN=GLRX3 PE=1 SV=2	4.40	2.07	2.02	1.36	1.73	2.62	1.52	2.85	0.00	0.00	3.41	2.89	0.79	3.63	0.00	1.24	0.00	1.38	0.00	3.76	0.55	3.74	0.00	1.02	2.07	1.34	>0.999999999999999	1.5
P40763	Signal transducer and activator of transcription 3 OS=Homo sapiens GN=STAT3 PE=1 SV=2	4.88	1.84	1.79	0.61	0.77	3.49	1.86	0.63	0.79	0.00	4.54	3.39	0.35	1.61	0.89	0.28	0.00	0.00	0.00	2.07	0.49	1.66	0.61	1.14	2.05	0.76	>0.999999999999999	2.7
P82970	High mobility group nucleosome-binding domain-containing protein 5 OS=Homo sapiens GN	8.55	5.07	0.00	0.00	2.11	0.00	1.86	1.74	0.00	0.00	4.17	1.10	0.00	0.00	0.00	0.00	0.00	0.00	0.00	1.43	0.00	0.00	0.00	0.00	2.05	0.12	>0.999999999999999	17.3
Q7KZF4	Staphylococcal nuclease domain-containing protein 1 OS=Homo sapiens GN=SND1 PE=1 S	3.18	3.00	2.35	0.45	0.00	6.70	0.51	0.95	0.83	0.00	4.73	1.69	0.53	3.42	1.11	0.69	0.57	1.99	0.95	3.24	0.09	6.62	0.00	0.97	2.03	1.18	>0.999999999999999	1.7
Q5JXB2	Putative ubiquitin-conjugating enzyme E2 N-like OS=Homo sapiens GN=UBE2NL PE=5 SV=	0.00	0.00	0.00	6.52	0.00	0.00	7.25	6.82	1.71	2.09	0.00	0.00	3.77	0.00	2.22	2.97	3.77	6.60	0.00	5.57	0.00	8.94	0.00	2.44	2.03	3.02	>0.999999999999999	0.7
P53999	Activated RNA polymerase II transcriptional coactivator p15 OS=Homo sapiens GN=SUB1 F	0.00	5.53	0.00	0.00	0.00	3.49	0.00	3.80	2.10	2.50	4.54	2.39	2.11	0.00	0.00	1.66	4.52	0.00	0.00	0.00	1.63	0.00	3.68	0.00	2.03	1.13	>0.999999999999999	1.8
Q5SXH7	PH domain-containing protein C10orf81 OS=Homo sapiens GN=C10orf81 PE=1 SV=2	4.88	1.38	0.67	0.91	5.37	4.08	1.01	0.00	0.24	0.00	3.79	1.99	0.53	0.00	0.00	0.00	0.00	0.00	0.00	0.00	0.00	0.00	0.00	0.34	2.03	0.07	>0.999999999999999	28.1
P01598	Ig kappa chain V-I region EU OS=Homo sapiens PE=1 SV=1	0.00	6.68	3.25	4.40	0.00	4.22	0.00	4.60	1.15	0.00	0.00	0.00	0.00	5.84	0.00	2.00	5.47	0.00	0.00	0.00	0.89	6.03	4.45	0.00	2.02	2.06	>0.999999999999999	1.0
Q7L1Q6	Basic leucine zipper and W2 domain-containing protein 1 OS=Homo sapiens GN=BZW1 PE	5.37	1.61	0.78	3.34	1.34	3.20	1.18	2.22	1.19	0.00	2.65	1.39	0.61	4.43	0.78	0.48	0.00	1.07	0.00	1.81	0.22	0.00	0.00	1.70	2.02	0.93	>0.999999999999999	2.2
P84243	Histone H3.3 OS=Homo sapiens GN=H3F3A PE=1 SV=2	5.37	18.90	0.00	0.00	0.00	0.00	0.00	0.00	0.00	0.00	0.00	0.00	0.00	0.00	0.00	0.00	0.00	0.00	0.00	0.00	0.00	0.00	0.00	0.00	2.02	0.00	>0.999999999999999	#DIV/0!
Q14019	Coactosin-like protein OS=Homo sapiens GN=COTL1 PE=1 SV=3	0.00	0.00	0.00	3.18	0.00	3.06	3.54	7.45	0.83	2.19	3.98	0.00	0.00	0.00	0.00	0.00	3.96	0.00	3.33	0.00	0.00	33.25	3.22	0.00	2.02	3.65	>0.999999999999999	0.6
P01700	Ig lambda chain V-I region HA OS=Homo sapiens PE=1 SV=1	7.08	6.68	0.00	0.00	5.56	0.00	4.89	0.00	0.00	0.00	0.00	0.00	0.00	0.00	0.00	0.00	0.00	0.00	0.00	0.00	0.00	0.00	0.00	0.00	2.02	0.00	>0.999999999999999	#DIV/0!
O60763	General vesicular transport factor p115 OS=Homo sapiens GN=USO1 PE=1 SV=2	7.57	3.00	0.34	1.36	2.49	3.93	0.51	1.43	0.24	0.00	2.46	0.90	0.26	1.21	1.00	0.21	0.57	0.46	0.48	2.46	0.09	1.25	0.92	0.74	2.02	0.80	>0.999999999999999	2.5
P25774	Cathepsin S OS=Homo sapiens GN=CTSS PE=1 SV=3	0.00	6.45	1.01	1.36	0.00	5.82	1.52	2.85	0.71	1.88	1.70	0.90	1.58	1.81	1.00	1.24	0.00	1.38	0.00	2.33	0.00	3.74	2.76	1.02	2.02	1.41	>0.999999999999999	1.4
Q92499	ATP-dependent RNA helicase DDX1 OS=Homo sapiens GN=DDX1 PE=1 SV=2	4.15	3.92	1.34	0.61	3.26	2.47	1.35	0.00	0.83	0.83	3.22	2.09	0.70	1.61	0.00	0.83	0.00	2.61	0.00	2.20	0.12	1.66	0.61	0.45	2.01	0.90	>0.999999999999999	2.2
Q9BZQ8	Protein Niban OS=Homo sapiens GN=FAM129A PE=1 SV=1	4.88	3.00	1.12	1.52	0.00	5.24	0.00	1.59	0.67	0.00	2.46	3.59	0.53	4.03	0.00	0.41	0.57	1.53	0.48	0.78	0.31	2.08	0.00	1.36	2.01	1.01	>0.999999999999999	2.0
Q13526	Peptidyl-prolyl cis-trans isomerase NIMA-interacting 1 OS=Homo sapiens GN=PIN1 PE=1 S\	4.40	4.15	4.48	0.00	0.00	5.82	0.00	0.00	0.00	0.00	3.41	1.79	0.00	0.00	0.00	0.00	0.00	0.00	0.00	0.00	0.00	3.74	0.00	0.00	2.00	0.31	>0.999999999999999	6.4
Q96HN2	Putative adenosylhomocysteinase 3 OS=Homo sapiens GN=AHCYL2 PE=1 SV=1	6.59	3.46	1.68	0.00	0.00	6.70	0.84	0.79	1.55	0.00	1.89	0.50	0.44	6.65	0.00	0.69	0.00	3.22	0.00	4.28	0.15	1.04	1.53	1.53	2.00	1.63	>0.999999999999999	1.2
Q9HCY8	Protein S100-A14 OS=Homo sapiens GN=S100A14 PE=1 SV=1	7.08	0.00	0.00	0.00	0.00	4.22	0.00	4.60	1.15	6.89	0.00	0.00	0.00	5.84	3.22	0.00	0.00	0.00	0.00	3.76	0.00	0.00	0.00	1.65	1.99	1.21	>0.999999999999999	1.7
Q14376	UDP-glucose 4-epimerase OS=Homo sapiens GN=GALE PE=1 SV=2	2.20	2.07	2.02	1.36	1.73	5.68	1.52	1.43	0.71	0.00	3.41	1.79	0.79	3.63	0.00	1.24	1.70	5.98	0.00	3.63	0.55	3.74	0.00	1.02	1.99	1.86	>0.999999999999999	1.1
P21281	V-type proton ATPase subunit B, brain isoform OS=Homo sapiens GN=ATP6V1B2 PE=1 SV	6.11	4.15	2.80	0.91	0.00	2.62	0.00	1.90	0.71	0.63	2.27	1.79	0.00	5.04	2.00	1.24	0.00	1.84	0.00	5.18	0.00	9.97	0.92	1.42	1.99	2.30	>0.999999999999999	0.9
P55058	Phospholipid transfer protein OS=Homo sapiens GN=PLTP PE=1 SV=1	2.93	4.38	3.81	1.82	0.00	1.75	3.21	1.90	0.48	1.25	1.14	1.20	1.05	2.42	1.33	0.83	1.13	2.91	0.95	1.55	0.37	2.49	1.84	0.00	1.99	1.41	>0.999999999999999	1.4
P04424	Argininosuccinate lyase OS=Homo sapiens GN=ASL PE=1 SV=4	4.88	8.30	2.24	0.91	0.00	4.08	1.01	0.00	0.00	0.00	1.14	1.29	1.14	1.21	0.00	0.41	0.00	0.00	0.00	0.00	0.00	2.70	0.00	0.00	1.99	0.46	>0.999999999999999	4.4
O00303	Eukaryotic translation initiation factor 3 subunit F OS=Homo sapiens GN=EIF3F PE=1 SV=1	7.08	2.07	3.25	1.36	1.73	2.62	1.52	0.00	0.71	0.00	1.70	1.79	0.79	1.81	1.00	0.00	0.00	0.00	0.00	1.17	0.55	0.00	0.00	0.51	1.99	0.49	>0.999999999999999	4.1
P49327	Fatty acid synthase OS=Homo sapiens GN=FASN PE=1 SV=3	3.91	5.07	2.02	0.91	0.77	5.68	1.01	0.32	0.87	0.00	1.89	1.39	0.44	5.04	1.22	1.66	0.00	2.76	0.79	3.11	0.22	1.04	0.31	1.02	1.99	1.47	>0.999999999999999	1.4
Q9H2H8	Peptidyl-prolyl cis-trans isomerase-like 3 OS=Homo sapiens GN=PPIL3 PE=1 SV=1	9.77	4.15	2.02	2.73	0.00	2.62	0.00	0.00	0.71	0.00	0.00	1.79	0.00	3.63	0.00	0.00	0.00	0.00	0.00	0.00	0.55	0.00	0.00	1.02	1.98	0.43	>0.999999999999999	4.6
Q86SR0	Secreted Ly-6/uPAR-related protein 2 OS=Homo sapiens GN=SLURP2 PE=2 SV=1	0.00	7.38	0.00	0.00	0.00	4.66	5.40	5.07	1.27	0.00	0.00	0.00	0.00	0.00	0.00	2.21	0.00	0.00	0.00	4.15	0.00	0.00	4.91	1.82	1.98	1.09	>0.999999999999999	1.8
Q8TBC4	NEDD8-activating enzyme E1 catalytic subunit OS=Homo sapiens GN=UBA3 PE=1 SV=2	4.88	3.00	2.24	0.91	2.49	5.24	1.01	0.00	0.24	0.00	2.46	1.29	0.53	1.21	0.67	0.41	0.00	0.00	0.00	0.78	0.00	0.00	0.00	0.34	1.98	0.33	>0.999999999999999	6.0
Q13200	26S proteasome non-ATPase regulatory subunit 2 OS=Homo sapiens GN=PSMD2 PE=1 S\	4.15	2.30	3.25	1.06	0.00	5.53	1.18	1.11	0.83	0.31	1.89	2.09	0.26	2.82	1.11	0.69	0.00	2.61	0.48	2.72	0.09	2.08	0.46	0.79	1.98	1.18	>0.999999999999999	1.7
O60884	DnaJ homolog subfamily A member 2 OS=Homo sapiens GN=DNAJA2 PE=1 SV=1	3.66	3.46	0.78	1.06	0.00	3.35	1.18	2.38	0.28	0.00	4.35	3.19	0.00	1.41	0.00	0.00	0.00	0.00	0.00	0.91	0.22	0.00	0.00	0.00	1.97	0.21	>0.999999999999999	9.4
Q9NYL9	Tropomodulin-3 OS=Homo sapiens GN=TMOD3 PE=1 SV=1	6.59	1.84	3.03	0.00	1.53	3.93	2.87	1.27	0.32	0.00	1.51	0.80	1.49	1.61	0.00	0.00	0.00	1.23	0.00	3.50	0.00	3.53	1.23	0.45	1.97	1.09	>0.999999999999999	1.8
Q09666	Neuroblast differentiation-associated protein AHNAK OS=Homo sapiens GN=AHNAK PE=1 S	4.15	3.23	2.13	1.36	0.96	3.49	1.01	2.22	0.40	0.42	3.22	1.10	0.61	4.23	1.33	1.03	0.57	1.07	1.59	3.50	0.15	0.62	0.46	0.62	1.97	1.32	>0.999999999999999	1.5
Q8TC71	Mitochondria-eating protein OS=Homo sapiens GN=SPATA18 PE=1 SV=1	2.69	2.54	0.00	0.76	2.11	7.42	1.86	0.79	0.00	0.00	3.22	2.29	0.00	0.00	0.00	0.00	0.00	0.00	0.00	0.00	0.00	0.00	0.00	0.00	1.97	0.00	>0.999999999999999	#DIV/0!
Q6UWW0	Lipocalin-15 OS=Homo sapiens GN=LCN15 PE=1 SV=1	8.55	0.00	1.79	0.00	0.00	2.33	2.70	0.00	0.00	0.00	6.63	1.59	0.00	0.00	0.00	0.00	0.00	2.45	0.00	0.00	0.00	3.32	0.00	0.91	1.97	0.56	>0.999999999999999	3.5
P01769	Ig heavy chain V-III region GA OS=Homo sapiens PE=1 SV=1	0.00	0.00	0.00	3.94	11.12	0.00	4.39	4.12	0.00	0.00	0.00	0.00	2.28	5.24	2.89	1.79	4.90	3.99	0.00	0.00	0.00	5.40	0.00	0.00	1.96	2.21	>0.999999999999999	0.9
P62714	Serine/threonine-protein phosphatase 2A catalytic subunit beta isoform OS=Homo sapiens (	2.20	2.07	3.36	2.88	0.00	4.37	0.00	0.00	0.00	0.00	5.68	2.99	0.00	1.81	0.00	2.07	0.00	2.91	1.43	3.89	0.28	0.00	0.00	1.70	1.96	1.17	>0.999999999999999	1.7
Q9UBE0	SUMO-activating enzyme subunit 1 OS=Homo sapiens GN=SAE1 PE=1 SV=1	4.40	6.45	2.02	1.36	0.00	4.08	1.52	0.00	1.11	0.00	1.70	0.90	0.00	1.81	0.00	0.00	0.00	0.00	0.00	2.33	0.00	0.00	0.00	1.02	1.96	0.43	>0.999999999999999	4.6
Q14116	Interleukin-18 OS=Homo sapiens GN=IL18 PE=1 SV=1	7.82	3.46	5.71	2.27	0.00	2.18	0.00	0.00	0.59	0.00	0.00	1.49	0.00	3.02	0.00	2.21	0.00	0.00	0.00	1.94	0.46	0.00	0.00	1.82	1.96	0.79	>0.999999999999999	2.5
Q8WWA0	Intelectin-1 OS=Homo sapiens GN=ITLN1 PE=1 SV=1	2.20	0.00	3.47	4.70	1.73	0.00	0.00	1.43	5.71	0.00	0.00	4.28	3.77	0.00	3.44	1.38	1.70	0.00	1.43	1.17	3.20	0.00	1.38	3.18	1.96	1.72	>0.999999999999999	1.1
P01031	Complement C5 OS=Homo sapiens GN=C5 PE=1 SV=4	0.49	0.69	1.23	5.76	7.29	1.31	1.86	0.00	0.08	1.98	1.33	1.49	1.84	5.84	5.89	2.35	6.79	0.77	4.91	1.68	0.52	4.36	2.91	0.74	1.96	3.22	>0.999999999999999	0.6
Q9HB71	Calcyclin-binding protein OS=Homo sapiens GN=CACYBP PE=1 SV=2	3.18	6.22	3.03	0.00	0.00	6.26	0.00	0.00	1.07	0.00	2.46	1.29	0.00	0.00	0.00	0.00	0.00	0.00	0.00	0.00	0.00	0.00	0.00	0.00	1.96	0.00	>0.999999999999999	#DIV/0!
Q9NUU7	ATP-dependent RNA helicase DDX19A OS=Homo sapiens GN=DDX19A PE=1 SV=1	4.64	6.22	2.13	0.00	1.15	1.75	2.02	1.90	0.24	0.00	0.00	3.39	0.00	2.42	0.67	0.00	0.00	2.91	0.00	1.55	0.00	2.49	0.00	0.68	1.95	0.89	>0.999999999999999	2.2
Q6GMV3	Putative peptidyl-tRNA hydrolase PTRHD1 OS=Homo sapiens GN=PTRHD1 PE=1 SV=1	0.00	4.84	0.00	0.00	4.03	3.06	0.00	7.45	0.00	0.00	3.98	0.00	0.00	0.00	0.00	3.24	0.00	7.21	0.00	0.00	0.00	4.36	0.00	0.00	1.95	1.23	>0.999999999999999	1.6
Q13740	CD166 antigen OS=Homo sapiens GN=ALCAM PE=1 SV=2	1.22	2.30	2.47	1.52	3.07	2.33	1.69	2.54	0.40	0.00	4.17	1.59	0.44	1.01	0.56	0.69	0.00	0.77	0.00	2.07	0.00	0.00	0.77	0.91	1.94	0.60	>0.999999999999999	3.2
Q9UBT2	SUMO-activating enzyme subunit 2 OS=Homo sapiens GN=UBA2 PE=1 SV=2	7.57	2.07	1.57	0.76	0.96	4.51	0.84	0.00	0.79	0.00	1.70	2.49	0.44	1.01	0.00	0.34	0.00	0.77	0.00	1.17	0.00	0.00	0.00	0.51	1.94	0.35	>0.999999999999999	5.5
O60506	Heterogeneous nuclear ribonucleoprotein Q OS=Homo sapiens GN=SYNCRIP PE=1 SV=2	2.44	4.61	1.68	1.52	1.92	2.91	0.84	1.59	0.79	1.04	2.84	1.00	1.32	3.02	0.00	1.03	0.94	1.53	0.79	1.94	0.00	2.08	0.77	0.57	1.93	1.17	>0.999999999999999	1.7
P17858	6-phosphofructokinase, liver type OS=Homo sapiens GN=PFKL PE=1 SV=6	3.91	4.84	2.35	0.61	0.77	3.64	1.35	0.00	0.48	0.00	3.03	2.09	0.70	3.22	0.89	0.28	0.00	0.00	0.00	2.07	0.00	2.49	0.00	0.91	1.92	0.88	>0.999999999999999	2.2
Q9UNH7	Sorting nexin-6 OS=Homo sapiens GN=SNX6 PE=1 SV=1	5.62	0.00	2.58	0.00	0.00	4.51	1.18	1.11	0.59	0.00	4.35	3.09	0.61	1.41	0.78	0.48	0.00	1.07	2.38	1.94	0.00	3.12	0.00	0.40	1.92	1.02	>0.999999999999999	1.9
O95747	Serine/threonine-protein kinase OSR1 OS=Homo sapiens GN=OXSR1 PE=1 SV=1	2.93	1.38	2.02	2.73	2.30	2.62	2.02	1.90	0.48	0.00	3.41	1.20	1.05	2.42	0.67	1.24	0.00	0.00	0.00	2.33	0.37	1.25	1.84	0.34	1.92	0.96	>0.999999999999999	2.0
P39023	60S ribosomal protein L3 OS=Homo sapiens GN=RPL3 PE=1 SV=2	3.66	3.46	1.68	1.06	1.34	3.35	1.18	1.11	0.28	0.73	4.35	0.70	0.61	3.02	0.78	0.48	0.00	1.07	2.38	1.94	0.00	1.45	1.07	0.85	1.91	1.14	>0.999999999999999	1.7
P19012	Keratin, type I cytoskeletal 15 OS=Homo sapiens GN=KRT15 PE=1 SV=3	0.00	10.83	0.00	0.00	0.00	0.00	4.89	0.00	0.00	7.10	0.00	0.00	0.00	9.47	0.00	0.00	0.00	0.00	9.04	0.00	0.00	0.00	0.00	0.00	1.90	1.54	>0.999999999999999	1.2
A6NKN8	Purkinje cell protein 4-like protein 1 OS=Homo sapiens GN=PCP4L1 PE=2 SV=3	0.00	0.00	0.00	0.00	0.00	6.99	0.00	0.00	1.90	0.00	9.09	4.78	0.00	0.00	0.00	0.00	0.00	0.00	0.00	0.00	0.00	0.00	0.00	0.00	1.90	0.00	>0.999999999999999	#DIV/0!
P22234	Multifunctional protein ADE2 OS=Homo sapiens GN=PAICS PE=1 SV=3	5.37	3.23	0.78	0.00	0.00	5.82	0.00	2.22	0.56	0.73	2.65	1.39	0.00	6.24	0.00	0.00	0.00	0.00	0.00	5.18	0.43	6.44	2.15	0.00	1.90	1.70	>0.999999999999999	1.1
P42766	60S ribosomal protein L35 OS=Homo sapiens GN=RPL35 PE=1 SV=2	5.62	0.00	0.00	3.49	0.00	3.35	0.00	3.65	0.00	0.00	4.35	2.29	2.02	10.48	0.00	0.00	0.00	3.53	0.00	2.98	0.71	0.00	3.53	0.00	1.90	1.94	>0.999999999999999	1.0

P60891	Ribose-phosphate pyrophosphokinase 1 OS=Homo sapiens GN=PRPS1 PE=1 SV=2	0.00	7.15	3.47	0.00	1.73	10.33	0.00	0.00	0.00	0.00	0.00	0.00	0.00	1.81	2.22	0.00	0.00	0.00	0.00	0.00	0.00	0.00	0.00	0.00	1.89	0.34	>0.999999999999999	5.6
Q9UI30	tRNA methyltransferase 112 homolog OS=Homo sapiens GN=TRMT112 PE=1 SV=1	12.94	0.00	0.00	0.00	0.00	3.49	0.00	3.80	0.00	0.00	0.00	2.39	0.00	0.00	0.00	0.00	0.00	3.68	0.00	3.11	0.00	0.00	0.00	1.36	1.89	0.68	>0.999999999999999	2.8
P02655	Apolipoprotein C-II OS=Homo sapiens GN=APOC2 PE=1 SV=1	0.00	0.00	0.00	4.55	0.00	0.00	5.06	0.00	0.00	7.31	5.68	0.00	2.63	0.00	3.33	0.00	0.00	0.00	29.96	0.00	2.16	6.23	4.60	0.00	1.88	4.08	>0.999999999999999	0.5
P32455	Interferon-induced guanylate-binding protein 1 OS=Homo sapiens GN=GBP1 PE=1 SV=2	3.66	2.30	0.56	0.00	0.00	5.68	0.84	0.00	1.31	0.00	4.92	3.29	0.00	3.02	0.00	0.69	0.00	0.77	0.00	1.30	1.02	6.86	2.30	1.19	1.88	1.43	>0.999999999999999	1.3
P31431	Syndecan-4 OS=Homo sapiens GN=SDC4 PE=1 SV=2	3.91	3.69	1.79	2.43	3.07	2.33	0.00	0.00	0.63	0.00	3.03	1.59	1.40	6.65	0.00	2.28	0.00	0.00	0.00	4.28	1.02	0.00	0.00	0.00	1.87	1.30	>0.999999999999999	1.4
P82979	SAP domain-containing ribonucleoprotein OS=Homo sapiens GN=SARNP PE=1 SV=3	3.42	0.00	1.57	0.00	0.00	6.99	0.00	2.22	1.19	0.00	5.68	1.39	0.00	2.82	1.56	0.97	0.00	0.00	0.00	3.89	0.00	2.91	0.00	0.00	1.87	1.01	>0.999999999999999	1.9
O95968	Secretoglobin family 1D member 1 OS=Homo sapiens GN=SCGB1D1 PE=1 SV=1	8.30	0.00	3.81	0.00	0.00	4.95	0.00	5.39	0.00	0.00	0.00	0.00	0.00	0.00	0.00	0.00	0.00	5.21	0.00	4.41	0.00	0.00	0.00	0.00	1.87	0.80	>0.999999999999999	2.3
P23246	Splicing factor, proline- and glutamine-rich OS=Homo sapiens GN=SFPQ PE=1 SV=2	3.18	3.00	2.02	1.97	0.77	1.31	1.52	2.85	0.16	0.94	3.41	1.29	0.35	2.62	1.00	0.62	1.70	1.99	1.43	1.68	0.12	0.83	1.99	0.74	1.87	1.26	>0.999999999999999	1.5
P20339	Ras-related protein Rab-5A OS=Homo sapiens GN=RAB5A PE=1 SV=2	0.00	3.23	1.57	0.00	0.00	6.99	0.00	0.00	1.90	0.00	5.68	2.99	0.00	0.00	0.00	0.00	0.00	0.00	0.00	0.00	0.00	0.00	2.15	0.79	1.86	0.25	>0.999999999999999	7.6
P35244	Replication protein A 14 kDa subunit OS=Homo sapiens GN=RPA3 PE=1 SV=1	0.00	5.76	2.80	3.79	0.00	0.00	4.22	0.00	0.99	0.00	4.73	0.00	0.00	0.00	2.78	1.72	0.00	0.00	0.00	3.24	0.00	0.00	0.00	1.42	1.86	0.76	>0.999999999999999	2.4
P00918	Carbonic anhydrase 2 OS=Homo sapiens GN=CA2 PE=1 SV=2	0.00	2.54	0.00	1.67	2.11	5.53	1.86	1.74	0.95	0.00	2.08	3.79	0.00	2.22	1.22	1.66	0.00	5.83	8.56	4.92	0.74	2.29	3.68	1.36	1.86	2.71	>0.999999999999999	0.7
Q4LDG9	Dynein light chain 1, axonemal OS=Homo sapiens GN=DNAL1 PE=1 SV=1	3.91	3.69	1.79	2.43	3.07	0.00	2.70	0.00	0.00	0.00	3.03	1.59	0.00	0.00	0.00	0.00	0.00	0.00	0.00	0.00	0.00	0.00	0.00	0.00	1.85	0.00	>0.999999999999999	#DIV/0!
Q86X76	Nitrilase homolog 1 OS=Homo sapiens GN=NIT1 PE=1 SV=2	4.64	2.07	1.01	1.36	1.73	4.37	0.00	0.00	1.19	0.00	1.70	4.08	0.79	6.04	1.00	2.83	0.00	0.00	0.00	1.17	0.28	3.95	1.38	1.70	1.85	1.59	>0.999999999999999	1.2
P55884	Eukaryotic translation initiation factor 3 subunit B OS=Homo sapiens GN=EIF3B PE=1 SV=3	4.64	3.46	1.23	1.06	1.34	3.35	1.18	1.11	0.75	0.00	2.08	1.89	0.61	3.02	0.44	0.76	0.75	0.61	0.63	1.94	0.34	0.83	1.07	0.40	1.84	0.95	>0.999999999999999	1.9
Q9H008	Phospholysine phosphohistidine inorganic pyrophosphate phosphatase OS=Homo sapiens	2.69	5.53	1.23	1.67	2.11	1.60	1.86	1.74	0.44	0.00	2.08	1.10	0.96	2.22	1.22	0.76	2.07	1.69	1.74	1.43	0.34	2.29	1.69	0.62	1.84	1.42	>0.999999999999999	1.3
P22102	Trifunctional purine biosynthetic protein adenosine-3 OS=Homo sapiens GN=GART PE=1 S\	6.59	4.38	1.79	0.45	0.58	3.35	0.51	0.95	0.36	0.00	1.14	1.89	0.26	0.60	0.33	0.21	0.00	0.00	0.00	1.17	0.00	0.62	0.00	0.34	1.83	0.29	>0.999999999999999	6.2
P04430	Ig kappa chain V-I region BAN OS=Homo sapiens PE=1 SV=1	0.00	0.00	7.40	4.40	5.56	0.00	0.00	4.60	0.00	0.00	0.00	0.00	0.00	0.00	3.22	2.00	12.44	0.00	4.60	0.00	0.00	0.00	0.00	0.00	1.83	1.86	>0.999999999999999	1.0
O75569	Interferon-inducible double stranded RNA-dependent protein kinase activator A OS=Homo s	4.88	2.30	1.12	1.52	1.92	2.91	0.00	0.00	0.40	0.00	3.79	3.09	0.88	2.01	0.00	0.69	0.00	1.53	0.00	2.59	0.00	0.00	0.00	1.14	1.83	0.74	>0.999999999999999	2.5
Q8N1G4	Leucine-rich repeat-containing protein 47 OS=Homo sapiens GN=LRRC47 PE=1 SV=1	3.91	1.15	1.23	3.34	2.11	3.20	1.86	0.79	0.20	0.00	3.03	1.10	0.00	1.01	0.00	1.10	0.00	0.77	0.00	3.63	0.00	0.00	0.00	0.91	1.83	0.62	>0.999999999999999	3.0
P23588	Eukaryotic translation initiation factor 4B OS=Homo sapiens GN=EIF4B PE=1 SV=2	2.44	2.30	2.24	1.52	1.92	1.46	3.37	1.59	0.20	0.00	2.84	1.99	0.88	3.02	0.56	0.34	0.00	2.30	0.00	1.30	0.15	2.08	1.53	0.57	1.82	1.06	>0.999999999999999	1.7
P11940	Polyadenylate-binding protein 1 OS=Homo sapiens GN=PABPC1 PE=1 SV=2	3.66	3.46	2.80	1.36	0.00	2.91	0.00	0.00	1.23	0.94	2.84	2.49	1.32	1.81	1.67	0.00	0.00	3.83	0.00	1.94	0.00	0.00	0.00	0.85	1.81	0.95	>0.999999999999999	1.9
Q9H0P0	Cytosolic 5'-nucleotidase 3 OS=Homo sapiens GN=NT5C3 PE=1 SV=3	4.40	2.07	0.00	0.00	1.73	4.08	0.00	2.85	0.36	0.00	3.41	2.79	0.00	0.00	0.00	0.00	0.00	0.00	0.00	2.33	0.00	1.87	1.38	0.00	1.81	0.47	>0.999999999999999	3.9
P48147	Prolyl endopeptidase OS=Homo sapiens GN=PREP PE=1 SV=2	4.15	3.00	1.46	0.61	0.00	6.99	0.00	0.63	0.52	0.00	1.51	2.69	0.00	4.43	0.00	1.86	0.00	0.61	0.00	1.04	0.00	1.66	0.00	1.25	1.80	0.90	>0.999999999999999	2.0
P62249	40S ribosomal protein S16 OS=Homo sapiens GN=RPS16 PE=1 SV=2	0.00	4.61	2.24	0.00	0.00	10.92	0.00	0.00	1.78	0.00	0.00	1.99	0.00	4.03	0.00	0.00	0.00	0.00	0.00	2.59	0.00	0.00	0.00	0.00	1.80	0.55	>0.999999999999999	3.3
P50914	60S ribosomal protein L14 OS=Homo sapiens GN=RPL14 PE=1 SV=4	3.42	0.00	3.36	0.00	0.00	7.13	0.00	0.00	0.56	0.00	5.68	1.39	0.00	2.82	3.33	2.07	0.00	2.15	2.22	3.89	0.00	0.00	0.00	0.00	1.80	1.37	>0.999999999999999	1.3
P15170	Eukaryotic peptide chain release factor GTP-binding subunit ERF3A OS=Homo sapiens GN=	2.93	5.76	0.00	1.82	1.15	4.80	0.00	0.95	0.99	0.00	1.14	1.89	0.53	6.65	0.00	0.83	0.00	0.00	0.00	2.46	0.00	2.49	0.00	0.68	1.79	1.14	>0.999999999999999	1.6
P10643	Complement component C7 OS=Homo sapiens GN=C7 PE=1 SV=2	2.69	1.61	2.47	2.73	4.22	0.44	1.86	1.11	0.12	1.46	2.65	0.00	1.58	2.82	2.00	1.52	4.15	1.07	1.11	0.39	0.09	2.91	1.69	0.62	1.78	1.66	>0.999999999999999	1.1
P02743	Serum amyloid P-component OS=Homo sapiens GN=APCS PE=1 SV=2	3.18	0.00	1.46	4.25	2.49	1.89	0.00	0.00	0.00	1.36	5.30	1.29	2.46	5.64	5.00	1.93	2.45	1.99	4.44	1.68	0.86	2.70	4.29	0.74	1.77	2.85	>0.999999999999999	0.6
Q13907	Isopentenyl-diphosphate Delta-isomerase 1 OS=Homo sapiens GN=IDI1 PE=1 SV=2	6.59	6.22	0.00	1.97	2.49	3.93	0.00	0.00	0.00	0.00	0.00	0.00	0.00	5.44	0.00	0.90	0.00	1.99	0.00	0.00	0.00	0.00	0.00	1.53	1.77	0.82	>0.999999999999999	2.2
P30046	D-dopachrome decarboxylase OS=Homo sapiens GN=DDT PE=1 SV=3	0.00	6.22	0.00	0.00	0.00	8.88	0.00	0.00	0.00	0.00	0.00	6.08	0.00	0.00	0.00	0.00	0.00	0.00	0.00	0.00	0.83	0.00	0.00	0.00	1.76	0.07	>0.999999999999999	25.5
Q16772	Glutathione S-transferase A3 OS=Homo sapiens GN=GSTA3 PE=1 SV=3	0.00	0.00	0.00	0.00	21.10	0.00	0.00	0.00	0.00	0.00	0.00	0.00	0.00	0.00	0.00	0.00	0.00	0.00	0.00	0.00	0.00	0.00	0.00	0.00	1.76	0.00	>0.999999999999999	#DIV/0!
Q9UJU6	Drebrin-like protein OS=Homo sapiens GN=DBNL PE=1 SV=1	1.71	1.61	1.57	1.06	2.68	3.20	2.36	1.11	0.87	0.00	2.65	2.19	1.23	6.04	1.56	2.07	1.32	1.07	1.11	2.85	0.68	4.57	2.15	1.70	1.75	2.20	>0.999999999999999	0.8
P28070	Proteasome subunit beta type-4 OS=Homo sapiens GN=PSMB4 PE=1 SV=4	2.69	2.54	2.69	1.67	2.11	1.60	1.86	1.74	0.95	0.00	2.08	1.10	0.00	2.22	1.22	1.66	0.00	3.68	1.74	3.11	0.74	4.99	1.69	0.00	1.75	1.75	>0.999999999999999	1.0
P29350	Tyrosine-protein phosphatase non-receptor type 6 OS=Homo sapiens GN=PTPN6 PE=1 SV	3.66	1.15	1.12	2.27	0.96	3.06	0.84	0.00	0.20	2.19	3.98	1.49	1.32	3.02	0.00	0.34	0.94	0.77	0.00	1.30	0.00	5.61	4.14	0.28	1.74	1.48	>0.999999999999999	1.2
Q8NCW5	NAD(P)H-hydrate epimerase OS=Homo sapiens GN=APOA1BP PE=1 SV=2	8.55	2.30	1.12	3.34	1.92	1.46	0.00	0.00	0.00	0.00	0.00	2.19	0.00	2.01	0.00	0.00	0.00	0.00	0.00	0.00	0.31	0.00	0.00	0.57	1.74	0.24	>0.999999999999999	7.2
O43681	ATPase ASNA1 OS=Homo sapiens GN=ASNA1 PE=1 SV=2	6.84	1.84	2.02	1.21	0.00	2.62	0.00	1.27	0.71	0.00	1.51	2.79	0.70	3.63	0.89	0.55	0.00	0.00	0.00	1.04	0.25	1.66	0.00	0.45	1.73	0.76	>0.999999999999999	2.3
P13667	Protein disulfide-isomerase A4 OS=Homo sapiens GN=PDIA4 PE=1 SV=2	2.20	2.07	1.57	0.61	0.77	6.99	0.67	3.01	0.36	0.42	0.76	1.39	0.35	1.81	0.00	0.28	0.75	1.38	0.63	1.81	0.12	1.87	0.00	0.51	1.73	0.79	>0.999999999999999	2.2
O95865	N(G),N(G)-dimethylarginine dimethylaminohydrolase 2 OS=Homo sapiens GN=DDAH2 PE=1	9.04	2.54	0.00	0.00	0.00	3.49	0.00	0.00	0.44	0.00	0.00	5.28	0.96	2.22	0.00	0.76	0.00	0.00	0.00	1.43	0.00	0.00	1.69	0.00	1.73	0.59	>0.999999999999999	2.9
Q7Z4S6	Kinesin-like protein KIF21A OS=Homo sapiens GN=KIF21A PE=1 SV=2	5.13	3.46	1.01	0.30	2.49	1.31	0.84	1.74	0.36	0.00	2.84	1.29	0.26	0.40	0.00	0.21	0.38	0.46	0.00	1.43	0.06	0.00	0.31	0.17	1.73	0.31	>0.999999999999999	5.7
P0C0L4	Complement C4-A OS=Homo sapiens GN=C4A PE=1 SV=1	0.00	0.00	0.00	0.00	0.00	0.00	0.00	2.85	0.00	7.10	10.79	0.00	0.00	0.00	0.00	0.00	0.00	6.14	18.23	4.79	0.00	0.00	7.52	0.00	1.73	3.06	>0.999999999999999	0.6
O15371	Eukaryotic translation initiation factor 3 subunit D OS=Homo sapiens GN=EIF3D PE=1 SV=1	5.37	2.30	3.14	0.76	0.96	4.08	0.00	0.00	0.63	0.00	1.89	1.59	0.44	4.43	1.11	1.10	0.00	0.77	0.00	2.85	0.49	0.00	0.77	0.57	1.73	1.04	>0.999999999999999	1.7
P62244	40S ribosomal protein S15a OS=Homo sapiens GN=RPS15A PE=1 SV=2	5.62	0.00	0.00	0.00	0.00	7.42	0.00	0.00	0.91	0.00	4.35	2.29	0.00	4.63	0.00	0.00	0.00	3.53	0.00	2.98	0.00	0.00	0.00	1.31	1.72	1.04	>0.999999999999999	1.7
P41218	Myeloid cell nuclear differentiation antigen OS=Homo sapiens GN=MNDA PE=1 SV=1	0.00	3.46	0.78	2.27	1.34	2.18	2.53	2.38	0.00	4.28	1.33	0.00	1.32	3.02	0.00	0.00	2.83	0.00	0.00	0.00	0.00	10.60	2.30	0.00	1.71	1.67	>0.999999999999999	1.0
Q96PD5	N-acetylmuramoyl-L-alanine amidase OS=Homo sapiens GN=PGLYRP2 PE=1 SV=1	4.15	2.54	0.56	0.76	4.41	0.73	0.84	0.79	0.20	1.77	2.08	1.69	0.44	3.42	1.89	0.34	4.34	0.77	1.74	1.43	0.34	3.53	2.61	0.28	1.71	1.76	>0.999999999999999	1.0
P40429	60S ribosomal protein L13a OS=Homo sapiens GN=RPL13A PE=1 SV=2	0.00	6.91	3.36	4.55	0.00	4.37	0.00	0.00	1.19	0.00	0.00	0.00	0.00	9.87	0.00	2.07	0.00	4.60	0.00	3.89	0.00	0.00	0.00	1.70	1.70	1.84	>0.999999999999999	0.9
P63151	Serine/threonine-protein phosphatase 2A 55 kDa regulatory subunit B alpha isoform OS=Ho	4.88	3.00	0.00	1.97	2.49	0.00	2.19	2.06	0.00	0.00	2.46	1.29	0.00	5.64	1.44	0.90	0.00	1.99	2.06	5.70	0.00	2.70	0.00	0.00	1.70	1.70	>0.999999999999999	1.0
P13798	Acylamino-acid-releasing enzyme OS=Homo sapiens GN=APEH PE=1 SV=4	4.15	5.07	0.90	0.61	0.00	4.66	0.00	0.63	0.32	0.00	2.27	1.69	0.70	3.42	1.33	0.83	0.00	3.99	0.00	3.37	0.00	1.66	1.23	0.68	1.69	1.43	>0.999999999999999	1.2
P47897	Glutamine--tRNA ligase OS=Homo sapiens GN=QARS PE=1 SV=1	1.95	2.54	1.79	1.21	0.77	4.95	0.00	1.74	0.79	0.42	3.03	1.10	0.00	2.22	0.89	0.76	0.00	1.69	0.00	3.11	0.49	1.66	0.00	1.14	1.69	1.00	>0.999999999999999	1.7
P14324	Farnesyl pyrophosphate synthase OS=Homo sapiens GN=FDPS PE=1 SV=4	3.42	3.23	1.57	1.06	1.34	2.04	1.18	1.11	0.56	0.73	2.65	1.39	0.00	1.41	0.78	0.48	0.00	3.37	0.00	0.91	0.22	0.00	1.07	0.40	1.69	0.72	>0.999999999999999	2.3
P68402	Platelet-activating factor acetylhydrolase IB subunit beta OS=Homo sapiens GN=PAFAH1B2	3.18	0.00	1.46	1.97	2.49	4.08	2.19	0.00	1.11	0.00	2.46	1.29	1.14	0.00	1.44	1.93	2.45	0.00	2.06	1.68	0.40	2.70	0.00	0.00	1.69	1.15	>0.999999999999999	1.5
O95394	Phosphoacetylglucosamine mutase OS=Homo sapiens GN=PGM3 PE=1 SV=1	2.69	2.54	1.90	0.76	2.11	3.49	0.84	0.00	0.44	0.00	0.95	4.48	0.44	4.83	1.89	0.76	0.00	0.77	0.00	2.20	0.52	2.29	1.69	0.97	1.68	1.36	>0.999999999999999	1.2
P42224	Signal transducer and activator of transcription 1-alpha/beta OS=Homo sapiens GN=STAT1	2.93	1.84	1.34	0.61	0.00	3.64	0.67	0.63	2.18	0.00	4.73	1.59	0.00	2.42	0.00	0.83	0.00	2.45	0.63	2.59	0.77	0.83	3.07	0.45	1.68	1.17	>0.999999999999999	1.4
P46926	Glucosamine-6-phosphate isomerase 1 OS=Homo sapiens GN=GNPDA1 PE=1 SV=1	8.06	0.00	1.12	1.52	0.00	0.00	0.00	0.00	1.31	0.00	1.89	6.18	0.00	9.47	2.33	1.45	1.89	0.00	0.00	4.28	0.00	6.86	1.53	1.87	1.67	2.47	>0.999999999999999	0.7
P61313	60S ribosomal protein L15 OS=Homo sapiens GN=RPL15 PE=1 SV=2	0.00	3.23	1.57	0.00	0.00	4.37	0.00	0.00	0.56	0.00	8.90	1.39	0.00	0.00	0.00	0.97	0.00	2.15	0.00	0.00	0.43	0.00	0.00	0.79	1.67	0.36	>0.999999999999999	4.6
O60888	Protein CutA OS=Homo sapiens GN=CUTA PE=1 SV=2	4.40	4.15	2.02	0.00	0.00	2.62	3.04	0.00	0.00	0.00	0.00	3.79	1.58	3.63	2.00	1.24	3.39	2.76	0.00	2.33	0.55	0.00	2.76	1.02	1.67	1.77	>0.999999999999999	0.9
O43865	Putative adenosylhomocysteinase 2 OS=Homo sapiens GN=AHCYL1 PE=1 SV=2	9.28	3.92	0.00	0.00	0.00	6.55	0.00	0.00	0.00	0.00	0.00	0.00	0.00	0.00	0.00	0.00	0.00	0.00	0.00	0.00	0.00	0.00	0.00	0.00	1.65	0.00	>0.999999999999999	#DIV/0!
P09496	Clathrin light chain A OS=Homo sapiens GN=CLTA PE=1 SV=1	0.00	2.77	1.34	1.82	0.00	5.97	0.00	1.90	1.03	0.00	2.27	2.59	1.05	2.42	0.00	0.83	0.00	1.84	0.00	1.55	0.37	2.49	0.00	0.68	1.64	0.94	>0.999999999999999	1.8
Q07812	Apoptosis regulator BAX OS=Homo sapiens GN=BAX PE=1 SV=1	3.91	3.69	1.79	0.00	0.00	2.33	2.70	0.00	0.63	0.00	3.03	1.59	0.00	3.22	0.00	0.00	0.00	0.00	0.00	0.00	0.49	3.32	0.00	0.00	1.64	0.59	>0.999999999999999	2.8
Q16222	UDP-N-acetylhexosamine pyrophosphorylase OS=Homo sapiens GN=UAP1 PE=1 SV=3	1.47	1.38	0.67	0.00	0.00	7.86	0.00	1.74	0.71	0.00	3.41	2.39	0.53	4.83	0.67	0.76	0.00	0.00	0.00	1.43	0.55	1.25	0.92	0.62	1.64	0.96	>0.999999999999999	1.7
P18827	Syndecan-1 OS=Homo sapiens GN=SDC1 PE=1 SV=3	5.13	2.30	1.12	1.52	1.92	1.46	1.69	1.59	0.00	0.00	1.89	1.00	0.88	2.01	1.11	0.69	1.89	0.00	1.59	1.30	0.31	2.08	0.00	0.00	1.63	0.99	>0.999999999999999	1.7
P51148	Ras-related protein Rab-5C OS=Homo sapiens GN=RAB5C PE=1 SV=2	0.00	0.00	0.00	0.00	0.00	0.00	0.00	2.22	0.00	3.13	9.28	4.88	0.00	9.87	0.00	2.07	0.00	4.60	0.00	3.89	0.00	0.00	0.00	0.00	1.63	1.70	>0.999999999999999	1.0
O76070	Gamma-synuclein OS=Homo sapiens GN=SNCG PE=1 SV=2	0.00	0.00	2.91	0.00	0.00	14.26	0.00	0.00	2.30	0.00	0.00	0.00	0.00	0.00	0.00	6.76	0.00	8.90	0.00	7.51	0.00	0.00	0.00	1.48	1.62	2.05	>0.999999999999999	0.8
Q16401	26S proteasome non-ATPase regulatory subunit 5 OS=Homo sapiens GN=PSMD5 PE=1 S\	2.93	2.77	3.70	0.91	1.15	1.75	2.02	0.00	0.71	0.00	2.27	1.20	1.05	2.42	2.78	0.83	0.00	0.92	0.00	3.24	0.55	0.00	0.92	1.42	1.62	1.18	>0.999999999999999	1.4
Q8WW22	DnaJ homolog subfamily A member 4 OS=Homo sapiens GN=DNAJA4 PE=1 SV=1	3.66	3.46	1.68	0.00	2.88	2.18	1.18	0.00	0.00	0.00	2.84	1.49	0.61	1.41	0.00	0.00	1.32	0.00	0.00	0.00	0.22	0.00	2.30	0.00	1.61	0.49	>0.999999999999999	3.3
Q8WVY7	Ubiquitin-like domain-containing CTD phosphatase 1 OS=Homo sapiens GN=UBLCP1 PE=1	4.64	4.38	1.01	1.36	1.73	2.77	0.00	1.43	0.36	0.00	1.70	0.00	0.00	0.00	0.00	0.62	1.70	0.00	0.00	1.17	0.00	0.00	0.00	0.51	1.61	0.33	>0.999999999999999	4.8
Q9NP72	Ras-related protein Rab-18 OS=Homo sapiens GN=RAB18 PE=1 SV=1	7.57	3.23	0.00	0.00	0.00	4.51	0.00	0.00	0.00	0.00	2.65	1.39	0.00	0.00	0.00	0.00	0.00	0.00	0.00	0.00	0.00	0.00	0.00	0.00	1.61	0.00	>0.999999999999999	#DIV/0!
P04211	Ig lambda chain V region 4A OS=Homo sapiens PE=4 SV=1	0.00	6.22	0.00	0.00	5.18	0.00	0.00	0.00	0.00	2.82	5.11	0.00	2.37	5.44	3.00	0.00	5.09	4.14	0.00	0.00	0.00	5.61	0.00	0.00	1.61	2.14	>0.999999999999999	0.8
O60234	Glia maturation factor gamma OS=Homo sapiens GN=GMFG PE=1 SV=1	0.00	0.00	0.00	3.03	0.00	0.00	3.37	0.00	0.00	4.59	8.33	0.00	1.75	8.86	2.22	0.00	3.77	0.00	0.00	0.00	0.00	9.14	6.75	0.00	1.61	2.71	>0.999999999999999	0.6
Q01082	Spectrin beta chain, brain 1 OS=Homo sapiens GN=SPTBN1 PE=1 SV=2	4.64	2.30	0.67	0.76	0.77	4.08	0.34	0.32	0.28	0.52	3.22	1.39	0.35	2.01	0.22	0.07	0.19	1.38	0.00	0.91	0.03	0.83	0.61	0.34	1.61	0.58	>0.999999999999999	2.8
P63010	AP-2 complex subunit beta OS=Homo sapiens GN=AP2B1 PE=1 SV=1	4.15	4.61	1.90	0.00	0.00	3.49	0.00	0.00	0.95	0.00	2.46	1.69	0.00	2.62	0.00	0.00	0.00	0.00	0.00	0.00	0.00	0.00	0.00	0.57	1.61	0.27	>0.999999999999999	6.0
Q5VW32	BRO1 domain-containing protein BROX OS=Homo sapiens GN=BROX PE=1 SV=1	5.62	1.61	1.68	1.06	1.34	1.02	0.00	0.00	0.28	0.00	4.35	2.29	0.61	3.02	0.00	0.00	0.00	1.07	0.00	0.00	0.46	0.00	0.00	0.40	1.60	0.46	>0.999999999999999	3.5
P12955	Xaa-Pro dipeptidase OS=Homo sapiens GN=PEPD PE=1 SV=3	4.64	2.77	0.00	0.91	1.15	2.77	1.01	0.95	0.24	0.00	3.60	1.20	0.00	2.42	1.33	0.83	1.13	0.92	0.95	2.46	0.00	1.25	2.91	0.34	1.60	1.21	>0.999999999999999	1.3
O15372	Eukaryotic translation initiation factor 3 subunit H OS=Homo sapiens GN=EIF3H PE=1 SV=1	1.95	1.84	0.90	1.21	1.53	3.93	1.35	1.27	0.32	0.00	3.22	1.69	0.70	1.61	0.89	0.55	1.51	0.00	1.27	2.20	0.25	1.66	1.23	0.45	1.60	1.03	>0.999999999999999	1.6
Q16666	Gamma-interferon-inducible protein 16 OS=Homo sapiens GN=IFI16 PE=1 SV=3	4.88	2.54	0.90	0.00	0.77	4.22	0.00	2.54	0.32	0.42	1.51	1.10	0.35	2.22	0.44	0.00	0.00	1.69	0.63	1.43	0.00	0.00	0.61	0.23	1.60	0.63	>0.999999999999999	2.5
Q13045	Protein flightless-1 homolog OS=Homo sapiens GN=FLII PE=1 SV=2	4.64	2.77	1.01	1.06	1.34	2.04	1.18	1.43	0.56	0.52	1.70	0.90	1.05	1.81	0.22	0.34	0.94	0.31	0.32	0.65	0.15	2.49	0.31	0.68	1.60	0.77	>0.999999999999999	2.1
Q8NHW5	60S acidic ribosomal protein P0-like OS=Homo sapiens GN=RPLP0P6 PE=5 SV=1	0.00	0.00	0.00	0.00	14.00	0.00	0.00	5.07	0.00	0.00	0.00	0.00	0.00	0.00	0.00	0.00	0.00	0.00	0.00	0.00	0.00	6.65	0.00	0.00	1.59	0.55	>0.999999999999999	2.9
O60925	Prefoldin subunit 1 OS=Homo sapiens GN=PFDN1 PE=1 SV=2	5.86	0.00	2.69	3.64	0.00	3.49	0.00	0.00	0.95	0.00	0.00	2.39	2.11	4.83	0.00	1.66	0.00	0.00	0.00	0.00	0.00	4.99	0.00	1.36	1.59	1.25	>0.999999999999999	1.3
Q9Y3D6	Mitochondrial fission 1 protein OS=Homo sapiens GN=FIS1 PE=1 SV=2	4.88	0.00	2.24	0.00	0.00	2.91	0.00	0.00	0.79	0.00	3.79	4.38	0.00	0.00	0.00	0.00	0.00	0.00	0.00	5.70	0.00	0.00	0.00	0.00	1.58	0.48	>0.999999999999999	3.3
O95379	Tumor necrosis factor alpha-induced protein 8 OS=Homo sapiens GN=TNFAIP8 PE=2 SV=1	3.42	3.23	1.57	0.00	2.68	2.04	0.00	0.00	0.56	1.46	2.65	1.39	0.00	2.82	0.00	0.00	2.64	0.00	0.00	1.81	0.00	2.91	0.00	0.79	1.58	0.91	>0.999999999999999	1.7
P41250	Glycine--tRNA ligase OS=Homo sapiens GN=GARS PE=1 SV=3	2.93	3.92	0.90	0.61	1.53	3.06	0.00	0.63	0.48	0.00	3.22	1.69	0.00	4.23	0.89	0.28	0.75	0.61	0.63	2.20	0.12	2.49	0.61	0.68	1.58	1.13	>0.999999999999999	1.4
Q96K17	Transcription factor BTF3 homolog 4 OS=Homo sapiens GN=BTF3L4 PE=1 SV=1	10.50	4.61	0.00	0.00	0.00	0.00	0.00	0.00	0.00	0.00	3.79	0.00	0.00	0.00	0.00	0.00	0.00	3.07	0.00	2.59	0.00	0.00	0.00	1.08	1.57	0.56	>0.999999999999999	2.8
Q9UDX4	SEC14-like protein 3 OS=Homo sapiens GN=SEC14L3 PE=2 SV=1	12.46	1.61	0.00	0.00	0.00	1.02	0.00	2.38	0.00	0.00	1.33	0.00	0.00	0.00	0.00	0.00	0.00	0.00	0.00	0.00	0.00	0.00	0.00	0.00	1.57	0.00	>0.999999999999999	#DIV/0!
Q9UM54	Unconventional myosin-VI OS=Homo sapiens GN=MYO6 PE=1 SV=4	4.64	2.07	2.13	0.30	1.34	3.93	0.67	0.32	0.44	0.00	1.33	1.59	0.00	3.22	0.44	0.62	0.00	1.07	0.00	2.07	0.00	0.42	0.00	0.11	1.56	0.66	>0.999999999999999	2.4
Q9UGM3	Deleted in malignant brain tumors 1 protein OS=Homo sapiens GN=DMBT1 PE=1 SV=2	1.95	3.00	1.57	1.36	1.73	1.46	2.36	1.43	0.52	0.94	1.51	0.90	0.44	1.01	1.44	0.69	2.64	2.61	1.27	1.81	0.28	1.87	0.77	0.74	1.56	1.30	>0.999999999999999	1.2
Q02790	Peptidyl-prolyl cis-trans isomerase FKBP4 OS=Homo sapiens GN=FKBP4 PE=1 SV=3	6.84	3.00	2.24	0.00	0.00	1.89	1.01	0.95	0.79	0.00	0.00	1.99	0.00	1.21	0.00	0.41	0.00	0.92	0.00	1.68	0.00	0.00	0.92	1.14	1.56	0.52	>0.999999999999999	3.0
P68431	Histone H3.1 OS=Homo sapiens GN=HIST1H3A PE=1 SV=2	0.00	18.67	0.00	0.00	0.00	0.00	0.00	0.00	0.00	0.00	0.00	0.00	0.00	0.00	0.00	0.00	0.00	0.00	0.00	0.00	0.00	0.00	0.00	0.00	1.56	0.00	>0.999999999999999	#DIV/0!
Q8IWE2	Protein NOXP20 OS=Homo sapiens GN=FAM114A1 PE=1 SV=2	2.69	3.92	2.58	0.00	0.00	1.60	2.87	0.79	0.44	0.00	2.08	1.69	0.00	2.22	0.56	1.17	0.00	1.69	0.00	1.43	0.00	1.04	0.00	0.97	1.55	0.76	>0.999999999999999	2.1
P62753	40S ribosomal protein S6 OS=Homo sapiens GN=RPS6 PE=1 SV=1	5.86	2.77	4.37	0.00	0.00	3.49	0.00	0.00	0.95	0.00	0.00	1.20	0.00	2.42	0.00	0.00	0.00	1.84	0.00	1.55	0.00	0.00	0.00	0.00	1.55	0.48	>0.999999999999999	3.2
P28065	Proteasome subunit beta type-9 OS=Homo sapiens GN=PSMB9 PE=1 SV=2	3.42	3.23	0.00	2.12	0.00	4.51	0.00	0.00	1.23	0.00	2.65	1.39	1.23	2.82	0.00	0.97	0.00	2.15	0.00	1.81	0.00	0.00	2.15	0.79	1.55	0.99	>0.999999999999999	1.6
P50452	Serpin B8 OS=Homo sapiens GN=SERPINB8 PE=1 SV=2	3.91	0.00	2.80	2.43	0.00	3.64	0.00	0.00	0.99	0.00	3.03	1.59	0.00	5.04	0.00	1.10	0.00	0.00	0.00	3.24	0.00	5.19	0.00	0.00	1.53	1.21	>0.999999999999999	1.3
P48735	Isocitrate dehydrogenase [NADP], mitochondrial OS=Homo sapiens GN=IDH2 PE=1 SV=2	3.18	3.00	2.35	0.00	0.00	5.39	0.00	3.33	0.52	0.63	0.00	0.00	0.00	4.23	0.00	0.00	0.00	0.00	0.00	1.68	0.00	0.00	0.00	0.00	1.53	0.49	>0.999999999999999	3.1
Q8IY31	Intraflagellar transport protein 20 homolog OS=Homo sapiens GN=IFT20 PE=1 SV=1	11.97	0.00	0.00	0.00	4.22	0.00	0.00	0.00	0.00	0.00	0.00	2.19	0.00	0.00	0.00	0.00	0.00	0.00	0.00	0.00	0.00	0.00	0.00	0.00	1.53	0.00	>0.999999999999999	#DIV/0!
P09132	Signal recognition particle 19 kDa protein OS=Homo sapiens GN=SRP19 PE=1 SV=3	5.13	4.84	0.00	0.00	0.00	3.06	0.00	0.00	0.83	0.00	0.00	4.48	1.84	4.23	0.00	0.00	0.00	0.00	0.00	2.72	0.65	0.00	0.00	0.00	1.53	0.79	>0.999999999999999	1.9
P28482	Mitogen-activated protein kinase 1 OS=Homo sapiens GN=MAPK1 PE=1 SV=3	8.79	3.69	0.90	0.00	0.00	0.00	0.00	0.00	0.32	0.00	3.03	1.59	0.70	3.22	0.00	0.00	0.00	0.00	0.00	1.04	0.00	3.32	0.00	0.00	1.53	0.69	>0.999999999999999	2.2
Q99439	Calponin-2 OS=Homo sapiens GN=CNN2 PE=1 SV=4	2.44	2.30	2.24	1.52	0.00	2.91	0.00	0.00	0.00	2.09	3.79	1.00	0.88	0.00	0.00	0.00	1.89	0.00	0.00	0.00	0.00	4.16	1.53	0.57	1.52	0.75	>0.999999999999999	2.0
O95716	Ras-related protein Rab-3D OS=Homo sapiens GN=RAB3D PE=1 SV=1	0.00	10.83	0.00	0.00	0.00	0.00	0.00	7.45	0.00	0.00	0.00	0.00	0.00	5.84	0.00	0.00	0.00	0.00	0.00	0.00	0.00	0.00	0.00	0.00	1.52	0.49	>0.999999999999999	3.1
P55042	GTP-binding protein RAD OS=Homo sapiens GN=RRAD PE=1 SV=2	5.13	0.00	1.12	0.00	1.92	4.80	0.00	3.33	0.00	0.00	1.89	0.00	0.00	0.00	0.00	0.00	0.00	0.00	0.00	0.00	0.00	0.00	0.00	0.00	1.52	0.00	>0.999999999999999	#DIV/0!
Q8TDQ7	Glucosamine-6-phosphate isomerase 2 OS=Homo sapiens GN=GNPDA2 PE=1 SV=1	5.37	5.07	0.00	0.00	0.00	3.20	1.86	0.00	0.44	0.00	0.00	2.19	0.96	2.22	0.00	0.76	0.00	1.69	0.00	2.85	0.34	0.00	0.00	0.00	1.51	0.73	>0.999999999999999	2.1
P61221	ATP-binding cassette sub-family E member 1 OS=Homo sapiens GN=ABCE1 PE=1 SV=1	3.66	2.30	1.68	1.52	0.96	2.18	0.84	0.79	0.20	0.00	1.89	2.09	0.44	2.01	0.00	0.00	0.00	0.77	0.79	1.30	0.15	1.04	0.00	0.28	1.51	0.57	>0.999999999999999	2.7
P12532	Creatine kinase U-type, mitochondrial OS=Homo sapiens GN=CKMT1A PE=1 SV=1	5.37	1.61	1.57	0.00	0.00	4.51	0.00	1.11	1.23	0.00	1.33	1.39	0.61	0.00	3.44	0.97	0.00	2.15	0.00	0.00	0.22	0.00	0.00	0.40	1.51	0.65	>0.999999999999999	2.3
Q16543	Hsp90 co-chaperone Cdc37 OS=Homo sapiens GN=CDC37 PE=1 SV=1	1.71	0.00	0.78	1.06	1.34	6.26	0.00	1.11	0.59	0.00	2.84	2.39	0.00	1.41	0.00	0.48	0.00	0.00	1.11	1.94	0.74	1.45	0.00	0.85	1.51	0.67	>0.999999999999999	2.3
Q15785	Mitochondrial import receptor subunit TOM34 OS=Homo sapiens GN=TOMM34 PE=1 SV=2	4.88	2.07	0.00	0.00	0.00	6.40	0.00	0.00	0.00	0.00	3.79	0.90	0.79	1.81	0.00	0.00	0.00	0.00	0.00	1.17	0.00	0.00	0.00	0.00	1.50	0.31	>0.999999999999999	4.8
P34896	Serine hydroxymethyltransferase, cytosolic OS=Homo sapiens GN=SHMT1 PE=1 SV=1	10.50	1.38	0.67	0.00	0.00	3.93	0.00	0.00	0.24	0.00	0.00	1.29	0.00	0.00	0.67	0.41	0.00	0.00	0.00	0.00	0.00	2.70	0.00	0.34	1.50	0.34	>0.999999999999999	4.4
P22079	Lactoperoxidase OS=Homo sapiens GN=LPO PE=1 SV=2	0.00	0.92	4.82	1.21	2.49	0.00	3.71	3.49	0.52	0.42	0.00	0.40	2.81	0.81	3.56	0.55	6.03	2.61	0.63	2.20	0.40	2.70	0.00	0.23	1.50	1.88	>0.999999999999999	0.8
Q9Y315	Putative deoxyribose-phosphate aldolase OS=Homo sapiens GN=DERA PE=1 SV=2	4.88	2.07	1.01	0.00	0.00	4.51	0.00	1.43	0.36	0.00	1.70	1.99	0.00	6.24	0.00	0.00	0.00	1.38	0.00	2.59	0.28	0.00	1.38	0.51	1.50	1.03	>0.999999999999999	1.5
P22059	Oxysterol-binding protein 1 OS=Homo sapiens GN=OSBP PE=1 SV=1	3.66	3.46	1.68	1.06	0.77	2.91	0.67	0.63	0.20	0.00	1.33	1.49	0.35	3.02	0.44	0.00	0.00	0.00	0.00	1.43	0.12	0.83	1.07	0.23	1.50	0.62	>0.999999999999999	2.4
P49247	Ribose-5-phosphate isomerase OS=Homo sapiens GN=RPIA PE=1 SV=3	5.13	0.00	1.12	0.00	0.00	4.80	0.00	0.00	0.40	0.00	1.89	4.58	0.00	2.01	0.00	1.45	0.00	1.53	0.00	2.72	0.00	0.00	0.00	1.87	1.49	0.80	>0.999999999999999	1.9
Q5XKE5	Keratin, type II cytoskeletal 79 OS=Homo sapiens GN=KRT79 PE=1 SV=2	0.00	4.15	1.34	2.73	0.00	0.00	2.02	1.90	0.48	1.88	3.41	0.00	1.05	3.63	0.00	0.83	0.00	1.84	2.85	0.00	0.00	2.49	1.84	0.68	1.49	1.27	>0.999999999999999	1.2
P21283	V-type proton ATPase subunit C 1 OS=Homo sapiens GN=ATP6V1C1 PE=1 SV=4	3.91	0.00	0.78	0.00	1.34	4.95	1.18	0.00	0.28	0.00	3.03	2.39	0.00	1.41	1.78	0.00	0.00	3.68	0.00	3.11	0.00	0.00	1.07	1.93	1.49	1.08	>0.999999999999999	1.4
Q16774	Guanylate kinase OS=Homo sapiens GN=GUK1 PE=1 SV=2	8.06	3.46	0.00	0.00	0.00	2.18	0.00	0.00	1.31	0.00	2.84	0.00	0.00	0.00	0.00	0.00	0.00	0.00	0.00	0.00	0.00	0.00	0.00	0.85	1.49	0.07	>0.999999999999999	21.0
Q96FV0	Leucine-rich repeat-containing protein 46 OS=Homo sapiens GN=LRRC46 PE=2 SV=1	2.20	4.38	1.01	0.00	3.64	0.00	0.00	1.43	0.36	0.00	1.70	3.09	0.00	0.00	0.00	0.00	0.00	0.00	0.00	0.00	0.00	0.00	0.00	0.00	1.48	0.00	>0.999999999999999	#DIV/0!
Q15717	ELAV-like protein 1 OS=Homo sapiens GN=ELAVL1 PE=1 SV=2	2.20	2.07	0.00	0.00	1.73	1.31	0.00	1.43	0.36	0.00	5.68	2.99	0.00	1.81	0.00	0.00	0.00	1.70	1.43	1.17	0.28	0.00	0.00	0.51	1.48	0.57	>0.999999999999999	2.6
Q14677	Clathrin interactor 1 OS=Homo sapiens GN=CLINT1 PE=1 SV=1	3.66	1.15	1.12	0.76	0.96	3.06	2.53	0.79	0.40	0.00	0.00	3.29	0.44	1.01	1.11	0.34	0.00	0.00	0.00	0.65	0.15	1.04	0.00	0.28	1.48	0.42	>0.999999999999999	3.5
Q15843	NEDD8 OS=Homo sapiens GN=NEDD8 PE=1 SV=1	0.00	0.00	4.26	0.00	0.00	0.00	0.00	6.02	3.61	0.00	0.00	3.79	3.33	0.00	0.00	0.00	0.00	5.83	0.00	4.92	0.00	7.90	5.83	5.17	1.47	2.75	>0.999999999999999	0.5
P62333	26S protease regulatory subunit 10B OS=Homo sapiens GN=PSMC6 PE=1 SV=1	1.71	0.00	1.68	1.06	0.00	6.26	0.00	0.00	0.59	0.73	1.33	4.28	0.61	3.02	0.78	0.48	0.00	0.00	0.00	1.94	0.74	1.45	2.30	0.40	1.47	0.98	>0.999999999999999	1.5
Q9BV40	Vesicle-associated membrane protein 8 OS=Homo sapiens GN=VAMP8 PE=1 SV=1	0.00	6.91	3.36	0.00	0.00	4.37	0.00	0.00	0.00	0.00	0.00	2.99	2.63	13.90	3.33	2.07	0.00	0.00	0.00	3.89	0.00	0.00	0.00	1.70	1.47	2.29	>0.999999999999999	0.6
P61923	Coatomer subunit zeta-1 OS=Homo sapiens GN=COPZ1 PE=1 SV=1	0.00	3.92	0.00	0.00	0.00	8.59	0.00	2.69	0.67	0.00	0.00	1.69	0.00	3.42	1.89	0.00	0.00	0.00	0.00	4.66	0.00	0.00	0.00	0.97	1.46	0.91	>0.999999999999999	1.6
Q99447	Ethanolamine-phosphate cytidylyltransferase OS=Homo sapiens GN=PCYT2 PE=1 SV=1	5.86	1.61	0.00	0.00	1.34	3.49	0.00	0.00	0.00	0.00	2.84	2.39	0.00	0.00	0.00	0.00	0.00	0.00	0.00	0.00	0.00	0.00	0.00	0.00	1.46	0.00	>0.999999999999999	#DIV/0!
P69891	Hemoglobin subunit gamma-1 OS=Homo sapiens GN=HBG1 PE=1 SV=2	0.00	0.00	0.00	0.00	8.82	0.00	0.00	0.00	0.00	0.00	8.71	0.00	0.00	0.00	0.00	0.00	0.00	0.00	0.00	0.00	0.00	0.00	0.00	0.00	1.46	0.00	>0.999999999999999	#DIV/0!
P01824	Ig heavy chain V-II region WAH OS=Homo sapiens PE=1 SV=1	0.00	0.00	0.00	3.64	10.36	0.00	0.00	0.00	0.95	2.50	0.00	0.00	0.00	0.00	2.67	1.66	0.00	0.00	0.00	0.00	0.74	0.00	0.00	0.00	1.45	0.42	>0.999999999999999	3.4
P02812	Basic salivary proline-rich protein 2 OS=Homo sapiens GN=PRB2 PE=1 SV=3	1.95	1.84	1.90	5.61	3.26	0.00	1.35	0.00	0.67	0.83	0.00	0.00	0.70	1.61	0.89	0.00	6.98	0.00	0.00	1.04	0.52	0.00	0.00	1.48	1.45	1.10	>0.999999999999999	1.3
P53618	Coatomer subunit beta OS=Homo sapiens GN=COPB1 PE=1 SV=3	1.47	3.69	1.46	0.45	0.00	6.84	0.51	0.48	0.63	0.00	0.57	1.29	0.26	1.81	0.33	0.21	0.57	1.38	0.00	0.39	0.18	0.00	0.00	0.34	1.45	0.46	>0.999999999999999	3.2
A0MZ66	Shootin-1 OS=Homo sapiens GN=KIAA1598 PE=1 SV=4	2.20	2.07	0.56	0.76	1.73	3.64	0.84	0.79	0.00	0.00	1.70	3.09	0.79	1.01	0.00	0.34	0.00	1.38	0.00	1.17	0.00	1.04	0.77	0.28	1.45	0.56	>0.999999999999999	2.6
P60866	40S ribosomal protein S20 OS=Homo sapiens GN=RPS20 PE=1 SV=1	0.00	0.00	2.80	3.79	0.00	8.30	0.00	0.00	0.00	0.00	0.00	2.49	0.00	5.04	0.00	0.00	0.00	0.00	0.00	3.24	0.00	0.00	0.00	1.42	1.45	0.81	>0.999999999999999	1.8
Q9UH65	Switch-associated protein 70 OS=Homo sapiens GN=SWAP70 PE=1 SV=1	3.66	3.46	2.91	0.00	0.00	2.91	0.00	1.59	0.40	0.00	0.95	1.49	0.00	3.02	0.56	0.69	0.00	1.53	0.00	1.30	0.00	1.04	0.77	1.48	1.45	0.86	>0.999999999999999	1.7
Q96GG9	DCN1-like protein 1 OS=Homo sapiens GN=DCUN1D1 PE=1 SV=1	5.62	5.30	1.23	0.00	0.00	1.60	0.00	0.00	0.44	0.00	2.08	1.10	0.00	2.22	1.22	0.00	0.00	0.00	0.00	1.43	0.00	0.00	0.00	1.31	1.45	0.51	>0.999999999999999	2.8
P55735	Protein SEC13 homolog OS=Homo sapiens GN=SEC13 PE=1 SV=3	2.20	4.38	1.01	1.36	0.00	4.37	0.00	1.43	0.00	0.00	1.70	0.90	0.00	1.81	0.00	0.00	0.00	0.00	0.00	2.46	0.00	0.00	0.00	1.08	1.45	0.45	>0.999999999999999	3.2
P83731	60S ribosomal protein L24 OS=Homo sapiens GN=RPL24 PE=1 SV=1	0.00	4.38	4.59	0.00	0.00	2.77	0.00	0.00	0.00	0.00	3.60	1.89	0.00	3.83	0.00	1.31	0.00	2.91	0.00	5.31	0.00	0.00	0.00	1.08	1.44	1.20	>0.999999999999999	1.2
Q92841	Probable ATP-dependent RNA helicase DDX17 OS=Homo sapiens GN=DDX17 PE=1 SV=2	3.18	0.92	1.46	1.21	0.00	3.20	1.35	0.00	0.00	0.00	4.17	1.69	0.00	0.00	0.00	0.00	0.00	0.00	2.06	0.00	0.00	0.00	0.00	0.74	1.43	0.23	>0.999999999999999	6.1
P32320	Cytidine deaminase OS=Homo sapiens GN=CDA PE=1 SV=2	0.00	0.00	0.00	11.22	3.84	0.00	0.00	0.00	0.00	2.09	0.00	0.00	1.75	0.00	0.00	0.00	0.00	0.00	0.00	0.00	0.00	9.35	0.00	0.00	1.43	0.93	>0.999999999999999	1.5
P08185	Corticosteroid-binding globulin OS=Homo sapiens GN=SERPINA6 PE=1 SV=1	1.71	0.00	2.69	2.27	1.34	0.00	1.18	2.38	0.28	0.73	4.54	0.00	1.32	1.41	4.67	1.03	6.22	1.07	2.38	0.91	0.46	6.86	1.07	0.40	1.43	2.32	>0.999999999999999	0.6
P38919	Eukaryotic initiation factor 4A-III OS=Homo sapiens GN=EIF4A3 PE=1 SV=4	5.37	1.61	0.00	0.00	1.34	3.20	3.71	0.00	0.56	0.00	1.33	0.00	0.00	0.00	0.00	0.00	0.00	2.15	0.00	0.91	0.00	0.00	0.00	0.00	1.43	0.25	>0.999999999999999	5.6
Q53EL6	Programmed cell death protein 4 OS=Homo sapiens GN=PDCD4 PE=1 SV=2	3.18	0.00	0.67	0.00	0.00	5.24	1.01	2.06	0.24	0.00	1.14	3.59	0.00	2.62	0.00	0.00	0.00	0.00	0.00	1.68	0.00	0.00	0.00	1.14	1.43	0.45	>0.999999999999999	3.1
Q9H2U2	Inorganic pyrophosphatase 2, mitochondrial OS=Homo sapiens GN=PPA2 PE=1 SV=2	2.20	0.00	2.02	1.36	1.73	4.08	1.52	0.00	0.71	0.00	1.70	1.79	0.00	0.00	1.00	0.62	0.00	1.38	1.43	0.00	0.00	0.00	1.38	0.51	1.43	0.53	>0.999999999999999	2.7
Q9Y6G9	Cytoplasmic dynein 1 light intermediate chain 1 OS=Homo sapiens GN=DYNC1LI1 PE=1 SV	4.40	5.76	1.34	0.91	0.00	0.00	1.01	0.00	0.71	0.00	1.14	1.79	0.53	2.42	0.67	0.00	0.00	0.92	0.00	3.24	0.18	1.25	0.92	0.34	1.42	0.87	>0.999999999999999	1.6
Q6BCY4	NADH-cytochrome b5 reductase 2 OS=Homo sapiens GN=CYB5R2 PE=1 SV=1	2.69	5.07	0.00	1.67	2.11	1.60	0.00	0.00	0.44	0.00	0.00	3.49	0.00	0.00	0.00	0.76	0.00	0.00	0.00	1.43	0.00	0.00	0.00	0.00	1.42	0.18	>0.999999999999999	7.8
O14579	Coatomer subunit epsilon OS=Homo sapiens GN=COPE PE=1 SV=3	4.88	2.30	1.12	1.52	0.00	2.91	0.00	0.00	0.40	0.00	1.89	1.99	0.00	2.01	0.00	0.00	0.00	0.00	0.00	1.30	0.62	0.00	0.00	0.57	1.42	0.37	>0.999999999999999	3.8
Q96A49	Synapse-associated protein 1 OS=Homo sapiens GN=SYAP1 PE=1 SV=1	4.15	0.00	1.90	1.21	1.53	0.00	1.35	1.27	0.67	0.00	3.22	1.69	1.49	1.61	0.89	0.00	0.00	0.00	0.00	1.04	0.00	0.00	0.00	0.97	1.42	0.50	>0.999999999999999	2.8
P61160	Actin-related protein 2 OS=Homo sapiens GN=ACTR2 PE=1 SV=1	1.71	1.61	0.78	1.06	1.34	2.18	1.18	0.00	0.28	0.73	4.54	1.49	0.61	3.02	1.67	0.48	1.32	2.30	1.11	0.00	0.46	4.99	3.68	1.36	1.41	1.75	>0.999999999999999	0.8
P23142	Fibulin-1 OS=Homo sapiens GN=FBLN1 PE=1 SV=4	0.98	1.84	0.90	1.97	4.22	1.16	0.67	0.63	0.32	1.36	2.46	0.40	1.93	4.43	2.44	2.55	3.20	0.61	2.69	1.68	0.00	0.83	1.99	0.23	1.41	1.88	>0.999999999999999	0.7
O43390	Heterogeneous nuclear ribonucleoprotein R OS=Homo sapiens GN=HNRNPR PE=1 SV=1	3.42	2.07	1.01	0.00	0.96	2.91	0.00	0.00	0.36	0.94	3.79	1.39	0.00	0.00	0.00	0.00	0.94	0.00	0.79	1.17	0.15	1.04	0.00	0.51	1.40	0.38	>0.999999999999999	3.7
P07814	Bifunctional glutamate/proline--tRNA ligase OS=Homo sapiens GN=EPRS PE=1 SV=5	2.93	3.69	1.12	0.00	0.38	4.80	0.00	0.63	0.56	0.00	1.89	0.80	0.35	2.82	0.22	0.41	0.00	1.23	0.00	2.07	0.00	1.66	0.00	0.34	1.40	0.76	>0.999999999999999	1.8
Q96A72	Protein mago nashi homolog 2 OS=Homo sapiens GN=MAGOHB PE=1 SV=1	4.88	0.00	2.24	0.00	0.00	6.26	3.37	0.00	0.00	0.00	0.00	0.00	0.00	0.00	0.00	0.00	0.00	0.00	0.00	2.59	0.00	0.00	0.00	1.14	1.40	0.31	>0.999999999999999	4.5
O94903	Proline synthase co-transcribed bacterial homolog protein OS=Homo sapiens GN=PROSC F	2.69	0.00	1.23	0.00	2.11	5.24	1.86	0.00	0.44	0.00	2.08	1.10	0.96	0.00	0.00	0.00	2.07	1.69	1.74	0.00	0.00	2.29	0.00	0.62	1.39	0.78	>0.999999999999999	1.8
O14980	Exportin-1 OS=Homo sapiens GN=XPO1 PE=1 SV=1	2.69	1.15	0.56	2.12	0.96	4.37	0.84	0.79	0.32	0.00	1.51	1.39	0.00	0.60	0.33	0.34	0.57	0.00	0.48	1.04	0.25	2.29	0.00	0.62	1.39	0.54	>0.999999999999999	2.6
Q8WZA0	Protein LZIC OS=Homo sapiens GN=LZIC PE=1 SV=1	3.91	7.84	0.00	0.00	0.00	4.95	0.00	0.00	0.00	0.00	0.00	0.00	0.00	0.00	0.00	0.00	0.00	0.00	0.00	0.00	0.00	0.00	0.00	0.00	1.39	0.00	>0.999999999999999	#DIV/0!
Q13596	Sorting nexin-1 OS=Homo sapiens GN=SNX1 PE=1 SV=3	2.69	4.15	0.67	1.67	0.00	2.62	1.01	0.00	0.00	0.00	2.08	1.79	0.00	3.63	0.00	1.24	0.00	1.69	0.00	2.33	0.00	1.25	0.00	1.02	1.39	0.93	>0.999999999999999	1.5
P14923	Junction plakoglobin OS=Homo sapiens GN=JUP PE=1 SV=3	0.98	1.84	1.34	0.61	0.77	1.75	1.35	0.63	0.67	2.30	3.22	1.20	0.35	1.61	0.89	0.83	0.00	1.84	1.27	2.20	0.12	0.00	0.00	0.68	1.39	0.82	>0.999999999999999	1.7
O14744	Protein arginine N-methyltransferase 5 OS=Homo sapiens GN=PRMT5 PE=1 SV=4	2.20	4.38	0.00	0.00	2.68	2.04	1.52	0.00	0.16	0.00	2.65	0.90	0.79	1.81	1.00	0.00	0.00	0.61	0.00	0.00	0.00	0.00	0.00	0.00	1.38	0.35	>0.999999999999999	3.9
Q13155	Aminoacyl tRNA synthase complex-interacting multifunctional protein 2 OS=Homo sapiens (	2.20	2.07	2.13	0.00	1.73	2.77	1.52	1.43	0.00	0.00	1.70	0.90	0.79	1.81	0.00	0.00	0.00	1.38	0.00	1.17	0.00	0.00	0.00	0.00	1.37	0.43	>0.999999999999999	3.2
Q92882	Osteoclast-stimulating factor 1 OS=Homo sapiens GN=OSTF1 PE=1 SV=2	3.42	0.00	0.00	2.12	2.68	4.37	0.00	0.00	0.00	0.00	2.65	0.00	0.00	2.82	1.56	0.00	0.00	0.00	0.00	1.81	0.43	0.00	0.00	0.79	1.37	0.62	>0.999999999999999	2.2
P06681	Complement C2 OS=Homo sapiens GN=C2 PE=1 SV=2	0.98	0.92	1.79	3.18	4.03	1.16	0.67	1.90	0.16	0.00	0.76	0.80	0.70	0.81	3.33	2.07	3.96	1.84	4.12	1.04	0.25	0.83	1.23	0.91	1.36	1.76	>0.999999999999999	0.8
A6NDG6	Phosphoglycolate phosphatase OS=Homo sapiens GN=PGP PE=1 SV=1	2.44	2.30	1.12	0.00	0.00	4.66	0.00	0.00	0.00	0.00	3.79	1.99	0.00	4.03	0.00	0.00	0.00	1.53	0.00	0.00	0.00	2.08	0.00	0.00	1.36	0.64	>0.999999999999999	2.1
Q9Y2Q3	Glutathione S-transferase kappa 1 OS=Homo sapiens GN=GSTK1 PE=1 SV=3	3.18	0.00	3.14	0.00	2.49	1.89	2.19	2.06	0.00	0.00	0.00	1.29	1.14	0.00	0.00	0.90	0.00	1.99	2.06	1.68	0.40	2.70	0.00	1.59	1.35	1.04	>0.999999999999999	1.3
Q13153	Serine/threonine-protein kinase PAK 1 OS=Homo sapiens GN=PAK1 PE=1 SV=2	2.69	3.92	0.56	0.76	0.96	3.35	0.84	0.79	0.67	0.00	0.00	1.69	0.00	0.00	1.22	0.34	0.94	0.00	0.00	1.43	0.00	0.00	0.00	0.28	1.35	0.35	>0.999999999999999	3.8
Q53FA7	Quinone oxidoreductase PIG3 OS=Homo sapiens GN=TP53I3 PE=1 SV=2	2.20	2.07	0.00	1.36	0.00	4.51	1.52	0.00	0.00	0.00	3.60	0.90	0.00	1.81	1.00	2.14	0.00	0.00	0.00	2.46	0.28	3.95	1.38	1.08	1.35	1.17	>0.999999999999999	1.1
Q6P996	Pyridoxal-dependent decarboxylase domain-containing protein 1 OS=Homo sapiens GN=PD	4.88	0.00	2.80	0.00	0.00	4.95	0.00	1.27	0.63	0.00	0.00	1.59	0.70	3.22	0.89	0.00	0.00	0.61	0.63	3.76	0.25	0.00	1.23	0.45	1.34	0.98	>0.999999999999999	1.4
O60547	GDP-mannose 4,6 dehydratase OS=Homo sapiens GN=GMDS PE=1 SV=1	1.95	3.69	1.79	0.00	0.00	3.64	1.35	0.00	1.39	0.00	1.51	0.80	0.00	3.22	0.89	0.00	0.00	1.23	0.00	0.00	0.77	1.66	1.23	0.45	1.34	0.79	>0.999999999999999	1.7
P09382	Galectin-1 OS=Homo sapiens GN=LGALS1 PE=1 SV=2	0.00	5.30	2.58	0.00	0.00	0.00	3.88	0.00	0.00	0.00	4.35	0.00	0.00	4.63	0.00	1.59	0.00	0.00	0.00	0.00	0.00	10.39	0.00	0.00	1.34	1.38	>0.999999999999999	1.0
Q9Y6E0	Serine/threonine-protein kinase 24 OS=Homo sapiens GN=STK24 PE=1 SV=1	7.08	1.61	0.00	0.00	0.00	5.53	0.00	0.00	0.56	0.00	1.33	0.00	0.00	2.82	0.00	0.00	0.00	0.00	0.00	2.72	0.00	0.00	0.00	0.00	1.34	0.46	>0.999999999999999	2.9
P53611	Geranylgeranyl transferase type-2 subunit beta OS=Homo sapiens GN=RABGGTB PE=1 S\	2.20	4.15	1.01	0.00	0.00	1.31	3.04	1.43	0.36	0.00	1.70	0.90	0.79	0.00	0.00	0.00	0.00	0.00	0.00	1.17	0.00	0.00	1.38	0.51	1.34	0.32	>0.999999999999999	4.2
Q16186	Proteasomal ubiquitin receptor ADRM1 OS=Homo sapiens GN=ADRM1 PE=1 SV=2	3.91	3.69	0.90	0.00	0.00	2.33	0.00	0.00	0.63	0.00	3.03	1.59	0.70	0.00	0.89	0.55	0.00	2.45	0.00	1.04	0.49	0.00	1.23	0.91	1.34	0.69	>0.999999999999999	1.9
A0AVT1	Ubiquitin-like modifier-activating enzyme 6 OS=Homo sapiens GN=UBA6 PE=1 SV=1	2.69	3.23	0.67	0.91	0.58	2.04	1.01	0.48	0.56	0.31	2.08	1.39	0.26	2.22	0.67	0.41	1.13	0.92	0.48	2.33	0.09	1.25	0.92	0.45	1.33	0.93	>0.999999999999999	1.4
P17844	Probable ATP-dependent RNA helicase DDX5 OS=Homo sapiens GN=DDX5 PE=1 SV=1	3.66	1.15	0.00	0.00	0.96	3.78	0.00	0.00	0.59	0.00	3.79	1.99	0.00	1.01	0.00	0.00	0.00	1.53	0.00	1.94	0.15	0.00	0.00	0.85	1.33	0.46	>0.999999999999999	2.9
P27694	Replication protein A 70 kDa DNA-binding subunit OS=Homo sapiens GN=RPA1 PE=1 SV=2	3.66	2.30	0.00	1.52	0.00	4.66	0.00	0.00	0.40	0.00	1.89	1.49	0.00	2.01	0.56	0.34	0.00	0.00	0.00	0.65	0.00	0.00	0.00	0.57	1.33	0.34	>0.999999999999999	3.9
Q12904	Aminoacyl tRNA synthase complex-interacting multifunctional protein 1 OS=Homo sapiens (	2.44	4.61	1.12	1.52	0.00	2.91	0.00	0.00	0.40	0.00	1.89	1.00	0.88	0.00	1.11	0.69	0.00	1.53	0.00	2.59	0.31	0.00	0.00	1.14	1.32	0.69	>0.999999999999999	1.9
Q9UL25	Ras-related protein Rab-21 OS=Homo sapiens GN=RAB21 PE=1 SV=3	7.08	6.68	0.00	0.00	0.00	2.04	0.00	0.00	0.00	0.00	0.00	0.00	0.00	2.82	0.00	0.00	0.00	0.00	0.00	0.00	0.00	0.00	0.00	0.79	1.32	0.30	>0.999999999999999	4.4
P18440	Arylamine N-acetyltransferase 1 OS=Homo sapiens GN=NAT1 PE=1 SV=2	2.44	2.30	1.12	0.00	1.92	1.46	1.69	1.59	0.40	0.00	1.89	1.00	0.00	2.01	0.00	0.00	0.00	0.00	0.00	0.00	0.00	2.08	1.53	0.00	1.32	0.47	>0.999999999999999	2.8
Q96BW5	Phosphotriesterase-related protein OS=Homo sapiens GN=PTER PE=1 SV=1	1.95	1.84	1.90	0.00	0.00	2.47	0.00	2.69	0.67	0.00	1.51	2.69	2.37	5.44	1.89	1.86	0.00	5.83	0.00	4.92	0.00	1.66	1.23	2.16	1.31	2.28	>0.999999999999999	0.6
Q9Y2V2	Calcium-regulated heat stable protein 1 OS=Homo sapiens GN=CARHSP1 PE=1 SV=2	0.00	0.00	2.35	0.00	0.00	6.84	0.00	0.00	1.86	0.00	0.00	4.68	0.00	0.00	0.00	1.45	0.00	3.22	0.00	0.00	0.00	0.00	0.00	0.00	1.31	0.39	>0.999999999999999	3.4
P54105	Methylosome subunit pICln OS=Homo sapiens GN=CLNS1A PE=1 SV=1	3.18	0.00	1.46	1.97	0.00	1.89	2.19	2.06	0.52	0.00	2.46	0.00	0.00	2.62	0.00	0.90	0.00	0.00	0.00	1.68	0.00	0.00	0.00	0.00	1.31	0.43	>0.999999999999999	3.0
P62304	Small nuclear ribonucleoprotein E OS=Homo sapiens GN=SNRPE PE=1 SV=1	0.00	7.38	3.59	0.00	0.00	4.66	0.00	0.00	0.00	0.00	0.00	0.00	0.00	0.00	0.00	0.00	0.00	0.00	0.00	0.00	0.00	0.00	0.00	0.00	1.30	0.00	>0.999999999999999	#DIV/0!
Q99933	BAG family molecular chaperone regulator 1 OS=Homo sapiens GN=BAG1 PE=1 SV=4	4.40	1.84	0.90	0.00	1.53	4.08	1.35	0.00	0.71	0.00	0.00	0.80	0.00	1.61	0.00	0.55	0.00	1.23	0.00	2.33	0.00	0.00	1.23	1.02	1.30	0.66	>0.999999999999999	2.0
Q9Y6Q5	AP-1 complex subunit mu-2 OS=Homo sapiens GN=AP1M2 PE=1 SV=4	1.71	1.61	1.57	0.00	0.00	7.13	0.00	0.00	0.56	0.00	0.00	2.99	0.00	0.00	0.00	0.00	0.00	0.00	0.00	0.91	0.22	0.00	0.00	0.40	1.30	0.13	>0.999999999999999	10.2
O75821	Eukaryotic translation initiation factor 3 subunit G OS=Homo sapiens GN=EIF3G PE=1 SV=2	4.64	0.00	2.13	0.00	0.00	2.77	1.52	0.00	0.00	0.00	3.60	0.90	0.00	3.83	0.00	1.31	0.00	2.91	0.00	1.17	0.28	0.00	0.00	1.08	1.30	0.88	>0.999999999999999	1.5
P55263	Adenosine kinase OS=Homo sapiens GN=ADK PE=1 SV=2	4.15	0.00	1.90	0.00	0.00	1.16	1.35	0.00	1.03	0.00	0.00	5.88	0.70	3.42	0.00	0.55	0.00	0.00	0.00	2.20	0.25	1.66	1.23	0.45	1.29	0.87	>0.999999999999999	1.5
O00410	Importin-5 OS=Homo sapiens GN=IPO5 PE=1 SV=4	4.15	2.54	0.90	0.45	0.58	2.47	0.00	0.48	0.32	0.00	1.51	1.99	0.70	1.01	0.00	0.34	0.00	0.00	0.00	0.65	0.15	0.00	0.00	0.45	1.28	0.28	>0.999999999999999	4.6
P18621	60S ribosomal protein L17 OS=Homo sapiens GN=RPL17 PE=1 SV=3	3.91	3.69	0.00	2.43	0.00	2.33	0.00	0.00	0.00	0.00	3.03	0.00	0.00	6.65	0.00	1.10	0.00	0.00	0.00	4.28	0.00	0.00	0.00	0.91	1.28	1.08	>0.999999999999999	1.2
Q52LJ0	Protein FAM98B OS=Homo sapiens GN=FAM98B PE=1 SV=1	7.08	0.00	0.00	1.36	0.00	2.62	0.00	0.00	0.00	0.00	3.41	0.90	0.00	0.00	0.00	0.00	0.00	2.76	0.00	0.00	0.00	0.00	0.00	0.00	1.28	0.23	>0.999999999999999	5.6
P01719	Ig lambda chain V-V region DEL OS=Homo sapiens PE=1 SV=1	0.00	0.00	0.00	4.55	5.75	0.00	5.06	0.00	0.00	0.00	0.00	0.00	0.00	0.00	3.33	2.07	13.01	0.00	4.76	0.00	0.00	0.00	0.00	0.00	1.28	1.93	>0.999999999999999	0.7
P61254	60S ribosomal protein L26 OS=Homo sapiens GN=RPL26 PE=1 SV=1	10.50	0.00	4.82	0.00	0.00	0.00	0.00	0.00	0.00	0.00	0.00	0.00	0.00	0.00	0.00	0.00	0.00	0.00	0.00	0.00	0.00	0.00	0.00	0.00	1.28	0.00	>0.999999999999999	#DIV/0!
Q9NZL9	Methionine adenosyltransferase 2 subunit beta OS=Homo sapiens GN=MAT2B PE=1 SV=1	2.20	2.07	1.01	0.00	0.00	7.57	0.00	0.00	0.71	0.00	1.70	0.00	0.00	1.81	0.00	0.00	0.00	0.00	0.00	0.00	0.00	3.74	0.00	0.51	1.27	0.51	>0.999999999999999	2.5
Q14258	E3 ubiquitin/ISG15 ligase TRIM25 OS=Homo sapiens GN=TRIM25 PE=1 SV=2	1.22	2.07	2.13	0.76	0.96	3.64	1.52	0.00	0.36	0.00	1.70	0.90	0.44	1.81	0.56	0.00	0.00	0.77	0.00	0.65	0.00	1.04	0.00	0.28	1.27	0.46	>0.999999999999999	2.8
O14933	Ubiquitin/ISG15-conjugating enzyme E2 L6 OS=Homo sapiens GN=UBE2L6 PE=1 SV=4	0.00	4.38	0.00	2.88	0.00	2.77	0.00	0.00	1.63	0.00	3.60	0.00	0.00	3.83	0.00	1.31	0.00	2.91	0.00	0.00	0.59	8.52	0.00	1.08	1.27	1.52	>0.999999999999999	0.8
P36873	Serine/threonine-protein phosphatase PP1-gamma catalytic subunit OS=Homo sapiens GN=	0.00	2.07	0.00	2.73	3.45	0.00	0.00	2.85	3.17	0.94	0.00	0.00	0.79	0.00	0.00	2.00	3.39	0.00	2.85	3.76	0.00	8.31	2.76	0.00	1.27	1.99	>0.999999999999999	0.6
P08571	Monocyte differentiation antigen CD14 OS=Homo sapiens GN=CD14 PE=1 SV=2	0.00	0.00	1.90	0.00	4.99	0.00	2.87	1.27	1.03	0.83	1.51	0.80	0.00	0.00	0.00	2.89	0.00	3.20	0.00	1.27	0.00	0.52	1.66	1.23	0.97	1.27	0.98	>0.999999999999999	1.3
Q15084	Protein disulfide-isomerase A6 OS=Homo sapiens GN=PDIA6 PE=1 SV=1	1.71	3.23	2.47	0.00	0.00	2.04	0.00	4.76	0.28	0.73	0.00	0.00	0.00	4.43	0.78	0.00	0.00	0.00	1.11	1.81	0.00	1.07	0.79	1.27	0.83	>0.999999999999999	1.5
P08118	Beta-microseminoprotein OS=Homo sapiens GN=MSMB PE=1 SV=1	0.00	0.00	0.00	0.00	4.79	3.64	4.22	0.00	0.00	0.00	0.00	2.49	2.19	0.00	0.00	0.00	0.00	0.00	3.96	0.00	0.77	0.00	0.00	1.42	1.26	0.70	>0.999999999999999	1.8
P55210	Caspase-7 OS=Homo sapiens GN=CASP7 PE=1 SV=1	0.00	4.61	0.00	0.00	0.00	6.40	0.00	0.00	1.23	0.00	1.89	1.00	0.00	4.03	0.00	0.69	0.00	0.00	0.00	4.02	0.31	0.00	0.00	0.00	1.26	0.75	>0.999999999999999	1.7
Q9UGI8	Testin OS=Homo sapiens GN=TES PE=1 SV=1	5.13	1.61	0.78	1.06	0.00	1.02	1.18	0.00	0.28	0.00	2.65	1.39	0.61	1.41	0.78	0.48	0.00	0.00	0.00	0.91	0.22	0.00	0.00	0.00	1.26	0.37	>0.999999999999999	3.4
Q14210	Lymphocyte antigen 6D OS=Homo sapiens GN=LY6D PE=1 SV=1	0.00	0.00	2.80	0.00	0.00	3.64	0.00	3.96	2.18	0.00	0.00	2.49	0.00	5.04	2.78	0.00	0.00	0.00	3.96	3.24	0.00	0.00	0.00	1.42	1.26	1.37	>0.999999999999999	0.9
Q12805	EGF-containing fibulin-like extracellular matrix protein 1 OS=Homo sapiens GN=EFEMP1 PE	1.47	4.15	0.67	3.79	2.30	0.87	0.00	0.95	0.24	0.00	0.00	0.60	0.53	3.63	0.67	0.83	1.13	0.92	0.95	0.78	0.18	0.00	1.84	0.00	1.25	0.95	>0.999999999999999	1.3
P07476	Involucrin OS=Homo sapiens GN=IVL PE=1 SV=2	0.00	1.15	2.35	0.00	0.00	0.00	0.84	6.18	0.20	3.34	0.95	0.00	0.44	3.02	0.00	2.21	0.00	1.53	0.79	1.30	0.31	1.04	0.77	0.28	1.25	0.97	>0.999999999999999	1.3
P56192	Methionine--tRNA ligase, cytoplasmic OS=Homo sapiens GN=MARS PE=1 SV=2	2.44	3.92	0.78	0.45	0.00	4.22	0.00	0.48	0.12	0.00	1.89	0.70	0.00	0.60	0.33	0.00	0.00	0.46	0.00	2.20	0.09	2.08	0.46	0.57	1.25	0.57	>0.999999999999999	2.2
P30419	Glycylpeptide N-tetradecanoyltransferase 1 OS=Homo sapiens GN=NMT1 PE=1 SV=2	2.93	2.77	0.67	0.91	1.15	1.75	1.01	0.95	0.48	0.00	1.14	1.20	0.53	1.21	0.67	0.41	0.00	0.92	1.90	0.78	0.18	1.25	0.92	0.34	1.25	0.76	>0.999999999999999	1.6
Q96DG6	Carboxymethylenebutenolidase homolog OS=Homo sapiens GN=CMBL PE=1 SV=1	9.53	0.00	1.34	0.00	2.30	1.75	0.00	0.00	0.00	0.00	0.00	0.00	0.00	0.00	0.00	0.00	0.00	0.00	0.00	0.00	0.00	0.00	0.00	0.00	1.24	0.00	>0.999999999999999	#DIV/0!
P61758	Prefoldin subunit 3 OS=Homo sapiens GN=VBP1 PE=1 SV=3	0.00	0.00	0.00	0.00	2.88	4.51	0.00	0.00	0.00	1.57	2.84	3.09	0.00	0.00	0.00	0.00	0.00	0.00	0.00	0.00	0.46	3.12	0.00	0.00	1.24	0.30	>0.999999999999999	4.2
O95757	Heat shock 70 kDa protein 4L OS=Homo sapiens GN=HSPA4L PE=1 SV=3	2.69	3.23	1.23	0.00	0.00	2.04	0.00	0.00	0.44	0.00	4.17	1.10	0.00	0.00	0.33	0.00	0.00	0.00	0.00	0.00	0.34	0.00	0.00	0.00	1.24	0.06	>0.999999999999999	22.1
Q9UBG3	Cornulin OS=Homo sapiens GN=CRNN PE=1 SV=1	0.00	3.00	0.67	0.00	0.00	0.00	5.90	2.06	0.00	2.09	1.14	0.00	0.00	2.62	0.00	1.38	0.00	0.00	2.06	0.78	0.00	0.00	0.00	0.34	1.24	0.60	>0.999999999999999	2.1
P43353	Aldehyde dehydrogenase family 3 member B1 OS=Homo sapiens GN=ALDH3B1 PE=1 SV=	6.84	0.00	0.00	0.00	0.00	2.91	0.00	4.44	0.00	0.63	0.00	0.00	0.00	0.00	0.00	0.00	0.00	0.00	0.00	0.00	0.00	0.00	0.00	0.00	1.23	0.00	>0.999999999999999	#DIV/0!
P60228	Eukaryotic translation initiation factor 3 subunit E OS=Homo sapiens GN=EIF3E PE=1 SV=1	1.47	1.38	3.03	0.00	0.00	3.93	0.00	0.00	0.52	0.00	2.46	1.99	0.00	2.62	0.67	1.38	0.00	1.99	0.00	1.68	0.40	0.00	0.00	0.74	1.23	0.79	>0.999999999999999	1.6
P06899	Histone H2B type 1-J OS=Homo sapiens GN=HIST1H2BJ PE=1 SV=3	0.00	0.00	0.00	0.00	0.00	0.00	0.00	0.00	0.00	14.72	0.00	0.00	0.00	0.00	0.00	0.00	0.00	0.00	22.35	0.00	0.00	11.43	0.00	0.00	1.23	2.82	>0.999999999999999	0.4
P53041	Serine/threonine-protein phosphatase 5 OS=Homo sapiens GN=PPP5C PE=1 SV=1	2.93	2.77	0.67	0.00	0.00	2.62	0.00	0.95	0.00	0.00	2.27	2.49	0.00	1.21	0.00	0.41	0.00	0.00	0.00	0.78	0.18	1.25	0.92	0.68	1.23	0.45	>0.999999999999999	2.7
O75822	Eukaryotic translation initiation factor 3 subunit J OS=Homo sapiens GN=EIF3J PE=1 SV=2	5.86	5.53	1.23	0.00	0.00	0.00	0.00	0.00	0.95	0.00	0.00	1.10	0.00	2.22	0.00	0.76	0.00	0.00	0.00	1.43	0.00	0.00	0.00	0.62	1.22	0.42	>0.999999999999999	2.9
P18077	60S ribosomal protein L35a OS=Homo sapiens GN=RPL35A PE=1 SV=2	6.59	0.00	3.03	0.00	0.00	3.93	0.00	0.00	1.07	0.00	0.00	0.00	0.00	0.00	0.00	0.00	0.00	4.14	0.00	8.03	0.83	0.00	0.00	0.00	1.22	1.08	>0.999999999999999	1.1
P09238	Stromelysin-2 OS=Homo sapiens GN=MMP10 PE=1 SV=1	0.00	0.00	0.00	0.00	0.00	6.11	0.00	4.28	0.00	0.00	0.00	4.18	0.00	0.00	0.00	0.00	0.00	0.00	0.00	0.00	0.00	0.00	0.00	1.08	1.21	0.09	>0.999999999999999	13.5
Q15404	Ras suppressor protein 1 OS=Homo sapiens GN=RSU1 PE=1 SV=3	2.69	2.54	1.23	0.00	2.11	1.60	1.86	0.00	0.44	0.00	2.08	0.00	0.96	2.22	0.00	0.00	0.00	0.00	0.00	1.43	0.00	2.29	1.69	0.00	1.21	0.71	>0.999999999999999	1.7
O00233	26S proteasome non-ATPase regulatory subunit 9 OS=Homo sapiens GN=PSMD9 PE=1 S\	3.18	0.00	5.15	0.00	0.00	1.89	0.00	0.00	0.52	0.00	2.46	1.29	0.00	2.62	1.44	0.00	0.00	0.00	0.00	3.76	0.40	0.00	0.00	0.74	1.21	0.75	>0.999999999999999	1.6
Q9BQ61	Uncharacterized protein C19orf43 OS=Homo sapiens GN=C19orf43 PE=1 SV=1	4.40	4.15	0.00	0.00	0.00	0.00	0.00	0.00	0.71	0.00	3.41	1.79	0.00	0.00	0.00	0.00	0.00	0.00	0.00	0.00	0.00	0.00	0.00	1.02	1.21	0.09	>0.999999999999999	14.1
Q96IJ6	Mannose-1-phosphate guanyltransferase alpha OS=Homo sapiens GN=GMPPA PE=1 SV=	3.66	5.30	0.00	0.00	0.00	4.51	0.00	0.00	0.28	0.00	0.00	0.70	0.00	0.00	0.78	0.00	0.00	0.00	0.00	0.91	0.00	1.45	0.00	0.00	1.20	0.26	>0.999999999999999	4.6
P25713	Metallothionein-3 OS=Homo sapiens GN=MT3 PE=1 SV=1	0.00	0.00	0.00	0.00	0.00	6.26	0.00	0.00	0.00	0.00	8.14	0.00	0.00	0.00	0.00	0.00	0.00	0.00	0.00	0.00	0.00	0.00	0.00	2.44	1.20	0.20	>0.999999999999999	5.9
P00167	Cytochrome b5 OS=Homo sapiens GN=CYB5A PE=1 SV=2	0.00	0.00	2.47	0.00	0.00	0.00	0.00	7.77	0.00	0.00	4.17	0.00	0.00	0.00	0.00	0.00	0.00	0.00	0.00	0.00	0.00	0.00	0.00	0.00	1.20	0.00	>0.999999999999999	#DIV/0!
O75594	Peptidoglycan recognition protein 1 OS=Homo sapiens GN=PGLYRP1 PE=1 SV=1	0.00	7.61	0.00	0.00	0.00	0.00	0.00	2.38	0.00	1.57	2.84	0.00	2.89	3.02	0.00	0.00	0.00	0.00	0.00	0.00	0.00	15.79	8.13	0.00	1.20	2.49	>0.999999999999999	0.5
P04114	Apolipoprotein B-100 OS=Homo sapiens GN=APOB PE=1 SV=2	0.24	1.38	0.34	1.21	6.14	0.00	0.67	0.00	0.44	2.19	1.14	0.60	1.49	2.62	3.00	0.41	1.51	0.15	6.82	0.13	0.59	5.19	1.38	0.34	1.20	1.97	>0.999999999999999	0.6
Q16698	2,4-dienoyl-CoA reductase, mitochondrial OS=Homo sapiens GN=DECR1 PE=1 SV=1	2.20	2.07	1.01	0.00	0.00	1.31	0.00	4.76	0.36	0.94	1.70	0.00	0.79	1.81	1.00	0.00	0.00	1.38	1.43	1.17	0.00	0.00	0.00	0.00	1.20	0.63	>0.999999999999999	1.9
Q9H977	WD repeat-containing protein 54 OS=Homo sapiens GN=WDR54 PE=1 SV=1	4.64	0.00	0.00	0.00	1.73	4.37	0.00	0.00	0.00	0.00	3.60	0.00	0.00	0.00	0.00	0.00	0.00	0.00	0.00	0.00	0.00	0.00	0.00	0.51	1.19	0.04	>0.999999999999999	28.0
Q6S8J3	POTE ankyrin domain family member E OS=Homo sapiens GN=POTEE PE=1 SV=3	0.00	0.00	3.03	0.00	0.00	4.37	0.00	3.17	1.07	0.00	0.00	2.69	0.00	0.00	0.00	1.59	0.00	0.00	0.00	0.00	0.00	0.00	0.00	0.00	1.19	0.13	>0.999999999999999	9.0
P19013	Keratin, type II cytoskeletal 4 OS=Homo sapiens GN=KRT4 PE=1 SV=4	0.00	2.77	0.00	0.00	0.00	1.75	1.01	0.00	0.00	8.77	0.00	0.00	0.00	1.21	1.33	0.00	0.00	0.00	6.34	0.00	0.00	0.00	0.00	0.34	1.19	0.77	>0.999999999999999	1.5

Q71UM5	40S ribosomal protein S27-like OS=Homo sapiens GN=RPS27L PE=1 SV=3	0.00	0.00	4.03	0.00	0.00	5.24	0.00	0.00	1.43	0.00	0.00	3.59	0.00	7.25	4.00	0.00	0.00	5.52	0.00	4.66	1.11	0.00	0.00	2.04	1.19	2.05	>0.999999999999999	0.6
O15355	Protein phosphatase 1G OS=Homo sapiens GN=PPM1G PE=1 SV=1	4.15	1.15	1.90	0.00	0.96	0.73	1.86	0.79	0.67	0.00	0.95	1.10	0.00	1.01	0.00	0.34	0.00	0.00	0.00	0.65	0.15	1.04	0.00	0.28	1.19	0.29	>0.999999999999999	4.1
Q7Z5P4	17-beta-hydroxysteroid dehydrogenase 13 OS=Homo sapiens GN=HSD17B13 PE=2 SV=1	4.88	0.00	0.00	0.00	6.14	0.00	0.00	3.17	0.00	0.00	0.00	0.00	0.00	0.00	0.00	0.00	0.00	0.00	0.00	0.00	0.00	0.00	0.00	0.00	1.18	0.00	>0.999999999999999	#DIV/0!
Q6NXT2	Histone H3.3C OS=Homo sapiens GN=H3F3C PE=1 SV=3	0.00	0.00	2.58	0.00	0.00	3.35	3.88	0.00	0.00	0.00	4.35	0.00	0.00	4.63	2.56	1.59	0.00	0.00	0.00	0.00	0.00	0.00	3.53	0.00	1.18	1.03	>0.999999999999999	1.2
O43813	LanC-like protein 1 OS=Homo sapiens GN=LANCL1 PE=1 SV=1	3.66	0.00	1.68	0.00	2.88	3.35	0.00	0.00	0.28	0.00	0.00	2.29	0.00	0.00	1.67	1.03	0.00	0.00	0.00	1.94	0.00	1.45	1.07	0.40	1.18	0.63	>0.999999999999999	1.9
P30711	Glutathione S-transferase theta-1 OS=Homo sapiens GN=GSTT1 PE=1 SV=4	2.93	0.00	0.00	1.82	4.99	0.00	4.39	0.00	0.00	0.00	0.00	0.00	1.05	0.00	0.00	0.00	2.26	0.00	0.00	1.55	0.00	0.00	0.00	0.00	1.18	0.41	>0.999999999999999	2.9
P20290	Transcription factor BTF3 OS=Homo sapiens GN=BTF3 PE=1 SV=1	3.66	3.46	1.68	0.00	0.00	2.18	2.53	0.00	0.59	0.00	0.00	0.00	0.00	3.02	0.00	0.00	0.00	0.00	0.00	0.00	0.00	0.00	2.30	0.00	1.18	0.44	>0.999999999999999	2.7
P06576	ATP synthase subunit beta, mitochondrial OS=Homo sapiens GN=ATP5B PE=1 SV=3	1.47	0.00	0.67	0.00	0.00	0.87	3.21	3.96	0.48	3.44	0.00	0.00	0.53	2.42	0.67	0.00	0.00	0.00	0.95	0.00	0.00	0.00	0.00	0.00	1.17	0.38	>0.999999999999999	3.1
P62633	Cellular nucleic acid-binding protein OS=Homo sapiens GN=CNBP PE=1 SV=1	3.91	0.00	1.79	0.00	3.07	2.33	0.00	0.00	1.39	0.00	0.00	1.59	1.40	3.22	0.00	1.10	0.00	0.00	2.54	2.07	0.49	3.32	0.00	1.99	1.17	1.35	>0.999999999999999	0.9
Q9BY44	Eukaryotic translation initiation factor 2A OS=Homo sapiens GN=EIF2A PE=1 SV=3	2.44	0.00	1.12	1.52	0.96	4.95	0.00	0.00	0.20	0.00	1.89	1.00	0.88	2.01	0.00	0.34	0.00	0.77	0.00	2.07	0.15	0.00	0.00	0.57	1.17	0.57	>0.999999999999999	2.1
Q8NBF2	NHL repeat-containing protein 2 OS=Homo sapiens GN=NHLRC2 PE=1 SV=1	1.95	1.84	1.46	1.21	0.77	1.89	1.35	0.00	0.32	0.00	2.46	0.80	0.35	1.61	0.44	0.90	0.00	0.61	0.00	1.04	0.25	1.66	0.61	0.23	1.17	0.64	>0.999999999999999	1.8
P21266	Glutathione S-transferase Mu 3 OS=Homo sapiens GN=GSTM3 PE=1 SV=3	2.93	0.00	2.91	0.00	0.00	1.75	0.00	0.00	0.00	0.00	2.27	4.18	0.00	0.00	0.00	0.00	0.00	0.00	0.00	0.00	0.00	0.00	0.00	0.68	1.17	0.06	>0.999999999999999	20.6
Q13151	Heterogeneous nuclear ribonucleoprotein A0 OS=Homo sapiens GN=HNRNPA0 PE=1 SV=1	2.69	0.00	0.00	0.00	0.00	5.24	0.00	0.00	1.74	0.00	2.08	2.29	0.00	2.22	0.00	0.00	0.00	0.00	0.00	0.00	0.00	0.00	0.00	0.00	1.17	0.18	>0.999999999999999	6.3
P61201	COP9 signalosome complex subunit 2 OS=Homo sapiens GN=COPS2 PE=1 SV=1	3.18	3.00	1.46	0.00	1.15	1.89	0.00	0.00	0.24	0.00	1.14	1.99	0.53	0.00	0.00	0.00	0.00	0.00	0.00	0.78	0.00	0.00	0.00	0.74	1.17	0.17	>0.999999999999999	6.9
Q969H8	UPF0556 protein C19orf10 OS=Homo sapiens GN=C19orf10 PE=1 SV=1	4.40	4.15	0.00	0.00	0.00	2.62	0.00	2.85	0.00	0.00	0.00	0.00	0.00	3.63	0.00	0.00	0.00	0.00	0.00	2.33	0.00	0.00	0.00	1.02	1.17	0.58	>0.999999999999999	2.0
P49321	Nuclear autoantigenic sperm protein OS=Homo sapiens GN=NASP PE=1 SV=2	3.91	1.84	0.90	0.00	0.77	1.75	1.35	0.63	0.16	0.00	1.51	1.20	0.35	1.61	0.44	0.28	0.00	0.61	0.00	1.55	0.12	0.83	0.00	0.45	1.17	0.52	>0.999999999999999	2.2
O75608	Acyl-protein thioesterase 1 OS=Homo sapiens GN=LYPLA1 PE=1 SV=1	3.18	3.00	1.46	1.97	2.49	0.00	0.00	0.00	0.52	0.00	0.00	1.29	0.00	0.00	0.00	0.90	0.00	0.00	0.00	0.00	0.40	0.00	0.00	0.74	1.16	0.17	>0.999999999999999	6.8
P09758	Tumor-associated calcium signal transducer 2 OS=Homo sapiens GN=TACSTD2 PE=1 SV=	2.20	0.00	1.01	1.36	1.73	1.31	0.00	3.01	0.36	1.98	0.00	0.90	0.00	1.81	1.00	1.31	0.00	0.00	1.43	2.46	0.28	0.00	0.00	0.00	1.15	0.69	>0.999999999999999	1.7
O94979	Protein transport protein Sec31A OS=Homo sapiens GN=SEC31A PE=1 SV=3	3.18	1.84	1.12	0.76	0.00	2.62	0.84	0.79	0.71	0.00	0.95	1.00	0.44	2.01	0.56	0.90	0.38	0.77	0.00	1.68	0.31	0.42	0.31	0.57	1.15	0.69	>0.999999999999999	1.7
Q9H4G0	Band 4.1-like protein 1 OS=Homo sapiens GN=EPB41L1 PE=1 SV=2	2.44	1.61	1.12	0.00	1.34	1.46	0.00	0.48	0.56	0.00	3.41	1.39	0.00	1.41	0.78	0.21	0.00	0.46	0.48	1.30	0.00	0.00	0.00	0.57	1.15	0.43	>0.999999999999999	2.7
Q7Z5L0	Vitelline membrane outer layer protein 1 homolog OS=Homo sapiens GN=VMO1 PE=1 SV=1	3.66	0.00	1.68	0.00	0.00	4.80	0.00	0.00	0.59	1.57	0.00	1.49	1.32	0.00	1.67	0.00	0.00	2.30	0.00	4.28	0.00	0.00	0.00	1.87	1.15	0.95	>0.999999999999999	1.2
P09936	Ubiquitin carboxyl-terminal hydrolase isozyme L1 OS=Homo sapiens GN=UCHL1 PE=1 SV=	3.18	0.00	1.46	1.97	2.49	1.89	0.00	0.00	0.00	0.00	0.00	2.79	0.00	0.00	0.00	0.00	0.00	0.00	0.00	0.00	0.00	0.00	0.00	0.00	1.15	0.00	>0.999999999999999	#DIV/0!
P84098	60S ribosomal protein L19 OS=Homo sapiens GN=RPL19 PE=1 SV=1	3.42	0.00	0.00	2.12	0.00	2.04	0.00	0.00	0.56	0.00	2.65	2.99	0.00	2.82	1.56	0.00	0.00	2.15	4.76	1.81	0.00	0.00	0.00	0.79	1.15	1.16	>0.999999999999999	1.0
Q13618	Cullin-3 OS=Homo sapiens GN=CUL3 PE=1 SV=2	0.98	1.61	1.23	1.06	0.00	2.91	0.67	0.63	0.28	0.42	2.84	1.10	0.35	1.41	0.78	0.28	0.00	0.00	0.00	0.91	0.00	0.83	0.00	0.40	1.14	0.41	>0.999999999999999	2.8
Q14152	Eukaryotic translation initiation factor 3 subunit A OS=Homo sapiens GN=EIF3A PE=1 SV=1	2.93	2.30	1.34	0.91	0.00	2.77	0.00	0.63	0.48	0.00	0.76	1.49	0.18	0.40	0.22	0.14	0.00	0.00	0.32	0.78	0.25	0.00	0.00	0.11	1.13	0.20	>0.999999999999999	5.7
P60903	Protein S100-A10 OS=Homo sapiens GN=S100A10 PE=1 SV=2	0.00	0.00	3.59	4.85	0.00	0.00	0.00	5.07	0.00	0.00	0.00	0.00	0.00	6.45	0.00	0.00	0.00	0.00	0.00	0.00	0.00	0.00	0.00	0.00	1.13	0.54	>0.999999999999999	2.1
Q9UK76	Hematological and neurological expressed 1 protein OS=Homo sapiens GN=HN1 PE=1 SV=	5.13	0.00	2.35	0.00	0.00	3.06	0.00	0.00	0.83	0.00	0.00	2.09	0.00	4.23	0.00	0.00	0.00	0.00	0.00	2.72	0.65	0.00	0.00	1.19	1.12	0.73	>0.999999999999999	1.5
Q9BVG4	UPF0368 protein Cxorf26 OS=Homo sapiens GN=CXorf26 PE=1 SV=1	3.18	0.00	1.46	1.97	0.00	1.89	2.19	0.00	0.00	0.00	0.00	2.69	0.00	0.00	1.44	0.00	0.00	0.00	0.00	1.68	0.00	2.70	0.00	0.74	1.11	0.55	>0.999999999999999	2.0
Q8WXC6	Myeloma-overexpressed gene 2 protein OS=Homo sapiens GN=MYEOV2 PE=2 SV=3	0.00	13.37	0.00	0.00	0.00	0.00	0.00	0.00	0.00	0.00	0.00	0.00	0.00	0.00	0.00	0.00	0.00	0.00	0.00	0.00	0.00	0.00	0.00	0.00	1.11	0.00	>0.999999999999999	#DIV/0!
P46108	Adapter molecule crk OS=Homo sapiens GN=CRK PE=1 SV=2	2.44	2.30	1.12	0.00	0.00	3.06	0.00	0.00	0.40	0.00	1.89	2.09	0.00	2.01	0.00	0.00	0.00	0.00	0.00	0.00	0.31	0.00	0.00	0.57	1.11	0.24	>0.999999999999999	4.6
P06132	Uroporphyrinogen decarboxylase OS=Homo sapiens GN=UROD PE=1 SV=2	4.15	1.84	0.90	0.00	1.53	0.00	1.35	0.00	0.32	0.00	1.51	1.69	0.00	1.61	0.00	0.00	0.00	0.00	0.00	1.04	0.25	0.00	0.00	0.45	1.11	0.28	>0.999999999999999	4.0
P40616	ADP-ribosylation factor-like protein 1 OS=Homo sapiens GN=ARL1 PE=1 SV=1	3.91	0.00	1.79	0.00	0.00	2.33	0.00	0.00	0.63	0.00	3.03	1.59	0.00	0.00	0.00	0.00	0.00	0.00	0.00	2.07	0.00	0.00	0.00	0.00	1.11	0.17	>0.999999999999999	6.4
P25705	ATP synthase subunit alpha, mitochondrial OS=Homo sapiens GN=ATP5A1 PE=1 SV=1	0.00	1.15	0.00	0.76	0.00	0.00	0.00	7.13	0.20	3.97	0.00	0.00	0.44	0.00	0.00	0.34	0.00	0.00	2.69	0.00	0.00	1.04	0.00	0.00	1.10	0.38	>0.999999999999999	2.9
P14317	Hematopoietic lineage cell-specific protein OS=Homo sapiens GN=HCLS1 PE=1 SV=3	3.18	1.38	0.67	0.91	2.49	0.87	1.01	0.00	0.24	0.00	1.14	1.29	0.00	0.00	0.67	0.00	0.00	0.00	0.00	0.78	0.00	1.25	2.91	0.74	1.10	0.53	>0.999999999999999	2.1
Q9Y5K8	V-type proton ATPase subunit D OS=Homo sapiens GN=ATP6V1D PE=1 SV=1	2.93	0.00	1.34	0.00	0.00	3.64	0.00	0.00	0.48	0.00	2.27	2.49	0.00	0.00	0.00	0.83	0.00	0.00	0.00	0.00	0.00	0.00	1.84	0.00	1.10	0.22	>0.999999999999999	4.9
P27169	Serum paraoxonase/arylesterase 1 OS=Homo sapiens GN=PON1 PE=1 SV=3	1.95	0.00	0.00	0.00	0.00	3.26	0.00	6.41	0.00	0.00	1.51	0.00	0.70	0.00	0.00	0.00	1.51	0.00	9.67	0.00	1.17	1.66	2.61	0.00	1.09	1.44	>0.999999999999999	0.8
P04899	Guanine nucleotide-binding protein G(i) subunit alpha-2 OS=Homo sapiens GN=GNAI2 PE=	4.15	0.00	0.00	0.00	0.00	0.00	0.00	1.27	0.00	6.16	1.51	0.00	0.70	0.00	0.00	0.00	0.00	0.00	0.00	1.27	0.00	5.40	0.00	0.00	1.09	0.61	>0.999999999999999	1.8
P03952	Plasma kallikrein OS=Homo sapiens GN=KLKB1 PE=1 SV=1	0.98	2.07	1.57	2.88	2.68	0.00	1.52	0.00	0.00	0.94	0.00	0.40	0.35	2.82	4.67	0.97	3.58	0.61	3.01	1.17	0.59	4.99	1.38	0.51	1.09	2.05	>0.999999999999999	0.5
P57737	Coronin-7 OS=Homo sapiens GN=CORO7 PE=1 SV=2	4.15	1.61	0.34	1.06	1.34	2.04	0.00	1.11	0.12	0.00	0.57	0.70	0.61	1.41	0.00	0.00	0.00	0.46	0.00	0.00	0.00	0.62	0.46	0.00	1.09	0.30	>0.999999999999999	3.7
O43708	Maleylacetoacetate isomerase OS=Homo sapiens GN=GSTZ1 PE=1 SV=3	0.00	3.23	1.57	2.12	0.00	2.04	0.00	0.00	0.00	0.00	2.65	1.39	0.00	5.84	0.00	0.97	0.00	0.00	0.00	3.76	0.43	2.91	0.00	0.00	1.08	1.16	>0.999999999999999	0.9
P54619	5'-AMP-activated protein kinase subunit gamma-1 OS=Homo sapiens GN=PRKAG1 PE=1 S	4.40	2.07	1.01	0.00	0.00	1.31	0.00	0.00	0.71	0.00	1.70	1.79	0.00	0.00	0.00	0.00	0.00	0.00	0.00	1.17	0.28	0.00	0.00	1.02	1.08	0.21	>0.999999999999999	5.3
Q00688	Peptidyl-prolyl cis-trans isomerase FKBP3 OS=Homo sapiens GN=FKBP3 PE=1 SV=1	3.18	3.00	1.46	0.00	0.00	4.08	0.00	0.00	0.00	0.00	0.00	1.29	0.00	2.62	1.44	0.90	0.00	1.99	0.00	3.63	0.00	0.00	0.00	0.00	1.08	0.88	>0.999999999999999	1.2
P50570	Dynamin-2 OS=Homo sapiens GN=DNM2 PE=1 SV=2	2.44	0.69	0.34	1.06	0.00	2.04	1.18	1.59	0.28	0.00	1.89	1.39	0.61	1.41	0.33	0.00	0.00	0.46	0.00	1.30	0.00	0.00	0.00	0.40	1.08	0.38	>0.999999999999999	2.9
P26368	Splicing factor U2AF 65 kDa subunit OS=Homo sapiens GN=U2AF2 PE=1 SV=4	1.47	3.00	1.46	0.91	0.00	2.77	0.00	0.00	0.24	0.00	2.46	0.60	0.00	2.62	0.00	0.41	0.00	0.00	0.00	0.78	0.00	0.00	0.00	0.00	1.07	0.32	>0.999999999999999	3.4
O75884	Putative hydrolase RBBP9 OS=Homo sapiens GN=RBBP9 PE=1 SV=2	3.91	0.00	0.00	0.00	0.00	4.95	0.00	0.00	0.63	0.00	0.00	3.39	0.00	0.00	0.00	0.00	0.00	0.00	0.00	2.07	0.00	0.00	0.00	1.93	1.07	0.33	>0.999999999999999	3.2
P04280	Basic salivary proline-rich protein 1 OS=Homo sapiens GN=PRB1 PE=1 SV=2	0.00	0.00	3.14	1.36	0.00	0.00	3.04	1.43	2.02	1.88	0.00	0.00	0.00	5.64	3.11	0.00	9.61	0.00	1.43	0.00	1.57	5.82	0.00	0.51	1.07	2.31	>0.999999999999999	0.5
Q9NR50	Translation initiation factor eIF-2B subunit gamma OS=Homo sapiens GN=EIF2B3 PE=1 SV	3.18	4.84	1.46	0.00	0.00	0.87	0.00	0.00	0.00	0.00	2.46	0.00	0.00	1.21	0.00	0.00	0.00	0.00	0.00	1.68	0.00	0.00	0.00	0.00	1.07	0.24	>0.999999999999999	4.4
Q9UNX3	60S ribosomal protein L26-like 1 OS=Homo sapiens GN=RPL26L1 PE=1 SV=1	0.00	0.00	0.00	0.00	0.00	6.26	3.37	3.17	0.00	0.00	0.00	0.00	1.75	4.03	0.00	1.38	0.00	0.00	3.17	2.59	1.32	4.16	0.00	1.14	1.07	1.63	>0.999999999999999	0.7
Q99613	Eukaryotic translation initiation factor 3 subunit C OS=Homo sapiens GN=EIF3C PE=1 SV=1	3.91	2.30	1.46	0.45	0.58	2.33	0.00	0.95	0.79	0.00	0.00	0.00	0.53	4.03	0.00	0.69	0.00	0.92	0.00	1.30	0.40	0.00	0.46	0.57	1.06	0.74	>0.999999999999999	1.4
Q9NR46	Endophilin-B2 OS=Homo sapiens GN=SH3GLB2 PE=1 SV=1	3.91	0.00	1.79	0.00	0.00	4.80	0.00	0.00	0.63	0.00	0.00	1.59	0.00	1.41	0.00	0.00	0.00	0.00	0.00	0.00	0.00	0.00	0.00	0.00	1.06	0.12	>0.999999999999999	9.0
Q9Y3B8	Oligoribonuclease, mitochondrial OS=Homo sapiens GN=REXO2 PE=1 SV=3	2.93	2.77	1.34	0.00	0.00	1.75	0.00	0.00	0.48	0.00	2.27	1.20	0.00	2.42	1.33	0.83	0.00	0.00	1.90	3.37	0.00	2.49	0.00	0.00	1.06	1.03	>0.999999999999999	1.0
Q9NZZ3	Charged multivesicular body protein 5 OS=Homo sapiens GN=CHMP5 PE=1 SV=1	3.42	3.23	0.00	0.00	0.00	2.04	0.00	0.00	0.00	0.00	2.65	1.39	0.00	0.00	0.00	0.00	0.00	0.00	0.00	1.81	0.00	0.00	0.00	0.00	1.06	0.15	>0.999999999999999	7.0
Q16629	Serine/arginine-rich splicing factor 7 OS=Homo sapiens GN=SRSF7 PE=1 SV=1	2.93	2.77	1.34	0.00	0.00	3.78	0.00	1.90	0.00	0.00	0.00	0.00	0.00	0.00	0.00	0.00	0.00	1.84	0.00	1.55	0.00	0.00	0.00	0.00	1.06	0.28	>0.999999999999999	3.7
Q8N8N7	Prostaglandin reductase 2 OS=Homo sapiens GN=PTGR2 PE=1 SV=1	1.95	1.84	0.90	0.00	0.00	1.16	1.35	0.00	0.32	0.00	3.41	1.79	0.00	0.00	0.89	0.00	0.00	0.00	0.00	0.00	0.00	0.00	0.00	0.00	1.06	0.07	>0.999999999999999	14.3
Q9HD89	Resistin OS=Homo sapiens GN=RETN PE=2 SV=1	0.00	0.00	3.14	4.25	0.00	0.00	0.00	0.00	0.00	0.00	5.30	0.00	2.46	0.00	3.11	0.00	5.28	0.00	0.00	0.00	0.00	13.51	0.00	0.00	1.06	2.03	>0.999999999999999	0.5
P35270	Sepiapterin reductase OS=Homo sapiens GN=SPR PE=1 SV=1	6.11	0.00	2.80	0.00	0.00	1.75	2.02	0.00	0.00	0.00	0.00	0.00	0.00	5.04	1.33	0.00	0.00	0.00	0.00	0.00	0.00	0.00	0.00	0.00	1.06	0.53	>0.999999999999999	2.0
Q9Y3D0	Mitotic spindle-associated MMXD complex subunit MIP18 OS=Homo sapiens GN=FAM96B	4.64	0.00	0.00	0.00	0.00	6.11	0.00	0.00	0.00	0.00	0.00	1.89	0.00	0.00	0.00	0.00	0.00	0.00	0.00	0.00	0.00	0.00	0.00	0.00	1.05	0.00	>0.999999999999999	#DIV/0!
Q92905	COP9 signalosome complex subunit 5 OS=Homo sapiens GN=COPS5 PE=1 SV=4	4.40	2.07	1.01	1.36	0.00	1.31	1.52	0.00	0.00	0.00	0.00	0.90	0.79	0.00	0.00	0.62	0.00	1.38	1.43	1.17	0.28	0.00	0.00	0.51	1.05	0.51	>0.999999999999999	2.0
P42025	Beta-centractin OS=Homo sapiens GN=ACTR1B PE=1 SV=1	3.91	0.00	0.00	0.00	0.00	3.64	0.00	0.00	0.00	0.00	1.51	3.49	0.00	3.22	0.00	0.00	0.00	0.00	0.00	0.00	0.00	1.66	0.00	0.00	1.05	0.41	>0.999999999999999	2.6
Q9BZZ5	Apoptosis inhibitor 5 OS=Homo sapiens GN=API5 PE=1 SV=3	0.00	2.54	1.23	0.00	0.00	1.60	1.01	0.95	0.00	0.00	3.41	1.79	0.53	1.21	0.00	0.00	0.00	0.00	0.00	0.78	0.00	0.00	0.00	0.62	1.04	0.26	>0.999999999999999	4.0
P53990	IST1 homolog OS=Homo sapiens GN=IST1 PE=1 SV=1	1.95	1.84	0.90	1.21	1.53	1.16	0.00	1.27	0.32	0.00	1.51	0.80	0.70	3.42	0.00	0.55	0.00	0.00	0.00	1.04	0.00	1.66	0.00	0.45	1.04	0.65	>0.999999999999999	1.6
Q96M27	Protein PRRC1 OS=Homo sapiens GN=PRRC1 PE=1 SV=1	3.66	1.61	1.68	0.00	0.00	1.02	0.00	1.11	0.59	0.00	1.33	1.49	0.00	3.02	1.67	0.48	0.00	0.00	1.11	1.94	0.00	0.00	0.00	0.40	1.04	0.72	>0.999999999999999	1.4
P06730	Eukaryotic translation initiation factor 4E OS=Homo sapiens GN=EIF4E PE=1 SV=2	3.18	3.00	0.00	1.97	0.00	1.89	0.00	0.00	0.00	0.00	2.46	0.00	0.00	5.64	0.00	0.00	0.00	0.00	0.00	1.68	0.00	0.00	0.00	0.74	1.04	0.67	>0.999999999999999	1.5
Q8TF09	Dynein light chain roadblock-type 2 OS=Homo sapiens GN=DYNLRB2 PE=1 SV=1	0.00	0.00	0.00	0.00	6.14	0.00	0.00	5.07	1.27	0.00	0.00	0.00	0.00	0.00	0.00	0.00	0.00	0.00	0.00	0.00	0.00	0.00	0.00	0.00	1.04	0.00	>0.999999999999999	#DIV/0!
P02745	Complement C1q subcomponent subunit A OS=Homo sapiens GN=C1QA PE=1 SV=2	0.00	3.00	1.46	1.97	2.49	0.00	2.19	0.00	0.00	1.36	0.00	0.00	0.00	2.62	1.44	0.90	2.45	0.00	2.06	0.00	0.00	0.00	0.00	0.00	1.04	0.79	>0.999999999999999	1.3
Q8WXF1	Paraspeckle component 1 OS=Homo sapiens GN=PSPC1 PE=1 SV=1	4.40	1.38	1.23	0.00	1.15	1.60	0.00	0.00	0.00	0.00	2.08	0.60	0.00	0.00	0.00	0.00	0.00	0.00	0.00	0.00	0.00	0.00	0.00	0.34	1.04	0.03	>0.999999999999999	36.5
P46778	60S ribosomal protein L21 OS=Homo sapiens GN=RPL21 PE=1 SV=2	4.40	0.00	2.02	0.00	0.00	2.62	0.00	0.00	0.00	0.00	3.41	0.00	0.00	0.00	0.00	0.00	0.00	0.00	0.00	0.00	0.00	0.00	0.00	0.00	1.04	0.00	>0.999999999999999	#DIV/0!
P02746	Complement C1q subcomponent subunit B OS=Homo sapiens GN=C1QB PE=1 SV=3	0.00	2.77	1.34	3.94	2.30	0.00	2.02	0.00	0.00	0.00	0.00	0.00	1.05	5.24	1.33	0.83	2.26	0.00	1.90	1.55	0.37	2.49	0.00	0.00	1.03	1.42	>0.999999999999999	0.7
P49441	Inositol polyphosphate 1-phosphatase OS=Homo sapiens GN=INPP1 PE=1 SV=1	1.71	5.53	0.00	0.00	0.00	3.49	0.00	0.00	0.28	0.00	1.33	0.00	0.00	0.00	0.00	0.00	0.00	1.07	0.00	0.00	0.00	0.00	0.00	0.40	1.03	0.12	>0.999999999999999	8.4
Q8TEX9	Importin-4 OS=Homo sapiens GN=IPO4 PE=1 SV=2	1.95	2.54	0.90	0.45	0.58	3.06	0.51	0.00	0.12	0.00	1.14	1.10	0.26	0.00	0.00	0.00	0.00	0.00	0.00	0.78	0.09	0.00	0.00	0.17	1.03	0.11	>0.999999999999999	9.5
Q13510	Acid ceramidase OS=Homo sapiens GN=ASAH1 PE=1 SV=5	0.00	3.46	1.68	0.00	0.00	3.49	0.00	2.38	0.59	0.00	0.00	0.70	0.61	3.02	0.78	1.03	1.32	3.68	0.00	1.94	0.22	1.45	0.00	0.00	1.03	1.17	>0.999999999999999	0.9
Q9NZJ9	Diphosphoinositol polyphosphate phosphohydrolase 2 OS=Homo sapiens GN=NUDT4 PE=	3.91	0.00	0.00	0.00	3.07	0.00	0.00	0.00	0.63	0.00	3.03	1.59	1.40	0.00	0.00	0.00	0.00	0.00	0.00	0.00	0.00	0.00	0.00	0.00	1.02	0.12	>0.999999999999999	8.7
P26373	60S ribosomal protein L13 OS=Homo sapiens GN=RPL13 PE=1 SV=4	0.00	3.23	1.57	0.00	0.00	6.84	0.00	0.00	0.56	0.00	0.00	0.00	0.00	0.00	0.00	0.00	0.00	0.00	2.22	1.81	0.00	0.00	0.00	0.00	1.02	0.34	>0.999999999999999	3.0
P31942	Heterogeneous nuclear ribonucleoprotein H3 OS=Homo sapiens GN=HNRNPH3 PE=1 SV=2	2.20	0.00	2.13	0.00	0.00	1.31	0.00	0.00	0.00	0.00	3.60	2.89	0.00	0.00	0.00	0.00	0.00	0.00	0.00	0.00	0.00	0.00	0.00	0.00	1.01	0.00	>0.999999999999999	#DIV/0!
Q14444	Caprin-1 OS=Homo sapiens GN=CAPRIN1 PE=1 SV=2	3.18	3.00	1.01	0.61	0.77	1.31	0.67	0.00	0.36	0.00	0.76	0.40	0.00	1.81	0.44	0.28	0.00	0.00	0.00	0.52	0.12	1.87	0.00	0.23	1.00	0.44	>0.999999999999999	2.3
Q96CV9	Optineurin OS=Homo sapiens GN=OPTN PE=1 SV=2	3.91	1.15	0.00	0.76	0.00	3.06	0.00	0.79	0.40	0.00	0.95	1.00	0.00	1.01	0.00	0.34	0.00	0.77	0.00	1.30	0.15	0.00	0.00	0.28	1.00	0.32	>0.999999999999999	3.1
Q6UWP8	Suprabasin OS=Homo sapiens GN=SBSN PE=2 SV=1	0.00	0.00	1.46	0.00	0.00	0.00	4.72	4.44	0.00	1.36	0.00	0.00	0.00	2.62	0.00	0.00	0.00	0.00	2.06	5.83	0.00	0.00	0.00	0.74	1.00	0.94	>0.999999999999999	1.1
Q7Z4W1	L-xylulose reductase OS=Homo sapiens GN=DCXR PE=1 SV=2	0.00	2.77	1.46	0.00	0.00	3.93	0.00	0.00	2.46	0.00	0.00	1.29	0.00	0.00	0.00	0.00	0.00	0.00	0.00	1.68	0.40	0.00	0.00	0.00	0.99	0.17	>0.999999999999999	5.7
A6NIZ1	Ras-related protein Rap-1b-like protein OS=Homo sapiens PE=2 SV=1	0.00	7.84	0.00	0.00	0.00	0.00	2.70	0.00	1.35	0.00	0.00	0.00	0.00	0.00	0.00	1.78	0.00	3.02	0.00	0.00	0.49	0.00	0.00	0.00	0.99	0.44	>0.999999999999999	2.2
Q9UEW8	STE20/SPS1-related proline-alanine-rich protein kinase OS=Homo sapiens GN=STK39 PE=	2.69	0.00	1.23	0.76	0.96	1.60	1.86	0.00	0.20	0.00	2.08	0.50	0.44	2.22	1.22	0.00	0.00	0.00	0.00	0.00	0.15	1.04	0.00	0.62	0.99	0.47	>0.999999999999999	2.1
P30040	Endoplasmic reticulum resident protein 29 OS=Homo sapiens GN=ERP29 PE=1 SV=4	0.00	0.00	2.69	0.00	0.00	3.49	1.86	3.80	0.00	0.00	0.00	0.00	0.00	2.22	1.22	0.00	0.00	0.00	0.00	3.11	0.00	2.29	1.69	0.00	0.99	0.88	>0.999999999999999	1.1
Q14C86	GTPase-activating protein and VPS9 domain-containing protein 1 OS=Homo sapiens GN=G	2.93	1.84	0.22	0.30	1.15	1.46	0.34	0.32	0.40	0.00	2.27	0.60	0.35	0.00	0.00	0.00	0.00	0.00	0.00	0.00	0.06	0.00	0.00	0.00	0.99	0.03	>0.999999999999999	28.7
Q9BVM4	Gamma-glutamylaminecyclotransferase OS=Homo sapiens GN=A2LD1 PE=1 SV=2	4.64	0.00	0.00	0.00	3.64	2.77	0.00	0.00	0.75	0.00	0.00	0.00	0.00	0.00	0.00	0.00	0.00	0.00	0.00	0.00	0.00	0.00	0.00	0.00	0.98	0.00	>0.999999999999999	#DIV/0!
P13726	Tissue factor OS=Homo sapiens GN=F3 PE=1 SV=1	5.13	0.00	0.00	0.00	0.00	1.46	0.00	3.33	0.83	1.04	0.00	0.00	0.00	0.00	0.00	0.00	0.00	1.53	1.59	1.30	0.00	2.08	1.53	0.00	0.98	0.67	>0.999999999999999	1.5
O00757	Fructose-1,6-bisphosphatase isozyme 2 OS=Homo sapiens GN=FBP2 PE=1 SV=2	2.20	0.00	1.01	1.36	0.00	1.31	1.52	1.43	0.36	0.00	1.70	0.90	0.00	1.81	1.00	0.62	1.70	1.38	0.00	1.17	0.28	1.87	1.38	0.51	0.98	0.98	>0.999999999999999	1.0
Q9H993	UPF0364 protein C6orf211 OS=Homo sapiens GN=C6orf211 PE=1 SV=1	4.88	3.00	0.67	0.00	0.00	0.87	1.01	0.00	0.00	0.00	0.00	1.29	0.53	1.21	0.00	0.41	0.00	0.00	0.00	0.78	0.18	0.00	0.00	0.00	0.98	0.26	>0.999999999999999	3.8
O43684	Mitotic checkpoint protein BUB3 OS=Homo sapiens GN=BUB3 PE=1 SV=1	4.40	2.07	0.00	1.36	0.00	2.62	0.00	0.00	0.36	0.00	0.00	0.90	0.00	0.00	0.00	0.00	0.00	0.00	0.00	1.17	0.00	0.00	0.00	0.00	0.98	0.10	>0.999999999999999	10.0
Q13177	Serine/threonine-protein kinase PAK 2 OS=Homo sapiens GN=PAK2 PE=1 SV=3	0.00	5.76	0.00	0.00	0.00	0.00	0.00	0.00	0.71	0.00	3.41	1.79	0.00	3.63	1.33	0.00	0.00	0.00	0.00	0.00	0.00	3.74	0.00	0.00	0.97	0.72	>0.999999999999999	1.3
Q12798	Centrin-1 OS=Homo sapiens GN=CETN1 PE=1 SV=1	4.15	3.92	1.90	0.00	0.00	0.00	0.00	0.00	0.00	0.00	0.00	1.69	0.00	0.00	0.00	0.00	0.00	0.00	0.00	0.00	0.00	0.00	0.00	0.00	0.97	0.00	>0.999999999999999	#DIV/0!
O60437	Periplakin OS=Homo sapiens GN=PPL PE=1 SV=4	1.95	1.84	0.90	0.30	0.00	1.89	0.84	0.48	0.20	1.98	0.95	0.30	0.26	1.01	1.11	1.03	0.00	0.46	0.48	1.30	0.00	0.62	0.46	0.57	0.97	0.61	>0.999999999999999	1.6
P41252	Isoleucine--tRNA ligase, cytoplasmic OS=Homo sapiens GN=IARS PE=1 SV=2	2.20	3.92	0.56	0.00	0.00	2.77	0.00	1.11	0.20	0.00	0.38	0.50	0.00	1.01	0.00	0.00	0.00	0.77	0.00	1.17	0.00	0.00	0.31	0.40	0.97	0.30	>0.999999999999999	3.2
P10809	60 kDa heat shock protein, mitochondrial OS=Homo sapiens GN=HSPD1 PE=1 SV=2	1.22	0.00	1.90	0.76	0.00	1.60	0.84	1.74	0.20	2.40	0.95	0.00	0.96	0.00	1.22	0.34	0.00	1.69	0.79	0.65	0.00	0.00	0.00	0.00	0.97	0.47	>0.999999999999999	2.1
O15347	High mobility group protein B3 OS=Homo sapiens GN=HMGB3 PE=1 SV=4	0.00	3.46	0.00	2.27	0.00	2.18	0.00	2.38	1.23	0.00	0.00	0.00	1.32	6.24	0.00	0.00	0.00	4.75	0.00	0.00	0.00	0.00	0.00	0.85	0.96	1.10	>0.999999999999999	0.9
P12830	Cadherin-1 OS=Homo sapiens GN=CDH1 PE=1 SV=3	1.71	1.61	1.12	0.45	0.58	1.46	1.18	1.11	0.28	0.00	1.33	0.70	0.26	0.60	0.78	0.21	0.57	0.46	1.11	0.91	0.09	0.00	0.46	0.17	0.96	0.47	>0.999999999999999	2.1
Q96CX2	BTB/POZ domain-containing protein KCTD12 OS=Homo sapiens GN=KCTD12 PE=1 SV=1	2.20	0.00	0.00	0.00	0.00	1.31	0.00	0.00	0.00	0.00	7.95	0.00	0.00	0.00	0.00	0.00	0.00	0.00	0.00	0.00	0.00	0.00	1.38	0.00	0.96	0.12	>0.999999999999999	8.3
Q5XUX1	F-box/WD repeat-containing protein 9 OS=Homo sapiens GN=FBXW9 PE=1 SV=2	2.93	1.38	0.67	0.00	0.00	2.77	0.00	0.00	0.24	0.00	2.27	1.20	0.53	0.00	0.00	0.00	0.00	0.00	0.00	0.00	0.00	0.00	0.00	0.00	0.95	0.04	>0.999999999999999	21.8
O43747	AP-1 complex subunit gamma-1 OS=Homo sapiens GN=AP1G1 PE=1 SV=5	2.69	0.92	1.68	0.00	0.77	2.18	0.00	0.00	0.44	0.00	2.08	0.70	0.35	0.81	0.44	0.28	0.00	0.61	0.00	0.52	0.00	0.83	0.61	0.62	0.95	0.42	>0.999999999999999	2.3
Q9NT62	Ubiquitin-like-conjugating enzyme ATG3 OS=Homo sapiens GN=ATG3 PE=1 SV=1	2.20	0.00	1.01	0.00	0.00	2.77	1.52	0.00	0.36	0.00	1.70	1.89	0.00	0.00	2.11	0.00	1.70	0.00	1.43	1.17	0.28	3.95	1.38	1.08	0.95	1.09	>0.999999999999999	0.9
Q5SRE7	Phytanoyl-CoA dioxygenase domain-containing protein 1 OS=Homo sapiens GN=PHYHD1 P	2.44	0.00	1.12	0.00	0.00	4.95	0.00	0.00	0.00	0.00	1.89	1.00	0.00	2.01	0.00	0.69	0.00	0.00	0.00	0.00	0.00	0.00	0.00	0.00	0.95	0.23	>0.999999999999999	4.2
Q92696	Geranylgeranyl transferase type-2 subunit alpha OS=Homo sapiens GN=RABGGTA PE=1 S	2.44	2.30	0.56	0.76	0.96	2.33	0.00	0.79	0.20	0.00	0.00	1.00	0.44	0.00	0.00	0.00	0.00	0.00	0.00	2.72	0.15	1.04	0.00	0.57	0.95	0.41	>0.999999999999999	2.3
O95777	N-alpha-acetyltransferase 38, NatC auxiliary subunit OS=Homo sapiens GN=NAA38 PE=1 S	0.00	0.00	0.00	0.00	0.00	11.21	0.00	0.00	0.00	0.00	0.00	0.00	0.00	0.00	0.00	0.00	0.00	0.00	0.00	0.00	0.00	0.00	0.00	0.00	0.93	0.00	>0.999999999999999	#DIV/0!
Q6UXB3	Ly6/PLAUR domain-containing protein 2 OS=Homo sapiens GN=LYPD2 PE=2 SV=1	0.00	0.00	0.00	0.00	0.00	3.64	0.00	3.96	0.99	2.61	0.00	0.00	2.19	0.00	0.00	1.72	0.00	3.83	3.96	3.24	0.00	0.00	3.83	0.00	0.93	1.57	>0.999999999999999	0.6
Q5EBL8	PDZ domain-containing protein 11 OS=Homo sapiens GN=PDZD11 PE=1 SV=2	5.13	0.00	0.00	0.00	0.00	0.00	0.00	0.00	0.00	0.00	3.98	2.09	0.00	0.00	0.00	0.00	0.00	0.00	0.00	0.00	0.00	0.00	0.00	0.00	0.93	0.00	>0.999999999999999	#DIV/0!
O60256	Phosphoribosyl pyrophosphate synthase-associated protein 2 OS=Homo sapiens GN=PRPS	0.00	1.84	0.90	0.00	0.00	5.24	0.00	0.00	0.00	0.00	1.51	1.69	0.00	0.00	0.00	0.00	0.00	0.00	0.00	0.00	0.00	1.66	0.00	0.00	0.93	0.14	>0.999999999999999	6.7
Q9H4G4	Golgi-associated plant pathogenesis-related protein 1 OS=Homo sapiens GN=GLIPR2 PE=1	0.00	0.00	0.00	0.00	0.00	2.91	0.00	0.00	0.00	4.49	3.79	0.00	0.00	0.00	0.00	0.00	0.00	0.00	0.00	0.00	0.00	0.00	0.00	0.00	0.93	0.00	>0.999999999999999	#DIV/0!
O95782	AP-2 complex subunit alpha-1 OS=Homo sapiens GN=AP2A1 PE=1 SV=3	1.47	0.69	0.34	0.00	1.15	3.93	0.00	0.00	0.00	0.00	2.46	0.90	0.00	0.00	0.00	1.00	0.00	0.00	0.00	0.00	0.18	0.00	0.00	0.34	0.93	0.13	>0.999999999999999	7.3
Q7L2H7	Eukaryotic translation initiation factor 3 subunit M OS=Homo sapiens GN=EIF3M PE=1 SV=1	1.95	1.84	0.90	0.00	0.00	3.64	1.35	0.00	0.63	0.00	0.00	0.80	0.70	1.61	0.00	0.55	0.00	1.23	1.27	1.04	0.00	0.00	0.00	0.91	0.93	0.61	>0.999999999999999	1.5
P46783	40S ribosomal protein S10 OS=Homo sapiens GN=RPS10 PE=1 SV=1	0.00	0.00	0.00	0.00	0.00	5.68	0.00	0.00	1.55	0.00	0.00	3.88	0.00	0.00	0.00	0.00	0.00	0.00	0.00	0.00	0.00	0.00	0.00	0.00	0.93	0.00	>0.999999999999999	#DIV/0!
Q96IU4	Abhydrolase domain-containing protein 14B OS=Homo sapiens GN=ABHD14B PE=1 SV=1	0.00	3.46	1.68	0.00	0.00	2.18	0.00	0.00	0.59	0.00	0.00	3.19	0.00	0.00	1.67	1.03	0.00	0.00	0.00	1.94	0.00	0.00	2.30	1.82	0.93	0.73	>0.999999999999999	1.3
P12004	Proliferating cell nuclear antigen OS=Homo sapiens GN=PCNA PE=1 SV=1	2.69	0.00	0.00	0.00	0.00	1.60	1.86	1.74	0.00	0.00	2.08	1.10	0.00	0.00	0.00	0.76	0.00	0.00	0.00	1.43	0.34	2.29	1.69	0.62	0.92	0.59	>0.999999999999999	1.6
Q9Y5S9	RNA-binding protein 8A OS=Homo sapiens GN=RBM8A PE=1 SV=1	0.00	0.00	4.15	0.00	0.00	0.00	0.00	0.00	0.00	0.00	3.22	3.69	0.00	0.00	0.00	0.00	0.00	0.00	0.00	0.00	0.52	3.53	2.61	0.00	0.92	0.56	>0.999999999999999	1.7
P37840	Alpha-synuclein OS=Homo sapiens GN=SNCA PE=1 SV=1	0.00	0.00	0.00	0.00	4.60	0.00	4.05	0.00	0.00	0.00	0.00	2.39	0.00	0.00	0.00	0.00	0.00	0.00	0.00	0.00	0.00	0.00	0.00	0.00	0.92	0.00	>0.999999999999999	#DIV/0!
Q9Y570	Protein phosphatase methylesterase 1 OS=Homo sapiens GN=PPME1 PE=1 SV=3	0.00	1.84	1.79	1.21	0.00	3.64	0.00	0.00	0.00	0.00	0.00	2.49	0.00	0.00	0.00	0.00	0.00	0.00	0.00	1.04	0.00	0.00	0.00	0.00	0.91	0.09	>0.999999999999999	10.6
Q9Y376	Calcium-binding protein 39 OS=Homo sapiens GN=CAB39 PE=1 SV=1	1.95	0.00	0.00	0.00	0.00	2.47	0.00	0.00	0.67	0.94	3.22	1.69	0.00	5.44	1.89	0.55	0.00	1.23	0.00	0.00	0.25	7.69	1.23	0.00	0.91	1.52	>0.999999999999999	0.6
Q6UX06	Olfactomedin-4 OS=Homo sapiens GN=OLFM4 PE=1 SV=1	0.00	5.76	0.00	0.00	0.00	0.00	1.01	0.00	0.00	4.17	0.00	0.00	0.53	1.21	0.00	0.00	0.00	0.00	0.00	0.00	0.00	3.74	2.76	0.00	0.91	0.69	>0.999999999999999	1.3
Q96AE4	Far upstream element-binding protein 1 OS=Homo sapiens GN=FUBP1 PE=1 SV=3	2.44	1.15	1.12	1.52	0.96	3.06	0.00	0.00	0.20	0.00	0.00	0.50	0.44	0.00	0.00	0.00	0.00	0.00	0.00	0.65	0.00	0.00	0.00	0.28	0.91	0.11	>0.999999999999999	8.0
Q9H0N5	Pterin-4-alpha-carbinolamine dehydratase 2 OS=Homo sapiens GN=PCBD2 PE=1 SV=4	5.86	0.00	2.69	0.00	0.00	0.00	0.00	0.00	0.00	0.00	0.00	2.39	0.00	4.83	0.00	0.00	0.00	0.00	0.00	0.00	0.00	0.00	0.00	0.00	0.91	0.40	>0.999999999999999	2.3
P01772	Ig heavy chain V-III region KOL OS=Homo sapiens PE=1 SV=1	0.00	0.00	0.00	3.64	0.00	3.49	0.00	3.80	0.00	0.00	0.00	0.00	0.00	4.83	0.00	0.00	4.52	3.68	3.80	3.11	0.00	4.99	0.00	0.00	0.91	2.08	>0.999999999999999	0.4
Q96E39	RNA binding motif protein, X-linked-like-1 OS=Homo sapiens GN=RBMXL1 PE=1 SV=1	1.95	0.00	1.79	1.21	0.00	0.00	1.35	0.00	0.00	0.00	3.03	1.59	0.00	0.00	0.89	0.00	1.51	0.00	0.00	0.00	0.25	1.66	2.45	0.00	0.91	0.56	>0.999999999999999	1.6
Q96FV2	Secernin-2 OS=Homo sapiens GN=SCRN2 PE=2 SV=3	0.00	3.46	0.00	0.00	1.34	2.18	0.00	1.11	0.00	0.00	1.33	1.49	0.00	1.41	0.00	0.48	0.00	0.00	0.00	1.94	0.00	0.00	0.00	0.00	0.91	0.32	>0.999999999999999	2.8
Q9UKM9	RNA-binding protein Raly OS=Homo sapiens GN=RALY PE=1 SV=1	2.44	0.00	1.12	1.52	0.00	1.46	0.00	0.00	0.40	0.00	3.98	0.00	0.00	2.01	0.00	0.00	0.00	0.00	0.00	1.30	0.00	0.00	0.00	0.57	0.91	0.32	>0.999999999999999	2.8
Q9BQA1	Methylosome protein 50 OS=Homo sapiens GN=WDR77 PE=1 SV=1	2.20	2.07	1.01	0.00	0.00	0.00	1.52	1.43	0.00	0.00	1.70	0.90	0.00	0.00	0.00	0.00	0.00	0.00	0.00	0.00	0.28	0.00	0.00	0.51	0.90	0.07	>0.999999999999999	13.7
P05091	Aldehyde dehydrogenase, mitochondrial OS=Homo sapiens GN=ALDH2 PE=1 SV=2	0.00	0.00	0.00	0.00	0.00	3.64	0.00	5.23	0.00	1.88	0.00	0.00	2.19	5.04	2.78	1.24	0.00	0.00	0.00	3.24	0.00	0.00	0.00	0.00	0.90	1.21	>0.999999999999999	0.7
P49788	Retinoic acid receptor responder protein 1 OS=Homo sapiens GN=RARRES1 PE=2 SV=2	0.00	2.30	1.12	1.52	0.00	4.80	0.00	0.00	0.00	0.00	0.00	1.00	1.84	6.65	1.11	0.69	3.96	0.00	0.00	2.72	0.00	2.08	0.00	1.87	0.90	1.74	>0.999999999999999	0.5
Q9UBK7	Rab-like protein 2A OS=Homo sapiens GN=RABL2A PE=2 SV=1	3.18	0.00	0.00	0.00	0.00	6.26	0.00	0.00	0.00	0.00	0.00	1.29	0.00	0.00	0.00	0.00	0.00	0.00	0.00	0.00	0.00	0.00	0.00	0.00	0.89	0.00	>0.999999999999999	#DIV/0!
P62877	E3 ubiquitin-protein ligase RBX1 OS=Homo sapiens GN=RBX1 PE=1 SV=1	0.00	0.00	3.03	0.00	0.00	3.93	0.00	0.00	1.07	0.00	0.00	2.69	0.00	5.44	0.00	0.00	0.00	4.14	0.00	3.50	0.00	0.00	0.00	0.00	0.89	1.09	>0.999999999999999	0.8
Q9P287	BRCA2 and CDKN1A-interacting protein OS=Homo sapiens GN=BCCIP PE=1 SV=1	7.33	2.07	0.00	0.00	0.00	1.31	0.00	0.00	0.00	0.00	0.00	0.00	0.79	0.00	0.00	0.00	0.00	0.00	0.00	1.17	0.00	0.00	0.00	0.00	0.89	0.16	>0.999999999999999	5.5
P38646	Stress-70 protein, mitochondrial OS=Homo sapiens GN=HSPA9 PE=1 SV=2	0.98	0.00	0.45	0.00	0.77	2.77	0.00	4.76	0.00	0.94	0.00	0.00	0.79	1.81	1.00	0.28	0.00	1.38	0.63	0.52	0.00	0.00	0.00	0.00	0.89	0.53	>0.999999999999999	1.7
P53365	Arfaptin-2 OS=Homo sapiens GN=ARFIP2 PE=1 SV=1	4.40	0.00	0.00	0.00	1.73	1.31	0.00	1.43	0.00	0.00	1.70	0.00	0.00	0.00	0.00	0.00	0.00	0.00	0.00	1.17	0.00	0.00	0.00	0.00	0.88	0.10	>0.999999999999999	9.1
Q16777	Histone H2A type 2-C OS=Homo sapiens GN=HIST2H2AC PE=1 SV=4	0.00	0.00	0.00	0.00	10.55	0.00	0.00	0.00	0.00	0.00	0.00	0.00	0.00	0.00	0.00	0.00	0.00	0.00	0.00	0.00	0.00	0.00	0.00	0.00	0.88	0.00	>0.999999999999999	#DIV/0!
Q5TDH0	Protein DDI1 homolog 2 OS=Homo sapiens GN=DDI2 PE=1 SV=1	1.71	0.00	0.00	0.00	0.00	2.18	0.00	1.11	0.28	0.00	4.54	0.70	0.00	1.41	0.00	0.48	0.00	0.00	0.00	1.94	0.00	0.00	0.00	0.00	0.88	0.32	>0.999999999999999	2.7
A6NJ16	Putative V-set and immunoglobulin domain-containing protein 6 OS=Homo sapiens GN=VSI	0.00	5.76	0.00	0.00	0.00	0.00	0.00	0.00	0.00	0.00	4.73	0.00	0.00	0.00	0.00	0.00	0.00	3.83	0.00	0.00	0.00	0.00	3.83	0.00	0.87	0.64	>0.999999999999999	1.4
Q6P6B1	Uncharacterized protein C8orf47 OS=Homo sapiens GN=C8orf47 PE=2 SV=1	4.15	0.00	0.90	0.00	0.00	3.93	0.00	0.00	0.00	0.00	1.51	0.00	0.00	0.00	0.00	0.00	0.00	0.00	0.00	0.00	0.00	0.00	0.00	0.00	0.87	0.00	>0.999999999999999	#DIV/0!
Q8NDH3	Probable aminopeptidase NPEPL1 OS=Homo sapiens GN=NPEPL1 PE=1 SV=3	2.93	1.38	0.67	0.00	1.15	0.87	0.00	0.00	0.00	0.00	2.27	1.20	0.00	1.21	0.00	0.00	0.00	0.00	0.00	0.78	0.00	0.00	0.00	0.00	0.87	0.17	>0.999999999999999	5.3
P10155	60 kDa SS-A/Ro ribonucleoprotein OS=Homo sapiens GN=TROVE2 PE=1 SV=2	2.69	0.00	1.23	0.00	0.00	3.35	0.00	0.00	0.00	0.00	2.08	1.10	0.00	3.42	0.00	0.76	0.00	0.00	0.00	0.65	0.00	0.00	0.77	0.62	0.87	0.52	>0.999999999999999	1.7
Q8WVM8	Sec1 family domain-containing protein 1 OS=Homo sapiens GN=SCFD1 PE=1 SV=4	2.20	3.23	1.01	0.00	0.96	2.04	0.00	0.79	0.20	0.00	0.00	0.00	0.44	1.81	0.00	0.62	0.00	0.77	0.00	0.00	0.15	0.00	0.00	0.00	0.87	0.32	>0.999999999999999	2.7
Q12765	Secernin-1 OS=Homo sapiens GN=SCRN1 PE=1 SV=2	1.71	1.61	2.58	0.00	1.34	2.18	0.00	0.00	0.28	0.00	0.00	0.70	0.00	4.63	0.78	1.03	0.00	1.07	0.00	0.91	0.00	0.00	0.00	0.85	0.87	0.77	>0.999999999999999	1.1
P13716	Delta-aminolevulinic acid dehydratase OS=Homo sapiens GN=ALAD PE=1 SV=1	0.00	0.00	2.13	0.00	0.00	4.37	0.00	1.43	0.75	0.00	1.70	0.00	0.00	1.81	1.00	0.62	0.00	1.38	0.00	1.17	0.28	1.87	0.00	0.51	0.86	0.72	>0.999999999999999	1.2
Q5TFE4	5'-nucleotidase domain-containing protein 1 OS=Homo sapiens GN=NT5DC1 PE=1 SV=1	1.47	1.38	0.00	0.91	1.15	0.87	1.18	0.95	0.00	0.00	1.14	1.29	0.00	4.03	0.00	0.41	0.00	0.00	0.00	0.00	0.00	0.00	0.00	0.34	0.86	0.40	>0.999999999999999	2.2
P22531	Small proline-rich protein 2E OS=Homo sapiens GN=SPRR2E PE=2 SV=2	0.00	0.00	0.00	0.00	0.00	0.00	0.00	0.00	0.00	10.33	0.00	0.00	0.00	0.00	0.00	0.00	0.00	0.00	0.00	0.00	0.00	0.00	0.00	0.00	0.86	0.00	>0.999999999999999	#DIV/0!
P54920	Alpha-soluble NSF attachment protein OS=Homo sapiens GN=NAPA PE=1 SV=3	2.44	0.00	2.35	1.52	0.00	0.00	0.00	0.00	0.83	1.04	0.00	2.09	0.00	0.00	0.00	0.69	0.00	0.00	0.00	2.72	0.31	2.08	0.00	0.00	0.86	0.48	>0.999999999999999	1.8
P26640	Valine--tRNA ligase OS=Homo sapiens GN=VARS PE=1 SV=4	2.44	0.46	0.56	0.30	0.96	1.75	0.00	0.32	0.40	0.00	1.89	1.20	0.18	1.41	0.22	0.14	0.00	0.77	0.00	0.26	0.15	0.42	0.00	0.28	0.86	0.32	>0.999999999999999	2.7
Q9P2J5	Leucine--tRNA ligase, cytoplasmic OS=Homo sapiens GN=LARS PE=1 SV=2	1.22	2.30	0.78	0.00	0.00	3.49	0.00	0.32	0.20	0.00	0.95	1.00	0.00	1.01	0.00	0.14	0.00	0.77	0.00	0.91	0.00	0.00	0.00	0.28	0.86	0.26	>0.999999999999999	3.3
Q9C010	cAMP-dependent protein kinase inhibitor beta OS=Homo sapiens GN=PKIB PE=2 SV=1	10.26	0.00	0.00	0.00	0.00	0.00	0.00	0.00	0.00	0.00	0.00	0.00	8.77	0.00	0.00	0.00	0.00	6.44	0.00	0.00	0.00	0.00	0.00	0.00	0.85	1.27	>0.999999999999999	0.7
Q9UBW8	COP9 signalosome complex subunit 7a OS=Homo sapiens GN=COPS7A PE=1 SV=1	5.62	2.54	0.00	0.00	0.00	2.08	0.00	0.00	0.00	0.00	2.08	0.00	0.00	0.00	0.00	0.00	0.00	0.00	0.00	1.43	0.00	0.00	0.00	0.00	0.85	0.12	>0.999999999999999	7.2
Q9NPF4	Probable tRNA threonylcarbamoyladenosine biosynthesis protein OSGEP OS=Homo sapien	0.00	2.07	1.01	1.36	0.00	1.31	1.52	0.00	0.36	0.00	1.70	0.90	0.00	1.81	0.00	0.62	0.00	0.00	0.00	1.17	0.00	0.00	0.00	0.51	0.85	0.34	>0.999999999999999	2.5
Q9H477	Ribokinase OS=Homo sapiens GN=RBKS PE=1 SV=1	0.00	4.61	0.00	0.00	0.00	1.46	0.00	3.17	0.00	0.00	0.00	1.00	0.00	0.00	0.00	0.00	0.00	0.00	0.00	1.30	0.00	0.00	0.00	0.00	0.85	0.11	>0.999999999999999	7.9
P53367	Arfaptin-1 OS=Homo sapiens GN=ARFIP1 PE=1 SV=2	0.00	1.84	0.00	0.00	0.00	0.00	0.00	1.27	0.63	0.83	3.03	2.59	0.00	1.61	0.00	0.00	0.00	0.00	0.00	0.00	0.00	0.00	0.00	0.00	0.85	0.13	>0.999999999999999	6.3
O60936	Nucleolar protein 3 OS=Homo sapiens GN=NOL3 PE=1 SV=1	3.42	0.00	0.00	0.00	2.68	0.00	0.00	0.00	0.00	0.00	2.65	1.39	1.23	2.82	1.56	0.97	0.00	0.00	0.00	3.76	0.00	0.00	0.00	0.00	0.85	0.86	>0.999999999999999	1.0
Q8WVJ2	NudC domain-containing protein 2 OS=Homo sapiens GN=NUDCD2 PE=1 SV=1	4.64	0.00	0.00	0.00	0.00	0.00	0.00	0.00	0.00	0.00	3.60	1.89	0.00	0.00	0.00	0.00	0.00	0.00	0.00	2.46	0.00	0.00	0.00	1.08	0.84	0.30	>0.999999999999999	2.9
P49902	Cytosolic purine 5'-nucleotidase OS=Homo sapiens GN=NT5C2 PE=1 SV=1	2.44	2.30	0.56	0.00	0.00	2.33	0.00	0.00	0.00	0.52	0.95	1.00	0.00	1.01	0.56	0.34	0.00	1.53	0.00	0.65	0.49	0.00	0.00	0.91	0.84	0.46	>0.999999999999999	1.8
P06311	Ig kappa chain V-III region IARC/BL41 OS=Homo sapiens PE=1 SV=1	0.00	0.00	6.05	0.00	0.00	0.00	4.05	0.00	0.00	0.00	0.00	0.00	2.11	0.00	6.00	1.66	4.52	0.00	0.00	3.11	0.00	4.99	3.68	0.00	0.84	2.17	>0.999999999999999	0.4
Q9HC84	Mucin-5B OS=Homo sapiens GN=MUC5B PE=1 SV=3	0.00	3.23	0.11	0.00	2.30	0.00	1.69	0.63	0.04	1.98	0.00	0.10	0.00	0.20	0.00	0.00	0.38	0.15	2.69	0.26	0.06	0.83	0.46	0.11	0.84	0.43	>0.999999999999999	2.0
P12724	Eosinophil cationic protein OS=Homo sapiens GN=RNASE3 PE=1 SV=2	0.00	4.15	0.00	0.00	0.00	0.00	3.04	2.85	0.00	0.00	0.00	0.00	0.00	0.00	0.00	0.00	0.00	0.00	2.85	0.00	0.00	0.00	2.76	0.00	0.84	0.47	>0.999999999999999	1.8
Q9HC35	Echinoderm microtubule-associated protein-like 4 OS=Homo sapiens GN=EML4 PE=1 SV=3	0.73	2.07	1.01	0.00	0.00	1.75	0.00	0.48	0.12	0.00	2.27	1.59	0.00	2.42	0.67	0.21	0.00	0.46	0.00	1.55	0.09	0.62	0.92	0.51	0.84	0.62	>0.999999999999999	1.3
Q969E4	Transcription elongation factor A protein-like 3 OS=Homo sapiens GN=TCEAL3 PE=1 SV=1	0.00	3.46	0.00	0.00	2.88	2.18	0.00	0.00	0.00	0.00	0.00	1.49	0.00	0.00	0.00	0.00	0.00	0.00	0.00	1.94	0.00	0.00	0.00	0.00	0.83	0.16	>0.999999999999999	5.2
Q9Y2S2	Lambda-crystallin homolog OS=Homo sapiens GN=CRYL1 PE=1 SV=3	4.64	0.00	0.00	0.00	0.00	2.77	0.00	0.00	0.00	0.00	1.70	0.90	0.00	0.00	0.00	0.00	0.00	0.00	0.00	0.00	0.00	0.00	0.00	0.00	0.83	0.00	>0.999999999999999	#DIV/0!
Q96QR8	Transcriptional activator protein Pur-beta OS=Homo sapiens GN=PURB PE=1 SV=3	2.44	2.30	2.35	0.00	0.00	0.00	0.00	0.00	0.00	0.00	1.89	1.00	0.00	0.00	0.00	0.69	0.00	1.53	0.00	0.00	0.00	0.00	0.00	0.57	0.83	0.23	>0.999999999999999	3.6
P36507	Dual specificity mitogen-activated protein kinase kinase 2 OS=Homo sapiens GN=MAP2K2	5.86	1.61	0.00	0.00	0.00	1.02	0.00	0.00	0.00	0.00	0.00	1.49	0.00	3.02	0.00	0.00	0.00	0.00	0.00	0.00	0.00	0.00	0.00	0.00	0.83	0.25	>0.999999999999999	3.3
P62917	60S ribosomal protein L8 OS=Homo sapiens GN=RPL8 PE=1 SV=2	0.00	0.00	1.34	0.00	0.00	8.15	0.00	0.00	0.48	0.00	0.00	0.00	0.00	0.00	0.00	0.00	0.00	3.83	0.00	3.24	0.37	0.00	0.00	0.00	0.83	0.62	>0.999999999999999	1.3
Q14134	Tripartite motif-containing protein 29 OS=Homo sapiens GN=TRIM29 PE=1 SV=2	2.44	0.00	1.79	0.76	0.00	0.73	0.00	1.59	0.20	0.52	0.95	1.00	0.00	4.23	1.11	0.69	0.00	1.53	0.00	2.07	0.15	0.00	0.00	0.00	0.83	0.82	>0.999999999999999	1.0
P62266	40S ribosomal protein S23 OS=Homo sapiens GN=RPS23 PE=1 SV=3	0.00	0.00	0.00	0.00	0.00	3.06	0.00	0.00	0.83	0.00	3.98	2.09	0.00	0.00	0.00	0.00	0.00	0.00	0.00	0.00	0.00	0.00	0.00	0.00	0.83	0.00	>0.999999999999999	#DIV/0!
Q9NSK0	Kinesin light chain 4 OS=Homo sapiens GN=KLC4 PE=1 SV=3	2.44	0.00	1.68	0.00	0.00	2.91	0.00	0.00	0.00	0.00	1.89	1.00	0.00	0.00	0.00	0.00	0.00	0.00	0.00	0.00	0.00	0.00	0.00	0.28	0.83	0.02	>0.999999999999999	35.0
P51668	Ubiquitin-conjugating enzyme E2 D1 OS=Homo sapiens GN=UBE2D1 PE=1 SV=1	0.00	0.00	0.00	0.00	0.00	0.00	0.00	0.00	1.74	0.00	3.79	4.38	0.00	0.00	0.00	0.00	0.00	0.00	0.00	0.00	0.00	0.00	0.00	0.00	0.83	0.00	>0.999999999999999	#DIV/0!
P47895	Aldehyde dehydrogenase family 1 member A3 OS=Homo sapiens GN=ALDH1A3 PE=1 SV=	0.00	2.77	2.02	0.00	0.00	2.62	0.00	0.00	0.71	0.00	0.00	1.79	0.00	3.63	0.00	1.72	0.00	2.76	2.85	3.24	0.00	3.74	1.84	2.73	0.83	1.88	>0.999999999999999	0.4
Q9H1E3	Nuclear ubiquitous casein and cyclin-dependent kinases substrate OS=Homo sapiens GN=N	0.00	2.77	1.34	0.00	0.00	0.00	0.00	4.12	0.48	0.00	0.00	1.20	0.00	0.00	0.00	0.00	0.00	0.00	0.00	3.37	0.37	0.00	0.00	0.00	0.83	0.31	>0.999999999999999	2.6
Q32P51	Heterogeneous nuclear ribonucleoprotein A1-like 2 OS=Homo sapiens GN=HNRNPA1L2 PE	0.00	4.61	2.24	3.03	0.00	0.00	0.00	0.00	0.00	0.00	0.00	0.00	0.00	0.00	0.00	0.00	3.77	1.53	1.59	0.00	0.00	4.16	0.00	0.00	0.82	0.92	>0.999999999999999	0.9
P52434	DNA-directed RNA polymerases I, II, and III subunit RPABC3 OS=Homo sapiens GN=POLR	4.88	0.00	0.00	0.00	0.00	2.91	0.00	0.00	0.00	2.09	0.00	0.00	0.00	4.03	2.22	1.38	0.00	0.00	0.00	2.59	0.00	0.00	0.00	1.14	0.82	0.95	>0.999999999999999	0.9
Q8WZA9	Immunity-related GTPase family Q protein OS=Homo sapiens GN=IRGQ PE=1 SV=1	2.69	1.15	0.56	0.76	0.00	4.22	0.00	0.00	0.00	0.00	0.00	0.50	0.00	1.01	0.00	0.00	0.00	0.77	0.00	1.43	0.15	1.04	0.00	0.62	0.82	0.42	>0.999999999999999	2.0
Q6FI81	Anamorsin OS=Homo sapiens GN=CIAPIN1 PE=1 SV=2	2.44	2.30	1.12	0.00	0.00	0.00	0.00	1.59	0.40	0.00	0.00	1.99	0.00	0.00	0.00	0.00	0.00	0.00	0.00	1.30	0.00	0.00	0.00	0.57	0.82	0.16	>0.999999999999999	5.3
Q04637	Eukaryotic translation initiation factor 4 gamma 1 OS=Homo sapiens GN=EIF4G1 PE=1 SV=	1.47	3.23	0.67	0.61	0.00	2.04	0.00	0.32	0.32	0.21	0.38	0.60	0.00	1.21	0.22	0.14	0.00	0.00	0.00	1.04	0.18	0.83	0.00	0.57	0.82	0.35	>0.999999999999999	2.3
Q2VIR3	Putative eukaryotic translation initiation factor 2 subunit 3-like protein OS=Homo sapiens GN	0.00	6.45	0.00	0.00	0.00	0.00	3.37	0.00	0.00	0.00	0.00	0.00	0.53	0.00	1.44	0.00	1.13	0.00	0.00	0.00	0.00	1.25	1.99	0.00	0.82	0.53	>0.999999999999999	1.5
O75531	Barrier-to-autointegration factor OS=Homo sapiens GN=BANF1 PE=1 SV=1	0.00	0.00	0.00	0.00	0.00	0.00	0.00	0.00	0.00	0.00	6.44	3.39	0.00	0.00	0.00	0.00	0.00	0.00	0.00	0.00	0.00	0.00	0.00	0.00	0.82	0.00	>0.999999999999999	#DIV/0!
Q9Y3C6	Peptidyl-prolyl cis-trans isomerase-like 1 OS=Homo sapiens GN=PPIL1 PE=1 SV=1	4.40	0.00	2.02	0.00	0.00	0.00	0.00	0.00	0.00	0.00	3.41	0.00	0.00	0.00	0.00	0.00	0.00	0.00	0.00	2.33	0.00	0.00	0.00	0.00	0.82	0.19	>0.999999999999999	4.2
Q16864	V-type proton ATPase subunit F OS=Homo sapiens GN=ATP6V1F PE=1 SV=2	0.00	5.99	0.00	0.00	0.00	3.78	0.00	0.00	0.00	0.00	0.00	0.00	0.00	0.00	0.00	0.00	0.00	0.00	0.00	0.00	0.00	0.00	0.00	0.00	0.81	0.00	>0.999999999999999	#DIV/0!
Q13310	Polyadenylate-binding protein 4 OS=Homo sapiens GN=PABPC4 PE=1 SV=1	2.20	3.46	1.68	0.00	0.00	0.00	0.00	0.00	0.59	0.00	0.95	0.90	0.00	1.81	0.00	0.00	0.00	0.00	0.00	1.94	0.00	0.00	0.00	0.00	0.81	0.31	>0.999999999999999	2.6
P84090	Enhancer of rudimentary homolog OS=Homo sapiens GN=ERH PE=1 SV=1	6.84	0.00	0.00	0.00	0.00	0.00	0.00	0.00	0.00	2.92	0.00	0.00	0.00	5.64	0.00	0.00	0.00	4.29	0.00	0.00	0.00	0.00	4.29	0.00	0.81	1.19	>0.999999999999999	0.7
Q15018	BRISC complex subunit Abro1 OS=Homo sapiens GN=FAM175B PE=1 SV=2	1.71	0.00	0.78	1.06	0.00	2.18	1.18	0.00	0.00	0.00	1.33	1.49	0.61	1.41	0.78	0.00	0.00	0.00	0.00	0.91	0.00	1.45	1.07	0.40	0.81	0.55	>0.999999999999999	1.5
P36969	Phospholipid hydroperoxide glutathione peroxidase, mitochondrial OS=Homo sapiens GN=G	0.00	0.00	0.00	0.00	0.00	4.66	0.00	0.00	0.00	0.00	0.00	5.08	0.00	3.02	0.00	0.00	0.00	0.00	0.00	1.94	0.00	0.00	0.00	0.00	0.81	0.41	>0.999999999999999	2.0
P42830	C-X-C motif chemokine 5 OS=Homo sapiens GN=CXCL5 PE=1 SV=1	0.00	0.00	0.00	9.71	0.00	0.00	0.00	0.00	0.00	0.00	0.00	0.00	2.46	0.00	0.00	0.00	0.00	0.00	0.00	0.00	0.00	0.00	0.00	0.00	0.81	0.20	>0.999999999999999	4.0
Q9BRT3	Migration and invasion enhancer 1 OS=Homo sapiens GN=MIEN1 PE=1 SV=1	6.59	0.00	3.03	0.00	0.00	0.00	0.00	0.00	0.00	0.00	0.00	0.00	0.00	0.00	0.00	0.00	0.00	0.00	0.00	0.00	0.00	0.00	0.00	0.00	0.80	0.00	>0.999999999999999	#DIV/0!
Q6ZN66	Guanylate-binding protein 6 OS=Homo sapiens GN=GBP6 PE=2 SV=1	1.22	1.15	0.00	0.76	0.96	1.31	0.84	0.79	0.20	0.52	0.95	0.90	0.44	1.81	0.00	0.00	0.00	0.00	0.00	0.65	0.00	0.00	0.00	0.28	0.80	0.27	>0.999999999999999	3.0
O95361	Tripartite motif-containing protein 16 OS=Homo sapiens GN=TRIM16 PE=1 SV=3	0.00	2.30	0.56	0.00	1.92	3.20	0.00	0.00	0.00	0.00	0.00	1.59	0.44	1.01	1.11	0.34	0.94	0.00	0.00	2.07	0.31	0.00	0.00	0.57	0.80	0.57	>0.999999999999999	1.4
Q9BWD1	Acetyl-CoA acetyltransferase, cytosolic OS=Homo sapiens GN=ACAT2 PE=1 SV=2	6.35	0.00	0.90	0.00	0.00	2.33	0.00	0.00	0.00	0.00	0.00	0.00	0.70	1.61	0.00	0.00	0.00	0.00	0.00	0.00	0.00	0.00	1.23	0.00	0.80	0.30	>0.999999999999999	2.7
Q13263	Transcription intermediary factor 1-beta OS=Homo sapiens GN=TRIM28 PE=1 SV=5	3.66	0.00	0.45	1.06	0.77	1.60	0.67	0.63	0.00	0.00	0.00	0.70	0.00	0.00	0.44	0.28	0.00	0.00	0.00	0.91	0.00	0.00	0.61	0.00	0.80	0.19	>0.999999999999999	4.3
P41236	Protein phosphatase inhibitor 2 OS=Homo sapiens GN=PPP1R2 PE=1 SV=2	0.00	0.00	0.00	0.00	0.00	2.18	0.00	0.00	0.00	0.00	5.87	1.49	0.00	0.00	1.67	0.00	0.00	0.00	0.00	1.94	0.00	0.00	0.00	0.00	0.80	0.30	>0.999999999999999	2.6
P49588	Alanine--tRNA ligase, cytoplasmic OS=Homo sapiens GN=AARS PE=1 SV=2	0.73	2.07	0.67	0.45	0.58	1.89	0.00	0.48	0.24	0.00	1.14	1.29	0.00	2.62	0.00	0.21	0.00	0.00	0.00	0.78	0.28	0.00	0.46	0.17	0.80	0.38	>0.999999999999999	2.1
Q13283	Ras GTPase-activating protein-binding protein 1 OS=Homo sapiens GN=G3BP1 PE=1 SV=1	0.00	1.38	0.67	0.00	1.15	2.91	0.00	0.95	0.00	0.00	1.14	1.29	0.00	0.00	0.67	0.00	0.00	0.00	0.00	0.00	0.18	0.00	0.00	0.00	0.79	0.07	>0.999999999999999	11.2
P25325	3-mercaptopyruvate sulfurtransferase OS=Homo sapiens GN=MPST PE=1 SV=3	2.44	0.00	0.00	0.00	0.00	3.06	0.00	0.00	0.00	0.00	1.89	2.09	0.00	0.00	0.00	0.00	0.00	0.00	0.00	0.00	0.00	0.00	0.00	0.00	0.79	0.00	>0.999999999999999	#DIV/0!
O75506	Heat shock factor-binding protein 1 OS=Homo sapiens GN=HSBP1 PE=1 SV=1	0.00	9.45	0.00	0.00	0.00	0.00	0.00	0.00	0.00	0.00	0.00	0.00	0.00	0.00	0.00	0.00	0.00	0.00	0.00	0.00	0.00	0.00	0.00	0.00	0.79	0.00	>0.999999999999999	#DIV/0!
Q96B54	Zinc finger protein 428 OS=Homo sapiens GN=ZNF428 PE=1 SV=2	0.00	3.69	0.00	0.00	0.00	5.09	0.00	0.00	0.63	0.00	0.00	0.00	0.00	0.00	0.00	0.00	0.00	0.00	0.00	0.00	0.00	0.00	0.00	0.00	0.78	0.00	>0.999999999999999	#DIV/0!
P53396	ATP-citrate synthase OS=Homo sapiens GN=ACLY PE=1 SV=3	0.73	1.15	0.56	0.45	0.58	2.91	0.51	0.00	0.12	0.31	0.95	1.10	0.00	1.01	0.33	0.21	0.00	0.46	0.00	0.65	0.09	1.66	0.46	0.00	0.78	0.41	>0.999999999999999	1.9
Q8TAX7	Mucin-7 OS=Homo sapiens GN=MUC7 PE=1 SV=2	0.00	1.84	0.00	0.00	0.00	0.00	1.35	2.69	0.32	0.83	1.51	0.80	0.70	0.00	0.00	0.00	3.20	0.00	1.27	1.04	0.25	1.66	1.23	0.00	0.78	0.78	>0.999999999999999	1.0
Q9H0N0	Ras-related protein Rab-6C OS=Homo sapiens GN=RAB6C PE=2 SV=2	2.93	2.77	0.00	0.00	0.00	1.75	0.00	1.90	0.00	0.00	0.00	0.00	0.00	0.00	0.00	0.00	0.00	0.00	0.00	0.00	0.00	0.00	0.00	0.00	0.78	0.00	>0.999999999999999	#DIV/0!
P57764	Gasdermin-D OS=Homo sapiens GN=GSDMD PE=1 SV=1	3.18	0.00	0.00	0.00	0.00	1.89	0.00	0.95	0.24	0.00	2.46	0.60	0.00	0.00	0.00	0.00	0.00	0.00	0.00	0.78	0.00	2.70	0.00	0.34	0.78	0.32	>0.999999999999999	2.4
O14908	PDZ domain-containing protein GIPC1 OS=Homo sapiens GN=GIPC1 PE=1 SV=2	4.64	0.00	0.00	0.00	0.00	2.77	0.00	0.00	0.00	0.00	0.00	1.89	0.00	1.81	0.00	0.00	0.00	1.38	0.00	1.17	0.00	0.00	0.00	0.00	0.77	0.36	>0.999999999999999	2.1
P46777	60S ribosomal protein L5 OS=Homo sapiens GN=RPL5 PE=1 SV=3	0.00	0.00	3.59	0.00	0.00	2.91	0.00	0.00	0.79	0.00	0.00	1.99	0.00	4.03	0.00	1.38	0.00	1.53	0.00	1.30	0.00	0.00	0.00	0.00	0.77	0.69	>0.999999999999999	1.1
P98088	Mucin-5AC (Fragments) OS=Homo sapiens GN=MUC5AC PE=1 SV=3	0.24	2.77	0.11	0.00	0.38	0.29	0.67	0.16	0.16	4.07	0.00	0.40	0.00	0.40	0.00	0.07	0.00	0.61	6.66	1.81	0.06	0.83	0.31	0.74	0.77	0.96	>0.999999999999999	0.8
Q15437	Protein transport protein Sec23B OS=Homo sapiens GN=SEC23B PE=1 SV=2	1.95	0.00	1.34	0.00	0.00	2.91	0.67	0.63	0.16	0.00	0.76	0.80	0.35	1.61	0.44	0.28	0.75	1.84	0.00	2.07	0.25	0.83	0.00	0.23	0.77	0.72	>0.999999999999999	1.1
Q9H4A5	Golgi phosphoprotein 3-like OS=Homo sapiens GN=GOLPH3L PE=1 SV=1	0.00	4.84	0.00	1.52	0.00	1.46	0.00	0.00	0.40	0.00	0.00	1.00	0.00	0.00	0.00	0.00	0.00	0.00	0.00	0.00	0.00	0.00	0.00	0.00	0.77	0.00	>0.999999999999999	#DIV/0!
P05120	Plasminogen activator inhibitor 2 OS=Homo sapiens GN=SERPINB2 PE=1 SV=2	1.71	1.61	0.00	0.00	1.34	4.51	0.00	0.00	0.00	0.00	0.00	0.00	1.32	1.41	0.00	0.00	1.32	1.07	2.38	0.91	0.46	1.45	1.07	0.00	0.76	0.95	>0.999999999999999	0.8
Q15046	Lysine--tRNA ligase OS=Homo sapiens GN=KARS PE=1 SV=3	2.44	2.30	0.56	0.76	0.00	1.46	0.00	0.00	0.20	0.00	0.95	0.50	0.00	1.01	0.00	0.34	0.00	0.77	0.00	0.65	0.15	0.00	0.00	0.28	0.76	0.27	>0.999999999999999	2.9
Q9NSD9	Phenylalanine--tRNA ligase beta subunit OS=Homo sapiens GN=FARSB PE=1 SV=3	2.44	1.15	0.56	0.00	0.00	3.06	0.00	0.00	0.00	0.00	0.95	1.00	0.88	3.22	0.00	0.00	0.00	0.77	0.00	2.07	0.00	1.04	0.00	0.57	0.76	0.71	>0.999999999999999	1.1
O14618	Copper chaperone for superoxide dismutase OS=Homo sapiens GN=CCS PE=1 SV=1	2.69	2.54	1.23	0.00	0.00	1.60	0.00	0.00	0.00	0.00	0.00	1.10	0.00	0.00	0.00	0.00	0.00	0.00	0.00	0.00	0.00	0.00	0.00	0.62	0.76	0.05	>0.999999999999999	14.7
Q9H2G2	STE20-like serine/threonine-protein kinase OS=Homo sapiens GN=SLK PE=1 SV=1	0.00	2.07	0.22	1.06	0.00	2.04	0.34	0.79	0.48	0.00	0.95	1.20	0.61	2.82	0.22	0.62	0.00	0.77	0.32	1.81	0.15	1.87	0.31	0.97	0.76	0.87	>0.999999999999999	0.9
Q6MZZ7	Calpain-13 OS=Homo sapiens GN=CAPN13 PE=1 SV=2	0.98	2.07	0.00	0.00	0.00	1.89	0.67	0.00	0.52	0.00	1.70	1.29	0.00	3.63	0.00	0.00	0.00	0.00	0.00	0.52	0.00	1.87	0.00	0.51	0.76	0.54	>0.999999999999999	1.4
P81605	Dermcidin OS=Homo sapiens GN=DCD PE=1 SV=2	0.00	0.00	0.00	0.00	0.00	4.37	0.00	4.76	0.00	0.00	0.00	0.00	0.00	0.00	0.00	2.07	0.00	0.00	0.00	0.00	0.00	6.23	0.00	0.00	0.76	0.69	>0.999999999999999	1.1
Q5T6V5	UPF0553 protein C9orf64 OS=Homo sapiens GN=C9orf64 PE=1 SV=1	1.95	0.00	0.00	0.00	0.00	3.93	0.00	0.00	0.00	0.00	1.51	1.69	0.00	0.00	0.00	0.00	0.00	0.00	0.00	0.00	0.00	0.00	0.00	0.00	0.76	0.00	>0.999999999999999	#DIV/0!
P84103	Serine/arginine-rich splicing factor 3 OS=Homo sapiens GN=SRSF3 PE=1 SV=1	0.00	0.00	0.00	0.00	0.00	5.39	0.00	0.00	0.00	0.00	0.00	3.69	0.00	0.00	0.00	0.00	0.00	0.00	0.00	0.00	0.00	0.00	0.00	0.00	0.76	0.00	>0.999999999999999	#DIV/0!
P14735	Insulin-degrading enzyme OS=Homo sapiens GN=IDE PE=1 SV=4	0.73	0.69	0.34	0.45	0.58	3.49	1.35	0.00	0.24	0.00	0.57	0.60	0.00	0.60	0.00	0.21	0.00	0.00	0.48	0.00	0.18	2.29	0.00	0.17	0.75	0.33	>0.999999999999999	2.3
Q9BV20	Methylthioribose-1-phosphate isomerase OS=Homo sapiens GN=MRI1 PE=1 SV=1	4.15	1.84	1.90	0.00	0.00	0.00	0.00	0.00	0.32	0.00	0.00	0.80	0.00	0.00	0.00	0.00	0.00	0.00	0.00	1.04	0.00	0.00	0.00	0.45	0.75	0.12	>0.999999999999999	6.0
O75369	Filamin-B OS=Homo sapiens GN=FLNB PE=1 SV=2	1.22	0.92	0.56	0.76	0.00	3.20	0.00	0.16	0.40	0.42	0.95	0.40	0.18	2.22	0.00	0.55	0.00	0.92	0.00	2.33	0.18	1.45	0.92	0.28	0.75	0.75	>0.999999999999999	1.0
P57721	Poly(rC)-binding protein 3 OS=Homo sapiens GN=PCBP3 PE=1 SV=2	0.00	0.00	0.00	0.00	5.18	0.00	0.00	2.69	1.07	0.00	0.00	0.00	0.00	0.00	0.00	0.00	0.00	0.00	0.00	0.00	0.00	0.00	0.00	0.00	0.75	0.00	>0.999999999999999	#DIV/0!
Q7RTV0	PHD finger-like domain-containing protein 5A OS=Homo sapiens GN=PHF5A PE=1 SV=1	6.35	0.00	0.00	0.00	0.00	0.00	0.00	0.00	0.00	0.00	0.00	2.59	0.00	0.00	0.00	0.00	0.00	0.00	0.00	0.00	0.00	0.00	0.00	0.00	0.75	0.00	>0.999999999999999	#DIV/0!
O43490	Prominin-1 OS=Homo sapiens GN=PROM1 PE=1 SV=1	0.73	0.69	0.34	0.00	0.00	2.04	0.51	1.59	0.12	0.73	1.33	0.30	0.26	0.00	0.33	0.21	0.57	0.00	0.48	0.00	0.00	0.00	0.46	0.40	0.74	0.23	>0.999999999999999	3.3
P49589	Cysteine--tRNA ligase, cytoplasmic OS=Homo sapiens GN=CARS PE=1 SV=3	3.91	0.92	0.45	0.00	0.00	2.33	0.00	0.00	0.16	0.00	0.76	0.40	0.00	1.61	0.00	0.28	0.00	0.00	0.00	1.04	0.00	0.00	0.00	0.45	0.74	0.28	>0.999999999999999	2.6
O00534	von Willebrand factor A domain-containing protein 5A OS=Homo sapiens GN=VWA5A PE=2	1.95	1.84	0.00	0.00	0.00	3.64	0.00	0.00	0.32	0.00	0.76	0.40	0.00	0.81	0.00	0.28	0.00	0.00	0.00	1.04	0.00	0.00	0.00	0.23	0.74	0.20	>0.999999999999999	3.8
Q99685	Monoglyceride lipase OS=Homo sapiens GN=MGLL PE=1 SV=2	2.44	0.00	0.00	0.00	0.00	1.46	0.00	0.00	0.00	0.00	3.98	1.00	0.00	0.00	0.00	0.00	0.00	0.00	0.00	0.00	0.00	0.00	0.00	0.00	0.74	0.00	>0.999999999999999	#DIV/0!

P53609	Geranylgeranyl transferase type-1 subunit beta OS=Homo sapiens GN=PGGT1B PE=1 SV=	0.00	1.84	0.90	0.00	1.53	1.16	0.00	0.00	0.32	0.00	1.51	1.59	0.00	0.00	0.00	0.55	0.00	0.00	0.00	2.07	0.00	1.66	0.00	0.00	0.74	0.36	>0.999999999999999	2.1
Q9UBF2	Coatomer subunit gamma-2 OS=Homo sapiens GN=COPG2 PE=1 SV=1	3.42	0.69	1.12	0.00	0.00	2.62	0.00	0.00	0.00	0.00	0.00	1.00	0.00	1.41	0.00	0.00	0.57	0.00	0.00	0.00	0.00	0.00	0.00	0.57	0.74	0.21	>0.999999999999999	3.5
Q16555	Dihydropyrimidinase-related protein 2 OS=Homo sapiens GN=DPYSL2 PE=1 SV=1	4.15	0.00	0.56	0.76	0.00	0.73	0.00	0.00	0.00	0.00	0.95	1.69	0.00	0.00	0.00	0.00	0.00	0.00	0.00	0.00	0.00	0.00	0.00	0.00	0.74	0.00	>0.999999999999999	#DIV/0!
P49006	MARCKS-related protein OS=Homo sapiens GN=MARCKSL1 PE=1 SV=2	0.00	3.92	0.00	0.00	0.00	0.00	0.00	0.00	0.00	0.00	3.22	1.69	0.00	3.42	0.00	0.00	0.00	0.00	0.00	2.20	0.00	3.53	2.61	0.00	0.74	0.98	>0.999999999999999	0.8
Q9NP79	Vacuolar protein sorting-associated protein VTA1 homolog OS=Homo sapiens GN=VTA1 PE	2.44	0.00	1.12	0.00	0.00	0.00	1.69	1.59	0.00	0.00	0.00	1.99	0.00	0.00	0.00	0.00	0.00	0.00	0.00	0.00	0.00	0.00	0.00	0.00	0.74	0.00	>0.999999999999999	#DIV/0!
P45877	Peptidyl-prolyl cis-trans isomerase C OS=Homo sapiens GN=PPIC PE=1 SV=1	3.66	0.00	0.00	2.27	2.88	0.00	0.00	0.00	0.00	0.00	0.00	0.00	0.00	3.02	0.00	0.00	0.00	0.00	2.38	0.00	0.00	3.12	0.00	0.00	0.73	0.71	>0.999999999999999	1.0
P40123	Adenylyl cyclase-associated protein 2 OS=Homo sapiens GN=CAP2 PE=1 SV=1	6.59	0.00	0.00	0.00	0.00	0.87	0.00	0.00	0.00	0.00	0.00	1.29	0.00	0.00	0.00	0.00	0.00	0.00	0.00	0.00	0.00	0.00	0.00	0.00	0.73	0.00	>0.999999999999999	#DIV/0!
Q07021	Complement component 1 Q subcomponent-binding protein, mitochondrial OS=Homo sapie	0.00	0.00	0.00	0.00	0.00	1.46	0.00	3.49	0.87	1.04	1.89	0.00	0.00	2.01	1.11	0.69	0.00	1.53	1.59	2.85	0.00	0.00	1.53	0.57	0.73	0.99	>0.999999999999999	0.7
P35573	Glycogen debranching enzyme OS=Homo sapiens GN=AGL PE=1 SV=3	2.44	0.92	0.67	0.00	0.00	2.62	0.00	0.00	0.56	0.00	1.14	0.40	0.18	0.00	0.22	0.00	0.00	0.00	0.00	0.78	0.00	0.42	0.00	0.11	0.73	0.14	>0.999999999999999	5.1
P07998	Ribonuclease pancreatic OS=Homo sapiens GN=RNASE1 PE=1 SV=4	0.00	0.00	2.13	0.00	0.00	0.00	0.00	3.01	0.00	0.00	3.60	0.00	0.00	0.00	0.00	0.00	3.58	0.00	0.00	0.00	0.00	0.00	2.91	1.08	0.73	0.63	>0.999999999999999	1.2
Q53H82	Beta-lactamase-like protein 2 OS=Homo sapiens GN=LACTB2 PE=1 SV=2	2.44	0.00	2.35	0.00	0.00	3.06	0.00	0.00	0.83	0.00	0.00	0.00	0.00	0.00	0.00	0.00	0.00	0.00	0.00	2.72	0.00	0.00	0.00	0.00	0.72	0.23	>0.999999999999999	3.2
Q8IXM2	Chromatin complexes subunit BAP18 OS=Homo sapiens GN=BAP18 PE=1 SV=1	4.64	0.00	2.13	0.00	0.00	0.00	0.00	0.00	0.00	0.00	0.00	1.89	0.00	0.00	0.00	0.00	0.00	0.00	0.00	0.00	0.00	0.00	0.00	0.00	0.72	0.00	>0.999999999999999	#DIV/0!
P25685	DnaJ homolog subfamily B member 1 OS=Homo sapiens GN=DNAJB1 PE=1 SV=4	0.00	2.07	1.01	0.00	0.00	2.62	0.00	0.00	0.36	0.00	1.70	0.90	0.00	0.00	0.00	0.00	0.00	0.00	1.43	1.17	0.00	0.00	0.00	0.00	0.72	0.22	>0.999999999999999	3.3
Q08623	Pseudouridine-5'-monophosphatase OS=Homo sapiens GN=HDHD1 PE=1 SV=3	0.00	0.00	0.00	0.00	2.49	4.08	0.00	2.06	0.00	0.00	0.00	0.00	0.00	0.00	0.00	0.00	0.00	0.00	0.00	0.00	0.00	2.70	0.00	0.00	0.72	0.23	>0.999999999999999	3.2
Q9H446	RWD domain-containing protein 1 OS=Homo sapiens GN=RWDD1 PE=1 SV=1	2.93	0.00	0.00	0.00	0.00	1.75	0.00	0.00	0.48	0.00	2.27	1.20	0.00	2.42	0.00	0.00	0.00	0.00	0.00	1.55	0.00	0.00	0.00	0.68	0.72	0.39	>0.999999999999999	1.9
P09497	Clathrin light chain B OS=Homo sapiens GN=CLTB PE=1 SV=1	0.00	0.00	0.00	0.00	0.00	6.55	0.00	2.06	0.00	0.00	0.00	0.00	0.00	0.00	0.00	0.90	0.00	1.99	2.06	3.63	0.40	2.70	1.99	0.00	0.72	1.14	>0.999999999999999	0.6
Q9UL15	BAG family molecular chaperone regulator 5 OS=Homo sapiens GN=BAG5 PE=1 SV=1	3.18	1.38	0.00	0.00	0.00	2.91	0.00	0.00	0.00	0.00	1.14	0.00	0.00	0.00	0.00	0.00	0.00	0.00	0.00	0.00	0.00	0.00	0.00	0.34	0.72	0.03	>0.999999999999999	25.3
P35637	RNA-binding protein FUS OS=Homo sapiens GN=FUS PE=1 SV=1	1.47	1.38	0.67	0.91	1.15	0.00	1.01	0.00	0.24	0.00	1.14	0.60	0.53	1.21	0.00	0.41	0.00	0.00	0.00	0.00	0.18	1.25	0.00	0.34	0.71	0.33	>0.999999999999999	2.2
Q13557	Calcium/calmodulin-dependent protein kinase type II subunit delta OS=Homo sapiens GN=C	2.93	0.00	1.34	0.91	1.15	0.00	0.00	0.95	0.00	0.00	0.00	1.20	0.53	0.00	0.00	1.72	0.00	0.00	0.00	1.55	0.37	0.00	0.00	0.00	0.71	0.35	>0.999999999999999	2.0
Q9UNF1	Melanoma-associated antigen D2 OS=Homo sapiens GN=MAGED2 PE=1 SV=2	1.22	1.15	1.12	0.00	0.00	1.46	1.69	0.00	0.40	0.00	0.95	0.50	0.00	1.01	0.00	0.34	0.00	0.77	0.00	1.30	0.00	0.00	0.00	0.28	0.71	0.31	>0.999999999999999	2.3
O00193	Small acidic protein OS=Homo sapiens GN=SMAP PE=1 SV=1	3.91	0.00	0.00	0.00	0.00	2.33	0.00	0.00	0.63	0.00	0.00	1.59	0.00	0.00	0.00	0.00	0.00	0.00	0.00	0.00	0.00	0.00	0.00	0.00	0.71	0.00	>0.999999999999999	#DIV/0!
P07951	Tropomyosin beta chain OS=Homo sapiens GN=TPM2 PE=1 SV=1	5.13	0.00	0.00	0.00	0.00	0.00	0.00	0.00	0.00	0.00	0.00	3.29	0.00	4.23	0.00	0.00	1.89	0.00	0.00	0.00	0.00	0.00	0.00	0.00	0.70	0.51	>0.999999999999999	1.4
P00367	Glutamate dehydrogenase 1, mitochondrial OS=Homo sapiens GN=GLUD1 PE=1 SV=2	1.22	1.15	0.56	0.00	0.00	1.60	0.00	2.69	0.00	1.15	0.00	0.00	0.44	1.01	0.00	0.34	0.00	0.77	0.79	0.65	0.00	0.00	0.00	0.28	0.70	0.36	>0.999999999999999	2.0
Q9NVA2	Septin-11 OS=Homo sapiens GN=SEPT11 PE=1 SV=3	3.42	1.61	0.00	0.00	0.00	3.06	0.00	0.00	0.28	0.00	0.00	0.00	0.00	2.82	0.00	0.00	0.00	0.00	0.00	1.81	0.00	1.45	0.00	0.79	0.70	0.57	>0.999999999999999	1.2
P61289	Proteasome activator complex subunit 3 OS=Homo sapiens GN=PSME3 PE=1 SV=1	2.69	2.54	0.00	0.00	0.00	1.60	0.00	0.00	0.44	0.00	0.00	1.10	0.00	0.00	0.00	0.00	0.00	0.00	0.00	0.00	0.00	0.00	0.00	0.00	0.70	0.00	>0.999999999999999	#DIV/0!
Q16204	Coiled-coil domain-containing protein 6 OS=Homo sapiens GN=CCDC6 PE=1 SV=2	3.18	1.38	0.67	0.00	0.00	0.87	0.00	0.95	0.00	0.00	0.00	1.29	0.00	2.62	0.00	0.00	0.00	0.00	0.00	0.78	0.18	0.00	0.00	0.74	0.70	0.36	>0.999999999999999	1.9
O75351	Vacuolar protein sorting-associated protein 4B OS=Homo sapiens GN=VPS4B PE=1 SV=2	5.13	0.00	0.78	0.00	0.00	1.02	0.00	0.00	0.00	0.00	0.00	1.39	0.00	1.41	0.00	0.00	0.48	0.00	0.00	0.91	0.00	0.00	0.00	0.00	0.69	0.23	>0.999999999999999	3.0
P02794	Ferritin heavy chain OS=Homo sapiens GN=FTH1 PE=1 SV=2	8.30	0.00	0.00	0.00	0.00	0.00	0.00	0.00	0.00	0.00	0.00	0.00	0.00	0.00	0.00	0.00	0.00	0.00	0.00	0.00	0.00	0.00	0.00	0.00	0.69	0.00	>0.999999999999999	#DIV/0!
O00148	ATP-dependent RNA helicase DDX39A OS=Homo sapiens GN=DDX39A PE=1 SV=2	0.00	0.00	0.00	0.00	1.34	0.00	2.36	4.60	0.00	0.00	0.00	0.00	0.61	0.00	2.33	0.00	2.64	0.00	0.00	0.00	0.00	2.91	1.07	0.00	0.69	0.80	>0.999999999999999	0.9
Q9UKY7	Protein CDV3 homolog OS=Homo sapiens GN=CDV3 PE=1 SV=1	2.93	0.00	0.00	0.00	0.00	0.00	0.00	1.90	0.00	0.00	2.27	1.20	0.00	0.00	0.00	0.00	0.00	0.00	0.00	0.00	0.00	0.00	0.00	0.00	0.69	0.00	>0.999999999999999	#DIV/0!
P49207	60S ribosomal protein L34 OS=Homo sapiens GN=RPL34 PE=1 SV=3	0.00	0.00	0.00	0.00	0.00	8.30	0.00	0.00	0.00	0.00	0.00	0.00	0.00	0.00	2.78	0.00	0.00	0.00	0.00	3.24	0.77	0.00	0.00	1.42	0.69	0.68	>0.999999999999999	1.0
P61956	Small ubiquitin-related modifier 2 OS=Homo sapiens GN=SUMO2 PE=1 SV=3	0.00	0.00	8.29	0.00	0.00	0.00	0.00	0.00	0.00	0.00	0.00	0.00	0.00	0.00	0.00	0.00	0.00	0.00	0.00	0.00	0.00	0.00	0.00	0.00	0.69	0.00	>0.999999999999999	#DIV/0!
Q92945	Far upstream element-binding protein 2 OS=Homo sapiens GN=KHSRP PE=1 SV=4	0.98	0.92	1.01	0.00	0.00	1.31	0.00	0.00	0.00	0.00	2.65	1.39	0.00	0.00	0.00	0.28	0.00	0.00	0.00	1.17	0.00	0.00	0.00	0.23	0.69	0.14	>0.999999999999999	5.0
P02814	Submaxillary gland androgen-regulated protein 3B OS=Homo sapiens GN=SMR3B PE=1 SV	0.00	0.00	0.00	0.00	8.25	0.00	0.00	0.00	0.00	0.00	0.00	0.00	0.00	0.00	0.00	0.00	8.11	0.00	0.00	0.00	0.00	0.00	0.00	0.00	0.69	0.68	>0.999999999999999	1.0
P60510	Serine/threonine-protein phosphatase 4 catalytic subunit OS=Homo sapiens GN=PPP4C PE	2.20	2.07	0.00	0.00	0.00	0.00	0.00	0.00	0.36	0.00	3.60	0.00	0.00	1.81	0.00	0.00	0.00	0.00	0.00	0.00	0.00	0.00	0.00	0.00	0.69	0.15	>0.999999999999999	4.5
P01814	Ig heavy chain V-II region OU OS=Homo sapiens PE=1 SV=1	0.00	0.00	2.69	0.00	0.00	0.00	0.00	0.00	0.00	5.53	0.00	0.00	0.00	0.00	0.00	0.00	0.00	0.00	0.00	0.00	0.00	11.01	0.00	0.00	0.69	0.92	>0.999999999999999	0.7
P04278	Sex hormone-binding globulin OS=Homo sapiens GN=SHBG PE=1 SV=2	1.95	0.00	0.00	1.21	0.00	0.00	2.70	0.00	0.00	0.83	1.51	0.00	0.00	0.00	0.00	0.00	0.00	0.00	3.80	1.04	0.25	0.00	0.00	0.45	0.68	0.46	>0.999999999999999	1.5
Q16576	Histone-binding protein RBBP7 OS=Homo sapiens GN=RBBP7 PE=1 SV=1	1.71	1.61	0.00	0.00	0.00	0.00	0.00	0.00	0.00	0.00	2.65	2.19	0.00	0.00	0.00	0.00	0.00	0.00	0.00	0.00	0.00	0.00	0.00	0.00	0.68	0.00	>0.999999999999999	#DIV/0!
Q13409	Cytoplasmic dynein 1 intermediate chain 2 OS=Homo sapiens GN=DYNC1I2 PE=1 SV=3	2.20	1.15	1.01	0.76	0.00	1.31	0.00	0.00	0.00	0.00	1.70	0.00	0.79	1.81	0.00	0.62	0.00	0.00	0.00	0.00	0.00	0.00	0.00	0.28	0.68	0.29	>0.999999999999999	2.3
Q13148	TAR DNA-binding protein 43 OS=Homo sapiens GN=TARDBP PE=1 SV=1	1.71	0.00	0.78	1.06	0.00	1.02	0.00	0.00	0.00	0.00	2.84	0.70	0.00	1.41	0.78	0.00	0.00	0.00	0.00	0.00	0.22	0.00	0.00	0.40	0.68	0.23	>0.999999999999999	2.9
Q9NWS1	PCNA-interacting partner OS=Homo sapiens GN=PARPBP PE=1 SV=3	1.22	1.15	0.00	0.76	0.96	0.73	0.84	0.79	0.20	0.00	0.95	0.50	0.44	1.01	0.56	0.34	0.94	0.77	0.79	0.65	0.15	1.04	0.77	0.28	0.67	0.64	>0.999999999999999	1.0
Q9UNS2	COP9 signalosome complex subunit 3 OS=Homo sapiens GN=COPS3 PE=1 SV=3	1.71	1.61	0.78	0.00	0.00	2.04	1.18	0.00	0.00	0.00	0.00	0.70	0.00	0.00	0.00	0.00	0.00	0.00	0.00	0.00	0.00	0.00	0.00	0.00	0.67	0.00	>0.999999999999999	#DIV/0!
Q8TAT6	Nuclear protein localization protein 4 homolog OS=Homo sapiens GN=NPLOC4 PE=1 SV=3	2.44	0.00	0.00	0.00	0.00	2.18	0.00	0.00	0.00	0.00	1.89	1.49	0.00	0.00	0.00	0.00	0.00	0.00	0.00	0.65	0.00	0.00	0.00	0.00	0.67	0.05	>0.999999999999999	12.4
Q96HY6	DDRGK domain-containing protein 1 OS=Homo sapiens GN=DDRGK1 PE=1 SV=2	2.20	0.00	1.01	0.00	0.00	1.31	0.00	1.43	0.36	0.00	1.70	0.00	0.79	1.81	1.00	0.00	0.00	1.38	0.00	0.00	0.00	0.00	0.00	1.08	0.67	0.51	>0.999999999999999	1.3
P12268	Inosine-5'-monophosphate dehydrogenase 2 OS=Homo sapiens GN=IMPDH2 PE=1 SV=2	0.00	1.38	0.67	0.00	0.00	1.75	0.00	0.00	0.00	0.00	3.60	0.60	0.00	0.00	0.00	0.00	0.00	0.00	0.00	0.00	0.18	0.00	0.00	0.00	0.67	0.02	>0.999999999999999	43.3
P27635	60S ribosomal protein L10 OS=Homo sapiens GN=RPL10 PE=1 SV=4	3.18	3.00	0.00	0.00	0.00	0.00	0.00	0.00	0.52	0.00	0.00	1.29	0.00	0.00	0.00	0.00	0.00	0.00	0.00	0.00	0.00	0.00	0.00	0.00	0.67	0.00	>0.999999999999999	#DIV/0!
P50995	Annexin A11 OS=Homo sapiens GN=ANXA11 PE=1 SV=1	2.93	1.38	0.00	0.00	0.00	1.75	0.00	1.90	0.00	0.00	0.00	0.00	0.53	0.00	0.00	0.00	0.00	0.00	0.00	0.00	0.00	0.00	0.00	0.00	0.66	0.04	>0.999999999999999	15.1
P61970	Nuclear transport factor 2 OS=Homo sapiens GN=NUTF2 PE=1 SV=1	5.62	0.00	0.00	0.00	0.00	0.00	0.00	0.00	0.00	0.00	0.00	2.29	0.00	0.00	0.00	0.00	0.00	0.00	0.00	0.00	0.71	0.00	0.00	0.00	0.66	0.06	>0.999999999999999	11.2
Q9Y5P4	Collagen type IV alpha-3-binding protein OS=Homo sapiens GN=COL4A3BP PE=1 SV=1	4.88	1.15	0.56	0.00	0.00	1.31	0.00	0.00	0.00	0.00	0.00	0.00	0.00	1.81	0.00	0.00	0.00	0.77	0.79	0.00	0.00	1.04	0.00	0.00	0.66	0.37	>0.999999999999999	1.8
P09012	U1 small nuclear ribonucleoprotein A OS=Homo sapiens GN=SNRPA PE=1 SV=3	0.00	2.54	0.00	1.67	0.00	1.60	0.00	0.00	0.00	0.00	2.08	0.00	0.00	0.00	0.00	0.00	0.00	0.00	0.00	0.00	0.00	0.00	0.00	0.00	0.66	0.00	>0.999999999999999	#DIV/0!
O76094	Signal recognition particle 72 kDa protein OS=Homo sapiens GN=SRP72 PE=1 SV=3	3.42	0.92	0.45	0.00	0.00	2.04	0.00	0.00	0.16	0.00	0.00	0.90	0.00	0.00	0.00	0.00	0.00	0.00	0.00	1.81	0.00	0.00	0.00	0.51	0.66	0.19	>0.999999999999999	3.4
P55000	Secreted Ly-6/uPAR-related protein 1 OS=Homo sapiens GN=SLURP1 PE=1 SV=2	0.00	0.00	3.25	0.00	0.00	0.00	0.00	4.60	0.00	0.00	0.00	0.00	0.00	0.00	0.00	0.00	0.00	0.00	0.00	0.00	0.00	0.00	0.00	0.00	0.65	0.00	>0.999999999999999	#DIV/0!
P05089	Arginase-1 OS=Homo sapiens GN=ARG1 PE=1 SV=2	0.00	2.30	0.00	1.52	1.92	0.00	0.00	0.00	0.00	2.09	0.00	0.00	0.88	0.00	0.00	0.00	0.00	0.00	0.00	0.00	0.00	15.17	6.75	0.00	0.65	1.90	>0.999999999999999	0.3
P20160	Azurocidin OS=Homo sapiens GN=AZU1 PE=1 SV=3	0.00	5.99	0.00	1.82	0.00	0.00	0.00	0.00	0.00	0.00	0.00	0.00	0.00	0.00	0.00	0.00	0.00	0.00	0.00	0.00	0.00	0.00	0.00	0.00	0.65	0.00	>0.999999999999999	#DIV/0!
Q03154	Aminoacylase-1 OS=Homo sapiens GN=ACY1 PE=1 SV=1	1.71	1.61	0.00	0.00	0.00	2.18	0.00	0.00	0.00	0.00	0.00	2.29	0.00	1.41	0.00	0.00	0.00	0.00	0.00	1.94	0.00	0.00	0.00	0.00	0.65	0.28	>0.999999999999999	2.3
P98179	Putative RNA-binding protein 3 OS=Homo sapiens GN=RBM3 PE=1 SV=1	4.88	0.00	0.00	0.00	0.00	2.91	0.00	0.00	0.00	0.00	0.00	0.00	0.00	0.00	0.00	0.00	0.00	0.00	0.00	0.00	0.00	0.00	0.00	0.00	0.65	0.00	>0.999999999999999	#DIV/0!
Q8IZ83	Aldehyde dehydrogenase family 16 member A1 OS=Homo sapiens GN=ALDH16A1 PE=1 S	0.98	0.92	0.45	0.00	0.00	1.75	0.00	0.00	0.16	0.42	1.51	1.59	0.35	0.81	0.44	0.00	0.00	0.61	0.00	0.52	0.00	1.66	0.61	0.45	0.65	0.46	>0.999999999999999	1.4
P30048	Thioredoxin-dependent peroxide reductase, mitochondrial OS=Homo sapiens GN=PRDX3 P	0.00	2.77	1.34	0.00	0.00	1.75	0.00	1.90	0.00	0.00	0.00	0.00	0.00	0.00	0.00	0.00	2.26	0.00	0.00	0.00	0.00	0.00	0.00	0.00	0.65	0.19	>0.999999999999999	3.4
Q9UDY2	Tight junction protein ZO-2 OS=Homo sapiens GN=TJP2 PE=1 SV=2	2.44	1.15	0.56	0.00	0.00	3.06	0.34	0.00	0.20	0.00	0.00	0.00	0.00	1.01	0.22	0.34	0.00	0.31	0.00	0.26	0.15	1.04	0.00	0.00	0.65	0.28	>0.999999999999999	2.3
Q14203	Dynactin subunit 1 OS=Homo sapiens GN=DCTN1 PE=1 SV=3	1.22	1.15	0.56	0.00	0.38	1.75	0.34	0.79	0.40	0.00	0.95	0.20	0.18	0.40	0.22	0.14	0.00	1.84	0.00	1.55	0.00	0.42	0.31	0.11	0.64	0.43	>0.999999999999999	1.5
Q9Y5K5	Ubiquitin carboxyl-terminal hydrolase isozyme L5 OS=Homo sapiens GN=UCHL5 PE=1 SV=	4.40	2.07	0.00	0.00	0.00	0.00	0.00	0.00	0.36	0.00	0.00	0.90	0.00	1.81	0.00	0.00	0.00	0.00	0.00	0.00	0.00	0.00	0.00	0.00	0.64	0.15	>0.999999999999999	4.3
Q16651	Prostasin OS=Homo sapiens GN=PRSS8 PE=1 SV=1	0.00	0.00	0.00	1.36	0.00	0.00	1.52	1.43	0.75	0.94	1.70	0.00	1.67	1.81	0.00	0.00	1.70	0.00	0.00	0.00	0.00	1.87	2.91	0.51	0.64	0.87	>0.999999999999999	0.7
P35580	Myosin-10 OS=Homo sapiens GN=MYH10 PE=1 SV=3	2.93	2.77	0.00	0.00	0.00	0.00	0.00	0.00	0.59	0.00	0.00	1.39	0.00	0.00	0.00	0.00	0.00	0.00	0.00	0.00	0.00	0.00	0.00	0.00	0.64	0.00	>0.999999999999999	#DIV/0!
Q15418	Ribosomal protein S6 kinase alpha-1 OS=Homo sapiens GN=RPS6KA1 PE=1 SV=2	1.95	0.92	0.45	0.00	0.00	1.75	0.00	0.00	0.16	0.00	0.76	1.69	0.00	0.81	0.00	0.00	0.00	0.00	0.00	0.00	0.12	0.00	0.00	0.68	0.64	0.13	>0.999999999999999	4.8
Q9HC24	Protein lifeguard 4 OS=Homo sapiens GN=TMBIM4 PE=1 SV=3	3.18	3.00	1.46	0.00	0.00	0.00	0.00	0.00	0.00	0.00	0.00	0.00	0.00	0.00	0.00	0.00	0.00	0.00	0.00	0.00	0.00	0.00	0.00	0.00	0.64	0.00	>0.999999999999999	#DIV/0!
P60022	Beta-defensin 1 OS=Homo sapiens GN=DEFB1 PE=1 SV=1	0.00	0.00	0.00	0.00	0.00	0.00	7.59	0.00	0.00	0.00	0.00	0.00	0.00	0.00	0.00	0.00	0.00	0.00	0.00	5.83	0.00	0.00	0.00	0.00	0.63	0.49	>0.999999999999999	1.3
P55010	Eukaryotic translation initiation factor 5 OS=Homo sapiens GN=EIF5 PE=1 SV=2	0.00	1.61	0.78	1.06	0.00	2.04	0.00	1.11	0.28	0.00	0.00	0.70	0.00	1.41	0.00	0.48	0.00	0.00	0.00	0.91	0.00	0.00	0.00	0.40	0.63	0.27	>0.999999999999999	2.4
Q07866	Kinesin light chain 1 OS=Homo sapiens GN=KLC1 PE=1 SV=2	2.44	0.00	0.56	0.00	0.00	4.08	0.00	0.00	0.00	0.00	0.00	0.50	0.00	0.00	0.00	0.00	0.00	0.00	0.00	0.65	0.00	0.00	0.00	0.00	0.63	0.05	>0.999999999999999	11.7
P53384	Cytosolic Fe-S cluster assembly factor NUBP1 OS=Homo sapiens GN=NUBP1 PE=1 SV=2	2.20	0.00	1.01	0.00	1.73	0.00	0.00	0.00	0.00	0.00	1.70	0.90	0.00	1.81	0.00	0.62	0.00	0.00	0.00	1.17	0.00	0.00	0.00	0.51	0.63	0.34	>0.999999999999999	1.8
Q9BV86	Alpha N-terminal protein methyltransferase 1A OS=Homo sapiens GN=METTL11A PE=1 SV	3.18	0.00	0.00	0.00	0.00	1.89	0.00	0.00	0.00	0.00	2.46	0.00	0.00	2.62	0.00	0.00	0.00	1.99	0.00	1.68	0.00	0.00	0.00	0.74	0.63	0.59	>0.999999999999999	1.1
Q96GX9	Probable methylthioribulose-1-phosphate dehydratase OS=Homo sapiens GN=APIP PE=1 S	2.93	2.77	0.00	1.82	0.00	0.00	0.00	0.00	0.00	0.00	0.00	0.00	0.00	0.00	0.00	0.00	0.00	0.00	0.00	1.55	0.00	0.00	0.00	0.00	0.63	0.13	>0.999999999999999	4.8
Q9UHB9	Signal recognition particle 68 kDa protein OS=Homo sapiens GN=SRP68 PE=1 SV=2	1.22	0.00	0.56	0.00	0.00	2.04	0.00	0.00	0.00	0.00	1.70	1.99	0.00	1.81	0.56	0.00	0.00	0.00	0.00	0.65	0.15	0.00	0.00	0.28	0.63	0.29	>0.999999999999999	2.2
P08397	Porphobilinogen deaminase OS=Homo sapiens GN=HMBS PE=1 SV=2	4.15	0.00	0.00	0.00	0.00	0.00	1.35	0.00	0.32	0.00	0.00	1.69	0.00	0.00	0.00	0.55	0.00	0.00	0.00	1.04	0.00	0.00	0.00	0.45	0.63	0.17	>0.999999999999999	3.7
O43852	Calumenin OS=Homo sapiens GN=CALU PE=1 SV=2	0.00	0.00	1.01	0.00	0.00	0.00	1.52	4.60	0.36	0.00	0.00	0.00	1.67	0.00	0.00	0.62	1.70	1.38	0.00	0.00	0.00	1.87	1.38	0.00	0.62	0.72	>0.999999999999999	0.9
Q9P2E9	Ribosome-binding protein 1 OS=Homo sapiens GN=RRBP1 PE=1 SV=4	1.71	2.54	0.22	0.00	0.00	2.33	0.00	0.32	0.16	0.00	0.00	0.20	0.00	5.24	0.22	0.28	0.00	1.69	0.00	3.37	0.12	0.42	0.00	0.40	0.62	0.98	>0.999999999999999	0.6
Q92871	Phosphomannomutase 1 OS=Homo sapiens GN=PMM1 PE=1 SV=2	2.69	0.00	0.00	0.00	0.00	1.60	0.00	0.00	0.00	0.00	2.08	1.10	0.00	0.00	0.00	0.00	0.00	0.00	0.00	0.00	0.00	0.00	0.00	0.00	0.62	0.00	>0.999999999999999	#DIV/0!
P36980	Complement factor H-related protein 2 OS=Homo sapiens GN=CFHR2 PE=1 SV=1	0.00	0.00	0.00	7.43	0.00	0.00	0.00	0.00	0.00	0.00	0.00	0.00	0.00	0.00	0.00	2.41	0.00	0.00	0.00	0.00	0.00	0.00	3.37	0.00	0.62	0.48	>0.999999999999999	1.3
Q92623	Tetratricopeptide repeat protein 9A OS=Homo sapiens GN=TTC9 PE=2 SV=3	0.00	0.00	1.57	0.00	0.00	2.04	0.00	0.00	1.15	0.00	2.65	0.00	0.00	2.82	0.00	0.00	0.00	0.00	0.00	1.81	0.00	0.00	0.00	0.79	0.62	0.45	>0.999999999999999	1.4
O95445	Apolipoprotein M OS=Homo sapiens GN=APOM PE=1 SV=2	0.00	0.00	0.00	0.00	0.00	0.00	5.74	0.00	0.00	1.67	0.00	0.00	0.00	0.00	1.78	0.00	0.00	0.00	8.56	0.00	0.00	0.00	0.00	0.00	0.62	0.86	>0.999999999999999	0.7
Q99622	Protein C10 OS=Homo sapiens GN=C12orf57 PE=1 SV=1	0.00	0.00	0.00	0.00	0.00	3.78	0.00	0.00	1.03	0.00	0.00	2.59	0.00	5.24	0.00	0.00	0.00	0.00	0.00	0.00	0.00	0.00	0.00	0.00	0.62	0.44	>0.999999999999999	1.4
Q92526	T-complex protein 1 subunit zeta-2 OS=Homo sapiens GN=CCT6B PE=1 SV=5	0.00	7.38	0.00	0.00	0.00	0.00	0.00	0.00	0.00	0.00	0.00	0.00	0.00	0.00	0.00	0.00	0.00	0.00	0.00	0.00	0.00	0.00	0.00	0.00	0.61	0.00	>0.999999999999999	#DIV/0!
P04003	C4b-binding protein alpha chain OS=Homo sapiens GN=C4BPA PE=1 SV=2	0.00	0.00	1.12	1.52	1.92	0.73	0.00	0.00	0.00	2.09	0.00	0.00	0.44	1.01	6.55	1.79	0.00	0.00	2.38	0.65	0.00	3.12	0.00	0.00	0.61	1.33	>0.999999999999999	0.5
P27816	Microtubule-associated protein 4 OS=Homo sapiens GN=MAP4 PE=1 SV=3	1.95	1.15	0.34	0.00	0.96	0.73	0.00	0.79	0.00	0.00	0.95	0.50	0.00	0.00	0.00	0.00	0.00	0.00	0.00	0.00	0.00	0.00	0.00	0.17	0.61	0.01	>0.999999999999999	43.3
Q8NHQ9	ATP-dependent RNA helicase DDX55 OS=Homo sapiens GN=DDX55 PE=1 SV=3	1.22	1.15	0.00	0.00	0.96	0.73	0.84	0.79	0.20	0.52	0.95	0.00	0.44	1.01	0.56	0.00	0.94	0.00	0.79	0.65	0.15	1.04	0.77	0.28	0.61	0.55	>0.999999999999999	1.1
O95373	Importin-7 OS=Homo sapiens GN=IPO7 PE=1 SV=1	1.22	0.69	0.34	0.45	0.00	2.47	0.00	0.48	0.44	0.00	0.95	0.30	0.00	0.00	0.00	0.21	0.00	0.77	0.00	1.04	0.09	0.00	0.00	0.28	0.61	0.20	>0.999999999999999	3.1
Q92688	Acidic leucine-rich nuclear phosphoprotein 32 family member B OS=Homo sapiens GN=ANP	0.00	0.00	0.00	0.00	0.00	0.00	0.00	6.18	0.00	1.15	0.00	0.00	3.42	0.00	0.00	0.00	0.00	0.00	0.00	3.11	0.00	0.00	0.00	0.00	0.61	0.54	>0.999999999999999	1.1
P63279	SUMO-conjugating enzyme UBC9 OS=Homo sapiens GN=UBE2I PE=1 SV=1	4.64	0.00	0.00	0.00	0.00	0.00	0.00	0.00	0.75	0.00	0.00	1.89	1.67	0.00	0.00	0.00	0.00	0.00	0.00	0.00	0.00	0.00	0.00	1.08	0.61	0.23	>0.999999999999999	2.7
P22894	Neutrophil collagenase OS=Homo sapiens GN=MMP8 PE=1 SV=1	0.00	1.38	0.00	1.97	1.15	0.00	1.01	0.00	0.00	0.63	1.14	0.00	1.14	1.21	0.00	0.00	0.00	0.00	0.00	0.00	0.00	5.61	4.14	0.00	0.61	1.01	>0.999999999999999	0.6
Q8N1Q1	Carbonic anhydrase 13 OS=Homo sapiens GN=CA13 PE=1 SV=1	0.00	0.00	2.69	0.00	0.00	3.49	0.00	0.00	0.00	0.00	0.00	1.10	0.00	4.83	0.00	1.66	0.00	0.00	0.00	4.92	0.34	0.00	0.00	0.62	0.61	1.03	>0.999999999999999	0.6
Q15286	Ras-related protein Rab-35 OS=Homo sapiens GN=RAB35 PE=1 SV=1	0.00	0.00	0.00	0.00	0.00	7.28	0.00	0.00	0.00	0.00	0.00	0.00	0.00	0.00	0.00	0.00	0.00	0.00	0.00	0.00	0.00	0.00	0.00	0.00	0.61	0.00	>0.999999999999999	#DIV/0!
Q13098	COP9 signalosome complex subunit 1 OS=Homo sapiens GN=GPS1 PE=1 SV=4	1.47	1.38	1.34	0.00	0.00	0.00	0.00	0.95	0.24	0.00	0.00	1.89	0.00	1.21	1.33	0.00	0.00	0.00	0.00	0.00	0.37	0.00	0.00	0.68	0.61	0.30	>0.999999999999999	2.0
P50851	Lipopolysaccharide-responsive and beige-like anchor protein OS=Homo sapiens GN=LRBA	1.47	0.92	0.78	0.30	0.19	1.75	0.34	0.32	0.04	0.00	0.76	0.40	0.18	0.20	0.11	0.14	0.00	0.15	0.00	0.39	0.03	0.21	0.00	0.06	0.61	0.12	>0.999999999999999	5.0
Q15063	Periostin OS=Homo sapiens GN=POSTN PE=1 SV=2	0.00	0.69	2.13	0.00	3.64	0.00	0.51	0.00	0.28	0.00	0.00	0.00	0.96	0.00	1.67	1.03	3.58	0.00	8.08	0.00	0.00	0.62	0.00	0.17	0.60	1.34	>0.999999999999999	0.4
P78527	DNA-dependent protein kinase catalytic subunit OS=Homo sapiens GN=PRKDC PE=1 SV=3	1.47	0.92	0.34	0.15	0.19	1.75	0.17	0.16	0.00	0.00	1.70	0.40	0.09	0.20	0.11	0.00	0.00	0.15	0.00	0.13	0.00	0.21	0.15	0.11	0.60	0.10	>0.999999999999999	6.3
Q86XW9	Thioredoxin domain-containing protein 6 OS=Homo sapiens GN=NME9 PE=1 SV=1	0.00	2.07	0.00	0.00	0.00	1.31	1.52	1.43	0.00	0.00	0.00	0.90	0.00	0.00	0.00	0.00	0.00	0.00	0.00	1.17	0.00	0.00	0.00	0.00	0.60	0.10	>0.999999999999999	6.2
O43633	Charged multivesicular body protein 2a OS=Homo sapiens GN=CHMP2A PE=1 SV=1	0.00	0.00	1.46	0.00	0.00	1.89	0.00	2.06	0.52	0.00	0.00	1.29	0.00	2.62	1.44	0.00	0.00	0.00	0.00	1.68	0.00	2.70	0.00	0.74	0.60	0.77	>0.999999999999999	0.8
O14929	Histone acetyltransferase type B catalytic subunit OS=Homo sapiens GN=HAT1 PE=1 SV=1	3.42	0.00	0.00	0.00	0.00	1.02	0.00	1.11	0.28	0.00	0.00	1.39	0.00	0.00	0.00	0.00	0.00	0.00	0.00	0.00	0.00	0.00	0.00	0.00	0.60	0.00	>0.999999999999999	#DIV/0!
P21333	Filamin-A OS=Homo sapiens GN=FLNA PE=1 SV=4	0.00	2.30	0.00	1.06	0.00	0.00	0.34	0.00	0.00	3.03	0.38	0.10	0.00	0.20	0.00	0.00	0.00	0.00	0.00	0.13	0.00	3.53	1.84	0.00	0.60	0.48	>0.999999999999999	1.3
Q9NQG5	Regulation of nuclear pre-mRNA domain-containing protein 1B OS=Homo sapiens GN=RPR	4.64	0.00	0.00	0.00	0.00	1.31	0.00	0.00	0.36	0.00	0.00	0.90	0.00	0.00	0.00	0.00	0.00	0.00	0.00	0.00	0.00	0.00	0.00	0.00	0.60	0.00	>0.999999999999999	#DIV/0!
Q16531	DNA damage-binding protein 1 OS=Homo sapiens GN=DDB1 PE=1 SV=1	1.22	1.15	0.34	0.00	0.00	2.33	0.00	0.00	0.12	0.00	0.95	1.10	0.00	0.00	0.00	0.00	0.00	0.00	0.00	0.65	0.00	0.00	0.46	0.00	0.60	0.09	>0.999999999999999	6.5
P63220	40S ribosomal protein S21 OS=Homo sapiens GN=RPS21 PE=1 SV=1	0.00	0.00	0.00	0.00	0.00	0.00	0.00	0.00	0.00	0.00	7.20	0.00	0.00	0.00	0.00	0.00	0.00	0.00	0.00	0.00	0.00	0.00	0.00	0.00	0.60	0.00	>0.999999999999999	#DIV/0!
Q4G0F5	Vacuolar protein sorting-associated protein 26B OS=Homo sapiens GN=VPS26B PE=1 SV=	1.95	0.00	0.00	0.00	1.73	0.00	0.00	0.00	0.00	0.00	1.70	1.79	0.00	0.00	0.00	0.00	0.00	0.00	0.00	0.00	0.00	0.00	0.00	0.00	0.60	0.00	>0.999999999999999	#DIV/0!
Q16658	Fascin OS=Homo sapiens GN=FSCN1 PE=1 SV=3	1.47	0.00	1.34	1.82	0.00	0.87	0.00	0.00	0.48	0.00	1.14	0.00	0.00	5.24	0.67	1.31	1.13	0.92	1.90	0.78	0.80	2.49	0.00	0.68	0.59	1.33	>0.999999999999999	0.4
Q15126	Phosphomevalonate kinase OS=Homo sapiens GN=PMVK PE=1 SV=3	0.00	0.00	0.00	0.00	0.00	2.18	0.00	0.00	0.59	0.00	2.84	1.49	0.00	0.00	0.00	0.00	0.00	0.00	0.00	0.00	0.00	0.00	0.00	0.85	0.59	0.07	>0.999999999999999	8.4
Q8IW45	ATP-dependent (S)-NAD(P)H-hydrate dehydratase OS=Homo sapiens GN=CARKD PE=1 S	2.20	0.00	0.00	0.00	0.00	1.31	0.00	0.00	0.00	0.00	1.70	1.89	0.00	0.00	0.00	0.00	0.00	0.00	0.00	1.17	0.00	0.00	0.00	1.08	0.59	0.19	>0.999999999999999	3.2
P01780	Ig heavy chain V-III region JON OS=Homo sapiens PE=1 SV=1	0.00	0.00	0.00	0.00	0.00	0.00	0.00	4.28	0.00	2.82	0.00	0.00	2.37	0.00	3.00	0.00	5.09	0.00	0.00	0.00	0.00	0.00	4.14	0.00	0.59	1.22	>0.999999999999999	0.5
P63098	Calcineurin subunit B type 1 OS=Homo sapiens GN=PPP3R1 PE=1 SV=2	0.00	3.92	0.00	0.00	0.00	2.47	0.00	0.00	0.67	0.00	0.00	0.00	0.00	0.00	0.00	0.00	0.00	0.00	0.00	0.00	0.00	0.00	0.00	0.00	0.59	0.00	>0.999999999999999	#DIV/0!
Q53T59	HCLS1-binding protein 3 OS=Homo sapiens GN=HS1BP3 PE=1 SV=1	3.91	0.00	0.00	0.00	0.00	2.33	0.00	0.00	0.00	0.00	0.00	0.80	0.00	0.00	0.00	0.00	0.00	0.00	0.00	0.00	0.00	0.00	0.00	0.00	0.59	0.00	>0.999999999999999	#DIV/0!
P61966	AP-1 complex subunit sigma-1A OS=Homo sapiens GN=AP1S1 PE=1 SV=1	4.40	0.00	0.00	0.00	0.00	2.62	0.00	0.00	0.00	0.00	0.00	0.00	0.00	0.00	0.00	0.00	0.00	0.00	0.00	0.00	0.00	0.00	0.00	0.00	0.58	0.00	>0.999999999999999	#DIV/0!
Q1KMD3	Heterogeneous nuclear ribonucleoprotein U-like protein 2 OS=Homo sapiens GN=HNRNPUL	1.95	1.84	0.00	0.00	0.00	1.75	0.00	0.63	0.00	0.83	0.00	0.00	0.00	0.00	0.00	0.00	0.00	1.23	0.00	0.00	0.00	0.00	0.61	0.23	0.58	0.17	>0.999999999999999	3.4
Q9NZL4	Hsp70-binding protein 1 OS=Homo sapiens GN=HSPBP1 PE=1 SV=1	1.95	0.00	0.00	0.00	1.53	1.16	0.00	0.00	0.00	0.00	1.51	0.80	0.00	0.00	0.00	0.00	0.00	0.00	0.00	0.00	0.00	0.00	0.00	0.00	0.58	0.00	>0.999999999999999	#DIV/0!
Q9UHB6	LIM domain and actin-binding protein 1 OS=Homo sapiens GN=LIMA1 PE=1 SV=1	0.98	0.92	0.90	0.00	0.77	1.16	0.00	1.27	0.16	0.00	0.00	0.80	0.35	0.81	0.44	0.55	0.00	0.61	0.00	1.04	0.00	0.00	0.00	0.23	0.58	0.34	>0.999999999999999	1.7
Q9P0L0	Vesicle-associated membrane protein-associated protein A OS=Homo sapiens GN=VAPA P	2.93	0.00	0.00	0.00	0.00	1.75	0.00	0.00	0.00	0.00	2.27	0.00	0.00	2.42	0.00	0.00	0.00	0.00	0.00	1.55	0.00	0.00	0.00	0.00	0.58	0.33	>0.999999999999999	1.7
O60784	Target of Myb protein 1 OS=Homo sapiens GN=TOM1 PE=1 SV=2	1.47	1.38	0.67	0.00	0.00	1.89	0.00	0.00	0.24	0.00	0.00	1.29	0.53	0.00	0.67	0.00	0.00	0.00	0.00	0.00	0.00	1.25	0.00	0.00	0.58	0.20	>0.999999999999999	2.8
Q9BYT3	Serine/threonine-protein kinase 33 OS=Homo sapiens GN=STK33 PE=1 SV=1	1.47	1.38	0.00	0.00	1.15	1.75	0.00	0.00	0.00	0.00	0.00	1.20	0.00	0.00	0.00	0.00	0.00	0.00	0.00	0.00	0.00	0.00	0.00	0.00	0.58	0.00	>0.999999999999999	#DIV/0!
Q9H7X7	Rab-like protein 5 OS=Homo sapiens GN=RABL5 PE=2 SV=1	3.91	0.00	0.00	0.00	0.00	0.00	0.00	0.00	0.00	0.00	3.03	0.00	0.00	0.00	0.00	0.00	0.00	0.00	0.00	0.00	0.00	0.00	0.00	0.00	0.58	0.00	>0.999999999999999	#DIV/0!
Q9GZZ1	N-alpha-acetyltransferase 50 OS=Homo sapiens GN=NAA50 PE=1 SV=1	0.00	0.00	0.00	0.00	0.00	0.00	0.00	0.00	0.00	0.00	3.22	3.69	0.00	3.42	0.00	0.00	0.00	0.00	0.00	0.00	0.00	0.00	0.00	0.97	0.58	0.37	>0.999999999999999	1.6
Q13492	Phosphatidylinositol-binding clathrin assembly protein OS=Homo sapiens GN=PICALM PE=	0.00	1.15	0.56	0.00	0.00	2.18	0.00	0.79	0.36	0.00	0.95	0.90	0.44	1.01	0.00	0.00	0.00	0.00	0.00	1.17	0.00	1.04	0.77	0.28	0.57	0.39	>0.999999999999999	1.5
P14314	Glucosidase 2 subunit beta OS=Homo sapiens GN=PRKCSH PE=1 SV=2	0.00	0.00	0.00	0.00	0.00	1.60	0.00	4.76	0.00	0.52	0.00	0.00	0.00	1.01	0.00	0.00	0.00	0.00	0.00	0.65	0.00	0.00	0.00	0.00	0.57	0.14	>0.999999999999999	4.2
Q9BRL6	Serine/arginine-rich splicing factor 8 OS=Homo sapiens GN=SRSF8 PE=1 SV=1	2.44	0.00	1.23	0.00	0.00	3.20	0.00	0.00	0.00	0.00	0.00	0.00	0.00	0.00	0.00	0.00	0.00	0.00	0.00	0.00	0.00	0.00	0.00	0.00	0.57	0.00	>0.999999999999999	#DIV/0!
P01702	Ig lambda chain V-I region NIG-64 OS=Homo sapiens PE=1 SV=1	0.00	0.00	0.00	0.00	0.00	0.00	0.00	0.00	1.19	0.00	5.68	0.00	0.00	0.00	3.33	2.07	0.00	0.00	0.00	0.00	0.00	0.00	4.60	1.70	0.57	0.98	>0.999999999999999	0.6
P09493	Tropomyosin alpha-1 chain OS=Homo sapiens GN=TPM1 PE=1 SV=2	0.00	0.00	0.00	0.00	0.00	6.84	0.00	0.00	0.00	0.00	0.00	0.00	0.00	0.00	0.00	0.00	0.00	0.00	0.00	0.00	0.00	0.00	0.00	0.00	0.57	0.00	>0.999999999999999	#DIV/0!
Q13576	Ras GTPase-activating-like protein IQGAP2 OS=Homo sapiens GN=IQGAP2 PE=1 SV=4	1.47	0.00	0.67	0.00	0.00	2.47	0.00	0.00	0.00	0.00	1.33	0.90	0.00	0.00	0.22	0.00	0.00	0.00	0.00	0.52	0.00	0.00	0.00	0.00	0.57	0.06	>0.999999999999999	9.2
Q99627	COP9 signalosome complex subunit 8 OS=Homo sapiens GN=COPS8 PE=1 SV=1	0.00	3.23	1.57	0.00	0.00	2.04	0.00	0.00	0.00	0.00	0.00	0.00	0.00	0.00	0.00	0.00	0.00	0.00	0.00	0.00	0.00	0.00	0.00	0.00	0.57	0.00	>0.999999999999999	#DIV/0!
O00505	Importin subunit alpha-3 OS=Homo sapiens GN=KPNA3 PE=1 SV=2	2.93	0.00	0.67	0.91	0.00	0.87	0.00	0.00	0.24	0.00	0.00	1.20	0.00	0.00	0.00	0.00	0.00	0.00	0.00	0.00	0.00	0.00	0.00	0.00	0.57	0.00	>0.999999999999999	#DIV/0!
P34096	Ribonuclease 4 OS=Homo sapiens GN=RNASE4 PE=1 SV=3	0.00	0.00	0.00	0.00	0.00	0.00	0.00	6.82	0.00	0.00	0.00	0.00	0.00	0.00	0.00	1.38	0.00	0.00	0.00	0.00	0.00	0.00	0.00	0.00	0.57	0.11	>0.999999999999999	4.9
P50502	Hsc70-interacting protein OS=Homo sapiens GN=ST13 PE=1 SV=2	0.00	0.00	0.00	0.00	0.00	0.00	0.00	0.00	0.00	0.00	6.82	0.00	0.00	0.00	0.00	0.00	0.00	0.00	0.00	0.00	0.00	0.00	0.00	0.00	0.57	0.00	>0.999999999999999	#DIV/0!
P30504	HLA class I histocompatibility antigen, Cw-4 alpha chain OS=Homo sapiens GN=HLA-C PE=	0.00	0.00	0.00	0.00	0.00	0.00	0.00	0.00	0.00	0.00	6.82	0.00	0.00	0.00	0.00	0.00	0.00	0.00	0.00	0.00	0.00	0.00	0.00	0.00	0.57	0.00	>0.999999999999999	#DIV/0!
Q9NQC8	Intraflagellar transport protein 46 homolog OS=Homo sapiens GN=IFT46 PE=2 SV=1	2.44	0.00	0.00	0.00	0.00	1.46	0.00	0.00	0.00	0.00	1.89	1.00	0.00	0.00	0.00	0.00	0.00	0.00	0.00	0.00	0.00	0.00	0.00	0.00	0.57	0.00	>0.999999999999999	#DIV/0!
P58499	Protein FAM3B OS=Homo sapiens GN=FAM3B PE=1 SV=2	0.00	0.00	0.00	0.00	2.49	0.00	0.00	4.28	0.00	0.00	0.00	0.00	0.00	0.00	1.44	0.00	5.09	0.00	0.00	0.00	0.00	0.00	0.00	0.74	0.56	0.61	>0.999999999999999	0.9
P21399	Cytoplasmic aconitate hydratase OS=Homo sapiens GN=ACO1 PE=1 SV=3	2.44	0.69	0.34	0.00	0.00	1.46	0.00	0.00	0.28	0.00	0.57	1.00	0.00	2.01	0.00	0.00	0.00	0.46	0.00	0.39	0.00	0.00	0.00	0.17	0.56	0.25	>0.999999999999999	2.2
Q7L5N1	COP9 signalosome complex subunit 6 OS=Homo sapiens GN=COPS6 PE=1 SV=1	2.20	0.00	1.01	0.00	0.00	1.31	0.00	0.00	0.36	0.00	0.00	1.89	0.00	0.00	0.00	0.00	0.00	0.00	0.00	1.17	0.28	0.00	0.00	0.51	0.56	0.16	>0.999999999999999	3.5
Q96CD0	F-box/LRR-repeat protein 8 OS=Homo sapiens GN=FBXL8 PE=1 SV=1	1.95	0.00	0.00	0.00	1.53	2.47	0.00	0.00	0.00	0.00	0.00	0.80	0.00	0.00	0.00	0.00	0.00	0.00	0.00	0.00	0.00	0.00	0.00	0.00	0.56	0.00	>0.999999999999999	#DIV/0!
A6NKL6	Transmembrane protein 200C OS=Homo sapiens GN=TMEM200C PE=2 SV=2	0.00	1.15	0.00	0.76	0.96	0.73	0.84	0.79	0.00	0.52	0.95	0.00	0.44	1.01	0.00	0.00	0.94	0.00	0.79	0.00	0.15	1.04	0.00	0.28	0.56	0.39	>0.999999999999999	1.4
O15145	Actin-related protein 2/3 complex subunit 3 OS=Homo sapiens GN=ARPC3 PE=1 SV=3	0.00	0.00	0.00	0.00	0.00	2.33	2.70	0.00	0.00	1.67	0.00	0.00	0.00	0.00	0.00	0.00	0.00	0.00	2.45	0.00	0.00	3.32	2.45	0.00	0.56	0.69	>0.999999999999999	0.8
P57076	Uncharacterized protein C21orf59 OS=Homo sapiens GN=C21orf59 PE=1 SV=1	0.00	0.00	0.00	0.00	0.00	4.80	0.00	0.00	0.00	0.00	1.89	0.00	0.00	0.00	0.00	0.00	0.00	0.00	0.00	0.00	0.00	0.00	0.00	0.00	0.56	0.00	>0.999999999999999	#DIV/0!
P30084	Enoyl-CoA hydratase, mitochondrial OS=Homo sapiens GN=ECHS1 PE=1 SV=4	0.00	0.00	0.00	0.00	0.00	3.20	0.00	3.49	0.00	0.00	0.00	0.00	0.00	2.01	2.44	0.00	0.00	0.00	1.59	1.30	0.00	0.00	1.53	0.00	0.56	0.74	>0.999999999999999	0.8
Q8WWI1	LIM domain only protein 7 OS=Homo sapiens GN=LMO7 PE=1 SV=3	1.22	1.15	0.78	0.30	0.00	1.60	0.51	0.32	0.28	0.21	0.00	0.30	0.00	1.01	0.56	0.21	0.00	0.31	0.00	0.91	0.15	0.00	0.00	0.40	0.56	0.29	>0.999999999999999	1.9
Q9NVD7	Alpha-parvin OS=Homo sapiens GN=PARVA PE=1 SV=1	3.91	0.00	0.00	0.00	0.00	1.16	0.00	0.00	0.00	0.00	0.00	1.59	0.00	1.61	0.00	0.00	0.00	0.00	0.00	1.04	0.00	0.00	0.00	0.00	0.56	0.22	>0.999999999999999	2.5
O75396	Vesicle-trafficking protein SEC22b OS=Homo sapiens GN=SEC22B PE=1 SV=4	0.00	0.00	0.00	0.00	0.00	2.04	0.00	4.60	0.00	0.00	0.00	0.00	0.00	0.00	0.00	0.00	0.00	0.00	0.00	0.00	0.00	0.00	0.00	0.00	0.55	0.00	>0.999999999999999	#DIV/0!
Q7L576	Cytoplasmic FMR1-interacting protein 1 OS=Homo sapiens GN=CYFIP1 PE=1 SV=1	1.22	1.15	0.22	0.30	0.00	2.04	0.34	0.00	0.48	0.00	0.38	0.50	0.18	0.00	0.22	0.00	0.00	0.00	0.32	0.26	0.06	0.00	0.00	0.28	0.55	0.11	>0.999999999999999	5.0
P31513	Dimethylaniline monooxygenase [N-oxide-forming] 3 OS=Homo sapiens GN=FMO3 PE=1 S\	1.22	0.00	0.00	0.00	0.00	1.60	0.00	3.80	0.00	0.00	0.00	0.00	0.00	0.00	0.00	0.00	0.00	0.00	0.00	0.00	0.00	0.00	0.00	0.00	0.55	0.00	>0.999999999999999	#DIV/0!
O75131	Copine-3 OS=Homo sapiens GN=CPNE3 PE=1 SV=1	2.69	0.00	0.00	0.76	0.00	0.73	0.00	0.79	0.00	1.15	0.00	0.50	0.00	0.00	0.00	0.00	0.00	0.00	0.00	0.65	0.00	0.00	0.00	0.00	0.55	0.05	>0.999999999999999	10.2
Q9H6S3	Epidermal growth factor receptor kinase substrate 8-like protein 2 OS=Homo sapiens GN=EI	1.95	0.92	0.45	0.00	0.00	1.89	0.00	0.63	0.00	0.00	0.76	0.00	0.00	0.00	0.44	0.00	0.00	0.00	0.00	1.04	0.00	0.00	0.00	0.00	0.55	0.12	>0.999999999999999	4.5
P08579	U2 small nuclear ribonucleoprotein B'' OS=Homo sapiens GN=SNRPB2 PE=1 SV=1	3.18	0.00	1.46	1.97	0.00	0.00	0.00	0.00	0.00	0.00	0.00	0.00	0.00	0.00	0.00	0.00	0.00	0.00	0.00	1.68	0.00	0.00	0.00	0.00	0.55	0.14	>0.999999999999999	3.9
Q14166	Tubulin--tyrosine ligase-like protein 12 OS=Homo sapiens GN=TTLL12 PE=1 SV=2	2.20	0.00	0.45	0.00	0.00	2.77	0.00	0.63	0.16	0.00	0.00	0.40	0.35	1.81	0.44	0.97	0.00	2.15	0.00	1.17	0.00	0.00	0.00	0.00	0.55	0.57	>0.999999999999999	1.0
Q9BVJ7	Dual specificity protein phosphatase 23 OS=Homo sapiens GN=DUSP23 PE=1 SV=1	0.00	4.61	0.00	0.00	0.00	0.00	0.00	0.00	0.00	0.00	0.00	1.99	0.00	4.03	0.00	0.00	0.00	0.00	0.00	0.00	0.00	0.00	0.00	1.14	0.55	0.43	>0.999999999999999	1.3
Q9GZT8	NIF3-like protein 1 OS=Homo sapiens GN=NIF3L1 PE=1 SV=2	1.95	0.00	0.00	0.00	0.00	2.33	0.00	0.00	0.00	0.00	1.51	0.80	0.00	1.61	0.89	0.55	0.00	0.00	0.00	1.04	0.00	0.00	0.00	0.00	0.55	0.34	>0.999999999999999	1.6
O00592	Podocalyxin OS=Homo sapiens GN=PODXL PE=1 SV=2	1.47	1.38	0.00	0.00	1.15	0.00	1.01	0.95	0.00	0.63	0.00	0.00	0.53	0.00	0.00	0.00	1.13	0.00	0.00	0.00	0.00	1.25	0.92	0.00	0.55	0.32	>0.999999999999999	1.7
P01613	Ig kappa chain V-I region Ni OS=Homo sapiens PE=1 SV=1	0.00	0.00	0.00	0.00	0.00	0.00	0.00	0.00	0.00	6.57	0.00	0.00	0.00	0.00	0.00	0.00	0.00	0.00	0.00	0.00	0.00	0.00	0.00	0.00	0.55	0.00	>0.999999999999999	#DIV/0!
P15328	Folate receptor alpha OS=Homo sapiens GN=FOLR1 PE=1 SV=3	0.00	2.54	0.00	0.00	0.00	0.00	1.86	1.74	0.44	0.00	0.00	0.00	0.00	0.00	0.00	0.00	2.07	0.00	0.00	0.00	0.34	0.00	0.00	0.00	0.55	0.20	>0.999999999999999	2.7
Q9P0R6	GSK3-beta interaction protein OS=Homo sapiens GN=GSKIP PE=1 SV=2	0.00	0.00	0.00	0.00	0.00	0.00	0.00	3.49	0.87	0.00	0.00	2.19	0.00	0.00	0.00	0.00	0.00	0.00	0.00	0.00	0.00	0.00	0.00	0.00	0.55	0.00	>0.999999999999999	#DIV/0!
O75891	Cytosolic 10-formyltetrahydrofolate dehydrogenase OS=Homo sapiens GN=ALDH1L1 PE=1	1.71	2.30	0.78	0.00	0.00	1.02	0.00	0.00	0.71	0.00	0.00	0.00	0.00	3.63	0.00	0.00	0.00	1.07	0.00	2.72	0.00	0.00	1.07	0.40	0.54	0.74	>0.999999999999999	0.7
Q14657	L antigen family member 3 OS=Homo sapiens GN=LAGE3 PE=1 SV=2	5.62	0.00	0.00	0.00	0.00	0.00	0.00	0.00	0.91	0.00	0.00	0.00	0.00	0.00	0.00	0.00	0.00	0.00	0.00	0.00	0.00	0.00	0.00	0.00	0.54	0.00	>0.999999999999999	#DIV/0!
Q9UI12	V-type proton ATPase subunit H OS=Homo sapiens GN=ATP6V1H PE=1 SV=1	0.00	0.00	0.67	0.00	0.00	2.77	0.00	0.00	0.75	0.00	1.14	1.20	0.00	1.21	0.00	0.83	0.00	0.92	0.00	1.55	0.00	1.25	0.92	0.34	0.54	0.58	>0.999999999999999	0.9
Q9BQG2	Peroxisomal NADH pyrophosphatase NUDT12 OS=Homo sapiens GN=NUDT12 PE=1 SV=1	3.18	0.00	0.67	0.91	1.15	0.00	0.00	0.00	0.00	0.00	0.00	0.60	0.00	0.00	0.00	0.41	0.00	0.00	0.00	0.78	0.00	0.00	0.00	0.00	0.54	0.10	>0.999999999999999	5.5
Q9UMS4	Pre-mRNA-processing factor 19 OS=Homo sapiens GN=PRPF19 PE=1 SV=1	1.47	0.00	0.00	0.00	0.00	2.77	0.00	0.00	0.00	0.00	2.27	0.00	0.00	0.00	0.00	0.00	0.00	0.00	0.00	0.78	0.00	0.00	0.00	0.00	0.54	0.06	>0.999999999999999	8.4
Q9UBB5	Methyl-CpG-binding domain protein 2 OS=Homo sapiens GN=MBD2 PE=1 SV=1	1.95	0.00	0.90	1.21	0.00	1.16	0.00	1.27	0.00	0.00	0.00	0.00	0.70	1.61	0.00	0.55	0.00	0.00	0.00	0.00	0.00	0.00	0.00	0.00	0.54	0.24	>0.999999999999999	2.3
P17213	Bactericidal permeability-increasing protein OS=Homo sapiens GN=BPI PE=1 SV=4	0.00	3.00	0.00	0.91	0.00	0.00	1.01	0.95	0.00	0.63	0.00	0.00	0.53	1.21	0.00	0.00	0.00	0.92	0.00	0.00	0.00	1.25	0.92	0.00	0.54	0.40	>0.999999999999999	1.3
Q9NVE7	Pantothenate kinase 4 OS=Homo sapiens GN=PANK4 PE=1 SV=1	2.93	0.92	0.00	0.00	0.00	1.16	0.67	0.00	0.00	0.00	0.00	0.80	0.00	2.42	0.44	0.00	0.00	0.00	0.00	0.52	0.00	0.83	0.00	0.00	0.54	0.35	>0.999999999999999	1.5
Q9UDY4	DnaJ homolog subfamily B member 4 OS=Homo sapiens GN=DNAJB4 PE=1 SV=1	4.40	2.07	0.00	0.00	0.00	0.00	0.00	0.00	0.00	0.00	0.00	0.00	0.00	0.00	0.00	0.62	0.00	0.00	0.00	0.00	0.00	0.00	0.00	0.00	0.54	0.05	>0.999999999999999	10.4
O75533	Splicing factor 3B subunit 1 OS=Homo sapiens GN=SF3B1 PE=1 SV=3	1.71	1.15	0.56	0.00	0.38	0.29	0.34	0.00	0.00	0.00	1.33	0.70	0.00	1.01	0.00	0.00	0.00	0.00	0.00	0.91	0.00	0.00	0.00	0.11	0.54	0.17	>0.999999999999999	3.2
Q99798	Aconitate hydratase, mitochondrial OS=Homo sapiens GN=ACO2 PE=1 SV=2	0.00	0.00	0.45	0.00	0.00	2.33	0.00	3.33	0.32	0.00	0.00	0.00	0.70	0.81	0.44	0.28	0.00	0.61	0.00	0.52	0.00	0.00	0.00	0.00	0.54	0.28	>0.999999999999999	1.9
O43809	Cleavage and polyadenylation specificity factor subunit 5 OS=Homo sapiens GN=NUDT21 F	0.00	0.00	0.00	0.00	0.00	0.00	0.00	0.00	0.00	0.00	5.11	1.29	0.00	0.00	0.00	0.00	0.00	0.00	0.00	0.00	0.00	0.00	1.99	0.00	0.53	0.17	>0.999999999999999	3.2
P61011	Signal recognition particle 54 kDa protein OS=Homo sapiens GN=SRP54 PE=1 SV=1	1.47	1.38	0.00	0.00	0.00	0.87	0.00	0.95	0.00	0.00	1.14	0.60	0.00	1.21	0.00	0.00	0.00	0.00	0.00	0.78	0.00	0.00	0.00	0.00	0.53	0.17	>0.999999999999999	3.2
Q9NS86	LanC-like protein 2 OS=Homo sapiens GN=LANCL2 PE=1 SV=1	3.18	0.00	0.00	0.00	0.00	1.89	0.00	0.00	0.00	0.00	0.00	1.29	0.00	0.00	0.00	0.00	0.00	0.00	0.00	0.00	0.00	0.00	0.00	0.00	0.53	0.00	>0.999999999999999	#DIV/0!
P09661	U2 small nuclear ribonucleoprotein A' OS=Homo sapiens GN=SNRPA1 PE=1 SV=2	2.93	0.00	0.00	0.00	0.00	1.75	0.00	0.00	0.48	0.00	0.00	1.20	0.00	0.00	0.00	0.00	0.00	0.00	0.00	0.00	0.00	0.00	0.00	0.00	0.53	0.00	>0.999999999999999	#DIV/0!
Q8TDR0	TRAF3-interacting protein 1 OS=Homo sapiens GN=TRAF3IP1 PE=1 SV=1	0.98	1.84	0.45	0.00	0.77	0.00	0.00	0.00	0.00	0.00	1.51	0.80	0.00	0.00	0.00	0.00	0.00	0.00	0.00	0.00	0.00	0.00	0.00	0.00	0.53	0.00	>0.999999999999999	#DIV/0!
P05543	Thyroxine-binding globulin OS=Homo sapiens GN=SERPINA7 PE=1 SV=2	1.71	0.00	0.78	0.00	2.88	0.00	0.00	0.00	0.28	0.00	0.00	0.70	0.61	0.00	0.00	0.48	0.00	0.00	0.00	0.00	0.00	0.00	0.00	0.00	0.53	0.09	>0.999999999999999	5.8
O43847	Nardilysin OS=Homo sapiens GN=NRD1 PE=1 SV=2	1.95	0.46	0.56	0.00	0.38	2.33	0.00	0.00	0.08	0.00	0.38	0.20	0.18	1.01	0.00	0.55	0.00	0.31	0.00	0.65	0.06	0.42	0.00	0.28	0.53	0.29	>0.999999999999999	1.8
Q9UIV8	Serpin B13 OS=Homo sapiens GN=SERPINB13 PE=2 SV=2	0.00	0.00	1.68	0.00	0.00	1.02	2.53	1.11	0.00	0.00	0.00	0.00	0.00	3.02	0.78	0.48	1.32	2.30	3.80	1.94	0.74	1.45	0.00	0.40	0.53	1.35	>0.999999999999999	0.4
P51570	Galactokinase OS=Homo sapiens GN=GALK1 PE=1 SV=1	1.95	0.00	0.90	0.00	0.00	1.16	0.00	0.00	0.00	0.00	1.51	0.80	0.00	1.61	0.00	0.55	0.00	0.00	0.00	1.04	0.00	0.00	0.00	0.00	0.53	0.27	>0.999999999999999	2.0
P00748	Coagulation factor XII OS=Homo sapiens GN=F12 PE=1 SV=3	0.00	0.00	0.00	0.76	2.88	0.00	1.69	0.00	0.00	0.00	0.00	1.00	0.00	0.00	1.11	0.69	2.83	1.53	5.07	0.65	0.15	2.08	0.00	0.28	0.53	1.20	>0.999999999999999	0.4
P18428	Lipopolysaccharide-binding protein OS=Homo sapiens GN=LBP PE=1 SV=3	0.00	0.00	0.67	0.91	2.49	0.00	1.01	0.00	0.00	0.63	0.00	0.60	0.00	0.00	0.67	0.00	1.13	0.00	0.95	0.00	0.00	1.25	0.00	0.00	0.53	0.33	>0.999999999999999	1.6
O94874	E3 UFM1-protein ligase 1 OS=Homo sapiens GN=UFL1 PE=1 SV=2	0.00	0.92	0.00	0.61	0.00	3.49	0.00	1.11	0.16	0.00	0.00	0.00	0.00	0.00	0.00	0.00	0.00	0.00	0.00	0.52	0.00	0.00	0.00	0.00	0.52	0.04	>0.999999999999999	12.1
Q92616	Translational activator GCN1 OS=Homo sapiens GN=GCN1L1 PE=1 SV=6	2.20	0.92	0.34	0.00	0.00	1.46	0.17	0.00	0.04	0.00	0.57	0.60	0.00	0.60	0.00	0.00	0.00	0.00	0.00	0.52	0.06	0.00	0.15	0.11	0.52	0.12	>0.999999999999999	4.3
P29144	Tripeptidyl-peptidase 2 OS=Homo sapiens GN=TPP2 PE=1 SV=4	1.22	1.15	0.56	0.30	0.38	1.75	0.34	0.00	0.00	0.00	0.38	0.20	0.18	0.00	0.00	0.14	0.00	0.00	0.00	0.91	0.00	0.42	0.00	0.00	0.52	0.14	>0.999999999999999	3.8
Q11128	Alpha-(1,3)-fucosyltransferase OS=Homo sapiens GN=FUT5 PE=2 SV=1	0.00	1.84	0.90	1.21	0.00	2.33	0.00	0.00	0.00	0.00	0.00	0.00	0.00	1.61	0.00	0.55	1.51	1.23	0.00	0.00	0.00	0.00	0.00	0.00	0.52	0.41	>0.999999999999999	1.3
P07996	Thrombospondin-1 OS=Homo sapiens GN=THBS1 PE=1 SV=2	0.49	0.46	0.00	0.00	0.96	0.73	0.84	1.27	0.20	0.83	0.00	0.50	0.88	0.00	0.00	0.34	5.09	0.77	1.27	0.65	0.00	0.42	0.31	0.00	0.52	0.81	>0.999999999999999	0.6
P14678	Small nuclear ribonucleoprotein-associated proteins B and B' OS=Homo sapiens GN=SNRP	0.00	0.00	1.57	0.00	0.00	2.04	0.00	0.00	0.00	0.00	2.65	0.00	0.00	0.00	0.00	0.00	0.00	0.00	0.00	0.00	0.00	0.00	0.00	0.00	0.52	0.00	>0.999999999999999	#DIV/0!
Q99598	Translin-associated protein X OS=Homo sapiens GN=TSNAX PE=1 SV=1	0.00	4.84	0.00	0.00	0.00	0.00	0.00	0.00	0.40	0.00	0.00	1.00	0.00	0.00	1.11	0.00	0.00	0.00	0.00	0.00	0.00	0.00	0.00	0.00	0.52	0.09	>0.999999999999999	5.6
P04745	Alpha-amylase 1 OS=Homo sapiens GN=AMY1A PE=1 SV=2	1.47	0.00	0.00	0.00	1.15	0.87	1.01	0.00	0.48	0.63	0.00	0.60	0.53	1.21	0.00	0.41	0.00	0.00	0.95	0.00	0.18	0.00	0.00	0.34	0.52	0.30	>0.999999999999999	1.7
Q8N9W5	UPF0470 protein C19orf51 OS=Homo sapiens GN=C19orf51 PE=2 SV=3	1.47	1.38	0.00	0.00	0.00	1.60	0.00	0.00	0.00	0.00	1.14	0.60	0.00	0.00	0.00	0.00	0.00	0.00	0.00	0.00	0.00	0.00	0.00	0.00	0.52	0.00	>0.999999999999999	#DIV/0!
Q9P016	Thymocyte nuclear protein 1 OS=Homo sapiens GN=THYN1 PE=1 SV=1	3.18	3.00	0.00	0.00	0.00	0.00	0.00	0.00	0.00	0.00	0.00	0.00	0.00	0.00	0.00	0.00	0.00	0.00	0.00	0.00	0.00	0.00	0.00	0.00	0.51	0.00	>0.999999999999999	#DIV/0!
Q9H7C9	UPF0366 protein C11orf67 OS=Homo sapiens GN=C11orf67 PE=1 SV=1	0.00	0.00	0.00	0.00	0.00	3.64	0.00	0.00	0.00	0.00	0.00	2.49	0.00	0.00	0.00	0.00	0.00	0.00	0.00	0.00	0.00	0.00	0.00	0.00	0.51	0.00	>0.999999999999999	#DIV/0!
P35858	Insulin-like growth factor-binding protein complex acid labile subunit OS=Homo sapiens GN=	1.22	0.00	0.56	0.76	1.92	0.00	0.00	0.00	0.20	0.52	0.95	0.00	0.44	0.00	0.56	0.00	1.89	0.00	1.59	0.00	0.15	2.08	0.00	0.00	0.51	0.56	>0.999999999999999	0.9
Q9GZZ9	Ubiquitin-like modifier-activating enzyme 5 OS=Homo sapiens GN=UBA5 PE=1 SV=1	0.00	1.61	1.68	0.00	0.00	1.02	0.00	0.00	0.28	0.00	0.00	1.49	0.61	0.00	0.00	0.00	0.00	1.07	0.00	0.00	0.00	0.00	0.00	0.00	0.51	0.14	>0.999999999999999	3.6
Q9NQ39	Putative 40S ribosomal protein S10-like OS=Homo sapiens GN=RPS10P5 PE=5 SV=1	4.15	0.00	1.90	0.00	0.00	0.00	0.00	0.00	0.00	0.00	0.00	0.00	0.00	0.00	0.00	1.17	0.00	0.00	0.00	2.20	0.00	0.00	0.00	0.97	0.50	0.36	>0.999999999999999	1.4
Q14498	RNA-binding protein 39 OS=Homo sapiens GN=RBM39 PE=1 SV=2	1.47	0.00	1.23	0.00	0.00	1.60	0.00	0.00	0.00	0.00	1.14	0.60	0.00	1.21	0.00	0.00	0.00	0.00	0.00	1.43	0.00	0.00	0.00	0.00	0.50	0.22	>0.999999999999999	2.3
P15428	15-hydroxyprostaglandin dehydrogenase [NAD(+)] OS=Homo sapiens GN=HPGD PE=1 SV=	2.69	0.00	0.00	0.00	0.00	0.00	0.00	0.00	0.95	0.00	0.00	2.39	0.00	4.83	1.22	1.66	0.00	0.00	1.74	0.00	0.34	0.00	0.00	0.62	0.50	0.87	>0.999999999999999	0.6
P00746	Complement factor D OS=Homo sapiens GN=CFD PE=1 SV=5	0.00	0.00	0.00	1.82	2.30	0.00	0.00	1.90	0.00	0.00	0.00	0.00	0.00	0.00	0.00	0.00	0.00	0.00	0.00	1.55	0.00	0.00	0.00	0.00	0.50	0.13	>0.999999999999999	3.9
P23193	Transcription elongation factor A protein 1 OS=Homo sapiens GN=TCEA1 PE=1 SV=2	4.88	0.00	1.12	0.00	0.00	0.00	0.00	0.00	0.00	0.00	0.00	0.00	0.00	0.00	0.00	0.00	0.00	0.00	0.00	0.00	0.00	0.00	0.00	0.00	0.50	0.00	>0.999999999999999	#DIV/0!
O60739	Eukaryotic translation initiation factor 1b OS=Homo sapiens GN=EIF1B PE=1 SV=2	0.00	0.00	0.00	0.00	0.00	0.00	0.00	0.00	0.00	0.00	0.00	5.98	0.00	0.00	0.00	0.00	0.00	0.00	0.00	0.00	0.00	0.00	0.00	3.41	0.50	0.28	>0.999999999999999	1.8
P32929	Cystathionine gamma-lyase OS=Homo sapiens GN=CTH PE=1 SV=3	1.71	3.46	0.78	0.00	0.00	0.00	0.00	0.00	0.00	0.00	0.00	0.00	0.00	0.00	0.00	0.00	0.00	0.00	0.00	0.00	0.00	0.00	0.00	0.00	0.50	0.00	>0.999999999999999	#DIV/0!
P59827	BPI fold-containing family B member 4 OS=Homo sapiens GN=BPIFB4 PE=2 SV=2	0.00	0.00	1.79	0.00	0.96	0.00	0.00	0.79	0.20	1.67	0.00	0.50	0.44	0.00	0.56	0.00	5.28	0.77	0.00	0.00	0.15	0.00	0.00	0.91	0.49	0.68	>0.999999999999999	0.7
Q9HB07	UPF0160 protein MYG1, mitochondrial OS=Homo sapiens GN=C12orf10 PE=1 SV=2	0.00	0.00	0.00	0.00	0.00	2.33	0.00	1.27	0.00	0.00	1.51	0.80	0.00	0.00	0.00	0.00	0.00	0.00	0.00	0.00	0.00	0.00	1.23	0.00	0.49	0.10	>0.999999999999999	4.8
P52943	Cysteine-rich protein 2 OS=Homo sapiens GN=CRIP2 PE=1 SV=1	0.00	0.00	0.00	0.00	0.00	4.51	0.00	0.00	0.00	0.00	0.00	1.39	0.00	0.00	1.67	0.00	0.00	0.00	0.00	0.00	0.00	0.00	0.00	0.00	0.49	0.14	>0.999999999999999	3.5
Q96S44	TP53-regulating kinase OS=Homo sapiens GN=TP53RK PE=1 SV=2	2.93	0.00	0.00	0.00	0.00	1.75	0.00	0.00	0.00	0.00	0.00	1.20	0.00	0.00	0.00	0.00	0.00	0.00	0.00	1.55	0.00	0.00	0.00	0.00	0.49	0.13	>0.999999999999999	3.8
Q13501	Sequestosome-1 OS=Homo sapiens GN=SQSTM1 PE=1 SV=1	1.71	0.00	1.57	0.00	0.00	2.04	0.00	0.00	0.56	0.00	0.00	0.00	0.00	0.00	0.00	0.00	0.00	1.07	0.00	0.91	0.00	0.00	0.00	0.00	0.49	0.17	>0.999999999999999	3.0
Q99714	3-hydroxyacyl-CoA dehydrogenase type-2 OS=Homo sapiens GN=HSD17B10 PE=1 SV=3	0.00	0.00	0.00	0.00	0.00	1.75	0.00	4.12	0.00	0.00	0.00	0.00	0.00	0.00	0.00	0.00	0.00	0.00	0.00	0.00	0.00	0.00	0.00	0.00	0.49	0.00	>0.999999999999999	#DIV/0!
P01742	Ig heavy chain V-I region EU OS=Homo sapiens PE=1 SV=1	0.00	0.00	3.03	0.00	0.00	0.00	0.00	0.00	0.00	2.82	0.00	0.00	0.00	0.00	0.00	1.86	0.00	4.14	0.00	0.00	0.00	0.00	0.00	0.00	0.49	0.50	>0.999999999999999	1.0
Q08J23	tRNA (cytosine(34)-C(5))-methyltransferase OS=Homo sapiens GN=NSUN2 PE=1 SV=2	1.95	0.92	0.00	0.00	0.00	1.16	0.00	0.63	0.00	0.00	0.76	0.40	0.35	0.00	0.00	0.00	0.00	0.00	0.00	1.04	0.00	0.00	0.61	0.00	0.49	0.17	>0.999999999999999	2.9
Q8TE77	Protein phosphatase Slingshot homolog 3 OS=Homo sapiens GN=SSH3 PE=1 SV=2	2.20	0.00	0.45	0.00	0.77	0.58	0.00	0.00	0.16	0.00	0.76	0.90	0.00	0.81	0.44	0.00	0.00	0.00	0.00	1.81	0.00	0.00	0.00	0.23	0.48	0.27	>0.999999999999999	1.8
Q9UI42	Carboxypeptidase A4 OS=Homo sapiens GN=CPA4 PE=1 SV=2	0.00	1.61	0.78	0.00	0.00	0.00	1.18	2.22	0.00	0.00	0.00	0.00	0.00	1.41	0.00	0.00	0.00	0.00	0.00	0.00	0.00	1.45	0.00	0.00	0.48	0.24	>0.999999999999999	2.0
O95817	BAG family molecular chaperone regulator 3 OS=Homo sapiens GN=BAG3 PE=1 SV=3	1.22	0.00	0.56	0.00	0.00	0.73	0.84	1.74	0.20	0.00	0.00	0.50	0.00	0.00	0.56	0.00	0.94	0.77	0.00	0.00	0.00	0.00	0.00	0.00	0.48	0.19	>0.999999999999999	2.6
Q04446	1,4-alpha-glucan-branching enzyme OS=Homo sapiens GN=GBE1 PE=1 SV=3	0.98	0.00	1.46	0.61	0.77	0.58	0.00	0.63	0.00	0.00	0.76	0.00	0.00	0.00	0.44	0.00	0.00	0.00	0.00	0.00	0.12	0.00	0.00	0.00	0.48	0.05	>0.999999999999999	10.2
Q9P289	Serine/threonine-protein kinase MST4 OS=Homo sapiens GN=MST4 PE=1 SV=2	0.00	1.61	0.78	0.00	1.34	0.00	0.00	0.00	0.00	0.00	1.33	0.70	0.61	3.02	0.78	0.48	1.32	2.30	0.00	0.00	0.00	1.45	0.00	0.85	0.48	0.90	>0.999999999999999	0.5
Q86SG5	Protein S100-A7A OS=Homo sapiens GN=S100A7A PE=2 SV=3	0.00	0.00	0.00	0.00	5.75	0.00	0.00	0.00	0.00	0.00	0.00	0.00	0.00	0.00	0.00	0.00	0.00	0.00	0.00	0.00	0.00	0.00	0.00	0.00	0.48	0.00	>0.999999999999999	#DIV/0!
P18065	Insulin-like growth factor-binding protein 2 OS=Homo sapiens GN=IGFBP2 PE=1 SV=2	0.00	2.07	0.00	0.00	0.00	1.31	0.00	1.43	0.00	0.94	0.00	0.00	0.00	0.00	1.00	0.00	3.58	0.00	0.00	0.00	0.00	0.00	0.00	0.00	0.48	0.38	>0.999999999999999	1.3
Q8IUE6	Histone H2A type 2-B OS=Homo sapiens GN=HIST2H2AB PE=1 SV=3	0.00	0.00	0.00	0.00	0.00	0.00	0.00	0.00	0.00	5.74	0.00	0.00	0.00	0.00	0.00	0.00	0.00	0.00	0.00	0.00	0.00	0.00	0.00	0.00	0.48	0.00	>0.999999999999999	#DIV/0!
Q08211	ATP-dependent RNA helicase A OS=Homo sapiens GN=DHX9 PE=1 SV=4	1.71	0.00	0.56	0.00	0.00	0.73	0.34	0.00	0.08	0.00	1.33	1.00	0.00	0.00	0.00	0.00	0.00	0.00	0.00	0.91	0.00	0.00	0.00	0.00	0.48	0.08	>0.999999999999999	6.3
Q14697	Neutral alpha-glucosidase AB OS=Homo sapiens GN=GANAB PE=1 SV=3	0.73	0.00	0.67	0.00	0.58	1.31	0.00	1.43	0.12	0.00	0.57	0.30	0.26	0.60	0.00	0.00	0.00	0.00	0.48	0.39	0.00	1.25	0.00	0.00	0.48	0.25	>0.999999999999999	1.9
Q9BTE1	Dynactin subunit 5 OS=Homo sapiens GN=DCTN5 PE=1 SV=1	3.91	0.00	1.79	0.00	0.00	0.00	0.00	0.00	0.00	0.00	0.00	0.00	0.00	0.00	0.00	0.00	0.00	0.00	0.00	0.00	0.00	0.00	0.00	0.00	0.48	0.00	>0.999999999999999	#DIV/0!
Q9P0W8	Spermatogenesis-associated protein 7 OS=Homo sapiens GN=SPATA7 PE=2 SV=3	0.00	1.15	0.56	0.76	0.00	0.73	0.84	0.00	0.20	0.00	0.95	0.50	0.00	1.01	0.56	0.34	0.94	0.77	0.00	0.65	0.15	1.04	0.77	0.28	0.47	0.54	>0.999999999999999	0.9
Q15233	Non-POU domain-containing octamer-binding protein OS=Homo sapiens GN=NONO PE=1 S	3.18	0.00	0.00	0.00	0.00	1.89	0.00	0.00	0.00	0.00	0.00	0.60	0.00	0.00	0.00	0.00	0.00	0.00	0.00	0.00	0.00	0.00	0.00	0.00	0.47	0.00	>0.999999999999999	#DIV/0!
O60825	6-phosphofructo-2-kinase/fructose-2,6-bisphosphatase 2 OS=Homo sapiens GN=PFKFB2 F	0.00	1.38	0.67	0.91	1.15	0.00	0.00	0.95	0.00	0.00	0.00	0.60	0.00	2.22	0.00	0.41	0.00	0.00	0.00	0.78	0.00	0.00	0.00	0.00	0.47	0.28	>0.999999999999999	1.7
P49411	Elongation factor Tu, mitochondrial OS=Homo sapiens GN=TUFM PE=1 SV=2	0.00	0.00	0.78	0.00	0.00	0.00	0.00	4.60	0.28	0.00	0.00	0.00	0.61	1.41	0.78	0.00	0.00	0.00	0.00	0.00	0.00	0.00	0.00	0.00	0.47	0.23	>0.999999999999999	2.0
Q9ULV4	Coronin-1C OS=Homo sapiens GN=CORO1C PE=1 SV=1	0.00	1.38	0.00	0.91	0.00	2.77	0.00	0.00	0.00	0.00	0.00	0.60	0.00	1.21	0.00	0.41	0.00	1.99	0.00	0.00	0.00	0.00	0.00	0.00	0.47	0.30	>0.999999999999999	1.6

P17050	Alpha-N-acetylgalactosaminidase OS=Homo sapiens GN=NAGA PE=1 SV=2	0.00	3.46	0.00	0.00	0.00	2.18	0.00	0.00	0.00	0.00	0.00	0.00	0.00	1.41	0.00	0.00	0.00	0.00	0.00	0.00	0.00	0.00	0.00	0.00	0.47	0.12	>0.999999999999999	4.0
Q9Y6W5	Wiskott-Aldrich syndrome protein family member 2 OS=Homo sapiens GN=WASF2 PE=1 SV	2.93	0.00	0.00	0.00	0.00	1.75	0.00	0.95	0.00	0.00	0.00	0.00	0.00	0.00	0.00	0.00	0.00	0.92	0.00	0.00	0.18	1.25	0.92	0.34	0.47	0.30	>0.999999999999999	1.6
Q86UP2	Kinectin OS=Homo sapiens GN=KTN1 PE=1 SV=1	0.98	2.07	0.00	0.00	0.00	0.29	0.00	0.00	0.00	0.00	2.08	0.20	0.00	0.00	0.00	0.00	0.00	0.00	0.00	0.00	0.00	0.00	0.00	0.00	0.47	0.00	>0.999999999999999	#DIV/0!
P51812	Ribosomal protein S6 kinase alpha-3 OS=Homo sapiens GN=RPS6KA3 PE=1 SV=1	0.00	0.00	0.45	0.00	0.00	1.16	0.00	0.00	0.00	0.00	2.27	1.69	0.00	0.00	0.00	0.00	0.00	0.00	0.00	0.52	0.12	0.00	0.00	0.00	0.46	0.05	>0.999999999999999	8.7
P19367	Hexokinase-1 OS=Homo sapiens GN=HK1 PE=1 SV=3	0.73	0.69	0.67	0.45	0.00	0.87	0.00	1.59	0.24	0.31	0.00	0.00	0.00	0.00	0.67	0.21	0.00	0.00	0.95	2.20	0.00	0.62	0.46	0.17	0.46	0.44	>0.999999999999999	1.1
Q9H0D6	5'-3' exoribonuclease 2 OS=Homo sapiens GN=XRN2 PE=1 SV=1	1.47	0.69	0.34	0.45	0.00	0.87	0.00	0.00	0.00	0.00	1.14	0.60	0.00	0.00	0.00	0.00	0.00	0.00	0.00	0.00	0.00	0.00	0.00	0.17	0.46	0.01	>0.999999999999999	32.6
Q07000	HLA class I histocompatibility antigen, Cw-15 alpha chain OS=Homo sapiens GN=HLA-C PE	0.00	0.00	0.00	0.00	0.00	0.00	0.00	4.12	1.43	0.00	0.00	0.00	0.00	0.00	0.00	0.00	0.00	0.00	0.00	0.00	0.00	5.40	0.00	0.00	0.46	0.45	>0.999999999999999	1.0
P07099	Epoxide hydrolase 1 OS=Homo sapiens GN=EPHX1 PE=1 SV=1	0.00	0.00	0.00	0.00	0.00	0.00	0.00	5.55	0.00	0.00	0.00	0.00	0.53	0.00	0.67	0.00	0.00	0.00	0.00	0.00	0.00	0.00	0.00	0.00	0.46	0.10	>0.999999999999999	4.7
Q8TD06	Anterior gradient protein 3 homolog OS=Homo sapiens GN=AGR3 PE=1 SV=1	0.00	0.00	0.00	0.00	0.00	5.53	0.00	0.00	0.00	0.00	0.00	0.00	0.00	0.00	0.00	0.00	0.00	0.00	0.00	0.00	0.00	0.00	0.00	0.00	0.46	0.00	>0.999999999999999	#DIV/0!
Q96CN7	Isochorismatase domain-containing protein 1 OS=Homo sapiens GN=ISOC1 PE=1 SV=3	0.00	0.00	1.12	0.00	1.92	0.00	0.00	0.00	0.40	0.00	0.00	2.09	0.00	2.01	0.00	0.00	0.00	0.00	0.00	0.00	0.00	0.00	0.00	0.00	0.46	0.17	>0.999999999999999	2.7
Q9NX46	Poly(ADP-ribose) glycohydrolase ARH3 OS=Homo sapiens GN=ADPRHL2 PE=1 SV=1	1.95	0.00	0.90	0.00	0.00	1.16	0.00	0.00	0.71	0.00	0.00	0.80	0.00	1.61	0.00	1.24	0.00	0.00	0.00	1.04	0.00	0.00	0.00	0.00	0.46	0.32	>0.999999999999999	1.4
P50579	Methionine aminopeptidase 2 OS=Homo sapiens GN=METAP2 PE=1 SV=1	0.00	1.38	0.00	0.00	0.00	1.89	0.00	0.00	0.52	0.00	1.14	0.60	0.00	0.00	0.00	0.00	0.00	0.00	0.00	0.00	0.00	0.00	0.00	0.00	0.46	0.00	>0.999999999999999	#DIV/0!
P05141	ADP/ATP translocase 2 OS=Homo sapiens GN=SLC25A5 PE=1 SV=7	0.00	0.00	0.00	0.00	0.00	0.00	0.00	3.33	0.00	2.19	0.00	0.00	0.00	0.00	0.00	0.00	0.00	0.00	3.33	0.00	0.00	0.00	0.00	0.00	0.46	0.28	>0.999999999999999	1.7
O60841	Eukaryotic translation initiation factor 5B OS=Homo sapiens GN=EIF5B PE=1 SV=4	1.22	1.15	0.78	0.00	0.00	1.02	0.00	0.00	0.00	0.00	1.33	0.00	0.00	0.00	0.00	0.00	0.00	0.31	0.00	1.55	0.06	0.00	0.00	0.00	0.46	0.16	>0.999999999999999	2.9
Q8N8S7	Protein enabled homolog OS=Homo sapiens GN=ENAH PE=1 SV=2	1.22	1.15	0.00	0.00	0.96	0.00	0.00	0.00	0.20	0.00	0.95	1.00	0.00	1.01	0.00	0.00	0.00	0.00	0.00	0.00	0.00	0.00	0.00	0.00	0.46	0.08	>0.999999999999999	5.4
P53582	Methionine aminopeptidase 1 OS=Homo sapiens GN=METAP1 PE=1 SV=2	1.71	0.00	0.78	0.00	0.00	1.02	0.00	0.00	0.63	0.00	1.33	0.00	0.61	1.41	0.00	0.48	0.00	0.00	0.00	0.00	0.00	0.00	0.00	0.00	0.46	0.21	>0.999999999999999	2.2
P10619	Lysosomal protective protein OS=Homo sapiens GN=CTSA PE=1 SV=2	0.00	0.00	0.67	0.91	1.15	1.75	0.00	0.95	0.00	0.00	0.00	0.00	0.53	1.21	0.00	0.41	1.13	1.84	0.95	1.55	0.00	2.49	1.84	0.34	0.45	1.03	>0.999999999999999	0.4
Q13131	5'-AMP-activated protein kinase catalytic subunit alpha-1 OS=Homo sapiens GN=PRKAA1 F	2.44	0.00	0.56	0.00	0.96	1.46	0.00	0.00	0.00	0.00	0.00	0.00	0.00	1.01	0.00	0.00	0.00	0.00	0.00	0.00	0.00	0.00	0.00	0.00	0.45	0.08	>0.999999999999999	5.4
Q96QR1	Secretoglobin family 3A member 1 OS=Homo sapiens GN=SCGB3A1 PE=1 SV=2	0.00	0.00	0.00	0.00	0.00	0.00	0.00	5.39	0.00	0.00	0.00	0.00	2.98	0.00	0.00	5.45	0.00	0.00	0.00	0.00	0.00	0.00	0.00	0.00	0.45	0.70	>0.999999999999999	0.6
O60610	Protein diaphanous homolog 1 OS=Homo sapiens GN=DIAPH1 PE=1 SV=2	0.49	1.15	0.22	0.30	0.00	1.75	0.00	0.00	0.40	0.00	0.38	0.70	0.00	1.41	0.00	0.00	0.38	0.31	0.00	0.91	0.15	1.45	0.31	0.28	0.45	0.43	>0.999999999999999	1.0
Q8NE71	ATP-binding cassette sub-family F member 1 OS=Homo sapiens GN=ABCF1 PE=1 SV=2	1.71	0.69	0.34	0.00	0.00	2.04	0.00	0.00	0.31	0.00	0.00	0.30	0.26	0.00	0.00	0.00	0.00	0.00	0.00	0.00	0.00	0.00	0.00	0.00	0.45	0.02	>0.999999999999999	20.5
Q9NUP9	Protein lin-7 homolog C OS=Homo sapiens GN=LIN7C PE=1 SV=1	0.00	0.00	1.68	0.00	0.00	2.18	0.00	0.00	0.00	0.00	0.00	1.49	0.00	0.00	0.00	0.00	0.00	0.00	0.00	0.00	0.00	0.00	0.00	0.00	0.45	0.00	>0.999999999999999	#DIV/0!
O75663	TIP41-like protein OS=Homo sapiens GN=TIPRL PE=1 SV=2	2.69	0.00	0.00	0.00	0.00	0.00	0.00	0.00	0.44	0.00	0.00	2.19	0.00	0.00	0.00	0.00	0.00	0.00	0.00	0.00	0.00	0.00	0.00	0.00	0.44	0.00	>0.999999999999999	#DIV/0!
Q8WVF1	Protein OSCP1 OS=Homo sapiens GN=OSCP1 PE=1 SV=4	0.00	0.00	0.00	0.00	0.00	3.49	0.00	1.11	0.00	0.00	0.00	0.70	0.61	0.00	0.00	0.00	0.00	0.00	0.00	0.00	0.00	0.00	0.00	0.00	0.44	0.05	>0.999999999999999	8.6
O95218	Zinc finger Ran-binding domain-containing protein 2 OS=Homo sapiens GN=ZRANB2 PE=1	4.40	0.00	0.00	0.00	0.00	0.00	0.00	0.00	0.00	0.00	0.00	0.90	0.00	0.00	0.00	0.00	0.00	0.00	0.00	0.00	0.00	0.00	0.00	0.00	0.44	0.00	>0.999999999999999	#DIV/0!
P03950	Angiogenin OS=Homo sapiens GN=ANG PE=1 SV=1	0.00	0.00	2.24	3.03	0.00	0.00	0.00	0.00	0.00	0.00	0.00	0.00	0.00	4.03	0.00	1.38	3.77	0.00	11.57	0.00	0.00	0.00	0.00	0.00	0.44	1.73	>0.999999999999999	0.3
Q15149	Plectin OS=Homo sapiens GN=PLEC PE=1 SV=3	0.73	0.46	0.67	0.15	0.19	1.75	0.17	0.32	0.04	0.21	0.38	0.20	0.18	1.61	0.44	0.55	0.00	0.15	0.16	1.04	0.00	0.21	0.15	0.17	0.44	0.39	>0.999999999999999	1.1
Q9Y5K6	CD2-associated protein OS=Homo sapiens GN=CD2AP PE=1 SV=1	0.00	1.15	1.57	0.00	0.00	0.73	0.00	0.00	0.36	0.00	0.95	0.50	0.00	1.81	0.00	0.00	0.00	0.00	0.00	0.65	0.00	0.00	0.77	0.28	0.44	0.29	>0.999999999999999	1.5
O43504	Hepatitis B virus X-interacting protein OS=Homo sapiens GN=HBXIP PE=1 SV=1	0.00	0.00	0.00	0.00	0.00	5.24	0.00	0.00	0.00	0.00	0.00	0.00	0.00	0.00	0.00	0.00	0.00	0.00	0.00	0.00	0.00	0.00	0.00	0.00	0.44	0.00	>0.999999999999999	#DIV/0!
P20073	Annexin A7 OS=Homo sapiens GN=ANXA7 PE=1 SV=3	3.18	0.00	0.00	0.00	0.00	0.00	0.00	2.06	0.00	0.00	0.00	0.00	0.53	0.00	0.00	0.00	0.00	0.00	0.00	0.78	0.00	0.00	0.00	0.00	0.44	0.11	>0.999999999999999	4.0
Q2M2I5	Keratin, type I cytoskeletal 24 OS=Homo sapiens GN=KRT24 PE=1 SV=1	0.00	0.00	0.00	0.00	0.00	0.00	0.00	0.00	0.00	5.22	0.00	0.00	0.00	0.00	0.00	0.00	0.00	0.00	0.00	0.00	0.00	0.00	0.00	0.00	0.43	0.00	>0.999999999999999	#DIV/0!
Q9UN36	Protein NDRG2 OS=Homo sapiens GN=NDRG2 PE=1 SV=2	0.00	1.84	0.90	0.00	0.00	2.47	0.00	0.00	0.00	0.00	0.00	0.00	0.70	1.61	0.00	0.55	0.00	0.00	0.00	1.04	0.00	1.66	0.00	0.97	0.43	0.54	>0.999999999999999	0.8
P67870	Casein kinase II subunit beta OS=Homo sapiens GN=CSNK2B PE=1 SV=1	0.00	0.00	1.46	0.00	0.00	0.00	0.00	0.00	0.00	0.00	2.46	1.29	0.00	0.00	0.00	0.00	0.00	0.00	0.00	1.68	0.00	0.00	0.00	0.74	0.43	0.20	>0.999999999999999	2.2
Q17RN3	Protein FAM98C OS=Homo sapiens GN=FAM98C PE=2 SV=1	2.20	0.00	0.00	0.00	0.00	1.31	0.00	0.00	0.00	0.00	1.70	0.00	0.00	0.00	0.00	0.00	0.00	0.00	0.00	0.00	0.00	0.00	0.00	0.00	0.43	0.00	>0.999999999999999	#DIV/0!
Q99615	DnaJ homolog subfamily C member 7 OS=Homo sapiens GN=DNAJC7 PE=1 SV=2	1.47	1.38	0.00	0.00	0.00	1.75	0.00	0.00	0.00	0.00	0.00	0.60	0.53	0.00	0.00	0.00	0.00	0.00	0.00	0.00	0.00	0.00	0.00	0.00	0.43	0.04	>0.999999999999999	9.9
Q02539	Histone H1.1 OS=Homo sapiens GN=HIST1H1A PE=1 SV=3	0.00	3.46	1.68	0.00	0.00	0.00	0.00	0.00	0.00	0.00	0.00	0.00	0.00	3.02	0.00	0.00	0.00	0.00	2.38	0.00	0.00	0.00	0.00	0.00	0.43	0.45	>0.999999999999999	1.0
Q6QNY1	Biogenesis of lysosome-related organelles complex 1 subunit 2 OS=Homo sapiens GN=BLC	5.13	0.00	0.00	0.00	0.00	0.00	0.00	0.00	0.00	0.00	0.00	0.00	0.00	0.00	0.00	0.00	0.00	0.00	0.00	0.00	0.00	0.00	0.00	0.00	0.43	0.00	>0.999999999999999	#DIV/0!
P21741	Midkine OS=Homo sapiens GN=MDK PE=1 SV=1	5.13	0.00	0.00	0.00	0.00	0.00	0.00	0.00	0.00	0.00	0.00	0.00	0.00	4.23	0.00	0.00	0.00	0.00	3.33	0.00	0.00	0.00	0.00	0.00	0.43	0.63	>0.999999999999999	0.7
O75800	Zinc finger MYND domain-containing protein 10 OS=Homo sapiens GN=ZMYND10 PE=1 SV	1.47	0.00	0.00	0.00	0.00	3.06	0.00	0.00	0.00	0.00	0.00	0.60	0.00	0.00	0.00	0.00	0.00	0.00	0.00	0.00	0.00	0.00	0.00	0.00	0.43	0.00	>0.999999999999999	#DIV/0!
Q96E17	Ras-related protein Rab-3C OS=Homo sapiens GN=RAB3C PE=2 SV=1	0.00	0.00	0.00	0.00	0.00	0.00	0.00	0.00	0.00	0.00	5.11	0.00	0.00	5.44	0.00	0.00	0.00	0.00	0.00	3.50	0.00	0.00	0.00	0.00	0.43	0.74	>0.999999999999999	0.6
Q14126	Desmoglein-2 OS=Homo sapiens GN=DSG2 PE=1 SV=2	1.22	0.69	0.56	0.45	0.00	0.44	0.00	0.48	0.20	0.00	0.57	0.50	0.00	0.60	0.00	0.21	0.00	0.00	0.48	0.00	0.00	0.00	0.00	0.28	0.43	0.13	>0.999999999999999	3.2
Q5JSH3	WD repeat-containing protein 44 OS=Homo sapiens GN=WDR44 PE=1 SV=1	0.00	2.30	0.34	0.00	0.00	1.02	0.00	0.00	0.12	0.00	0.57	0.70	0.00	0.60	0.00	0.00	0.00	0.00	0.00	0.00	0.00	0.00	0.00	0.00	0.42	0.05	>0.999999999999999	8.3
P16949	Stathmin OS=Homo sapiens GN=STMN1 PE=1 SV=3	0.00	0.00	0.00	3.03	0.00	0.00	0.00	0.00	0.00	0.00	0.00	1.99	0.00	4.03	0.00	0.00	0.00	0.00	0.00	0.00	0.00	4.16	0.00	0.00	0.42	0.68	>0.999999999999999	0.6
Q9Y4L1	Hypoxia up-regulated protein 1 OS=Homo sapiens GN=HYOU1 PE=1 SV=1	0.00	0.00	1.01	0.00	0.00	1.75	0.00	1.90	0.36	0.00	0.00	0.00	0.00	0.60	0.33	0.00	0.00	0.00	0.48	0.00	0.00	0.00	0.00	0.00	0.42	0.12	>0.999999999999999	3.5
Q9Y6D6	Brefeldin A-inhibited guanine nucleotide-exchange protein 1 OS=Homo sapiens GN=ARFGE	0.49	0.69	0.67	0.30	0.00	0.87	0.00	0.00	0.24	0.00	0.95	0.80	0.18	1.61	0.22	0.21	0.00	0.00	0.00	0.26	0.00	0.00	0.00	0.11	0.42	0.22	>0.999999999999999	1.9
Q14117	Dihydropyrimidinase OS=Homo sapiens GN=DPYS PE=1 SV=1	1.47	0.00	0.00	0.00	0.00	1.75	0.00	0.00	0.00	0.00	0.00	1.79	0.00	0.00	0.00	0.00	0.00	0.00	0.00	0.00	0.00	0.00	0.00	0.00	0.42	0.00	>0.999999999999999	#DIV/0!
Q8NEU8	DCC-interacting protein 13-beta OS=Homo sapiens GN=APPL2 PE=1 SV=3	0.98	0.92	0.00	0.00	0.00	0.00	0.00	0.00	0.00	0.00	1.70	1.39	0.00	0.00	0.00	0.00	0.00	0.00	0.00	0.00	0.00	0.00	0.00	0.00	0.42	0.00	>0.999999999999999	#DIV/0!
O14907	Tax1-binding protein 3 OS=Homo sapiens GN=TAX1BP3 PE=1 SV=2	0.00	0.00	0.00	0.00	0.00	0.00	0.00	3.96	0.99	0.00	0.00	0.00	2.19	0.00	0.00	0.00	0.00	0.00	0.00	3.24	0.00	0.00	0.00	0.00	0.41	0.45	>0.999999999999999	0.9
P10253	Lysosomal alpha-glucosidase OS=Homo sapiens GN=GAA PE=1 SV=4	0.00	1.38	0.34	0.00	0.00	1.89	0.00	0.48	0.00	0.00	0.57	0.30	0.00	0.60	0.00	0.00	0.00	0.46	0.00	0.78	0.00	0.00	0.00	0.00	0.41	0.15	>0.999999999999999	2.7
Q9NTX5	Ethylmalonyl-CoA decarboxylase OS=Homo sapiens GN=ECHDC1 PE=1 SV=2	0.00	0.00	0.00	0.00	0.00	3.06	0.00	0.00	0.00	0.00	1.89	0.00	0.00	0.00	0.00	0.00	0.00	1.53	0.00	0.00	0.00	0.00	0.00	0.00	0.41	0.13	>0.999999999999999	3.2
Q9Y285	Phenylalanine--tRNA ligase alpha subunit OS=Homo sapiens GN=FARSA PE=1 SV=3	1.47	0.00	0.00	0.00	0.00	1.75	0.00	0.00	0.00	0.00	1.14	0.60	0.53	0.00	0.00	0.00	0.00	0.00	0.00	0.00	0.00	0.00	0.00	0.00	0.41	0.04	>0.999999999999999	9.4
P07919	Cytochrome b-c1 complex subunit 6, mitochondrial OS=Homo sapiens GN=UQCRH PE=1 S	0.00	0.00	0.00	0.00	0.00	0.00	0.00	4.91	0.00	0.00	0.00	0.00	0.00	0.00	0.00	0.00	0.00	0.00	0.00	0.00	0.00	0.00	0.00	0.00	0.41	0.00	>0.999999999999999	#DIV/0!
P11279	Lysosome-associated membrane glycoprotein 1 OS=Homo sapiens GN=LAMP1 PE=1 SV=3	0.00	1.61	0.00	0.00	0.00	2.18	0.00	1.11	0.00	0.00	0.00	0.00	0.00	1.41	0.78	0.48	0.00	1.07	0.00	0.91	0.00	1.45	1.07	0.00	0.41	0.60	>0.999999999999999	0.7
B2RPK0	Putative high mobility group protein B1-like 1 OS=Homo sapiens GN=HMGB1P1 PE=5 SV=1	0.00	0.00	0.00	0.00	0.00	0.00	0.00	0.00	0.00	4.91	0.00	0.00	0.00	0.00	0.00	0.00	0.00	0.00	0.00	0.00	0.00	0.00	2.15	0.00	0.41	0.18	>0.999999999999999	2.3
Q03393	6-pyruvoyl tetrahydrobiopterin synthase OS=Homo sapiens GN=PTS PE=1 SV=1	0.00	0.00	0.00	0.00	0.00	2.91	0.00	0.00	0.00	0.00	0.00	1.99	0.00	0.00	0.00	0.00	0.00	0.00	0.00	0.00	0.00	0.00	0.00	1.14	0.41	0.09	>0.999999999999999	4.3
Q15102	Platelet-activating factor acetylhydrolase IB subunit gamma OS=Homo sapiens GN=PAFAH	0.00	0.00	0.00	0.00	0.00	0.00	0.00	0.00	0.52	0.00	0.00	4.38	0.00	0.00	0.00	0.00	0.00	0.00	0.00	0.00	0.40	0.00	0.00	0.00	0.41	0.03	>0.999999999999999	12.2
Q04323	UBX domain-containing protein 1 OS=Homo sapiens GN=UBXN1 PE=1 SV=2	2.44	0.00	0.00	0.00	0.00	1.46	0.00	0.00	0.00	0.00	0.00	1.00	0.00	0.00	0.00	0.00	0.00	0.00	0.00	0.00	0.00	0.00	0.00	0.00	0.41	0.00	>0.999999999999999	#DIV/0!
P40855	Peroxisomal biogenesis factor 19 OS=Homo sapiens GN=PEX19 PE=1 SV=1	2.44	0.00	0.00	0.00	0.00	1.46	0.00	0.00	0.00	0.00	0.00	1.00	0.00	0.00	0.00	0.00	0.00	0.00	0.00	0.00	0.00	0.00	0.00	0.00	0.41	0.00	>0.999999999999999	#DIV/0!
Q16775	Hydroxyacylglutathione hydrolase, mitochondrial OS=Homo sapiens GN=HAGH PE=1 SV=2	0.00	2.30	1.12	0.00	0.00	1.46	0.00	0.00	0.00	0.00	0.00	0.00	0.00	0.00	0.00	0.00	0.00	0.00	0.00	0.00	0.00	0.00	0.00	0.00	0.41	0.00	>0.999999999999999	#DIV/0!
P05026	Sodium/potassium-transporting ATPase subunit beta-1 OS=Homo sapiens GN=ATP1B1 PE	0.00	0.00	0.00	0.00	0.00	1.31	0.00	3.17	0.36	0.00	0.00	0.00	0.00	0.00	0.00	0.62	0.00	1.38	0.00	0.00	0.00	0.00	0.00	0.00	0.40	0.17	>0.999999999999999	2.4
P07686	Beta-hexosaminidase subunit beta OS=Homo sapiens GN=HEXB PE=1 SV=3	0.00	1.15	0.56	0.00	0.00	2.33	0.00	0.79	0.00	0.00	0.00	0.00	0.00	2.22	0.00	0.76	0.00	1.69	0.00	1.43	0.00	0.00	0.00	0.91	0.40	0.58	>0.999999999999999	0.7
Q96LB3	Intraflagellar transport protein 74 homolog OS=Homo sapiens GN=IFT74 PE=2 SV=1	1.22	1.15	0.56	0.00	0.00	0.00	0.00	0.00	0.00	0.00	1.89	0.00	0.00	0.00	0.00	0.00	0.00	0.00	0.00	0.00	0.00	0.00	0.00	0.00	0.40	0.00	>0.999999999999999	#DIV/0!
P35611	Alpha-adducin OS=Homo sapiens GN=ADD1 PE=1 SV=2	0.98	0.92	0.00	0.61	0.77	0.00	0.00	0.00	0.00	0.00	0.76	0.80	0.00	0.81	0.00	0.00	0.00	0.00	0.00	0.00	0.00	0.00	0.00	0.00	0.40	0.07	>0.999999999999999	6.0
P08133	Annexin A6 OS=Homo sapiens GN=ANXA6 PE=1 SV=3	0.00	0.00	0.00	0.00	0.00	0.00	0.00	0.00	0.00	4.80	0.00	0.00	0.00	0.00	0.00	0.00	0.00	0.00	0.00	0.00	0.00	0.00	0.00	0.00	0.40	0.00	>0.999999999999999	#DIV/0!
O43866	CD5 antigen-like OS=Homo sapiens GN=CD5L PE=1 SV=1	0.00	0.00	0.00	1.21	3.26	0.00	0.00	0.00	0.32	0.00	0.00	0.00	0.70	1.61	1.89	0.55	1.51	1.23	0.00	0.00	0.25	1.66	1.23	0.00	0.40	0.89	>0.999999999999999	0.5
Q9NQ88	Probable fructose-2,6-bisphosphatase TIGAR OS=Homo sapiens GN=TIGAR PE=1 SV=1	0.00	0.00	0.00	0.00	0.00	1.60	0.00	0.00	0.00	0.00	2.08	1.10	0.00	2.22	0.00	0.76	0.00	0.00	0.00	1.43	0.34	2.29	0.00	0.62	0.40	0.64	>0.999999999999999	0.6
Q99426	Tubulin-folding cofactor B OS=Homo sapiens GN=TBCB PE=1 SV=2	2.93	0.00	0.00	1.82	0.00	0.00	0.00	0.00	0.00	0.00	0.00	0.00	0.00	0.00	0.00	0.00	0.00	0.00	0.00	0.00	0.00	0.00	0.00	0.00	0.40	0.00	>0.999999999999999	#DIV/0!
Q06033	Inter-alpha-trypsin inhibitor heavy chain H3 OS=Homo sapiens GN=ITIH3 PE=1 SV=2	0.00	0.69	0.00	0.45	1.92	0.00	0.51	0.00	0.00	0.31	0.57	0.30	0.00	0.00	0.78	0.21	1.32	0.00	1.59	0.00	0.09	1.45	1.07	0.17	0.40	0.56	>0.999999999999999	0.7
Q92734	Protein TFG OS=Homo sapiens GN=TFG PE=1 SV=2	0.00	0.00	1.79	0.00	0.00	1.16	0.00	0.00	0.99	0.00	0.00	0.80	0.00	1.61	0.00	0.55	0.00	0.00	0.00	1.04	0.00	0.00	0.00	0.45	0.40	0.30	>0.999999999999999	1.3
Q14141	Septin-6 OS=Homo sapiens GN=SEPT6 PE=1 SV=4	3.42	0.00	0.00	0.00	0.00	0.00	0.00	0.00	0.00	0.00	1.33	0.00	0.00	0.00	0.00	0.00	0.00	0.00	0.00	0.00	0.00	0.00	0.00	0.00	0.40	0.00	>0.999999999999999	#DIV/0!
P10644	cAMP-dependent protein kinase type I-alpha regulatory subunit OS=Homo sapiens GN=PRK	0.00	0.00	0.90	0.00	1.53	0.00	0.00	0.00	0.00	0.00	1.51	0.80	0.70	1.61	0.89	0.00	0.00	1.23	1.27	1.04	0.00	1.66	0.00	0.45	0.40	0.74	>0.999999999999999	0.5
Q9NZT2	Opioid growth factor receptor OS=Homo sapiens GN=OGFR PE=1 SV=3	0.00	0.92	0.00	0.00	0.00	0.58	0.00	0.63	0.00	0.00	1.70	0.90	0.35	1.81	0.00	0.28	0.00	0.00	0.00	1.17	0.00	0.00	0.00	0.00	0.39	0.30	>0.999999999999999	1.3
O75947	ATP synthase subunit d, mitochondrial OS=Homo sapiens GN=ATP5H PE=1 SV=3	0.00	0.00	0.00	0.00	0.00	0.00	0.00	2.85	0.00	1.88	0.00	0.00	0.00	0.00	0.00	0.00	0.00	0.00	0.00	0.00	0.00	0.00	0.00	0.00	0.39	0.00	>0.999999999999999	#DIV/0!
P22307	Non-specific lipid-transfer protein OS=Homo sapiens GN=SCP2 PE=1 SV=2	1.47	0.00	0.67	0.00	0.00	1.60	0.00	0.95	0.00	0.00	0.00	0.00	0.53	0.00	0.00	0.00	0.00	0.00	0.00	0.78	0.00	0.00	0.00	0.00	0.39	0.11	>0.999999999999999	3.6
P57735	Ras-related protein Rab-25 OS=Homo sapiens GN=RAB25 PE=1 SV=2	0.00	0.00	0.00	0.00	0.00	2.04	0.00	0.00	0.00	0.00	2.65	0.00	0.00	0.00	0.00	0.00	0.00	0.00	0.00	0.00	0.00	2.91	0.00	0.79	0.39	0.31	>0.999999999999999	1.3
Q96FT9	Intraflagellar transport protein 43 homolog OS=Homo sapiens GN=IFT43 PE=1 SV=3	0.00	0.00	0.00	0.00	0.00	2.04	0.00	0.00	0.00	0.00	2.65	0.00	0.00	0.00	0.00	0.00	0.00	0.00	0.00	0.00	0.00	0.00	0.00	0.00	0.39	0.00	>0.999999999999999	#DIV/0!
P08621	U1 small nuclear ribonucleoprotein 70 kDa OS=Homo sapiens GN=SNRNP70 PE=1 SV=2	1.47	1.38	0.67	0.00	0.00	0.00	0.00	0.00	0.00	0.00	1.14	0.00	0.00	0.00	0.00	0.00	0.00	0.00	0.00	0.00	0.00	0.00	0.00	0.00	0.39	0.00	>0.999999999999999	#DIV/0!
P48739	Phosphatidylinositol transfer protein beta isoform OS=Homo sapiens GN=PITPNB PE=1 SV	2.44	0.00	0.00	0.00	0.00	0.00	0.00	0.00	0.00	0.00	0.00	2.19	0.88	2.01	0.00	0.00	0.00	0.00	1.59	2.85	0.00	2.08	0.00	0.57	0.39	0.83	>0.999999999999999	0.5
Q9Y263	Phospholipase A-2-activating protein OS=Homo sapiens GN=PLAA PE=1 SV=2	0.98	0.00	0.45	0.00	0.00	1.60	0.00	0.63	0.16	0.00	0.00	0.80	0.00	0.81	0.00	0.00	0.00	0.00	0.00	0.52	0.12	0.00	0.00	0.00	0.38	0.12	>0.999999999999999	3.2
P09622	Dihydrolipoyl dehydrogenase, mitochondrial OS=Homo sapiens GN=DLD PE=1 SV=2	0.00	1.38	0.67	0.00	0.00	0.00	0.00	1.90	0.00	0.63	0.00	0.00	1.05	1.21	0.00	0.00	0.00	0.00	0.00	0.00	0.00	0.00	0.00	0.00	0.38	0.19	>0.999999999999999	2.0
Q05315	Eosinophil lysophospholipase OS=Homo sapiens GN=CLC PE=1 SV=3	0.00	0.00	0.00	0.00	0.00	0.00	3.71	0.00	0.87	0.00	0.00	0.00	0.00	0.00	0.00	0.00	0.00	0.00	0.00	0.00	0.00	0.00	0.00	0.00	0.38	0.00	>0.999999999999999	#DIV/0!
P16930	Fumarylacetoacetase OS=Homo sapiens GN=FAH PE=1 SV=2	0.00	0.00	0.00	1.06	0.00	2.18	0.00	0.00	0.00	0.00	1.33	0.00	0.00	1.41	0.00	0.48	0.00	0.00	0.00	0.00	0.00	1.45	0.00	0.00	0.38	0.28	>0.999999999999999	1.4
P11172	Uridine 5'-monophosphate synthase OS=Homo sapiens GN=UMPS PE=1 SV=1	1.47	1.38	0.00	0.00	0.00	0.87	0.00	0.00	0.24	0.00	0.00	0.60	0.00	0.00	0.00	0.00	0.00	0.00	0.00	0.78	0.00	0.00	0.00	0.00	0.38	0.06	>0.999999999999999	5.9
P51452	Dual specificity protein phosphatase 3 OS=Homo sapiens GN=DUSP3 PE=1 SV=1	0.00	0.00	0.00	0.00	0.00	2.33	0.00	0.00	0.63	0.00	0.00	1.59	0.00	0.00	0.00	0.00	0.00	0.00	0.00	0.00	0.49	0.00	0.00	0.00	0.38	0.04	>0.999999999999999	9.2
Q96AG4	Leucine-rich repeat-containing protein 59 OS=Homo sapiens GN=LRRC59 PE=1 SV=1	0.00	0.00	1.01	0.00	0.00	1.31	0.00	1.43	0.79	0.00	0.00	0.00	0.00	0.00	1.00	0.00	0.00	0.00	0.00	0.00	0.00	0.00	0.00	0.00	0.38	0.08	>0.999999999999999	4.5
O00203	AP-3 complex subunit beta-1 OS=Homo sapiens GN=AP3B1 PE=1 SV=3	1.95	0.69	0.34	0.00	0.00	0.73	0.00	0.00	0.32	0.00	0.00	0.50	0.00	1.01	0.00	0.00	0.00	0.46	0.00	0.39	0.15	0.00	0.00	0.17	0.38	0.18	>0.999999999999999	2.1
Q96IZ0	PRKC apoptosis WT1 regulator protein OS=Homo sapiens GN=PAWR PE=1 SV=1	2.20	0.00	1.01	0.00	0.00	1.31	0.00	0.00	0.00	0.00	0.00	0.00	0.00	0.00	0.00	0.00	0.00	0.00	0.00	0.00	0.00	0.00	0.00	0.00	0.38	0.00	>0.999999999999999	#DIV/0!
Q01844	RNA-binding protein EWS OS=Homo sapiens GN=EWSR1 PE=1 SV=1	0.00	0.00	0.56	0.76	0.00	1.46	0.00	0.79	0.00	0.00	0.95	0.00	0.00	1.01	0.00	0.00	0.00	0.00	0.00	0.65	0.00	0.00	0.00	0.00	0.38	0.14	>0.999999999999999	2.7
Q9C005	Protein dpy-30 homolog OS=Homo sapiens GN=DPY30 PE=1 SV=1	0.00	0.00	0.00	0.00	0.00	4.51	0.00	0.00	0.00	0.00	0.00	0.00	0.00	0.00	0.00	0.00	0.00	0.00	0.00	0.00	0.00	0.00	0.00	0.00	0.38	0.00	>0.999999999999999	#DIV/0!
Q96GX2	Putative ataxin-7-like protein 3B OS=Homo sapiens GN=ATXN7L3B PE=3 SV=2	0.00	0.00	0.00	0.00	0.00	4.51	0.00	0.00	0.00	0.00	0.00	0.00	0.00	0.00	0.00	0.00	0.00	0.00	0.00	0.00	0.00	0.00	0.00	0.00	0.38	0.00	>0.999999999999999	#DIV/0!
Q15836	Vesicle-associated membrane protein 3 OS=Homo sapiens GN=VAMP3 PE=1 SV=3	0.00	0.00	0.00	0.00	0.00	4.51	0.00	0.00	0.00	0.00	0.00	0.00	0.00	0.00	0.00	0.00	0.00	0.00	0.00	0.00	0.00	0.00	0.00	0.00	0.38	0.00	>0.999999999999999	#DIV/0!
Q9P0J7	E3 ubiquitin-protein ligase KCMF1 OS=Homo sapiens GN=KCMF1 PE=1 SV=2	0.00	1.84	0.00	0.00	0.00	0.00	0.00	0.00	0.32	0.00	1.51	0.80	0.00	0.00	0.00	0.00	0.00	0.00	0.00	1.04	0.00	0.00	0.00	0.00	0.37	0.09	>0.999999999999999	4.3
Q9H5N1	Rab GTPase-binding effector protein 2 OS=Homo sapiens GN=RABEP2 PE=1 SV=2	0.00	1.15	0.00	0.00	0.00	2.33	0.00	0.79	0.20	0.00	0.00	0.00	0.00	0.00	0.00	0.00	0.00	0.00	0.00	0.00	0.00	0.00	0.00	0.00	0.37	0.00	>0.999999999999999	#DIV/0!
O15217	Glutathione S-transferase A4 OS=Homo sapiens GN=GSTA4 PE=1 SV=1	3.18	0.00	0.00	0.00	0.00	0.00	0.00	0.00	0.00	0.00	0.00	1.29	0.00	0.00	0.00	0.00	0.00	0.00	0.00	3.63	0.00	0.00	0.00	0.74	0.37	0.36	>0.999999999999999	1.0
P28799	Granulins OS=Homo sapiens GN=GRN PE=1 SV=2	0.00	2.30	0.00	0.00	0.00	0.00	0.84	0.79	0.00	0.52	0.00	0.00	0.00	1.01	0.00	0.00	0.94	0.77	0.00	1.94	0.00	0.00	0.77	0.00	0.37	0.45	>0.999999999999999	0.8
Q7LG56	Ribonucleoside-diphosphate reductase subunit M2 B OS=Homo sapiens GN=RRM2B PE=1	1.95	0.00	0.00	0.00	0.00	2.47	0.00	0.00	0.00	0.00	0.00	0.00	0.00	0.00	0.00	0.00	0.00	0.00	0.00	0.00	0.00	0.00	0.00	0.00	0.37	0.00	>0.999999999999999	#DIV/0!
Q92973	Transportin-1 OS=Homo sapiens GN=TNPO1 PE=1 SV=2	0.73	0.00	0.67	0.00	0.00	1.89	0.51	0.00	0.00	0.00	0.00	0.60	0.26	0.60	0.00	0.00	0.00	0.46	0.95	0.39	0.09	0.62	0.00	0.17	0.37	0.30	>0.999999999999999	1.2
Q9BXJ9	N-alpha-acetyltransferase 15, NatA auxiliary subunit OS=Homo sapiens GN=NAA15 PE=1 S	1.47	0.00	1.12	0.00	0.00	0.44	0.00	0.00	0.24	0.00	1.14	0.00	0.00	0.00	0.00	0.00	0.00	0.00	0.00	0.39	0.00	0.00	0.00	0.17	0.37	0.05	>0.999999999999999	7.9
Q9H773	dCTP pyrophosphatase 1 OS=Homo sapiens GN=DCTPP1 PE=1 SV=1	4.40	0.00	0.00	0.00	0.00	0.00	0.00	0.00	0.00	0.00	0.00	0.00	0.00	0.00	0.00	0.00	0.00	0.00	0.00	0.00	0.00	0.00	0.00	0.00	0.37	0.00	>0.999999999999999	#DIV/0!
O43252	Bifunctional 3'-phosphoadenosine 5'-phosphosulfate synthase 1 OS=Homo sapiens GN=PA	1.22	0.00	0.56	0.00	0.96	0.00	0.00	0.00	0.20	0.00	0.95	0.50	0.00	0.00	0.00	0.00	0.00	0.00	0.00	0.00	0.00	0.00	0.00	0.00	0.37	0.00	>0.999999999999999	#DIV/0!
Q8N573	Oxidation resistance protein 1 OS=Homo sapiens GN=OXR1 PE=1 SV=2	1.71	0.00	0.34	0.00	1.92	0.00	0.00	0.00	0.12	0.00	0.00	0.30	0.26	0.00	0.00	0.21	0.00	0.00	0.00	0.91	0.00	0.00	0.00	0.00	0.37	0.11	>0.999999999999999	3.2
Q5RHP9	Uncharacterized protein C1orf173 OS=Homo sapiens GN=C1orf173 PE=2 SV=1	1.95	0.92	0.00	0.00	0.00	0.29	0.00	0.63	0.00	0.00	0.38	0.20	0.00	0.00	0.00	0.00	0.00	0.00	0.00	0.00	0.00	0.00	0.00	0.00	0.36	0.00	>0.999999999999999	#DIV/0!
Q8NFW8	N-acylneuraminate cytidylyltransferase OS=Homo sapiens GN=CMAS PE=1 SV=2	0.00	0.00	0.00	0.00	0.00	4.37	0.00	0.00	0.00	0.00	0.00	0.00	0.61	0.00	0.00	0.48	0.00	1.07	0.00	1.81	0.00	0.00	0.00	0.00	0.36	0.33	>0.999999999999999	1.1
P01699	Ig lambda chain V-I region VOR OS=Homo sapiens PE=1 SV=1	0.00	0.00	0.00	0.00	0.00	4.37	0.00	0.00	0.00	0.00	0.00	0.00	0.00	0.00	0.00	0.00	0.00	0.00	0.00	0.00	0.00	0.00	0.00	0.00	0.36	0.00	>0.999999999999999	#DIV/0!
Q99436	Proteasome subunit beta type-7 OS=Homo sapiens GN=PSMB7 PE=1 SV=1	2.69	0.00	0.00	1.67	0.00	0.00	0.00	0.00	0.00	0.00	0.00	0.00	0.96	0.00	0.00	0.76	0.00	0.00	0.00	1.43	0.00	0.00	0.00	0.00	0.36	0.26	>0.999999999999999	1.4
Q86U42	Polyadenylate-binding protein 2 OS=Homo sapiens GN=PABPN1 PE=1 SV=3	0.00	0.00	0.00	0.00	0.00	1.46	0.00	0.00	0.00	0.00	1.89	1.00	0.00	0.00	0.00	0.00	0.00	0.00	0.00	0.00	0.00	0.00	0.00	0.00	0.36	0.00	>0.999999999999999	#DIV/0!
P29466	Caspase-1 OS=Homo sapiens GN=CASP1 PE=1 SV=1	0.00	0.00	0.00	0.00	0.00	3.35	0.00	0.00	0.28	0.00	0.00	0.70	0.00	0.00	0.00	0.00	0.00	0.00	0.00	1.94	0.00	0.00	0.00	0.40	0.36	0.20	>0.999999999999999	1.8
P13807	Glycogen [starch] synthase, muscle OS=Homo sapiens GN=GYS1 PE=1 SV=2	0.00	0.00	0.45	0.00	0.77	0.58	0.00	0.00	0.16	0.83	1.51	0.00	0.00	0.00	0.00	0.00	0.00	0.00	0.00	0.52	0.00	0.83	0.00	0.00	0.36	0.11	>0.999999999999999	3.2
P43243	Matrin-3 OS=Homo sapiens GN=MATR3 PE=1 SV=2	0.00	0.00	0.34	0.00	0.00	1.02	0.00	0.00	0.00	0.00	2.65	0.30	0.00	0.00	0.00	0.00	0.00	0.00	0.00	0.39	0.00	0.00	0.00	0.00	0.36	0.03	>0.999999999999999	11.1
O14645	Axonemal dynein light intermediate polypeptide 1 OS=Homo sapiens GN=DNALI1 PE=2 SV	2.69	0.00	0.00	0.00	0.00	1.60	0.00	0.00	0.00	0.00	0.00	0.00	0.00	0.00	0.00	0.00	0.00	0.00	0.00	0.00	0.00	0.00	0.00	0.00	0.36	0.00	>0.999999999999999	#DIV/0!
O95372	Acyl-protein thioesterase 2 OS=Homo sapiens GN=LYPLA2 PE=1 SV=1	3.18	0.00	0.00	0.00	0.00	0.00	0.00	0.00	1.11	0.00	0.00	0.00	0.00	2.62	0.00	0.00	0.00	0.00	0.00	0.00	0.00	0.00	0.00	0.74	0.36	0.28	>0.999999999999999	1.3
Q9H6Z4	Ran-binding protein 3 OS=Homo sapiens GN=RANBP3 PE=1 SV=1	0.00	1.15	0.00	0.00	0.96	0.73	0.00	0.00	0.00	0.00	0.95	0.50	0.00	0.00	0.00	0.00	0.00	0.00	0.00	1.43	0.00	0.00	0.00	0.28	0.36	0.14	>0.999999999999999	2.5
P21108	Ribose-phosphate pyrophosphokinase 3 OS=Homo sapiens GN=PRPS1L1 PE=1 SV=2	0.00	0.00	0.00	0.00	0.00	0.00	0.00	0.00	0.00	0.00	0.00	4.28	0.00	0.00	0.00	0.00	0.00	0.00	0.00	0.00	0.00	0.00	0.00	0.00	0.36	0.00	>0.999999999999999	#DIV/0!
P35813	Protein phosphatase 1A OS=Homo sapiens GN=PPM1A PE=1 SV=1	1.95	0.00	0.00	0.00	0.00	2.33	0.00	0.00	0.00	0.00	0.00	0.00	0.70	1.61	0.00	0.00	0.00	0.00	0.00	1.04	0.00	0.00	0.00	0.45	0.36	0.32	>0.999999999999999	1.1
Q6PD74	Alpha- and gamma-adaptin-binding protein p34 OS=Homo sapiens GN=AAGAB PE=1 SV=1	0.00	2.07	0.00	0.00	0.00	1.31	0.00	0.00	0.00	0.00	0.00	0.90	0.00	0.00	0.00	0.00	0.00	0.00	0.00	0.00	0.00	0.00	0.00	0.00	0.36	0.00	>0.999999999999999	#DIV/0!
Q15599	Na(+)/H(+) exchange regulatory cofactor NHE-RF2 OS=Homo sapiens GN=SLC9A3R2 PE=	2.20	2.07	0.00	0.00	0.00	0.00	0.00	0.00	0.00	0.00	0.00	0.00	0.00	0.00	0.00	0.62	0.00	0.00	0.00	0.00	0.00	0.00	0.00	0.00	0.36	0.05	>0.999999999999999	6.9
P01594	Ig kappa chain V-I region AU OS=Homo sapiens PE=1 SV=1	0.00	0.00	0.00	4.25	0.00	0.00	0.00	0.00	0.00	0.00	0.00	0.00	0.00	0.00	0.00	0.00	0.00	0.00	0.00	0.00	0.86	0.00	0.00	0.00	0.35	0.07	>0.999999999999999	4.9
Q9UHD9	Ubiquilin-2 OS=Homo sapiens GN=UBQLN2 PE=1 SV=2	0.00	1.15	0.00	0.00	0.00	1.46	0.84	0.79	0.00	0.00	0.00	0.00	0.88	1.01	0.00	0.34	0.00	0.00	0.00	1.30	0.15	0.00	0.77	0.00	0.35	0.37	>0.999999999999999	1.0
Q96AX2	Ras-related protein Rab-37 OS=Homo sapiens GN=RAB37 PE=1 SV=3	0.00	0.00	0.00	0.00	0.00	4.22	0.00	0.00	0.00	0.00	0.00	0.00	0.00	0.00	0.00	0.00	0.00	0.00	0.00	0.00	0.00	0.00	0.00	0.00	0.35	0.00	>0.999999999999999	#DIV/0!
Q9UMX0	Ubiquilin-1 OS=Homo sapiens GN=UBQLN1 PE=1 SV=2	0.00	0.00	0.56	0.00	0.00	0.00	0.00	0.00	0.44	0.00	3.22	0.00	0.00	2.22	0.00	0.00	0.00	0.00	0.00	0.00	0.00	0.00	0.00	0.28	0.35	0.21	>0.999999999999999	1.7
A1Z1Q3	MACRO domain-containing protein 2 OS=Homo sapiens GN=MACROD2 PE=1 SV=1	3.18	0.00	0.00	0.00	0.00	1.02	0.00	0.00	0.00	0.00	0.00	0.00	0.00	0.00	0.00	0.00	0.00	0.00	0.00	0.00	0.00	0.00	0.00	0.00	0.35	0.00	>0.999999999999999	#DIV/0!
P62253	Ubiquitin-conjugating enzyme E2 G1 OS=Homo sapiens GN=UBE2G1 PE=1 SV=3	0.00	0.00	0.00	0.00	0.00	2.47	0.00	0.00	0.00	0.00	0.00	1.69	0.00	0.00	0.00	0.00	0.00	0.00	0.00	0.00	0.00	0.00	0.00	0.00	0.35	0.00	>0.999999999999999	#DIV/0!
Q92900	Regulator of nonsense transcripts 1 OS=Homo sapiens GN=UPF1 PE=1 SV=2	0.73	0.00	0.00	0.00	0.58	1.16	0.51	0.00	0.00	0.31	0.57	0.30	0.00	0.00	0.00	0.00	0.00	0.00	0.00	0.00	0.00	0.00	0.00	0.00	0.35	0.00	>0.999999999999999	#DIV/0!
P34913	Epoxide hydrolase 2 OS=Homo sapiens GN=EPHX2 PE=1 SV=2	1.22	0.00	0.56	0.00	0.00	0.73	0.00	0.00	0.20	0.00	0.95	0.50	0.00	0.00	0.00	0.00	0.00	0.77	0.00	0.00	0.00	0.00	0.00	0.00	0.35	0.06	>0.999999999999999	5.4
Q86V88	Magnesium-dependent phosphatase 1 OS=Homo sapiens GN=MDP1 PE=1 SV=1	4.15	0.00	0.00	0.00	0.00	0.00	0.00	0.00	0.00	0.00	0.00	0.00	0.00	0.00	0.00	0.00	0.00	0.00	0.00	0.00	0.00	0.00	0.00	0.00	0.35	0.00	>0.999999999999999	#DIV/0!
O00629	Importin subunit alpha-4 OS=Homo sapiens GN=KPNA4 PE=1 SV=1	1.47	0.00	0.67	0.00	0.00	0.87	0.00	0.00	0.00	0.00	1.14	0.00	0.00	0.00	0.00	0.00	0.00	0.00	0.00	0.78	0.00	0.00	0.00	0.00	0.35	0.06	>0.999999999999999	5.3
Q13790	Apolipoprotein F OS=Homo sapiens GN=APOF PE=1 SV=2	0.00	0.00	0.00	0.00	0.00	0.00	3.21	0.00	0.00	0.94	0.00	0.00	0.79	0.00	1.00	0.00	0.00	0.00	1.43	0.00	0.59	3.95	1.38	0.00	0.35	0.76	>0.999999999999999	0.5
Q96NG3	Tetratricopeptide repeat protein 25 OS=Homo sapiens GN=TTC25 PE=1 SV=2	0.98	0.00	0.00	0.00	1.73	0.00	0.67	0.00	0.00	0.00	0.76	0.00	0.00	0.00	0.00	0.00	0.00	0.00	0.00	0.00	0.00	0.00	0.00	0.00	0.34	0.00	>0.999999999999999	#DIV/0!
Q9UBC2	Epidermal growth factor receptor substrate 15-like 1 OS=Homo sapiens GN=EPS15L1 PE=1	2.69	0.69	0.34	0.00	0.00	0.00	0.00	0.00	0.12	0.00	0.00	0.30	0.00	0.00	0.00	0.00	0.00	0.00	0.00	0.00	0.00	0.00	0.00	0.00	0.34	0.00	>0.999999999999999	#DIV/0!
P30461	HLA class I histocompatibility antigen, B-13 alpha chain OS=Homo sapiens GN=HLA-B PE=2	0.00	0.00	0.00	0.00	0.00	0.00	0.00	4.12	0.00	0.00	0.00	0.00	0.00	0.00	0.00	0.00	0.00	0.00	0.00	0.00	0.00	0.00	0.00	0.00	0.34	0.00	>0.999999999999999	#DIV/0!
Q9Y3A5	Ribosome maturation protein SBDS OS=Homo sapiens GN=SBDS PE=1 SV=4	0.00	0.00	0.00	0.00	0.00	1.60	0.00	0.00	0.44	0.00	2.08	0.00	0.00	0.00	0.00	0.00	0.00	0.00	0.00	1.43	0.00	0.00	0.00	0.00	0.34	0.12	>0.999999999999999	2.9
Q8IZP0	Abl interactor 1 OS=Homo sapiens GN=ABI1 PE=1 SV=4	0.00	1.38	0.00	0.00	0.00	0.87	1.01	0.00	0.24	0.00	0.00	0.60	0.53	1.21	0.00	0.00	0.00	0.00	0.00	0.00	0.00	1.25	0.00	0.34	0.34	0.28	>0.999999999999999	1.2
Q99747	Gamma-soluble NSF attachment protein OS=Homo sapiens GN=NAPG PE=1 SV=1	2.20	0.00	1.01	0.00	0.00	0.00	0.00	0.00	0.00	0.00	0.00	0.90	0.00	1.81	0.00	0.00	0.00	0.00	0.00	1.17	0.00	0.00	0.00	1.14	0.34	0.34	>0.999999999999999	1.0
P19957	Elafin OS=Homo sapiens GN=PI3 PE=1 SV=3	0.00	0.00	0.00	4.09	0.00	0.00	0.00	0.00	0.00	0.00	0.00	0.00	0.00	12.29	0.00	1.86	5.09	0.00	0.00	0.00	0.00	0.00	0.00	1.53	0.34	1.73	>0.999999999999999	0.2
Q14244	Ensconsin OS=Homo sapiens GN=MAP7 PE=1 SV=1	0.98	0.92	0.45	0.00	0.00	0.58	0.00	0.00	0.00	0.00	0.76	0.40	0.00	0.81	0.00	0.28	0.00	0.61	0.00	1.04	0.00	0.00	0.00	0.45	0.34	0.27	>0.999999999999999	1.3
P19525	Interferon-induced, double-stranded RNA-activated protein kinase OS=Homo sapiens GN=E	0.00	1.15	0.00	0.76	0.00	0.73	0.00	0.00	0.00	0.00	0.95	0.50	0.00	0.00	0.00	0.00	0.00	0.00	0.00	0.65	0.00	0.00	0.00	0.00	0.34	0.05	>0.999999999999999	6.3
O00170	AH receptor-interacting protein OS=Homo sapiens GN=AIP PE=1 SV=2	0.00	0.00	0.00	0.00	0.00	1.31	1.52	0.00	0.36	0.00	0.00	0.90	0.00	3.63	0.00	0.00	0.00	0.00	0.00	1.17	0.28	0.00	0.00	0.51	0.34	0.47	>0.999999999999999	0.7
Q9UJC5	SH3 domain-binding glutamic acid-rich-like protein 2 OS=Homo sapiens GN=SH3BGRL2 PE	0.00	0.00	0.00	0.00	0.00	4.08	0.00	0.00	0.00	0.00	0.00	0.00	0.00	5.64	0.00	0.00	0.00	0.00	0.00	0.00	0.00	0.00	0.00	1.59	0.34	0.60	>0.999999999999999	0.6
Q53GD3	Choline transporter-like protein 4 OS=Homo sapiens GN=SLC44A4 PE=2 SV=2	0.98	0.92	0.00	0.00	0.77	0.00	0.00	0.63	0.00	0.00	0.76	0.00	0.00	0.00	0.00	0.00	0.00	0.00	0.00	0.00	0.12	0.00	0.00	0.23	0.34	0.03	>0.999999999999999	11.6
P54819	Adenylate kinase 2, mitochondrial OS=Homo sapiens GN=AK2 PE=1 SV=2	0.00	0.00	1.46	0.00	0.00	0.00	0.00	2.06	0.52	0.00	0.00	0.00	0.00	0.00	0.00	0.00	0.00	0.00	0.00	0.00	0.00	0.00	0.00	0.00	0.34	0.00	>0.999999999999999	#DIV/0!
O00429	Dynamin-1-like protein OS=Homo sapiens GN=DNM1L PE=1 SV=2	0.98	0.00	0.00	0.00	0.00	0.58	0.00	0.00	0.16	0.00	1.51	0.80	0.35	0.00	0.00	0.00	0.00	0.00	0.00	0.52	0.00	0.00	0.00	0.00	0.34	0.07	>0.999999999999999	4.6
P00387	NADH-cytochrome b5 reductase 3 OS=Homo sapiens GN=CYB5R3 PE=1 SV=3	2.44	0.00	0.00	0.00	0.00	0.00	0.00	1.59	0.00	0.00	0.00	0.00	0.00	4.03	0.00	0.00	0.00	1.53	0.00	0.00	0.31	0.00	0.00	0.57	0.34	0.54	>0.999999999999999	0.6
O95396	Adenylyltransferase and sulfurtransferase MOCS3 OS=Homo sapiens GN=MOCS3 PE=1 SV	1.71	1.61	0.00	0.00	0.00	0.00	0.00	0.00	0.00	0.00	0.00	0.70	0.00	0.00	0.00	0.00	0.00	0.00	0.00	0.00	0.00	0.00	0.00	0.40	0.34	0.03	>0.999999999999999	10.1
O75534	Cold shock domain-containing protein E1 OS=Homo sapiens GN=CSDE1 PE=1 SV=2	0.98	0.92	0.00	0.00	0.00	1.02	0.00	0.00	0.00	0.00	0.00	1.10	0.00	0.81	0.44	0.00	0.00	0.61	0.00	0.91	0.00	0.00	0.00	0.00	0.33	0.23	>0.999999999999999	1.4
O43765	Small glutamine-rich tetratricopeptide repeat-containing protein alpha OS=Homo sapiens GN	0.00	0.00	1.12	0.00	0.00	1.46	0.00	0.00	0.40	0.00	0.00	1.00	0.00	2.01	0.00	0.00	0.00	0.00	0.00	4.15	0.00	0.00	0.00	0.57	0.33	0.56	>0.999999999999999	0.6
Q07666	KH domain-containing, RNA-binding, signal transduction-associated protein 1 OS=Homo sa	0.00	1.61	0.00	0.00	0.00	1.02	0.00	0.00	0.00	0.00	1.33	0.00	0.00	0.00	0.00	0.00	0.00	0.00	0.00	0.00	0.00	0.00	0.00	0.00	0.33	0.00	>0.999999999999999	#DIV/0!
P38159	RNA-binding motif protein, X chromosome OS=Homo sapiens GN=RBMX PE=1 SV=3	0.00	0.00	0.00	0.00	0.00	3.64	0.00	0.00	0.32	0.00	0.00	0.00	0.00	0.00	0.00	0.00	0.00	0.00	0.00	0.00	0.00	0.00	0.00	0.00	0.33	0.00	>0.999999999999999	#DIV/0!
Q93008	Probable ubiquitin carboxyl-terminal hydrolase FAF-X OS=Homo sapiens GN=USP9X PE=1	1.47	0.46	0.11	0.30	0.00	0.44	0.00	0.00	0.00	0.00	0.76	0.40	0.00	0.20	0.11	0.00	0.00	0.15	0.00	0.26	0.00	0.00	0.00	0.00	0.33	0.06	>0.999999999999999	5.4
O95758	Polypyrimidine tract-binding protein 3 OS=Homo sapiens GN=PTBP3 PE=1 SV=2	0.00	0.00	0.56	0.00	0.00	0.00	0.00	0.79	0.00	0.00	2.08	0.50	0.00	0.00	0.00	0.00	0.00	0.00	0.00	0.00	0.00	0.00	0.00	0.00	0.33	0.00	>0.999999999999999	#DIV/0!
Q9TNN7	HLA class I histocompatibility antigen, Cw-5 alpha chain OS=Homo sapiens GN=HLA-C PE=	0.00	3.92	0.00	0.00	0.00	0.00	0.00	0.00	0.00	0.00	0.00	0.00	0.00	0.00	0.00	0.00	0.00	1.23	0.00	0.00	0.00	0.00	0.00	0.00	0.33	0.10	>0.999999999999999	3.2
O43414	ERI1 exoribonuclease 3 OS=Homo sapiens GN=ERI3 PE=1 SV=2	0.00	0.00	0.00	0.00	0.00	1.31	0.00	0.00	0.00	0.00	1.70	0.90	0.00	0.00	0.00	0.00	0.00	0.00	0.00	0.00	0.00	0.00	0.00	0.00	0.33	0.00	>0.999999999999999	#DIV/0!
Q9NRV9	Heme-binding protein 1 OS=Homo sapiens GN=HEBP1 PE=1 SV=1	3.91	0.00	0.00	0.00	0.00	0.00	0.00	0.00	0.00	0.00	0.00	0.00	0.00	0.00	0.00	0.00	0.00	0.00	0.00	0.00	0.00	0.00	0.00	0.00	0.33	0.00	>0.999999999999999	#DIV/0!
Q96IM9	DPY30 domain-containing protein 2 OS=Homo sapiens GN=DYDC2 PE=2 SV=1	3.91	0.00	0.00	0.00	0.00	0.00	0.00	0.00	0.00	0.00	0.00	0.00	0.00	0.00	0.00	0.00	0.00	0.00	0.00	0.00	0.00	0.00	0.00	0.00	0.33	0.00	>0.999999999999999	#DIV/0!
P32321	Deoxycytidylate deaminase OS=Homo sapiens GN=DCTD PE=1 SV=2	3.91	0.00	0.00	0.00	0.00	0.00	0.00	0.00	0.00	0.00	0.00	0.00	0.00	0.00	0.00	0.00	0.00	0.00	0.00	0.00	0.00	0.00	0.00	0.00	0.33	0.00	>0.999999999999999	#DIV/0!
Q08AM6	Protein VAC14 homolog OS=Homo sapiens GN=VAC14 PE=1 SV=1	0.98	0.00	0.45	0.00	0.00	1.16	0.00	0.00	0.16	0.00	0.76	0.40	0.00	0.00	0.00	0.00	0.00	0.00	0.00	0.52	0.12	0.00	0.00	0.23	0.33	0.07	>0.999999999999999	4.5
Q96BK5	PIN2/TERF1-interacting telomerase inhibitor 1 OS=Homo sapiens GN=PINX1 PE=1 SV=2	2.20	0.00	0.00	0.00	0.00	0.00	0.00	0.00	0.00	0.00	1.70	0.00	0.00	0.00	0.00	0.62	0.00	0.00	0.00	1.17	0.00	0.00	0.00	0.00	0.33	0.15	>0.999999999999999	2.2
P35030	Trypsin-3 OS=Homo sapiens GN=PRSS3 PE=1 SV=2	2.44	0.00	0.00	0.00	0.00	1.46	0.00	0.00	0.00	0.00	0.00	0.00	0.00	0.00	0.00	0.00	0.00	0.00	0.00	0.00	0.00	0.00	0.00	0.00	0.32	0.00	>0.999999999999999	#DIV/0!
Q9UK22	F-box only protein 2 OS=Homo sapiens GN=FBXO2 PE=1 SV=2	2.44	0.00	0.00	0.00	0.00	1.46	0.00	0.00	0.00	0.00	0.00	0.00	0.00	0.00	0.00	0.00	0.00	0.00	0.00	0.00	0.00	0.00	0.00	0.00	0.32	0.00	>0.999999999999999	#DIV/0!
Q9NUJ1	Abhydrolase domain-containing protein 10, mitochondrial OS=Homo sapiens GN=ABHD10 F	2.44	0.00	0.00	0.00	0.00	1.46	0.00	0.00	0.00	0.00	0.00	0.00	0.88	0.00	0.00	0.00	0.00	0.00	0.00	0.00	0.00	0.00	0.00	0.00	0.32	0.07	>0.999999999999999	4.4
Q14409	Putative glycerol kinase 3 OS=Homo sapiens GN=GK3P PE=5 SV=2	0.00	1.15	0.56	0.00	0.00	0.00	0.00	0.79	0.44	0.00	0.95	0.00	0.00	2.22	0.00	0.00	0.00	0.77	0.00	0.65	0.00	0.00	0.77	0.28	0.32	0.39	>0.999999999999999	0.8
Q9BUH6	Uncharacterized protein C9orf142 OS=Homo sapiens GN=C9orf142 PE=1 SV=2	0.00	0.00	0.00	0.00	0.00	0.00	0.00	0.00	0.59	0.00	0.00	3.29	0.00	0.00	0.00	1.03	0.00	0.00	0.00	0.00	0.00	0.00	0.00	0.00	0.32	0.09	>0.999999999999999	3.8
O75602	Sperm-associated antigen 6 OS=Homo sapiens GN=SPAG6 PE=1 SV=1	1.47	0.00	0.67	0.00	0.00	0.00	0.00	0.00	0.00	0.00	1.14	0.60	0.00	0.00	0.00	0.00	0.00	0.00	0.00	0.00	0.00	0.00	0.00	0.00	0.32	0.00	>0.999999999999999	#DIV/0!
P10319	HLA class I histocompatibility antigen, B-58 alpha chain OS=Homo sapiens GN=HLA-B PE=2	0.00	0.00	0.00	0.00	0.00	0.00	0.00	0.00	0.00	3.86	0.00	0.00	0.00	0.00	0.00	0.00	0.00	0.00	0.00	0.00	0.00	0.00	0.00	0.00	0.32	0.00	>0.999999999999999	#DIV/0!
P12259	Coagulation factor V OS=Homo sapiens GN=F5 PE=1 SV=4	0.00	0.00	0.34	0.45	0.77	0.15	1.18	0.63	0.04	0.10	0.19	0.00	0.00	0.00	0.00	0.00	0.75	0.15	0.63	0.00	0.09	0.00	0.15	0.17	0.32	0.16	>0.999999999999999	2.0
Q9NQT8	Kinesin-like protein KIF13B OS=Homo sapiens GN=KIF13B PE=1 SV=1	0.73	0.69	0.22	0.30	0.00	1.02	0.00	0.00	0.20	0.00	0.38	0.30	0.18	0.40	0.00	0.00	0.00	0.31	0.00	0.91	0.00	0.00	0.00	0.11	0.32	0.16	>0.999999999999999	2.0
Q15427	Splicing factor 3B subunit 4 OS=Homo sapiens GN=SF3B4 PE=1 SV=1	1.71	0.00	0.00	0.00	0.00	1.02	0.00	1.11	0.00	0.00	0.00	0.00	0.00	0.00	0.00	0.00	0.00	1.07	0.00	0.00	0.00	1.45	1.07	0.00	0.32	0.30	>0.999999999999999	1.1
O00178	GTP-binding protein 1 OS=Homo sapiens GN=GTPBP1 PE=1 SV=3	2.20	0.00	0.45	0.61	0.00	0.58	0.00	0.00	0.00	0.00	0.00	0.00	0.35	0.81	0.00	0.00	0.00	0.61	0.00	0.52	0.00	0.00	0.00	0.00	0.32	0.19	>0.999999999999999	1.7
A0AVF1	Tetratricopeptide repeat protein 26 OS=Homo sapiens GN=TTC26 PE=2 SV=1	1.22	1.15	0.00	0.00	0.00	1.46	0.00	0.00	0.00	0.00	0.00	0.00	0.00	0.00	0.00	0.00	0.00	0.00	0.00	0.00	0.00	0.00	0.00	0.00	0.32	0.00	>0.999999999999999	#DIV/0!
P02735	Serum amyloid A protein OS=Homo sapiens GN=SAA1 PE=1 SV=2	0.00	0.00	2.80	0.00	0.00	0.00	0.00	0.00	0.99	0.00	0.00	0.00	0.00	5.04	6.33	0.00	4.71	0.00	0.00	0.00	0.77	0.00	0.00	0.00	0.32	1.40	>0.999999999999999	0.2
Q9GZM3	DNA-directed RNA polymerase II subunit RPB11-b1 OS=Homo sapiens GN=POLR2J2 PE=1	0.00	0.00	0.00	0.00	0.00	3.78	0.00	0.00	0.00	0.00	0.00	0.00	0.00	0.00	0.00	0.00	0.00	0.00	0.00	0.00	0.00	0.00	0.00	0.00	0.32	0.00	>0.999999999999999	#DIV/0!
Q96LJ8	UBX domain-containing protein 10 OS=Homo sapiens GN=UBXN10 PE=2 SV=1	2.69	0.00	0.00	0.00	0.00	0.00	0.00	0.00	0.00	0.00	0.00	1.10	0.00	0.00	0.00	0.00	0.00	0.00	0.00	0.00	0.00	0.00	0.00	0.00	0.32	0.00	>0.999999999999999	#DIV/0!
Q8TF05	Serine/threonine-protein phosphatase 4 regulatory subunit 1 OS=Homo sapiens GN=PPP4F	2.20	0.69	0.34	0.00	0.00	0.44	0.00	0.00	0.12	0.00	0.00	0.00	0.26	0.60	0.33	0.00	0.00	0.00	0.00	0.78	0.00	0.00	0.46	0.00	0.32	0.20	>0.999999999999999	1.6
Q5HYA8	Meckelin OS=Homo sapiens GN=TMEM67 PE=1 SV=2	0.73	0.69	0.00	0.00	0.58	0.44	0.00	0.48	0.00	0.00	0.57	0.30	0.00	0.60	0.00	0.00	0.00	0.00	0.00	0.00	0.00	0.62	0.00	0.17	0.31	0.12	>0.999999999999999	2.7
O43795	Unconventional myosin-Ib OS=Homo sapiens GN=MYO1B PE=2 SV=3	0.49	1.15	0.56	0.00	0.38	0.29	0.00	0.32	0.08	0.00	0.00	0.50	0.00	0.40	0.00	0.00	0.00	0.00	0.00	0.26	0.06	0.00	0.00	0.11	0.31	0.07	>0.999999999999999	4.5
O60613	15 kDa selenoprotein OS=Homo sapiens GN=SEP15 PE=1 SV=3	0.00	0.00	0.00	0.00	0.00	0.00	0.00	3.01	0.75	0.00	0.00	0.00	1.67	0.00	0.00	0.00	0.00	0.00	0.00	0.00	0.00	0.00	0.00	0.00	0.31	0.14	>0.999999999999999	2.3
P15924	Desmoplakin OS=Homo sapiens GN=DSP PE=1 SV=3	0.24	0.23	0.11	0.00	0.00	0.29	0.17	0.32	0.04	1.98	0.38	0.00	0.09	0.00	0.11	0.07	0.19	0.00	0.00	0.00	0.03	0.00	0.00	0.11	0.31	0.05	>0.999999999999999	6.3
P50897	Palmitoyl-protein thioesterase 1 OS=Homo sapiens GN=PPT1 PE=1 SV=1	0.00	2.30	0.00	0.00	0.00	1.46	0.00	0.00	0.00	0.00	0.00	0.00	0.00	2.01	0.00	0.00	0.00	0.00	0.00	0.00	0.00	0.00	0.00	0.00	0.31	0.17	>0.999999999999999	1.9
Q29960	HLA class I histocompatibility antigen, Cw-16 alpha chain OS=Homo sapiens GN=HLA-C PE	0.00	0.00	0.00	0.00	0.00	0.00	0.00	0.00	0.00	3.76	0.00	0.00	0.00	0.00	0.00	0.00	0.00	0.00	0.00	0.00	0.00	0.00	0.00	0.00	0.31	0.00	>0.999999999999999	#DIV/0!
P41240	Tyrosine-protein kinase CSK OS=Homo sapiens GN=CSK PE=1 SV=1	0.00	0.00	0.00	0.91	0.00	0.87	0.00	0.00	0.24	0.00	1.14	0.60	0.00	1.21	0.00	0.00	0.00	0.00	0.00	0.00	0.00	0.00	0.92	0.34	0.31	0.21	>0.999999999999999	1.5
Q9NUQ8	ATP-binding cassette sub-family F member 3 OS=Homo sapiens GN=ABCF3 PE=1 SV=2	0.00	0.92	0.45	0.61	0.00	0.58	0.00	0.63	0.16	0.00	0.00	0.40	0.00	0.81	0.00	0.00	0.00	0.00	0.00	0.52	0.12	0.00	0.00	0.23	0.31	0.14	>0.999999999999999	2.2
Q8NEP3	Dynein assembly factor 1, axonemal OS=Homo sapiens GN=DNAAF1 PE=1 SV=5	0.00	0.92	0.00	0.00	0.00	0.00	0.00	0.00	0.00	0.00	1.51	1.29	0.00	0.00	0.00	0.00	0.00	0.00	0.00	0.00	0.00	0.00	0.00	0.00	0.31	0.00	>0.999999999999999	#DIV/0!
P62841	40S ribosomal protein S15 OS=Homo sapiens GN=RPS15 PE=1 SV=2	0.00	0.00	0.00	0.00	0.00	2.91	0.00	0.00	0.79	0.00	0.00	0.00	0.00	0.00	0.00	0.00	0.00	0.00	0.00	0.00	0.00	0.00	0.00	0.00	0.31	0.00	>0.999999999999999	#DIV/0!
Q53FT3	Uncharacterized protein C11orf73 OS=Homo sapiens GN=C11orf73 PE=1 SV=2	0.00	3.69	0.00	0.00	0.00	0.00	0.00	0.00	0.00	0.00	0.00	0.00	0.00	3.22	0.00	0.00	0.00	0.00	2.07	0.00	0.00	0.00	0.00	0.00	0.31	0.44	>0.999999999999999	0.7
P53602	Diphosphomevalonate decarboxylase OS=Homo sapiens GN=MVD PE=1 SV=1	0.00	3.69	0.00	0.00	0.00	0.00	0.00	0.00	0.00	0.00	0.00	0.00	0.00	0.00	0.00	0.00	0.00	0.00	0.00	0.00	0.00	0.00	0.00	0.00	0.31	0.00	>0.999999999999999	#DIV/0!
Q969H4	Connector enhancer of kinase suppressor of ras 1 OS=Homo sapiens GN=CNKSR1 PE=1 S	0.98	0.92	0.45	0.00	0.00	0.58	0.00	0.00	0.00	0.00	0.76	0.00	0.00	0.00	0.00	0.00	0.00	0.00	0.00	0.00	0.00	0.00	0.00	0.00	0.31	0.00	>0.999999999999999	#DIV/0!
Q9Y6G5	COMM domain-containing protein 10 OS=Homo sapiens GN=COMMD10 PE=1 SV=1	0.00	0.00	0.00	0.00	0.00	2.18	0.00	0.00	0.00	0.00	0.00	1.49	0.00	0.00	0.00	0.00	0.00	0.00	0.00	0.00	0.00	0.00	0.00	0.00	0.31	0.00	>0.999999999999999	#DIV/0!
Q9H0A8	COMM domain-containing protein 4 OS=Homo sapiens GN=COMMD4 PE=1 SV=1	3.66	0.00	0.00	0.00	0.00	0.00	0.00	0.00	0.00	0.00	0.00	0.00	0.00	0.00	0.00	0.00	0.00	0.00	0.00	0.00	0.00	0.00	0.00	0.00	0.31	0.00	>0.999999999999999	#DIV/0!
Q86X83	COMM domain-containing protein 2 OS=Homo sapiens GN=COMMD2 PE=1 SV=2	3.66	0.00	0.00	0.00	0.00	0.00	0.00	0.00	0.00	0.00	0.00	0.00	0.00	0.00	0.00	0.00	0.00	0.00	0.00	0.00	0.00	0.00	0.00	0.00	0.31	0.00	>0.999999999999999	#DIV/0!
O95352	Ubiquitin-like modifier-activating enzyme ATG7 OS=Homo sapiens GN=ATG7 PE=1 SV=1	0.00	0.00	0.45	0.00	0.00	1.89	0.00	0.00	0.16	0.00	0.76	0.40	0.00	1.61	0.44	0.00	0.00	0.00	0.00	0.00	0.12	0.83	0.00	0.23	0.30	0.27	>0.999999999999999	1.1
P46060	Ran GTPase-activating protein 1 OS=Homo sapiens GN=RANGAP1 PE=1 SV=1	0.00	0.00	0.00	0.76	0.00	1.60	0.00	0.00	0.20	0.00	0.00	1.10	0.00	1.01	0.00	0.00	0.00	0.77	0.00	2.07	0.00	0.00	0.00	0.62	0.30	0.37	>0.999999999999999	0.8
P50453	Serpin B9 OS=Homo sapiens GN=SERPINB9 PE=1 SV=1	0.00	0.00	0.00	0.00	0.00	3.64	0.00	0.00	0.00	0.00	0.00	0.00	0.00	0.00	0.00	0.00	0.00	0.00	0.00	0.00	0.00	0.00	0.00	0.00	0.30	0.00	>0.999999999999999	#DIV/0!
O75348	V-type proton ATPase subunit G 1 OS=Homo sapiens GN=ATP6V1G1 PE=1 SV=3	0.00	0.00	0.00	0.00	0.00	3.64	0.00	0.00	0.00	0.00	0.00	0.00	0.00	0.00	0.00	0.00	0.00	0.00	0.00	0.00	0.00	0.00	0.00	0.00	0.30	0.00	>0.999999999999999	#DIV/0!
Q5JS54	Proteasome assembly chaperone 4 OS=Homo sapiens GN=PSMG4 PE=2 SV=2	0.00	0.00	0.00	0.00	0.00	3.64	0.00	0.00	0.00	0.00	0.00	0.00	0.00	0.00	0.00	0.00	0.00	0.00	0.00	0.00	0.00	0.00	0.00	0.00	0.30	0.00	>0.999999999999999	#DIV/0!
P51808	Dynein light chain Tctex-type 3 OS=Homo sapiens GN=DYNLT3 PE=1 SV=1	0.00	0.00	0.00	0.00	0.00	3.64	0.00	0.00	0.00	0.00	0.00	0.00	0.00	0.00	2.78	0.00	0.00	0.00	0.00	3.24	0.00	0.00	0.00	0.00	0.30	0.50	>0.999999999999999	0.6
Q9Y2Q5	Ragulator complex protein LAMTOR2 OS=Homo sapiens GN=LAMTOR2 PE=1 SV=1	0.00	0.00	0.00	0.00	0.00	3.64	0.00	0.00	0.00	0.00	0.00	0.00	0.00	0.00	0.00	0.00	0.00	3.83	0.00	0.00	0.00	5.19	0.00	0.00	0.30	0.75	>0.999999999999999	0.4
O75882	Attractin OS=Homo sapiens GN=ATRN PE=1 SV=2	0.00	0.00	0.45	0.61	0.38	0.29	0.34	0.32	0.08	0.21	0.76	0.20	0.18	0.00	0.44	0.14	1.51	0.00	0.00	0.26	0.00	0.42	0.31	0.11	0.30	0.28	>0.999999999999999	1.1
P31937	3-hydroxyisobutyrate dehydrogenase, mitochondrial OS=Homo sapiens GN=HIBADH PE=1	2.20	0.00	0.00	0.00	0.00	0.00	0.00	1.43	0.00	0.00	0.00	0.00	0.79	0.00	0.00	0.00	0.00	0.00	0.00	0.00	0.00	0.00	0.00	0.00	0.30	0.07	>0.999999999999999	4.6
P62879	Guanine nucleotide-binding protein G(I)/G(S)/G(T) subunit beta-2 OS=Homo sapiens GN=GN	2.20	0.00	0.00	0.00	0.00	0.00	0.00	1.43	0.00	0.00	0.00	0.00	0.00	0.00	0.00	0.00	0.00	0.00	0.00	0.00	0.00	0.00	0.00	0.00	0.30	0.00	>0.999999999999999	#DIV/0!
Q9Y2W2	WW domain-binding protein 11 OS=Homo sapiens GN=WBP11 PE=1 SV=1	1.22	0.00	0.00	0.00	0.00	0.00	0.00	0.00	0.00	0.00	1.89	0.50	0.00	0.00	0.00	0.00	0.00	0.00	0.00	0.65	0.00	0.00	0.00	0.00	0.30	0.05	>0.999999999999999	5.6
P05023	Sodium/potassium-transporting ATPase subunit alpha-1 OS=Homo sapiens GN=ATP1A1 PE	0.73	0.00	0.34	0.00	0.00	0.87	0.00	1.43	0.24	0.00	0.00	0.00	0.26	0.60	0.33	0.21	0.00	0.00	0.95	0.78	0.00	0.00	0.00	0.00	0.30	0.26	>0.999999999999999	1.2
O15195	Villin-like protein OS=Homo sapiens GN=VILL PE=2 SV=3	0.73	0.00	0.00	0.00	0.00	1.60	0.00	0.00	0.00	0.00	0.57	0.70	0.00	0.60	0.00	0.00	0.00	0.00	0.00	0.39	0.00	0.00	0.00	0.17	0.30	0.10	>0.999999999999999	3.1
Q96HJ3	Coiled-coil domain-containing protein 34 OS=Homo sapiens GN=CCDC34 PE=2 SV=2	0.00	0.00	0.90	0.00	1.53	1.16	0.00	0.00	0.00	0.00	0.00	0.00	0.00	0.00	0.00	0.00	0.00	1.23	0.00	0.00	0.00	0.00	0.00	0.00	0.30	0.10	>0.999999999999999	2.9
Q9NZB2	Constitutive coactivator of PPAR-gamma-like protein 1 OS=Homo sapiens GN=FAM120A PE	1.22	0.69	0.00	0.00	0.00	0.44	0.00	0.00	0.00	0.00	0.95	0.30	0.00	0.00	0.00	0.00	0.00	0.00	0.00	0.00	0.00	0.00	0.00	0.00	0.30	0.00	>0.999999999999999	#DIV/0!
Q9Y295	Developmentally-regulated GTP-binding protein 1 OS=Homo sapiens GN=DRG1 PE=1 SV=1	0.00	0.00	0.00	0.00	0.00	2.47	0.00	0.00	0.32	0.00	0.00	0.80	0.00	1.61	0.00	0.00	0.00	0.00	0.00	0.00	0.00	0.00	0.00	0.45	0.30	0.17	>0.999999999999999	1.7
A6NI72	Putative neutrophil cytosol factor 1B OS=Homo sapiens GN=NCF1B PE=5 SV=2	0.00	0.00	0.00	1.06	0.00	0.00	0.00	0.00	0.00	2.50	0.00	0.00	0.00	0.00	0.00	0.00	0.00	0.00	0.00	0.00	0.00	3.12	2.30	0.00	0.30	0.45	>0.999999999999999	0.7
Q15293	Reticulocalbin-1 OS=Homo sapiens GN=RCN1 PE=1 SV=1	0.00	0.00	0.00	0.00	0.00	2.62	0.00	0.00	0.00	0.94	0.00	0.00	0.00	0.00	0.00	0.00	0.00	1.38	0.00	1.17	0.00	0.00	0.00	0.51	0.30	0.25	>0.999999999999999	1.2
P20742	Pregnancy zone protein OS=Homo sapiens GN=PZP PE=1 SV=4	0.00	0.00	0.00	0.00	2.30	0.00	0.00	0.00	0.00	1.25	0.00	0.00	0.00	0.00	1.67	0.00	2.26	0.00	0.00	0.00	0.00	0.00	0.00	0.00	0.30	0.33	>0.999999999999999	0.9
Q9H4M9	EH domain-containing protein 1 OS=Homo sapiens GN=EHD1 PE=1 SV=2	0.00	0.00	0.00	0.76	0.00	0.00	0.84	0.79	0.00	1.15	0.00	0.00	0.00	0.00	0.00	0.00	0.00	0.00	0.00	0.00	0.00	2.29	0.77	0.00	0.30	0.25	>0.999999999999999	1.2
O15231	Zinc finger protein 185 OS=Homo sapiens GN=ZNF185 PE=1 SV=3	0.98	0.92	1.01	0.00	0.00	0.00	0.00	0.63	0.00	0.00	0.00	0.00	0.00	0.81	0.00	0.00	0.00	0.61	0.00	0.52	0.00	0.00	0.00	0.51	0.30	0.20	>0.999999999999999	1.4
Q5VYK3	Proteasome-associated protein ECM29 homolog OS=Homo sapiens GN=ECM29 PE=1 SV=	0.49	0.46	0.34	0.00	0.38	0.87	0.00	0.00	0.12	0.00	0.57	0.30	0.00	0.00	0.00	0.00	0.00	0.00	0.00	0.26	0.00	0.42	0.00	0.11	0.29	0.07	>0.999999999999999	4.5
Q9H0B6	Kinesin light chain 2 OS=Homo sapiens GN=KLC2 PE=1 SV=1	0.00	0.00	0.00	1.52	0.96	0.00	0.84	0.00	0.20	0.00	0.00	0.00	0.44	1.01	0.00	0.00	0.00	0.00	0.00	0.65	0.00	1.04	0.00	0.00	0.29	0.26	>0.999999999999999	1.1
Q13619	Cullin-4A OS=Homo sapiens GN=CUL4A PE=1 SV=3	0.98	0.00	0.00	0.00	0.00	0.58	0.00	0.00	0.00	0.00	0.76	1.20	0.00	0.00	0.00	0.00	0.00	0.00	0.00	0.52	0.00	0.00	0.00	0.00	0.29	0.04	>0.999999999999999	6.8
Q99816	Tumor susceptibility gene 101 protein OS=Homo sapiens GN=TSG101 PE=1 SV=2	0.00	0.00	0.00	0.00	0.00	2.33	1.18	0.00	0.00	0.00	0.00	0.00	0.00	1.41	0.00	0.00	0.00	0.00	0.00	0.91	0.22	0.00	0.00	0.00	0.29	0.21	>0.999999999999999	1.4
Q9H832	Ubiquitin-conjugating enzyme E2 Z OS=Homo sapiens GN=UBE2Z PE=1 SV=2	2.20	0.00	0.00	0.00	0.00	1.31	0.00	0.00	0.00	0.00	0.00	0.00	0.00	0.00	0.00	0.00	0.00	0.00	0.00	1.17	0.00	0.00	0.00	0.00	0.29	0.10	>0.999999999999999	3.0
Q9BV68	RING finger protein 126 OS=Homo sapiens GN=RNF126 PE=1 SV=1	2.20	0.00	0.00	0.00	0.00	1.31	0.00	0.00	0.00	0.00	0.00	0.00	0.00	0.00	0.00	0.00	0.00	0.00	0.00	0.00	0.00	0.00	0.00	0.00	0.29	0.00	>0.999999999999999	#DIV/0!
P35542	Serum amyloid A-4 protein OS=Homo sapiens GN=SAA4 PE=1 SV=2	0.00	0.00	0.00	3.49	0.00	0.00	0.00	0.00	0.00	0.00	0.00	0.00	0.00	0.00	0.00	0.00	0.00	0.00	0.00	0.00	0.00	0.00	0.00	0.00	0.29	0.00	>0.999999999999999	#DIV/0!
Q96A65	Exocyst complex component 4 OS=Homo sapiens GN=EXOC4 PE=1 SV=1	1.47	0.69	0.00	0.45	0.00	0.87	0.00	0.00	0.00	0.00	0.00	0.00	0.00	0.60	0.00	0.00	0.00	0.00	0.00	0.39	0.00	0.00	0.00	0.00	0.29	0.08	>0.999999999999999	3.5
Q9UI46	Dynein intermediate chain 1, axonemal OS=Homo sapiens GN=DNAI1 PE=1 SV=1	0.98	0.00	0.00	0.00	0.77	0.58	0.00	0.00	0.00	0.00	0.76	0.40	0.00	0.00	0.00	0.00	0.00	0.00	0.00	0.00	0.00	0.00	0.00	0.00	0.29	0.00	>0.999999999999999	#DIV/0!

Q96M32	Adenylate kinase 7 OS=Homo sapiens GN=AK7 PE=1 SV=3	1.95	0.00	0.00	0.00	0.77	0.00	0.00	0.00	0.00	0.00	0.76	0.00	0.00	0.00	0.00	0.00	0.00	0.00	0.00	0.00	0.00	0.00	0.00	0.00	0.29	0.00	>0.999999999999999	#DIV/0!
P48556	26S proteasome non-ATPase regulatory subunit 8 OS=Homo sapiens GN=PSMD8 PE=1 S\	0.00	0.00	0.00	0.00	0.00	1.16	0.00	0.00	0.00	0.00	1.51	0.80	0.00	0.00	0.00	0.00	0.00	0.00	0.00	0.00	0.52	1.66	1.23	0.00	0.29	0.28	>0.999999999999999	1.0
P59780	AP-3 complex subunit sigma-2 OS=Homo sapiens GN=AP3S2 PE=2 SV=1	0.00	3.46	0.00	0.00	0.00	0.00	0.00	0.00	0.00	0.00	0.00	0.00	0.00	0.00	0.00	0.00	0.00	0.00	0.00	0.00	0.00	0.00	0.00	0.00	0.29	0.00	>0.999999999999999	#DIV/0!
P84095	Rho-related GTP-binding protein RhoG OS=Homo sapiens GN=RHOG PE=1 SV=1	0.00	0.00	0.00	0.00	0.00	0.00	0.00	0.00	0.00	3.44	0.00	0.00	0.00	0.00	0.00	0.00	0.00	0.00	0.00	0.00	0.00	6.86	0.00	0.00	0.29	0.57	>0.999999999999999	0.5
P49915	GMP synthase [glutamine-hydrolyzing] OS=Homo sapiens GN=GMPS PE=1 SV=1	0.98	0.00	0.00	0.00	0.00	1.31	0.00	0.00	0.00	0.00	0.76	0.40	0.00	0.81	0.00	0.28	0.00	0.00	0.00	0.00	0.12	0.00	0.00	0.23	0.29	0.12	>0.999999999999999	2.4
P19878	Neutrophil cytosol factor 2 OS=Homo sapiens GN=NCF2 PE=1 SV=2	0.00	0.00	0.00	1.67	0.00	0.00	0.00	0.00	0.00	1.77	0.00	0.00	0.00	0.00	0.00	0.00	0.00	0.00	0.00	0.00	0.15	2.29	0.77	0.00	0.29	0.27	>0.999999999999999	1.1
P07954	Fumarate hydratase, mitochondrial OS=Homo sapiens GN=FH PE=1 SV=3	0.00	0.00	0.67	0.00	0.00	2.77	0.00	0.00	0.00	0.00	0.00	0.00	0.53	0.00	1.33	0.41	0.00	1.84	0.00	1.55	0.00	1.25	0.00	0.34	0.29	0.60	>0.999999999999999	0.5
P20930	Filaggrin OS=Homo sapiens GN=FLG PE=1 SV=3	0.00	0.92	0.34	0.00	0.00	0.15	0.34	1.27	0.12	0.21	0.00	0.10	0.00	0.81	0.22	0.07	0.38	0.15	0.32	0.26	0.03	0.42	0.15	0.23	0.29	0.25	>0.999999999999999	1.1
Q5T764	Interferon-induced protein with tetratricopeptide repeats 1B OS=Homo sapiens GN=IFIT1B F	0.00	1.38	0.00	0.91	0.00	0.00	0.00	0.00	0.00	0.00	1.14	0.00	0.00	0.00	0.00	0.00	0.00	0.00	0.00	0.00	0.00	0.00	0.00	0.00	0.29	0.00	>0.999999999999999	#DIV/0!
Q96A05	V-type proton ATPase subunit E 2 OS=Homo sapiens GN=ATP6V1E2 PE=1 SV=1	0.00	0.00	1.46	1.97	0.00	0.00	0.00	0.00	0.00	0.00	0.00	0.00	0.00	0.00	1.44	0.00	0.00	0.00	0.00	0.00	0.00	0.00	0.00	0.00	0.29	0.12	>0.999999999999999	2.4
Q5JR98	Tctex1 domain-containing protein 4 OS=Homo sapiens GN=TCTEX1D4 PE=1 SV=1	3.42	0.00	0.00	0.00	0.00	0.00	0.00	0.00	0.00	0.00	0.00	0.00	0.00	0.00	0.00	0.00	0.00	0.00	0.00	0.00	0.00	0.00	0.00	0.00	0.28	0.00	>0.999999999999999	#DIV/0!
P56597	Nucleoside diphosphate kinase homolog 5 OS=Homo sapiens GN=NME5 PE=1 SV=1	3.42	0.00	0.00	0.00	0.00	0.00	0.00	0.00	0.00	0.00	0.00	0.00	0.00	0.00	0.00	0.00	0.00	0.00	0.00	0.00	0.00	0.00	0.00	0.00	0.28	0.00	>0.999999999999999	#DIV/0!
O76071	Probable cytosolic iron-sulfur protein assembly protein CIAO1 OS=Homo sapiens GN=CIAO	0.00	2.07	0.00	0.00	0.00	1.31	0.00	0.00	0.00	0.00	0.00	0.00	0.00	1.81	0.00	0.00	0.00	0.00	0.00	0.00	0.00	0.00	0.00	0.00	0.28	0.15	>0.999999999999999	1.9
Q9BQE5	Apolipoprotein L2 OS=Homo sapiens GN=APOL2 PE=1 SV=1	0.00	0.00	0.00	0.00	0.00	1.31	0.00	0.00	0.36	0.00	1.70	0.00	0.00	1.81	0.00	0.00	0.00	2.91	0.00	1.17	0.00	0.00	0.00	0.51	0.28	0.53	>0.999999999999999	0.5
Q86Y56	HEAT repeat-containing protein 2 OS=Homo sapiens GN=HEATR2 PE=1 SV=4	0.73	0.69	0.00	0.00	0.00	0.44	0.00	0.00	0.00	0.00	0.00	1.49	0.00	0.00	0.00	0.00	0.00	0.00	0.00	0.00	0.00	0.00	0.00	0.00	0.28	0.00	>0.999999999999999	#DIV/0!
Q92890	Ubiquitin fusion degradation protein 1 homolog OS=Homo sapiens GN=UFD1L PE=1 SV=3	0.00	0.00	0.00	0.00	0.00	1.46	0.00	0.00	0.00	0.00	1.89	0.00	0.00	0.00	0.00	0.69	0.00	0.00	0.00	0.00	0.00	0.00	0.00	0.00	0.28	0.06	>0.999999999999999	4.9
O75382	Tripartite motif-containing protein 3 OS=Homo sapiens GN=TRIM3 PE=1 SV=2	0.98	0.00	0.45	0.00	0.77	0.00	0.00	0.00	0.00	0.00	0.76	0.40	0.00	0.00	0.00	0.00	0.00	0.00	0.00	0.00	0.00	0.00	0.00	0.00	0.28	0.00	>0.999999999999999	#DIV/0!
P52758	Ribonuclease UK114 OS=Homo sapiens GN=HRSP12 PE=1 SV=1	0.00	0.00	0.00	0.00	0.00	3.35	0.00	0.00	0.00	0.00	0.00	0.00	0.00	0.00	0.00	0.00	0.00	0.00	0.00	0.00	0.00	0.00	0.00	0.00	0.28	0.00	>0.999999999999999	#DIV/0!
P60673	Profilin-3 OS=Homo sapiens GN=PFN3 PE=2 SV=1	0.00	0.00	0.00	0.00	0.00	3.35	0.00	0.00	0.00	0.00	0.00	0.00	0.00	0.00	0.00	0.00	0.00	3.53	0.00	0.00	0.00	0.00	0.00	0.00	0.28	0.29	>0.999999999999999	0.9
Q8N163	Protein KIAA1967 OS=Homo sapiens GN=KIAA1967 PE=1 SV=2	0.00	0.69	0.34	0.00	0.00	0.87	0.00	0.00	0.00	0.00	1.14	0.30	0.00	0.00	0.00	0.00	0.00	0.00	0.00	0.39	0.00	0.00	0.00	0.00	0.28	0.03	>0.999999999999999	8.6
Q08209	Serine/threonine-protein phosphatase 2B catalytic subunit alpha isoform OS=Homo sapiens	0.00	0.00	0.00	0.00	0.00	1.60	0.00	0.00	0.00	0.00	1.14	0.60	0.00	0.00	0.00	0.41	0.00	0.00	0.00	0.78	0.00	2.29	0.92	0.34	0.28	0.39	>0.999999999999999	0.7
Q96CW1	AP-2 complex subunit mu OS=Homo sapiens GN=AP2M1 PE=1 SV=2	0.00	1.61	0.00	0.00	0.00	1.02	0.00	0.00	0.00	0.00	0.00	0.70	0.00	0.00	0.78	0.00	0.00	0.00	0.00	0.00	0.22	0.00	0.00	0.00	0.28	0.08	>0.999999999999999	3.4
P51159	Ras-related protein Rab-27A OS=Homo sapiens GN=RAB27A PE=1 SV=3	0.00	0.00	0.00	1.97	0.00	0.00	0.00	0.00	0.00	1.36	0.00	0.00	0.00	0.00	0.00	0.00	0.00	0.00	0.00	0.00	0.00	2.70	0.00	0.00	0.28	0.23	>0.999999999999999	1.2
Q9Y4P1	Cysteine protease ATG4B OS=Homo sapiens GN=ATG4B PE=1 SV=2	1.71	1.61	0.00	0.00	0.00	0.00	0.00	0.00	0.00	0.00	0.00	0.00	0.00	0.00	0.00	0.00	0.00	0.00	0.00	0.91	0.00	0.00	0.00	0.40	0.28	0.11	>0.999999999999999	2.5
P56945	Breast cancer anti-estrogen resistance protein 1 OS=Homo sapiens GN=BCAR1 PE=1 SV=:	0.98	0.00	0.45	0.00	0.00	0.58	0.00	0.00	0.16	0.00	0.76	0.40	0.00	0.00	0.44	0.28	0.00	0.00	0.00	0.52	0.00	0.00	0.00	0.00	0.28	0.10	>0.999999999999999	2.7
P22792	Carboxypeptidase N subunit 2 OS=Homo sapiens GN=CPN2 PE=1 SV=3	0.00	0.00	0.56	0.76	0.96	0.00	0.84	0.00	0.20	0.00	0.00	0.00	0.44	0.00	0.56	0.00	0.94	0.00	0.79	0.00	0.15	0.00	0.77	0.00	0.28	0.30	>0.999999999999999	0.9
Q9Y6I9	Testis-expressed sequence 264 protein OS=Homo sapiens GN=TEX264 PE=1 SV=1	0.00	2.30	0.00	0.00	0.00	0.00	0.00	0.00	0.00	0.00	0.00	1.00	0.00	0.00	0.00	0.00	0.00	0.00	0.00	0.00	0.00	0.00	0.00	0.00	0.28	0.00	>0.999999999999999	#DIV/0!
Q9Y2X3	Nucleolar protein 58 OS=Homo sapiens GN=NOP58 PE=1 SV=1	0.00	0.00	0.00	0.76	0.00	0.73	0.84	0.00	0.00	0.00	0.95	0.00	0.00	0.00	0.00	0.00	0.00	0.00	0.00	0.00	0.00	0.00	0.00	0.00	0.27	0.00	>0.999999999999999	#DIV/0!
P01889	HLA class I histocompatibility antigen, B-7 alpha chain OS=Homo sapiens GN=HLA-B PE=1	0.00	0.00	0.00	0.00	0.00	2.47	0.00	0.00	0.00	0.00	0.00	0.80	0.00	0.00	0.00	1.17	0.00	0.00	0.00	0.00	0.25	0.00	0.00	0.45	0.27	0.16	>0.999999999999999	1.7
Q92530	Proteasome inhibitor PI31 subunit OS=Homo sapiens GN=PSMF1 PE=1 SV=2	0.00	0.00	1.23	0.00	0.00	1.60	0.00	0.00	0.44	0.00	0.00	0.00	0.00	0.00	0.00	0.00	0.00	0.00	0.00	0.00	0.00	0.00	0.00	0.00	0.27	0.00	>0.999999999999999	#DIV/0!
Q5T1M5	FK506-binding protein 15 OS=Homo sapiens GN=FKBP15 PE=1 SV=2	1.22	0.00	0.22	0.00	0.00	0.29	0.00	0.00	0.08	0.00	0.95	0.50	0.00	0.40	0.00	0.00	0.00	0.00	0.00	0.00	0.00	0.00	0.00	0.00	0.27	0.03	>0.999999999999999	8.1
Q9P258	Protein RCC2 OS=Homo sapiens GN=RCC2 PE=1 SV=2	1.47	0.00	0.67	0.00	0.00	0.87	0.00	0.00	0.24	0.00	0.00	0.00	0.00	0.00	0.00	0.41	0.00	0.00	0.00	0.78	0.00	0.00	0.00	0.00	0.27	0.10	>0.999999999999999	2.7
Q9Y6N5	Sulfide:quinone oxidoreductase, mitochondrial OS=Homo sapiens GN=SQRDL PE=1 SV=1	0.00	0.00	0.00	0.00	0.00	0.00	0.00	2.22	0.28	0.73	0.00	0.00	0.00	0.00	0.78	0.00	0.00	0.00	0.00	0.00	0.00	0.00	0.00	0.00	0.27	0.06	>0.999999999999999	4.2
P09914	Interferon-induced protein with tetratricopeptide repeats 1 OS=Homo sapiens GN=IFIT1 PE=	1.47	0.00	0.00	0.00	0.00	1.75	0.00	0.00	0.00	0.00	0.00	0.00	0.00	0.00	0.00	0.00	0.00	0.00	0.00	0.00	0.00	0.00	0.00	0.00	0.27	0.00	>0.999999999999999	#DIV/0!
Q5T1J5	Putative coiled-coil-helix-coiled-coil-helix domain-containing protein CHCHD2P9, mitochondri	0.00	0.00	0.00	0.00	0.00	3.20	0.00	0.00	0.00	0.00	0.00	0.00	0.00	0.00	0.00	0.00	0.00	0.00	0.00	0.00	0.00	0.00	0.00	0.00	0.27	0.00	>0.999999999999999	#DIV/0!
O94817	Ubiquitin-like protein ATG12 OS=Homo sapiens GN=ATG12 PE=1 SV=1	0.00	0.00	0.00	0.00	0.00	3.20	0.00	0.00	0.00	0.00	0.00	0.00	0.00	0.00	0.00	0.00	0.00	0.00	0.00	0.00	0.00	0.00	0.00	0.00	0.27	0.00	>0.999999999999999	#DIV/0!
O75688	Protein phosphatase 1B OS=Homo sapiens GN=PPM1B PE=1 SV=1	1.47	0.00	0.00	0.00	0.00	0.00	0.00	0.00	0.00	0.00	1.14	0.60	0.00	0.00	0.00	0.00	0.00	0.00	0.00	0.00	0.00	1.25	0.00	0.00	0.27	0.10	>0.999999999999999	2.6
P09417	Dihydropteridine reductase OS=Homo sapiens GN=QDPR PE=1 SV=2	0.00	0.00	0.00	0.00	0.00	1.89	0.00	0.00	0.00	0.00	0.00	1.29	0.00	2.62	0.00	0.00	0.00	0.00	0.00	1.68	0.00	2.70	0.00	0.00	0.27	0.58	>0.999999999999999	0.5
Q9NVS9	Pyridoxine-5'-phosphate oxidase OS=Homo sapiens GN=PNPO PE=1 SV=1	0.00	0.00	0.00	0.00	0.00	0.00	0.00	0.00	0.00	0.00	2.08	1.10	0.00	0.00	0.00	0.00	0.00	0.00	0.00	1.43	0.00	0.00	0.00	0.62	0.26	0.17	>0.999999999999999	1.6
Q9NXG2	THUMP domain-containing protein 1 OS=Homo sapiens GN=THUMPD1 PE=1 SV=2	0.00	0.00	0.90	0.00	0.00	1.16	0.00	0.00	0.32	0.00	0.00	0.80	0.00	0.00	0.00	0.00	0.00	1.23	0.00	0.00	0.00	0.00	0.00	0.45	0.26	0.14	>0.999999999999999	1.9
Q6PGP7	Tetratricopeptide repeat protein 37 OS=Homo sapiens GN=TTC37 PE=1 SV=1	0.49	0.92	0.22	0.00	0.00	0.58	0.00	0.00	0.00	0.00	0.76	0.20	0.00	0.00	0.00	0.00	0.00	0.00	0.00	0.26	0.00	0.00	0.00	0.00	0.26	0.02	>0.999999999999999	12.2
Q8NI22	Multiple coagulation factor deficiency protein 2 OS=Homo sapiens GN=MCFD2 PE=1 SV=1	0.00	0.00	0.00	0.00	0.00	0.00	0.00	3.17	0.00	0.00	0.00	0.00	1.75	0.00	0.00	0.00	0.00	0.00	0.00	2.59	0.00	0.00	0.00	0.00	0.26	0.36	>0.999999999999999	0.7
Q04837	Single-stranded DNA-binding protein, mitochondrial OS=Homo sapiens GN=SSBP1 PE=1 S\	0.00	0.00	0.00	0.00	0.00	0.00	0.00	3.17	0.00	0.00	0.00	0.00	1.75	0.00	0.00	0.00	0.00	0.00	0.00	0.00	0.00	0.00	0.00	0.00	0.26	0.15	>0.999999999999999	1.8
Q676U5	Autophagy-related protein 16-1 OS=Homo sapiens GN=ATG16L1 PE=1 SV=2	1.22	1.15	0.56	0.00	0.00	0.00	0.00	0.00	0.20	0.00	0.00	0.00	0.00	1.01	0.00	0.00	0.00	0.00	0.00	0.65	0.00	0.00	0.00	0.00	0.26	0.14	>0.999999999999999	1.9
Q9NRF8	CTP synthase 2 OS=Homo sapiens GN=CTPS2 PE=1 SV=1	0.00	0.00	0.00	0.00	0.96	0.73	0.00	0.00	0.00	0.00	0.95	0.50	0.00	0.00	0.00	0.00	0.00	0.00	0.00	0.00	0.00	0.00	0.00	0.00	0.26	0.00	>0.999999999999999	#DIV/0!
P28676	Grancalcin OS=Homo sapiens GN=GCA PE=1 SV=2	0.00	0.00	0.00	0.00	0.00	0.00	0.00	0.00	0.00	3.13	0.00	0.00	0.00	0.00	0.00	0.00	0.00	0.00	0.00	0.00	0.00	0.00	0.00	0.00	0.26	0.00	>0.999999999999999	#DIV/0!
P06888	Ig lambda chain V-I region EPS OS=Homo sapiens PE=1 SV=1	0.00	0.00	0.00	0.00	0.00	0.00	0.00	0.00	0.00	3.13	0.00	0.00	0.00	0.00	0.00	0.00	5.66	0.00	0.00	0.00	0.00	6.23	0.00	0.00	0.26	0.99	>0.999999999999999	0.3
Q92743	Serine protease HTRA1 OS=Homo sapiens GN=HTRA1 PE=1 SV=1	0.00	0.00	0.67	0.91	0.00	0.00	1.01	0.00	0.52	0.00	0.00	0.00	1.14	0.00	0.67	0.00	0.00	0.92	0.00	0.78	0.18	0.00	0.00	0.74	0.26	0.37	>0.999999999999999	0.7
Q96EP5	DAZ-associated protein 1 OS=Homo sapiens GN=DAZAP1 PE=1 SV=1	0.00	0.00	0.00	0.00	0.00	0.00	0.00	0.00	0.00	0.00	1.51	1.59	0.00	0.00	0.89	0.00	0.00	0.00	0.00	0.00	0.00	0.00	0.00	0.45	0.26	0.11	>0.999999999999999	2.3
Q9Y2L1	Exosome complex exonuclease RRP44 OS=Homo sapiens GN=DIS3 PE=1 SV=2	0.73	0.00	0.34	0.00	0.00	0.87	0.00	0.00	0.00	0.00	0.57	0.60	0.00	0.60	0.00	0.21	0.00	0.46	0.00	0.78	0.00	0.00	0.00	0.17	0.26	0.18	>0.999999999999999	1.4
Q93009	Ubiquitin carboxyl-terminal hydrolase 7 OS=Homo sapiens GN=USP7 PE=1 SV=2	1.22	0.00	0.00	0.00	0.00	0.44	0.00	0.00	0.00	0.00	0.95	0.50	0.00	0.00	0.00	0.00	0.00	0.00	0.00	0.39	0.00	0.00	0.00	0.00	0.26	0.03	>0.999999999999999	8.0
O95400	CD2 antigen cytoplasmic tail-binding protein 2 OS=Homo sapiens GN=CD2BP2 PE=1 SV=1	2.20	0.00	0.00	0.00	0.00	0.00	0.00	0.00	0.00	0.00	0.00	0.90	0.00	0.00	0.00	0.00	0.00	0.00	0.00	0.00	0.00	0.00	0.00	0.00	0.26	0.00	>0.999999999999999	#DIV/0!
P35222	Catenin beta-1 OS=Homo sapiens GN=CTNNB1 PE=1 SV=1	0.98	0.00	0.00	0.00	0.00	1.16	0.00	0.63	0.32	0.00	0.00	0.00	0.00	0.00	0.00	0.00	0.00	1.23	0.00	1.04	0.00	0.00	0.00	0.00	0.26	0.19	>0.999999999999999	1.4
Q9H0R4	Haloacid dehalogenase-like hydrolase domain-containing protein 2 OS=Homo sapiens GN=I	0.00	0.00	1.34	0.00	0.00	1.75	0.00	0.00	0.00	0.00	0.00	0.00	0.00	0.00	0.00	0.00	0.00	0.00	0.00	0.00	0.00	0.00	0.00	0.00	0.26	0.00	>0.999999999999999	#DIV/0!
Q9ULA0	Aspartyl aminopeptidase OS=Homo sapiens GN=DNPEP PE=1 SV=1	0.00	0.00	0.00	0.00	0.00	1.89	0.00	0.95	0.24	0.00	0.00	0.00	0.53	0.00	0.00	0.00	0.00	0.92	0.00	1.68	0.00	0.00	0.00	0.00	0.26	0.26	>0.999999999999999	1.0
O94855	Protein transport protein Sec24D OS=Homo sapiens GN=SEC24D PE=1 SV=2	0.00	0.69	0.34	0.45	0.00	0.44	0.51	0.00	0.36	0.00	0.00	0.30	0.26	0.60	0.00	0.21	0.00	0.46	0.00	0.78	0.18	0.00	0.00	0.34	0.26	0.24	>0.999999999999999	1.1
P05186	Alkaline phosphatase, tissue-nonspecific isozyme OS=Homo sapiens GN=ALPL PE=1 SV=4	0.00	0.00	0.00	0.00	0.00	0.87	0.00	0.95	0.00	1.25	0.00	0.00	0.00	2.42	0.00	0.00	0.00	0.00	0.00	0.78	0.00	0.00	0.00	0.00	0.26	0.27	>0.999999999999999	1.0
P55795	Heterogeneous nuclear ribonucleoprotein H2 OS=Homo sapiens GN=HNRNPH2 PE=1 SV='	0.00	0.00	0.00	0.00	0.00	3.06	0.00	0.00	0.00	0.00	0.00	0.00	0.00	0.00	0.00	0.00	0.00	0.00	0.00	0.00	0.00	0.00	0.00	0.00	0.25	0.00	>0.999999999999999	#DIV/0!
P31323	cAMP-dependent protein kinase type II-beta regulatory subunit OS=Homo sapiens GN=PRK	0.00	0.00	0.00	1.06	0.00	1.02	0.00	0.00	0.28	0.00	0.00	0.70	0.61	0.00	0.78	0.48	0.00	0.00	0.00	0.91	0.00	1.45	0.00	0.40	0.25	0.39	>0.999999999999999	0.7
Q13057	Bifunctional coenzyme A synthase OS=Homo sapiens GN=COASY PE=1 SV=4	0.00	0.00	0.00	0.00	0.00	1.60	0.00	0.00	0.00	0.00	0.95	0.50	0.00	1.01	0.00	0.00	0.00	0.00	0.00	0.65	0.00	0.00	0.00	0.28	0.25	0.16	>0.999999999999999	1.6
Q9Y2A7	Nck-associated protein 1 OS=Homo sapiens GN=NCKAP1 PE=1 SV=1	0.73	0.69	0.56	0.00	0.00	0.44	0.00	0.00	0.32	0.00	0.00	0.30	0.00	0.60	0.89	0.21	0.00	0.00	0.00	0.00	0.09	0.00	0.46	0.00	0.25	0.19	>0.999999999999999	1.3
P10153	Non-secretory ribonuclease OS=Homo sapiens GN=RNASE2 PE=1 SV=2	0.00	0.00	0.00	0.00	0.00	0.00	3.04	0.00	0.00	0.00	0.00	0.00	0.00	0.00	0.00	0.00	0.00	0.00	2.85	0.00	0.00	0.00	2.76	0.00	0.25	0.47	>0.999999999999999	0.5
P09543	2',3'-cyclic-nucleotide 3'-phosphodiesterase OS=Homo sapiens GN=CNP PE=1 SV=2	1.71	0.00	0.00	0.00	0.00	0.00	0.00	0.00	0.00	0.00	1.33	0.00	0.00	1.41	0.00	0.00	0.00	0.00	0.00	0.91	0.00	1.45	0.00	0.00	0.25	0.31	>0.999999999999999	0.8
Q15382	GTP-binding protein Rheb OS=Homo sapiens GN=RHEB PE=1 SV=1	0.00	0.00	0.00	0.00	0.00	0.00	0.00	0.00	0.00	0.00	3.03	0.00	0.00	0.00	0.00	0.00	0.00	0.00	0.00	0.00	0.00	0.00	0.00	0.00	0.25	0.00	>0.999999999999999	#DIV/0!
P01596	Ig kappa chain V-I region CAR OS=Homo sapiens PE=1 SV=1	0.00	0.00	0.00	0.00	0.00	0.00	0.00	0.00	0.00	3.03	0.00	0.00	0.00	0.00	0.00	0.00	0.00	0.00	0.00	0.00	0.00	0.00	0.00	0.00	0.25	0.00	>0.999999999999999	#DIV/0!
P10620	Microsomal glutathione S-transferase 1 OS=Homo sapiens GN=MGST1 PE=1 SV=1	0.00	0.00	0.00	0.00	0.00	0.00	0.00	3.01	0.00	0.00	0.00	0.00	0.00	0.00	0.00	0.00	0.00	0.00	0.00	0.00	0.00	0.00	0.00	0.00	0.25	0.00	>0.999999999999999	#DIV/0!
Q8NEZ5	F-box only protein 22 OS=Homo sapiens GN=FBXO22 PE=1 SV=1	1.71	0.00	0.00	0.00	0.00	1.02	0.00	0.00	0.28	0.00	0.00	0.00	0.00	0.00	0.00	0.00	0.00	1.07	0.00	0.00	0.00	0.00	0.00	0.00	0.25	0.09	>0.999999999999999	2.8
P32456	Interferon-induced guanylate-binding protein 2 OS=Homo sapiens GN=GBP2 PE=2 SV=3	0.00	1.15	0.00	0.00	0.00	1.46	0.00	0.00	0.40	0.00	0.00	0.00	0.00	0.00	0.00	0.00	0.00	0.00	0.00	0.00	0.00	0.00	0.00	0.28	0.25	0.02	>0.999999999999999	10.6
P32926	Desmoglein-3 OS=Homo sapiens GN=DSG3 PE=1 SV=2	0.00	0.00	0.00	0.00	0.00	0.00	0.51	0.48	0.00	0.31	1.70	0.00	0.00	0.00	0.00	0.00	0.57	0.00	0.00	0.00	0.09	0.62	1.38	0.00	0.25	0.22	>0.999999999999999	1.1
O75015	Low affinity immunoglobulin gamma Fc region receptor III-B OS=Homo sapiens GN=FCGR3	0.00	3.00	0.00	0.00	0.00	0.00	0.00	0.00	0.00	0.00	0.00	0.00	0.00	0.00	0.00	0.00	0.00	0.00	0.00	0.00	0.00	0.00	0.00	0.00	0.25	0.00	>0.999999999999999	#DIV/0!
P00736	Complement C1r subcomponent OS=Homo sapiens GN=C1R PE=1 SV=2	0.00	0.00	0.00	2.58	0.00	0.00	0.00	0.00	0.00	0.42	0.00	0.00	0.00	0.81	0.89	0.28	1.51	0.00	2.06	0.52	0.00	1.66	0.00	0.23	0.25	0.66	>0.999999999999999	0.4
Q9BS26	Endoplasmic reticulum resident protein 44 OS=Homo sapiens GN=ERP44 PE=1 SV=1	0.00	0.00	0.00	1.06	0.00	0.00	1.18	0.00	0.00	0.73	0.00	0.00	0.61	0.00	0.00	0.00	0.00	0.00	0.00	0.00	0.00	1.45	0.00	0.00	0.25	0.17	>0.999999999999999	1.4
Q15714	TSC22 domain family protein 1 OS=Homo sapiens GN=TSC22D1 PE=1 SV=3	0.73	0.69	0.34	0.00	0.00	0.44	0.00	0.48	0.00	0.00	0.00	0.30	0.00	0.00	0.00	0.00	0.00	0.00	0.00	0.39	0.00	0.00	0.00	0.00	0.25	0.03	>0.999999999999999	7.6
Q9NXJ5	Pyroglutamyl-peptidase 1 OS=Homo sapiens GN=PGPEP1 PE=1 SV=1	0.00	0.00	1.57	0.00	0.00	0.00	0.00	0.00	0.00	0.00	0.00	1.39	0.00	0.00	0.00	0.00	0.00	0.00	0.00	0.00	0.00	0.00	0.00	0.00	0.25	0.00	>0.999999999999999	#DIV/0!
P16070	CD44 antigen OS=Homo sapiens GN=CD44 PE=1 SV=3	0.00	0.92	0.45	0.61	0.00	0.58	0.00	0.00	0.00	0.00	0.00	0.40	0.00	0.81	0.00	0.28	0.00	0.61	0.63	0.52	0.12	0.00	0.61	0.00	0.25	0.30	>0.999999999999999	0.8
P06865	Beta-hexosaminidase subunit alpha OS=Homo sapiens GN=HEXA PE=1 SV=2	0.00	1.15	0.00	0.00	0.00	1.60	0.00	0.00	0.20	0.00	0.00	0.00	0.00	2.22	0.00	0.00	0.00	0.77	0.00	0.65	0.15	1.04	0.00	0.28	0.25	0.43	>0.999999999999999	0.6
P0CG29	Glutathione S-transferase theta-2 OS=Homo sapiens GN=GSTT2 PE=1 SV=1	0.00	0.00	0.00	0.00	0.00	1.75	0.00	0.00	0.00	0.00	0.00	1.20	0.00	0.00	0.00	0.00	0.00	0.00	0.00	0.00	0.37	0.00	0.00	0.00	0.25	0.03	>0.999999999999999	8.0
P52594	Arf-GAP domain and FG repeat-containing protein 1 OS=Homo sapiens GN=AGFG1 PE=1 S	1.47	0.00	0.00	0.00	0.00	0.87	0.00	0.00	0.00	0.00	0.00	0.60	0.00	0.00	0.00	0.00	0.00	0.00	0.00	0.00	0.00	0.00	0.00	0.00	0.24	0.00	>0.999999999999999	#DIV/0!
P31749	RAC-alpha serine/threonine-protein kinase OS=Homo sapiens GN=AKT1 PE=1 SV=2	1.47	0.00	0.00	0.00	0.00	0.87	0.00	0.00	0.00	0.00	0.00	0.60	0.00	0.00	0.00	0.00	0.00	0.00	0.00	0.00	0.00	0.00	0.00	0.00	0.24	0.00	>0.999999999999999	#DIV/0!
Q05655	Protein kinase C delta type OS=Homo sapiens GN=PRKCD PE=1 SV=2	0.98	0.92	0.00	0.00	0.00	0.00	0.00	0.63	0.00	0.00	0.00	0.40	0.00	0.00	0.00	0.00	0.00	0.00	0.00	0.52	0.00	0.83	0.61	0.23	0.24	0.18	>0.999999999999999	1.3
Q15080	Neutrophil cytosol factor 4 OS=Homo sapiens GN=NCF4 PE=1 SV=2	0.00	0.00	0.00	0.00	0.00	0.00	0.00	0.00	0.00	2.92	0.00	0.00	0.00	0.00	0.00	0.00	0.00	0.00	0.00	0.00	0.00	3.74	0.00	0.00	0.24	0.31	>0.999999999999999	0.8
P42166	Lamina-associated polypeptide 2, isoform alpha OS=Homo sapiens GN=TMPO PE=1 SV=2	0.00	0.00	0.00	0.00	0.00	0.58	0.00	0.63	0.00	0.00	1.70	0.00	0.00	0.00	0.00	0.00	0.00	1.38	0.00	0.00	0.00	0.83	0.00	0.00	0.24	0.18	>0.999999999999999	1.3
O75390	Citrate synthase, mitochondrial OS=Homo sapiens GN=CS PE=1 SV=2	0.00	0.00	0.00	0.91	0.00	0.87	0.00	0.00	0.00	0.00	1.14	0.00	0.00	0.00	0.67	0.00	0.00	0.92	0.95	0.78	0.00	0.00	0.92	0.00	0.24	0.35	>0.999999999999999	0.7
P35749	Myosin-11 OS=Homo sapiens GN=MYH11 PE=1 SV=3	0.00	0.00	0.00	0.00	0.00	2.91	0.00	0.00	0.00	0.00	0.00	0.00	0.00	0.00	0.00	0.00	0.00	0.00	0.00	0.00	0.00	0.00	0.00	0.00	0.24	0.00	>0.999999999999999	#DIV/0!
Q99614	Tetratricopeptide repeat protein 1 OS=Homo sapiens GN=TTC1 PE=1 SV=1	0.00	0.00	0.00	0.00	0.00	2.91	0.00	0.00	0.00	0.00	0.00	0.00	0.00	0.00	0.00	0.00	0.00	0.00	0.00	0.00	0.00	0.00	0.00	0.00	0.24	0.00	>0.999999999999999	#DIV/0!
Q14232	Translation initiation factor eIF-2B subunit alpha OS=Homo sapiens GN=EIF2B1 PE=1 SV=1	0.00	0.00	0.00	0.00	0.00	0.00	0.00	0.00	0.00	0.00	1.89	1.00	0.00	0.00	0.00	0.00	0.00	0.00	0.00	0.00	0.00	2.08	0.00	0.00	0.24	0.17	>0.999999999999999	1.4
Q7L5Y1	Mitochondrial enolase superfamily member 1 OS=Homo sapiens GN=ENOSF1 PE=1 SV=1	0.00	0.00	0.00	0.00	0.00	0.00	0.00	0.00	0.00	0.00	0.00	2.89	0.00	0.00	0.00	0.00	0.00	0.00	0.00	0.00	0.00	0.00	0.00	0.00	0.24	0.00	>0.999999999999999	#DIV/0!
Q9H0U4	Ras-related protein Rab-1B OS=Homo sapiens GN=RAB1B PE=1 SV=1	0.00	0.00	0.00	0.00	2.88	0.00	0.00	0.00	0.00	0.00	0.00	0.00	0.00	0.00	0.00	0.00	0.00	7.98	2.38	0.00	0.00	0.00	0.00	0.00	0.24	0.86	>0.999999999999999	0.3
Q7Z6Z7	E3 ubiquitin-protein ligase HUWE1 OS=Homo sapiens GN=HUWE1 PE=1 SV=3	1.47	0.23	0.11	0.00	0.00	0.44	0.00	0.00	0.12	0.00	0.19	0.30	0.00	0.20	0.00	0.07	0.00	0.00	0.00	0.39	0.06	0.00	0.00	0.06	0.24	0.06	>0.999999999999999	3.7
Q04826	HLA class I histocompatibility antigen, B-40 alpha chain OS=Homo sapiens GN=HLA-B PE='	1.95	0.00	0.90	0.00	0.00	0.00	0.00	0.00	0.00	0.00	0.00	0.00	0.00	0.00	0.00	0.00	0.00	0.00	0.00	4.79	0.00	0.00	0.00	0.00	0.24	0.40	>0.999999999999999	0.6
O00487	26S proteasome non-ATPase regulatory subunit 14 OS=Homo sapiens GN=PSMD14 PE=1	0.00	0.00	0.00	0.00	0.00	1.46	0.00	0.00	0.40	0.00	0.00	1.00	0.00	2.01	0.00	0.00	0.00	0.00	0.00	1.30	0.00	0.00	0.00	0.00	0.24	0.28	>0.999999999999999	0.9
O14896	Interferon regulatory factor 6 OS=Homo sapiens GN=IRF6 PE=1 SV=1	0.00	0.00	0.00	0.00	0.00	0.87	0.00	0.00	0.24	0.00	1.14	0.60	0.00	0.00	0.00	0.00	0.00	0.00	0.00	0.00	0.00	0.00	0.00	0.00	0.24	0.00	>0.999999999999999	#DIV/0!
O43143	Putative pre-mRNA-splicing factor ATP-dependent RNA helicase DHX15 OS=Homo sapiens	0.98	0.00	0.45	0.00	0.00	1.02	0.00	0.00	0.00	0.00	0.00	0.40	0.00	0.00	0.00	0.00	0.00	0.00	0.00	0.00	0.00	0.00	0.00	0.00	0.24	0.00	>0.999999999999999	#DIV/0!
Q9Y6B6	GTP-binding protein SAR1b OS=Homo sapiens GN=SAR1B PE=1 SV=1	0.00	0.00	0.00	0.00	0.00	0.00	0.00	0.00	0.00	0.00	2.84	0.00	0.00	0.00	0.00	0.00	0.00	0.00	0.00	0.00	0.00	0.00	0.00	0.00	0.24	0.00	>0.999999999999999	#DIV/0!
Q99909	Protein SSX3 OS=Homo sapiens GN=SSX3 PE=1 SV=2	0.00	0.00	0.00	0.00	0.00	0.00	0.00	0.00	0.00	0.00	2.84	0.00	0.00	0.00	0.00	0.00	0.00	0.00	0.00	0.00	0.00	0.00	0.00	0.00	0.24	0.00	>0.999999999999999	#DIV/0!
Q13670	Putative postmeiotic segregation increased 2-like protein 11 OS=Homo sapiens GN=PMS2P	0.00	0.00	1.23	0.00	0.00	1.60	0.00	0.00	0.00	0.00	0.00	0.00	0.00	0.00	0.00	0.00	0.00	0.00	0.00	0.00	0.00	0.00	0.00	0.00	0.24	0.00	>0.999999999999999	#DIV/0!
Q86TU7	Histone-lysine N-methyltransferase setd3 OS=Homo sapiens GN=SETD3 PE=1 SV=1	0.00	1.15	0.00	0.00	0.00	0.73	0.00	0.00	0.00	0.00	0.95	0.00	0.00	0.00	0.00	0.00	0.00	0.00	0.00	0.00	0.00	0.00	0.00	0.00	0.24	0.00	>0.999999999999999	#DIV/0!
Q9H069	Leucine-rich repeat-containing protein 48 OS=Homo sapiens GN=LRRC48 PE=2 SV=2	1.22	0.00	0.00	0.00	0.00	1.60	0.00	0.00	0.00	0.00	0.00	0.00	0.44	0.00	0.00	0.00	0.00	0.00	0.00	0.00	0.00	0.00	0.00	0.00	0.24	0.04	>0.999999999999999	6.4
P08493	Matrix Gla protein OS=Homo sapiens GN=MGP PE=1 SV=2	0.00	0.00	0.00	0.00	0.00	0.00	0.00	0.00	0.00	2.82	0.00	0.00	0.00	0.00	0.00	0.00	0.00	0.00	0.00	0.00	0.00	0.00	0.00	0.00	0.23	0.00	>0.999999999999999	#DIV/0!
P08637	Low affinity immunoglobulin gamma Fc region receptor III-A OS=Homo sapiens GN=FCGR3.	0.00	0.00	0.00	1.67	0.00	0.00	0.00	0.00	0.00	1.15	0.00	0.00	0.00	0.00	0.00	0.00	0.00	0.00	0.00	0.00	0.00	4.99	1.69	0.00	0.23	0.56	>0.999999999999999	0.4
O60232	Sjoegren syndrome/scleroderma autoantigen 1 OS=Homo sapiens GN=SSSCA1 PE=1 SV=	0.00	0.00	0.00	0.00	0.00	2.18	0.00	0.00	0.59	0.00	0.00	0.00	0.00	0.00	0.00	0.00	0.00	0.00	0.00	0.00	0.00	0.00	0.00	0.00	0.23	0.00	>0.999999999999999	#DIV/0!
Q8WYA0	Intraflagellar transport protein 81 homolog OS=Homo sapiens GN=IFT81 PE=1 SV=1	0.00	0.00	0.00	0.00	0.00	0.58	0.00	0.63	0.00	0.00	0.76	0.80	0.00	0.00	0.00	0.00	0.00	0.00	0.00	0.00	0.00	0.00	0.00	0.00	0.23	0.00	>0.999999999999999	#DIV/0!
P46937	Yorkie homolog OS=Homo sapiens GN=YAP1 PE=1 SV=2	0.00	2.77	0.00	0.00	0.00	0.00	0.00	0.00	0.00	0.00	0.00	0.00	0.00	0.00	0.00	0.00	0.00	0.00	0.00	0.00	0.00	0.00	0.00	0.00	0.23	0.00	>0.999999999999999	#DIV/0!
Q15417	Calponin-3 OS=Homo sapiens GN=CNN3 PE=1 SV=1	0.00	0.00	0.00	0.00	0.00	2.77	0.00	0.00	0.00	0.00	0.00	0.00	0.00	0.00	0.00	0.00	0.00	0.00	0.00	0.00	0.00	0.00	0.00	0.00	0.23	0.00	>0.999999999999999	#DIV/0!
Q5TBC7	Bcl-2-like protein 15 OS=Homo sapiens GN=BCL2L15 PE=2 SV=1	0.00	0.00	0.00	0.00	0.00	2.77	0.00	0.00	0.00	0.00	0.00	0.00	0.00	0.00	0.00	0.00	0.00	0.00	0.00	0.59	0.00	0.00	0.00	0.00	0.23	0.05	>0.999999999999999	4.7
Q9UIL1	Short coiled-coil protein OS=Homo sapiens GN=SCOC PE=1 SV=2	0.00	0.00	0.00	0.00	0.00	2.77	0.00	0.00	0.00	0.00	0.00	0.00	0.00	0.00	0.00	0.00	0.00	0.00	0.00	0.00	0.00	0.00	0.00	0.00	0.23	0.00	>0.999999999999999	#DIV/0!
O95273	Cyclin-D1-binding protein 1 OS=Homo sapiens GN=CCNDBP1 PE=1 SV=2	1.95	0.00	0.00	0.00	0.00	0.00	0.00	0.00	0.00	0.00	0.00	0.80	0.00	0.00	0.00	0.00	0.00	0.00	0.00	0.00	0.00	0.00	0.00	0.45	0.23	0.04	>0.999999999999999	6.1
Q9NWY4	UPF0609 protein C4orf27 OS=Homo sapiens GN=C4orf27 PE=1 SV=2	1.95	0.00	0.00	0.00	0.00	0.00	0.00	0.00	0.00	0.00	0.00	0.80	0.00	0.00	0.00	0.00	0.00	0.00	0.00	0.00	0.00	0.00	0.00	0.00	0.23	0.00	>0.999999999999999	#DIV/0!
P30566	Adenylosuccinate lyase OS=Homo sapiens GN=ADSL PE=1 SV=2	1.47	0.00	0.67	0.00	0.00	0.00	0.00	0.00	0.00	0.00	0.00	0.60	0.00	0.00	0.00	0.00	0.00	0.00	0.00	0.00	0.00	0.00	0.00	0.00	0.23	0.00	>0.999999999999999	#DIV/0!
Q9Y383	Putative RNA-binding protein Luc7-like 2 OS=Homo sapiens GN=LUC7L2 PE=1 SV=2	1.71	0.00	0.00	0.00	0.00	1.02	0.00	0.00	0.00	0.00	0.00	0.00	0.00	0.00	0.00	0.00	0.00	0.00	0.00	0.00	0.00	0.00	0.00	0.00	0.23	0.00	>0.999999999999999	#DIV/0!
Q8TAX9	Gasdermin-B OS=Homo sapiens GN=GSDMB PE=1 SV=2	1.71	0.00	0.00	0.00	0.00	1.02	0.00	0.00	0.00	0.00	0.00	0.00	0.00	0.00	0.00	0.00	0.00	0.00	0.00	0.00	0.00	0.00	0.00	0.00	0.23	0.00	>0.999999999999999	#DIV/0!
O14791	Apolipoprotein L1 OS=Homo sapiens GN=APOL1 PE=1 SV=5	0.00	0.00	0.00	1.21	0.00	0.00	0.00	0.00	0.00	0.00	1.51	0.00	0.00	0.00	2.67	0.00	1.51	0.00	1.27	0.00	0.25	0.00	1.23	0.45	0.23	0.61	>0.999999999999999	0.4
Q9Y371	Endophilin-B1 OS=Homo sapiens GN=SH3GLB1 PE=1 SV=1	0.00	0.00	0.00	0.00	1.53	1.16	0.00	0.00	0.00	0.00	0.00	0.00	0.00	0.00	0.00	0.00	0.00	0.00	0.00	0.00	0.00	0.00	0.00	0.00	0.22	0.00	>0.999999999999999	#DIV/0!
P07738	Bisphosphoglycerate mutase OS=Homo sapiens GN=BPGM PE=1 SV=2	0.00	0.00	0.00	0.00	0.00	1.60	0.00	0.00	0.00	0.00	0.00	1.10	0.00	0.00	0.00	0.00	0.00	0.00	0.00	0.00	0.34	0.00	0.00	0.00	0.22	0.03	>0.999999999999999	8.0
O95989	Diphosphoinositol polyphosphate phosphohydrolase 1 OS=Homo sapiens GN=NUDT3 PE='	0.00	0.00	0.00	0.00	0.00	0.00	0.00	2.69	0.00	0.00	0.00	0.00	0.00	0.00	1.89	1.17	0.00	2.61	0.00	0.00	0.00	0.00	0.00	0.97	0.22	0.55	>0.999999999999999	0.4
Q15369	Transcription elongation factor B polypeptide 1 OS=Homo sapiens GN=TCEB1 PE=1 SV=1	0.00	0.00	0.00	0.00	0.00	0.00	0.00	0.00	0.00	0.00	0.00	2.69	0.00	0.00	0.00	0.00	0.00	0.00	0.00	0.00	0.00	0.00	0.00	0.00	0.22	0.00	>0.999999999999999	#DIV/0!
A6NHG4	D-dopachrome decarboxylase-like protein OS=Homo sapiens GN=DDTL PE=2 SV=1	0.00	0.00	2.69	0.00	0.00	0.00	0.00	0.00	0.00	0.00	0.00	0.00	2.11	0.00	0.00	0.00	0.00	0.00	0.00	0.00	0.00	0.00	0.00	0.00	0.22	0.18	>0.999999999999999	1.3
P52790	Hexokinase-3 OS=Homo sapiens GN=HK3 PE=1 SV=2	0.00	0.00	0.00	1.06	0.00	0.00	0.00	0.00	0.00	1.04	0.57	0.00	0.00	0.00	0.00	0.00	1.32	0.00	0.00	0.00	0.09	7.90	2.15	0.00	0.22	0.95	>0.999999999999999	0.2
Q9NPQ8	Synembryn-A OS=Homo sapiens GN=RIC8A PE=1 SV=3	1.22	0.00	0.00	0.00	0.00	0.00	0.00	0.00	0.00	0.00	0.95	0.50	0.00	0.00	0.00	0.00	0.00	0.00	0.00	0.00	0.00	0.00	0.00	0.00	0.22	0.00	>0.999999999999999	#DIV/0!
Q9UM07	Protein-arginine deiminase type-4 OS=Homo sapiens GN=PADI4 PE=1 SV=2	0.00	0.00	0.00	0.00	0.00	0.00	0.67	0.00	0.00	1.98	0.00	0.00	0.35	0.00	0.00	0.00	0.00	0.00	0.00	0.00	0.00	0.00	0.61	0.00	0.22	0.08	>0.999999999999999	2.8
Q9H3P7	Golgi resident protein GCP60 OS=Homo sapiens GN=ACBD3 PE=1 SV=4	0.00	0.00	1.23	0.00	0.00	0.73	0.00	0.00	0.20	0.00	0.00	0.50	0.00	1.01	0.00	0.00	0.00	0.00	0.00	0.00	0.00	0.00	0.00	0.00	0.22	0.08	>0.999999999999999	2.6
Q8TC07	TBC1 domain family member 15 OS=Homo sapiens GN=TBC1D15 PE=1 SV=2	0.00	0.92	0.00	0.00	0.00	1.16	0.00	0.00	0.16	0.00	0.00	0.40	0.00	0.00	0.00	0.00	0.00	0.00	0.00	0.00	0.00	0.00	0.00	0.00	0.22	0.00	>0.999999999999999	#DIV/0!
P42785	Lysosomal Pro-X carboxypeptidase OS=Homo sapiens GN=PRCP PE=1 SV=1	0.00	1.38	0.00	0.00	0.00	0.00	1.01	0.00	0.24	0.00	0.00	0.00	0.00	0.00	0.00	0.00	0.00	0.00	0.00	0.00	0.00	0.00	0.92	0.00	0.22	0.08	>0.999999999999999	2.9
Q99623	Prohibitin-2 OS=Homo sapiens GN=PHB2 PE=1 SV=2	0.00	0.00	0.00	0.00	0.00	0.00	0.00	1.59	0.00	1.04	0.00	0.00	0.00	0.00	0.00	0.00	0.00	0.00	0.00	0.00	0.00	0.00	0.00	0.00	0.22	0.00	>0.999999999999999	#DIV/0!
P27824	Calnexin OS=Homo sapiens GN=CANX PE=1 SV=2	0.00	0.00	0.00	0.00	0.00	0.00	0.00	1.59	0.00	1.04	0.00	0.00	0.00	0.00	0.00	0.00	0.00	0.00	0.00	0.00	0.00	0.00	0.00	0.00	0.22	0.00	>0.999999999999999	#DIV/0!
Q309B1	Tripartite motif-containing protein 16-like protein OS=Homo sapiens GN=TRIM16L PE=2 SV=	1.95	0.00	0.00	0.00	0.00	0.00	0.00	0.00	0.67	0.00	0.00	0.00	0.00	0.00	0.00	0.00	0.00	0.00	0.00	0.00	0.00	0.00	0.00	0.00	0.22	0.00	>0.999999999999999	#DIV/0!
Q9H9S4	Calcium-binding protein 39-like OS=Homo sapiens GN=CAB39L PE=1 SV=3	0.00	0.00	0.00	0.00	0.00	2.62	0.00	0.00	0.00	0.00	0.00	0.00	0.00	0.00	0.00	0.00	0.00	0.00	0.00	0.00	0.00	0.00	0.00	0.45	0.22	0.04	>0.999999999999999	5.8
P09668	Pro-cathepsin H OS=Homo sapiens GN=CTSH PE=1 SV=4	0.00	0.00	0.00	0.00	0.00	2.62	0.00	0.00	0.00	0.00	0.00	0.00	0.00	0.00	0.00	0.00	0.00	0.00	0.00	0.00	0.00	0.00	0.00	0.51	0.22	0.04	>0.999999999999999	5.1
Q96FN9	Probable D-tyrosyl-tRNA(Tyr) deacylase 2 OS=Homo sapiens GN=C14orf126 PE=2 SV=1	0.00	0.00	0.00	0.00	0.00	2.62	0.00	0.00	0.00	0.00	0.00	0.00	0.00	0.00	0.00	0.00	0.00	0.00	0.00	0.00	0.00	0.00	0.00	0.00	0.22	0.00	>0.999999999999999	#DIV/0!
Q9BRK5	45 kDa calcium-binding protein OS=Homo sapiens GN=SDF4 PE=1 SV=1	0.00	0.00	0.00	0.00	0.00	0.00	1.35	1.27	0.00	0.00	0.00	0.00	0.00	0.00	0.89	0.00	4.90	1.23	0.00	0.00	0.25	0.00	0.00	0.00	0.22	0.61	>0.999999999999999	0.4
P19021	Peptidyl-glycine alpha-amidating monooxygenase OS=Homo sapiens GN=PAM PE=1 SV=2	0.73	0.00	0.00	0.00	0.58	0.00	0.51	0.48	0.00	0.31	0.00	0.00	0.26	0.00	0.00	0.00	0.00	0.00	0.48	0.00	0.00	0.00	0.00	0.00	0.22	0.06	>0.999999999999999	3.5
Q9NWV8	BRISC and BRCA1-A complex member 1 OS=Homo sapiens GN=BABAM1 PE=1 SV=1	0.00	0.00	0.00	0.00	0.00	0.00	0.00	0.00	0.00	0.00	1.70	0.90	0.00	0.00	0.00	0.00	0.00	0.00	0.00	0.00	0.00	0.00	0.00	0.00	0.22	0.00	>0.999999999999999	#DIV/0!
Q96DH6	RNA-binding protein Musashi homolog 2 OS=Homo sapiens GN=MSI2 PE=1 SV=1	0.00	0.00	0.00	0.00	0.00	0.00	0.00	0.00	0.00	0.00	1.70	0.90	0.00	0.00	0.00	0.00	0.00	0.00	0.00	1.17	0.00	0.00	0.00	0.00	0.22	0.10	>0.999999999999999	2.2
Q9H361	Polyadenylate-binding protein 3 OS=Homo sapiens GN=PABPC3 PE=1 SV=2	0.00	0.00	0.00	0.00	0.96	0.00	0.84	0.79	0.00	0.00	0.00	0.00	0.00	0.00	0.00	0.34	0.00	0.00	0.00	0.00	0.46	0.00	0.77	0.00	0.22	0.13	>0.999999999999999	1.6
P21926	CD9 antigen OS=Homo sapiens GN=CD9 PE=1 SV=4	0.00	0.00	0.00	0.00	0.00	0.00	0.00	2.06	0.52	0.00	0.00	0.00	0.00	0.00	0.00	0.00	0.00	0.00	4.28	0.00	0.00	0.00	0.00	0.00	0.21	0.36	>0.999999999999999	0.6
P52306	Rap1 GTPase-GDP dissociation stimulator 1 OS=Homo sapiens GN=RAP1GDS1 PE=1 SV=	1.22	1.15	0.00	0.00	0.00	0.00	0.00	0.00	0.20	0.00	0.00	0.00	0.00	0.00	0.00	0.00	0.00	0.00	0.00	0.00	0.00	0.00	0.00	0.00	0.21	0.00	>0.999999999999999	#DIV/0!
Q15393	Splicing factor 3B subunit 3 OS=Homo sapiens GN=SF3B3 PE=1 SV=4	0.49	0.00	0.00	0.00	0.00	0.73	0.00	0.00	0.20	0.00	0.95	0.20	0.00	0.00	0.00	0.00	0.00	0.31	0.00	0.26	0.06	0.00	0.00	0.00	0.21	0.05	>0.999999999999999	4.1
P27216	Annexin A13 OS=Homo sapiens GN=ANXA13 PE=1 SV=3	2.20	0.00	0.00	0.00	0.00	0.00	0.00	0.00	0.36	0.00	0.00	0.00	0.00	1.81	0.00	0.62	0.00	1.38	0.00	0.00	0.28	0.00	0.00	0.51	0.21	0.38	>0.999999999999999	0.6
Q07617	Sperm-associated antigen 1 OS=Homo sapiens GN=SPAG1 PE=1 SV=3	0.73	1.38	0.00	0.00	0.00	0.44	0.00	0.00	0.00	0.00	0.00	0.00	0.00	0.00	0.00	0.00	0.00	0.00	0.00	0.39	0.00	0.00	0.00	0.00	0.21	0.03	>0.999999999999999	6.6
Q5T4S7	E3 ubiquitin-protein ligase UBR4 OS=Homo sapiens GN=UBR4 PE=1 SV=1	0.73	0.23	0.11	0.15	0.19	0.44	0.17	0.00	0.04	0.00	0.19	0.30	0.09	0.20	0.00	0.07	0.00	0.00	0.00	0.26	0.00	0.00	0.00	0.06	0.21	0.06	>0.999999999999999	3.8
Q9BUL8	Programmed cell death protein 10 OS=Homo sapiens GN=PDCD10 PE=1 SV=1	0.00	0.00	0.00	0.00	0.00	0.00	0.00	0.00	1.15	0.00	0.00	1.39	0.00	5.84	0.00	0.97	0.00	0.00	0.00	3.76	0.00	2.91	0.00	0.79	0.21	1.19	>0.999999999999999	0.2
Q9UJC3	Protein Hook homolog 1 OS=Homo sapiens GN=HOOK1 PE=1 SV=2	1.95	0.00	0.00	0.00	0.00	0.58	0.00	0.00	0.00	0.00	0.00	0.00	0.00	0.00	0.00	0.00	0.00	0.00	0.00	0.00	0.00	0.00	0.00	0.00	0.21	0.00	>0.999999999999999	#DIV/0!
Q00341	Vigilin OS=Homo sapiens GN=HDLBP PE=1 SV=2	0.00	0.00	0.22	0.00	0.00	1.02	0.00	0.00	0.40	0.00	0.38	0.50	0.18	1.01	0.00	0.00	0.00	0.00	0.00	0.91	0.15	0.00	0.00	0.00	0.21	0.19	>0.999999999999999	1.1
Q9Y5X1	Sorting nexin-9 OS=Homo sapiens GN=SNX9 PE=1 SV=1	1.22	0.00	0.56	0.00	0.00	0.73	0.00	0.00	0.00	0.00	0.00	0.00	0.00	0.00	0.00	0.00	0.00	0.00	0.00	0.00	0.00	0.00	0.00	0.00	0.21	0.00	>0.999999999999999	#DIV/0!
O95671	N-acetylserotonin O-methyltransferase-like protein OS=Homo sapiens GN=ASMTL PE=1 SV	1.22	0.00	0.56	0.00	0.00	0.73	0.00	0.00	0.00	0.00	0.00	0.00	0.00	1.01	0.00	0.00	0.00	0.00	0.00	0.00	0.00	0.00	0.00	0.00	0.21	0.08	>0.999999999999999	2.5
Q9HCD5	Nuclear receptor coactivator 5 OS=Homo sapiens GN=NCOA5 PE=1 SV=2	0.00	1.15	0.00	0.00	0.96	0.00	0.00	0.00	0.40	0.00	0.00	0.00	0.00	0.00	0.00	0.00	0.00	0.00	0.00	0.00	0.00	0.00	0.00	0.00	0.21	0.00	>0.999999999999999	#DIV/0!
O60635	Tetraspanin-1 OS=Homo sapiens GN=TSPAN1 PE=1 SV=2	0.00	0.00	0.00	0.00	0.00	0.00	2.02	0.00	0.48	0.00	0.00	0.00	0.00	0.00	0.00	0.00	0.00	0.00	0.00	0.00	0.00	0.00	0.00	0.00	0.21	0.00	>0.999999999999999	#DIV/0!
Q8N6T3	ADP-ribosylation factor GTPase-activating protein 1 OS=Homo sapiens GN=ARFGAP1 PE='	1.71	0.00	0.78	0.00	0.00	0.00	0.00	0.00	0.00	0.00	0.00	0.00	0.00	0.00	0.00	0.00	0.00	0.00	0.00	0.00	0.00	0.00	0.00	0.00	0.21	0.00	>0.999999999999999	#DIV/0!
Q9Y606	tRNA pseudouridine synthase A, mitochondrial OS=Homo sapiens GN=PUS1 PE=1 SV=3	1.71	0.00	0.78	0.00	0.00	0.00	0.00	0.00	0.00	0.00	0.00	0.00	0.00	0.00	0.00	0.00	0.00	0.00	0.00	0.00	0.00	0.00	0.00	0.00	0.21	0.00	>0.999999999999999	#DIV/0!
Q8NHP1	Aflatoxin B1 aldehyde reductase member 4 OS=Homo sapiens GN=AKR7L PE=2 SV=6	0.00	0.00	2.13	0.00	0.00	0.00	0.00	0.00	0.36	0.00	0.00	0.00	0.00	0.00	0.00	0.00	0.00	2.91	0.00	0.00	0.28	3.95	0.00	0.00	0.21	0.59	>0.999999999999999	0.3
P01893	Putative HLA class I histocompatibility antigen, alpha chain H OS=Homo sapiens GN=HLA-H	0.00	0.00	0.00	0.00	0.00	2.47	0.00	0.00	0.00	0.00	0.00	0.00	0.00	0.00	0.00	0.00	0.00	0.00	0.00	0.00	0.00	0.00	0.00	0.00	0.21	0.00	>0.999999999999999	#DIV/0!
O43324	Eukaryotic translation elongation factor 1 epsilon-1 OS=Homo sapiens GN=EEF1E1 PE=1 S	0.00	0.00	0.00	0.00	0.00	2.47	0.00	0.00	0.00	0.00	0.00	0.00	0.00	0.00	0.00	0.00	0.00	0.00	0.00	0.00	0.00	0.00	0.00	0.00	0.21	0.00	>0.999999999999999	#DIV/0!
O14796	SH2 domain-containing protein 1B OS=Homo sapiens GN=SH2D1B PE=1 SV=2	0.00	0.00	2.47	0.00	0.00	0.00	0.00	0.00	0.00	0.00	0.00	0.00	0.00	0.00	0.00	0.00	0.00	0.00	0.00	0.00	0.00	0.00	0.00	0.00	0.21	0.00	>0.999999999999999	#DIV/0!
Q9HD15	Steroid receptor RNA activator 1 OS=Homo sapiens GN=SRA1 PE=1 SV=1	0.00	0.00	0.00	0.00	0.00	0.00	0.00	0.00	0.00	0.00	2.46	0.00	0.00	0.00	0.00	0.00	0.00	0.00	0.00	1.68	0.00	0.00	0.00	0.00	0.21	0.14	>0.999999999999999	1.5
Q14118	Dystroglycan OS=Homo sapiens GN=DAG1 PE=1 SV=2	0.00	0.00	0.34	0.00	0.58	0.00	0.51	0.48	0.00	0.00	0.57	0.00	0.26	0.00	0.00	0.21	1.32	0.00	0.00	0.00	0.09	0.00	0.00	0.17	0.21	0.17	>0.999999999999999	1.2
Q15560	Transcription elongation factor A protein 2 OS=Homo sapiens GN=TCEA2 PE=1 SV=1	0.00	0.00	0.00	0.00	0.00	1.46	0.00	0.00	0.00	0.00	0.00	1.00	0.00	0.00	0.00	0.00	0.00	0.00	0.00	0.00	0.00	0.00	0.00	0.00	0.20	0.00	>0.999999999999999	#DIV/0!
Q8NFF5	FAD synthase OS=Homo sapiens GN=FLAD1 PE=1 SV=1	1.22	0.00	0.00	0.00	0.00	0.73	0.00	0.00	0.00	0.00	0.00	0.50	0.00	0.00	0.00	0.00	0.00	0.00	0.00	0.00	0.00	0.00	0.00	0.00	0.20	0.00	>0.999999999999999	#DIV/0!
P07992	DNA excision repair protein ERCC-1 OS=Homo sapiens GN=ERCC1 PE=1 SV=1	2.44	0.00	0.00	0.00	0.00	0.00	0.00	0.00	0.00	0.00	0.00	0.00	0.00	0.00	0.00	0.00	0.00	0.00	0.00	0.00	0.00	0.00	0.00	0.00	0.20	0.00	>0.999999999999999	#DIV/0!
Q9NRD1	F-box only protein 6 OS=Homo sapiens GN=FBXO6 PE=1 SV=1	2.44	0.00	0.00	0.00	0.00	0.00	0.00	0.00	0.00	0.00	0.00	0.00	0.88	0.00	0.00	0.00	0.00	0.00	0.00	0.00	0.00	0.00	0.00	0.57	0.20	0.12	>0.999999999999999	1.7
Q9NVM6	DnaJ homolog subfamily C member 17 OS=Homo sapiens GN=DNAJC17 PE=1 SV=1	2.44	0.00	0.00	0.00	0.00	0.00	0.00	0.00	0.00	0.00	0.00	0.00	0.00	0.00	0.00	0.00	0.00	0.00	0.00	0.00	0.00	0.00	0.00	0.00	0.20	0.00	>0.999999999999999	#DIV/0!
O75792	Ribonuclease H2 subunit A OS=Homo sapiens GN=RNASEH2A PE=1 SV=2	2.44	0.00	0.00	0.00	0.00	0.00	0.00	0.00	0.00	0.00	0.00	0.00	0.00	0.00	0.00	0.00	0.00	0.00	0.00	0.00	0.00	0.00	0.00	0.00	0.20	0.00	>0.999999999999999	#DIV/0!
P41226	Ubiquitin-like modifier-activating enzyme 7 OS=Homo sapiens GN=UBA7 PE=1 SV=2	0.73	0.00	0.00	0.00	0.00	0.87	0.00	0.00	0.24	0.00	0.00	0.60	0.00	0.00	0.00	0.00	0.00	0.00	0.00	0.39	0.00	0.62	0.00	0.00	0.20	0.08	>0.999999999999999	2.4
P27361	Mitogen-activated protein kinase 3 OS=Homo sapiens GN=MAPK3 PE=1 SV=4	0.00	0.00	0.00	0.00	0.00	1.16	0.00	1.27	0.00	0.00	0.00	0.00	0.00	0.00	0.00	0.55	0.00	0.00	0.00	0.00	0.00	0.00	0.00	0.91	0.20	0.12	>0.999999999999999	1.7
Q8N4P3	Guanosine-3',5'-bis(diphosphate) 3'-pyrophosphohydrolase MESH1 OS=Homo sapiens GN=	0.00	0.00	0.00	0.00	2.43	0.00	0.00	0.00	0.00	0.00	0.00	0.00	0.00	0.00	0.00	0.00	0.00	0.00	0.00	0.00	0.00	0.00	0.00	0.00	0.20	0.00	>0.999999999999999	#DIV/0!
Q8NBL1	Protein O-glucosyltransferase 1 OS=Homo sapiens GN=POGLUT1 PE=1 SV=1	1.71	0.00	0.00	0.00	0.00	0.00	0.00	0.00	0.00	0.00	0.00	0.70	0.00	0.00	0.00	0.00	0.00	0.00	0.00	0.00	0.00	0.00	0.00	0.40	0.20	0.03	>0.999999999999999	6.1
P30622	CAP-Gly domain-containing linker protein 1 OS=Homo sapiens GN=CLIP1 PE=1 SV=2	0.49	0.00	0.22	0.00	0.00	0.58	0.34	0.00	0.00	0.00	0.76	0.00	0.18	0.00	0.00	0.00	0.00	0.00	0.00	0.00	0.00	0.00	0.00	0.00	0.20	0.01	>0.999999999999999	13.6
Q6ZVX7	Non-specific cytotoxic cell receptor protein 1 homolog OS=Homo sapiens GN=NCCRP1 PE=	0.00	0.00	1.23	0.00	0.00	0.00	0.00	0.00	0.00	1.15	0.00	0.00	0.00	0.00	1.22	0.76	0.00	0.00	0.00	1.43	0.00	0.00	0.00	0.00	0.20	0.28	>0.999999999999999	0.7
Q99828	Calcium and integrin-binding protein 1 OS=Homo sapiens GN=CIB1 PE=1 SV=4	0.00	0.00	0.00	0.00	0.00	0.00	0.00	2.38	0.00	0.00	0.00	0.00	0.00	0.00	0.00	0.00	0.00	0.00	0.00	1.94	0.00	0.00	0.00	0.00	0.20	0.16	>0.999999999999999	1.2
P30626	Sorcin OS=Homo sapiens GN=SRI PE=1 SV=1	0.00	0.00	0.00	0.00	0.00	0.00	0.00	2.38	0.00	0.00	0.00	0.00	0.00	0.00	0.00	0.00	0.00	0.00	0.00	0.00	0.00	0.00	0.00	0.00	0.20	0.00	>0.999999999999999	#DIV/0!
Q9Y394	Dehydrogenase/reductase SDR family member 7 OS=Homo sapiens GN=DHRS7 PE=1 SV=	0.00	0.00	0.00	0.00	0.00	0.00	0.00	1.43	0.00	0.94	0.00	0.00	0.00	0.00	0.00	0.00	0.00	0.00	0.00	0.00	0.00	0.00	0.00	0.00	0.20	0.00	>0.999999999999999	#DIV/0!
P09525	Annexin A4 OS=Homo sapiens GN=ANXA4 PE=1 SV=4	0.00	0.00	0.00	0.00	0.00	0.00	0.00	1.43	0.00	0.94	0.00	0.00	0.00	0.00	0.00	0.00	0.00	0.00	0.00	0.00	0.00	0.00	0.00	0.00	0.20	0.00	>0.999999999999999	#DIV/0!
Q3ZCM7	Tubulin beta-8 chain OS=Homo sapiens GN=TUBB8 PE=1 SV=2	0.00	0.00	2.35	0.00	0.00	0.00	0.00	0.00	0.00	0.00	0.00	0.00	0.00	0.00	0.00	0.00	0.00	0.00	0.00	0.00	0.00	0.00	0.00	0.00	0.20	0.00	>0.999999999999999	#DIV/0!
Q9NRG4	N-lysine methyltransferase SMYD2 OS=Homo sapiens GN=SMYD2 PE=1 SV=2	0.00	0.00	0.00	0.00	0.00	1.02	0.00	0.00	0.00	0.00	1.33	0.00	0.00	0.00	0.00	0.00	0.00	0.00	0.00	0.00	0.00	0.00	0.00	0.00	0.20	0.00	>0.999999999999999	#DIV/0!
Q9UKK3	Poly [ADP-ribose] polymerase 4 OS=Homo sapiens GN=PARP4 PE=1 SV=3	0.73	0.00	0.22	0.00	0.00	0.73	0.00	0.00	0.08	0.00	0.38	0.20	0.00	0.00	0.00	0.14	0.00	0.00	0.00	0.39	0.00	0.00	0.00	0.11	0.20	0.05	>0.999999999999999	3.7
Q6YP21	Kynurenine--oxoglutarate transaminase 3 OS=Homo sapiens GN=CCBL2 PE=1 SV=1	1.47	0.00	0.00	0.00	0.00	0.87	0.00	0.00	0.00	0.00	0.00	0.00	0.00	0.00	0.00	0.00	0.00	0.00	0.00	0.00	0.18	0.00	0.00	0.34	0.19	0.04	>0.999999999999999	4.5
Q8NCA5	Protein FAM98A OS=Homo sapiens GN=FAM98A PE=1 SV=1	1.47	0.00	0.00	0.00	0.00	0.87	0.00	0.00	0.00	0.00	0.00	0.00	0.00	0.00	0.00	0.00	0.00	0.00	0.00	0.00	0.00	0.00	0.00	0.34	0.19	0.03	>0.999999999999999	6.9
Q96DE0	U8 snoRNA-decapping enzyme OS=Homo sapiens GN=NUDT16 PE=1 SV=2	0.00	0.00	0.00	0.00	0.00	2.33	0.00	0.00	0.00	0.00	0.00	0.00	0.00	0.00	0.00	0.00	0.00	0.00	0.00	0.00	0.00	0.00	0.00	0.00	0.19	0.00	>0.999999999999999	#DIV/0!
Q53HC9	Protein TSSC1 OS=Homo sapiens GN=TSSC1 PE=1 SV=2	0.00	0.00	0.00	0.00	0.00	2.33	0.00	0.00	0.00	0.00	0.00	0.00	0.00	0.00	0.00	0.00	0.00	0.00	0.00	0.00	0.00	0.00	0.00	0.00	0.19	0.00	>0.999999999999999	#DIV/0!
Q6ZNW5	GDP-D-glucose phosphorylase C15orf58 OS=Homo sapiens GN=C15orf58 PE=1 SV=2	0.00	0.00	0.00	0.00	0.00	2.33	0.00	0.00	0.00	0.00	0.00	0.00	0.00	0.00	0.00	0.00	0.00	0.00	0.00	0.00	0.00	0.00	0.00	0.00	0.19	0.00	>0.999999999999999	#DIV/0!
Q96AZ6	Interferon-stimulated gene 20 kDa protein OS=Homo sapiens GN=ISG20 PE=1 SV=2	0.00	0.00	0.00	0.00	0.00	2.33	0.00	0.00	0.00	0.00	0.00	0.00	0.00	0.00	0.00	0.00	0.00	0.00	0.00	0.00	0.00	0.00	0.00	0.00	0.19	0.00	>0.999999999999999	#DIV/0!
Q5T6J7	Probable gluconokinase OS=Homo sapiens GN=C9orf103 PE=2 SV=1	0.00	0.00	0.00	0.00	0.00	2.33	0.00	0.00	0.00	0.00	0.00	0.00	0.00	0.00	0.00	0.00	0.00	0.00	0.00	0.00	0.00	0.00	0.00	0.00	0.19	0.00	>0.999999999999999	#DIV/0!
P50479	PDZ and LIM domain protein 4 OS=Homo sapiens GN=PDLIM4 PE=1 SV=2	0.00	0.00	1.01	0.00	0.00	1.31	0.00	0.00	0.00	0.00	0.00	0.00	0.00	0.00	0.00	0.00	0.00	0.00	0.00	0.00	0.00	0.00	0.00	0.00	0.19	0.00	>0.999999999999999	#DIV/0!
P52888	Thimet oligopeptidase OS=Homo sapiens GN=THOP1 PE=1 SV=2	0.00	0.92	0.00	0.00	0.00	0.00	0.00	0.63	0.00	0.00	0.76	0.00	0.00	0.81	0.00	0.00	0.00	0.00	0.00	0.00	0.00	0.00	0.00	0.00	0.19	0.07	>0.999999999999999	2.9
O14948	Transcription factor EC OS=Homo sapiens GN=TFEC PE=1 SV=1	0.00	0.00	0.00	0.00	0.00	0.00	0.00	0.00	0.00	0.00	1.51	0.80	0.00	0.00	0.00	0.00	0.00	0.00	0.00	0.00	0.00	0.00	0.00	0.00	0.19	0.00	>0.999999999999999	#DIV/0!
P30039	Phenazine biosynthesis-like domain-containing protein OS=Homo sapiens GN=PBLD PE=1 :	0.00	2.30	0.00	0.00	0.00	0.00	0.00	0.00	0.00	0.00	0.00	0.00	0.00	0.00	0.00	0.00	0.00	0.00	0.00	0.00	0.00	0.00	0.00	0.00	0.19	0.00	>0.999999999999999	#DIV/0!
O00533	Neural cell adhesion molecule L1-like protein OS=Homo sapiens GN=CHL1 PE=1 SV=4	0.49	0.00	0.00	0.00	0.00	0.29	0.00	0.00	0.08	0.00	0.95	0.50	0.00	0.00	0.00	0.00	0.00	0.00	0.00	0.00	0.00	0.00	0.00	0.11	0.19	0.01	>0.999999999999999	20.3
P78386	Keratin, type II cuticular Hb5 OS=Homo sapiens GN=KRT85 PE=1 SV=1	0.00	0.00	0.00	0.00	2.30	0.00	0.00	0.00	0.00	0.00	0.00	0.00	0.00	0.00	0.00	0.00	0.00	0.00	0.00	0.00	0.00	0.00	0.00	0.00	0.19	0.00	>0.999999999999999	#DIV/0!
P46063	ATP-dependent DNA helicase Q1 OS=Homo sapiens GN=RECQL PE=1 SV=3	0.98	0.00	0.00	0.00	0.00	1.31	0.00	0.00	0.00	0.00	0.00	0.00	0.00	0.00	0.00	0.00	0.00	0.00	0.00	0.00	0.00	0.00	0.00	0.00	0.19	0.00	>0.999999999999999	#DIV/0!
P15498	Proto-oncogene vav OS=Homo sapiens GN=VAV1 PE=1 SV=4	1.71	0.00	0.00	0.00	0.58	0.00	0.00	0.00	0.00	0.00	0.00	0.00	0.26	0.00	0.00	0.00	0.00	0.00	0.00	0.00	0.00	0.00	0.00	0.17	0.19	0.04	>0.999999999999999	5.3
Q9P2R3	Ankyrin repeat and FYVE domain-containing protein 1 OS=Homo sapiens GN=ANKFY1 PE=	0.73	0.69	0.00	0.00	0.00	0.44	0.00	0.00	0.12	0.00	0.00	0.30	0.00	0.00	0.00	0.00	0.00	0.00	0.00	0.00	0.00	0.00	0.00	0.00	0.19	0.00	>0.999999999999999	#DIV/0!
Q13231	Chitotriosidase-1 OS=Homo sapiens GN=CHIT1 PE=1 SV=1	0.00	0.00	0.00	0.91	0.00	0.00	0.00	0.00	0.00	1.36	0.00	0.00	0.00	0.00	0.00	0.00	0.00	0.00	0.00	0.00	0.00	2.70	0.92	0.00	0.19	0.30	>0.999999999999999	0.6
Q9NZP8	Complement C1r subcomponent-like protein OS=Homo sapiens GN=C1RL PE=1 SV=2	0.00	0.00	0.00	0.00	1.15	0.87	0.00	0.00	0.24	0.00	0.00	0.00	0.00	0.00	1.44	0.00	0.00	0.00	0.00	0.00	0.00	0.00	0.92	0.00	0.19	0.20	>0.999999999999999	1.0
Q01581	Hydroxymethylglutaryl-CoA synthase, cytoplasmic OS=Homo sapiens GN=HMGCS1 PE=1 S	0.00	1.38	0.00	0.00	0.00	0.87	0.00	0.00	0.00	0.00	0.00	0.00	0.53	1.21	0.00	0.00	0.00	0.92	0.00	0.78	0.00	0.00	0.00	0.34	0.19	0.31	>0.999999999999999	0.6
Q8WX93	Palladin OS=Homo sapiens GN=PALLD PE=1 SV=3	0.49	0.00	0.22	0.00	0.38	0.58	0.00	0.00	0.00	0.00	0.38	0.20	0.00	0.00	0.00	0.00	0.00	0.31	0.00	0.26	0.00	0.00	0.00	0.00	0.19	0.05	>0.999999999999999	4.0
P16278	Beta-galactosidase OS=Homo sapiens GN=GLB1 PE=1 SV=2	0.00	0.92	0.00	0.00	0.00	1.31	0.00	0.00	0.00	0.00	0.00	0.00	0.00	0.00	0.00	0.00	0.00	0.00	0.00	0.00	0.00	0.00	0.00	0.00	0.19	0.00	>0.999999999999999	#DIV/0!
P11233	Ras-related protein Ral-A OS=Homo sapiens GN=RALA PE=1 SV=1	0.00	0.00	0.00	0.00	0.00	0.00	0.00	2.22	0.00	0.00	0.00	0.00	0.00	0.00	0.00	0.00	0.00	0.00	0.00	0.00	0.00	0.00	0.00	0.00	0.18	0.00	>0.999999999999999	#DIV/0!
Q04206	Transcription factor p65 OS=Homo sapiens GN=RELA PE=1 SV=2	0.00	1.15	0.56	0.00	0.00	0.00	0.00	0.00	0.00	0.00	0.00	0.50	0.00	0.00	0.00	0.34	0.00	0.77	0.00	1.43	0.00	0.00	0.00	0.00	0.18	0.21	>0.999999999999999	0.9
Q96P70	Importin-9 OS=Homo sapiens GN=IPO9 PE=1 SV=3	1.47	0.00	0.00	0.00	0.00	0.44	0.00	0.00	0.00	0.00	0.00	0.30	0.00	0.00	0.00	0.00	0.00	0.00	0.00	0.39	0.00	0.00	0.00	0.00	0.18	0.03	>0.999999999999999	5.7
Q9H0Q0	Protein FAM49A OS=Homo sapiens GN=FAM49A PE=1 SV=1	2.20	0.00	0.00	0.00	0.00	0.00	0.00	0.00	0.00	0.00	0.00	0.00	0.00	0.00	0.00	0.00	0.00	0.00	0.00	0.00	0.00	0.00	0.00	0.00	0.18	0.00	>0.999999999999999	#DIV/0!
Q96GY0	Protein FAM164A OS=Homo sapiens GN=FAM164A PE=1 SV=2	2.20	0.00	0.00	0.00	0.00	0.00	0.00	0.00	0.00	0.00	0.00	0.00	0.00	0.00	0.00	0.00	0.00	0.00	0.00	0.00	0.00	0.00	0.00	0.00	0.18	0.00	>0.999999999999999	#DIV/0!
Q96HL8	SH3 domain-containing YSC84-like protein 1 OS=Homo sapiens GN=SH3YL1 PE=1 SV=1	2.20	0.00	0.00	0.00	0.00	0.00	0.00	0.00	0.00	0.00	0.00	0.00	0.00	0.00	0.00	0.00	0.00	0.00	0.00	0.00	0.00	0.00	0.00	0.00	0.18	0.00	>0.999999999999999	#DIV/0!
P04839	Cytochrome b-245 heavy chain OS=Homo sapiens GN=CYBB PE=1 SV=2	0.00	0.00	0.00	0.00	0.00	0.00	0.00	0.00	0.00	2.19	0.00	0.00	0.00	0.00	0.00	0.00	0.00	0.00	0.00	0.00	0.00	2.08	0.77	0.00	0.18	0.24	>0.999999999999999	0.8
P35241	Radixin OS=Homo sapiens GN=RDX PE=1 SV=1	0.00	0.00	0.00	0.00	0.00	2.18	0.00	0.00	0.00	0.00	0.00	0.00	0.00	0.00	0.00	0.00	0.00	0.00	0.00	0.00	0.00	0.00	0.00	0.00	0.18	0.00	>0.999999999999999	#DIV/0!
Q00765	Receptor expression-enhancing protein 5 OS=Homo sapiens GN=REEP5 PE=1 SV=3	0.00	0.00	0.00	0.00	0.00	2.18	0.00	0.00	0.00	0.00	0.00	0.00	0.00	0.00	1.67	0.00	0.00	0.00	0.00	0.00	0.00	0.00	0.00	0.00	0.18	0.14	>0.999999999999999	1.3
Q9H2C0	Gigaxonin OS=Homo sapiens GN=GAN PE=1 SV=1	1.22	0.00	0.56	0.00	0.00	0.00	0.00	0.00	0.40	0.00	0.00	0.00	0.00	0.00	0.00	0.34	0.00	0.00	0.00	1.94	0.00	0.00	0.00	0.28	0.18	0.21	>0.999999999999999	0.8
Q8N0U7	Uncharacterized protein C1orf87 OS=Homo sapiens GN=C1orf87 PE=2 SV=1	1.22	0.00	0.00	0.00	0.00	0.00	0.00	0.00	0.00	0.00	0.95	0.00	0.00	0.00	0.00	0.00	0.00	0.00	0.00	0.00	0.00	0.00	0.00	0.00	0.18	0.00	>0.999999999999999	#DIV/0!
Q7L775	EPM2A-interacting protein 1 OS=Homo sapiens GN=EPM2AIP1 PE=1 SV=1	1.22	0.00	0.00	0.00	0.00	0.00	0.00	0.00	0.00	0.00	0.95	0.00	0.00	0.00	0.00	0.00	0.00	0.00	0.00	0.00	0.00	0.00	0.00	0.00	0.18	0.00	>0.999999999999999	#DIV/0!
Q9NU19	TBC1 domain family member 22B OS=Homo sapiens GN=TBC1D22B PE=1 SV=3	0.00	0.00	0.00	0.00	1.15	0.00	1.01	0.00	0.00	0.00	0.00	0.00	0.00	0.00	0.00	0.00	0.00	0.00	0.00	0.00	0.00	0.00	0.00	0.00	0.18	0.00	>0.999999999999999	#DIV/0!
P17812	CTP synthase 1 OS=Homo sapiens GN=CTPS PE=1 SV=2	1.22	0.00	0.00	0.00	0.00	0.73	0.00	0.00	0.20	0.00	0.00	0.00	0.00	0.00	0.00	0.00	0.00	0.00	0.00	0.00	0.00	0.00	0.00	0.00	0.18	0.00	>0.999999999999999	#DIV/0!
Q8N6H7	ADP-ribosylation factor GTPase-activating protein 2 OS=Homo sapiens GN=ARFGAP2 PE='	0.00	0.00	0.67	0.00	0.00	0.87	0.00	0.00	0.00	0.00	0.00	0.60	0.00	0.00	0.00	0.00	0.00	0.00	0.00	0.00	0.00	0.00	0.00	0.34	0.18	0.03	>0.999999999999999	6.3
P60763	Ras-related C3 botulinum toxin substrate 3 OS=Homo sapiens GN=RAC3 PE=1 SV=1	0.00	0.00	0.00	0.00	0.00	0.00	0.00	0.00	2.14	0.00	0.00	0.00	0.00	0.00	0.00	0.00	0.00	0.00	0.00	0.00	0.00	16.00	0.00	0.00	0.18	1.33	>0.999999999999999	0.1
P26196	Probable ATP-dependent RNA helicase DDX6 OS=Homo sapiens GN=DDX6 PE=1 SV=2	1.47	0.00	0.67	0.00	0.00	0.00	0.00	0.00	0.00	0.00	0.00	0.00	0.00	0.00	0.00	0.00	0.00	0.00	0.00	0.00	0.00	0.00	0.00	0.00	0.18	0.00	>0.999999999999999	#DIV/0!
P00505	Aspartate aminotransferase, mitochondrial OS=Homo sapiens GN=GOT2 PE=1 SV=3	0.00	0.00	0.00	0.00	0.00	1.02	0.00	1.11	0.00	0.00	0.00	0.00	0.00	0.00	0.00	0.00	0.00	0.00	0.00	0.00	0.00	0.00	0.00	0.00	0.18	0.00	>0.999999999999999	#DIV/0!
Q9NV70	Exocyst complex component 1 OS=Homo sapiens GN=EXOC1 PE=1 SV=4	0.73	1.38	0.00	0.00	0.00	0.00	0.00	0.00	0.00	0.00	0.00	0.00	0.00	0.00	0.00	0.00	0.00	0.00	0.00	0.00	0.00	0.00	0.00	0.00	0.18	0.00	>0.999999999999999	#DIV/0!
Q3YEC7	Rab-like protein 1 OS=Homo sapiens GN=PARF PE=1 SV=2	0.00	0.92	0.45	0.00	0.00	0.58	0.00	0.00	0.16	0.00	0.00	0.00	0.00	0.00	0.00	0.00	0.00	0.00	0.00	0.00	0.00	0.00	0.00	0.23	0.18	0.02	>0.999999999999999	9.3
O95456	Proteasome assembly chaperone 1 OS=Homo sapiens GN=PSMG1 PE=1 SV=1	0.00	0.00	0.00	0.00	0.00	0.00	0.00	0.00	0.00	0.00	0.00	2.09	0.00	0.00	0.00	0.00	0.00	0.00	0.00	0.00	0.00	0.00	0.00	0.00	0.17	0.00	>0.999999999999999	#DIV/0!
O15498	Synaptobrevin homolog YKT6 OS=Homo sapiens GN=YKT6 PE=1 SV=1	0.00	0.00	0.00	0.00	0.00	0.00	0.00	0.00	0.59	0.00	0.00	1.49	0.00	0.00	0.00	0.00	0.00	2.30	0.00	0.00	0.00	0.00	0.00	0.85	0.17	0.26	>0.999999999999999	0.7
Q9NR31	GTP-binding protein SAR1a OS=Homo sapiens GN=SAR1A PE=1 SV=1	0.00	0.00	0.00	0.00	0.00	0.00	0.00	0.00	0.59	0.00	0.00	1.49	0.00	3.02	0.00	0.00	0.00	0.00	0.00	0.00	0.46	0.00	0.00	0.00	0.17	0.29	>0.999999999999999	0.6

P55008	Allograft inflammatory factor 1 OS=Homo sapiens GN=AIF1 PE=1 SV=1	0.00	0.00	0.00	0.00	0.00	0.00	0.00	0.00	0.00	2.09	0.00	0.00	0.00	0.00	0.00	0.00	0.00	0.00	0.00	0.00	0.00	0.00	3.07	0.00	0.17	0.26	>0.999999999999999	0.7
Q9H0E2	Toll-interacting protein OS=Homo sapiens GN=TOLLIP PE=1 SV=1	0.00	0.00	0.00	0.00	0.00	0.00	0.00	0.00	0.00	0.00	2.08	0.00	0.00	0.00	0.00	0.00	0.00	0.00	0.00	0.00	0.00	0.00	0.00	0.00	0.17	0.00	>0.999999999999999	#DIV/0!
Q99538	Legumain OS=Homo sapiens GN=LGMN PE=1 SV=1	0.00	0.00	0.78	0.00	0.00	1.02	0.00	0.00	0.28	0.00	0.00	0.00	0.00	0.00	0.00	0.00	0.00	0.00	0.00	0.00	0.00	0.00	0.00	0.40	0.17	0.03	>0.999999999999999	5.2
Q9UHD1	Cysteine and histidine-rich domain-containing protein 1 OS=Homo sapiens GN=CHORDC1 F	0.00	2.07	0.00	0.00	0.00	0.00	0.00	0.00	0.00	0.00	0.00	0.00	0.00	1.81	0.00	0.00	0.00	0.00	0.00	0.00	0.00	0.00	0.00	0.00	0.17	0.15	>0.999999999999999	1.1
Q96IY4	Carboxypeptidase B2 OS=Homo sapiens GN=CPB2 PE=1 SV=2	0.00	0.00	0.00	0.00	1.34	0.00	0.00	0.00	0.00	0.73	0.00	0.00	0.00	0.00	0.78	1.52	0.00	0.00	1.11	0.00	0.00	0.00	1.07	0.40	0.17	0.41	>0.999999999999999	0.4
Q9P2M7	Cingulin OS=Homo sapiens GN=CGN PE=1 SV=2	0.49	0.00	0.56	0.00	0.00	1.02	0.00	0.00	0.00	0.00	0.00	0.00	0.00	0.40	0.00	0.00	0.00	0.00	0.00	0.00	0.00	0.00	0.00	0.00	0.17	0.03	>0.999999999999999	5.1
Q5R3I4	Tetratricopeptide repeat protein 38 OS=Homo sapiens GN=TTC38 PE=1 SV=1	1.47	0.00	0.00	0.00	0.00	0.00	0.00	0.00	0.00	0.00	0.00	0.60	0.00	0.00	0.00	0.00	0.00	0.00	0.00	0.00	0.00	0.00	0.00	0.00	0.17	0.00	>0.999999999999999	#DIV/0!
Q15125	3-beta-hydroxysteroid-Delta(8),Delta(7)-isomerase OS=Homo sapiens GN=EBP PE=1 SV=3	0.00	0.00	0.00	0.00	0.00	0.00	0.00	2.06	0.00	0.00	0.00	0.00	0.00	0.00	0.00	0.00	0.00	0.00	0.00	0.00	0.00	0.00	0.00	0.00	0.17	0.00	>0.999999999999999	#DIV/0!
P00740	Coagulation factor IX OS=Homo sapiens GN=F9 PE=1 SV=2	0.00	0.00	0.00	0.91	1.15	0.00	0.00	0.00	0.00	0.00	0.00	0.00	0.00	0.00	0.67	0.00	0.00	0.00	0.95	0.00	0.00	0.00	0.00	0.00	0.17	0.13	>0.999999999999999	1.3
P15169	Carboxypeptidase N catalytic chain OS=Homo sapiens GN=CPN1 PE=1 SV=1	0.00	0.00	0.00	0.91	1.15	0.00	0.00	0.00	0.00	0.00	0.00	0.00	0.00	0.00	0.00	0.00	0.00	0.00	4.44	0.00	0.00	0.00	0.00	0.00	0.17	0.37	>0.999999999999999	0.5
Q14155	Rho guanine nucleotide exchange factor 7 OS=Homo sapiens GN=ARHGEF7 PE=1 SV=2	0.98	0.92	0.00	0.00	0.00	0.00	0.00	0.00	0.16	0.00	0.00	0.00	0.00	0.00	0.00	0.00	0.00	0.00	0.00	0.00	0.00	0.00	0.00	0.00	0.17	0.00	>0.999999999999999	#DIV/0!
O95544	NAD kinase OS=Homo sapiens GN=NADK PE=1 SV=1	0.00	0.00	0.00	0.00	0.00	0.00	0.00	0.00	0.00	0.73	1.33	0.00	0.00	0.00	0.00	0.00	0.00	0.00	0.00	0.00	0.00	2.91	0.00	0.00	0.17	0.24	>0.999999999999999	0.7
P19388	DNA-directed RNA polymerases I, II, and III subunit RPABC1 OS=Homo sapiens GN=POLR	0.00	0.00	0.00	0.00	0.00	2.04	0.00	0.00	0.00	0.00	0.00	0.00	0.00	0.00	0.00	0.00	0.00	0.00	0.00	0.00	0.00	0.00	0.00	0.00	0.17	0.00	>0.999999999999999	#DIV/0!
Q8N1A6	UPF0462 protein C4orf33 OS=Homo sapiens GN=C4orf33 PE=1 SV=2	0.00	0.00	0.00	0.00	0.00	2.04	0.00	0.00	0.00	0.00	0.00	0.00	0.00	0.00	0.00	0.00	0.00	0.00	0.00	0.00	0.00	0.00	0.00	0.00	0.17	0.00	>0.999999999999999	#DIV/0!
Q8WUD1	Ras-related protein Rab-2B OS=Homo sapiens GN=RAB2B PE=1 SV=1	0.00	0.00	0.00	0.00	0.00	2.04	0.00	0.00	0.00	0.00	0.00	0.00	0.00	0.00	0.00	0.97	0.00	0.00	2.22	0.00	0.00	6.03	0.00	1.65	0.17	0.90	>0.999999999999999	0.2
Q86WR0	Coiled-coil domain-containing protein 25 OS=Homo sapiens GN=CCDC25 PE=1 SV=2	0.00	0.00	0.00	0.00	0.00	2.04	0.00	0.00	0.00	0.00	0.00	0.00	0.00	0.00	0.00	0.00	0.00	0.00	0.00	1.81	0.00	0.00	0.00	0.00	0.17	0.15	>0.999999999999999	1.1
Q8WZ82	Ovarian cancer-associated gene 2 protein OS=Homo sapiens GN=OVCA2 PE=1 SV=1	0.00	0.00	0.00	0.00	0.00	2.04	0.00	0.00	0.00	0.00	0.00	0.00	0.00	2.82	0.00	0.00	0.00	0.00	0.00	1.81	0.43	0.00	0.00	0.79	0.17	0.49	>0.999999999999999	0.3
Q6ZWC4	Putative uncharacterized protein LOC100128429 OS=Homo sapiens PE=5 SV=1	0.00	0.00	0.00	0.00	0.00	2.04	0.00	0.00	0.00	0.00	0.00	0.00	0.00	0.00	0.00	0.00	0.00	0.00	0.00	0.00	0.00	0.00	0.00	0.00	0.17	0.00	>0.999999999999999	#DIV/0!
P52294	Importin subunit alpha-1 OS=Homo sapiens GN=KPNA1 PE=1 SV=3	0.00	0.00	0.00	0.00	0.00	1.60	0.00	0.00	0.44	0.00	0.00	0.00	0.00	0.00	0.00	0.00	0.00	0.00	0.00	0.00	0.00	0.00	0.00	0.00	0.17	0.00	>0.999999999999999	#DIV/0!
Q9Y5Y2	Cytosolic Fe-S cluster assembly factor NUBP2 OS=Homo sapiens GN=NUBP2 PE=1 SV=1	0.00	0.00	0.00	0.00	0.00	1.60	0.00	0.00	0.44	0.00	0.00	0.00	0.00	0.00	0.00	0.00	0.00	0.00	0.00	0.00	0.00	0.00	0.00	0.00	0.17	0.00	>0.999999999999999	#DIV/0!
Q8TE68	Epidermal growth factor receptor kinase substrate 8-like protein 1 OS=Homo sapiens GN=El	0.98	0.00	0.90	0.00	0.00	0.00	0.00	0.00	0.16	0.00	0.00	0.00	0.00	0.00	0.00	0.00	0.00	0.00	0.00	0.52	0.25	0.00	0.00	0.23	0.17	0.08	>0.999999999999999	2.0
Q9Y6Y8	SEC23-interacting protein OS=Homo sapiens GN=SEC23IP PE=1 SV=1	0.73	0.00	0.00	0.00	0.00	0.87	0.00	0.00	0.12	0.00	0.00	0.30	0.00	0.00	0.00	0.00	0.00	0.00	0.00	0.00	0.00	0.00	0.00	0.00	0.17	0.00	>0.999999999999999	#DIV/0!
O94788	Retinal dehydrogenase 2 OS=Homo sapiens GN=ALDH1A2 PE=1 SV=3	0.00	0.00	2.02	0.00	0.00	0.00	0.00	0.00	0.00	0.00	0.00	0.00	0.00	0.00	0.00	0.00	0.00	0.00	0.00	0.00	0.00	0.00	0.00	0.00	0.17	0.00	>0.999999999999999	#DIV/0!
Q9UQM7	Calcium/calmodulin-dependent protein kinase type II subunit alpha OS=Homo sapiens GN=(	0.00	0.00	0.00	0.00	0.00	0.87	0.00	0.00	0.00	0.00	1.14	0.00	0.00	0.00	0.00	0.00	0.00	0.92	0.00	0.00	0.00	0.00	0.00	0.34	0.17	0.11	>0.999999999999999	1.6
Q13325	Interferon-induced protein with tetratricopeptide repeats 5 OS=Homo sapiens GN=IFIT5 PE=	0.00	0.00	0.00	0.00	0.00	0.87	0.00	0.00	0.00	0.00	1.14	0.00	0.00	0.00	0.00	0.00	0.00	0.00	0.00	0.00	0.00	0.00	0.00	0.00	0.17	0.00	>0.999999999999999	#DIV/0!
Q96JB5	CDK5 regulatory subunit-associated protein 3 OS=Homo sapiens GN=CDK5RAP3 PE=1 SV	0.00	0.00	0.00	0.00	0.00	0.87	0.00	0.00	0.00	0.00	1.14	0.00	0.00	0.00	0.00	0.00	0.00	0.00	0.00	0.00	0.00	0.00	0.00	0.00	0.17	0.00	>0.999999999999999	#DIV/0!
P51659	Peroxisomal multifunctional enzyme type 2 OS=Homo sapiens GN=HSD17B4 PE=1 SV=3	0.98	0.00	0.45	0.00	0.00	0.58	0.00	0.00	0.00	0.00	0.00	0.00	0.00	0.00	0.00	0.00	0.00	0.00	0.00	0.00	0.00	0.00	0.00	0.00	0.17	0.00	>0.999999999999999	#DIV/0!
Q9Y223	Bifunctional UDP-N-acetylglucosamine 2-epimerase/N-acetylmannosamine kinase OS=Hom	0.00	0.00	0.00	0.00	0.00	1.16	0.67	0.00	0.16	0.00	0.00	0.00	0.35	1.61	0.44	0.00	0.75	0.00	0.63	1.04	0.12	0.83	0.00	0.23	0.17	0.50	>0.999999999999999	0.3
Q7Z3J2	UPF0505 protein C16orf62 OS=Homo sapiens GN=C16orf62 PE=1 SV=2	0.00	0.69	0.00	0.00	0.00	0.44	0.00	0.00	0.00	0.00	0.57	0.30	0.00	0.00	0.00	0.00	0.00	0.00	0.39	0.00	0.00	0.00	0.00	0.17	0.17	0.05	>0.999999999999999	3.6
Q14CN4	Keratin, type II cytoskeletal 72 OS=Homo sapiens GN=KRT72 PE=1 SV=2	0.00	0.00	0.00	0.00	0.00	0.00	0.00	0.00	0.00	1.98	0.00	0.00	0.00	0.00	0.00	0.00	0.00	0.00	0.00	0.00	0.00	0.00	0.00	0.00	0.17	0.00	>0.999999999999999	#DIV/0!
P11215	Integrin alpha-M OS=Homo sapiens GN=ITGAM PE=1 SV=2	0.00	0.00	0.00	0.00	0.00	0.00	0.00	0.00	0.00	1.98	0.00	0.00	0.00	0.00	0.00	0.00	0.00	0.00	0.00	0.00	0.00	1.04	0.00	0.00	0.17	0.09	>0.999999999999999	1.9
Q9UJY5	ADP-ribosylation factor-binding protein GGA1 OS=Homo sapiens GN=GGA1 PE=1 SV=1	1.22	0.00	0.56	0.00	0.00	0.00	0.00	0.00	0.20	0.00	0.00	0.00	0.00	0.00	0.00	0.00	0.00	0.00	0.00	0.00	0.00	0.00	0.00	0.00	0.16	0.00	>0.999999999999999	#DIV/0!
Q8IYD1	Eukaryotic peptide chain release factor GTP-binding subunit ERF3B OS=Homo sapiens GN:	0.00	0.00	1.12	0.00	0.00	0.00	0.84	0.00	0.00	0.00	0.00	0.00	0.00	0.00	1.11	0.00	0.00	0.00	0.00	0.00	0.15	0.00	0.77	0.00	0.16	0.17	>0.999999999999999	1.0
Q9P2H3	Intraflagellar transport protein 80 homolog OS=Homo sapiens GN=IFT80 PE=1 SV=3	0.00	0.00	0.45	0.00	0.00	0.00	0.00	0.00	0.00	0.00	1.51	0.00	0.00	0.00	0.00	0.00	0.00	0.00	0.00	0.00	0.00	0.00	0.00	0.00	0.16	0.00	>0.999999999999999	#DIV/0!
Q08752	Peptidyl-prolyl cis-trans isomerase D OS=Homo sapiens GN=PPID PE=1 SV=3	0.00	0.00	0.00	0.00	0.00	1.16	0.00	0.00	0.00	0.00	0.00	0.80	0.00	0.00	0.00	0.00	0.00	0.00	0.00	0.00	0.00	0.00	0.00	0.00	0.16	0.00	>0.999999999999999	#DIV/0!
Q9NXR7	BRCA1-A complex subunit BRE OS=Homo sapiens GN=BRE PE=1 SV=2	0.00	0.00	0.00	0.00	0.00	1.16	0.00	0.00	0.00	0.00	0.00	0.80	0.00	0.00	0.00	0.00	0.00	0.00	0.00	0.00	0.00	0.00	0.00	0.00	0.16	0.00	>0.999999999999999	#DIV/0!
Q03426	Mevalonate kinase OS=Homo sapiens GN=MVK PE=1 SV=1	1.95	0.00	0.00	0.00	0.00	0.00	0.00	0.00	0.00	0.00	0.00	0.00	0.00	0.00	0.00	0.00	0.00	0.00	0.00	0.00	0.00	0.00	0.00	0.00	0.16	0.00	>0.999999999999999	#DIV/0!
Q67FW5	UDP-GlcNAc:betaGal beta-1,3-N-acetylglucosaminyltransferase-like protein 1 OS=Homo sa|	1.95	0.00	0.00	0.00	0.00	0.00	0.00	0.00	0.00	0.00	0.00	0.00	0.00	0.00	0.00	0.00	0.00	0.00	0.00	0.00	0.00	0.00	0.00	0.00	0.16	0.00	>0.999999999999999	#DIV/0!
Q15942	Zyxin OS=Homo sapiens GN=ZYX PE=1 SV=1	1.22	0.00	0.00	0.00	0.00	0.73	0.00	0.00	0.00	0.00	0.00	0.00	0.00	1.01	0.00	0.00	0.00	0.00	0.00	0.00	0.00	0.00	0.00	0.00	0.16	0.08	>0.999999999999999	1.9
Q9BZE9	Tether containing UBX domain for GLUT4 OS=Homo sapiens GN=ASPSCR1 PE=1 SV=1	1.22	0.00	0.00	0.00	0.00	0.73	0.00	0.00	0.00	0.00	0.00	0.00	0.00	1.01	0.00	0.00	0.00	0.00	0.00	0.00	0.00	0.00	0.00	0.00	0.16	0.08	>0.999999999999999	1.9
O14976	Cyclin-G-associated kinase OS=Homo sapiens GN=GAK PE=1 SV=2	0.49	0.00	0.22	0.00	0.00	0.73	0.00	0.00	0.00	0.00	0.00	0.50	0.00	0.00	0.00	0.00	0.00	0.00	0.00	0.00	0.00	0.00	0.00	0.00	0.16	0.00	>0.999999999999999	#DIV/0!
O75636	Ficolin-3 OS=Homo sapiens GN=FCN3 PE=1 SV=2	0.00	0.00	0.00	0.00	1.92	0.00	0.00	0.00	0.00	0.00	0.00	0.00	0.00	0.00	0.00	0.00	0.00	0.00	0.00	0.00	0.00	0.00	0.00	0.00	0.16	0.00	>0.999999999999999	#DIV/0!
Q15485	Ficolin-2 OS=Homo sapiens GN=FCN2 PE=1 SV=2	0.00	0.00	0.00	0.00	1.92	0.00	0.00	0.00	0.00	0.00	0.00	0.00	0.00	0.00	0.00	0.00	1.89	0.00	0.00	0.00	0.00	0.00	0.00	0.00	0.16	0.16	>0.999999999999999	1.0
Q9UIA9	Exportin-7 OS=Homo sapiens GN=XPO7 PE=1 SV=3	0.00	0.69	0.00	0.00	0.00	0.73	0.00	0.00	0.00	0.00	0.00	0.50	0.00	0.60	0.00	0.00	0.00	0.00	0.39	0.00	1.04	0.00	0.00	0.00	0.16	0.17	>0.999999999999999	0.9
O43447	Peptidyl-prolyl cis-trans isomerase H OS=Homo sapiens GN=PPIH PE=1 SV=1	0.00	0.00	1.90	0.00	0.00	0.00	0.00	0.00	0.00	0.00	0.00	0.00	0.00	0.00	0.00	0.00	0.00	0.00	0.00	0.00	0.00	0.00	0.00	0.97	0.16	0.08	>0.999999999999999	2.0
A8MPT4	Glutathione S-transferase theta-4 OS=Homo sapiens GN=GSTT4 PE=3 SV=2	0.00	0.00	0.00	0.00	0.00	0.00	0.00	1.90	0.00	0.00	0.00	0.00	0.00	0.00	0.00	0.83	0.00	0.00	0.00	0.00	0.00	0.00	0.00	0.00	0.16	0.07	>0.999999999999999	2.3
P30042	ES1 protein homolog, mitochondrial OS=Homo sapiens GN=C21orf33 PE=1 SV=3	0.00	0.00	0.00	0.00	0.00	0.00	0.00	1.90	0.00	0.00	0.00	0.00	0.00	0.00	0.00	0.00	0.00	0.00	0.00	0.00	0.00	0.00	0.00	0.00	0.16	0.00	>0.999999999999999	#DIV/0!
P38117	Electron transfer flavoprotein subunit beta OS=Homo sapiens GN=ETFB PE=1 SV=3	0.00	0.00	0.00	0.00	0.00	0.00	0.00	1.90	0.00	0.00	0.00	0.00	0.00	0.00	0.00	0.00	0.00	0.00	0.00	0.00	0.00	0.00	0.00	0.00	0.16	0.00	>0.999999999999999	#DIV/0!
Q5H9R7	Serine/threonine-protein phosphatase 6 regulatory subunit 3 OS=Homo sapiens GN=PPP6F	0.73	0.69	0.00	0.00	0.00	0.00	0.00	0.48	0.00	0.00	0.00	0.00	0.00	0.00	0.00	0.00	0.00	0.00	0.00	0.00	0.00	0.00	0.00	0.00	0.16	0.00	>0.999999999999999	#DIV/0!
Q96I99	Succinyl-CoA ligase [GDP-forming] subunit beta, mitochondrial OS=Homo sapiens GN=SUC	0.00	0.00	0.78	0.00	0.00	0.00	0.00	1.11	0.00	0.00	0.00	0.00	0.00	0.00	0.00	0.00	0.00	0.00	0.00	0.00	0.00	0.00	0.00	0.00	0.16	0.00	>0.999999999999999	#DIV/0!
Q5VSP4	Putative lipocalin 1-like protein 1 OS=Homo sapiens GN=LCN1P1 PE=5 SV=1	0.00	0.00	0.00	0.00	0.00	0.00	0.00	0.00	0.00	0.00	0.00	1.89	0.00	3.83	0.00	0.00	0.00	0.00	0.00	0.59	0.00	0.00	0.00	0.00	0.16	0.37	>0.999999999999999	0.4
P05161	Ubiquitin-like protein ISG15 OS=Homo sapiens GN=ISG15 PE=1 SV=5	0.00	0.00	0.00	0.00	0.00	0.00	0.00	0.00	0.00	0.00	0.00	1.89	0.00	0.00	0.00	0.00	0.00	0.00	0.00	0.00	0.00	0.00	0.00	0.00	0.16	0.00	>0.999999999999999	#DIV/0!
Q66LE6	Serine/threonine-protein phosphatase 2A 55 kDa regulatory subunit B delta isoform OS=Hor	0.00	0.00	0.00	0.00	0.00	1.89	0.00	0.00	0.00	0.00	0.00	0.00	0.00	0.00	0.00	0.00	0.00	0.00	0.00	0.00	0.00	0.00	0.00	0.00	0.16	0.00	>0.999999999999999	#DIV/0!
P78330	Phosphoserine phosphatase OS=Homo sapiens GN=PSPH PE=1 SV=2	0.00	0.00	0.00	0.00	0.00	1.89	0.00	0.00	0.00	0.00	0.00	0.00	0.00	0.00	0.00	0.00	0.00	0.00	0.00	0.00	0.00	0.00	0.00	0.00	0.16	0.00	>0.999999999999999	#DIV/0!
Q9UBQ5	Eukaryotic translation initiation factor 3 subunit K OS=Homo sapiens GN=EIF3K PE=1 SV=1	0.00	0.00	0.00	0.00	0.00	1.89	0.00	0.00	0.00	0.00	0.00	0.00	0.00	0.00	0.00	0.00	0.00	1.68	0.00	0.00	0.00	0.00	0.00	0.00	0.16	0.14	>0.999999999999999	1.1
Q5VWZ2	Lysophospholipase-like protein 1 OS=Homo sapiens GN=LYPLAL1 PE=1 SV=3	0.00	0.00	0.00	0.00	0.00	1.89	0.00	0.00	0.00	0.00	0.00	0.00	0.00	0.00	0.00	0.00	0.00	0.00	0.00	0.00	0.00	0.00	0.00	0.00	0.16	0.00	>0.999999999999999	#DIV/0!
Q14696	LDLR chaperone MESD OS=Homo sapiens GN=MESDC2 PE=1 SV=2	0.00	0.00	0.00	0.00	0.00	1.89	0.00	0.00	0.00	0.00	0.00	0.00	0.00	0.00	0.00	0.00	0.00	0.00	0.00	0.00	0.00	0.00	0.00	0.00	0.16	0.00	>0.999999999999999	#DIV/0!
O75934	Pre-mRNA-splicing factor SPF27 OS=Homo sapiens GN=BCAS2 PE=1 SV=1	0.00	0.00	0.00	0.00	0.00	1.89	0.00	0.00	0.00	0.00	0.00	0.00	0.00	0.00	0.00	0.00	0.00	0.00	0.00	0.00	0.00	0.00	0.00	0.00	0.16	0.00	>0.999999999999999	#DIV/0!
Q7L9L4	MOB kinase activator 1B OS=Homo sapiens GN=MOB1B PE=1 SV=3	0.00	0.00	0.00	0.00	0.00	1.89	0.00	0.00	0.00	0.00	0.00	0.00	1.14	0.00	0.00	0.00	0.00	0.00	0.00	0.00	2.70	0.00	0.00	0.00	0.16	0.32	>0.999999999999999	0.5
Q96LR2	Leucine rich adaptor protein 1 OS=Homo sapiens GN=LURAP1 PE=1 SV=1	0.00	0.00	0.00	0.00	0.00	1.89	0.00	0.00	0.00	0.00	0.00	0.00	0.00	0.00	0.00	0.00	0.00	0.00	0.00	0.00	0.00	0.00	0.00	0.00	0.16	0.00	>0.999999999999999	#DIV/0!
Q9UMF0	Intercellular adhesion molecule 5 OS=Homo sapiens GN=ICAM5 PE=1 SV=3	0.00	0.69	0.00	0.00	0.00	0.58	0.00	0.00	0.00	0.31	0.00	0.30	0.00	0.00	0.00	0.00	0.00	0.00	0.00	0.62	0.00	0.00	0.00	0.00	0.16	0.05	>0.999999999999999	3.0
Q8N1N4	Keratin, type II cytoskeletal 78 OS=Homo sapiens GN=KRT78 PE=2 SV=2	0.00	0.00	0.00	0.00	0.00	0.00	0.00	0.00	0.00	1.88	0.00	0.00	0.00	0.00	0.00	0.00	0.00	0.00	0.00	0.00	0.00	0.00	0.00	0.00	0.16	0.00	>0.999999999999999	#DIV/0!
Q9Y2J8	Protein-arginine deiminase type-2 OS=Homo sapiens GN=PADI2 PE=1 SV=2	0.00	0.00	0.00	0.00	0.00	0.00	0.00	0.00	0.00	1.88	0.00	0.00	0.00	0.00	0.00	0.00	0.00	0.00	0.00	0.00	0.00	0.00	0.00	0.00	0.16	0.00	>0.999999999999999	#DIV/0!
P30536	Translocator protein OS=Homo sapiens GN=TSPO PE=1 SV=3	0.00	0.00	0.00	0.00	0.00	0.00	0.00	0.00	0.00	1.88	0.00	0.00	0.00	0.00	0.00	0.00	0.00	0.00	0.00	0.00	0.00	0.00	0.00	0.00	0.16	0.00	>0.999999999999999	#DIV/0!
P62993	Growth factor receptor-bound protein 2 OS=Homo sapiens GN=GRB2 PE=1 SV=1	0.00	0.00	0.00	0.00	0.00	0.00	0.00	0.00	0.52	1.36	0.00	0.00	0.00	0.00	0.00	0.00	0.00	0.00	1.68	0.00	5.82	0.00	0.00	0.00	0.16	0.63	>0.999999999999999	0.2
Q9Y6V7	Probable ATP-dependent RNA helicase DDX49 OS=Homo sapiens GN=DDX49 PE=1 SV=1	0.00	0.00	0.67	0.00	0.00	0.00	0.00	0.95	0.24	0.00	0.00	0.00	0.00	0.00	0.67	0.00	0.00	0.00	0.00	0.00	0.00	0.00	0.00	0.00	0.16	0.06	>0.999999999999999	2.8
P24821	Tenascin OS=Homo sapiens GN=TNC PE=1 SV=3	0.00	0.69	0.11	0.00	0.77	0.00	0.00	0.00	0.00	0.00	0.19	0.10	0.26	0.00	0.89	0.00	0.00	0.63	0.13	0.00	0.00	0.15	0.00	0.00	0.15	0.17	>0.999999999999999	0.9
Q9UBR2	Cathepsin Z OS=Homo sapiens GN=CTSZ PE=1 SV=1	0.00	0.00	0.00	0.00	0.00	1.46	0.00	0.00	0.40	0.00	0.00	0.00	0.00	4.03	0.00	0.69	0.00	0.00	0.00	1.30	0.00	2.08	0.00	0.00	0.15	0.67	>0.999999999999999	0.2
P46109	Crk-like protein OS=Homo sapiens GN=CRKL PE=1 SV=1	0.00	0.00	0.00	0.00	0.00	1.46	0.00	0.00	0.40	0.00	0.00	0.00	0.00	0.00	0.00	0.00	0.00	0.00	0.00	0.00	0.00	0.00	0.00	0.00	0.15	0.00	>0.999999999999999	#DIV/0!
Q9NZ08	Endoplasmic reticulum aminopeptidase 1 OS=Homo sapiens GN=ERAP1 PE=1 SV=3	0.00	0.00	0.00	0.00	0.00	1.31	0.00	0.00	0.24	0.00	0.00	0.30	0.00	0.00	0.00	0.00	0.00	0.46	0.00	0.78	0.00	0.00	0.00	0.00	0.15	0.10	>0.999999999999999	1.5
Q9P0M6	Core histone macro-H2A.2 OS=Homo sapiens GN=H2AFY2 PE=1 SV=3	0.00	1.84	0.00	0.00	0.00	0.00	0.00	0.00	0.00	0.00	0.00	0.00	0.00	0.00	0.00	0.00	0.00	0.00	0.00	0.00	0.00	0.00	0.00	0.00	0.15	0.00	>0.999999999999999	#DIV/0!
Q9H2F3	3 beta-hydroxysteroid dehydrogenase type 7 OS=Homo sapiens GN=HSD3B7 PE=1 SV=2	0.00	1.84	0.00	0.00	0.00	0.00	0.00	0.00	0.00	0.00	0.00	0.00	0.00	0.00	0.00	0.00	0.00	0.00	0.00	0.00	0.00	0.00	0.00	0.00	0.15	0.00	>0.999999999999999	#DIV/0!
P22695	Cytochrome b-c1 complex subunit 2, mitochondrial OS=Homo sapiens GN=UQCRC2 PE=1	0.00	0.00	0.00	0.00	0.00	0.00	0.00	1.11	0.00	0.73	0.00	0.00	0.00	0.00	0.00	0.00	0.00	0.00	0.00	0.00	0.00	0.00	0.00	0.00	0.15	0.00	>0.999999999999999	#DIV/0!
Q6JBY9	CapZ-interacting protein OS=Homo sapiens GN=RCSD1 PE=1 SV=1	0.00	0.00	0.00	0.00	0.00	0.00	0.00	1.11	0.00	0.73	0.00	0.00	0.00	0.00	0.00	0.00	0.00	0.00	0.00	0.00	1.45	0.00	0.00	0.00	0.15	0.12	>0.999999999999999	1.3
Q96HE7	ERO1-like protein alpha OS=Homo sapiens GN=ERO1L PE=1 SV=2	0.00	0.00	0.00	0.00	0.00	0.87	0.00	0.95	0.00	0.00	0.00	0.00	0.00	0.00	0.00	0.00	0.00	0.00	0.00	0.00	0.00	0.00	0.00	0.00	0.15	0.00	>0.999999999999999	#DIV/0!
Q09028	Histone-binding protein RBBP4 OS=Homo sapiens GN=RBBP4 PE=1 SV=3	0.00	0.00	0.78	0.00	0.00	1.02	0.00	0.00	0.00	0.00	0.00	0.00	0.61	0.00	0.00	0.00	1.07	0.00	1.81	0.00	1.45	0.00	0.00	0.00	0.15	0.41	>0.999999999999999	0.4
Q8TBX8	Phosphatidylinositol-5-phosphate 4-kinase type-2 gamma OS=Homo sapiens GN=PIP4K2C	0.00	0.00	0.78	0.00	0.00	1.02	0.00	0.00	0.00	0.00	0.00	0.00	0.00	0.00	0.00	0.00	0.00	0.00	0.00	0.00	0.00	0.00	0.00	0.00	0.15	0.00	>0.999999999999999	#DIV/0!
P00973	2'-5'-oligoadenylate synthase 1 OS=Homo sapiens GN=OAS1 PE=1 SV=4	0.00	0.00	0.78	0.00	0.00	1.02	0.00	0.00	0.00	0.00	0.00	0.00	0.00	1.41	0.00	0.00	0.00	0.00	0.00	0.00	0.00	0.00	0.00	0.00	0.15	0.12	>0.999999999999999	1.3
Q96HR9	Receptor expression-enhancing protein 6 OS=Homo sapiens GN=REEP6 PE=1 SV=1	0.00	0.00	1.79	0.00	0.00	0.00	0.00	0.00	0.00	0.00	0.00	0.00	0.00	0.00	0.00	0.00	0.00	0.00	0.00	0.00	0.00	0.00	0.00	0.00	0.15	0.00	>0.999999999999999	#DIV/0!
Q9UHJ6	Sedoheptulokinase OS=Homo sapiens GN=SHPK PE=1 SV=3	0.00	0.00	0.67	0.00	0.00	0.87	0.00	0.00	0.24	0.00	0.00	0.00	0.00	0.00	0.00	1.38	0.00	0.00	0.00	0.78	0.00	0.00	0.00	0.34	0.15	0.21	>0.999999999999999	0.7
O75367	Core histone macro-H2A.1 OS=Homo sapiens GN=H2AFY PE=1 SV=4	0.00	0.00	0.00	0.00	0.00	0.00	0.00	0.00	0.00	1.77	0.00	0.00	0.00	0.00	0.00	0.00	0.00	0.00	0.00	0.00	0.00	0.00	0.00	0.00	0.15	0.00	>0.999999999999999	#DIV/0!
Q9GZN8	UPF0687 protein C20orf27 OS=Homo sapiens GN=C20orf27 PE=1 SV=3	0.00	0.00	0.00	0.00	0.00	0.00	0.00	0.00	0.00	1.77	0.00	0.00	0.00	0.00	0.00	0.00	0.00	0.00	0.00	0.00	0.00	3.53	2.61	0.00	0.15	0.51	>0.999999999999999	0.3
Q13435	Splicing factor 3B subunit 2 OS=Homo sapiens GN=SF3B2 PE=1 SV=2	0.00	0.00	0.00	0.45	0.00	0.44	0.00	0.00	0.00	0.00	0.57	0.30	0.00	0.60	0.00	0.00	0.00	0.00	0.00	0.00	0.00	0.00	0.00	0.00	0.15	0.05	>0.999999999999999	2.9
Q92599	Septin-8 OS=Homo sapiens GN=SEPT8 PE=1 SV=4	0.00	0.00	0.00	0.00	0.00	1.75	0.00	0.00	0.00	0.00	0.00	0.00	0.00	0.00	0.00	0.00	0.00	0.00	0.00	0.00	0.00	0.00	0.00	0.00	0.15	0.00	>0.999999999999999	#DIV/0!
Q01081	Splicing factor U2AF 35 kDa subunit OS=Homo sapiens GN=U2AF1 PE=1 SV=3	0.00	0.00	0.00	0.00	0.00	1.75	0.00	0.00	0.00	0.00	0.00	0.00	0.00	0.00	0.00	0.00	0.00	0.00	0.00	0.00	0.00	0.00	0.00	0.00	0.15	0.00	>0.999999999999999	#DIV/0!
Q08170	Serine/arginine-rich splicing factor 4 OS=Homo sapiens GN=SRSF4 PE=1 SV=2	0.00	0.00	0.00	0.00	0.00	1.75	0.00	0.00	0.00	0.00	0.00	0.00	0.00	0.00	0.00	0.00	0.00	0.00	0.00	0.00	0.00	0.00	0.00	0.00	0.15	0.00	>0.999999999999999	#DIV/0!
Q6UWP2	Dehydrogenase/reductase SDR family member 11 OS=Homo sapiens GN=DHRS11 PE=1 S	0.00	0.00	0.00	0.00	0.00	1.75	0.00	0.00	0.00	0.00	0.00	0.00	0.00	0.00	0.00	0.00	1.84	0.00	0.00	0.00	0.00	0.00	0.00	0.00	0.15	0.15	>0.999999999999999	0.9
Q9H8W4	Pleckstrin homology domain-containing family F member 2 OS=Homo sapiens GN=PLEKHF	0.00	0.00	0.00	0.00	0.00	1.75	0.00	0.00	0.00	0.00	0.00	0.00	0.00	0.00	0.00	0.00	0.00	0.00	0.00	0.00	0.00	0.00	0.00	0.00	0.15	0.00	>0.999999999999999	#DIV/0!
Q8NHM4	Putative trypsin-6 OS=Homo sapiens GN=TRY6 PE=5 SV=1	0.00	0.00	0.00	0.00	0.00	1.75	0.00	0.00	0.00	0.00	0.00	0.00	0.00	0.00	0.00	0.83	0.00	0.00	0.00	0.00	0.00	0.00	0.00	0.00	0.15	0.07	>0.999999999999999	2.1
Q86V81	THO complex subunit 4 OS=Homo sapiens GN=ALYREF PE=1 SV=3	0.00	0.00	0.00	0.00	0.00	1.75	0.00	0.00	0.00	0.00	0.00	0.00	0.00	0.00	0.00	0.00	0.00	0.00	0.00	0.00	0.00	0.00	0.00	0.00	0.15	0.00	>0.999999999999999	#DIV/0!
P51572	B-cell receptor-associated protein 31 OS=Homo sapiens GN=BCAP31 PE=1 SV=3	0.00	0.00	0.00	0.00	0.00	1.75	0.00	0.00	0.00	0.00	0.00	0.00	0.00	0.00	0.00	0.00	0.00	0.00	0.00	0.00	0.00	0.00	0.00	0.00	0.15	0.00	>0.999999999999999	#DIV/0!
Q712K3	Ubiquitin-conjugating enzyme E2 R2 OS=Homo sapiens GN=UBE2R2 PE=1 SV=1	0.00	0.00	0.00	0.00	0.00	1.75	0.00	0.00	0.00	0.00	0.00	0.00	0.00	0.00	0.00	0.00	0.00	0.00	0.00	0.00	0.00	0.00	0.00	0.00	0.15	0.00	>0.999999999999999	#DIV/0!
Q07955	Serine/arginine-rich splicing factor 1 OS=Homo sapiens GN=SRSF1 PE=1 SV=2	0.00	0.00	0.00	0.00	0.00	1.75	0.00	0.00	0.00	0.00	0.00	0.00	1.05	0.00	0.00	0.00	0.00	0.00	0.00	0.00	0.00	0.00	0.00	0.00	0.15	0.09	>0.999999999999999	1.7
O43240	Kallikrein-10 OS=Homo sapiens GN=KLK10 PE=1 SV=3	0.00	0.00	0.00	0.00	0.00	0.00	0.00	1.74	0.00	0.00	0.00	0.00	0.00	0.00	0.00	0.00	0.00	0.00	0.00	0.00	0.00	0.00	0.00	0.00	0.15	0.00	>0.999999999999999	#DIV/0!
Q12874	Splicing factor 3A subunit 3 OS=Homo sapiens GN=SF3A3 PE=1 SV=1	0.00	0.00	0.00	0.00	0.00	0.00	0.00	0.00	0.00	0.00	1.14	0.60	0.00	0.00	0.00	0.00	0.00	0.00	0.00	0.00	0.00	0.00	0.00	0.00	0.14	0.00	>0.999999999999999	#DIV/0!
Q5VV41	Rho guanine nucleotide exchange factor 16 OS=Homo sapiens GN=ARHGEF16 PE=1 SV='	0.00	0.00	0.00	0.00	0.00	1.16	0.00	0.00	0.16	0.00	0.00	0.40	0.00	0.00	0.00	0.00	0.00	0.00	0.52	0.00	0.00	0.00	0.00	0.23	0.14	0.06	>0.999999999999999	2.3
Q13362	Serine/threonine-protein phosphatase 2A 56 kDa regulatory subunit gamma isoform OS=Ho	1.22	0.00	0.00	0.00	0.00	0.00	0.00	0.00	0.00	0.00	0.00	0.50	0.00	0.00	0.00	0.00	0.00	0.00	0.00	0.00	0.00	0.00	0.00	0.00	0.14	0.00	>0.999999999999999	#DIV/0!
Q15813	Tubulin-specific chaperone E OS=Homo sapiens GN=TBCE PE=1 SV=1	1.22	0.00	0.00	0.00	0.00	0.00	0.00	0.00	0.00	0.00	0.00	0.50	0.00	0.00	0.00	0.00	0.00	0.00	0.00	0.00	0.00	0.00	0.00	0.00	0.14	0.00	>0.999999999999999	#DIV/0!
Q9Y5X3	Sorting nexin-5 OS=Homo sapiens GN=SNX5 PE=1 SV=1	0.00	0.00	0.00	0.00	0.00	1.02	0.00	0.00	0.00	0.00	0.00	0.70	0.00	0.00	0.00	0.00	1.32	0.00	0.00	0.00	1.45	0.00	0.00	0.85	0.14	0.30	>0.999999999999999	0.5
Q5VYY2	Lipase member M OS=Homo sapiens GN=LIPM PE=2 SV=2	0.00	0.00	0.00	0.00	0.00	1.02	0.00	0.00	0.00	0.00	0.00	0.70	0.00	0.00	0.00	0.00	0.00	0.00	0.00	0.00	0.00	0.00	0.00	0.00	0.14	0.00	>0.999999999999999	#DIV/0!
Q49AR2	UPF0489 protein C5orf22 OS=Homo sapiens GN=C5orf22 PE=1 SV=2	1.71	0.00	0.00	0.00	0.00	0.00	0.00	0.00	0.00	0.00	0.00	0.00	0.00	0.00	0.00	0.00	0.00	0.00	0.00	0.00	0.00	0.00	0.00	0.00	0.14	0.00	>0.999999999999999	#DIV/0!
Q9BSL1	Ubiquitin-associated domain-containing protein 1 OS=Homo sapiens GN=UBAC1 PE=1 SV=	1.71	0.00	0.00	0.00	0.00	0.00	0.00	0.00	0.00	0.00	0.00	0.00	0.00	0.00	0.00	0.00	0.00	0.00	0.00	0.00	0.00	0.00	0.00	0.00	0.14	0.00	>0.999999999999999	#DIV/0!
Q13363	C-terminal-binding protein 1 OS=Homo sapiens GN=CTBP1 PE=1 SV=2	1.71	0.00	0.00	0.00	0.00	0.00	0.00	0.00	0.00	0.00	0.00	0.00	0.00	0.00	0.00	0.00	0.00	0.00	0.00	0.00	0.00	0.00	0.00	0.00	0.14	0.00	>0.999999999999999	#DIV/0!
Q8NFZ5	TNFAIP3-interacting protein 2 OS=Homo sapiens GN=TNIP2 PE=1 SV=1	1.71	0.00	0.00	0.00	0.00	0.00	0.00	0.00	0.00	0.00	0.00	0.00	0.00	0.00	0.00	0.00	0.00	0.00	0.00	0.00	0.00	0.00	0.00	0.00	0.14	0.00	>0.999999999999999	#DIV/0!
Q13685	Angio-associated migratory cell protein OS=Homo sapiens GN=AAMP PE=1 SV=2	1.71	0.00	0.00	0.00	0.00	0.00	0.00	0.00	0.00	0.00	0.00	0.00	0.00	0.00	0.00	0.00	0.00	0.00	0.00	0.00	0.00	0.00	0.00	0.00	0.14	0.00	>0.999999999999999	#DIV/0!
P45984	Mitogen-activated protein kinase 9 OS=Homo sapiens GN=MAPK9 PE=1 SV=2	1.71	0.00	0.00	0.00	0.00	0.00	0.00	0.00	0.00	0.00	0.00	0.00	0.00	0.00	0.00	0.00	0.00	0.00	0.00	0.22	0.00	0.00	0.00	0.00	0.14	0.02	>0.999999999999999	7.9
Q99418	Cytohesin-2 OS=Homo sapiens GN=CYTH2 PE=1 SV=2	1.71	0.00	0.00	0.00	0.00	0.00	0.00	0.00	0.00	0.00	0.00	0.00	0.00	0.00	0.00	0.00	0.00	0.00	0.00	0.00	0.00	0.00	0.00	0.00	0.14	0.00	>0.999999999999999	#DIV/0!
Q9Y303	Putative N-acetylglucosamine-6-phosphate deacetylase OS=Homo sapiens GN=AMDHD2 F	1.71	0.00	0.00	0.00	0.00	0.00	0.00	0.00	0.00	0.00	0.00	0.00	0.00	0.00	0.00	0.00	0.00	0.00	0.00	0.00	0.00	0.00	0.00	0.00	0.14	0.00	>0.999999999999999	#DIV/0!
Q9Y366	Intraflagellar transport protein 52 homolog OS=Homo sapiens GN=IFT52 PE=2 SV=3	1.71	0.00	0.00	0.00	0.00	0.00	0.00	0.00	0.00	0.00	0.00	0.00	0.00	0.00	0.00	0.00	0.00	0.00	0.00	0.00	0.00	0.00	0.00	0.00	0.14	0.00	>0.999999999999999	#DIV/0!
Q9HB40	Retinoid-inducible serine carboxypeptidase OS=Homo sapiens GN=SCPEP1 PE=1 SV=1	0.00	0.00	0.00	0.00	0.00	0.87	0.00	0.00	0.24	0.00	0.00	0.60	0.00	1.21	0.00	0.41	0.00	0.00	0.95	0.78	0.00	0.00	0.00	0.34	0.14	0.31	>0.999999999999999	0.5
P07225	Vitamin K-dependent protein S OS=Homo sapiens GN=PROS1 PE=1 SV=1	0.00	0.00	0.00	0.00	0.77	0.00	0.00	0.00	0.00	0.94	0.00	0.00	0.00	0.81	1.44	0.00	0.75	0.00	1.43	0.00	0.00	0.83	0.00	0.23	0.14	0.46	>0.999999999999999	0.3
O75312	Zinc finger protein ZPR1 OS=Homo sapiens GN=ZNF259 PE=1 SV=1	1.47	0.00	0.00	0.00	0.00	0.00	0.00	0.00	0.24	0.00	0.00	0.00	0.00	0.00	0.00	0.00	0.00	0.00	0.00	0.00	0.00	0.00	0.00	0.00	0.14	0.00	>0.999999999999999	#DIV/0!
Q00005	Serine/threonine-protein phosphatase 2A 55 kDa regulatory subunit B beta isoform OS=Hon	0.00	0.00	1.46	0.00	0.00	0.00	0.00	0.00	0.24	0.00	0.00	0.00	0.00	0.00	0.00	0.00	1.13	0.00	0.00	0.18	0.00	0.00	0.00	0.74	0.14	0.17	>0.999999999999999	0.8
P45973	Chromobox protein homolog 5 OS=Homo sapiens GN=CBX5 PE=1 SV=1	0.00	0.00	1.68	0.00	0.00	0.00	0.00	0.00	0.00	0.00	0.00	0.00	0.00	0.00	0.00	0.00	0.00	0.00	0.00	0.00	0.00	0.00	0.00	0.00	0.14	0.00	>0.999999999999999	#DIV/0!
Q08AF3	Schlafen family member 5 OS=Homo sapiens GN=SLFN5 PE=1 SV=1	0.00	0.00	0.00	0.00	0.00	0.87	0.00	0.00	0.24	0.00	0.57	0.00	0.00	0.00	0.00	0.00	0.00	0.00	0.00	0.00	0.00	0.00	0.00	0.00	0.14	0.00	>0.999999999999999	#DIV/0!
Q5SNT6	WASH complex subunit FAM21B OS=Homo sapiens GN=FAM21B PE=1 SV=2	0.49	0.46	0.00	0.00	0.00	0.73	0.00	0.00	0.00	0.00	0.00	0.00	0.00	0.00	0.00	0.00	0.00	0.00	0.00	0.00	0.00	0.00	0.00	0.00	0.14	0.00	>0.999999999999999	#DIV/0!
O95793	Double-stranded RNA-binding protein Staufen homolog 1 OS=Homo sapiens GN=STAU1 PE	0.00	0.00	0.00	0.00	0.00	0.73	0.00	0.00	0.00	0.00	0.95	0.00	0.00	0.00	0.00	0.00	0.00	0.00	0.00	0.00	0.00	0.00	0.00	0.00	0.14	0.00	>0.999999999999999	#DIV/0!
P13498	Cytochrome b-245 light chain OS=Homo sapiens GN=CYBA PE=1 SV=3	0.00	0.00	0.00	0.00	0.00	0.00	0.00	0.00	0.00	1.67	0.00	0.00	0.00	0.00	0.00	0.00	0.00	0.00	0.00	0.00	0.00	0.00	0.00	0.00	0.14	0.00	>0.999999999999999	#DIV/0!
P10163	Basic salivary proline-rich protein 4 OS=Homo sapiens GN=PRB4 PE=1 SV=3	0.00	0.00	1.23	0.00	0.00	0.00	0.00	0.00	0.44	0.00	0.00	0.00	0.00	0.00	0.00	0.00	0.00	0.00	0.00	0.00	0.00	0.00	0.00	0.00	0.14	0.00	>0.999999999999999	#DIV/0!
Q9UKY3	Putative inactive carboxylesterase 4 OS=Homo sapiens GN=CES1P1 PE=5 SV=2	0.00	0.00	0.00	1.67	0.00	0.00	0.00	0.00	0.00	0.00	0.00	0.00	0.00	0.00	0.00	0.00	1.69	0.00	0.00	0.00	0.00	1.69	0.00	0.00	0.14	0.28	>0.999999999999999	0.5
Q8N999	Uncharacterized protein C12orf29 OS=Homo sapiens GN=C12orf29 PE=1 SV=2	0.00	0.00	0.00	0.00	0.00	1.31	0.00	0.00	0.36	0.00	0.00	0.00	0.00	0.00	0.00	0.00	0.00	0.00	0.00	0.00	0.00	0.00	0.00	0.00	0.14	0.00	>0.999999999999999	#DIV/0!
Q13616	Cullin-1 OS=Homo sapiens GN=CUL1 PE=1 SV=2	0.00	0.00	0.45	0.61	0.00	0.58	0.00	0.00	0.00	0.00	0.00	0.00	0.00	0.00	0.00	0.00	0.00	0.00	0.00	0.00	0.00	0.00	0.00	0.00	0.14	0.00	>0.999999999999999	#DIV/0!
P78559	Microtubule-associated protein 1A OS=Homo sapiens GN=MAP1A PE=1 SV=6	0.49	0.23	0.00	0.00	0.00	0.44	0.00	0.00	0.00	0.00	0.38	0.10	0.00	0.00	0.00	0.00	0.00	0.00	0.00	0.00	0.00	0.00	0.00	0.00	0.14	0.00	>0.999999999999999	#DIV/0!
Q16539	Mitogen-activated protein kinase 14 OS=Homo sapiens GN=MAPK14 PE=1 SV=3	0.00	0.00	0.00	0.00	0.00	0.00	0.00	0.00	0.00	0.83	0.00	0.80	0.00	0.00	0.00	0.00	0.00	0.00	0.00	0.00	0.00	0.00	0.00	0.45	0.14	0.04	>0.999999999999999	3.6
Q9Y4X5	E3 ubiquitin-protein ligase ARIH1 OS=Homo sapiens GN=ARIH1 PE=1 SV=2	0.00	0.00	0.00	0.00	0.00	0.73	0.00	0.00	0.40	0.00	0.00	0.50	0.00	0.00	0.00	0.00	0.00	0.00	0.00	0.00	0.00	0.00	0.00	0.00	0.14	0.00	>0.999999999999999	#DIV/0!
Q5QJ74	Tubulin-specific chaperone cofactor E-like protein OS=Homo sapiens GN=TBCEL PE=2 SV=	0.00	1.61	0.00	0.00	0.00	0.00	0.00	0.00	0.00	0.00	0.00	0.00	0.00	0.00	0.00	0.00	0.00	0.00	0.00	0.00	0.00	0.00	0.00	0.00	0.13	0.00	>0.999999999999999	#DIV/0!
Q96KP1	Exocyst complex component 2 OS=Homo sapiens GN=EXOC2 PE=1 SV=1	0.73	0.00	0.00	0.00	0.00	0.87	0.00	0.00	0.00	0.00	0.00	0.00	0.00	0.00	0.00	0.00	0.00	0.00	0.00	0.00	0.00	0.00	0.00	0.00	0.13	0.00	>0.999999999999999	#DIV/0!
Q96H20	Vacuolar-sorting protein SNF8 OS=Homo sapiens GN=SNF8 PE=1 SV=1	0.00	0.00	0.00	0.00	0.00	1.60	0.00	0.00	0.00	0.00	0.00	0.00	0.00	0.00	0.00	0.00	0.00	0.00	0.00	0.00	0.00	0.00	0.00	0.00	0.13	0.00	>0.999999999999999	#DIV/0!
Q9NVT9	Armadillo repeat-containing protein 1 OS=Homo sapiens GN=ARMC1 PE=1 SV=1	0.00	0.00	0.00	0.00	0.00	1.60	0.00	0.00	0.00	0.00	0.00	0.00	0.00	0.00	0.00	0.00	0.00	0.00	0.00	0.00	0.00	0.00	0.00	0.00	0.13	0.00	>0.999999999999999	#DIV/0!
Q8N6M3	Fat storage-inducing transmembrane protein 2 OS=Homo sapiens GN=FITM2 PE=2 SV=1	0.00	0.00	0.00	0.00	0.00	1.60	0.00	0.00	0.00	0.00	0.00	0.00	0.00	0.00	0.00	0.00	0.00	0.00	0.00	0.00	0.00	0.00	0.00	0.00	0.13	0.00	>0.999999999999999	#DIV/0!
Q86U90	YrdC domain-containing protein, mitochondrial OS=Homo sapiens GN=YRDC PE=1 SV=1	0.00	0.00	0.00	0.00	0.00	1.60	0.00	0.00	0.00	0.00	0.00	0.00	0.00	0.00	0.00	0.00	0.00	0.00	0.00	0.00	0.00	0.00	0.00	0.00	0.13	0.00	>0.999999999999999	#DIV/0!
Q5T013	Putative hydroxypyruvate isomerase OS=Homo sapiens GN=HYI PE=2 SV=2	0.00	0.00	0.00	0.00	0.00	1.60	0.00	0.00	0.00	0.00	0.00	0.00	0.00	0.00	0.00	0.00	0.00	0.00	0.00	0.00	0.00	0.00	0.00	0.00	0.13	0.00	>0.999999999999999	#DIV/0!
O75937	DnaJ homolog subfamily C member 8 OS=Homo sapiens GN=DNAJC8 PE=1 SV=2	0.00	0.00	0.00	0.00	0.00	1.60	0.00	0.00	0.00	0.00	0.00	0.00	0.00	0.00	0.00	0.00	0.00	0.00	0.00	0.00	0.00	0.00	0.00	0.00	0.13	0.00	>0.999999999999999	#DIV/0!
Q9NX08	COMM domain-containing protein 8 OS=Homo sapiens GN=COMMD8 PE=1 SV=1	0.00	0.00	0.00	0.00	0.00	0.00	0.00	0.00	0.00	0.00	0.00	1.59	0.00	0.00	0.00	0.00	0.00	0.00	0.00	0.00	0.00	0.00	0.00	0.00	0.13	0.00	>0.999999999999999	#DIV/0!
Q9BV57	1,2-dihydroxy-3-keto-5-methylthiopentene dioxygenase OS=Homo sapiens GN=ADI1 PE=1	0.00	0.00	0.00	0.00	0.00	0.00	0.00	0.00	0.00	0.00	0.00	1.59	0.00	0.00	0.00	0.00	0.00	0.00	0.00	0.00	0.00	0.00	0.00	0.00	0.13	0.00	>0.999999999999999	#DIV/0!
O96007	Molybdopterin synthase catalytic subunit OS=Homo sapiens GN=MOCS2 PE=1 SV=1	0.00	0.00	0.00	0.00	0.00	0.00	0.00	0.00	0.00	0.00	0.00	1.59	0.00	0.00	0.00	0.00	0.00	0.00	0.00	0.00	0.00	0.00	0.00	0.00	0.13	0.00	>0.999999999999999	#DIV/0!
Q9BRG1	Vacuolar protein-sorting-associated protein 25 OS=Homo sapiens GN=VPS25 PE=1 SV=1	0.00	0.00	0.00	0.00	0.00	0.00	0.00	0.00	0.00	0.00	0.00	1.59	0.00	0.00	0.00	0.00	0.00	0.00	0.00	0.00	0.00	0.00	0.00	0.00	0.13	0.00	>0.999999999999999	#DIV/0!
Q05086	Ubiquitin-protein ligase E3A OS=Homo sapiens GN=UBE3A PE=1 SV=4	0.73	0.00	0.00	0.00	0.00	0.44	0.00	0.00	0.12	0.00	0.00	0.30	0.00	0.60	0.00	0.00	0.00	0.46	0.00	0.00	0.00	0.00	0.00	0.17	0.13	0.10	>0.999999999999999	1.3
P45880	Voltage-dependent anion-selective channel protein 2 OS=Homo sapiens GN=VDAC2 PE=1	0.00	0.00	0.00	0.00	0.00	0.00	0.00	1.59	0.00	0.00	0.00	0.00	0.00	0.00	0.00	0.00	0.00	0.00	0.00	0.00	0.00	0.00	0.00	0.00	0.13	0.00	>0.999999999999999	#DIV/0!
Q9UBX7	Kallikrein-11 OS=Homo sapiens GN=KLK11 PE=1 SV=2	0.00	0.00	0.00	0.00	0.00	0.00	0.00	1.59	0.00	0.00	0.00	0.00	0.00	0.00	0.00	0.00	0.00	0.00	0.00	0.31	0.00	0.00	0.00	0.00	0.13	0.03	>0.999999999999999	5.1
P21912	Succinate dehydrogenase [ubiquinone] iron-sulfur subunit, mitochondrial OS=Homo sapiens	0.00	0.00	0.00	0.00	0.00	0.00	0.00	1.59	0.00	0.00	0.00	0.00	0.00	0.00	0.00	0.00	0.00	0.00	0.00	0.00	0.00	0.00	0.00	0.00	0.13	0.00	>0.999999999999999	#DIV/0!
Q16836	Hydroxyacyl-coenzyme A dehydrogenase, mitochondrial OS=Homo sapiens GN=HADH PE=	0.00	0.00	0.00	0.00	0.00	0.00	0.00	1.59	0.00	0.00	0.00	0.00	0.00	0.00	0.00	0.00	0.00	0.00	0.00	0.00	0.00	0.00	0.00	0.00	0.13	0.00	>0.999999999999999	#DIV/0!
P31930	Cytochrome b-c1 complex subunit 1, mitochondrial OS=Homo sapiens GN=UQCRC1 PE=1	0.00	0.00	0.00	0.00	0.00	0.00	0.00	0.95	0.00	0.63	0.00	0.00	0.00	0.00	0.00	0.00	0.00	0.00	0.95	0.00	0.00	0.00	0.00	0.00	0.13	0.08	>0.999999999999999	1.7
O95786	Probable ATP-dependent RNA helicase DDX58 OS=Homo sapiens GN=DDX58 PE=1 SV=2	0.00	0.69	0.00	0.00	0.00	0.87	0.00	0.00	0.00	0.00	0.00	0.00	0.00	0.60	0.00	0.00	0.00	0.00	0.39	0.09	0.00	0.00	0.00	0.17	0.13	0.10	>0.999999999999999	1.2
Q9Y450	HBS1-like protein OS=Homo sapiens GN=HBS1L PE=1 SV=1	0.98	0.00	0.00	0.00	0.00	0.58	0.00	0.00	0.00	0.00	0.00	0.00	0.00	0.00	0.00	0.00	0.00	0.00	0.00	0.00	0.00	0.00	0.00	0.00	0.13	0.00	>0.999999999999999	#DIV/0!
Q93034	Cullin-5 OS=Homo sapiens GN=CUL5 PE=1 SV=4	0.98	0.00	0.00	0.00	0.00	0.58	0.00	0.00	0.00	0.00	0.00	0.00	0.00	0.00	0.00	0.00	0.00	0.00	0.00	0.00	0.00	0.00	0.00	0.00	0.13	0.00	>0.999999999999999	#DIV/0!
Q9Y608	Leucine-rich repeat flightless-interacting protein 2 OS=Homo sapiens GN=LRRFIP2 PE=1 S\	0.98	0.00	0.00	0.00	0.00	0.58	0.00	0.00	0.00	0.00	0.00	0.00	0.00	0.00	0.00	0.00	0.00	0.00	0.00	0.00	0.00	0.00	0.00	0.00	0.13	0.00	>0.999999999999999	#DIV/0!
P39748	Flap endonuclease 1 OS=Homo sapiens GN=FEN1 PE=1 SV=1	0.00	0.00	0.00	1.21	0.00	0.00	0.00	0.00	0.32	0.00	0.00	0.00	0.00	0.00	0.00	0.00	0.00	0.00	0.00	0.25	0.00	0.00	0.00	0.00	0.13	0.02	>0.999999999999999	6.2
Q04756	Hepatocyte growth factor activator OS=Homo sapiens GN=HGFAC PE=1 SV=1	0.00	0.00	0.56	0.00	0.96	0.00	0.00	0.00	0.00	0.00	0.00	0.00	0.00	0.00	0.00	0.00	0.94	0.00	0.00	0.15	0.00	0.00	0.00	0.00	0.13	0.09	>0.999999999999999	1.4
Q13449	Limbic system-associated membrane protein OS=Homo sapiens GN=LSAMP PE=1 SV=2	0.00	0.00	0.00	0.00	0.00	0.00	1.52	0.00	0.00	0.00	0.00	0.00	0.00	0.00	0.00	0.00	0.00	0.00	0.00	0.00	0.00	0.00	0.00	0.00	0.13	0.00	>0.999999999999999	#DIV/0!
Q9H4A6	Golgi phosphoprotein 3 OS=Homo sapiens GN=GOLPH3 PE=1 SV=1	0.00	0.00	1.12	0.00	0.00	0.00	0.00	0.00	0.40	0.00	0.00	0.00	0.88	0.00	0.00	0.69	0.00	0.00	0.00	1.30	0.00	0.00	0.00	0.57	0.13	0.29	>0.999999999999999	0.4
Q9GZN7	Protein rogdi homolog OS=Homo sapiens GN=ROGDI PE=2 SV=1	0.00	0.00	0.00	1.52	0.00	0.00	0.00	0.00	0.00	0.00	0.00	0.00	0.00	0.00	0.00	0.00	0.00	0.00	0.00	0.00	0.00	0.00	0.00	0.00	0.13	0.00	>0.999999999999999	#DIV/0!
Q9H892	Tetratricopeptide repeat protein 12 OS=Homo sapiens GN=TTC12 PE=2 SV=2	0.00	0.00	0.00	0.00	0.00	0.00	0.00	0.00	0.00	0.00	1.51	0.00	0.00	0.00	0.00	0.00	0.00	0.00	0.00	0.00	0.00	0.00	0.00	0.00	0.13	0.00	>0.999999999999999	#DIV/0!
Q66K66	Transmembrane protein 198 OS=Homo sapiens GN=TMEM198 PE=2 SV=1	0.00	0.00	0.00	0.00	0.00	0.00	0.00	0.00	0.00	0.00	1.51	0.00	0.00	0.00	0.00	0.89	0.00	0.00	0.00	0.00	0.00	0.00	0.00	0.45	0.13	0.11	>0.999999999999999	1.1
Q6IA69	Glutamine-dependent NAD(+) synthetase OS=Homo sapiens GN=NADSYN1 PE=1 SV=3	0.00	0.92	0.00	0.00	0.00	0.58	0.00	0.00	0.00	0.00	0.00	0.00	0.00	0.81	0.00	0.00	0.00	0.00	0.00	0.00	0.00	0.00	0.00	0.23	0.13	0.09	>0.999999999999999	1.5
Q9HA65	TBC1 domain family member 17 OS=Homo sapiens GN=TBC1D17 PE=2 SV=2	0.00	0.92	0.00	0.00	0.00	0.58	0.00	0.00	0.00	0.00	0.00	0.00	0.00	0.00	0.00	0.00	0.00	0.00	0.00	0.00	0.00	0.00	0.00	0.00	0.13	0.00	>0.999999999999999	#DIV/0!
P16455	Methylated-DNA--protein-cysteine methyltransferase OS=Homo sapiens GN=MGMT PE=1 S	0.00	0.00	0.00	0.00	0.00	0.00	0.00	0.00	0.00	0.00	0.00	1.49	0.00	0.00	0.00	0.00	0.00	0.00	0.00	0.00	0.00	0.00	0.00	0.00	0.12	0.00	>0.999999999999999	#DIV/0!
Q9Y6A4	UPF0468 protein C16orf80 OS=Homo sapiens GN=C16orf80 PE=1 SV=1	0.00	0.00	0.00	0.00	0.00	0.00	0.00	0.00	0.00	0.00	0.00	1.49	0.00	0.00	0.00	0.00	0.00	0.00	0.00	0.00	0.00	0.00	0.00	0.00	0.12	0.00	>0.999999999999999	#DIV/0!
Q13951	Core-binding factor subunit beta OS=Homo sapiens GN=CBFB PE=1 SV=2	0.00	0.00	0.00	0.00	0.00	0.00	0.00	0.00	0.00	0.00	0.00	1.49	0.00	0.00	0.00	0.00	0.00	0.00	0.00	0.00	0.00	2.30	0.00	0.00	0.12	0.19	>0.999999999999999	0.6
Q9BXX3	Ankyrin repeat domain-containing protein 30A OS=Homo sapiens GN=ANKRD30A PE=1 SV	0.00	0.00	0.22	0.00	0.00	0.29	0.00	0.32	0.08	0.00	0.38	0.20	0.18	0.40	0.22	0.14	0.00	0.31	0.32	0.26	0.06	0.00	0.00	0.11	0.12	0.17	>0.999999999999999	0.7
Q9UHY1	Nuclear receptor-binding protein OS=Homo sapiens GN=NRBP1 PE=1 SV=1	0.00	0.00	0.56	0.00	0.00	0.73	0.00	0.00	0.20	0.00	0.00	0.00	0.00	1.01	0.00	0.00	0.00	0.00	0.00	0.00	1.04	0.00	0.00	0.00	0.12	0.17	>0.999999999999999	0.7
Q9BRX2	Protein pelota homolog OS=Homo sapiens GN=PELO PE=1 SV=2	0.00	0.00	0.00	0.00	0.00	1.16	0.00	0.00	0.32	0.00	0.00	0.00	0.00	0.00	0.00	0.00	0.00	0.00	1.04	0.00	0.00	0.00	0.00	0.00	0.12	0.09	>0.999999999999999	1.4
P55196	Afadin OS=Homo sapiens GN=MLLT4 PE=1 SV=3	0.00	0.46	0.00	0.00	0.00	0.44	0.00	0.00	0.00	0.00	0.38	0.20	0.00	0.00	0.00	0.00	0.00	0.00	0.00	0.00	0.00	0.00	0.00	0.00	0.12	0.00	>0.999999999999999	#DIV/0!
O15198	Mothers against decapentaplegic homolog 9 OS=Homo sapiens GN=SMAD9 PE=1 SV=1	0.00	0.00	0.00	0.00	0.00	0.87	0.00	0.00	0.00	0.00	0.00	0.60	0.00	0.00	0.00	0.00	0.00	0.00	0.00	0.00	0.00	0.00	0.00	0.00	0.12	0.00	>0.999999999999999	#DIV/0!
P78344	Eukaryotic translation initiation factor 4 gamma 2 OS=Homo sapiens GN=EIF4G2 PE=1 SV=	0.73	0.00	0.00	0.00	0.00	0.44	0.00	0.00	0.00	0.00	0.00	0.30	0.00	0.00	0.00	0.00	0.00	0.00	0.00	0.00	0.00	0.00	0.00	0.00	0.12	0.00	>0.999999999999999	#DIV/0!
Q5GLZ8	Probable E3 ubiquitin-protein ligase HERC4 OS=Homo sapiens GN=HERC4 PE=1 SV=1	0.73	0.00	0.00	0.00	0.00	0.44	0.00	0.00	0.00	0.00	0.00	0.30	0.26	0.60	0.00	0.00	0.00	0.46	0.00	0.00	0.00	0.00	0.00	0.17	0.12	0.12	>0.999999999999999	1.0
Q9UMY4	Sorting nexin-12 OS=Homo sapiens GN=SNX12 PE=1 SV=3	0.00	0.00	0.00	0.00	0.00	0.00	0.00	0.00	1.47	0.00	0.00	0.00	0.00	3.42	0.00	0.00	0.00	2.61	0.00	0.00	0.00	0.00	2.61	0.97	0.12	0.80	>0.999999999999999	0.2
Q16630	Cleavage and polyadenylation specificity factor subunit 6 OS=Homo sapiens GN=CPSF6 PE	1.47	0.00	0.00	0.00	0.00	0.00	0.00	0.00	0.00	0.00	0.00	0.00	0.00	0.00	0.00	0.00	0.00	0.00	0.00	0.00	0.00	0.00	0.00	0.00	0.12	0.00	>0.999999999999999	#DIV/0!
Q9UBS8	E3 ubiquitin-protein ligase RNF14 OS=Homo sapiens GN=RNF14 PE=1 SV=1	1.47	0.00	0.00	0.00	0.00	0.00	0.00	0.00	0.00	0.00	0.00	0.00	0.00	0.00	0.00	0.00	0.00	0.00	0.00	0.00	0.00	0.00	0.00	0.00	0.12	0.00	>0.999999999999999	#DIV/0!
Q05519	Serine/arginine-rich splicing factor 11 OS=Homo sapiens GN=SRSF11 PE=1 SV=1	1.47	0.00	0.00	0.00	0.00	0.00	0.00	0.00	0.00	0.00	0.00	0.00	0.00	0.00	0.00	0.00	0.00	0.00	0.00	0.00	0.00	0.00	0.00	0.00	0.12	0.00	>0.999999999999999	#DIV/0!
Q8N9M5	Transmembrane protein 102 OS=Homo sapiens GN=TMEM102 PE=1 SV=1	1.47	0.00	0.00	0.00	0.00	0.00	0.00	0.00	0.00	0.00	0.00	0.00	0.00	0.00	0.00	0.00	0.00	0.00	0.00	0.00	0.00	0.00	0.00	0.00	0.12	0.00	>0.999999999999999	#DIV/0!
Q9UJA5	tRNA (adenine(58)-N(1))-methyltransferase non-catalytic subunit TRM6 OS=Homo sapiens (	1.47	0.00	0.00	0.00	0.00	0.00	0.00	0.00	0.00	0.00	0.00	0.00	0.00	0.00	0.00	0.00	0.00	0.00	0.00	0.00	0.00	0.00	0.00	0.00	0.12	0.00	>0.999999999999999	#DIV/0!
A6NCN2	Keratin-81-like protein OS=Homo sapiens PE=2 SV=3	1.47	0.00	0.00	0.00	0.00	0.00	0.00	0.00	0.00	0.00	0.00	0.00	0.00	0.00	0.00	0.00	0.00	0.00	0.00	0.00	0.00	0.00	0.00	0.00	0.12	0.00	>0.999999999999999	#DIV/0!
Q13043	Serine/threonine-protein kinase 4 OS=Homo sapiens GN=STK4 PE=1 SV=2	1.47	0.00	0.00	0.00	0.00	0.00	0.00	0.00	0.00	0.00	0.00	0.00	0.00	0.00	0.00	0.00	0.00	0.00	0.00	0.00	0.00	0.00	0.00	0.00	0.12	0.00	>0.999999999999999	#DIV/0!
Q14254	Flotillin-2 OS=Homo sapiens GN=FLOT2 PE=1 SV=2	0.00	0.00	0.00	0.00	0.00	0.00	0.00	0.00	1.46	0.00	0.00	0.00	0.00	0.00	0.00	0.00	0.00	0.00	0.00	0.00	0.00	0.00	0.00	0.00	0.12	0.00	>0.999999999999999	#DIV/0!
Q9BZK3	Putative nascent polypeptide-associated complex subunit alpha-like protein OS=Homo sapie	0.00	0.00	0.00	0.00	0.00	0.00	0.00	0.00	0.00	1.46	0.00	0.00	0.00	0.00	0.00	0.00	0.00	4.91	0.00	0.00	6.44	0.00	0.00	0.00	0.12	0.95	>0.999999999999999	0.1
Q6ZN28	Metastasis-associated in colon cancer protein 1 OS=Homo sapiens GN=MACC1 PE=1 SV=2	0.00	0.00	0.00	0.00	0.00	1.46	0.00	0.00	0.00	0.00	0.00	0.00	0.00	0.00	0.00	0.00	0.00	0.00	0.00	0.00	0.00	0.00	0.00	0.17	0.12	0.01	>0.999999999999999	8.5
Q9NSC7	Alpha-N-acetylgalactosaminide alpha-2,6-sialyltransferase 1 OS=Homo sapiens GN=ST6GA	0.00	0.00	0.00	0.00	0.00	1.46	0.00	0.00	0.00	0.00	0.00	0.00	0.00	1.01	0.00	0.00	0.00	0.00	0.00	0.00	0.00	0.00	0.00	0.28	0.12	0.11	>0.999999999999999	1.1
O14562	Ubiquitin domain-containing protein UBFD1 OS=Homo sapiens GN=UBFD1 PE=1 SV=2	0.00	0.00	0.00	0.00	0.00	1.46	0.00	0.00	0.00	0.00	0.00	0.00	0.00	0.00	0.00	0.00	0.00	0.00	0.00	0.00	0.00	0.00	0.00	0.00	0.12	0.00	>0.999999999999999	#DIV/0!
P42126	Enoyl-CoA delta isomerase 1, mitochondrial OS=Homo sapiens GN=ECI1 PE=1 SV=1	0.00	0.00	0.00	0.00	0.00	1.46	0.00	0.00	0.00	0.00	0.00	0.00	0.88	0.00	0.00	0.00	0.00	0.00	0.00	0.00	0.00	0.00	0.00	0.00	0.12	0.07	>0.999999999999999	1.7
P80217	Interferon-induced 35 kDa protein OS=Homo sapiens GN=IFI35 PE=1 SV=5	0.00	0.00	0.00	0.00	0.00	1.46	0.00	0.00	0.00	0.00	0.00	0.00	0.00	0.00	0.00	0.00	0.00	0.00	0.00	0.00	2.08	0.00	0.00	0.00	0.12	0.17	>0.999999999999999	0.7
Q8NFV4	Abhydrolase domain-containing protein 11 OS=Homo sapiens GN=ABHD11 PE=1 SV=1	0.00	0.00	0.00	0.00	0.00	1.46	0.00	0.00	0.00	0.00	0.00	0.00	0.00	0.00	0.00	0.00	0.00	0.00	0.00	0.00	0.00	0.00	0.00	0.00	0.12	0.00	>0.999999999999999	#DIV/0!
A6NDU8	UPF0600 protein C5orf51 OS=Homo sapiens GN=C5orf51 PE=1 SV=1	0.00	0.00	0.00	0.00	0.00	1.46	0.00	0.00	0.00	0.00	0.00	0.00	0.00	0.00	0.00	0.00	0.00	0.00	0.00	0.00	0.00	0.00	0.00	0.00	0.12	0.00	>0.999999999999999	#DIV/0!
Q9H223	EH domain-containing protein 4 OS=Homo sapiens GN=EHD4 PE=1 SV=1	0.00	0.00	0.00	0.00	0.00	0.00	0.00	0.00	0.00	0.00	0.95	0.50	0.00	0.00	0.00	0.00	0.77	0.00	0.00	0.00	0.00	0.00	0.00	0.00	0.12	0.06	>0.999999999999999	1.9
P19838	Nuclear factor NF-kappa-B p105 subunit OS=Homo sapiens GN=NFKB1 PE=1 SV=2	0.00	0.00	0.34	0.00	0.00	0.00	0.00	0.00	0.24	0.00	0.57	0.30	0.00	0.00	0.00	0.21	0.00	0.00	0.00	0.00	0.00	0.00	0.00	0.17	0.12	0.03	>0.999999999999999	3.8
P10314	HLA class I histocompatibility antigen, A-32 alpha chain OS=Homo sapiens GN=HLA-A PE=	0.00	0.00	0.00	0.00	0.00	0.00	0.00	0.00	1.43	0.00	0.00	0.00	0.00	0.00	0.00	0.00	0.00	0.00	0.00	0.00	0.00	0.00	0.00	0.00	0.12	0.00	>0.999999999999999	#DIV/0!
P16435	NADPH--cytochrome P450 reductase OS=Homo sapiens GN=POR PE=1 SV=2	0.00	0.00	0.00	0.00	0.00	0.00	0.00	1.43	0.00	0.00	0.00	0.00	0.00	0.00	0.00	0.00	0.00	0.00	0.00	0.00	0.00	0.00	0.00	0.00	0.12	0.00	>0.999999999999999	#DIV/0!
O14975	Very long-chain acyl-CoA synthetase OS=Homo sapiens GN=SLC27A2 PE=1 SV=2	0.00	0.00	0.00	0.00	0.00	0.00	0.00	1.43	0.00	0.00	0.00	0.00	0.00	0.00	0.00	0.00	0.00	0.00	0.00	0.00	0.00	0.00	0.00	0.00	0.12	0.00	>0.999999999999999	#DIV/0!
Q14257	Reticulocalbin-2 OS=Homo sapiens GN=RCN2 PE=1 SV=1	0.00	0.00	0.00	0.00	0.00	0.00	0.00	1.43	0.00	0.00	0.00	0.00	0.00	0.00	0.00	0.00	0.00	0.00	1.17	0.00	0.00	0.00	0.00	0.00	0.12	0.10	>0.999999999999999	1.2
Q9UJZ1	Stomatin-like protein 2 OS=Homo sapiens GN=STOML2 PE=1 SV=1	0.00	0.00	0.00	0.00	0.00	0.00	0.00	1.43	0.00	0.00	0.00	0.00	0.00	0.00	0.00	0.00	0.00	0.00	0.00	0.00	0.00	0.00	0.00	0.00	0.12	0.00	>0.999999999999999	#DIV/0!
Q7Z2Z2	Elongation factor Tu GTP-binding domain-containing protein 1 OS=Homo sapiens GN=EFTU	0.73	0.69	0.00	0.00	0.00	0.00	0.00	0.00	0.00	0.00	0.00	0.00	0.00	0.00	0.00	0.00	0.00	0.00	0.00	0.00	0.00	0.00	0.00	0.00	0.12	0.00	>0.999999999999999	#DIV/0!
Q7RTR2	Protein NLRC3 OS=Homo sapiens GN=NLRC3 PE=2 SV=2	0.73	0.69	0.00	0.00	0.00	0.00	0.00	0.00	0.00	0.00	0.00	0.00	0.00	0.00	0.00	0.00	0.00	0.00	0.00	0.00	0.00	0.00	0.00	0.00	0.12	0.00	>0.999999999999999	#DIV/0!
Q93052	Lipoma-preferred partner OS=Homo sapiens GN=LPP PE=1 SV=1	1.22	0.00	0.00	0.00	0.00	0.00	0.00	0.00	0.20	0.00	0.00	0.00	0.00	1.01	0.00	0.00	0.00	0.00	0.00	0.00	0.00	0.00	0.00	0.00	0.12	0.08	>0.999999999999999	1.4
Q9NUQ6	SPATS2-like protein OS=Homo sapiens GN=SPATS2L PE=1 SV=2	1.22	0.00	0.00	0.00	0.00	0.00	0.00	0.00	0.20	0.00	0.00	0.00	0.00	0.00	0.00	0.00	0.00	0.00	0.00	0.00	0.00	0.00	0.00	0.00	0.12	0.00	>0.999999999999999	#DIV/0!
Q9P260	LisH domain and HEAT repeat-containing protein KIAA1468 OS=Homo sapiens GN=KIAA14	0.49	0.00	0.00	0.00	0.00	0.73	0.00	0.00	0.00	0.00	0.00	0.20	0.00	0.00	0.00	0.00	0.00	0.00	0.00	0.00	0.00	0.00	0.00	0.11	0.12	0.01	>0.999999999999999	12.5
Q8NCM8	Cytoplasmic dynein 2 heavy chain 1 OS=Homo sapiens GN=DYNC2H1 PE=1 SV=4	0.73	0.00	0.00	0.00	0.00	0.00	0.00	0.00	0.00	0.00	0.57	0.10	0.00	0.00	0.00	0.00	0.00	0.00	0.00	0.00	0.00	0.00	0.00	0.00	0.12	0.00	>0.999999999999999	#DIV/0!
Q7Z4H3	HD domain-containing protein 2 OS=Homo sapiens GN=HDDC2 PE=1 SV=1	0.00	0.00	0.00	0.00	0.00	0.00	0.00	0.00	0.00	0.00	0.00	1.39	0.00	0.00	0.00	0.00	0.00	0.00	0.00	0.00	0.00	0.00	0.00	0.00	0.12	0.00	>0.999999999999999	#DIV/0!
Q9UHR4	Brain-specific angiogenesis inhibitor 1-associated protein 2-like protein 1 OS=Homo sapiens	0.00	1.38	0.00	0.00	0.00	0.00	0.00	0.00	0.00	0.00	0.00	0.00	0.00	0.00	0.00	0.00	0.00	0.00	0.00	0.00	0.00	0.00	0.00	0.00	0.12	0.00	>0.999999999999999	#DIV/0!
Q96A33	Coiled-coil domain-containing protein 47 OS=Homo sapiens GN=CCDC47 PE=1 SV=1	0.00	1.38	0.00	0.00	0.00	0.00	0.00	0.00	0.00	0.00	0.00	0.00	0.00	0.00	0.00	0.00	0.00	0.00	0.00	0.00	0.00	0.00	0.00	0.00	0.12	0.00	>0.999999999999999	#DIV/0!
Q92831	Histone acetyltransferase KAT2B OS=Homo sapiens GN=KAT2B PE=1 SV=3	0.73	0.00	0.34	0.00	0.00	0.00	0.00	0.00	0.00	0.31	0.00	0.00	0.00	0.00	0.00	0.21	0.00	0.00	0.00	0.39	0.00	0.00	0.00	0.00	0.12	0.05	>0.999999999999999	2.3
Q8N474	Secreted frizzled-related protein 1 OS=Homo sapiens GN=SFRP1 PE=1 SV=1	0.00	0.00	1.01	0.00	0.00	0.00	0.00	0.00	0.36	0.00	0.00	0.00	0.00	0.00	0.00	0.00	0.00	0.00	0.00	0.00	0.00	0.00	0.00	0.00	0.11	0.00	>0.999999999999999	#DIV/0!
Q6P4A8	Phospholipase B-like 1 OS=Homo sapiens GN=PLBD1 PE=1 SV=2	0.00	0.00	0.00	0.00	0.00	0.00	0.84	0.00	0.00	0.52	0.00	0.00	0.00	1.01	0.00	0.00	0.00	0.00	0.00	0.00	0.00	0.00	0.00	0.00	0.11	0.08	>0.999999999999999	1.4
P05160	Coagulation factor XIII B chain OS=Homo sapiens GN=F13B PE=1 SV=3	0.00	0.00	0.00	1.36	0.00	0.00	0.00	0.00	0.00	0.00	0.00	0.00	0.00	0.81	0.44	0.28	0.00	0.00	0.63	0.00	0.00	0.00	0.00	0.00	0.11	0.18	>0.999999999999999	0.6
Q7L5D6	Golgi to ER traffic protein 4 homolog OS=Homo sapiens GN=GET4 PE=1 SV=1	0.00	0.00	0.00	1.36	0.00	0.00	0.00	0.00	0.00	0.00	0.00	0.00	0.00	0.00	0.00	0.00	0.00	0.00	0.00	1.17	0.00	0.00	0.00	0.00	0.11	0.10	>0.999999999999999	1.2
Q5K651	Sterile alpha motif domain-containing protein 9 OS=Homo sapiens GN=SAMD9 PE=1 SV=1	0.00	0.46	0.00	0.00	0.00	0.58	0.00	0.32	0.00	0.00	0.00	0.00	0.00	0.40	0.00	0.00	0.00	0.31	0.32	0.26	0.00	0.00	0.00	0.11	0.11	0.12	>0.999999999999999	1.0
Q9BVC6	Transmembrane protein 109 OS=Homo sapiens GN=TMEM109 PE=1 SV=1	0.00	0.00	0.00	0.00	0.00	0.00	0.00	0.00	0.00	1.36	0.00	0.00	0.00	0.00	0.00	0.00	0.00	0.00	0.00	0.00	0.00	0.00	0.00	0.00	0.11	0.00	>0.999999999999999	#DIV/0!
P15941	Mucin-1 OS=Homo sapiens GN=MUC1 PE=1 SV=3	0.00	0.00	0.00	0.00	0.00	0.44	0.00	0.79	0.12	0.00	0.00	0.00	0.26	0.00	0.00	0.00	0.00	0.00	0.00	0.00	0.00	0.00	0.00	0.00	0.11	0.02	>0.999999999999999	5.1
Q99935	Proline-rich protein 1 OS=Homo sapiens GN=PROL1 PE=1 SV=2	0.00	0.00	1.34	0.00	0.00	0.00	0.00	0.00	0.00	0.00	0.00	0.00	0.00	0.00	0.00	0.00	0.00	0.00	0.00	0.00	0.00	0.00	0.00	0.00	0.11	0.00	>0.999999999999999	#DIV/0!
Q2TAA2	Isoamyl acetate-hydrolyzing esterase 1 homolog OS=Homo sapiens GN=IAH1 PE=1 SV=1	0.00	0.00	1.34	0.00	0.00	0.00	0.00	0.00	0.00	0.00	0.00	0.00	0.00	0.00	0.00	0.00	0.00	0.00	0.00	0.00	0.00	0.00	0.00	0.00	0.11	0.00	>0.999999999999999	#DIV/0!
Q8N142	Adenylosuccinate synthetase isozyme 1 OS=Homo sapiens GN=ADSSL1 PE=1 SV=1	0.00	0.00	0.00	0.00	1.34	0.00	0.00	0.00	0.00	0.00	0.00	0.00	0.00	0.00	0.00	0.00	0.00	0.00	0.00	0.00	0.00	0.00	0.00	0.00	0.11	0.00	>0.999999999999999	#DIV/0!
Q15113	Procollagen C-endopeptidase enhancer 1 OS=Homo sapiens GN=PCOLCE PE=1 SV=2	0.00	0.00	0.00	0.00	1.34	0.00	0.00	0.00	0.00	0.00	0.00	0.00	0.00	0.00	0.00	0.00	0.00	0.00	0.00	0.00	0.00	0.00	0.00	0.00	0.11	0.00	>0.999999999999999	#DIV/0!
Q709C8	Vacuolar protein sorting-associated protein 13C OS=Homo sapiens GN=VPS13C PE=1 SV=	0.49	0.46	0.00	0.00	0.00	0.29	0.00	0.00	0.00	0.00	0.00	0.10	0.00	0.00	0.00	0.00	0.00	0.00	0.00	0.00	0.00	0.00	0.00	0.00	0.11	0.00	>0.999999999999999	#DIV/0!
Q14746	Conserved oligomeric Golgi complex subunit 2 OS=Homo sapiens GN=COG2 PE=1 SV=1	0.00	0.00	0.00	0.00	0.00	0.58	0.00	0.00	0.00	0.00	0.76	0.00	0.00	0.00	0.00	0.00	0.00	0.00	0.00	0.00	0.00	0.00	0.00	0.00	0.11	0.00	>0.999999999999999	#DIV/0!
Q5VZK9	Leucine-rich repeat-containing protein 16A OS=Homo sapiens GN=LRRC16A PE=1 SV=1	0.00	0.46	0.00	0.00	0.00	0.29	0.00	0.00	0.00	0.00	0.38	0.20	0.00	0.81	0.22	0.00	0.00	0.00	0.00	0.00	0.00	0.00	0.00	0.11	0.11	0.10	>0.999999999999999	1.2
P16615	Sarcoplasmic/endoplasmic reticulum calcium ATPase 2 OS=Homo sapiens GN=ATP2A2 PE	0.73	0.00	0.00	0.00	0.00	0.00	0.00	0.48	0.12	0.00	0.00	0.00	0.00	0.00	0.00	0.00	0.00	0.00	0.00	0.00	0.00	0.00	0.00	0.00	0.11	0.00	>0.999999999999999	#DIV/0!
Q9BXS5	AP-1 complex subunit mu-1 OS=Homo sapiens GN=AP1M1 PE=1 SV=3	0.00	0.00	0.00	0.00	0.00	0.00	0.00	0.00	0.00	0.00	1.33	0.00	0.00	0.00	0.00	0.00	0.00	0.00	0.00	0.00	0.00	0.00	0.00	0.00	0.11	0.00	>0.999999999999999	#DIV/0!
O76054	SEC14-like protein 2 OS=Homo sapiens GN=SEC14L2 PE=1 SV=1	0.00	0.00	0.00	0.00	0.00	0.00	0.00	0.00	0.00	0.00	1.33	0.00	0.00	0.00	0.00	0.00	0.00	0.00	0.00	0.00	0.00	0.00	0.00	0.00	0.11	0.00	>0.999999999999999	#DIV/0!
Q6IQ49	UPF0667 protein C1orf55 OS=Homo sapiens GN=C1orf55 PE=1 SV=1	0.00	0.00	0.00	0.00	0.00	0.00	0.00	0.00	0.00	0.00	1.33	0.00	0.00	0.00	0.00	0.00	0.00	0.00	0.00	0.00	0.00	0.00	0.00	0.00	0.11	0.00	>0.999999999999999	#DIV/0!

P49748	Very long-chain specific acyl-CoA dehydrogenase, mitochondrial OS=Homo sapiens GN=AC	0.00	0.00	0.00	0.00	0.00	0.00	0.00	0.79	0.00	0.52	0.00	0.00	0.00	0.00	0.00	0.00	0.00	0.00	0.00	0.00	0.00	0.00	0.00	0.00	0.11	0.00	>0.999999999999999	#DIV/0!
P07711	Cathepsin L1 OS=Homo sapiens GN=CTSL1 PE=1 SV=2	0.00	0.00	0.00	0.00	0.00	1.31	0.00	0.00	0.00	0.00	0.00	0.00	0.00	0.00	0.00	0.62	0.00	0.00	0.00	0.00	0.00	0.00	0.00	0.00	0.11	0.05	>0.999999999999999	2.1
P16422	Epithelial cell adhesion molecule OS=Homo sapiens GN=EPCAM PE=1 SV=2	0.00	0.00	0.00	0.00	0.00	1.31	0.00	0.00	0.00	0.00	0.00	0.00	0.00	0.00	0.00	0.00	0.00	0.00	0.00	0.00	0.00	0.00	0.00	0.00	0.11	0.00	>0.999999999999999	#DIV/0!
Q13546	Receptor-interacting serine/threonine-protein kinase 1 OS=Homo sapiens GN=RIPK1 PE=1	0.00	0.00	0.00	0.00	0.00	1.31	0.00	0.00	0.00	0.00	0.00	0.00	0.00	0.00	0.00	0.00	0.00	0.00	0.00	0.00	0.00	0.00	0.00	0.00	0.11	0.00	>0.999999999999999	#DIV/0!
Q9P2W9	Syntaxin-18 OS=Homo sapiens GN=STX18 PE=1 SV=1	0.00	0.00	0.00	0.00	0.00	1.31	0.00	0.00	0.00	0.00	0.00	0.00	0.00	0.00	0.00	0.00	0.00	0.00	0.00	0.00	0.00	0.00	0.00	0.00	0.11	0.00	>0.999999999999999	#DIV/0!
Q96GA7	Serine dehydratase-like OS=Homo sapiens GN=SDSL PE=1 SV=1	0.00	0.00	0.00	0.00	0.00	1.31	0.00	0.00	0.00	0.00	0.00	0.00	0.00	1.81	0.00	0.00	0.00	0.00	0.00	0.00	0.00	4.02	0.00	0.00	0.11	0.49	>0.999999999999999	0.2
Q9GZT4	Serine racemase OS=Homo sapiens GN=SRR PE=1 SV=1	0.00	0.00	0.00	0.00	0.00	1.31	0.00	0.00	0.00	0.00	0.00	0.00	0.79	0.00	0.00	0.00	0.00	0.00	0.00	0.00	0.00	0.00	0.00	0.00	0.11	0.07	>0.999999999999999	1.7
P13804	Electron transfer flavoprotein subunit alpha, mitochondrial OS=Homo sapiens GN=ETFA PE:	0.00	0.00	0.00	0.00	0.00	1.31	0.00	0.00	0.00	0.00	0.00	0.00	0.00	0.00	0.00	0.00	0.00	0.00	0.00	0.00	0.00	0.00	0.00	0.00	0.11	0.00	>0.999999999999999	#DIV/0!
Q13287	N-myc-interactor OS=Homo sapiens GN=NMI PE=1 SV=2	0.00	0.00	0.00	0.00	0.00	1.31	0.00	0.00	0.00	0.00	0.00	0.00	0.00	0.00	0.00	0.00	0.00	0.00	0.00	0.00	0.00	0.00	0.00	0.51	0.11	0.04	>0.999999999999999	2.6
P0C870	JmjC domain-containing protein 7 OS=Homo sapiens GN=JMJD7 PE=2 SV=1	0.00	0.00	0.00	0.00	0.00	1.31	0.00	0.00	0.00	0.00	0.00	0.00	0.00	0.00	0.00	0.00	0.00	0.00	0.00	0.00	0.00	0.00	0.00	0.00	0.11	0.00	>0.999999999999999	#DIV/0!
O15067	Phosphoribosylformylglycinamidine synthase OS=Homo sapiens GN=PFAS PE=1 SV=4	0.00	0.00	0.56	0.00	0.00	0.29	0.00	0.00	0.08	0.00	0.38	0.00	0.00	0.00	0.00	0.00	0.00	0.00	0.00	0.00	0.00	0.00	0.00	0.00	0.11	0.00	>0.999999999999999	#DIV/0!
Q86YR7	Probable guanine nucleotide exchange factor MCF2L2 OS=Homo sapiens GN=MCF2L2 PE	0.73	0.00	0.00	0.00	0.58	0.00	0.00	0.00	0.00	0.00	0.00	0.00	0.00	0.00	0.00	0.00	0.00	0.00	0.00	0.00	0.00	0.00	0.00	0.00	0.11	0.00	>0.999999999999999	#DIV/0!
Q9UBB4	Ataxin-10 OS=Homo sapiens GN=ATXN10 PE=1 SV=1	0.00	0.00	0.00	0.00	0.00	0.00	0.00	0.00	0.00	0.00	0.00	1.29	0.00	0.00	0.00	0.00	0.00	0.00	0.00	0.00	0.00	0.00	0.00	0.00	0.11	0.00	>0.999999999999999	#DIV/0!
P41227	N-alpha-acetyltransferase 10 OS=Homo sapiens GN=NAA10 PE=1 SV=1	0.00	0.00	0.00	0.00	0.00	0.00	0.00	0.00	0.00	0.00	0.00	1.29	1.14	0.00	0.00	0.00	0.00	1.99	0.00	1.68	0.00	0.00	1.99	0.00	0.11	0.57	>0.999999999999999	0.2
P53992	Protein transport protein Sec24C OS=Homo sapiens GN=SEC24C PE=1 SV=3	0.00	0.00	0.00	0.00	0.00	0.87	0.00	0.00	0.12	0.00	0.00	0.30	0.00	0.60	0.00	0.21	0.00	0.00	0.00	0.00	0.00	0.00	0.00	0.17	0.11	0.08	>0.999999999999999	1.3
Q14738	Serine/threonine-protein phosphatase 2A 56 kDa regulatory subunit delta isoform OS=Homo	0.00	0.00	0.56	0.00	0.00	0.73	0.00	0.00	0.00	0.00	0.00	0.00	0.00	0.00	0.00	0.00	0.00	0.00	0.00	0.00	0.00	0.00	0.00	0.00	0.11	0.00	>0.999999999999999	#DIV/0!
Q9UBW5	Bridging integrator 2 OS=Homo sapiens GN=BIN2 PE=1 SV=3	0.00	0.00	0.00	0.76	0.00	0.00	0.00	0.00	0.00	0.52	0.00	0.00	0.44	0.00	0.00	0.00	0.94	0.00	0.00	0.00	0.00	2.29	0.77	0.00	0.11	0.37	>0.999999999999999	0.3
P58107	Epiplakin OS=Homo sapiens GN=EPPK1 PE=1 SV=2	0.24	0.00	0.00	0.15	0.00	0.29	0.17	0.00	0.00	0.42	0.00	0.00	0.09	0.20	0.00	0.00	0.19	0.00	0.32	0.00	0.03	0.21	0.00	0.11	0.11	0.10	>0.999999999999999	1.1
Q9P035	3-hydroxyacyl-CoA dehydratase 3 OS=Homo sapiens GN=PTPLAD1 PE=1 SV=2	0.00	0.00	0.00	0.00	0.00	0.00	0.00	1.27	0.00	0.00	0.00	0.00	0.00	0.00	0.00	0.00	0.00	0.00	0.00	0.00	0.00	0.00	0.00	0.00	0.11	0.00	>0.999999999999999	#DIV/0!
Q9C0C2	182 kDa tankyrase-1-binding protein OS=Homo sapiens GN=TNKS1BP1 PE=1 SV=4	0.98	0.00	0.00	0.00	0.00	0.29	0.00	0.00	0.00	0.00	0.00	0.00	0.00	0.00	0.22	0.14	0.00	0.00	0.00	0.26	0.00	0.00	0.00	0.00	0.11	0.05	>0.999999999999999	2.0
O95721	Synaptosomal-associated protein 29 OS=Homo sapiens GN=SNAP29 PE=1 SV=1	0.00	0.00	1.23	0.00	0.00	0.00	0.00	0.00	0.00	0.00	0.00	0.00	0.00	0.00	0.00	0.00	0.00	0.00	0.00	0.00	0.00	0.00	0.00	0.00	0.10	0.00	>0.999999999999999	#DIV/0!
Q9BTW9	Tubulin-specific chaperone D OS=Homo sapiens GN=TBCD PE=1 SV=2	1.22	0.00	0.00	0.00	0.00	0.00	0.00	0.00	0.00	0.00	0.00	0.00	0.00	0.00	0.00	0.00	0.00	0.00	0.00	0.00	0.00	0.00	0.00	0.00	0.10	0.00	>0.999999999999999	#DIV/0!
Q9NUL3	Double-stranded RNA-binding protein Staufen homolog 2 OS=Homo sapiens GN=STAU2 PE	1.22	0.00	0.00	0.00	0.00	0.00	0.00	0.00	0.00	0.00	0.00	0.00	0.00	0.00	0.00	0.00	0.00	0.00	0.00	0.00	0.00	0.00	0.00	0.00	0.10	0.00	>0.999999999999999	#DIV/0!
Q9NXV6	CDKN2A-interacting protein OS=Homo sapiens GN=CDKN2AIP PE=1 SV=3	1.22	0.00	0.00	0.00	0.00	0.00	0.00	0.00	0.00	0.00	0.00	0.00	0.00	0.00	0.00	0.00	0.00	0.00	0.00	0.00	0.00	0.00	0.00	0.00	0.10	0.00	>0.999999999999999	#DIV/0!
O60684	Importin subunit alpha-7 OS=Homo sapiens GN=KPNA6 PE=1 SV=1	1.22	0.00	0.00	0.00	0.00	0.00	0.00	0.00	0.00	0.00	0.00	0.00	0.00	1.01	0.00	0.00	0.00	0.00	0.00	0.00	0.00	0.00	0.00	0.00	0.10	0.08	>0.999999999999999	1.2
Q96HC4	PDZ and LIM domain protein 5 OS=Homo sapiens GN=PDLIM5 PE=1 SV=5	1.22	0.00	0.00	0.00	0.00	0.00	0.00	0.00	0.00	0.00	0.00	0.00	0.00	1.01	0.00	0.00	0.00	0.00	0.00	2.07	0.00	0.00	0.00	0.00	0.10	0.26	>0.999999999999999	0.4
P40222	Alpha-taxilin OS=Homo sapiens GN=TXLNA PE=1 SV=3	1.22	0.00	0.00	0.00	0.00	0.00	0.00	0.00	0.00	0.00	0.00	0.00	0.00	0.00	0.00	0.00	0.00	0.00	0.00	0.00	0.00	0.00	0.00	0.00	0.10	0.00	>0.999999999999999	#DIV/0!
Q96K76	Ubiquitin carboxyl-terminal hydrolase 47 OS=Homo sapiens GN=USP47 PE=1 SV=3	0.49	0.00	0.00	0.00	0.00	0.29	0.00	0.00	0.24	0.00	0.00	0.20	0.00	0.00	0.00	0.14	0.00	0.00	0.32	0.26	0.00	0.00	0.00	0.11	0.10	0.07	>0.999999999999999	1.5
O00154	Cytosolic acyl coenzyme A thioester hydrolase OS=Homo sapiens GN=ACOT7 PE=1 SV=3	0.00	0.00	0.90	0.00	0.00	0.00	0.00	0.00	0.32	0.00	0.00	0.00	0.00	0.00	0.00	0.00	0.00	0.00	0.00	1.04	0.25	1.66	0.00	0.45	0.10	0.28	>0.999999999999999	0.4
Q66K74	Microtubule-associated protein 1S OS=Homo sapiens GN=MAP1S PE=1 SV=2	0.00	0.00	0.34	0.00	0.00	0.00	0.00	0.00	0.00	0.00	0.57	0.30	0.00	0.60	0.00	0.00	0.00	0.00	0.00	0.00	0.00	0.00	0.00	0.00	0.10	0.05	>0.999999999999999	2.0
Q8N960	Centrosomal protein of 120 kDa OS=Homo sapiens GN=CEP120 PE=2 SV=2	0.00	0.69	0.00	0.00	0.00	0.00	0.51	0.00	0.00	0.00	0.00	0.00	0.00	0.00	0.00	0.00	0.00	0.00	0.00	0.00	0.09	0.00	0.00	0.00	0.10	0.01	>0.999999999999999	13.0
A6NIH7	Protein unc-119 homolog B OS=Homo sapiens GN=UNC119B PE=1 SV=1	0.00	0.00	0.00	0.00	0.00	0.00	0.00	0.00	0.00	0.00	0.00	1.20	0.00	0.00	0.00	0.00	0.00	0.00	0.00	0.00	0.00	0.00	0.00	0.00	0.10	0.00	>0.999999999999999	#DIV/0!
Q9H3H3	UPF0696 protein C11orf68 OS=Homo sapiens GN=C11orf68 PE=1 SV=2	0.00	0.00	0.00	0.00	0.00	0.00	0.00	0.00	0.00	0.00	0.00	1.20	0.00	0.00	0.00	0.00	0.00	0.00	0.00	0.00	0.00	0.00	0.00	0.00	0.10	0.00	>0.999999999999999	#DIV/0!
Q9BUJ2	Heterogeneous nuclear ribonucleoprotein U-like protein 1 OS=Homo sapiens GN=HNRNPUL	0.00	0.00	0.00	0.00	0.00	0.00	0.00	0.00	0.00	0.31	0.57	0.30	0.00	0.00	0.00	0.00	0.00	0.00	0.00	0.39	0.00	0.62	0.00	0.00	0.10	0.08	>0.999999999999999	1.2
Q6IN85	Serine/threonine-protein phosphatase 4 regulatory subunit 3A OS=Homo sapiens GN=SMEI	0.73	0.00	0.00	0.00	0.00	0.44	0.00	0.00	0.00	0.00	0.00	0.00	0.00	0.00	0.00	0.00	0.00	0.00	0.00	0.00	0.00	0.00	0.00	0.00	0.10	0.00	>0.999999999999999	#DIV/0!
Q96T76	MMS19 nucleotide excision repair protein homolog OS=Homo sapiens GN=MMS19 PE=1 S	0.73	0.00	0.00	0.00	0.00	0.44	0.00	0.00	0.00	0.00	0.00	0.00	0.00	0.00	0.00	0.00	0.00	0.00	0.00	0.39	0.00	0.00	0.00	0.00	0.10	0.03	>0.999999999999999	3.0
P46976	Glycogenin-1 OS=Homo sapiens GN=GYG1 PE=1 SV=4	0.00	0.00	0.00	0.00	0.00	1.16	0.00	0.00	0.00	0.00	0.00	0.00	0.00	0.00	0.00	0.00	0.00	0.00	0.00	0.00	0.00	0.00	0.00	0.00	0.10	0.00	>0.999999999999999	#DIV/0!
Q13190	Syntaxin-5 OS=Homo sapiens GN=STX5 PE=1 SV=2	0.00	0.00	0.00	0.00	0.00	1.16	0.00	0.00	0.00	0.00	0.00	0.00	0.00	0.00	0.00	0.00	0.00	0.00	0.00	0.00	0.00	0.00	0.00	0.00	0.10	0.00	>0.999999999999999	#DIV/0!
P49903	Selenide, water dikinase 1 OS=Homo sapiens GN=SEPHS1 PE=1 SV=2	0.00	0.00	0.00	0.00	0.00	1.16	0.00	0.00	0.00	0.00	0.00	0.00	0.00	0.00	0.00	0.00	0.00	0.00	0.00	0.00	0.00	0.00	0.00	0.00	0.10	0.00	>0.999999999999999	#DIV/0!
Q0VDD7	Uncharacterized protein C19orf57 OS=Homo sapiens GN=C19orf57 PE=1 SV=2	0.00	0.00	0.00	0.00	0.00	0.00	0.00	0.00	0.00	0.00	0.76	0.40	0.00	0.81	0.56	0.00	0.00	0.00	0.65	0.00	0.00	1.04	0.00	0.00	0.10	0.25	>0.999999999999999	0.4
Q96MW5	Conserved oligomeric Golgi complex subunit 8 OS=Homo sapiens GN=COG8 PE=1 SV=2	0.00	1.15	0.00	0.00	0.00	0.00	0.00	0.00	0.00	0.00	0.00	0.00	0.00	0.00	0.00	0.00	0.00	0.00	0.00	0.00	0.00	0.00	0.00	0.00	0.10	0.00	>0.999999999999999	#DIV/0!
Q56NI9	N-acetyltransferase ESCO2 OS=Homo sapiens GN=ESCO2 PE=1 SV=1	0.00	1.15	0.00	0.00	0.00	0.00	0.00	0.00	0.00	0.00	0.00	0.00	0.00	0.00	0.00	0.00	0.00	0.00	0.00	0.00	0.00	0.00	0.00	0.00	0.10	0.00	>0.999999999999999	#DIV/0!
O75475	PC4 and SFRS1-interacting protein OS=Homo sapiens GN=PSIP1 PE=1 SV=1	0.00	1.15	0.00	0.00	0.00	0.00	0.00	0.00	0.00	0.00	0.00	0.00	0.00	0.00	0.00	0.00	0.00	0.00	0.00	0.00	0.00	0.00	0.00	0.00	0.10	0.00	>0.999999999999999	#DIV/0!
P27918	Properdin OS=Homo sapiens GN=CFP PE=1 SV=2	0.00	0.00	0.00	0.00	1.15	0.00	0.00	0.00	0.00	0.00	0.00	0.00	0.00	1.21	0.00	0.41	0.00	0.00	0.95	0.00	0.00	1.25	0.92	0.00	0.10	0.40	>0.999999999999999	0.2
P11597	Cholesteryl ester transfer protein OS=Homo sapiens GN=CETP PE=1 SV=2	0.00	0.00	0.00	0.00	1.15	0.00	0.00	0.00	0.00	0.00	0.00	0.00	0.00	0.00	0.00	0.00	0.00	0.00	0.95	0.00	0.00	0.00	0.00	0.00	0.10	0.08	>0.999999999999999	1.2
P01605	Ig kappa chain V-I region Lay OS=Homo sapiens PE=1 SV=1	0.00	0.00	0.00	0.00	0.00	0.00	0.00	0.00	1.15	0.00	0.00	0.00	0.00	0.00	3.22	0.00	0.00	0.00	0.00	3.76	0.00	0.00	0.00	0.00	0.10	0.58	>0.999999999999999	0.2
P35232	Prohibitin OS=Homo sapiens GN=PHB PE=1 SV=1	0.00	0.00	0.00	0.00	0.00	0.00	0.00	0.00	0.00	1.15	0.00	0.00	0.00	0.00	0.00	0.00	0.00	0.00	0.00	0.00	0.00	0.00	0.00	0.00	0.10	0.00	>0.999999999999999	#DIV/0!
Q9P0G3	Kallikrein-14 OS=Homo sapiens GN=KLK14 PE=1 SV=2	0.00	0.00	0.00	0.00	0.00	0.00	0.00	0.00	0.00	1.15	0.00	0.00	0.00	0.00	0.00	0.00	0.00	0.00	0.00	0.00	0.00	0.00	0.00	0.00	0.10	0.00	>0.999999999999999	#DIV/0!
O15400	Syntaxin-7 OS=Homo sapiens GN=STX7 PE=1 SV=4	0.00	0.00	0.00	0.00	0.00	0.00	0.00	0.00	0.00	1.15	0.00	0.00	0.00	2.22	0.00	0.00	0.00	1.69	0.00	0.00	0.00	0.00	1.69	0.00	0.10	0.47	>0.999999999999999	0.2
P08311	Cathepsin G OS=Homo sapiens GN=CTSG PE=1 SV=2	0.00	0.00	0.00	0.00	0.00	0.00	0.00	0.00	0.00	1.15	0.00	0.00	0.00	0.00	0.00	0.00	0.00	0.00	0.00	0.00	0.00	0.00	0.00	0.00	0.10	0.00	>0.999999999999999	#DIV/0!
Q5FWF4	Zinc finger Ran-binding domain-containing protein 3 OS=Homo sapiens GN=ZRANB3 PE=2	0.00	0.00	0.34	0.00	0.00	0.00	0.51	0.00	0.00	0.00	0.00	0.30	0.00	0.00	0.00	0.00	0.00	0.00	0.00	0.00	0.00	0.00	0.00	0.00	0.10	0.00	>0.999999999999999	#DIV/0!
Q0VDF9	Heat shock 70 kDa protein 14 OS=Homo sapiens GN=HSPA14 PE=1 SV=1	0.00	0.00	0.00	0.00	0.00	0.00	0.00	0.00	0.00	0.00	1.14	0.00	0.00	0.00	0.00	0.00	0.00	0.00	0.00	0.78	0.00	0.00	0.00	0.00	0.09	0.06	>0.999999999999999	1.5
Q6NUJ1	Proactivator polypeptide-like 1 OS=Homo sapiens GN=PSAPL1 PE=2 SV=2	0.00	0.00	0.00	0.00	0.00	0.00	0.00	0.00	0.00	0.00	1.14	0.00	0.00	0.00	0.00	0.00	0.00	0.00	0.00	0.00	0.00	1.25	0.00	0.00	0.09	0.10	>0.999999999999999	0.9
Q6P474	Pyridoxal-dependent decarboxylase domain-containing protein 2 OS=Homo sapiens GN=PD	0.00	0.00	0.00	0.00	0.00	0.00	0.00	0.00	0.00	0.00	1.14	0.00	0.00	0.00	0.00	0.41	0.00	0.00	0.00	0.00	0.00	0.00	0.00	0.00	0.09	0.03	>0.999999999999999	2.7
Q9H1Z4	WD repeat-containing protein 13 OS=Homo sapiens GN=WDR13 PE=1 SV=2	0.00	0.00	0.00	0.00	0.00	0.00	0.00	0.00	0.00	0.00	1.14	0.00	0.00	0.00	0.00	0.00	0.00	0.00	0.00	0.00	0.00	0.00	0.00	0.00	0.09	0.00	>0.999999999999999	#DIV/0!
O43670	Zinc finger protein 207 OS=Homo sapiens GN=ZNF207 PE=1 SV=1	0.00	0.00	0.00	0.00	0.00	0.00	0.00	0.00	0.00	0.00	1.14	0.00	0.00	0.00	0.00	0.00	0.00	0.00	0.00	0.00	0.00	0.00	0.00	0.00	0.09	0.00	>0.999999999999999	#DIV/0!
Q9H190	Syntenin-2 OS=Homo sapiens GN=SDCBP2 PE=2 SV=2	0.00	0.00	1.12	0.00	0.00	0.00	0.00	0.00	0.00	0.00	0.00	0.00	0.00	0.00	0.00	0.69	0.00	0.00	0.00	1.30	0.00	0.00	0.00	0.00	0.09	0.17	>0.999999999999999	0.6
Q9HBL8	NmrA-like family domain-containing protein 1 OS=Homo sapiens GN=NMRAL1 PE=1 SV=1	0.00	0.00	1.12	0.00	0.00	0.00	0.00	0.00	0.00	0.00	0.00	0.00	0.00	0.00	0.00	0.00	0.00	0.00	0.00	0.00	0.00	0.00	0.00	0.00	0.09	0.00	>0.999999999999999	#DIV/0!
Q8NGA8	Olfactory receptor 4F17 OS=Homo sapiens GN=OR4F17 PE=2 SV=1	0.00	0.00	1.12	0.00	0.00	0.00	0.00	0.00	0.00	0.00	0.00	0.00	0.00	0.00	0.00	0.00	0.00	0.00	0.00	0.00	0.00	0.00	0.00	0.00	0.09	0.00	>0.999999999999999	#DIV/0!
Q9UNP9	Peptidyl-prolyl cis-trans isomerase E OS=Homo sapiens GN=PPIE PE=1 SV=1	0.00	0.00	1.12	0.00	0.00	0.00	0.00	0.00	0.00	0.00	0.00	0.00	0.00	0.00	0.00	0.00	0.00	0.00	0.00	0.00	0.00	0.00	0.00	0.00	0.09	0.00	>0.999999999999999	#DIV/0!
Q9UG01	Intraflagellar transport protein 172 homolog OS=Homo sapiens GN=IFT172 PE=1 SV=2	0.73	0.00	0.00	0.00	0.00	0.00	0.00	0.00	0.00	0.00	0.38	0.00	0.00	0.00	0.00	0.00	0.00	0.00	0.00	0.00	0.00	0.00	0.00	0.00	0.09	0.00	>0.999999999999999	#DIV/0!
Q9NP61	ADP-ribosylation factor GTPase-activating protein 3 OS=Homo sapiens GN=ARFGAP3 PE=	0.00	0.00	0.00	0.00	0.00	0.87	0.00	0.00	0.24	0.00	0.00	0.00	0.00	0.00	0.00	0.00	0.00	0.00	0.00	0.00	0.00	0.00	0.00	0.00	0.09	0.00	>0.999999999999999	#DIV/0!
Q9P2R7	Succinyl-CoA ligase [ADP-forming] subunit beta, mitochondrial OS=Homo sapiens GN=SUCl	0.00	0.00	0.00	0.00	0.00	0.00	0.00	1.11	0.00	0.00	0.00	0.00	0.00	0.00	0.00	0.00	0.00	0.00	0.00	0.00	0.00	0.00	0.00	0.00	0.09	0.00	>0.999999999999999	#DIV/0!
Q9Y6D5	Brefeldin A-inhibited guanine nucleotide-exchange protein 2 OS=Homo sapiens GN=ARFGE	0.49	0.00	0.00	0.00	0.00	0.00	0.00	0.00	0.12	0.00	0.00	0.50	0.00	0.60	0.00	0.00	0.00	0.00	0.00	0.00	0.00	0.00	0.00	0.00	0.09	0.05	>0.999999999999999	1.8
P32189	Glycerol kinase OS=Homo sapiens GN=GK PE=1 SV=3	0.00	0.00	0.00	0.00	0.00	0.00	0.00	0.00	0.00	0.00	0.00	1.10	0.00	0.00	0.00	0.00	0.00	0.00	0.00	0.00	0.00	0.00	0.00	0.00	0.09	0.00	>0.999999999999999	#DIV/0!
P15927	Replication protein A 32 kDa subunit OS=Homo sapiens GN=RPA2 PE=1 SV=1	0.00	0.00	0.00	0.00	0.00	0.00	0.00	0.00	0.00	0.00	0.00	1.10	0.00	2.22	0.00	0.76	0.00	0.00	0.00	1.43	0.00	0.00	0.00	0.00	0.09	0.37	>0.999999999999999	0.2
Q00169	Phosphatidylinositol transfer protein alpha isoform OS=Homo sapiens GN=PITPNA PE=1 SV	0.00	0.00	0.00	0.00	0.00	0.00	0.00	0.00	0.00	0.00	0.00	1.10	0.00	2.01	0.00	0.00	0.00	0.00	0.00	0.00	0.00	0.00	0.00	0.00	0.09	0.17	>0.999999999999999	0.5
P11678	Eosinophil peroxidase OS=Homo sapiens GN=EPX PE=1 SV=2	0.00	0.00	0.00	0.00	0.00	0.00	0.67	0.00	0.00	0.42	0.00	0.00	0.00	0.00	0.00	0.00	0.00	0.00	0.00	0.00	0.00	0.00	0.00	0.00	0.09	0.00	>0.999999999999999	#DIV/0!
Q9Y5L0	Transportin-3 OS=Homo sapiens GN=TNPO3 PE=1 SV=3	0.00	0.00	0.34	0.00	0.00	0.44	0.00	0.00	0.00	0.00	0.00	0.30	0.00	0.00	0.00	0.00	0.00	0.00	0.00	0.00	0.00	0.00	0.00	0.00	0.09	0.00	>0.999999999999999	#DIV/0!
O76041	Nebulette OS=Homo sapiens GN=NEBL PE=2 SV=1	0.00	0.00	0.34	0.00	0.00	0.44	0.00	0.00	0.00	0.00	0.00	0.30	0.00	0.00	0.00	0.00	0.00	0.00	0.00	0.00	0.00	0.00	0.00	0.00	0.09	0.00	>0.999999999999999	#DIV/0!
Q03252	Lamin-B2 OS=Homo sapiens GN=LMNB2 PE=1 SV=3	0.00	0.00	0.00	0.00	0.00	0.00	0.00	0.00	0.00	1.04	0.00	0.00	0.00	0.00	0.00	0.00	0.00	0.00	0.00	0.00	0.00	0.00	0.00	0.00	0.09	0.00	>0.999999999999999	#DIV/0!
P27105	Erythrocyte band 7 integral membrane protein OS=Homo sapiens GN=STOM PE=1 SV=3	0.00	0.00	0.00	0.00	0.00	0.00	0.00	0.00	0.00	1.04	0.00	0.00	0.00	0.00	0.00	0.00	0.00	0.00	0.00	0.00	0.00	0.00	0.00	0.00	0.09	0.00	>0.999999999999999	#DIV/0!
Q6P3X3	Tetratricopeptide repeat protein 27 OS=Homo sapiens GN=TTC27 PE=1 SV=1	0.73	0.00	0.00	0.00	0.00	0.00	0.00	0.00	0.00	0.00	0.00	0.30	0.00	0.00	0.00	0.00	0.00	0.00	0.00	0.00	0.00	0.00	0.00	0.00	0.09	0.00	>0.999999999999999	#DIV/0!
O15027	Protein transport protein Sec16A OS=Homo sapiens GN=SEC16A PE=1 SV=3	0.24	0.23	0.11	0.00	0.19	0.15	0.00	0.00	0.00	0.00	0.00	0.10	0.00	0.00	0.00	0.00	0.00	0.00	0.00	0.13	0.00	0.00	0.00	0.00	0.09	0.01	>0.999999999999999	7.9
P11117	Lysosomal acid phosphatase OS=Homo sapiens GN=ACP2 PE=1 SV=3	0.00	0.00	0.00	0.00	0.00	1.02	0.00	0.00	0.00	0.00	0.00	0.00	0.00	0.00	0.00	0.00	0.00	0.00	0.00	0.00	0.00	0.00	0.00	0.40	0.08	0.03	>0.999999999999999	2.6
P04181	Ornithine aminotransferase, mitochondrial OS=Homo sapiens GN=OAT PE=1 SV=1	0.00	0.00	0.00	0.00	0.00	1.02	0.00	0.00	0.00	0.00	0.00	0.00	0.61	0.00	0.00	0.00	0.00	0.00	0.00	0.00	0.00	0.00	0.00	0.00	0.08	0.05	>0.999999999999999	1.7
Q4V328	GRIP1-associated protein 1 OS=Homo sapiens GN=GRIPAP1 PE=1 SV=1	0.00	0.00	0.00	0.00	0.00	1.02	0.00	0.00	0.00	0.00	0.00	0.00	0.00	0.00	0.00	0.00	0.00	0.00	0.00	0.00	0.00	0.00	0.00	0.00	0.08	0.00	>0.999999999999999	#DIV/0!
Q9BQ67	Glutamate-rich WD repeat-containing protein 1 OS=Homo sapiens GN=GRWD1 PE=1 SV=1	0.00	0.00	0.00	0.00	0.00	1.02	0.00	0.00	0.00	0.00	0.00	0.00	0.00	0.00	0.00	0.00	0.00	0.00	0.00	0.00	0.00	0.00	0.00	0.00	0.08	0.00	>0.999999999999999	#DIV/0!
Q86TX2	Acyl-coenzyme A thioesterase 1 OS=Homo sapiens GN=ACOT1 PE=1 SV=1	0.00	0.00	0.00	0.00	0.00	1.02	0.00	0.00	0.00	0.00	0.00	0.00	0.00	0.00	0.00	0.00	0.00	0.00	0.00	0.91	0.00	0.00	0.00	0.00	0.08	0.08	>0.999999999999999	1.1
Q9Y4P8	WD repeat domain phosphoinositide-interacting protein 2 OS=Homo sapiens GN=WIPI2 PE:	0.00	0.00	0.00	0.00	0.00	1.02	0.00	0.00	0.00	0.00	0.00	0.00	0.00	0.00	0.00	0.00	0.00	0.00	0.00	0.00	0.00	0.00	0.00	0.00	0.08	0.00	>0.999999999999999	#DIV/0!
P22830	Ferrochelatase, mitochondrial OS=Homo sapiens GN=FECH PE=1 SV=2	0.00	0.00	0.00	0.00	0.00	1.02	0.00	0.00	0.00	0.00	0.00	0.00	0.00	0.00	0.00	0.00	0.00	0.00	0.00	0.00	0.00	0.00	0.00	0.00	0.08	0.00	>0.999999999999999	#DIV/0!
P18754	Regulator of chromosome condensation OS=Homo sapiens GN=RCC1 PE=1 SV=1	0.00	0.00	0.00	0.00	0.00	1.02	0.00	0.00	0.00	0.00	0.00	0.00	0.00	0.00	0.00	0.00	0.00	1.07	0.00	0.91	0.00	0.00	0.00	0.00	0.08	0.17	>0.999999999999999	0.5
Q8WU90	Zinc finger CCCH domain-containing protein 15 OS=Homo sapiens GN=ZC3H15 PE=1 SV='	0.00	0.00	0.00	0.00	0.00	1.02	0.00	0.00	0.00	0.00	0.00	0.00	0.00	0.00	0.00	0.00	0.00	1.07	0.00	0.00	0.00	0.00	0.00	0.00	0.08	0.09	>0.999999999999999	0.9
Q9Y2T2	AP-3 complex subunit mu-1 OS=Homo sapiens GN=AP3M1 PE=1 SV=1	0.00	0.00	0.00	0.00	0.00	1.02	0.00	0.00	0.00	0.00	0.00	0.00	0.00	0.00	0.00	0.00	0.00	0.00	0.00	0.91	0.00	0.00	0.00	0.00	0.08	0.08	>0.999999999999999	1.1
Q15561	Transcriptional enhancer factor TEF-3 OS=Homo sapiens GN=TEAD4 PE=1 SV=3	0.00	0.00	0.00	0.00	0.00	1.02	0.00	0.00	0.00	0.00	0.00	0.00	0.00	0.00	0.00	0.00	0.00	0.00	0.00	0.00	0.00	0.00	0.00	0.00	0.08	0.00	>0.999999999999999	#DIV/0!
Q9NQ55	Suppressor of SWI4 1 homolog OS=Homo sapiens GN=PPAN PE=1 SV=1	0.00	0.00	0.00	0.00	0.00	0.00	1.01	0.00	0.00	0.00	0.00	0.00	0.00	0.00	0.00	0.00	0.00	0.00	0.00	0.00	0.00	0.00	0.00	0.00	0.08	0.00	>0.999999999999999	#DIV/0!
Q6P2I3	Fumarylacetoacetate hydrolase domain-containing protein 2B OS=Homo sapiens GN=FAHD	0.00	0.00	1.01	0.00	0.00	0.00	0.00	0.00	0.00	0.00	0.00	0.00	0.00	0.00	0.00	0.00	0.00	0.00	0.00	0.00	0.00	0.00	0.00	0.00	0.08	0.00	>0.999999999999999	#DIV/0!
Q92834	X-linked retinitis pigmentosa GTPase regulator OS=Homo sapiens GN=RPGR PE=1 SV=2	0.00	0.00	0.00	0.00	0.00	0.44	0.00	0.00	0.00	0.00	0.57	0.00	0.00	0.00	0.00	0.00	0.00	0.00	0.00	0.00	0.00	0.00	0.00	0.00	0.08	0.00	>0.999999999999999	#DIV/0!
Q92600	Cell differentiation protein RCD1 homolog OS=Homo sapiens GN=RQCD1 PE=1 SV=1	0.00	0.00	0.00	0.00	0.00	0.00	0.00	0.00	0.00	0.00	0.00	1.00	0.00	0.00	0.00	0.00	0.00	0.00	0.00	0.00	0.00	0.00	0.00	0.00	0.08	0.00	>0.999999999999999	#DIV/0!
Q14D04	Ventricular zone-expressed PH domain-containing protein homolog 1 OS=Homo sapiens GN	0.00	0.69	0.00	0.00	0.00	0.00	0.00	0.00	0.00	0.00	0.00	0.30	0.00	0.60	0.00	0.28	0.00	0.00	0.00	0.39	0.12	0.00	0.00	0.00	0.08	0.12	>0.999999999999999	0.7
O00139	Kinesin-like protein KIF2A OS=Homo sapiens GN=KIF2A PE=1 SV=3	0.00	0.00	0.00	0.00	0.00	0.58	0.00	0.00	0.00	0.00	0.00	0.40	0.00	0.00	0.00	0.00	0.00	0.00	0.00	0.00	0.00	0.00	0.00	0.00	0.08	0.00	>0.999999999999999	#DIV/0!
Q15057	Arf-GAP with coiled-coil, ANK repeat and PH domain-containing protein 2 OS=Homo sapiens	0.00	0.00	0.00	0.00	0.00	0.58	0.00	0.00	0.00	0.00	0.00	0.40	0.00	0.00	0.00	0.00	0.00	0.00	0.00	0.00	0.00	0.00	0.00	0.00	0.08	0.00	>0.999999999999999	#DIV/0!
Q9Y496	Kinesin-like protein KIF3A OS=Homo sapiens GN=KIF3A PE=1 SV=4	0.00	0.00	0.00	0.00	0.00	0.58	0.00	0.00	0.00	0.00	0.00	0.40	0.00	0.00	0.00	0.00	0.00	0.00	0.00	0.00	0.00	0.00	0.00	0.00	0.08	0.00	>0.999999999999999	#DIV/0!
Q9NR19	Acetyl-coenzyme A synthetase, cytoplasmic OS=Homo sapiens GN=ACSS2 PE=1 SV=1	0.00	0.00	0.00	0.00	0.00	0.58	0.00	0.00	0.00	0.00	0.00	0.40	0.00	0.00	0.00	0.00	0.00	0.00	0.00	0.00	0.00	0.00	0.00	0.00	0.08	0.00	>0.999999999999999	#DIV/0!
P04626	Receptor tyrosine-protein kinase erbB-2 OS=Homo sapiens GN=ERBB2 PE=1 SV=1	0.49	0.00	0.00	0.00	0.00	0.29	0.00	0.00	0.00	0.00	0.00	0.20	0.00	0.00	0.00	0.00	0.00	0.00	0.00	0.00	0.00	0.00	0.00	0.00	0.08	0.00	>0.999999999999999	#DIV/0!
O14617	AP-3 complex subunit delta-1 OS=Homo sapiens GN=AP3D1 PE=1 SV=1	0.49	0.00	0.00	0.00	0.00	0.29	0.00	0.00	0.20	0.00	0.00	0.00	0.00	0.00	0.00	0.00	0.00	0.00	0.00	0.26	0.00	0.00	0.00	0.11	0.08	0.03	>0.999999999999999	2.6
Q9C040	Tripartite motif-containing protein 2 OS=Homo sapiens GN=TRIM2 PE=1 SV=1	0.98	0.00	0.00	0.00	0.00	0.00	0.00	0.00	0.00	0.00	0.00	0.00	0.00	0.00	0.00	0.00	0.00	0.00	0.00	0.00	0.00	0.00	0.00	0.00	0.08	0.00	>0.999999999999999	#DIV/0!
Q14161	ARF GTPase-activating protein GIT2 OS=Homo sapiens GN=GIT2 PE=1 SV=2	0.98	0.00	0.00	0.00	0.00	0.00	0.00	0.00	0.00	0.00	0.00	0.00	0.00	0.00	0.00	0.00	0.00	0.00	0.00	0.00	0.00	0.00	0.00	0.00	0.08	0.00	>0.999999999999999	#DIV/0!
Q96MC2	Coiled-coil domain-containing protein 164 OS=Homo sapiens GN=CCDC164 PE=2 SV=2	0.98	0.00	0.00	0.00	0.00	0.00	0.00	0.00	0.00	0.00	0.00	0.00	0.00	0.00	0.00	0.00	0.00	0.00	0.00	0.00	0.00	0.00	0.00	0.00	0.08	0.00	>0.999999999999999	#DIV/0!
Q8N4P2	Tetratricopeptide repeat protein 30B OS=Homo sapiens GN=TTC30B PE=2 SV=2	0.98	0.00	0.00	0.00	0.00	0.00	0.00	0.00	0.00	0.00	0.00	0.00	0.00	0.00	0.00	0.00	0.00	0.00	0.00	0.00	0.00	0.00	0.00	0.00	0.08	0.00	>0.999999999999999	#DIV/0!
A8MT70	Zinc finger B-box domain-containing protein 1 OS=Homo sapiens GN=ZBBX PE=2 SV=3	0.98	0.00	0.00	0.00	0.00	0.00	0.00	0.00	0.00	0.00	0.00	0.00	0.00	0.00	0.00	0.00	0.00	0.00	0.00	0.00	0.00	0.00	0.00	0.00	0.08	0.00	>0.999999999999999	#DIV/0!
Q8ND24	RING finger protein 214 OS=Homo sapiens GN=RNF214 PE=1 SV=2	0.98	0.00	0.00	0.00	0.00	0.00	0.00	0.00	0.00	0.00	0.00	0.00	0.00	0.00	0.00	0.00	0.00	0.00	0.00	0.00	0.00	0.00	0.00	0.00	0.08	0.00	>0.999999999999999	#DIV/0!
O15066	Kinesin-like protein KIF3B OS=Homo sapiens GN=KIF3B PE=1 SV=1	0.98	0.00	0.00	0.00	0.00	0.00	0.00	0.00	0.00	0.00	0.00	0.00	0.00	0.00	0.00	0.00	0.00	0.00	0.00	0.00	0.00	0.00	0.00	0.00	0.08	0.00	>0.999999999999999	#DIV/0!
Q68DL7	Uncharacterized protein C18orf63 OS=Homo sapiens GN=C18orf63 PE=2 SV=2	0.98	0.00	0.00	0.00	0.00	0.00	0.00	0.00	0.00	0.00	0.00	0.00	0.00	0.00	0.00	0.00	0.00	0.00	0.00	0.00	0.00	0.00	0.00	0.00	0.08	0.00	>0.999999999999999	#DIV/0!
Q9UBN7	Histone deacetylase 6 OS=Homo sapiens GN=HDAC6 PE=1 SV=2	0.00	0.46	0.22	0.00	0.00	0.29	0.00	0.00	0.00	0.00	0.00	0.00	0.00	0.00	0.00	0.00	0.00	0.00	0.00	0.65	0.00	0.00	0.00	0.00	0.08	0.05	>0.999999999999999	1.5
Q02383	Semenogelin-2 OS=Homo sapiens GN=SEMG2 PE=1 SV=1	0.00	0.00	0.00	0.00	0.96	0.00	0.00	0.00	0.00	0.00	0.00	0.00	0.44	0.00	0.00	0.00	0.00	4.15	0.00	0.00	0.00	0.00	0.77	0.00	0.08	0.45	>0.999999999999999	0.2
Q12965	Unconventional myosin-Ie OS=Homo sapiens GN=MYO1E PE=1 SV=2	0.00	0.00	0.00	0.00	0.00	0.44	0.00	0.00	0.00	0.52	0.00	0.00	0.00	0.00	0.00	0.00	0.00	0.00	0.00	0.00	0.00	0.00	0.00	0.00	0.08	0.00	>0.999999999999999	#DIV/0!
Q8IVW4	Cyclin-dependent kinase-like 3 OS=Homo sapiens GN=CDKL3 PE=2 SV=1	0.00	0.00	0.00	0.76	0.00	0.00	0.00	0.00	0.20	0.00	0.00	0.00	0.00	0.00	0.56	0.34	0.00	0.00	0.00	0.00	0.00	1.04	0.00	0.00	0.08	0.16	>0.999999999999999	0.5
Q02413	Desmoglein-1 OS=Homo sapiens GN=DSG1 PE=1 SV=2	0.00	0.00	0.00	0.00	0.00	0.00	0.95	0.00	0.00	0.00	0.00	0.00	0.00	0.00	0.00	0.21	0.00	0.00	0.00	0.00	0.00	0.62	0.00	0.00	0.08	0.07	>0.999999999999999	1.1
P48723	Heat shock 70 kDa protein 13 OS=Homo sapiens GN=HSPA13 PE=1 SV=1	0.00	0.00	0.00	0.00	0.00	0.00	0.95	0.00	0.00	0.00	0.00	0.00	0.00	0.00	0.00	0.00	0.00	0.00	0.00	0.00	0.00	0.00	0.00	0.00	0.08	0.00	>0.999999999999999	#DIV/0!
Q13217	DnaJ homolog subfamily C member 3 OS=Homo sapiens GN=DNAJC3 PE=1 SV=1	0.00	0.00	0.00	0.00	0.00	0.00	0.95	0.00	0.00	0.00	0.00	0.00	0.00	0.00	0.67	0.00	0.00	0.00	0.00	0.00	0.00	0.00	0.00	0.00	0.08	0.06	>0.999999999999999	1.4
P55084	Trifunctional enzyme subunit beta, mitochondrial OS=Homo sapiens GN=HADHB PE=1 SV=	0.00	0.00	0.00	0.00	0.00	0.00	0.95	0.00	0.00	0.00	0.00	0.00	0.00	0.00	0.00	0.00	0.00	0.00	0.00	0.00	0.00	0.00	0.00	0.00	0.08	0.00	>0.999999999999999	#DIV/0!
P19438	Tumor necrosis factor receptor superfamily member 1A OS=Homo sapiens GN=TNFRSF1A	0.00	0.00	0.00	0.00	0.00	0.00	0.95	0.00	0.00	0.00	0.00	0.00	0.00	0.00	0.00	0.00	0.00	0.00	0.00	0.00	0.00	0.00	0.00	0.00	0.08	0.00	>0.999999999999999	#DIV/0!
Q9BXM9	FSD1-like protein OS=Homo sapiens GN=FSD1L PE=2 SV=2	0.00	0.00	0.00	0.00	0.00	0.00	0.00	0.00	0.00	0.00	0.95	0.00	0.00	0.00	0.00	0.00	0.00	0.00	0.00	0.00	0.00	0.00	0.00	0.00	0.08	0.00	>0.999999999999999	#DIV/0!
Q13573	SNW domain-containing protein 1 OS=Homo sapiens GN=SNW1 PE=1 SV=1	0.00	0.00	0.00	0.00	0.00	0.00	0.00	0.00	0.00	0.00	0.95	0.00	0.00	0.00	0.00	0.00	0.00	0.00	0.00	0.00	0.00	0.00	0.00	0.00	0.08	0.00	>0.999999999999999	#DIV/0!
Q9P2L0	WD repeat-containing protein 35 OS=Homo sapiens GN=WDR35 PE=1 SV=3	0.00	0.00	0.00	0.00	0.00	0.73	0.00	0.00	0.00	0.00	0.00	0.20	0.00	0.00	0.00	0.00	0.00	0.00	0.00	0.00	0.00	0.00	0.00	0.00	0.08	0.00	>0.999999999999999	#DIV/0!
Q06124	Tyrosine-protein phosphatase non-receptor type 11 OS=Homo sapiens GN=PTPN11 PE=1 :	0.00	0.00	0.00	0.00	0.00	0.73	0.00	0.00	0.20	0.00	0.00	0.00	0.00	1.01	0.00	0.00	0.00	0.00	0.00	0.00	0.00	0.00	0.00	0.00	0.08	0.08	>0.999999999999999	0.9
P57772	Selenocysteine-specific elongation factor OS=Homo sapiens GN=EEFSEC PE=1 SV=4	0.00	0.00	0.00	0.00	0.00	0.73	0.00	0.00	0.20	0.00	0.00	0.00	0.00	0.00	0.00	0.00	0.00	0.00	0.00	0.00	0.00	0.00	0.00	0.00	0.08	0.00	>0.999999999999999	#DIV/0!
Q16891	Mitochondrial inner membrane protein OS=Homo sapiens GN=IMMT PE=1 SV=1	0.00	0.92	0.00	0.00	0.00	0.00	0.00	0.00	0.00	0.00	0.00	0.00	0.00	0.00	0.00	0.00	0.00	0.00	0.00	0.00	0.00	0.00	0.00	0.00	0.08	0.00	>0.999999999999999	#DIV/0!
Q8IYI6	Exocyst complex component 8 OS=Homo sapiens GN=EXOC8 PE=1 SV=2	0.00	0.92	0.00	0.00	0.00	0.00	0.00	0.00	0.00	0.00	0.00	0.00	0.00	0.00	0.00	0.00	0.00	0.00	0.00	0.00	0.00	0.00	0.00	0.00	0.08	0.00	>0.999999999999999	#DIV/0!
Q9H9A5	CCR4-NOT transcription complex subunit 10 OS=Homo sapiens GN=CNOT10 PE=1 SV=1	0.00	0.92	0.00	0.00	0.00	0.00	0.00	0.00	0.00	0.00	0.00	0.00	0.00	0.00	0.00	0.00	0.00	0.00	0.00	0.00	0.00	0.00	0.00	0.00	0.08	0.00	>0.999999999999999	#DIV/0!
Q7Z4V5	Hepatoma-derived growth factor-related protein 2 OS=Homo sapiens GN=HDGFRP2 PE=1 :	0.00	0.92	0.00	0.00	0.00	0.00	0.00	0.00	0.00	0.00	0.00	0.00	0.35	0.81	0.00	0.00	0.00	0.00	0.00	0.00	0.00	0.00	0.00	0.00	0.08	0.10	>0.999999999999999	0.8
P83436	Conserved oligomeric Golgi complex subunit 7 OS=Homo sapiens GN=COG7 PE=1 SV=1	0.00	0.92	0.00	0.00	0.00	0.00	0.00	0.00	0.00	0.00	0.00	0.00	0.00	0.00	0.00	0.00	0.00	0.00	0.00	0.00	0.00	0.00	0.00	0.23	0.08	0.02	>0.999999999999999	4.1
Q7Z589	Protein EMSY OS=Homo sapiens GN=EMSY PE=1 SV=2	0.00	0.00	0.22	0.00	0.00	0.00	0.32	0.00	0.00	0.00	0.38	0.00	0.18	0.40	0.00	0.00	0.38	0.31	0.32	0.26	0.00	0.42	0.31	0.00	0.08	0.21	>0.999999999999999	0.4
O14964	Hepatocyte growth factor-regulated tyrosine kinase substrate OS=Homo sapiens GN=HGS I	0.00	0.00	0.00	0.00	0.00	0.00	0.00	0.00	0.16	0.00	0.76	0.00	0.00	1.61	0.00	0.28	0.00	0.00	0.00	0.00	0.00	0.00	0.00	0.23	0.08	0.18	>0.999999999999999	0.4
P08254	Stromelysin-1 OS=Homo sapiens GN=MMP3 PE=1 SV=2	0.00	0.00	0.67	0.00	0.00	0.00	0.00	0.00	0.24	0.00	0.00	0.00	0.00	0.00	0.00	0.00	0.00	0.00	0.00	0.78	0.00	0.00	0.00	0.00	0.08	0.06	>0.999999999999999	1.2
Q13451	Peptidyl-prolyl cis-trans isomerase FKBP5 OS=Homo sapiens GN=FKBP5 PE=1 SV=2	0.00	0.00	0.00	0.91	0.00	0.00	0.00	0.00	0.00	0.00	0.00	0.00	0.00	0.00	0.00	0.00	0.00	0.00	0.00	0.00	0.00	0.00	0.00	0.00	0.08	0.00	>0.999999999999999	#DIV/0!
Q02487	Desmocollin-2 OS=Homo sapiens GN=DSC2 PE=1 SV=1	0.00	0.00	0.34	0.00	0.00	0.00	0.00	0.00	0.00	0.00	0.57	0.00	0.00	0.00	0.00	0.00	0.00	0.00	0.00	0.00	0.00	0.00	0.00	0.00	0.08	0.00	>0.999999999999999	#DIV/0!
Q9Y5K3	Choline-phosphate cytidylyltransferase B OS=Homo sapiens GN=PCYT1B PE=1 SV=1	0.00	0.00	0.90	0.00	0.00	0.00	0.00	0.00	0.00	0.00	0.00	0.00	0.00	0.00	0.00	0.00	0.00	0.00	0.00	0.00	0.00	0.00	0.00	0.00	0.07	0.00	>0.999999999999999	#DIV/0!
Q9NZQ9	Tropomodulin-4 OS=Homo sapiens GN=TMOD4 PE=2 SV=1	0.00	0.00	0.90	0.00	0.00	0.00	0.00	0.00	0.00	0.00	0.00	0.00	0.00	0.00	0.00	0.00	0.00	0.00	0.00	0.00	0.00	0.00	0.00	0.00	0.07	0.00	>0.999999999999999	#DIV/0!
Q9Y2M2	Uncharacterized protein C3orf32 OS=Homo sapiens GN=C3orf32 PE=2 SV=1	0.00	0.00	0.90	0.00	0.00	0.00	0.00	0.00	0.00	0.00	0.00	0.00	0.00	0.00	0.00	0.00	0.00	0.00	0.00	0.00	0.00	0.00	0.00	0.00	0.07	0.00	>0.999999999999999	#DIV/0!
P15289	Arylsulfatase A OS=Homo sapiens GN=ARSA PE=1 SV=3	0.00	0.00	0.00	0.00	0.00	0.87	0.00	0.00	0.00	0.00	0.00	0.00	0.00	0.00	0.00	0.00	0.00	0.00	0.00	0.00	0.00	0.00	0.00	0.00	0.07	0.00	>0.999999999999999	#DIV/0!
Q96MA6	Adenylate kinase 8 OS=Homo sapiens GN=AK8 PE=1 SV=1	0.00	0.00	0.00	0.00	0.00	0.87	0.00	0.00	0.00	0.00	0.00	0.00	0.00	0.00	0.00	0.00	0.00	0.00	0.00	0.00	0.00	0.00	0.00	0.00	0.07	0.00	>0.999999999999999	#DIV/0!
Q9UN86	Ras GTPase-activating protein-binding protein 2 OS=Homo sapiens GN=G3BP2 PE=1 SV=2	0.00	0.00	0.00	0.00	0.00	0.87	0.00	0.00	0.00	0.00	0.00	0.00	0.00	0.00	0.00	0.00	0.00	0.00	0.00	0.00	0.00	0.00	0.00	0.00	0.07	0.00	>0.999999999999999	#DIV/0!
Q9H074	Polyadenylate-binding protein-interacting protein 1 OS=Homo sapiens GN=PAIP1 PE=1 SV=	0.00	0.00	0.00	0.00	0.00	0.87	0.00	0.00	0.00	0.00	0.00	0.00	0.00	0.00	0.00	0.00	0.00	0.00	0.00	0.00	0.00	0.00	0.00	0.00	0.07	0.00	>0.999999999999999	#DIV/0!
Q9HD40	O-phosphoseryl-tRNA(Sec) selenium transferase OS=Homo sapiens GN=SEPSECS PE=1 S	0.00	0.00	0.00	0.00	0.00	0.87	0.00	0.00	0.00	0.00	0.00	0.00	0.00	0.00	0.00	0.00	0.00	0.00	0.00	0.00	0.00	0.00	0.00	0.00	0.07	0.00	>0.999999999999999	#DIV/0!
Q96NY8	Poliovirus receptor-related protein 4 OS=Homo sapiens GN=PVRL4 PE=1 SV=1	0.00	0.00	0.00	0.00	0.00	0.87	0.00	0.00	0.00	0.00	0.00	0.00	0.00	0.00	0.00	0.41	0.00	0.00	0.00	0.00	0.00	0.00	0.00	0.00	0.07	0.03	>0.999999999999999	2.1
O60832	H/ACA ribonucleoprotein complex subunit 4 OS=Homo sapiens GN=DKC1 PE=1 SV=3	0.00	0.00	0.00	0.00	0.00	0.87	0.00	0.00	0.00	0.00	0.00	0.00	0.00	0.00	0.00	0.00	0.00	0.00	0.00	0.00	0.00	0.00	0.00	0.00	0.07	0.00	>0.999999999999999	#DIV/0!
Q8IV08	Phospholipase D3 OS=Homo sapiens GN=PLD3 PE=1 SV=1	0.00	0.00	0.00	0.00	0.00	0.87	0.00	0.00	0.00	0.00	0.00	0.00	0.00	0.00	0.00	0.00	0.00	0.00	0.00	0.00	0.00	0.00	0.00	0.00	0.07	0.00	>0.999999999999999	#DIV/0!
Q86SX3	Uncharacterized protein C14orf80 OS=Homo sapiens GN=C14orf80 PE=2 SV=2	0.00	0.00	0.00	0.00	0.00	0.87	0.00	0.00	0.00	0.00	0.00	0.00	0.00	0.00	0.00	0.41	0.00	0.00	0.00	0.78	0.00	0.00	0.00	0.00	0.07	0.10	>0.999999999999999	0.7
P16118	6-phosphofructo-2-kinase/fructose-2,6-bisphosphatase 1 OS=Homo sapiens GN=PFKFB1 F	0.00	0.00	0.00	0.00	0.00	0.87	0.00	0.00	0.00	0.00	0.00	0.00	0.00	0.00	0.00	0.00	0.00	0.00	0.00	0.00	0.00	0.00	0.00	0.00	0.07	0.00	>0.999999999999999	#DIV/0!
O75886	Signal transducing adapter molecule 2 OS=Homo sapiens GN=STAM2 PE=1 SV=1	0.00	0.00	0.00	0.00	0.00	0.87	0.00	0.00	0.00	0.00	0.00	0.00	0.00	0.00	0.00	0.00	0.00	0.00	0.00	0.00	0.00	0.00	0.00	0.00	0.07	0.00	>0.999999999999999	#DIV/0!
Q16647	Prostacyclin synthase OS=Homo sapiens GN=PTGIS PE=1 SV=1	0.00	0.00	0.00	0.00	0.00	0.87	0.00	0.00	0.00	0.00	0.00	0.00	0.00	0.00	0.00	0.00	0.00	0.00	0.00	0.00	0.00	0.00	0.00	0.00	0.07	0.00	>0.999999999999999	#DIV/0!
Q5T124	UBX domain-containing protein 11 OS=Homo sapiens GN=UBXN11 PE=2 SV=2	0.00	0.00	0.00	0.00	0.00	0.87	0.00	0.00	0.00	0.00	0.00	0.00	0.00	0.00	0.00	0.00	0.00	0.00	0.00	0.00	0.00	0.00	0.00	0.00	0.07	0.00	>0.999999999999999	#DIV/0!
Q9UPN7	Serine/threonine-protein phosphatase 6 regulatory subunit 1 OS=Homo sapiens GN=PPP6R	0.00	0.00	0.00	0.00	0.00	0.00	0.00	0.00	0.00	0.00	0.57	0.30	0.00	0.00	0.00	0.00	0.00	0.00	0.00	0.00	0.00	0.00	0.00	0.00	0.07	0.00	>0.999999999999999	#DIV/0!
Q70J99	Protein unc-13 homolog D OS=Homo sapiens GN=UNC13D PE=1 SV=1	0.00	0.00	0.00	0.00	0.00	0.00	0.00	0.00	0.00	0.83	0.00	0.00	0.00	0.00	0.00	0.00	0.00	0.00	0.00	0.00	0.00	0.00	0.00	0.00	0.07	0.00	>0.999999999999999	#DIV/0!
P05107	Integrin beta-2 OS=Homo sapiens GN=ITGB2 PE=1 SV=2	0.00	0.00	0.00	0.00	0.00	0.00	0.00	0.00	0.00	0.83	0.00	0.00	0.00	0.00	0.00	0.00	0.00	0.00	0.00	0.00	0.00	0.00	0.00	0.00	0.07	0.00	>0.999999999999999	#DIV/0!
Q13835	Plakophilin-1 OS=Homo sapiens GN=PKP1 PE=1 SV=2	0.00	0.00	0.00	0.00	0.00	0.00	0.00	0.00	0.00	0.83	0.00	0.00	0.00	0.00	0.00	0.00	0.00	0.00	0.00	0.00	0.00	0.00	0.00	0.00	0.07	0.00	>0.999999999999999	#DIV/0!
P61421	V-type proton ATPase subunit d 1 OS=Homo sapiens GN=ATP6V0D1 PE=1 SV=1	0.00	0.00	0.00	0.00	0.00	0.00	0.00	0.00	0.00	0.83	0.00	0.00	0.00	0.00	0.00	0.00	0.00	0.00	0.00	0.00	0.00	0.00	0.00	0.00	0.07	0.00	>0.999999999999999	#DIV/0!
Q9H2M3	Betaine--homocysteine S-methyltransferase 2 OS=Homo sapiens GN=BHMT2 PE=1 SV=1	0.00	0.00	0.00	0.00	0.00	0.00	0.00	0.00	0.00	0.83	0.00	0.00	0.00	0.00	0.00	0.00	0.00	0.00	0.00	0.00	0.00	0.00	0.00	0.00	0.07	0.00	>0.999999999999999	#DIV/0!
C9JRZ8	Aldo-keto reductase family 1 member B15 OS=Homo sapiens GN=AKR1B15 PE=2 SV=1	0.00	0.00	0.00	0.00	0.00	0.00	0.00	0.00	0.00	0.83	0.00	0.00	0.00	0.00	0.00	0.00	0.00	0.00	0.00	0.00	0.00	0.00	0.00	0.00	0.07	0.00	>0.999999999999999	#DIV/0!
P36222	Chitinase-3-like protein 1 OS=Homo sapiens GN=CHI3L1 PE=1 SV=2	0.00	0.00	0.00	0.00	0.00	0.00	0.00	0.00	0.00	0.83	0.00	0.00	0.00	0.00	0.00	0.00	0.00	0.00	0.00	0.00	0.00	1.66	0.00	0.00	0.07	0.14	>0.999999999999999	0.5
P28325	Cystatin-D OS=Homo sapiens GN=CST5 PE=1 SV=1	0.00	0.00	0.00	0.00	0.00	0.00	0.00	0.00	0.83	0.00	0.00	0.00	0.00	0.00	0.00	0.00	0.00	21.30	0.00	0.00	0.00	0.00	0.00	1.19	0.07	1.87	>0.999999999999999	0.0
O75643	U5 small nuclear ribonucleoprotein 200 kDa helicase OS=Homo sapiens GN=SNRNP200 PE	0.00	0.00	0.11	0.00	0.00	0.15	0.00	0.00	0.00	0.00	0.57	0.00	0.00	0.00	0.00	0.00	0.00	0.00	0.00	0.00	0.00	0.00	0.00	0.00	0.07	0.00	>0.999999999999999	#DIV/0!
Q96G28	Coiled-coil domain-containing protein 104 OS=Homo sapiens GN=CCDC104 PE=1 SV=2	0.00	0.00	0.00	0.00	0.00	0.00	0.00	0.00	0.00	0.00	0.00	0.80	0.00	0.00	0.00	0.00	0.00	0.00	0.00	0.00	0.00	0.00	0.00	0.00	0.07	0.00	>0.999999999999999	#DIV/0!
Q9HCN4	GPN-loop GTPase 1 OS=Homo sapiens GN=GPN1 PE=1 SV=1	0.00	0.00	0.00	0.00	0.00	0.00	0.00	0.00	0.00	0.00	0.00	0.80	0.00	0.00	0.00	0.00	0.00	0.00	0.00	0.00	0.00	0.00	0.00	0.00	0.07	0.00	>0.999999999999999	#DIV/0!
Q13247	Serine/arginine-rich splicing factor 6 OS=Homo sapiens GN=SRSF6 PE=1 SV=2	0.00	0.00	0.00	0.00	0.00	0.00	0.00	0.00	0.00	0.00	0.00	0.80	0.00	0.00	0.00	0.00	0.00	0.00	0.00	0.00	0.00	0.00	0.00	0.00	0.07	0.00	>0.999999999999999	#DIV/0!
Q53EV4	Leucine-rich repeat-containing protein 23 OS=Homo sapiens GN=LRRC23 PE=2 SV=2	0.00	0.00	0.00	0.00	0.00	0.00	0.00	0.00	0.00	0.00	0.00	0.80	0.00	0.00	0.00	0.00	0.00	0.00	0.00	0.00	0.00	0.00	0.00	0.00	0.07	0.00	>0.999999999999999	#DIV/0!
P55039	Developmentally-regulated GTP-binding protein 2 OS=Homo sapiens GN=DRG2 PE=1 SV=1	0.00	0.00	0.00	0.00	0.00	0.00	0.00	0.00	0.00	0.00	0.00	0.80	0.00	0.00	0.00	0.00	0.00	0.00	0.00	0.00	0.00	0.00	0.00	0.00	0.07	0.00	>0.999999999999999	#DIV/0!
Q9H0C2	ADP/ATP translocase 4 OS=Homo sapiens GN=SLC25A31 PE=1 SV=1	0.00	0.00	0.00	0.00	0.00	0.00	0.00	0.00	0.79	0.00	0.00	0.00	0.00	0.00	1.00	0.00	0.00	0.00	0.00	0.00	0.00	0.00	0.00	0.00	0.07	0.08	>0.999999999999999	0.8
P53680	AP-2 complex subunit sigma OS=Homo sapiens GN=AP2S1 PE=1 SV=2	0.00	0.00	0.00	0.00	0.00	0.00	0.00	0.00	0.79	0.00	0.00	0.00	0.00	0.00	0.00	0.00	0.00	0.00	0.00	0.00	0.00	0.00	0.00	0.00	0.07	0.00	>0.999999999999999	#DIV/0!
Q96PC3	AP-1 complex subunit sigma-3 OS=Homo sapiens GN=AP1S3 PE=2 SV=1	0.00	0.00	0.00	0.00	0.00	0.00	0.00	0.00	0.79	0.00	0.00	0.00	0.00	0.00	0.00	0.00	0.00	0.00	0.00	2.33	0.00	0.00	0.00	0.00	0.07	0.19	>0.999999999999999	0.3
Q86X29	Lipolysis-stimulated lipoprotein receptor OS=Homo sapiens GN=LSR PE=1 SV=4	0.00	0.00	0.00	0.00	0.00	0.00	0.00	0.79	0.00	0.00	0.00	0.00	0.00	0.00	0.00	0.00	0.00	0.00	0.00	0.00	0.15	0.00	0.00	0.00	0.07	0.01	>0.999999999999999	5.1
O95831	Apoptosis-inducing factor 1, mitochondrial OS=Homo sapiens GN=AIFM1 PE=1 SV=1	0.00	0.00	0.00	0.00	0.00	0.00	0.00	0.79	0.00	0.00	0.00	0.00	0.00	0.00	0.00	0.00	0.00	0.00	0.00	0.00	0.00	0.00	0.00	0.00	0.07	0.00	>0.999999999999999	#DIV/0!
P04844	Dolichyl-diphosphooligosaccharide--protein glycosyltransferase subunit 2 OS=Homo sapiens	0.00	0.00	0.00	0.00	0.00	0.00	0.00	0.79	0.00	0.00	0.00	0.00	0.00	0.00	0.00	0.00	0.00	0.00	0.00	0.00	0.00	0.00	0.00	0.00	0.07	0.00	>0.999999999999999	#DIV/0!
O00748	Cocaine esterase OS=Homo sapiens GN=CES2 PE=1 SV=1	0.00	0.00	0.00	0.00	0.00	0.00	0.00	0.79	0.00	0.00	0.00	0.00	0.44	0.00	0.00	0.00	0.00	0.00	0.00	0.00	0.00	0.00	0.00	0.00	0.07	0.04	>0.999999999999999	1.8
P55259	Pancreatic secretory granule membrane major glycoprotein GP2 OS=Homo sapiens GN=GF	0.00	0.00	0.00	0.00	0.00	0.00	0.00	0.79	0.00	0.00	0.00	0.00	0.00	0.00	0.00	0.00	0.00	0.00	0.00	0.00	0.00	0.00	0.00	0.00	0.07	0.00	>0.999999999999999	#DIV/0!
O94826	Mitochondrial import receptor subunit TOM70 OS=Homo sapiens GN=TOMM70A PE=1 SV=	0.00	0.00	0.00	0.00	0.00	0.00	0.00	0.79	0.00	0.00	0.00	0.00	0.00	0.00	0.00	0.00	0.00	0.00	0.00	0.00	0.00	0.00	0.00	0.00	0.07	0.00	>0.999999999999999	#DIV/0!
Q15642	Cdc42-interacting protein 4 OS=Homo sapiens GN=TRIP10 PE=1 SV=3	0.00	0.00	0.00	0.00	0.00	0.00	0.00	0.79	0.00	0.00	0.00	0.00	0.00	0.00	0.00	0.00	0.00	0.77	0.00	0.00	0.00	0.00	0.00	0.00	0.07	0.06	>0.999999999999999	1.0
Q2M329	Coiled-coil domain-containing protein 96 OS=Homo sapiens GN=CCDC96 PE=2 SV=2	0.00	0.00	0.00	0.00	0.00	0.00	0.00	0.79	0.00	0.00	0.00	0.00	0.00	0.00	0.00	0.00	0.00	0.00	0.00	0.00	0.00	0.00	0.00	0.00	0.07	0.00	>0.999999999999999	#DIV/0!
Q96JH7	Deubiquitinating protein VCIP135 OS=Homo sapiens GN=VCPIP1 PE=1 SV=2	0.00	0.00	0.00	0.00	0.00	0.29	0.00	0.00	0.00	0.00	0.00	0.50	0.00	0.00	0.00	0.00	0.00	0.00	0.00	0.00	0.00	0.00	0.00	0.11	0.07	0.01	>0.999999999999999	7.0
O75116	Rho-associated protein kinase 2 OS=Homo sapiens GN=ROCK2 PE=1 SV=4	0.49	0.00	0.00	0.00	0.00	0.29	0.00	0.00	0.00	0.00	0.00	0.00	0.00	0.40	0.00	0.00	0.00	0.00	0.00	0.26	0.00	0.00	0.00	0.00	0.06	0.06	>0.999999999999999	1.2
Q8NDA8	HEAT repeat-containing protein 7A OS=Homo sapiens GN=HEATR7A PE=1 SV=3	0.49	0.00	0.00	0.00	0.00	0.29	0.00	0.00	0.00	0.00	0.00	0.00	0.00	0.00	0.00	0.00	0.00	0.00	0.00	0.00	0.00	0.00	0.00	0.00	0.06	0.00	>0.999999999999999	#DIV/0!
Q15075	Early endosome antigen 1 OS=Homo sapiens GN=EEA1 PE=1 SV=2	0.49	0.00	0.00	0.00	0.00	0.29	0.00	0.00	0.00	0.00	0.00	0.00	0.00	0.00	0.00	0.00	0.00	0.31	0.00	0.26	0.00	0.00	0.00	0.00	0.06	0.05	>0.999999999999999	1.4
Q08378	Golgin subfamily A member 3 OS=Homo sapiens GN=GOLGA3 PE=1 SV=2	0.49	0.00	0.00	0.00	0.00	0.29	0.00	0.00	0.00	0.00	0.00	0.00	0.00	0.00	0.00	0.00	0.00	0.00	0.00	0.00	0.00	0.00	0.00	0.00	0.06	0.00	>0.999999999999999	#DIV/0!
Q2M389	WASH complex subunit 7 OS=Homo sapiens GN=KIAA1033 PE=1 SV=2	0.00	0.00	0.00	0.00	0.00	0.00	0.00	0.00	0.08	0.00	0.00	0.70	0.00	0.00	0.00	0.00	0.00	0.00	0.00	0.00	0.00	0.00	0.00	0.11	0.06	0.01	>0.999999999999999	6.8
O60502	Bifunctional protein NCOAT OS=Homo sapiens GN=MGEA5 PE=1 SV=2	0.00	0.00	0.34	0.00	0.00	0.44	0.00	0.00	0.00	0.00	0.00	0.00	0.00	0.00	0.00	0.00	0.00	0.00	0.00	0.00	0.00	0.00	0.00	0.00	0.06	0.00	>0.999999999999999	#DIV/0!
O94832	Unconventional myosin-Id OS=Homo sapiens GN=MYO1D PE=1 SV=2	0.00	0.00	0.34	0.00	0.00	0.44	0.00	0.00	0.00	0.00	0.00	0.00	0.00	0.00	0.00	0.00	0.00	0.00	0.00	0.39	0.00	0.00	0.00	0.00	0.06	0.03	>0.999999999999999	2.0
P80108	Phosphatidylinositol-glycan-specific phospholipase D OS=Homo sapiens GN=GPLD1 PE=1 :	0.00	0.00	0.00	0.00	0.77	0.00	0.00	0.00	0.00	0.00	0.00	0.00	0.00	0.00	0.00	0.00	0.00	0.00	0.00	0.00	0.00	0.00	0.00	0.00	0.06	0.00	>0.999999999999999	#DIV/0!
Q15477	Helicase SKI2W OS=Homo sapiens GN=SKIV2L PE=1 SV=3	0.00	0.00	0.56	0.00	0.00	0.00	0.00	0.00	0.00	0.00	0.00	0.20	0.00	0.00	0.00	0.00	0.00	0.00	0.00	0.00	0.00	0.00	0.00	0.11	0.06	0.01	>0.999999999999999	6.7
P49959	Double-strand break repair protein MRE11A OS=Homo sapiens GN=MRE11A PE=1 SV=3	0.00	0.00	0.00	0.00	0.00	0.00	0.00	0.00	0.00	0.00	0.76	0.00	0.00	0.00	0.00	0.00	0.00	0.00	0.00	0.00	0.00	0.00	0.00	0.00	0.06	0.00	>0.999999999999999	#DIV/0!
Q92845	Kinesin-associated protein 3 OS=Homo sapiens GN=KIFAP3 PE=1 SV=2	0.00	0.00	0.00	0.00	0.00	0.00	0.00	0.00	0.00	0.00	0.76	0.00	0.00	0.00	0.00	0.00	0.00	0.00	0.00	0.00	0.00	0.00	0.00	0.00	0.06	0.00	>0.999999999999999	#DIV/0!
O43290	U4/U6.U5 tri-snRNP-associated protein 1 OS=Homo sapiens GN=SART1 PE=1 SV=1	0.00	0.00	0.00	0.00	0.00	0.00	0.00	0.00	0.00	0.00	0.76	0.00	0.00	0.00	0.00	0.00	0.00	0.00	0.00	0.00	0.00	0.00	0.00	0.00	0.06	0.00	>0.999999999999999	#DIV/0!
P08253	72 kDa type IV collagenase OS=Homo sapiens GN=MMP2 PE=1 SV=2	0.00	0.00	0.00	0.00	0.00	0.00	0.00	0.00	0.00	0.00	0.76	0.00	0.00	0.00	0.00	0.00	0.00	0.00	0.00	0.00	0.00	0.00	0.00	0.00	0.06	0.00	>0.999999999999999	#DIV/0!
Q5JVL4	EF-hand domain-containing protein 1 OS=Homo sapiens GN=EFHC1 PE=1 SV=1	0.00	0.00	0.00	0.00	0.00	0.00	0.00	0.00	0.00	0.00	0.76	0.00	0.00	0.00	0.00	0.00	0.00	0.00	0.00	0.00	0.00	0.00	0.00	0.00	0.06	0.00	>0.999999999999999	#DIV/0!
O95477	ATP-binding cassette sub-family A member 1 OS=Homo sapiens GN=ABCA1 PE=1 SV=3	0.00	0.00	0.00	0.00	0.00	0.00	0.17	0.48	0.00	0.10	0.00	0.00	0.00	0.00	0.00	0.00	0.00	0.00	0.16	0.00	0.00	0.00	0.00	0.00	0.06	0.01	>0.999999999999999	4.7
P54802	Alpha-N-acetylglucosaminidase OS=Homo sapiens GN=NAGLU PE=1 SV=2	0.00	0.00	0.00	0.00	0.00	0.58	0.00	0.00	0.16	0.00	0.00	0.00	0.35	0.81	0.00	0.00	0.00	0.61	0.00	0.00	0.00	0.00	0.00	0.23	0.06	0.17	>0.999999999999999	0.4
Q0JRZ9	FCH domain only protein 2 OS=Homo sapiens GN=FCHO2 PE=1 SV=2	0.00	0.00	0.00	0.00	0.00	0.58	0.00	0.00	0.16	0.00	0.00	0.00	0.00	0.00	0.00	0.00	0.00	0.00	0.00	0.00	0.00	0.00	0.00	0.00	0.06	0.00	>0.999999999999999	#DIV/0!
Q9UQ88	Cyclin-dependent kinase 11A OS=Homo sapiens GN=CDK11A PE=1 SV=4	0.00	0.00	0.00	0.00	0.00	0.58	0.00	0.00	0.16	0.00	0.00	0.00	0.00	0.81	0.00	0.00	0.00	0.00	0.00	0.00	0.00	0.00	0.00	0.23	0.06	0.09	>0.999999999999999	0.7
Q9H269	Vacuolar protein sorting-associated protein 16 homolog OS=Homo sapiens GN=VPS16 PE=	0.00	0.00	0.00	0.00	0.00	0.44	0.00	0.00	0.00	0.00	0.00	0.30	0.00	0.00	0.00	0.00	0.00	0.00	0.00	0.00	0.00	0.00	0.00	0.00	0.06	0.00	>0.999999999999999	#DIV/0!
P09874	Poly [ADP-ribose] polymerase 1 OS=Homo sapiens GN=PARP1 PE=1 SV=4	0.73	0.00	0.00	0.00	0.00	0.00	0.00	0.00	0.00	0.00	0.00	0.00	0.00	0.00	0.00	0.00	0.00	0.46	0.00	0.00	0.00	0.00	0.00	0.00	0.06	0.04	>0.999999999999999	1.6
O15084	Serine/threonine-protein phosphatase 6 regulatory ankyrin repeat subunit A OS=Homo sapie	0.73	0.00	0.00	0.00	0.00	0.00	0.00	0.00	0.00	0.00	0.00	0.00	0.00	0.00	0.00	0.00	0.00	0.00	0.00	0.00	0.00	0.00	0.00	0.00	0.06	0.00	>0.999999999999999	#DIV/0!
Q6IA86	Elongator complex protein 2 OS=Homo sapiens GN=ELP2 PE=1 SV=2	0.73	0.00	0.00	0.00	0.00	0.00	0.00	0.00	0.00	0.00	0.00	0.00	0.00	0.00	0.00	0.00	0.00	0.00	0.00	0.00	0.00	0.00	0.00	0.00	0.06	0.00	>0.999999999999999	#DIV/0!
Q9H3U1	Protein unc-45 homolog A OS=Homo sapiens GN=UNC45A PE=1 SV=1	0.73	0.00	0.00	0.00	0.00	0.00	0.00	0.00	0.00	0.00	0.00	0.00	0.26	0.00	0.00	0.00	0.00	0.00	0.00	0.78	0.00	0.00	0.00	0.00	0.06	0.09	>0.999999999999999	0.7
O00160	Unconventional myosin-If OS=Homo sapiens GN=MYO1F PE=1 SV=3	0.73	0.00	0.00	0.00	0.00	0.00	0.00	0.00	0.00	0.00	0.00	0.00	0.00	0.00	0.00	0.00	0.00	0.00	0.00	0.00	0.00	2.29	0.00	0.00	0.06	0.19	>0.999999999999999	0.3
Q6V1X1	Dipeptidyl peptidase 8 OS=Homo sapiens GN=DPP8 PE=1 SV=1	0.73	0.00	0.00	0.00	0.00	0.00	0.00	0.00	0.00	0.00	0.00	0.00	0.00	0.00	0.00	0.00	0.00	0.00	0.00	0.00	0.00	0.00	0.00	0.00	0.06	0.00	>0.999999999999999	#DIV/0!
Q13099	Intraflagellar transport protein 88 homolog OS=Homo sapiens GN=IFT88 PE=2 SV=2	0.73	0.00	0.00	0.00	0.00	0.00	0.00	0.00	0.00	0.00	0.00	0.00	0.00	0.00	0.00	0.00	0.00	0.00	0.00	0.00	0.00	0.00	0.00	0.00	0.06	0.00	>0.999999999999999	#DIV/0!
Q92817	Envoplakin OS=Homo sapiens GN=EVPL PE=1 SV=3	0.00	0.00	0.00	0.00	0.00	0.00	0.00	0.00	0.00	0.73	0.00	0.00	0.00	0.00	0.00	0.00	0.00	0.00	0.00	0.00	0.00	0.00	0.00	0.00	0.06	0.00	>0.999999999999999	#DIV/0!
P32121	Beta-arrestin-2 OS=Homo sapiens GN=ARRB2 PE=1 SV=2	0.00	0.00	0.00	0.00	0.00	0.00	0.00	0.00	0.00	0.73	0.00	0.00	0.00	0.00	0.00	0.00	0.00	0.00	0.00	0.00	0.00	0.00	0.00	0.00	0.06	0.00	>0.999999999999999	#DIV/0!
Q9C075	Keratin, type I cytoskeletal 23 OS=Homo sapiens GN=KRT23 PE=1 SV=2	0.00	0.00	0.00	0.00	0.00	0.00	0.00	0.00	0.00	0.73	0.00	0.00	0.00	0.00	0.00	0.00	0.00	0.00	0.00	0.00	0.00	0.00	0.00	0.00	0.06	0.00	>0.999999999999999	#DIV/0!
Q9NP62	Chorion-specific transcription factor GCMa OS=Homo sapiens GN=GCM1 PE=2 SV=1	0.00	0.00	0.00	0.00	0.00	0.00	0.00	0.00	0.00	0.73	0.00	0.00	0.00	0.00	0.00	0.00	0.00	0.00	0.00	0.00	0.00	0.00	0.00	0.00	0.06	0.00	>0.999999999999999	#DIV/0!
Q96PK6	RNA-binding protein 14 OS=Homo sapiens GN=RBM14 PE=1 SV=2	0.00	0.00	0.00	0.00	0.00	0.73	0.00	0.00	0.00	0.00	0.00	0.00	0.00	0.00	0.00	0.00	0.00	0.00	0.00	0.00	0.00	0.00	0.00	0.00	0.06	0.00	>0.999999999999999	#DIV/0!
Q8WVV9	Heterogeneous nuclear ribonucleoprotein L-like OS=Homo sapiens GN=HNRPLL PE=1 SV=	0.00	0.00	0.00	0.00	0.00	0.73	0.00	0.00	0.00	0.00	0.00	0.00	0.00	0.00	0.00	0.00	0.00	0.00	0.00	0.00	0.00	0.00	0.00	0.00	0.06	0.00	>0.999999999999999	#DIV/0!
Q86UE4	Protein LYRIC OS=Homo sapiens GN=MTDH PE=1 SV=2	0.00	0.00	0.00	0.00	0.00	0.73	0.00	0.00	0.00	0.00	0.00	0.00	0.00	0.00	0.00	0.00	0.00	0.00	0.00	0.00	0.00	0.00	0.00	0.00	0.06	0.00	>0.999999999999999	#DIV/0!
O14772	Fucose-1-phosphate guanylyltransferase OS=Homo sapiens GN=FPGT PE=1 SV=2	0.00	0.00	0.00	0.00	0.00	0.73	0.00	0.00	0.00	0.00	0.00	0.00	0.00	0.00	0.00	0.00	0.00	0.00	0.00	0.00	0.00	0.00	0.00	0.00	0.06	0.00	>0.999999999999999	#DIV/0!
Q8N1V2	WD repeat-containing protein 16 OS=Homo sapiens GN=WDR16 PE=1 SV=3	0.00	0.00	0.00	0.00	0.00	0.73	0.00	0.00	0.00	0.00	0.00	0.00	0.00	0.00	0.00	0.00	0.00	0.00	0.00	0.00	0.00	0.00	0.00	0.00	0.06	0.00	>0.999999999999999	#DIV/0!
Q12899	Tripartite motif-containing protein 26 OS=Homo sapiens GN=TRIM26 PE=2 SV=1	0.00	0.00	0.00	0.00	0.00	0.73	0.00	0.00	0.00	0.00	0.00	0.00	0.00	0.00	0.00	0.00	0.00	0.00	0.00	0.00	0.00	0.00	0.00	0.00	0.06	0.00	>0.999999999999999	#DIV/0!
Q96RF0	Sorting nexin-18 OS=Homo sapiens GN=SNX18 PE=1 SV=2	0.00	0.00	0.00	0.00	0.00	0.73	0.00	0.00	0.00	0.00	0.00	0.00	0.00	0.00	0.00	0.00	0.00	0.00	0.00	0.00	0.00	0.00	0.00	0.28	0.06	0.02	>0.999999999999999	2.6
Q53GS9	U4/U6.U5 tri-snRNP-associated protein 2 OS=Homo sapiens GN=USP39 PE=1 SV=2	0.00	0.00	0.00	0.00	0.00	0.73	0.00	0.00	0.00	0.00	0.00	0.00	0.00	0.00	0.00	0.00	0.00	0.00	0.00	0.00	0.00	0.00	0.00	0.00	0.06	0.00	>0.999999999999999	#DIV/0!
Q9Y2Y0	ADP-ribosylation factor-like protein 2-binding protein OS=Homo sapiens GN=ARL2BP PE=1	0.00	0.00	0.00	0.00	0.00	0.00	0.00	0.00	0.71	0.00	0.00	0.00	0.00	0.00	0.00	0.00	0.00	0.00	0.00	0.00	0.00	0.00	0.00	0.00	0.06	0.00	>0.999999999999999	#DIV/0!
Q9NWV4	UPF0587 protein C1orf123 OS=Homo sapiens GN=C1orf123 PE=1 SV=1	0.00	0.00	0.00	0.00	0.00	0.00	0.00	0.00	0.71	0.00	0.00	0.00	0.00	3.63	2.00	2.76	0.00	0.00	0.00	2.33	0.00	3.74	0.00	1.02	0.06	1.29	>0.999999999999999	0.0
Q6A1A2	Putative 3-phosphoinositide-dependent protein kinase 2 OS=Homo sapiens GN=PDPK2 PE:	0.00	0.00	0.00	0.00	0.00	0.00	0.00	0.00	0.00	0.00	0.00	0.70	0.00	0.00	0.00	0.00	0.00	0.00	0.00	0.00	0.00	0.00	0.00	0.00	0.06	0.00	>0.999999999999999	#DIV/0!
P45985	Dual specificity mitogen-activated protein kinase kinase 4 OS=Homo sapiens GN=MAP2K4 I	0.00	0.00	0.00	0.00	0.00	0.00	0.00	0.00	0.00	0.00	0.00	0.70	0.00	0.00	0.00	0.00	0.00	0.00	0.00	0.00	0.00	0.00	0.00	0.00	0.06	0.00	>0.999999999999999	#DIV/0!
Q8IUI8	Cytokine receptor-like factor 3 OS=Homo sapiens GN=CRLF3 PE=1 SV=2	0.00	0.00	0.00	0.00	0.00	0.00	0.00	0.00	0.00	0.00	0.00	0.70	0.00	0.00	0.00	0.00	0.00	0.00	0.00	0.00	0.00	0.00	1.07	0.00	0.06	0.09	>0.999999999999999	0.6
Q9BYX2	TBC1 domain family member 2A OS=Homo sapiens GN=TBC1D2 PE=1 SV=3	0.00	0.69	0.00	0.00	0.00	0.00	0.00	0.00	0.00	0.00	0.00	0.00	0.00	0.00	0.00	0.00	0.00	0.00	0.00	0.39	0.00	0.00	0.00	0.00	0.06	0.03	>0.999999999999999	1.8
B1AL46	Protein FAM22E OS=Homo sapiens GN=FAM22E PE=1 SV=3	0.00	0.69	0.00	0.00	0.00	0.00	0.00	0.00	0.00	0.00	0.00	0.00	0.00	0.00	0.00	0.00	0.00	0.00	0.00	0.00	0.00	0.00	0.00	0.00	0.06	0.00	>0.999999999999999	#DIV/0!
Q9H2K8	Serine/threonine-protein kinase TAO3 OS=Homo sapiens GN=TAOK3 PE=1 SV=2	0.00	0.69	0.00	0.00	0.00	0.00	0.00	0.00	0.00	0.00	0.00	0.00	0.00	0.00	0.00	0.00	0.00	0.00	0.00	0.00	0.00	0.00	0.00	0.00	0.06	0.00	>0.999999999999999	#DIV/0!
O14802	DNA-directed RNA polymerase III subunit RPC1 OS=Homo sapiens GN=POLR3A PE=1 SV:	0.00	0.00	0.00	0.30	0.00	0.00	0.00	0.00	0.00	0.00	0.38	0.00	0.00	0.00	0.22	0.00	0.00	0.00	0.00	0.00	0.00	0.00	0.00	0.00	0.06	0.02	>0.999999999999999	3.1

O43665	Regulator of G-protein signaling 10 OS=Homo sapiens GN=RGS10 PE=1 SV=2	0.00	0.00	0.00	0.00	0.00	0.00	0.00	0.00	0.67	0.00	0.00	0.00	0.00	0.00	0.00	0.00	0.00	0.00	0.00	0.00	0.00	0.00	0.00	0.00	0.06	0.00	>0.999999999999999	#DIV/0!
P39880	Homeobox protein cut-like 1 OS=Homo sapiens GN=CUX1 PE=1 SV=3	0.00	0.46	0.00	0.00	0.00	0.00	0.00	0.00	0.00	0.21	0.00	0.00	0.00	0.00	0.00	0.00	0.00	0.00	0.00	0.00	0.00	0.00	0.00	0.00	0.06	0.00	>0.999999999999999	#DIV/0!
Q92859	Neogenin OS=Homo sapiens GN=NEO1 PE=1 SV=2	0.00	0.46	0.00	0.00	0.00	0.00	0.00	0.00	0.00	0.21	0.00	0.00	0.00	0.00	0.00	0.00	0.00	0.00	0.00	0.00	0.00	0.00	0.00	0.00	0.06	0.00	>0.999999999999999	#DIV/0!
O75935	Dynactin subunit 3 OS=Homo sapiens GN=DCTN3 PE=1 SV=1	0.00	0.00	0.00	0.00	0.00	0.00	0.00	0.00	0.63	0.00	0.00	0.00	0.00	0.00	0.00	0.00	0.00	0.00	0.00	0.00	0.00	0.00	0.00	0.00	0.05	0.00	>0.999999999999999	#DIV/0!
O95202	LETM1 and EF-hand domain-containing protein 1, mitochondrial OS=Homo sapiens GN=LET	0.00	0.00	0.00	0.00	0.00	0.00	0.00	0.63	0.00	0.00	0.00	0.00	0.00	0.00	0.00	0.00	0.00	0.00	0.00	0.00	0.00	0.00	0.00	0.00	0.05	0.00	>0.999999999999999	#DIV/0!
P40939	Trifunctional enzyme subunit alpha, mitochondrial OS=Homo sapiens GN=HADHA PE=1 SV	0.00	0.00	0.00	0.00	0.00	0.00	0.00	0.63	0.00	0.00	0.00	0.00	0.35	0.00	0.44	0.00	0.00	0.00	0.00	0.00	0.00	0.00	0.00	0.00	0.05	0.07	>0.999999999999999	0.8
O75582	Ribosomal protein S6 kinase alpha-5 OS=Homo sapiens GN=RPS6KA5 PE=1 SV=1	0.00	0.00	0.00	0.00	0.00	0.00	0.00	0.63	0.00	0.00	0.00	0.00	0.00	0.00	0.00	0.00	0.00	0.00	0.00	0.00	0.00	0.00	0.00	0.00	0.05	0.00	>0.999999999999999	#DIV/0!
Q9UN76	Sodium- and chloride-dependent neutral and basic amino acid transporter B(0+) OS=Homo	0.00	0.00	0.00	0.00	0.00	0.00	0.00	0.63	0.00	0.00	0.00	0.00	0.00	0.00	0.00	0.00	0.00	0.61	0.00	0.00	0.00	0.00	0.00	0.00	0.05	0.05	>0.999999999999999	1.0
Q99523	Sortilin OS=Homo sapiens GN=SORT1 PE=1 SV=3	0.00	0.00	0.00	0.00	0.00	0.00	0.00	0.63	0.00	0.00	0.00	0.00	0.00	0.00	0.00	0.00	0.00	0.00	0.00	0.00	0.12	0.00	0.00	0.00	0.05	0.01	>0.999999999999999	5.1
P43304	Glycerol-3-phosphate dehydrogenase, mitochondrial OS=Homo sapiens GN=GPD2 PE=1 S	0.00	0.00	0.00	0.00	0.00	0.00	0.00	0.63	0.00	0.00	0.00	0.00	0.00	0.00	0.00	0.00	0.00	0.00	0.00	0.00	0.00	0.00	0.00	0.00	0.05	0.00	>0.999999999999999	#DIV/0!
Q96B97	SH3 domain-containing kinase-binding protein 1 OS=Homo sapiens GN=SH3KBP1 PE=1 SV	0.00	0.00	0.00	0.00	0.00	0.00	0.00	0.63	0.00	0.00	0.00	0.00	0.00	0.00	0.00	0.00	0.00	0.00	0.00	0.52	0.00	1.87	0.00	0.00	0.05	0.20	>0.999999999999999	0.3
Q6UWH4	Protein FAM198B OS=Homo sapiens GN=FAM198B PE=2 SV=1	0.00	0.00	0.00	0.00	0.00	0.00	0.00	0.00	0.00	0.63	0.00	0.00	0.00	0.00	0.00	0.00	0.00	0.00	0.00	0.00	0.00	0.00	0.00	0.00	0.05	0.00	>0.999999999999999	#DIV/0!
Q49MG5	Microtubule-associated protein 9 OS=Homo sapiens GN=MAP9 PE=1 SV=3	0.00	0.00	0.00	0.61	0.00	0.00	0.00	0.00	0.00	0.00	0.00	0.00	0.00	0.00	0.00	0.00	0.00	0.00	0.00	0.00	0.00	0.00	0.00	0.00	0.05	0.00	>0.999999999999999	#DIV/0!
O95359	Transforming acidic coiled-coil-containing protein 2 OS=Homo sapiens GN=TACC2 PE=1 SV	0.00	0.00	0.11	0.00	0.00	0.29	0.00	0.00	0.00	0.00	0.00	0.20	0.00	0.00	0.00	0.00	0.00	0.00	0.00	0.00	0.00	0.00	0.00	0.00	0.05	0.00	>0.999999999999999	#DIV/0!
Q86X45	Protein TILB homolog OS=Homo sapiens GN=LRRC6 PE=2 SV=3	0.00	0.00	0.00	0.00	0.00	0.00	0.00	0.00	0.00	0.00	0.00	0.60	0.00	0.00	0.00	0.00	0.00	0.00	0.00	0.00	0.00	0.00	0.00	0.00	0.05	0.00	>0.999999999999999	#DIV/0!
Q9P0V9	Septin-10 OS=Homo sapiens GN=SEPT10 PE=1 SV=2	0.00	0.00	0.00	0.00	0.00	0.00	0.00	0.00	0.00	0.00	0.00	0.60	0.00	0.00	0.00	0.00	0.00	0.00	0.00	0.78	0.00	0.00	0.00	0.00	0.05	0.06	>0.999999999999999	0.8
Q01543	Friend leukemia integration 1 transcription factor OS=Homo sapiens GN=FLI1 PE=1 SV=1	0.00	0.00	0.00	0.00	0.00	0.00	0.00	0.00	0.00	0.00	0.00	0.60	0.00	0.00	0.00	0.00	0.00	0.00	0.00	0.00	0.00	0.00	0.00	0.00	0.05	0.00	>0.999999999999999	#DIV/0!
Q9H173	Nucleotide exchange factor SIL1 OS=Homo sapiens GN=SIL1 PE=1 SV=1	0.00	0.00	0.00	0.00	0.00	0.00	0.00	0.00	0.00	0.00	0.00	0.60	0.00	0.00	0.00	0.00	0.00	0.00	0.00	0.00	0.00	0.00	0.00	0.00	0.05	0.00	>0.999999999999999	#DIV/0!
P14902	Indoleamine 2,3-dioxygenase 1 OS=Homo sapiens GN=IDO1 PE=1 SV=1	0.00	0.00	0.00	0.00	0.00	0.00	0.00	0.00	0.59	0.00	0.00	0.00	0.00	0.00	0.00	0.00	0.00	0.00	0.00	0.00	0.00	0.00	0.00	0.00	0.05	0.00	>0.999999999999999	#DIV/0!
A4D0S4	Laminin subunit beta-4 OS=Homo sapiens GN=LAMB4 PE=2 SV=1	0.00	0.00	0.00	0.00	0.00	0.00	0.00	0.00	0.00	0.21	0.38	0.00	0.00	0.00	0.00	0.00	0.00	0.00	0.00	0.26	0.00	0.00	0.00	0.00	0.05	0.02	>0.999999999999999	2.3
Q8NFP9	Neurobeachin OS=Homo sapiens GN=NBEA PE=1 SV=3	0.00	0.00	0.00	0.00	0.00	0.58	0.00	0.00	0.00	0.00	0.00	0.00	0.00	0.00	0.00	0.00	0.00	0.00	0.00	0.00	0.00	0.00	0.00	0.00	0.05	0.00	>0.999999999999999	#DIV/0!
Q9Y3L3	SH3 domain-binding protein 1 OS=Homo sapiens GN=SH3BP1 PE=1 SV=3	0.00	0.00	0.00	0.00	0.00	0.58	0.00	0.00	0.00	0.00	0.00	0.00	0.00	0.00	0.00	0.00	0.00	0.00	0.00	0.00	0.12	0.83	0.00	0.00	0.05	0.08	>0.999999999999999	0.6
O14639	Actin-binding LIM protein 1 OS=Homo sapiens GN=ABLIM1 PE=1 SV=3	0.00	0.00	0.00	0.00	0.00	0.58	0.00	0.00	0.00	0.00	0.00	0.00	0.00	0.00	0.00	0.00	0.00	0.00	0.00	0.00	0.00	0.00	0.00	0.00	0.05	0.00	>0.999999999999999	#DIV/0!
Q9H6T3	RNA polymerase II-associated protein 3 OS=Homo sapiens GN=RPAP3 PE=1 SV=2	0.00	0.00	0.00	0.00	0.00	0.58	0.00	0.00	0.00	0.00	0.00	0.00	0.00	0.00	0.00	0.00	0.00	0.00	0.00	0.00	0.00	0.00	0.00	0.00	0.05	0.00	>0.999999999999999	#DIV/0!
Q8IVT2	Uncharacterized protein C19orf21 OS=Homo sapiens GN=C19orf21 PE=1 SV=1	0.00	0.00	0.00	0.00	0.00	0.58	0.00	0.00	0.00	0.00	0.00	0.00	0.00	0.00	0.00	0.00	0.00	0.00	0.00	0.52	0.00	0.00	0.00	0.00	0.05	0.04	>0.999999999999999	1.1
O75843	AP-1 complex subunit gamma-like 2 OS=Homo sapiens GN=AP1G2 PE=1 SV=1	0.00	0.00	0.00	0.00	0.00	0.58	0.00	0.00	0.00	0.00	0.00	0.00	0.00	0.00	0.00	0.00	0.00	0.00	0.00	0.00	0.00	0.00	0.00	0.00	0.05	0.00	>0.999999999999999	#DIV/0!
O75175	CCR4-NOT transcription complex subunit 3 OS=Homo sapiens GN=CNOT3 PE=1 SV=1	0.00	0.00	0.00	0.00	0.00	0.58	0.00	0.00	0.00	0.00	0.00	0.00	0.00	0.00	0.00	0.00	0.00	0.00	0.00	0.00	0.00	0.00	0.00	0.00	0.05	0.00	>0.999999999999999	#DIV/0!
Q9Y2V7	Conserved oligomeric Golgi complex subunit 6 OS=Homo sapiens GN=COG6 PE=2 SV=2	0.00	0.00	0.00	0.00	0.00	0.58	0.00	0.00	0.00	0.00	0.00	0.00	0.00	0.00	0.00	0.00	0.00	0.00	0.00	0.00	0.00	0.00	0.00	0.00	0.05	0.00	>0.999999999999999	#DIV/0!
Q8IUC4	Rhophilin-2 OS=Homo sapiens GN=RHPN2 PE=1 SV=1	0.00	0.00	0.00	0.00	0.00	0.58	0.00	0.00	0.00	0.00	0.00	0.00	0.00	0.00	0.00	0.00	0.00	0.00	0.00	0.00	0.00	0.00	0.00	0.00	0.05	0.00	>0.999999999999999	#DIV/0!
Q86V21	Acetoacetyl-CoA synthetase OS=Homo sapiens GN=AACS PE=1 SV=1	0.00	0.00	0.00	0.00	0.00	0.58	0.00	0.00	0.00	0.00	0.00	0.00	0.00	0.00	0.00	0.00	0.00	0.00	0.00	0.00	0.00	0.00	0.00	0.00	0.05	0.00	>0.999999999999999	#DIV/0!
Q01432	AMP deaminase 3 OS=Homo sapiens GN=AMPD3 PE=1 SV=1	0.00	0.00	0.00	0.00	0.00	0.58	0.00	0.00	0.00	0.00	0.00	0.00	0.00	0.00	0.00	0.00	0.00	0.00	0.00	0.00	0.00	0.83	0.00	0.00	0.05	0.07	>0.999999999999999	0.7
Q9NPB8	Glycerophosphocholine phosphodiesterase GPCPD1 OS=Homo sapiens GN=GPCPD1 PE=	0.00	0.00	0.00	0.00	0.00	0.58	0.00	0.00	0.00	0.00	0.00	0.00	0.00	0.00	0.00	0.00	0.00	0.00	0.00	0.52	0.00	0.00	0.00	0.00	0.05	0.04	>0.999999999999999	1.1
O15211	Ral guanine nucleotide dissociation stimulator-like 2 OS=Homo sapiens GN=RGL2 PE=1 SV	0.00	0.00	0.00	0.00	0.00	0.58	0.00	0.00	0.00	0.00	0.00	0.00	0.00	0.00	0.00	0.00	0.00	0.00	0.00	0.00	0.00	0.00	0.00	0.00	0.05	0.00	>0.999999999999999	#DIV/0!
Q8NE18	Putative methyltransferase NSUN7 OS=Homo sapiens GN=NSUN7 PE=2 SV=4	0.00	0.00	0.00	0.00	0.00	0.58	0.00	0.00	0.00	0.00	0.00	0.00	0.00	0.00	0.00	0.00	0.00	0.00	0.00	0.00	0.00	0.00	0.00	0.00	0.05	0.00	>0.999999999999999	#DIV/0!
Q9HCE7	E3 ubiquitin-protein ligase SMURF1 OS=Homo sapiens GN=SMURF1 PE=1 SV=2	0.00	0.00	0.00	0.00	0.00	0.58	0.00	0.00	0.00	0.00	0.00	0.00	0.00	0.00	0.00	0.00	0.00	0.00	0.00	0.00	0.00	0.00	0.00	0.00	0.05	0.00	>0.999999999999999	#DIV/0!
Q9UPT5	Exocyst complex component 7 OS=Homo sapiens GN=EXOC7 PE=1 SV=3	0.00	0.00	0.00	0.00	0.00	0.58	0.00	0.00	0.00	0.00	0.00	0.00	0.00	0.00	0.00	0.00	0.00	0.00	0.00	0.00	0.00	0.00	0.00	0.00	0.05	0.00	>0.999999999999999	#DIV/0!
Q9UKF5	Disintegrin and metalloproteinase domain-containing protein 29 OS=Homo sapiens GN=ADA	0.00	0.00	0.00	0.00	0.58	0.00	0.00	0.00	0.00	0.00	0.00	0.00	0.00	0.00	0.00	0.00	0.00	0.00	0.00	0.00	0.00	0.00	0.00	0.00	0.05	0.00	>0.999999999999999	#DIV/0!
Q86XP3	ATP-dependent RNA helicase DDX42 OS=Homo sapiens GN=DDX42 PE=1 SV=1	0.00	0.00	0.00	0.00	0.00	0.00	0.00	0.00	0.00	0.00	0.57	0.00	0.00	0.60	0.00	0.00	0.00	0.00	0.00	0.00	0.00	0.00	0.00	0.00	0.05	0.05	>0.999999999999999	0.9
Q8IYT8	Serine/threonine-protein kinase ULK2 OS=Homo sapiens GN=ULK2 PE=1 SV=3	0.00	0.00	0.00	0.00	0.00	0.00	0.00	0.00	0.00	0.00	0.57	0.00	0.00	0.00	0.00	0.00	0.00	0.00	0.00	0.00	0.09	0.00	0.00	0.00	0.05	0.01	>0.999999999999999	6.1
Q9NTZ6	RNA-binding protein 12 OS=Homo sapiens GN=RBM12 PE=1 SV=1	0.00	0.00	0.00	0.00	0.00	0.00	0.00	0.00	0.00	0.00	0.57	0.00	0.00	0.00	0.00	0.00	0.00	0.00	0.00	0.00	0.00	0.00	0.00	0.00	0.05	0.00	>0.999999999999999	#DIV/0!
Q15020	Squamous cell carcinoma antigen recognized by T-cells 3 OS=Homo sapiens GN=SART3 P	0.00	0.00	0.00	0.00	0.00	0.00	0.00	0.00	0.00	0.00	0.57	0.00	0.00	0.00	0.00	0.00	0.00	0.00	0.00	0.00	0.00	0.00	0.00	0.00	0.05	0.00	>0.999999999999999	#DIV/0!
O15131	Importin subunit alpha-6 OS=Homo sapiens GN=KPNA5 PE=1 SV=2	0.00	0.00	0.56	0.00	0.00	0.00	0.00	0.00	0.00	0.00	0.00	0.00	0.00	0.00	0.00	0.00	0.00	0.00	0.00	0.00	0.00	0.00	0.00	0.00	0.05	0.00	>0.999999999999999	#DIV/0!
O14773	Tripeptidyl-peptidase 1 OS=Homo sapiens GN=TPP1 PE=1 SV=2	0.00	0.00	0.56	0.00	0.00	0.00	0.00	0.00	0.00	0.00	0.00	0.00	0.00	1.01	0.00	0.34	0.00	0.00	0.00	0.00	0.00	0.00	0.00	0.28	0.05	0.14	>0.999999999999999	0.3
Q9BW04	Specifically androgen-regulated gene protein OS=Homo sapiens GN=SARG PE=1 SV=2	0.00	0.00	0.56	0.00	0.00	0.00	0.00	0.00	0.00	0.00	0.00	0.00	0.00	1.01	0.56	0.34	0.00	0.00	0.00	0.65	0.00	0.00	0.00	0.28	0.05	0.24	>0.999999999999999	0.2
Q9BV36	Melanophilin OS=Homo sapiens GN=MLPH PE=1 SV=1	0.00	0.00	0.56	0.00	0.00	0.00	0.00	0.00	0.00	0.00	0.00	0.00	0.00	0.00	0.00	0.00	0.00	0.00	0.00	0.65	0.00	0.00	0.00	0.00	0.05	0.05	>0.999999999999999	0.9
Q13144	Translation initiation factor eIF-2B subunit epsilon OS=Homo sapiens GN=EIF2B5 PE=1 SV=	0.00	0.00	0.00	0.00	0.00	0.00	0.00	0.00	0.16	0.00	0.00	0.40	0.00	0.00	0.00	0.00	0.00	0.00	0.00	0.00	0.00	0.00	0.00	0.00	0.05	0.00	>0.999999999999999	#DIV/0!
Q92538	Golgi-specific brefeldin A-resistance guanine nucleotide exchange factor 1 OS=Homo sapier	0.00	0.00	0.00	0.00	0.00	0.44	0.00	0.00	0.12	0.00	0.00	0.00	0.00	0.00	0.00	0.00	0.00	0.00	0.00	0.00	0.00	0.00	0.00	0.00	0.05	0.00	>0.999999999999999	#DIV/0!
Q9Y6K5	2'-5'-oligoadenylate synthase 3 OS=Homo sapiens GN=OAS3 PE=1 SV=3	0.00	0.00	0.00	0.00	0.00	0.44	0.00	0.00	0.12	0.00	0.00	0.00	0.00	0.00	0.00	0.00	0.57	0.00	0.00	0.39	0.09	0.00	0.00	0.00	0.05	0.09	>0.999999999999999	0.5
Q9BVM2	Protein DPCD OS=Homo sapiens GN=DPCD PE=1 SV=2	0.00	0.00	0.00	0.00	0.00	0.00	0.00	0.00	0.56	0.00	0.00	0.00	0.00	0.00	0.00	0.00	0.00	0.00	0.00	0.00	0.00	0.00	0.00	0.00	0.05	0.00	>0.999999999999999	#DIV/0!
Q92896	Golgi apparatus protein 1 OS=Homo sapiens GN=GLG1 PE=1 SV=2	0.00	0.00	0.22	0.00	0.00	0.00	0.00	0.32	0.00	0.00	0.00	0.00	0.00	0.00	0.22	0.00	0.00	0.00	0.32	0.00	0.00	0.00	0.00	0.00	0.05	0.04	>0.999999999999999	1.0
Q9P1Z9	Uncharacterized protein C9orf174 OS=Homo sapiens GN=C9orf174 PE=1 SV=2	0.00	0.00	0.22	0.30	0.00	0.00	0.00	0.00	0.00	0.00	0.00	0.00	0.00	0.00	0.00	0.00	0.00	0.00	0.00	0.00	0.00	0.00	0.00	0.00	0.04	0.00	>0.999999999999999	#DIV/0!
Q16610	Extracellular matrix protein 1 OS=Homo sapiens GN=ECM1 PE=1 SV=2	0.00	0.00	0.00	0.00	0.00	0.00	0.00	0.00	0.00	0.52	0.00	0.00	0.00	0.00	0.00	0.00	0.00	0.00	0.00	0.65	0.00	0.00	0.00	0.00	0.04	0.05	>0.999999999999999	0.8
Q14739	Lamin-B receptor OS=Homo sapiens GN=LBR PE=1 SV=2	0.00	0.00	0.00	0.00	0.00	0.00	0.00	0.00	0.00	0.52	0.00	0.00	0.00	0.00	0.00	0.00	0.00	0.00	0.00	0.00	0.00	0.00	0.00	0.00	0.04	0.00	>0.999999999999999	#DIV/0!
Q8IUX8	Epidermal growth factor-like protein 6 OS=Homo sapiens GN=EGFL6 PE=2 SV=1	0.00	0.00	0.00	0.00	0.00	0.00	0.00	0.00	0.00	0.52	0.00	0.00	0.00	0.00	0.00	0.00	0.00	0.00	0.00	0.00	0.00	0.00	0.00	0.00	0.04	0.00	>0.999999999999999	#DIV/0!
Q96BQ1	Protein FAM3D OS=Homo sapiens GN=FAM3D PE=1 SV=1	0.00	0.00	0.00	0.00	0.00	0.00	0.00	0.00	0.52	0.00	0.00	0.00	0.00	0.00	1.44	0.00	0.00	0.00	0.00	0.00	0.00	0.00	0.00	0.00	0.04	0.12	>0.999999999999999	0.4
Q12955	Ankyrin-3 OS=Homo sapiens GN=ANK3 PE=1 SV=3	0.00	0.00	0.11	0.00	0.00	0.00	0.00	0.00	0.16	0.04	0.19	0.00	0.00	0.00	0.11	0.00	0.00	0.00	0.00	0.13	0.00	0.00	0.15	0.00	0.04	0.03	>0.999999999999999	1.3
O00186	Syntaxin-binding protein 3 OS=Homo sapiens GN=STXBP3 PE=1 SV=2	0.00	0.00	0.00	0.00	0.00	0.00	0.00	0.00	0.00	0.00	0.00	0.50	0.00	0.00	0.00	0.00	0.00	0.00	0.00	0.65	0.00	0.00	0.00	0.00	0.04	0.05	>0.999999999999999	0.8
Q9HBG6	Intraflagellar transport protein 122 homolog OS=Homo sapiens GN=IFT122 PE=1 SV=2	0.00	0.00	0.00	0.00	0.00	0.00	0.00	0.00	0.00	0.00	0.00	0.50	0.00	0.00	0.00	0.00	0.00	0.00	0.00	0.00	0.00	0.00	0.00	0.00	0.04	0.00	>0.999999999999999	#DIV/0!
Q8ND83	SLAIN motif-containing protein 1 OS=Homo sapiens GN=SLAIN1 PE=1 SV=3	0.00	0.00	0.00	0.00	0.00	0.00	0.00	0.00	0.00	0.00	0.00	0.50	0.00	0.00	0.56	0.00	0.00	0.00	0.00	0.00	0.00	0.00	0.00	0.00	0.04	0.05	>0.999999999999999	0.9
Q15637	Splicing factor 1 OS=Homo sapiens GN=SF1 PE=1 SV=4	0.00	0.00	0.00	0.00	0.00	0.00	0.00	0.00	0.00	0.00	0.00	0.50	0.00	0.00	0.00	0.00	0.00	0.00	0.00	0.00	0.00	0.00	0.00	0.00	0.04	0.00	>0.999999999999999	#DIV/0!
Q9NYV6	RNA polymerase I-specific transcription initiation factor RRN3 OS=Homo sapiens GN=RRN3	0.00	0.00	0.00	0.00	0.00	0.00	0.00	0.00	0.00	0.00	0.00	0.50	0.00	0.00	0.00	0.00	0.00	0.00	0.00	0.00	0.00	0.00	0.00	0.00	0.04	0.00	>0.999999999999999	#DIV/0!
Q9NRR5	Ubiquilin-4 OS=Homo sapiens GN=UBQLN4 PE=1 SV=2	0.00	0.00	0.00	0.00	0.00	0.00	0.00	0.00	0.00	0.00	0.00	0.50	0.00	0.00	0.56	0.00	0.00	0.00	0.00	0.00	0.00	1.04	0.00	0.00	0.04	0.13	>0.999999999999999	0.3
O14841	5-oxoprolinase OS=Homo sapiens GN=OPLAH PE=1 SV=3	0.00	0.00	0.00	0.00	0.00	0.29	0.00	0.00	0.00	0.00	0.00	0.20	0.00	0.00	0.00	0.14	0.00	0.31	0.00	0.00	0.00	0.42	0.00	0.00	0.04	0.07	>0.999999999999999	0.6
Q66K14	TBC1 domain family member 9B OS=Homo sapiens GN=TBC1D9B PE=1 SV=3	0.00	0.00	0.00	0.00	0.00	0.00	0.29	0.00	0.00	0.00	0.00	0.20	0.00	0.00	0.00	0.00	0.00	0.00	0.00	0.00	0.00	0.00	0.00	0.00	0.04	0.00	>0.999999999999999	#DIV/0!
O95163	Elongator complex protein 1 OS=Homo sapiens GN=IKBKAP PE=1 SV=3	0.49	0.00	0.00	0.00	0.00	0.00	0.00	0.00	0.00	0.00	0.00	0.00	0.00	0.00	0.00	0.00	0.00	0.00	0.00	0.00	0.00	0.00	0.00	0.00	0.04	0.00	>0.999999999999999	#DIV/0!
Q9H2M9	Rab3 GTPase-activating protein non-catalytic subunit OS=Homo sapiens GN=RAB3GAP2 P	0.49	0.00	0.00	0.00	0.00	0.00	0.00	0.00	0.00	0.00	0.00	0.00	0.00	0.00	0.00	0.00	0.00	0.00	0.00	0.00	0.00	0.00	0.00	0.00	0.04	0.00	>0.999999999999999	#DIV/0!
Q6Y7W6	PERQ amino acid-rich with GYF domain-containing protein 2 OS=Homo sapiens GN=GIGYF	0.49	0.00	0.00	0.00	0.00	0.00	0.00	0.00	0.00	0.00	0.00	0.00	0.00	0.00	0.00	0.00	0.00	0.00	0.00	0.00	0.00	0.00	0.00	0.00	0.04	0.00	>0.999999999999999	#DIV/0!
Q9BZH6	WD repeat-containing protein 11 OS=Homo sapiens GN=WDR11 PE=1 SV=1	0.49	0.00	0.00	0.00	0.00	0.00	0.00	0.00	0.00	0.00	0.00	0.00	0.00	0.00	0.00	0.00	0.00	0.00	0.00	0.26	0.00	0.00	0.00	0.00	0.04	0.02	>0.999999999999999	1.9
O95759	TBC1 domain family member 8 OS=Homo sapiens GN=TBC1D8 PE=1 SV=3	0.49	0.00	0.00	0.00	0.00	0.00	0.00	0.00	0.00	0.00	0.00	0.00	0.00	0.00	0.00	0.00	0.00	0.00	0.00	0.00	0.00	0.00	0.00	0.00	0.04	0.00	>0.999999999999999	#DIV/0!
Q9Y4F5	Protein KIAA0284 OS=Homo sapiens GN=KIAA0284 PE=1 SV=4	0.49	0.00	0.00	0.00	0.00	0.00	0.00	0.00	0.00	0.00	0.00	0.00	0.00	0.00	0.00	0.00	0.00	0.00	0.00	0.00	0.00	0.00	0.00	0.00	0.04	0.00	>0.999999999999999	#DIV/0!
Q5T5P2	Sickle tail protein homolog OS=Homo sapiens GN=SKT PE=1 SV=2	0.49	0.00	0.00	0.00	0.00	0.00	0.00	0.00	0.00	0.00	0.00	0.00	0.00	0.00	0.00	0.00	0.00	0.00	0.00	0.00	0.00	0.00	0.00	0.00	0.04	0.00	>0.999999999999999	#DIV/0!
Q96MR6	WD repeat-containing protein 65 OS=Homo sapiens GN=WDR65 PE=1 SV=3	0.49	0.00	0.00	0.00	0.00	0.00	0.00	0.00	0.00	0.00	0.00	0.00	0.00	0.00	0.00	0.00	0.00	0.00	0.00	0.00	0.00	0.00	0.00	0.00	0.04	0.00	>0.999999999999999	#DIV/0!
Q5JTH9	RRP12-like protein OS=Homo sapiens GN=RRP12 PE=1 SV=2	0.49	0.00	0.00	0.00	0.00	0.00	0.00	0.00	0.00	0.00	0.00	0.00	0.00	0.00	0.00	0.00	0.00	0.00	0.00	0.00	0.00	0.00	0.00	0.00	0.04	0.00	>0.999999999999999	#DIV/0!
P15313	V-type proton ATPase subunit B, kidney isoform OS=Homo sapiens GN=ATP6V1B1 PE=1 S	0.00	0.00	0.00	0.00	0.00	0.00	0.00	0.00	0.48	0.00	0.00	0.00	0.00	0.00	0.00	0.00	0.00	0.00	0.00	1.55	0.00	0.00	0.00	0.00	0.04	0.13	>0.999999999999999	0.3
Q96B36	Proline-rich AKT1 substrate 1 OS=Homo sapiens GN=AKT1S1 PE=1 SV=1	0.00	0.00	0.00	0.00	0.00	0.00	0.00	0.00	0.48	0.00	0.00	0.00	0.00	0.00	0.00	0.00	0.00	0.00	0.00	0.00	0.00	0.00	0.00	0.00	0.04	0.00	>0.999999999999999	#DIV/0!
Q8IYM0	Protein FAM186B OS=Homo sapiens GN=FAM186B PE=2 SV=2	0.00	0.00	0.00	0.00	0.00	0.00	0.00	0.48	0.00	0.00	0.00	0.00	0.00	0.00	0.00	0.00	0.00	0.00	0.00	0.00	0.00	0.00	0.00	0.00	0.04	0.00	>0.999999999999999	#DIV/0!
Q9C0E2	Exportin-4 OS=Homo sapiens GN=XPO4 PE=1 SV=2	0.00	0.46	0.00	0.00	0.00	0.00	0.00	0.00	0.00	0.00	0.00	0.00	0.00	0.00	0.00	0.00	0.00	0.00	0.00	0.00	0.00	0.00	0.00	0.00	0.04	0.00	>0.999999999999999	#DIV/0!
Q13029	PR domain zinc finger protein 2 OS=Homo sapiens GN=PRDM2 PE=1 SV=3	0.00	0.46	0.00	0.00	0.00	0.00	0.00	0.00	0.00	0.00	0.00	0.00	0.00	0.00	0.00	0.00	0.00	0.00	0.00	0.00	0.00	0.00	0.00	0.00	0.04	0.00	>0.999999999999999	#DIV/0!
Q96T51	RUN and FYVE domain-containing protein 1 OS=Homo sapiens GN=RUFY1 PE=1 SV=2	0.00	0.00	0.45	0.00	0.00	0.00	0.00	0.00	0.00	0.00	0.00	0.00	0.00	0.00	0.00	0.00	0.00	0.00	0.00	0.00	0.00	0.00	0.00	0.00	0.04	0.00	>0.999999999999999	#DIV/0!
Q14139	Ubiquitin conjugation factor E4 A OS=Homo sapiens GN=UBE4A PE=1 SV=2	0.00	0.00	0.00	0.00	0.00	0.44	0.00	0.00	0.00	0.00	0.00	0.00	0.00	0.00	0.00	0.00	0.00	0.00	0.00	0.00	0.00	0.00	0.00	0.00	0.04	0.00	>0.999999999999999	#DIV/0!
P82094	TATA element modulatory factor OS=Homo sapiens GN=TMF1 PE=1 SV=2	0.00	0.00	0.00	0.00	0.00	0.44	0.00	0.00	0.00	0.00	0.00	0.00	0.00	0.00	0.00	0.00	0.00	0.00	0.00	0.00	0.00	0.00	0.00	0.00	0.04	0.00	>0.999999999999999	#DIV/0!
Q5JWF2	Guanine nucleotide-binding protein G(s) subunit alpha isoforms XLas OS=Homo sapiens GN	0.00	0.00	0.00	0.00	0.00	0.44	0.00	0.00	0.00	0.00	0.00	0.00	0.00	0.00	0.00	0.00	0.00	0.00	0.00	0.00	0.00	0.00	0.46	0.00	0.04	0.04	>0.999999999999999	0.9
O14936	Peripheral plasma membrane protein CASK OS=Homo sapiens GN=CASK PE=1 SV=3	0.00	0.00	0.00	0.00	0.00	0.44	0.00	0.00	0.00	0.00	0.00	0.00	0.00	0.00	0.00	0.00	0.00	0.00	0.00	0.00	0.00	0.00	0.00	0.00	0.04	0.00	>0.999999999999999	#DIV/0!
Q05397	Focal adhesion kinase 1 OS=Homo sapiens GN=PTK2 PE=1 SV=2	0.00	0.00	0.00	0.00	0.00	0.44	0.00	0.00	0.00	0.00	0.00	0.00	0.00	0.00	0.00	0.00	0.00	0.00	0.00	0.00	0.00	0.00	0.00	0.00	0.04	0.00	>0.999999999999999	#DIV/0!
Q658Y4	Protein FAM91A1 OS=Homo sapiens GN=FAM91A1 PE=1 SV=3	0.00	0.00	0.00	0.00	0.00	0.44	0.00	0.00	0.00	0.00	0.00	0.00	0.00	0.00	0.00	0.00	0.00	0.00	0.00	0.00	0.00	0.00	0.00	0.00	0.04	0.00	>0.999999999999999	#DIV/0!
Q15276	Rab GTPase-binding effector protein 1 OS=Homo sapiens GN=RABEP1 PE=1 SV=2	0.00	0.00	0.00	0.00	0.00	0.44	0.00	0.00	0.00	0.00	0.00	0.00	0.00	0.00	0.00	0.00	0.00	0.00	0.00	0.00	0.00	0.00	0.00	0.00	0.04	0.00	>0.999999999999999	#DIV/0!
Q8WTW3	Conserved oligomeric Golgi complex subunit 1 OS=Homo sapiens GN=COG1 PE=1 SV=1	0.00	0.00	0.00	0.00	0.00	0.44	0.00	0.00	0.00	0.00	0.00	0.00	0.00	0.00	0.00	0.00	0.00	0.00	0.00	0.00	0.00	0.00	0.00	0.00	0.04	0.00	>0.999999999999999	#DIV/0!
Q9BXP5	Serrate RNA effector molecule homolog OS=Homo sapiens GN=SRRT PE=1 SV=1	0.00	0.00	0.00	0.00	0.00	0.44	0.00	0.00	0.00	0.00	0.00	0.00	0.00	0.00	0.00	0.00	0.00	0.00	0.00	0.39	0.00	0.00	0.00	0.00	0.04	0.03	>0.999999999999999	1.1
Q15042	Rab3 GTPase-activating protein catalytic subunit OS=Homo sapiens GN=RAB3GAP1 PE=1	0.00	0.00	0.00	0.00	0.00	0.44	0.00	0.00	0.00	0.00	0.00	0.00	0.00	0.00	0.00	0.00	0.00	0.00	0.00	0.00	0.00	0.00	0.00	0.17	0.04	0.01	>0.999999999999999	2.6
Q9Y3P9	Rab GTPase-activating protein 1 OS=Homo sapiens GN=RABGAP1 PE=1 SV=3	0.00	0.00	0.00	0.00	0.00	0.44	0.00	0.00	0.00	0.00	0.00	0.00	0.00	0.00	0.00	0.00	0.00	0.00	0.00	0.00	0.00	0.00	0.00	0.00	0.04	0.00	>0.999999999999999	#DIV/0!
Q9H9H4	Vacuolar protein sorting-associated protein 37B OS=Homo sapiens GN=VPS37B PE=1 SV=	0.00	0.00	0.00	0.00	0.00	0.00	0.00	0.00	0.44	0.00	0.00	0.00	0.00	2.22	0.00	0.00	0.00	0.00	0.00	0.00	0.00	0.00	0.00	0.00	0.04	0.18	>0.999999999999999	0.2
Q16270	Insulin-like growth factor-binding protein 7 OS=Homo sapiens GN=IGFBP7 PE=1 SV=1	0.00	0.00	0.00	0.00	0.00	0.00	0.00	0.00	0.44	0.00	0.00	0.00	0.00	0.00	0.00	0.00	0.00	0.00	0.00	0.00	0.00	0.00	0.00	0.00	0.04	0.00	>0.999999999999999	#DIV/0!
P46939	Utrophin OS=Homo sapiens GN=UTRN PE=1 SV=2	0.24	0.00	0.00	0.00	0.00	0.00	0.00	0.00	0.00	0.00	0.19	0.00	0.00	0.00	0.00	0.00	0.00	0.00	0.00	0.00	0.00	0.21	0.00	0.00	0.04	0.02	>0.999999999999999	2.1
Q8TE73	Dynein heavy chain 5, axonemal OS=Homo sapiens GN=DNAH5 PE=1 SV=3	0.24	0.00	0.00	0.00	0.00	0.00	0.00	0.00	0.00	0.00	0.19	0.00	0.00	0.00	0.00	0.00	0.00	0.00	0.00	0.00	0.00	0.00	0.00	0.00	0.04	0.00	>0.999999999999999	#DIV/0!
O43149	Zinc finger ZZ-type and EF-hand domain-containing protein 1 OS=Homo sapiens GN=ZZEF	0.24	0.00	0.00	0.00	0.00	0.00	0.00	0.00	0.00	0.00	0.19	0.00	0.09	0.00	0.00	0.00	0.00	0.00	0.00	0.00	0.00	0.00	0.00	0.06	0.04	0.01	>0.999999999999999	3.0
P30876	DNA-directed RNA polymerase II subunit RPB2 OS=Homo sapiens GN=POLR2B PE=1 SV=	0.00	0.00	0.22	0.00	0.00	0.00	0.00	0.00	0.00	0.00	0.00	0.20	0.00	0.00	0.00	0.00	0.00	0.00	0.00	0.00	0.00	0.00	0.00	0.00	0.04	0.00	>0.999999999999999	#DIV/0!
Q86UX7	Fermitin family homolog 3 OS=Homo sapiens GN=FERMT3 PE=1 SV=1	0.00	0.00	0.00	0.00	0.00	0.00	0.00	0.00	0.00	0.42	0.00	0.00	0.00	0.00	0.00	0.00	0.00	0.00	0.00	0.00	0.00	1.87	0.00	0.00	0.03	0.16	>0.999999999999999	0.2
Q6XPR3	Repetin OS=Homo sapiens GN=RPTN PE=1 SV=1	0.00	0.00	0.00	0.00	0.00	0.00	0.00	0.00	0.00	0.42	0.00	0.00	0.00	0.00	0.00	0.00	0.00	0.00	0.00	0.00	0.00	0.00	0.00	0.00	0.03	0.00	>0.999999999999999	#DIV/0!
P08575	Receptor-type tyrosine-protein phosphatase C OS=Homo sapiens GN=PTPRC PE=1 SV=2	0.00	0.00	0.00	0.00	0.00	0.00	0.00	0.00	0.00	0.42	0.00	0.00	0.00	0.00	0.00	0.00	0.00	0.00	0.00	0.00	0.00	0.00	0.00	0.00	0.03	0.00	>0.999999999999999	#DIV/0!
Q9NR71	Neutral ceramidase OS=Homo sapiens GN=ASAH2 PE=1 SV=2	0.00	0.00	0.00	0.00	0.00	0.00	0.00	0.00	0.00	0.42	0.00	0.00	0.00	0.00	0.00	0.00	0.00	0.00	0.00	0.00	0.00	0.00	0.00	0.00	0.03	0.00	>0.999999999999999	#DIV/0!
P09917	Arachidonate 5-lipoxygenase OS=Homo sapiens GN=ALOX5 PE=1 SV=2	0.00	0.00	0.00	0.00	0.00	0.00	0.00	0.00	0.00	0.42	0.00	0.00	0.00	0.00	0.00	0.00	0.00	0.00	0.00	0.00	0.00	0.00	0.00	0.00	0.03	0.00	>0.999999999999999	#DIV/0!
Q8IVF2	Protein AHNAK2 OS=Homo sapiens GN=AHNAK2 PE=1 SV=2	0.00	0.00	0.00	0.00	0.00	0.15	0.00	0.16	0.00	0.00	0.00	0.10	0.00	0.00	0.00	0.00	0.00	0.15	0.00	0.13	0.00	0.00	0.00	0.06	0.03	0.03	>0.999999999999999	1.2
Q9C0I1	Myotubularin-related protein 12 OS=Homo sapiens GN=MTMR12 PE=1 SV=2	0.00	0.00	0.00	0.00	0.00	0.00	0.00	0.00	0.00	0.00	0.00	0.40	0.00	0.00	0.00	0.00	0.00	0.00	0.00	0.00	0.00	0.00	0.00	0.00	0.03	0.00	>0.999999999999999	#DIV/0!
Q6TDU7	Cancer susceptibility candidate protein 1 OS=Homo sapiens GN=CASC1 PE=2 SV=2	0.00	0.00	0.00	0.00	0.00	0.00	0.00	0.00	0.00	0.00	0.00	0.40	0.00	0.00	0.00	0.00	0.00	0.00	0.00	0.00	0.00	0.00	0.00	0.00	0.03	0.00	>0.999999999999999	#DIV/0!
Q17RC7	Exocyst complex component 3-like protein 4 OS=Homo sapiens GN=EXOC3L4 PE=2 SV=2	0.00	0.00	0.00	0.00	0.00	0.00	0.00	0.00	0.00	0.00	0.00	0.40	0.00	0.00	0.00	0.00	0.00	0.00	0.00	0.00	0.00	0.00	0.00	0.00	0.03	0.00	>0.999999999999999	#DIV/0!
Q9UKG1	DCC-interacting protein 13-alpha OS=Homo sapiens GN=APPL1 PE=1 SV=1	0.00	0.00	0.00	0.00	0.00	0.00	0.00	0.00	0.00	0.00	0.00	0.40	0.00	0.00	0.00	0.00	0.00	0.00	0.00	0.00	0.00	0.00	0.00	0.00	0.03	0.00	>0.999999999999999	#DIV/0!
Q9BYT8	Neurolysin, mitochondrial OS=Homo sapiens GN=NLN PE=1 SV=1	0.00	0.00	0.00	0.00	0.00	0.00	0.00	0.00	0.00	0.00	0.00	0.40	0.00	0.00	0.00	0.00	0.00	0.00	0.00	0.00	0.00	0.00	0.00	0.00	0.03	0.00	>0.999999999999999	#DIV/0!
Q5TH69	Brefeldin A-inhibited guanine nucleotide-exchange protein 3 OS=Homo sapiens GN=ARFGE	0.24	0.00	0.00	0.00	0.00	0.15	0.00	0.00	0.00	0.00	0.00	0.00	0.00	0.00	0.00	0.00	0.00	0.00	0.00	0.00	0.00	0.00	0.00	0.00	0.03	0.00	>0.999999999999999	#DIV/0!
Q9ULT8	E3 ubiquitin-protein ligase HECTD1 OS=Homo sapiens GN=HECTD1 PE=1 SV=3	0.24	0.00	0.00	0.00	0.00	0.15	0.00	0.00	0.00	0.00	0.00	0.00	0.00	0.00	0.00	0.00	0.00	0.00	0.00	0.13	0.00	0.00	0.00	0.00	0.03	0.01	>0.999999999999999	3.0
Q13464	Rho-associated protein kinase 1 OS=Homo sapiens GN=ROCK1 PE=1 SV=1	0.00	0.00	0.00	0.00	0.00	0.00	0.00	0.00	0.00	0.00	0.38	0.00	0.00	0.00	0.00	0.00	0.00	0.31	0.00	0.00	0.00	0.00	0.31	0.00	0.03	0.05	>0.999999999999999	0.6
O75694	Nuclear pore complex protein Nup155 OS=Homo sapiens GN=NUP155 PE=1 SV=1	0.00	0.00	0.00	0.00	0.00	0.00	0.00	0.00	0.00	0.00	0.38	0.00	0.00	0.00	0.00	0.00	0.00	0.00	0.00	0.00	0.00	0.00	0.00	0.00	0.03	0.00	>0.999999999999999	#DIV/0!
Q8TBY9	WD repeat-containing protein 66 OS=Homo sapiens GN=WDR66 PE=1 SV=2	0.00	0.00	0.00	0.00	0.00	0.00	0.00	0.00	0.00	0.00	0.38	0.00	0.00	0.00	0.00	0.00	0.00	0.00	0.00	0.00	0.00	0.00	0.00	0.00	0.03	0.00	>0.999999999999999	#DIV/0!
Q14997	Proteasome activator complex subunit 4 OS=Homo sapiens GN=PSME4 PE=1 SV=2	0.00	0.00	0.00	0.00	0.00	0.29	0.00	0.00	0.08	0.00	0.00	0.00	0.00	0.00	0.00	0.00	0.00	0.00	0.00	0.00	0.00	0.00	0.00	0.00	0.03	0.00	>0.999999999999999	#DIV/0!
Q96JY6	PDZ and LIM domain protein 2 OS=Homo sapiens GN=PDLIM2 PE=1 SV=1	0.00	0.00	0.00	0.00	0.00	0.00	0.00	0.00	0.36	0.00	0.00	0.00	0.00	0.00	0.00	0.00	0.00	0.00	0.00	0.00	0.00	0.00	0.00	0.51	0.03	0.04	>0.999999999999999	0.7
Q96EU7	C1GALT1-specific chaperone 1 OS=Homo sapiens GN=C1GALT1C1 PE=1 SV=1	0.00	0.00	0.00	0.00	0.00	0.00	0.00	0.00	0.36	0.00	0.00	0.00	0.00	0.00	0.00	0.00	0.00	0.00	0.00	0.00	0.00	0.00	0.00	0.00	0.03	0.00	>0.999999999999999	#DIV/0!
O75436	Vacuolar protein sorting-associated protein 26A OS=Homo sapiens GN=VPS26A PE=1 SV=	0.00	0.00	0.00	0.00	0.00	0.00	0.00	0.00	0.36	0.00	0.00	0.00	0.00	0.00	0.00	0.00	0.00	0.00	0.00	2.33	0.00	0.00	0.00	0.00	0.03	0.19	>0.999999999999999	0.2
O00273	DNA fragmentation factor subunit alpha OS=Homo sapiens GN=DFFA PE=1 SV=1	0.00	0.00	0.00	0.00	0.00	0.00	0.00	0.00	0.36	0.00	0.00	0.00	0.00	0.00	0.00	0.00	0.00	0.00	0.00	0.00	0.00	0.00	0.00	0.00	0.03	0.00	>0.999999999999999	#DIV/0!
Q9H4A3	Serine/threonine-protein kinase WNK1 OS=Homo sapiens GN=WNK1 PE=1 SV=2	0.24	0.00	0.00	0.00	0.00	0.00	0.00	0.00	0.00	0.00	0.00	0.10	0.00	0.00	0.00	0.00	0.00	0.00	0.00	0.00	0.00	0.00	0.00	0.00	0.03	0.00	>0.999999999999999	#DIV/0!
Q9H4L5	Oxysterol-binding protein-related protein 3 OS=Homo sapiens GN=OSBPL3 PE=1 SV=1	0.00	0.00	0.34	0.00	0.00	0.00	0.00	0.00	0.00	0.00	0.00	0.00	0.00	0.00	0.00	0.00	0.00	0.00	0.00	0.00	0.00	0.00	0.00	0.00	0.03	0.00	>0.999999999999999	#DIV/0!
O00268	Transcription initiation factor TFIID subunit 4 OS=Homo sapiens GN=TAF4 PE=1 SV=2	0.00	0.00	0.34	0.00	0.00	0.00	0.00	0.00	0.00	0.00	0.00	0.00	0.00	0.00	0.00	0.00	0.21	0.00	0.00	0.00	0.00	0.00	0.00	0.00	0.03	0.02	>0.999999999999999	1.6
O60716	Catenin delta-1 OS=Homo sapiens GN=CTNND1 PE=1 SV=1	0.00	0.00	0.34	0.00	0.00	0.00	0.00	0.00	0.00	0.00	0.00	0.00	0.00	0.00	0.00	0.00	0.00	0.00	0.48	0.00	0.00	0.00	0.00	0.00	0.03	0.04	>0.999999999999999	0.7
O95049	Tight junction protein ZO-3 OS=Homo sapiens GN=TJP3 PE=1 SV=2	0.00	0.00	0.34	0.00	0.00	0.00	0.00	0.00	0.00	0.00	0.00	0.00	0.00	0.00	0.00	0.00	0.00	0.00	0.00	0.00	0.00	0.00	0.00	0.00	0.03	0.00	>0.999999999999999	#DIV/0!
Q9UGM5	Fetuin-B OS=Homo sapiens GN=FETUB PE=1 SV=2	0.00	0.00	0.00	0.00	0.00	0.00	0.00	0.00	0.32	0.00	0.00	0.00	0.00	0.00	0.00	0.00	0.00	0.00	0.00	0.00	0.00	0.00	0.00	0.00	0.03	0.00	>0.999999999999999	#DIV/0!
Q8IU85	Calcium/calmodulin-dependent protein kinase type 1D OS=Homo sapiens GN=CAMK1D PE	0.00	0.00	0.00	0.00	0.00	0.00	0.00	0.00	0.32	0.00	0.00	0.00	0.00	0.00	0.00	0.55	0.00	0.00	0.00	0.00	0.00	0.00	0.00	0.00	0.03	0.05	>0.999999999999999	0.6
Q99961	Endophilin-A2 OS=Homo sapiens GN=SH3GL1 PE=1 SV=1	0.00	0.00	0.00	0.00	0.00	0.00	0.00	0.00	0.32	0.00	0.00	0.00	0.00	0.00	0.00	0.00	0.00	0.00	0.00	0.00	0.00	0.00	0.00	0.00	0.03	0.00	>0.999999999999999	#DIV/0!
A8K2U0	Alpha-2-macroglobulin-like protein 1 OS=Homo sapiens GN=A2ML1 PE=1 SV=3	0.00	0.00	0.00	0.00	0.00	0.00	0.00	0.32	0.00	0.00	0.00	0.00	0.00	0.00	0.00	0.00	0.00	0.00	0.00	0.00	0.00	0.00	0.00	0.00	0.03	0.00	>0.999999999999999	#DIV/0!
Q5D862	Filaggrin-2 OS=Homo sapiens GN=FLG2 PE=1 SV=1	0.00	0.00	0.00	0.00	0.00	0.00	0.00	0.00	0.31	0.00	0.00	0.00	0.00	0.00	0.00	0.00	0.00	0.00	0.00	0.00	0.00	0.00	0.00	0.00	0.03	0.00	>0.999999999999999	#DIV/0!
Q0VD83	Apolipoprotein B receptor OS=Homo sapiens GN=APOBR PE=1 SV=1	0.00	0.00	0.00	0.00	0.00	0.00	0.00	0.00	0.31	0.00	0.00	0.00	0.00	0.00	0.00	0.00	0.00	0.00	0.00	0.00	0.00	0.62	0.46	0.00	0.03	0.09	>0.999999999999999	0.3
Q92619	Minor histocompatibility protein HA-1 OS=Homo sapiens GN=HMHA1 PE=1 SV=2	0.00	0.00	0.00	0.00	0.00	0.00	0.00	0.00	0.31	0.00	0.00	0.00	0.00	0.00	0.00	0.00	0.00	0.00	0.00	0.00	0.00	0.00	0.00	0.00	0.03	0.00	>0.999999999999999	#DIV/0!
A8MQ14	Zinc finger protein 850 OS=Homo sapiens GN=ZNF850 PE=3 SV=2	0.00	0.00	0.00	0.00	0.00	0.00	0.00	0.00	0.31	0.00	0.00	0.00	0.00	0.00	0.00	0.00	0.00	0.00	0.00	0.00	0.00	0.00	0.00	0.00	0.03	0.00	>0.999999999999999	#DIV/0!
Q53GL7	Poly [ADP-ribose] polymerase 10 OS=Homo sapiens GN=PARP10 PE=1 SV=2	0.00	0.00	0.00	0.00	0.00	0.00	0.00	0.00	0.00	0.00	0.00	0.30	0.00	0.00	0.00	0.00	0.00	0.00	0.00	0.00	0.00	0.00	0.00	0.00	0.02	0.00	>0.999999999999999	#DIV/0!
P42566	Epidermal growth factor receptor substrate 15 OS=Homo sapiens GN=EPS15 PE=1 SV=2	0.00	0.00	0.00	0.00	0.00	0.00	0.00	0.00	0.00	0.00	0.00	0.30	0.00	0.60	0.00	0.00	0.00	0.00	0.00	0.00	0.00	0.00	0.00	0.00	0.02	0.05	>0.999999999999999	0.5
Q5T447	E3 ubiquitin-protein ligase HECTD3 OS=Homo sapiens GN=HECTD3 PE=1 SV=1	0.00	0.00	0.00	0.00	0.00	0.00	0.00	0.00	0.00	0.00	0.00	0.30	0.00	0.00	0.00	0.00	0.00	0.00	0.00	0.00	0.00	0.00	0.00	0.00	0.02	0.00	>0.999999999999999	#DIV/0!
Q5T0F9	Coiled-coil and C2 domain-containing protein 1B OS=Homo sapiens GN=CC2D1B PE=1 SV=	0.00	0.00	0.00	0.00	0.00	0.00	0.00	0.00	0.00	0.00	0.00	0.30	0.00	0.00	0.00	0.00	0.00	0.00	0.00	0.00	0.00	0.00	0.00	0.00	0.02	0.00	>0.999999999999999	#DIV/0!
P20273	B-cell receptor CD22 OS=Homo sapiens GN=CD22 PE=1 SV=2	0.00	0.00	0.00	0.00	0.00	0.00	0.00	0.00	0.00	0.00	0.00	0.30	0.00	0.00	0.00	0.00	0.00	0.00	0.00	0.00	0.00	0.00	0.00	0.00	0.02	0.00	>0.999999999999999	#DIV/0!
O15327	Type II inositol-3,4-bisphosphate 4-phosphatase OS=Homo sapiens GN=INPP4B PE=2 SV=	0.00	0.00	0.00	0.00	0.00	0.00	0.00	0.00	0.00	0.00	0.00	0.30	0.00	0.00	0.00	0.00	0.00	0.00	0.00	0.00	0.00	0.00	0.00	0.00	0.02	0.00	>0.999999999999999	#DIV/0!
P46379	Large proline-rich protein BAG6 OS=Homo sapiens GN=BAG6 PE=1 SV=2	0.00	0.00	0.00	0.00	0.00	0.00	0.00	0.00	0.00	0.00	0.00	0.30	0.00	0.00	0.00	0.00	0.00	0.00	0.00	0.00	0.00	0.00	0.00	0.00	0.02	0.00	>0.999999999999999	#DIV/0!
Q15154	Pericentriolar material 1 protein OS=Homo sapiens GN=PCM1 PE=1 SV=4	0.00	0.00	0.00	0.15	0.00	0.15	0.00	0.00	0.00	0.00	0.00	0.00	0.00	0.00	0.00	0.00	0.00	0.00	0.00	0.00	0.00	0.00	0.00	0.00	0.02	0.00	>0.999999999999999	#DIV/0!
Q92954	Proteoglycan 4 OS=Homo sapiens GN=PRG4 PE=1 SV=2	0.00	0.00	0.00	0.00	0.00	0.29	0.00	0.00	0.00	0.00	0.00	0.00	0.00	0.00	0.00	0.00	0.00	0.00	0.00	0.26	0.00	0.00	0.00	0.00	0.02	0.02	>0.999999999999999	1.1
O43861	Probable phospholipid-transporting ATPase IIB OS=Homo sapiens GN=ATP9B PE=2 SV=4	0.00	0.00	0.00	0.00	0.00	0.29	0.00	0.00	0.00	0.00	0.00	0.00	0.00	0.00	0.00	0.00	0.00	0.00	0.00	0.00	0.00	0.00	0.00	0.00	0.02	0.00	>0.999999999999999	#DIV/0!
Q9ULV0	Unconventional myosin-Vb OS=Homo sapiens GN=MYO5B PE=1 SV=3	0.00	0.00	0.00	0.00	0.00	0.29	0.00	0.00	0.00	0.00	0.00	0.00	0.00	0.00	0.00	0.00	0.00	0.00	0.00	0.00	0.00	0.00	0.00	0.00	0.02	0.00	>0.999999999999999	#DIV/0!
Q15047	Histone-lysine N-methyltransferase SETDB1 OS=Homo sapiens GN=SETDB1 PE=1 SV=1	0.00	0.00	0.00	0.00	0.00	0.29	0.00	0.00	0.00	0.00	0.00	0.00	0.18	0.00	0.00	0.00	0.00	0.00	0.00	0.00	0.00	0.00	0.00	0.00	0.02	0.01	>0.999999999999999	1.7
Q12888	Tumor suppressor p53-binding protein 1 OS=Homo sapiens GN=TP53BP1 PE=1 SV=2	0.00	0.00	0.00	0.00	0.00	0.29	0.00	0.00	0.00	0.00	0.00	0.00	0.00	0.00	0.00	0.00	0.00	0.00	0.00	0.00	0.00	0.00	0.00	0.00	0.02	0.00	>0.999999999999999	#DIV/0!
Q86WI3	Protein NLRC5 OS=Homo sapiens GN=NLRC5 PE=1 SV=3	0.00	0.00	0.00	0.00	0.00	0.29	0.00	0.00	0.00	0.00	0.00	0.00	0.00	0.00	0.00	0.00	0.00	0.00	0.00	0.00	0.00	0.00	0.00	0.00	0.02	0.00	>0.999999999999999	#DIV/0!
Q63HN8	E3 ubiquitin-protein ligase RNF213 OS=Homo sapiens GN=RNF213 PE=1 SV=3	0.00	0.00	0.00	0.00	0.00	0.15	0.00	0.00	0.04	0.00	0.00	0.10	0.00	0.00	0.00	0.00	0.00	0.00	0.00	0.13	0.00	0.00	0.00	0.00	0.02	0.01	>0.999999999999999	2.2
Q9NR09	Baculoviral IAP repeat-containing protein 6 OS=Homo sapiens GN=BIRC6 PE=1 SV=2	0.24	0.00	0.00	0.00	0.00	0.00	0.00	0.00	0.04	0.00	0.00	0.00	0.00	0.00	0.00	0.00	0.00	0.00	0.00	0.00	0.00	0.00	0.00	0.00	0.02	0.00	>0.999999999999999	#DIV/0!
P48426	Phosphatidylinositol-5-phosphate 4-kinase type-2 alpha OS=Homo sapiens GN=PIP4K2A PI	0.00	0.00	0.00	0.00	0.00	0.00	0.00	0.00	0.28	0.00	0.00	0.00	0.00	0.00	0.00	0.00	0.00	0.00	0.00	0.00	0.00	0.00	0.00	0.00	0.02	0.00	>0.999999999999999	#DIV/0!
Q8N3C0	Activating signal cointegrator 1 complex subunit 3 OS=Homo sapiens GN=ASCC3 PE=1 SV	0.24	0.00	0.00	0.00	0.00	0.00	0.00	0.00	0.00	0.00	0.00	0.00	0.00	0.00	0.00	0.00	0.00	0.00	0.00	0.00	0.00	0.00	0.00	0.00	0.02	0.00	>0.999999999999999	#DIV/0!
Q5THJ4	Vacuolar protein sorting-associated protein 13D OS=Homo sapiens GN=VPS13D PE=1 SV=	0.24	0.00	0.00	0.00	0.00	0.00	0.00	0.00	0.00	0.00	0.00	0.00	0.00	0.00	0.00	0.00	0.00	0.00	0.00	0.00	0.00	0.00	0.00	0.00	0.02	0.00	>0.999999999999999	#DIV/0!
Q9Y3S1	Serine/threonine-protein kinase WNK2 OS=Homo sapiens GN=WNK2 PE=1 SV=4	0.24	0.00	0.00	0.00	0.00	0.00	0.00	0.00	0.00	0.00	0.00	0.00	0.00	0.00	0.00	0.00	0.00	0.00	0.00	0.00	0.00	0.00	0.00	0.00	0.02	0.00	>0.999999999999999	#DIV/0!
P42858	Huntingtin OS=Homo sapiens GN=HTT PE=1 SV=2	0.24	0.00	0.00	0.00	0.00	0.00	0.00	0.00	0.00	0.00	0.00	0.00	0.00	0.00	0.00	0.00	0.00	0.00	0.00	0.00	0.00	0.00	0.00	0.00	0.02	0.00	>0.999999999999999	#DIV/0!
Q8WXX0	Dynein heavy chain 7, axonemal OS=Homo sapiens GN=DNAH7 PE=1 SV=2	0.24	0.00	0.00	0.00	0.00	0.00	0.00	0.00	0.00	0.00	0.00	0.00	0.00	0.00	0.00	0.00	0.00	0.00	0.00	0.00	0.00	0.00	0.00	0.00	0.02	0.00	>0.999999999999999	#DIV/0!
P52789	Hexokinase-2 OS=Homo sapiens GN=HK2 PE=1 SV=2	0.00	0.00	0.00	0.00	0.00	0.00	0.00	0.00	0.24	0.00	0.00	0.00	0.00	0.60	0.00	0.21	0.00	0.46	0.00	0.39	0.00	0.00	0.00	0.34	0.02	0.17	>0.999999999999999	0.1
Q92575	UBX domain-containing protein 4 OS=Homo sapiens GN=UBXN4 PE=1 SV=2	0.00	0.00	0.00	0.00	0.00	0.00	0.00	0.00	0.24	0.00	0.00	0.00	0.00	0.00	0.00	0.00	0.00	0.00	0.00	0.00	0.00	0.00	0.00	0.00	0.02	0.00	>0.999999999999999	#DIV/0!
Q15208	Serine/threonine-protein kinase 38 OS=Homo sapiens GN=STK38 PE=1 SV=1	0.00	0.00	0.00	0.00	0.00	0.00	0.00	0.00	0.24	0.00	0.00	0.00	0.00	0.00	0.00	0.00	0.00	0.00	0.00	0.00	0.18	0.00	0.00	0.00	0.02	0.02	>0.999999999999999	1.3
O14879	Interferon-induced protein with tetratricopeptide repeats 3 OS=Homo sapiens GN=IFIT3 PE=	0.00	0.00	0.00	0.00	0.00	0.00	0.00	0.00	0.24	0.00	0.00	0.00	0.00	0.00	0.00	0.00	0.00	0.00	0.00	0.00	0.00	0.00	0.00	0.00	0.02	0.00	>0.999999999999999	#DIV/0!
P16870	Carboxypeptidase E OS=Homo sapiens GN=CPE PE=1 SV=1	0.00	0.00	0.00	0.00	0.00	0.00	0.00	0.00	0.24	0.00	0.00	0.00	0.00	0.00	0.00	0.00	0.00	0.00	0.00	0.00	0.18	0.00	0.92	0.34	0.02	0.12	>0.999999999999999	0.2
Q9UNF0	Protein kinase C and casein kinase substrate in neurons protein 2 OS=Homo sapiens GN=F	0.00	0.00	0.00	0.00	0.00	0.00	0.00	0.00	0.24	0.00	0.00	0.00	0.00	1.21	0.00	0.00	0.00	0.00	0.00	0.00	0.00	0.00	0.00	0.00	0.02	0.10	>0.999999999999999	0.2
Q8IZF6	Probable G-protein coupled receptor 112 OS=Homo sapiens GN=GPR112 PE=2 SV=2	0.00	0.23	0.00	0.00	0.00	0.00	0.00	0.00	0.00	0.00	0.00	0.00	0.00	0.00	0.00	0.00	0.00	0.00	0.00	0.00	0.00	0.00	0.00	0.00	0.02	0.00	>0.999999999999999	#DIV/0!
Q9UKL3	CASP8-associated protein 2 OS=Homo sapiens GN=CASP8AP2 PE=1 SV=1	0.00	0.23	0.00	0.00	0.00	0.00	0.00	0.00	0.00	0.00	0.00	0.00	0.00	0.00	0.00	0.00	0.00	0.00	0.00	0.00	0.00	0.00	0.00	0.00	0.02	0.00	>0.999999999999999	#DIV/0!
Q9C0C9	Ubiquitin-conjugating enzyme E2 O OS=Homo sapiens GN=UBE2O PE=1 SV=3	0.00	0.00	0.22	0.00	0.00	0.00	0.00	0.00	0.00	0.00	0.00	0.00	0.00	0.00	0.00	0.00	0.00	0.00	0.00	0.00	0.00	0.00	0.00	0.00	0.02	0.00	>0.999999999999999	#DIV/0!
Q8IZX4	Transcription initiation factor TFIID subunit 1-like OS=Homo sapiens GN=TAF1L PE=1 SV=1	0.00	0.00	0.22	0.00	0.00	0.00	0.00	0.00	0.00	0.00	0.00	0.00	0.00	0.00	0.00	0.00	0.00	0.00	0.00	0.00	0.00	0.00	0.00	0.00	0.02	0.00	>0.999999999999999	#DIV/0!
Q02817	Mucin-2 OS=Homo sapiens GN=MUC2 PE=1 SV=2	0.00	0.00	0.00	0.00	0.00	0.00	0.00	0.00	0.00	0.21	0.00	0.00	0.00	0.00	0.00	0.00	0.00	0.00	1.11	0.00	0.00	0.00	0.00	0.00	0.02	0.09	>0.999999999999999	0.2
Q9UQE7	Structural maintenance of chromosomes protein 3 OS=Homo sapiens GN=SMC3 PE=1 SV=	0.00	0.00	0.00	0.00	0.00	0.00	0.00	0.00	0.00	0.21	0.00	0.00	0.00	0.00	0.00	0.00	0.00	0.00	0.00	0.00	0.00	0.00	0.00	0.00	0.02	0.00	>0.999999999999999	#DIV/0!
Q52LW3	Rho GTPase-activating protein 29 OS=Homo sapiens GN=ARHGAP29 PE=1 SV=2	0.00	0.00	0.00	0.00	0.00	0.00	0.00	0.00	0.00	0.21	0.00	0.00	0.00	0.00	0.00	0.00	0.00	0.00	0.00	0.00	0.00	0.00	0.00	0.00	0.02	0.00	>0.999999999999999	#DIV/0!
Q12913	Receptor-type tyrosine-protein phosphatase eta OS=Homo sapiens GN=PTPRJ PE=1 SV=3	0.00	0.00	0.00	0.00	0.00	0.00	0.00	0.00	0.00	0.21	0.00	0.00	0.00	0.00	0.00	0.00	0.00	0.00	0.00	0.00	0.00	0.00	0.00	0.00	0.02	0.00	>0.999999999999999	#DIV/0!
A6NCI4	von Willebrand factor A domain-containing protein 3A OS=Homo sapiens GN=VWA3A PE=2	0.00	0.00	0.00	0.00	0.00	0.00	0.00	0.00	0.00	0.21	0.00	0.00	0.00	0.00	0.00	0.00	0.00	0.00	0.00	0.00	0.00	0.00	0.00	0.00	0.02	0.00	>0.999999999999999	#DIV/0!
P52701	DNA mismatch repair protein Msh6 OS=Homo sapiens GN=MSH6 PE=1 SV=2	0.00	0.00	0.00	0.00	0.00	0.00	0.00	0.00	0.00	0.00	0.00	0.20	0.00	0.00	0.00	0.00	0.00	0.00	0.00	0.00	0.00	0.00	0.00	0.00	0.02	0.00	>0.999999999999999	#DIV/0!
O60271	C-Jun-amino-terminal kinase-interacting protein 4 OS=Homo sapiens GN=SPAG9 PE=1 SV=	0.00	0.00	0.00	0.00	0.00	0.00	0.00	0.00	0.00	0.00	0.00	0.20	0.00	0.40	0.00	0.00	0.00	0.00	0.00	0.00	0.00	0.00	0.00	0.00	0.02	0.03	>0.999999999999999	0.5
Q96PP8	Guanylate-binding protein 5 OS=Homo sapiens GN=GBP5 PE=2 SV=1	0.00	0.00	0.00	0.00	0.00	0.00	0.00	0.00	0.20	0.00	0.00	0.00	0.00	0.00	0.00	0.00	0.00	0.00	0.00	0.00	0.00	0.00	0.00	0.00	0.02	0.00	>0.999999999999999	#DIV/0!
Q86X55	Histone-arginine methyltransferase CARM1 OS=Homo sapiens GN=CARM1 PE=1 SV=3	0.00	0.00	0.00	0.00	0.00	0.00	0.00	0.00	0.20	0.00	0.00	0.00	0.00	0.00	0.00	0.00	0.00	0.00	0.00	0.00	0.00	0.00	0.00	0.00	0.02	0.00	>0.999999999999999	#DIV/0!
Q9UKG9	Peroxisomal carnitine O-octanoyltransferase OS=Homo sapiens GN=CROT PE=1 SV=2	0.00	0.00	0.00	0.00	0.00	0.00	0.00	0.00	0.20	0.00	0.00	0.00	0.00	0.00	0.00	0.00	0.00	0.00	0.00	0.00	0.00	0.00	0.00	0.00	0.02	0.00	>0.999999999999999	#DIV/0!
O95340	Bifunctional 3'-phosphoadenosine 5'-phosphosulfate synthase 2 OS=Homo sapiens GN=PA	0.00	0.00	0.00	0.00	0.00	0.00	0.00	0.00	0.20	0.00	0.00	0.00	0.00	0.00	0.00	0.00	0.00	0.00	0.00	0.00	0.00	0.00	0.00	0.00	0.02	0.00	>0.999999999999999	#DIV/0!
Q8TF72	Protein Shroom3 OS=Homo sapiens GN=SHROOM3 PE=1 SV=2	0.00	0.00	0.00	0.00	0.00	0.00	0.00	0.00	0.00	0.00	0.19	0.00	0.00	0.00	0.00	0.07	0.19	0.00	0.00	0.00	0.00	0.00	0.00	0.00	0.02	0.02	>0.999999999999999	0.7
A6NHR9	Structural maintenance of chromosomes flexible hinge domain-containing protein 1 OS=Hon	0.00	0.00	0.00	0.00	0.00	0.00	0.00	0.00	0.00	0.00	0.19	0.00	0.00	0.00	0.00	0.00	0.00	0.00	0.00	0.00	0.00	0.00	0.00	0.00	0.02	0.00	>0.999999999999999	#DIV/0!
P29728	2'-5'-oligoadenylate synthase 2 OS=Homo sapiens GN=OAS2 PE=1 SV=3	0.00	0.00	0.00	0.00	0.00	0.00	0.00	0.00	0.16	0.00	0.00	0.00	0.00	0.00	0.00	0.00	0.00	0.00	0.00	0.00	0.00	0.00	0.00	0.00	0.01	0.00	>0.999999999999999	#DIV/0!
Q96PP9	Guanylate-binding protein 4 OS=Homo sapiens GN=GBP4 PE=1 SV=2	0.00	0.00	0.00	0.00	0.00	0.00	0.00	0.00	0.16	0.00	0.00	0.00	0.00	0.00	0.00	0.00	0.00	0.00	0.00	0.00	0.00	0.00	0.00	0.00	0.01	0.00	>0.999999999999999	#DIV/0!
Q96QF7	Acidic repeat-containing protein OS=Homo sapiens GN=ACRC PE=2 SV=1	0.00	0.00	0.00	0.00	0.00	0.00	0.00	0.00	0.16	0.00	0.00	0.00	0.00	0.00	0.00	0.00	0.00	0.00	0.00	0.00	0.00	0.00	0.00	0.00	0.01	0.00	>0.999999999999999	#DIV/0!
O14672	Disintegrin and metalloproteinase domain-containing protein 10 OS=Homo sapiens GN=ADA	0.00	0.00	0.00	0.00	0.00	0.00	0.00	0.00	0.16	0.00	0.00	0.00	0.00	0.00	0.00	0.00	0.00	0.00	0.00	0.00	0.00	0.00	0.00	0.00	0.01	0.00	>0.999999999999999	#DIV/0!
Q96KG9	N-terminal kinase-like protein OS=Homo sapiens GN=SCYL1 PE=1 SV=1	0.00	0.00	0.00	0.00	0.00	0.00	0.00	0.00	0.16	0.00	0.00	0.00	0.00	0.00	0.00	0.00	0.00	0.00	0.00	0.52	0.00	0.00	0.00	0.23	0.01	0.06	>0.999999999999999	0.2
P11277	Spectrin beta chain, erythrocyte OS=Homo sapiens GN=SPTB PE=1 SV=5	0.00	0.00	0.00	0.15	0.00	0.00	0.00	0.00	0.00	0.00	0.00	0.00	0.00	0.00	0.00	0.00	0.00	0.00	0.00	0.00	0.00	0.00	0.00	0.00	0.01	0.00	>0.999999999999999	#DIV/0!
Q14669	Probable E3 ubiquitin-protein ligase TRIP12 OS=Homo sapiens GN=TRIP12 PE=1 SV=1	0.00	0.00	0.00	0.00	0.00	0.15	0.00	0.00	0.00	0.00	0.00	0.00	0.00	0.00	0.00	0.00	0.00	0.00	0.00	0.00	0.00	0.00	0.00	0.00	0.01	0.00	>0.999999999999999	#DIV/0!
Q99102	Mucin-4 OS=Homo sapiens GN=MUC4 PE=1 SV=4	0.00	0.00	0.00	0.00	0.00	0.15	0.00	0.00	0.00	0.00	0.00	0.00	0.00	0.00	0.00	0.00	0.00	0.00	0.00	0.13	0.00	0.00	0.00	0.06	0.01	0.02	>0.999999999999999	0.8
Q8IWI9	MAX gene-associated protein OS=Homo sapiens GN=MGA PE=1 SV=2	0.00	0.00	0.00	0.00	0.00	0.15	0.00	0.00	0.00	0.00	0.00	0.00	0.00	0.00	0.00	0.00	0.00	0.00	0.00	0.00	0.00	0.00	0.00	0.00	0.01	0.00	>0.999999999999999	#DIV/0!
O14787	Transportin-2 OS=Homo sapiens GN=TNPO2 PE=1 SV=3	0.00	0.00	0.00	0.00	0.00	0.00	0.00	0.00	0.12	0.00	0.00	0.00	0.00	0.00	0.00	0.00	0.00	0.00	0.00	0.00	0.00	0.00	0.00	0.00	0.01	0.00	>0.999999999999999	#DIV/0!
Q9ULE0	Protein WWC3 OS=Homo sapiens GN=WWC3 PE=2 SV=3	0.00	0.00	0.00	0.00	0.00	0.00	0.00	0.00	0.12	0.00	0.00	0.00	0.00	0.00	0.00	0.00	0.00	0.00	0.00	0.00	0.00	0.00	0.00	0.00	0.01	0.00	>0.999999999999999	#DIV/0!
P08519	Apolipoprotein(a) OS=Homo sapiens GN=LPA PE=1 SV=1	0.00	0.00	0.11	0.00	0.00	0.00	0.00	0.00	0.00	0.00	0.00	0.00	0.00	0.20	0.11	0.00	0.00	0.00	0.00	0.00	0.00	0.00	0.00	0.00	0.01	0.03	>0.999999999999999	0.4
Q92614	Unconventional myosin-XVIIIa OS=Homo sapiens GN=MYO18A PE=1 SV=3	0.00	0.00	0.00	0.00	0.00	0.00	0.00	0.00	0.10	0.00	0.00	0.00	0.00	0.00	0.00	0.00	0.00	0.00	0.00	0.00	0.00	0.00	0.00	0.00	0.01	0.00	>0.999999999999999	#DIV/0!
Q9NZM1	Myoferlin OS=Homo sapiens GN=MYOF PE=1 SV=1	0.00	0.00	0.00	0.00	0.00	0.00	0.00	0.00	0.10	0.00	0.00	0.00	0.00	0.00	0.00	0.00	0.00	0.00	0.00	0.00	0.00	0.00	0.00	0.00	0.01	0.00	>0.999999999999999	#DIV/0!
Q8N3K9	Cardiomyopathy-associated protein 5 OS=Homo sapiens GN=CMYA5 PE=1 SV=3	0.00	0.00	0.00	0.00	0.00	0.00	0.00	0.00	0.00	0.00	0.00	0.10	0.00	0.00	0.00	0.00	0.00	0.00	0.00	0.00	0.00	0.00	0.00	0.00	0.01	0.00	>0.999999999999999	#DIV/0!
Q12768	WASH complex subunit strumpellin OS=Homo sapiens GN=KIAA0196 PE=1 SV=1	0.00	0.00	0.00	0.00	0.00	0.00	0.00	0.00	0.08	0.00	0.00	0.00	0.00	0.00	0.00	0.00	0.00	0.00	0.00	0.00	0.00	0.00	0.00	0.00	0.01	0.00	>0.999999999999999	#DIV/0!
Q9Y3R5	Protein dopey-2 OS=Homo sapiens GN=DOPEY2 PE=1 SV=5	0.00	0.00	0.00	0.00	0.00	0.00	0.00	0.00	0.04	0.00	0.00	0.00	0.00	0.00	0.00	0.00	0.00	0.00	0.00	0.00	0.00	0.00	0.00	0.00	0.00	0.00	>0.999999999999999	#DIV/0!
Q13085	Acetyl-CoA carboxylase 1 OS=Homo sapiens GN=ACACA PE=1 SV=2	0.00	0.00	0.00	0.00	0.00	0.00	0.00	0.00	0.04	0.00	0.00	0.00	0.00	0.00	0.00	0.00	0.00	0.00	0.00	0.00	0.00	0.00	0.00	0.00	0.00	0.00	>0.999999999999999	#DIV/0!
O14602	Eukaryotic translation initiation factor 1A, Y-chromosomal OS=Homo sapiens GN=EIF1AY P	0.00	0.00	0.00	0.00	0.00	0.00	0.00	0.00	0.00	0.00	0.00	0.00	0.00	0.00	0.00	0.00	0.00	0.00	0.00	2.59	0.00	0.00	0.00	0.00	0.00	0.22	>0.999999999999999	0.0
Q92597	Protein NDRG1 OS=Homo sapiens GN=NDRG1 PE=1 SV=1	0.00	0.00	0.00	0.00	0.00	0.00	0.00	0.00	0.00	0.00	0.00	0.00	0.00	1.61	0.00	0.00	0.00	0.00	0.00	0.00	0.00	0.00	0.00	0.45	0.00	0.17	>0.999999999999999	0.0
Q9Y4E8	Ubiquitin carboxyl-terminal hydrolase 15 OS=Homo sapiens GN=USP15 PE=1 SV=3	0.00	0.00	0.00	0.00	0.00	0.00	0.00	0.00	0.00	0.00	0.00	0.00	0.00	0.00	0.00	0.00	0.00	0.46	0.00	0.00	0.09	1.25	0.00	0.00	0.00	0.15	>0.999999999999999	0.0
P00488	Coagulation factor XIII A chain OS=Homo sapiens GN=F13A1 PE=1 SV=4	0.00	0.00	0.00	0.00	0.00	0.00	0.00	0.00	0.00	0.00	0.00	0.00	0.00	0.00	0.00	0.00	0.00	0.00	0.00	0.00	0.00	0.83	0.00	0.00	0.00	0.07	>0.999999999999999	0.0
Q9HA64	Ketosamine-3-kinase OS=Homo sapiens GN=FN3KRP PE=1 SV=2	0.00	0.00	0.00	0.00	0.00	0.00	0.00	0.00	0.00	0.00	0.00	0.00	0.00	0.00	0.00	0.69	0.00	0.00	0.00	0.00	0.00	0.00	0.00	0.00	0.00	0.06	>0.999999999999999	0.0
O00507	Probable ubiquitin carboxyl-terminal hydrolase FAF-Y OS=Homo sapiens GN=USP9Y PE=1	0.00	0.00	0.00	0.00	0.00	0.00	0.00	0.00	0.00	0.00	0.00	0.00	0.00	0.00	0.00	0.00	0.00	0.00	0.00	0.03	0.00	0.00	0.00	0.00	0.00	0.00	>0.999999999999999	0.0
O43278	Kunitz-type protease inhibitor 1 OS=Homo sapiens GN=SPINT1 PE=1 SV=2	0.00	0.00	0.00	0.00	0.00	0.00	0.00	0.00	0.00	0.00	0.00	0.00	0.00	0.00	0.56	0.34	0.00	0.00	0.00	0.65	0.00	0.00	0.00	0.00	0.00	0.13	>0.999999999999999	0.0
Q9C0F3	Zinc finger protein 436 OS=Homo sapiens GN=ZNF436 PE=2 SV=2	0.00	0.00	0.00	0.00	0.00	0.00	0.00	0.00	0.00	0.00	0.00	0.00	0.00	1.21	0.00	0.00	0.00	0.00	0.00	0.00	0.00	1.25	0.00	0.00	0.00	0.20	>0.999999999999999	0.0
Q02750	Dual specificity mitogen-activated protein kinase kinase 1 OS=Homo sapiens GN=MAP2K1 I	0.00	0.00	0.00	0.00	0.00	0.00	0.00	0.00	0.00	0.00	0.00	0.00	0.00	0.00	0.00	0.00	0.00	0.00	0.00	0.00	0.00	1.66	0.00	0.00	0.00	0.14	>0.999999999999999	0.0
P17096	High mobility group protein HMG-I/HMG-Y OS=Homo sapiens GN=HMGA1 PE=1 SV=3	0.00	0.00	0.00	0.00	0.00	0.00	0.00	0.00	0.00	0.00	0.00	0.00	0.00	0.00	0.00	0.00	0.00	0.00	0.00	3.89	0.00	0.00	0.00	0.00	0.00	0.32	>0.999999999999999	0.0
P30613	Pyruvate kinase isozymes R/L OS=Homo sapiens GN=PKLR PE=1 SV=2	0.00	0.00	0.00	0.00	0.00	0.00	0.00	0.00	0.00	0.00	0.00	0.00	0.00	2.22	0.00	0.00	0.00	0.00	0.00	0.00	0.00	0.00	0.00	0.00	0.00	0.18	>0.999999999999999	0.0
Q9UHX1	Poly(U)-binding-splicing factor PUF60 OS=Homo sapiens GN=PUF60 PE=1 SV=1	0.00	0.00	0.00	0.00	0.00	0.00	0.00	0.00	0.00	0.00	0.00	0.00	0.00	0.00	0.00	0.00	0.00	0.00	0.00	0.65	0.00	0.00	0.00	0.00	0.00	0.05	>0.999999999999999	0.0
Q8TD33	Secretoglobin family 1C member 1 OS=Homo sapiens GN=SCGB1C1 PE=2 SV=2	0.00	0.00	0.00	0.00	0.00	0.00	0.00	0.00	0.00	0.00	0.00	0.00	2.89	0.00	0.00	0.00	6.22	0.00	0.00	0.00	0.00	0.00	0.00	0.00	0.00	0.76	>0.999999999999999	0.0
P34949	Mannose-6-phosphate isomerase OS=Homo sapiens GN=MPI PE=1 SV=2	0.00	0.00	0.00	0.00	0.00	0.00	0.00	0.00	0.00	0.00	0.00	0.00	0.00	0.00	0.00	0.00	0.00	1.07	0.00	0.00	0.00	0.00	0.00	0.40	0.00	0.12	>0.999999999999999	0.0
P78318	Immunoglobulin-binding protein 1 OS=Homo sapiens GN=IGBP1 PE=1 SV=1	0.00	0.00	0.00	0.00	0.00	0.00	0.00	0.00	0.00	0.00	0.00	0.00	0.00	1.61	0.00	0.00	0.00	0.00	0.00	0.00	0.00	0.00	0.00	0.00	0.00	0.13	>0.999999999999999	0.0
Q9UJT2	Testis-specific serine kinase substrate OS=Homo sapiens GN=TSKS PE=1 SV=3	0.00	0.00	0.00	0.00	0.00	0.00	0.00	0.00	0.00	0.00	0.00	0.00	0.00	0.00	0.00	0.00	0.00	0.00	0.00	0.00	0.00	0.00	0.00	0.28	0.00	0.02	>0.999999999999999	0.0
Q5JNZ5	Putative 40S ribosomal protein S26-like 1 OS=Homo sapiens GN=RPS26P11 PE=5 SV=1	0.00	0.00	0.00	0.00	0.00	0.00	0.00	0.00	0.00	0.00	0.00	0.00	0.00	5.24	0.00	0.00	0.00	0.00	0.00	0.00	0.00	0.00	0.00	0.00	0.00	0.44	>0.999999999999999	0.0
Q8N7X1	RNA-binding motif protein, X-linked-like-3 OS=Homo sapiens GN=RBMXL3 PE=2 SV=2	0.00	0.00	0.00	0.00	0.00	0.00	0.00	0.00	0.00	0.00	0.00	0.00	0.26	0.00	0.00	0.21	0.00	0.00	0.00	0.00	0.00	0.00	0.00	0.17	0.00	0.05	>0.999999999999999	0.0
P61457	Pterin-4-alpha-carbinolamine dehydratase OS=Homo sapiens GN=PCBD1 PE=1 SV=2	0.00	0.00	0.00	0.00	0.00	0.00	0.00	0.00	0.00	0.00	0.00	0.00	0.00	5.64	0.00	0.00	0.00	0.00	0.00	0.00	0.00	0.00	0.00	0.00	0.00	0.47	>0.999999999999999	0.0
Q3ZCW2	Galectin-related protein OS=Homo sapiens GN=LGALSL PE=1 SV=2	0.00	0.00	0.00	0.00	0.00	0.00	0.00	0.00	0.00	0.00	0.00	0.00	0.00	0.00	0.00	0.00	0.00	0.00	0.00	2.33	0.00	0.00	0.00	0.00	0.00	0.19	>0.999999999999999	0.0
O94973	AP-2 complex subunit alpha-2 OS=Homo sapiens GN=AP2A2 PE=1 SV=2	0.00	0.00	0.00	0.00	0.00	0.00	0.00	0.00	0.00	0.00	0.00	0.00	0.00	0.60	0.00	0.00	0.00	0.00	0.00	0.39	0.00	0.00	0.46	0.00	0.00	0.12	>0.999999999999999	0.0
Q8N668	COMM domain-containing protein 1 OS=Homo sapiens GN=COMMD1 PE=1 SV=1	0.00	0.00	0.00	0.00	0.00	0.00	0.00	0.00	0.00	0.00	0.00	0.00	0.00	0.00	0.00	0.00	0.00	0.00	0.00	2.07	0.00	0.00	0.00	0.00	0.00	0.17	>0.999999999999999	0.0
Q9UNN5	FAS-associated factor 1 OS=Homo sapiens GN=FAF1 PE=1 SV=2	0.00	0.00	0.00	0.00	0.00	0.00	0.00	0.00	0.00	0.00	0.00	0.00	0.00	0.00	0.00	0.00	0.00	0.00	0.00	0.52	0.00	0.00	0.00	0.00	0.00	0.04	>0.999999999999999	0.0
Q9UEY8	Gamma-adducin OS=Homo sapiens GN=ADD3 PE=1 SV=1	0.00	0.00	0.00	0.00	0.00	0.00	0.00	0.00	0.00	0.00	0.00	0.00	0.35	0.00	0.00	0.00	0.00	0.00	0.00	0.00	0.00	0.00	0.00	0.00	0.00	0.03	>0.999999999999999	0.0
P49407	Beta-arrestin-1 OS=Homo sapiens GN=ARRB1 PE=1 SV=2	0.00	0.00	0.00	0.00	0.00	0.00	0.00	0.00	0.00	0.00	0.00	0.00	0.00	0.00	0.00	0.00	0.00	0.00	0.00	0.91	0.00	0.00	0.00	0.00	0.00	0.08	>0.999999999999999	0.0

P08237	6-phosphofructokinase, muscle type OS=Homo sapiens GN=PFKM PE=1 SV=2	0.00	0.00	0.00	0.00	0.00	0.00	0.00	0.00	0.00	0.00	0.00	0.00	0.00	0.00	0.00	0.00	0.00	0.00	0.00	0.00	0.00	0.83	0.00	0.00	0.00	0.07	>0.999999999999999	0.0
Q9UJW0	Dynactin subunit 4 OS=Homo sapiens GN=DCTN4 PE=1 SV=1	0.00	0.00	0.00	0.00	0.00	0.00	0.00	0.00	0.00	0.00	0.00	0.00	0.00	1.21	0.00	0.00	0.00	0.00	0.00	0.00	0.00	0.00	0.00	0.00	0.00	0.10	>0.999999999999999	0.0
Q9UK45	U6 snRNA-associated Sm-like protein LSm7 OS=Homo sapiens GN=LSM7 PE=1 SV=1	0.00	0.00	0.00	0.00	0.00	0.00	0.00	0.00	0.00	0.00	0.00	0.00	0.00	0.00	0.00	0.00	0.00	0.00	0.00	0.00	0.00	0.00	4.60	0.00	0.00	0.38	>0.999999999999999	0.0
Q7LBR1	Charged multivesicular body protein 1b OS=Homo sapiens GN=CHMP1B PE=1 SV=1	0.00	0.00	0.00	0.00	0.00	0.00	0.00	0.00	0.00	0.00	0.00	0.00	0.00	3.02	0.00	0.00	0.00	0.00	0.00	0.00	0.00	0.00	0.00	0.00	0.00	0.25	>0.999999999999999	0.0
Q7L190	Developmental pluripotency-associated protein 4 OS=Homo sapiens GN=DPPA4 PE=1 SV=	0.00	0.00	0.00	0.00	0.00	0.00	0.00	0.00	0.00	0.00	0.00	0.00	0.00	0.00	0.00	0.00	0.00	0.00	0.00	0.00	0.00	0.00	0.00	0.57	0.00	0.05	>0.999999999999999	0.0
Q9UL36	Zinc finger protein 236 OS=Homo sapiens GN=ZNF236 PE=2 SV=2	0.00	0.00	0.00	0.00	0.00	0.00	0.00	0.00	0.00	0.00	0.00	0.00	0.00	0.00	0.00	0.14	0.00	0.31	0.00	0.00	0.00	0.00	0.00	0.00	0.00	0.04	>0.999999999999999	0.0
Q8NGK0	Olfactory receptor 51G2 OS=Homo sapiens GN=OR51G2 PE=2 SV=1	0.00	0.00	0.00	0.00	0.00	0.00	0.00	0.00	0.00	0.00	0.00	0.00	0.00	1.81	0.00	0.00	0.00	0.00	0.00	0.00	0.00	0.00	0.00	0.00	0.00	0.15	>0.999999999999999	0.0
Q9H1R3	Myosin light chain kinase 2, skeletal/cardiac muscle OS=Homo sapiens GN=MYLK2 PE=1 S	0.00	0.00	0.00	0.00	0.00	0.00	0.00	0.00	0.00	0.00	0.00	0.00	0.00	0.00	0.00	0.00	0.00	0.00	0.00	0.00	0.00	1.04	0.00	0.00	0.00	0.09	>0.999999999999999	0.0
Q9NZC9	SWI/SNF-related matrix-associated actin-dependent regulator of chromatin subfamily A-like	0.00	0.00	0.00	0.00	0.00	0.00	0.00	0.00	0.00	0.00	0.00	0.00	0.00	0.60	0.00	0.00	0.00	0.00	0.00	0.00	0.00	0.00	0.00	0.00	0.00	0.05	>0.999999999999999	0.0
O00442	RNA 3'-terminal phosphate cyclase OS=Homo sapiens GN=RTCD1 PE=1 SV=1	0.00	0.00	0.00	0.00	0.00	0.00	0.00	0.00	0.00	0.00	0.00	0.00	0.70	0.00	0.00	0.00	0.00	0.00	0.00	1.04	0.00	0.00	0.00	0.00	0.00	0.14	>0.999999999999999	0.0
Q5T2P8	Annexin A8-like protein 1 OS=Homo sapiens GN=ANXA8L1 PE=2 SV=1	0.00	0.00	0.00	0.00	0.00	0.00	0.00	0.00	0.00	0.00	0.00	0.00	0.00	0.00	0.00	0.00	0.00	0.00	1.43	0.00	0.00	0.00	0.00	0.00	0.00	0.12	>0.999999999999999	0.0
P61020	Ras-related protein Rab-5B OS=Homo sapiens GN=RAB5B PE=1 SV=1	0.00	0.00	0.00	0.00	0.00	0.00	0.00	0.00	0.00	0.00	0.00	0.00	0.00	6.04	0.00	0.00	0.00	0.00	0.00	0.00	0.00	9.97	0.00	0.00	0.00	1.33	>0.999999999999999	0.0
O14974	Protein phosphatase 1 regulatory subunit 12A OS=Homo sapiens GN=PPP1R12A PE=1 SV	0.00	0.00	0.00	0.00	0.00	0.00	0.00	0.00	0.00	0.00	0.00	0.00	0.00	0.00	0.00	0.00	0.00	0.00	0.00	0.00	0.00	0.62	0.46	0.17	0.00	0.10	>0.999999999999999	0.0
P29373	Cellular retinoic acid-binding protein 2 OS=Homo sapiens GN=CRABP2 PE=1 SV=2	0.00	0.00	0.00	0.00	0.00	0.00	0.00	0.00	0.00	0.00	0.00	0.00	0.00	4.23	0.00	0.00	0.00	0.00	0.00	0.00	0.00	4.36	0.00	0.00	0.00	0.72	>0.999999999999999	0.0
B5MCN3	Putative SEC14-like protein 6 OS=Homo sapiens GN=SEC14L6 PE=5 SV=1	0.00	0.00	0.00	0.00	0.00	0.00	0.00	0.00	0.00	0.00	0.00	0.00	0.00	1.41	0.00	0.00	0.00	0.00	0.00	0.00	0.00	0.00	0.00	0.00	0.00	0.12	>0.999999999999999	0.0
Q8NBS9	Thioredoxin domain-containing protein 5 OS=Homo sapiens GN=TXNDC5 PE=1 SV=2	0.00	0.00	0.00	0.00	0.00	0.00	0.00	0.00	0.00	0.00	0.00	0.00	0.00	0.00	0.00	0.00	0.00	0.00	0.00	0.91	0.22	0.00	0.00	0.40	0.00	0.13	>0.999999999999999	0.0
P07305	Histone H1.0 OS=Homo sapiens GN=H1F0 PE=1 SV=3	0.00	0.00	0.00	0.00	0.00	0.00	0.00	0.00	0.00	0.00	0.00	0.00	0.00	0.00	0.00	0.00	0.00	0.00	2.54	0.00	0.00	0.00	0.00	0.00	0.00	0.21	>0.999999999999999	0.0
Q00978	Interferon regulatory factor 9 OS=Homo sapiens GN=IRF9 PE=1 SV=1	0.00	0.00	0.00	0.00	0.00	0.00	0.00	0.00	0.00	0.00	0.00	0.00	0.00	0.00	0.00	0.00	0.00	1.07	0.00	0.00	0.00	0.00	0.00	0.00	0.00	0.09	>0.999999999999999	0.0
Q92572	AP-3 complex subunit sigma-1 OS=Homo sapiens GN=AP3S1 PE=1 SV=1	0.00	0.00	0.00	0.00	0.00	0.00	0.00	0.00	0.00	0.00	0.00	0.00	0.00	0.00	0.00	0.00	0.00	0.00	0.00	1.94	0.00	0.00	0.00	0.00	0.00	0.16	>0.999999999999999	0.0
Q9Y237	Peptidyl-prolyl cis-trans isomerase NIMA-interacting 4 OS=Homo sapiens GN=PIN4 PE=1 S'	0.00	0.00	0.00	0.00	0.00	0.00	0.00	0.00	0.00	0.00	0.00	0.00	0.00	0.00	0.00	0.00	0.00	0.00	0.00	0.00	0.00	5.19	0.00	0.00	0.00	0.43	>0.999999999999999	0.0
Q9UK99	F-box only protein 3 OS=Homo sapiens GN=FBXO3 PE=1 SV=3	0.00	0.00	0.00	0.00	0.00	0.00	0.00	0.00	0.00	0.00	0.00	0.00	0.00	0.00	0.00	0.41	0.00	0.00	0.00	0.78	0.00	0.00	0.00	0.00	0.00	0.10	>0.999999999999999	0.0
Q9Y6E2	Basic leucine zipper and W2 domain-containing protein 2 OS=Homo sapiens GN=BZW2 PE=	0.00	0.00	0.00	0.00	0.00	0.00	0.00	0.00	0.00	0.00	0.00	0.00	0.00	0.00	0.00	0.00	0.00	0.00	0.00	0.91	0.00	0.00	0.00	0.00	0.00	0.08	>0.999999999999999	0.0
Q92520	Protein FAM3C OS=Homo sapiens GN=FAM3C PE=1 SV=1	0.00	0.00	0.00	0.00	0.00	0.00	0.00	0.00	0.00	0.00	0.00	0.00	0.00	0.00	0.00	0.00	0.00	0.00	0.00	0.00	0.00	0.00	0.00	0.74	0.00	0.06	>0.999999999999999	0.0
P04066	Tissue alpha-L-fucosidase OS=Homo sapiens GN=FUCA1 PE=1 SV=4	0.00	0.00	0.00	0.00	0.00	0.00	0.00	0.00	0.00	0.00	0.00	0.00	0.00	2.62	0.00	0.00	0.00	0.00	0.00	1.68	0.00	1.25	0.00	0.34	0.00	0.49	>0.999999999999999	0.0
A6NMY6	Putative annexin A2-like protein OS=Homo sapiens GN=ANXA2P2 PE=5 SV=2	0.00	0.00	0.00	0.00	0.00	0.00	0.00	0.00	0.00	0.00	0.00	0.00	0.00	0.00	3.11	0.00	0.00	0.00	0.00	0.00	0.00	0.00	0.00	0.00	0.00	0.26	>0.999999999999999	0.0
Q9Y2T7	Y-box-binding protein 2 OS=Homo sapiens GN=YBX2 PE=1 SV=2	0.00	0.00	0.00	0.00	0.00	0.00	0.00	0.00	0.00	0.00	0.00	0.00	0.00	0.00	0.00	0.00	1.70	0.00	0.00	0.00	0.00	0.00	1.38	0.00	0.00	0.26	>0.999999999999999	0.0
Q8NI51	Transcriptional repressor CTCFL OS=Homo sapiens GN=CTCFL PE=1 SV=2	0.00	0.00	0.00	0.00	0.00	0.00	0.00	0.00	0.00	0.00	0.00	0.00	0.00	0.00	0.00	0.00	0.00	0.00	0.63	0.00	0.00	0.00	0.00	0.00	0.00	0.05	>0.999999999999999	0.0
Q8WU03	Glycine N-acyltransferase-like protein 2 OS=Homo sapiens GN=GLYATL2 PE=2 SV=1	0.00	0.00	0.00	0.00	0.00	0.00	0.00	0.00	0.00	0.00	0.00	0.00	0.00	0.00	1.11	0.00	0.00	0.00	0.00	0.00	0.00	0.00	0.00	0.00	0.00	0.09	>0.999999999999999	0.0
P67775	Serine/threonine-protein phosphatase 2A catalytic subunit alpha isoform OS=Homo sapiens	0.00	0.00	0.00	0.00	0.00	0.00	0.00	0.00	0.00	0.00	0.00	0.00	0.79	0.00	0.00	0.00	1.70	0.00	0.00	0.00	0.00	6.23	2.91	0.00	0.00	0.97	>0.999999999999999	0.0
P01764	Ig heavy chain V-III region VH26 OS=Homo sapiens PE=1 SV=1	0.00	0.00	0.00	0.00	0.00	0.00	0.00	0.00	0.00	0.00	0.00	0.00	0.00	0.00	0.00	0.00	11.50	0.00	0.00	0.00	0.00	0.00	9.36	0.00	0.00	1.74	>0.999999999999999	0.0
Q9H788	SH2 domain-containing protein 4A OS=Homo sapiens GN=SH2D4A PE=1 SV=1	0.00	0.00	0.00	0.00	0.00	0.00	0.00	0.00	0.00	0.00	0.00	0.00	0.00	0.00	0.00	0.00	0.00	0.00	0.00	0.78	0.00	0.00	0.00	0.00	0.00	0.06	>0.999999999999999	0.0
Q15582	Transforming growth factor-beta-induced protein ig-h3 OS=Homo sapiens GN=TGFBI PE=1	0.00	0.00	0.00	0.00	0.00	0.00	0.00	0.00	0.00	0.00	0.00	0.00	0.00	0.00	0.44	0.00	0.00	0.00	0.00	0.00	0.00	0.00	0.00	0.00	0.00	0.04	>0.999999999999999	0.0
P60033	CD81 antigen OS=Homo sapiens GN=CD81 PE=1 SV=1	0.00	0.00	0.00	0.00	0.00	0.00	0.00	0.00	0.00	0.00	0.00	0.00	0.00	0.00	1.44	0.00	0.00	0.00	0.00	0.00	0.00	0.00	0.00	0.00	0.00	0.12	>0.999999999999999	0.0
Q9Y2B0	Protein canopy homolog 2 OS=Homo sapiens GN=CNPY2 PE=1 SV=1	0.00	0.00	0.00	0.00	0.00	0.00	0.00	0.00	0.00	0.00	0.00	0.00	0.00	0.00	1.78	0.00	0.00	0.00	0.00	0.00	0.00	0.00	0.00	0.00	0.00	0.15	>0.999999999999999	0.0
Q9BWS9	Chitinase domain-containing protein 1 OS=Homo sapiens GN=CHID1 PE=1 SV=1	0.00	0.00	0.00	0.00	0.00	0.00	0.00	0.00	0.00	0.00	0.00	0.00	1.32	0.00	0.00	0.00	0.00	0.00	0.00	0.00	0.00	0.00	0.00	0.00	0.00	0.11	>0.999999999999999	0.0
O95571	Protein ETHE1, mitochondrial OS=Homo sapiens GN=ETHE1 PE=1 SV=2	0.00	0.00	0.00	0.00	0.00	0.00	0.00	0.00	0.00	0.00	0.00	0.00	1.05	0.00	0.00	0.83	0.00	1.84	0.00	0.00	0.00	0.00	0.00	0.00	0.00	0.31	>0.999999999999999	0.0
Q8TD47	40S ribosomal protein S4, Y isoform 2 OS=Homo sapiens GN=RPS4Y2 PE=1 SV=3	0.00	0.00	0.00	0.00	0.00	0.00	0.00	0.00	0.00	0.00	0.00	0.00	0.00	4.83	1.22	0.76	0.00	0.00	0.00	0.00	0.00	0.00	0.00	0.00	0.00	0.57	>0.999999999999999	0.0

Table S4. Young OC-enriched proteins

			Young			
			Average [emPAI]		Significance	Fold difference
No	Accession	Description	OC	ANC	-log (Adjusted P value)	OC/ANC
1	O60739	Eukaryotic translation initiation factor 1b	11.4	0.4	5.3	28.4
2	P54652	Heat shock-related 70 kDa protein 2	8.0	0.3	2.3	27.4
3	P62314	Small nuclear ribonucleoprotein Sm D1	8.5	0.5	2.6	17.8
4	Q8WYR4	Radial spoke head 1 homolog	8.5	1.0	2.2	8.8
5	P08758	Annexin A5	11.0	2.5	2.9	4.4
6	Q99417	C-Myc-binding protein	14.0	3.7	4.6	3.8
7	Q9Y3Z3	SAM domain and HD domain-containing protein 1	17.9	4.9	7.7	3.6
8	P63208	S-phase kinase-associated protein 1	25.2	7.1	>15	3.5
9	Q9NQR4	Omega-amidase NIT2	14.7	4.3	4.7	3.4
10	P00325	Alcohol dehydrogenase 1B	16.6	4.9	6.1	3.4
11	P02144	Myoglobin	11.9	3.5	2.8	3.4
12	P80723	Brain acid soluble protein 1	24.7	7.5	>15	3.3
13	P49458	Signal recognition particle 9 kDa protein	10.6	3.3	2.0	3.2
14	Q96NY7	Chloride intracellular channel protein 6	11.1	3.6	2.1	3.0
15	P62888	60S ribosomal protein L30	13.2	4.4	3.2	3.0
16	Q02878	60S ribosomal protein L6	9.6	3.3	1.3	2.9
17	Q13885	Tubulin beta-2A chain	19.9	7.1	7.5	2.8
18	Q9NQ48	Leucine zipper transcription factor-like protein 1	12.6	4.5	2.6	2.8
19	P07355	Annexin A2	16.9	6.2	5.0	2.7
20	Q9Y265	RuvB-like 1	14.1	5.3	3.2	2.7
21	P0CW22	40S ribosomal protein S17-like	11.4	4.2	1.9	2.7
22	Q71U36	Tubulin alpha-1A chain	34.5	12.9	>15	2.7
23	P60900	Proteasome subunit alpha type-6	12.0	4.5	2.1	2.6
24	P18085	ADP-ribosylation factor 4	10.7	4.1	1.5	2.6
25	P31150	Rab GDP dissociation inhibitor alpha	17.1	6.7	4.7	2.5
26	P09104	Gamma-enolase	11.3	4.6	1.6	2.5
27	P41567	Eukaryotic translation initiation factor 1	21.4	8.7	7.4	2.5
28	P51858	Hepatoma-derived growth factor	24.3	9.9	9.6	2.4
29	P36405	ADP-ribosylation factor-like protein 3	15.2	6.4	3.1	2.4
30	P05386	60S acidic ribosomal protein P1	11.8	5.0	1.6	2.3
31	Q9H4A4	Aminopeptidase B	12.3	5.3	1.7	2.3
32	P49773	Histidine triad nucleotide-binding protein 1	20.0	8.7	5.7	2.3
33	P61204	ADP-ribosylation factor 3	20.0	8.7	5.7	2.3
34	P10768	S-formylglutathione hydrolase	13.9	6.1	2.4	2.3
35	P30050	60S ribosomal protein L12	24.5	10.9	8.5	2.2
36	P62820	Ras-related protein Rab-1A	16.1	7.4	3.1	2.2
37	P30043	Flavin reductase (NADPH)	17.4	8.1	3.6	2.1
38	P68371	Tubulin beta-4B chain	22.9	10.8	6.6	2.1
39	Q99832	T-complex protein 1 subunit eta	13.0	6.3	1.5	2.1
40	P17987	T-complex protein 1 subunit alpha	17.3	8.6	3.0	2.0
41	O00764	Pyridoxal kinase	12.7	6.4	1.3	2.0
42	P00441	Superoxide dismutase [Cu-Zn]	38.2	19.2	>15	2.0
43	Q9BW30	Tubulin polymerization-promoting protein family member 3	39.6	20.1	>15	2.0
44	Q99497	Protein DJ-1	37.2	19.0	>15	2.0
45	P23396	40S ribosomal protein S3	17.9	9.4	3.0	1.9
46	P16050	Arachidonate 15-lipoxygenase	24.6	12.8	6.1	1.9
47	P06454	Prothymosin alpha	14.5	7.6	1.7	1.9
48	P07437	Tubulin beta chain	22.4	11.7	5.0	1.9
49	O60701	UDP-glucose 6-dehydrogenase	16.8	8.8	2.5	1.9
50	P25786	Proteasome subunit alpha type-1	13.7	7.2	1.4	1.9
51	P37802	Transgelin-2	25.6	13.8	6.2	1.9
52	P05388	60S acidic ribosomal protein P0	14.8	8.1	1.6	1.8
53	P22626	Heterogeneous nuclear ribonucleoproteins A2/B1	17.1	9.5	2.2	1.8
54	Q96KP4	Cytosolic non-specific dipeptidase	20.4	11.4	3.3	1.8
55	Q13228	Selenium-binding protein 1	22.7	12.9	4.1	1.8
56	P28838	Cytosol aminopeptidase	18.2	10.7	2.1	1.7
57	P55072	Transitional endoplasmic reticulum ATPase	18.3	10.8	2.1	1.7
58	P09211	Glutathione S-transferase P	70.3	42.1	>15	1.7
59	P00326	Alcohol dehydrogenase 1C	49.7	30.3	>15	1.6
60	O00151	PDZ and LIM domain protein 1	17.7	10.9	1.6	1.6
61	P50395	Rab GDP dissociation inhibitor beta	37.0	22.8	9.4	1.6
62	P04083	Annexin A1	43.4	26.9	13.1	1.6
63	P68366	Tubulin alpha-4A chain	19.6	12.2	2.1	1.6
64	P07900	Heat shock protein HSP 90-alpha	24.3	15.1	3.5	1.6
65	P10599	Thioredoxin	27.9	17.4	4.9	1.6
66	P05387	60S acidic ribosomal protein P2	51.0	32.0	>15	1.6
67	P29401	Transketolase	20.5	13.0	2.2	1.6
68	P68036	Ubiquitin-conjugating enzyme E2 L3	20.6	13.1	2.1	1.6
69	P14550	Alcohol dehydrogenase [NADP(+)]	35.1	22.8	6.9	1.5
70	P62258	14-3-3 protein epsilon	35.4	23.1	6.8	1.5
71	P06703	Protein S100-A6	27.2	17.8	3.7	1.5
72	P02652	Apolipoprotein A-II	32.3	21.3	5.4	1.5
73	P13489	Ribonuclease inhibitor	21.5	14.2	2.0	1.5

74	P40394	Alcohol dehydrogenase class 4 mu/sigma chain	28.5	18.9	3.9	1.5
75	P30044	Peroxiredoxin-5, mitochondrial	52.0	34.5	>15	1.5
76	P08263	Glutathione S-transferase A1	32.3	21.5	5.1	1.5
77	P30085	UMP-CMP kinase	20.3	13.6	1.6	1.5
78	P60981	Destrin	19.6	13.1	1.4	1.5
79	P32119	Peroxiredoxin-2	23.5	16.0	2.1	1.5
80	P08238	Heat shock protein HSP 90-beta	25.9	17.8	2.6	1.5
81	P04075	Fructose-bisphosphate aldolase A	48.5	33.5	10.5	1.4
82	P00352	Retinal dehydrogenase 1	80.3	55.9	>15	1.4
83	Q13938	Calcyphosin	50.8	36.0	10.2	1.4
84	P30041	Peroxiredoxin-6	30.2	21.7	3.0	1.4
85	P27348	14-3-3 protein theta	30.3	21.9	2.8	1.4
86	P60174	Triosephosphate isomerase	59.9	44.2	11.8	1.4
87	P31949	Protein S100-A11	43.7	32.4	5.7	1.3

Table S5. Elderly OC-enriched proteins

No	Accession	Description	Elderly			
			Average [emPAI]		Fold difference	
			OC	ANC	-log (Adjusted P value)	OC/ANC
1	P62805	Histone H4	23.3	4.4	1.5	5.3
2	Q71U36	Tubulin alpha-1A chain	27.5	5.9	2.3	4.6
3	P69905	Hemoglobin subunit alpha	98.7	29.6	15.0	3.3
4	P04083	Annexin A1	40.2	18.6	2.3	2.2
5	P02042	Hemoglobin subunit delta	38.9	18.5	1.9	2.1
6	P68871	Hemoglobin subunit beta	126.5	61.4	15.0	2.1
7	Q13938	Calcyphosin	45.0	23.7	2.2	1.9
8	P30044	Peroxiredoxin-5, mitochondrial	46.7	27.3	1.6	1.7
9	P60174	Triosephosphate isomerase	69.8	48.2	2.3	1.4
10	P01834	Ig kappa chain C region	274.5	198.4	15.0	1.4

Table S6. Age-associated OC proteins

Data from shotgun proteome							
			OC				
			Average [emPAI]		Significance	Fold difference	
No	Accession	Description	Elderly	Young	-log (Adjusted P value)	Elderly/Young	
1	P06702	Protein S100-A9	155.2	9.5	>15	16.4	
2	P05109	Protein S100-A8	221.8	14.5	>15	15.3	
3	P02042	Hemoglobin subunit delta	38.9	3.0	>15	12.9	
4	P62805	Histone H4	23.3	2.1	7.7	11.3	
5	P68871	Hemoglobin subunit beta	126.5	15.9	>15	8.0	
6	P69905	Hemoglobin subunit alpha	98.7	16.9	>15	5.8	
7	P02766	Transthyretin	17.6	4.7	1.9	3.7	
8	P01834	Ig kappa chain C region	274.5	102.6	>15	2.7	
9	P80188	Neutrophil gelatinase-associated lipocalin	28.4	14.5	2.4	2.0	
10	P02788	Lactotransferrin	51.0	29.8	7.7	1.7	
11	P07737	Profilin-1	44.8	27.3	4.8	1.6	
12	P60709	Actin, cytoplasmic 1	43.2	27.4	3.6	1.6	
13	O00299	Chloride intracellular channel protein 1	34.6	48.3	2.4	0.7	
14	P50395	Rab GDP dissociation inhibitor beta	24.8	37.0	1.6	0.7	
15	P14550	Alcohol dehydrogenase [NADP(+)]	23.2	35.1	1.5	0.7	
16	P04080	Cystatin-B	23.5	35.7	1.6	0.7	
17	P01040	Cystatin-A	32.3	49.4	4.4	0.7	
18	P30086	Phosphatidylethanolamine-binding protein 1	29.7	47.2	4.8	0.6	
19	Q99497	Protein DJ-1	23.3	37.2	2.4	0.6	
20	P09211	Glutathione S-transferase P	40.5	70.3	>15	0.6	

Table S7. Proteins associated with olfaction

			Young & Elderly (n=24)	
No		Protein	Spearman's Rho	*P* value
1	P13693	Translationally-controlled tumor protein	0.54	0.0061
2	Q14157	Ubiquitin-associated protein 2-like	0.53	0.0082
3	Q8TD33	Secretoglobin family 1C member 1	0.52	0.0085
4	P08185	Corticosteroid-binding globulin	0.50	0.0126
5	Q92597	Protein NDRG1	0.49	0.0152
6	P59827	BPI fold-containing family B member 4	0.49	0.0163
7	Q9UJU6	Drebrin-like protein	0.48	0.0187
8	Q99436	Proteasome subunit beta type-7	0.47	0.0196
9	P27216	Annexin A13	0.46	0.0223
10	Q9NQ39	Putative 40S ribosomal protein S10-like	0.46	0.0227
11	O95236	Apolipoprotein L3	0.46	0.0238
12	P35268	60S ribosomal protein L22	0.45	0.0282
13	P49458	Signal recognition particle 9 kDa protein	0.44	0.0326
14	P62158	Calmodulin	0.44	0.0327
15	P41567	Eukaryotic translation initiation factor 1	0.44	0.0332
16	Q99426	Tubulin-folding cofactor B	0.43	0.0339
17	Q6UWW0	Lipocalin-15	0.43	0.0349
18	Q9HB40	Retinoid-inducible serine carboxypeptidase	0.43	0.0364
19	P01040	Cystatin-A	0.42	0.0417
20	Q13838	Spliceosome RNA helicase DDX39B	0.42	0.0423
21	Q9UBI6	Guanine nucleotide-binding protein G(I)/G(S)/G(O) subunit gamma-12	0.42	0.0425
22	Q8NBS9	Thioredoxin domain-containing protein 5	0.42	0.0435
23	Q9Y5K3	Choline-phosphate cytidylyltransferase B	0.42	0.0436
24	O14791	Apolipoprotein L1	0.42	0.0437
25	P01019	Angiotensinogen	0.42	0.0437
26	P52597	Heterogeneous nuclear ribonucleoprotein F	0.41	0.0438
27	Q9H4A6	Golgi phosphoprotein 3	0.41	0.0447
28	P14174	Macrophage migration inhibitory factor	0.41	0.0463
29	Q9NWV4	UPF0587 protein C1orf123	0.41	0.0462
30	Q9NRV9	Heme-binding protein 1	0.41	0.0468
31	O00625	Pirin	0.40	0.0498
32	P35527	Keratin, type I cytoskeletal 9	-0.40	0.0499
33	O60256	Phosphoribosyl pyrophosphate synthase-associated protein 2	-0.41	0.0462
34	P63104	14-3-3 protein zeta/delta	-0.41	0.0457
35	P35908	Keratin, type II cytoskeletal 2 epidermal	-0.41	0.0455
36	P53609	Geranylgeranyl transferase type-1 subunit beta	-0.41	0.0455
37	P41218	Myeloid cell nuclear differentiation antigen	-0.41	0.0453
38	Q9NXR7	BRCA1-A complex subunit BRE	-0.41	0.0443
39	O15198	Mothers against decapentaplegic homolog 9	-0.41	0.0443
40	Q9NR19	Acetyl-coenzyme A synthetase, cytoplasmic	-0.41	0.0443
41	Q9H7C9	UPF0366 protein C11orf67	-0.41	0.0443
42	P38919	Eukaryotic initiation factor 4A-III	-0.41	0.0438
43	P52943	Cysteine-rich protein 2	-0.42	0.0435
44	P02538	Keratin, type II cytoskeletal 6A	-0.42	0.0434
45	P07996	Thrombospondin-1	-0.42	0.0417
46	P39880	Homeobox protein cut-like 1	-0.42	0.0417
47	Q96JH7	Deubiquitinating protein VCIP135	-0.42	0.0403
48	Q86XW9	Thioredoxin domain-containing protein 6	-0.42	0.0402
49	Q13546	Receptor-interacting serine/threonine-protein kinase 1	-0.42	0.0387
50	P15924	Desmoplakin	-0.43	0.0375
51	Q5JSH3	WD repeat-containing protein 44	-0.43	0.037
52	Q99615	DnaJ homolog subfamily C member 7	-0.43	0.0362
53	P06702	Protein S100-A9	-0.43	0.0348
54	Q9Y6I9	Testis-expressed sequence 264 protein	-0.44	0.0333
55	P52566	Rho GDP-dissociation inhibitor 2	-0.44	0.032
56	P04745	Alpha-amylase 1	-0.44	0.0296
57	P04083	Annexin A1	-0.45	0.0287
58	Q8N9W5	UPF0470 protein C19orf51	-0.45	0.026
59	P04434	Ig kappa chain V-III region VH (Fragment)	-0.46	0.0252
60	Q06033	Inter-alpha-trypsin inhibitor heavy chain H3	-0.46	0.0239
61	Q8IZP0	Abl interactor 1	-0.47	0.0204

62	A6NHG4	D-dopachrome decarboxylase	-0.47	0.0197
63	P13646	Keratin, type I cytoskeletal 13	-0.48	0.0189
64	Q8TC07	TBC1 domain family member 15	-0.48	0.0179
65	Q6IA69	Glutamine-dependent NAD(+) synthetase	-0.48	0.0178
66	Q9HA65	TBC1 domain family member 17	-0.48	0.0178
67	P07738	Bisphosphoglycerate mutase	-0.48	0.0169
68	Q9Y6G5	COMM domain-containing protein 10	-0.48	0.0169
69	P05109	Protein S100-A8	-0.49	0.0158
70	P17213	Bactericidal permeability-increasing protein	-0.49	0.015
71	Q9UHD9	Ubiquilin-2	-0.50	0.0128
72	Q53FA7	Quinone oxidoreductase PIG3	-0.50	0.0128
73	Q14D04	Ventricular zone-expressed PH domain-containing protein homolog 1	-0.50	0.0127
74	Q96B54	Zinc finger protein 428	-0.50	0.012
75	O76071	Probable cytosolic iron-sulfur protein assembly protein CIAO1	-0.50	0.012
76	P19013	Keratin, type II cytoskeletal 4	-0.51	0.0116
77	P21333	Filamin-A	-0.52	0.0099
78	P18065	Insulin-like growth factor-binding protein 2	-0.52	0.0097
79	P19012	Keratin, type I cytoskeletal 15	-0.52	0.0091
80	Q6UX06	Olfactomedin-4	-0.52	0.0086
81	O43237	Cytoplasmic dynein 1 light intermediate chain 2	-0.52	0.0085
82	P02533	Keratin, type I cytoskeletal 14	-0.57	0.0033
83	P53611	Geranylgeranyl transferase type-2 subunit beta	-0.58	0.0029

Supplementary figure S1

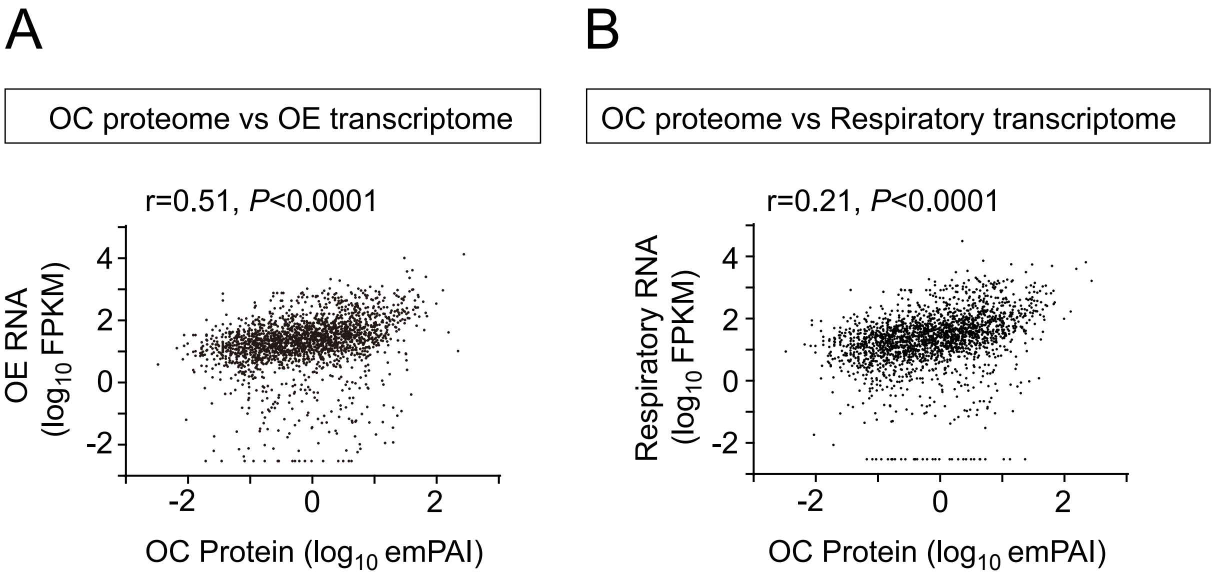

Supplementary figure S1 Comparison between shotgun proteomics and anti-body based multiplex assay
Scatter plot to deduce the correlation between shotgun proteome and antibody-based multiplex assay. X axis, log2 of IL-1RA concentration (pg/μl) determined by multiplex assay. Y axis, log2 of IL-RA concentration (emPAI/μl) determined by shotgun proteome analysis. Data from 22 subjects including both young and elderly are plotted. Data from shotgun proteomics showed linearity against different amounts of IL-1RA in multiplex assay. Spearman rank correlation coefficient (*r*) was calculated and shown inside this figure.

Supplementary figure S2

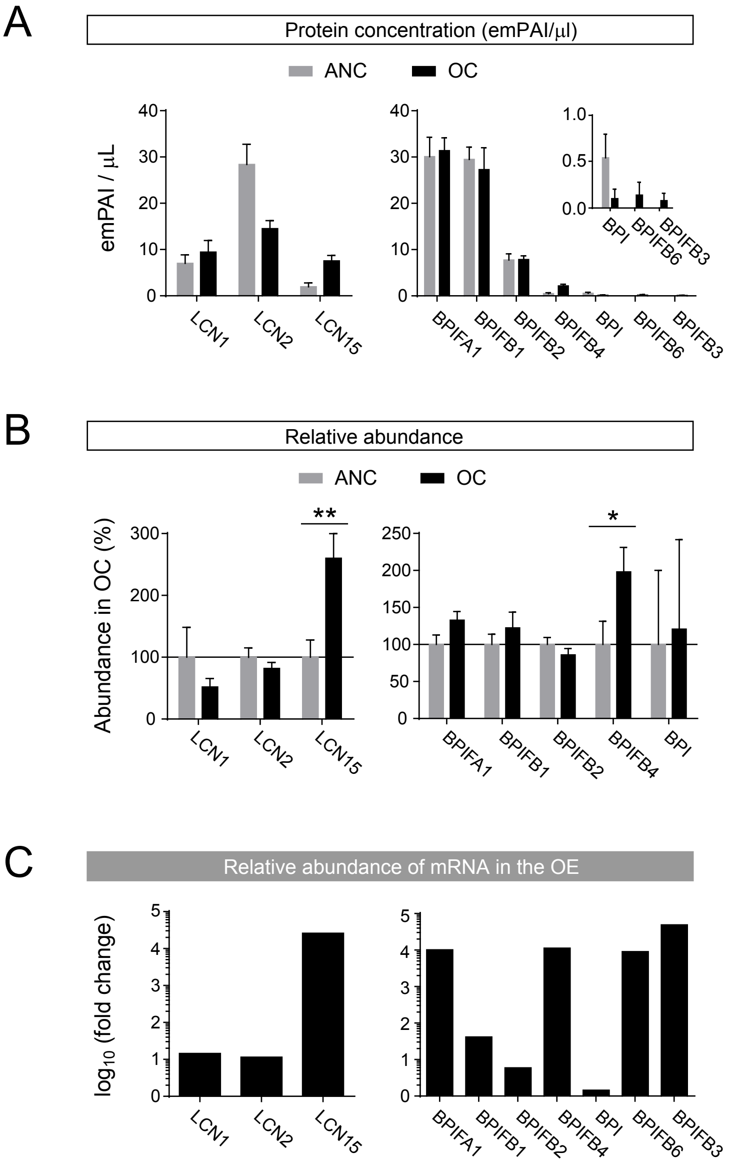

Supplementary figure S2 Regional difference in levels of lipocalins and BPI-fold containing proteins (A) Concentration (emPAI/μl) of each protein in young nasal mucus. LCN15 and BPIFB4 were enriched in OC mucus. BPIFB3 and BPIFB6 were detected only from OC mucus. (B) Average expression level (emPAI/μl) of each protein is set to 100% and relative expression levels in OC mucus are shown. (C) Relative mRNA-expression level in olfactory epithelium compared to non-olfactory tissues. Data is from a previous report[18)].

Supplementary figure S3

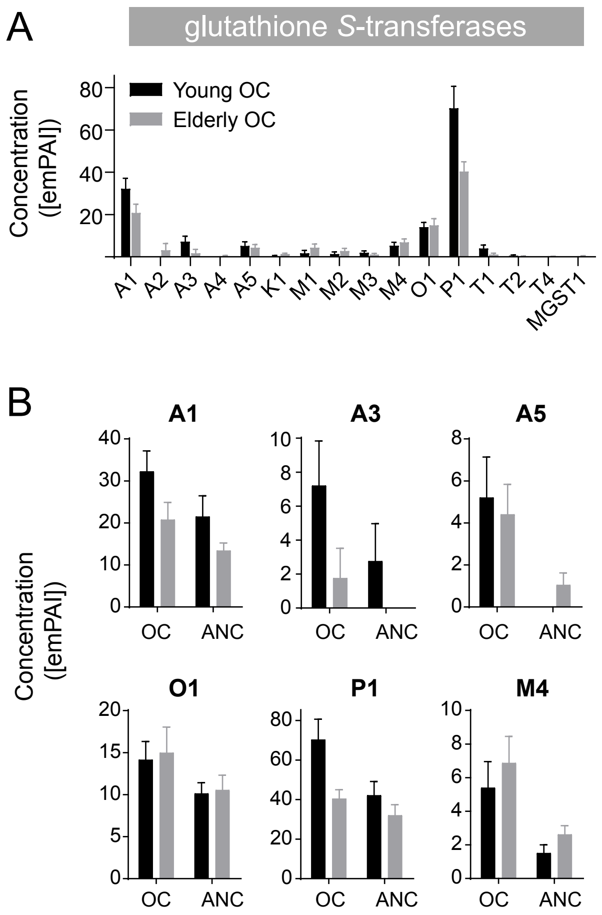

Supplementary figure S3 Age-related change in GST gene family in proteome analysis
Concentration of each GST gene family proteins in young and elderly mucus. (A) emPAI concentrations of each GST gene (Mean ± SE, n = 12). (B) Enlarged view of figure (A) for each protein.

Supplementary figure S4

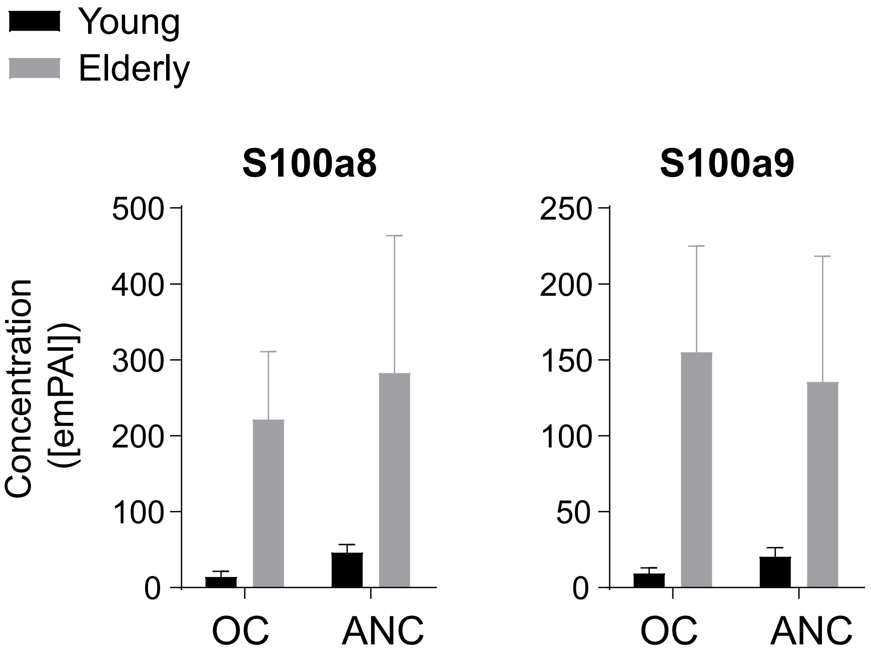

Supplementary figure S4 Age-related change in S100a8 and S100a9 gene family in proteome analysis
Each bar shows average emPAI concentration with standard error (n = 12).
